# Mutational spectrum and precision oncology for biliary tract carcinoma

**DOI:** 10.7150/thno.56539

**Published:** 2021-03-04

**Authors:** Jianzhen Lin, Yinghao Cao, Xu Yang, Guangyu Li, Yang Shi, Dongxu Wang, Junyu Long, Yang Song, Jinzhu Mao, Fucun Xie, Yi Bai, Lei Zhang, Xiaobo Yang, Xueshuai Wan, Anqiang Wang, Mei Guan, Lin Zhao, Ke Hu, Jie Pan, Li Huo, Xin Lu, Yilei Mao, Xinting Sang, Henghui Zhang, Kai Wang, Xiaoyue Wang, Haitao Zhao

**Affiliations:** 1 Department of Liver Surgery, Chinese Academy of Medical Sciences and Peking Union Medical College (CAMS & PUMC), Peking Union Medical College Hospital, No. 1 Shuaifuyuan, Wangfujing, Beijing 100730, China.; 2 Pancreas Center, The First Affiliated Hospital of Nanjing Medical University; Pancreas Institute, Nanjing Medical University, Nanjing 210000, China.; 3 Institute of Basic Medical Sciences (IBMS), Chinese Academy of Medical Sciences & Peking Union Medical College (CAMS & PUMC), Beijing 100730, China.; 4 Multidisciplinary Molecular Tumor Board of Hepatobiliary Tumors (Departments of Liver Surgery, Medical Oncology, Radiology, Radiotherapy and Nuclear Medicine), Peking Union Medical College Hospital, No. 1 Shuaifuyuan, Wangfujing, Beijing, China.; 5 School of Mathematical Sciences, Peking University, Beijing 100871, China.; 6 Department of Gastrointestinal Surgery, Peking University Cancer Hospital & Institute, Beijing 100142, China.; 7 OrigiMed Co. Ltd, Shanghai 201114, China.; 8 Institute of Infectious Diseases, Beijing Ditan Hospital, Capital Medical University, Beijing; Genecast Precision Medicine Technology Institute, Beijing 100089, China.

**Keywords:** biliary tract cancer, precision medicine, targeted therapy, genomic alterations, molecular screening

## Abstract

**Background:** The genomic spectrum of biliary tract carcinoma (BTC) has been characterized and is associated with distinct anatomic and etiologic subtypes, yet limited studies have linked genomic alterations with personalized therapies in BTC patients.

**Methods:** This study analyzed 803 patients with BTC:164 with gallbladder cancer, 475 with intrahepatic cholangiocarcinoma (ICC) and 164 with extrahepatic cholangiocarcinoma. We determined genomic alterations, mutational signatures related to etiology and histopathology and prognostic biomarkers. Personalized targeted therapies for patients harboring potentially actionable targets (PATs) were investigated.

**Results:** The median tumor mutation burden (TMB) was 1.23 Mut/Mb, with 4.1% of patients having hypermutated BTCs. Unlike the results obtained from the Western population, the most frequently altered cancer-related genes in our cohort included *TP53* (53%), *KRAS* (26%), *ARID1A* (18%), *LRP1B* (14%) and *CDKN2A* (14%). Germline mutations occurred mostly in DNA damage repair genes. Notably, 35.8% of the ICCs harbored aristolochic acid related signatures and an elevated TMB. *TP53* and *KRAS* mutations and amplified 7q31.2 were demonstrated to negatively affect patient prognosis. Moreover, 19 genes were proposed to be PATs in BTCs, with 25.4% of patients harboring these PATs. Forty-six patients received PAT-matched targeted therapies, achieving a 26.1% objective response rate; the median progression-free survival (PFS) was 5.0 months, with 56.8% of patients obtaining PFS benefits.

**Conclusions:** Extensive genomic diversity and heterogeneity were observed among BTC patients, with contributions according to potential etiology exposures, anatomical subtypes and clinicopathological characteristics. We also demonstrated that patients with refractory BTCs who have PATs can derive considerable benefit from receiving a matched therapy, initiating further prospective clinical trials guided by molecular profiling among this aggressive cancer.

## Introduction

Biliary tract carcinoma (BTC) is an aggressive malignancy that arises from epithelial cells lining the bile duct. Histopathological types of BTC are classified based on the anatomical location and include intrahepatic cholangiocarcinoma (ICC), extrahepatic cholangiocarcinoma (ECC) and gallbladder cancer (GBC) 1. The incidence and mortality rate of BTC have been increasing in the past few decades 2,3. Due to the insidious onset and absence of symptoms at an early stage, only 10% of BTC patients are considered candidates for surgery at their initial diagnosis 4. Thus, the 5-year survival rate of patients with unresectable BTCs is less than 10% 5,6. As all nonradical surgeries and adjunctive therapies are palliative 7, novel therapies especially for BTC are urgently needed, especially for patients who progress after first-line gemcitabine-based chemotherapy.

Previous molecular profiling studies through whole-exome sequencing (WES) or targeted panel sequencing (TPS) have revealed recurrent genomic alterations in BTC patients 8,9. Frequent alterations in *IDH1/2*, *FGFR1/2/3*, *EPHA2*, and *BAP1* were noted predominantly in ICC, whereas mutations in *KRAS*, *ARID1B*, *ELF3* and *PBRM1* occurred preferentially in ECC. GBC harbors common alterations in *TP53* and *ERBBs*. Moreover, according to observations from early-stage clinical trials, personalized molecular targeted therapies have emerged as a potentially promising strategy for treating patients with refractory BTCs 10,11. However, the etiologic factors from different populations leading to various genotypes and molecular phenotypes are poorly understood, and more genomic data and clinical practice are required to uncover the prognostic or predictive implications of genetic alterations for patients with advanced BTC.

To identify underlying genomic targets with clinical translational significance, we conducted a comprehensive analysis of genome profiles from a Chinese cohort of 803 BTC patients. We systematically delineated the somatic mutational spectrum and germline alterations in BTC patients and investigated their links with the etiological background, prognosis and therapeutic responses for personalized molecular targeted therapies.

## Methods and materials

### Patients and samples

The entire dataset included a total of 803 BTC patients from two major cohorts. Data on 92 patients with surgically resected ICCs from a previously published cohort were obtained from the Sequence Read Archive (SRA) under accession number *SRP045202* 12. Another cohort was established from the Precision Treatment of Hepatobiliary Cancers (PTHBC, NCT02715089) program. A total of 711 histopathology confirmed BTC tumors and paired tumor-free samples were collected, and written informed consent for tumor genomics profiling via WES or TPS was obtained from each participant. In the PTHBC cohort, if patients initially enrolled in the molecular screening program were eligible for biomarker guided treatments (BGTs) with actionable molecular alterations, our Molecular Tumor Board (MTB) at the leading center (Peking Union Medical College Hospital, PUMCH) was responsible for recommending a concrete regimen or a registered trial to proceed with personalized targeted therapies under the premise of well-informed explanations about all underlying benefits and risks for receiving optional treatments and a hard copy of another signed consent form. The study protocol and informed consent were approved by the Institutional Ethics Review Committee at PUMCH. The study was conducted in accordance with the Declaration of Helsinki and Good Clinical Practice guidelines.

In total, 803 BTC patients underwent genomic profiling, and the baseline clinicopathological characteristics are summarized in Table 1 (detailed clinicopathological data was presented in Supplementary Table S1). A total of 160 BTC tumors and matched control samples were subjected to WES, with a median coverage of 108× in coding exons in tumors and 72× in paired control samples. For TPS, we used a hybridization capture-based next-generation sequencing (NGS) platform (Cancer Sequencing YS panel, CSYS 13) with an average ultradeep sequencing depth of 1021× that has been demonstrated 14 to highly and accurately identify genomic alterations in whole exons and selected introns of 450 cancer-associated genes (Table S2); tumor samples from 643 BTC patients were analyzed.

### Determination of potentially actionable targets (PATs) in BTCs

The two leading evidence-based knowledge databases, OncoKB 15 and ESMO Scale for Clinical Actionability of molecular Targets (ESCAT) 16, were referenced to determine the potential actionabilities of genetic alterations in druggable targets. OncoKB is a precision oncology knowledge base that comprehensively considers the guidelines and recommendations from the FDA, National Comprehensive Cancer Network (NCCN) and medical literature. The ESCAT was launched by the European Society for Medical Oncology (ESMO) Translational Research and Precision Medicine Working Group to facilitate the implementation of precision medicine in the clinical management of cancer.

In our study, we defined PATs as genomic alterations classified according to the scoring system of OncoKB (tier ≤level-3A) or ESCAT (tiers ≤II-B), and their matched targeted therapies have shown compelling clinical efficacy in treating BTC or other tumors. Consequently, a total of 19 genes could be classified as PATs in BTCs (Table S3).

### MTB and treatment allocation

The MTB held multidisciplinary face-to-face meetings approximately twice a month and was attended by oncologists, surgeons, radiologists, pathologists, genetic scientists and bioinformatics specialists, all of whom were from the Chinese Academy of Medical Science (CAMS) & Peking Medical Union College (PUMC) and PUMCH. Clinical trial coordinators or navigators also participated in the multidisciplinary meetings of the MTB.

The MTB generally discussed the rationality of biomarker-guided therapies and developed a concrete regimen for patients who harbor potentially druggable targets. Notably, participants with 19 PAT genes were considered candidates to receive personalized targeted therapies (in-PATs), and patients carrying druggable targets of OncoKB level-3A/B or ESCAT tier-III with strong desires and no standard therapeutic regimen for advanced tumors were considered off-PATs. The prioritization to assign personalized targeted therapies according to druggable targets was as follows: (I) those with 19 PAT genes had the highest priority; (II) if multiple PATs were simultaneously detected in a single patient, the optimal evidence between OncoKB and the ESCAT was accepted (for instance, if a patient with advanced ICC was identified as having both the MSI-H status and *ERBB2* amplification, the optimal level of the MSI-H status was ESCAT's I-C, whereas the optimal level of *ERBB2* amplification was OncoKB's level-2B; thus, the MTB developed a regimen of pembrolizumab for this patient with MSI-H ICC); and (III) if patients harbored mutant *EGFR*, considering that mutations in EGFR exons 17-20 are scant in BTCs, the overexpression of IHC 2+/3+ was required for each patient to receive anti-EGFR treatment with afatinib 17.

The patient registry included optional consent to collect real-world outcomes longitudinally for research purposes. Once the patients received targeted treatments, follow-up was conducted to evaluate the efficacy and safety of the drugs until the determination of overall survival (OS). Patients with primary eligibility were those with at least one druggable target who required palliative care after systematic chemotherapy. Patients were required to have an Eastern Cooperative Oncology Group (ECOG) performance status of 0-2 and normal baseline organ and bone marrow functions. All patients scheduled to receive targeted drugs had at least one measurable lesion that was used to assess the therapeutic response according to the Response Evaluation Criteria in Solid Tumors (RECIST, v1.1) 18.

### Statistical analysis

All statistical analyses were performed using R version 3.5.7. Continuous variables are expressed as the mean ± SD if they were normally distributed; otherwise, they are expressed as the median with the interquartile range. A two-tailed unpaired t-test was used for comparisons between two groups. Fisher's exact test and post-hoc tests were used for comparisons between multiple groups. Variables associated with disease-free survival (DFS) and OS were identified using univariable and multivariable Cox proportional hazards regression models. Kaplan-Meier plots (log-rank tests) were used to describe prognostic factors related to DFS and OS. The R package “ggplot2” was used to draw figures. All reported P values are two-tailed, and P < 0.05 was considered statistically significant.

For further details regarding the materials and methods used, please refer to the **Supplementary methods**.

## Results

### Populational characteristics and tumor mutation loads

**Figure 1:**
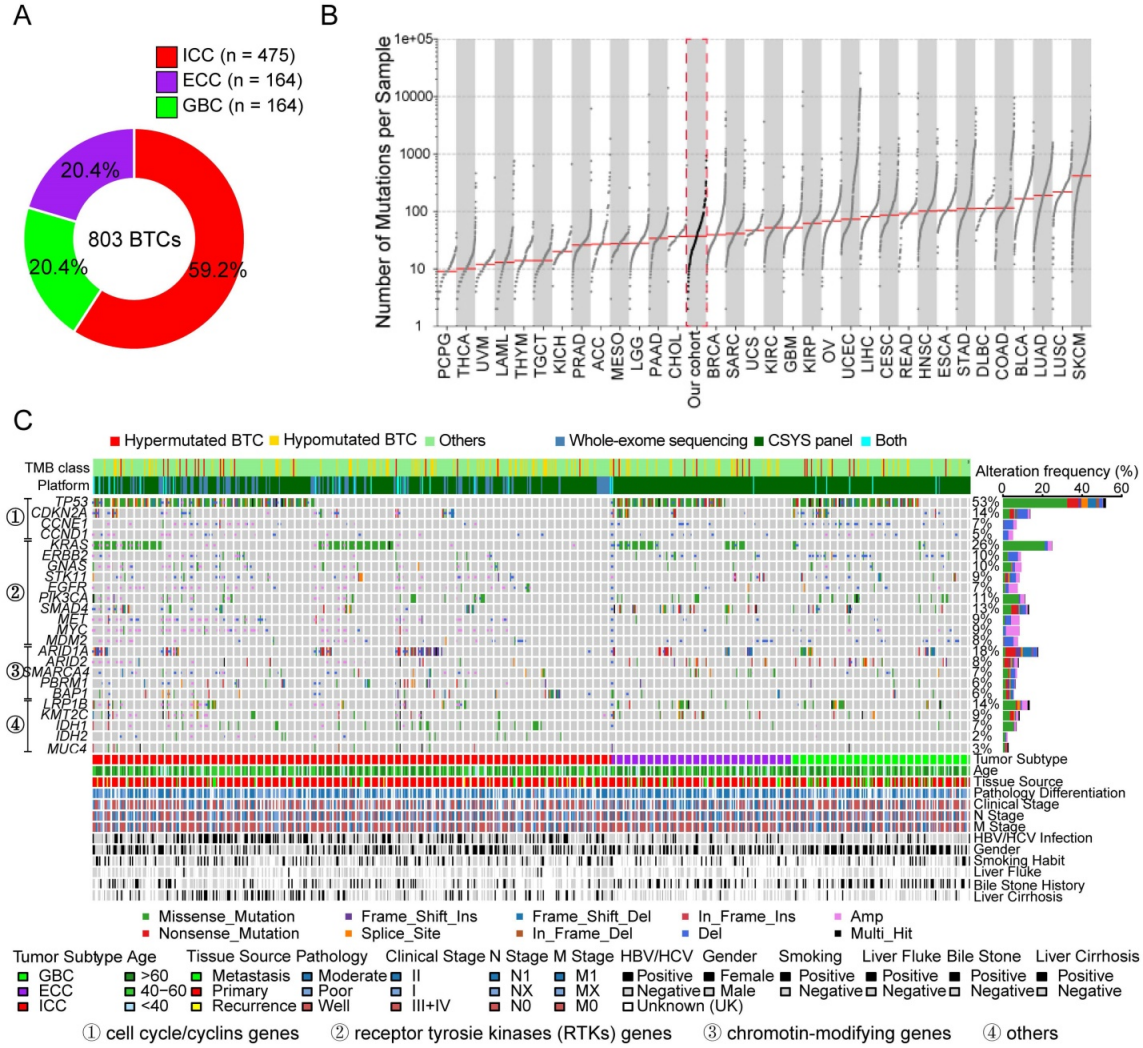
** Genomic mutation profiles of 803 BTC patients. (A)** Numbers and proportions of the three anatomical subtypes of BTC. **(B)** Landscape of tumor mutation burden (TMB) across the major tumor types; the median level of TMB for each tumor type and our cohort is highlighted. **(C)** Mutation profiles of driver genes detected by *MutSigCV* and frequently mutated BTC-related genes. Mutant frequencies in the cohort are shown on the right, and associated clinicopathological characteristics for all 803 patients are shown at the bottom.

The genomic profiles of 803 patients with BTC (475 with ICC, 164 with ECC and 164 with GBC) were analyzed (Figure 1A). In total, 27,042 somatic single-nucleotide variants (SNVs) and 15,425 small indels were identified (a full list of the SNVs and indels is provided in Table S4). Due to the different processing methods between WES and TPS, we sequenced a subset of 31 tumor samples by both WES and TPS, achieving excellent concordance in the variant allele fractions (VAFs) identified between modalities (*R^2^* = 0.81, Figure S1A); at the gene level, copy-number variations (CNVs) were highly consistent between WES and TPS (Figure S1B). Next, the tumor mutation burden (TMB) was estimated for the entire cohort. We separately defined a threshold for hypermutated BTC considering the sequencing depth, VAF and targeted region of each method (WES, ≥9.36 Mut/Mb; TPS, ≥16.1 Mut/Mb, Figure S2A). As a result, 4.1% (33/803) of patients were identified as having hypermutated tumors. We also noticed that TMB was highly consistent between sequencing experiments (*R^2^* = 0.93, p < 0.001, Figure S2B). The median (IQR) TMB determined by WES was 1.23 (0.7-2.34) Mut/Mb, which was similar to that for the TCGA-CHOL cohort (Figure 1B). Among the different subtypes of BTC, the highest TMB was observed in GBC (Figure S2C-D).

### Somatic mutational spectrum

A total of 37 significantly mutated genes (SMGs) were determined in 160 pairs of WES data (q < 0.1, Table S5) by using *MutSigCV* 19. In addition to these SMGs, we searched for somatic alterations in 68 putative oncodriver genes of BTC (Supplementary methods and Table S6) in the entire series of 803 tumor samples (Figure 1C). Consequently, the recurrently altered genes were determined to be associated with the cell cycle, receptor tyrosine kinases (RTKs) and chromatin-regulating modifiers, consistent with a previous report 20. *TP53* (53%), *KRAS* (26%), *ARID1A* (18%) and *LRP1B* (16%) were the most frequently mutated genes. Notably, *IDH1* and *IDH2* mutations, which are common (approximately 25%) in cholangiocarcinoma (CCA) patients of Western populations 21, were observed in only 7% and 2% of Chinese BTC patients, respectively. Furthermore, an alteration in* KMT2C* (also known as *MLL3*) was detected in 9% of BTC patients, suggesting that a subset of these patients exhibit abnormal histone methylases and demethylases. Next, the mutation simultaneity or exclusivity status of these driver mutations across the three subtypes of BTC was explored. Coaltered statuses were observed for *TP53*:*CDKN2A*, *TP53*:*TERT*, and *TP53*:*LRP1B*, as well as for *KRAS*:*CDKN2A*, *KRAS*:*SMAD4* and *ARID1A*:*SMAD4* (Figure S3A). In contrast, *TP53* mutations and *ARID1A*, *MUC4*, *IDH1* or *EPHA2* mutations were identified exclusively in BTCs (Figure S3B), and patients who carried *KRAS* mutations showed mutual exclusivity with patients identified as carrying *LRP1B*, *TERT*, *PIK3CA*, *IDH1* or *EPHA2* mutations (Figure S3C).

**Figure 2:**
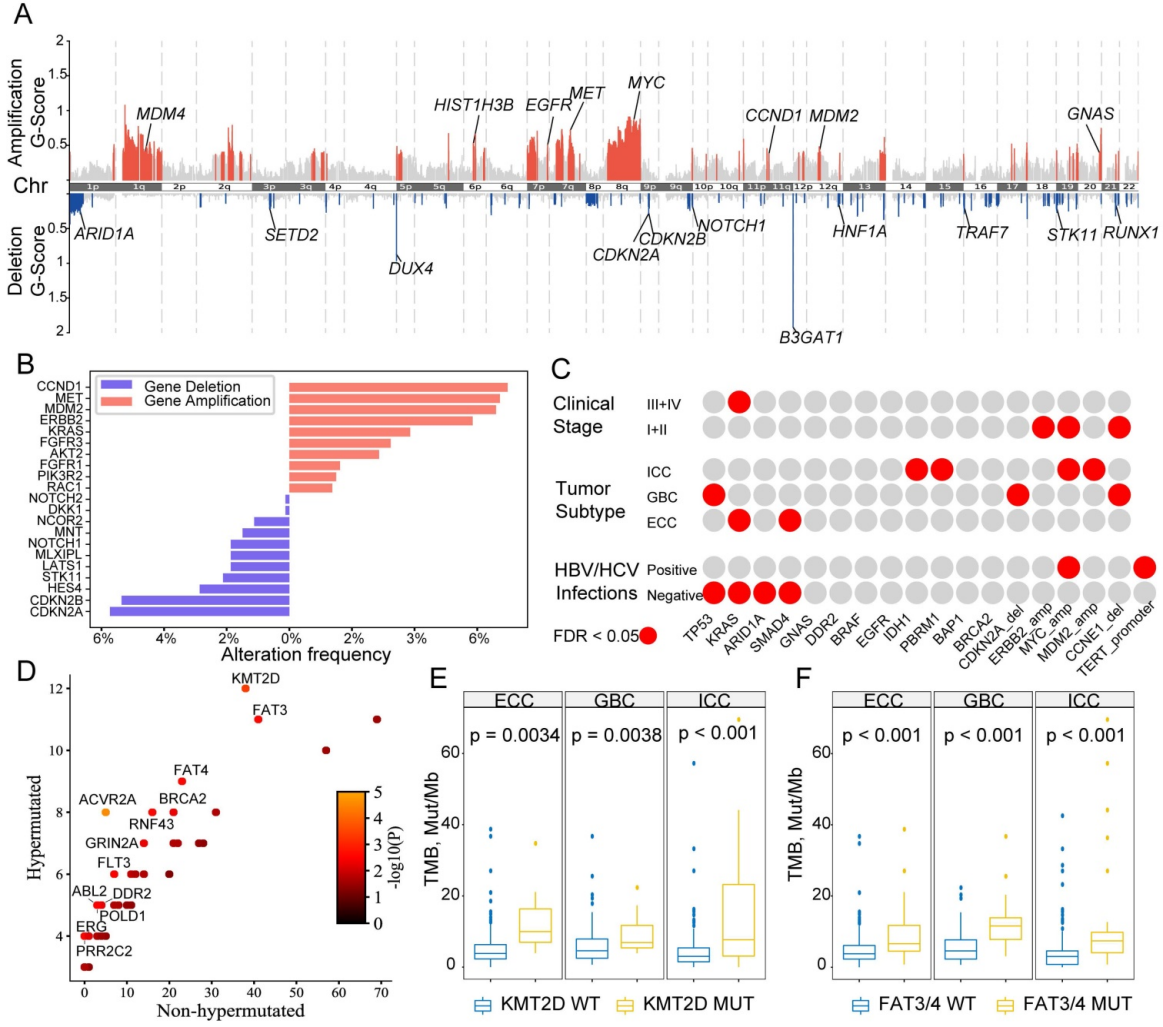
** Identification of chromosomal somatic copy-number alterations (SCNAs) in putative cancer driver genes. (A)** Significant (q < 0.1) focal SCNAs along all chromosomes. The vertical axis indicates the G-scores generated from *GISTIC2*, which considers the amplitude of the aberration and the frequency of its occurrence across samples. Recurrent SCNAs of putative cancer driver genes are also highlighted. **(B)** Proportions of patients from the entire cohort (n = 803) for with significantly amplified (red) or deleted (blue) cancer driver genes. **(C)** Correlations between driver mutations and clinical phenotypes in the entire cohort; significant correlations are highlighted in red. Two-sided Fisher's exact tests were then performed, and an FDR cutoff of 0.05 was used for reported genes. **(D)** Scatter plots depicting the mutational frequencies (percentage of patients) between patients with hypermutated tumors and non-hypermutated tumors in our cohort. Each dot represents one gene, and dots are color coded according to the P-values (-log10(P) uncorrected) shown in the legend. Statistics shown were derived from two-sided Fisher's exact tests. **(E-F)** Comparisons of TMB between mutant and wild-type KMT2D (E) or FAT3/4 (F) separated by histological subtypes.

To delineate significant CNV events, *GISTIC2* 22 analysis using WES data for tumor tissue and normal tissue revealed 129 focal chromosome amplifications and 111 focal chromosome deletions (Table S7). We observed significant amplifications in known oncogenes and deletions in tumor-suppressor genes (TSGs, Figure 2A). For instance, *CCND1*, *MET*, *MYC*, *EGFR* and *MDM2/4* were significantly amplified, but *CDKN2A/2B*, *ARID1A* and *STK11* were significantly deleted. The populational frequencies of these CNV-driving alterations in the entire series of 803 BTCs were also analyzed (Figure 2B), and the most commonly amplified oncogene was *CCND1* (6.97%), followed by *MET* (6.72%), *MDM2* (6.6%) and *ERBB2* (5.85%), whereas the main TSGs were *CDKN2A* (5.73%) and *CDKN2B* (5.35%).

We next correlated these genomic mutations with clinical features in the BTC cohort (Figure 2C). *TP53* mutations occurred more commonly in patients with GBC, whereas *KRAS* mutations were enriched in patients with late-stage BTC (phases III-IV) and ECC. Patients with ICC more frequently harbored *IDH1* and *PBRM1* mutations, as well amplifications of *MYC* and *MDM2*. Notably, we found a potential impact of hepatitis viral (type B or C) infection on mutational frequencies: BTC patients with hepatitis B/C virus infections had significantly lower rates of mutations in *TP53*, *KRAS*, *ARID1A* and *SMAD4*, with increased rates of mutation in the *TERT* promoter region and *MYC* amplification. Moreover, when comparing driver genes between hypermutated and other BTCs, 13 genes among the hypermutated tumors (Figure 2D) including *KMT2D*, *FAT3/4* (Figure 2E-2F), and several DNA damage repair (DDR) genes, such as *BRCA2*, *DDR2* and *POLD1*, showed significant differences in frequency.

Collectively, all identified somatic alterations in the three BTC subtypes (ICC, ECC and GBC) were assembled to illustrate a mutational landscape (Figure S4). Genomic alterations, including somatic mutations and CNVs were classified into 10 canonical oncogenic signaling pathways 23. After excluding synonymous mutations and germline alterations, the BTC mutational landscape indicated that the most frequently altered pathways were receptor tyrosine kinase (RTK)/RAS (72.5%, 582/803) and p53 (66%, 530/803) signaling, consistent with a previous report 24. Among the three subtypes of BTC, ICC showed the highest rate of mutations in the PI3K, Notch and Myc pathways. *SMAD4* inactivation and alterations in transforming growth factor (TGF)-β family receptors were more recurrently altered in ECC, and mutations in p53 and the cell cycle pathway were most commonly observed in GBC.

### DDR mutants and germline mutations

**Figure 3:**
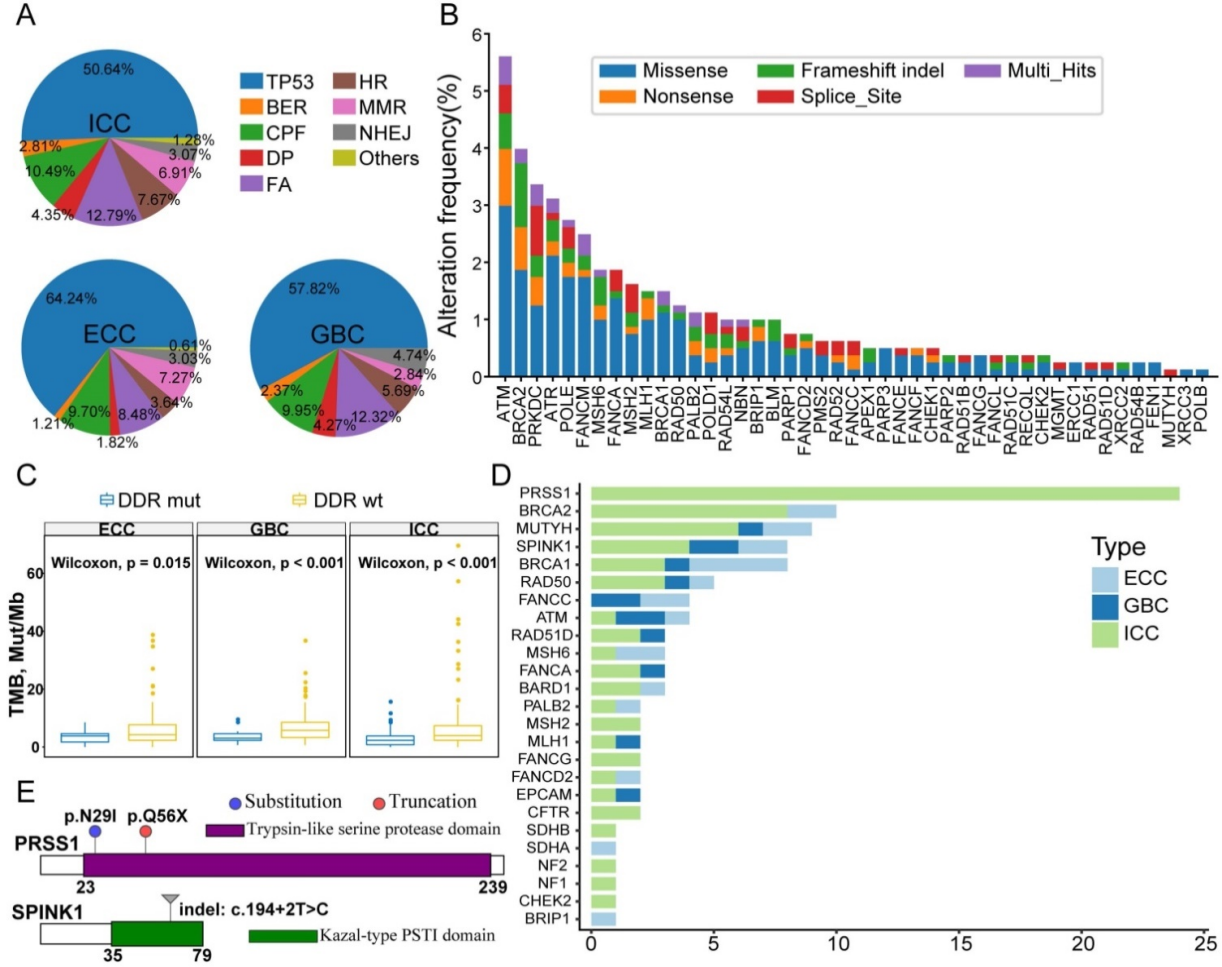
** Identification of DDR mutants and germline variants. (A)** Percentage of patients with the three different BTC subtypes with somatic mutations in DDR genes classified by *TP53* and various functional families of non-*TP53* DDR genes. **(B)** Somatic mutation spectrum of non-*TP53* DDR genes in BTC ranked by their prevalence. The color of the bars represents the type of genomic alteration. **(C)** Patients harboring mutated DDR genes have a significantly higher TMB than those with wild-type DDR; this was consistent across the three individual BTC subtypes. **(D)** Numbers of patients with pathogenic or pathogenic-likely germline variants; the findings indicate that *PRSS1*, *BRCA2*, *MUTYH*, *SPINK1* and *BRCA1* mutations are recurrent germline mutations in BTC patients. **(E)** Annotations and locations of *PRSS1* (upper) and *SPINK1* (bottom) mutations in our cohort.

DDR mutants represent some tumors harboring sporadic mutations in DDR genes, and DDR deficiency is a hallmark of BTC 25. Thus, we assessed the prevalence of alterations in DDR genes to provide insight into those genes in our cohort. Considering that most DDR genes have not yet been determined to have oncogenic effects in BTCs as well as the cotargeted gene pool between WES and TPS, 47 DDR genes involved in TP53, covering eight major functional DDR pathways, were estimated for the entire cohort (Table S8). A total of 65.5% (526/803) of BTC patients were classified as having DDR mutants, 284 of which were due to *TP53* mutations. Indeed, *TP53* was the most commonly altered DDR gene in all three different pathologic subtypes of BTC (Figure 3A); checkpoint factors (CPFs), Fanconi anemia (FA) and mismatch repair (MMR) were the main functional categories of non-*TP53* DDR mutants. Among the 242 patients with non-*TP53* DDR mutations, the most commonly altered DDR genes were *ATM*, *BRCA2*, *PRKDC*, *ATR* and *POLE* (Figure 3B). Accordingly, among the different subtypes of BTCs, we observed a significantly higher TMB in patients with mutated DDR genes than in those with wild-type DDR genes (Figure 3C).

Pathogenic germline mutations are found sporadically in pancreaticobiliary cancers, and our previous study revealed that ICC patients with germline *BRCA2 (gBRCA2)* mutations had susceptible genetic hereditary phenomena in their families 14. Thus, we inferred known pathogenic germline alterations in the present cohort using a validated computational prediction method combined with manual calibrations. This analysis showed that 96 of 803 (12%) patients carried pathogenic or likely pathogenic germline mutations (Table S9), all of which were heterozygous alterations. Notably, our results demonstrated that genetic predisposition toward chronic hereditary pancreatitis and pancreatic carcinogenesis, which involve *PRSS1* and *SPINK1*, in BTC patients was associated with susceptibility to germline mutations (Figure 3D). A total of 24 ICC patients harbored *PRSS1* missense mutations in germline tissues (23 altered loci were N29I and 1 locus was Q56X, Figure 3E). Eight patients, including 4 with ICC, 2 with ECC and 2 with GBC, carried a germline *SPINK1* indel (c.194+2T > C, Figure 3E). For the 32 patients, the median age of cancer diagnosis was 54.5 years (IQR: 48-61); 8 patients reported a family history of cancer, though only one patient had a personal history of chronic and recurrent pancreatitis. Our data indicate for the first time that gain-of-function mutations in the *PRSS1* gene and loss-of-function mutations in the *SPINK1* gene in the germline are prevalent in BTC patients. In addition, other germline mutations occurred mostly in DDR genes, with* BRCA2* (n = 10), *MUTYH* (n = 9) and *BRCA1* (n = 8) being the most common in the BTC patients in this study.

### Identification of aristolochic acid (AA)-related mutational signatures in ICC patients and prognostic alterations

**Figure 4:**
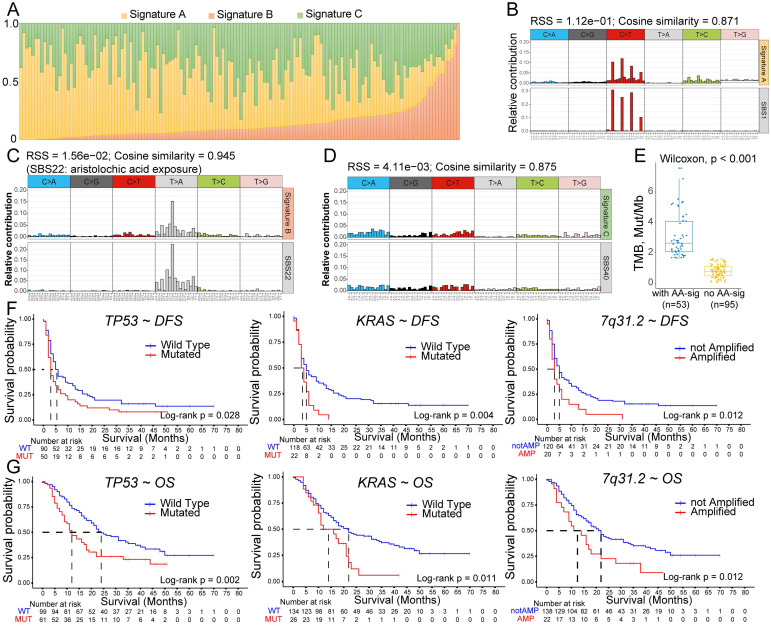
** Analyses of mutational signatures in ICC and prognostic alterations. (A)** Contribution of the three mutational signatures among 148 ICC patients. **(B)** Signature A identified from ICC samples is linked to COSMIC age-related single-base substitutions (SBSs) of SBS1. **(C)** Aristolochic acid (AA) signature (signature B) mutations were identified in 53 of 148 ICC tumors. The relative mutation frequencies of all 96 trinucleotide mutation patterns are plotted, with AA-related mutation patterns labeled in gray. **(D)** Signature C is linked to COSMIC age-related SBS40. **(E)** Comparisons of TMB in tumors with and without the AA signature. The line and box represent the median and upper and lower quartiles, respectively. **(F)** Negative impacts of *TP53* and *KRAS* mutations and 7q31.2 amplification on postoperative disease-free survival (DFS) in 140 patients. **(G)** Negative impacts of *TP53* and *KRAS* mutations and 7q31.2 amplification on postoperative overall survival (OS) in 160 patients.

The various etiologies of BTCs may point to distinct somatic mutational signatures of single-base substitutions (SBSs). Considering that most WES data (152 of 160) for our present cohort were generated from ICC patients, we extracted mutational signatures by using non-negative matrix factorization (NMF) analysis of SBSs in ICCs. Because hypermutated tumors have been demonstrated to possess different substitution patterns 24, we excluded 4 hypermutated ICCs. This NMF analysis revealed three unique signatures (Figure 4A): signature A, with a dominant pattern of C > T (Figure 4B); signature B, dominated by A:T > T:A transversions (Figure 4C); and signature C, without a dominant SBS pattern (Figure 4D). We further compared these three signatures to the SBS Signatures of Human Cancer in the COSMIC database 26. Signature A and signature C correlated with SBS1 (correlation similarity of 0.871) and SBS40 (correlation similarity of 0.875), both of which reported to correlate with patient age. Intriguingly, signature B showed a strong correlation with SBS22, with a cosine correlation similarity of 0.945, suggesting that AA exposure may contribute to carcinogenesis in Chinese ICC patients. In total, 35.8% (53/148) of our ICC patients were identified as having an AA signature (false discovery rate < 0.05). Consistent with previous reports in hepatocellular carcinoma (HCC) 27, TMB in AA signature-containing ICCs was found to be significantly higher than that in non-AA ICCs (Figure 4E), demonstrating that AA exposure might lead to DNA damage and the accumulation of somatic mutations in cholangiocarcinoma.

To comprehensively assess the prognostic significance of these mutations in ICCs, we conducted survival analyses on patients for whom WES data were available using both univariate and multivariate Cox regression models for DFS and OS. Univariate analysis revealed that *TP53* mutations, *KRAS* mutations and 7q31.2 amplification had significantly negative impacts on both DFS and OS (Figure 4F-4G). Other genomic risk factors for short DFS included 7p15.2, 5p15.33 and 17q21.2 amplifications; in contrast, patients harboring mutated *EPHA2* or *IDH1* had a significantly improved OS (Table S10). The multivariate Cox regression model, which combined prognostic SMGs, somatic copy-number alterations (SCNAs) and clinicopathological features, revealed that 7q31.2 amplification and an early clinical stage (phase I) were able to independently predict DFS; *KRAS* mutations and vascular invasion were independent variables for OS (Table S10). Both *TP53* and *KRAS* have been demonstrated to play a tumor-promoting role in BTC 20,28. Notably, our study is the first to indicate that 7q31.2 amplification is likely an oncogenic focal region in ICCs. This chromosomal region harbors several protein-coding genes that have been validated as oncogenes in solid tumors, such as *MET*, *CAV* (caveolin) and *WNT2* 29-31, which may account for its tumor-promoting role in ICC.

### Annotations of PATs and translational personalized therapies

**Figure 5:**
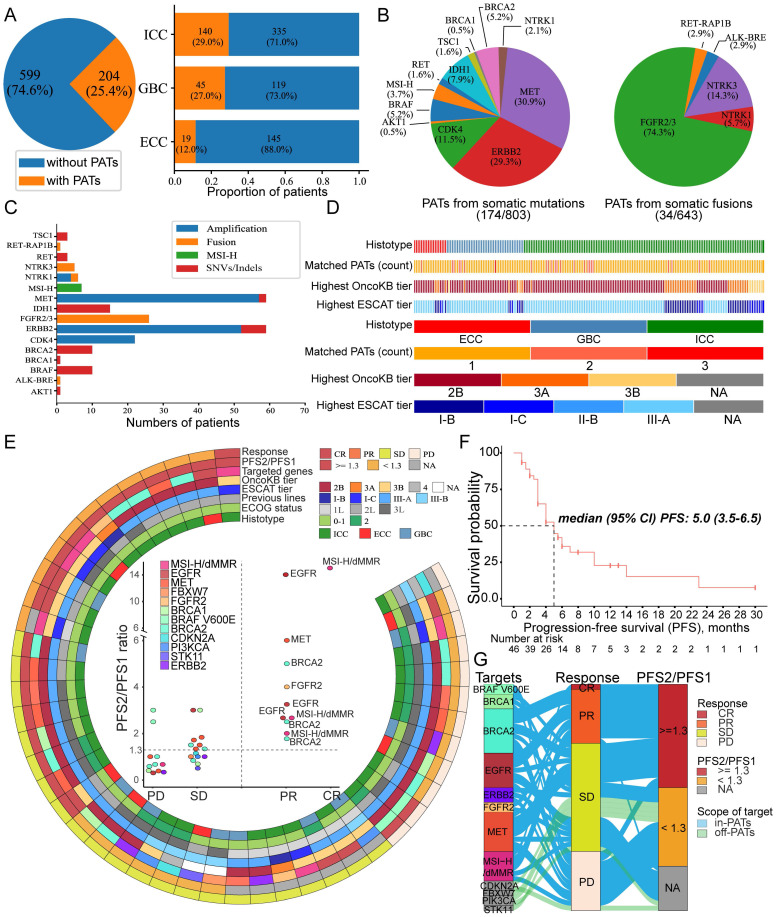
** Potentially actionable targets (PATs) and biomarker-guided targeted therapies in BTC patients. (A)** Pie graph of the percentage of patients harboring at least one PAT in the entire cohort (803 patients with BTCs); the populational proportion of PATs in each subtype of BTCs is shown as a bar plot. **(B)** Pie graphs show the percentage of PATs in mutation-originated targets (left) for all 803 BTCs and fusion-originated targets (right) among 643 patients who underwent CSYS panel analysis. As some patients had multiple PATs, the percentages do not add up to 100%. **(C)** The stacking diagram represents the number of patients and number of mutational sources for each PAT. **(D)** The upper scattered heatmap indicates the histotype, PAT count, relative highest OncoKB tier and highest ESCAT tier for each patient with available PATs among 204 patients. The colored bars at the bottom indicate the different levels of each parameter. **(E)** Efficacy-pathologic-genomic landscape of umbrella-setting BGTs in 46 patients with advanced or metastatic BTCs. The central scatter plot shows the response outcomes (x-axis) and PFS2/PFS1 ratio (y-axis) for each PAT. Targets with a PFS2/PFS1 ratio ≥ 1.3 and a responsive status (CR or PR) are highlighted in the center plot. **(F)** Survival analysis of progression-free survival (PFS) estimated by Kaplan-Meier curves among 46 patients who received BGTs. **(G)** The flow diagram in the left panel illustrates the list of druggable targets, the corresponding therapeutic response and the PFS2/PFS1 ratio. The colors of the curved belts indicate whether the druggable targets belonged to the 19 genes proposed as PATs in BTC, and the widths of the belts indicate different frequencies for each target at every level. *Abbreviations*: MSI-H: microsatellite instability-high; CR: complete response; PR: partial response; SD: stable disease; PD: progressive disease; PFS: progression-free survival; ECOG: Eastern Cooperative Oncology Group; dMMR: deficient mismatch repair.

There have been massive efforts to translate genomic alterations into actionable targets 32,33. In this study, we defined PATs as genomic alterations classified by the scoring system of OncoKB 15 (tier ≤level-3A) or the ESCAT 16 (tiers ≤II-B), as their matched molecular targeted therapies have shown compelling clinical efficacy in treating BTC and other tumors. Therefore, we propose 19 genes as PATs in BTC (Table S3). In this context, 204 (25.4%) patients harbored at least one PAT and 34 patients harbored two or more PATs (Figure 5A). Among the different subtypes, the lowest proportion of PAT carriers was found among ECC patients (p < 0.001, Figure 5A). Of the population with available PATs, SCNA-originating PATs, including *MET* (29.3%), *ERBB2* (29.3%) and *CDK4* (11.5%), were commonly identified (Figure 5B). Conversely, PATs originating from SNVs or indels were less common due to the low proportions of the designated mutated loci in BTC. For example, *EGFR* mutations in exons 18-21 are common druggable targets in lung cancer 34; although it is designated as a PAT in other tumors, mutations at this specific locus are absent in BTC, suggesting a potential risk of narrowing the genome-driven targeted therapy coverage population when only accurate mutated loci are considered. Additionally, several rearrangements involving gene fusions, such as *FGFR2/3* and *NTRK1/2/3*, are encouraging as biomarkers for guiding targeted treatments. Accordingly, we also investigated somatic fusion-originating PATs in 643 patients whose tumor samples were examined using the CYCS platform. We found that 4% (26/643) of patients harbored *FGFR2/3* fusions (Figure 5B), with multiple fusion partners identified (Table S11). In addition, sporadic *NTRK1/3* fusions were detected in 6 patients, indicating that potent treatment with larotrectinib may be suitable for only ~1% of BTC patients 35.

dMMR tumors are highly sensitive to immune checkpoint blockade (ICB) targeted PD1/PDL1 therapy, regardless of the cancer tissue of origin 36,37. Moreover, dMMR tumors are characterized by sequence alterations in microsatellites, and the cancer genome can accumulate vast somatic mutations, suggesting that microsatellite instability-high (MSI-H) is a directly-related genomic presentation 38. In our cohort, only 1.2% (10/803) of BTC patients had an MSI-H status (Figure 5C), suggesting that a limited proportion of patients with advanced BTC may be candidates for in-label ICB systematic therapy. Overall, the clinical evidence is not strong for most PATs identified in BTC patients, and these PATs are usually recommended as tier level-2B of OncoKB or tier II-B of the ESCAT (Figure 5D).

Limited studies have investigated the therapeutic responses to mutation-guided personalized treatments in patients with advanced BTCs. Herein, we describe 46 patients with refractory BTCs with actionable molecular anomalies who received personalized targeted therapies (Table S12). We classified the MTB recommendations as in-PAT personalized therapies and off-PAT personalized therapies, with the scope of the former being defined as the proposed 19 genes mentioned above. In this cohort, 40 patients received in-PAT personalized therapies, and 6 patients received personalized therapies based on off-PAT as level-3B/tier-III due to their strong willingness but the lack of an effective chemotherapy regimen for refractory and metastatic tumors.

The baseline clinicopathological features of these patients are summarized in Table S13, and it worth mentioning that 46% (21/46) of the patients had ≥ 3 prior lines of systematic antitumor treatments. Detailed information about the clinicopathological features and therapeutic responses is presented in Figure 5E. Primary outcomes for evaluating efficacy included the objective response rate (ORR) and progression-free survival (PFS). Considering that PFS becomes shorter with each line of therapy administered, we also calculated the PFS ratio (PFS2/PFS1: the ratio of PFS during precision oncology (PFS2) compared with that of the immediate prior line of unmatched therapy (PFS1)). PFS2/PFS1 ≥1.3 indicates a therapeutic benefit, as based on the work of Von Hoff *et al* 39 and the “I-PREDICT” trial 40. Overall, the ORR was 26.1% (12/46), with a median PFS of 5.0 (95% CI: 3.5-6.5) months (Figure 5F). Nine patients had an unmeasurable PFS1, and 21 of 37 (56.8%) patients achieved a PFS2/PFS1 ratio of ≥ 1.3.

To filter out responsive genomic variants, we next determined druggable alterations in patients who achieved both a responsive status (completed or partial response, CR or PR) and PFS2/PFS1 ratio ≥ 1.3 (Figure 5G). Our results showed that the MSI-H/dMMR status, *BRCA2* truncating mutations, *EGFR* activating mutations, *MET* amplifications and *FGFR2* fusions were promising targets to achieve satisfactory therapeutic efficacy. In the overall study population, the druggable targets of patients with a PFS2/PFS1 ratio ≥ 1.3 were largely in-PAT genes, highlighting the superiority of PATs with high-level evidence scoring by OncoKB and the ESCAT. Notably, 3 of 5 patients with truncated gBRCA2 achieved PR when treated with olaparib, which was consistent with previous reports that gBRCA2 may be a leading biomarker when utilizing poly ADP-ribose polymerase (PARP) inhibitors for pancreatobiliary tumors 41. More impressively, 6 patients who were defined as having MSI-H BTCs showed a high ORR (67%, 4/6) with anti-PD1 immunotherapy, confirming that the MSI-H or dMMR status is an encouraging biomarker for immune-checkpoint inhibitors. Overall, our data suggest that the personalized approach to manage precise targeted therapy is promising for patients with aggressive metastatic and refractory BTC. According to evidence-based recommendations from the ESCAT and OncoKB, the 19 PAT genes might facilitate further personalized selection for translational precision oncology in BTC patients.

## Discussion

In this study, a large cohort was examined to comprehensively investigate genomic alterations and translational precision oncology in patients with BTC, including genetic profiling, genomic alterations associated with etiological features and personalized anti-tumor therapies. Our results highlight extensive genomic diversity and heterogeneity among patients with BTC, with contributions according to potential etiology exposure, the anatomical subtype and clinicopathological characteristics. In addition, we preliminarily demonstrate that patients with refractory BTCs who have PATs can derive considerable benefit from receiving a matched therapy, initiating further prospective clinical trials guided by molecular profiling among this aggressive cancer.

Concerning the frequently mutated genes identified in our cohort, we observed a different spectrum of frequent mutations and altered signaling pathways than that reported for the Western BTC population. The mutational frequencies of *TP53, LRP1B, IDH1* and *FGFR* fusions varied between our cohort and the MSKCC CCA cohort 21. As hepatitis virus (HBV or HCV) infection is a major etiological feature of Chinese BTC patients 42, mutations in *TERT* and its promoter regions were more common among our BTC patients with a chronic hepatitis history. At the level of germline variants, our results highlight a potential role for trypsinogen-regulating genes, including *PRSS1* and *SPINK1*, in predisposition toward BTC, though the significantly increased risk for pancreatic adenocarcinoma associated with these two genes remains controversial 43,44. More importantly, we found AA signature mutations in 35.8% of ICC patients, suggesting that AA-containing herb exposure should be considered an etiological risk for ICC, particularly in China. Further investigation using experimental models or in clinical practice is needed to exclude the formal possibility that other chemicals unrelated to AA might also induce a mutational signature resembling the AA signature in biliary tumors. Consistent with observations in patients with urinary tract carcinomas, renal cell carcinoma and HCC 45,46, patients with ICC and a positive AA signature had a significantly higher TMB than those without this signature. In addition, we found a significantly high TMB in mutated *KMT2D* or *FAT3* BTCs. *KMT2D* is responsible for histone modification and chromatin remodeling, suggesting that disruption of the histone modification pathway is a contributing factor to an increase in TMB. Moreover, Salem *et al* found that microsatellite stable (MSS) colorectal carcinomas (CRCs) had a lower absolute incidence of *KMT2D* mutations than MSI-H CRCs 47, and *KMT2D* is a homologous recombination gene 48, implying that *KMT2D* mutations are associated with genomic instability. *FAT3* is a member of the family of human homologs to the Drosophila melanogaster transmembrane receptor for Hippo signaling and tumor-suppressor fat 49 and showed a significant association with TMB-high in small cell lung cancer 50. Thus, it is worthwhile to investigate whether patients with the AA signature harboring KMT2D and FAT3 mutations are responsive to ICB targeted PD1/PDL1.

Based on clinical survival prognoses, we provide three independent predictive genomic biomarkers, *TP53*, *KRAS* and chromosome 7q31.2, that have negative effects on DFS and OS in BTC patients. Both *TP53* and *KRAS* mutants have been demonstrated to be significantly associated with poor survival outcomes in several previous studies 24,51,52, but we did not observe the prognostic impacts of other genetic alterations, including *ARID1A*, *BAP1*, *CDKN2A/B*, *MUC17* and FGF pathway mutations 20,28. These discrepancies between our study and others are speculated to result from the heterogeneity in the baseline characteristics of the patient cohorts included.

Notably, we propose a subset of 19 genes as PATs in patients with advanced BTC, with assistance from evidence-based recommendations from OncoKB and the ESCAT. Due to the rigorous standards in defining PATs in our study and the shortage of approved biomarker-driven targeted therapies for BTC, the proportion (25.4%) of BTC patients harboring PATs was less than that in previous studies, which reported actionable alterations in 40-50% of patients 20,21,24. Nonetheless, our clinical practice of mutation-guided personalized therapies demonstrated an encouraging response rate among patients with refractory BTCs, particularly accentuating the scope of PATs, and further investigations of genome-driven oncology and personalized therapies among BTC patients should be performed. Nevertheless, the bottleneck of translational precision oncology in BTC patients is still the limited coverage of potent targets, which restricts the clinical utilization of precision oncology. For example, MSI-H/dMMR BTCs were identified in only approximately 1.5% of patients, and less than 1% of BTCs harbored *NTRK* fusions; *IDH1* mutations occurred in only 6% of patients with BTC in our cohort, and the proportion of *FGFR2/3* fusions was small (4%). Hence, more clinical investigations and proof-of-concept designed trials need to be implemented, perhaps involving combinational targeted treatment through multiple target inhibition or immunotherapy combined with chemotherapy. In addition, BGTs for BTC patients may be improved with more available targeted drugs that effectively inhibit genomic alterations such as *KRAS* and SWI/SNF subunit mutations, *CCND1* and *MET* amplifications, and *CDKN2A/2B* deletions. Promising PATs and therapeutic biomarkers for patients with advanced BTCs include cabozantinib for patients with mutated *MET* 53 and CDK4/6 inhibitors for those harboring *CCND1* and *CDKN2A/AB* mutations 54,55. Furthermore, the introduction of multiomics, including proteomics, transcriptomics and single-cell omics, into clinical translational treatments will enrich the scope of druggable targets in BTCs 56.

Limitations of our present study must be acknowledged. Our lack of matching transcriptomics and proteomics data leaves many of the novel observations uncorroborated in terms of expression. Although we provide a comprehensive landscape and clinical relevance of the genotypes of BTC, the introduction of multiomics into clinical translational treatments will promote the upgrade from genotypes to phenotypes in this aggressive malignancy. In addition, the rate of conversion from actual clinical treatment in the entire cohort was low; only 46 patients received PAT-matched targeted therapies. This might result from the limited coverage of PATs in BTC patients, the shortage of available molecular targeted drugs and insufficient clinical trials to provide patients with therapeutic opportunities.

In summary, we report the genomic mutational profiles of 803 BTC patients, and our work offers a feasible translational genome-driven precision oncology strategy involving the core parts of the mutational landscape, prognostic prediction and personalized antitumor treatments in BTC patients, inspiring the potential of precision oncology from evidence-based PATs for this rare but intractable disease. Our findings, while not definitive, suggest that our definition of a PAT is indeed a valid approach for identifying candidates for personalized treatments among patients with advanced BTCs.

## Supplementary Material

Supplementary methods, figures and tables.

## Figures and Tables

**Table 1:** Clinicopathological characteristics of the study population (n = 803)

Age (mean, IQR)	58.5 (52-66)
Sex (Male, n, %)	456 (56.8%)
**Histopathological Type (n, %)**	
Intrahepatic cholangiocarcinoma (ICC)	475 (59.2%)
Extrahepatic cholangiocarcinoma (ECC)	164 (20.4%)
Gallbladder cancer (GBC)	164 (20.4%)
**Sample Source (n, %)**	
Primary tumors	705 (87.8%)
Recurrent tumors	12 (1.5%)
Biopsy of metastatic sites	86 (10.7%)
**Clinical Stage (AJCC 7^th^, n, %)**	
I	134 (16.7%)
II	177 (22%)
III/IV	381 (47.4%)
Unknown	111 (13.9%)
**Lymphatic Metastasis (n, %)**	
N0	385 (47.9%)
N1	197 (24.6%)
Nx	221 (27.5%)
**HBV/HCV Infection (n, %)**	
Positive	214 (26.7%)
Negative	541 (67.4%)
Unknown	48 (5.9%)
**Liver Cirrhosis (n, %)**	
Positive	112 (13.9%)
Negative	367 (45.7%)
Unknown	324 (40.3%)
**Liver Fluke Infestation (n, %)**	
Etiologically confirmed	7 (1%)
Others	796 (99%)
**Biliary Stone Disease (n, %)**	
Positive	161 (20%)
Negative	292 (36.4%)
Unknown	350 (43.6%)

Note: AJCC: American Joint Committee on Cancer; HBV: hepatitis B virus; HCV: HBV: hepatitis C virus.

## Abbreviations

AA: aristolochic acid
BTC: biliary tract carcinoma
BGTs: biomarker guided treatments
CCA: cholangiocarcinoma
CNVs: copy-number variations
DDR: DNA damage repair
DFS: disease-free survival
dMMR: mismatch repair deficiency
ECC: extrahepatic cholangiocarcinoma
GBC: gallbladder cancer
HCC: hepatocellular carcinoma
HCCA: mixed hepato-cholangiocarcinoma
ICB: immune checkpoint blockade
ICC: intrahepatic cholangiocarcinoma
MSI: microsatellite instability
MTB: Molecular Tumor Board
NMF: non-negative matrix factorization
ORR: objective response rate
OS: overall survival
PARP: poly ADP-ribose polymerase
PATs: potentially actionable targets
PFS: progression-free survival
RTKs: receptor tyrosine kinases
SBSs: single-base substitutions
SMGs: significantly mutated genes
TMB: tumor mutation burden
TPS: targeted panel sequencing
WES: whole-exome sequencing

## References

[1] Benavides M, Antón A, Gallego J (2015). Biliary tract cancers: SEOM clinical guidelines. Clin Transl Oncol.

[2] Hundal R, Shaffer EA (2014). Gallbladder cancer: epidemiology and outcome. Clin Epidemiol.

[3] Bertuccio P, Malvezzi M, Carioli G (2019). Global trends in mortality from intrahepatic and extrahepatic cholangiocarcinoma. J Hepatol.

[4] Zhu AX, Hong TS, Hezel AF, Kooby DA (2010). Current management of gallbladder carcinoma. Oncologist.

[5] Marinelli I, Guido A, Fuccio L (2017). Clinical Target Volume in Biliary Carcinoma: A Systematic Review of Pathological Studies. Anticancer Res.

[6] Mavros MN, Economopoulos KP, Alexiou VG, Pawlik TM (2014). Treatment and Prognosis for Patients With Intrahepatic Cholangiocarcinoma: Systematic Review and Meta-analysis. JAMA Surg.

[7] Banales JM, Cardinale V, Carpino G (2016). Expert consensus document: Cholangiocarcinoma: current knowledge and future perspectives consensus statement from the European Network for the Study of Cholangiocarcinoma (ENS-CCA). Nat Rev Gastroenterol Hepatol.

[8] Dong L-Q, Shi Y, Ma L-J (2018). Spatial and temporal clonal evolution of intrahepatic cholangiocarcinoma. J Hepatol.

[9] Rizzo A, Ricci AD, Tavolari S, Brandi G (2020). Circulating Tumor DNA in Biliary Tract Cancer: Current Evidence and Future Perspectives. Cancer Genomics Proteomics.

[10] Massa A, Varamo C, Vita F (2020). Evolution of the Experimental Models of Cholangiocarcinoma. Cancers (Basel).

[11] Athauda A, Fong C, Lau DK (2020). Broadening the therapeutic horizon of advanced biliary tract cancer through molecular characterisation. Cancer Treat Rev.

[12] Zou S, Li J, Zhou H (2014). Mutational landscape of intrahepatic cholangiocarcinoma. Nat Commun.

[13] Cao J, Chen L, Li H (2019). An Accurate and Comprehensive Clinical Sequencing Assay for Cancer Targeted and Immunotherapies. Oncologist.

[14] Lin J, Shi J, Guo H (2019). Alterations in DNA Damage Repair Genes in Primary Liver Cancer. Clin Cancer Res.

[15] Chakravarty D, Gao J, Phillips S (2017). OncoKB: A Precision Oncology Knowledge Base. JCO Precision Oncology.

[16] Mateo J, Chakravarty D, Dienstmann R (2018). A framework to rank genomic alterations as targets for cancer precision medicine: the ESMO Scale for Clinical Actionability of molecular Targets (ESCAT). Ann Oncol.

[17] Suder A, Ang JE, Kyle F (2015). A phase I study of daily afatinib, an irreversible ErbB family blocker, in combination with weekly paclitaxel in patients with advanced solid tumours. Eur J Cancer.

[18] Schwartz LH, Litière S, de Vries E (2016). RECIST 1.1-Update and clarification: From the RECIST committee. Eur J Cancer.

[19] Lawrence MS, Stojanov P, Polak P (2013). Mutational heterogeneity in cancer and the search for new cancer-associated genes. Nature.

[20] Wardell CP, Fujita M, Yamada T (2018). Genomic characterization of biliary tract cancers identifies driver genes and predisposing mutations. J Hepatol.

[21] Lowery MA, Ptashkin R, Jordan E (2018). Comprehensive Molecular Profiling of Intrahepatic and Extrahepatic Cholangiocarcinomas: Potential Targets for Intervention. Clin Cancer Res.

[22] Mermel CH, Schumacher SE, Hill B, Meyerson ML, Beroukhim R, Getz G (2011). GISTIC2.0 facilitates sensitive and confident localization of the targets of focal somatic copy-number alteration in human cancers. Genome Biol.

[23] Sanchez-Vega F, Mina M, Armenia J (2018). Oncogenic Signaling Pathways in The Cancer Genome Atlas. Cell.

[24] Nakamura H, Arai Y, Totoki Y (2015). Genomic spectra of biliary tract cancer. Nat Genet.

[25] Chae H, Kim D, Yoo C (2019). Therapeutic relevance of targeted sequencing in management of patients with advanced biliary tract cancer: DNA damage repair gene mutations as a predictive biomarker. Eur J Cancer.

[26] Tate JG, Bamford S, Jubb HC (2019). COSMIC: the Catalogue of Somatic Mutations In Cancer. Nucleic Acids Res.

[27] Ng AWT, Poon SL, Huang MN (2017). Aristolochic acids and their derivatives are widely implicated in liver cancers in Taiwan and throughout Asia. Sci Transl Med.

[28] Javle M, Bekaii-Saab T, Jain A (2016). Biliary cancer: Utility of next-generation sequencing for clinical management. Cancer.

[29] Dai R, Li J, Fu J (2012). The tyrosine kinase c-Met contributes to the pro-tumorigenic function of the p38 kinase in human bile duct cholangiocarcinoma cells. J Biol Chem.

[30] Smith LM, Nesterova A, Ryan MC (2008). CD133/prominin-1 is a potential therapeutic target for antibody-drug conjugates in hepatocellular and gastric cancers. Br J Cancer.

[31] Sirica AE, Dumur CI, Campbell DJW, Almenara JA, Ogunwobi OO, Dewitt JL (2009). Intrahepatic cholangiocarcinoma progression: prognostic factors and basic mechanisms. Clin Gastroenterol Hepatol.

[32] Valle JW, Lamarca A, Goyal L, Barriuso J, Zhu AX (2017). New Horizons for Precision Medicine in Biliary Tract Cancers. Cancer Discov.

[33] Su S-C, Lin C-W, Liu Y-F (2017). Exome Sequencing of Oral Squamous Cell Carcinoma Reveals Molecular Subgroups and Novel Therapeutic Opportunities. Theranostics.

[34] Rosell R, Moran T, Queralt C (2009). Screening for epidermal growth factor receptor mutations in lung cancer. N Engl J Med.

[35] Drilon A, Laetsch TW, Kummar S (2018). Efficacy of Larotrectinib in TRK Fusion-Positive Cancers in Adults and Children. N Engl J Med.

[36] Mandal R, Samstein RM, Lee K-W (2019). Genetic diversity of tumors with mismatch repair deficiency influences anti-PD-1 immunotherapy response. Science.

[37] Le DT, Uram JN, Wang H (2015). PD-1 Blockade in Tumors with Mismatch-Repair Deficiency. N Engl J Med.

[38] Nowak JA, Yurgelun MB, Bruce JL (2017). Detection of Mismatch Repair Deficiency and Microsatellite Instability in Colorectal Adenocarcinoma by Targeted Next-Generation Sequencing. J Mol Diagn.

[39] Von Hoff DD, Stephenson JJ Jr, Rosen P (2010). Pilot study using molecular profiling of patients' tumors to find potential targets and select treatments for their refractory cancers. J Clin Oncol.

[40] Sicklick JK, Kato S, Okamura R (2019). Molecular profiling of cancer patients enables personalized combination therapy: the I-PREDICT study. Nat Med.

[41] Golan T, Hammel P, Reni M (2019). Maintenance Olaparib for Germline *BRCA* -Mutated Metastatic Pancreatic Cancer. N Engl J Med.

[42] Zhang H, Zhu B, Zhang H, Liang J, Zeng W (2016). HBV Infection Status and the Risk of Cholangiocarcinoma in Asia: A Meta-Analysis. Biomed Res Int. 2016/11/23 ed.

[43] Schubert S, Traub F, Brakensiek K (2014). CFTR, SPINK1, PRSS1, and CTRC mutations are not associated with pancreatic cancer in German patients. Pancreas.

[44] Zhan W, Shelton CA, Greer PJ, Brand RE, Whitcomb DC (2018). Germline Variants and Risk for Pancreatic Cancer: A Systematic Review and Emerging Concepts. Pancreas.

[45] Poon SL, Huang MN, Choo Y (2015). Mutation signatures implicate aristolochic acid in bladder cancer development. Genome Med.

[46] Rosenquist TA, Grollman AP (2016). Mutational signature of aristolochic acid: Clue to the recognition of a global disease. DNA Repair (Amst).

[47] Salem ME, Battaglin F, Goldberg RM (2020). Molecular Analyses of Left- and Right-Sided Tumors in Adolescents and Young Adults with Colorectal Cancer. Oncologist.

[48] Heeke AL, Xiu J, Elliott A (2020). Actionable co-alterations in breast tumors with pathogenic mutations in the homologous recombination DNA damage repair pathway. Breast Cancer Res Treat.

[49] Katoh M (2012). Function and cancer genomics of FAT family genes (review). Int J Oncol.

[50] Kachroo S, Shao C, Desai K, He J, Jin F, Sen S (2021). Association of clinico-genomic characteristics with tumor mutational burden in small cell lung cancer patients. Future Oncol.

[51] Chae H (2019). Therapeutic relevance of targeted sequencing in management of patients with advanced biliary tract cancer: DNA damage repair gene mutations as a predictive biomarker. Eur J Cancer.

[52] Jusakul A, Cutcutache I, Yong CH (2017). Whole-Genome and Epigenomic Landscapes of Etiologically Distinct Subtypes of Cholangiocarcinoma. Cancer Discov.

[53] Goyal L, Zheng H, Yurgelun MB (2017). A phase 2 and biomarker study of cabozantinib in patients with advanced cholangiocarcinoma. Cancer.

[54] Song X, Liu X, Wang H (2019). Combined CDK4/6 and Pan-mTOR Inhibition Is Synergistic Against Intrahepatic Cholangiocarcinoma. Clin Cancer Res.

[55] Sittithumcharee G, Suppramote O, Vaeteewoottacharn K (2019). Dependency of Cholangiocarcinoma on Cyclin D-Dependent Kinase Activity. Hepatology.

[56] Olivier M, Asmis R, Hawkins GA, Howard TD, Cox LA (2019). The Need for Multi-Omics Biomarker Signatures in Precision Medicine. Int J Mol Sci.

