# Supplementary material for: Mutational spectrum and precision oncology for biliary tract carcinoma
Source: Theranostics. 2021 Mar 4;11(10):4585–98. doi: 10.7150/thno.56539 (PMC7978308; doi:10.7150/thno.56539)
Supplement: Supplementary file 1 — Supplementary methods, figures and tables. [file thnov11p4585s1.pdf]

Supplementary Material

Supplementary Methods

Sample preparation and genomic profiling

All tumor tissues (fresh-frozen, FF; or formalin fixed paraffin-embedded, FFPE) obtained from participants were reviewed by two independent pathologists before sample disposal to confirm the pathologic diagnoses of biliary tract carcinomas (BTCs). Serial sections on tissue slides was performed to evaluate tumor content and percentage. Only samples with estimated tumor purity >20% on histopathologic assessment were further subjected to genomic profiling. Tumor purity assessed in our study is the proportion of cancer cells in the tissues. Tumor tissue section from each patient was hematoxylin-eosin (HE) stained, and the proportion of cancer cells was then estimated by pathologists. For sections with tumor purity over 20%, bulk tumor tissues were subjected to extract DNA and sequencing. For those tumor tissues with purity less than 20%, micro-resected tumor section was performed to remove non-cancerous cells and stroma to improve tumor purity. DNAs were extracted from paired tumor and blood samples using DNA Extraction Kits (Qiagen). DNAs yield and quality were quantified using the Qubit 3.0 (Invitrogen) and NanoDrop 2000 (Thermo Scientific) and the integrity was further visually inspected by agarose gel electrophoresis.

Genomic alterations profiling was performed in 2 major next-generation sequencing (NGS) platform, including whole-exome sequencing (WES) and a custom hybridization capture-based targeted panel sequencing (TPS)[1] provided by OrigiMed (Cancer Sequencing YS panel, CSYS, Shanghai, China). WES libraries were prepared from DNA extracted from tumor and normal samples using the Agilent's SureSelect Human All Exon V6 Kit (Agilent Technologies Inc.), and the sequencing was conducted using the Illumina Hiseq *XTEN* platform for paired-end 150bp reads. For CSYS platform, 50–250ng DNA was used to construct libraries, targeted genomic regions were hybrid-captured, and sequence reads were generated using Illumina (San Diego, CA) instruments with the referred procedures followed the steps described in Frampton et al,[2].

WES data analysis

Somatic mutation calling and filtering

Paired-end reads from paired tumor and normal-control samples were aligned to the human reference genome (GRCh37) using the Burrows-Wheeler Aligner (BWA) (version 0.7.10)[3]. Somatic mutation calling was processed using GATK (version 3.7)[4] followed the GATK Best Practices by using its Mutect2 function with duplicate and unmapped reads removed. A stringent downstream filter comprised of the following criteria was used to obtained high quality somatic variants: Variant Allele Fraction (VAF) ≥5% with >5 variant supporting reads in the tumor sample, and VAF <1% in the normal-control samples; a minimum of 5X coverage; strand bias ≤0.95. Variants with PASS tag was retained and annotated by ANNOVAR (version: 2018-04-16)[5] using multiple specific databases (e.g. ExAC03, dbNSFP33a, ClinVar-20170130, avsnp150, Cosmic70). To make a possible comparison with target sequencing, non-coding variants in the introns, upstream or downstream of coding regions in 3'- and 5'-UTRs, and in intergenic regions were discarded.

Tumor mutation burden (TMB)

TMB analysis of WES data was performed followed with Chalmers et al,[6]. Briefly, all base substitutions and indels identified by WES in the coding region of genes as non-synonymous alterations, were all counted, except alterations listed as known somatic alterations in COSMIC or truncations in tumor suppressor genes were not counted. Known germline alterations in dbSNP or occurring with two or more counts in the ExAC database were not counted. TMB was calculated as the total number of mutation counts divided by the size of the coding region of the whole-exome territory.

Analysis of significantly mutated genes

MutSigCV (version 1.41)[7] was performed on the somatic variations of WES data to identify the significantly mutated genes that were mutated more often than expected by chance. MutsigCV's q-value < 0.1 was taken as threshold to detect significantly mutated genes. Populational frequencies of mutated genes was calculated by integrating WES and TPS samples together. In addition, mutual co-occurrence and exclusive event analysis on top 20 significantly mutated genes was performed by "somaticInteractions" function from Maftools (version 2.2.10)[8], using pair-wise Fisher's Exact test to detect such significant pair of genes. Then "oncostrip" function was used to visualize mutually exclusive set of genes for *TP53* and *KRAS*.

Somatic copy-number alterations (SCNAs) calling

According to the Agilent Target BED file, copy number for each segment was first analyzed by Control-FREEC (version 11.4)[9] and segments with same copy number were merged together to

generate a unique segment file. GISTIC2 (version 2.0.23)[10] was then used to calculate significant copy number gains and losses at a confidence level 0.99. Focal amplifications and deletions were selected using a threshold of GISTIC's q-value <= 0.01.

Identifications and annotations of germline variants

Germline variants were identified using GATK (version 3.7) and its HaplotypeCaller function in single-sample mode with duplicate and unmapped reads removed. Variants were then filtered using VariantFiltration function of GATK with parameters "DP <4 || QD <2.0 || FS >60.0 || MQ <40.0 || MQRankSum <-12.5 || ReadPosRankSum <-8.0" and "DP <4 || QD <2.0 || FS >200.0 || ReadPosRankSum <-20.0" for SNVs and indels, respectively. Furthermore, to exclude potential tumor-in-normal contamination, germline mutations with VAF in normal-control sample <30%, and co-localizing with any known somatic mutation, which germline variants located at the same protein residue as recurrent (n>=3) somatic mutations in TCGA MC3 MAF[11] were further filtered. After the filtration, variants annotations were firstly obtained by ANNOVAR (version: 2018-04-16), and the evidence for pathogenic variations mainly derived from the public databases, including the Human Gene Mutation Database, Clinvar, Sorting Intolerant From Tolerant (SIFT), and the standard from American College of Medical Genetics (ACMG). A further manual review was performed to reduce the false positive variants.

Determination of MSI status

Microsatellites in the human reference genome (GRCh37) was identified using RepeatFinder (version 1.0)[12] with default parameters. To detect the microsatellite instability status, MANTIS (version 1.0.4)[13] was used with modified parameters "-mrq 20.0 -mlq 25.0 -mlc 20 -mrr 1" to fit for the whole-exome data, taken both tumor and non-tumor bam files as inputs. Any Step-Wise Difference (DIF) value calculated by MANTIS greater than or equal to 0.4 is treated as unstable. For 160 BTCs samples, only one patient was classified as MSI-H tumor, and MSI-PCR testing as the gold standard method was also performed to confirm the MSI-H status in tumor tissues.

TPS analysis for detecting somatic alterations

The raw sequencing data underwent stringent quality control of read depth and ratio of target capture. Data quality was inspected and controlled by examining sequencing coverage and uniformity, and a suite of customized bioinformatics pipelines was applied for discovery of SNVs,

short and long indels, SCNAs and gene rearrangements. The bioinformatics pipelines of CSTS have been described in our previous reports[1].

TMB was calculated from TPS data for each sample by counting the number of somatic mutations, including coding SNVs and indels, per megabase (Mb) of the sequence examined. Known somatic mutations in the Catalog of Somatic Mutations in Cancer (COSMIC) and known germline polymorphisms in the U.S. National Center for Biotechnology Information's Single Nucleotide Polymorphism Database (dbSNP) were not counted[6].

TPS panel designed 572 microsatellite loci (MSLs) targeted region as candidate MSI markers, which were done as previously described[13,14]. Using custom predictive model analyzed by a random forest algorithm combined with an orthogonal validation of PCR testing or IHC method as the gold standard, MSI status for each tumor sample could be judged. PCR products were analyzed by Sanger sequencing using an ABI 3730 Genetic Analyzer (Thermo Fisher Scientific, Waltham, MA).

Finally, all the detected mutations were compared with our in-house database of genomic changes specific to clinical annotation. A manual review process was performed to ensure no false positives or mistakes in clinical annotations, including complex variant curation and clinical relevance inference based on our in-house database. Based on the annotated mutations, a succinct report with relevant references to literature and clinical trials was generated.

Identification of hypermutated tumors

To determine a threshold of hypermutation in BTCs, a segmented linear regression analysis or ‘‘broken-stick analysis’’ was performed on the mutation burdens from WES cohort and TPS cohort, which refers to method from Campbell's colleagues[15]. Briefly, using the R package "Segmented", an iterative process was used to determine segment breakpoints at which a statistically significant change in the slope of adjacent regression lines occurred[16]. Consequently, the threshold was determined to 9.36 Mut/Mb in WES and 16.1 Mut/Mb in TPS, respectively.

Mutational landscape construction

A mutational case was defined as sample with no less than one non-silent mutation in coding region or SCNAs detected in individual genes. We determine 68 potential driver mutation genes of BTCs (Supplementary Table S4) as following: (1) genes that simultaneously captured by both WES and

CSYS platform; (2) significantly mutated genes identified by MutSigSV from our WES data; (3) recurrent mutations summarized from three previous studies which investigated the genomic landscape of BTCs, including Nakamura et al[17], Jusakul et al[18], and Montal et al[19]; (4) Tier1 cancer-related genes presented in COSMIC Cancer Gene Census database (version 91, https://cancer.sanger.ac.uk/census). The mutational landscape was constructed according to the populational frequencies (>5%) with a slight modification and integrated the mutations of *IDH2* and *MUC4* which showed significantly different rate as comparing with previous cohorts. The mutational spectrum was visualized by Maftools (version 2.2.10).

Mutations enriched for oncogenic pathways

Recurrent alterations of BTCs presented in Figure 1C could be roughly classified into four major functional families, that are cell cycle/cyclins genes, receptor tyrosine kinases genes, chromatin-modifying genes and others. Furthermore, ten curated pathways with oncogene and tumor suppressor gene information were retrieved from a study of TCGA program[20]. The mutational map of these 10 pathways was constructed according to the paper with a slight modification by integrating the description of percentage of mutational cases for each subtype of BTCs (Supplementary Figure S5).

Determining the scope of DNA damage response (DDR) genes

The scope and definition of DDR genes were collected from Human DNA Repair Genes (https://www.mdanderson.org/documents/Labs/Wood-Laboratory/human-dna-repair-genes.html). As most DDR genes have not yet been determined to have oncogenic or pathogenic effects in BTCs, we narrowed the scope of DDR genes into 47 coding genes that could be captured by both WES and CSYS platform (Supplementary Table S6), listing as: *TP53, MUTYH, APEX1, PARP1, PARP2, PARP3, MGMT, MSH2, MSH6, MLH1, PMS2, ERCC1, RAD51, RAD51B, RAD51D, XRCC2, XRCC3, RAD52, RAD54L, RAD54B, BRCA1, RAD50, MRE11A, NBN, FANCA, FANCC, BRCA2, FANCD2, FANCE, FANCF, FANCG, BRIP1, FANCL, FANCM, PALB2, RAD51C, PRKDC, POLB, POLD1, POLE, FEN1, BLM, ATM, RECQL, ATR, CHEK1* and *CHEK2*.

Mutational signature analysis and identification of aristolochic acid (AA) signature

Mutational signatures were inferred for ICC samples performed WES from our cohort. Firstly, we excluded 4 hypermutated ICCs as considering hypermutated tumors has been demonstrated to have significantly different substitution patterns. Next, the 96 mutational subtypes generated from somatic SNVs based on 6 single-base substitutions (SBSs, C>A, C>G, C>T, T>A, T>C and T>G) in 16 possible combinations of neighboring bases for each SBS were used as input data to infer their contributions to observed mutations. We implemented a non-negative matrix factorization (NMF) approach[21] to decipher the mutational signatures (Signature A, B, and C) of all samples with the number based on the signature stability and Frobenius reconstruction errors. Finally, three mutational signatures (A, B, and C) were linked to the known COSMIC cancer signatures (https://cancer.sanger.ac.uk/cosmic/signatures) with comparing cosine similarities to infer their exposure contributions.

Mutational signature activity (mSigAct, v2.0.0-branch) was applied to assess presence of the AA signature in all ICC samples. The mSigAct software[22] provided a signature presence test to infer whether the observed mutation spectrum could be better explained with a contribution from the AA mutational signature (COSMIC signature 22) than without it and compared them with a likelihood ratio test. The null hypothesis was that the mutational counts were generated without the AA signature and the alternative hypothesis was that they were from the AA signature. The test was then carried out by a standard likelihood ratio test on these two hypotheses. The mSigAct revealed strong evidence of AA exposure with FDR < 0.05.

Immunohistochemistry for expressions of MMR genes

Whole sections from available FFPE of BTCs tissues were prepared into to 4 or 5 μm-thick on glass slides. For samples identified as MSI-H tumors, the primary antibodies used were anti-MSH2 (Invitrogen, clone 25D12), anti-MSH6 (Ventana, clone 44), anti-MLH1 (Ventana, clone M1) and anti-PMS2 (Agilent Dako, Clone EP51). The secondary antibodies were added to all sections, including the negative control slides. During interpretation, loss of nuclear staining in the tumor cells with positively stained internal control was counted as an abnormal result for 4 MMR genes.

References

1. Cao J, Chen L, Li H, et al. An Accurate and Comprehensive Clinical Sequencing Assay for Cancer Targeted and Immunotherapies. Oncologist. 2019; 24: e1294–302.

2. Frampton GM, Fichtenholtz A, Otto GA, et al. Development and validation of a clinical cancer genomic profiling test based on massively parallel DNA sequencing. Nat Biotechnol. 2013; 31: 1023–31.

3. Li H, Durbin R. Fast and accurate short read alignment with Burrows-Wheeler transform. Bioinformatics. 2009; 25: 1754–60.

4. Cibulskis K, Lawrence MS, Carter SL, et al. Sensitive detection of somatic point mutations in impure and heterogeneous cancer samples. Nat Biotechnol. 2013; 31: 213–9.

5. Wang K, Li M, Hakonarson H. ANNOVAR: functional annotation of genetic variants from high-throughput sequencing data. Nucleic Acids Res. 2010; 38: e164–e164.

6. Chalmers ZR, Connelly CF, Fabrizio D, et al. Analysis of 100,000 human cancer genomes reveals the landscape of tumor mutational burden. Genome Med. 2017; 9: 34–34.

7. Lawrence MS, Stojanov P, Polak P, et al. Mutational heterogeneity in cancer and the search for new cancer-associated genes. Nature. 2013; 499: 214–8.

8. Mayakonda A, Lin D-C, Assenov Y, Plass C, Koeffler HP. Maftools: efficient and comprehensive analysis of somatic variants in cancer. Genome Res. 2018; 28: 1747–56.

9. Boeva V, Popova T, Bleakley K, et al. Control-FREEC: a tool for assessing copy number and allelic content using next-generation sequencing data. Bioinformatics. 2012; 28: 423–5.

10. Mermel CH, Schumacher SE, Hill B, Meyerson ML, Beroukhim R, Getz G. GISTIC2.0 facilitates sensitive and confident localization of the targets of focal somatic copy-number alteration in human cancers. Genome Biol. 2011; 12: R41–R41.

11. Huang K-L, Mashl RJ, Wu Y, et al. Pathogenic Germline Variants in 10,389 Adult Cancers. Cell. 2018; 173: 355-370.e14.

12. Volfovsky N, Haas BJ, Salzberg SL. A clustering method for repeat analysis in DNA sequences. Genome Biol. 2001; 2: RESEARCH0027–RESEARCH0027.

13. Kautto EA, Bonneville R, Miya J, et al. Performance evaluation for rapid detection of pan-cancer microsatellite instability with MANTIS. Oncotarget. 2017; 8: 7452–63.

14. Hause RJ, Pritchard CC, Shendure J, Salipante SJ. Classification and characterization of microsatellite instability across 18 cancer types. Nat Med. 2016; 22: 1342–50.

15. Campbell BB, Light N, Fabrizio D, et al. Comprehensive Analysis of Hypermutation in Human Cancer. Cell [Internet]. 2017; 171. Available at: https://pubmed.ncbi.nlm.nih.gov/29056344

16. Muggeo VMR. Interval estimation for the breakpoint in segmented regression: a smoothed score-based approach. Australian & New Zealand Journal of Statistics. 2017; 59: 311–22.

17. Nakamura H, Arai Y, Totoki Y, et al. Genomic spectra of biliary tract cancer. Nat Genet. 2015; 47: 1003–10.

18. Jusakul A, Cutcutache I, Yong CH, et al. Whole-Genome and Epigenomic Landscapes of Etiologically Distinct Subtypes of Cholangiocarcinoma. Cancer Discov. 2017; 7: 1116–35.

19. Montal R, Sia D, Montironi C, et al. Molecular classification and therapeutic targets in extrahepatic cholangiocarcinoma. Journal of Hepatology. 2020; S0168827820301665.

20. Sanchez-Vega F. Oncogenic Signaling Pathways in The Cancer Genome Atlas. : 28.

21. Gaujoux R, Seoighe C. A flexible R package for nonnegative matrix factorization. BMC Bioinformatics. 2010; 11: 367.

22. Ng AWT, Poon SL, Huang MN, et al. Aristolochic acids and their derivatives are widely implicated in liver cancers in Taiwan and throughout Asia. Sci Transl Med [Internet]. 2017; 9. Available at: https://pubmed.ncbi.nlm.nih.gov/29046434

Supplementary Figures

Figure S1. Correlation analysis of alterations in cocaptured genes between whole-exome sequencing (WES) and targeted panel sequencing (TPS). (A) Linear correlation between the variant allele fractions of cocaptured somatic single-nucleotide variants (SNVs, blue line) and indels (dark line) derived from WES and those derived from the TPS panel. (B) Identification of copy-number variants (CNVs) in individual genes from WES and TPS. The dashed line indicates the diploid copy-number (normal), and the vertical axis represents the number of copy-number gains (red) or copy-number losses (blue).

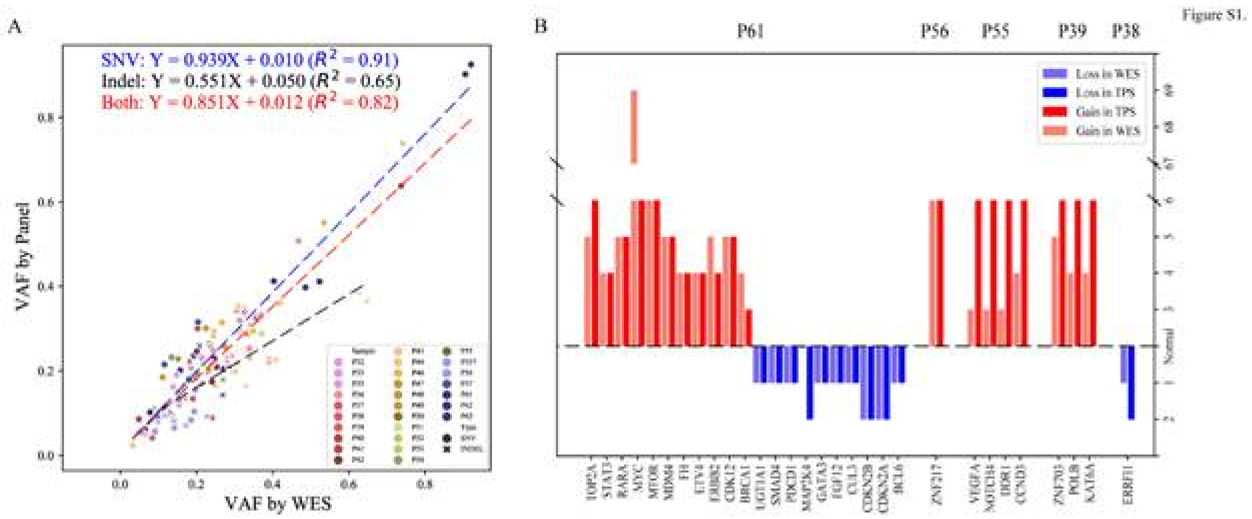

Figure S2. Distribution of somatic tumor mutation burden (TMB) and differences among the three subtypes of biliary tract cancer. (A) Distribution of TMB assessed by WES (top) or TPS (bottom) for each patient highlights the hypermutated group. Hypermutated cases are separated on the left, and the most drastic change point in the mutation rate (vertical dashed line) was chosen as the cutoff to discriminate hypermutated cases from non-hypermutated cases. (B) Linear correlation between TMB evaluated from WES and that evaluated from TPS. (C-D) Landscape of TMB across the major tumor types in WES data (C) or TPS data (D). The median level of TMB for each tumor type and in our cohort is highlighted.

Figure S2.

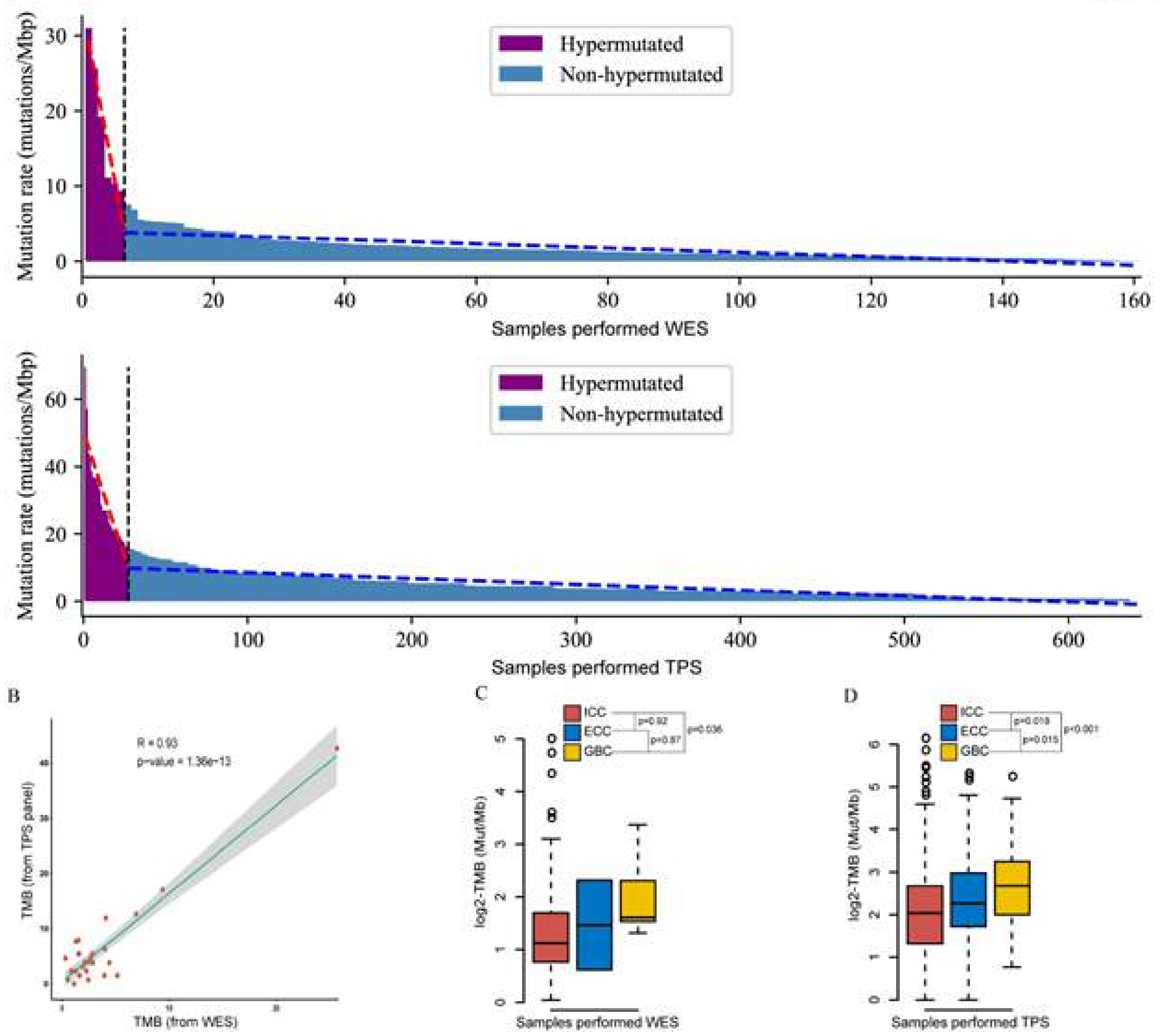

Figure S3. Somatic interactions of cooccurring or exclusively occurring mutations among the top-20 significantly mutated genes in 803 BTC patients. (A) Pairwise Fisher's exact tests were performed to detect significant pairs of genes. The cooccurring relationships are colored by a green box, while the exclusively occurring mutations are colored by a pink box. (B) *TP53* mutations are exclusive to alterations in *ARID1A*, *MUC4*, *IDH1* and *EPHA2*. (C) *KRAS* mutations are exclusively associated with alterations in *LRP1B*, *TERT*, *PIK3CA*, *IDH1* and *EPHA2*.

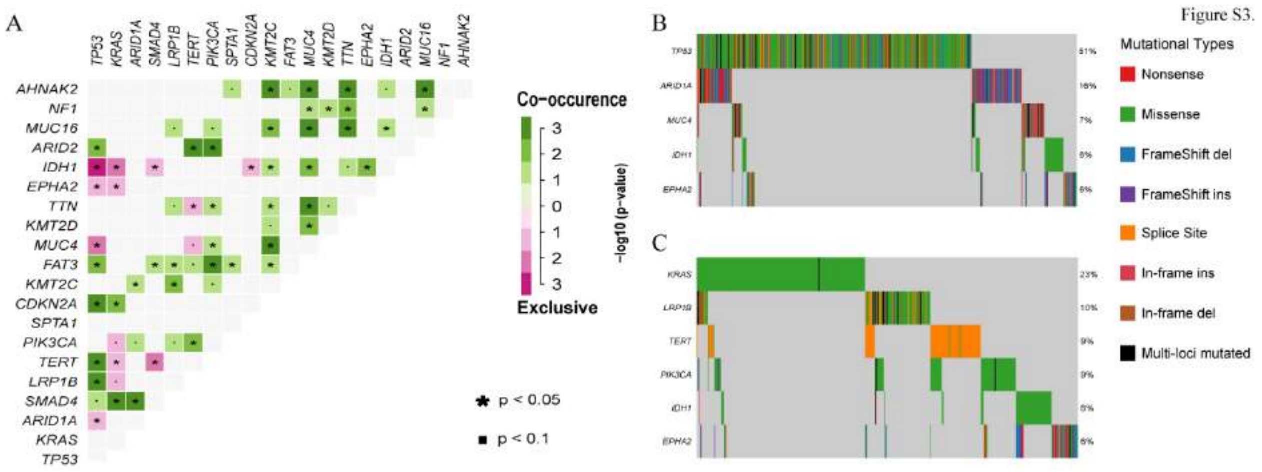

Figure S4. Integration of somatic mutations and SCNAs to decipher the mutational landscape of BTC patients. Pathway components and interactions in the ten selected oncogenic pathways are plotted. Somatic alteration (mutation and CNV) spectra of components in these pathways among the three subtypes (ICC, ECC, and GBC). The numbers of altered samples in each subtype are labeled in the boxes, and the color intensity indicates the average frequency of the alteration in each subtype. The numbers of patients harboring altered pathways are compiled in the top left corner of the plot.

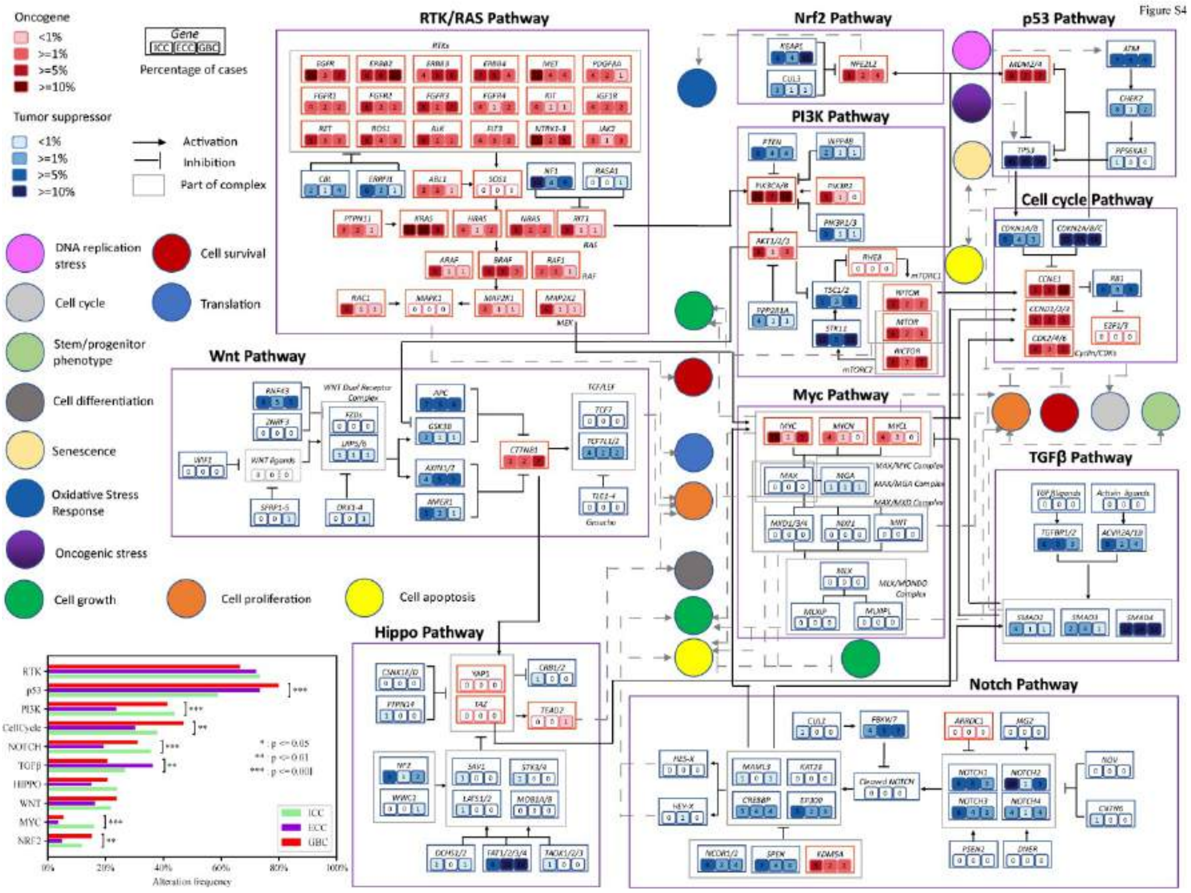

Supplementary Tables

Table S1. Detailed clinicopathological features of 803 BTC patients. (*status: 1-relapse/death; 0-censored)

Sample_ID	Tumor_Type	Age	Sex	Seq_Method	AJCC_Stage	Tissue_Source	Pathology_Differentiation	HBV/HCV_Infection	N_Stage	M_Stage	Vascular_Invasion	CA19-9>200	RFS_status	RFS	OS_Status	OS
SH_01	ICC	61	Male	WES	II	primary tumor	moderate	Negative	N0	M0	negative	negative	1	13	0	51
SH_02	ICC	44	Female	WES	II	primary tumor	moderate	Negative	N0	M0	positive	negative	1	1	1	11
SH_03	ICC	59	Female	WES	III+IV	primary tumor	moderate	Negative	N0	M0	positive	negative	1	5	1	11
SH_05	ICC	43	Female	WES	II	primary tumor	moderate	Positive	N0	M0	negative	negative	0	51	0	51
SH_06	ICC	48	Male	WES	I	primary tumor	moderate	Positive	N0	M0	negative	negative	0	51	0	51
SH_07	ICC	61	Female	WES	I	primary tumor	moderate	Negative	N0	M0	negative	positive	1	1	1	23
SH_08	ICC	45	Male	WES	III+IV	primary tumor	moderate	Positive	N0	M0	negative	NA	0	49	0	49
SH_09	ICC	62	Male	WES	II	primary tumor	moderate	Positive	N0	M0	negative	negative	1	2	1	18
SH_10	ICC	46	Male	WES	III+IV	primary tumor	moderate	Positive	N1	M0	negative	negative	1	0	1	38
SH_100	ICC	38	Female	WES	II	primary tumor	moderate	Positive	N0	M0	negative	negative	1	3	0	34
SH_101	ICC	54	Male	WES	I	primary tumor	moderate	Negative	N0	M0	negative	negative	1	9	0	34
SH_107	ICC	65	Male	WES	II	primary tumor	moderate	Negative	N0	M0	negative	negative	1	5	1	21
SH_108	ICC	57	Female	WES	II	primary tumor	moderate	Negative	N0	M0	negative	positive	1	2	1	8
SH_110	ICC	51	Male	WES	II	primary tumor	moderate	Positive	N0	M0	positive	negative	1	NA	1	4
SH_111	ICC	85	Male	WES	I	primary tumor	moderate	Negative	N0	M0	negative	positive	1	6	1	10
SH_113	ICC	40	Male	WES	I	primary tumor	moderate	Positive	N0	M0	negative	negative	1	12	1	31
SH_115	ICC	51	Female	WES	II	primary tumor	moderate	Negative	N0	M0	negative	positive	1	2	1	5
SH_118	ICC	43	Male	WES	I	primary tumor	moderate	Positive	N0	M0	negative	negative	1	6	1	16
SH_119	ICC	44	Male	WES	I	primary tumor	moderate	Positive	N0	M0	negative	negative	0	32	0	32
SH_120	ICC	51	Male	WES	III+IV	primary tumor	moderate	Positive	N0	M1	negative	positive	1	8	1	22
SH_121	ICC	48	Male	WES	II	primary tumor	moderate	Positive	N0	M0	positive	negative	1	9	1	15
SH_122	ICC	49	Male	WES	I	primary tumor	moderate	Negative	N0	M0	negative	negative	0	32	0	32
SH_123	ICC	64	Female	WES	III+IV	primary tumor	moderate	Negative	N0	M0	negative	negative	1	3	1	9
SH_125	ICC	35	Female	WES	II	primary tumor	moderate	Positive	N0	M0	positive	negative	1	6	1	14
SH_127	ICC	40	Male	WES	I	primary tumor	moderate	Positive	N0	M0	negative	negative	NA	NA	1	2
SH_128	ICC	63	Male	WES	I	primary tumor	poor	Positive	N0	M0	negative	negative	1	4	1	7
SH_129	ICC	68	Female	WES	I	primary tumor	moderate	Negative	N0	M0	negative	positive	1	1	0	42
SH_13	ICC	47	Male	WES	I	primary tumor	moderate	Positive	N0	M0	negative	negative	NA	NA	0	50
SH_130	ICC	54	Male	WES	III+IV	primary tumor	poor	Positive	N1	M0	negative	negative	NA	NA	1	7
SH_131	ICC	48	Male	WES	II	primary tumor	moderate	Positive	N0	M0	positive	negative	1	1	1	3
SH_132	ICC	70	Female	WES	I	primary tumor	moderate	Positive	N0	M0	negative	negative	NA	NA	1	1
SH_133	ICC	66	Male	WES	III+IV	primary tumor	moderate	Negative	N1	M0	negative	negative	1	0	1	26
SH_134	ICC	59	Female	WES	I	primary tumor	moderate	Negative	N0	M0	negative	positive	1	10	1	12
SH_135	ICC	68	Male	WES	I	primary tumor	moderate	Negative	N0	M0	negative	negative	NA	NA	1	3
SH_137	ICC	58	Female	WES	II	primary tumor	poor	Negative	N0	M0	negative	positive	NA	NA	1	4
SH_139	ICC	65	Male	WES	II	primary tumor	moderate	Negative	N0	M0	negative	negative	1	3	1	9
SH_140	ICC	59	Female	WES	III+IV	primary tumor	moderate	Positive	N1	M0	negative	negative	1	9	1	11
SH_141	ICC	62	Male	WES	III+IV	primary tumor	moderate	Negative	N1	M0	positive	negative	1	2	1	9
SH_142	ICC	52	Male	WES	I	primary tumor	moderate	Positive	N0	M0	negative	negative	NA	NA	1	8
SH_143	ICC	54	Male	WES	II	primary tumor	moderate	Positive	N0	M0	positive	negative	1	2	1	5
SH_144	ICC	62	Male	WES	I	primary tumor	moderate	Negative	N0	M0	negative	negative	NA	NA	1	18
SH_146	ICC	60	Male	WES	I	primary tumor	moderate	Negative	N0	M0	negative	positive	1	1	1	6
SH_147	ICC	48	Male	WES	II	primary tumor	poor	Positive	N0	M0	positive	negative	1	1	0	28
SH_16	ICC	63	Female	WES	II	primary tumor	moderate	Positive	N0	M0	negative	negative	NA	NA	1	5
SH_17	ICC	55	Male	WES	III+IV	primary tumor	moderate	Negative	N1	M1	negative	positive	1	3	1	6
SH_18	ICC	50	Male	WES	I	primary tumor	moderate	Positive	N0	M0	negative	negative	1	1	1	4
SH_19	ICC	56	Female	WES	I	primary tumor	well	Positive	N0	M0	negative	NA	NA	NA	0	49
SH_20	ICC	44	Male	WES	I	primary tumor	moderate	Positive	N0	M0	negative	negative	NA	NA	1	16
SH_24	ICC	55	Female	WES	II	primary tumor	moderate	Negative	N0	M0	positive	positive	1	3	1	6
SH_25	ICC	52	Male	WES	I	primary tumor	moderate	Positive	N0	M0	negative	negative	0	51	0	51
SH_26	ICC	49	Male	WES	II	primary tumor	moderate	Positive	N0	M0	positive	negative	1	27	1	33
SH_28	ICC	48	Male	WES	III+IV	primary tumor	moderate	Positive	N0	M1	positive	negative	NA	NA	1	1
SH_29	ICC	74	Female	WES	III+IV	primary tumor	moderate	Negative	N1	M0	negative	positive	1	2	1	4
SH_33	ICC	38	Female	WES	I	primary tumor	moderate	Negative	N0	M0	negative	positive	1	3	1	5
SH_34	ICC	52	Male	WES	I	primary tumor	moderate	Negative	N0	M0	negative	positive	1	5	1	6
SH_35	ICC	72	Male	WES	I	primary tumor	moderate	Positive	N0	M0	negative	negative	1	5	1	28
SH_41	ICC	36	Male	WES	I	primary tumor	moderate	Positive	N0	M0	negative	negative	1	15	1	18
SH_42	ICC	43	Female	WES	II	primary tumor	moderate	Positive	N0	M0	positive	negative	1	32	0	48
SH_44	ICC	73	Female	WES	II	primary tumor	moderate	Negative	N0	M0	positive	negative	1	NA	1	0
SH_46	ICC	52	Female	WES	III+IV	primary tumor	moderate	Negative	N1	M0	negative	negative	1	4	1	21
SH_48	ICC	56	Male	WES	I	primary tumor	moderate	Positive	N0	M0	negative	negative	0	49	0	49
SH_50	ICC	65	Female	WES	I	primary tumor	moderate	Positive	N0	M0	negative	negative	1	31	0	47
SH_51	ICC	52	Male	WES	II	primary tumor	moderate	Negative	N0	M0	positive	negative	1	1	1	22
SH_52	ICC	49	Male	WES	II	primary tumor	moderate	Positive	N0	M0	positive	positive	1	4	1	7
SH_53	ICC	69	Male	WES	I	primary tumor	moderate	Negative	N0	M0	negative	negative	1	10	0	48
SH_56	ICC	38	Male	WES	III+IV	primary tumor	moderate	Positive	N0	M1	negative	negative	1	1	1	4
SH_58	ICC	41	Male	WES	II	primary tumor	moderate	Positive	N0	M0	positive	negative	1	3	1	6
SH_59	ICC	60	Female	WES	III+IV	primary tumor	moderate	Positive	N0	M1	negative	positive	1	NA	1	4
SH_61	ICC	39	Female	WES	III+IV	primary tumor	moderate	Negative	N1	M0	negative	positive	1	1	1	8
SH_63	ICC	52	Female	WES	III+IV	primary tumor	moderate	Negative	N1	M0	negative	positive	1	5	1	12
SH_64	ICC	51	Male	WES	III+IV	primary tumor	moderate	Positive	N1	M0	positive	positive	NA	NA	1	2
SH_66	ICC	63	Female	WES	I	primary tumor	moderate	Negative	N0	M0	negative	positive	NA	NA	1	11
SH_67	ICC	66	Male	WES	I	primary tumor	moderate	Negative	N0	M0	negative	negative	NA	NA	0	46
SH_69	ICC	50	Male	WES	III+IV	primary tumor	moderate	Positive	N1	M0	negative	positive	1	21	1	23
SH_70	ICC	54	Female	WES	II	primary tumor	moderate	Negative	N0	M0	negative	negative	1	3	1	9
SH_71	ICC	59	Male	WES	I	primary tumor	moderate	Positive	N0	M0	negative	negative	0	46	0	46
SH_74	ICC	38	Male	WES	I	primary tumor	moderate	Negative	N0	M0	negative	negative	0	41	0	41
SH_79	ICC	43	Female	WES	II	primary tumor	moderate	Positive	N0	M0	negative	negative	0	35	0	35
SH_80	ICC	59	Female	WES	I	primary tumor	moderate	Negative	N0	M0	negative	negative	1	3	1	20
SH_81	ICC	38	Male	WES	III+IV	primary tumor	moderate	Negative	N1	M0	negative	positive	1	2	1	22
SH_82	ICC	43	Female	WES	I	primary tumor	moderate	Negative	N0	M0	negative	negative	1	13	1	34
SH_85	ICC	57	Female	WES	II	primary tumor	moderate	Positive	N0	M0	negative	negative	1	3	1	5
SH_86	ICC	66	Male	WES	I	primary tumor	moderate	Negative	N0	M0	negative	positive	0	35	0	35
SH_87	ICC	53	Male	WES	II	primary tumor	moderate	Positive	N0	M0	positive	negative	1	4	1	12
SH_88	ICC	56	Male	WES	I	primary tumor	moderate	Positive	N0	M0	negative	positive	1	21	0	35
SH_89	ICC	64	Female	WES	I	primary tumor	moderate	Negative	N0	M0	negative	negative	1	9	1	31
SH_90	ICC	56	Male	WES	III+IV	primary tumor	moderate	Negative	N1	M0	negative	negative	1	2	1	7
SH_91	ICC	65	Female	WES	I	primary tumor	moderate	Negative	N0	M0	negative	negative	0	39	0	39
SH_94	ICC	51	Male	WES	II	primary tumor	moderate	Positive	N0	M0	negative	negative	0	34	0	34
SH_95	ICC	48	Male	WES	II	primary tumor	moderate	Positive	N0	M0	negative	positive	1	0	1	9
SH_98	ICC	63	Male	WES	III+IV	primary tumor	moderate	Negative	N1	M0	negative	negative	NA	NA	1	5
SH_99	ICC	65	Male	WES	II	primary tumor	moderate	Negative	N0	M0	negative	negative	NA	NA	0	35
XH_P01	ICC	43	Male	WES	III+IV	primary tumor	poor	Negative	N1	M0	negative	negative	1	3	1	10
XH_P02	ICC	25	Female	WES	III+IV	primary tumor	moderate	Negative	N0	M1	negative	positive	1	2	1	13
XH_P03	ICC	60	Female	WES	III+IV	primary tumor	moderate	Negative	N1	M0	negative	positive	1	16	1	20
XH_P04	ICC	45	Female	WES	II	primary tumor	poor	Positive	NX	M0	positive	negative	1	5	0	41
XH_P05	ICC	43	Female	WES	III+IV	primary tumor	poor	Positive	N1	M1	negative	negative	1	2	1	11
XH_P06	ICC	62	Male	WES	I	primary tumor	poor	Negative	NX	M0	negative	negative	1	46	0	48
XH_P07	ICC	59	Female	WES	III+IV	primary tumor	moderate	Negative	N1	M0	negative	negative	1	14	1	18
XH_P08	ICC	63	Female	WES	III+IV	primary tumor	poor	Negative	N1	M0	negative	negative	1	2	1	16
XH_P09	ICC	63	Male	WES	II	primary tumor	moderate	Negative	N0	M0	negative	negative	1	16	1	22
XH_P10	ICC	44	Male	WES	III+IV	primary tumor	poor	Positive	N1	M1	negative	negative	1	2	1	9
XH_P11	ICC	45	Male	WES	I	primary tumor	poor	Positive	N0	M0	negative	negative	1	3	1	14
XH_P12	ICC	57	Female	WES	II	primary tumor	poor	Positive	N0	M0	negative	negative	1	32	1	50
XH_P13	ICC	65	Female	WES	III+IV	primary tumor	moderate	Negative	N1	M0	negative	negative	1	14	1	17
XH_P15	ICC	83	Male	WES	II	primary tumor	moderate	Negative	NX	M0	negative	positive	1	5	1	17
XH_P16	ICC	62	Male	WES	I	primary tumor	moderate	Negative	N0	M0	negative	negative	0	60	0	60
XH_P17	ICC	63	Female	WES	II	primary tumor	poor	Positive	N0	M0	negative	negative	0	70	0	70
XH_P18	ICC	66	Male	WES	III+IV	primary tumor	poor	Negative	N1	M0	negative	negative	1	3	1	11
XH_P20	ICC	62	Male	WES	I	primary tumor	poor	Positive	NX	M0	negative	negative	1	4	0	16
XH_P21	ICC	55	Male	WES	II	primary tumor	poor	Negative	N0	M0	negative	negative	1	2	1	25
XH_P24	ICC	34	Male	WES	II	primary tumor	poor	Negative	NX	M0	negative	negative	0	42	0	42

XH_P25	ICC	56	Male	WES	II	primary tumor	poor	Positive	NX	M0	negative	positive	1	13	1	18
XH_P27	ICC	34	Male	WES	II	primary tumor	poor	Negative	N0	M0	negative	negative	1	3	1	10
XH_P28	ICC	52	Male	WES	II	primary tumor	poor	Negative	N0	M0	negative	negative	1	20	0	60
XH_P29	ICC	49	Male	WES	II	primary tumor	moderate	Negative	N0	M0	negative	negative	1	4	1	7
XH_P30	ICC	48	Male	WES	I	primary tumor	poor	Negative	N0	M0	negative	negative	1	13	1	31
XH_P31	ICC	62	Female	WES	II	primary tumor	moderate	Positive	N0	M0	positive	negative	1	6	1	17
XH_P32	ICC	51	Female	WES	III+IV	primary tumor	poor	Negative	N1	M1	positive	positive	1	1	1	12
XH_P33	ICC	62	Male	WES	III+IV	primary tumor	moderate	Positive	N1	M0	negative	positive	1	18	1	35
XH_P34	ICC	51	Female	WES	II	primary tumor	moderate	Negative	N0	M0	positive	negative	1	9	1	14
XH_P35	ICC	68	Male	WES	III+IV	primary tumor	moderate	Negative	NX	M0	positive	negative	1	3	1	19
XH_P36	ICC	40	Female	WES	III+IV	primary tumor	moderate	Negative	N0	M0	negative	positive	1	5	0	34
XH_P37	ICC	61	Female	WES	III+IV	primary tumor	poor	Negative	N1	M0	negative	positive	1	5	1	16
XH_P38	ICC	53	Female	WES	II	primary tumor	poor	Negative	N0	M0	negative	negative	1	10	1	44
XH_P39	ICC	69	Female	WES	III+IV	primary tumor	moderate	Negative	N1	M0	negative	positive	1	3	1	10
XH_P40	ECC	47	Male	WES	I	primary tumor	poor	Negative	N0	M0	negative	negative	1	32	1	40
XH_P41	ICC	61	Male	WES	III+IV	primary tumor	moderate	Negative	N1	M0	negative	negative	1	6	1	12
XH_P42	ICC	51	Male	WES	I	primary tumor	poor	Negative	N0	M0	negative	negative	1	2	0	54
XH_P43	ICC	42	Male	WES	III+IV	primary tumor	moderate	Negative	N1	M0	positive	positive	1	16	1	48
XH_P44	ICC	52	Male	WES	III+IV	primary tumor	moderate	Negative	N1	M0	negative	positive	1	3	1	7
XH_P45	ICC	38	Female	WES	I	primary tumor	moderate	Negative	N0	M0	negative	negative	1	19	1	42
XH_P46	ICC	65	Male	WES	I	primary tumor	poor	Negative	N0	M0	negative	positive	1	4	1	16
XH_P47	ICC	67	Male	WES	I	primary tumor	poor	Negative	N0	M0	negative	positive	1	6	0	18
XH_P48	ICC	52	Male	WES	III+IV	primary tumor	poor	Negative	N1	M0	negative	negative	1	6	0	30
XH_P49	ICC	65	Female	WES	III+IV	primary tumor	poor	Positive	N1	M1	negative	positive	1	6	0	20
XH_P50	ICC	51	Male	WES	III+IV	metastatic tumor	moderate	Negative	N1	M1	negative	negative	1	3	1	15
XH_P51	ICC	53	Female	WES	I	primary tumor	poor	Positive	N0	M0	negative	negative	1	12	0	30
XH_P52	GBC	52	Male	WES	II	metastatic tumor	moderate	Negative	N0	M0	negative	positive	1	3	1	22
XH_P53	GBC	58	Female	WES	III+IV	primary tumor	poor	Negative	N0	M0	negative	negative	1	2	0	22
XH_P54	ICC	57	Female	WES	III+IV	primary tumor	moderate	Negative	N1	M1	negative	negative	1	3	1	12
XH_P55	GBC	56	Female	WES	III+IV	primary tumor	moderate	Negative	N0	M0	negative	negative	1	2	1	24
XH_P557	ECC	72	Male	WES	III+IV	primary tumor	moderate	Negative	N1	M0	negative	positive	1	2	0	17
XH_P56	ICC	77	Male	WES	I	primary tumor	moderate	Positive	N0	M0	negative	positive	1	3	0	14
XH_P57	GBC	69	Female	WES	III+IV	primary tumor	moderate	Negative	N1	M0	negative	positive	1	2	0	15
XH_P61	GBC	58	Male	WES	III+IV	primary tumor	poor	Negative	N1	M0	positive	negative	1	1	0	5
XH_P62	ICC	36	Male	WES	III+IV	primary tumor	moderate	Negative	N0	M1	negative	negative	1	1	0	13
XH_P63	ICC	63	Female	WES	III+IV	primary tumor	poor	Negative	N0	M1	negative	positive	1	2	0	12
XH_P87	ICC	54	Male	WES	I	primary tumor	moderate	Negative	N0	M0	negative	negative	0	18	0	21
XH_P88	ICC	62	Male	WES	I	primary tumor	poor	Negative	N0	M0	negative	negative	1	18	0	21
XH_P89	ICC	77	Female	WES	I	primary tumor	moderate	Negative	N0	M0	negative	positive	1	7	1	11
XH_P90	ICC	68	Female	WES	III+IV	primary tumor	poor	Negative	N1	M1	negative	negative	1	2	1	5
XH_P91	ICC	70	Female	WES	III+IV	primary tumor	poor	Negative	N1	M0	negative	negative	1	4	0	17
XH_P92	ICC	57	Male	WES	II	primary tumor	poor	Negative	N0	M0	negative	positive	1	7	0	18
XH_P93	ICC	57	Male	WES	II	primary tumor	poor	Negative	N0	M0	negative	negative	1	1	1	4
XH_P94	ICC	32	Male	WES	III+IV	primary tumor	poor	Negative	N1	M1	positive	positive	1	1	0	16
XH_P95	ICC	38	Female	WES	I	primary tumor	poor	Negative	N0	M0	negative	negative	0	8	0	8
XH_P96	ICC	67	Female	WES	I	primary tumor	moderate	Negative	N0	M0	negative	negative	0	6	0	16
XH_P97	GBC	55	Male	WES	II	primary tumor	poor	Negative	N0	M0	negative	negative	1	4	0	19
XH_P99	ICC	49	Male	WES	II	primary tumor	poor	Negative	N0	M0	negative	positive	1	4	0	11
116K0135A1	ICC	44	Male	CSYS	NA	primary tumor	poor	Negative	NX	MX	NA	NA	NA	NA	NA	NA
116L0197A1	ECC	44	Female	CSYS	NA	primary tumor	poor	Negative	N1	M0	NA	NA	NA	NA	NA	NA
116L0221A1	ICC	67	Male	CSYS	III+IV	metastatic tumor	NA	Negative	NX	M1	NA	NA	NA	NA	NA	NA
116L0254A1	ECC	48	Male	CSYS	I	primary tumor	moderate	Negative	N0	M0	NA	NA	NA	NA	NA	NA
116L0263A1	GBC	58	Female	CSYS	NA	metastatic tumor	NA	Negative	NX	MX	NA	NA	NA	NA	NA	NA
116L0279A1	ECC	64	Female	CSYS	NA	primary tumor	poor	Negative	N0	M0	NA	NA	NA	NA	NA	NA
116L0287A1	ICC	52	Male	CSYS	NA	primary tumor	moderate	Negative	NX	MX	NA	NA	NA	NA	NA	NA
117B0454A1	GBC	74	Female	CSYS	NA	metastatic tumor	moderate	Negative	NX	MX	NA	NA	NA	NA	NA	NA
117B0488A1	ECC	69	Female	CSYS	I	primary tumor	moderate	Negative	N0	M0	NA	NA	NA	NA	NA	NA
117B0545A1	GBC	54	Female	CSYS	III+IV	primary tumor	poor	Negative	N1	MX	NA	NA	NA	NA	NA	NA
117C0618A1	GBC	67	Female	CSYS	III+IV	primary tumor	poor	Negative	N1	MX	NA	NA	NA	NA	NA	NA
117C0646A1	ICC	62	Male	CSYS	NA	primary tumor	moderate	Negative	NX	MX	NA	NA	NA	NA	NA	NA
117C0651A1	ECC	63	Male	CSYS	I	primary tumor	moderate	Negative	N0	M0	NA	NA	NA	NA	NA	NA
117C0664A1	ICC	47	Male	CSYS	I	primary tumor	moderate	Positive	N0	M0	NA	NA	NA	NA	NA	NA
117C0733A1	ECC	50	Male	CSYS	NA	primary tumor	poor	Negative	N0	M0	NA	NA	NA	NA	NA	NA
117C0866A1	GBC	58	Male	CSYS	NA	primary tumor	well	Negative	NX	MX	NA	NA	NA	NA	NA	NA
117D0978A1	ICC	38	Male	CSYS	NA	primary tumor	poor	Negative	NX	MX	NA	NA	NA	NA	NA	NA
117D0997A1	ECC	66	Male	CSYS	III+IV	primary tumor	poor	Negative	N1	M0	NA	NA	NA	NA	NA	NA
117D1007A1	ECC	64	Male	CSYS	III+IV	primary tumor	moderate	Negative	N1	M0	NA	NA	NA	NA	NA	NA
117D1030A1	ICC	82	Male	CSYS	III+IV	metastatic tumor	poor	Negative	NX	M1	NA	NA	NA	NA	NA	NA
117D1074R1	ICC	60	Male	CSYS	NA	primary tumor	NA	Negative	NX	MX	NA	NA	NA	NA	NA	NA
117D1101A1	ICC	54	Male	CSYS	III+IV	primary tumor	poor	Negative	N1	M0	NA	NA	NA	NA	NA	NA
117D1130A1	ECC	44	Female	CSYS	III+IV	metastatic tumor	NA	Negative	N1	M1	NA	NA	NA	NA	NA	NA
117D1192A1	ICC	79	Male	CSYS	III+IV	primary tumor	moderate	Negative	N1	M0	NA	NA	NA	NA	NA	NA
117E1223A1	ECC	83	Male	CSYS	NA	primary tumor	moderate	Negative	N1	M0	NA	NA	NA	NA	NA	NA
117E1250R1	GBC	62	Male	CSYS	III+IV	metastatic tumor	moderate	NA	N1	M0	NA	NA	NA	NA	NA	NA
117E1259R1	ICC	49	Male	CSYS	III+IV	metastatic tumor	poor	Positive	NX	M1	NA	NA	NA	NA	NA	NA
117E1261R1	ICC	40	Male	CSYS	I	primary tumor	poor	Negative	N0	M0	NA	NA	NA	NA	NA	NA
117E1396A1	ICC	69	Male	CSYS	NA	primary tumor	NA	Negative	NX	MX	NA	NA	NA	NA	NA	NA
117E1425A1	ICC	60	Male	CSYS	III+IV	primary tumor	moderate	Positive	N1	M1	NA	NA	NA	NA	NA	NA
117E1443A1	ECC	68	Male	CSYS	I	primary tumor	moderate	Negative	N0	M0	NA	NA	NA	NA	NA	NA
117E1534A1	GBC	52	Female	CSYS	III+IV	primary tumor	moderate	Negative	N0	M1	NA	NA	NA	NA	NA	NA
117F1556A1	ICC	66	Female	CSYS	NA	primary tumor	NA	Negative	NX	MX	NA	NA	NA	NA	NA	NA
117F1640A1	ICC	63	Male	CSYS	I	primary tumor	moderate	Negative	N0	M0	NA	NA	NA	NA	NA	NA
117F1648A2	ICC	62	Male	CSYS	III+IV	primary tumor	poor	Negative	NX	M1	NA	NA	NA	NA	NA	NA
117F1689A1	ICC	69	Male	CSYS	NA	primary tumor	poor	Negative	NX	MX	NA	NA	NA	NA	NA	NA
117F1759A1	ECC	57	Female	CSYS	III+IV	metastatic tumor	poor	Negative	NX	M1	NA	NA	NA	NA	NA	NA
117F1767A1	ICC	54	Male	CSYS	NA	primary tumor	poor	Negative	NX	MX	NA	NA	NA	NA	NA	NA
117F1770A1	GBC	72	Male	CSYS	III+IV	primary tumor	moderate	Negative	N0	M0	NA	NA	NA	NA	NA	NA
117F1811A1	GBC	66	Male	CSYS	III+IV	primary tumor	poor	Negative	N1	M0	NA	NA	NA	NA	NA	NA
117G2016A1	ECC	54	Female	CSYS	I	primary tumor	poor	Negative	N0	M0	NA	NA	NA	NA	NA	NA
117G2026A1	GBC	66	Male	CSYS	III+IV	metastatic tumor	poor	Negative	NX	M1	NA	NA	NA	NA	NA	NA
117G2087A1	ECC	60	Male	CSYS	III+IV	metastatic tumor	poor	Negative	NX	M1	NA	NA	NA	NA	NA	NA
117H2198A1	ICC	43	Male	CSYS	NA	primary tumor	NA	Negative	NX	MX	NA	NA	NA	NA	NA	NA
117H2255A1	GBC	58	Female	CSYS	III+IV	primary tumor	poor	Negative	N1	M0	NA	NA	NA	NA	NA	NA
117H2289A1	ICC	50	Male	CSYS	III+IV	primary tumor	poor	Positive	NX	M1	NA	NA	NA	NA	NA	NA
117H2404A1	ECC	39	Male	CSYS	III+IV	primary tumor	poor	Negative	N1	M0	NA	NA	NA	NA	NA	NA
117H2427A1	GBC	60	Male	CSYS	III+IV	primary tumor	moderate	Negative	N0	M1	NA	NA	NA	NA	NA	NA
117H2467A1	ICC	46	Male	CSYS	III+IV	recurrent tumor	moderate	NA	NX	MX	NA	NA	NA	NA	NA	NA
117H2489A1	ICC	62	Female	CSYS	III+IV	primary tumor	moderate	Negative	NX	M1	NA	NA	NA	NA	NA	NA
117H2552A1	ECC	70	Female	CSYS	II	primary tumor	moderate	Negative	N0	M0	NA	NA	NA	NA	NA	NA
117I2596A1	ECC	48	Male	CSYS	I	primary tumor	moderate	Negative	N0	M0	NA	NA	NA	NA	NA	NA
117I2661A1	ICC	46	Male	CSYS	III+IV	primary tumor	poor	Negative	NX	MX	NA	NA	NA	NA	NA	NA
117I2687A1	ICC	66	Female	CSYS	III+IV	primary tumor	poor	Positive	N1	M0	NA	NA	NA	NA	NA	NA
117I2735A1	ECC	54	Male	CSYS	II	primary tumor	moderate	Negative	N0	M0	NA	NA	NA	NA	NA	NA
117I2766A1	ECC	60	Female	CSYS	II	primary tumor	well	Negative	N0	M0	NA	NA	NA	NA	NA	NA
117I2799A1	ICC	51	Male	CSYS	III+IV	primary tumor	poor	Negative	N1	M0	NA	NA	NA	NA	NA	NA
117I2805A1	ECC	61	Male	CSYS	II	primary tumor	poor	Negative	N1	M0	NA	NA	NA	NA	NA	NA
117I2844A1	ECC	70	Male	CSYS	III+IV	recurrent tumor	moderate	Negative	NX	M1	NA	NA	NA	NA	NA	NA
117I2845A1	ICC	73	Male	CSYS	NA	primary tumor	NA	Negative	NX	MX	NA	NA	NA	NA	NA	NA
117I2858A1	ICC	72	Female	CSYS	NA	primary tumor	well	Positive	N0	MX	NA	NA	NA	NA	NA	NA
117I2888A1	ECC	69	Male	CSYS	I	primary tumor	moderate	Negative	N0	M0	NA	NA	NA	NA	NA	NA
117I2908A1	GBC	55	Male	CSYS	III+IV	primary tumor	poor	Positive	N1	M0	NA	NA	NA	NA	NA	NA
117I2919A1	ICC	62	Female	CSYS	NA	primary tumor	NA	Negative	NX	MX	NA	NA	NA	NA	NA	NA
117I2931A1	GBC	61	Male	CSYS	II	primary tumor	moderate	Negative	N0	M0	NA	NA	NA	NA	NA	NA
117I2978A1	ICC	63	Female	CSYS	III+IV	primary tumor	poor	Negative	N0	M0	NA	NA	NA	NA	NA	NA
117J3004A1	ICC	61	Male	CSYS	II	primary tumor	moderate	Negative	N0	M0	NA	NA	NA	NA	NA	NA

117J3045A1	ICC	37	Male	CSYS	NA	primary tumor	poor	Positive	NX	MX	NA	NA	NA	NA	NA	NA
117J3086A1	ICC	86	Male	CSYS	III+IV	primary tumor	poor	Negative	N1	M1	NA	NA	NA	NA	NA	NA
117J3117A1	GBC	55	Male	CSYS	III+IV	primary tumor	NA	Negative	N0	M0	NA	NA	NA	NA	NA	NA
117J3131A1	ICC	53	Male	CSYS	I	primary tumor	moderate	Positive	N0	M0	NA	NA	NA	NA	NA	NA
117J3133A1	ECC	58	Female	CSYS	I	primary tumor	moderate	Negative	N0	M0	NA	NA	NA	NA	NA	NA
117J3136A1	GBC	57	Female	CSYS	III+IV	metastatic tumor	NA	NA	NX	M1	NA	NA	NA	NA	NA	NA
117J3193A1	ECC	50	Female	CSYS	III+IV	primary tumor	moderate	Negative	NX	M1	NA	NA	NA	NA	NA	NA
117J3213A1	ICC	49	Male	CSYS	NA	primary tumor	moderate	Positive	NX	MX	NA	NA	NA	NA	NA	NA
117J3237A1	GBC	31	Female	CSYS	III+IV	metastatic tumor	moderate	Negative	N0	M0	NA	NA	NA	NA	NA	NA
117J3239A1	GBC	61	Female	CSYS	III+IV	primary tumor	poor	Negative	N1	M0	NA	NA	NA	NA	NA	NA
117J3282A1	GBC	72	Male	CSYS	III+IV	primary tumor	poor	Negative	N1	M0	NA	NA	NA	NA	NA	NA
117J3295A1	ECC	56	Female	CSYS	III+IV	primary tumor	moderate	Negative	N1	M0	NA	NA	NA	NA	NA	NA
117J3306A1	ICC	58	Male	CSYS	II	primary tumor	moderate	Positive	N0	M0	NA	NA	NA	NA	NA	NA
117J3318A1	GBC	73	Male	CSYS	III+IV	primary tumor	moderate	Negative	N1	M0	NA	NA	NA	NA	NA	NA
117K3377A1	ICC	74	Male	CSYS	NA	primary tumor	NA	Negative	N0	M0	NA	NA	NA	NA	NA	NA
117K3386A1	ICC	65	Male	CSYS	III+IV	primary tumor	moderate	Positive	NX	M1	NA	NA	NA	NA	NA	NA
117K3403A1	ECC	53	Male	CSYS	I	primary tumor	poor	Negative	N0	M0	NA	NA	NA	NA	NA	NA
117K3508A1	GBC	51	Female	CSYS	III+IV	metastatic tumor	poor	Negative	N1	M1	NA	NA	NA	NA	NA	NA
117K3520A1	ICC	53	Female	CSYS	III+IV	primary tumor	well	Negative	N1	M0	NA	NA	NA	NA	NA	NA
117K3527A1	ICC	67	Female	CSYS	I	primary tumor	poor	Positive	N0	M0	NA	NA	NA	NA	NA	NA
117K3556A1	ICC	60	Male	CSYS	III+IV	primary tumor	poor	Positive	NX	MX	NA	NA	NA	NA	NA	NA
117K3576A1	ICC	54	Male	CSYS	III+IV	primary tumor	moderate	Negative	N1	M0	NA	NA	NA	NA	NA	NA
117K3596A1	ECC	75	Female	CSYS	II	primary tumor	poor	Negative	N0	M0	NA	NA	NA	NA	NA	NA
117K3609A1	ICC	48	Male	CSYS	III+IV	primary tumor	poor	Positive	N1	M1	NA	NA	NA	NA	NA	NA
117K3620A1	ICC	52	Male	CSYS	III+IV	primary tumor	moderate	NA	NX	M1	NA	NA	NA	NA	NA	NA
117K3660A1	GBC	73	Female	CSYS	NA	primary tumor	moderate	Negative	NX	MX	NA	NA	NA	NA	NA	NA
117K3669A1	ICC	54	Female	CSYS	III+IV	primary tumor	poor	Negative	N1	M1	NA	NA	NA	NA	NA	NA
117K3688A1	GBC	49	Female	CSYS	III+IV	primary tumor	poor	Negative	N0	M0	NA	NA	NA	NA	NA	NA
117K3699A1	ICC	67	Female	CSYS	III+IV	metastatic tumor	moderate	Negative	NX	M1	NA	NA	NA	NA	NA	NA
117K3728A1	ECC	61	Female	CSYS	II	primary tumor	poor	Negative	N1	M0	NA	NA	NA	NA	NA	NA
117K3750A1	ICC	52	Male	CSYS	III+IV	primary tumor	poor	NA	N1	M0	NA	NA	NA	NA	NA	NA
117L3780A1	ECC	60	Male	CSYS	I	primary tumor	moderate	Negative	N0	M0	NA	NA	NA	NA	NA	NA
117L3815A1	GBC	57	Female	CSYS	III+IV	primary tumor	well	Negative	NX	M1	NA	NA	NA	NA	NA	NA
117L3816A1	ICC	58	Female	CSYS	III+IV	primary tumor	poor	Positive	N1	M0	NA	NA	NA	NA	NA	NA
117L3817A1	ICC	65	Male	CSYS	II	primary tumor	poor	Negative	N0	M0	NA	NA	NA	NA	NA	NA
117L3842A1	ICC	40	Male	CSYS	II	primary tumor	poor	Positive	N0	M0	NA	NA	NA	NA	NA	NA
117L3851A1	ECC	60	Female	CSYS	III+IV	metastatic tumor	NA	Negative	NX	M1	NA	NA	NA	NA	NA	NA
117L3938A1	GBC	49	Female	CSYS	III+IV	primary tumor	poor	Negative	N0	M0	NA	NA	NA	NA	NA	NA
117L3940A1	ICC	65	Male	CSYS	III+IV	primary tumor	well	Negative	N0	M1	NA	NA	NA	NA	NA	NA
117L3952A1	ICC	51	Female	CSYS	III+IV	primary tumor	moderate	NA	N1	M0	NA	NA	NA	NA	NA	NA
117L3964A1	ICC	52	Female	CSYS	I	primary tumor	moderate	Negative	N0	M0	NA	NA	NA	NA	NA	NA
117L3965A1	ICC	64	Male	CSYS	II	primary tumor	poor	Negative	N0	M0	NA	NA	NA	NA	NA	NA
117L4005A1	ICC	51	Male	CSYS	III+IV	primary tumor	poor	Positive	N0	M1	NA	NA	NA	NA	NA	NA
117L4040A1	GBC	54	Female	CSYS	III+IV	primary tumor	poor	Negative	N1	M0	NA	NA	NA	NA	NA	NA
117L4049A1	ICC	63	Male	CSYS	I	primary tumor	moderate	Negative	N0	M0	NA	NA	NA	NA	NA	NA
117L4084A1	ICC	66	Male	CSYS	I	primary tumor	moderate	Negative	N0	M0	NA	NA	NA	NA	NA	NA
117L4087A1	ECC	59	Female	CSYS	II	primary tumor	NA	Negative	N1	M0	NA	NA	NA	NA	NA	NA
117L4089A1	ECC	46	Female	CSYS	II	primary tumor	poor	Negative	N1	M0	NA	NA	NA	NA	NA	NA
117L4100A1	ICC	54	Male	CSYS	III+IV	primary tumor	moderate	Positive	N1	M0	NA	NA	NA	NA	NA	NA
117L4103A1	GBC	50	Female	CSYS	III+IV	metastatic tumor	poor	Negative	NX	M1	NA	NA	NA	NA	NA	NA
117L4125A1	ICC	65	Female	CSYS	III+IV	metastatic tumor	moderate	Negative	NX	M1	NA	NA	NA	NA	NA	NA
117L4126A1	ICC	62	Male	CSYS	III+IV	recurrent tumor	poor	Positive	NX	M1	NA	NA	NA	NA	NA	NA
117L4133A1	GBC	52	Female	CSYS	II	primary tumor	poor	Negative	N0	M0	NA	NA	NA	NA	NA	NA
117L4139A1	ECC	63	Male	CSYS	I	primary tumor	moderate	NA	N0	M0	NA	NA	NA	NA	NA	NA
118A4239A1	ECC	73	Female	CSYS	II	primary tumor	poor	Negative	N1	M0	NA	NA	NA	NA	NA	NA
118A4277A1	ICC	68	Female	CSYS	III+IV	primary tumor	poor	Negative	N1	M0	NA	NA	NA	NA	NA	NA
118A4280A1	GBC	79	Male	CSYS	III+IV	primary tumor	poor	Negative	N0	M0	NA	NA	NA	NA	NA	NA
118A4284A1	ICC	52	Male	CSYS	II	primary tumor	moderate	Positive	N0	M0	NA	NA	NA	NA	NA	NA
118A4298A1	ICC	57	Male	CSYS	III+IV	primary tumor	poor	Negative	NX	M1	NA	NA	NA	NA	NA	NA
118A4330A1	ECC	74	Male	CSYS	II	primary tumor	moderate	Negative	N0	M0	NA	NA	NA	NA	NA	NA
118A4336A1	GBC	73	Male	CSYS	III+IV	primary tumor	moderate	Negative	N1	M0	NA	NA	NA	NA	NA	NA
118A4375A1	ICC	67	Male	CSYS	III+IV	primary tumor	moderate	Positive	NX	M1	NA	NA	NA	NA	NA	NA
118A4386A1	ICC	57	Female	CSYS	III+IV	primary tumor	poor	Negative	N1	M0	NA	NA	NA	NA	NA	NA
118A4387A1	GBC	74	Male	CSYS	III+IV	metastatic tumor	NA	Negative	NX	M1	NA	NA	NA	NA	NA	NA
118A4434A1	ICC	43	Female	CSYS	II	primary tumor	well	Positive	N0	M0	NA	NA	NA	NA	NA	NA
118A4438A1	ICC	67	Female	CSYS	II	primary tumor	poor	Negative	N0	M0	NA	NA	NA	NA	NA	NA
118A4486A1	GBC	66	Male	CSYS	III+IV	primary tumor	well	Negative	N0	M0	NA	NA	NA	NA	NA	NA
118A4554A1	GBC	70	Male	CSYS	II	primary tumor	poor	Negative	N0	M0	NA	NA	NA	NA	NA	NA
118A4682A2	ICC	57	Male	CSYS	II	primary tumor	moderate	Positive	N0	M0	NA	NA	NA	NA	NA	NA
118A4689A1	ECC	34	Female	CSYS	II	primary tumor	NA	Negative	N0	M0	NA	NA	NA	NA	NA	NA
118A4698A1	ECC	56	Female	CSYS	NA	primary tumor	poor	Negative	NX	MX	NA	NA	NA	NA	NA	NA
118A4765A2	ICC	59	Female	CSYS	I	primary tumor	moderate	Negative	N0	M0	NA	NA	NA	NA	NA	NA
118B4778A1	ICC	52	Male	CSYS	III+IV	metastatic tumor	moderate	Negative	NX	M1	NA	NA	NA	NA	NA	NA
118B4782A1	ICC	80	Female	CSYS	III+IV	primary tumor	moderate	Negative	N0	M1	NA	NA	NA	NA	NA	NA
118B4789A1	ECC	80	Female	CSYS	III+IV	metastatic tumor	NA	Negative	NX	M1	NA	NA	NA	NA	NA	NA
118B4791A2	ECC	76	Male	CSYS	II	primary tumor	moderate	Positive	N1	M0	NA	NA	NA	NA	NA	NA
118B4864A2	ICC	59	Female	CSYS	NA	primary tumor	poor	Negative	NX	MX	NA	NA	NA	NA	NA	NA
118B4888A1	ICC	62	Female	CSYS	NA	primary tumor	moderate	Negative	N0	MX	NA	NA	NA	NA	NA	NA
118B4909A1	GBC	67	Female	CSYS	III+IV	metastatic tumor	NA	Negative	NX	M1	NA	NA	NA	NA	NA	NA
118B4937A1	ICC	61	Female	CSYS	III+IV	primary tumor	NA	Negative	NX	M1	NA	NA	NA	NA	NA	NA
118B5017A1	ECC	60	Male	CSYS	II	primary tumor	poor	Negative	N1	M0	NA	NA	NA	NA	NA	NA
118B5044A1	ECC	68	Male	CSYS	II	primary tumor	moderate	NA	N1	M0	NA	NA	NA	NA	NA	NA
118B5053A1	ECC	60	Female	CSYS	II	primary tumor	poor	Negative	N0	M0	NA	NA	NA	NA	NA	NA
118B5073A1	GBC	62	Female	CSYS	III+IV	metastatic tumor	NA	Negative	N1	M1	NA	NA	NA	NA	NA	NA
118B5110A1	ICC	74	Male	CSYS	NA	primary tumor	poor	Negative	NX	MX	NA	NA	NA	NA	NA	NA
118B5118A1	ICC	65	Male	CSYS	NA	primary tumor	moderate	Negative	NX	MX	NA	NA	NA	NA	NA	NA
118B5121A1	ECC	62	Male	CSYS	II	primary tumor	poor	Negative	N0	M0	NA	NA	NA	NA	NA	NA
118B5127A1	ECC	63	Female	CSYS	II	primary tumor	moderate	Negative	N0	M0	NA	NA	NA	NA	NA	NA
118C5128A1	ICC	65	Male	CSYS	I	primary tumor	moderate	Negative	N0	M0	NA	NA	NA	NA	NA	NA
118C5132A1	ICC	55	Male	CSYS	III+IV	primary tumor	moderate	Negative	N1	M1	NA	NA	NA	NA	NA	NA
118C5169A1	ICC	60	Female	CSYS	III+IV	primary tumor	moderate	Negative	N1	M0	NA	NA	NA	NA	NA	NA
118C5218A1	ICC	63	Male	CSYS	III+IV	primary tumor	poor	Negative	NX	M1	NA	NA	NA	NA	NA	NA
118C5252A1	ICC	57	Male	CSYS	NA	primary tumor	NA	Positive	NX	MX	NA	NA	NA	NA	NA	NA
118C5275A1	ICC	61	Female	CSYS	III+IV	primary tumor	poor	Negative	N1	M0	NA	NA	NA	NA	NA	NA
118C5293A1	ICC	71	Male	CSYS	NA	primary tumor	moderate	Negative	NX	MX	NA	NA	NA	NA	NA	NA
118C5350A2	ECC	66	Male	CSYS	NA	primary tumor	poor	Negative	NX	MX	NA	NA	NA	NA	NA	NA
118C5506A1	ICC	64	Male	CSYS	III+IV	primary tumor	poor	NA	N1	M0	NA	NA	NA	NA	NA	NA
118C5592A1	ICC	76	Female	CSYS	NA	primary tumor	NA	Positive	NX	MX	NA	NA	NA	NA	NA	NA
118C5597A1	ECC	67	Male	CSYS	II	primary tumor	moderate	Negative	N0	M0	NA	NA	NA	NA	NA	NA
118C5599A1	ICC	59	Female	CSYS	III+IV	primary tumor	poor	Negative	N1	M0	NA	NA	NA	NA	NA	NA
118C5623A1	GBC	53	Female	CSYS	NA	primary tumor	poor	Negative	NX	MX	NA	NA	NA	NA	NA	NA
118C5635A1	ICC	59	Male	CSYS	III+IV	primary tumor	poor	Negative	N1	M0	NA	NA	NA	NA	NA	NA
118C5668A1	ICC	65	Female	CSYS	III+IV	primary tumor	poor	Positive	N1	M0	NA	NA	NA	NA	NA	NA
118C5749A1	GBC	35	Male	CSYS	II	primary tumor	moderate	Positive	N0	M0	NA	NA	NA	NA	NA	NA
118C5765A1	ICC	50	Male	CSYS	III+IV	primary tumor	moderate	Negative	N1	M0	NA	NA	NA	NA	NA	NA
118C5766A1	ECC	72	Female	CSYS	II	primary tumor	moderate	Negative	N1	M0	NA	NA	NA	NA	NA	NA
118C5792A1	ICC	65	Male	CSYS	NA	primary tumor	NA	NA	NX	MX	NA	NA	NA	NA	NA	NA
118C5833A1	ICC	61	Male	CSYS	I	primary tumor	moderate	NA	N0	M0	NA	NA	NA	NA	NA	NA
118C5910A1	ICC	73	Female	CSYS	III+IV	primary tumor	NA	Positive	N1	M0	NA	NA	NA	NA	NA	NA
118D5912A3	ICC	53	Male	CSYS	III+IV	metastatic tumor	NA	Negative	NX	MX	NA	NA	NA	NA	NA	NA
118D5925A1	ECC	64	Female	CSYS	II	primary tumor	poor	Negative	N1	M0	NA	NA	NA	NA	NA	NA
118D5949A1	ECC	63	Male	CSYS	III+IV	metastatic tumor	moderate	Negative	NX	M1	NA	NA	NA	NA	NA	NA
118D5960A1	ICC	44	Male	CSYS	III+IV	primary tumor	NA	Positive	N1	M0	NA	NA	NA	NA	NA	NA
118D5965A1	ECC	53	Male	CSYS	III+IV	primary tumor	moderate	Negative	NX	M1	NA	NA	NA	NA	NA	NA
118D6010J1	ECC	58	Female	CSYS	III+IV	primary tumor	moderate	Negative	N0	M0	NA	NA	NA	NA	NA	NA

118D6094D1	GBC	74	Male	CSYS	II	primary tumor	poor	Negative	N0	M0	NA	NA	NA	NA	NA	NA
118D6120A1	GBC	54	Female	CSYS	II	primary tumor	moderate	Negative	N0	M0	NA	NA	NA	NA	NA	NA
118D6166A1	ICC	72	Female	CSYS	III+IV	primary tumor	poor	Negative	N0	M0	NA	NA	NA	NA	NA	NA
118D6220A1	ICC	46	Female	CSYS	III+IV	primary tumor	well	Negative	NX	M1	NA	NA	NA	NA	NA	NA
118D6255A1	ICC	56	Female	CSYS	III+IV	primary tumor	poor	Positive	NX	M1	NA	NA	NA	NA	NA	NA
118D6295A1	ECC	38	Female	CSYS	III+IV	primary tumor	poor	Negative	NX	M1	NA	NA	NA	NA	NA	NA
118D6350A1	ICC	69	Male	CSYS	III+IV	primary tumor	moderate	Positive	N0	M0	NA	NA	NA	NA	NA	NA
118D6360A1	ICC	61	Female	CSYS	II	primary tumor	moderate	Positive	N0	M0	NA	NA	NA	NA	NA	NA
118D6403A1	ICC	73	Male	CSYS	NA	primary tumor	poor	Negative	NX	MX	NA	NA	NA	NA	NA	NA
118D6420A1	GBC	81	Male	CSYS	I	primary tumor	well	Negative	N0	M0	NA	NA	NA	NA	NA	NA
118D6519A1	ECC	47	Male	CSYS	III+IV	primary tumor	poor	NA	NX	M1	NA	NA	NA	NA	NA	NA
118D6546A1	GBC	57	Female	CSYS	III+IV	metastatic tumor	NA	Negative	NX	M1	NA	NA	NA	NA	NA	NA
118D6605A1	ECC	71	Female	CSYS	I	primary tumor	poor	Negative	N0	M0	NA	NA	NA	NA	NA	NA
118D6683A1	ICC	66	Male	CSYS	III+IV	primary tumor	poor	Negative	NX	M1	NA	NA	NA	NA	NA	NA
118D6684A1	ICC	60	Male	CSYS	III+IV	primary tumor	poor	Negative	NX	MX	NA	NA	NA	NA	NA	NA
118D6719A1	ICC	69	Male	CSYS	III+IV	primary tumor	moderate	Negative	N1	M0	NA	NA	NA	NA	NA	NA
118D6898A1	ECC	65	Male	CSYS	III+IV	primary tumor	well	Negative	N1	M0	NA	NA	NA	NA	NA	NA
118D6984A1	ECC	73	Male	CSYS	II	primary tumor	poor	Negative	N1	M0	NA	NA	NA	NA	NA	NA
118D6985A1	ICC	56	Male	CSYS	III+IV	primary tumor	poor	Negative	N1	M0	NA	NA	NA	NA	NA	NA
118E7021A1	GBC	82	Male	CSYS	II	primary tumor	poor	Negative	N0	M0	NA	NA	NA	NA	NA	NA
118E7059A1	ICC	62	Male	CSYS	NA	primary tumor	poor	Negative	NX	MX	NA	NA	NA	NA	NA	NA
118E7103A1	ECC	42	Female	CSYS	II	primary tumor	well	Negative	N0	M0	NA	NA	NA	NA	NA	NA
118E7142A1	ECC	51	Male	CSYS	II	primary tumor	moderate	Negative	N1	M0	NA	NA	NA	NA	NA	NA
118E7171A1	GBC	66	Female	CSYS	II	primary tumor	moderate	Negative	N0	M0	NA	NA	NA	NA	NA	NA
118E7187A1	ICC	83	Female	CSYS	I	primary tumor	moderate	Negative	N0	M0	NA	NA	NA	NA	NA	NA
118E7191A1	ICC	65	Female	CSYS	I	primary tumor	moderate	Negative	N0	M0	NA	NA	NA	NA	NA	NA
118E7218A1	ECC	66	Male	CSYS	III+IV	metastatic tumor	moderate	Negative	NX	M1	NA	NA	NA	NA	NA	NA
118E7249A1	GBC	45	Female	CSYS	II	primary tumor	moderate	Negative	N0	M0	NA	NA	NA	NA	NA	NA
118E7270A1	ECC	55	Male	CSYS	II	primary tumor	moderate	Negative	N0	M0	NA	NA	NA	NA	NA	NA
118E7290A1	ECC	31	Male	CSYS	III+IV	metastatic tumor	NA	Negative	N1	M1	NA	NA	NA	NA	NA	NA
118E7304A1	ECC	59	Male	CSYS	II	metastatic tumor	poor	Negative	N0	M0	NA	NA	NA	NA	NA	NA
118E7306A1	ICC	63	Male	CSYS	NA	primary tumor	poor	NA	NX	MX	NA	NA	NA	NA	NA	NA
118E7325A1	GBC	53	Female	CSYS	II	primary tumor	poor	Negative	N0	M0	NA	NA	NA	NA	NA	NA
118E7344A1	ICC	60	Male	CSYS	III+IV	primary tumor	poor	Negative	N0	M0	NA	NA	NA	NA	NA	NA
118E7407A1	ECC	72	Male	CSYS	III+IV	primary tumor	moderate	Negative	N1	M1	NA	NA	NA	NA	NA	NA
118E7446A1	ICC	65	Female	CSYS	III+IV	primary tumor	moderate	Negative	N0	M0	NA	NA	NA	NA	NA	NA
118E7470A1	ICC	54	Female	CSYS	I	primary tumor	moderate	Negative	N0	M0	NA	NA	NA	NA	NA	NA
118E7477A1	ICC	62	Male	CSYS	III+IV	primary tumor	poor	Negative	N0	M0	NA	NA	NA	NA	NA	NA
118E7484A1	ICC	59	Male	CSYS	III+IV	metastatic tumor	poor	Positive	NX	M1	NA	NA	NA	NA	NA	NA
118E7554A2	GBC	54	Female	CSYS	III+IV	metastatic tumor	NA	Negative	N0	M1	NA	NA	NA	NA	NA	NA
118E7614A1	ICC	45	Male	CSYS	II	primary tumor	moderate	Negative	N0	M0	NA	NA	NA	NA	NA	NA
118E7632A1	ICC	49	Female	CSYS	NA	primary tumor	poor	Negative	NX	MX	NA	NA	NA	NA	NA	NA
118E7633A1	ICC	71	Male	CSYS	I	primary tumor	poor	Positive	N0	M0	NA	NA	NA	NA	NA	NA
118E7744A1	ECC	61	Male	CSYS	I	primary tumor	moderate	NA	N0	M0	NA	NA	NA	NA	NA	NA
118E7805A1	ICC	64	Female	CSYS	II	primary tumor	poor	Positive	N0	M0	NA	NA	NA	NA	NA	NA
118E7868A1	ECC	63	Female	CSYS	I	primary tumor	moderate	Negative	N0	M0	NA	NA	NA	NA	NA	NA
118E7888A1	GBC	64	Female	CSYS	III+IV	primary tumor	poor	Negative	N0	M1	NA	NA	NA	NA	NA	NA
118E7901A1	ICC	48	Male	CSYS	III+IV	primary tumor	moderate	NA	N0	M1	NA	NA	NA	NA	NA	NA
118E7926A1	ICC	43	Female	CSYS	III+IV	primary tumor	poor	Positive	NX	M1	NA	NA	NA	NA	NA	NA
118E7940A1	ECC	77	Male	CSYS	II	primary tumor	poor	NA	N1	M0	NA	NA	NA	NA	NA	NA
118E7964A1	ICC	67	Male	CSYS	III+IV	metastatic tumor	poor	Negative	NX	MX	NA	NA	NA	NA	NA	NA
118E7982A2	ECC	58	Male	CSYS	II	primary tumor	moderate	NA	N1	M0	NA	NA	NA	NA	NA	NA
118F7997A1	ICC	48	Female	CSYS	III+IV	primary tumor	moderate	Negative	N0	M0	NA	NA	NA	NA	NA	NA
118F8082A1	ICC	68	Male	CSYS	III+IV	metastatic tumor	moderate	Negative	NX	M1	NA	NA	NA	NA	NA	NA
118F8090A1	ICC	54	Female	CSYS	NA	primary tumor	poor	Negative	NX	MX	NA	NA	NA	NA	NA	NA
118F8094A1	ECC	47	Male	CSYS	II	primary tumor	moderate	Negative	N1	M0	NA	NA	NA	NA	NA	NA
118F8096A1	ECC	59	Male	CSYS	II	primary tumor	poor	Negative	N1	M0	NA	NA	NA	NA	NA	NA
118F8114A1	GBC	52	Male	CSYS	III+IV	metastatic tumor	poor	Negative	N1	M0	NA	NA	NA	NA	NA	NA
118F8152A1	ICC	59	Male	CSYS	III+IV	primary tumor	poor	Positive	N1	M0	NA	NA	NA	NA	NA	NA
118F8157A1	ICC	58	Female	CSYS	III+IV	metastatic tumor	NA	Negative	NX	M1	NA	NA	NA	NA	NA	NA
118F8167A1	ECC	44	Male	CSYS	III+IV	metastatic tumor	NA	Negative	NX	M1	NA	NA	NA	NA	NA	NA
118F8170A1	GBC	55	Female	CSYS	III+IV	metastatic tumor	NA	NA	NX	M1	NA	NA	NA	NA	NA	NA
118F8189A1	ICC	61	Female	CSYS	III+IV	primary tumor	poor	Negative	N0	M1	NA	NA	NA	NA	NA	NA
118F8201A1	ICC	75	Female	CSYS	III+IV	primary tumor	poor	Negative	N0	M0	NA	NA	NA	NA	NA	NA
118F8208A1	ECC	35	Female	CSYS	NA	primary tumor	moderate	Negative	NX	MX	NA	NA	NA	NA	NA	NA
118F8237A1	ICC	61	Male	CSYS	II	primary tumor	poor	Negative	N0	M0	NA	NA	NA	NA	NA	NA
118F8276A1	ICC	57	Female	CSYS	III+IV	primary tumor	moderate	NA	N0	M0	NA	NA	NA	NA	NA	NA
118F8372A1	GBC	67	Female	CSYS	II	primary tumor	poor	NA	N0	M0	NA	NA	NA	NA	NA	NA
118F8377A1	GBC	72	Female	CSYS	III+IV	primary tumor	poor	Negative	N1	M0	NA	NA	NA	NA	NA	NA
118F8392A1	ECC	64	Male	CSYS	II	primary tumor	moderate	Negative	N1	M0	NA	NA	NA	NA	NA	NA
118F8415A1	ECC	55	Female	CSYS	III+IV	metastatic tumor	moderate	Negative	NX	M1	NA	NA	NA	NA	NA	NA
118F8458A1	ICC	42	Male	CSYS	III+IV	primary tumor	poor	Negative	N1	M0	NA	NA	NA	NA	NA	NA
118F8489A1	ICC	47	Male	CSYS	III+IV	metastatic tumor	NA	Negative	NX	M1	NA	NA	NA	NA	NA	NA
118F8522A1	ICC	68	Female	CSYS	I	primary tumor	poor	Negative	N0	M0	NA	NA	NA	NA	NA	NA
118F8546A1	ICC	46	Male	CSYS	NA	primary tumor	poor	Negative	NX	MX	NA	NA	NA	NA	NA	NA
118F8558A1	ICC	67	Male	CSYS	III+IV	primary tumor	moderate	Negative	N0	M0	NA	NA	NA	NA	NA	NA
118F8601A1	ICC	50	Male	CSYS	III+IV	primary tumor	moderate	Negative	N0	M0	NA	NA	NA	NA	NA	NA
118F8632A1	GBC	66	Female	CSYS	III+IV	primary tumor	poor	Negative	N0	M0	NA	NA	NA	NA	NA	NA
118F8646A1	ECC	82	Female	CSYS	III+IV	primary tumor	poor	Negative	N1	M1	NA	NA	NA	NA	NA	NA
118F8770A1	ICC	55	Female	CSYS	III+IV	primary tumor	moderate	Negative	N1	M0	NA	NA	NA	NA	NA	NA
118F8792A1	ICC	64	Male	CSYS	III+IV	primary tumor	poor	Positive	N0	M0	NA	NA	NA	NA	NA	NA
118F8797A1	ICC	55	Male	CSYS	III+IV	primary tumor	poor	Negative	N1	M0	NA	NA	NA	NA	NA	NA
118F8813A2	ECC	72	Male	CSYS	I	primary tumor	well	Positive	N0	M0	NA	NA	NA	NA	NA	NA
118F8844A1	GBC	62	Male	CSYS	III+IV	metastatic tumor	moderate	Negative	N1	M0	NA	NA	NA	NA	NA	NA
118F8850A1	ICC	60	Female	CSYS	III+IV	primary tumor	poor	Negative	N1	M0	NA	NA	NA	NA	NA	NA
118F8903A1	GBC	59	Male	CSYS	III+IV	primary tumor	moderate	Negative	N1	M0	NA	NA	NA	NA	NA	NA
118G1224A1	ICC	39	Male	CSYS	III+IV	primary tumor	moderate	Negative	N1	M1	NA	NA	NA	NA	NA	NA
118G1344A1	ICC	57	Male	CSYS	III+IV	primary tumor	poor	Negative	N0	M0	NA	NA	NA	NA	NA	NA
118G1348A1	GBC	48	Female	CSYS	II	primary tumor	poor	Negative	N0	M0	NA	NA	NA	NA	NA	NA
118G8977A1	ICC	44	Male	CSYS	NA	primary tumor	poor	Positive	NX	MX	NA	NA	NA	NA	NA	NA
118G9081A1	ICC	59	Male	CSYS	III+IV	primary tumor	moderate	Positive	N1	M1	NA	NA	NA	NA	NA	NA
118G9119A1	ICC	46	Female	CSYS	I	primary tumor	poor	Negative	N0	M0	NA	NA	NA	NA	NA	NA
118G9120A1	ECC	52	Female	CSYS	NA	primary tumor	poor	Negative	NX	MX	NA	NA	NA	NA	NA	NA
118G9233A1	ICC	71	Male	CSYS	III+IV	primary tumor	poor	Negative	N0	M0	NA	NA	NA	NA	NA	NA
118G9262A1	ICC	51	Female	CSYS	I	primary tumor	poor	Negative	N0	M0	NA	NA	NA	NA	NA	NA
118G9348A1	GBC	55	Male	CSYS	II	primary tumor	moderate	Negative	N0	M0	NA	NA	NA	NA	NA	NA
118G9364A1	ICC	65	Male	CSYS	III+IV	primary tumor	poor	Negative	NX	M1	NA	NA	NA	NA	NA	NA
118G9391A1	ICC	65	Male	CSYS	III+IV	primary tumor	poor	NA	N0	M0	NA	NA	NA	NA	NA	NA
118G9397A1	GBC	47	Male	CSYS	II	primary tumor	moderate	Negative	N0	M0	NA	NA	NA	NA	NA	NA
118G9417A2	ECC	67	Male	CSYS	II	primary tumor	moderate	NA	N0	M0	NA	NA	NA	NA	NA	NA
118G9421A1	ICC	71	Male	CSYS	III+IV	primary tumor	poor	Negative	N1	M0	NA	NA	NA	NA	NA	NA
118G9423A1	GBC	75	Female	CSYS	III+IV	primary tumor	poor	Negative	N0	M0	NA	NA	NA	NA	NA	NA
118G9448A1	GBC	78	Male	CSYS	III+IV	metastatic tumor	poor	NA	NX	M1	NA	NA	NA	NA	NA	NA
118G9512A1	ECC	66	Male	CSYS	III+IV	primary tumor	moderate	Negative	N1	M0	NA	NA	NA	NA	NA	NA
118G9558J1	GBC	54	Female	CSYS	III+IV	primary tumor	moderate	Negative	N0	M1	NA	NA	NA	NA	NA	NA
118G9559A1	ECC	68	Female	CSYS	II	primary tumor	moderate	Negative	N0	M0	NA	NA	NA	NA	NA	NA
118G9581A1	ICC	55	Female	CSYS	NA	primary tumor	NA	Negative	NX	MX	NA	NA	NA	NA	NA	NA
118G9603A1	GBC	54	Female	CSYS	II	primary tumor	moderate	Negative	N0	M0	NA	NA	NA	NA	NA	NA
118G9617A1	ICC	24	Male	CSYS	III+IV	primary tumor	moderate	Positive	NX	M1	NA	NA	NA	NA	NA	NA
118G9676A1	ICC	61	Male	CSYS	III+IV	primary tumor	moderate	Positive	NX	MX	NA	NA	NA	NA	NA	NA
118G9722A1	ICC	44	Male	CSYS	I	primary tumor	moderate	Negative	N0	M0	NA	NA	NA	NA	NA	NA
118G9724A1	ICC	56	Female	CSYS	I	primary tumor	moderate	Negative	N0	M0	NA	NA	NA	NA	NA	NA
118G9729A1	ICC	37	Male	CSYS	NA	primary tumor	poor	Positive	NX	MX	NA	NA	NA	NA	NA	NA
118G9742A1	ICC	65	Male	CSYS	III+IV	primary tumor	poor	Negative	N1	M0	NA	NA	NA	NA	NA	NA
118G9781A1	ICC	61	Male	CSYS	III+IV	primary tumor	poor	Positive	N1	M0	NA	NA	NA	NA	NA	NA

118G9797A1	GBC	47	Female	CSYS	II	primary tumor	poor	Negative	N0	M0	NA	NA	NA	NA	NA	NA
118G9912A1	GBC	64	Male	CSYS	III+IV	primary tumor	poor	Negative	N0	M0	NA	NA	NA	NA	NA	NA
118G9955A1	GBC	50	Male	CSYS	I	primary tumor	poor	Negative	N0	M0	NA	NA	NA	NA	NA	NA
118G9970A1	ECC	49	Male	CSYS	NA	primary tumor	moderate	Negative	NX	MX	NA	NA	NA	NA	NA	NA
118H1438M1	ICC	56	Female	CSYS	I	primary tumor	moderate	Negative	N0	M0	NA	NA	NA	NA	NA	NA
118H1479A1	ICC	69	Female	CSYS	III+IV	primary tumor	poor	Negative	N1	M0	NA	NA	NA	NA	NA	NA
118H1515A1	GBC	67	Male	CSYS	III+IV	primary tumor	moderate	Negative	N1	M0	NA	NA	NA	NA	NA	NA
118H1577A1	ICC	74	Male	CSYS	III+IV	primary tumor	NA	Negative	NX	M1	NA	NA	NA	NA	NA	NA
118H1607A1	ICC	35	Male	CSYS	III+IV	primary tumor	poor	Negative	N0	M0	NA	NA	NA	NA	NA	NA
118H1642A1	ECC	65	Male	CSYS	III+IV	primary tumor	poor	Negative	N1	M0	NA	NA	NA	NA	NA	NA
118H1685A1	ICC	55	Male	CSYS	II	primary tumor	poor	Negative	N0	M0	NA	NA	NA	NA	NA	NA
118H1689A1	ICC	69	Male	CSYS	III+IV	metastatic tumor	NA	Negative	NX	M1	NA	NA	NA	NA	NA	NA
118H1733A1	ECC	43	Male	CSYS	NA	primary tumor	poor	Negative	NX	MX	NA	NA	NA	NA	NA	NA
118H1787A1	GBC	59	Female	CSYS	III+IV	metastatic tumor	NA	Negative	NX	M1	NA	NA	NA	NA	NA	NA
118H1840A1	ICC	65	Male	CSYS	NA	primary tumor	poor	Negative	NX	MX	NA	NA	NA	NA	NA	NA
118H2136A1	ECC	63	Female	CSYS	III+IV	metastatic tumor	moderate	Negative	NX	M1	NA	NA	NA	NA	NA	NA
118H2149S1	ICC	73	Male	CSYS	III+IV	primary tumor	moderate	NA	N1	M1	NA	NA	NA	NA	NA	NA
118H2157A1	ICC	53	Male	CSYS	NA	primary tumor	poor	Positive	NX	MX	NA	NA	NA	NA	NA	NA
118H2438A1	ECC	73	Female	CSYS	II	primary tumor	moderate	Negative	N0	M0	NA	NA	NA	NA	NA	NA
118H2472A1	ICC	66	Male	CSYS	NA	primary tumor	moderate	Negative	NX	MX	NA	NA	NA	NA	NA	NA
118H2473A1	ECC	78	Male	CSYS	I	primary tumor	moderate	Negative	N0	M0	NA	NA	NA	NA	NA	NA
118H2475M1	ECC	55	Male	CSYS	III+IV	primary tumor	moderate	Negative	N1	M0	NA	NA	NA	NA	NA	NA
118H2476A1	ICC	55	Male	CSYS	III+IV	primary tumor	moderate	Positive	NX	M1	NA	NA	NA	NA	NA	NA
118H2503A1	GBC	60	Male	CSYS	III+IV	primary tumor	moderate	Negative	N1	M1	NA	NA	NA	NA	NA	NA
118H2531A3	GBC	27	Female	CSYS	NA	metastatic tumor	NA	NA	NX	MX	NA	NA	NA	NA	NA	NA
118H2540A1	ECC	58	Male	CSYS	III+IV	primary tumor	poor	Positive	N1	M0	NA	NA	NA	NA	NA	NA
118H2588A1	ECC	78	Male	CSYS	NA	primary tumor	poor	Negative	NX	MX	NA	NA	NA	NA	NA	NA
118H2593A1	ICC	74	Female	CSYS	I	primary tumor	moderate	Positive	N0	M0	NA	NA	NA	NA	NA	NA
118H2626A1	ICC	65	Male	CSYS	III+IV	recurrent tumor	NA	Positive	NX	MX	NA	NA	NA	NA	NA	NA
118H2716A1	ICC	58	Male	CSYS	III+IV	primary tumor	poor	Negative	N1	M0	NA	NA	NA	NA	NA	NA
118H2758A1	ECC	54	Female	CSYS	I	primary tumor	moderate	Negative	N0	M0	NA	NA	NA	NA	NA	NA
118I2919A1	ICC	56	Male	CSYS	III+IV	metastatic tumor	NA	Positive	NX	M1	NA	NA	NA	NA	NA	NA
118I2978M1	ICC	69	Male	CSYS	III+IV	metastatic tumor	moderate	Negative	N0	M0	NA	NA	NA	NA	NA	NA
118I3162A1	ICC	63	Male	CSYS	III+IV	primary tumor	moderate	Positive	N1	M0	NA	NA	NA	NA	NA	NA
118I3202A1	ICC	55	Female	CSYS	III+IV	recurrent tumor	moderate	Negative	NX	MX	NA	NA	NA	NA	NA	NA
118I3402A1	ICC	53	Male	CSYS	II	primary tumor	poor	Positive	N0	M0	NA	NA	NA	NA	NA	NA
118I3426A1	ECC	67	Female	CSYS	I	primary tumor	moderate	NA	N0	M0	NA	NA	NA	NA	NA	NA
118I3457A2	ICC	57	Female	CSYS	NA	primary tumor	moderate	Positive	NX	MX	NA	NA	NA	NA	NA	NA
118I3472M1	GBC	47	Female	CSYS	III+IV	primary tumor	moderate	Negative	N1	M0	NA	NA	NA	NA	NA	NA
118I3488A1	ICC	67	Female	CSYS	III+IV	primary tumor	poor	Positive	N1	M0	NA	NA	NA	NA	NA	NA
118I3510A1	ICC	55	Female	CSYS	III+IV	primary tumor	poor	NA	N1	M0	NA	NA	NA	NA	NA	NA
118I3529A1	ECC	73	Male	CSYS	II	primary tumor	poor	Positive	N0	M0	NA	NA	NA	NA	NA	NA
118I3532A1	ICC	59	Male	CSYS	III+IV	primary tumor	poor	Positive	N1	M0	NA	NA	NA	NA	NA	NA
118I3573A1	GBC	61	Female	CSYS	III+IV	metastatic tumor	moderate	Negative	NX	M1	NA	NA	NA	NA	NA	NA
118I3576A1	ICC	65	Male	CSYS	II	primary tumor	poor	Positive	N0	M0	NA	NA	NA	NA	NA	NA
118I3592A2	ECC	63	Male	CSYS	NA	primary tumor	poor	NA	N1	MX	NA	NA	NA	NA	NA	NA
118I3607A2	ICC	60	Female	CSYS	III+IV	primary tumor	poor	Positive	N0	M0	NA	NA	NA	NA	NA	NA
118I3629A1	ECC	59	Female	CSYS	III+IV	primary tumor	moderate	Negative	N1	M0	NA	NA	NA	NA	NA	NA
118I3652A1	ICC	75	Male	CSYS	III+IV	primary tumor	NA	Positive	N1	M1	NA	NA	NA	NA	NA	NA
118I3743M1	GBC	64	Female	CSYS	III+IV	metastatic tumor	NA	Negative	NX	M1	NA	NA	NA	NA	NA	NA
118I3752D1	ICC	53	Male	CSYS	NA	primary tumor	poor	Positive	NX	MX	NA	NA	NA	NA	NA	NA
118I3763A1	GBC	65	Male	CSYS	II	primary tumor	poor	Negative	N0	M0	NA	NA	NA	NA	NA	NA
118I3817A1	ICC	43	Male	CSYS	III+IV	primary tumor	poor	Positive	N0	M0	NA	NA	NA	NA	NA	NA
118I3847A1	ICC	63	Male	CSYS	III+IV	primary tumor	poor	Negative	N0	M0	NA	NA	NA	NA	NA	NA
118I3850D1	ECC	69	Female	CSYS	I	primary tumor	moderate	Positive	N0	M0	NA	NA	NA	NA	NA	NA
118I3860A1	ICC	62	Male	CSYS	III+IV	primary tumor	poor	Negative	N1	M0	NA	NA	NA	NA	NA	NA
118I3960A1	GBC	72	Male	CSYS	III+IV	primary tumor	poor	NA	N0	M0	NA	NA	NA	NA	NA	NA
118I3967M1	ICC	75	Male	CSYS	NA	primary tumor	poor	Negative	NX	MX	NA	NA	NA	NA	NA	NA
118I4062A1	ICC	69	Male	CSYS	III+IV	recurrent tumor	moderate	Negative	NX	MX	NA	NA	NA	NA	NA	NA
118J4224A1	ICC	57	Male	CSYS	III+IV	recurrent tumor	poor	Positive	NX	MX	NA	NA	NA	NA	NA	NA
118J4228A1	ICC	62	Female	CSYS	III+IV	primary tumor	moderate	Negative	N1	M0	NA	NA	NA	NA	NA	NA
118J4287M1	ICC	55	Male	CSYS	I	primary tumor	moderate	Positive	N0	M0	NA	NA	NA	NA	NA	NA
118J4299M1	GBC	68	Female	CSYS	NA	metastatic tumor	poor	NA	NX	MX	NA	NA	NA	NA	NA	NA
118J4384A1	ICC	69	Female	CSYS	NA	primary tumor	NA	Positive	NX	MX	NA	NA	NA	NA	NA	NA
118J4450M1	ICC	54	Male	CSYS	III+IV	primary tumor	moderate	Negative	NX	M1	NA	NA	NA	NA	NA	NA
118J4625A1	ICC	60	Male	CSYS	III+IV	primary tumor	poor	Negative	N1	M0	NA	NA	NA	NA	NA	NA
118J4646M1	ICC	60	Male	CSYS	III+IV	primary tumor	poor	Negative	N0	M0	NA	NA	NA	NA	NA	NA
118J4748M1	ICC	63	Male	CSYS	III+IV	primary tumor	poor	Positive	N0	M0	NA	NA	NA	NA	NA	NA
118J4750A1	ICC	55	Male	CSYS	III+IV	metastatic tumor	poor	Negative	N1	M0	NA	NA	NA	NA	NA	NA
118J4755M1	ICC	45	Male	CSYS	II	primary tumor	poor	Positive	N0	M0	NA	NA	NA	NA	NA	NA
118J4761A1	ICC	69	Male	CSYS	III+IV	primary tumor	poor	Positive	N1	M0	NA	NA	NA	NA	NA	NA
118J4779J1	ECC	51	Male	CSYS	II	primary tumor	poor	NA	N0	M0	NA	NA	NA	NA	NA	NA
118J4845A1	ICC	66	Male	CSYS	II	primary tumor	moderate	Positive	N0	M0	NA	NA	NA	NA	NA	NA
118J4887A1	GBC	58	Female	CSYS	III+IV	primary tumor	poor	Negative	N0	M0	NA	NA	NA	NA	NA	NA
118J4963M1	GBC	56	Female	CSYS	NA	primary tumor	NA	Positive	N1	MX	NA	NA	NA	NA	NA	NA
118J5065A1	ICC	63	Male	CSYS	III+IV	primary tumor	poor	Positive	NX	MX	NA	NA	NA	NA	NA	NA
118J5083M1	ICC	42	Female	CSYS	III+IV	primary tumor	moderate	NA	N1	M1	NA	NA	NA	NA	NA	NA
118J5085M1	ICC	51	Female	CSYS	NA	primary tumor	poor	Positive	NX	MX	NA	NA	NA	NA	NA	NA
118J5119A1	ECC	58	Male	CSYS	III+IV	metastatic tumor	moderate	Negative	NX	M1	NA	NA	NA	NA	NA	NA
118J5135A1	ICC	74	Male	CSYS	III+IV	primary tumor	poor	Negative	N0	M0	NA	NA	NA	NA	NA	NA
118J5144A1	GBC	38	Female	CSYS	III+IV	primary tumor	moderate	Negative	N0	M0	NA	NA	NA	NA	NA	NA
118J5169M1	ICC	75	Female	CSYS	III+IV	primary tumor	moderate	Negative	NX	M1	NA	NA	NA	NA	NA	NA
118J5206M1	ECC	39	Female	CSYS	III+IV	metastatic tumor	poor	Positive	NX	M1	NA	NA	NA	NA	NA	NA
118J5249M1	ECC	59	Female	CSYS	II	primary tumor	poor	Positive	N1	M0	NA	NA	NA	NA	NA	NA
118J5276A1	ECC	55	Female	CSYS	I	primary tumor	poor	Positive	N0	M0	NA	NA	NA	NA	NA	NA
118J5402A1	ICC	50	Male	CSYS	III+IV	metastatic tumor	poor	Positive	N1	M0	NA	NA	NA	NA	NA	NA
118J5518M1	ICC	34	Male	CSYS	III+IV	primary tumor	poor	Positive	NX	M1	NA	NA	NA	NA	NA	NA
118J5583A1	ICC	67	Male	CSYS	NA	primary tumor	poor	Positive	NX	MX	NA	NA	NA	NA	NA	NA
118K5630A1	ICC	65	Male	CSYS	III+IV	primary tumor	moderate	Positive	N1	M0	NA	NA	NA	NA	NA	NA
118K5635M1	ECC	54	Male	CSYS	II	primary tumor	moderate	Negative	N0	M0	NA	NA	NA	NA	NA	NA
118K5684M1	GBC	74	Male	CSYS	III+IV	metastatic tumor	moderate	Positive	NX	M1	NA	NA	NA	NA	NA	NA
118K5718A1	ICC	66	Male	CSYS	II	primary tumor	poor	Positive	N0	M0	NA	NA	NA	NA	NA	NA
118K5909A1	GBC	67	Female	CSYS	NA	primary tumor	moderate	Positive	NX	MX	NA	NA	NA	NA	NA	NA
118K5971J1	ICC	42	Male	CSYS	I	primary tumor	moderate	Positive	N0	M0	NA	NA	NA	NA	NA	NA
118K5984A1	ICC	56	Female	CSYS	III+IV	primary tumor	moderate	Positive	NX	M1	NA	NA	NA	NA	NA	NA
118K5992M1	ICC	60	Male	CSYS	NA	primary tumor	NA	Positive	NX	MX	NA	NA	NA	NA	NA	NA
118K5993A1	GBC	52	Female	CSYS	III+IV	recurrent tumor	NA	Positive	NX	MX	NA	NA	NA	NA	NA	NA
118K6072M1	ICC	56	Male	CSYS	III+IV	primary tumor	moderate	Positive	N1	M0	NA	NA	NA	NA	NA	NA
118K6080A1	GBC	68	Female	CSYS	III+IV	primary tumor	moderate	Positive	N1	M0	NA	NA	NA	NA	NA	NA
118K6130M1	ICC	52	Female	CSYS	I	primary tumor	poor	Negative	N0	M0	NA	NA	NA	NA	NA	NA
118K6186A1	ICC	63	Male	CSYS	III+IV	primary tumor	moderate	NA	N0	M0	NA	NA	NA	NA	NA	NA
118K6209A1	ICC	69	Female	CSYS	NA	primary tumor	moderate	Negative	NX	MX	NA	NA	NA	NA	NA	NA
118K6272A1	GBC	52	Female	CSYS	II	primary tumor	moderate	Positive	N0	M0	NA	NA	NA	NA	NA	NA
118K6285M1	GBC	66	Female	CSYS	III+IV	primary tumor	poor	Negative	N0	M0	NA	NA	NA	NA	NA	NA
118K6312M1	GBC	54	Female	CSYS	NA	primary tumor	moderate	Negative	NX	M0	NA	NA	NA	NA	NA	NA
118K6336A1	ECC	53	Male	CSYS	II	primary tumor	moderate	Negative	N0	M0	NA	NA	NA	NA	NA	NA
118K6349A1	ECC	74	Female	CSYS	II	primary tumor	moderate	Negative	N0	M0	NA	NA	NA	NA	NA	NA
118K6372A3	ICC	68	Male	CSYS	III+IV	primary tumor	poor	Negative	N1	M0	NA	NA	NA	NA	NA	NA
118K6377A1	GBC	68	Male	CSYS	II	primary tumor	well	Negative	N0	M0	NA	NA	NA	NA	NA	NA
118K6454A1	ICC	61	Female	CSYS	NA	primary tumor	NA	Negative	NX	MX	NA	NA	NA	NA	NA	NA
118K6479M1	GBC	56	Male	CSYS	III+IV	primary tumor	poor	Positive	NX	M1	NA	NA	NA	NA	NA	NA
118K6509A1	GBC	48	Female	CSYS	I	primary tumor	moderate	Negative	N0	M0	NA	NA	NA	NA	NA	NA
118K6518A1	ICC	62	Female	CSYS	II	primary tumor	moderate	Negative	N0	M0	NA	NA	NA	NA	NA	NA
118K6557A1	GBC	68	Male	CSYS	III+IV	primary tumor	well	Negative	N0	M1	NA	NA	NA	NA	NA	NA
118K6580A1	GBC	72	Male	CSYS	NA	primary tumor	moderate	Positive	NX	MX	NA	NA	NA	NA	NA	NA

118K6595M2	GBC	56	Female	CSYS	III+IV	primary tumor	NA	Positive	NX	M1	NA	NA	NA	NA	NA	NA
118K6631A1	ICC	46	Male	CSYS	III+IV	primary tumor	moderate	Positive	N1	M0	NA	NA	NA	NA	NA	NA
118K6637M1	ECC	74	Female	CSYS	NA	primary tumor	NA	Negative	NX	MX	NA	NA	NA	NA	NA	NA
118K6653A1	GBC	68	Male	CSYS	III+IV	metastatic tumor	NA	Negative	NX	M1	NA	NA	NA	NA	NA	NA
118K6674A1	ECC	71	Male	CSYS	III+IV	metastatic tumor	poor	Negative	N1	M0	NA	NA	NA	NA	NA	NA
118K6703A1	ICC	61	Male	CSYS	II	primary tumor	moderate	Negative	N0	M0	NA	NA	NA	NA	NA	NA
118K6789A1	ICC	65	Female	CSYS	III+IV	primary tumor	moderate	Positive	N1	M1	NA	NA	NA	NA	NA	NA
118K6855M1	ECC	53	Male	CSYS	I	primary tumor	poor	Negative	N0	M0	NA	NA	NA	NA	NA	NA
118K6924A1	ICC	62	Female	CSYS	I	primary tumor	poor	Negative	N0	M0	NA	NA	NA	NA	NA	NA
118K6958A1	GBC	66	Male	CSYS	III+IV	metastatic tumor	NA	Negative	NX	M1	NA	NA	NA	NA	NA	NA
118K6975A1	ECC	65	Female	CSYS	II	primary tumor	moderate	Positive	N0	M0	NA	NA	NA	NA	NA	NA
118K6977A1	GBC	64	Female	CSYS	III+IV	metastatic tumor	poor	Negative	NX	M1	NA	NA	NA	NA	NA	NA
118K6986A1	ICC	53	Male	CSYS	III+IV	primary tumor	poor	Positive	NX	MX	NA	NA	NA	NA	NA	NA
118K7052M1	ICC	46	Female	CSYS	III+IV	primary tumor	moderate	Positive	N0	M1	NA	NA	NA	NA	NA	NA
118K7082A1	ICC	62	Female	CSYS	III+IV	primary tumor	poor	Negative	N1	M0	NA	NA	NA	NA	NA	NA
118K7083M1	ICC	65	Female	CSYS	III+IV	primary tumor	moderate	Positive	N0	M0	NA	NA	NA	NA	NA	NA
118L7106A1	GBC	59	Male	CSYS	I	primary tumor	moderate	Negative	N0	M0	NA	NA	NA	NA	NA	NA
118L7131A1	ECC	72	Male	CSYS	III+IV	metastatic tumor	moderate	Negative	NX	M1	NA	NA	NA	NA	NA	NA
118L7133A1	ICC	40	Male	CSYS	NA	primary tumor	poor	Positive	NX	MX	NA	NA	NA	NA	NA	NA
118L7161A1	GBC	62	Female	CSYS	I	primary tumor	moderate	Negative	N0	M0	NA	NA	NA	NA	NA	NA
118L7198A1	ICC	56	Male	CSYS	III+IV	primary tumor	poor	Negative	N0	M0	NA	NA	NA	NA	NA	NA
118L7202A1	GBC	45	Female	CSYS	III+IV	metastatic tumor	moderate	Negative	NX	M1	NA	NA	NA	NA	NA	NA
118L7212A1	ICC	69	Female	CSYS	I	primary tumor	poor	Negative	N0	M0	NA	NA	NA	NA	NA	NA
118L7229A1	GBC	71	Female	CSYS	I	primary tumor	moderate	Negative	N0	M0	NA	NA	NA	NA	NA	NA
118L7236A1	ICC	47	Female	CSYS	III+IV	primary tumor	NA	Positive	NX	M1	NA	NA	NA	NA	NA	NA
118L7265A2	ECC	49	Male	CSYS	II	primary tumor	moderate	Negative	N0	MX	NA	NA	NA	NA	NA	NA
118L7275A1	ECC	63	Female	CSYS	III+IV	primary tumor	poor	Positive	NX	M1	NA	NA	NA	NA	NA	NA
118L7370A1	ICC	65	Female	CSYS	III+IV	primary tumor	moderate	Positive	N1	M0	NA	NA	NA	NA	NA	NA
118L7386A1	ICC	56	Male	CSYS	III+IV	primary tumor	moderate	Negative	N1	M0	NA	NA	NA	NA	NA	NA
118L7404M1	GBC	61	Female	CSYS	III+IV	metastatic tumor	poor	Negative	NX	M1	NA	NA	NA	NA	NA	NA
118L7413A1	ECC	60	Female	CSYS	III+IV	metastatic tumor	poor	Positive	NX	M1	NA	NA	NA	NA	NA	NA
118L7497M1	ICC	70	Male	CSYS	III+IV	primary tumor	NA	Positive	NX	M1	NA	NA	NA	NA	NA	NA
118L7516M1	ICC	71	Female	CSYS	II	primary tumor	moderate	Positive	N0	M0	NA	NA	NA	NA	NA	NA
118L7537A1	ECC	51	Male	CSYS	II	primary tumor	poor	Positive	N1	M0	NA	NA	NA	NA	NA	NA
118L7560M1	ICC	56	Male	CSYS	I	primary tumor	moderate	Negative	N0	M0	NA	NA	NA	NA	NA	NA
118L7574A1	ICC	56	Female	CSYS	III+IV	primary tumor	poor	Negative	N1	M0	NA	NA	NA	NA	NA	NA
118L7607A1	ICC	63	Male	CSYS	NA	primary tumor	poor	Negative	NX	MX	NA	NA	NA	NA	NA	NA
118L7654M1	ICC	68	Male	CSYS	NA	primary tumor	moderate	Positive	NX	MX	NA	NA	NA	NA	NA	NA
118L7656M1	ICC	62	Male	CSYS	I	primary tumor	moderate	Negative	N0	M0	NA	NA	NA	NA	NA	NA
118L7756A1	ICC	47	Male	CSYS	I	primary tumor	moderate	Negative	N0	M0	NA	NA	NA	NA	NA	NA
118L7757A1	ICC	39	Male	CSYS	I	primary tumor	poor	Positive	N0	M0	NA	NA	NA	NA	NA	NA
118L7820J1	ICC	31	Male	CSYS	NA	primary tumor	poor	Negative	NX	MX	NA	NA	NA	NA	NA	NA
118L7848M1	ICC	69	Female	CSYS	III+IV	primary tumor	moderate	Positive	N1	M0	NA	NA	NA	NA	NA	NA
118L7853A1	ECC	53	Male	CSYS	II	primary tumor	poor	Negative	N0	M0	NA	NA	NA	NA	NA	NA
118L7860A1	GBC	82	Male	CSYS	III+IV	primary tumor	poor	Negative	N1	M1	NA	NA	NA	NA	NA	NA
118L7867A1	ICC	43	Female	CSYS	III+IV	primary tumor	moderate	Positive	NX	M1	NA	NA	NA	NA	NA	NA
118L7895A1	ECC	30	Male	CSYS	III+IV	metastatic tumor	poor	Positive	NX	M1	NA	NA	NA	NA	NA	NA
118L8016M1	ECC	33	Male	CSYS	I	primary tumor	moderate	Negative	N0	M0	NA	NA	NA	NA	NA	NA
118L8023M2	ICC	74	Male	CSYS	III+IV	metastatic tumor	poor	Negative	NX	M1	NA	NA	NA	NA	NA	NA
118L8029M1	ICC	55	Male	CSYS	III+IV	primary tumor	moderate	Negative	N0	M0	NA	NA	NA	NA	NA	NA
118L8044A1	ECC	64	Female	CSYS	III+IV	primary tumor	poor	Positive	NX	M1	NA	NA	NA	NA	NA	NA
118L8083A1	ICC	59	Male	CSYS	I	primary tumor	poor	Positive	N0	M0	NA	NA	NA	NA	NA	NA
118L8213A1	ICC	66	Female	CSYS	I	recurrent tumor	poor	Negative	N0	M0	NA	NA	NA	NA	NA	NA
118L8367M1	GBC	49	Female	CSYS	III+IV	primary tumor	poor	Negative	N0	M0	NA	NA	NA	NA	NA	NA
118L8370M1	ECC	64	Male	CSYS	I	primary tumor	moderate	Negative	N0	M0	NA	NA	NA	NA	NA	NA
118L8457A1	ICC	32	Male	CSYS	III+IV	primary tumor	poor	Positive	N0	M0	NA	NA	NA	NA	NA	NA
118L8475A1	ICC	52	Female	CSYS	III+IV	primary tumor	poor	Positive	N0	M0	NA	NA	NA	NA	NA	NA
118L8606A1	GBC	60	Male	CSYS	III+IV	primary tumor	poor	Negative	N1	MX	NA	NA	NA	NA	NA	NA
118L8638M2	ICC	51	Female	CSYS	I	primary tumor	moderate	Negative	N0	M0	NA	NA	NA	NA	NA	NA
118L8642M1	GBC	64	Female	CSYS	III+IV	metastatic tumor	poor	Negative	N0	M1	NA	NA	NA	NA	NA	NA
118L8875M1	ICC	63	Female	CSYS	III+IV	primary tumor	moderate	Positive	N0	M0	NA	NA	NA	NA	NA	NA
118L8884A1	ECC	54	Male	CSYS	II	primary tumor	poor	NA	N0	M0	NA	NA	NA	NA	NA	NA
118L8913A1	ECC	73	Male	CSYS	II	primary tumor	moderate	Positive	N0	M0	NA	NA	NA	NA	NA	NA
118L8983A1	ECC	73	Male	CSYS	II	primary tumor	poor	Negative	N0	M0	NA	NA	NA	NA	NA	NA
118L8989A1	ECC	67	Male	CSYS	II	primary tumor	poor	Positive	N0	M0	NA	NA	NA	NA	NA	NA
118L9109A2	ICC	54	Female	CSYS	III+IV	primary tumor	poor	Negative	N1	M0	NA	NA	NA	NA	NA	NA
118L9110M1	ICC	51	Male	CSYS	II	primary tumor	moderate	Positive	N0	M0	NA	NA	NA	NA	NA	NA
119A1499J1	ICC	60	Male	CSYS	NA	primary tumor	poor	Positive	NX	MX	NA	NA	NA	NA	NA	NA
119A1568M1	ECC	61	Male	CSYS	III+IV	metastatic tumor	moderate	Positive	NX	M1	NA	NA	NA	NA	NA	NA
119A1621M1	GBC	65	Female	CSYS	III+IV	primary tumor	moderate	Negative	N0	M0	NA	NA	NA	NA	NA	NA
119A1715M1	ICC	73	Female	CSYS	III+IV	primary tumor	poor	Negative	NX	MX	NA	NA	NA	NA	NA	NA
119A1725M1	ICC	71	Female	CSYS	III+IV	primary tumor	poor	Negative	NX	M1	NA	NA	NA	NA	NA	NA
119A1933A1	ICC	66	Female	CSYS	NA	primary tumor	poor	Negative	NX	MX	NA	NA	NA	NA	NA	NA
119A1949A1	ICC	65	Female	CSYS	III+IV	primary tumor	poor	Negative	N1	MX	NA	NA	NA	NA	NA	NA
119A9209D1	ECC	62	Male	CSYS	II	primary tumor	moderate	Negative	N1	M0	NA	NA	NA	NA	NA	NA
119A9336A1	ECC	58	Male	CSYS	III+IV	primary tumor	moderate	Negative	NX	M0	NA	NA	NA	NA	NA	NA
119A9340A1	ECC	46	Male	CSYS	NA	metastatic tumor	poor	Positive	NX	MX	NA	NA	NA	NA	NA	NA
119A9694A1	ICC	67	Male	CSYS	I	primary tumor	moderate	Positive	N0	M0	NA	NA	NA	NA	NA	NA
119A9698A1	GBC	79	Female	CSYS	II	primary tumor	poor	Negative	N0	M0	NA	NA	NA	NA	NA	NA
119A9702A1	ICC	43	Male	CSYS	NA	primary tumor	moderate	Negative	NX	MX	NA	NA	NA	NA	NA	NA
119A9806A1	ECC	72	Male	CSYS	III+IV	primary tumor	moderate	Negative	N1	MX	NA	NA	NA	NA	NA	NA
119A9889A1	ICC	65	Male	CSYS	III+IV	primary tumor	poor	NA	N1	M0	NA	NA	NA	NA	NA	NA
216L0152A1	ICC	72	Male	CSYS	III+IV	primary tumor	NA	Negative	NX	M1	NA	NA	NA	NA	NA	NA
216L0276A1	ICC	61	Female	CSYS	NA	primary tumor	NA	Negative	NX	MX	NA	NA	NA	NA	NA	NA
217A0361A1	ICC	50	Female	CSYS	NA	primary tumor	NA	Negative	N1	M0	NA	NA	NA	NA	NA	NA
217B0420A1	ECC	63	Male	CSYS	III+IV	metastatic tumor	poor	Negative	N0	M0	NA	NA	NA	NA	NA	NA
217B0528A1	ICC	44	Female	CSYS	NA	primary tumor	NA	Negative	NX	MX	NA	NA	NA	NA	NA	NA
217C0588A1	ICC	55	Female	CSYS	III+IV	primary tumor	poor	Positive	N1	M0	NA	NA	NA	NA	NA	NA
217C0745A1	ICC	48	Female	CSYS	III+IV	primary tumor	moderate	Negative	N1	M0	NA	NA	NA	NA	NA	NA
217D0937A1	ICC	56	Male	CSYS	II	primary tumor	moderate	Negative	N0	M0	NA	NA	NA	NA	NA	NA
217E1258A1	ICC	53	Female	CSYS	III+IV	metastatic tumor	poor	Positive	N1	M0	NA	NA	NA	NA	NA	NA
217E1387A1	ICC	65	Female	CSYS	III+IV	recurrent tumor	NA	Positive	NX	MX	NA	NA	NA	NA	NA	NA
217F1558A1	ICC	67	Male	CSYS	NA	primary tumor	NA	Negative	NX	MX	NA	NA	NA	NA	NA	NA
217F1599A1	ECC	54	Female	CSYS	II	primary tumor	poor	Negative	N0	M0	NA	NA	NA	NA	NA	NA
217F1625A1	ICC	88	Female	CSYS	NA	primary tumor	NA	Negative	NX	MX	NA	NA	NA	NA	NA	NA
217F1660A1	GBC	53	Female	CSYS	III+IV	metastatic tumor	NA	Negative	NX	M1	NA	NA	NA	NA	NA	NA
217F1870A1	ICC	83	Male	CSYS	II	metastatic tumor	NA	Negative	N0	M0	NA	NA	NA	NA	NA	NA
217G1915A1	GBC	73	Male	CSYS	III+IV	metastatic tumor	poor	Negative	NX	M1	NA	NA	NA	NA	NA	NA
217G2113A1	ICC	59	Male	CSYS	III+IV	primary tumor	moderate	Negative	N1	M0	NA	NA	NA	NA	NA	NA
217H2225A1	GBC	51	Female	CSYS	NA	primary tumor	poor	Negative	NX	MX	NA	NA	NA	NA	NA	NA
217H2254A1	ICC	57	Male	CSYS	III+IV	primary tumor	poor	NA	N1	M0	NA	NA	NA	NA	NA	NA
217H2400A1	ICC	28	Male	CSYS	NA	primary tumor	NA	Negative	NX	MX	NA	NA	NA	NA	NA	NA
217H2423A1	ECC	59	Male	CSYS	NA	primary tumor	NA	Negative	NX	MX	NA	NA	NA	NA	NA	NA
217H2487A1	ICC	48	Female	CSYS	NA	primary tumor	NA	Negative	NX	MX	NA	NA	NA	NA	NA	NA
217I2570A1	GBC	59	Male	CSYS	III+IV	metastatic tumor	NA	Positive	N0	M1	NA	NA	NA	NA	NA	NA
217I2920A1	GBC	60	Female	CSYS	III+IV	metastatic tumor	poor	Negative	NX	M1	NA	NA	NA	NA	NA	NA
217I2921A1	GBC	80	Female	CSYS	III+IV	metastatic tumor	NA	Negative	NX	M1	NA	NA	NA	NA	NA	NA
217J3201A1	ICC	68	Male	CSYS	III+IV	primary tumor	poor	Negative	N0	M0	NA	NA	NA	NA	NA	NA
217K3355A1	ICC	83	Female	CSYS	NA	primary tumor	NA	Positive	NX	MX	NA	NA	NA	NA	NA	NA
217K3692A1	GBC	77	Female	CSYS	NA	metastatic tumor	NA	Negative	NX	MX	NA	NA	NA	NA	NA	NA
217K3729A1	ICC	71	Male	CSYS	III+IV	primary tumor	poor	Positive	N1	M0	NA	NA	NA	NA	NA	NA
217K3730A1	ICC	54	Female	CSYS	II	primary tumor	poor	Negative	N0	M0	NA	NA	NA	NA	NA	NA
217L3903A1	ICC	50	Male	CSYS	NA	primary tumor	poor	Positive	NX	MX	NA	NA	NA	NA	NA	NA
218A4699A1	ICC	73	Female	CSYS	NA	primary tumor	NA	Positive	NX	MX	NA	NA	NA	NA	NA	NA
218B4967A1	ICC	71	Male	CSYS	NA	primary tumor	NA	Positive	NX	MX	NA	NA	NA	NA	NA	NA

218C5441A1	ICC	56	Female	CSYS	NA	primary tumor	NA	Negative	NX	MX	NA	NA	NA	NA	NA	NA
218C5575A1	ICC	58	Male	CSYS	III+IV	metastatic tumor	poor	Positive	NX	M1	NA	NA	NA	NA	NA	NA
218C5588A2	GBC	44	Female	CSYS	III+IV	metastatic tumor	NA	Negative	NX	M1	NA	NA	NA	NA	NA	NA
218C5780A1	ICC	32	Female	CSYS	NA	primary tumor	NA	Positive	NX	MX	NA	NA	NA	NA	NA	NA
218D5915A1	GBC	58	Male	CSYS	II	primary tumor	moderate	Negative	N0	M0	NA	NA	NA	NA	NA	NA
218D6413A1	ICC	50	Male	CSYS	NA	primary tumor	NA	Positive	NX	MX	NA	NA	NA	NA	NA	NA
218D6471A1	ICC	66	Male	CSYS	III+IV	primary tumor	poor	Positive	NX	MX	NA	NA	NA	NA	NA	NA
218D6874A1	ICC	62	Female	CSYS	III+IV	primary tumor	poor	Negative	NX	M1	NA	NA	NA	NA	NA	NA
218E7563A1	ICC	60	Female	CSYS	NA	primary tumor	NA	Negative	NX	MX	NA	NA	NA	NA	NA	NA
218E7564A1	ICC	71	Male	CSYS	I	primary tumor	NA	Negative	N0	M0	NA	NA	NA	NA	NA	NA
218E7890A1	ICC	53	Female	CSYS	III+IV	primary tumor	poor	NA	N0	M0	NA	NA	NA	NA	NA	NA
218F8298A1	ICC	42	Female	CSYS	NA	primary tumor	NA	NA	NX	MX	NA	NA	NA	NA	NA	NA
218F8400A1	ICC	52	Male	CSYS	NA	primary tumor	poor	NA	N1	MX	NA	NA	NA	NA	NA	NA
218F8651A1	ECC	55	Female	CSYS	II	primary tumor	moderate	Negative	N1	M0	NA	NA	NA	NA	NA	NA
218G8980A1	ECC	79	Female	CSYS	I	primary tumor	moderate	Negative	N0	M0	NA	NA	NA	NA	NA	NA
218G9808A1	ICC	49	Female	CSYS	NA	primary tumor	NA	Negative	N1	M0	NA	NA	NA	NA	NA	NA
218G9947A1	ECC	53	Male	CSYS	III+IV	metastatic tumor	moderate	Negative	NX	M1	NA	NA	NA	NA	NA	NA
218G9952A1	ICC	60	Female	CSYS	III+IV	primary tumor	poor	Negative	N1	M1	NA	NA	NA	NA	NA	NA
218H1383A1	ICC	55	Male	CSYS	II	primary tumor	poor	Positive	N0	M0	NA	NA	NA	NA	NA	NA
218H1627A1	ECC	78	Male	CSYS	I	primary tumor	poor	Negative	N0	M0	NA	NA	NA	NA	NA	NA
218H1955A1	ICC	53	Male	CSYS	III+IV	primary tumor	poor	Positive	N0	M0	NA	NA	NA	NA	NA	NA
218H2052A1	ICC	45	Female	CSYS	III+IV	primary tumor	poor	Negative	N1	M0	NA	NA	NA	NA	NA	NA
218H2159A1	ECC	62	Male	CSYS	II	primary tumor	moderate	NA	N0	M0	NA	NA	NA	NA	NA	NA
218I2875A1	ICC	46	Female	CSYS	NA	primary tumor	NA	Negative	NX	MX	NA	NA	NA	NA	NA	NA
218I3043A1	GBC	62	Female	CSYS	II	primary tumor	poor	Negative	N0	M0	NA	NA	NA	NA	NA	NA
218I3048A1	ECC	60	Male	CSYS	III+IV	primary tumor	poor	NA	N0	M1	NA	NA	NA	NA	NA	NA
218J4306A1	ICC	49	Male	CSYS	III+IV	recurrent tumor	moderate	Positive	NX	MX	NA	NA	NA	NA	NA	NA
218J4308A1	ICC	37	Male	CSYS	NA	primary tumor	moderate	Positive	N1	MX	NA	NA	NA	NA	NA	NA
218J4341A1	ICC	40	Male	CSYS	NA	primary tumor	poor	Positive	NX	MX	NA	NA	NA	NA	NA	NA
218J4343A1	ICC	76	Female	CSYS	III+IV	metastatic tumor	moderate	Negative	NX	M1	NA	NA	NA	NA	NA	NA
218J4612A1	ICC	36	Female	CSYS	III+IV	primary tumor	moderate	Negative	N1	M0	NA	NA	NA	NA	NA	NA
218J5499A1	ICC	44	Male	CSYS	III+IV	primary tumor	poor	Negative	N1	M0	NA	NA	NA	NA	NA	NA
218K6790A1	ICC	68	Male	CSYS	III+IV	recurrent tumor	poor	Positive	NX	MX	NA	NA	NA	NA	NA	NA
218K6907A1	ICC	54	Female	CSYS	NA	primary tumor	poor	Positive	NX	MX	NA	NA	NA	NA	NA	NA
218L7136A1	ECC	34	Female	CSYS	III+IV	primary tumor	moderate	Positive	N1	M0	NA	NA	NA	NA	NA	NA
218L7137A1	ICC	56	Male	CSYS	III+IV	primary tumor	moderate	Positive	N0	M1	NA	NA	NA	NA	NA	NA
218L7990A1	ICC	49	Male	CSYS	III+IV	primary tumor	poor	Positive	N1	M0	NA	NA	NA	NA	NA	NA
218L9107A1	GBC	55	Female	CSYS	III+IV	metastatic tumor	NA	Negative	NX	M1	NA	NA	NA	NA	NA	NA
219A1770D1	ECC	61	Female	CSYS	II	primary tumor	poor	Negative	N1	M0	NA	NA	NA	NA	NA	NA
219A9920A1	ICC	55	Female	CSYS	III+IV	metastatic tumor	poor	Positive	NX	MX	NA	NA	NA	NA	NA	NA
24059085A1	ICC	63	Male	CSYS	III+IV	primary tumor	moderate	Negative	NX	MX	NA	NA	NA	NA	NA	NA
618F8157A2	ICC	58	Female	CSYS	III+IV	metastatic tumor	NA	Negative	NX	M1	NA	NA	NA	NA	NA	NA
618F8167A2	ECC	44	Male	CSYS	III+IV	metastatic tumor	NA	Negative	NX	M1	NA	NA	NA	NA	NA	NA
ASCO795410	ICC	54	Male	CSYS	NA	primary tumor	NA	Negative	NX	MX	NA	NA	NA	NA	NA	NA
CASE111111	GBC	69	Female	CSYS	II	primary tumor	poor	Negative	N0	M0	NA	NA	NA	NA	NA	NA
CASE222222	GBC	74	Male	CSYS	III+IV	primary tumor	moderate	Negative	N0	M0	NA	NA	NA	NA	NA	NA
CASE333333	GBC	54	Female	CSYS	II	primary tumor	poor	Negative	N0	M0	NA	NA	NA	NA	NA	NA
CASE444444	GBC	64	Female	CSYS	II	primary tumor	poor	Negative	N0	M0	NA	NA	NA	NA	NA	NA
CASE555555	GBC	40	Female	CSYS	III+IV	primary tumor	poor	Negative	N0	MX	NA	NA	NA	NA	NA	NA
R18C5410R2	ECC	68	Male	CSYS	I	primary tumor	moderate	Negative	N0	M0	NA	NA	NA	NA	NA	NA
R18C5411R2	ECC	53	Female	CSYS	I	primary tumor	poor	Negative	N0	M0	NA	NA	NA	NA	NA	NA
R18D6313R1	GBC	45	Female	CSYS	NA	primary tumor	NA	Negative	NX	MX	NA	NA	NA	NA	NA	NA
R18D6314R1	GBC	37	Female	CSYS	NA	primary tumor	moderate	Negative	N0	MX	NA	NA	NA	NA	NA	NA
R18D6315R1	GBC	73	Male	CSYS	NA	primary tumor	poor	Negative	NX	MX	NA	NA	NA	NA	NA	NA
R18D6316R1	GBC	63	Male	CSYS	II	primary tumor	moderate	Negative	N0	M0	NA	NA	NA	NA	NA	NA
R18D6317R1	GBC	60	Female	CSYS	NA	primary tumor	poor	Negative	NX	MX	NA	NA	NA	NA	NA	NA
R18D6417R1	GBC	55	Female	CSYS	III+IV	primary tumor	poor	Negative	N1	M0	NA	NA	NA	NA	NA	NA
R18D6463R1	GBC	69	Female	CSYS	II	primary tumor	poor	Negative	N0	M0	NA	NA	NA	NA	NA	NA
R18D6470R1	GBC	58	Male	CSYS	II	primary tumor	poor	Negative	N0	M0	NA	NA	NA	NA	NA	NA
R18D6483R1	GBC	54	Female	CSYS	III+IV	primary tumor	poor	Negative	NX	M1	NA	NA	NA	NA	NA	NA
R18D6488R1	GBC	37	Female	CSYS	III+IV	primary tumor	poor	Negative	N1	M0	NA	NA	NA	NA	NA	NA
R18D6489R1	GBC	51	Female	CSYS	III+IV	primary tumor	moderate	Negative	N0	M1	NA	NA	NA	NA	NA	NA
R18D6500R1	GBC	65	Female	CSYS	II	primary tumor	poor	Negative	N0	M0	NA	NA	NA	NA	NA	NA
R18D6508R1	GBC	67	Male	CSYS	III+IV	primary tumor	poor	Negative	N0	M0	NA	NA	NA	NA	NA	NA
R18D6510R1	GBC	64	Female	CSYS	II	primary tumor	poor	Negative	N0	M0	NA	NA	NA	NA	NA	NA
R18D6521R1	GBC	79	Female	CSYS	II	primary tumor	poor	Negative	N0	M0	NA	NA	NA	NA	NA	NA
R18D6523R1	GBC	74	Female	CSYS	III+IV	primary tumor	poor	NA	N1	M0	NA	NA	NA	NA	NA	NA
R18D6529R1	GBC	52	Female	CSYS	III+IV	primary tumor	poor	Negative	N1	M0	NA	NA	NA	NA	NA	NA
R18D6531R1	GBC	56	Male	CSYS	III+IV	primary tumor	poor	Positive	N0	M0	NA	NA	NA	NA	NA	NA
R18D6542R1	GBC	50	Female	CSYS	II	primary tumor	poor	Negative	N0	M0	NA	NA	NA	NA	NA	NA
R18D6543R1	GBC	45	Male	CSYS	II	primary tumor	poor	Negative	N0	M0	NA	NA	NA	NA	NA	NA
R18D6640R1	GBC	65	Male	CSYS	III+IV	primary tumor	poor	Negative	N0	M0	NA	NA	NA	NA	NA	NA
R18D6641R1	GBC	69	Female	CSYS	II	primary tumor	poor	Negative	N0	M0	NA	NA	NA	NA	NA	NA
R18D6643R1	GBC	85	Female	CSYS	II	primary tumor	poor	Negative	N0	M0	NA	NA	NA	NA	NA	NA
R18D6713R1	GBC	82	Female	CSYS	II	primary tumor	moderate	Negative	N0	M0	NA	NA	NA	NA	NA	NA
R18D6714R1	GBC	55	Male	CSYS	III+IV	primary tumor	poor	Positive	N0	M0	NA	NA	NA	NA	NA	NA
R18D6831R1	ICC	67	Male	CSYS	NA	primary tumor	poor	Negative	NX	MX	NA	NA	NA	NA	NA	NA
R18D6917S2	GBC	68	Female	CSYS	III+IV	primary tumor	moderate	Negative	N1	M0	NA	NA	NA	NA	NA	NA
R18E7186R1	ECC	60	Male	CSYS	NA	primary tumor	NA	Negative	N0	M0	NA	NA	NA	NA	NA	NA
R18F8364R1	GBC	71	Female	CSYS	II	primary tumor	moderate	NA	N0	M0	NA	NA	NA	NA	NA	NA
R18F8368R1	GBC	68	Male	CSYS	I	primary tumor	moderate	Negative	N0	M0	NA	NA	NA	NA	NA	NA
R18F8384R1	GBC	63	Female	CSYS	II	primary tumor	moderate	Negative	N0	M0	NA	NA	NA	NA	NA	NA
R18F8385R1	GBC	70	Female	CSYS	NA	primary tumor	poor	NA	NX	MX	NA	NA	NA	NA	NA	NA
R18F8390R1	GBC	61	Female	CSYS	III+IV	primary tumor	poor	Negative	N1	M0	NA	NA	NA	NA	NA	NA
R18F8410R1	GBC	59	Female	CSYS	III+IV	primary tumor	poor	Negative	N0	M1	NA	NA	NA	NA	NA	NA
R18F8428R1	GBC	71	Female	CSYS	III+IV	primary tumor	moderate	Negative	N0	M0	NA	NA	NA	NA	NA	NA
R18F8429R1	GBC	52	Female	CSYS	II	primary tumor	poor	Negative	N0	M0	NA	NA	NA	NA	NA	NA
R18F8432R1	GBC	58	Female	CSYS	III+IV	primary tumor	poor	Positive	NX	M1	NA	NA	NA	NA	NA	NA
R18F8433R1	GBC	68	Female	CSYS	II	primary tumor	moderate	Negative	N0	M0	NA	NA	NA	NA	NA	NA
R18F8442R1	GBC	73	Male	CSYS	III+IV	primary tumor	moderate	Negative	N0	M0	NA	NA	NA	NA	NA	NA
R18F8445R1	GBC	62	Female	CSYS	II	primary tumor	poor	Negative	N0	M0	NA	NA	NA	NA	NA	NA
R18F8636M2	ICC	68	Female	CSYS	NA	primary tumor	NA	Negative	NX	MX	NA	NA	NA	NA	NA	NA
R18G9202R1	ECC	53	Female	CSYS	I	primary tumor	moderate	Negative	N0	M0	NA	NA	NA	NA	NA	NA
R18G9203R1	ECC	59	Male	CSYS	II	primary tumor	moderate	Negative	N0	M0	NA	NA	NA	NA	NA	NA
R18G9211R1	ECC	49	Female	CSYS	III+IV	primary tumor	moderate	Negative	N0	M0	NA	NA	NA	NA	NA	NA
R18G9220R1	ICC	38	Female	CSYS	III+IV	primary tumor	moderate	NA	N1	M0	NA	NA	NA	NA	NA	NA
R18G9241R1	ECC	54	Male	CSYS	II	primary tumor	moderate	Negative	N0	M0	NA	NA	NA	NA	NA	NA
R18G9243R1	ECC	62	Male	CSYS	II	primary tumor	moderate	Negative	N0	M0	NA	NA	NA	NA	NA	NA
R18G9244R1	ECC	77	Male	CSYS	I	primary tumor	moderate	NA	N0	M0	NA	NA	NA	NA	NA	NA
R18G9245R1	ICC	56	Male	CSYS	III+IV	primary tumor	moderate	Negative	N0	M0	NA	NA	NA	NA	NA	NA
R18G9249R1	ECC	52	Male	CSYS	II	primary tumor	moderate	Negative	N0	M0	NA	NA	NA	NA	NA	NA
R18G9250R1	ECC	58	Male	CSYS	III+IV	primary tumor	moderate	NA	N0	M0	NA	NA	NA	NA	NA	NA
R18G9251R1	ECC	73	Male	CSYS	III+IV	primary tumor	moderate	Negative	N0	M1	NA	NA	NA	NA	NA	NA
R18G9255R1	ECC	67	Male	CSYS	I	primary tumor	moderate	NA	N0	M0	NA	NA	NA	NA	NA	NA
R18G9257R1	ECC	83	Male	CSYS	I	primary tumor	moderate	Negative	N0	M0	NA	NA	NA	NA	NA	NA
R18G9258R1	ECC	54	Male	CSYS	I	primary tumor	moderate	Negative	N0	M0	NA	NA	NA	NA	NA	NA
R18G9465R1	ECC	65	Male	CSYS	II	primary tumor	moderate	Negative	N0	M0	NA	NA	NA	NA	NA	NA
R18G9477R1	ECC	61	Male	CSYS	III+IV	primary tumor	moderate	Negative	N1	M0	NA	NA	NA	NA	NA	NA
R18G9484R1	ECC	51	Male	CSYS	II	primary tumor	moderate	Negative	N0	M0	NA	NA	NA	NA	NA	NA
R18G9499R1	ECC	44	Male	CSYS	III+IV	primary tumor	moderate	Negative	N1	M0	NA	NA	NA	NA	NA	NA
R18G9501R1	ECC	40	Female	CSYS	I	primary tumor	poor	Negative	N0	M0	NA	NA	NA	NA	NA	NA
R18G9503R1	ECC	61	Male	CSYS	III+IV	primary tumor	moderate	Negative	N1	M0	NA	NA	NA	NA	NA	NA
R18G9504R1	ECC	60	Male	CSYS	I	primary tumor	moderate	Negative	N0	M0	NA	NA	NA	NA	NA	NA
R18G9508R1	ECC	38	Female	CSYS	III+IV	primary tumor	moderate	Negative	N0	M0	NA	NA	NA	NA	NA	NA
R18G9509R1	ECC	60	Female	CSYS	I	primary tumor	moderate	Negative	N0	M0	NA	NA	NA	NA	NA	NA

R18H2220M1	ECC	71	Female	CSYS	II	primary tumor	moderate	Negative	N0	M0	NA	NA	NA	NA	NA	NA

Table S2. Gene list of the capture-based NGS platform (Cancer Sequencing YS panel, CSYS).

**Exons of 450 cancer-related genes**									
ABL1	ABL2	ACVR1B	ACVR2A	ADAM29	ADGRA2	AKT1	AKT2	AKT3	ALK
AMER1	APC	APEX1	AR	ARAF	ARFRP1	ARID1A	ARID1B	ARID2	ASXL1
ATF1	ATM	ATR	ATRX	AURKA	AURKB	AXIN1	AXIN2	AXL	BAP1
BARD1	BCL2	BCL2L1	BCL2L11	BCL2L2	BCL6	BCOR	BCORL1	BCR	BIRC5
BLK	BLM	BMPR1A	BMX	BRAF	BRCA1	BRCA2	BRD4	BRIP1	BTG1
BTK	CAMTA1	CARD11	CBFB	CBL	CCND1	CCND2	CCND3	CCNE1	CD274
CD79A	CD79B	CDC73	CDH1	CDK12	CDK4	CDK6	CDK8	CDKN1A	CDKN1B
CDKN2A	CDKN2B	CDKN2C	CEBPA	CFTR	CHD2	CHD4	CHEK1	CHEK2	CIC
COL1A1	CRBN	CREB3L1	CREB3L2	CREBBP	CRKL	CRLF2	CSF1	CSF1R	CSK
CSNK1A1	CTCF	CTNNA1	CTNNB1	CUL3	CXCR4	CYLD	CYP17A1	CYP2D6	DAXX
DDR1	DDR2	DICER1	DNMT3A	DOT1L	DPYD	EGF	EGFR	EMSY	EP300
EPCAM	EPHA2	EPHA3	EPHA5	EPHA7	EPHB1	ERBB2	ERBB3	ERBB4	ERCC1
ERG	ERRFI1	ESR1	ETV1	ETV4	ETV5	ETV6	EWSR1	EZH2	FAM135B
FAM46C	FANCA	FANCC	FANCD2	FANCE	FANCF	FANCG	FANCL	FANCM	FAS
FAT1	FAT3	FAT4	FBXW7	FEN1	FEV	FGF10	FGF12	FGF14	FGF19
FGF23	FGF3	FGF4	FGF6	FGF7	FGFR1	FGFR2	FGFR3	FGFR4	FGR
FH	FLCN	FLI1	FLT1	FLT3	FLT4	FOS	FOXL2	FOXO1	FOXP1
FRS2	FUBP1	FUS	FYN	GABRA6	GATA1	GATA2	GATA3	GATA4	GATA6
GID4	GLI1	GLI2	GLI3	GNA11	GNA13	GNAQ	GNAS	GRIN2A	GRM3
GSK3B	H3F3A	HCK	HDAC9	HGF	HNF1A	HRAS	HSD3B1	HSP90AA1	HTATIP2
IDH1	IDH2	IGF1R	IGF2	IKBKE	IKZF1	IL7R	INHBA	INPP4B	IRF2
IRF4	IRS2	ITK	JAK1	JAK2	JAK3	JUN	KAT6A	KDM5A	KDM5B
KDM5C	KDM6A	KDR	KEAP1	KEL	KIT	KLHL6	KMT2A	KMT2C	KMT2D
KRAS	LCK	LIMK1	LMO1	LRP1	LRP1B	LRP2	LYN	LZTR1	MACC1
MAGI2	MAP2K1	MAP2K2	MAP2K4	MAP3K1	MAP3K13	MAP4K5	MCL1	MDM2	MDM4
MED12	MEF2B	MEN1	MERTK	MET	MGMT	MITF	MLH1	MPL	MRE11A
MS4A1	MSH2	MSH6	MST1R	MTOR	MUTYH	MYB	MYC	MYCL	MYCN
MYD88	NBN	NCOA2	NCOR1	NEK11	NF1	NF2	NFE2L2	NFIB	NFKBIA
NKX2-1	NOTCH1	NOTCH2	NOTCH3	NOTCH4	NPM1	NR4A3	NRAS	NRG1	NRG3
NSD1	NTRK1	NTRK2	NTRK3	NUP93	PAK3	PALB2	PARK2	PARP1	PARP2
PARP3	PARP4	PAX5	PBRM1	PCA3	PDCD1	PDCD1LG2	PDGFB	PDGFRA	PDGFRB
PDK1	PIK3C2B	PIK3CA	PIK3CB	PIK3CD	PIK3CG	PIK3R1	PIK3R2	PKD2	PLA2G1B
PLCG2	PMS2	POLB	POLD1	POLE	PPP2R1A	PRDM1	PREX2	PRKACA	PRKAR1A
PRKCI	PRKDC	PRSS1	PRSS8	PTCH1	PTEN	PTK2	PTK6	PTPN11	QKI
RAC1	RAD50	RAD51	RAD51B	RAD51C	RAD51D	RAD52	RAD54B	RAD54L	RAF1
RANBP2	RARA	RB1	RBM10	RECQL	REL	RELA	RELB	RET	RHBDF2
RHOA	RICTOR	RNF43	ROCK1	ROCK2	ROS1	RPTOR	RUNX1	RUNX1T1	RXRA
SDHA	SDHB	SDHC	SDHD	SETD2	SF3B1	SIK1	SLIT2	SMAD2	SMAD3
SMAD4	SMARCA4	SMARCB1	SMARCD1	SMO	SNCAIP	SOCS1	SOX10	SOX2	SOX9
SPEN	SPINK1	SPOP	SPTA1	SRC	SRMS	SS18	SSX1	STAG2	STAT3
STAT4	STK11	STK24	SUFU	SYK	TAF1	TBX3	TCF7L2	TEK	TERT
TET1	TET2	TET3	TFE3	TGFBR1	TGFBR2	TIE1	TIPARP	TMPRSS2	TNFAIP3
TNFRSF14	TNFSF11	TNFSF13B	TNK2	TOP1	TOP2A	TP53	TPMT	TSC1	TSC2
TSHR	TYK2	U2AF1	UGT1A1	VEGFA	VHL	WEE1	WEE2	WHSC1	WISP3

WT1	XIAP	XPO1	XRCC2	XRCC3	YES1	ZBTB2	ZNF217	ZNF703	ZNF750
**Selected introns of 38 genes frequently rearranged in cancer**									
ALK	BCR	BRAF	BRCA1	BRCA2	DDR2	EGFR	FGFR1	FGFR2	FGFR3
KIT	MET	MSH2	NTRK1	NTRK2	PDGFRA	RAF1	RARA	RET	ROS1
AKT3	ERBB2	ERBB4	NTRK3	BCL2	BRD4	ETV1	ETV4	ETV5	ETV6
EWSR1	MYB	NOTCH2	SS18	TMPRSS2	NOTCH1	NRG1	PDGFB	JAK2	

Table S3. Potentially actionable targets (PATs) in BTC patients.

Targets	Mutation Types	Drugs	OncoKB level	ESCAT tier
ABL1	E255K, E255V, F317C, F317I, F317L, F317V, F359C, F359I, F359V, T315A, Y253H, BCR-ABL1 Fusion	Bosutinib	2B	III-A
ALK	Fusions	Crizotinib	2B	III-A
BRAF	V600E	Encorafenib; Cetuximab; Binimetinib; Dabrafenib; Panitumumab; Trametinib	2B	III-A
BRCA1	Oncogenic Mutations	Olaparib	2B	III-A
BRCA2	Oncogenic Mutations	Olaparib	2B	III-A
CDK4	Amplification	Abemaciclib; Palbociclib	2B	III-A
EGFR	A763_Y764insFQEA	Afatinib; Erlotinib; Gefitinib	2B	III-A
ERBB2	Amplification, Oncogenic Mutations	Ado-Trastuzumab Emtansine; Trastuzumab; Carboplatin-Taxol Regimen	2B	III-A
KIT	C809G, D816A, D816E, D816F, D816G, D816H, D816N, D816V, D816Y, D820A, D820E, D820G, D820Y, N822H, N822I, N822K, N822Y, R796G, Y823D, T670I, V654A, D816, Oncogenic Mutations	Sorafenib; Imatinib	2B	III-A
MET	Amplification, D1010mut, Exon 14 Deletion, Exon 14 splice mutation, Y1003mut	Cabozantinib; Crizotinib	2B	III-A
PDGFRA	Oncogenic Mutations, D842V	Imatinib, Dasatinib	2B	III-A
RET	Fusions, Oncogenic Mutations	Cabozantinib; Vandetanib; LOXO-292	2B	III-A
TSC1	Oncogenic Mutations	Everolimus	2B	III-A
TSC2	Oncogenic Mutations	Everolimus	2B	III-A
FGFR2	Fusions	BGJ398; Debio1347; AZD4547; Erdafitinib	3A	I-B
IDH1	Oncogenic Mutations	BGJ398; Debio1347; AZD4547; Erdafitinib	3A	I-B
AKT1	E17K	AZD5363	3B	II-B
NTRK	Fusions	larotrectinib	3B	I-C
MSI-H	dMMR	pembrolizumab	3B	I-C

Table S4. The somatic variants from coding regions of 803 patients with biliary tract carcinoma.											
**Sample.ID**	**Chr.**	**Start_Pos**	**End_Pos**	**Variant_Class**	**Variant_Type**	**Ref_Allele**	**Tumor_Allele1**	**Tumor_Allele2**	**Gene Symbol**	**DNA_CHANGE**	**AA_CHANGE**
116K0135A1	12	25398284	25398284	Missense_Mutation	SNP	C	T	T	KRAS	c.35G>A	p.G12D
116K0135A1	17	7577142	7577142	Nonsense_Mutation	SNP	C	A	A	TP53	c.796G>T	p.G266*
116K0135A1	2	47698190	47698190	Missense_Mutation	SNP	A	G	G	MSH2	c.1748A>G	p.N583S
116K0135A1	1	158606546	158606546	Missense_Mutation	SNP	T	G	G	SPTA1	c.5195A>C	p.K1732T
116K0135A1	1	158609707	158609707	Missense_Mutation	SNP	G	T	T	SPTA1	c.4828C>A	p.Q1610K
116L0197A1		-	-	Missense_Mutation	SNP	-	-	-	TP53	:.654_655insGT(	p.V218dup
116L0197A1	17	7578212	7578212	Nonsense_Mutation	SNP	G	A	A	TP53	c.637C>T	p.R213*
116L0197A1	11	92534643	92534643	Missense_Mutation	SNP	A	G	G	FAT3	c.8464A>G	p.I2822V
116L0197A1	2	48025777	48025777	Missense_Mutation	SNP	A	G	G	MSH6	c.655A>G	p.S219G
116L0197A1	1	40366499	40366499	Missense_Mutation	SNP	G	T	T	MYCL	c.698C>A	p.P233H
116L0197A1	8	69002826	69002826	Missense_Mutation	SNP	C	A	A	PREX2	c.2126C>A	p.A709E
116L0197A1	1	158615147	158615147	Missense_Mutation	SNP	G	A	A	SPTA1	c.4025C>T	p.A1342V
116L0197A1	X	123181290	123181290	Missense_Mutation	SNP	C	T	T	STAG2	c.754C>T	p.R252W
116L0221A1	3	52439128	52439128	Nonsense_Mutation	SNP	G	A	A	BAP1	c.1114C>T	p.Q372*
116L0221A1		-	-	Frame_Shift_Ins	INS	-	-	-	PBRM1	c.3550dupA	P.M1184Nfs*11
116L0254A1		-	-	In_Frame_Del	INS	-	-	-	BRAF	c.1457_1471del	P.N486_P490del
116L0254A1	16	2112992	2112992	Missense_Mutation	SNP	G	A	A	TSC2	c.1381G>A	p.V461M
116L0254A1	17	7577575	7577575	Missense_Mutation	SNP	A	C	C	TP53	c.706T>G	p.Y236D
116L0254A1	7	2976787	2976787	Missense_Mutation	SNP	C	T	T	CARD11	c.1225G>A	p.E409K
116L0254A1	17	11984782	11984782	Nonsense_Mutation	SNP	C	T	T	MAP2K4	c.328C>T	p.R110*
116L0254A1	18	48603023	48603023	Nonsense_Mutation	SNP	C	T	T	SMAD4	c.1324C>T	p.Q442*
116L0254A1	19	11138553	11138553	Missense_Mutation	SNP	C	G	G	SMARCA4	c.3309C>G	p.C1103W
116L0263A1	17	37881426	37881426	Missense_Mutation	SNP	A	G	G	ERBB2	c.2528A>G	p.D843G
116L0263A1	17	37868208	37868208	Missense_Mutation	SNP	C	T	T	ERBB2	c.839C>T	p.S280F
116L0263A1	1	156845889	156845889	Missense_Mutation	SNP	C	T	T	NTRK1	c.1501C>T	p.R501C
116L0263A1	17	7577557	7577557	Missense_Mutation	SNP	A	T	T	TP53	c.724T>A	p.C242S
116L0263A1	9	21971000	21971000	Nonsense_Mutation	SNP	C	A	A	CDKN2A	c.358G>T	p.E120*
116L0263A1	9	21994137	21994137	Splice_Site	SNP	C	A	A	CDKN2A	c.193+1G>T	-
116L0263A1	3	41266137	41266137	Missense_Mutation	SNP	C	T	T	CTNNB1	c.134C>T	p.S45F
116L0263A1		-	-	Frame_Shift_Del	INS	-	-	-	SMAD4	c.854del	p.N285Tfs*51
116L0263A1	3	170015084	170015084	Splice_Site	SNP	C	G	G	PRKCI	c.1498-8C>G	-
116L0279A1	12	25398284	25398284	Missense_Mutation	SNP	C	A	A	KRAS	c.35G>T	p.G12V
116L0279A1	6	93955072	93955072	Missense_Mutation	SNP	A	T	T	EPHA7	c.2826T>A	p.D942E
116L0279A1	20	57429454	57429454	Missense_Mutation	SNP	C	T	T	GNAS	c.947C>T	p.T316M
116L0279A1	12	49435757	49435757	Nonsense_Mutation	SNP	G	T	T	KMT2D	c.6126C>A	p.C2042*
116L0287A1	8	38283676	38283676	Missense_Mutation	SNP	C	T	T	FGFR1	c.709G>A	p.G237S
116L0287A1	5	149513266	149513266	Missense_Mutation	SNP	G	A	A	PDGFRB	c.817C>T	p.R273C
116L0287A1	4	88989068	88989068	Missense_Mutation	SNP	C	A	A	PKD2	c.2377C>A	p.H793N
116L0287A1	2	198267484	198267484	Missense_Mutation	SNP	G	A	A	SF3B1	c.1873C>T	p.R625C
117B0454A1		-	-	Frame_Shift_Del	INS	-	-	-	BAP1	c.1746delC	p.S583Rfs*34
117B0454A1		-	-	Frame_Shift_Del	INS	-	-	-	PBRM1	c.1704_1705del	p.K569Nfs*3
117B0454A1	17	7577138	7577138	Missense_Mutation	SNP	C	G	G	TP53	c.800G>C	p.R267P
117B0454A1	1	16475389	16475389	Missense_Mutation	SNP	G	C	C	EPHA2	c.307C>G	p.R103G
117B0488A1	12	25398284	25398284	Missense_Mutation	SNP	C	T	T	KRAS	c.35G>A	p.G12D
117B0488A1	17	7577539	7577539	Missense_Mutation	SNP	G	A	A	TP53	c.742C>T	p.R248W
117B0488A1	X	63411777	63411777	Missense_Mutation	SNP	C	T	T	AMER1	c.1390G>A	p.A464T
117B0545A1	1	16261091	16261091	Missense_Mutation	SNP	C	T	T	SPEN	c.8356C>T	p.H2786Y
117B0545A1	12	56478854	56478854	Missense_Mutation	SNP	G	C	C	ERBB3	c.310G>C	p.V104L
117B0545A1	2	142012219	142012219	Splice_Site	SNP	A	G	G	LRP1B	c.344-9T>C	-
117B0545A1		-	-	Frame_Shift_Del	INS	-	-	-	LRP1B	c.3794del	p.S1265Lfs*23
117C0618A1	12	52374945	52374945	Missense_Mutation	SNP	A	G	G	ACVR1B	c.773A>G	p.H258R
117C0618A1	1	27094336	27094336	Missense_Mutation	SNP	G	T	T	ARID1A	c.3044G>T	p.G1015V
117C0618A1	21	39774503	39774503	Missense_Mutation	SNP	G	A	A	ERG	c.670C>T	p.R224W
117C0618A1	20	57430298	57430298	Missense_Mutation	SNP	C	T	T	GNAS	c.1978C>T	p.R660C
117C0618A1	1	202736067	202736067	Missense_Mutation	SNP	C	T	T	KDM5B	c.698G>A	p.R233H
117C0618A1	12	49420606	49420606	Missense_Mutation	SNP	C	T	T	KMT2D	c.15143G>A	p.R5048H
117C0618A1		-	-	Frame_Shift_Ins	INS	-	-	-	PBRM1	c.4535_4536du	p.E1513Rfs*35
117C0618A1		-	-	Splice_Site	SNP	-	-	-	RECQL	c.868-3delT	-
117C0618A1	1	11169753	11169753	Missense_Mutation	SNP	G	A	A	MTOR	c.7400C>T	p.A2467V
117C0618A1	17	29557904	29557904	Nonsense_Mutation	SNP	C	G	G	NF1	c.3158C>G	p.S1053*
117C0618A1		-	-	In_Frame_Del	INS	-	-	-	NF2	c.380del	p.L127*
117C0618A1	1	179198519	179198519	Missense_Mutation	SNP	A	G	G	ABL2	c.14T>C	p.V5A
117C0618A1	10	89717778	89717778	Splice_Site	SNP	T	C	C	PTEN	c.801+2T>C	-
117C0618A1	17	7574003	7574003	Nonsense_Mutation	SNP	G	A	A	TP53	c.1024C>T	p.R342*
117C0618A1		-	-	Frame_Shift_Del	INS	-	-	-	TP53	c.902del	p.P301Qfs*44
117C0618A1		-	-	Missense_Mutation	SNP	-	-	-	CTNNB1	c.133_135del	p.S45del
117C0618A1	15	99250981	99250981	Missense_Mutation	SNP	C	A	A	IGF1R	c.285C>A	p.S95R
117C0618A1	5	156679629	156679629	Missense_Mutation	SNP	C	T	T	ITK	c.1804C>T	p.R602W
117C0618A1	9	98240363	98240363	Missense_Mutation	SNP	G	A	A	PTCH1	c.1321C>T	p.R441C
117C0618A1	3	187443373	187443373	Missense_Mutation	SNP	G	A	A	BCL6	c.1753C>T	p.R585W
117C0618A1	X	129148573	129148573	Nonsense_Mutation	SNP	C	T	T	BCORL1	c.1825C>T	p.R609*
117C0618A1	1	51436158	51436158	Missense_Mutation	SNP	A	G	G	CDKN2C	c.118A>G	p.T40A
117C0618A1	X	48652558	48652558	Missense_Mutation	SNP	C	T	T	GATA1	c.1229C>T	p.P410L
117C0618A1	11	64572285	64572285	Missense_Mutation	SNP	G	A	A	MEN1	c.1354C>T	p.R452W
117C0618A1	2	47672796	47672796	Missense_Mutation	SNP	G	T	T	MSH2	c.1386G>T	p.Q462H
117C0618A1		-	-	Frame_Shift_Ins	INS	-	-	-	PIK3CB	c.1810dup	p.R604Pfs*29
117C0618A1		-	-	Frame_Shift_Del	INS	-	-	-	RNF43	c.1976del	p.G659Vfs*41
117C0618A1	14	81609778	81609778	Missense_Mutation	SNP	C	T	T	TSHR	c.1376C>T	p.A459V
117C0646A1	11	118374777	118374777	Missense_Mutation	SNP	G	A	A	KMT2A	c.8170G>A	p.D2724N
117C0646A1	8	93074800	93074800	Splice_Site	SNP	C	A	A	RUNX1T1	c.-20G>T	-
117C0646A1	12	56478932	56478932	Missense_Mutation	SNP	G	T	T	ERBB3	c.388G>T	p.A130S
117C0646A1	9	135798734	135798734	Splice_Site	SNP	C	T	T	TSC1	c.508+1G>A	-
117C0646A1	13	32914817	32914817	Missense_Mutation	SNP	G	A	A	BRCA2	c.6325G>A	p.V2109I
117C0646A1	17	7577534	7577534	Missense_Mutation	SNP	C	G	G	TP53	c.747G>C	p.R249S
117C0646A1	20	30659499	30659499	Missense_Mutation	SNP	G	A	A	HCK	c.34G>A	p.G12R
117C0646A1	4	55973998	55973998	Missense_Mutation	SNP	T	G	G	KDR	c.1318A>C	p.T440P
117C0646A1	1	204426884	204426884	Missense_Mutation	SNP	G	A	A	PIK3C2B	c.1685C>T	p.P562L
117C0646A1	16	10274172	10274172	Missense_Mutation	SNP	C	T	T	GRIN2A	c.97G>A	p.A33T
117C0646A1	8	48690301	48690301	Missense_Mutation	SNP				PRKDC	c.11987T>A	p.L3996Q
117C0651A1		-	-	Frame_Shift_Ins	INS	-	-	-	ARID1A	2635_2636insCA	p.V879Afs*58
117C0651A1	11	108199955	108199955	Nonsense_Mutation	SNP	C	T	T	ATM	c.7297C>T	p.Q2433*
117C0651A1		-	-	Frame_Shift_Ins	INS	-	-	-	EPHA2	c.279dup	p.I94Yfs*4
117C0651A1	2	141660539	141660539	Missense_Mutation	SNP	C	A	A	LRP1B	c.3716G>T	p.C1239F
117C0651A1	15	67482870	67482870	Missense_Mutation	SNP	C	T	T	SMAD3	c.1274C>T	p.S425F
117C0664A1	12	115112364	115112364	Missense_Mutation	SNP	C	T	T	TBX3	c.1376G>A	p.R459H
117C0664A1	10	114905771	114905771	Nonsense_Mutation	SNP	C	T	T	TCF7L2	c.721C>T	p.Q241*

117C0664A1	X	48651642	48651642	Missense_Mutation	SNP	C	T	T	GATA1	c.808C>T	p.R270W
117C0733A1	21	44524456	44524456	Missense_Mutation	SNP	G	T	T	U2AF1	c.101C>A	p.S34Y
117C0733A1	12	25398284	25398284	Missense_Mutation	SNP	C	A	A	KRAS	c.35G>T	p.G12V
117C0733A1	9	101908873	101908873	Nonsense_Mutation	SNP	C	T	T	TGFBR1	c.1237C>T	p.R413*
117C0733A1	6	32170055	32170055	Missense_Mutation	SNP	C	T	T	NOTCH4	c.3553G>A	p.G1185R
117C0866A1		-	-	Frame_Shift_Ins	INS	-	-	-	STK11	c.400dup	p.C134Lfs*29
117C0866A1	13	32912691	32912691	Missense_Mutation	SNP	A	T	T	BRCA2	c.4199A>T	p.H1400L
117C0866A1	3	30691929	30691929	Missense_Mutation	SNP	A	G	G	TGFBR2	c.431A>G	p.N144S
117D0978A1	3	178936082	178936082	Missense_Mutation	SNP	G	A	A	PIK3CA	c.1624G>A	p.E542K
117D0978A1	17	7578406	7578406	Missense_Mutation	SNP	C	T	T	TP53	c.524G>A	p.R175H
117D0997A1	12	25398284	25398284	Missense_Mutation	SNP	C	G	G	KRAS	c.35G>C	p.G12A
117D0997A1	9	21971017	21971017	Missense_Mutation	SNP	G	C	C	CDKN2A	c.341C>G	p.P114R
117D0997A1	17	7578268	7578268	Missense_Mutation	SNP	A	G	G	TP53	c.581T>C	p.L194P
117D1007A1	12	25398284	25398284	Missense_Mutation	SNP	C	A	A	KRAS	c.35G>T	p.G12V
117D1007A1	18	48604760	48604760	Missense_Mutation	SNP	C	T	T	SMAD4	c.1582C>T	p.H528Y
117D1007A1	17	7577018	7577018	Splice_Site	SNP	C	G	G	TP53	c.919+1G>C	-
117D1007A1	9	21971101	21971101	Missense_Mutation	SNP	G	T	T	CDKN2A	c.257C>A	p.A86D
117D1030A1	1	7151437	7151437	Splice_Site	SNP	G	A	A	CAMTA1	c.302+6G>A	-
117D1030A1	12	12038903	12038903	Missense_Mutation	SNP	G	T	T	ETV6	c.1196G>T	p.R399L
117D1030A1	12	25380275	25380275	Missense_Mutation	SNP	T	G	G	KRAS	c.183A>C	p.Q61H
117D1030A1	19	1220717	1220717	Splice_Site	SNP	G	C	C	STK11	c.734+1G>C	-
117D1030A1	X	66765161	66765161	Missense_Mutation	SNP	A	T	T	AR	c.173A>T	p.Q58L
117D1030A1	6	32163723	32163723	Missense_Mutation	SNP	G	T	T	NOTCH4	c.5503C>A	p.R1835S
117D1030A1	17	56774083	56774083	Missense_Mutation	SNP	C	T	T	RAD51C	c.434C>T	p.P145L
117D1074R1	1	120612014	120612014	Missense_Mutation	SNP	C	A	A	NOTCH2	c.7G>T	p.A3S
117D1074R1	6	157528292	157528292	Missense_Mutation	SNP	C	T	T	ARID1B	c.6017C>T	p.A2006V
117D1074R1	17	63554344	63554344	Missense_Mutation	SNP	T	C	C	AXIN2	c.395A>G	p.K132R
117D1074R1		-	-	Frame_Shift_Del	INS	-	-	-	BAP1	c.586del	p.W196Gfs*35
117D1074R1	17	59878736	59878736	Missense_Mutation	SNP	G	A	A	BRIP1	c.1018C>T	p.L340F
117D1074R1	3	89528647	89528647	Missense_Mutation	SNP	G	C	C	EPHA3	c.2947G>C	p.V983L
117D1074R1	16	9857127	9857127	Missense_Mutation	SNP	G	A	A	GRIN2A	c.4274C>T	p.S1425L
117D1074R1	16	9857217	9857217	Missense_Mutation	SNP	G	A	A	GRIN2A	c.4184C>T	p.A1395V
117D1074R1	1	120612013	120612013	Missense_Mutation	SNP	G	A	A	NOTCH2	c.8C>T	p.A3V
117D1074R1	1	17349159	17349159	Missense_Mutation	SNP	G	A	A	SDHB	c.709C>T	p.P237S
117D1074R1	19	17950435	17950435	Missense_Mutation	SNP	C	T	T	JAK3	c.1292G>A	p.R431Q
117D1101A1	17	7577142	7577142	Missense_Mutation	SNP	C	T	T	TP53	c.796G>A	p.G266R
117D1101A1		-	-	Frame_Shift_Ins	INS	-	-	-	CFTR	c.50dup	p.S18Qfs*27
117D1130A1	6	117717432	117717432	Splice_Site	SNP	T	C	C	ROS1	c.780-5A>G	-
117D1130A1	17	7578212	7578212	Nonsense_Mutation	SNP	G	A	A	TP53	c.637C>T	p.R213*
117D1130A1	X	66765455	66765455	Missense_Mutation	SNP	A	G	G	AR	c.467A>G	p.D156G
117D1130A1	11	92534643	92534643	Missense_Mutation	SNP	A	G	G	FAT3	c.8464A>G	p.I2822V
117D1130A1	2	48025777	48025777	Missense_Mutation	SNP	A	G	G	MSH6	c.655A>G	p.S219G
117D1130A1	1	40366499	40366499	Missense_Mutation	SNP	G	T	T	MYCL	c.698C>A	p.P233H
117D1130A1	8	69002826	69002826	Missense_Mutation	SNP	C	A	A	PREX2	c.2126C>A	p.A709E
117D1130A1	X	123181290	123181290	Missense_Mutation	SNP	C	T	T	STAG2	c.754C>T	p.R252W
117D1192A1	13	32915174	32915174	Missense_Mutation	SNP	G	C	C	BRCA2	c.6682G>C	p.V2228L
117D1192A1		-	-	Frame_Shift_Del	INS	-	-	-	TGFBR2	c.677del	p.D226Afs*38
117E1223A1	12	25398284	25398284	Missense_Mutation	SNP	C	T	T	KRAS	c.35G>A	p.G12D
117E1223A1	13	32914817	32914817	Missense_Mutation	SNP	G	A	A	BRCA2	c.6325G>A	p.V2109I
117E1223A1	17	7578492	7578492	Nonsense_Mutation	SNP	C	T	T	TP53	c.438G>A	p.W146*
117E1223A1	X	66764995	66764995	Missense_Mutation	SNP	G	A	A	AR	c.7G>A	p.V3M
117E1223A1	9	21970899	21970899	Splice_Site	SNP	A	G	G	CDKN2A	c.457+2T>C	-
117E1223A1	3	51980306	51980306	Missense_Mutation	SNP	G	A	A	PARP3	c.1223G>A	p.R408H
117E1223A1	11	128638021	128638021	Missense_Mutation	SNP	C	T	T	FLI1	c.239C>T	p.P80L
117E1223A1	7	142655047	142655047	Missense_Mutation	SNP	C	T	T	KEL	c.539G>A	p.R180H
117E1223A1	6	161969931	161969931	Missense_Mutation	SNP	G	T	T	PRKN	c.1038C>A	p.D346E
117E1223A1	1	16261134	16261134	Missense_Mutation	SNP	C	T	T	SPEN	c.8399C>T	p.A2800V
117E1250R1		-	-	Splice_Site	SNP	-	-	-	TP53	c.77_96+8del	-
117E1250R1	17	74475297	74475297	Missense_Mutation	SNP	C	T	T	RHBDF2	c.422G>A	p.R141H
117E1250R1		-	-	Missense_Mutation	SNP	-	-	-	ARID1B	C.121_123dup	P.S41dup
117E1250R1	15	90628339	90628339	Splice_Site	SNP	G	A	A	IDH2	c.1081-9C>T	-
117E1250R1	12	49420858	49420858	Missense_Mutation	SNP	C	T	T	KMT2D	c.14891G>A	p.R4964H
117E1250R1	19	42777428	42777428	Missense_Mutation	SNP	A	G	G	CIC	c.1493A>G	p.N498S
117E1250R1	14	45665454	45665454	Missense_Mutation	SNP	C	T	T	FANCM	c.5420C>T	p.T1807I
117E1250R1	4	187540652	187540652	Missense_Mutation	SNP	A	C	C	FAT1	c.7088T>G	p.V2363G
117E1250R1	11	92087587	92087587	Missense_Mutation	SNP	C	T	T	FAT3	c.2309C>T	p.T770M
117E1259R1	10	123243238	123243238	Nonsense_Mutation	SNP	G	A	A	FGFR2	c.2275C>T	p.R759*
117E1259R1	17	7577534	7577534	Missense_Mutation	SNP	C	A	A	TP53	c.747G>T	p.R249S
117E1259R1	3	130992408	130992408	Missense_Mutation	SNP	C	T	T	NEK11	c.1708C>T	p.R570C
117E1259R1	9	98239029	98239029	Splice_Site	SNP	T	C	C	PTCH1	c.1602+12A>G	-
117E1259R1	1	27105700	27105700	Missense_Mutation	SNP	C	T	T	ARID1A	c.5311C>T	p.P1771S
117E1259R1	3	142217611	142217611	Missense_Mutation	SNP	T	G	G	ATR	c.5386A>C	p.K1796Q
117E1259R1	4	66230894	66230894	Missense_Mutation	SNP	G	A	A	EPHA5	c.2077C>T	p.R693C
117E1259R1	6	93979351	93979351	Missense_Mutation	SNP	C	G	G	EPHA7	c.1477G>C	p.V493L
117E1259R1	3	134898755	134898755	Missense_Mutation	SNP	G	A	A	EPHB1	c.1813G>A	p.E605K
117E1259R1	16	89877407	89877407	Missense_Mutation	SNP	G	C	C	FANCA	c.356C>G	p.S119C
117E1259R1	9	97879592	97879592	Splice_Site	SNP	C	G	G	FANCC	c.1072+5G>C	-
117E1259R1	16	56839548	56839548	Splice_Site	SNP	G	A	A	NUP93	c.489+4G>A	-
117E1259R1	8	48872522	48872522	Splice_Site	SNP	C	T	T	PRKDC	c.154+11G>A	-
117E1259R1	12	1034604	1034604	Splice_Site	SNP	T	C	C	RAD52	c.543+12A>G	-
117E1259R1		-	-	Splice_Site	SNP	-	-	-	RELA	c.1033+6delG	-
117E1259R1		-	-	Frame_Shift_Del	INS	-	-	-	SDHA	c.1945_1946del	p.L649Efs*4
117E1259R1	1	16260531	16260531	Missense_Mutation	SNP	C	T	T	SPEN	c.7796C>T	p.S2599L
117E1259R1	14	81609751	81609751	Missense_Mutation	SNP	G	A	A	TSHR	c.1349G>A	p.R450H
117E1261R1	10	43601830	43601830	Missense_Mutation	SNP	G	A	A	RET	c.874G>A	p.V292M
117E1261R1	8	38271189	38271189	Missense_Mutation	SNP	C	A	A	FGFR1	c.2426G>T	p.R809L
117E1261R1	17	7574035	7574035	Splice_Site	SNP	T	C	C	TP53	c.994-2A>G	-
117E1261R1	1	16459821	16459821	Missense_Mutation	SNP	G	A	A	EPHA2	c.1907C>T	p.S636L
117E1261R1	9	98218566	98218566	Missense_Mutation	SNP	C	T	T	PTCH1	c.3298G>A	p.V1100I
117E1261R1		-	-	In_Frame_Del	INS	-	-	-	RB1	c.1641_1658del	p.K548_C553del
117E1261R1	16	396430	396430	Missense_Mutation	SNP	G	C	C	AXIN1	c.596C>G	p.P199R
117E1261R1		-	-	Frame_Shift_Del	INS	-	-	-	CYP17A1	985_987delinsA	p.Y329Kfs*90
117E1261R1	3	185783775	185783775	Missense_Mutation	SNP	T	C	C	ETV5	c.737A>G	p.Q246R
117E1261R1	16	89871721	89871721	Missense_Mutation	SNP	G	C	C	FANCA	c.676C>G	p.Q226E
117E1261R1	4	187541859	187541859	Missense_Mutation	SNP	C	T	T	FAT1	c.5881G>A	p.G1961S
117E1261R1	4	187509906	187509906	Missense_Mutation	SNP	G	C	C	FAT1	c.13607C>G	p.P4536R
117E1261R1	20	57429772	57429772	Missense_Mutation	SNP	A	T	T	GNAS	c.1265A>T	p.Q422L
117E1261R1	7	151970833	151970833	Missense_Mutation	SNP	G	C	C	KMT2C	c.969C>G	p.I323M

117E1261R1	2	141004742	141004742	Splice_Site	SNP	C	T	T	LRP1B	c.13248-11G>A	-
117E1261R1	2	170030506	170030506	Missense_Mutation	SNP	C	T	T	LRP2	c.10937G>A	p.R3646H
117E1261R1	2	170097611	170097611	Missense_Mutation	SNP	C	T	T	LRP2	c.3932G>A	p.R1311H
117E1261R1	2	170135979	170135979	Missense_Mutation	SNP	G	A	A	LRP2	c.1468C>T	p.R490C
117E1261R1	12	133236099	133236099	Splice_Site	SNP	G	C	C	POLE	c.3061-4C>G	-
117E1261R1	2	74273621	74273621	Missense_Mutation	SNP	C	T	T	TET3	c.577C>T	p.R193C
117E1261R1	17	38548848	38548848	Missense_Mutation	SNP	C	A	A	TOP2A	c.3953G>T	p.R1318L
117E1261R1	19	10478867	10478867	Missense_Mutation	SNP	C	T	T	TYK2	c.329G>A	p.R110Q
117E1396A1	1	162740252	162740252	Missense_Mutation	SNP	C	T	T	DDR2	c.1454C>T	p.S485F
117E1396A1	12	25398285	25398285	Missense_Mutation	SNP	C	A	A	KRAS	c.34G>T	p.G12C
117E1396A1	17	7578469	7578469	Missense_Mutation	SNP	C	A	A	TP53	c.461G>T	p.G154V
117E1396A1	9	21971186	21971186	Nonsense_Mutation	SNP	G	A	A	CDKN2A	c.172C>T	p.R58*
117E1396A1	1	9783284	9783284	Missense_Mutation	SNP	A	G	G	PIK3CD	c.2528A>G	p.N843S
117E1396A1	X	76814235	76814235	Missense_Mutation	SNP	C	A	A	ATRX	c.6409G>T	p.A2137S
117E1396A1	1	97847966	97847966	Missense_Mutation	SNP	G	A	A	DPYD	c.1957C>T	p.L653F
117E1396A1	11	92534624	92534624	Missense_Mutation	SNP	A	C	C	FAT3	c.8445A>C	p.E2815D
117E1396A1	13	102379019	102379019	Missense_Mutation	SNP	C	A	A	FGF14	c.565G>T	p.G189W
117E1396A1	20	57415798	57415798	Nonsense_Mutation	SNP	G	T	T	GNAS	c.637G>T	p.E213*
117E1396A1	4	142949972	142949972	Missense_Mutation	SNP	C	A	A	INPP4B	c.2738G>T	p.R913I
117E1396A1	7	142641494	142641494	Splice_Site	SNP	A	G	G	KEL	c.1414-7T>C	-
117E1396A1	2	141122329	141122329	Nonsense_Mutation	SNP	C	A	A	LRP1B	c.11032G>T	p.E3678*
117E1396A1	1	40363571	40363571	Missense_Mutation	SNP	T	C	C	MYCL	c.568A>G	p.T190A
117E1396A1	1	120483388	120483388	Splice_Site	SNP	C	A	A	NOTCH2	c.2982-9G>T	-
117E1396A1	10	83635580	83635580	Missense_Mutation	SNP	A	T	T	NRG3	c.484A>T	p.T162S
117E1396A1	10	83635460	83635460	Missense_Mutation	SNP	C	A	A	NRG3	c.364C>A	p.P122T
117E1396A1	8	48872717	48872717	Splice_Site	SNP	C	A	A	PRKDC	c.-31G>T	-
117E1396A1	1	158592947	158592947	Missense_Mutation	SNP	T	A	A	SPTA1	c.5946A>T	p.Q1982H
117E1425A1	5	161116687	161116687	Missense_Mutation	SNP	C	A	A	GABRA6	c.575C>A	p.P192Q
117E1443A1	9	133760796	133760796	Missense_Mutation	SNP	G	A	A	ABL1	c.3176G>A	p.C1059Y
117E1443A1	3	178916646	178916646	Missense_Mutation	SNP	G	C	C	PIK3CA	c.33G>C	p.W11C
117E1443A1	17	7578442	7578442	Missense_Mutation	SNP	T	C	C	TP53	c.488A>G	p.Y163C
117E1443A1	9	21971116	21971116	Missense_Mutation	SNP	G	A	A	CDKN2A	c.242C>T	p.P81L
117E1443A1		-	-	In_Frame_Del	INS	-	-	-	ARID1B	c.369_392del	p.Q124_Q131del
117E1443A1		-	-	Frame_Shift_Del	INS	-	-	-	ATR	c.4670del	p.K1557Sfs*8
117E1443A1	11	92533398	92533398	Missense_Mutation	SNP	G	A	A	FAT3	c.7219G>A	p.A2407T
117E1443A1	1	206653375	206653375	Missense_Mutation	SNP	G	C	C	IKBKE	c.1259G>C	p.G420A
117E1443A1	2	141473639	141473639	Missense_Mutation	SNP	A	T	T	LRP1B	c.5926T>A	p.L1976I
117E1443A1		-	-	In_Frame_Del	INS	-	-	-	SMAD4	c.998_1009del	p.V333_G336del
117E1534A1	5	38982019	38982019	Missense_Mutation	SNP	G	A	A	RICTOR	c.703C>T	p.L235F
117E1534A1	5	156672790	156672790	Missense_Mutation	SNP	G	A	A	ITK	c.1504G>A	p.G502R
117E1534A1	15	41021015	41021015	Missense_Mutation	SNP	G	C	C	RAD51	c.637G>C	p.E213Q
117E1534A1	5	121736682	121736682	Missense_Mutation	SNP	A	G	G	SNCAIP	c.59A>G	p.D20G
117E1534A1	X	123020156	123020156	Missense_Mutation	SNP	G	A	A	XIAP	c.644G>A	p.R215H
117F1556A1	15	90631838	90631838	Missense_Mutation	SNP	C	T	T	IDH2	c.515G>A	p.R172K
117F1556A1		-	-	Frame_Shift_Ins	INS	-	-	-	ARID1A	c.1113dup	p.Q372Afs*28
117F1640A1	15	90631628	90631628	Missense_Mutation	SNP	G	T	T	IDH2	c.641C>A	p.A214E
117F1640A1	17	7579406	7579406	Nonsense_Mutation	SNP	G	T	T	TP53	c.281C>A	p.S94*
117F1640A1	17	48262951	48262951	Missense_Mutation	SNP	C	T	T	COL1A1	c.4307G>A	p.R1436H
117F1640A1		-	-	Splice_Site	SNP	-	-	-	CDK8	c.204+2dupT	-
117F1640A1	11	92532310	92532310	Missense_Mutation	SNP	G	A	A	FAT3	c.6131G>A	p.R2044H
117F1640A1	8	11606603	11606603	Splice_Site	SNP	G	A	A	GATA4	c.783+9G>A	-
117F1640A1	17	74470540	74470540	Missense_Mutation	SNP	C	G	G	RHBDF2	c.1466G>C	p.R489P
117F1640A1	2	198266713	198266713	Missense_Mutation	SNP	C	T	T	SF3B1	c.2219G>A	p.G740E
117F1648A2	2	141215115	141215115	Nonsense_Mutation	SNP	G	T	T	LRP1B	c.9731C>A	p.S3244*
117F1648A2	2	209113113	209113113	Missense_Mutation	SNP	G	A	A	IDH1	c.394C>T	p.R132C
117F1648A2	7	42012189	42012189	Missense_Mutation	SNP	C	T	T	GLI3	c.1850G>A	p.R617H
117F1689A1	2	209113113	209113113	Missense_Mutation	SNP	G	A	A	IDH1	c.394C>T	p.R132C
117F1689A1		-	-	Frame_Shift_Del	INS	-	-	-	EPHA2	C.351_357del	P.F119Sfs*41
117F1689A1	7	151962169	151962169	Missense_Mutation	SNP	G	T	T	KMT2C	c.1138C>A	p.R380S
117F1759A1	11	108213974	108213974	Missense_Mutation	SNP	G	T	T	ATM	c.8294G>T	p.G2765V
117F1759A1	8	38273409	38273409	Missense_Mutation	SNP	C	T	T	FGFR1	c.1833G>A	p.M611I
117F1759A1	19	1220649	1220649	Missense_Mutation	SNP	G	A	A	STK11	c.667G>A	p.E223K
117F1759A1		-	-	Frame_Shift_Del	INS	-	-	-	ARID1A	c.264_270del	p.G89Afs*10
117F1759A1	19	10599915	10599915	Missense_Mutation	SNP	C	A	A	KEAP1	c.1661G>T	p.R554L
117F1759A1	12	133263850	133263850	Nonsense_Mutation	SNP	C	A	A	POLE	c.52G>T	p.E18*
117F1759A1	19	45504848	45504848	Missense_Mutation	SNP	C	G	G	RELB	c.11C>G	p.S4C
117F1759A1	19	11144147	11144147	Missense_Mutation	SNP	G	A	A	SMARCA4	c.3728G>A	p.R1243Q
117F1767A1	12	25398285	25398285	Missense_Mutation	SNP	C	G	G	KRAS	c.34G>C	p.G12R
117F1767A1		-	-	Frame_Shift_Ins	INS	-	-	-	TP53	C.243_244dup	P.P82Hfs*42
117F1767A1	1	27102076	27102076	Nonsense_Mutation	SNP	G	T	T	ARID1A	c.5002G>T	p.E1668*
117F1767A1	6	157521932	157521932	Nonsense_Mutation	SNP	G	T	T	ARID1B	c.4204G>T	p.E1402*
117F1767A1	16	3808012	3808012	Missense_Mutation	SNP	G	A	A	CREBBP	3407C>T;3414C	[S1136F;I1138M]
117F1767A1	7	151875055	151875055	Missense_Mutation	SNP	G	C	C	KMT2C	c.7483C>G	p.Q2495E
117F1770A1		-	-	Frame_Shift_Ins	INS	-	-	-	ATM	c.5712dup	p.S1905Ifs*25
117F1770A1		-	-	Frame_Shift_Del	INS	-	-	-	STK11	c.785del	p.K262Sfs*25
117F1770A1	2	121726437	121726437	Missense_Mutation	SNP	G	A	A	GLI2	c.791G>A	p.R264Q
117F1770A1	12	57589078	57589078	Missense_Mutation	SNP	C	G	G	LRP1	c.8333C>G	p.S2778C
117F1770A1	18	18548826	18548826	Splice_Site	SNP	C	T	T	ROCK1	c.2915-5G>A	-
117F1770A1	17	70120232	70120232	Nonsense_Mutation	SNP	C	T	T	SOX9	c.1234C>T	p.Q412*
117F1770A1	13	108922284	108922284	Missense_Mutation	SNP	C	T	T	TNFSF13B	c.41C>T	p.S14F
117F1811A1	19	1221989	1221989	Nonsense_Mutation	SNP	C	T	T	STK11	c.904C>T	p.Q302*
117F1811A1	14	75745705	75745705	Missense_Mutation	SNP	A	G	G	FOS	c.20A>G	p.N7S
117G2016A1	2	209113113	209113113	Missense_Mutation	SNP	G	A	A	IDH1	c.394C>T	p.R132C
117G2016A1	7	151891600	151891600	Nonsense_Mutation	SNP	G	A	A	KMT2C	c.4432C>T	p.Q1478*
117G2016A1	1	11172979	11172979	Splice_Site	SNP	G	T	T	MTOR	c.7301-5C>A	-
117G2026A1		-	-	Frame_Shift_Del	INS	-	-	-	ARID1A	c.4982_4985del	p.M1661Kfs*11
117G2026A1	17	7577559	7577559	Missense_Mutation	SNP	G	A	A	TP53	c.722C>T	p.S241F
117G2026A1	19	10610285	10610285	Missense_Mutation	SNP	G	A	A	KEAP1	c.425C>T	p.T142M
117G2087A1	7	140481402	140481402	Missense_Mutation	SNP	C	G	G	BRAF	c.1406G>C	p.G469A
117G2087A1	17	7574034	7574034	Splice_Site	SNP	C	T	T	TP53	c.994-1G>A	-
117G2087A1		-	-	Frame_Shift_Ins	INS	-	-	-	ARID1A	c.4518_4519dup	p.N1507Ifs*21
117G2087A1	1	27102132	27102132	Nonsense_Mutation	SNP	G	A	A	ARID1A	c.5058G>A	p.W1686*
117G2087A1	22	41572870	41572870	Nonsense_Mutation	SNP	C	T	T	EP300	c.5155C>T	p.Q1719*
117G2087A1	3	134670774	134670774	Missense_Mutation	SNP	G	A	A	EPHB1	c.685G>A	p.A229T
117G2087A1	4	143191848	143191848	Missense_Mutation	SNP	C	G	G	INPP4B	c.583G>C	p.D195H
117G2087A1	7	142638388	142638388	Missense_Mutation	SNP	C	T	T	KEL	c.2150G>A	p.C717Y
117G2087A1	19	15280950	15280950	Missense_Mutation	SNP	C	G	G	NOTCH3	c.5146G>C	p.E1716Q
117G2087A1	8	68965472	68965472	Missense_Mutation	SNP	C	T	T	PREX2	c.1084C>T	p.R362W

117G2087A1		-	-	Frame_Shift_Del	INS	-	-	-	SMAD4	c.1586del	p.L529Yfs*8
117H2198A1		-	-	Frame_Shift_Del	INS	-	-	-	TP53	c.902del	p.P301Qfs*44
117H2198A1	2	141200100	141200100	Missense_Mutation	SNP	C	G	G	LRP1B	c.10387G>C	p.A3463P
117H2255A1	11	69633620	69633620	Missense_Mutation	SNP	C	A	A	FGF3	c.82G>T	p.D28Y
117H2255A1		-	-	Frame_Shift_Ins	INS	-	-	-	ARID2	C.282dup	P.R95Afs*16
117H2255A1		-	-	Frame_Shift_Del	INS	-	-	-	ARID2	C.1261del	P.V421Cfs*19
117H2255A1	15	88472463	88472463	Missense_Mutation	SNP	A	T	T	NTRK3	c.2092T>A	p.F698I
117H2255A1	2	178098953	178098953	Missense_Mutation	SNP	C	T	T	NFE2L2	c.92G>A	p.G31E
117H2255A1	20	54945564	54945564	Missense_Mutation	SNP	C	G	G	AURKA	c.1006G>C	p.E336Q
117H2255A1	3	178936082	178936082	Missense_Mutation	SNP	G	A	A	PIK3CA	c.1624G>A	p.E542K
117H2255A1	6	157099560	157099560	Missense_Mutation	SNP	C	T	T	ARID1B	c.497C>T	p.A166V
117H2255A1	7	117232211	117232211	Missense_Mutation	SNP	G	A	A	CFTR	c.1990G>A	p.E664K
117H2255A1	X	39923194	39923194	Missense_Mutation	SNP	C	G	G	BCOR	c.3514G>C	p.E1172Q
117H2255A1	X	39923194	39923194	Missense_Mutation	SNP	C	T	T	BCOR	c.3514G>A	p.E1172K
117H2289A1	1	27102066	27102066	Splice_Site	SNP	A	T	T	ARID1A	c.4994-2A>T	-
117H2289A1	11	92533302	92533302	Missense_Mutation	SNP	A	T	T	FAT3	c.7123A>T	p.S2375C
117H2289A1		-	-	Frame_Shift_Del	INS	-	-	-	TP53	c.153del	p.E51Dfs*72
117H2289A1	19	15285084	15285084	Missense_Mutation	SNP	C	T	T	NOTCH3	c.4531G>A	p.G1511S
117H2289A1	19	15302470	15302470	Splice_Site	SNP	T	A	A	NOTCH3	c.803-2A>T	-
117H2289A1	2	11351887	11351887	Missense_Mutation	SNP	T	A	A	ROCK2	c.2123A>T	p.H708L
117H2289A1	7	6027263	6027263	Splice_Site	SNP	T	A	A	PMS2	c.1145-12A>T	-
117H2289A1	X	53244031	53244031	Splice_Site	SNP	T	A	A	KDM5C	c.964-2A>T	-
117H2404A1		-	-	Splice_Site	SNP	-	-	-	PIK3CD	c.1690-3delC	-
117H2404A1	1	16477434	16477434	Missense_Mutation	SNP	G	A	A	EPHA2	c.110C>T	p.A37V
117H2404A1		-	-	Frame_Shift_Del	INS	-	-	-	ARID1A	c.4555del	p.Q1519Rfs*8
117H2404A1		-	-	Frame_Shift_Del	INS	-	-	-	ARID1A	c.5548del	p.D1850Tfs*33
117H2404A1	12	416672	416672	Missense_Mutation	SNP	T	C	C	KDM5A	c.3878A>G	p.E1293G
117H2404A1	12	25398279	25398279	Missense_Mutation	SNP	C	T	T	KRAS	c.40G>A	p.V14I
117H2404A1		-	-	Frame_Shift_Del	INS	-	-	-	KMT2D	c.7061del	p.P2354Lfs*30
117H2404A1		-	-	Frame_Shift_Del	INS	-	-	-	KMT2D	c.1940del	p.P647Hfs*283
117H2404A1	13	25060405	25060405	Missense_Mutation	SNP	C	T	T	PARP4	c.1253G>A	p.G418D
117H2404A1	14	68758655	68758655	Missense_Mutation	SNP	G	A	A	RAD51B	c.811G>A	p.A271T
117H2404A1	15	67482825	67482825	Missense_Mutation	SNP	T	A	A	SMAD3	c.1229T>A	p.V410D
117H2404A1		-	-	Frame_Shift_Del	INS	-	-	-	BLM	c.3651del	p.K1217Nfs*62
117H2404A1		-	-	Frame_Shift_Del	INS	-	-	-	AXIN1	c.978del	p.S327Pfs*87
117H2404A1		-	-	In_Frame_Del	INS	-	-	-	CDH1	c.2064_2065del	p.C688*
117H2404A1	19	11132462	11132462	Missense_Mutation	SNP	T	C	C	SMARCA4	c.2678T>C	p.L893P
117H2404A1	19	42795540	42795540	Missense_Mutation	SNP	G	A	A	CIC	c.2620G>A	p.G874S
117H2404A1		-	-	Frame_Shift_Ins	INS	-	-	-	POLD1	c.3231dup	p.I1078Hfs*28
117H2404A1	2	74273951	74273951	Missense_Mutation	SNP	A	G	G	TET3	c.907A>G	p.R303G
117H2404A1		-	-	Frame_Shift_Del	INS	-	-	-	ACVR2A	c.677_680del	p.K226Sfs*15
117H2404A1		-	-	Frame_Shift_Del	INS	-	-	-	ACVR2A	c.1310del	p.K437Rfs*5
117H2404A1	2	170092549	170092549	Missense_Mutation	SNP	C	T	T	LRP2	c.4721G>A	p.G1574D
117H2404A1		-	-	Splice_Site	SNP	-	-	-	SRC	c.-4-9_-4-8delT	-
117H2404A1		-	-	Missense_Mutation	SNP	-	-	-	CTNNB1	c.133_135del	p.S45del
117H2404A1		-	-	Frame_Shift_Del	INS	-	-	-	GATA2	c.599del	p.G200Vfs*18
117H2404A1	4	20258283	20258283	Splice_Site	SNP	T	G	G	SLIT2	c.180-12T>G	-
117H2404A1	4	55151582	55151582	Missense_Mutation	SNP	C	A	A	PDGFRA	c.2368C>A	p.L790I
117H2404A1	4	66361144	66361144	Missense_Mutation	SNP	A	G	G	EPHA5	c.1028T>C	p.F343S
117H2404A1	4	187527264	187527264	Missense_Mutation	SNP	G	A	A	FAT1	c.10310C>T	p.A3437V
117H2404A1	4	187557310	187557310	Missense_Mutation	SNP	G	A	A	FAT1	c.4052C>T	p.P1351L
117H2404A1	6	41905071	41905071	Missense_Mutation	SNP	T	A	A	CCND3	c.476A>T	p.D159V
117H2404A1	6	157256712	157256712	Splice_Site	SNP	T	C	C	ARID1B	c.2037+2T>C	-
117H2404A1	6	157517367	157517367	Missense_Mutation	SNP	A	G	G	ARID1B	c.3931A>G	p.N1311D
117H2404A1	6	162683710	162683710	Missense_Mutation	SNP	C	T	T	PRKN	c.259G>A	p.D87N
117H2404A1	6	163984691	163984691	Missense_Mutation	SNP	T	C	C	QKI	c.874T>C	p.Y292H
117H2404A1		-	-	Frame_Shift_Del	INS	-	-	-	MAGI2	c.277del	p.L93Sfs*81
117H2404A1		-	-	Frame_Shift_Del	INS	-	-	-	ABL1	c.2409del	p.R804Gfs*3
117H2404A1		-	-	Frame_Shift_Del	INS	-	-	-	KDM6A	c.1237del	p.T413Lfs*26
117H2404A1	X	66765161	66765161	Missense_Mutation	SNP	A	T	T	AR	c.173A>T	p.Q58L
117H2427A1	11	92573800	92573800	Missense_Mutation	SNP	C	T	T	FAT3	c.10441C>T	p.P3481S
117H2427A1	11	119146732	119146732	Missense_Mutation	SNP	C	T	T	CBL	c.895C>T	p.R299C
117H2427A1	12	49443583	49443583	Nonsense_Mutation	SNP	G	C	C	KMT2D	c.3788C>G	p.S1263*
117H2427A1	16	56782202	56782202	Nonsense_Mutation	SNP	C	T	T	NUP93	c.43C>T	p.Q15*
117H2427A1	17	7578275	7578275	Nonsense_Mutation	SNP	G	A	A	TP53	c.574C>T	p.Q192*
117H2427A1	17	11998985	11998985	Nonsense_Mutation	SNP	C	T	T	MAP2K4	c.487C>T	p.Q163*
117H2427A1	17	70119789	70119789	Missense_Mutation	SNP	G	A	A	SOX9	c.791G>A	p.R264K
117H2427A1	19	2210734	2210734	Missense_Mutation	SNP	C	T	T	DOT1L	c.1231C>T	p.R411C
117H2427A1	2	178098953	178098953	Missense_Mutation	SNP	C	G	G	NFE2L2	c.92G>C	p.G31A
117H2427A1	20	36031708	36031708	Missense_Mutation	SNP	G	C	C	SRC	c.1537G>C	p.E513Q
117H2427A1	3	178936082	178936082	Missense_Mutation	SNP	G	A	A	PIK3CA	c.1624G>A	p.E542K
117H2427A1	3	183273347	183273347	Missense_Mutation	SNP	G	C	C	KLHL6	c.95C>G	p.S32C
117H2427A1		-	-	In_Frame_Del	INS	-	-	-	CDKN2A	c.132del	p.Y44*
117H2427A1	X	53239895	53239895	Nonsense_Mutation	SNP	C	A	A	KDM5C	c.1546G>T	p.E516*
117H2427A1	X	76855262	76855262	Missense_Mutation	SNP	C	T	T	ATRX	c.5725G>A	p.E1909K
117H2427A1		-	-	Frame_Shift_Del	INS	-	-	-	STAG2	c.2848_2849del	p.E950Tfs*12
117H2467A1	11	46337914	46337914	Missense_Mutation	SNP	C	T	T	CREB3L1	c.1109C>T	p.T370I
117H2467A1	2	209113113	209113113	Missense_Mutation	SNP	G	A	A	IDH1	c.394C>T	p.R132C
117H2467A1	3	178952085	178952085	Missense_Mutation	SNP	A	T	T	PIK3CA	c.3140A>T	p.H1047L
117H2489A1	1	158590091	158590091	Missense_Mutation	SNP	G	T	T	SPTA1	c.6286C>A	p.Q2096K
117H2489A1	1	226567659	226567659	Missense_Mutation	SNP	G	A	A	PARP1	c.1507C>T	p.L503F
117H2489A1	11	92532310	92532310	Missense_Mutation	SNP	G	A	A	FAT3	c.6131G>A	p.R2044H
117H2489A1	12	25398284	25398284	Missense_Mutation	SNP	C	T	T	KRAS	c.35G>A	p.G12D
117H2489A1		-	-	Splice_Site	SNP	-	-	-	TP53	93+1_993+3deli	-
117H2489A1		-	-	Frame_Shift_Ins	INS	-	-	-	SMAD4	:.1399_1414dup	p.P472Rfs*27
117H2489A1	22	41574752	41574752	Missense_Mutation	SNP	C	T	T	EP300	c.7037C>T	p.S2346F
117H2489A1		-	-	In_Frame_Del	INS	-	-	-	CDKN2A	c.90_96delinsT	p.L31_L32del
117H2552A1	1	158614075	158614075	Missense_Mutation	SNP	G	A	A	SPTA1	c.4306C>T	p.R1436W
117H2552A1	12	25380275	25380275	Missense_Mutation	SNP	T	G	G	KRAS	c.183A>C	p.Q61H
117H2552A1	12	115118688	115118688	Missense_Mutation	SNP	C	T	T	TBX3	c.653G>A	p.G218E
117H2552A1	15	67473760	67473760	Missense_Mutation	SNP	T	A	A	SMAD3	c.840T>A	p.N280K
117H2552A1	17	7577084	7577084	Missense_Mutation	SNP	T	A	A	TP53	c.854A>T	p.E285V
117H2552A1	19	11132468	11132468	Missense_Mutation	SNP	A	T	T	SMARCA4	c.2684A>T	p.Q895L
117H2552A1	3	41266137	41266137	Missense_Mutation	SNP	C	T	T	CTNNB1	c.134C>T	p.S45F
117H2552A1	3	47164663	47164663	Missense_Mutation	SNP	T	A	A	SETD2	c.1463A>T	p.Y488F
117H2552A1	5	1293461	1293461	Missense_Mutation	SNP	C	T	T	TERT	c.1540G>A	p.V514M
117H2552A1	7	6026741	6026741	Missense_Mutation	SNP	T	C	C	PMS2	c.1655A>G	p.H552R
117H2552A1		-	-	Frame_Shift_Ins	INS	-	-	-	RBM10	:.1620_1627dup	p.T543Rfs*164

117I2596A1	1	158582613	158582613	Missense_Mutation	SNP	C	T	T	SPTA1	c.7128G>A	p.M2376I
117I2596A1	11	8251907	8251907	Missense_Mutation	SNP	C	T	T	LMO1	c.170G>A	p.R57H
117I2596A1	13	102521064	102521064	Splice_Site	SNP	G	A	A	FGF14	c.423+11C>T	-
117I2596A1	16	9857380	9857380	Missense_Mutation	SNP	T	C	C	GRIN2A	c.4021A>G	p.S1341G
117I2596A1		-	-	In_Frame_Ins	INS	-	-	-	TP53	66_467insCCGT	.T155_R156insPV
117I2596A1	18	48575102	48575102	Nonsense_Mutation	SNP	G	A	A	SMAD4	c.296G>A	p.W99*
117I2596A1	2	140990778	140990778	Missense_Mutation	SNP	C	T	T	LRP1B	c.13777G>A	p.G4593S
117I2596A1	7	140453127	140453127	Missense_Mutation	SNP	C	T	T	BRAF	c.1808G>A	p.R603Q
117I2596A1	7	151845726	151845726	Missense_Mutation	SNP	T	A	A	KMT2C	c.13286A>T	p.H4429L
117I2661A1	12	49434040	49434040	Nonsense_Mutation	SNP	C	A	A	KMT2D	c.7513G>T	p.E2505*
117I2661A1		-	-	Splice_Site	SNP	-	-	-	CHD2	907-12_4907-9	-
117I2661A1	17	29657313	29657313	Splice_Site	SNP	G	A	A	NF1	c.5610-1G>A	-
117I2661A1	2	198263263	198263263	Missense_Mutation	SNP	C	G	G	SF3B1	c.3056G>C	p.R1019T
117I2661A1	4	126371893	126371893	Missense_Mutation	SNP	T	A	A	FAT4	c.9722T>A	p.F3241Y
117I2661A1	9	137300846	137300846	Missense_Mutation	SNP	G	A	A	RXRA	c.491G>A	p.R164H
117I2687A1	1	16255661	16255661	Missense_Mutation	SNP	G	T	T	SPEN	c.2926G>T	p.V976L
117I2687A1	6	394867	394867	Missense_Mutation	SNP	C	T	T	IRF4	c.263C>T	p.P88L
117I2735A1	1	158615041	158615041	Missense_Mutation	SNP	T	A	A	SPTA1	c.4131A>T	p.R1377S
117I2735A1	11	108151875	108151875	Missense_Mutation	SNP	G	A	A	ATM	c.3556G>A	p.E1186K
117I2735A1	12	46233261	46233261	Missense_Mutation	SNP	C	T	T	ARID2	c.1480C>T	p.H494Y
117I2735A1	15	88428973	88428973	Splice_Site	SNP	A	T	T	NTRK3	c.2134-7T>A	-
117I2735A1	17	7578190	7578190	Missense_Mutation	SNP	T	C	C	TP53	c.659A>G	p.Y220C
117I2735A1	18	48591888	48591888	Missense_Mutation	SNP	G	C	C	SMAD4	c.1051G>C	p.D351H
117I2735A1	4	153249504	153249504	Missense_Mutation	SNP	C	G	G	FBXW7	c.1274G>C	p.W425S
117I2735A1		-	-	Frame_Shift_Del	INS	-	-	-	APC	c.4612_4613del	p.E1538Ifs*5
117I2735A1		-	-	Frame_Shift_Del	INS	-	-	-	ARID1B	c.502_550del	p.G168Tfs*82
117I2735A1	7	140481411	140481411	Missense_Mutation	SNP	C	G	G	BRAF	c.1397G>C	p.G466A
117I2735A1	9	98211585	98211585	Missense_Mutation	SNP	C	G	G	PTCH1	c.3570G>C	p.L1190F
117I2735A1	X	44929487	44929487	Nonsense_Mutation	SNP	C	T	T	KDM6A	c.2743C>T	p.Q915*
117I2735A1	X	70641188	70641188	Missense_Mutation	SNP	C	G	G	TAF1	c.4474C>G	p.Q1492E
117I2766A1	1	8075447	8075447	Splice_Site	SNP	G	C	C	ERRFI1	c.126-3C>G	-
117I2766A1	1	43770579	43770579	Missense_Mutation	SNP	T	G	G	TIE1	c.116T>G	p.F39C
117I2766A1	10	89624310	89624310	Splice_Site	SNP	C	G	G	PTEN	c.79+5C>G	-
117I2766A1	2	141609279	141609279	Missense_Mutation	SNP	A	C	C	LRP1B	c.4653T>G	p.S1551R
117I2766A1	7	73535316	73535316	Missense_Mutation	SNP	C	T	T	LIMK1	c.1718C>T	p.P573L
117I2799A1	12	6702686	6702686	Missense_Mutation	SNP	C	T	T	CHD4	c.2410G>A	p.V804I
117I2799A1	17	7577556	7577556	Missense_Mutation	SNP	C	G	G	TP53	c.725G>C	p.C242S
117I2805A1	1	27106747	27106747	Nonsense_Mutation	SNP	G	T	T	ARID1A	c.6358G>T	p.E2120*
117I2805A1	17	7578496	7578496	Missense_Mutation	SNP	A	G	G	TP53	c.434T>C	p.L145P
117I2805A1	17	12016658	12016658	Missense_Mutation	SNP	G	A	A	MAP2K4	c.794G>A	p.G265D
117I2805A1	18	48575671	48575671	Nonsense_Mutation	SNP	C	G	G	SMAD4	c.431C>G	p.S144*
117I2805A1		-	-	Frame_Shift_Ins	INS	-	-	-	RBM10	c.1268dup	p.T424Yfs*19
117I2844A1		-	-	Frame_Shift_Del	INS	-	-	-	FGF6	c.328del	p.H110Tfs*19
117I2844A1		-	-	Frame_Shift_Del	INS	-	-	-	NF1	c.3720_3726del	p.R1241Wfs*23
117I2844A1	17	29684098	29684098	Missense_Mutation	SNP	T	G	G	NF1	c.7859T>G	p.L2620R
117I2844A1	3	30691911	30691911	Missense_Mutation	SNP	G	T	T	TGFBR2	c.413G>T	p.C138F
117I2844A1	7	140453193	140453193	Missense_Mutation	SNP	T	A	A	BRAF	c.1742A>T	p.N581I
117I2845A1	1	16235953	16235953	Missense_Mutation	SNP	A	G	G	SPEN	c.1019A>G	p.Q340R
117I2845A1	1	27087961	27087961	Nonsense_Mutation	SNP	C	T	T	ARID1A	c.2248C>T	p.R750*
117I2845A1	1	156846188	156846188	Splice_Site	SNP	C	A	A	NTRK1	c.1615-4C>A	-
117I2845A1	10	43614982	43614982	Missense_Mutation	SNP	C	T	T	RET	c.2396C>T	p.P799L
117I2845A1	10	70333117	70333117	Missense_Mutation	SNP	C	T	T	TET1	c.1022C>T	p.A341V
117I2845A1	11	108114722	108114722	Missense_Mutation	SNP	A	G	G	ATM	c.539A>G	p.Q180R
117I2845A1	17	7577120	7577120	Missense_Mutation	SNP	C	A	A	TP53	c.818G>T	p.R273L
117I2845A1	17	70120193	70120193	Missense_Mutation	SNP	A	T	T	SOX9	c.1195A>T	p.T399S
117I2845A1	17	80789374	80789374	Nonsense_Mutation	SNP	G	T	T	ZNF750	c.957C>A	p.Y319*
117I2845A1	19	17955124	17955124	Missense_Mutation	SNP	G	T	T	JAK3	c.103C>A	p.P35T
117I2845A1	2	225342966	225342966	Missense_Mutation	SNP	C	T	T	CUL3	c.2126G>A	p.R709Q
117I2845A1	3	178936091	178936091	Missense_Mutation	SNP	G	A	A	PIK3CA	c.1633G>A	p.E545K
117I2845A1	6	43738502	43738502	Missense_Mutation	SNP	G	A	A	VEGFA	c.59G>A	p.R20Q
117I2845A1	8	69058436	69058436	Splice_Site	SNP	C	G	G	PREX2	c.4088-8C>G	-
117I2845A1		-	-	Frame_Shift_Del	INS	-	-	-	CDKN2A	c.253del	p.A85Lfs*61
117I2858A1	1	115256529	115256529	Missense_Mutation	SNP	T	C	C	NRAS	c.182A>G	p.Q61R
117I2858A1	1	158615326	158615326	Missense_Mutation	SNP	C	T	T	SPTA1	c.3955G>A	p.E1319K
117I2858A1	2	209113113	209113113	Missense_Mutation	SNP	G	A	A	IDH1	c.394C>T	p.R132C
117I2888A1		-	-	Frame_Shift_Del	INS	-	-	-	ARID1A	559_2561delins	p.P854Ifs*5
117I2888A1	12	25398284	25398284	Missense_Mutation	SNP	C	A	A	KRAS	c.35G>T	p.G12V
117I2888A1	12	56495760	56495760	Missense_Mutation	SNP	A	T	T	ERBB3	c.3950A>T	p.E1317V
117I2888A1	18	48604667	48604667	Missense_Mutation	SNP	C	T	T	SMAD4	c.1489C>T	p.R497C
117I2888A1	21	44524472	44524472	Missense_Mutation	SNP	G	T	T	U2AF1	c.85C>A	p.H29N
117I2888A1	X	44945149	44945149	Missense_Mutation	SNP	A	T	T	KDM6A	c.3629A>T	p.N1210I
117I2888A1	X	47039620	47039620	Nonsense_Mutation	SNP	C	T	T	RBM10	c.1072C>T	p.Q358*
117I2908A1	1	2492165	2492165	Splice_Site	SNP	C	T	T	TNFRSF14	c.551+12C>T	-
117I2908A1	17	7578450	7578450	Missense_Mutation	SNP	C	T	T	TP53	[480G>A;481G>	o.[M160I;A161S]
117I2908A1		-	-	In_Frame_Del	INS	-	-	-	GATA6	c.531_548del	o.A178_A183del
117I2908A1	19	10600527	10600527	Missense_Mutation	SNP	T	C	C	KEAP1	c.1328A>G	p.Y443C
117I2908A1	3	30713733	30713733	Missense_Mutation	SNP	C	A	A	TGFBR2	c.1058C>A	p.S353Y
117I2908A1	6	93982034	93982034	Missense_Mutation	SNP	T	A	A	EPHA7	c.1431A>T	p.E477D
117I2919A1	1	156838381	156838381	Missense_Mutation	SNP	G	A	A	NTRK1	c.659G>A	p.R220Q
117I2919A1		-	-	Frame_Shift_Ins	INS	-	-	-	ARID2	C.5249dup	P.N1750Kfs*10
117I2919A1	17	7578518	7578518	Missense_Mutation	SNP	C	G	G	TP53	c.412G>C	p.A138P
117I2919A1	17	37868208	37868208	Missense_Mutation	SNP	C	A	A	ERBB2	c.929C>A	p.S310Y
117I2919A1	19	15271796	15271796	Missense_Mutation	SNP	G	C	C	NOTCH3	c.6643C>G	p.P2215A
117I2919A1	2	141598583	141598583	Missense_Mutation	SNP	G	A	A	LRP1B	c.5018C>T	p.T1673M
117I2919A1	3	89456470	89456470	Missense_Mutation	SNP	C	T	T	EPHA3	c.1646C>T	p.A549V
117I2919A1	3	134898755	134898755	Missense_Mutation	SNP	G	A	A	EPHB1	c.1813G>A	p.E605K
117I2919A1		-	-	In_Frame_Del	INS	-	-	-	APC	C.2853_2858de	P.Y951*
117I2919A1		-	-	Frame_Shift_Ins	INS	-	-	-	APC	C.4666dup	P.T1556Nfs*3
117I2931A1	1	204402554	204402554	Splice_Site	SNP	C	T	T	PIK3C2B	c.3844-1G>A	-
117I2931A1	11	125499172	125499172	Missense_Mutation	SNP	A	G	G	CHEK1	c.335A>G	p.H112R
117I2931A1	17	7578265	7578265	Missense_Mutation	SNP	A	T	T	TP53	c.584T>A	p.I195N
117I2931A1	17	66511703	66511703	Missense_Mutation	SNP	G	C	C	PRKAR1A	c.163G>C	p.E55Q
117I2931A1	18	19761540	19761540	Splice_Site	SNP	G	A	A	GATA6	c.1428+1G>A	-
117I2931A1	18	48584552	48584552	Nonsense_Mutation	SNP	C	G	G	SMAD4	c.725C>G	p.S242*
117I2931A1	3	130889693	130889693	Missense_Mutation	SNP	C	T	T	NEK11	c.1361C>T	p.S454L
117I2931A1	3	178936091	178936091	Missense_Mutation	SNP	G	A	A	PIK3CA	c.1633G>A	p.E545K
117I2931A1	4	110864995	110864995	Splice_Site	SNP	C	G	G	EGF	c.510-3C>G	-
117I2931A1	5	112175897	112175897	Nonsense_Mutation	SNP	G	T	T	APC	c.4606G>T	p.E1536*

117I2931A1	6	152129279	152129279	Missense_Mutation	SNP	C	G	G	ESR1	c.232C>G	p.L78V
117I2931A1	7	14027714	14027714	Missense_Mutation	SNP	C	T	T	ETV1	c.130G>A	p.E44K
117I2931A1	8	93003987	93003987	Missense_Mutation	SNP	G	A	A	RUNX1T1	c.760C>T	p.H254Y
117I2978A1	11	108141854	108141854	Nonsense_Mutation	SNP	G	T	T	ATM	c.2902G>T	p.E968*
117I2978A1	12	25398284	25398284	Missense_Mutation	SNP	C	T	T	KRAS	c.35G>A	p.G12D
117J3004A1		-	-	Frame_Shift_Del	INS	-	-	-	ARID1A	c.267_295del	p.S90Pfs*11
117J3004A1		-	-	Frame_Shift_Ins	INS	-	-	-	TP53	c.437_462dup	p.T155Gfs*24
117J3004A1	3	52610556	52610556	Splice_Site	SNP	C	T	T	PBRM1	c.3616+1G>A	-
117J3004A1	3	178916725	178916725	Missense_Mutation	SNP	C	T	T	PIK3CA	c.112C>T	p.R38C
117J3004A1	5	131972837	131972837	Missense_Mutation	SNP	G	A	A	RAD50	c.3420G>A	p.M1140I
117J3045A1	13	110437380	110437380	Missense_Mutation	SNP	G	A	A	IRS2	c.1021C>T	p.R341C
117J3045A1	17	63010902	63010902	Missense_Mutation	SNP	T	A	A	GNA13	c.607A>T	p.T203S
117J3086A1		-	-	In_Frame_Del	INS	-	-	-	SPEN	c.1711_1728del	p.E571_K576del
117J3086A1	13	48919262	48919262	Nonsense_Mutation	SNP	A	T	T	RB1	c.427A>T	p.K143*
117J3086A1	14	95572475	95572475	Nonsense_Mutation	SNP	C	A	A	DICER1	c.2890G>T	p.E964*
117J3086A1	17	7577539	7577539	Missense_Mutation	SNP	G	A	A	TP53	c.742C>T	p.R248W
117J3086A1	17	37876038	37876038	Splice_Site	SNP	A	T	T	ERBB2	c.1899-2A>T	-
117J3086A1	2	29416137	29416137	Missense_Mutation	SNP	C	T	T	ALK	c.4816G>A	p.D1606N
117J3086A1		-	-	Frame_Shift_Del	INS	-	-	-	ACVR2A	c.834del	p.K280Rfs*31
117J3086A1		-	-	Missense_Mutation	SNP	-	-	-	KIT	972_1974delinsT	p.G658W
117J3086A1	5	138163231	138163231	Missense_Mutation	SNP	T	G	G	CTNNA1	c.886T>G	p.F296V
117J3086A1	7	77756727	77756727	Missense_Mutation	SNP	C	A	A	MAGI2	c.3210G>T	p.R1070S
117J3117A1		-	-	Splice_Site	SNP	-	-	-	RB1	2520+1_2520+4d	-
117J3117A1	17	7577548	7577548	Missense_Mutation	SNP	C	T	T	TP53	c.733G>A	p.G245S
117J3117A1	7	55238870	55238870	Missense_Mutation	SNP	G	A	A	EGFR	c.1883G>A	p.C628Y
117J3117A1	7	151970960	151970960	Splice_Site	SNP	G	A	A	KMT2C	c.850-8C>T	-
117J3131A1	11	92495113	92495113	Missense_Mutation	SNP	T	G	G	FAT3	c.3761T>G	p.F1254C
117J3131A1	16	2138234	2138234	Missense_Mutation	SNP	T	C	C	TSC2	c.5167T>C	p.S1723P
117J3131A1	16	89877212	89877212	Splice_Site	SNP	T	A	A	FANCA	c.427-2A>T	-
117J3131A1	17	7577550	7577550	Missense_Mutation	SNP	C	T	T	TP53	c.731G>A	p.G244D
117J3131A1	18	19751966	19751966	Missense_Mutation	SNP	T	A	A	GATA6	c.861T>A	p.S287R
117J3131A1	2	48028142	48028142	Nonsense_Mutation	SNP	G	A	A	MSH6	c.3020G>A	p.W1007*
117J3131A1	7	20199458	20199458	Missense_Mutation	SNP	C	T	T	MACC1	c.526G>A	p.E176K
117J3131A1	8	37691530	37691530	Missense_Mutation	SNP	G	T	T	ADGRA2	c.1492G>T	p.D498Y
117J3131A1	8	41791594	41791594	Missense_Mutation	SNP	T	C	C	KAT6A	c.4144A>G	p.T1382A
117J3133A1	1	16258497	16258497	Missense_Mutation	SNP	A	T	T	SPEN	c.5762A>T	p.K1921I
117J3133A1	11	2161474	2161474	Missense_Mutation	SNP	G	A	A	IGF2	c.53C>T	p.A18V
117J3133A1	11	128651900	128651900	Nonsense_Mutation	SNP	C	T	T	FLI1	c.637C>T	p.R213*
117J3133A1	17	7578526	7578526	Missense_Mutation	SNP	C	A	A	TP53	c.404G>T	p.C135F
117J3133A1	19	11141552	11141552	Missense_Mutation	SNP	G	A	A	SMARCA4	c.3529G>A	p.D1177N
117J3133A1	19	41743892	41743892	Missense_Mutation	SNP	C	G	G	AXL	c.827C>G	p.P276R
117J3133A1	20	57415595	57415595	Missense_Mutation	SNP	G	A	A	GNAS	c.434G>A	p.R145H
117J3133A1	20	57415892	57415892	Missense_Mutation	SNP	G	A	A	GNAS	c.731G>A	p.R244H
117J3133A1	3	71050193	71050193	Missense_Mutation	SNP	T	G	G	FOXP1	c.992A>C	p.H331P
117J3133A1		-	-	Frame_Shift_Del	INS	-	-	-	FOXL2	.892_893delins	P.H298Ffs*58
117J3133A1	3	178916617	178916617	Missense_Mutation	SNP	C	T	T	PIK3CA	c.4C>T	p.P2S
117J3133A1	4	187540509	187540509	Missense_Mutation	SNP	A	G	G	FAT1	c.7231T>C	p.C2411R
117J3133A1	7	151900051	151900051	Nonsense_Mutation	SNP	T	A	A	KMT2C	c.4060A>T	p.K1354*
117J3133A1	9	139404312	139404312	Missense_Mutation	SNP	C	T	T	NOTCH1	c.2842G>A	p.E948K
117J3136A1	12	46242663	46242663	Missense_Mutation	SNP	G	A	A	ARID2	c.1625G>A	p.R542Q
117J3136A1	12	51207840	51207840	Missense_Mutation	SNP	G	A	A	ATF1	c.376G>A	p.D126N
117J3136A1	17	7577538	7577538	Missense_Mutation	SNP	C	T	T	TP53	c.743G>A	p.R248Q
117J3136A1	17	37868208	37868208	Missense_Mutation	SNP	C	A	A	ERBB2	c.929C>A	p.S310Y
117J3136A1	2	25505279	25505279	Missense_Mutation	SNP	G	A	A	DNMT3A	c.479C>T	p.S160F
117J3136A1	3	47164273	47164273	Nonsense_Mutation	SNP	G	C	C	SETD2	c.1853C>G	p.S618*
117J3136A1	3	142272239	142272239	Missense_Mutation	SNP	C	T	T	ATR	c.2635G>A	p.A879T
117J3136A1	X	44936044	44936044	Missense_Mutation	SNP	G	T	T	KDM6A	c.2961G>T	p.K987N
117J3193A1	10	43609948	43609948	Missense_Mutation	SNP	T	G	G	RET	c.1900T>G	p.C634G
117J3193A1	12	25380276	25380276	Missense_Mutation	SNP	T	C	C	KRAS	c.182A>G	p.Q61R
117J3193A1	17	7578406	7578406	Missense_Mutation	SNP	C	T	T	TP53	c.524G>A	p.R175H
117J3193A1	3	52651369	52651369	Missense_Mutation	SNP	C	G	G	PBRM1	c.1727G>C	p.R576P
117J3193A1	5	176721790	176721790	Missense_Mutation	SNP	A	C	C	NSD1	c.7421A>C	p.Q2474P
117J3193A1	7	42011956	42011956	Missense_Mutation	SNP	C	T	T	GLI3	c.2083G>A	p.V695I
117J3193A1	7	42018223	42018223	Missense_Mutation	SNP	G	A	A	GLI3	c.1622C>T	p.T541M
117J3213A1	11	108202605	108202605	Splice_Site	SNP	G	C	C	ATM	c.7630-1G>C	-
117J3213A1	12	25398284	25398284	Missense_Mutation	SNP	C	A	A	KRAS	c.35G>T	p.G12V
117J3213A1	9	80537112	80537112	Missense_Mutation	SNP	T	A	A	GNAQ	c.286A>T	p.T96S
117J3237A1	1	27094280	27094280	Splice_Site	SNP	G	A	A	ARID1A	c.2989-1G>A	-
117J3237A1	17	7577517	7577517	Missense_Mutation	SNP	A	G	G	TP53	c.764T>C	p.I255T
117J3237A1		-	-	Frame_Shift_Ins	INS	-	-	-	SMAD4	c.807dup	p.G270Wfs*3
117J3237A1	2	109381151	109381151	Missense_Mutation	SNP	G	A	A	RANBP2	c.4156G>A	p.V1386I
117J3237A1	5	112173704	112173704	Nonsense_Mutation	SNP	C	T	T	APC	c.2413C>T	p.R805*
117J3237A1	7	42005672	42005672	Missense_Mutation	SNP	T	A	A	GLI3	c.2999A>T	p.H1000L
117J3239A1	1	9783248	9783248	Missense_Mutation	SNP	C	T	T	PIK3CD	c.2492C>T	p.S831L
117J3239A1	1	244006417	244006417	Splice_Site	SNP	G	C	C	AKT3	c.46+10C>G	-
117J3239A1	11	76174909	76174909	Nonsense_Mutation	SNP	C	T	T	EMSY	c.616C>T	p.R206*
117J3239A1	11	108206593	108206593	Missense_Mutation	SNP	G	C	C	ATM	c.8173G>C	p.D2725H
117J3239A1	11	108213982	108213982	Nonsense_Mutation	SNP	G	T	T	ATM	c.8302G>T	p.E2768*
117J3239A1	11	118366470	118366470	Missense_Mutation	SNP	C	G	G	KMT2A	c.5419C>G	p.Q1807E
117J3239A1	12	133202857	133202857	Missense_Mutation	SNP	T	C	C	POLE	c.6377A>G	p.N2126S
117J3239A1	13	32913221	32913221	Missense_Mutation	SNP	G	C	C	BRCA2	c.4729G>C	p.E1577Q
117J3239A1	13	32913587	32913587	Missense_Mutation	SNP	G	A	A	BRCA2	c.5095G>A	p.D1699N
117J3239A1	15	91354500	91354500	Missense_Mutation	SNP	G	A	A	BLM	c.3940G>A	p.E1314K
117J3239A1	16	3790535	3790535	Missense_Mutation	SNP	C	G	G	CREBBP	c.3998G>C	p.R1333T
117J3239A1	17	7573991	7573991	Missense_Mutation	SNP	C	T	T	TP53	c.1036G>A	p.E346K
117J3239A1	17	37868177	37868177	Splice_Site	SNP	T	G	G	ERBB2	c.902-4T>G	-
117J3239A1	17	40481778	40481778	Missense_Mutation	SNP	C	T	T	STAT3	c.1126G>A	p.A376T
117J3239A1	17	59761343	59761343	Missense_Mutation	SNP	C	T	T	BRIP1	c.3064G>A	p.E1022K
117J3239A1	17	63554530	63554530	Missense_Mutation	SNP	G	A	A	AXIN2	c.209C>T	p.S70F
117J3239A1	17	76219630	76219630	Missense_Mutation	SNP	G	A	A	BIRC5	c.424G>A	p.D142N
117J3239A1		-	-	Frame_Shift_Del	INS	-	-	-	GATA6	c.1182del	p.S395Pfs*26
117J3239A1	18	60985744	60985744	Missense_Mutation	SNP	C	G	G	BCL2	c.156G>C	p.Q52H
117J3239A1	19	50910347	50910347	Missense_Mutation	SNP	G	C	C	POLD1	c.1602G>C	p.E534D
117J3239A1	2	141625284	141625284	Missense_Mutation	SNP	G	T	T	LRP1B	c.4454C>A	p.T1485K
117J3239A1	2	141747173	141747173	Missense_Mutation	SNP	G	T	T	LRP1B	c.2698C>A	p.P900T
117J3239A1	2	141771315	141771315	Splice_Site	SNP	C	A	A	LRP1B	c.2191-1G>T	-
117J3239A1	2	142004929	142004929	Splice_Site	SNP	A	G	G	LRP1B	c.464-6T>C	-
117J3239A1	2	170077050	170077050	Missense_Mutation	SNP	G	C	C	LRP2	c.5562C>G	p.I1854M

117J3239A1	2	178098945	178098945	Missense_Mutation	SNP	G	C	C	NFE2L2	c.100C>G	p.R34G
117J3239A1	22	29115403	29115403	Missense_Mutation	SNP	G	C	C	CHEK2	c.663C>G	p.I221M
117J3239A1	3	138665213	138665213	Missense_Mutation	SNP	C	T	T	FOXL2	c.352G>A	p.E118K
117J3239A1	3	142274791	142274791	Missense_Mutation	SNP	G	C	C	ATR	c.2269C>G	p.Q757E
117J3239A1	3	142277502	142277502	Missense_Mutation	SNP	G	T	T	ATR	c.1849C>A	p.L617I
117J3239A1	3	178941890	178941890	Missense_Mutation	SNP	G	A	A	PIK3CA	c.2209G>A	p.E737K
117J3239A1	3	185155396	185155396	Missense_Mutation	SNP	C	A	A	MAP3K13	c.637C>A	p.H213N
117J3239A1	3	185190815	185190815	Missense_Mutation	SNP	G	T	T	MAP3K13	c.1696G>T	p.A566S
117J3239A1	4	1977011	1977011	Splice_Site	SNP	C	T	T	NSD2	c.3515-10C>T	-
117J3239A1	4	126238326	126238326	Missense_Mutation	SNP	C	T	T	FAT4	c.760C>T	p.H254Y
117J3239A1	4	126370128	126370128	Missense_Mutation	SNP	G	A	A	FAT4	c.7957G>A	p.E2653K
117J3239A1	5	149437141	149437141	Missense_Mutation	SNP	G	A	A	CSF1R	c.2147C>T	p.S716F
117J3239A1	6	93967194	93967194	Missense_Mutation	SNP	C	G	G	EPHA7	c.2158G>C	p.D720H
117J3239A1	8	38314954	38314954	Missense_Mutation	SNP	C	G	G	FGFR1	c.11G>C	p.W4S
117J3239A1	8	48691327	48691327	Missense_Mutation	SNP				PRKDC	c.11612G>C	p.R3871T
117J3239A1	9	101900314	101900314	Missense_Mutation	SNP	C	G	G	TGFBR1	c.748C>G	p.Q250E
117J3282A1	11	108201043	108201043	Nonsense_Mutation	SNP	T	A	A	ATM	c.7410T>A	p.Y2470*
117J3282A1	19	1220434	1220434	Missense_Mutation	SNP	A	C	C	STK11	c.527A>C	p.D176A
117J3282A1	19	42777250	42777250	Missense_Mutation	SNP	G	A	A	CIC	c.1315G>A	p.E439K
117J3282A1	3	142272717	142272717	Missense_Mutation	SNP	T	C	C	ATR	c.2482A>G	p.I828V
117J3295A1	1	156834205	156834205	Missense_Mutation	SNP	G	T	T	NTRK1	c.272G>T	p.G91V
117J3295A1		-	-	Frame_Shift_Del	INS	-	-	-	PTEN	C.243del	P.F81Lfs*18
117J3306A1	1	27057874	27057874	Nonsense_Mutation	SNP	C	T	T	ARID1A	c.1582C>T	p.Q528*
117J3306A1	1	162729667	162729667	Nonsense_Mutation	SNP	G	A	A	DDR2	c.753G>A	p.W251*
117J3306A1	10	123263446	123263446	Missense_Mutation	SNP	C	T	T	FGFR2	c.1297G>A	p.E433K
117J3306A1		-	-	In_Frame_Ins	INS	-	-	-	FGFR2	168insAACTTCT1	389_C390insNFLIA
117J3306A1	17	7579373	7579373	Missense_Mutation	SNP	C	T	T	TP53	c.314G>A	p.G105D
117J3306A1	2	48033372	48033372	Missense_Mutation	SNP	G	A	A	MSH6	c.3676G>A	p.A1226T
117J3306A1	20	57429649	57429649	Missense_Mutation	SNP	C	T	T	GNAS	c.1142C>T	p.P381L
117J3306A1	20	57429659	57429659	Missense_Mutation	SNP	C	T	T	GNAS	c.1339C>T	p.P447S
117J3306A1	3	52442055	52442055	Missense_Mutation	SNP	G	C	C	BAP1	c.294C>G	p.S98R
117J3306A1		-	-	Frame_Shift_Ins	INS	-	-	-	FOXP1	c.1629_1630ins	p.R544Sfs*9
117J3318A1	12	46125084	46125084	Nonsense_Mutation	SNP	C	T	T	ARID2	c.271C>T	p.Q91*
117J3318A1	12	56492633	56492633	Missense_Mutation	SNP	A	G	G	ERBB3	c.2783A>G	p.E928G
117J3318A1	17	7578392	7578392	Missense_Mutation	SNP	C	T	T	TP53	c.538G>A	p.E180K
117J3318A1		-	-	Frame_Shift_Del	INS	-	-	-	SIK1	C.351del	P.N118Tfs*4
117J3318A1	3	41268766	41268766	Missense_Mutation	SNP	A	T	T	CTNNB1	c.1004A>T	p.K335I
117J3318A1	12	25398284	25398284	Missense_Mutation	SNP	C	A	A	KRAS	c.35G>T	p.G12V
117K3377A1	1	16258484	16258484	Missense_Mutation	SNP	C	T	T	SPEN	c.5749C>T	p.R1917C
117K3377A1		-	-	Frame_Shift_Del	INS	-	-	-	STK11	c.298del	p.Q100Nfs*3
117K3377A1	6	157517297	157517297	Splice_Site	SNP	A	G	G	ARID1B	c.3863-2A>G	-
117K3386A1		-	-	Frame_Shift_Ins	INS	-	-	-	EPHA2	c.1320dup	p.K441Qfs*13
117K3386A1	1	51436176	51436176	Splice_Site	SNP	A	G	G	CDKN2C	c.129+7A>G	-
117K3386A1	10	43597793	43597793	Missense_Mutation	SNP	G	A	A	RET	c.341G>A	p.R114H
117K3386A1		-	-	In_Frame_Del	INS	-	-	-	FGFR2	c.772_813del	p.L258_G271del
117K3386A1	19	33792353	33792353	Missense_Mutation	SNP	C	A	A	CEBPA	c.968G>T	p.R323L
117K3386A1	3	10183830	10183830	Missense_Mutation	SNP	C	T	T	VHL	c.299C>T	p.T100M
117K3386A1	4	55946257	55946257	Missense_Mutation	SNP	C	T	T	KDR	c.3922G>A	p.G1308R
117K3403A1	1	43805738	43805738	Missense_Mutation	SNP	T	A	A	MPL	c.794T>A	p.L265H
117K3403A1	1	65303679	65303679	Missense_Mutation	SNP	T	C	C	JAK1	c.3076A>G	p.K1026E
117K3403A1	1	98015244	98015244	Missense_Mutation	SNP	G	T	T	DPYD	c.1396C>A	p.P466T
117K3403A1	1	98205939	98205939	Splice_Site	SNP	T	A	A	DPYD	c.321+9A>T	-
117K3403A1		-	-	Frame_Shift_Ins	INS	-	-	-	ABL2	c.2518dup	p.S840Kfs*52
117K3403A1	1	193117007	193117007	Missense_Mutation	SNP	A	G	G	CDC73	c.740A>G	p.K247R
117K3403A1	1	193181580	193181580	Missense_Mutation	SNP	A	T	T	CDC73	c.1127A>T	p.N376I
117K3403A1	11	46321682	46321682	Missense_Mutation	SNP	T	A	A	CREB3L1	c.299T>A	p.L100H
117K3403A1	11	92495345	92495345	Splice_Site	SNP	T	A	A	FAT3	c.3984+9T>A	-
117K3403A1		-	-	Frame_Shift_Del	INS	-	-	-	KMT2D	c.6594del	p.Y2199Ifs*65
117K3403A1	14	50929435	50929435	Missense_Mutation	SNP	T	C	C	MAP4K5	c.884A>G	p.N295S
117K3403A1	17	7578291	7578291	Splice_Site	SNP	T	A	A	TP53	c.560-2A>T	-
117K3403A1	17	40485698	40485698	Missense_Mutation	SNP	T	C	C	STAT3	c.1042A>G	p.K348E
117K3403A1	19	15355518	15355518	Splice_Site	SNP	T	A	A	BRD4	c.2211+3A>T	-
117K3403A1	2	141474278	141474278	Missense_Mutation	SNP	C	G	G	LRP1B	c.5866G>C	p.E1956Q
117K3403A1	2	212248591	212248591	Missense_Mutation	SNP	T	A	A	ERBB4	c.3676A>T	p.I1226L
117K3403A1	2	212488781	212488781	Splice_Site	SNP	A	T	T	ERBB4	c.2080-12T>A	-
117K3403A1	20	31024668	31024668	Missense_Mutation	SNP	A	T	T	ASXL1	c.4153A>T	p.R1385W
117K3403A1	21	39817423	39817423	Missense_Mutation	SNP	A	T	T	ERG	c.161T>A	p.M54K
117K3403A1	3	10123082	10123082	Missense_Mutation	SNP	A	T	T	FANCD2	c.3158A>T	p.Q1053L
117K3403A1	3	195596414	195596414	Splice_Site	SNP	T	A	A	TNK2	c.1733-2A>T	-
117K3403A1	4	126371728	126371728	Missense_Mutation	SNP	A	T	T	FAT4	c.9557A>T	p.D3186V
117K3403A1	5	251451	251451	Splice_Site	SNP	A	T	T	SDHA	c.1664-2A>T	-
117K3403A1	6	157522010	157522010	Missense_Mutation	SNP	A	T	T	ARID1B	c.4282A>T	p.S1428C
117K3403A1	8	69046362	69046362	Missense_Mutation	SNP	T	A	A	PREX2	c.3835T>A	p.S1279T
117K3403A1	8	92972637	92972637	Missense_Mutation	SNP	T	A	A	RUNX1T1	c.1537A>T	p.I513F
117K3403A1	8	93115083	93115083	Splice_Site	SNP	A	T	T	RUNX1T1	c.28+2T>A	-
117K3403A1	X	48650475	48650475	Nonsense_Mutation	SNP	G	T	T	GATA1	c.445G>T	p.G149*
117K3508A1	17	7577534	7577534	Missense_Mutation	SNP	C	A	A	TP53	c.747G>T	p.R249S
117K3508A1	22	21348029	21348029	Missense_Mutation	SNP	T	C	C	LZTR1	c.1339T>C	p.F447L
117K3508A1	3	181430615	181430615	Missense_Mutation	SNP	G	A	A	SOX2	c.467G>A	p.R156H
117K3508A1	7	41729517	41729517	Missense_Mutation	SNP	A	T	T	INHBA	c.1012T>A	p.W338R
117K3508A1	9	21971145	21971145	Missense_Mutation	SNP	G	T	T	CDKN2A	c.213C>A	p.N71K
117K3520A1	12	25398284	25398284	Missense_Mutation	SNP	C	A	A	KRAS	c.35G>T	p.G12V
117K3520A1	18	19752072	19752072	Missense_Mutation	SNP	T	C	C	GATA6	c.967T>C	p.Y323H
117K3520A1	18	48591892	48591892	Missense_Mutation	SNP	G	A	A	SMAD4	c.1055G>A	p.G352E
117K3520A1	18	48593406	48593406	Missense_Mutation	SNP	G	A	A	SMAD4	c.1157G>A	p.G386D
117K3527A1	12	25398284	25398284	Missense_Mutation	SNP	C	T	T	KRAS	c.35G>A	p.G12D
117K3527A1	12	46233111	46233111	Splice_Site	SNP	G	A	A	ARID2	c.1331-1G>A	-
117K3527A1	13	28578283	28578283	Missense_Mutation	SNP	G	A	A	FLT3	c.2888C>T	p.S963L
117K3527A1	17	7577108	7577108	Missense_Mutation	SNP	C	T	T	TP53	c.830G>A	p.C277Y
117K3527A1	19	45918127	45918127	Missense_Mutation	SNP	C	T	T	ERCC1	c.694G>A	p.V232I
117K3527A1	22	41489103	41489103	Splice_Site	SNP	G	A	A	EP300	c.94+1G>A	-
117K3527A1	3	138664879	138664879	Missense_Mutation	SNP	G	A	A	FOXL2	c.686C>T	p.A229V
117K3527A1	7	106508826	106508826	Missense_Mutation	SNP	G	A	A	PIK3CG	c.820G>A	p.V274I
117K3556A1	10	131506271	131506271	Missense_Mutation	SNP	C	A	A	MGMT	c.331C>A	p.P111T
117K3556A1	11	108117798	108117798	Missense_Mutation	SNP	C	T	T	ATM	c.1009C>T	p.R337C
117K3556A1	2	121726436	121726436	Nonsense_Mutation	SNP	C	T	T	GLI2	c.790C>T	p.R264*
117K3556A1	3	178916725	178916725	Missense_Mutation	SNP	C	T	T	PIK3CA	c.112C>T	p.R38C
117K3556A1	5	138266623	138266623	Missense_Mutation	SNP	A	G	G	CTNNA1	c.2297A>G	p.H766R

117K3556A1	5	156668740	156668740	Splice_Site	SNP	A	G	G	ITK	c.1060+10A>G	-
117K3556A1	7	42004287	42004287	Missense_Mutation	SNP	T	G	G	GLI3	c.4384A>C	p.I1462L
117K3576A1	1	158619676	158619676	Missense_Mutation	SNP	C	T	T	SPTA1	c.3539G>A	p.G1180D
117K3576A1	17	29548950	29548950	Splice_Site	SNP	A	G	G	NF1	c.1721+3A>G	-
117K3576A1	3	49936071	49936071	Nonsense_Mutation	SNP	A	T	T	MST1R	c.1599T>A	p.C533*
117K3576A1	7	55087043	55087043	Missense_Mutation	SNP	C	G	G	EGFR	c.73C>G	p.L25V
117K3596A1	10	114919749	114919749	Missense_Mutation	SNP	C	G	G	TCF7L2	c.1371C>G	p.C457W
117K3596A1	12	25398285	25398285	Missense_Mutation	SNP	C	A	A	KRAS	c.34G>T	p.G12C
117K3596A1		-	-	Frame_Shift_Ins	INS	-	-	-	TP53	C.756dup	P.T253Hfs*11
117K3596A1	5	149515139	149515139	Missense_Mutation	SNP	G	A	A	PDGFRB	c.343C>T	p.R115W
117K3609A1	19	15378374	15378374	Splice_Site	SNP	G	A	A	BRD4	c.424-12C>T	-
117K3609A1	2	178098800	178098800	Missense_Mutation	SNP	T	C	C	NFE2L2	c.245A>G	p.E82G
117K3609A1	3	30691943	30691943	Missense_Mutation	SNP	T	A	A	TGFBR2	c.445T>A	p.F149I
117K3609A1	3	30732948	30732948	Missense_Mutation	SNP	T	C	C	TGFBR2	c.1561T>C	p.W521R
117K3609A1	3	52443761	52443761	Splice_Site	SNP	T	C	C	BAP1	c.38-2A>G	-
117K3620A1	1	27056352	27056352	Nonsense_Mutation	SNP	C	T	T	ARID1A	c.1348C>T	p.Q450*
117K3620A1		-	-	Frame_Shift_Ins	INS	-	-	-	ARID1A	c.6747dup	p.E2250Rfs*28
117K3620A1	16	68845758	68845758	Missense_Mutation	SNP	G	A	A	CDH1	c.1004G>A	p.R335Q
117K3620A1	2	178095644	178095644	Missense_Mutation	SNP	C	G	G	NFE2L2	c.1687G>C	p.E563Q
117K3620A1	22	38370170	38370170	Missense_Mutation	SNP	G	A	A	SOX10	c.733C>T	p.P245S
117K3620A1	7	41729816	41729816	Missense_Mutation	SNP	A	G	G	INHBA	c.713T>C	p.L238P
117K3620A1	8	139165017	139165017	Missense_Mutation	SNP	T	G	G	FAM135B	c.1701A>C	p.E567D
117K3660A1	12	56481922	56481922	Missense_Mutation	SNP	G	A	A	ERBB3	c.850G>A	p.G284R
117K3660A1	17	7578271	7578271	Missense_Mutation	SNP	T	C	C	TP53	c.578A>G	p.H193R
117K3660A1	18	48604690	48604690	Missense_Mutation	SNP	T	G	G	SMAD4	c.1512T>G	p.S504R
117K3669A1	1	27101099	27101099	Nonsense_Mutation	SNP	C	T	T	ARID1A	c.4381C>T	p.R1461*
117K3669A1		-	-	Frame_Shift_Del	INS	-	-	-	SPTA1	C.1349del	P.K450Rfs*43
117K3669A1	12	56495328	56495328	Missense_Mutation	SNP	G	A	A	ERBB3	c.3518G>A	p.R1173Q
117K3669A1	17	70118937	70118937	Missense_Mutation	SNP	C	T	T	SOX9	c.509C>T	p.P170L
117K3669A1	19	2216398	2216398	Missense_Mutation	SNP	G	A	A	DOT1L	c.2042G>A	p.R681H
117K3669A1	2	209113112	209113112	Missense_Mutation	SNP	C	A	A	IDH1	c.395G>T	p.R132L
117K3669A1	20	57429158	57429158	Missense_Mutation	SNP	G	A	A	GNAS	c.838G>A	p.A280T
117K3669A1	22	21350115	21350115	Missense_Mutation	SNP	G	A	A	LZTR1	c.2023G>A	p.G675R
117K3669A1	3	52439278	52439278	Nonsense_Mutation	SNP	G	A	A	BAP1	c.964C>T	p.Q322*
117K3669A1		-	-	Frame_Shift_Del	INS	-	-	-	PBRM1	C.3833del	P.P1278Lfs*10
117K3669A1	4	1806600	1806600	Missense_Mutation	SNP	G	A	A	FGFR3	c.1316G>A	p.R439H
117K3669A1	4	126412679	126412679	Missense_Mutation	SNP	G	C	C	FAT4	c.14702G>C	p.G4901A
117K3669A1		-	-	Frame_Shift_Del	INS	-	-	-	APC	C.4056_4072de	P.F1354Ifs*15
117K3669A1	5	131972883	131972883	Missense_Mutation	SNP	C	T	T	RAD50	c.3466C>T	p.R1156C
117K3669A1	5	147207669	147207669	Missense_Mutation	SNP	T	C	C	SPINK1	c.110A>G	p.N37S
117K3669A1	6	117683996	117683996	Missense_Mutation	SNP	T	C	C	ROS1	c.3151A>G	p.I1051V
117K3669A1	7	152346028	152346028	Missense_Mutation	SNP	T	C	C	XRCC2	c.542A>G	p.E181G
117K3669A1	X	48891040	48891040	Missense_Mutation	SNP	C	T	T	TFE3	c.1076G>A	p.R359Q
117K3688A1	10	123325192	123325192	Nonsense_Mutation	SNP	G	A	A	FGFR2	c.136C>T	p.Q46*
117K3688A1	12	25398284	25398284	Missense_Mutation	SNP	C	G	G	KRAS	c.35G>C	p.G12A
117K3688A1	12	46245033	46245033	Nonsense_Mutation	SNP	C	T	T	ARID2	c.3127C>T	p.Q1043*
117K3688A1	12	46245376	46245376	Nonsense_Mutation	SNP	C	G	G	ARID2	c.3470C>G	p.S1157*
117K3688A1	12	115112364	115112364	Missense_Mutation	SNP	C	T	T	TBX3	c.1376G>A	p.R459H
117K3688A1		-	-	Frame_Shift_Del	INS	-	-	-	FOS	c.894_895del	p.F298Lfs*51
117K3688A1	16	31191485	31191485	Splice_Site	SNP	C	G	G	FUS	c.-51C>G	-
117K3688A1		-	-	In_Frame_Del	INS	-	-	-	TP53	c.469_486del	p.V157_I162del
117K3688A1	17	56432344	56432344	Missense_Mutation	SNP	G	A	A	RNF43	c.2312C>T	p.S771L
117K3688A1	2	47656970	47656970	Missense_Mutation	SNP	G	A	A	MSH2	c.1166G>A	p.R389Q
117K3688A1	2	121745999	121745999	Missense_Mutation	SNP	G	A	A	GLI2	c.2509G>A	p.E837K
117K3688A1	3	41274855	41274855	Missense_Mutation	SNP	C	T	T	CTNNB1	c.1105C>T	p.H369Y
117K3688A1	3	178936082	178936082	Missense_Mutation	SNP	G	A	A	PIK3CA	c.1624G>A	p.E542K
117K3688A1	5	176618955	176618955	Missense_Mutation	SNP	G	C	C	NSD1	c.998G>C	p.R333T
117K3688A1	5	176638333	176638333	Missense_Mutation	SNP	C	T	T	NSD1	c.2933C>T	p.P978L
117K3688A1	7	92462399	92462399	Splice_Site	SNP	G	A	A	CDK6	c.233+6C>T	-
117K3688A1	7	106508682	106508682	Missense_Mutation	SNP	C	T	T	PIK3CG	c.676C>T	p.R226C
117K3688A1	8	48766767	48766767	Missense_Mutation	SNP				PRKDC	c.6769G>C	p.E2257Q
117K3699A1	12	25398285	25398285	Missense_Mutation	SNP	C	A	A	KRAS	c.34G>T	p.G12C
117K3699A1		-	-	Frame_Shift_Ins	INS	-	-	-	SOX9	C.1074_1075dup	P.P359Rfs*25
117K3699A1	20	57484420	57484420	Missense_Mutation	SNP	C	T	T	GNAS	c.601C>T	p.R201C
117K3699A1	3	49397742	49397742	Missense_Mutation	SNP	G	A	A	RHOA	c.482C>T	p.A161V
117K3699A1		-	-	Frame_Shift_Ins	INS	-	-	-	APC	C.4666dup	P.T1556Nfs*3
117K3699A1	9	101907038	101907038	Missense_Mutation	SNP	A	T	T	TGFBR1	c.998A>T	p.D333V
117K3728A1	1	27059236	27059236	Nonsense_Mutation	SNP	C	T	T	ARID1A	c.1873C>T	p.Q625*
117K3728A1	12	25398284	25398284	Missense_Mutation	SNP	C	T	T	KRAS	c.35G>A	p.G12D
117K3728A1		-	-	Frame_Shift_Del	INS	-	-	-	TP53	c.1179_1180del	p.D393Efs*77
117K3728A1	19	11170486	11170486	Missense_Mutation	SNP	G	A	A	SMARCA4	c.4789G>A	p.E1597K
117K3728A1	7	78150951	78150951	Missense_Mutation	SNP	C	T	T	MAGI2	c.550G>A	p.G184S
117K3750A1	1	2491310	2491310	Missense_Mutation	SNP	T	C	C	TNFRSF14	c.353T>C	p.V118A
117K3750A1	1	9782576	9782576	Splice_Site	SNP	G	A	A	PIK3CD	c.2348-10G>A	-
117K3750A1	1	16257510	16257510	Missense_Mutation	SNP	G	A	A	SPEN	c.4775G>A	p.R1592Q
117K3750A1	1	65301152	65301152	Missense_Mutation	SNP	A	G	G	JAK1	c.3296T>C	p.M1099T
117K3750A1	1	65309758	65309758	Missense_Mutation	SNP	T	C	C	JAK1	c.2392A>G	p.T798A
117K3750A1	1	162740216	162740216	Missense_Mutation	SNP	G	A	A	DDR2	c.1418G>A	p.R473H
117K3750A1	1	179095717	179095717	Missense_Mutation	SNP	T	A	A	ABL2	c.482A>T	p.Y161F
117K3750A1	1	204438447	204438447	Nonsense_Mutation	SNP	G	A	A	PIK3C2B	c.484C>T	p.R162*
117K3750A1	1	226590082	226590082	Splice_Site	SNP	T	C	C	PARP1	c.121-2A>G	-
117K3750A1		-	-	In_Frame_Del	INS	-	-	-	TCF7L2	c.80_82del	p.27_28del
117K3750A1	10	123298201	123298201	Missense_Mutation	SNP	A	T	T	FGFR2	c.653T>A	p.M218K
117K3750A1		-	-	Missense_Mutation	SNP	-	-	-	NUP98	C.1585_1587de	P.K529del
117K3750A1	11	118390416	118390416	Nonsense_Mutation	SNP	C	T	T	KMT2A	c.11230C>T	p.R3744*
117K3750A1		-	-	Splice_Site	SNP	-	-	-	ARID2	c.419-10dupT	-
117K3750A1	12	49433223	49433223	Missense_Mutation	SNP	T	C	C	KMT2D	c.8224A>G	p.T2742A
117K3750A1		-	-	Frame_Shift_Del	INS	-	-	-	KMT2D	C.7061del	P.P2354Lfs*30
117K3750A1	12	50483664	50483664	Splice_Site	SNP	T	C	C	SMARCD1	c.772-3T>C	-
117K3750A1	12	50484355	50484355	Missense_Mutation	SNP	T	A	A	SMARCD1	c.1115T>A	p.I372N
117K3750A1	12	57560824	57560824	Missense_Mutation	SNP	C	T	T	LRP1	c.2909C>T	p.S970L
117K3750A1	12	57595294	57595294	Missense_Mutation	SNP	G	A	A	LRP1	c.10360G>A	p.A3454T
117K3750A1	12	115109887	115109887	Missense_Mutation	SNP	G	A	A	TBX3	c.1991C>T	p.A664V
117K3750A1	13	102568842	102568842	Missense_Mutation	SNP	G	A	A	FGF14	c.154C>T	p.R52C
117K3750A1	14	45620619	45620619	Missense_Mutation	SNP	G	A	A	FANCM	c.938G>A	p.R313H
117K3750A1	16	3790400	3790400	Missense_Mutation	SNP	C	T	T	CREBBP	c.4133G>A	p.R1378Q
117K3750A1	16	9862740	9862740	Missense_Mutation	SNP	C	T	T	GRIN2A	c.2563G>A	p.D855N
117K3750A1		-	-	In_Frame_Del	INS	-	-	-	THAP11	C.330_338del	P.Q130_Q132del

117K3750A1	16	81969962	81969962	Missense_Mutation	SNP	G	A	A	PLCG2	c.3031G>A	p.A1011T
117K3750A1	17	7578406	7578406	Missense_Mutation	SNP	C	T	T	TP53	c.524G>A	p.R175H
117K3750A1	17	11984788	11984788	Missense_Mutation	SNP	G	T	T	MAP2K4	c.334G>T	p.A112S
117K3750A1	17	11998928	11998928	Nonsense_Mutation	SNP	C	T	T	MAP2K4	c.430C>T	p.Q144*
117K3750A1		-	-	Frame_Shift_Del	INS	-	-	-	RNF43	C.1976del	P.G659Vfs*41
117K3750A1		-	-	Frame_Shift_Del	INS	-	-	-	BRIP1	C.2990_2993de	P.T997Rfs*61
117K3750A1		-	-	Frame_Shift_Del	INS	-	-	-	ROCK1	C.3921del	P.K1307Nfs*3
117K3750A1	19	42383667	42383667	Missense_Mutation	SNP	G	A	A	CD79A	c.442G>A	p.E148K
117K3750A1	19	45525373	45525373	Missense_Mutation	SNP				RELB	c.563C>A	p.P188H
117K3750A1		-	-	Splice_Site	SNP	-	-	-	RGPD4	c.73-3delT	-
117K3750A1		-	-	Missense_Mutation	SNP	-	-	-	RANBP2	C.6090_6092de	P.E2030del
117K3750A1	2	121747898	121747898	Missense_Mutation	SNP	C	T	T	GLI2	c.4408C>T	p.R1470W
117K3750A1	2	141274532	141274532	Missense_Mutation	SNP	C	A	A	LRP1B	c.8075G>T	p.G2692V
117K3750A1		-	-	Frame_Shift_Del	INS	-	-	-	ACVR2A	C.1310del	P.K437Rfs*5
117K3750A1		-	-	Frame_Shift_Del	INS	-	-	-	CASP8	C.1326_1327de	P.C443Pfs*12
117K3750A1	2	215633992	215633992	Missense_Mutation	SNP	A	T	T	BARD1	c.1359T>A	p.D453E
117K3750A1	20	30661518	30661518	Splice_Site	SNP	G	A	A	HCK	c.164-1G>A	-
117K3750A1	20	57429088	57429088	Missense_Mutation	SNP	G	A	A	GNAS	c.581G>A	p.R194Q
117K3750A1	21	39755530	39755530	Missense_Mutation	SNP	G	A	A	ERG	c.1184C>T	p.P395L
117K3750A1		-	-	Frame_Shift_Del	INS	-	-	-	EWSR1	C.1616_1617de	P.E539Vfs*5
117K3750A1	22	41566575	41566575	Missense_Mutation	SNP	G	T	T	EP300	c.4452G>T	p.K1484N
117K3750A1		-	-	Frame_Shift_Del	INS	-	-	-	TGFBR2	C.382_383del	P.K128Afs*3
117K3750A1	3	71021235	71021235	Missense_Mutation	SNP	G	A	A	FOXP1	c.1627C>T	p.R543C
117K3750A1		-	-	Frame_Shift_Ins	INS	-	-	-	PRKCI	C.79dup	P.R27Pfs*16
117K3750A1		-	-	Missense_Mutation	SNP	-	-	-	TNK2	C.920_922del	P.F307del
117K3750A1	4	1807638	1807638	Missense_Mutation	SNP	C	T	T	FGFR3	c.1807C>T	p.R603W
117K3750A1	4	106155916	106155916	Nonsense_Mutation	SNP	C	T	T	TET2	c.817C>T	p.Q273*
117K3750A1		-	-	Frame_Shift_Ins	INS	-	-	-	FAT4	C.2738dup	P.Q914Pfs*8
117K3750A1		-	-	Frame_Shift_Del	INS	-	-	-	FAT1	C.1607_1608de	P.L536Qfs*25
117K3750A1		-	-	Missense_Mutation	SNP	-	-	-	MAP3K1	C.3767_3769de	P.S1256del
117K3750A1	5	149503808	149503808	Splice_Site	SNP	G	A	A	PDGFRB	c.2023+5C>T	-
117K3750A1	5	149512334	149512334	Missense_Mutation	SNP	G	A	A	PDGFRB	c.1106C>T	p.T369M
117K3750A1	6	30863256	30863256	Missense_Mutation	SNP	C	A	A	DDR1	c.1589C>A	p.P530H
117K3750A1	6	32166736	32166736	Missense_Mutation	SNP	C	T	T	NOTCH4	c.4502G>A	p.R1501Q
117K3750A1	6	36651948	36651948	Missense_Mutation	SNP	C	A	A	CDKN1A	c.70C>A	p.P24T
117K3750A1	6	138192644	138192644	Missense_Mutation	SNP	G	A	A	TNFAIP3	c.280G>A	p.A94T
117K3750A1	7	86415839	86415839	Missense_Mutation	SNP	C	T	T	GRM3	c.731C>T	p.A244V
117K3750A1		-	-	Splice_Site	SNP	-	-	-	BRAF	c.1993-11delT	-
117K3750A1	7	151845112	151845112	Splice_Site	SNP	C	T	T	KMT2C	c.13894+6G>A	-
117K3750A1	8	11400849	11400849	Missense_Mutation	SNP	C	T	T	BLK	c.116C>T	p.P39L
117K3750A1	8	48843298	48843298	Missense_Mutation	SNP	G	A	A	PRKDC	c.1826C>T	p.A609V
117K3750A1	9	98231242	98231242	Missense_Mutation	SNP	G	A	A	PTCH1	c.2041C>T	p.P681S
117K3750A1		-	-	Splice_Site	SNP	-	-	-	TGFBR1	c.1131-3delT	-
117K3750A1		-	-	Frame_Shift_Ins	INS	-	-	-	NR4A3	C.449dup	P.Q151Afs*89
117K3750A1		-	-	Splice_Site	SNP	-	-	-	KDM6A	737-7_3737-6de	-
117K3750A1	X	47038783	47038783	Nonsense_Mutation	SNP	G	T	T	RBM10	c.790G>T	p.E264*
117K3750A1		-	-	Frame_Shift_Del	INS	-	-	-	BCORL1	C.4451del	P.P1484Qfs*7
117L3780A1		-	-	Frame_Shift_Del	INS	-	-	-	ARID1A	c.6744_6747del	p.H2248Qfs*18
117L3780A1	12	25398284	25398284	Missense_Mutation	SNP	C	A	A	KRAS	c.35G>T	p.G12V
117L3780A1	18	48593387	48593387	Splice_Site	SNP	A	T	T	SMAD4	c.1140-2A>T	-
117L3815A1	1	156843595	156843595	Missense_Mutation	SNP	G	A	A	NTRK1	c.1021G>A	p.V341M
117L3815A1	13	41134628	41134628	Missense_Mutation	SNP	C	T	T	FOXO1	c.1000G>A	p.E334K
117L3815A1	17	7576855	7576855	Nonsense_Mutation	SNP	G	A	A	TP53	c.991C>T	p.Q331*
117L3815A1	17	41203134	41203134	Missense_Mutation	SNP	T	C	C	BRCA1	c.5278A>G	p.I1760V
117L3815A1	18	19751791	19751791	Missense_Mutation	SNP	C	G	G	GATA6	c.686C>G	p.S229W
117L3815A1	2	141200065	141200065	Splice_Site	SNP	A	G	G	LRP1B	c.10414+8T>C	-
117L3815A1	3	3215926	3215926	Missense_Mutation	SNP	T	C	C	CRBN	c.194A>G	p.E65G
117L3815A1	4	1906094	1906094	Missense_Mutation	SNP	C	A	A	NSD2	c.749C>A	p.T250N
117L3815A1	7	13975403	13975403	Missense_Mutation	SNP	C	T	T	ETV1	c.484G>A	p.D162N
117L3815A1	7	106522569	106522569	Missense_Mutation	SNP	G	A	A	PIK3CG	c.2546G>A	p.R849Q
117L3816A1	11	108200961	108200961	Missense_Mutation	SNP	G	A	A	ATM	c.7328G>A	p.R2443Q
117L3816A1	12	46231410	46231410	Nonsense_Mutation	SNP	C	G	G	ARID2	c.1250C>G	p.S417*
117L3816A1		-	-	Frame_Shift_Del	INS	-	-	-	TP53	.383_387delins	P.P128Lfs*41
117L3816A1	18	19752152	19752152	Missense_Mutation	SNP	G	T	T	GATA6	c.1047G>T	p.W349C
117L3816A1	19	11169494	11169494	Missense_Mutation	SNP	C	A	A	SMARCA4	c.4660C>A	p.L1554I
117L3816A1	20	36030892	36030892	Missense_Mutation	SNP	C	T	T	SRC	c.1171C>T	p.R391C
117L3816A1	4	187584566	187584566	Missense_Mutation	SNP	A	G	G	FAT1	c.3467T>C	p.V1156A
117L3816A1	8	93004079	93004079	Missense_Mutation	SNP	G	A	A	RUNX1T1	c.668C>T	p.S223L
117L3816A1	X	47429308	47429308	Missense_Mutation	SNP	G	A	A	ARAF	c.1436G>A	p.R479H
117L3817A1	11	108224492	108224492	Splice_Site	SNP	G	C	C	ATM	c.8672-1G>C	-
117L3817A1	13	43155275	43155275	Missense_Mutation	SNP	G	A	A	TNFSF11	c.14G>A	p.R5K
117L3817A1	16	2130195	2130195	Missense_Mutation	SNP	G	C	C	TSC2	c.3427G>C	p.D1143H
117L3817A1	19	11144798	11144798	Splice_Site	SNP	G	T	T	SMARCA4	c.3874-1G>T	-
117L3817A1	2	212576772	212576772	Splice_Site	SNP	G	A	A	ERBB4	c.1124+3C>T	-
117L3817A1	3	41266113	41266113	Missense_Mutation	SNP	C	T	T	CTNNB1	c.110C>T	p.S37F
117L3817A1	3	89391031	89391031	Missense_Mutation	SNP	G	T	T	EPHA3	c.1097G>T	p.C366F
117L3817A1	4	187541076	187541076	Missense_Mutation	SNP	C	T	T	FAT1	c.6664G>A	p.D2222N
117L3817A1	7	77764356	77764356	Missense_Mutation	SNP	G	A	A	MAGI2	c.3013C>T	p.R1005C
117L3842A1	11	119149250	119149250	Nonsense_Mutation	SNP	C	T	T	CBL	c.1258C>T	p.R420*
117L3842A1		-	-	In_Frame_Del	INS	-	-	-	TP53	c.512_520del	E171_R174delinsG
117L3842A1	4	187557957	187557957	Missense_Mutation	SNP	T	C	C	FAT1	c.3754A>G	p.I1252V
117L3851A1	1	27105892	27105892	Missense_Mutation	SNP	C	A	A	ARID1A	c.5503C>A	p.Q1835K
117L3851A1	1	120459238	120459238	Missense_Mutation	SNP	C	T	T	NOTCH2	c.6107G>A	p.R2036Q
117L3851A1	12	25398284	25398284	Missense_Mutation	SNP	C	A	A	KRAS	c.35G>T	p.G12V
117L3851A1	16	3778618	3778618	Missense_Mutation	SNP	C	A	A	CREBBP	c.6430G>T	p.A2144S
117L3851A1	17	41245489	41245489	Missense_Mutation	SNP	G	T	T	BRCA1	c.2059C>A	p.Q687K
117L3851A1	17	78921158	78921158	Splice_Site	SNP	G	A	A	RPTOR	c.3265+7G>A	-
117L3851A1	19	10610154	10610154	Missense_Mutation	SNP	C	A	A	KEAP1	c.556G>T	p.G186C
117L3851A1	2	198266834	198266834	Missense_Mutation	SNP	T	C	C	SF3B1	c.2098A>G	p.K700E
117L3851A1	8	71053502	71053502	Missense_Mutation	SNP	C	T	T	NCOA2	c.2945G>A	p.R982Q
117L3938A1	10	43606743	43606743	Missense_Mutation	SNP	C	T	T	RET	c.1352C>T	p.T451M
117L3938A1		-	-	Frame_Shift_Del	INS	-	-	-	TP53	c.626_627del	p.R209Kfs*6
117L3938A1	7	117282657	117282657	Splice_Site	SNP	A	G	G	CFTR	c.3873+10A>G	-
117L3940A1	12	25398284	25398284	Missense_Mutation	SNP	C	A	A	KRAS	c.35G>T	p.G12V
117L3940A1	12	50479995	50479995	Nonsense_Mutation	SNP	G	T	T	SMARCD1	c.229G>T	p.G77*
117L3940A1	17	62009553	62009553	Splice_Site	SNP	A	G	G	CD79B	c.67+2T>C	-
117L3940A1	3	178936082	178936082	Missense_Mutation	SNP	G	A	A	PIK3CA	c.1624G>A	p.E542K
117L3940A1	4	126367653	126367653	Nonsense_Mutation	SNP	C	T	T	FAT4	c.7399C>T	p.Q2467*

117L3952A1	1	65303760	65303760	Missense_Mutation	SNP	A	G	G	JAK1	c.2995T>C	p.Y999H
117L3952A1	1	156843554	156843554	Missense_Mutation	SNP	T	C	C	NTRK1	c.980T>C	p.F327S
117L3952A1	10	70405595	70405595	Missense_Mutation	SNP	C	A	A	TET1	c.3109C>A	p.Q1037K
117L3952A1		-	-	In_Frame_Del	INS	-	-	-	PARP4	333delinsGAAT	40_I445delinsNLVF
117L3952A1		-	-	Frame_Shift_Ins	INS	-	-	-	BRCA2	C.4169dup	P.L1390Ffs*13
117L3952A1	19	42778531	42778531	Missense_Mutation	SNP	C	A	A	CIC	c.2596C>A	p.P866T
117L3952A1		-	-	In_Frame_Del	INS	-	-	-	ROS1	C.5341_5362de	P.I1781*
117L3952A1	6	117710734	117710734	Missense_Mutation	SNP	T	C	C	ROS1	c.1538A>G	p.D513G
117L3964A1	12	25398284	25398284	Missense_Mutation	SNP	C	A	A	KRAS	c.35G>T	p.G12V
117L3964A1	12	49420739	49420739	Missense_Mutation	SNP	C	T	T	KMT2D	c.15010G>A	p.E5004K
117L3965A1		-	-	Frame_Shift_Del	INS	-	-	-	ARID1A	C.5038_5042de	P.L1680Gfs*16
117L3965A1	12	46244247	46244247	Nonsense_Mutation	SNP	G	T	T	ARID2	c.2341G>T	p.G781*
117L3965A1	13	32930610	32930610	Missense_Mutation	SNP	G	A	A	BRCA2	c.7481G>A	p.R2494Q
117L3965A1		-	-	Frame_Shift_Ins	INS	-	-	-	PBRM1	C.4198dup	.M1400Nfs*109
117L3965A1	5	176518789	176518789	Missense_Mutation	SNP	A	T	T	FGFR4	c.707A>T	p.N236I
117L3965A1	6	162206852	162206852	Missense_Mutation	SNP	G	A	A	PRKN	c.823C>T	p.R275W
117L4005A1		-	-	Frame_Shift_Ins	INS	-	-	-	ARID1A	c.4933dup	p.S1645Ffs*3
117L4005A1		-	-	Frame_Shift_Del	INS	-	-	-	CHD4	c.4376_4377del	p.Y1459Cfs*10
117L4005A1	17	48275831	48275831	Missense_Mutation	SNP	T	G	G	COL1A1	c.506A>C	p.E169A
117L4040A1	13	41134892	41134892	Missense_Mutation	SNP	T	C	C	FOXO1	c.736A>G	p.S246G
117L4040A1	15	88680700	88680700	Missense_Mutation	SNP	T	G	G	NTRK3	c.557A>C	p.N186T
117L4040A1	17	7577507	7577507	Missense_Mutation	SNP	T	G	G	TP53	c.774A>C	p.E258D
117L4040A1		-	-	In_Frame_Del	INS	-	-	-	NF1	C.5300_5314de	A1767_K1771del
117L4040A1		-	-	Frame_Shift_Del	INS	-	-	-	BRCA1	C.4485del	P.R1495Sfs*10
117L4040A1	6	32187569	32187569	Splice_Site	SNP	A	G	G	NOTCH4	c.1316-6T>C	-
117L4040A1	8	37688272	37688272	Missense_Mutation	SNP	C	T	T	ADGRA2	c.763C>T	p.R255C
117L4049A1	1	16458871	16458871	Splice_Site	SNP	A	G	G	EPHA2	c.2115+2T>C	-
117L4049A1		-	-	Frame_Shift_Ins	INS	-	-	-	PBRM1	810_1811insAA	p.G604Efs*7
117L4049A1	7	42188023	42188023	Missense_Mutation	SNP	C	T	T	GLI3	c.169G>A	p.A57T
117L4084A1	12	25398284	25398284	Missense_Mutation	SNP	C	T	T	KRAS	c.35G>A	p.G12D
117L4084A1		-	-	Frame_Shift_Del	INS	-	-	-	PBRM1	c.1284_1285del	p.S429Tfs*14
117L4084A1	6	30862458	30862458	Splice_Site	SNP	G	A	A	DDR1	c.1513+10G>A	-
117L4084A1	7	13975478	13975478	Missense_Mutation	SNP	G	T	T	ETV1	c.409C>A	p.P137T
117L4084A1	8	93017416	93017416	Missense_Mutation	SNP	G	T	T	RUNX1T1	c.557C>A	p.T186N
117L4084A1		-	-	Frame_Shift_Del	INS	-	-	-	AMER1	353_1362delins	p.E452Hfs*5
117L4087A1		-	-	Frame_Shift_Del	INS	-	-	-	MEN1	c.249_252del	p.I85Sfs*33
117L4087A1	17	7576857	7576857	Missense_Mutation	SNP	A	G	G	TP53	c.989T>C	p.L330P
117L4087A1	3	49927438	49927438	Missense_Mutation	SNP	C	T	T	MST1R	c.3866G>A	p.R1289H
117L4087A1	4	126239421	126239421	Missense_Mutation	SNP	C	T	T	FAT4	c.1855C>T	p.R619C
117L4087A1	7	18767243	18767243	Missense_Mutation	SNP	G	C	C	HDAC9	c.1763G>C	p.R588P
117L4089A1		-	-	Frame_Shift_Ins	INS	-	-	-	ARID1A	C.6341dup	P.Q2115Afs*35
117L4089A1	10	114925329	114925329	Missense_Mutation	SNP	C	T	T	TCF7L2	c.1163C>T	p.A388V
117L4089A1	12	56478786	56478786	Missense_Mutation	SNP	G	A	A	ERBB3	c.242G>A	p.R81Q
117L4089A1	13	28588663	28588663	Missense_Mutation	SNP	C	G	G	FLT3	c.2785G>C	p.D929H
117L4089A1	13	102568833	102568833	Missense_Mutation	SNP	C	T	T	FGF14	c.163G>A	p.G55S
117L4089A1		-	-	Frame_Shift_Del	INS	-	-	-	AXIN1	C.1523del	P.G508Vfs*197
117L4089A1	16	3788627	3788627	Missense_Mutation	SNP	G	A	A	CREBBP	c.4327C>T	p.R1443C
117L4089A1	17	7577548	7577548	Missense_Mutation	SNP	C	A	A	TP53	c.733G>T	p.G245C
117L4089A1	17	12011119	12011119	Missense_Mutation	SNP	A	T	T	MAP2K4	c.526A>T	p.I176F
117L4089A1		-	-	Splice_Site	SNP	-	-	-	COL1A1	c.1300-7delC	-
117L4089A1	17	48272920	48272920	Splice_Site	SNP	A	G	G	COL1A1	c.1155+8T>C	-
117L4089A1		-	-	Frame_Shift_Del	INS	-	-	-	AXIN2	C.1994del	P.G665Afs*24
117L4089A1		-	-	In_Frame_Del	INS	-	-	-	SMAD4	C.94_95del	P.S32*
117L4089A1		-	-	Frame_Shift_Del	INS	-	-	-	SMAD4	C.158_162del	P.E53Gfs*15
117L4089A1		-	-	In_Frame_Del	INS	-	-	-	SMAD4	C.1313_1314de	P.F438*
117L4089A1	19	15285059	15285059	Missense_Mutation	SNP	A	G	G	NOTCH3	c.4556T>C	p.L1519P
117L4089A1	19	17945927	17945927	Missense_Mutation	SNP	T	G	G	JAK3	c.2012A>C	p.D671A
117L4089A1	2	47637413	47637413	Nonsense_Mutation	SNP	C	T	T	MSH2	c.547C>T	p.Q183*
117L4089A1	2	191895741	191895741	Missense_Mutation	SNP	G	A	A	STAT4	c.2177C>T	p.A726V
117L4089A1	2	212426705	212426705	Missense_Mutation	SNP	G	T	T	ERBB4	c.2410C>A	p.L804M
117L4089A1	3	52437639	52437639	Missense_Mutation	SNP	G	A	A	BAP1	c.1522C>T	p.R508C
117L4089A1	3	181430166	181430166	Missense_Mutation	SNP	G	T	T	SOX2	c.18G>T	p.E6D
117L4089A1	3	183211878	183211878	Missense_Mutation	SNP	A	G	G	KLHL6	c.1339T>C	p.C447R
117L4089A1	5	180046110	180046110	Splice_Site	SNP	C	A	A	FLT4	c.2762-1G>T	-
117L4089A1	7	41730086	41730086	Missense_Mutation	SNP	G	A	A	INHBA	c.443C>T	p.S148L
117L4089A1	7	81358991	81358991	Missense_Mutation	SNP	T	C	C	HGF	c.970A>G	p.I324V
117L4089A1	7	151873888	151873888	Nonsense_Mutation	SNP	G	A	A	KMT2C	c.8650C>T	p.R2884*
117L4089A1	8	139323164	139323164	Splice_Site	SNP	C	A	A	FAM135B	c.78-1G>T	-
117L4089A1	9	21971120	21971120	Nonsense_Mutation	SNP	G	A	A	CDKN2A	c.238C>T	p.R80*
117L4089A1	9	133760147	133760147	Missense_Mutation	SNP	G	A	A	ABL1	c.2527G>A	p.V843M
117L4089A1	9	135776154	135776154	Missense_Mutation	SNP	A	G	G	TSC1	c.2573T>C	p.V858A
117L4089A1		-	-	Frame_Shift_Ins	INS	-	-	-	KDM6A	C.1271dup	P.L425Tfs*7
117L4089A1	X	44966679	44966679	Missense_Mutation	SNP	G	T	T	KDM6A	c.3903G>T	p.K1301N
117L4089A1	X	53224583	53224583	Missense_Mutation	SNP	G	A	A	KDM5C	c.3130C>T	p.H1044Y
117L4089A1	X	53245002	53245002	Missense_Mutation	SNP	C	T	T	KDM5C	c.938G>A	p.R313Q
117L4100A1	3	52441213	52441213	Missense_Mutation	SNP	A	C	C	BAP1	c.557T>G	p.L186R
117L4100A1	5	180057710	180057710	Missense_Mutation	SNP	C	T	T	FLT4	c.245G>A	p.R82Q
117L4103A1		-	-	Frame_Shift_Ins	INS	-	-	-	EPHA2	C.1000dup	P.Y334Lfs*47
117L4103A1	1	27105553	27105553	Nonsense_Mutation	SNP	C	T	T	ARID1A	c.5164C>T	p.R1722*
117L4103A1	12	25398284	25398284	Missense_Mutation	SNP	C	T	T	KRAS	c.35G>A	p.G12D
117L4103A1	3	52598207	52598207	Missense_Mutation	SNP	C	T	T	PBRM1	c.3659G>A	p.R1220K
117L4103A1	3	178936091	178936091	Missense_Mutation	SNP	G	A	A	PIK3CA	c.1633G>A	p.E545K
117L4103A1	5	156649874	156649874	Missense_Mutation	SNP	G	A	A	ITK	c.497G>A	p.R166Q
117L4103A1	7	151960190	151960190	Missense_Mutation	SNP	G	C	C	KMT2C	c.1210C>G	p.L404V
117L4103A1	8	11400840	11400840	Missense_Mutation	SNP	C	T	T	BLK	c.107C>T	p.P36L
117L4125A1		-	-	Frame_Shift_Del	INS	-	-	-	EPHA2	c.921del	p.C307Wfs*86
117L4125A1	12	69229720	69229720	Missense_Mutation	SNP	A	T	T	MDM2	c.796A>T	p.S266C
117L4125A1	2	209113112	209113112	Missense_Mutation	SNP	C	A	A	IDH1	c.395G>T	p.R132L
117L4126A1	1	204515977	204515977	Missense_Mutation	SNP	A	T	T	MDM4	c.875A>T	p.D292V
117L4126A1	1	226253379	226253379	Missense_Mutation	SNP	G	C	C	H3F3A	c.151G>C	p.E51Q
117L4126A1	11	69631202	69631202	Splice_Site	SNP	T	A	A	FGF3	c.221-11A>T	-
117L4126A1	11	76261137	76261137	Missense_Mutation	SNP	A	C	C	EMSY	c.3916A>C	p.T1306P
117L4126A1		-	-	Frame_Shift_Del	INS	-	-	-	POLE	C.4090del	P.R1364Vfs*5
117L4126A1		-	-	Splice_Site	SNP	-	-	-	TP53	c.373_375+9del	-
117L4126A1	19	15281220	15281220	Missense_Mutation	SNP	C	A	A	NOTCH3	c.5036G>T	p.G1679V
117L4126A1	2	121745934	121745934	Missense_Mutation	SNP	C	T	T	GLI2	c.2444C>T	p.S815L
117L4126A1	4	153249384	153249384	Missense_Mutation	SNP	C	A	A	FBXW7	c.1394G>T	p.R465L
117L4126A1	7	117232062	117232062	Missense_Mutation	SNP	A	T	T	CFTR	c.1841A>T	p.D614V

117L4126A1	9	87636352	87636352	Nonsense_Mutation	SNP	G	T	T	NTRK2	c.2517G>T	p.*839Y
117L4133A1	11	94800480	94800480	Missense_Mutation	SNP	G	C	C	SRSF8	c.90G>C	p.L30F
117L4133A1	17	7577082	7577082	Missense_Mutation	SNP	C	T	T	TP53	c.856G>A	p.E286K
117L4133A1	18	48604655	48604655	Missense_Mutation	SNP	G	A	A	SMAD4	c.1477G>A	p.D493N
117L4133A1	6	36651918	36651918	Missense_Mutation	SNP	G	A	A	CDKN1A	c.40G>A	p.G14S
117L4133A1	7	142649593	142649593	Splice_Site	SNP	C	T	T	KEL	c.1203+3G>A	-
117L4139A1	10	8100573	8100573	Missense_Mutation	SNP	T	A	A	GATA3	c.547T>A	p.C183S
117L4139A1	10	43595967	43595967	Missense_Mutation	SNP	C	T	T	RET	c.134C>T	p.A45V
117L4139A1	11	108115753	108115753	Missense_Mutation	SNP	G	C	C	ATM	c.901G>C	p.G301R
117L4139A1	11	108163495	108163495	Missense_Mutation	SNP	A	T	T	ATM	c.4586A>T	p.Y1529F
117L4139A1		-	-	Splice_Site	SNP	-	-	-	RB1	1390-38_1396d	-
117L4139A1	17	7576927	7576927	Splice_Site	SNP	C	A	A	TP53	c.920-1G>T	-
117L4139A1		-	-	Frame_Shift_Del	INS	-	-	-	LZTR1	c.2312del	p.Q771Rfs*18
117L4139A1	3	185797820	185797820	Missense_Mutation	SNP	G	C	C	ETV5	c.436C>G	p.Q146E
117L4139A1	7	18767372	18767372	Missense_Mutation	SNP	C	A	A	HDAC9	c.1892C>A	p.S631Y
118A4239A1	16	3900929	3900929	Missense_Mutation	SNP	C	G	G	CREBBP	c.167G>C	p.S56T
118A4239A1	17	7577538	7577538	Missense_Mutation	SNP	C	T	T	TP53	c.743G>A	p.R248Q
118A4239A1	17	56435317	56435317	Missense_Mutation	SNP	G	A	A	RNF43	c.1820C>T	p.S607L
118A4239A1	18	48603147	48603147	Splice_Site	SNP	G	A	A	SMAD4	c.1447+1G>A	-
118A4239A1	4	1962801	1962801	Missense_Mutation	SNP	G	A	A	NSD2	c.3295G>A	p.E1099K
118A4239A1	5	176563022	176563022	Missense_Mutation	SNP	A	T	T	NSD1	c.918A>T	p.E306D
118A4277A1	11	118376686	118376686	Nonsense_Mutation	SNP	C	A	A	KMT2A	c.10079C>A	p.S3360*
118A4277A1	12	25398284	25398284	Missense_Mutation	SNP	C	T	T	KRAS	c.35G>A	p.G12D
118A4277A1		-	-	Frame_Shift_Del	INS	-	-	-	ARID2	C.2032_2038de	P.A678Lfs*21
118A4277A1	12	121426784	121426784	Missense_Mutation	SNP	C	T	T	HNF1A	c.475C>T	p.R159W
118A4277A1	17	7577548	7577548	Missense_Mutation	SNP	C	T	T	TP53	c.733G>A	p.G245S
118A4277A1	19	14208444	14208444	Missense_Mutation	SNP	A	G	G	PRKACA	c.589T>C	p.W197R
118A4280A1	1	17349155	17349155	Missense_Mutation	SNP	A	T	T	SDHB	c.713T>A	p.F238Y
118A4280A1		-	-	Missense_Mutation	SNP	-	-	-	FAM46C	.857_858delinsT	p.R286I
118A4280A1	1	158647473	158647473	Splice_Site	SNP	G	A	A	SPTA1	c.957+7C>T	-
118A4280A1		-	-	Frame_Shift_Del	INS	-	-	-	ARID2	C.2560_2561de	P.V854Nfs*96
118A4280A1		-	-	Frame_Shift_Del	INS	-	-	-	RB1	_2058delinsCC	p.F684Pfs*7
118A4280A1	17	7578190	7578190	Missense_Mutation	SNP	T	C	C	TP53	c.659A>G	p.Y220C
118A4280A1	19	10610252	10610252	Missense_Mutation	SNP	A	T	T	KEAP1	c.458T>A	p.L153H
118A4280A1	19	42777783	42777783	Missense_Mutation	SNP	C	G	G	CIC	c.1848C>G	p.F616L
118A4280A1		-	-	Missense_Mutation	SNP	-	-	-	LRP1B	5740_5741delins	p.G1914L
118A4280A1	22	23523338	23523338	Missense_Mutation	SNP	C	T	T	BCR	c.191C>T	p.A64V
118A4280A1	3	178922328	178922328	Missense_Mutation	SNP	C	G	G	PIK3CA	c.1097C>G	p.P366R
118A4280A1	3	187443373	187443373	Missense_Mutation	SNP	G	A	A	BCL6	c.1753C>T	p.R585W
118A4280A1	X	47045541	47045541	Missense_Mutation	SNP	G	T	T	RBM10	c.2508G>T	p.R836S
118A4284A1	16	10031854	10031854	Missense_Mutation	SNP	C	A	A	GRIN2A	c.969G>T	p.Q323H
118A4284A1	19	11143993	11143993	Missense_Mutation	SNP	C	T	T	SMARCA4	c.3574C>T	p.R1192C
118A4298A1	13	48939100	48939100	Missense_Mutation	SNP	T	C	C	RB1	c.932T>C	p.L311P
118A4298A1	17	78914407	78914407	Splice_Site	SNP	G	T	T	RPTOR	c.3025+6G>T	-
118A4298A1	19	4110622	4110622	Missense_Mutation	SNP	C	A	A	MAP2K2	c.335G>T	p.R112L
118A4298A1	2	209113112	209113112	Missense_Mutation	SNP	C	A	A	IDH1	c.395G>T	p.R132L
118A4298A1	6	138198224	138198224	Missense_Mutation	SNP	G	A	A	TNFAIP3	c.817G>A	p.V273I
118A4298A1	9	80336361	80336361	Missense_Mutation	SNP	T	A	A	GNAQ	c.958A>T	p.S320C
118A4330A1	1	27105529	27105529	Nonsense_Mutation	SNP	G	T	T	ARID1A	c.5140G>T	p.E1714*
118A4330A1	7	117149095	117149095	Missense_Mutation	SNP	G	T	T	CFTR	c.172G>T	p.D58Y
118A4330A1	9	21971120	21971120	Nonsense_Mutation	SNP	G	A	A	CDKN2A	c.238C>T	p.R80*
118A4336A1		-	-	Missense_Mutation	SNP	-	-	-	STK11	c.190_192dup	p.K64dup
118A4336A1	2	212615432	212615432	Splice_Site	SNP	G	A	A	ERBB4	c.557-3C>T	-
118A4336A1	7	151917611	151917611	Nonsense_Mutation	SNP	G	A	A	KMT2C	c.3709C>T	p.R1237*
118A4375A1	12	57579467	57579467	Missense_Mutation	SNP	G	A	A	LRP1	c.6617G>A	p.R2206H
118A4375A1	18	48604773	48604773	Missense_Mutation	SNP	C	A	A	SMAD4	c.1595C>A	p.A532D
118A4375A1	21	44524456	44524456	Missense_Mutation	SNP	G	A	A	U2AF1	c.101C>T	p.S34F
118A4375A1	8	32505505	32505505	Missense_Mutation	SNP	T	C	C	NRG1	c.269T>C	p.L90P
118A4375A1		-	-	Frame_Shift_Del	INS	-	-	-	AR	C.1484del	P.S495Tfs*15
118A4386A1		-	-	Frame_Shift_Del	INS	-	-	-	ARID1A	C.1015del	P.A339Lfs*24
118A4386A1	12	25398284	25398284	Missense_Mutation	SNP	C	T	T	KRAS	c.35G>A	p.G12D
118A4386A1	13	32912573	32912573	Missense_Mutation	SNP	C	G	G	BRCA2	c.4081C>G	p.Q1361E
118A4386A1	16	3900377	3900377	Missense_Mutation	SNP	G	A	A	CREBBP	c.719C>T	p.A240V
118A4386A1	18	48604643	48604643	Nonsense_Mutation	SNP	G	T	T	SMAD4	c.1465G>T	p.G489*
118A4386A1	20	36031747	36031747	Missense_Mutation	SNP	A	C	C	SRC	c.1576A>C	p.T526P
118A4386A1	5	67591097	67591097	Missense_Mutation	SNP	A	G	G	PIK3R1	c.1690A>G	p.N564D
118A4387A1	12	56491645	56491645	Missense_Mutation	SNP	G	T	T	ERBB3	c.2537G>T	p.S846I
118A4387A1	12	57858503	57858503	Missense_Mutation	SNP	C	T	T	GLI1	c.118C>T	p.R40W
118A4387A1	17	7577509	7577509	Missense_Mutation	SNP	C	T	T	TP53	c.772G>A	p.E258K
118A4387A1	2	191903933	191903933	Missense_Mutation	SNP	C	T	T	STAT4	c.1426G>A	p.D476N
118A4387A1		-	-	Missense_Mutation	SNP	-	-	-	PDGFRA	904_1905delins	p.S635L
118A4387A1	5	131924401	131924401	Missense_Mutation	SNP	T	A	A	RAD50	c.1074T>A	p.D358E
118A4387A1	9	21971120	21971120	Nonsense_Mutation	SNP	G	A	A	CDKN2A	c.238C>T	p.R80*
118A4434A1	3	178916944	178916944	Missense_Mutation	SNP	A	G	G	PIK3CA	c.331A>G	p.K111E
118A4438A1	12	115118843	115118843	Missense_Mutation	SNP	G	T	T	TBX3	c.498C>A	p.H166Q
118A4438A1	15	66727441	66727441	Missense_Mutation	SNP	T	G	G	MAP2K1	c.157T>G	p.F53V
118A4438A1		-	-	Frame_Shift_Ins	INS	-	-	-	FUS	c.621_624dup	p.G209Lfs*33
118A4438A1	17	70120517	70120517	Missense_Mutation	SNP	A	G	G	SOX9	c.1519A>G	p.T507A
118A4438A1		-	-	In_Frame_Del	INS	-	-	-	BAP1	c.1255delinsTG	p.K419*
118A4486A1	1	179090819	179090819	Nonsense_Mutation	SNP	T	A	A	ABL2	c.871A>T	p.K291*
118A4486A1	19	15288780	15288780	Missense_Mutation	SNP	G	A	A	NOTCH3	c.3959C>T	p.S1320L
118A4486A1	3	41266137	41266137	Missense_Mutation	SNP	C	T	T	CTNNB1	c.134C>T	p.S45F
118A4486A1	3	142178079	142178079	Missense_Mutation	SNP	G	C	C	ATR	c.7339C>G	p.P2447A
118A4554A1	10	89720789	89720789	Nonsense_Mutation	SNP	G	T	T	PTEN	c.940G>T	p.E314*
118A4554A1	11	92088480	92088480	Missense_Mutation	SNP	C	G	G	FAT3	c.3202C>G	p.R1068G
118A4554A1		-	-	Frame_Shift_Del	INS	-	-	-	TP53	.547_553delins	P.S183Mfs*24
118A4682A2	2	58468446	58468446	Missense_Mutation	SNP	C	T	T	FANCL	c.3G>A	p.M1I
118A4689A1	12	12022535	12022535	Missense_Mutation	SNP	C	T	T	ETV6	c.641C>T	p.P214L
118A4689A1	12	49423015	49423015	Nonsense_Mutation	SNP	C	A	A	KMT2D	c.14080G>T	p.E4694*
118A4689A1	17	7578406	7578406	Missense_Mutation	SNP	C	T	T	TP53	c.524G>A	p.R175H
118A4689A1	17	7579489	7579489	Missense_Mutation	SNP	C	T	T	TP53	c.198G>A	p.M66I
118A4689A1		-	-	Frame_Shift_Del	INS	-	-	-	SMAD2	C.503_506del	P.H168Pfs*39
118A4689A1	5	1268675	1268675	Missense_Mutation	SNP	C	T	T	TERT	c.2542G>A	p.D848N
118A4689A1	7	81339472	81339472	Missense_Mutation	SNP	A	G	G	HGF	c.1532T>C	p.L511S
118A4689A1	9	21968728	21968728	Missense_Mutation	SNP	G	A	A	CDKN2A	c.500C>T	p.A167V
118A4698A1	12	57598259	57598259	Missense_Mutation	SNP	G	A	A	LRP1	c.11018G>A	p.C3673Y
118A4698A1	2	209113113	209113113	Missense_Mutation	SNP	G	A	A	IDH1	c.394C>T	p.R132C
118A4698A1	3	71021168	71021168	Missense_Mutation	SNP	A	G	G	FOXP1	c.1694T>C	p.V565A

118A4698A1	5	149449595	149449595	Missense_Mutation	SNP	C	G	G	CSF1R	c.1351G>C	p.D451H
118A4698A1	7	140453136	140453136	Missense_Mutation	SNP	A	T	T	BRAF	c.1799T>A	p.V600E
118A4765A2	1	11190635	11190635	Missense_Mutation	SNP	C	T	T	MTOR	c.5564G>A	p.S1855N
118A4765A2	1	16456803	16456803	Nonsense_Mutation	SNP	T	A	A	EPHA2	c.2587A>T	p.K863*
118A4765A2	14	35871868	35871868	Missense_Mutation	SNP	T	C	C	NFKBIA	c.638A>G	p.E213G
118A4765A2	4	153244237	153244237	Missense_Mutation	SNP	G	T	T	FBXW7	c.1920C>A	p.S640R
118B4778A1	12	25398281	25398281	Missense_Mutation	SNP	C	T	T	KRAS	c.38G>A	p.G13D
118B4778A1	14	50971556	50971556	Missense_Mutation	SNP	C	G	G	MAP4K5	c.128G>C	p.G43A
118B4778A1		-	-	Frame_Shift_Del	INS	-	-	-	TP53	C.1045del	P.E349Nfs*21
118B4778A1	3	30715688	30715688	Missense_Mutation	SNP	C	A	A	TGFBR2	c.1346C>A	p.S449Y
118B4778A1	3	52436390	52436390	Nonsense_Mutation	SNP	G	A	A	BAP1	c.2104C>T	p.Q702*
118B4778A1	3	195611671	195611671	Splice_Site	SNP	C	A	A	TNK2	c.456+12G>T	-
118B4778A1	5	149510154	149510154	Missense_Mutation	SNP	G	C	C	PDGFRB	c.1315C>G	p.R439G
118B4778A1	9	87549162	87549162	Missense_Mutation	SNP	C	G	G	NTRK2	c.1719C>G	p.C573W
118B4778A1		-	-	Frame_Shift_Del	INS	-	-	-	AMER1	C.275_306del	P.K92Sfs*5
118B4782A1	15	66729181	66729181	Missense_Mutation	SNP	A	G	G	MAP2K1	c.389A>G	p.Y130C
118B4782A1	3	37067240	37067240	Missense_Mutation	SNP	T	A	A	MLH1	c.1151T>A	p.V384D
118B4782A1		-	-	Frame_Shift_Del	INS	-	-	-	CDKN2A	C.47_50del	P.L16Pfs*9
118B4789A1	19	1207162	1207162	Nonsense_Mutation	SNP	A	T	T	STK11	c.250A>T	p.K84*
118B4789A1	7	151873336	151873336	Nonsense_Mutation	SNP	G	A	A	KMT2C	c.9202C>T	p.Q3068*
118B4791A2	1	158614085	158614085	Missense_Mutation	SNP	C	G	G	SPTA1	c.4296G>C	p.L1432F
118B4791A2	11	92495145	92495145	Missense_Mutation	SNP	G	A	A	FAT3	c.3793G>A	p.E1265K
118B4791A2	11	92616277	92616277	Missense_Mutation	SNP	C	T	T	FAT3	c.12655C>T	p.R4219C
118B4791A2	11	92616454	92616454	Missense_Mutation	SNP	C	T	T	FAT3	c.12832C>T	p.R4278W
118B4791A2	11	94153350	94153350	Splice_Site	SNP	G	C	C	MRE11	c.2071-3C>G	-
118B4791A2	17	1303394	1303394	Missense_Mutation	SNP	C	T	T	YWHAE	c.11G>A	p.R4Q
118B4791A2		-	-	Frame_Shift_Del	INS	-	-	-	SMAD4	C.1245_1248de	P.D415Efs*20
118B4791A2	18	48603032	48603032	Nonsense_Mutation	SNP	C	T	T	SMAD4	c.1333C>T	p.R445*
118B4791A2	19	15288622	15288622	Missense_Mutation	SNP	C	T	T	NOTCH3	c.4117G>A	p.E1373K
118B4791A2		-	-	Frame_Shift_Ins	INS	-	-	-	NFE2L2	C.304dup	P.Y102Lfs*16
118B4791A2	3	37035147	37035147	Missense_Mutation	SNP	G	C	C	MLH1	c.109G>C	p.E37Q
118B4791A2	4	126238851	126238851	Missense_Mutation	SNP	G	A	A	FAT4	c.1285G>A	p.E429K
118B4791A2	7	42088134	42088134	Missense_Mutation	SNP	G	A	A	GLI3	c.635C>T	p.S212L
118B4791A2	7	140453155	140453155	Missense_Mutation	SNP	C	T	T	BRAF	c.1780G>A	p.D594N
118B4791A2	X	129147104	129147104	Missense_Mutation	SNP	G	A	A	BCORL1	c.356G>A	p.G119E
118B4864A2	19	18266930	18266930	Missense_Mutation	SNP	G	A	A	PIK3R2	c.241G>A	p.V81M
118B4864A2	2	209113113	209113113	Missense_Mutation	SNP	G	A	A	IDH1	c.394C>T	p.R132C
118B4864A2	3	138375035	138375035	Missense_Mutation	SNP	C	A	A	PIK3CB	c.3024G>T	p.L1008F
118B4864A2	8	69002809	69002809	Splice_Site	SNP	T	C	C	PREX2	c.2114-5T>C	-
118B4888A1	12	25398284	25398284	Missense_Mutation	SNP	C	T	T	KRAS	c.35G>A	p.G12D
118B4888A1	15	66727517	66727517	Missense_Mutation	SNP	A	G	G	MAP2K1	c.233A>G	p.N78S
118B4888A1	17	7576852	7576852	Splice_Site	SNP	C	T	T	TP53	c.993+1G>A	-
118B4888A1	18	48591919	48591919	Missense_Mutation	SNP	G	A	A	SMAD4	c.1082G>A	p.R361H
118B4888A1	X	76874372	76874372	Missense_Mutation	SNP	T	A	A	ATRX	c.5350A>T	p.I1784F
118B4909A1	12	50479178	50479178	Missense_Mutation	SNP	C	T	T	SMARCD1	c.26C>T	p.S9F
118B4909A1	13	48919331	48919331	Nonsense_Mutation	SNP	G	T	T	RB1	c.496G>T	p.E166*
118B4909A1	16	348049	348049	Nonsense_Mutation	SNP	G	T	T	AXIN1	c.1457C>A	p.S486*
118B4909A1		-	-	Frame_Shift_Ins	INS	-	-	-	PRSS8	c.882_903dup	p.D302Pfs*71
118B4909A1	17	7572986	7572986	Nonsense_Mutation	SNP	G	A	A	TP53	c.1123C>T	p.Q375*
118B4909A1	4	153249457	153249457	Missense_Mutation	SNP	G	A	A	FBXW7	c.1321C>T	p.R441W
118B4909A1	7	86394636	86394636	Nonsense_Mutation	SNP	C	T	T	GRM3	c.175C>T	p.R59*
118B4909A1	8	69033161	69033161	Nonsense_Mutation	SNP	C	T	T	PREX2	c.3601C>T	p.Q1201*
118B4937A1	1	27107195	27107195	Nonsense_Mutation	SNP	C	A	A	ARID1A	c.6806C>A	p.S2269*
118B4937A1	2	209113113	209113113	Missense_Mutation	SNP	G	A	A	IDH1	c.394C>T	p.R132C
118B4937A1	3	52643328	52643328	Splice_Site	SNP	C	A	A	PBRM1	c.2567+1G>T	-
118B4937A1	3	178916930	178916930	Missense_Mutation	SNP	G	T	T	PIK3CA	c.317G>T	p.G106V
118B5017A1	12	25398284	25398284	Missense_Mutation	SNP	C	T	T	KRAS	c.35G>A	p.G12D
118B5017A1	X	110439168	110439168	Missense_Mutation	SNP	G	T	T	PAK3	c.1209G>T	p.L403F
118B5044A1	12	25398284	25398284	Missense_Mutation	SNP	C	T	T	KRAS	c.35G>A	p.G12D
118B5044A1	16	31202119	31202119	Missense_Mutation	SNP	C	G	G	FUS	c.1349C>G	p.P450R
118B5044A1	17	7577538	7577538	Missense_Mutation	SNP	C	T	T	TP53	c.743G>A	p.R248Q
118B5044A1	17	56492700	56492700	Missense_Mutation	SNP	C	T	T	RNF43	c.239G>A	p.G80E
118B5044A1	6	138199731	138199731	Missense_Mutation	SNP	G	A	A	TNFAIP3	c.1149G>A	p.M383I
118B5053A1	12	25398284	25398284	Missense_Mutation	SNP	C	T	T	KRAS	c.35G>A	p.G12D
118B5053A1	17	7578539	7578539	Missense_Mutation	SNP	T	A	A	TP53	c.391A>T	p.N131Y
118B5053A1	8	93017450	93017450	Missense_Mutation	SNP	C	T	T	RUNX1T1	c.523G>A	p.A175T
118B5053A1	9	21971186	21971186	Nonsense_Mutation	SNP	G	A	A	CDKN2A	c.172C>T	p.R58*
118B5073A1	12	6692265	6692265	Missense_Mutation	SNP	G	C	C	CHD4	c.4075C>G	p.Q1359E
118B5073A1	17	17118505	17118505	Missense_Mutation	SNP	C	T	T	FLCN	c.1426G>A	p.D476N
118B5073A1	19	1220640	1220640	Nonsense_Mutation	SNP	C	T	T	STK11	c.658C>T	p.Q220*
118B5073A1	19	10600447	10600447	Missense_Mutation	SNP	G	A	A	KEAP1	c.1408C>T	p.R470C
118B5073A1	20	31022862	31022862	Missense_Mutation	SNP	C	A	A	ASXL1	c.2347C>A	p.P783T
118B5110A1	1	115258745	115258745	Missense_Mutation	SNP	C	G	G	NRAS	c.37G>C	p.G13R
118B5110A1	15	90631839	90631839	Missense_Mutation	SNP	T	A	A	IDH2	c.514A>T	p.R172W
118B5110A1	17	7578263	7578263	Nonsense_Mutation	SNP	G	A	A	TP53	c.586C>T	p.R196*
118B5110A1	17	7578454	7578454	Missense_Mutation	SNP	G	A	A	TP53	c.476C>T	p.A159V
118B5110A1		-	-	Frame_Shift_Ins	INS	-	-	-	BCL2L11	c.172_173dup	p.S59Afs*26
118B5110A1	3	52621446	52621446	Nonsense_Mutation	SNP	C	A	A	PBRM1	c.2971G>T	p.E991*
118B5110A1	5	121758671	121758671	Missense_Mutation	SNP	C	T	T	SNCAIP	c.239C>T	p.S80L
118B5118A1		-	-	Frame_Shift_Del	INS	-	-	-	ACVR1B	C.1009del	P.R337Gfs*4
118B5118A1		-	-	In_Frame_Del	INS	-	-	-	PARP2	C.1236_1241de	P.K413_E414del
118B5118A1	17	37666010	37666010	Missense_Mutation	SNP	G	A	A	CDK12	c.2662G>A	p.E888K
118B5118A1		-	-	Splice_Site	SNP	-	-	-	ETV4	C.-12_1del	-
118B5118A1	4	55597558	55597558	Missense_Mutation	SNP	G	A	A	KIT	c.2206G>A	p.A736T
118B5118A1	4	55599291	55599291	Missense_Mutation	SNP	C	A	A	KIT	c.2417C>A	p.T806K
118B5121A1	11	94200987	94200987	Nonsense_Mutation	SNP	G	A	A	MRE11	c.1090C>T	p.R364*
118B5121A1	12	25398282	25398282	Missense_Mutation	SNP	C	A	A	KRAS	c.37G>T	p.G13C
118B5121A1		-	-	In_Frame_Ins	INS	-	-	-	KMT2D	c.4484dup	p.Y1495*
118B5121A1	12	112926890	112926890	Missense_Mutation	SNP	A	G	G	PTPN11	c.1510A>G	p.M504V
118B5121A1	17	7578534	7578534	Missense_Mutation	SNP	C	G	G	TP53	c.396G>C	p.K132N
118B5121A1	17	63010545	63010545	Missense_Mutation	SNP	C	T	T	GNA13	c.964G>A	p.V322I
118B5121A1	18	48591891	48591891	Missense_Mutation	SNP	G	A	A	SMAD4	c.1054G>A	p.G352R
118B5121A1	7	140481402	140481402	Missense_Mutation	SNP	C	T	T	BRAF	c.1406G>A	p.G469E
118B5121A1	8	139164639	139164639	Missense_Mutation	SNP	G	C	C	FAM135B	c.2079C>G	p.S693R
118B5121A1	9	139391547	139391547	Missense_Mutation	SNP	G	A	A	NOTCH1	c.6644C>T	p.S2215L
118B5127A1		-	-	Frame_Shift_Del	INS	-	-	-	AXIN1	C.1632_1668de	P.R545Gfs*148
118C5128A1		-	-	Missense_Mutation	SNP	-	-	-	TET1	c.1035_1037del	p.H345del
118C5128A1	12	25398284	25398284	Missense_Mutation	SNP	C	A	A	KRAS	c.35G>T	p.G12V

118C5132A1	1	27097721	27097721	Nonsense_Mutation	SNP	G	T	T	ARID1A	c.3310G>T	p.E1104*
118C5132A1	2	48027494	48027494	Missense_Mutation	SNP	G	A	A	MSH6	c.2372G>A	p.R791H
118C5132A1		-	-	Frame_Shift_Del	INS	-	-	-	PBRM1	C.310del	P.E104Kfs*9
118C5132A1	4	153247367	153247367	Nonsense_Mutation	SNP	G	A	A	FBXW7	c.1435C>T	p.R479*
118C5169A1	5	131973805	131973805	Missense_Mutation	SNP	G	A	A	RAD50	c.3508G>A	p.D1170N
118C5169A1	X	48887754	48887754	Missense_Mutation	SNP	G	A	A	TFE3	c.1643C>T	p.S548F
118C5218A1	1	120497687	120497687	Missense_Mutation	SNP	C	T	T	NOTCH2	c.2195G>A	p.G732E
118C5218A1	17	29422382	29422382	Nonsense_Mutation	SNP	G	T	T	NF1	c.55G>T	p.E19*
118C5218A1	17	29527461	29527461	Nonsense_Mutation	SNP	C	T	T	NF1	c.910C>T	p.R304*
118C5218A1	9	87342561	87342561	Splice_Site	SNP	C	A	A	NTRK2	c.854-8C>A	-
118C5252A1		-	-	Frame_Shift_Ins	INS	-	-	-	TP53	C.216dup	P.V73Rfs*76
118C5252A1	2	209113113	209113113	Missense_Mutation	SNP	G	A	A	IDH1	c.394C>T	p.R132C
118C5252A1	22	21348283	21348283	Missense_Mutation	SNP	C	T	T	LZTR1	c.1424C>T	p.T475M
118C5252A1	4	106164897	106164897	Nonsense_Mutation	SNP	C	A	A	TET2	c.3765C>A	p.Y1255*
118C5275A1	10	123279564	123279564	Missense_Mutation	SNP	A	G	G	FGFR2	c.868T>C	p.W290R
118C5275A1	12	52377935	52377935	Missense_Mutation	SNP	A	G	G	ACVR1B	c.1087A>G	p.I363V
118C5275A1	19	1221211	1221211	Splice_Site	SNP	G	C	C	STK11	c.735-1G>C	-
118C5275A1	2	209113113	209113113	Missense_Mutation	SNP	G	A	A	IDH1	c.394C>T	p.R132C
118C5275A1	4	153247173	153247173	Missense_Mutation	SNP	T	G	G	FBXW7	c.1629A>C	p.R543S
118C5293A1	1	27056307	27056307	Nonsense_Mutation	SNP	C	T	T	ARID1A	c.1303C>T	p.Q435*
118C5293A1	1	158644142	158644142	Missense_Mutation	SNP	C	A	A	SPTA1	c.1327G>T	p.A443S
118C5293A1		-	-	Frame_Shift_Ins	INS	-	-	-	LRP1B	.4477_4478insT	p.S1493Lfs*14
118C5293A1		-	-	Missense_Mutation	SNP	-	-	-	FEV	c.465_467del	p.Q156del
118C5350A2	11	9607076	9607076	Splice_Site	SNP	T	A	A	WEE1	c.1470+2T>A	-
118C5350A2	12	25398284	25398284	Missense_Mutation	SNP	C	T	T	KRAS	c.35G>A	p.G12D
118C5350A2	12	46230571	46230571	Nonsense_Mutation	SNP	C	T	T	ARID2	c.820C>T	p.R274*
118C5350A2	17	7578460	7578460	Missense_Mutation	SNP	A	T	T	TP53	c.470T>A	p.V157D
118C5350A2	2	141709457	141709457	Nonsense_Mutation	SNP	G	T	T	LRP1B	c.2940C>A	p.C980*
118C5506A1	11	76257063	76257063	Missense_Mutation	SNP	A	G	G	EMSY	c.3496A>G	p.M1166V
118C5506A1	17	11998898	11998898	Missense_Mutation	SNP	C	T	T	MAP2K4	c.400C>T	p.R134W
118C5506A1	17	15968937	15968937	Missense_Mutation	SNP	C	T	T	NCOR1	c.4813G>A	p.A1605T
118C5506A1		-	-	Missense_Mutation	SNP	-	-	-	IDH1	.393_394delinsG	p.R132C
118C5592A1	1	16464453	16464453	Nonsense_Mutation	SNP	C	A	A	EPHA2	c.1207G>T	p.E403*
118C5592A1	2	29917717	29917717	Missense_Mutation	SNP	T	A	A	ALK	c.951A>T	p.R317S
118C5592A1		-	-	Frame_Shift_Ins	INS	-	-	-	BAP1	c.758dup	p.T254Dfs*30
118C5592A1	3	89468367	89468367	Missense_Mutation	SNP	A	T	T	EPHA3	c.1901A>T	p.E634V
118C5592A1	7	127569277	127569277	Nonsense_Mutation	SNP	C	T	T	SND1	c.1564C>T	p.Q522*
118C5592A1	7	151878482	151878482	Missense_Mutation	SNP	T	C	C	KMT2C	c.6463A>G	p.N2155D
118C5592A1	8	92983052	92983052	Missense_Mutation	SNP	C	T	T	RUNX1T1	c.1262G>A	p.R421H
118C5597A1	12	25398284	25398284	Missense_Mutation	SNP	C	A	A	KRAS	c.35G>T	p.G12V
118C5597A1	17	7578190	7578190	Missense_Mutation	SNP	T	C	C	TP53	c.659A>G	p.Y220C
118C5597A1		-	-	Frame_Shift_Ins	INS	-	-	-	SMAD4	c.1242dup	p.D415Rfs*14
118C5597A1		-	-	Frame_Shift_Del	INS	-	-	-	CDKN2A	c.135_150del	p.R46Sfs*2
118C5599A1	12	25398285	25398285	Missense_Mutation	SNP	C	G	G	KRAS	c.34G>C	p.G12R
118C5599A1	17	7577124	7577124	Missense_Mutation	SNP	C	T	T	TP53	c.814G>A	p.V272M
118C5599A1		-	-	Frame_Shift_Del	INS	-	-	-	SMAD4	c.1286_1295del	p.I429Mfs*4
118C5599A1	8	128751001	128751001	Missense_Mutation	SNP	G	A	A	MYC	c.538G>A	p.A180T
118C5599A1		-	-	Frame_Shift_Ins	INS	-	-	-	CDKN2A	c.277dup	p.T93Nfs*27
118C5623A1	1	202729652	202729652	Missense_Mutation	SNP	C	T	T	KDM5B	c.1076G>A	p.R359Q
118C5623A1	12	6702590	6702590	Missense_Mutation	SNP	G	A	A	CHD4	c.2506C>T	p.R836C
118C5623A1	12	49438276	49438276	Nonsense_Mutation	SNP	T	A	A	KMT2D	c.4993A>T	p.K1665*
118C5623A1		-	-	Missense_Mutation	SNP	-	-	-	TP53	c.456_458del	p.P153del
118C5623A1	17	76210392	76210392	Splice_Site	SNP	G	T	T	BIRC5	c.-6G>T	-
118C5623A1	19	15376218	15376218	Missense_Mutation	SNP	G	T	T	BRD4	c.796C>A	p.Q266K
118C5623A1	19	17948832	17948832	Missense_Mutation	SNP	C	T	T	JAK3	c.1610G>A	p.R537Q
118C5623A1	2	141283813	141283813	Missense_Mutation	SNP	C	A	A	LRP1B	c.7869G>T	p.K2623N
118C5623A1	4	1902652	1902652	Missense_Mutation	SNP	C	T	T	NSD2	c.271C>T	p.P91S
118C5623A1	7	148523608	148523608	Missense_Mutation	SNP	T	C	C	EZH2	c.845A>G	p.H282R
118C5635A1	1	27097802	27097802	Nonsense_Mutation	SNP	C	T	T	ARID1A	c.3391C>T	p.Q1131*
118C5635A1	12	25398285	25398285	Missense_Mutation	SNP	C	T	T	KRAS	c.34G>A	p.G12S
118C5635A1		-	-	In_Frame_Ins	INS	-	-	-	FOS	c.735dup	p.D246*
118C5635A1	17	7574003	7574003	Nonsense_Mutation	SNP	G	A	A	TP53	c.1024C>T	p.R342*
118C5635A1	17	11998898	11998898	Missense_Mutation	SNP	C	T	T	MAP2K4	c.400C>T	p.R134W
118C5635A1	2	198267471	198267471	Missense_Mutation	SNP	G	T	T	SF3B1	c.1886C>A	p.A629D
118C5635A1	8	68930080	68930080	Splice_Site	SNP	G	C	C	PREX2	c.142-1G>C	-
118C5635A1	9	21971116	21971116	Missense_Mutation	SNP	G	C	C	CDKN2A	c.242C>G	p.P81R
118C5668A1	1	78428586	78428586	Missense_Mutation	SNP	G	T	T	FUBP1	c.1213C>A	p.Q405K
118C5668A1	12	25398284	25398284	Missense_Mutation	SNP	C	T	T	KRAS	c.35G>A	p.G12D
118C5668A1	X	123224544	123224544	Missense_Mutation	SNP	C	T	T	STAG2	c.3397C>T	p.R1133W
118C5749A1	4	126241857	126241857	Missense_Mutation	SNP	G	A	A	FAT4	c.4291G>A	p.E1431K
118C5749A1	7	140453136	140453136	Missense_Mutation	SNP	A	T	T	BRAF	c.1799T>A	p.V600E
118C5765A1	7	140453136	140453136	Missense_Mutation	SNP	A	T	T	BRAF	c.1799T>A	p.V600E
118C5765A1		-	-	In_Frame_Ins	INS	-	-	-	RBM10	c.310_324dup	).D104_R108dup
118C5766A1	10	123279669	123279669	Missense_Mutation	SNP	G	A	A	FGFR2	c.763C>T	p.R255W
118C5766A1	12	4387995	4387995	Missense_Mutation	SNP	G	A	A	CCND2	c.481G>A	p.E161K
118C5766A1	12	46245246	46245246	Nonsense_Mutation	SNP	C	T	T	ARID2	c.3340C>T	p.Q1114*
118C5766A1	12	52380684	52380684	Missense_Mutation	SNP	G	A	A	ACVR1B	c.1342G>A	p.G448R
118C5766A1	17	7577085	7577085	Missense_Mutation	SNP	C	T	T	TP53	c.853G>A	p.E285K
118C5766A1		-	-	Frame_Shift_Del	INS	-	-	-	NF1	56delinsCTCTCT	p.L1253Sfs*17
118C5766A1	2	141130697	141130697	Missense_Mutation	SNP	C	T	T	LRP1B	c.10648G>A	p.E3550K
118C5766A1	2	170082929	170082929	Splice_Site	SNP	T	C	C	LRP2	c.5394+3A>G	-
118C5766A1	X	70602520	70602520	Splice_Site	SNP	G	A	A	TAF1	c.1725+7G>A	-
118C5792A1	16	396356	396356	Nonsense_Mutation	SNP	G	A	A	AXIN1	c.670C>T	p.Q224*
118C5833A1	1	65323437	65323437	Missense_Mutation	SNP	G	A	A	JAK1	c.1360C>T	p.R454W
118C5833A1	1	158582669	158582669	Nonsense_Mutation	SNP	C	A	A	SPTA1	c.7072G>T	p.E2358*
118C5833A1	13	32931974	32931974	Nonsense_Mutation	SNP	A	C	C	BRCA2	7713A>C;7742T:	[E2571D;L2581*]
118C5833A1	16	3828151	3828151	Missense_Mutation	SNP	G	C	C	CREBBP	c.1974C>G	p.I658M
118C5833A1	2	169999288	169999288	Missense_Mutation	SNP	T	G	G	LRP2	c.13004A>C	p.K4335T
118C5833A1	5	121759251	121759251	Missense_Mutation	SNP	G	C	C	SNCAIP	c.819G>C	p.K273N
118C5833A1	5	176523183	176523183	Splice_Site	SNP	G	A	A	FGFR4	c.1944+3G>A	-
118C5910A1	12	25398284	25398284	Missense_Mutation	SNP	C	T	T	KRAS	c.35G>A	p.G12D
118C5910A1	17	7578454	7578454	Missense_Mutation	SNP	G	A	A	TP53	c.476C>T	p.A159V
118D5912A3	1	158581097	158581097	Missense_Mutation	SNP	C	A	A	SPTA1	c.7217G>T	p.G2406V
118D5912A3	12	25380275	25380275	Missense_Mutation	SNP	T	G	G	KRAS	c.183A>C	p.Q61H
118D5912A3		-	-	Splice_Site	SNP	-	-	-	STK11	.290+3_290+4de	-
118D5912A3	3	89528621	89528621	Missense_Mutation	SNP	C	T	T	EPHA3	c.2921C>T	p.T974M
118D5912A3	4	126369874	126369874	Missense_Mutation	SNP	G	T	T	FAT4	c.7703G>T	p.R2568I
118D5912A3	5	180055906	180055906	Missense_Mutation	SNP	G	A	A	FLT4	c.1079C>T	p.A360V

118D5925A1	12	25398284	25398284	Missense_Mutation	SNP	C	A	A	KRAS	c.35G>T	p.G12V
118D5925A1	7	55259515	55259515	Missense_Mutation	SNP	T	G	G	EGFR	c.2573T>G	p.L858R
118D5925A1	9	21971111	21971111	Missense_Mutation	SNP	G	C	C	CDKN2A	c.247C>G	p.H83D
118D5949A1	12	25398284	25398284	Missense_Mutation	SNP	C	A	A	KRAS	c.35G>T	p.G12V
118D5949A1	17	7577559	7577559	Missense_Mutation	SNP	G	A	A	TP53	c.722C>T	p.S241F
118D5949A1	5	38967303	38967303	Missense_Mutation	SNP	G	A	A	RICTOR	c.1178C>T	p.A393V
118D5949A1	7	41729394	41729394	Missense_Mutation	SNP	G	A	A	INHBA	c.1135C>T	p.R379W
118D5960A1	12	56493528	56493528	Missense_Mutation	SNP	A	T	T	ERBB3	c.2936A>T	p.K979M
118D5960A1		-	-	Splice_Site	SNP	-	-	-	RB1	498_1498+1delir	-
118D5960A1	2	141747096	141747096	Splice_Site	SNP	T	C	C	LRP1B	c.2770+5A>G	-
118D5960A1	22	41521980	41521980	Nonsense_Mutation	SNP	C	G	G	EP300	c.842C>G	p.S281*
118D5960A1	3	37053586	37053586	Missense_Mutation	SNP	A	G	G	MLH1	c.673A>G	p.S225G
118D5960A1	7	81372666	81372666	Splice_Site	SNP	T	C	C	HGF	c.865+3A>G	-
118D5960A1	8	92972730	92972730	Missense_Mutation	SNP	C	A	A	RUNX1T1	c.1444G>T	p.G482C
118D5960A1	9	21994276	21994276	Nonsense_Mutation	SNP	G	A	A	CDKN2A	c.55C>T	p.R19*
118D5965A1		-	-	Frame_Shift_Del	INS	-	-	-	ARID1A	c.3238_3251del	p.L1080Ifs*20
118D5965A1		-	-	Frame_Shift_Ins	INS	-	-	-	NRG3	c.423_424insT	p.A142Cfs*14
118D5965A1	11	92577865	92577865	Missense_Mutation	SNP	C	T	T	FAT3	c.11332C>T	p.R3778C
118D5965A1		-	-	Splice_Site	SNP	-	-	-	ATM	235_1235+1delir	-
118D5965A1	11	108216637	108216637	Splice_Site	SNP	T	C	C	ATM	c.8584+2T>C	-
118D5965A1	12	25398285	25398285	Missense_Mutation	SNP	C	A	A	KRAS	c.34G>T	p.G12C
118D5965A1		-	-	Frame_Shift_Ins	INS	-	-	-	SMAD3	c.177_178dup	p.V60Afs*57
118D5965A1	18	48575197	48575197	Missense_Mutation	SNP	T	G	G	SMAD4	c.391T>G	p.Y131D
118D5965A1	19	4090604	4090604	Missense_Mutation	SNP	C	T	T	MAP2K2	c.1195G>A	p.A399T
118D5965A1	2	141253197	141253197	Missense_Mutation	SNP	A	T	T	LRP1B	c.8971T>A	p.C2991S
118D5965A1	2	170090092	170090092	Missense_Mutation	SNP	G	A	A	LRP2	c.4927C>T	p.R1643W
118D5965A1	7	106509436	106509436	Missense_Mutation	SNP	G	A	A	PIK3CG	c.1430G>A	p.R477H
118D6010J1	3	51979191	51979191	Missense_Mutation	SNP	C	T	T	PARP3	c.812C>T	p.P271L
118D6010J1	3	52441973	52441973	Splice_Site	SNP	C	T	T	BAP1	c.375+1G>A	-
118D6094D1	1	27058078	27058078	Missense_Mutation	SNP	C	A	A	ARID1A	c.1786C>A	p.R596S
118D6094D1	11	60235910	60235910	Missense_Mutation	SNP	C	T	T	MS4A1	c.863C>T	p.S288F
118D6094D1	17	7578550	7578550	Missense_Mutation	SNP	G	A	A	TP53	c.380C>T	p.S127F
118D6094D1		-	-	Frame_Shift_Del	INS	-	-	-	STK11	c.866_878del	p.M289Nfs*43
118D6094D1	3	51978529	51978529	Missense_Mutation	SNP	C	G	G	PARP3	c.436C>G	p.H146D
118D6094D1	3	134880937	134880937	Missense_Mutation	SNP	G	C	C	EPHB1	c.1500G>C	p.M500I
118D6094D1		-	-	Frame_Shift_Del	INS	-	-	-	JAK2	3142_3147delin	p.F1048Lfs*3
118D6094D1	9	21970901	21970901	Missense_Mutation	SNP	C	G	G	CDKN2A	c.457G>C	p.D153H
118D6094D1		-	-	In_Frame_Del	INS	-	-	-	KDM6A	c.491_494del	p.C164*
118D6120A1	12	52385664	52385664	Nonsense_Mutation	SNP	C	T	T	ACVR1B	c.1402C>T	p.Q468*
118D6120A1	12	57598196	57598196	Missense_Mutation	SNP	G	A	A	LRP1	c.10955G>A	p.R3652Q
118D6166A1	12	25398285	25398285	Missense_Mutation	SNP	C	A	A	KRAS	c.34G>T	p.G12C
118D6166A1	17	56492739	56492739	Missense_Mutation	SNP	C	T	T	RNF43	c.200G>A	p.G67D
118D6166A1		-	-	Frame_Shift_Del	INS	-	-	-	EP300	c.3954_3955del	p.G1319Rfs*5
118D6166A1	8	90967754	90967754	Missense_Mutation	SNP	T	C	C	NBN	c.1154A>G	p.K385R
118D6166A1	X	47040707	47040707	Nonsense_Mutation	SNP	C	T	T	RBM10	c.1342C>T	p.Q448*
118D6220A1		-	-	Frame_Shift_Del	INS	-	-	-	ARID1A	c.6001del	p.M2001Cfs*14
118D6220A1	1	115256529	115256529	Missense_Mutation	SNP	T	C	C	NRAS	c.182A>G	p.Q61R
118D6220A1	16	68863701	68863701	Splice_Site	SNP	G	A	A	CDH1	c.2439+1G>A	-
118D6220A1	17	7577539	7577539	Missense_Mutation	SNP	G	A	A	TP53	c.742C>T	p.R248W
118D6220A1	18	48604785	48604785	Missense_Mutation	SNP	T	G	G	SMAD4	c.1607T>G	p.L536R
118D6220A1	22	29687545	29687545	Splice_Site	SNP	C	T	T	EWSR1	c.975-6C>T	-
118D6220A1		-	-	In_Frame_Del	INS	-	-	-	CDKN2A	c.58_63del	p.A20_A21del
118D6255A1		-	-	Frame_Shift_Del	INS	-	-	-	BAP1	c.354del	p.F118Lfs*69
118D6295A1	1	43777390	43777390	Missense_Mutation	SNP	G	A	A	TIE1	c.1382G>A	p.R461H
118D6350A1		-	-	Frame_Shift_Del	INS	-	-	-	ARID1A	95_2000delinsA	p.I666Ffs*76
118D6350A1		-	-	In_Frame_Del	INS	-	-	-	ARID2	c.423_439del	p.Y141*
118D6350A1	3	178936082	178936082	Missense_Mutation	SNP	G	A	A	PIK3CA	c.1624G>A	p.E542K
118D6350A1	7	151879625	151879625	Nonsense_Mutation	SNP	C	A	A	KMT2C	c.5320G>T	p.E1774*
118D6350A1	X	63410813	63410813	Missense_Mutation	SNP	C	T	T	AMER1	c.2354G>A	p.S785N
118D6360A1	1	27101714	27101714	Splice_Site	SNP	A	T	T	ARID1A	c.4993+3A>T	-
118D6360A1	1	158583611	158583611	Missense_Mutation	SNP	G	A	A	SPTA1	c.6889C>T	p.R2297W
118D6360A1	12	25398284	25398284	Missense_Mutation	SNP	C	T	T	KRAS	c.35G>A	p.G12D
118D6360A1	14	95560464	95560464	Missense_Mutation	SNP	C	T	T	DICER1	c.5125G>A	p.D1709N
118D6360A1	14	95562619	95562619	Nonsense_Mutation	SNP	G	T	T	DICER1	c.4638C>A	p.Y1546*
118D6360A1		-	-	Frame_Shift_Del	INS	-	-	-	PALB2	c.1037_1041del	p.K346Tfs*13
118D6360A1	16	56865774	56865774	Missense_Mutation	SNP	A	G	G	NUP93	c.1106A>G	p.N369S
118D6360A1	22	30051652	30051652	Nonsense_Mutation	SNP	C	T	T	NF2	c.586C>T	p.R196*
118D6360A1	22	41566555	41566555	Missense_Mutation	SNP	C	T	T	EP300	c.4432C>T	p.R1478C
118D6360A1	5	131915116	131915116	Missense_Mutation	SNP	A	G	G	RAD50	c.473A>G	p.H158R
118D6360A1	7	106508703	106508703	Missense_Mutation	SNP	A	G	G	PIK3CG	c.697A>G	p.I233V
118D6403A1		-	-	In_Frame_Ins	INS	-	-	-	ARID1A	c.2445dup	p.N816*
118D6403A1	12	25398284	25398284	Missense_Mutation	SNP	C	T	T	KRAS	c.35G>A	p.G12D
118D6403A1	12	49433354	49433354	Missense_Mutation	SNP	T	C	C	KMT2D	c.8093A>G	p.N2698S
118D6403A1	16	9943687	9943687	Missense_Mutation	SNP	G	C	C	GRIN2A	c.1254C>G	p.I418M
118D6403A1	17	7578268	7578268	Missense_Mutation	SNP	A	C	C	TP53	c.581T>G	p.L194R
118D6403A1		-	-	Frame_Shift_Ins	INS	-	-	-	NCOR1	c.1573dup	p.T525Nfs*11
118D6403A1	18	48591890	48591890	Missense_Mutation	SNP	T	A	A	SMAD4	c.1053T>A	p.D351E
118D6403A1		-	-	Frame_Shift_Ins	INS	-	-	-	RANBP2	.534_535insTTT	p.K179Ffs*2
118D6403A1	3	178952085	178952085	Missense_Mutation	SNP	A	T	T	PIK3CA	c.3140A>T	p.H1047L
118D6420A1		-	-	Frame_Shift_Ins	INS	-	-	-	CDKN1B	c.204_205insCC	p.L70Pfs*2
118D6420A1		-	-	Frame_Shift_Ins	INS	-	-	-	CDKN1B	c.224_225dup	p.W76Sfs*44
118D6420A1	12	49434810	49434810	Missense_Mutation	SNP	C	T	T	KMT2D	c.6743G>A	p.R2248H
118D6420A1	12	115118961	115118961	Splice_Site	SNP	G	T	T	TBX3	c.390-10C>A	-
118D6420A1	22	21351626	21351626	Missense_Mutation	SNP	G	A	A	LZTR1	c.2512G>A	p.A838T
118D6420A1	3	41266104	41266104	Missense_Mutation	SNP	G	A	A	CTNNB1	c.101G>A	p.G34E
118D6420A1		-	-	Frame_Shift_Del	INS	-	-	-	FBXW7	C.798del	P.K266Nfs*3
118D6420A1	8	69039666	69039666	Splice_Site	SNP	C	T	T	PREX2	c.3766+12C>T	-
118D6420A1	8	139207558	139207558	Splice_Site	SNP	G	A	A	FAM135B	c.824-8C>T	-
118D6519A1	12	25398285	25398285	Missense_Mutation	SNP	C	A	A	KRAS	c.34G>T	p.G12C
118D6519A1	17	7578554	7578554	Missense_Mutation	SNP	A	T	T	TP53	c.376T>A	p.Y126N
118D6519A1	20	57430164	57430164	Missense_Mutation	SNP	C	T	T	GNAS	c.1657C>T	p.P553S
118D6519A1		-	-	In_Frame_Del	INS	-	-	-	CDKN2A	c.34_69del	p.S12_G23del
118D6546A1	1	27023855	27023855	Nonsense_Mutation	SNP	C	T	T	ARID1A	c.961C>T	p.Q321*
118D6546A1	11	92088462	92088462	Missense_Mutation	SNP	G	A	A	FAT3	c.3184G>A	p.V1062M
118D6546A1	15	49716719	49716719	Nonsense_Mutation	SNP	G	A	A	FGF7	c.225G>A	p.W75*
118D6546A1	15	67477203	67477203	Splice_Site	SNP	G	A	A	SMAD3	c.1009+1G>A	-
118D6546A1	18	48591918	48591918	Missense_Mutation	SNP	C	T	T	SMAD4	c.1081C>T	p.R361C
118D6546A1	2	198266834	198266834	Missense_Mutation	SNP	T	C	C	SF3B1	c.2098A>G	p.K700E

118D6546A1	7	42065982	42065982	Missense_Mutation	SNP	G	A	A	GLI3	c.1058C>T	p.A353V
118D6546A1	7	140453154	140453154	Missense_Mutation	SNP	T	C	C	BRAF	c.1781A>G	p.D594G
118D6605A1	1	27100207	27100207	Nonsense_Mutation	SNP	C	T	T	ARID1A	c.4003C>T	p.R1335*
118D6605A1	12	115111972	115111972	Nonsense_Mutation	SNP	G	A	A	TBX3	c.1768C>T	p.Q590*
118D6605A1	17	7577520	7577520	Missense_Mutation	SNP	A	T	T	TP53	c.761T>A	p.I254N
118D6605A1		-	-	Missense_Mutation	SNP	-	-	-	ERBB2	C.2090_2092de	P.V697del
118D6605A1	2	170050322	170050322	Missense_Mutation	SNP	C	T	T	LRP2	c.8779G>A	p.G2927S
118D6605A1	3	47103828	47103828	Nonsense_Mutation	SNP	G	A	A	SETD2	c.6118C>T	p.R2040*
118D6605A1	7	2976766	2976766	Missense_Mutation	SNP	C	T	T	CARD11	c.1246G>A	p.E416K
118D6605A1	7	50450283	50450283	Missense_Mutation	SNP	A	G	G	IKZF1	c.467A>G	p.Q156R
118D6605A1	7	140453193	140453193	Missense_Mutation	SNP	T	C	C	BRAF	c.1742A>G	p.N581S
118D6683A1	17	7577551	7577551	Missense_Mutation	SNP	C	T	T	TP53	c.730G>A	p.G244S
118D6683A1		-	-	Frame_Shift_Del	INS	-	-	-	PBRM1	c.1106del	p.G369Efs*35
118D6684A1	17	7577141	7577141	Missense_Mutation	SNP	C	T	T	TP53	c.797G>A	p.G266E
118D6684A1		-	-	Frame_Shift_Del	INS	-	-	-	TP53	c.452delinsAA	p.P151Qfs*30
118D6684A1	2	209113112	209113112	Missense_Mutation	SNP	C	A	A	IDH1	c.395G>T	p.R132L
118D6719A1	1	226252030	226252030	Splice_Site	SNP	G	A	A	H3F3A	c.-23-4G>A	-
118D6719A1	12	4488690	4488690	Missense_Mutation	SNP	C	T	T	FGF23	c.59G>A	p.S20N
118D6719A1	12	25398281	25398281	Missense_Mutation	SNP	C	T	T	KRAS	c.38G>A	p.G13D
118D6719A1	12	49447386	49447386	Missense_Mutation	SNP	A	C	C	KMT2D	c.712T>G	p.L238V
118D6719A1	14	45633742	45633742	Missense_Mutation	SNP	A	G	G	FANCM	c.1762A>G	p.I588V
118D6719A1	15	67473704	67473704	Missense_Mutation	SNP	G	A	A	SMAD3	c.784G>A	p.D262N
118D6719A1	15	93444499	93444499	Missense_Mutation	SNP	A	G	G	CHD2	c.32A>G	p.E11G
118D6719A1	16	89862323	89862323	Missense_Mutation	SNP	C	T	T	FANCA	c.997G>A	p.V333M
118D6719A1	17	7576852	7576852	Splice_Site	SNP	C	T	T	TP53	c.993+1G>A	-
118D6719A1	19	10602580	10602580	Missense_Mutation	SNP	C	T	T	KEAP1	c.998G>A	p.G333D
118D6719A1	2	141215125	141215125	Missense_Mutation	SNP	G	A	A	LRP1B	c.9721C>T	p.H3241Y
118D6719A1	2	198266611	198266611	Missense_Mutation	SNP	C	T	T	SF3B1	c.2225G>A	p.G742D
118D6719A1	3	41266137	41266137	Missense_Mutation	SNP	C	T	T	CTNNB1	c.134C>T	p.S45F
118D6719A1	6	30863250	30863250	Missense_Mutation	SNP	G	C	C	DDR1	c.1583G>C	p.G528A
118D6719A1	7	151846063	151846063	Nonsense_Mutation	SNP	G	A	A	KMT2C	c.12949C>T	p.Q4317*
118D6719A1	8	139160859	139160859	Missense_Mutation	SNP	C	T	T	FAM135B	c.3352G>A	p.V1118I
118D6719A1	9	21971029	21971029	Nonsense_Mutation	SNP	C	T	T	CDKN2A	c.329G>A	p.W110*
118D6719A1	9	137300951	137300951	Missense_Mutation	SNP	G	T	T	RXRA	c.596G>T	p.G199V
118D6719A1	X	47040904	47040904	Splice_Site	SNP	A	T	T	RBM10	c.1436-2A>T	-
118D6898A1	7	151845915	151845915	Missense_Mutation	SNP	A	T	T	KMT2C	c.13097T>A	p.I4366N
118D6898A1		-	-	Splice_Site	SNP	-	-	-	PRKDC	3465-5_3465-4c	-
118D6898A1	9	139391932	139391932	Missense_Mutation	SNP	G	A	A	NOTCH1	c.6259C>T	p.R2087W
118D6984A1	12	25398281	25398281	Missense_Mutation	SNP	C	T	T	KRAS	c.38G>A	p.G13D
118D6984A1	17	7578508	7578508	Missense_Mutation	SNP	C	T	T	TP53	c.422G>A	p.C141Y
118D6984A1	3	178938934	178938934	Missense_Mutation	SNP	G	A	A	PIK3CA	c.2176G>A	p.E726K
118D6984A1	9	21971036	21971036	Missense_Mutation	SNP	C	A	A	CDKN2A	c.322G>T	p.D108Y
118D6984A1		-	-	In_Frame_Del	INS	-	-	-	RXRA	c.84_92del	.M28_P31delinsI
118D6984A1		-	-	Frame_Shift_Del	INS	-	-	-	RBM10	c.705del	p.K235Nfs*31
118D6985A1	1	97771837	97771837	Missense_Mutation	SNP	C	A	A	DPYD	c.2075G>T	p.R692L
118D6985A1	12	57603962	57603962	Splice_Site	SNP	T	C	C	LRP1	c.12580+10T>C	-
118D6985A1	17	7577534	7577534	Missense_Mutation	SNP	C	A	A	TP53	c.747G>T	p.R249S
118D6985A1	19	4123888	4123888	Splice_Site	SNP	C	A	A	MAP2K2	c.-16G>T	-
118E7021A1	12	49441824	49441824	Missense_Mutation	SNP	C	A	A	KMT2D	c.4160G>T	p.G1387V
118E7021A1		-	-	Frame_Shift_Del	INS	-	-	-	TP53	.769_771delinsA	P.L257Mfs*88
118E7021A1	17	7578457	7578457	Missense_Mutation	SNP	C	A	A	TP53	c.473G>T	p.R158L
118E7021A1	19	30308420	30308420	Missense_Mutation	SNP	G	A	A	CCNE1	c.434G>A	p.R145Q
118E7021A1	2	141272296	141272296	Missense_Mutation	SNP	C	A	A	LRP1B	c.8195G>T	p.C2732F
118E7021A1	2	141812805	141812805	Missense_Mutation	SNP	C	G	G	LRP1B	c.1432G>C	p.D478H
118E7021A1	22	21348031	21348031	Missense_Mutation	SNP	C	G	G	LZTR1	c.1341C>G	p.F447L
118E7021A1	6	32551999	32551999	Missense_Mutation	SNP				HLA-DRB1	c.231C>G	p.D77E
118E7021A1	7	50455067	50455067	Missense_Mutation	SNP	A	G	G	IKZF1	c.614A>G	p.Y205C
118E7059A1	17	7577559	7577559	Missense_Mutation	SNP	G	T	T	TP53	c.722C>A	p.S241Y
118E7059A1	17	29546098	29546098	Nonsense_Mutation	SNP	C	T	T	NF1	c.1603C>T	p.Q535*
118E7059A1	17	29661945	29661945	Nonsense_Mutation	SNP	C	T	T	NF1	c.5902C>T	p.R1968*
118E7059A1	19	2213593	2213593	Missense_Mutation	SNP	C	G	G	DOT1L	c.1613C>G	p.A538G
118E7059A1	19	10602369	10602369	Nonsense_Mutation	SNP	C	T	T	KEAP1	c.1209G>A	p.W403*
118E7059A1		-	-	Frame_Shift_Del	INS	-	-	-	KEAP1	C.1006del	P.R336Dfs*64
118E7059A1	19	18267022	18267022	Splice_Site	SNP	A	G	G	PIK3R2	c.322+11A>G	-
118E7059A1	4	20490443	20490443	Nonsense_Mutation	SNP	C	T	T	SLIT2	c.613C>T	p.R205*
118E7059A1	4	153249384	153249384	Missense_Mutation	SNP	C	T	T	FBXW7	c.1394G>A	p.R465H
118E7059A1		-	-	Frame_Shift_Ins	INS	-	-	-	MAGI2	C.4262dup	P.A1422Rfs*19
118E7103A1	13	28622541	28622541	Missense_Mutation	SNP	T	G	G	FLT3	c.1076A>C	p.Y359S
118E7103A1	16	89818613	89818613	Missense_Mutation	SNP	T	C	C	FANCA	c.2999A>G	p.H1000R
118E7103A1	18	42531133	42531133	Missense_Mutation	SNP	G	A	A	SETBP1	c.1828G>A	p.V610I
118E7103A1	5	112173549	112173549	Missense_Mutation	SNP	A	G	G	APC	c.2258A>G	p.H753R
118E7103A1	X	48125796	48125796	Missense_Mutation	SNP	A	G	G	SSX1	c.541A>G	p.S181G
118E7142A1	12	25398284	25398284	Missense_Mutation	SNP	C	A	A	KRAS	c.35G>T	p.G12V
118E7142A1	17	7578406	7578406	Missense_Mutation	SNP	C	T	T	TP53	c.524G>A	p.R175H
118E7142A1		-	-	Splice_Site	SNP	-	-	-	MAP2K4	c.633+2dup	-
118E7142A1	2	212286784	212286784	Missense_Mutation	SNP	G	T	T	ERBB4	c.2912C>A	p.A971D
118E7142A1		-	-	Missense_Mutation	SNP	-	-	-	CDKN2A	C.130_132del	P.Y44del
118E7171A1	1	158637645	158637645	Splice_Site	SNP	T	C	C	SPTA1	c.2038+3A>G	-
118E7171A1	15	88669526	88669526	Nonsense_Mutation	SNP	G	A	A	NTRK3	c.1372C>T	p.R458*
118E7171A1	17	7577509	7577509	Nonsense_Mutation	SNP	C	A	A	TP53	c.772G>T	p.E258*
118E7171A1	17	12011106	12011106	Splice_Site	SNP	G	C	C	MAP2K4	c.514-1G>C	-
118E7171A1	3	70008488	70008488	Nonsense_Mutation	SNP	C	T	T	MITF	c.775C>T	p.R259*
118E7171A1	3	169998173	169998173	Missense_Mutation	SNP	G	C	C	PRKCI	c.864G>C	p.E288D
118E7171A1	5	180057057	180057057	Missense_Mutation	SNP	G	A	A	FLT4	c.562C>T	p.R188W
118E7171A1	9	98247979	98247979	Missense_Mutation	SNP	T	A	A	PTCH1	c.572A>T	p.Y191F
118E7187A1	10	123274822	123274822	Missense_Mutation	SNP	C	T	T	FGFR2	c.1096G>A	p.E366K
118E7187A1	12	25398284	25398284	Missense_Mutation	SNP	C	T	T	KRAS	c.35G>A	p.G12D
118E7187A1	13	28919596	28919596	Nonsense_Mutation	SNP	G	A	A	FLT1	c.2341C>T	p.R781*
118E7187A1	17	7577538	7577538	Missense_Mutation	SNP	C	T	T	TP53	c.743G>A	p.R248Q
118E7187A1	18	48591904	48591904	Missense_Mutation	SNP	C	G	G	SMAD4	c.1067C>G	p.P356R
118E7191A1	1	27023696	27023696	Nonsense_Mutation	SNP	C	T	T	ARID1A	c.802C>T	p.Q268*
118E7191A1	1	158651439	158651439	Missense_Mutation	SNP	G	A	A	SPTA1	c.409C>T	p.R137C
118E7191A1	12	25398284	25398284	Missense_Mutation	SNP	C	T	T	KRAS	c.35G>A	p.G12D
118E7191A1	18	48603080	48603080	Nonsense_Mutation	SNP	C	T	T	SMAD4	c.1381C>T	p.Q461*
118E7191A1	2	178098800	178098800	Missense_Mutation	SNP	T	A	A	NFE2L2	c.245A>T	p.E82V
118E7191A1	20	57484421	57484421	Missense_Mutation	SNP	G	A	A	GNAS	c.602G>A	p.R201H
118E7191A1		-	-	Frame_Shift_Del	INS	-	-	-	RBM10	c.495del	p.K165Nfs*101
118E7218A1	1	115256529	115256529	Missense_Mutation	SNP	T	A	A	NRAS	c.182A>T	p.Q61L

118E7218A1	11	64577316	64577316	Missense_Mutation	SNP	A	C	C	MEN1	c.266T>G	p.L89R
118E7218A1	17	7578212	7578212	Nonsense_Mutation	SNP	G	A	A	TP53	c.637C>T	p.R213*
118E7249A1	1	158624504	158624504	Missense_Mutation	SNP	C	A	A	SPTA1	c.2933G>T	p.G978V
118E7249A1	12	46287201	46287201	Splice_Site	SNP	A	G	G	ARID2	c.5148-2A>G	-
118E7249A1	17	7577570	7577570	Missense_Mutation	SNP	C	A	A	TP53	c.711G>T	p.M237I
118E7270A1	17	7578265	7578265	Missense_Mutation	SNP	A	G	G	TP53	c.584T>C	p.I195T
118E7270A1	2	141459401	141459401	Missense_Mutation	SNP	C	A	A	LRP1B	c.6316G>T	p.G2106W
118E7270A1	2	141771296	141771296	Nonsense_Mutation	SNP	C	A	A	LRP1B	c.2209G>T	p.E737*
118E7270A1	21	44524456	44524456	Missense_Mutation	SNP	G	A	A	U2AF1	c.101C>T	p.S34F
118E7270A1	4	20568947	20568947	Missense_Mutation	SNP	G	A	A	SLIT2	c.2788G>A	p.D930N
118E7270A1	4	153247289	153247289	Missense_Mutation	SNP	G	A	A	FBXW7	c.1513C>T	p.R505C
118E7290A1		-	-	Frame_Shift_Del	INS	-	-	-	TP53	.761_772delins	p.I254Kfs*6
118E7290A1	17	7579312	7579312	Splice_Site	SNP	C	T	T	TP53	c.375G>A	-
118E7290A1	6	117679130	117679130	Missense_Mutation	SNP	T	C	C	ROS1	c.3691A>G	p.I1231V
118E7290A1	6	151687723	151687723	Missense_Mutation	SNP	G	A	A	ZBTB2	c.478C>T	p.R160W
118E7304A1		-	-	In_Frame_Ins	INS	-	-	-	ARID2	c.1268dup	p.Y423*
118E7304A1	12	69964192	69964192	Missense_Mutation	SNP	C	T	T	FRS2	c.148C>T	p.R50C
118E7304A1	17	7579377	7579377	Nonsense_Mutation	SNP	G	A	A	TP53	c.310C>T	p.Q104*
118E7304A1	19	41726703	41726703	Missense_Mutation	SNP	A	T	T	AXL	c.248A>T	p.Q83L
118E7306A1	1	8073757	8073757	Nonsense_Mutation	SNP	C	T	T	ERRFI1	c.902G>A	p.W301*
118E7306A1	11	46332655	46332655	Missense_Mutation	SNP	G	A	A	CREB3L1	c.668G>A	p.R223H
118E7306A1	12	46245663	46245663	Missense_Mutation	SNP	G	T	T	ARID2	c.3757G>T	p.A1253S
118E7306A1	17	38563832	38563832	Missense_Mutation	SNP	C	T	T	TOP2A	c.1595G>A	p.R532H
118E7306A1	17	78727989	78727989	Splice_Site	SNP	A	T	T	RPTOR	c.830+4A>T	-
118E7306A1	2	109384745	109384745	Missense_Mutation	SNP	G	A	A	RANBP2	c.7750G>A	p.D2584N
118E7306A1	2	141055406	141055406	Missense_Mutation	SNP	G	A	A	LRP1B	c.12938C>T	p.P4313L
118E7306A1	20	52192647	52192647	Missense_Mutation	SNP	C	T	T	ZNF217	c.2656G>A	p.A886T
118E7306A1	4	187527243	187527243	Missense_Mutation	SNP	T	C	C	FAT1	c.10331A>G	p.N3444S
118E7306A1	7	2977554	2977554	Missense_Mutation	SNP	C	T	T	CARD11	c.1130G>A	p.R377Q
118E7306A1	7	80292448	80292448	Missense_Mutation	SNP	C	T	T	CD36	c.572C>T	p.P191L
118E7306A1	X	66766174	66766174	Missense_Mutation	SNP	G	C	C	AR	c.1186G>C	p.G396R
118E7325A1	1	158605703	158605703	Missense_Mutation	SNP	C	T	T	SPTA1	c.5432G>A	p.R1811Q
118E7325A1	15	93527621	93527621	Nonsense_Mutation	SNP	G	A	A	CHD2	c.3128G>A	p.W1043*
118E7325A1	17	7577099	7577099	Missense_Mutation	SNP	C	G	G	TP53	c.839G>C	p.R280T
118E7325A1	6	157527680	157527680	Missense_Mutation	SNP	G	A	A	ARID1B	c.5405G>A	p.R1802Q
118E7325A1	7	42004863	42004863	Missense_Mutation	SNP	C	T	T	GLI3	c.3808G>A	p.G1270S
118E7325A1	7	142641481	142641481	Nonsense_Mutation	SNP	G	A	A	KEL	c.1420C>T	p.Q474*
118E7344A1		-	-	Frame_Shift_Del	INS	-	-	-	TP53	C.347del	P.S116Lfs*7
118E7344A1	4	153271242	153271242	Missense_Mutation	SNP	C	T	T	FBXW7	c.536G>A	p.R179H
118E7344A1	7	81358997	81358997	Missense_Mutation	SNP	T	C	C	HGF	c.964A>G	p.N322D
118E7407A1	1	32741546	32741546	Missense_Mutation	SNP	C	A	A	LCK	c.513C>A	p.D171E
118E7407A1		-	-	Frame_Shift_Ins	INS	-	-	-	BRCA2	C.1759dup	P.T587Nfs*3
118E7407A1	17	7577108	7577108	Missense_Mutation	SNP	C	A	A	TP53	c.830G>T	p.C277F
118E7407A1		-	-	Frame_Shift_Del	INS	-	-	-	DOT1L	C.4330_4340del	P.L1444Gfs*90
118E7407A1	2	29497991	29497991	Missense_Mutation	SNP	C	G	G	ALK	c.2015G>C	p.R672T
118E7407A1		-	-	In_Frame_Del	INS	-	-	-	PRKCI	C.54_59del	P.S19_G20del
118E7407A1	4	110916047	110916047	Splice_Site	SNP	C	T	T	EGF	c.3005+11C>T	-
118E7407A1	5	176562150	176562150	Missense_Mutation	SNP	T	C	C	NSD1	c.46T>C	p.F16L
118E7446A1	12	112926910	112926910	Missense_Mutation	SNP	G	C	C	PTPN11	c.1530G>C	p.Q510H
118E7446A1	16	396533	396533	Nonsense_Mutation	SNP	T	A	A	AXIN1	c.493A>T	p.K165*
118E7446A1	17	7578394	7578394	Missense_Mutation	SNP	T	A	A	TP53	c.536A>T	p.H179L
118E7446A1	17	29483155	29483155	Splice_Site	SNP	G	A	A	NF1	c.204+11G>A	-
118E7446A1	18	23618578	23618578	Missense_Mutation	SNP	T	C	C	SS18	c.821A>G	p.Q274R
118E7446A1		-	-	Frame_Shift_Del	INS	-	-	-	GNAS	C.736del	P.R246Vfs*25
118E7446A1	22	30077445	30077445	Missense_Mutation	SNP	A	T	T	NF2	c.1592A>T	p.K531M
118E7446A1	8	48771477	48771477	Missense_Mutation	SNP				PRKDC	c.6277A>G	p.M2093V
118E7470A1	1	158612676	158612676	Missense_Mutation	SNP	C	A	A	SPTA1	c.4533G>T	p.E1511D
118E7470A1	11	108224531	108224531	Missense_Mutation	SNP	G	A	A	ATM	c.8710G>A	p.E2904K
118E7470A1	2	141473542	141473542	Missense_Mutation	SNP	C	T	T	LRP1B	c.6023G>A	p.G2008D
118E7470A1	3	52649463	52649463	Missense_Mutation	SNP	C	T	T	PBRM1	1828G>A;1870G	.[D610N;E624Q]
118E7477A1	12	25398284	25398284	Missense_Mutation	SNP	C	T	T	KRAS	c.35G>A	p.G12D
118E7477A1		-	-	Frame_Shift_Del	INS	-	-	-	RNF43	C.69_72del	P.G24Lfs*26
118E7477A1	2	191895815	191895815	Splice_Site	SNP	G	A	A	STAT4	c.2112-9C>T	-
118E7477A1	3	10076158	10076158	Missense_Mutation	SNP	G	A	A	FANCD2	c.211G>A	p.D71N
118E7477A1	X	70617240	70617240	Missense_Mutation	SNP	C	T	T	TAF1	c.3604C>T	p.R1202C
118E7484A1	1	155880444	155880444	Splice_Site	SNP	T	A	A	RIT1	c.106+3A>T	-
118E7484A1	1	158582656	158582656	Missense_Mutation	SNP	T	A	A	SPTA1	c.7085A>T	p.Q2362L
118E7484A1	13	32912396	32912396	Missense_Mutation	SNP	A	G	G	BRCA2	c.3904A>G	p.T1302A
118E7484A1	13	48923156	48923156	Nonsense_Mutation	SNP	A	T	T	RB1	c.604A>T	p.K202*
118E7484A1	14	45628487	45628487	Splice_Site	SNP	A	T	T	FANCM	c.1581+4A>T	-
118E7484A1	14	45644605	45644605	Missense_Mutation	SNP	A	T	T	FANCM	c.2648A>T	p.N883I
118E7484A1		-	-	In_Frame_Del	INS	-	-	-	TP53	c.598_609del	.N200_V203del
118E7484A1	19	2202777	2202777	Missense_Mutation	SNP	A	T	T	DOT1L	c.786A>T	p.E262D
118E7484A1	2	74328674	74328674	Nonsense_Mutation	SNP	G	T	T	TET3	c.4759G>T	p.G1587*
118E7484A1	21	39755484	39755484	Missense_Mutation	SNP	C	G	G	ERG	c.1230G>C	p.K410N
118E7484A1	3	47162071	47162071	Missense_Mutation	SNP	T	A	A	SETD2	c.4055A>T	p.Q1352L
118E7484A1		-	-	Frame_Shift_Del	INS	-	-	-	EPHA3	c.1547del	p.G516Dfs*29
118E7484A1	5	35857107	35857107	Missense_Mutation	SNP	A	T	T	IL7R	c.28A>T	p.M10L
118E7484A1	5	131972826	131972826	Missense_Mutation	SNP	A	T	T	RAD50	c.3409A>T	p.S1137C
118E7484A1	5	176523136	176523136	Missense_Mutation	SNP	G	T	T	FGFR4	c.1900G>T	p.A634S
118E7484A1	6	93967207	93967207	Missense_Mutation	SNP	T	A	A	EPHA7	c.2145A>T	p.E715D
118E7484A1	8	48766731	48766731	Missense_Mutation	SNP	T	A	A	PRKDC	c.6805A>T	p.N2269Y
118E7554A2		-	-	In_Frame_Del	INS	-	-	-	TP53	.723_735delins	.C242_G245del
118E7554A2	17	29665110	29665110	Nonsense_Mutation	SNP	C	T	T	NF1	c.6772C>T	p.R2258*
118E7554A2	22	42525787	42525787	Missense_Mutation	SNP	G	A	A	CYP2D6	c.305C>T	p.P102L
118E7554A2	3	134911420	134911420	Missense_Mutation	SNP	G	A	A	EPHB1	c.1885G>A	p.E629K
118E7554A2	3	142242884	142242884	Missense_Mutation	SNP	C	T	T	ATR	c.4103G>A	p.R1368Q
118E7554A2	3	181430278	181430278	Missense_Mutation	SNP	C	A	A	SOX2	c.130C>A	p.P44T
118E7554A2	8	139180187	139180187	Missense_Mutation	SNP	G	T	T	FAM135B	c.1209C>A	p.N403K
118E7554A2	X	70597003	70597003	Missense_Mutation	SNP	A	T	T	TAF1	c.736A>T	p.S246C
118E7554A2	X	129148828	129148828	Missense_Mutation	SNP	G	A	A	BCORL1	c.2080G>A	p.V694M
118E7614A1		-	-	Frame_Shift_Ins	INS	-	-	-	ARID1A	c.3027dup	p.K1010Qfs*4
118E7614A1	16	3778882	3778882	Missense_Mutation	SNP	C	T	T	CREBBP	c.6166G>A	p.V2056M
118E7614A1	3	30732969	30732969	Missense_Mutation	SNP	C	T	T	TGFBR2	c.1582C>T	p.R528C
118E7614A1		-	-	Frame_Shift_Del	INS	-	-	-	PBRM1	c.1335del	p.D446Ifs*7
118E7614A1		-	-	Frame_Shift_Del	INS	-	-	-	MAP3K1	c.1053_1063del	p.G352Yfs*29
118E7614A1		-	-	Frame_Shift_Ins	INS	-	-	-	ARID1B	c.3438dup	p.Q1147Sfs*7
118E7614A1	X	47044967	47044967	Nonsense_Mutation	SNP	C	T	T	RBM10	c.2293C>T	p.R765*

118E7632A1	1	7798423	7798423	Missense_Mutation	SNP	C	T	T	CAMTA1	c.4063C>T	p.P1355S
118E7632A1	1	16259091	16259091	Missense_Mutation	SNP	T	C	C	SPEN	c.6356T>C	p.V2119A
118E7632A1	1	156844702	156844702	Missense_Mutation	SNP	C	T	T	NTRK1	c.1238C>T	p.S413L
118E7632A1	10	70446106	70446106	Splice_Site	SNP	C	G	G	TET1	c.5053-7C>G	-
118E7632A1	12	25398285	25398285	Missense_Mutation	SNP	C	T	T	KRAS	c.34G>A	p.G12S
118E7632A1	12	49428067	49428067	Missense_Mutation	SNP	C	T	T	KMT2D	c.10523G>A	p.R3508Q
118E7632A1	15	99456264	99456264	Splice_Site	SNP	G	A	A	IGF1R	c.1590-9G>A	-
118E7632A1	17	29587401	29587401	Missense_Mutation	SNP	T	C	C	NF1	c.4445T>C	p.I1482T
118E7632A1	2	170063706	170063706	Missense_Mutation	SNP	C	T	T	LRP2	c.6524G>A	p.R2175Q
118E7632A1	2	198257053	198257053	Missense_Mutation	SNP	G	A	A	SF3B1	c.3889C>T	p.R1297C
118E7632A1	3	52677328	52677328	Missense_Mutation	SNP	T	C	C	PBRM1	c.931A>G	p.T311A
118E7632A1	4	55131089	55131089	Missense_Mutation	SNP	C	T	T	PDGFRA	c.632C>T	p.T211I
118E7632A1		-	-	In_Frame_Ins	INS	-	-	-	KIT	c.1712_1741dup	p.I571_H580dup
118E7632A1	4	153249385	153249385	Missense_Mutation	SNP	G	A	A	FBXW7	c.1393C>T	p.R465C
118E7632A1	5	112174282	112174282	Nonsense_Mutation	SNP	T	G	G	APC	c.2991T>G	p.Y997*
118E7632A1	6	117681126	117681126	Missense_Mutation	SNP	A	T	T	ROS1	c.3494T>A	p.V1165D
118E7632A1	X	47039404	47039404	Missense_Mutation	SNP	C	T	T	RBM10	c.1027C>T	p.R343C
118E7633A1	1	46740370	46740370	Missense_Mutation	SNP	A	G	G	RAD54L	c.1850A>G	p.Y617C
118E7633A1	1	59247890	59247890	Missense_Mutation	SNP	T	G	G	JUN	c.853A>C	p.K285Q
118E7633A1	11	60233587	60233587	Missense_Mutation	SNP	C	T	T	MS4A1	c.530C>T	p.S177F
118E7633A1	13	48881502	48881502	Nonsense_Mutation	SNP	G	A	A	RB1	c.224G>A	p.W75*
118E7633A1		-	-	Frame_Shift_Ins	INS	-	-	-	JAK3	c.2362dup	p.L788Pfs*13
118E7633A1	19	52715937	52715937	Splice_Site	SNP	A	G	G	PPP2R1A	c.504-2A>G	-
118E7633A1	4	55152030	55152030	Missense_Mutation	SNP	C	T	T	PDGFRA	c.2462C>T	p.A821V
118E7633A1	4	187557957	187557957	Missense_Mutation	SNP	T	C	C	FAT1	c.3754A>G	p.I1252V
118E7633A1	6	112035608	112035608	Missense_Mutation	SNP	G	A	A	FYN	c.286C>T	p.R96W
118E7633A1	X	48650564	48650564	Missense_Mutation	SNP	C	G	G	GATA1	c.534C>G	p.S178R
118E7744A1	12	25398284	25398284	Missense_Mutation	SNP	C	A	A	KRAS	c.35G>T	p.G12V
118E7744A1	17	7578457	7578457	Missense_Mutation	SNP	C	G	G	TP53	c.473G>C	p.R158P
118E7744A1	18	18622670	18622670	Missense_Mutation	SNP	C	T	T	ROCK1	c.676G>A	p.E226K
118E7805A1	16	9857071	9857071	Missense_Mutation	SNP	C	A	A	GRIN2A	c.4330G>T	p.V1444F
118E7805A1	17	7577121	7577121	Missense_Mutation	SNP	G	T	T	TP53	c.817C>A	p.R273S
118E7805A1	17	29677251	29677251	Nonsense_Mutation	SNP	A	T	T	NF1	c.7372A>T	p.R2458*
118E7805A1	17	38555286	38555286	Splice_Site	SNP	G	T	T	TOP2A	c.3288+6C>A	-
118E7805A1	17	74733439	74733439	Splice_Site	SNP	G	A	A	SRSF2	c.-197C>T	-
118E7805A1	6	32170300	32170300	Missense_Mutation	SNP	A	T	T	NOTCH4	c.3308T>A	p.L1103Q
118E7805A1	8	90983389	90983389	Splice_Site	SNP	T	A	A	NBN	c.702+12A>T	-
118E7868A1	12	25398281	25398281	Missense_Mutation	SNP	C	T	T	KRAS	c.38G>A	p.G13D
118E7868A1	17	7574003	7574003	Nonsense_Mutation	SNP	G	A	A	TP53	c.1024C>T	p.R342*
118E7868A1		-	-	Frame_Shift_Ins	INS	-	-	-	RBM10	c.931dup	p.S311Kfs*70
118E7888A1	11	22646687	22646687	Missense_Mutation	SNP	G	C	C	FANCF	c.670C>G	p.P224A
118E7888A1	11	76165796	76165796	Missense_Mutation	SNP	G	A	A	EMSY	c.249G>A	p.M83I
118E7888A1	11	92533642	92533642	Missense_Mutation	SNP	G	A	A	FAT3	c.7463G>A	p.G2488E
118E7888A1	12	57605284	57605284	Missense_Mutation	SNP	G	A	A	LRP1	c.13106G>A	p.R4369Q
118E7888A1	12	112926270	112926270	Missense_Mutation	SNP	C	T	T	PTPN11	c.1403C>T	p.T468M
118E7888A1	17	1268292	1268292	Missense_Mutation	SNP	C	G	G	YWHAE	c.125G>C	p.R42T
118E7888A1	17	7577156	7577156	Splice_Site	SNP	C	A	A	TP53	c.783-1G>T	-
118E7888A1	2	141283830	141283830	Missense_Mutation	SNP	C	G	G	LRP1B	c.7852G>C	p.D2618H
118E7888A1	3	178916836	178916836	Missense_Mutation	SNP	C	G	G	PIK3CA	c.223C>G	p.Q75E
118E7888A1	8	93003925	93003925	Nonsense_Mutation	SNP	G	T	T	RUNX1T1	c.822C>A	p.Y274*
118E7901A1	12	25398284	25398284	Missense_Mutation	SNP	C	A	A	KRAS	c.35G>T	p.G12V
118E7926A1	1	6947759	6947759	Missense_Mutation	SNP	C	A	A	CAMTA1	c.280C>A	p.P94T
118E7926A1		-	-	Frame_Shift_Del	INS	-	-	-	EPHA2	C.1379del	P.P460Rfs*33
118E7926A1	1	158612617	158612617	Missense_Mutation	SNP	G	A	A	SPTA1	c.4592C>T	p.A1531V
118E7926A1		-	-	Frame_Shift_Ins	INS	-	-	-	ABL2	c.1500dup	p.G501Rfs*8
118E7926A1	1	202710606	202710606	Missense_Mutation	SNP	C	A	A	KDM5B	c.2942G>T	p.G981V
118E7926A1		-	-	Splice_Site	SNP	-	-	-	ARID2	c.5364-5del	-
118E7926A1	12	49428423	49428423	Missense_Mutation	SNP	C	A	A	KMT2D	c.10382G>T	p.R3461M
118E7926A1		-	-	Frame_Shift_Del	INS	-	-	-	LRP1	C.12750del	P.S4251Lfs*91
118E7926A1	13	28623626	28623626	Missense_Mutation	SNP	G	A	A	FLT3	c.931C>T	p.R311W
118E7926A1	15	99467241	99467241	Missense_Mutation	SNP	G	T	T	IGF1R	c.2622G>T	p.E874D
118E7926A1	16	3778512	3778512	Missense_Mutation	SNP	T	C	C	CREBBP	c.6536A>G	p.N2179S
118E7926A1	16	50783930	50783930	Missense_Mutation	SNP	G	T	T	CYLD	c.321G>T	p.E107D
118E7926A1	17	7578406	7578406	Missense_Mutation	SNP	C	T	T	TP53	c.524G>A	p.R175H
118E7926A1		-	-	Frame_Shift_Del	INS	-	-	-	TP53	C.102del	P.L35Cfs*9
118E7926A1		-	-	Frame_Shift_Del	INS	-	-	-	NF1	C.2033del	P.P678Rfs*10
118E7926A1	17	78936362	78936362	Missense_Mutation	SNP	C	T	T	RPTOR	c.3794C>T	p.A1265V
118E7926A1	18	48575185	48575185	Missense_Mutation	SNP	T	C	C	SMAD4	c.379T>C	p.C127R
118E7926A1	19	30308407	30308407	Nonsense_Mutation	SNP	C	T	T	CCNE1	c.421C>T	p.Q141*
118E7926A1		-	-	Splice_Site	SNP	-	-	-	RANBP2	c.141-4dup	-
118E7926A1	2	121743882	121743882	Missense_Mutation	SNP	C	T	T	GLI2	c.1985C>T	p.S662L
118E7926A1	2	209113113	209113113	Missense_Mutation	SNP	G	A	A	IDH1	c.394C>T	p.R132C
118E7926A1	20	31023957	31023957	Missense_Mutation	SNP	C	T	T	ASXL1	c.3442C>T	p.R1148C
118E7926A1	20	57484420	57484420	Missense_Mutation	SNP	C	A	A	GNAS	c.601C>A	p.R201S
118E7926A1		-	-	Frame_Shift_Ins	INS	-	-	-	PBRM1	C.773dup	P.N258Kfs*6
118E7926A1	3	142269129	142269129	Missense_Mutation	SNP	G	T	T	ATR	c.2821C>A	p.L941I
118E7926A1		-	-	Frame_Shift_Del	INS	-	-	-	PRKCI	C.826del	P.T276Qfs*7
118E7926A1		-	-	Frame_Shift_Ins	INS	-	-	-	MAP3K13	c.1603dup	p.R535Kfs*45
118E7926A1		-	-	Splice_Site	SNP	-	-	-	SLIT2	c.1439-7del	-
118E7926A1	5	149513557	149513557	Missense_Mutation	SNP	C	T	T	PDGFRB	c.646G>A	p.V216I
118E7926A1	7	18684405	18684405	Missense_Mutation	SNP	T	A	A	HDAC9	c.1015T>A	p.S339T
118E7926A1	8	68864646	68864646	Missense_Mutation	SNP	G	A	A	PREX2	c.17G>A	p.R6H
118E7940A1	16	89851304	89851304	Missense_Mutation	SNP	G	C	C	FANCA	c.1428C>G	p.F476L
118E7940A1	17	15995380	15995380	Splice_Site	SNP	A	G	G	NCOR1	c.2821-8T>C	-
118E7940A1	19	41727824	41727824	Missense_Mutation	SNP	T	C	C	AXL	c.449T>C	p.V150A
118E7940A1	9	133760532	133760532	Missense_Mutation	SNP	G	A	A	ABL1	c.2912G>A	p.G971E
118E7964A1	10	89725215	89725215	Missense_Mutation	SNP	A	C	C	PTEN	c.1198A>C	p.I400L
118E7964A1		-	-	Frame_Shift_Del	INS	-	-	-	KMT2A	c.2641_2642del	p.E881Kfs*4
118E7964A1	12	133218777	133218777	Missense_Mutation	SNP	C	A	A	POLE	c.5159G>T	p.G1720V
118E7964A1		-	-	Splice_Site	SNP	-	-	-	NUP93	.2349_2349+1d	-
118E7964A1	17	7577581	7577581	Missense_Mutation	SNP	A	G	G	TP53	c.700T>C	p.Y234H
118E7964A1		-	-	Frame_Shift_Del	INS	-	-	-	GNA13	c.134_135del	p.V45Efs*56
118E7964A1		-	-	Frame_Shift_Del	INS	-	-	-	SMAD4	c.1558_1561del	p.E520Hfs*16
118E7964A1	19	10610224	10610224	Nonsense_Mutation	SNP	G	C	C	KEAP1	c.486C>G	p.Y162*
118E7964A1	2	198266713	198266713	Missense_Mutation	SNP	C	A	A	SF3B1	c.2219G>T	p.G740V
118E7964A1		-	-	Missense_Mutation	SNP	-	-	-	EP300	c.4357_4359del	p.P1453del
118E7964A1	3	134967244	134967244	Missense_Mutation	SNP	G	C	C	EPHB1	2583G>C;2590G	.[W861C;D864H]
118E7964A1	3	185774956	185774956	Missense_Mutation	SNP	A	G	G	ETV5	c.1117T>C	p.F373L

118E7964A1		-	-	Frame_Shift_Del	INS	-	-	-	SDHA	c.1583_1584del	p.V528Gfs*74
118E7964A1		-	-	Frame_Shift_Del	INS	-	-	-	APC	c.7724_7725del	p.S2575Cfs*7
118E7964A1		-	-	Frame_Shift_Del	INS	-	-	-	NSD1	c.3539_3540del	p.K1180Rfs*4
118E7964A1	6	41904310	41904310	Missense_Mutation	SNP	G	A	A	CCND3	c.698C>T	p.T233I
118E7964A1		-	-	Frame_Shift_Del	INS	-	-	-	KMT2C	c.3176_3177del	p.H1059Lfs*11
118E7964A1		-	-	Frame_Shift_Del	INS	-	-	-	CDKN2A	C.47_50del	P.L16Pfs*9
118E7982A2	1	8074337	8074337	Missense_Mutation	SNP	C	T	T	ERRFI1	c.322G>A	p.E108K
118E7982A2	1	110466159	110466159	Missense_Mutation	SNP	G	A	A	CSF1	c.916G>A	p.E306K
118E7982A2	11	118348894	118348894	Missense_Mutation	SNP	A	C	C	KMT2A	3547A>C;3554A	N1183H;K1185M]
118E7982A2		-	-	Frame_Shift_Del	INS	-	-	-	RB1	c.1252del	p.R418Efs*2
118E7982A2	14	45639831	45639831	Missense_Mutation	SNP	C	T	T	FANCM	c.2042C>T	p.S681L
118E7982A2	17	29664885	29664885	Missense_Mutation	SNP	G	A	A	NF1	c.6691G>A	p.E2231K
118E7982A2		-	-	Frame_Shift_Ins	INS	-	-	-	MSH6	c.3261dup	p.F1088Lfs*5
118E7982A2		-	-	Frame_Shift_Del	INS	-	-	-	ACVR2A	c.338del	p.K113Sfs*37
118E7982A2	4	126242661	126242661	Missense_Mutation	SNP	G	C	C	FAT4	c.5095G>C	p.E1699Q
118E7982A2	4	153245404	153245404	Missense_Mutation	SNP	G	A	A	FBXW7	c.1787C>T	p.S596F
118E7982A2		-	-	Missense_Mutation	SNP	-	-	-	FBXW7	c.1423_1425del	p.V475del
118E7982A2	6	36652071	36652071	Missense_Mutation	SNP	T	C	C	CDKN1A	c.193T>C	p.W65R
118E7982A2	8	141727804	141727804	Missense_Mutation	SNP	T	C	C	PTK2	c.2101A>G	p.I701V
118F7997A1	1	120471692	120471692	Missense_Mutation	SNP	C	T	T	NOTCH2	c.3799G>A	p.E1267K
118F7997A1	11	108235809	108235809	Missense_Mutation	SNP	G	C	C	ATM	c.8851G>C	p.V2951L
118F7997A1	12	25398284	25398284	Missense_Mutation	SNP	C	G	G	KRAS	c.35G>C	p.G12A
118F7997A1	2	141264356	141264356	Splice_Site	SNP	T	G	G	LRP1B	c.8521+9A>C	-
118F7997A1	3	142261571	142261571	Missense_Mutation	SNP	C	G	G	ATR	c.3386G>C	p.G1129A
118F8082A1	12	25398281	25398281	Missense_Mutation	SNP	C	T	T	KRAS	c.38G>A	p.G13D
118F8082A1	17	7577058	7577058	Nonsense_Mutation	SNP	C	A	A	TP53	c.880G>T	p.E294*
118F8082A1		-	-	Frame_Shift_Ins	INS	-	-	-	SMAD4	c.221dup	p.Q75Tfs*29
118F8090A1	1	9784994	9784994	Missense_Mutation	SNP	G	A	A	PIK3CD	c.2997G>A	p.K999K
118F8090A1	12	4409117	4409117	Missense_Mutation	SNP	C	T	T	CCND2	c.812C>T	p.S271L
118F8090A1	12	25398284	25398284	Missense_Mutation	SNP	C	T	T	KRAS	c.35G>A	p.G12D
118F8090A1	17	7577551	7577551	Missense_Mutation	SNP	C	A	A	TP53	c.730G>T	p.G244C
118F8090A1	18	48593558	48593558	Splice_Site	SNP	G	T	T	SMAD4	c.1308+1G>T	-
118F8094A1	12	25398285	25398285	Missense_Mutation	SNP	C	G	G	KRAS	c.34G>C	p.G12R
118F8094A1	13	29005422	29005422	Missense_Mutation	SNP	C	T	T	FLT1	c.839G>A	p.R280Q
118F8094A1	17	7578406	7578406	Missense_Mutation	SNP	C	T	T	TP53	c.524G>A	p.R175H
118F8094A1	7	151949114	151949114	Nonsense_Mutation	SNP	C	A	A	KMT2C	c.1531G>T	p.E511*
118F8094A1	9	21971077	21971077	Missense_Mutation	SNP	A	T	T	CDKN2A	c.281T>A	p.L94Q
118F8096A1		-	-	Frame_Shift_Ins	INS	-	-	-	TP53	C.750dup	P.I251Hfs*13
118F8096A1		-	-	Frame_Shift_Del	INS	-	-	-	TGFBR1	c.1347_1350del	p.L450Gfs*14
118F8114A1		-	-	Missense_Mutation	SNP	-	-	-	TP53	c.321_323del	p.G108del
118F8114A1		-	-	Missense_Mutation	SNP	-	-	-	EPHA5	948_2949delins	p.T983N
118F8114A1		-	-	Frame_Shift_Ins	INS	-	-	-	FBXW7	c.1463_1466dup	p.E489Dfs*2
118F8114A1	6	32188062	32188062	Splice_Site	SNP	C	T	T	NOTCH4	c.1160-1G>A	-
118F8114A1	8	128751112	128751112	Missense_Mutation	SNP	A	G	G	MYC	c.649A>G	p.S217G
118F8152A1	12	52374741	52374741	Splice_Site	SNP	T	C	C	ACVR1B	c.581-12T>C	-
118F8152A1	3	37067240	37067240	Missense_Mutation	SNP	T	A	A	MLH1	c.1151T>A	p.V384D
118F8152A1	3	89390226	89390226	Splice_Site	SNP	G	A	A	EPHA3	c.970+5G>A	-
118F8152A1		-	-	Frame_Shift_Del	INS	-	-	-	CDKN2A	C.47_50del	P.L16Pfs*9
118F8157A1	11	108128226	108128226	Nonsense_Mutation	SNP	G	T	T	ATM	c.2269G>T	p.G757*
118F8157A1	17	16004563	16004563	Splice_Site	SNP	C	A	A	NCOR1	c.2690+1G>T	-
118F8167A1	3	30713760	30713760	Missense_Mutation	SNP	A	G	G	TGFBR2	c.1085A>G	p.H362R
118F8167A1		-	-	Frame_Shift_Del	INS	-	-	-	BAP1	c.421del	p.H141Ifs*46
118F8167A1	7	77789586	77789586	Missense_Mutation	SNP	C	A	A	MAGI2	c.2601G>T	p.E867D
118F8170A1		-	-	Frame_Shift_Ins	INS	-	-	-	TP53	c.131_132insA	p.M44Ifs*8
118F8170A1	17	56435317	56435317	Missense_Mutation	SNP	G	A	A	RNF43	c.1820C>T	p.S607L
118F8170A1	19	10610618	10610618	Missense_Mutation	SNP	A	G	G	KEAP1	c.92T>C	p.V31A
118F8170A1	2	170068570	170068570	Missense_Mutation	SNP	G	T	T	LRP2	c.6188C>A	p.S2063Y
118F8170A1		-	-	Frame_Shift_Del	INS	-	-	-	BAP1	c.2129_2142del	p.H710Pfs*2
118F8170A1		-	-	In_Frame_Del	INS	-	-	-	PRKDC	c.11736del	p.C3912*
118F8189A1	17	7578557	7578557	Splice_Site	SNP	G	C	C	TP53	c.376-3C>G	-
118F8201A1	20	57485805	57485805	Missense_Mutation	SNP	C	T	T	GNAS	c.1106C>T	p.T369I
118F8201A1		-	-	Frame_Shift_Del	INS	-	-	-	BAP1	c.97_106del	p.Y33Rfs*36
118F8208A1	11	92086588	92086588	Missense_Mutation	SNP	C	A	A	FAT3	c.1310C>A	p.T437N
118F8208A1	11	108196235	108196235	Missense_Mutation	SNP	A	C	C	ATM	c.6771A>C	p.E2257D
118F8208A1	12	25398284	25398284	Missense_Mutation	SNP	C	T	T	KRAS	c.35G>A	p.G12D
118F8208A1	17	7577094	7577094	Missense_Mutation	SNP	G	A	A	TP53	c.844C>T	p.R282W
118F8208A1		-	-	In_Frame_Ins	INS	-	-	-	ZNF703	c.58_69dup	p.S20_G23dup
118F8237A1	1	158584994	158584994	Splice_Site	SNP	C	T	T	SPTA1	c.6788+12G>A	-
118F8237A1	12	25398281	25398281	Missense_Mutation	SNP	C	T	T	KRAS	c.38G>A	p.G13D
118F8237A1	17	7578406	7578406	Missense_Mutation	SNP	C	T	T	TP53	c.524G>A	p.R175H
118F8237A1	18	48593505	48593505	Missense_Mutation	SNP	G	C	C	SMAD4	c.1256G>C	p.G419A
118F8237A1	19	1220649	1220649	Missense_Mutation	SNP	G	A	A	STK11	c.667G>A	p.E223K
118F8237A1	2	61706035	61706035	Missense_Mutation	SNP	C	T	T	XPO1	c.3136G>A	p.D1046N
118F8237A1	22	29092948	29092948	Missense_Mutation	SNP	G	A	A	CHEK2	c.1036C>T	p.R346C
118F8237A1	3	187446190	187446190	Nonsense_Mutation	SNP	C	A	A	BCL6	c.1498G>T	p.E500*
118F8237A1	5	112162891	112162891	Nonsense_Mutation	SNP	C	T	T	APC	c.1495C>T	p.R499*
118F8276A1	12	25378562	25378562	Missense_Mutation	SNP	C	G	G	KRAS	c.436G>C	p.A146P
118F8276A1	17	7576852	7576852	Splice_Site	SNP	C	T	T	TP53	c.993+1G>A	-
118F8276A1	19	4117549	4117549	Missense_Mutation	SNP	A	C	C	MAP2K2	c.171T>G	p.F57L
118F8276A1	19	15296082	15296082	Missense_Mutation	SNP	G	A	A	NOTCH3	c.2282C>T	p.P761L
118F8276A1	3	30664717	30664717	Missense_Mutation	SNP	G	A	A	TGFBR2	c.121G>A	p.E41K
118F8372A1	1	158606546	158606546	Missense_Mutation	SNP	T	G	G	SPTA1	c.5195A>C	p.K1732T
118F8372A1		-	-	In_Frame_Del	INS	-	-	-	TP53	c.471_518del	p.A159_R174del
118F8372A1	19	11135028	11135028	Missense_Mutation	SNP	G	A	A	SMARCA4	c.2995G>A	p.D999N
118F8372A1		-	-	Splice_Site	SNP	-	-	-	RUNX1	.59-12_59-11de	-
118F8372A1	3	138665289	138665289	Missense_Mutation	SNP	C	G	G	FOXL2	c.276G>C	p.E92D
118F8377A1	12	46287315	46287315	Nonsense_Mutation	SNP	C	T	T	ARID2	c.5260C>T	p.R1754*
118F8377A1	17	7577099	7577099	Missense_Mutation	SNP	C	T	T	TP53	c.839G>A	p.R280K
118F8377A1	18	739820	739820	Splice_Site	SNP	G	A	A	YES1	c.1061-9C>T	-
118F8377A1		-	-	Frame_Shift_Del	INS	-	-	-	KEAP1	c.1626_1627del	p.E542Dfs*31
118F8377A1	19	42796446	42796446	Splice_Site	SNP	C	T	T	CIC	c.3009-6C>T	-
118F8377A1	2	142004783	142004783	Splice_Site	SNP	G	A	A	LRP1B	c.592+12C>T	-
118F8377A1	4	153273928	153273928	Splice_Site	SNP	G	C	C	FBXW7	c.-46C>G	-
118F8377A1	8	71068319	71068319	Missense_Mutation	SNP	C	T	T	NCOA2	c.2281G>A	p.E761K
118F8377A1	9	5126352	5126352	Missense_Mutation	SNP	G	A	A	JAK2	c.3197G>A	p.G1066D
118F8392A1	11	118355614	118355614	Missense_Mutation	SNP	G	T	T	KMT2A	c.4256G>T	p.G1419V
118F8392A1	12	25380275	25380275	Missense_Mutation	SNP	T	G	G	KRAS	c.183A>C	p.Q61H
118F8392A1		-	-	Frame_Shift_Ins	INS	-	-	-	STK11	c.157_158insT	p.D53Vfs*110

118F8415A1	1	27094361	27094361	Nonsense_Mutation	SNP	G	A	A	ARID1A	c.3069G>A	p.W1023*
118F8415A1	12	49444878	49444878	Missense_Mutation	SNP	C	A	A	KMT2D	c.2588G>T	p.R863L
118F8415A1	13	41134822	41134822	Missense_Mutation	SNP	C	T	T	FOXO1	c.806G>A	p.R269Q
118F8415A1	17	7577548	7577548	Missense_Mutation	SNP	C	T	T	TP53	c.733G>A	p.G245S
118F8415A1		-	-	Frame_Shift_Del	INS	-	-	-	SMAD4	c.793del	p.T265Lfs*71
118F8415A1	2	198299719	198299719	Missense_Mutation	SNP	G	A	A	SF3B1	c.5C>T	p.A2V
118F8415A1		-	-	Frame_Shift_Del	INS	-	-	-	PBRM1	c.2581del	p.I861Yfs*6
118F8415A1	7	2987341	2987341	Missense_Mutation	SNP	G	A	A	CARD11	c.88C>T	p.R30W
118F8415A1	7	151845790	151845790	Missense_Mutation	SNP	C	G	G	KMT2C	c.13222G>C	p.E4408Q
118F8458A1	1	158613185	158613185	Missense_Mutation	SNP	C	T	T	SPTA1	c.4369G>A	p.A1457T
118F8458A1	12	465645	465645	Missense_Mutation	SNP	G	A	A	KDM5A	c.731C>T	p.A244V
118F8458A1	12	49422858	49422858	Missense_Mutation	SNP	C	T	T	KMT2D	c.14237G>A	p.R4746Q
118F8458A1		-	-	Frame_Shift_Del	INS	-	-	-	KMT2D	c.5956del	p.T1986Pfs*61
118F8458A1	12	57863295	57863295	Missense_Mutation	SNP	G	T	T	GLI1	c.1267G>T	p.G423C
118F8458A1		-	-	Frame_Shift_Ins	INS	-	-	-	POLE	c.382dup	p.I128Nfs*76
118F8458A1	13	32953610	32953610	Missense_Mutation	SNP	A	G	G	BRCA2	c.8911A>G	p.K2971E
118F8458A1	13	110435825	110435825	Missense_Mutation	SNP	G	A	A	IRS2	c.2576C>T	p.T859M
118F8458A1	15	75093027	75093027	Missense_Mutation	SNP	T	C	C	CSK	c.562T>C	p.W188R
118F8458A1		-	-	Missense_Mutation	SNP	-	-	-	TP53	C.572_574del	P.P191del
118F8458A1		-	-	Frame_Shift_Ins	INS	-	-	-	MAP2K4	c.69dup	p.V24Rfs*17
118F8458A1		-	-	Missense_Mutation	SNP	-	-	-	GATA6	c.134_136del	p.S45del
118F8458A1	19	10610256	10610256	Missense_Mutation	SNP	C	T	T	KEAP1	c.454G>A	p.V152I
118F8458A1	19	15298722	15298722	Missense_Mutation	SNP	G	A	A	NOTCH3	c.1576C>T	p.P526S
118F8458A1		-	-	Splice_Site	SNP	-	-	-	CIC	176-14_3176-12	-
118F8458A1	2	16086115	16086115	Missense_Mutation	SNP	C	T	T	MYCN	c.1291C>T	p.H431Y
118F8458A1		-	-	Frame_Shift_Del	INS	-	-	-	ACVR2A	C.36_39del	P.F12Lfs*61
118F8458A1	2	170060692	170060692	Missense_Mutation	SNP	C	T	T	LRP2	c.7805G>A	p.G2602D
118F8458A1	22	41556665	41556665	Missense_Mutation	SNP	T	C	C	EP300	c.3610T>C	p.C1204R
118F8458A1	3	38182308	38182308	Missense_Mutation	SNP	C	T	T	MYD88	c.428C>T	p.S143F
118F8458A1	3	191888366	191888366	Missense_Mutation	SNP	C	T	T	FGF12	c.308G>A	p.R103H
118F8458A1	5	112176515	112176515	Missense_Mutation	SNP	C	A	A	APC	c.5224C>A	p.R1742S
118F8458A1	5	138269729	138269729	Missense_Mutation	SNP	T	C	C	CTNNA1	c.2672T>C	p.V891A
118F8458A1	6	106536126	106536126	Missense_Mutation	SNP	G	T	T	PRDM1	c.93G>T	p.E31D
118F8458A1	7	41729607	41729607	Missense_Mutation	SNP	G	A	A	INHBA	c.922C>T	p.R308C
118F8458A1		-	-	Frame_Shift_Del	INS	-	-	-	NRG1	c.472del	p.A158Rfs*5
118F8458A1	8	71068513	71068513	Missense_Mutation	SNP	T	C	C	NCOA2	c.2087A>G	p.D696G
118F8458A1	9	93624559	93624559	Missense_Mutation	SNP	G	A	A	SYK	c.650G>A	p.R217H
118F8458A1	9	93639919	93639919	Missense_Mutation	SNP	G	T	T	SYK	c.1179G>T	p.E393D
118F8458A1	9	137300076	137300076	Missense_Mutation	SNP	G	A	A	RXRA	c.361G>A	p.A121T
118F8458A1	X	66943638	66943638	Missense_Mutation	SNP	G	T	T	AR	c.2718G>T	p.K906N
118F8489A1	17	29541460	29541460	Splice_Site	SNP	T	A	A	NF1	c.1393-9T>A	-
118F8522A1		-	-	Frame_Shift_Del	INS	-	-	-	PTEN	c.219_222del	p.E73Dfs*25
118F8522A1		-	-	Splice_Site	SNP	-	-	-	PBRM1	1302-29_1313d	-
118F8522A1	6	41909260	41909260	Missense_Mutation	SNP	G	A	A	CCND3	c.128C>T	p.S43F
118F8522A1	7	151864290	151864290	Missense_Mutation	SNP	C	A	A	KMT2C	c.9691G>T	p.A3231S
118F8546A1	17	7577121	7577121	Missense_Mutation	SNP	G	A	A	TP53	c.817C>T	p.R273C
118F8546A1		-	-	Missense_Mutation	SNP	-	-	-	TP53	c.787_789del	p.N263del
118F8546A1	4	187629400	187629400	Missense_Mutation	SNP	C	T	T	FAT1	c.1582G>A	p.E528K
118F8546A1	7	116412023	116412023	Missense_Mutation	SNP	A	T	T	MET	c.3008A>T	p.Y1003F
118F8558A1	15	88679244	88679244	Missense_Mutation	SNP	C	T	T	NTRK3	c.793G>A	p.A265T
118F8558A1	17	7577518	7577518	Missense_Mutation	SNP	T	A	A	TP53	c.763A>T	p.I255F
118F8558A1	17	56437614	56437614	Splice_Site	SNP	T	C	C	RNF43	c.850-2A>G	-
118F8558A1	5	67591128	67591128	Missense_Mutation	SNP	G	C	C	PIK3R1	1721G>C;1730G	p.[R574T;R577K]
118F8558A1	7	42004379	42004379	Missense_Mutation	SNP	G	A	A	GLI3	c.4292C>T	p.P1431L
118F8558A1	7	140453154	140453154	Missense_Mutation	SNP	T	C	C	BRAF	c.1781A>G	p.D594G
118F8601A1	4	55946274	55946274	Missense_Mutation	SNP	G	A	A	KDR	c.3905C>T	p.T1302I
118F8601A1	9	101904982	101904982	Nonsense_Mutation	SNP	C	T	T	TGFBR1	c.970C>T	p.Q324*
118F8601A1		-	-	Frame_Shift_Del	INS	-	-	-	RBM10	c.1998del	p.E667Kfs*37
118F8632A1	12	49421846	49421846	Nonsense_Mutation	SNP	C	A	A	KMT2D	c.14461G>T	p.E4821*
118F8632A1	12	133250284	133250284	Missense_Mutation	SNP	C	G	G	POLE	c.1236G>C	p.K412N
118F8632A1	17	7578550	7578550	Missense_Mutation	SNP	G	A	A	TP53	c.380C>T	p.S127F
118F8632A1	19	11144113	11144113	Missense_Mutation	SNP	G	T	T	SMARCA4	3694G>T;3695G	p.G1232F
118F8632A1	6	112015871	112015871	Missense_Mutation	SNP	C	G	G	FYN	c.1079G>C	p.R360T
118F8632A1	7	18788726	18788726	Nonsense_Mutation	SNP	C	T	T	HDAC9	c.1999C>T	p.R667*
118F8646A1	12	25398284	25398284	Missense_Mutation	SNP	C	T	T	KRAS	c.35G>A	p.G12D
118F8646A1	13	110435910	110435910	Missense_Mutation	SNP	C	T	T	IRS2	c.2491G>A	p.G831R
118F8646A1		-	-	In_Frame_Ins	INS	-	-	-	SOX9	c.1089_1109dup	p.A371_P377dup
118F8646A1		-	-	Frame_Shift_Del	INS	-	-	-	STK11	c.787_790del	p.F264Rfs*22
118F8646A1	19	15288644	15288644	Missense_Mutation	SNP	C	G	G	NOTCH3	c.4095G>C	p.Q1365H
118F8646A1	5	112178000	112178000	Nonsense_Mutation	SNP	C	T	T	APC	c.6709C>T	p.R2237*
118F8770A1	11	118361941	118361941	Missense_Mutation	SNP	A	G	G	KMT2A	c.4727A>G	p.Y1576C
118F8770A1	12	25398284	25398284	Missense_Mutation	SNP	C	A	A	KRAS	c.35G>T	p.G12V
118F8770A1	12	57598946	57598946	Missense_Mutation	SNP	G	A	A	LRP1	c.11249G>A	p.R3750Q
118F8792A1		-	-	Missense_Mutation	SNP	-	-	-	FGFR2	c.1635_1637del	p.K545del
118F8792A1	3	10114944	10114944	Missense_Mutation	SNP	A	C	C	FANCD2	c.2613A>C	p.K871N
118F8792A1	3	52441430	52441430	Missense_Mutation	SNP	T	C	C	BAP1	c.422A>G	p.H141R
118F8797A1	10	89720649	89720649	Splice_Site	SNP	A	G	G	PTEN	c.802-2A>G	-
118F8797A1	13	48934234	48934234	Nonsense_Mutation	SNP	C	A	A	RB1	c.689C>A	p.S230*
118F8797A1	14	45645216	45645216	Nonsense_Mutation	SNP	A	T	T	FANCM	c.3259A>T	p.K1087*
118F8797A1	17	7577120	7577120	Missense_Mutation	SNP	C	T	T	TP53	c.818G>A	p.R273H
118F8797A1	17	29562760	29562760	Missense_Mutation	SNP	G	T	T	NF1	c.3840G>T	p.L1280F
118F8797A1	2	25523034	25523034	Missense_Mutation	SNP	G	A	A	DNMT3A	c.151C>T	p.P51S
118F8797A1	4	1953960	1953960	Splice_Site	SNP	C	T	T	NSD2	c.2137+2C>T	-
118F8797A1		-	-	Frame_Shift_Del	INS	-	-	-	RICTOR	c.3655del	p.S1219Vfs*11
118F8797A1	6	93974325	93974325	Missense_Mutation	SNP	T	A	A	EPHA7	c.1729A>T	p.I577F
118F8797A1	8	48767810	48767810	Nonsense_Mutation	SNP				PRKDC	c.6733A>T	p.K2245*
118F8797A1	X	129150082	129150082	Nonsense_Mutation	SNP	G	T	T	BCORL1	c.3334G>T	p.E1112*
118F8813A2		-	-	Frame_Shift_Del	INS	-	-	-	ARID1A	c.229del	p.A77Pfs*24
118F8813A2	11	92534248	92534248	Missense_Mutation	SNP	C	T	T	FAT3	c.8069C>T	p.S2690F
118F8813A2	12	46123909	46123909	Nonsense_Mutation	SNP	G	T	T	ARID2	c.175G>T	p.G59*
118F8813A2	17	7578440	7578440	Nonsense_Mutation	SNP	T	A	A	TP53	c.490A>T	p.K164*
118F8813A2	18	48575209	48575209	Nonsense_Mutation	SNP	C	T	T	SMAD4	c.403C>T	p.R135*
118F8813A2	3	49397721	49397721	Missense_Mutation	SNP	C	G	G	RHOA	c.503G>C	p.R168T
118F8813A2	3	49412969	49412969	Missense_Mutation	SNP	C	A	A	RHOA	c.54G>T	p.K18N
118F8813A2	3	191888403	191888403	Nonsense_Mutation	SNP	C	A	A	FGF12	c.271G>T	p.E91*
118F8813A2	5	161116166	161116166	Missense_Mutation	SNP	A	T	T	GABRA6	c.437A>T	p.Y146F
118F8813A2	8	32472087	32472087	Splice_Site	SNP	G	T	T	NRG1	c.451+5G>T	-
118F8844A1	1	16475389	16475389	Missense_Mutation	SNP	G	A	A	EPHA2	c.307C>T	p.R103C

118F8844A1	12	46246290	46246290	Nonsense_Mutation	SNP	C	T	T	ARID2	c.4384C>T	p.Q1462*
118F8844A1	12	57559938	57559938	Missense_Mutation	SNP	G	A	A	LRP1	c.2743G>A	p.D915N
118F8844A1	16	9943686	9943686	Missense_Mutation	SNP	C	T	T	GRIN2A	c.1255G>A	p.V419M
118F8844A1	17	38556655	38556655	Missense_Mutation	SNP	T	C	C	TOP2A	c.2806A>G	p.K936E
118F8844A1	3	178952074	178952074	Missense_Mutation	SNP	G	A	A	PIK3CA	c.3129G>A	p.M1043I
118F8844A1	5	225600	225600	Missense_Mutation	SNP	G	A	A	SDHA	c.379G>A	p.V127M
118F8844A1	5	176707786	176707786	Missense_Mutation	SNP	G	A	A	NSD1	c.5843G>A	p.R1948H
118F8850A1	1	16256981	16256981	Missense_Mutation	SNP	C	T	T	SPEN	c.4246C>T	p.R1416C
118F8850A1		-	-	Frame_Shift_Del	INS	-	-	-	ARID1A	C.827del	P.G276Efs*87
118F8850A1	1	43777462	43777462	Missense_Mutation	SNP	G	A	A	TIE1	c.1454G>A	p.R485Q
118F8850A1	1	162722894	162722894	Missense_Mutation	SNP	G	A	A	DDR2	c.92G>A	p.R31H
118F8850A1	1	202718170	202718170	Missense_Mutation	SNP	G	A	A	KDM5B	c.2027C>T	p.A676V
118F8850A1	10	8100645	8100645	Missense_Mutation	SNP	G	A	A	GATA3	c.619G>A	p.A207T
118F8850A1	11	92532114	92532114	Missense_Mutation	SNP	G	A	A	FAT3	c.5935G>A	p.G1979S
118F8850A1	11	108115753	108115753	Missense_Mutation	SNP	G	A	A	ATM	c.901G>A	p.G301S
118F8850A1	11	118365088	118365088	Missense_Mutation	SNP	G	A	A	KMT2A	c.5264G>A	p.S1755N
118F8850A1		-	-	Frame_Shift_Del	INS	-	-	-	KMT2D	C.7548del	P.N2517Ifs*26
118F8850A1		-	-	Frame_Shift_Ins	INS	-	-	-	LRP1	C.12575dup	P.D4193Rfs*9
118F8850A1	12	133245212	133245212	Splice_Site	SNP	G	A	A	POLE	c.2026+9C>T	-
118F8850A1	13	110436318	110436318	Missense_Mutation	SNP	C	T	T	IRS2	c.2083G>A	p.A695T
118F8850A1	15	88576153	88576153	Missense_Mutation	SNP	C	T	T	NTRK3	c.1520G>A	p.R507H
118F8850A1		-	-	Frame_Shift_Del	INS	-	-	-	CHD2	C.4173del	P.K1391Nfs*15
118F8850A1	16	9858165	9858165	Missense_Mutation	SNP	T	C	C	GRIN2A	c.3236A>G	p.N1079S
118F8850A1		-	-	Frame_Shift_Ins	INS	-	-	-	NF1	C.2033dup	P.I679Dfs*21
118F8850A1		-	-	Frame_Shift_Del	INS	-	-	-	NF1	C.7626_7627de	P.S2543Ffs*2
118F8850A1	17	37864598	37864598	Missense_Mutation	SNP	G	A	A	ERBB2	c.250G>A	p.V84M
118F8850A1	17	41267752	41267752	Missense_Mutation	SNP	A	T	T	BRCA1	[125T>A;127T>	p.[I42K;F43I]
118F8850A1	17	80789063	80789063	Missense_Mutation	SNP	G	A	A	ZNF750	c.1268C>T	p.T423M
118F8850A1	18	42643239	42643239	Missense_Mutation	SNP	G	A	A	SETBP1	c.4367G>A	p.R1456H
118F8850A1	19	15375253	15375253	Missense_Mutation	SNP	C	T	T	BRD4	c.1174G>A	p.D392N
118F8850A1	19	40746008	40746008	Missense_Mutation	SNP	C	T	T	AKT2	c.583G>A	p.A195T
118F8850A1	19	50905635	50905635	Splice_Site	SNP	G	A	A	POLD1	c.758+5G>A	-
118F8850A1		-	-	Frame_Shift_Del	INS	-	-	-	MSH2	c.473_474del	p.R159Tfs*18
118F8850A1		-	-	Frame_Shift_Ins	INS	-	-	-	TET3	C.5243dup	P.T1749Hfs*5
118F8850A1	2	121746027	121746027	Missense_Mutation	SNP	C	T	T	GLI2	c.2537C>T	p.P846L
118F8850A1	2	202150030	202150030	Nonsense_Mutation	SNP	C	T	T	CASP8	c.1345C>T	p.R449*
118F8850A1		-	-	Frame_Shift_Ins	INS	-	-	-	CASP8	C.1470dup	P.L491Tfs*6
118F8850A1		-	-	Frame_Shift_Ins	INS	-	-	-	ASXL1	C.1934dup	P.G646Wfs*12
118F8850A1		-	-	Frame_Shift_Del	INS	-	-	-	TGFBR2	c.717_718del	p.E240Afs*5
118F8850A1		-	-	Frame_Shift_Del	INS	-	-	-	PBRM1	C.2727del	P.K909Nfs*6
118F8850A1	3	170015091	170015091	Splice_Site	SNP	G	T	T	PRKCI	c.1498-1G>T	-
118F8850A1	5	1279521	1279521	Missense_Mutation	SNP	C	T	T	TERT	c.2015G>A	p.R672H
118F8850A1		-	-	Frame_Shift_Del	INS	-	-	-	APC	C.1742del	P.K581Rfs*9
118F8850A1	7	18975437	18975437	Missense_Mutation	SNP	G	A	A	HDAC9	c.2800G>A	p.G934S
118F8850A1	7	117250697	117250697	Missense_Mutation	SNP	A	G	G	CFTR	c.3113A>G	p.Q1038R
118F8850A1	7	151873322	151873322	Missense_Mutation	SNP	C	T	T	KMT2C	c.9216G>A	p.M3072I
118F8850A1	8	11607696	11607696	Missense_Mutation	SNP	A	G	G	GATA4	c.860A>G	p.E287G
118F8850A1	8	31498051	31498051	Missense_Mutation	SNP	G	A	A	NRG1	c.551G>A	p.R184H
118F8850A1	8	32505822	32505822	Missense_Mutation	SNP	C	T	T	NRG1	c.586C>T	p.R196W
118F8850A1	8	41790777	41790777	Missense_Mutation	SNP	G	A	A	KAT6A	c.4961C>T	p.P1654L
118F8850A1	8	42220198	42220198	Missense_Mutation	SNP	G	T	T	POLB	c.690G>T	p.K230N
118F8850A1	8	95419766	95419766	Missense_Mutation	SNP	A	G	G	RAD54B	c.682T>C	p.F228L
118F8850A1	9	21971120	21971120	Nonsense_Mutation	SNP	G	A	A	CDKN2A	c.238C>T	p.R80*
118F8850A1		-	-	Frame_Shift_Del	INS	-	-	-	TSC1	C.1808del	P.P603Rfs*26
118F8850A1	9	139404203	139404203	Missense_Mutation	SNP	G	A	A	NOTCH1	c.2951C>T	p.T984M
118F8850A1	X	47426763	47426763	Missense_Mutation	SNP	G	T	T	ARAF	c.1008G>T	p.K336N
118F8903A1	1	27101510	27101510	Missense_Mutation	SNP	C	T	T	ARID1A	c.4792C>T	p.R1598C
118F8903A1	12	56489567	56489567	Missense_Mutation	SNP	A	T	T	ERBB3	c.2032A>T	p.R678W
118F8903A1	15	88576199	88576199	Missense_Mutation	SNP	A	G	G	NTRK3	c.1474T>C	p.S492P
118F8903A1	17	7577121	7577121	Missense_Mutation	SNP	G	A	A	TP53	c.817C>T	p.R273C
118F8903A1	19	1207162	1207162	Nonsense_Mutation	SNP	A	T	T	STK11	c.250A>T	p.K84*
118F8903A1	19	10610285	10610285	Missense_Mutation	SNP	G	A	A	KEAP1	c.425C>T	p.T142M
118F8903A1	3	134851763	134851763	Missense_Mutation	SNP	G	A	A	EPHB1	c.1169G>A	p.R390H
118F8903A1	5	112177911	112177911	Nonsense_Mutation	SNP	C	G	G	APC	c.6620C>G	p.S2207*
118F8903A1	5	176517950	176517950	Missense_Mutation	SNP	A	G	G	FGFR4	c.448A>G	p.T150A
118F8903A1	8	48701588	48701588	Nonsense_Mutation	SNP				PRKDC	c.10780G>T	p.E3594*
118G1224A1		-	-	Frame_Shift_Del	INS	-	-	-	ARID1A	c.4750del	p.Q1584Rfs*28
118G1224A1	1	202743853	202743853	Missense_Mutation	SNP	C	T	T	KDM5B	c.293G>A	p.R98H
118G1224A1	12	25398284	25398284	Missense_Mutation	SNP	C	T	T	KRAS	c.35G>A	p.G12D
118G1224A1	17	7578442	7578442	Missense_Mutation	SNP	T	C	C	TP53	c.488A>G	p.Y163C
118G1224A1	22	23634808	23634808	Missense_Mutation	SNP	G	A	A	BCR	c.2863G>A	p.E955K
118G1224A1	5	121761117	121761117	Missense_Mutation	SNP	C	T	T	SNCAIP	c.1073C>T	p.A358V
118G1344A1	1	27101468	27101468	Nonsense_Mutation	SNP	C	T	T	ARID1A	c.4750C>T	p.Q1584*
118G1344A1	12	25398284	25398284	Missense_Mutation	SNP	C	A	A	KRAS	c.35G>T	p.G12V
118G1344A1	15	75093221	75093221	Missense_Mutation	SNP	A	G	G	CSK	c.680A>G	p.D227G
118G1344A1	17	7577538	7577538	Missense_Mutation	SNP	C	T	T	TP53	c.743G>A	p.R248Q
118G1344A1	X	44913082	44913082	Missense_Mutation	SNP	C	T	T	KDM6A	c.757C>T	p.H253Y
118G1348A1	10	89717708	89717708	Nonsense_Mutation	SNP	C	T	T	PTEN	c.733C>T	p.Q245*
118G1348A1		-	-	Frame_Shift_Del	INS	-	-	-	TP53	C.626_627del	P.R209Kfs*6
118G1348A1	4	187539330	187539330	Missense_Mutation	SNP	C	T	T	FAT1	c.8410G>A	p.D2804N
118G8977A1	12	49425544	49425544	Missense_Mutation	SNP	T	C	C	KMT2D	c.12944A>G	p.Q4315R
118G8977A1	17	7577609	7577609	Splice_Site	SNP	C	T	T	TP53	c.673-1G>A	-
118G9081A1	1	11190804	11190804	Missense_Mutation	SNP	C	T	T	MTOR	c.5395G>A	p.E1799K
118G9081A1		-	-	Frame_Shift_Del	INS	-	-	-	EPHA2	c.2459del	p.E820Gfs*7
118G9081A1		-	-	Frame_Shift_Del	INS	-	-	-	ARID1A	4_733delinsAA	p.G242Lfs*120
118G9081A1	12	25380275	25380275	Missense_Mutation	SNP	T	A	A	KRAS	c.183A>T	p.Q61H
118G9081A1	15	67477103	67477103	Missense_Mutation	SNP	T	C	C	SMAD3	c.910T>C	p.F304L
118G9081A1	19	15271517	15271517	Missense_Mutation	SNP	C	A	A	NOTCH3	c.6922G>T	p.G2308W
118G9081A1	3	52595851	52595851	Missense_Mutation	SNP	C	A	A	PBRM1	4064G>T;4076G	[G1355V;R1359L]
118G9081A1	4	175898265	175898265	Missense_Mutation	SNP	G	A	A	ADAM29	c.1589G>A	p.R530H
118G9081A1		-	-	In_Frame_Del	INS	-	-	-	ARID1B	c.3447_3454del	p.F1150*
118G9119A1	1	120510085	120510085	Missense_Mutation	SNP	A	C	C	NOTCH2	c.1424T>G	p.I475S
118G9119A1		-	-	Frame_Shift_Del	INS	-	-	-	BIRC3	3_603delinsGTC	p.Y195Vfs*21
118G9119A1	15	40998493	40998493	Splice_Site	SNP	G	T	T	RAD51	c.343+1G>T	-
118G9119A1	15	90631839	90631839	Missense_Mutation	SNP	T	A	A	IDH2	c.514A>T	p.R172W
118G9119A1		-	-	In_Frame_Ins	INS	-	-	-	BAP1	c.1847_1854du	p.G619*
118G9120A1	17	7577099	7577099	Missense_Mutation	SNP	C	T	T	TP53	c.839G>A	p.R280K
118G9233A1	1	158596763	158596763	Missense_Mutation	SNP	G	A	A	SPTA1	c.5699C>T	p.S1900F

118G9233A1	11	92543090	92543090	Missense_Mutation	SNP	G	A	A	FAT3	c.9329G>A	p.G3110D
118G9233A1		-	-	Frame_Shift_Del	INS	-	-	-	TP53	c.922_928del	p.L308Tfs*35
118G9233A1	2	170009390	170009390	Missense_Mutation	SNP	C	T	T	LRP2	c.12380G>A	p.R4127H
118G9233A1	6	94120602	94120602	Missense_Mutation	SNP	G	A	A	EPHA7	c.449C>T	p.A150V
118G9262A1	18	48591889	48591889	Missense_Mutation	SNP	A	G	G	SMAD4	c.1052A>G	p.D351G
118G9262A1	20	57484421	57484421	Missense_Mutation	SNP	G	A	A	GNAS	c.602G>A	p.R201H
118G9348A1	1	179086473	179086473	Missense_Mutation	SNP	C	T	T	ABL2	c.1402G>A	p.V468I
118G9348A1		-	-	Frame_Shift_Del	INS	-	-	-	TP53	c.184_211del	p.E62Pfs*52
118G9348A1	19	11134267	11134267	Missense_Mutation	SNP	G	T	T	SMARCA4	c.2933G>T	p.R978L
118G9348A1	6	152163886	152163886	Nonsense_Mutation	SNP	G	T	T	ESR1	c.607G>T	p.E203*
118G9364A1		-	-	Frame_Shift_Del	INS	-	-	-	ARID1A	c.245del	p.G82Vfs*19
118G9364A1	12	25380275	25380275	Missense_Mutation	SNP	T	G	G	KRAS	c.183A>C	p.Q61H
118G9364A1	17	7577539	7577539	Missense_Mutation	SNP	G	A	A	TP53	c.742C>T	p.R248W
118G9364A1		-	-	Frame_Shift_Ins	INS	-	-	-	TGFBR2	.1452_1453insA	p.E485Kfs*4
118G9364A1	9	21974679	21974679	Nonsense_Mutation	SNP	G	A	A	CDKN2A	c.148C>T	p.Q50*
118G9391A1		-	-	Frame_Shift_Del	INS	-	-	-	ARID1A	c.1273del	p.Q425Rfs*8
118G9391A1	12	25398284	25398284	Missense_Mutation	SNP	C	T	T	KRAS	c.35G>A	p.G12D
118G9391A1		-	-	Frame_Shift_Del	INS	-	-	-	TP53	c.642_643del	p.H214Qfs*7
118G9391A1	7	140477815	140477815	Missense_Mutation	SNP	A	T	T	BRAF	c.1493T>A	p.F498Y
118G9391A1	9	21971111	21971111	Missense_Mutation	SNP	G	A	A	CDKN2A	c.247C>T	p.H83Y
118G9397A1	12	57590958	57590958	Splice_Site	SNP	G	A	A	LRP1	c.9076+10G>A	-
118G9397A1		-	-	Missense_Mutation	SNP	-	-	-	TP53	c.764_766del	p.I255del
118G9397A1		-	-	Frame_Shift_Ins	INS	-	-	-	STK11	c.842dup	p.L282Afs*3
118G9397A1	8	41790808	41790808	Missense_Mutation	SNP	G	A	A	KAT6A	c.4930C>T	p.P1644S
118G9397A1	8	41806794	41806794	Missense_Mutation	SNP	C	G	G	KAT6A	c.1686G>C	p.W562C
118G9417A2	12	25398284	25398284	Missense_Mutation	SNP	C	T	T	KRAS	c.35G>A	p.G12D
118G9417A2	12	49428435	49428435	Missense_Mutation	SNP	A	G	G	KMT2D	c.10370T>C	p.L3457P
118G9417A2	14	95574391	95574391	Missense_Mutation	SNP	T	C	C	DICER1	c.2476A>G	p.T826A
118G9417A2	17	7577106	7577106	Missense_Mutation	SNP	G	T	T	TP53	c.832C>A	p.P278T
118G9417A2	17	7577144	7577144	Missense_Mutation	SNP	A	G	G	TP53	c.794T>C	p.L265P
118G9417A2	17	41222990	41222990	Missense_Mutation	SNP	G	C	C	BRCA1	c.4941C>G	p.N1647K
118G9417A2	18	48604722	48604722	Missense_Mutation	SNP	G	C	C	SMAD4	c.1544G>C	p.R515T
118G9417A2	3	71026870	71026870	Missense_Mutation	SNP	T	C	C	FOXP1	c.1352A>G	p.D451G
118G9417A2	3	156421477	156421477	Missense_Mutation	SNP	G	T	T	TIPARP	c.1512G>T	p.W504C
118G9417A2	4	1980613	1980613	Missense_Mutation	SNP	C	T	T	NSD2	c.4075C>T	p.R1359W
118G9417A2	9	139401365	139401365	Missense_Mutation	SNP	C	A	A	NOTCH1	3704G>T;3709A	.[S1235I;K1237Q]
118G9417A2	X	100617204	100617204	Missense_Mutation	SNP	C	T	T	BTK	c.545G>A	p.R182Q
118G9421A1	16	2135300	2135300	Missense_Mutation	SNP	G	A	A	TSC2	c.4639G>A	p.V1547I
118G9421A1		-	-	Frame_Shift_Del	INS	-	-	-	CDKN2A	C.152_156del	P.V51Dfs*67
118G9423A1	1	193104711	193104711	Nonsense_Mutation	SNP	C	T	T	CDC73	c.415C>T	p.R139*
118G9423A1		-	-	Missense_Mutation	SNP	-	-	-	KMT2D	597_7599delins	p.P2533S
118G9423A1	17	7577538	7577538	Missense_Mutation	SNP	C	T	T	TP53	c.743G>A	p.R248Q
118G9423A1	19	10610223	10610223	Missense_Mutation	SNP	G	T	T	KEAP1	c.487C>A	p.Q163K
118G9423A1	20	30661134	30661134	Missense_Mutation	SNP	C	A	A	HCK	c.133C>A	p.H45N
118G9423A1		-	-	Frame_Shift_Del	INS	-	-	-	SETD2	C.5214del	P.L1738Ffs*4
118G9423A1	3	130748692	130748692	Missense_Mutation	SNP	C	T	T	NEK11	c.140C>T	p.S47L
118G9423A1	4	66217130	66217130	Missense_Mutation	SNP	G	T	T	EPHA5	c.2485C>A	p.P829T
118G9448A1	1	27102193	27102193	Missense_Mutation	SNP	A	T	T	ARID1A	c.5119A>T	p.S1707C
118G9448A1	10	84738788	84738788	Nonsense_Mutation	SNP	C	T	T	NRG3	c.1495C>T	p.R499*
118G9448A1	10	89711975	89711975	Missense_Mutation	SNP	T	G	G	PTEN	c.593T>G	p.M198R
118G9448A1	12	46244139	46244139	Nonsense_Mutation	SNP	C	T	T	ARID2	c.2233C>T	p.Q745*
118G9448A1	12	46244959	46244959	Nonsense_Mutation	SNP	C	A	A	ARID2	c.3053C>A	p.S1018*
118G9448A1	12	56491597	56491597	Missense_Mutation	SNP	T	C	C	ERBB3	c.2489T>C	p.M830T
118G9448A1	16	9934597	9934597	Missense_Mutation	SNP	C	T	T	GRIN2A	c.1558G>A	p.E520K
118G9448A1	17	7574003	7574003	Nonsense_Mutation	SNP	G	A	A	TP53	c.1024C>T	p.R342*
118G9448A1		-	-	Frame_Shift_Ins	INS	-	-	-	TP53	c.162_163insT	p.T55Yfs*2
118G9448A1	19	14213709	14213709	Missense_Mutation	SNP	C	G	G	PRKACA	c.255G>C	p.Q85H
118G9448A1	2	11337440	11337440	Missense_Mutation	SNP	A	G	G	ROCK2	c.3314T>C	p.L1105P
118G9448A1	2	225362475	225362475	Nonsense_Mutation	SNP	T	A	A	CUL3	c.1702A>T	p.K568*
118G9448A1	3	89390914	89390914	Missense_Mutation	SNP	C	G	G	EPHA3	c.980C>G	p.S327C
118G9448A1	3	195594327	195594327	Missense_Mutation	SNP	G	A	A	TNK2	c.2797C>T	p.R933W
118G9448A1		-	-	Frame_Shift_Ins	INS	-	-	-	ETV1	c.414dup	p.T139Hfs*24
118G9448A1	8	69129904	69129904	Missense_Mutation	SNP	G	A	A	PREX2	c.4658G>A	p.G1553E
118G9512A1	1	11205035	11205035	Missense_Mutation	SNP	C	T	T	MTOR	c.4754G>A	p.R1585Q
118G9512A1	16	2124214	2124214	Missense_Mutation	SNP	A	G	G	TSC2	c.2369A>G	p.Y790C
118G9512A1	17	7578406	7578406	Missense_Mutation	SNP	C	T	T	TP53	c.524G>A	p.R175H
118G9512A1	22	42524339	42524339	Missense_Mutation	SNP	A	G	G	CYP2D6	c.680T>C	p.V227A
118G9512A1	3	30729980	30729980	Missense_Mutation	SNP	C	G	G	TGFBR2	c.1501C>G	p.P501A
118G9512A1	7	151845579	151845579	Missense_Mutation	SNP	C	T	T	KMT2C	c.13433G>A	p.R4478Q
118G9512A1	8	92983007	92983007	Missense_Mutation	SNP	C	T	T	RUNX1T1	c.1307G>A	p.R436Q
118G9512A1	X	47034459	47034459	Nonsense_Mutation	SNP	C	T	T	RBM10	c.544C>T	p.Q182*
118G9558J1	17	7578535	7578535	Missense_Mutation	SNP	T	C	C	TP53	c.395A>G	p.K132R
118G9558J1	18	45368223	45368223	Nonsense_Mutation	SNP	G	T	T	SMAD2	c.1289C>A	p.S430*
118G9558J1		-	-	Frame_Shift_Del	INS	-	-	-	STK11	c.482_483del	p.I161Rfs*8
118G9558J1	5	112128185	112128185	Missense_Mutation	SNP	C	T	T	APC	c.688C>T	p.R230C
118G9558J1	X	66941797	66941797	Missense_Mutation	SNP	T	G	G	AR	c.2441T>G	p.F814C
118G9559A1	12	25398284	25398284	Missense_Mutation	SNP	C	T	T	KRAS	c.35G>A	p.G12D
118G9559A1	17	7578442	7578442	Missense_Mutation	SNP	T	C	C	TP53	c.488A>G	p.Y163C
118G9559A1	18	48603032	48603032	Nonsense_Mutation	SNP	C	T	T	SMAD4	c.1333C>T	p.R445*
118G9559A1	7	151856061	151856061	Missense_Mutation	SNP	G	A	A	KMT2C	c.11557C>T	p.R3853W
118G9559A1		-	-	Frame_Shift_Ins	INS	-	-	-	CDKN2A	C.243dup	P.V82Rfs*38
118G9581A1		-	-	Frame_Shift_Ins	INS	-	-	-	APC	C.4666dup	P.T1556Nfs*3
118G9603A1	1	158624424	158624424	Missense_Mutation	SNP	T	G	G	SPTA1	c.3013A>C	p.T1005P
118G9603A1	13	28601290	28601290	Missense_Mutation	SNP	C	G	G	FLT3	c.2142G>C	p.W714C
118G9603A1	15	88423632	88423632	Missense_Mutation	SNP	G	A	A	NTRK3	c.2161C>T	p.R721C
118G9603A1		-	-	Frame_Shift_Del	INS	-	-	-	TP53	c.879_880del	p.E294Afs*11
118G9603A1		-	-	Frame_Shift_Del	INS	-	-	-	SMAD4	c.889del	p.H297Ifs*39
118G9603A1		-	-	Missense_Mutation	SNP	-	-	-	SMAD4	c.1137_1139del	p.R380del
118G9603A1	2	141607761	141607761	Missense_Mutation	SNP	C	T	T	LRP1B	c.4849G>A	p.E1617K
118G9603A1	3	41266124	41266124	Missense_Mutation	SNP	A	G	G	CTNNB1	c.121A>G	p.T41A
118G9603A1	3	87027819	87027819	Missense_Mutation	SNP	C	T	T	VGLL3	c.260G>A	p.R87H
118G9603A1	3	170011214	170011214	Missense_Mutation	SNP	G	C	C	PRKCI	c.1335G>C	p.E445D
118G9603A1	4	20525790	20525790	Missense_Mutation	SNP	C	A	A	SLIT2	c.1428C>A	p.F476L
118G9603A1	4	153249456	153249456	Missense_Mutation	SNP	C	T	T	FBXW7	c.1322G>A	p.R441Q
118G9603A1	7	2951868	2951868	Missense_Mutation	SNP	C	T	T	CARD11	c.3082G>A	p.E1028K
118G9603A1	7	41729606	41729606	Missense_Mutation	SNP	C	T	T	INHBA	c.923G>A	p.R308H
118G9603A1	7	151853002	151853002	Nonsense_Mutation	SNP	C	A	A	KMT2C	c.11953G>T	p.E3985*
118G9617A1	17	7577534	7577534	Missense_Mutation	SNP	C	A	A	TP53	c.747G>T	p.R249S

118G9617A1	19	10602300	10602300	Nonsense_Mutation	SNP	A	C	C	KEAP1	c.1278T>G	p.Y426*
118G9617A1	X	39934298	39934298	Missense_Mutation	SNP	C	G	G	BCOR	c.301G>C	p.G101R
118G9617A1	X	70345909	70345909	Nonsense_Mutation	SNP	C	T	T	MED12	c.2446C>T	p.R816*
118G9617A1	17	7577534	7577534	Missense_Mutation	SNP	C	A	A	TP53	c.747G>T	p.R249S
118G9617A1	X	39934298	39934298	Missense_Mutation	SNP	C	G	G	BCOR	c.301G>C	p.G101R
118G9676A1		-	-	Frame_Shift_Del	INS	-	-	-	ARID1A	c.609del	p.H203Qfs*29
118G9676A1	12	25398284	25398284	Missense_Mutation	SNP	C	T	T	KRAS	c.35G>A	p.G12D
118G9676A1	17	7578268	7578268	Missense_Mutation	SNP	A	G	G	TP53	c.581T>C	p.L194P
118G9676A1		-	-	In_Frame_Ins	INS	-	-	-	CDKN2A	c.131dup	p.Y44*
118G9722A1		-	-	Splice_Site	SNP	-	-	-	CYLD	51-16_2351-2de	-
118G9722A1	17	7577114	7577114	Missense_Mutation	SNP	C	T	T	TP53	c.824G>A	p.C275Y
118G9722A1	4	66213918	66213918	Nonsense_Mutation	SNP	C	A	A	EPHA5	c.2512G>T	p.G838*
118G9722A1	8	141810631	141810631	Missense_Mutation	SNP	C	T	T	PTK2	c.1086G>A	p.M362I
118G9724A1	17	29665743	29665743	Nonsense_Mutation	SNP	G	T	T	NF1	c.6841G>T	p.G2281*
118G9729A1		-	-	Frame_Shift_Del	INS	-	-	-	TP53	c.876del	p.E294Sfs*51
118G9729A1	4	143235911	143235911	Missense_Mutation	SNP	C	T	T	INPP4B	c.377G>A	p.R126Q
118G9729A1	8	69046362	69046362	Missense_Mutation	SNP	T	A	A	PREX2	c.3835T>A	p.S1279T
118G9729A1	9	21971117	21971117	Missense_Mutation	SNP	G	T	T	CDKN2A	c.241C>A	p.P81T
118G9742A1	11	92533107	92533107	Missense_Mutation	SNP	G	A	A	FAT3	c.6928G>A	p.V2310I
118G9742A1	12	25398284	25398284	Missense_Mutation	SNP	C	T	T	KRAS	c.35G>A	p.G12D
118G9742A1	17	48269373	48269373	Missense_Mutation	SNP	C	A	A	COL1A1	c.1996G>T	p.D666Y
118G9742A1	17	66518996	66518996	Missense_Mutation	SNP	G	T	T	PRKAR1A	c.277G>T	p.G93C
118G9742A1	2	48033780	48033780	Nonsense_Mutation	SNP	C	T	T	MSH6	c.3991C>T	p.R1331*
118G9742A1	2	198266834	198266834	Missense_Mutation	SNP	T	C	C	SF3B1	c.2098A>G	p.K700E
118G9742A1	8	139163791	139163791	Missense_Mutation	SNP	G	T	T	FAM135B	c.2927C>A	p.P976Q
118G9742A1	X	76907651	76907651	Nonsense_Mutation	SNP	G	A	A	ATRX	c.4510C>T	p.R1504*
118G9781A1	1	27942071	27942071	Missense_Mutation	SNP	T	C	C	FGR	c.892A>G	p.M298V
118G9781A1	1	158609403	158609403	Missense_Mutation	SNP	T	A	A	SPTA1	c.4949A>T	p.Q1650L
118G9781A1	12	69218398	69218398	Missense_Mutation	SNP	A	T	T	MDM2	c.490A>T	p.T164S
118G9781A1		-	-	Frame_Shift_Ins	INS	-	-	-	TP53	c.494_495dup	p.Q167Hfs*4
118G9781A1	20	31022763	31022763	Missense_Mutation	SNP	C	A	A	ASXL1	c.2248C>A	p.P750T
118G9781A1	3	47164685	47164685	Nonsense_Mutation	SNP	T	A	A	SETD2	c.1441A>T	p.R481*
118G9781A1	4	187538357	187538357	Splice_Site	SNP	T	A	A	FAT1	c.8879-2A>T	-
118G9781A1	5	176519743	176519743	Missense_Mutation	SNP	A	T	T	FGFR4	c.1015A>T	p.I339F
118G9781A1	8	69129852	69129852	Missense_Mutation	SNP	T	A	A	PREX2	c.4606T>A	p.C1536S
118G9797A1	1	120478095	120478095	Missense_Mutation	SNP	C	G	G	NOTCH2	c.3655G>C	p.G1219R
118G9797A1	15	45003751	45003751	Missense_Mutation	SNP	C	T	T	B2M	c.7C>T	p.R3C
118G9797A1	17	7577082	7577082	Missense_Mutation	SNP	C	T	T	TP53	c.856G>A	p.E286K
118G9797A1	2	11337395	11337395	Missense_Mutation	SNP	C	T	T	ROCK2	3359G>A;3364C	[R1120Q;Q1122E]
118G9797A1	2	141777614	141777614	Missense_Mutation	SNP	G	T	T	LRP1B	c.1847C>A	p.T616N
118G9912A1	1	158641935	158641935	Missense_Mutation	SNP	G	A	A	SPTA1	c.1402C>T	p.R468C
118G9912A1	2	212248377	212248377	Missense_Mutation	SNP	G	A	A	ERBB4	c.3890C>T	p.P1297L
118G9912A1	5	38950829	38950829	Splice_Site	SNP	T	C	C	RICTOR	c.3128-7A>G	-
118G9955A1	17	16024432	16024432	Missense_Mutation	SNP	C	A	A	NCOR1	c.1786G>T	p.A596S
118G9955A1	3	41266100	41266100	Missense_Mutation	SNP	T	C	C	CTNNB1	c.97T>C	p.S33P
118G9955A1		-	-	Splice_Site	SNP	-	-	-	IRF4	4-12_404-9delC	-
118G9955A1		-	-	In_Frame_Ins	INS	-	-	-	KAT6A	c.1536dup	p.E513*
118G9970A1	19	18266993	18266993	Missense_Mutation	SNP	G	A	A	PIK3R2	c.304G>A	p.D102N
118G9970A1	4	20525654	20525654	Missense_Mutation	SNP	C	T	T	SLIT2	c.1292C>T	p.P431L
118H1438M1	3	52442078	52442078	Missense_Mutation	SNP	A	G	G	BAP1	c.271T>C	p.C91R
118H1479A1		-	-	Frame_Shift_Del	INS	-	-	-	EPHA2	C.1436_1449de	P.S479Cfs*112
118H1479A1		-	-	Frame_Shift_Ins	INS	-	-	-	ARID2	C.2364dup	P.V789Cfs*52
118H1479A1		-	-	Splice_Site	SNP	-	-	-	POLE	5812-7_5812-6d	-
118H1479A1	15	90631839	90631839	Missense_Mutation	SNP	T	C	C	IDH2	c.514A>G	p.R172G
118H1479A1		-	-	Frame_Shift_Del	INS	-	-	-	TP53	C.810_825del	P.F270Lfs*70
118H1479A1	7	73530217	73530217	Missense_Mutation	SNP	A	G	G	LIMK1	c.1496A>G	p.K499R
118H1515A1	11	60231782	60231782	Missense_Mutation	SNP	C	A	A	MS4A1	c.301C>A	p.L101M
118H1515A1	17	7578239	7578239	Nonsense_Mutation	SNP	C	A	A	TP53	c.610G>T	p.E204*
118H1515A1	17	15983971	15983971	Missense_Mutation	SNP	A	G	G	NCOR1	c.3248T>C	p.V1083A
118H1515A1		-	-	Frame_Shift_Del	INS	-	-	-	STK11	c.610_613del	p.F204Rfs*82
118H1577A1		-	-	Splice_Site	SNP	-	-	-	MRE11	59+6_659+7delin	-
118H1577A1	17	7577124	7577124	Missense_Mutation	SNP	C	A	A	TP53	c.814G>T	p.V272L
118H1577A1	2	47601141	47601141	Missense_Mutation	SNP	A	G	G	EPCAM	c.379A>G	p.T127A
118H1577A1	2	141027805	141027805	Splice_Site	SNP	A	T	T	LRP1B	c.13247+6T>A	-
118H1577A1	2	170063350	170063350	Missense_Mutation	SNP	C	G	G	LRP2	c.6880G>C	p.D2294H
118H1577A1	6	135514976	135514976	Missense_Mutation	SNP	G	T	T	MYB	c.763G>T	p.V255F
118H1577A1	X	66765409	66765409	Missense_Mutation	SNP	G	T	T	AR	c.421G>T	p.A141S
118H1607A1	11	92085831	92085831	Missense_Mutation	SNP	G	A	A	FAT3	c.553G>A	p.A185T
118H1607A1		-	-	Frame_Shift_Del	INS	-	-	-	TP53	c.365_366del	p.V122Dfs*26
118H1607A1		-	-	Frame_Shift_Del	INS	-	-	-	NF1	c.3936del	p.D1313Ifs*14
118H1607A1	19	11141507	11141507	Missense_Mutation	SNP	G	A	A	SMARCA4	c.3484G>A	p.G1162S
118H1607A1	22	30051652	30051652	Nonsense_Mutation	SNP	C	T	T	NF2	c.586C>T	p.R196*
118H1642A1	12	56478817	56478817	Missense_Mutation	SNP	G	A	A	ERBB3	c.273G>A	p.M91I
118H1642A1	14	36986773	36986773	Missense_Mutation	SNP	C	A	A	NKX2-1	c.916G>T	p.A306S
118H1642A1	17	7577120	7577120	Missense_Mutation	SNP	C	T	T	TP53	c.818G>A	p.R273H
118H1642A1	22	23656785	23656785	Missense_Mutation	SNP	G	A	A	BCR	c.3610G>A	p.A1204T
118H1642A1	7	151856085	151856085	Nonsense_Mutation	SNP	C	A	A	KMT2C	c.11533G>T	p.E3845*
118H1685A1	1	27088759	27088759	Nonsense_Mutation	SNP	C	T	T	ARID1A	c.2368C>T	p.Q790*
118H1685A1	19	42797228	42797228	Missense_Mutation	SNP	C	T	T	CIC	c.3590C>T	p.S1197F
118H1689A1	18	48604778	48604778	Nonsense_Mutation	SNP	C	T	T	SMAD4	c.1600C>T	p.Q534*
118H1689A1	19	1220579	1220579	Splice_Site	SNP	G	C	C	STK11	c.598-1G>C	-
118H1733A1	12	25398284	25398284	Missense_Mutation	SNP	C	A	A	KRAS	c.35G>T	p.G12V
118H1733A1	17	7577539	7577539	Missense_Mutation	SNP	G	A	A	TP53	c.742C>T	p.R248W
118H1787A1	12	46230571	46230571	Nonsense_Mutation	SNP	C	T	T	ARID2	c.820C>T	p.R274*
118H1787A1	14	45623912	45623912	Missense_Mutation	SNP	C	T	T	FANCM	c.1196C>T	p.S399L
118H1787A1	17	7577082	7577082	Missense_Mutation	SNP	C	T	T	TP53	c.856G>A	p.E286K
118H1787A1	6	36653561	36653561	Missense_Mutation	SNP	C	T	T	CDKN1A	c.479C>T	p.S160F
118H1787A1	8	41905993	41905993	Missense_Mutation	SNP	C	T	T	KAT6A	c.503G>A	p.R168Q
118H1840A1	12	57567022	57567022	Missense_Mutation	SNP	C	T	T	LRP1	c.3235C>T	p.R1079W
118H1840A1	13	28626712	28626712	Missense_Mutation	SNP	T	C	C	FLT3	c.584A>G	p.E195G
118H1840A1	2	209113113	209113113	Missense_Mutation	SNP	G	A	A	IDH1	c.394C>T	p.R132C
118H1840A1	3	52643328	52643328	Splice_Site	SNP	C	T	T	PBRM1	c.2567+1G>A	-
118H1840A1	X	66942707	66942707	Nonsense_Mutation	SNP	G	T	T	AR	c.2488G>T	p.E830*
118H1840A1	X	76939715	76939715	Missense_Mutation	SNP	C	T	T	ATRX	c.1033G>A	p.A345T
118H2136A1	1	27106354	27106354	Nonsense_Mutation	SNP	C	T	T	ARID1A	c.5965C>T	p.R1989*
118H2136A1	10	43610033	43610033	Missense_Mutation	SNP	A	G	G	RET	c.1985A>G	p.K662R
118H2136A1	13	49030487	49030487	Splice_Site	SNP	T	A	A	RB1	c.1960+2T>A	-
118H2136A1	15	91358353	91358353	Missense_Mutation	SNP	G	C	C	BLM	c.4098G>C	p.K1366N

118H2136A1		-	-	Frame_Shift_Ins	INS	-	-	-	TP53	C.342dup	P.H115Afs*34
118H2136A1	17	63531744	63531744	Missense_Mutation	SNP	T	C	C	AXIN2	c.2237A>G	p.D746G
118H2136A1	17	78599496	78599496	Missense_Mutation	SNP	G	C	C	RPTOR	c.168G>C	p.K56N
118H2136A1		-	-	Frame_Shift_Del	INS	-	-	-	SMAD4	C.953del	P.P318Lfs*18
118H2136A1	2	170059470	170059470	Splice_Site	SNP	C	A	A	LRP2	c.8006-1G>T	-
118H2136A1	20	57429628	57429628	Missense_Mutation	SNP	C	T	T	GNAS	c.1121C>T	p.P374L
118H2136A1	3	12660112	12660112	Missense_Mutation	SNP	C	G	G	RAF1	c.109G>C	p.G37R
118H2136A1	3	138409835	138409835	Splice_Site	SNP	G	C	C	PIK3CB	c.2036+7C>G	-
118H2136A1	3	185191256	185191256	Missense_Mutation	SNP	C	G	G	MAP3K13	c.2137C>G	p.P713A
118H2136A1	7	106508522	106508522	Missense_Mutation	SNP	G	C	C	PIK3CG	c.516G>C	p.E172D
118H2136A1	7	140481402	140481402	Missense_Mutation	SNP	C	G	G	BRAF	c.1406G>C	p.G469A
118H2136A1	8	48715963	48715963	Nonsense_Mutation	SNP				PRKDC	c.9825G>A	p.W3275*
118H2136A1	8	48765245	48765245	Missense_Mutation	SNP	C	A	A	PRKDC	c.6993G>T	p.E2331D
118H2136A1	9	139393361	139393361	Missense_Mutation	SNP	T	C	C	NOTCH1	c.6170A>G	p.Q2057R
118H2136A1	X	76814163	76814163	Missense_Mutation	SNP	A	G	G	ATRX	c.6481T>C	p.Y2161H
118H2149S1	1	9777149	9777149	Missense_Mutation	SNP	C	T	T	PIK3CD	c.913C>T	p.P305S
118H2149S1	1	27940979	27940979	Missense_Mutation	SNP	G	A	A	FGR	c.1211C>T	p.A404V
118H2149S1	1	43770729	43770729	Missense_Mutation	SNP	C	T	T	TIE1	c.266C>T	p.T89M
118H2149S1	1	179100530	179100530	Missense_Mutation	SNP	C	T	T	ABL2	c.307G>A	p.G103R
118H2149S1	10	131265562	131265562	Splice_Site	SNP	T	C	C	MGMT	c.81+2T>C	-
118H2149S1	11	92534025	92534025	Missense_Mutation	SNP	G	A	A	FAT3	c.7846G>A	p.G2616S
118H2149S1	11	108204681	108204681	Missense_Mutation	SNP	A	G	G	ATM	c.7996A>G	p.T2666A
118H2149S1	11	119145633	119145633	Missense_Mutation	SNP	G	A	A	CBL	c.839G>A	p.R280Q
118H2149S1	11	128680615	128680615	Missense_Mutation	SNP	G	A	A	FLI1	c.1091G>A	p.G364D
118H2149S1	12	57563000	57563000	Missense_Mutation	SNP	G	A	A	LRP1	c.3073G>A	p.G1025R
118H2149S1	12	112915797	112915797	Missense_Mutation	SNP	C	T	T	PTPN11	c.1070C>T	p.T357M
118H2149S1	12	122492869	122492869	Missense_Mutation	SNP	G	A	A	BCL7A	c.598G>A	p.G200S
118H2149S1		-	-	Frame_Shift_Del	INS	-	-	-	FLT3	c.2955_2956del	p.P986Afs*27
118H2149S1	14	95599700	95599700	Nonsense_Mutation	SNP	C	T	T	DICER1	c.96G>A	p.W32*
118H2149S1	16	2131685	2131685	Missense_Mutation	SNP	G	A	A	TSC2	c.3700G>A	p.A1234T
118H2149S1	16	68867356	68867356	Missense_Mutation	SNP	G	A	A	CDH1	c.2603G>A	p.R868H
118H2149S1	17	37871746	37871746	Missense_Mutation	SNP	G	A	A	ERBB2	c.1270G>A	p.V424I
118H2149S1		-	-	Frame_Shift_Del	INS	-	-	-	RNF43	c.1976del	p.G659Vfs*41
118H2149S1	19	3114953	3114953	Missense_Mutation	SNP	A	C	C	GNA11	c.488A>C	p.D163A
118H2149S1	19	18273784	18273784	Missense_Mutation	SNP	G	A	A	PIK3R2	c.1117G>A	p.G373R
118H2149S1	19	50906837	50906837	Missense_Mutation	SNP	C	T	T	POLD1	c.1225C>T	p.R409W
118H2149S1	2	16085728	16085728	Missense_Mutation	SNP	C	T	T	MYCN	c.904C>T	p.R302C
118H2149S1	2	47637357	47637357	Missense_Mutation	SNP	G	A	A	MSH2	c.491G>A	p.G164E
118H2149S1	2	141272257	141272257	Missense_Mutation	SNP	C	T	T	LRP1B	c.8234G>A	p.C2745Y
118H2149S1	2	141747101	141747101	Missense_Mutation	SNP	C	A	A	LRP1B	c.2770G>T	p.A924S
118H2149S1	2	148680563	148680563	Missense_Mutation	SNP	G	A	A	ACVR2A	c.1099G>A	p.A367T
118H2149S1		-	-	Frame_Shift_Del	INS	-	-	-	ACVR2A	c.1310del	p.K437Rfs*5
118H2149S1	20	30689231	30689231	Missense_Mutation	SNP	G	A	A	HCK	c.1427G>A	p.R476H
118H2149S1	20	36031750	36031750	Missense_Mutation	SNP	G	A	A	SRC	c.1579G>A	p.E527K
118H2149S1	20	57430079	57430079	Missense_Mutation	SNP	G	A	A	GNAS	c.1759G>A	p.G587R
118H2149S1	20	57484421	57484421	Missense_Mutation	SNP	G	A	A	GNAS	c.602G>A	p.R201H
118H2149S1	22	21343116	21343116	Missense_Mutation	SNP	A	G	G	LZTR1	c.548A>G	p.Y183C
118H2149S1	22	24159067	24159067	Missense_Mutation	SNP	T	C	C	SMARCB1	c.739T>C	p.S247P
118H2149S1	22	42525829	42525829	Missense_Mutation	SNP	C	T	T	CYP2D6	c.263G>A	p.R88H
118H2149S1		-	-	Frame_Shift_Del	INS	-	-	-	XPC	c.2782_2785del	p.K928Qfs*18
118H2149S1	3	52651532	52651532	Nonsense_Mutation	SNP	G	A	A	PBRM1	c.1564C>T	p.R522*
118H2149S1	3	128205829	128205829	Missense_Mutation	SNP	C	T	T	GATA2	c.46G>A	p.V16M
118H2149S1	4	88986961	88986961	Missense_Mutation	SNP	A	G	G	PKD2	c.2288A>G	p.D763G
118H2149S1	4	106164898	106164898	Missense_Mutation	SNP	G	A	A	TET2	c.3766G>A	p.G1256S
118H2149S1	4	126329910	126329910	Missense_Mutation	SNP	T	A	A	FAT4	c.5881T>A	p.S1961T
118H2149S1	4	126369876	126369876	Missense_Mutation	SNP	G	A	A	FAT4	c.7705G>A	p.A2569T
118H2149S1	4	153244065	153244065	Missense_Mutation	SNP	G	T	T	FBXW7	c.2092C>A	p.L698M
118H2149S1	5	149456996	149456996	Missense_Mutation	SNP	G	A	A	CSF1R	c.732C>T	p.L244L
118H2149S1	5	156667179	156667179	Missense_Mutation	SNP	T	A	A	ITK	c.959T>A	p.I320N
118H2149S1	6	394941	394941	Missense_Mutation	SNP	C	T	T	IRF4	c.337C>T	p.R113W
118H2149S1	6	93965625	93965625	Missense_Mutation	SNP	C	T	T	EPHA7	c.2303G>A	p.S768N
118H2149S1	6	138202412	138202412	Missense_Mutation	SNP	G	A	A	TNFAIP3	c.2329G>A	p.G777S
118H2149S1	7	50450329	50450329	Missense_Mutation	SNP	G	T	T	IKZF1	c.513G>T	p.K171N
118H2149S1		-	-	Frame_Shift_Del	INS	-	-	-	CFTR	c.2835_2836del	p.K946Nfs*28
118H2149S1	8	139164544	139164544	Missense_Mutation	SNP	C	T	T	FAM135B	c.2174G>A	p.R725Q
118H2149S1	9	133760442	133760442	Missense_Mutation	SNP	C	T	T	ABL1	c.2822C>T	p.A941V
118H2157A1	11	92531778	92531778	Missense_Mutation	SNP	G	A	A	FAT3	c.5599G>A	p.V1867I
118H2157A1	15	99486181	99486181	Missense_Mutation	SNP	A	G	G	IGF1R	c.3487A>G	p.T1163A
118H2157A1		-	-	In_Frame_Del	INS	-	-	-	CYLD	c.1421_1434del	p.S474*
118H2157A1	17	7578190	7578190	Missense_Mutation	SNP	T	C	C	TP53	c.659A>G	p.Y220C
118H2157A1	17	29684041	29684041	Nonsense_Mutation	SNP	C	A	A	NF1	c.7802C>A	p.S2601*
118H2157A1	19	1226453	1226453	Missense_Mutation	SNP	G	T	T	STK11	c.1109G>T	p.G370V
118H2157A1	3	89456434	89456434	Missense_Mutation	SNP	G	A	A	EPHA3	c.1610G>A	p.G537D
118H2157A1	6	157511321	157511321	Missense_Mutation	SNP	A	G	G	ARID1B	c.3839A>G	p.D1280G
118H2157A1	9	36846891	36846891	Missense_Mutation	SNP	G	T	T	PAX5	c.1048C>A	p.Q350K
118H2438A1	1	16464509	16464509	Missense_Mutation	SNP	C	T	T	EPHA2	c.1151G>A	p.R384H
118H2438A1		-	-	Frame_Shift_Ins	INS	-	-	-	ARID1A	c.4303_4340dup	p.A1448Ifs*46
118H2438A1	17	7577559	7577559	Missense_Mutation	SNP	G	T	T	TP53	c.722C>A	p.S241Y
118H2438A1	18	48593397	48593397	Missense_Mutation	SNP	T	G	G	SMAD4	c.1148T>G	p.I383R
118H2438A1	2	170115694	170115694	Missense_Mutation	SNP	T	C	C	LRP2	c.2354A>G	p.N785S
118H2438A1	8	68864735	68864735	Missense_Mutation	SNP	C	T	T	PREX2	c.106C>T	p.R36W
118H2472A1	1	158622390	158622390	Missense_Mutation	SNP	C	T	T	SPTA1	c.3242G>A	p.R1081H
118H2472A1		-	-	Missense_Mutation	SNP	-	-	-	KRAS	c.35_36delinsTG	p.G12V
118H2472A1	2	198267483	198267483	Missense_Mutation	SNP	C	T	T	SF3B1	c.1874G>A	p.R625H
118H2472A1	3	52637554	52637554	Missense_Mutation	SNP	C	T	T	PBRM1	c.2762G>A	p.R921Q
118H2472A1	7	142458528	142458528	Missense_Mutation	SNP	G	A	A	PRSS1	c.163G>A	p.E55K
118H2472A1	9	21971120	21971120	Nonsense_Mutation	SNP	G	A	A	CDKN2A	c.238C>T	p.R80*
118H2473A1	10	84733640	84733640	Missense_Mutation	SNP	G	A	A	NRG3	c.1381G>A	p.D461N
118H2473A1	15	67482861	67482861	Missense_Mutation	SNP	C	T	T	SMAD3	c.1265C>T	p.S422F
118H2473A1	15	93515167	93515167	Splice_Site	SNP	T	C	C	CHD2	c.2352+10T>C	-
118H2473A1		-	-	Frame_Shift_Del	INS	-	-	-	TP53	c.994_1012del	p.I332Sfs*7
118H2473A1	17	29528441	29528441	Nonsense_Mutation	SNP	C	T	T	NF1	1198C>T;1205C	p.[Q400*;S402L]
118H2473A1	17	29528489	29528489	Nonsense_Mutation	SNP	C	T	T	NF1	c.1246C>T	p.R416*
118H2473A1	17	29667602	29667602	Missense_Mutation	SNP	G	A	A	NF1	c.7001G>A	p.G2334D
118H2473A1	19	2199922	2199922	Nonsense_Mutation	SNP	C	T	T	DOT1L	c.691C>T	p.R231*
118H2473A1	19	11136147	11136147	Missense_Mutation	SNP	A	C	C	SMARCA4	c.3131A>C	p.K1044T
118H2473A1	22	42525827	42525827	Missense_Mutation	SNP	C	T	T	CYP2D6	c.265G>A	p.E89K
118H2473A1	3	178952090	178952090	Missense_Mutation	SNP	G	C	C	PIK3CA	c.3145G>C	p.G1049R

118H2473A1	5	112137007	112137007	Missense_Mutation	SNP	C	T	T	APC	c.761C>T	p.S254L
118H2473A1	5	176662911	176662911	Missense_Mutation	SNP	A	C	C	NSD1	c.3886A>C	p.K1296Q
118H2473A1	9	139390602	139390602	Missense_Mutation	SNP	G	A	A	NOTCH1	c.7589C>T	p.S2530F
118H2475M1	12	25398284	25398284	Missense_Mutation	SNP	G	A	A	KRAS	c.35G>T	p.G12V
118H2475M1	2	198266834	198266834	Missense_Mutation	SNP	T	C	C	SF3B1	c.2098A>G	p.K700E
118H2475M1	7	117232475	117232475	Missense_Mutation	SNP	A	G	G	CFTR	c.2254A>G	p.I752V
118H2475M1	9	101900221	101900221	Missense_Mutation	SNP	G	T	T	TGFBR1	c.655G>T	p.V219F
118H2476A1		-	-	Frame_Shift_Del	INS	-	-	-	LRP1	.11695_11696de	p.S3899Cfs*4
118H2476A1		-	-	Missense_Mutation	SNP	-	-	-	CREBBP	c.5039_5041del	p.S1680del
118H2476A1		-	-	In_Frame_Ins	INS	-	-	-	MSH6	c.1608dup	p.K537*
118H2476A1	3	52442512	52442512	Missense_Mutation	SNP	T	C	C	BAP1	c.233A>G	p.N78S
118H2476A1		-	-	Frame_Shift_Ins	INS	-	-	-	TNFAIP3	c.655dup	p.E219Gfs*35
118H2476A1	7	140453136	140453136	Missense_Mutation	SNP	A	T	T	BRAF	c.1799T>A	p.V600E
118H2503A1	12	49433655	49433655	Nonsense_Mutation	SNP	G	C	C	KMT2D	c.7898C>G	p.S2633*
118H2503A1	16	23646503	23646503	Missense_Mutation	SNP	T	C	C	PALB2	c.1364A>G	p.N455S
118H2503A1	16	68867235	68867235	Missense_Mutation	SNP	G	A	A	CDH1	c.2482G>A	p.D828N
118H2503A1	17	7577505	7577505	Missense_Mutation	SNP	T	A	A	TP53	c.776A>T	p.D259V
118H2503A1	20	52193685	52193685	Nonsense_Mutation	SNP	G	A	A	ZNF217	c.1618C>T	p.Q540*
118H2503A1	3	47158212	47158212	Missense_Mutation	SNP	C	A	A	SETD2	c.4487G>T	p.R1496L
118H2503A1	3	52440271	52440271	Nonsense_Mutation	SNP	G	A	A	BAP1	c.781C>T	p.Q261*
118H2503A1	3	52598140	52598140	Missense_Mutation	SNP	C	A	A	PBRM1	c.3726G>T	p.Q1242H
118H2503A1	9	139401405	139401405	Missense_Mutation	SNP	C	T	T	NOTCH1	c.3664G>A	p.V1222M
118H2503A1	X	123197056	123197056	Splice_Site	SNP	G	C	C	STAG2	c.1821+1G>C	-
118H2531A3	11	128680614	128680614	Missense_Mutation	SNP	G	A	A	FLI1	c.1090G>A	p.G364S
118H2531A3	12	12870900	12870900	Missense_Mutation	SNP	C	G	G	CDKN1B	c.127C>G	p.R43G
118H2531A3	12	133254193	133254193	Missense_Mutation	SNP	G	A	A	POLE	c.691C>T	p.R231C
118H2531A3	17	7577094	7577094	Missense_Mutation	SNP	G	A	A	TP53	c.844C>T	p.R282W
118H2531A3	19	17940997	17940997	Missense_Mutation	SNP	C	G	G	JAK3	c.3127G>C	p.D1043H
118H2540A1		-	-	Frame_Shift_Ins	INS	-	-	-	ARID1A	c.4689dup	p.M1564Hfs*8
118H2540A1	10	104353749	104353749	Splice_Site	SNP	G	A	A	SUFU	c.684-1G>A	-
118H2540A1	17	7578505	7578505	Missense_Mutation	SNP	G	A	A	TP53	c.425C>T	p.P142L
118H2588A1	16	343642	343642	Nonsense_Mutation	SNP	G	A	A	AXIN1	c.2032C>T	p.Q678*
118H2588A1	17	7578433	7578433	Nonsense_Mutation	SNP	G	C	C	TP53	c.497C>G	p.S166*
118H2588A1	18	48604778	48604778	Nonsense_Mutation	SNP	C	T	T	SMAD4	c.1600C>T	p.Q534*
118H2588A1	19	15288868	15288868	Missense_Mutation	SNP	G	A	A	NOTCH3	c.3871C>T	p.R1291C
118H2588A1	2	141665655	141665655	Splice_Site	SNP	A	T	T	LRP1B	c.3320-9T>A	-
118H2588A1	22	41545883	41545883	Missense_Mutation	SNP	C	T	T	EP300	c.2498C>T	p.S833L
118H2588A1	3	178921553	178921553	Missense_Mutation	SNP	T	A	A	PIK3CA	c.1035T>A	p.N345K
118H2588A1	7	151853289	151853289	Splice_Site	SNP	C	G	G	KMT2C	c.11812+1G>C	-
118H2588A1	9	97869493	97869493	Nonsense_Mutation	SNP	G	C	C	FANCC	c.1388C>G	p.S463*
118H2593A1	1	120462972	120462972	Missense_Mutation	SNP	G	A	A	NOTCH2	c.5359C>T	p.R1787W
118H2593A1	12	25398284	25398284	Missense_Mutation	SNP	C	T	T	KRAS	c.35G>A	p.G12D
118H2593A1	20	36024580	36024580	Missense_Mutation	SNP	C	T	T	SRC	c.569C>T	p.S190L
118H2593A1		-	-	Frame_Shift_Del	INS	-	-	-	CDKN1A	C.251_288del	P.R84Hfs*32
118H2593A1	9	101904862	101904862	Nonsense_Mutation	SNP	G	T	T	TGFBR1	c.850G>T	p.E284*
118H2626A1	11	92257952	92257952	Missense_Mutation	SNP	T	A	A	FAT3	c.3445T>A	p.S1149T
118H2626A1	11	108216606	108216606	Missense_Mutation	SNP	A	G	G	ATM	c.8555A>G	p.Y2852C
118H2626A1	12	25398284	25398284	Missense_Mutation	SNP	C	A	A	KRAS	c.35G>T	p.G12V
118H2626A1	19	10463722	10463722	Missense_Mutation	SNP	C	T	T	TYK2	c.3080G>A	p.R1027H
118H2626A1	2	74317026	74317026	Missense_Mutation	SNP	G	A	A	TET3	c.2891G>A	p.R964Q
118H2626A1	4	55946257	55946257	Missense_Mutation	SNP	C	T	T	KDR	c.3922G>A	p.G1308R
118H2626A1	7	138252244	138252244	Missense_Mutation	SNP	A	G	G	TRIM24	c.1447A>G	p.I483V
118H2626A1	8	41800520	41800520	Splice_Site	SNP	T	A	A	KAT6A	c.2229-2A>T	-
118H2716A1	12	25398285	25398285	Missense_Mutation	SNP	C	T	T	KRAS	c.34G>A	p.G12S
118H2716A1	17	7578406	7578406	Missense_Mutation	SNP	C	T	T	TP53	c.524G>A	p.R175H
118H2716A1	9	101908834	101908834	Missense_Mutation	SNP	G	A	A	TGFBR1	c.1198G>A	p.D400N
118H2758A1		-	-	Splice_Site	SNP	-	-	-	PTEN	.209+4_209+7de	-
118H2758A1		-	-	Frame_Shift_Ins	INS	-	-	-	PTEN	c.920dup	p.R308Afs*4
118H2758A1	12	56482552	56482552	Missense_Mutation	SNP	G	A	A	ERBB3	c.1009G>A	p.G337R
118H2758A1	17	7578403	7578403	Missense_Mutation	SNP	C	T	T	TP53	c.527G>A	p.C176Y
118H2758A1		-	-	Frame_Shift_Del	INS	-	-	-	SMAD4	c.579_580del	p.E193Dfs*12
118H2758A1	19	10600330	10600330	Missense_Mutation	SNP	C	G	G	KEAP1	c.1525G>C	p.G509R
118H2758A1	2	141283484	141283484	Missense_Mutation	SNP	C	A	A	LRP1B	c.7955G>T	p.C2652F
118H2758A1		-	-	Frame_Shift_Del	INS	-	-	-	PIK3R1	c.622del	p.S208Lfs*2
118H2758A1		-	-	Frame_Shift_Del	INS	-	-	-	APC	c.5455_5458del	p.N1819Pfs*43
118I2919A1		-	-	Frame_Shift_Del	INS	-	-	-	EPHA2	c.1429_1432del	p.G477Tfs*15
118I2919A1	12	133202852	133202852	Missense_Mutation	SNP	C	T	T	POLE	c.6382G>A	p.D2128N
118I2919A1	17	7577534	7577534	Missense_Mutation	SNP	C	A	A	TP53	c.747G>T	p.R249S
118I2919A1	2	61753601	61753601	Missense_Mutation	SNP	G	A	A	XPO1	c.182C>T	p.T61I
118I2919A1	2	141643827	141643827	Missense_Mutation	SNP	G	T	T	LRP1B	c.3844C>A	p.L1282I
118I2919A1	6	41909240	41909240	Missense_Mutation	SNP	G	C	C	CCND3	c.148C>G	p.R50G
118I2919A1	X	123022478	123022478	Missense_Mutation	SNP	A	G	G	XIAP	c.887A>G	p.D296G
118I2978M1	15	67473693	67473693	Missense_Mutation	SNP	A	G	G	SMAD3	c.773A>G	p.D258G
118I2978M1	18	48575192	48575192	Missense_Mutation	SNP	A	G	G	SMAD4	c.386A>G	p.N129S
118I2978M1		-	-	Frame_Shift_Del	INS	-	-	-	PBRM1	C.3507del	P.I1170Sfs*23
118I2978M1		-	-	Frame_Shift_Ins	INS	-	-	-	ARID1B	C.1400_1410dup	P.A471Sfs*35
118I2978M1	X	44921964	44921964	Missense_Mutation	SNP	A	G	G	KDM6A	c.1498A>G	p.S500G
118I3162A1	1	120050217	120050217	Missense_Mutation	SNP	G	A	A	HSD3B1	c.118G>A	p.G40R
118I3162A1	12	25398284	25398284	Missense_Mutation	SNP	C	T	T	KRAS	c.35G>A	p.G12D
118I3162A1	17	7576928	7576928	Splice_Site	SNP	T	A	A	TP53	c.920-2A>T	-
118I3162A1	19	11143991	11143991	Missense_Mutation	SNP	A	G	G	SMARCA4	c.3572A>G	p.H1191R
118I3162A1	20	57484420	57484420	Missense_Mutation	SNP	C	T	T	GNAS	c.601C>T	p.R201C
118I3162A1	22	41574029	41574029	Missense_Mutation	SNP	G	T	T	EP300	c.6314G>T	p.G2105V
118I3162A1	4	20525511	20525511	Missense_Mutation	SNP	G	A	A	SLIT2	c.1259G>A	p.R420Q
118I3162A1	6	93979390	93979390	Splice_Site	SNP	A	G	G	EPHA7	c.1450-12T>C	-
118I3162A1	6	117718076	117718076	Splice_Site	SNP	A	T	T	ROS1	c.779+2T>A	-
118I3162A1		-	-	Frame_Shift_Del	INS	-	-	-	BCORL1	C.2495del	P.G832Afs*21
118I3202A1	12	49448150	49448150	Nonsense_Mutation	SNP	C	T	T	KMT2D	c.450G>A	p.W150*
118I3202A1	15	91312768	91312768	Missense_Mutation	SNP	G	T	T	BLM	c.2507G>T	p.R836M
118I3202A1	7	140453136	140453136	Missense_Mutation	SNP	A	T	T	BRAF	c.1799T>A	p.V600E
118I3202A1	9	98211397	98211397	Missense_Mutation	SNP	T	G	G	PTCH1	c.3758A>C	p.Q1253P
118I3202A1	X	129159159	129159159	Missense_Mutation	SNP	C	A	A	BCORL1	c.3883C>A	p.L1295M
118I3402A1	19	11135115	11135115	Splice_Site	SNP	G	A	A	SMARCA4	c.3081+1G>A	-
118I3426A1	1	7724489	7724489	Missense_Mutation	SNP	G	A	A	CAMTA1	c.1882G>A	p.E628K
118I3426A1	1	204394048	204394048	Nonsense_Mutation	SNP	C	A	A	PIK3C2B	c.4837G>T	p.E1613*
118I3426A1	12	56488302	56488302	Missense_Mutation	SNP	G	C	C	ERBB3	c.1821G>C	p.Q607H
118I3426A1	14	95592940	95592940	Missense_Mutation	SNP	C	G	G	DICER1	c.880G>C	p.D294H
118I3426A1	17	7578275	7578275	Nonsense_Mutation	SNP	G	A	A	TP53	c.574C>T	p.Q192*

118I3426A1	2	16082426	16082426	Missense_Mutation	SNP	G	C	C	MYCN	c.240G>C	p.E80D
118I3426A1	2	74275364	74275364	Missense_Mutation	SNP	C	T	T	TET3	c.2320C>T	p.H774Y
118I3426A1	2	141215176	141215176	Missense_Mutation	SNP	C	T	T	LRP1B	c.9670G>A	p.E3224K
118I3426A1	20	31023588	31023588	Missense_Mutation	SNP	A	G	G	ASXL1	c.3073A>G	p.K1025E
118I3426A1	22	21345973	21345973	Missense_Mutation	SNP	G	A	A	LZTR1	c.848G>A	p.R283Q
118I3426A1	3	49939840	49939840	Missense_Mutation	SNP	G	T	T	MST1R	c.1203C>A	p.F401L
118I3426A1	3	71050211	71050211	Splice_Site	SNP	C	G	G	FOXP1	c.975-1G>C	-
118I3426A1	5	112176873	112176873	Missense_Mutation	SNP	C	T	T	APC	c.5582C>T	p.S1861F
118I3426A1	6	152332800	152332800	Missense_Mutation	SNP	A	T	T	ESR1	c.1106A>T	p.D369V
118I3426A1	6	163983083	163983083	Missense_Mutation	SNP	G	A	A	QKI	c.616G>A	p.D206N
118I3426A1	7	18630059	18630059	Missense_Mutation	SNP	G	A	A	HDAC9	c.356G>A	p.R119H
118I3426A1	7	41730075	41730075	Missense_Mutation	SNP	G	A	A	INHBA	c.454C>T	p.R152C
118I3426A1	7	86468307	86468307	Missense_Mutation	SNP	G	C	C	GRM3	c.1477G>C	p.D493H
118I3426A1	7	117243617	117243617	Missense_Mutation	SNP	C	A	A	CFTR	c.2689C>A	p.H897N
118I3426A1	7	141430086	141430086	Splice_Site	SNP	G	C	C	WEE2	c.1679-9G>C	-
118I3426A1	9	21971036	21971036	Missense_Mutation	SNP	C	T	T	CDKN2A	c.322G>A	p.D108N
118I3426A1	X	66905906	66905906	Missense_Mutation	SNP	G	A	A	AR	c.1823G>A	p.R608Q
118I3426A1	X	76855029	76855029	Missense_Mutation	SNP	T	C	C	ATRX	c.5807A>G	p.K1936R
118I3457A2		-	-	Frame_Shift_Ins	INS	-	-	-	ROCK1	C.3921dup	P.W1308Mfs*11
118I3457A2		-	-	In_Frame_Del	INS	-	-	-	BAP1	C.149_150del	P.F50*
118I3472M1	4	20598129	20598129	Missense_Mutation	SNP	G	A	A	SLIT2	c.3412G>A	p.V1138I
118I3472M1	6	152265322	152265322	Nonsense_Mutation	SNP	C	T	T	ESR1	c.775C>T	p.R259*
118I3488A1	12	25398284	25398284	Missense_Mutation	SNP	C	A	A	KRAS	c.35G>T	p.G12V
118I3488A1	2	198266835	198266835	Missense_Mutation	SNP	C	A	A	SF3B1	2097G>T;2098A	).[Q699H;K700E]
118I3488A1	3	30715615	30715615	Missense_Mutation	SNP	A	G	G	TGFBR2	c.1273A>G	p.M425V
118I3488A1		-	-	Frame_Shift_Del	INS	-	-	-	RBM10	c.2581del	p.I861Lfs*25
118I3510A1	1	11187848	11187848	Missense_Mutation	SNP	T	C	C	MTOR	c.6049A>G	p.I2017V
118I3510A1		-	-	Frame_Shift_Del	INS	-	-	-	EPHA2	c.1379del	p.P460Rfs*33
118I3510A1	10	89692800	89692800	Missense_Mutation	SNP	C	A	A	PTEN	c.284C>A	p.P95Q
118I3510A1	11	118374156	118374156	Missense_Mutation	SNP	G	A	A	KMT2A	c.7549G>A	p.A2517T
118I3510A1	12	416835	416835	Missense_Mutation	SNP	G	A	A	KDM5A	c.3715C>T	p.R1239W
118I3510A1		-	-	Frame_Shift_Del	INS	-	-	-	ARID2	c.951_954del	p.L317Ffs*10
118I3510A1		-	-	Frame_Shift_Del	INS	-	-	-	KMT2D	c.7061del	p.P2354Lfs*30
118I3510A1		-	-	Frame_Shift_Del	INS	-	-	-	BRCA2	c.5351del	p.N1784Tfs*7
118I3510A1	17	7579312	7579312	Missense_Mutation	SNP	C	T	T	TP53	c.375G>A	p.T125T
118I3510A1	17	8110622	8110622	Missense_Mutation	SNP	G	A	A	AURKB	c.270C>T	p.N90N
118I3510A1	17	12044500	12044500	Missense_Mutation	SNP	G	A	A	MAP2K4	c.1123G>A	p.V375I
118I3510A1	17	37881332	37881332	Missense_Mutation	SNP	G	A	A	ERBB2	c.2524G>A	p.V842I
118I3510A1	17	63533790	63533790	Missense_Mutation	SNP	G	A	A	AXIN2	c.1364C>T	p.P455L
118I3510A1	17	70120496	70120496	Nonsense_Mutation	SNP	C	T	T	SOX9	c.1498C>T	p.Q500*
118I3510A1		-	-	Frame_Shift_Del	INS	-	-	-	ACVR2A	c.1310del	p.K437Rfs*5
118I3510A1		-	-	Frame_Shift_Del	INS	-	-	-	TGFBR2	c.383del	p.K128Sfs*35
118I3510A1	4	55964878	55964878	Missense_Mutation	SNP	G	A	A	KDR	c.2359C>T	p.R787W
118I3510A1	4	66356202	66356202	Missense_Mutation	SNP	A	C	C	EPHA5	c.1295T>G	p.L432R
118I3510A1		-	-	Frame_Shift_Del	INS	-	-	-	PIK3R1	c.607del	p.Y203Tfs*4
118I3510A1		-	-	Frame_Shift_Del	INS	-	-	-	CTNNA1	c.2213del	p.N738Ifs*53
118I3510A1	6	135518403	135518403	Missense_Mutation	SNP	T	C	C	MYB	c.1508T>C	p.V503A
118I3510A1		-	-	In_Frame_Del	INS	-	-	-	MAGI2	c.3207_3208del	p.Y1069*
118I3510A1	7	151836767	151836767	Missense_Mutation	SNP	T	C	C	KMT2C	c.14453A>G	p.E4818G
118I3510A1	7	151878034	151878034	Missense_Mutation	SNP	A	G	G	KMT2C	c.6911T>C	p.M2304T
118I3510A1	7	152007121	152007121	Missense_Mutation	SNP	A	G	G	KMT2C	c.779T>C	p.L260P
118I3510A1	8	71078959	71078959	Missense_Mutation	SNP	G	A	A	NCOA2	c.572C>T	p.P191L
118I3510A1	8	139164508	139164508	Missense_Mutation	SNP	G	C	C	FAM135B	c.2210C>G	p.T737R
118I3510A1	9	5126729	5126729	Missense_Mutation	SNP	C	T	T	JAK2	c.3337C>T	p.R1113C
118I3510A1	9	87570290	87570290	Missense_Mutation	SNP	C	T	T	NTRK2	c.2030C>T	p.A677V
118I3529A1	1	16482332	16482332	Splice_Site	SNP	C	T	T	EPHA2	c.85+11G>A	-
118I3529A1	11	92616148	92616148	Missense_Mutation	SNP	C	T	T	FAT3	c.12526C>T	p.R4176C
118I3529A1	17	7578460	7578460	Missense_Mutation	SNP	A	C	C	TP53	c.470T>G	p.V157G
118I3529A1		-	-	Frame_Shift_Del	INS	-	-	-	SMAD4	c.681del	p.S227Rfs*14
118I3529A1	4	55152038	55152038	Missense_Mutation	SNP	G	A	A	PDGFRA	c.2470G>A	p.V824I
118I3529A1	9	21971203	21971203	Missense_Mutation	SNP	A	T	T	CDKN2A	c.155T>A	p.M52K
118I3529A1		-	-	Frame_Shift_Del	INS	-	-	-	RBM10	c.2118_2121del	p.R706Sfs*95
118I3532A1	12	92538065	92538065	Missense_Mutation	SNP	T	A	A	BTG1	c.307A>T	p.T103S
118I3532A1	17	7577548	7577548	Missense_Mutation	SNP	C	T	T	TP53	c.733G>A	p.G245S
118I3532A1	18	48604665	48604665	Missense_Mutation	SNP	G	A	A	SMAD4	c.1487G>A	p.R496H
118I3532A1		-	-	Frame_Shift_Ins	INS	-	-	-	TGFBR2	c.229_232dup	p.E78Vfs*2
118I3532A1	9	5126342	5126342	Missense_Mutation	SNP	C	T	T	JAK2	c.3187C>T	p.R1063C
118I3573A1		-	-	Missense_Mutation	SNP	-	-	-	TET1	C.5877_5879de	P.E1959del
118I3573A1	12	493228	493228	Missense_Mutation	SNP	C	G	G	KDM5A	c.335G>C	p.R112T
118I3573A1	17	7577575	7577575	Missense_Mutation	SNP	A	G	G	TP53	c.706T>C	p.Y236H
118I3573A1		-	-	Frame_Shift_Ins	INS	-	-	-	STK11	C.842dup	P.L282Afs*3
118I3573A1	3	178936082	178936082	Missense_Mutation	SNP	G	A	A	PIK3CA	c.1624G>A	p.E542K
118I3573A1	3	178936980	178936980	Splice_Site	SNP	A	G	G	PIK3CA	c.1665-4A>G	-
118I3573A1	8	37555773	37555773	Missense_Mutation	SNP	G	A	A	ZNF703	c.1354G>A	p.E452K
118I3576A1	12	25398284	25398284	Missense_Mutation	SNP	C	G	G	KRAS	c.35G>C	p.G12A
118I3576A1	17	7577142	7577142	Missense_Mutation	SNP	C	T	T	TP53	c.796G>A	p.G266R
118I3592A2		-	-	Frame_Shift_Del	INS	-	-	-	ARID1A	c.3893del	p.G1298Efs*3
118I3592A2	1	156845877	156845877	Missense_Mutation	SNP	C	T	T	NTRK1	c.1489C>T	p.H497Y
118I3592A2	1	241682991	241682991	Missense_Mutation	SNP	G	C	C	FH	c.32C>G	p.S11W
118I3592A2	10	89624289	89624289	Missense_Mutation	SNP	C	G	G	PTEN	c.63C>G	p.F21L
118I3592A2	11	92526050	92526050	Missense_Mutation	SNP	G	C	C	FAT3	c.4729G>C	p.E1577Q
118I3592A2	12	56474020	56474020	Splice_Site	SNP	C	G	G	ERBB3	c.-65C>G	-
118I3592A2	12	56481922	56481922	Missense_Mutation	SNP	G	A	A	ERBB3	c.850G>A	p.G284R
118I3592A2	15	91312441	91312441	Missense_Mutation	SNP	G	A	A	BLM	c.2386G>A	p.E796K
118I3592A2	18	48603023	48603023	Nonsense_Mutation	SNP	C	T	T	SMAD4	c.1324C>T	p.Q442*
118I3592A2	19	11144536	11144536	Missense_Mutation	SNP	C	G	G	SMARCA4	c.3868C>G	p.L1290V
118I3592A2	20	62338444	62338444	Splice_Site	SNP				ARFRP1	c.-1-3C>A	-
118I3592A2	3	178936091	178936091	Missense_Mutation	SNP	G	A	A	PIK3CA	c.1633G>A	p.E545K
118I3592A2	4	126412807	126412807	Missense_Mutation	SNP	G	A	A	FAT4	c.14830G>A	p.V4944I
118I3592A2	7	55218984	55218984	Splice_Site	SNP	C	T	T	EGFR	c.560-3C>T	-
118I3592A2	8	71068334	71068334	Missense_Mutation	SNP	C	G	G	NCOA2	c.2266G>C	p.D756H
118I3592A2	9	101894916	101894916	Nonsense_Mutation	SNP	C	T	T	TGFBR1	c.469C>T	p.R157*
118I3592A2	X	44936067	44936067	Missense_Mutation	SNP	T	G	G	KDM6A	c.2828T>G	p.I943S
118I3592A2	X	47430749	47430749	Missense_Mutation	SNP	C	T	T	ARAF	c.1714C>T	p.R572W
118I3607A2	1	158589112	158589112	Missense_Mutation	SNP	C	G	G	SPTA1	c.6430G>C	p.E2144Q
118I3607A2	1	158613109	158613109	Splice_Site	SNP	G	C	C	SPTA1	c.4442+3C>G	-
118I3607A2		-	-	Frame_Shift_Ins	INS	-	-	-	TP53	C.902dup	P.G302Rfs*4
118I3607A2	17	37646932	37646932	Missense_Mutation	SNP	C	G	G	CDK12	c.2054C>G	p.S685C

118I3607A2	18	45372090	45372090	Missense_Mutation	SNP	G	T	T	SMAD2	c.989C>A	p.P330H
118I3607A2	19	41727883	41727883	Missense_Mutation	SNP	G	C	C	AXL	c.508G>C	p.D170H
118I3607A2	2	121746932	121746932	Missense_Mutation	SNP	G	A	A	GLI2	c.3442G>A	p.E1148K
118I3607A2	20	57429319	57429319	Missense_Mutation	SNP	C	T	T	GNAS	c.812C>T	p.T271M
118I3607A2	3	52692343	52692343	Splice_Site	SNP	G	T	T	PBRM1	c.529-12C>A	-
118I3607A2	3	178919314	178919314	Missense_Mutation	SNP	C	A	A	PIK3CA	c.799C>A	p.L267M
118I3607A2	4	185311889	185311889	Missense_Mutation	SNP	C	T	T	IRF2	c.709G>A	p.D237N
118I3607A2		-	-	Frame_Shift_Del	INS	-	-	-	MAGI2	C.3111del	P.L1039Wfs*35
118I3607A2	7	151945106	151945106	Missense_Mutation	SNP	T	C	C	KMT2C	c.2413A>G	p.S805G
118I3607A2	9	21971036	21971036	Missense_Mutation	SNP	C	T	T	CDKN2A	c.322G>A	p.D108N
118I3607A2	X	47045464	47045464	Nonsense_Mutation	SNP	C	T	T	RBM10	c.2431C>T	p.Q811*
118I3629A1		-	-	Frame_Shift_Del	INS	-	-	-	STK11	C.153_157del	P.M51Ifs*110
118I3652A1	10	70451566	70451566	Missense_Mutation	SNP	G	T	T	TET1	c.6406G>T	p.V2136F
118I3652A1	12	25398284	25398284	Missense_Mutation	SNP	C	T	T	KRAS	c.35G>A	p.G12D
118I3652A1		-	-	Frame_Shift_Del	INS	-	-	-	STK11	C.64_92del	P.M22Hfs*131
118I3652A1	2	141641582	141641582	Missense_Mutation	SNP	C	A	A	LRP1B	c.3973G>T	p.A1325S
118I3652A1	2	141751692	141751692	Missense_Mutation	SNP	T	G	G	LRP1B	c.2516A>C	p.E839A
118I3743M1		-	-	Frame_Shift_Ins	INS	-	-	-	EPHA2	:.1199_1203dup	p.L402Afs*7
118I3743M1	1	27100292	27100292	Splice_Site	SNP	G	A	A	ARID1A	c.4005-1G>A	-
118I3743M1	14	45623981	45623981	Missense_Mutation	SNP	G	A	A	FANCM	c.1265G>A	p.R422H
118I3743M1	17	7578457	7578457	Missense_Mutation	SNP	C	T	T	TP53	c.473G>A	p.R158H
118I3743M1		-	-	Frame_Shift_Ins	INS	-	-	-	SMAD4	c.692dup	p.S232Qfs*3
118I3743M1	20	57485014	57485014	Missense_Mutation	SNP	G	A	A	GNAS	c.848G>A	p.R283H
118I3743M1		-	-	Frame_Shift_Del	INS	-	-	-	KMT2C	c.4937del	p.G1646Vfs*19
118I3752D1	11	64573752	64573752	Missense_Mutation	SNP	A	C	C	MEN1	c.1001T>G	p.V334G
118I3752D1	12	69203080	69203080	Splice_Site	SNP	T	C	C	MDM2	c.99+8T>C	-
118I3752D1	13	43175020	43175020	Missense_Mutation	SNP	G	A	A	TNFSF11	c.216G>A	p.A72A
118I3752D1	17	17116985	17116985	Missense_Mutation	SNP	G	A	A	FLCN	c.1724C>T	p.S575L
118I3752D1	17	41244192	41244192	Missense_Mutation	SNP	G	C	C	BRCA1	c.3356C>G	p.T1119S
118I3752D1	19	10602748	10602748	Missense_Mutation	SNP	G	T	T	KEAP1	c.830C>A	p.T277K
118I3752D1	2	112751824	112751824	Splice_Site	SNP	C	A	A	MERTK	c.1297-4C>A	-
118I3752D1	3	69987112	69987112	Missense_Mutation	SNP	G	T	T	MITF	c.173G>T	p.G58V
118I3752D1	4	106197291	106197291	Missense_Mutation	SNP	G	A	A	TET2	c.5624G>A	p.C1875Y
118I3752D1	9	21971179	21971179	Missense_Mutation	SNP	G	A	A	CDKN2A	c.179C>T	p.A60V
118I3763A1		-	-	Frame_Shift_Del	INS	-	-	-	KMT2D	C.11684del	P.G3895Afs*84
118I3763A1	12	133257726	133257726	Missense_Mutation	SNP	G	A	A	POLE	c.202C>T	p.P68S
118I3763A1	16	3788629	3788629	Missense_Mutation	SNP	G	A	A	CREBBP	c.4325C>T	p.P1442L
118I3763A1	17	7577609	7577609	Splice_Site	SNP	C	A	A	TP53	c.673-1G>T	-
118I3763A1	2	141299380	141299380	Missense_Mutation	SNP	C	T	T	LRP1B	c.7355G>A	p.G2452E
118I3763A1	4	55991418	55991418	Missense_Mutation	SNP	C	T	T	KDR	c.43G>A	p.V15M
118I3763A1	X	39921616	39921616	Nonsense_Mutation	SNP	C	A	A	BCOR	c.4102G>T	p.E1368*
118I3817A1		-	-	In_Frame_Del	INS	-	-	-	TP53	C.789_803del	P.L264_N268del
118I3817A1	X	44918604	44918604	Missense_Mutation	SNP	G	A	A	KDM6A	c.1087G>A	p.E363K
118I3847A1	12	25398284	25398284	Missense_Mutation	SNP	C	A	A	KRAS	c.35G>T	p.G12V
118I3847A1	13	26956990	26956990	Nonsense_Mutation	SNP	C	T	T	CDK8	c.496C>T	p.R166*
118I3847A1	17	7577570	7577570	Missense_Mutation	SNP	C	T	T	TP53	c.711G>A	p.M237I
118I3847A1	22	29664332	29664332	Missense_Mutation	SNP	T	C	C	EWSR1	c.7T>C	p.S3P
118I3847A1	6	117681042	117681042	Missense_Mutation	SNP	T	A	A	ROS1	c.3578A>T	p.N1193I
118I3847A1	7	2959024	2959024	Missense_Mutation	SNP	G	A	A	CARD11	c.2492C>T	p.T831I
118I3850D1	16	23646483	23646483	Missense_Mutation	SNP	C	G	G	PALB2	c.1384G>C	p.E462Q
118I3850D1		-	-	Frame_Shift_Del	INS	-	-	-	PALB2	054_1056delins	p.E352Kfs*4
118I3850D1		-	-	Frame_Shift_Del	INS	-	-	-	BAP1	C.714del	P.I239Sfs*10
118I3860A1	1	27023685	27023685	Nonsense_Mutation	SNP	C	A	A	ARID1A	c.791C>A	p.S264*
118I3860A1	11	92534203	92534203	Missense_Mutation	SNP	C	T	T	FAT3	c.8024C>T	p.S2675L
118I3860A1	12	25398282	25398282	Missense_Mutation	SNP	C	A	A	KRAS	c.37G>T	p.G13C
118I3860A1	19	1220485	1220485	Missense_Mutation	SNP	C	G	G	STK11	c.578C>G	p.S193C
118I3860A1	19	1220629	1220629	Missense_Mutation	SNP	C	T	T	STK11	c.647C>T	p.S216F
118I3860A1	20	57429128	57429128	Missense_Mutation	SNP	G	A	A	GNAS	c.808G>A	p.A270T
118I3860A1	4	55980357	55980357	Missense_Mutation	SNP	T	A	A	KDR	c.734A>T	p.N245I
118I3860A1	7	151945622	151945622	Nonsense_Mutation	SNP	C	A	A	KMT2C	c.1897G>T	p.E633*
118I3960A1	1	32741508	32741508	Splice_Site	SNP	C	G	G	LCK	c.482-7C>G	-
118I3960A1	1	120471846	120471846	Splice_Site	SNP	G	C	C	NOTCH2	c.3656-11C>G	-
118I3960A1	1	150551604	150551604	Missense_Mutation	SNP	C	G	G	MCL1	c.403G>C	p.G135R
118I3960A1	12	133209059	133209059	Missense_Mutation	SNP	C	G	G	POLE	c.6172G>C	p.E2058Q
118I3960A1	14	95570075	95570075	Missense_Mutation	SNP	G	C	C	DICER1	c.3658C>G	p.Q1220E
118I3960A1	16	2129377	2129377	Missense_Mutation	SNP	C	T	T	TSC2	c.3232C>T	p.R1078W
118I3960A1	16	9858439	9858439	Missense_Mutation	SNP	G	C	C	GRIN2A	c.2962C>G	p.L988V
118I3960A1	16	89871757	89871757	Missense_Mutation	SNP	G	C	C	FANCA	c.640C>G	p.L214V
118I3960A1	17	7577099	7577099	Missense_Mutation	SNP	C	T	T	TP53	c.839G>A	p.R280K
118I3960A1	17	29556139	29556139	Missense_Mutation	SNP	G	A	A	NF1	c.2506G>A	p.E836K
118I3960A1	17	29557890	29557890	Nonsense_Mutation	SNP	G	A	A	NF1	c.3144G>A	p.W1048*
118I3960A1	17	33443866	33443866	Splice_Site	SNP	G	A	A	RAD51D	c.323+12C>T	-
118I3960A1	17	40489460	40489460	Nonsense_Mutation	SNP	C	A	A	STAT3	c.790G>T	p.E264*
118I3960A1	19	10599973	10599973	Missense_Mutation	SNP	C	G	G	KEAP1	c.1603G>C	p.E535Q
118I3960A1	19	10600378	10600378	Missense_Mutation	SNP	C	G	G	KEAP1	c.1477G>C	p.E493Q
118I3960A1	2	170060716	170060716	Missense_Mutation	SNP	T	C	C	LRP2	c.7781A>G	p.H2594R
118I3960A1	6	117641019	117641019	Splice_Site	SNP	G	A	A	ROS1	c.5940+12C>T	-
118I3960A1	8	68995502	68995502	Missense_Mutation	SNP	A	T	T	PREX2	c.1906A>T	p.I636F
118I3960A1	9	5044518	5044518	Nonsense_Mutation	SNP	C	T	T	JAK2	c.466C>T	p.Q156*
118I3967M1	1	27087440	27087440	Nonsense_Mutation	SNP	G	T	T	ARID1A	c.2014G>T	p.E672*
118I3967M1	1	46726267	46726267	Missense_Mutation	SNP	G	A	A	RAD54L	c.461G>A	p.R154Q
118I3967M1	1	158612287	158612287	Missense_Mutation	SNP	G	C	C	SPTA1	c.4651C>G	p.R1551G
118I3967M1	12	25398284	25398284	Missense_Mutation	SNP	C	T	T	KRAS	c.35G>A	p.G12D
118I3967M1	16	81888057	81888057	Missense_Mutation	SNP	A	T	T	PLCG2	c.202A>T	p.M68L
118I3967M1	19	10599916	10599916	Nonsense_Mutation	SNP	G	A	A	KEAP1	c.1660C>T	p.R554*
118I3967M1	19	17952448	17952448	Splice_Site	SNP	C	A	A	JAK3	c.984+1G>T	-
118I3967M1	4	126328026	126328026	Splice_Site	SNP	T	C	C	FAT4	c.5308-9T>C	-
118I3967M1	5	35873749	35873749	Missense_Mutation	SNP	A	C	C	IL7R	c.705A>C	p.S235S
118I3967M1	5	112128185	112128185	Missense_Mutation	SNP	C	T	T	APC	c.688C>T	p.R230C
118I3967M1		-	-	In_Frame_Ins	INS	-	-	-	ARID1B	c.363_368dup	.Q130_Q131dup
118I3967M1	6	157505488	157505488	Nonsense_Mutation	SNP	G	T	T	ARID1B	c.3469G>T	p.E1157*
118I3967M1	8	71041103	71041103	Missense_Mutation	SNP	C	G	G	NCOA2	c.3437G>C	p.G1146A
118I3967M1	9	133760285	133760285	Missense_Mutation	SNP	G	A	A	ABL1	c.2665G>A	p.A889T
118I3967M1	9	139391265	139391265	Missense_Mutation	SNP	T	A	A	NOTCH1	c.6926A>T	p.E2309V
118I3967M1	9	139409801	139409801	Missense_Mutation	SNP	G	A	A	NOTCH1	c.1955C>T	p.S652L
118I3967M1		-	-	Frame_Shift_Ins	INS	-	-	-	RBM10	c.627dup	p.K210Qfs*6
118I4062A1		-	-	Frame_Shift_Ins	INS	-	-	-	ARID1A	C.3216dup	P.W1073Mfs*32
118I4062A1	1	206658350	206658350	Nonsense_Mutation	SNP	G	T	T	IKBKE	c.1444G>T	p.G482*

118I4062A1	12	12871086	12871086	Nonsense_Mutation	SNP	G	T	T	CDKN1B	c.313G>T	p.E105*
118I4062A1	12	25398284	25398284	Missense_Mutation	SNP	C	T	T	KRAS	c.35G>A	p.G12D
118I4062A1	17	7578394	7578394	Missense_Mutation	SNP	T	A	A	TP53	c.536A>T	p.H179L
118J4224A1		-	-	Splice_Site	SNP	-	-	-	KMT2A	c.3569+1del	-
118J4224A1		-	-	Frame_Shift_Ins	INS	-	-	-	TP53	c.120_121dup	p.D41Gfs*4
118J4224A1	2	140995831	140995831	Missense_Mutation	SNP	C	T	T	LRP1B	c.13450G>A	p.G4484R
118J4224A1		-	-	Splice_Site	SNP	-	-	-	EGFR	2185-9_2185-8d	-
118J4224A1	9	98215869	98215869	Missense_Mutation	SNP	T	A	A	PTCH1	c.3340A>T	p.R1114W
118J4228A1	1	8074231	8074231	Missense_Mutation	SNP	G	A	A	ERRFI1	c.428C>T	p.A143V
118J4228A1	1	11259456	11259456	Missense_Mutation	SNP	G	C	C	MTOR	c.4112C>G	p.P1371R
118J4228A1	1	27106354	27106354	Nonsense_Mutation	SNP	C	T	T	ARID1A	c.5965C>T	p.R1989*
118J4228A1	12	6692520	6692520	Missense_Mutation	SNP	T	C	C	CHD4	c.3904A>G	p.I1302V
118J4228A1	17	7577538	7577538	Missense_Mutation	SNP	C	T	T	TP53	c.743G>A	p.R248Q
118J4228A1	18	48593406	48593406	Missense_Mutation	SNP	G	T	T	SMAD4	c.1157G>T	p.G386V
118J4228A1	2	47643508	47643508	Missense_Mutation	SNP	A	C	C	MSH2	c.1016A>C	p.Q339P
118J4228A1	7	81339562	81339562	Splice_Site	SNP	G	A	A	HGF	c.1445-3C>T	-
118J4228A1	X	39932447	39932447	Missense_Mutation	SNP	C	T	T	BCOR	c.2152G>A	p.D718N
118J4287M1	1	16262141	16262141	Missense_Mutation	SNP	C	T	T	SPEN	c.9406C>T	p.R3136C
118J4287M1	10	84738818	84738818	Missense_Mutation	SNP	G	A	A	NRG3	c.1525G>A	p.A509T
118J4287M1	10	123274774	123274774	Missense_Mutation	SNP	A	G	G	FGFR2	c.1144T>C	p.C382R
118J4287M1	14	105239435	105239435	Splice_Site	SNP	C	T	T	AKT1	c.958-6G>A	-
118J4287M1	16	9923507	9923507	Missense_Mutation	SNP	G	A	A	GRIN2A	c.1780C>T	p.P594S
118J4287M1	17	7579358	7579358	Missense_Mutation	SNP	C	T	T	TP53	c.329G>A	p.R110H
118J4287M1	17	56440640	56440640	Missense_Mutation	SNP	G	A	A	RNF43	c.578C>T	p.A193V
118J4287M1	2	48030630	48030630	Missense_Mutation	SNP	C	T	T	MSH6	c.3244C>T	p.P1082S
118J4287M1	2	61753549	61753549	Splice_Site	SNP	G	A	A	XPO1	c.228+6C>T	-
118J4287M1	2	109368337	109368337	Missense_Mutation	SNP	G	T	T	RANBP2	c.1642G>T	p.V548L
118J4287M1	2	141641379	141641379	Splice_Site	SNP	T	G	G	LRP1B	c.4169+7A>C	-
118J4287M1	3	52441235	52441235	Missense_Mutation	SNP	G	A	A	BAP1	c.535C>T	p.R179W
118J4287M1	3	142275266	142275266	Missense_Mutation	SNP	C	G	G	ATR	c.2037G>C	p.L679F
118J4287M1	3	178916930	178916930	Missense_Mutation	SNP	G	T	T	PIK3CA	c.317G>T	p.G106V
118J4287M1	3	185775016	185775016	Missense_Mutation	SNP	G	C	C	ETV5	c.1057C>G	p.P353A
118J4287M1	4	126372111	126372111	Missense_Mutation	SNP	G	T	T	FAT4	c.9940G>T	p.G3314C
118J4287M1	7	117243698	117243698	Missense_Mutation	SNP	G	A	A	CFTR	c.2770G>A	p.D924N
118J4287M1	8	48811106	48811106	Missense_Mutation	SNP	C	A	A	PRKDC	c.3388G>T	p.A1130S
118J4299M1		-	-	Frame_Shift_Del	INS	-	-	-	ARID1A	c.5198_5204del	p.E1733Gfs*35
118J4299M1		-	-	Frame_Shift_Del	INS	-	-	-	TP53	c.956del	p.K319Rfs*26
118J4299M1		-	-	Splice_Site	SNP	-	-	-	EGFR	c.1881-6dup	-
118J4384A1		-	-	Splice_Site	SNP	-	-	-	BAP1	c.67+2_67+4del	-
118J4450M1		-	-	Frame_Shift_Ins	INS	-	-	-	ERRFI1	c.336_339dup	p.G114Mfs*4
118J4450M1		-	-	Frame_Shift_Del	INS	-	-	-	ARID1A	c.2186_2196del	p.P729Lfs*84
118J4450M1	11	108213974	108213974	Missense_Mutation	SNP	G	T	T	ATM	c.8294G>T	p.G2765V
118J4450M1	8	32505402	32505402	Missense_Mutation	SNP	G	A	A	NRG1	c.166G>A	p.V56M
118J4450M1	X	47041442	47041442	Splice_Site	SNP	G	A	A	RBM10	c.1785+1G>A	-
118J4625A1	12	25398285	25398285	Missense_Mutation	SNP	C	A	A	KRAS	c.34G>T	p.G12C
118J4625A1		-	-	Frame_Shift_Ins	INS	-	-	-	KMT2D	c.15539dup	p.L5183Pfs*16
118J4625A1	2	170066064	170066064	Missense_Mutation	SNP	G	A	A	LRP2	c.6368C>T	p.A2123V
118J4625A1	2	198266713	198266713	Missense_Mutation	SNP	C	T	T	SF3B1	c.2219G>A	p.G740E
118J4646M1	1	158614125	158614125	Missense_Mutation	SNP	C	G	G	SPTA1	c.4256G>C	p.R1419T
118J4646M1	11	125514167	125514167	Splice_Site	SNP	A	T	T	CHEK1	c.1101+4A>T	-
118J4646M1	12	115112332	115112332	Missense_Mutation	SNP	C	T	T	TBX3	c.1408G>A	p.A470T
118J4646M1		-	-	In_Frame_Ins	INS	-	-	-	FLT1	c.2178_2180dup	p.*734Sext*1
118J4646M1		-	-	Frame_Shift_Del	INS	-	-	-	TP53	c.365_366del	p.V122Dfs*26
118J4646M1	17	48263861	48263861	Nonsense_Mutation	SNP	G	C	C	COL1A1	c.3822C>G	p.Y1274*
118J4646M1	19	40744823	40744823	Missense_Mutation	SNP	T	C	C	AKT2	c.697A>G	p.N233D
118J4646M1	2	25467502	25467502	Missense_Mutation	SNP	G	A	A	DNMT3A	c.1574C>T	p.A525V
118J4646M1	2	47690161	47690161	Splice_Site	SNP	T	G	G	MSH2	c.1387-9T>G	-
118J4646M1		-	-	Frame_Shift_Del	INS	-	-	-	BAP1	c.145del	p.L49Cfs*23
118J4646M1	7	117243635	117243635	Missense_Mutation	SNP	T	G	G	CFTR	c.2707T>G	p.Y903D
118J4748M1	19	11123691	11123691	Missense_Mutation	SNP	A	G	G	SMARCA4	c.2341A>G	p.M781V
118J4748M1	7	140453136	140453136	Missense_Mutation	SNP	A	T	T	BRAF	c.1799T>A	p.V600E
118J4748M1	8	139163664	139163664	Missense_Mutation	SNP	C	A	A	FAM135B	c.3054G>T	p.E1018D
118J4750A1	10	89692853	89692853	Missense_Mutation	SNP	A	C	C	PTEN	c.337A>C	p.S113R
118J4750A1	4	55593651	55593651	Missense_Mutation	SNP	C	T	T	KIT	c.1717C>T	p.P573S
118J4755M1	1	158618388	158618388	Missense_Mutation	SNP	C	T	T	SPTA1	c.3625G>A	p.A1209T
118J4755M1	17	7577568	7577568	Missense_Mutation	SNP	C	A	A	TP53	c.713G>T	p.C238F
118J4755M1	8	69030814	69030814	Missense_Mutation	SNP	C	G	G	PREX2	c.3356C>G	p.A1119G
118J4761A1	12	25398284	25398284	Missense_Mutation	SNP	C	T	T	KRAS	c.35G>A	p.G12D
118J4761A1		-	-	Frame_Shift_Del	INS	-	-	-	TP53	c.486del	p.Y163Tfs*7
118J4761A1		-	-	Frame_Shift_Ins	INS	-	-	-	FBXW7	c.1627dup	p.R543Kfs*8
118J4761A1	6	152265353	152265353	Missense_Mutation	SNP	G	A	A	ESR1	c.806G>A	p.R269H
118J4779J1		-	-	Splice_Site	SNP	-	-	-	ARID2	1715+2_1715+5	-
118J4779J1	15	66727442	66727442	Missense_Mutation	SNP	T	G	G	MAP2K1	c.158T>G	p.F53C
118J4779J1		-	-	Frame_Shift_Ins	INS	-	-	-	TP53	C.915dup	P.R306Afs*31
118J4779J1	19	10602367	10602367	Missense_Mutation	SNP	G	A	A	KEAP1	c.1211C>T	p.S404L
118J4779J1	5	112175897	112175897	Nonsense_Mutation	SNP	G	T	T	APC	c.4606G>T	p.E1536*
118J4845A1		-	-	Frame_Shift_Del	INS	-	-	-	ARID1A	C.5624del	P.P1875Hfs*8
118J4845A1	10	104592349	104592349	Missense_Mutation	SNP	A	T	T	CYP17A1	c.1058T>A	p.L353Q
118J4845A1	16	10032132	10032132	Missense_Mutation	SNP	A	T	T	GRIN2A	c.691T>A	p.C231S
118J4845A1	19	41745061	41745061	Splice_Site	SNP	C	G	G	AXL	c.1135-8C>G	-
118J4845A1	2	170089935	170089935	Missense_Mutation	SNP	G	A	A	LRP2	c.5084C>T	p.S1695L
118J4845A1	2	209113113	209113113	Missense_Mutation	SNP	G	A	A	IDH1	c.394C>T	p.R132C
118J4845A1	X	63411267	63411267	Missense_Mutation	SNP	G	A	A	AMER1	c.1900C>T	p.R634C
118J4887A1	1	7724066	7724066	Missense_Mutation	SNP	T	C	C	CAMTA1	c.1459T>C	p.C487R
118J4887A1	14	20823952	20823952	Missense_Mutation	SNP	C	T	T	PARP2	c.983C>T	p.S328L
118J4887A1	16	67116197	67116197	Missense_Mutation	SNP	C	T	T	CBFB	c.481C>T	p.R161W
118J4887A1	17	29533378	29533378	Nonsense_Mutation	SNP	C	T	T	NF1	c.1381C>T	p.R461*
118J4887A1	17	29576091	29576091	Nonsense_Mutation	SNP	C	G	G	NF1	c.4064C>G	p.S1355*
118J4887A1	18	48584552	48584552	Nonsense_Mutation	SNP	C	G	G	SMAD4	c.725C>G	p.S242*
118J4887A1		-	-	Splice_Site	SNP	-	-	-	ACVR2A	c.817-9_857del	-
118J4887A1	2	202149890	202149890	Nonsense_Mutation	SNP	T	A	A	CASP8	c.1205T>A	p.L402*
118J4887A1	3	178936091	178936091	Missense_Mutation	SNP	G	A	A	PIK3CA	c.1633G>A	p.E545K
118J4887A1	4	55956164	55956164	Missense_Mutation	SNP	G	A	A	KDR	c.3151C>T	p.R1051W
118J4887A1	7	148515112	148515112	Missense_Mutation	SNP	T	G	G	EZH2	c.1097A>C	p.N366T
118J4963M1	12	49421833	49421833	Missense_Mutation	SNP	C	T	T	KMT2D	c.14474G>A	p.R4825Q
118J4963M1	15	88476400	88476400	Missense_Mutation	SNP	T	C	C	NTRK3	c.1732A>G	p.T578A
118J4963M1		-	-	Frame_Shift_Del	INS	-	-	-	TP53	C.626_627del	P.R209Kfs*6
118J4963M1	17	29654575	29654575	Missense_Mutation	SNP	C	T	T	NF1	c.5327C>T	p.S1776L

118J4963M1	2	141356367	141356367	Missense_Mutation	SNP	T	G	G	LRP1B	c.7027A>C	p.S2343R
118J4963M1		-	-	In_Frame_Del	INS	-	-	-	ARID1B	C.363_380del	P.Q126_Q131del
118J5065A1	11	76162890	76162890	Splice_Site	SNP	T	A	A	EMSY	c.71-12T>A	-
118J5065A1	12	49446348	49446348	Missense_Mutation	SNP	T	C	C	KMT2D	c.1257A>G	p.L419L
118J5065A1	12	69968689	69968689	Missense_Mutation	SNP	G	T	T	FRS2	c.1481G>T	p.G494V
118J5065A1	13	49050868	49050868	Missense_Mutation	SNP	T	C	C	RB1	c.2552T>C	p.M851T
118J5065A1	17	7579494	7579494	Nonsense_Mutation	SNP	T	A	A	TP53	c.193A>T	p.R65*
118J5065A1	2	141986863	141986863	Missense_Mutation	SNP	T	C	C	LRP1B	c.739A>G	p.I247V
118J5065A1	20	57429815	57429815	Missense_Mutation	SNP	G	T	T	GNAS	c.1495G>T	p.A499S
118J5065A1		-	-	Frame_Shift_Del	INS	-	-	-	NOTCH4	C.989del	P.G330Afs*15
118J5083M1		-	-	Frame_Shift_Del	INS	-	-	-	ARID1A	delinsAGGTAGG	p.G314Rfs*48
118J5083M1	11	69458744	69458744	Missense_Mutation	SNP	G	A	A	CCND1	c.559G>A	p.A187T
118J5083M1	12	25398284	25398284	Missense_Mutation	SNP	C	T	T	KRAS	c.35G>A	p.G12D
118J5083M1	16	23646321	23646321	Nonsense_Mutation	SNP	T	A	A	PALB2	c.1546A>T	p.R516*
118J5083M1		-	-	Frame_Shift_Del	INS	-	-	-	SMAD4	C.321_330del	P.E108Mfs*11
118J5083M1	18	48581157	48581157	Nonsense_Mutation	SNP	C	G	G	SMAD4	c.461C>G	p.S154*
118J5083M1	19	42797344	42797344	Missense_Mutation	SNP	C	T	T	CIC	c.3706C>T	p.P1236S
118J5083M1	4	88979247	88979247	Missense_Mutation	SNP	A	T	T	PKD2	c.2011A>T	p.I671F
118J5083M1		-	-	In_Frame_Del	INS	-	-	-	TET2	C.3463_3479de	P.P1155*
118J5083M1		-	-	In_Frame_Del	INS	-	-	-	PIK3R1	C.1392_1403de	P.D464_Y467del
118J5083M1	7	148529748	148529748	Missense_Mutation	SNP	G	C	C	EZH2	c.341C>G	p.S114C
118J5085M1	1	98348861	98348861	Missense_Mutation	SNP	C	T	T	DPYD	c.109G>A	p.D37N
118J5085M1	12	56489534	56489534	Missense_Mutation	SNP	C	T	T	ERBB3	c.1999C>T	p.R667C
118J5085M1	15	93552474	93552474	Missense_Mutation	SNP	T	A	A	CHD2	c.4513T>A	p.C1505S
118J5085M1	2	209113113	209113113	Missense_Mutation	SNP	G	A	A	IDH1	c.394C>T	p.R132C
118J5119A1	10	43601922	43601922	Missense_Mutation	SNP	C	A	A	RET	c.966C>A	p.D322E
118J5119A1	17	7577539	7577539	Missense_Mutation	SNP	G	A	A	TP53	c.742C>T	p.R248W
118J5119A1	17	41607296	41607296	Missense_Mutation	SNP	C	T	T	ETV4	c.911G>A	p.R304Q
118J5119A1	2	140995765	140995765	Missense_Mutation	SNP	C	G	G	LRP1B	c.13516G>C	p.D4506H
118J5119A1	22	30074230	30074230	Missense_Mutation	SNP	T	A	A	NF2	c.1492T>A	p.F498I
118J5119A1	3	37081683	37081683	Missense_Mutation	SNP	G	A	A	MLH1	c.1565G>A	p.R522Q
118J5119A1	3	178921551	178921551	Missense_Mutation	SNP	A	G	G	PIK3CA	c.1033A>G	p.N345D
118J5119A1	5	38953598	38953598	Missense_Mutation	SNP	C	G	G	RICTOR	c.2755G>C	p.E919Q
118J5135A1	11	65427594	65427594	Splice_Site	SNP	C	A	A	RELA	c.427+1G>T	-
118J5135A1	11	92086687	92086687	Missense_Mutation	SNP	C	T	T	FAT3	c.1409C>T	p.P470L
118J5135A1	17	41219675	41219675	Missense_Mutation	SNP	G	A	A	BRCA1	c.5024C>T	p.T1675I
118J5135A1	3	52643347	52643347	Missense_Mutation	SNP	C	T	T	PBRM1	c.2549G>A	p.R850Q
118J5135A1		-	-	Splice_Site	SNP	-	-	-	PIK3R1	1746-28_1746-7	-
118J5135A1	6	112015644	112015644	Missense_Mutation	SNP	T	C	C	FYN	c.1207A>G	p.I403V
118J5135A1	8	92998483	92998483	Missense_Mutation	SNP	T	C	C	RUNX1T1	c.1037A>G	p.E346G
118J5144A1	17	37868208	37868208	Missense_Mutation	SNP	C	T	T	ERBB2	c.929C>T	p.S310F
118J5144A1	20	39751885	39751885	Missense_Mutation	SNP	G	A	A	TOP1	c.2246G>A	p.R749Q
118J5144A1	21	44524456	44524456	Missense_Mutation	SNP	G	A	A	U2AF1	c.101C>T	p.S34F
118J5144A1	5	112177203	112177203	Missense_Mutation	SNP	C	T	T	APC	c.5912C>T	p.S1971F
118J5169M1	1	202710848	202710848	Missense_Mutation	SNP	A	T	T	KDM5B	c.2700T>A	p.D900E
118J5169M1	12	1039246	1039246	Nonsense_Mutation	SNP	C	T	T	RAD52	c.251G>A	p.W84*
118J5169M1	12	4554445	4554445	Missense_Mutation	SNP	G	T	T	FGF6	c.292C>A	p.L98I
118J5169M1		-	-	Frame_Shift_Del	INS	-	-	-	CDKN1B	c.410del	p.P137Rfs*8
118J5169M1	12	25398285	25398285	Missense_Mutation	SNP	C	A	A	KRAS	c.34G>T	p.G12C
118J5169M1		-	-	Splice_Site	SNP	-	-	-	ARID2	c.4774-1del	-
118J5169M1	17	7577570	7577570	Missense_Mutation	SNP	C	T	T	TP53	c.711G>A	p.M237I
118J5169M1		-	-	Frame_Shift_Del	INS	-	-	-	SMAD4	c.814_818del	p.R272Cfs*6
118J5169M1	19	42788891	42788891	Missense_Mutation	SNP	C	T	T	CIC	c.35C>T	p.S12F
118J5169M1	3	10140458	10140458	Missense_Mutation	SNP	G	A	A	FANCD2	c.4240G>A	p.D1414N
118J5169M1		-	-	Frame_Shift_Ins	INS	-	-	-	KMT2C	12476_12477du	p.S4160Afs*5
118J5169M1	9	21994135	21994135	Splice_Site	SNP	T	C	C	CDKN2A	c.193+3A>G	-
118J5169M1	X	47039817	47039817	Splice_Site	SNP	G	A	A	RBM10	c.1161-1G>A	-
118J5206M1	12	25398284	25398284	Missense_Mutation	SNP	C	T	T	KRAS	c.35G>A	p.G12D
118J5206M1	12	46233257	46233257	Missense_Mutation	SNP	G	T	T	ARID2	c.1476G>T	p.Q492H
118J5206M1	17	7578461	7578461	Missense_Mutation	SNP	C	A	A	TP53	c.469G>T	p.V157F
118J5206M1		-	-	Frame_Shift_Del	INS	-	-	-	KDM6A	C.695del	P.L232Cfs*10
118J5249M1	12	46245027	46245027	Nonsense_Mutation	SNP	C	T	T	ARID2	c.3121C>T	p.Q1041*
118J5249M1	14	105237170	105237170	Missense_Mutation	SNP	G	C	C	AKT1	c.1275C>G	p.F425L
118J5249M1	17	7578382	7578382	Nonsense_Mutation	SNP	G	C	C	TP53	c.548C>G	p.S183*
118J5249M1	20	39704825	39704825	Missense_Mutation	SNP	C	G	G	TOP1	c.170C>G	p.S57C
118J5249M1	3	178936094	178936094	Missense_Mutation	SNP	C	G	G	PIK3CA	c.1636C>G	p.Q546E
118J5249M1	6	94120359	94120359	Missense_Mutation	SNP	C	T	T	EPHA7	c.692G>A	p.R231Q
118J5249M1	6	112029201	112029201	Missense_Mutation	SNP	G	C	C	FYN	c.367C>G	p.R123G
118J5249M1		-	-	Frame_Shift_Ins	INS	-	-	-	RBM10	c.819_820insTG	p.Q274Cfs*35
118J5249M1	X	53226131	53226131	Missense_Mutation	SNP	C	G	G	KDM5C	c.2718G>C	p.E906D
118J5276A1		-	-	Frame_Shift_Del	INS	-	-	-	SPEN	c.6948del	p.S2318Lfs*35
118J5276A1	1	16263745	16263745	Missense_Mutation	SNP	T	A	A	SPEN	c.10114T>A	p.S3372T
118J5276A1	1	120057209	120057209	Missense_Mutation	SNP	G	A	A	HSD3B1	c.1063G>A	p.E355K
118J5276A1	1	226252059	226252059	Missense_Mutation	SNP	C	G	G	H3F3A	c.7C>G	p.R3G
118J5276A1	12	427483	427483	Missense_Mutation	SNP	G	T	T	KDM5A	c.2686C>A	p.Q896K
118J5276A1	17	7578290	7578290	Splice_Site	SNP	C	A	A	TP53	c.560-1G>T	-
118J5276A1	19	11169566	11169566	Splice_Site	SNP	G	A	A	SMARCA4	c.4731+1G>A	-
118J5276A1	2	212566814	212566814	Missense_Mutation	SNP	C	T	T	ERBB4	c.1367G>A	p.G456E
118J5276A1	2	212589881	212589881	Missense_Mutation	SNP	A	T	T	ERBB4	c.661T>A	p.C221S
118J5276A1	X	39932902	39932902	Missense_Mutation	SNP	C	T	T	BCOR	c.1697G>A	p.R566H
118J5402A1	1	115256530	115256530	Missense_Mutation	SNP	G	T	T	NRAS	c.181C>A	p.Q61K
118J5402A1	15	88472464	88472464	Missense_Mutation	SNP	G	C	C	NTRK3	c.2091C>G	p.D697E
118J5402A1	17	7577551	7577551	Missense_Mutation	SNP	C	G	G	TP53	c.730G>C	p.G244R
118J5402A1	2	109357112	109357112	Missense_Mutation	SNP	C	T	T	RANBP2	c.950C>T	p.A317V
118J5402A1	2	141526925	141526925	Splice_Site	SNP	G	A	A	LRP1B	c.5627-12C>T	-
118J5402A1	4	143067101	143067101	Missense_Mutation	SNP	T	C	C	INPP4B	c.1612A>G	p.M538V
118J5402A1	4	153332838	153332838	Nonsense_Mutation	SNP	C	A	A	FBXW7	c.118G>T	p.E40*
118J5402A1	8	32505516	32505516	Missense_Mutation	SNP	G	A	A	NRG1	c.280G>A	p.G94S
118J5402A1	X	44733158	44733158	Splice_Site	SNP	C	T	T	KDM6A	c.162-12C>T	-
118J5518M1	3	41266097	41266097	Missense_Mutation	SNP	G	A	A	CTNNB1	c.94G>A	p.D32N
118J5518M1	3	47162341	47162341	Missense_Mutation	SNP	A	C	C	SETD2	c.3785T>G	p.F1262C
118J5583A1		-	-	Frame_Shift_Del	INS	-	-	-	ARID1A	c.5756_5787del	p.S1919Ffs*15
118J5583A1	1	115258747	115258747	Missense_Mutation	SNP	C	A	A	NRAS	c.35G>T	p.G12V
118J5583A1	1	158614191	158614191	Splice_Site	SNP	G	T	T	SPTA1	c.4195-5C>A	-
118J5583A1		-	-	Frame_Shift_Del	INS	-	-	-	TP53	c.109_134del	p.S37Vfs*6
118J5583A1	18	18550317	18550317	Missense_Mutation	SNP	C	T	T	ROCK1	c.2812G>A	p.V938I
118J5583A1	2	61708325	61708325	Missense_Mutation	SNP	T	C	C	XPO1	c.3064A>G	p.I1022V
118J5583A1	3	178951947	178951947	Missense_Mutation	SNP	T	G	G	PIK3CA	c.3002T>G	p.L1001R

118J5583A1	4	110890182	110890182	Missense_Mutation	SNP	C	T	T	EGF	c.1631C>T	p.S544F
118J5583A1	6	157100380	157100380	Missense_Mutation	SNP	G	T	T	ARID1B	c.1317G>T	p.E439D
118J5583A1	6	157517322	157517322	Missense_Mutation	SNP	A	G	G	ARID1B	c.3886A>G	p.M1296V
118J5583A1	X	129148987	129148987	Nonsense_Mutation	SNP	A	T	T	BCORL1	c.2239A>T	p.K747*
118K5630A1	1	120512184	120512184	Missense_Mutation	SNP	C	T	T	NOTCH2	c.1058G>A	p.C353Y
118K5630A1	17	7577538	7577538	Missense_Mutation	SNP	C	T	T	TP53	c.743G>A	p.R248Q
118K5630A1	4	153249385	153249385	Missense_Mutation	SNP	G	A	A	FBXW7	c.1393C>T	p.R465C
118K5630A1	7	140494154	140494154	Missense_Mutation	SNP	G	A	A	BRAF	c.1094C>T	p.S365L
118K5630A1		-	-	Frame_Shift_Del	INS	-	-	-	BCOR	c.3913_3917del	p.E1305Lfs*68
118K5635M1	1	27092809	27092809	Nonsense_Mutation	SNP	C	T	T	ARID1A	c.2830C>T	p.Q944*
118K5635M1	16	10274090	10274090	Missense_Mutation	SNP	G	A	A	GRIN2A	c.179C>T	p.A60V
118K5635M1	16	50811844	50811844	Missense_Mutation	SNP	T	A	A	CYLD	c.1130T>A	p.I377N
118K5635M1	17	7577016	7577016	Splice_Site	SNP	T	C	C	TP53	c.919+3A>G	-
118K5635M1	17	7577117	7577117	Missense_Mutation	SNP	A	T	T	TP53	c.821T>A	p.V274D
118K5635M1	2	141143491	141143491	Missense_Mutation	SNP	C	T	T	LRP1B	c.10502G>A	p.C3501Y
118K5635M1	2	170092375	170092375	Missense_Mutation	SNP	C	T	T	LRP2	c.4895G>A	p.R1632Q
118K5635M1	5	176522396	176522396	Nonsense_Mutation	SNP	C	T	T	FGFR4	c.1585C>T	p.R529*
118K5635M1		-	-	Frame_Shift_Del	INS	-	-	-	DDR1	c.1356_1375del	p.R453Dfs*20
118K5635M1	8	41792377	41792377	Missense_Mutation	SNP	T	C	C	KAT6A	c.3361A>G	p.I1121V
118K5635M1	X	44966786	44966786	Splice_Site	SNP	G	A	A	KDM6A	c.4005+5G>A	-
118K5684M1	12	25398284	25398284	Missense_Mutation	SNP	C	T	T	KRAS	c.35G>A	p.G12D
118K5684M1	5	1280264	1280264	Splice_Site	SNP	C	T	T	TERT	c.1950+9G>A	-
118K5684M1	5	56111590	56111590	Nonsense_Mutation	SNP	A	T	T	MAP3K1	c.190A>T	p.K64*
118K5684M1	6	94066572	94066572	Missense_Mutation	SNP	T	C	C	EPHA7	c.1187A>G	p.E396G
118K5718A1	12	46244022	46244022	Nonsense_Mutation	SNP	A	T	T	ARID2	c.2116A>T	p.K706*
118K5718A1	16	3781441	3781441	Missense_Mutation	SNP	C	A	A	CREBBP	c.4924G>T	p.V1642F
118K5718A1	17	7577534	7577534	Missense_Mutation	SNP	C	A	A	TP53	c.747G>T	p.R249S
118K5718A1	20	57429115	57429115	Missense_Mutation	SNP	C	T	T	GNAS	c.608C>T	p.A203V
118K5718A1	22	30032748	30032748	Nonsense_Mutation	SNP	G	A	A	NF2	c.123G>A	p.W41*
118K5718A1	5	1268630	1268630	Splice_Site	SNP	C	T	T	TERT	c.2582+5G>A	-
118K5718A1		-	-	Splice_Site	SNP	-	-	-	ITK	.515-12_1515-2	-
118K5718A1	7	140453987	140453987	Missense_Mutation	SNP	T	C	C	BRAF	c.1741A>G	p.N581D
118K5909A1	1	243858901	243858901	Missense_Mutation	SNP	G	A	A	AKT3	c.164C>T	p.S55L
118K5909A1	17	7577094	7577094	Missense_Mutation	SNP	G	A	A	TP53	c.844C>T	p.R282W
118K5909A1	17	59926534	59926534	Nonsense_Mutation	SNP	G	A	A	BRIP1	c.463C>T	p.Q155*
118K5909A1		-	-	Frame_Shift_Del	INS	-	-	-	DNMT3A	c.421_433del	p.E141Pfs*17
118K5909A1	2	212812256	212812256	Missense_Mutation	SNP	C	T	T	ERBB4	c.320G>A	p.G107E
118K5909A1		-	-	Frame_Shift_Del	INS	-	-	-	FBXW7	c.870del	p.Y291Mfs*51
118K5909A1	8	68864619	68864619	Splice_Site	SNP	C	T	T	PREX2	c.-11C>T	-
118K5909A1	9	21971186	21971186	Nonsense_Mutation	SNP	G	A	A	CDKN2A	c.172C>T	p.R58*
118K5909A1	X	70612764	70612764	Missense_Mutation	SNP	G	A	A	TAF1	c.3031G>A	p.G1011R
118K5971J1	12	25398284	25398284	Missense_Mutation	SNP	C	T	T	KRAS	c.35G>A	p.G12D
118K5984A1	12	25398284	25398284	Missense_Mutation	SNP	C	T	T	KRAS	c.35G>A	p.G12D
118K5984A1	17	7577105	7577105	Missense_Mutation	SNP	G	A	A	TP53	c.833C>T	p.P278L
118K5984A1	4	126373264	126373264	Missense_Mutation	SNP	G	A	A	FAT4	c.11093G>A	p.R3698Q
118K5984A1		-	-	Splice_Site	SNP	-	-	-	ATRX	c.3737-9del	-
118K5992M1	1	226567661	226567661	Missense_Mutation	SNP	G	C	C	PARP1	c.1505C>G	p.A502G
118K5992M1	10	89711894	89711894	Missense_Mutation	SNP	A	G	G	PTEN	c.512A>G	p.Q171R
118K5992M1	11	108235879	108235879	Missense_Mutation	SNP	C	T	T	ATM	c.8921C>T	p.P2974L
118K5992M1	12	25398284	25398284	Missense_Mutation	SNP	C	T	T	KRAS	c.35G>A	p.G12D
118K5992M1	12	49434990	49434990	Missense_Mutation	SNP	C	T	T	KMT2D	c.6563G>A	p.R2188H
118K5992M1	16	23641346	23641346	Missense_Mutation	SNP	G	A	A	PALB2	c.2129C>T	p.T710M
118K5992M1	17	7578217	7578217	Missense_Mutation	SNP	G	A	A	TP53	c.632C>T	p.T211I
118K5992M1	17	37676315	37676315	Missense_Mutation	SNP	G	A	A	CDK12	c.3070G>A	p.E1024K
118K5992M1	2	29416667	29416667	Missense_Mutation	SNP	T	C	C	ALK	c.4286A>G	p.Q1429R
118K5992M1	2	112751824	112751824	Splice_Site	SNP	C	A	A	MERTK	c.1297-4C>A	-
118K5992M1	2	173429747	173429747	Nonsense_Mutation	SNP	C	T	T	PDK1	c.637C>T	p.R213*
118K5992M1	20	31023931	31023931	Missense_Mutation	SNP	C	A	A	ASXL1	c.3416C>A	p.T1139K
118K5992M1	3	185783608	185783608	Missense_Mutation	SNP	C	T	T	ETV5	c.904G>A	p.D302N
118K5992M1	7	80276070	80276070	Missense_Mutation	SNP	G	T	T	CD36	c.14G>T	p.R5L
118K5992M1	7	127714549	127714549	Splice_Site	SNP	C	T	T	SND1	c.1780-5C>T	-
118K5992M1	8	141756950	141756950	Missense_Mutation	SNP	C	T	T	PTK2	c.1493G>A	p.R498H
118K5992M1	9	5557694	5557694	Missense_Mutation	SNP	C	A	A	PDCD1LG2	c.708C>A	p.F236L
118K5993A1	17	7578257	7578257	Nonsense_Mutation	SNP	C	A	A	TP53	c.592G>T	p.E198*
118K6072M1		-	-	In_Frame_Del	INS	-	-	-	ARID1A	7_4217delinsGT	p.Y1389*
118K6072M1	12	25398285	25398285	Missense_Mutation	SNP	C	G	G	KRAS	c.34G>C	p.G12R
118K6072M1	3	30691910	30691910	Missense_Mutation	SNP	T	G	G	TGFBR2	c.412T>G	p.C138G
118K6072M1	4	185339864	185339864	Splice_Site	SNP	T	G	G	IRF2	c.188-2A>C	-
118K6072M1	9	21971111	21971111	Missense_Mutation	SNP	G	A	A	CDKN2A	c.247C>T	p.H83Y
118K6080A1		-	-	In_Frame_Ins	INS	-	-	-	EPHA2	C.719dup	P.E241*
118K6080A1	17	7577022	7577022	Nonsense_Mutation	SNP	G	A	A	TP53	c.916C>T	p.R306*
118K6130M1		-	-	Frame_Shift_Ins	INS	-	-	-	ARID1A	c.1113dup	p.Q372Afs*28
118K6130M1	10	89653846	89653846	Missense_Mutation	SNP	C	A	A	PTEN	c.144C>A	p.N48K
118K6130M1	19	11143985	11143985	Missense_Mutation	SNP	G	A	A	SMARCA4	c.3566G>A	p.R1189Q
118K6186A1	1	46739143	46739143	Splice_Site	SNP	C	A	A	RAD54L	c.1486+6C>A	-
118K6186A1	1	78430841	78430841	Missense_Mutation	SNP	G	A	A	FUBP1	c.548C>T	p.P183L
118K6186A1	12	25398284	25398284	Missense_Mutation	SNP	C	T	T	KRAS	c.35G>A	p.G12D
118K6186A1	12	57593115	57593115	Missense_Mutation	SNP	C	T	T	LRP1	c.9797C>T	p.T3266M
118K6186A1	13	26978111	26978111	Missense_Mutation	SNP	G	A	A	CDK8	c.1288G>A	p.A430T
118K6186A1	16	67650786	67650786	Splice_Site	SNP	G	A	A	CTCF	c.1086+5G>A	-
118K6186A1	17	7578212	7578212	Nonsense_Mutation	SNP	G	A	A	TP53	c.637C>T	p.R213*
118K6186A1	18	48593443	48593443	Nonsense_Mutation	SNP	G	A	A	SMAD4	c.1194G>A	p.W398*
118K6186A1	19	10464891	10464891	Missense_Mutation	SNP	C	A	A	TYK2	c.2735G>T	p.S912I
118K6186A1	19	41744411	41744411	Missense_Mutation	SNP	G	A	A	AXL	c.1031G>A	p.R344Q
118K6186A1	19	50905864	50905864	Splice_Site	SNP	C	T	T	POLD1	c.841-5C>T	-
118K6186A1	2	141110639	141110639	Missense_Mutation	SNP	G	A	A	LRP1B	c.11533C>T	p.L3845F
118K6186A1		-	-	In_Frame_Del	INS	-	-	-	SRMS	c.1464_1465del	p.*489Sext*59
118K6186A1	5	131973900	131973900	Missense_Mutation	SNP	C	G	G	RAD50	c.3603C>G	p.C1201W
118K6186A1	5	161116252	161116252	Splice_Site	SNP	G	A	A	GABRA6	c.447-8G>A	-
118K6186A1	7	41730102	41730102	Missense_Mutation	SNP	C	T	T	INHBA	c.427G>A	p.E143K
118K6186A1	8	41791906	41791906	Missense_Mutation	SNP	G	A	A	KAT6A	c.3832C>T	p.R1278C
118K6186A1	9	21994140	21994140	Missense_Mutation	SNP	G	A	A	CDKN2A	c.191C>T	p.P64L
118K6186A1	9	35078227	35078227	Missense_Mutation	SNP	G	A	A	FANCG	c.421C>T	p.R141C
118K6209A1		-	-	Frame_Shift_Ins	INS	-	-	-	ARID1A	C.2172_2175du	P.P726Afs*92
118K6209A1	19	18273784	18273784	Missense_Mutation	SNP	G	A	A	PIK3R2	c.1117G>A	p.G373R
118K6209A1	2	209113113	209113113	Missense_Mutation	SNP	G	A	A	IDH1	c.394C>T	p.R132C
118K6209A1	3	52651426	52651426	Missense_Mutation	SNP	T	A	A	PBRM1	c.1670A>T	p.D557V
118K6272A1	1	11204742	11204742	Missense_Mutation	SNP	C	T	T	MTOR	c.4835G>A	p.R1612Q

118K6272A1	11	92532669	92532669	Missense_Mutation	SNP	G	A	A	FAT3	c.6490G>A	p.G2164R
118K6272A1	11	92533086	92533086	Missense_Mutation	SNP	G	T	T	FAT3	c.6907G>T	p.G2303W
118K6272A1	11	92616250	92616250	Missense_Mutation	SNP	C	T	T	FAT3	c.12628C>T	p.R4210W
118K6272A1	11	108138018	108138018	Missense_Mutation	SNP	G	C	C	ATM	c.2587G>C	p.D863H
118K6272A1		-	-	Frame_Shift_Del	INS	-	-	-	TP53	c.561_564del	p.L188Pfs*58
118K6272A1	17	48265353	48265353	Splice_Site	SNP	G	C	C	COL1A1	c.3262-9C>G	-
118K6272A1	19	10610205	10610205	Missense_Mutation	SNP	G	A	A	KEAP1	c.505C>T	p.R169C
118K6272A1	2	215645433	215645433	Missense_Mutation	SNP	T	A	A	BARD1	c.1165A>T	p.S389C
118K6272A1	22	21304119	21304119	Missense_Mutation	SNP	G	C	C	CRKL	c.898G>C	p.D300H
118K6272A1	3	156421343	156421343	Nonsense_Mutation	SNP	C	T	T	TIPARP	c.1378C>T	p.Q460*
118K6272A1	3	178922301	178922301	Missense_Mutation	SNP	G	A	A	PIK3CA	c.1070G>A	p.R357Q
118K6272A1	7	151845790	151845790	Missense_Mutation	SNP	C	G	G	KMT2C	c.13222G>C	p.E4408Q
118K6272A1	8	31497666	31497666	Missense_Mutation	SNP	C	G	G	NRG1	c.166C>G	p.P56A
118K6285M1	1	43817931	43817931	Missense_Mutation	SNP	G	T	T	MPL	c.1610G>T	p.R537L
118K6285M1		-	-	Frame_Shift_Del	INS	-	-	-	KMT2D	c.14312del	p.V4771Afs*26
118K6285M1	12	49434495	49434495	Missense_Mutation	SNP	G	A	A	KMT2D	c.7058C>T	p.P2353L
118K6285M1	16	56782164	56782164	Missense_Mutation	SNP	A	G	G	NUP93	c.5A>G	p.D2G
118K6285M1	16	56865836	56865836	Missense_Mutation	SNP	G	A	A	NUP93	c.1168G>A	p.V390M
118K6285M1	16	68842741	68842741	Missense_Mutation	SNP	C	A	A	CDH1	c.677C>A	p.A226D
118K6285M1	17	7578253	7578253	Missense_Mutation	SNP	C	A	A	TP53	c.596G>T	p.G199V
118K6285M1	17	59870987	59870987	Missense_Mutation	SNP	T	C	C	BRIP1	c.1444A>G	p.I482V
118K6285M1	18	19751656	19751656	Missense_Mutation	SNP	G	A	A	GATA6	c.551G>A	p.S184N
118K6285M1	19	10472988	10472988	Missense_Mutation	SNP	C	T	T	TYK2	c.1621G>A	p.G541R
118K6285M1		-	-	Splice_Site	SNP	-	-	-	EP300	c.1623-5dup	-
118K6285M1	3	119720881	119720881	Splice_Site	SNP	T	A	A	GSK3B	c.282+12A>T	-
118K6285M1	4	187532570	187532570	Missense_Mutation	SNP	G	T	T	FAT1	c.9823C>A	p.H3275N
118K6285M1	4	187541786	187541786	Missense_Mutation	SNP	G	T	T	FAT1	c.5954C>A	p.S1985Y
118K6285M1	5	251216	251216	Missense_Mutation	SNP	G	A	A	SDHA	c.1661G>A	p.R554Q
118K6285M1	5	156671429	156671429	Missense_Mutation	SNP	C	A	A	ITK	c.1390C>A	p.L464M
118K6285M1	6	33288998	33288998	Missense_Mutation	SNP	C	T	T	DAXX	c.554G>A	p.R185Q
118K6285M1	9	102590768	102590768	Missense_Mutation	SNP	C	A	A	NR4A3	c.444C>A	p.F148L
118K6312M1	17	7578503	7578503	Missense_Mutation	SNP	C	T	T	TP53	c.427G>A	p.V143M
118K6312M1		-	-	In_Frame_Del	INS	-	-	-	CDKN2A	c.83_100del	p.V28_E33del
118K6312M1		-	-	Frame_Shift_Del	INS	-	-	-	KDM6A	c.3388_3395del	p.N1130Sfs*18
118K6336A1	11	125499349	125499349	Missense_Mutation	SNP	G	A	A	CHEK1	c.418G>A	p.E140K
118K6336A1	16	2130267	2130267	Missense_Mutation	SNP	G	C	C	TSC2	c.3499G>C	p.E1167Q
118K6336A1		-	-	Frame_Shift_Del	INS	-	-	-	TP53	c.626_627del	p.R209Kfs*6
118K6336A1	18	42532639	42532639	Nonsense_Mutation	SNP	A	T	T	SETBP1	c.3334A>T	p.K1112*
118K6336A1	2	141283547	141283547	Missense_Mutation	SNP	G	C	C	LRP1B	c.7892C>G	p.T2631R
118K6336A1	2	212543774	212543774	Splice_Site	SNP	T	A	A	ERBB4	c.1622+3A>T	-
118K6336A1	3	41266101	41266101	Missense_Mutation	SNP	C	T	T	CTNNB1	c.98C>T	p.S33F
118K6336A1	4	153249478	153249478	Missense_Mutation	SNP	T	A	A	FBXW7	c.1300A>T	p.I434F
118K6336A1	7	20198075	20198075	Missense_Mutation	SNP	C	T	T	MACC1	c.1909G>A	p.E637K
118K6336A1	7	151921590	151921590	Missense_Mutation	SNP	T	C	C	KMT2C	c.3088A>G	p.I1030V
118K6349A1	11	92616149	92616149	Missense_Mutation	SNP	G	A	A	FAT3	c.12527G>A	p.R4176H
118K6349A1	17	37868208	37868208	Missense_Mutation	SNP	C	T	T	ERBB2	c.929C>T	p.S310F
118K6349A1	17	80789199	80789199	Missense_Mutation	SNP	C	T	T	ZNF750	c.1132G>A	p.E378K
118K6349A1	18	48575671	48575671	Nonsense_Mutation	SNP	C	G	G	SMAD4	c.431C>G	p.S144*
118K6349A1	3	189526226	189526226	Missense_Mutation	SNP	G	A	A	TP63	c.490G>A	p.A164T
118K6349A1	7	41729593	41729593	Missense_Mutation	SNP	C	G	G	INHBA	c.936G>C	p.L312F
118K6372A3		-	-	Frame_Shift_Del	INS	-	-	-	ARID1A	c.817del	p.A273Pfs*90
118K6372A3		-	-	Frame_Shift_Ins	INS	-	-	-	ARID1A	c.3216dup	p.W1073Mfs*32
118K6372A3		-	-	Splice_Site	SNP	-	-	-	FUBP1	c.1781-6dup	-
118K6372A3	1	158623086	158623086	Missense_Mutation	SNP	G	A	A	SPTA1	c.3166C>T	p.R1056C
118K6372A3	1	162729663	162729663	Missense_Mutation	SNP	T	C	C	DDR2	c.749T>C	p.V250A
118K6372A3	11	22646455	22646455	Missense_Mutation	SNP	T	C	C	FANCF	c.902A>G	p.D301G
118K6372A3	11	118376407	118376407	Missense_Mutation	SNP	A	C	C	KMT2A	c.9800A>C	p.N3267T
118K6372A3		-	-	Missense_Mutation	SNP	-	-	-	CHD4	c.4586_4588del	p.K1529del
118K6372A3	12	21628745	21628745	Missense_Mutation	SNP	A	C	C	RECQL	c.963T>G	p.C321W
118K6372A3		-	-	Splice_Site	SNP	-	-	-	KMT2D	c.13840-10del	-
118K6372A3	12	49445244	49445244	Missense_Mutation	SNP	G	A	A	KMT2D	c.2222C>T	p.P741L
118K6372A3	12	52385715	52385715	Nonsense_Mutation	SNP	C	T	T	ACVR1B	c.1453C>T	p.R485*
118K6372A3	12	56478854	56478854	Missense_Mutation	SNP	G	T	T	ERBB3	c.310G>T	p.V104L
118K6372A3		-	-	Frame_Shift_Ins	INS	-	-	-	APEX1	c.146dup	p.D50Rfs*28
118K6372A3		-	-	Frame_Shift_Ins	INS	-	-	-	FANCM	c.2604dup	p.E869Rfs*3
118K6372A3	16	67662352	67662352	Missense_Mutation	SNP	G	A	A	CTCF	c.1598G>A	p.R533H
118K6372A3		-	-	Frame_Shift_Ins	INS	-	-	-	CDH1	c.377dup	p.P127Afs*41
118K6372A3		-	-	Frame_Shift_Del	INS	-	-	-	USP6	c.3752del	p.G1251Afs*14
118K6372A3		-	-	Frame_Shift_Del	INS	-	-	-	TP53	c.532del	p.H178Tfs*69
118K6372A3	17	29541518	29541518	Missense_Mutation	SNP	C	A	A	NF1	c.1442C>A	p.P481H
118K6372A3	17	29661899	29661899	Nonsense_Mutation	SNP	G	A	A	NF1	c.5856G>A	p.W1952*
118K6372A3		-	-	Splice_Site	SNP	-	-	-	SPOP	c.353-12del	-
118K6372A3		-	-	Frame_Shift_Del	INS	-	-	-	RNF43	C.1976del	P.G659Vfs*41
118K6372A3	17	78897476	78897476	Splice_Site	SNP	A	G	G	RPTOR	c.2808+3A>G	-
118K6372A3	18	23632782	23632782	Missense_Mutation	SNP	G	T	T	SS18	c.413C>A	p.P138H
118K6372A3		-	-	Frame_Shift_Del	INS	-	-	-	ROCK2	c.412del	p.W138Gfs*31
118K6372A3		-	-	Missense_Mutation	SNP	-	-	-	XPO1	c.116_118del	p.E39del
118K6372A3	2	121746006	121746006	Missense_Mutation	SNP	C	T	T	GLI2	c.2516C>T	p.S839L
118K6372A3	2	170042491	170042491	Missense_Mutation	SNP	C	T	T	LRP2	c.9367G>A	p.D3123N
118K6372A3		-	-	Splice_Site	SNP	-	-	-	LRP2	c.770-5del	-
118K6372A3	22	21344765	21344765	Missense_Mutation	SNP	G	A	A	LZTR1	c.742G>A	p.G248R
118K6372A3		-	-	Missense_Mutation	SNP	-	-	-	SMARCB1	c.1091_1093del	p.K364del
118K6372A3	22	30057302	30057302	Nonsense_Mutation	SNP	C	T	T	NF2	c.784C>T	p.R262*
118K6372A3	22	41556717	41556717	Missense_Mutation	SNP	A	C	C	EP300	c.3662A>C	p.Q1221P
118K6372A3	3	49927438	49927438	Missense_Mutation	SNP	C	T	T	MST1R	c.3866G>A	p.R1289H
118K6372A3		-	-	Frame_Shift_Del	INS	-	-	-	PBRM1	c.773del	p.N258Mfs*25
118K6372A3	3	142268966	142268966	Splice_Site	SNP	C	T	T	ATR	c.2976+8G>A	-
118K6372A3	3	185191097	185191097	Missense_Mutation	SNP	G	A	A	MAP3K13	c.1978G>A	p.G660R
118K6372A3	4	126369973	126369973	Missense_Mutation	SNP	A	G	G	FAT4	c.7802A>G	p.Y2601C
118K6372A3	4	187539894	187539894	Missense_Mutation	SNP	C	T	T	FAT1	c.7846G>A	p.V2616I
118K6372A3		-	-	Splice_Site	SNP	-	-	-	ITK	1061-7_1061-6d	-
118K6372A3		-	-	Splice_Site	SNP	-	-	-	ARID1B	c.1927-3del	-
118K6372A3	6	157488293	157488293	Missense_Mutation	SNP	C	T	T	ARID1B	c.2999C>T	p.A1000V
118K6372A3	6	157522130	157522130	Missense_Mutation	SNP	C	T	T	ARID1B	c.4402C>T	p.R1468C
118K6372A3	6	157528657	157528657	Nonsense_Mutation	SNP	C	T	T	ARID1B	c.6382C>T	p.R2128*
118K6372A3	7	55221830	55221830	Missense_Mutation	SNP	G	A	A	EGFR	c.874G>A	p.V292M
118K6372A3	7	140481411	140481411	Missense_Mutation	SNP	C	A	A	BRAF	c.1397G>T	p.G466V
118K6372A3	7	140507836	140507836	Missense_Mutation	SNP	G	A	A	BRAF	c.635C>T	p.T212I

118K6372A3	9	102609909	102609909	Splice_Site	SNP	C	A	A	NR4A3	c.1633+12C>A	-
118K6377A1	1	27106308	27106308	Nonsense_Mutation	SNP	G	A	A	ARID1A	c.5919G>A	p.W1973*
118K6377A1		-	-	Frame_Shift_Del	INS	-	-	-	ARID2	c.1010_1020del	p.N337Rfs*8
118K6377A1	17	7577547	7577547	Missense_Mutation	SNP	C	T	T	TP53	c.734G>A	p.G245D
118K6377A1	18	48591891	48591891	Missense_Mutation	SNP	G	A	A	SMAD4	c.1054G>A	p.G352R
118K6377A1	6	163876338	163876338	Missense_Mutation	SNP	A	G	G	QKI	c.170A>G	p.Y57C
118K6454A1		-	-	Frame_Shift_Del	INS	-	-	-	EPHA2	c.1244delinsCT	p.R415Pfs*39
118K6454A1	1	115256529	115256529	Missense_Mutation	SNP	T	C	C	NRAS	c.182A>G	p.Q61R
118K6454A1	3	52439883	52439883	Nonsense_Mutation	SNP	G	A	A	BAP1	c.829C>T	p.Q277*
118K6479M1	11	60231786	60231786	Missense_Mutation	SNP	C	A	A	MS4A1	c.305C>A	p.A102E
118K6479M1	17	7578406	7578406	Missense_Mutation	SNP	C	T	T	TP53	c.524G>A	p.R175H
118K6479M1	3	41267364	41267364	Splice_Site	SNP	A	C	C	CTNNB1	c.936+12A>C	-
118K6479M1	4	88967920	88967920	Missense_Mutation	SNP	C	G	G	PKD2	c.1446C>G	p.F482L
118K6509A1	12	46230571	46230571	Nonsense_Mutation	SNP	C	T	T	ARID2	c.820C>T	p.R274*
118K6509A1	12	49426907	49426907	Nonsense_Mutation	SNP	G	A	A	KMT2D	c.11581C>T	p.Q3861*
118K6509A1	15	66729154	66729154	Missense_Mutation	SNP	G	C	C	MAP2K1	c.362G>C	p.C121S
118K6509A1	16	2103385	2103385	Nonsense_Mutation	SNP	C	T	T	TSC2	c.268C>T	p.Q90*
118K6509A1	16	2130180	2130180	Nonsense_Mutation	SNP	C	T	T	TSC2	c.3412C>T	p.R1138*
118K6509A1	17	7577511	7577511	Missense_Mutation	SNP	A	G	G	TP53	c.770T>C	p.L257P
118K6509A1	18	48575209	48575209	Nonsense_Mutation	SNP	C	T	T	SMAD4	c.403C>T	p.R135*
118K6509A1	18	48591918	48591918	Missense_Mutation	SNP	C	T	T	SMAD4	c.1081C>T	p.R361C
118K6509A1	2	148677797	148677797	Splice_Site	SNP	A	C	C	ACVR2A	c.963-2A>C	-
118K6509A1	5	231091	231091	Missense_Mutation	SNP	G	C	C	SDHA	c.871G>C	p.E291Q
118K6509A1	5	149502735	149502735	Missense_Mutation	SNP	G	A	A	PDGFRB	c.2053C>T	p.R685C
118K6509A1	X	129190052	129190052	Missense_Mutation	SNP	G	A	A	BCORL1	c.5077G>A	p.E1693K
118K6518A1	12	25398284	25398284	Missense_Mutation	SNP	C	A	A	KRAS	c.35G>T	p.G12V
118K6518A1	17	7577526	7577526	Missense_Mutation	SNP	A	G	G	TP53	c.755T>C	p.L252P
118K6518A1	20	31022853	31022853	Nonsense_Mutation	SNP	C	T	T	ASXL1	c.2338C>T	p.Q780*
118K6518A1		-	-	Frame_Shift_Del	INS	-	-	-	PBRM1	C.559del	P.E187Sfs*7
118K6518A1	5	112102032	112102032	Nonsense_Mutation	SNP	A	T	T	APC	c.145A>T	p.K49*
118K6557A1	1	43775149	43775149	Missense_Mutation	SNP	C	T	T	TIE1	c.1279C>T	p.R427C
118K6557A1	10	8115751	8115751	Missense_Mutation	SNP	G	A	A	GATA3	c.1100G>A	p.R367Q
118K6557A1	12	25398282	25398282	Missense_Mutation	SNP	C	A	A	KRAS	c.37G>T	p.G13C
118K6557A1	12	57859588	57859588	Missense_Mutation	SNP	G	A	A	GLI1	c.519G>A	p.M173I
118K6557A1		-	-	Missense_Mutation	SNP	-	-	-	TP53	c.764_766del	p.I255del
118K6557A1	17	29679367	29679367	Missense_Mutation	SNP	G	A	A	NF1	c.7550G>A	p.R2517Q
118K6557A1		-	-	Frame_Shift_Del	INS	-	-	-	IRF2	c.642del	p.S215Afs*2
118K6557A1	9	21971209	21971209	Splice_Site	SNP	T	A	A	CDKN2A	c.151-2A>T	-
118K6580A1	16	2124337	2124337	Missense_Mutation	SNP	C	T	T	TSC2	c.2492C>T	p.T831M
118K6580A1	17	7577509	7577509	Nonsense_Mutation	SNP	C	A	A	TP53	c.772G>T	p.E258*
118K6580A1	2	141665451	141665451	Missense_Mutation	SNP	A	G	G	LRP1B	c.3515T>C	p.L1172P
118K6580A1	7	20199837	20199837	Nonsense_Mutation	SNP	C	T	T	MACC1	c.147G>A	p.W49*
118K6595M2		-	-	Frame_Shift_Del	INS	-	-	-	MTOR	c.5274_5290del	p.Q1758Hfs*3
118K6595M2	12	57597207	57597207	Missense_Mutation	SNP	C	A	A	LRP1	c.10854C>A	p.F3618L
118K6595M2	17	7578406	7578406	Missense_Mutation	SNP	C	T	T	TP53	c.524G>A	p.R175H
118K6595M2	17	29509525	29509525	Splice_Site	SNP	G	C	C	NF1	c.731-1G>C	-
118K6595M2	17	48263830	48263830	Missense_Mutation	SNP	C	T	T	COL1A1	c.3853G>A	p.D1285N
118K6595M2	7	92462405	92462405	Missense_Mutation	SNP	C	T	T	CDK6	c.233G>A	p.R78K
118K6595M2	7	127724808	127724808	Missense_Mutation	SNP	C	G	G	SND1	c.2143C>G	p.R715G
118K6595M2	9	87635254	87635254	Missense_Mutation	SNP	C	G	G	NTRK2	c.2306C>G	p.P769R
118K6631A1	12	25398284	25398284	Missense_Mutation	SNP	C	A	A	KRAS	c.35G>T	p.G12V
118K6631A1	4	20620399	20620399	Missense_Mutation	SNP	T	C	C	SLIT2	c.4357T>C	p.C1453R
118K6637M1	12	25398285	25398285	Missense_Mutation	SNP	C	A	A	KRAS	c.34G>T	p.G12C
118K6637M1		-	-	Frame_Shift_Del	INS	-	-	-	TP53	c.626_627del	p.R209Kfs*6
118K6637M1	17	12013738	12013738	Missense_Mutation	SNP	A	T	T	MAP2K4	c.680A>T	p.H227L
118K6637M1	18	48575671	48575671	Nonsense_Mutation	SNP	C	G	G	SMAD4	c.431C>G	p.S144*
118K6637M1		-	-	Frame_Shift_Del	INS	-	-	-	AMER1	84_988delinsG[illegible]	p.C328Wfs*4
118K6653A1	17	7577114	7577114	Missense_Mutation	SNP	C	T	T	TP53	c.824G>A	p.C275Y
118K6653A1	22	21341825	21341825	Missense_Mutation	SNP	G	A	A	LZTR1	c.353G>A	p.R118H
118K6653A1	4	110882136	110882136	Nonsense_Mutation	SNP	C	T	T	EGF	c.1180C>T	p.R394*
118K6674A1	12	25380275	25380275	Missense_Mutation	SNP	T	G	G	KRAS	c.183A>C	p.Q61H
118K6674A1	17	7578534	7578534	Missense_Mutation	SNP	C	G	G	TP53	c.396G>C	p.K132N
118K6674A1	2	112765986	112765986	Missense_Mutation	SNP	G	A	A	MERTK	c.1894G>A	p.E632K
118K6674A1	2	212426807	212426807	Missense_Mutation	SNP	G	T	T	ERBB4	c.2308C>A	p.L770M
118K6674A1	3	52441219	52441219	Missense_Mutation	SNP	T	G	G	BAP1	c.551A>C	p.D184A
118K6674A1	7	80299287	80299287	Missense_Mutation	SNP	C	A	A	CD36	c.767C>A	p.P256H
118K6703A1		-	-	In_Frame_Del	INS	-	-	-	LZTR1	c.2248_2253del	p.G750_F751del
118K6703A1		-	-	In_Frame_Del	INS	-	-	-	BAP1	c.1063_1081del	p.Q355*
118K6703A1	3	183212058	183212058	Missense_Mutation	SNP	T	A	A	KLHL6	c.1159A>T	p.T387S
118K6703A1	7	140453193	140453193	Missense_Mutation	SNP	T	C	C	BRAF	c.1742A>G	p.N581S
118K6789A1	2	209113113	209113113	Missense_Mutation	SNP	G	A	A	IDH1	c.394C>T	p.R132C
118K6789A1	20	57428356	57428356	Missense_Mutation	SNP	G	T	T	GNAS	c.36G>T	p.M12I
118K6789A1	3	185146700	185146700	Missense_Mutation	SNP	A	G	G	MAP3K13	c.331A>G	p.S111G
118K6855M1	10	84738731	84738731	Missense_Mutation	SNP	G	T	T	NRG3	c.1438G>T	p.G480W
118K6855M1	11	92086957	92086957	Missense_Mutation	SNP	G	T	T	FAT3	c.1679G>T	p.S560I
118K6855M1	17	7578463	7578463	Missense_Mutation	SNP	C	G	G	TP53	c.467G>C	p.R156P
118K6855M1	18	48604790	48604790	Nonsense_Mutation	SNP	G	T	T	SMAD4	c.1612G>T	p.E538*
118K6924A1	12	25398284	25398284	Missense_Mutation	SNP	C	T	T	KRAS	c.35G>A	p.G12D
118K6924A1	3	30732996	30732996	Missense_Mutation	SNP	C	T	T	TGFBR2	c.1609C>T	p.R537C
118K6924A1		-	-	Frame_Shift_Del	INS	-	-	-	RBM10	c.1630del	p.H544Ifs*160
118K6958A1	1	27105586	27105586	Nonsense_Mutation	SNP	G	T	T	ARID1A	c.5197G>T	p.E1733*
118K6958A1	1	158626386	158626386	Missense_Mutation	SNP	T	C	C	SPTA1	c.2866A>G	p.K956E
118K6958A1	12	25398284	25398284	Missense_Mutation	SNP	C	T	T	KRAS	c.35G>A	p.G12D
118K6958A1	17	7577094	7577094	Missense_Mutation	SNP	G	A	A	TP53	c.844C>T	p.R282W
118K6958A1		-	-	Frame_Shift_Del	INS	-	-	-	SMAD4	C.563_564del	P.N188Tfs*17
118K6958A1	3	142241669	142241669	Missense_Mutation	SNP	A	C	C	ATR	c.4167T>G	p.D1389E
118K6958A1	8	11412317	11412317	Missense_Mutation	SNP	C	T	T	BLK	c.538C>T	p.R180C
118K6975A1	13	32931985	32931985	Missense_Mutation	SNP	C	A	A	BRCA2	c.7724C>A	p.T2575N
118K6975A1	17	7577121	7577121	Missense_Mutation	SNP	G	A	A	TP53	c.817C>T	p.R273C
118K6975A1	17	37868208	37868208	Missense_Mutation	SNP	C	T	T	ERBB2	c.929C>T	p.S310F
118K6975A1	2	170028581	170028581	Missense_Mutation	SNP	C	T	T	LRP2	c.11207G>A	p.R3736H
118K6975A1	7	140481402	140481402	Missense_Mutation	SNP	C	A	A	BRAF	c.1406G>T	p.G469V
118K6975A1	8	48710791	48710791	Splice_Site	SNP				PRKDC	c.10455+9C>T	-
118K6975A1	9	21971186	21971186	Nonsense_Mutation	SNP	G	A	A	CDKN2A	c.172C>T	p.R58*
118K6975A1	X	15540551	15540551	Nonsense_Mutation	SNP	C	G	G	BMX	c.593C>G	p.S198*
118K6977A1	1	43787162	43787162	Missense_Mutation	SNP	G	T	T	TIE1	c.3244G>T	p.V1082L
118K6977A1	11	65425793	65425793	Missense_Mutation	SNP	C	T	T	RELA	c.842G>A	p.S281N
118K6977A1	11	92616427	92616427	Missense_Mutation	SNP	G	C	C	FAT3	c.12805G>C	p.E4269Q

118K6977A1		-	-	Frame_Shift_Del	INS	-	-	-	TP53	C.254del	P.P85Lfs*38
118K6977A1	19	4090615	4090615	Missense_Mutation	SNP	G	T	T	MAP2K2	c.1184C>A	p.P395H
118K6977A1	3	30729864	30729864	Splice_Site	SNP	C	G	G	TGFBR2	c.1397-12C>G	-
118K6977A1	4	1808030	1808030	Missense_Mutation	SNP	G	C	C	FGFR3	c.2006G>C	p.R669P
118K6977A1	4	55968099	55968099	Missense_Mutation	SNP	C	G	G	KDR	c.2231G>C	p.G744A
118K6977A1		-	-	Frame_Shift_Del	INS	-	-	-	CSNK1A1	C.626_635del	P.Y209Cfs*13
118K6986A1	12	50480122	50480122	Missense_Mutation	SNP	G	A	A	SMARCD1	c.356G>A	p.R119Q
118K6986A1	16	50783661	50783661	Missense_Mutation	SNP	G	A	A	CYLD	c.52G>A	p.E18K
118K6986A1		-	-	Frame_Shift_Del	INS	-	-	-	TP53	c.923del	p.L308Rfs*37
118K6986A1	17	78854209	78854209	Splice_Site	SNP	G	C	C	RPTOR	c.1510-6G>C	-
118K6986A1	18	23632790	23632790	Missense_Mutation	SNP	C	A	A	SS18	c.405G>T	p.M135I
118K6986A1	2	25497848	25497848	Missense_Mutation	SNP	G	A	A	DNMT3A	c.601C>T	p.R201C
118K6986A1	2	29917793	29917793	Missense_Mutation	SNP	C	T	T	ALK	c.875G>A	p.R292H
118K6986A1	2	109383574	109383574	Missense_Mutation	SNP	G	T	T	RANBP2	c.6579G>T	p.E2193D
118K6986A1	22	21344757	21344757	Missense_Mutation	SNP	G	A	A	LZTR1	c.734G>A	p.G245E
118K6986A1	6	30858775	30858775	Missense_Mutation	SNP	A	C	C	DDR1	c.443A>C	p.E148A
118K6986A1	7	6017398	6017398	Splice_Site	SNP	T	A	A	PMS2	c.2276-10A>T	-
118K6986A1	7	42088124	42088124	Missense_Mutation	SNP	C	A	A	GLI3	c.645G>T	p.M215I
118K6986A1	7	80302163	80302163	Splice_Site	SNP	A	T	T	CD36	c.1199+4A>T	-
118K6986A1	X	53223818	53223818	Missense_Mutation	SNP	C	T	T	KDM5C	c.3541G>A	p.A1181T
118K7052M1	1	115256529	115256529	Missense_Mutation	SNP	T	C	C	NRAS	c.182A>G	p.Q61R
118K7052M1	13	103053877	103053877	Missense_Mutation	SNP	G	A	A	FGF14	c.152C>T	p.T51M
118K7052M1		-	-	Frame_Shift_Ins	INS	-	-	-	BAP1	c.476_510dup	p.V171Lfs*28
118K7052M1		-	-	Frame_Shift_Ins	INS	-	-	-	PBRM1	c.1248dup	p.Y417Ifs*3
118K7052M1	4	66231757	66231757	Missense_Mutation	SNP	C	A	A	EPHA5	c.1943G>T	p.R648I
118K7052M1	6	117674224	117674224	Missense_Mutation	SNP	A	G	G	ROS1	c.4250T>C	p.I1417T
118K7082A1	12	25398284	25398284	Missense_Mutation	SNP	C	T	T	KRAS	c.35G>A	p.G12D
118K7082A1	15	93522483	93522483	Missense_Mutation	SNP	C	G	G	CHD2	c.2846C>G	p.T949R
118K7082A1	17	7578271	7578271	Missense_Mutation	SNP	T	A	A	TP53	c.578A>T	p.H193L
118K7082A1	18	48581281	48581281	Nonsense_Mutation	SNP	C	G	G	SMAD4	c.585C>G	p.Y195*
118K7082A1		-	-	Frame_Shift_Del	INS	-	-	-	SMARCA4	426_432delinsT	p.Q144Cfs*158
118K7082A1	2	121708868	121708868	Missense_Mutation	SNP	C	T	T	GLI2	c.304C>T	p.R102W
118K7082A1	20	57484421	57484421	Missense_Mutation	SNP	G	A	A	GNAS	c.602G>A	p.R201H
118K7082A1		-	-	Frame_Shift_Del	INS	-	-	-	KMT2C	c.11228del	p.K3743Rfs*2
118K7082A1	8	41834792	41834792	Missense_Mutation	SNP	C	T	T	KAT6A	c.1097G>A	p.R366Q
118K7083M1	1	16459975	16459975	Splice_Site	SNP	C	T	T	EPHA2	c.1864+1G>A	-
118K7083M1		-	-	Frame_Shift_Ins	INS	-	-	-	TP53	C.93dup	P.L32Sfs*11
118K7083M1		-	-	Frame_Shift_Del	INS	-	-	-	NF1	C.1584del	P.L529Sfs*27
118K7083M1	3	178936082	178936082	Missense_Mutation	SNP	G	A	A	PIK3CA	c.1624G>A	p.E542K
118L7106A1		-	-	Frame_Shift_Del	INS	-	-	-	TP53	c.1002del	p.R335Vfs*10
118L7106A1		-	-	Frame_Shift_Del	INS	-	-	-	MSH2	c.1171_1202del	p.A391Tfs*15
118L7106A1	20	57485733	57485733	Splice_Site	SNP	C	T	T	GNAS	c.1039-5C>T	-
118L7106A1		-	-	Frame_Shift_Del	INS	-	-	-	MST1R	c.722_770del	p.H241Pfs*6
118L7106A1	4	1920319	1920319	Missense_Mutation	SNP	T	C	C	NSD2	c.1379T>C	p.F460S
118L7131A1		-	-	In_Frame_Del	INS	-	-	-	ARID2	c.2495_2503del	p.S832*
118L7131A1	17	5036249	5036249	Missense_Mutation	SNP	G	T	T	USP6	c.240G>T	p.M80I
118L7131A1	17	7578493	7578493	Nonsense_Mutation	SNP	C	T	T	TP53	c.437G>A	p.W146*
118L7131A1	4	153258986	153258986	Nonsense_Mutation	SNP	G	A	A	FBXW7	c.829C>T	p.Q277*
118L7133A1	17	7579414	7579414	Nonsense_Mutation	SNP	C	T	T	TP53	c.273G>A	p.W91*
118L7161A1	15	67457641	67457641	Missense_Mutation	SNP	C	A	A	SMAD3	c.451C>A	p.P151T
118L7161A1	17	7577108	7577108	Missense_Mutation	SNP	C	A	A	TP53	c.830G>T	p.C277F
118L7161A1		-	-	Frame_Shift_Del	INS	-	-	-	PIK3R1	C.98del	P.N33Ifs*5
118L7161A1		-	-	Frame_Shift_Ins	INS	-	-	-	APC	C.4666dup	P.T1556Nfs*3
118L7198A1	1	16475389	16475389	Missense_Mutation	SNP	G	A	A	EPHA2	c.307C>T	p.R103C
118L7198A1	10	123239508	123239508	Nonsense_Mutation	SNP	C	A	A	FGFR2	c.2329G>T	p.E777*
118L7198A1	17	7578475	7578475	Missense_Mutation	SNP	G	A	A	TP53	c.455C>T	p.P152L
118L7198A1	17	37868208	37868208	Missense_Mutation	SNP	C	T	T	ERBB2	c.929C>T	p.S310F
118L7198A1	3	52443729	52443729	Splice_Site	SNP	C	T	T	BAP1	c.67+1G>A	-
118L7198A1	7	128852243	128852243	Missense_Mutation	SNP	G	A	A	SMO	c.2315G>A	p.R772H
118L7198A1	7	140453193	140453193	Missense_Mutation	SNP	T	A	A	BRAF	c.1742A>T	p.N581I
118L7202A1	1	158624504	158624504	Missense_Mutation	SNP	C	A	A	SPTA1	c.2933G>T	p.G978V
118L7202A1	12	46287201	46287201	Splice_Site	SNP	A	G	G	ARID2	c.5148-2A>G	-
118L7202A1		-	-	Frame_Shift_Del	INS	-	-	-	CTCF	C.1331_1332de	P.T444Sfs*7
118L7202A1	17	7577570	7577570	Missense_Mutation	SNP	C	A	A	TP53	c.711G>T	p.M237I
118L7202A1	X	110463640	110463640	Missense_Mutation	SNP	G	A	A	PAK3	c.1600G>A	p.A534T
118L7212A1	1	51439730	51439730	Nonsense_Mutation	SNP	G	T	T	CDKN2C	c.295G>T	p.E99*
118L7212A1	11	118375313	118375313	Missense_Mutation	SNP	G	T	T	KMT2A	c.8706G>T	p.Q2902H
118L7212A1	12	46240644	46240644	Nonsense_Mutation	SNP	A	T	T	ARID2	c.1504A>T	p.R502*
118L7212A1		-	-	Frame_Shift_Del	INS	-	-	-	ARID2	c.1611_1626del	p.D537Efs*15
118L7212A1		-	-	Frame_Shift_Del	INS	-	-	-	KMT2D	:.192_193delins	p.R65Vfs*65
118L7212A1	17	7577534	7577534	Missense_Mutation	SNP	C	A	A	TP53	c.747G>T	p.R249S
118L7212A1		-	-	In_Frame_Del	INS	-	-	-	PIK3CA	c.1342_1353de	b.V448_G451del
118L7212A1	5	180047312	180047312	Splice_Site	SNP	C	A	A	FLT4	c.2407-4G>T	-
118L7212A1	7	2962378	2962378	Missense_Mutation	SNP	C	T	T	CARD11	c.2159G>A	p.R720Q
118L7229A1	1	16475388	16475388	Missense_Mutation	SNP	C	G	G	EPHA2	c.308G>C	p.R103P
118L7229A1		-	-	Frame_Shift_Del	INS	-	-	-	ARID1A	c.487_508del	p.A163Tfs*62
118L7229A1	2	141108580	141108580	Missense_Mutation	SNP	G	C	C	LRP1B	c.11678C>G	p.A3893G
118L7229A1	8	93107283	93107283	Missense_Mutation	SNP	G	C	C	RUNX1T1	c.238C>G	p.L80V
118L7236A1	1	27094328	27094328	Nonsense_Mutation	SNP	T	A	A	ARID1A	c.3036T>A	p.Y1012*
118L7236A1		-	-	Frame_Shift_Ins	INS	-	-	-	ACVR1B	:.1335_1338dup	p.L447Cfs*20
118L7236A1	19	1207085	1207085	Missense_Mutation	SNP	G	T	T	STK11	c.173G>T	p.G58V
118L7236A1	2	148680663	148680663	Missense_Mutation	SNP	G	A	A	ACVR2A	c.1199G>A	p.R400H
118L7236A1	X	129159276	129159276	Missense_Mutation	SNP	C	T	T	BCORL1	c.4000C>T	p.R1334W
118L7265A2	12	25398284	25398284	Missense_Mutation	SNP	C	T	T	KRAS	c.35G>A	p.G12D
118L7265A2	13	110434682	110434682	Missense_Mutation	SNP	C	T	T	IRS2	c.3719G>A	p.R1240H
118L7265A2	17	7578265	7578265	Missense_Mutation	SNP	A	G	G	TP53	c.584T>C	p.I195T
118L7265A2	2	141641467	141641467	Missense_Mutation	SNP	T	C	C	LRP1B	c.4088A>G	p.K1363R
118L7265A2	2	202136270	202136270	Missense_Mutation	SNP	A	G	G	CASP8	c.433A>G	p.S145G
118L7275A1	12	25398284	25398284	Missense_Mutation	SNP	C	T	T	KRAS	c.35G>A	p.G12D
118L7275A1	17	7578382	7578382	Nonsense_Mutation	SNP	G	T	T	TP53	c.548C>A	p.S183*
118L7275A1	9	135787732	135787732	Missense_Mutation	SNP	G	A	A	TSC1	c.850C>T	p.R284C
118L7370A1		-	-	Frame_Shift_Del	INS	-	-	-	KRAS	c.498_499del	p.H166Qfs*9
118L7370A1	12	25398285	25398285	Missense_Mutation	SNP	C	A	A	KRAS	c.34G>T	p.G12C
118L7370A1	22	24145600	24145600	Missense_Mutation	SNP	A	T	T	SMARCB1	c.619A>T	p.N207Y
118L7386A1	1	27099947	27099947	Nonsense_Mutation	SNP	C	T	T	ARID1A	c.3826C>T	p.R1276*
118L7386A1	14	75748096	75748096	Nonsense_Mutation	SNP	C	A	A	FOS	c.1112C>A	p.S371*
118L7386A1	17	59793342	59793342	Missense_Mutation	SNP	G	A	A	BRIP1	c.2462C>T	p.A821V
118L7386A1	22	21343161	21343161	Missense_Mutation	SNP	G	A	A	LZTR1	c.593G>A	p.R198K

118L7386A1		-	-	Missense_Mutation	SNP	-	-	-	PIK3CA	C.328_330del	P.E110del
118L7386A1		-	-	In_Frame_Del	INS	-	-	-	PDGFRB	C.181_204del	P.V61_E68del
118L7386A1	7	6426900	6426900	Missense_Mutation	SNP	A	C	C	RAC1	c.93A>C	p.E31D
118L7386A1	7	81336624	81336624	Missense_Mutation	SNP	C	T	T	HGF	c.1598G>A	p.R533Q
118L7386A1	8	48711869	48711869	Missense_Mutation	SNP				PRKDC	c.10198T>G	p.W3400G
118L7404M1	1	120466361	120466361	Missense_Mutation	SNP	T	G	G	NOTCH2	c.4758A>C	p.E1586D
118L7404M1	1	158609445	158609445	Missense_Mutation	SNP	G	C	C	SPTA1	c.4907C>G	p.A1636G
118L7404M1	12	49432299	49432299	Missense_Mutation	SNP	T	G	G	KMT2D	c.8840A>C	p.H2947P
118L7404M1	12	57560824	57560824	Missense_Mutation	SNP	C	T	T	LRP1	c.2909C>T	p.S970L
118L7404M1	13	25026701	25026701	Missense_Mutation	SNP	A	G	G	PARP4	c.2857T>C	p.F953L
118L7404M1	16	3786029	3786029	Splice_Site	SNP	G	A	A	CREBBP	c.4728+8C>T	-
118L7404M1	17	7578263	7578263	Nonsense_Mutation	SNP	G	A	A	TP53	c.586C>T	p.R196*
118L7404M1	19	15353845	15353845	Missense_Mutation	SNP	G	A	A	BRD4	c.3035C>T	p.P1012L
118L7404M1	2	170002327	170002327	Missense_Mutation	SNP	C	G	G	LRP2	c.12918G>C	p.E4306D
118L7404M1	22	24176339	24176339	Missense_Mutation	SNP	G	A	A	SMARCB1	c.1130G>A	p.R377H
118L7404M1	4	66356209	66356209	Missense_Mutation	SNP	C	T	T	EPHA5	c.1288G>A	p.D430N
118L7404M1	5	176636872	176636872	Missense_Mutation	SNP	A	G	G	NSD1	c.1472A>G	p.E491G
118L7404M1	6	157405841	157405841	Missense_Mutation	SNP	A	G	G	ARID1B	c.2083A>G	p.T695A
118L7404M1	7	18833040	18833040	Missense_Mutation	SNP	G	A	A	HDAC9	c.2278G>A	p.E760K
118L7404M1	9	97933432	97933432	Splice_Site	SNP	A	G	G	FANCC	c.457-7T>C	-
118L7413A1		-	-	In_Frame_Ins	INS	-	-	-	PTEN	c.439_456dup	).K147_L152dup
118L7413A1	7	142640096	142640096	Missense_Mutation	SNP	C	T	T	KEL	c.1807G>A	p.A603T
118L7497M1		-	-	Frame_Shift_Ins	INS	-	-	-	ARID1A	c.961_986dup	p.A330Rfs*42
118L7497M1	1	204499861	204499861	Missense_Mutation	SNP	A	G	G	MDM4	c.203A>G	p.Q68R
118L7497M1	10	88659835	88659835	Missense_Mutation	SNP	C	A	A	BMPR1A	c.482C>A	p.A161D
118L7497M1	11	92616320	92616320	Missense_Mutation	SNP	G	A	A	FAT3	c.12698G>A	p.R4233H
118L7497M1	13	28897002	28897002	Missense_Mutation	SNP	C	T	T	FLT1	c.2878G>A	p.V960I
118L7497M1	16	67644855	67644855	Missense_Mutation	SNP	A	T	T	CTCF	c.120A>T	p.L40F
118L7497M1	17	80789046	80789046	Missense_Mutation	SNP	C	T	T	ZNF750	c.1285G>A	p.G429S
118L7497M1	18	48593533	48593533	Missense_Mutation	SNP	G	T	T	SMAD4	c.1284G>T	p.K428N
118L7497M1	2	121746077	121746077	Missense_Mutation	SNP	T	G	G	GLI2	c.2587T>G	p.S863A
118L7497M1	X	44969471	44969471	Nonsense_Mutation	SNP	C	T	T	KDM6A	c.4153C>T	p.Q1385*
118L7516M1	12	121438953	121438953	Missense_Mutation	SNP	C	G	G	HNF1A	c.1854C>G	p.I618M
118L7516M1	13	49039470	49039470	Missense_Mutation	SNP	C	G	G	RB1	c.2455C>G	p.L819V
118L7516M1		-	-	In_Frame_Del	INS	-	-	-	IRS2	C.2096_2104de	P.A699_A701del
118L7516M1	14	95562437	95562437	Missense_Mutation	SNP	C	T	T	DICER1	c.4820G>A	p.R1607Q
118L7516M1	16	2124198	2124198	Splice_Site	SNP	C	T	T	TSC2	c.2356-3C>T	-
118L7516M1	16	56871605	56871605	Missense_Mutation	SNP	G	A	A	NUP93	c.1985G>A	p.R662K
118L7516M1	2	170034537	170034537	Splice_Site	SNP	C	A	A	LRP2	c.10170-1G>T	-
118L7516M1	2	209113113	209113113	Missense_Mutation	SNP	G	A	A	IDH1	c.394C>T	p.R132C
118L7516M1	3	47165993	47165993	Missense_Mutation	SNP	T	C	C	SETD2	c.133A>G	p.M45V
118L7516M1	3	195609000	195609000	Missense_Mutation	SNP	T	C	C	TNK2	c.809A>G	p.D270G
118L7516M1	4	1806153	1806153	Missense_Mutation	SNP	C	T	T	FGFR3	c.1172C>T	p.A391V
118L7516M1	4	187584755	187584755	Missense_Mutation	SNP	G	A	A	FAT1	c.3278C>T	p.T1093M
118L7537A1	1	27057961	27057961	Nonsense_Mutation	SNP	C	T	T	ARID1A	c.1669C>T	p.Q557*
118L7537A1		-	-	Splice_Site	SNP	-	-	-	SPTA1	376-30_3376-11	-
118L7537A1	17	7577539	7577539	Missense_Mutation	SNP	G	A	A	TP53	c.742C>T	p.R248W
118L7537A1	18	48575152	48575152	Missense_Mutation	SNP	C	A	A	SMAD4	c.346C>A	p.Q116K
118L7537A1		-	-	Frame_Shift_Del	INS	-	-	-	ARID1B	C.1160_1200de	P.A387Gfs*134
118L7537A1	7	140453155	140453155	Missense_Mutation	SNP	C	T	T	BRAF	c.1780G>A	p.D594N
118L7537A1	X	44929085	44929085	Nonsense_Mutation	SNP	G	T	T	KDM6A	c.2185G>T	p.E729*
118L7537A1	X	47032526	47032526	Splice_Site	SNP	G	T	T	RBM10	c.433-1G>T	-
118L7560M1	2	209113113	209113113	Missense_Mutation	SNP	G	A	A	IDH1	c.394C>T	p.R132C
118L7560M1		-	-	Frame_Shift_Del	INS	-	-	-	PBRM1	c.818_860del	p.A273Vfs*13
118L7574A1	17	7577609	7577609	Splice_Site	SNP	C	T	T	TP53	c.673-1G>A	-
118L7574A1	19	11123735	11123735	Nonsense_Mutation	SNP	C	A	A	SMARCA4	c.2385C>A	p.Y795*
118L7574A1	9	21971111	21971111	Missense_Mutation	SNP	G	A	A	CDKN2A	c.247C>T	p.H83Y
118L7574A1	X	76814140	76814140	Missense_Mutation	SNP	C	A	A	ATRX	c.6504G>T	p.Q2168H
118L7607A1	17	7577538	7577538	Missense_Mutation	SNP	C	T	T	TP53	c.743G>A	p.R248Q
118L7607A1	9	93641101	93641101	Missense_Mutation	SNP	A	G	G	SYK	c.1378A>G	p.K460E
118L7654M1		-	-	Frame_Shift_Ins	INS	-	-	-	ARID1A	C.748_758dup	P.S255Rfs*112
118L7654M1	12	49444501	49444501	Nonsense_Mutation	SNP	A	T	T	KMT2D	c.2870T>A	p.L957*
118L7654M1	14	102548726	102548726	Missense_Mutation	SNP	T	C	C	HSP90AA1	c.2177A>G	p.Y726C
118L7654M1	2	209113113	209113113	Missense_Mutation	SNP	G	A	A	IDH1	c.394C>T	p.R132C
118L7654M1	7	151846234	151846234	Missense_Mutation	SNP	A	C	C	KMT2C	c.12778T>G	p.S4260A
118L7654M1		-	-	Splice_Site	SNP	-	-	-	KMT2C	2775-26_12775-	-
118L7654M1	7	151893098	151893098	Splice_Site	SNP	T	A	A	KMT2C	c.4274-2A>T	-
118L7654M1		-	-	Frame_Shift_Del	INS	-	-	-	PTCH1	C.2144del	P.F715Sfs*31
118L7656M1		-	-	Frame_Shift_Del	INS	-	-	-	TNFRSF14	c.218_219del	p.T73Sfs*3
118L7656M1		-	-	Frame_Shift_Del	INS	-	-	-	EPHA2	c.987del	p.S330Pfs*63
118L7656M1		-	-	Frame_Shift_Ins	INS	-	-	-	ARID1A	c.676_679dup	p.P227Rfs*174
118L7656M1		-	-	Missense_Mutation	SNP	-	-	-	RARA	c.499_501del	p.K167del
118L7656M1		-	-	Frame_Shift_Del	INS	-	-	-	BCOR	c.524_527del	p.K175Rfs*40
118L7756A1		-	-	Frame_Shift_Ins	INS	-	-	-	ARID1A	C.3121dup	P.L1041Pfs*6
118L7756A1	10	89690802	89690802	Splice_Site	SNP	G	C	C	PTEN	c.210-1G>C	-
118L7756A1		-	-	Frame_Shift_Ins	INS	-	-	-	SMAD4	C.421dup	P.I141Nfs*2
118L7756A1	20	57484421	57484421	Missense_Mutation	SNP	G	A	A	GNAS	c.602G>A	p.R201H
118L7756A1	7	140481412	140481412	Missense_Mutation	SNP	C	G	G	BRAF	c.1396G>C	p.G466R
118L7757A1		-	-	Frame_Shift_Ins	INS	-	-	-	EPHA2	c.1153dup	p.Y385Lfs*69
118L7757A1	1	158612266	158612266	Missense_Mutation	SNP	C	T	T	SPTA1	c.4672G>A	p.V1558I
118L7757A1		-	-	Splice_Site	SNP	-	-	-	PARP1	c.2848+11del	-
118L7757A1	12	25380276	25380276	Missense_Mutation	SNP	T	A	A	KRAS	c.182A>T	p.Q61L
118L7757A1		-	-	Frame_Shift_Ins	INS	-	-	-	TP53	c.267dup	p.S90Lfs*59
118L7757A1	17	56440631	56440631	Splice_Site	SNP	C	G	G	RNF43	c.582+5G>C	-
118L7757A1	2	141643893	141643893	Missense_Mutation	SNP	C	T	T	LRP1B	c.3778G>A	p.A1260T
118L7757A1	2	170101208	170101208	Missense_Mutation	SNP	T	C	C	LRP2	c.3425A>G	p.N1142S
118L7757A1	2	212295774	212295774	Missense_Mutation	SNP	G	A	A	ERBB4	c.2539C>T	p.R847C
118L7757A1		-	-	Frame_Shift_Del	INS	-	-	-	CDKN2A	c.118_142del	p.A40Rfs*5
118L7820J1	1	158641959	158641959	Missense_Mutation	SNP	C	T	T	SPTA1	c.1378G>A	p.A460T
118L7820J1		-	-	Frame_Shift_Del	INS	-	-	-	ATM	C.3272_3273del	P.E1091Vfs*4
118L7820J1		-	-	Frame_Shift_Del	INS	-	-	-	PARP4	C.3032_3071de	P.H1011Lfs*16
118L7820J1	13	102375233	102375233	Missense_Mutation	SNP	G	A	A	FGF14	c.707C>T	p.A236V
118L7820J1	17	7574021	7574021	Nonsense_Mutation	SNP	C	A	A	TP53	c.1006G>T	p.E336*
118L7820J1		-	-	Missense_Mutation	SNP	-	-	-	ASXL1	3478_3479delins	p.G1160F
118L7820J1	3	52439898	52439898	Missense_Mutation	SNP	G	C	C	BAP1	c.814C>G	p.Q272E
118L7820J1	8	68999961	68999961	Missense_Mutation	SNP	C	A	A	PREX2	c.2030C>A	p.T677K
118L7848M1		-	-	Splice_Site	SNP	-	-	-	EPHA2	1865-10_1865-1	-
118L7848M1	12	25398284	25398284	Missense_Mutation	SNP	C	A	A	KRAS	c.35G>T	p.G12V

118L7848M1	12	133225654	133225654	Missense_Mutation	SNP	C	T	T	POLE	c.4010G>A	p.S1337N
118L7848M1	20	30662502	30662502	Missense_Mutation	SNP	G	A	A	HCK	c.343G>A	p.V115I
118L7848M1	4	20620585	20620585	Nonsense_Mutation	SNP	G	T	T	SLIT2	c.4543G>T	p.E1515*
118L7848M1		-	-	Frame_Shift_Del	INS	-	-	-	BCORL1	C.2228del	P.R743Pfs*40
118L7853A1		-	-	Frame_Shift_Del	INS	-	-	-	ARID1A	c.4912del	p.D1638Ifs*16
118L7853A1	12	46285669	46285669	Nonsense_Mutation	SNP	C	T	T	ARID2	c.5029C>T	p.R1677*
118L7853A1	15	88423551	88423551	Missense_Mutation	SNP	C	T	T	NTRK3	c.2242G>A	p.E748K
118L7853A1		-	-	Missense_Mutation	SNP	-	-	-	TP53	000_1001delins	p.G334L
118L7860A1	1	78430001	78430001	Missense_Mutation	SNP	C	T	T	FUBP1	c.878G>A	p.R293K
118L7860A1	11	92577541	92577541	Missense_Mutation	SNP	C	T	T	FAT3	c.11008C>T	p.R3670W
118L7860A1	12	46243472	46243472	Nonsense_Mutation	SNP	C	T	T	ARID2	c.1825C>T	p.Q609*
118L7860A1	12	46243484	46243484	Nonsense_Mutation	SNP	C	T	T	ARID2	c.1837C>T	p.Q613*
118L7860A1	12	46244709	46244709	Nonsense_Mutation	SNP	C	T	T	ARID2	c.2803C>T	p.Q935*
118L7860A1	12	46254641	46254641	Nonsense_Mutation	SNP	C	T	T	ARID2	c.4831C>T	p.Q1611*
118L7860A1	17	7577085	7577085	Nonsense_Mutation	SNP	C	A	A	TP53	c.853G>T	p.E285*
118L7860A1	17	7577538	7577538	Missense_Mutation	SNP	C	T	T	TP53	c.743G>A	p.R248Q
118L7860A1	17	48263773	48263773	Missense_Mutation	SNP	G	C	C	COL1A1	c.3910C>G	p.Q1304E
118L7860A1	2	170013946	170013946	Missense_Mutation	SNP	C	G	G	LRP2	c.11954G>C	p.G3985A
118L7860A1	3	178936037	178936037	Missense_Mutation	SNP	G	A	A	PIK3CA	c.1579G>A	p.D527N
118L7860A1	3	178936091	178936091	Missense_Mutation	SNP	G	A	A	PIK3CA	c.1633G>A	p.E545K
118L7860A1	3	183226037	183226037	Missense_Mutation	SNP	A	T	T	KLHL6	c.719T>A	p.M240K
118L7860A1	4	110901298	110901298	Splice_Site	SNP	A	G	G	EGF	c.2221+3A>G	-
118L7860A1	5	176665478	176665478	Missense_Mutation	SNP	G	C	C	NSD1	c.4162G>C	p.E1388Q
118L7860A1	6	117714466	117714466	Missense_Mutation	SNP	C	G	G	ROS1	c.1183G>C	p.E395Q
118L7860A1	8	11566174	11566174	Missense_Mutation	SNP	C	T	T	GATA4	c.353C>T	p.A118V
118L7860A1	9	21971036	21971036	Missense_Mutation	SNP	C	T	T	CDKN2A	c.322G>A	p.D108N
118L7867A1		-	-	Frame_Shift_Del	INS	-	-	-	ARID1A	c.3300del	p.Y1101Mfs*60
118L7867A1	12	56478983	56478983	Missense_Mutation	SNP	C	T	T	ERBB3	c.439C>T	p.P147S
118L7867A1	15	93486100	93486100	Missense_Mutation	SNP	C	T	T	CHD2	c.854C>T	p.A285V
118L7867A1		-	-	Frame_Shift_Del	INS	-	-	-	TP53	c.46del	p.Q16Rfs*28
118L7867A1	19	10602745	10602745	Missense_Mutation	SNP	G	A	A	KEAP1	c.833C>T	p.P278L
118L7867A1	19	15299917	15299917	Missense_Mutation	SNP	G	A	A	NOTCH3	c.1261C>T	p.R421C
118L7867A1	2	212286794	212286794	Nonsense_Mutation	SNP	T	A	A	ERBB4	c.2902A>T	p.K968*
118L7867A1	3	142278223	142278223	Nonsense_Mutation	SNP	C	T	T	ATR	c.1602G>A	p.W534*
118L7867A1	6	157505389	157505389	Nonsense_Mutation	SNP	G	T	T	ARID1B	c.3370G>T	p.E1124*
118L7895A1	1	16460082	16460082	Missense_Mutation	SNP	C	A	A	EPHA2	c.1758G>T	p.K586N
118L7895A1		-	-	In_Frame_Del	INS	-	-	-	TP53	c.794_817del	p.L265_V272del
118L7895A1	9	21971194	21971194	Missense_Mutation	SNP	C	T	T	CDKN2A	c.164G>A	p.G55D
118L7895A1	X	123019820	123019820	Missense_Mutation	SNP	C	T	T	XIAP	c.308C>T	p.T103M
118L8016M1	12	25398284	25398284	Missense_Mutation	SNP	C	A	A	KRAS	c.35G>T	p.G12V
118L8016M1	16	354440	354440	Missense_Mutation	SNP	C	T	T	AXIN1	c.1118G>A	p.R373H
118L8016M1	17	7578508	7578508	Missense_Mutation	SNP	C	T	T	TP53	c.422G>A	p.C141Y
118L8016M1	17	37880257	37880257	Missense_Mutation	SNP	C	G	G	ERBB2	c.2301C>G	p.I767M
118L8016M1	2	198267360	198267360	Missense_Mutation	SNP	T	G	G	SF3B1	c.1997A>C	p.K666T
118L8016M1	4	88996022	88996022	Missense_Mutation	SNP	G	A	A	PKD2	c.2581G>A	p.D861N
118L8016M1	7	116412023	116412023	Missense_Mutation	SNP	A	G	G	MET	c.3008A>G	p.Y1003C
118L8016M1	8	69017412	69017412	Missense_Mutation	SNP	C	T	T	PREX2	c.2755C>T	p.H919Y
118L8023M2		-	-	In_Frame_Ins	INS	-	-	-	EPHA2	c.719dup	p.E241*
118L8023M2	13	32912432	32912432	Nonsense_Mutation	SNP	A	T	T	BRCA2	c.3940A>T	p.K1314*
118L8023M2	20	31023598	31023598	Missense_Mutation	SNP	C	G	G	ASXL1	c.3083C>G	p.S1028W
118L8023M2	3	52443864	52443864	Missense_Mutation	SNP	C	T	T	BAP1	c.31G>A	p.D11N
118L8029M1		-	-	Frame_Shift_Del	INS	-	-	-	SPEN	C.3154del	P.I1052Sfs*40
118L8029M1		-	-	Frame_Shift_Del	INS	-	-	-	SPEN	C.7484del	P.P2495Lfs*4
118L8029M1		-	-	Frame_Shift_Del	INS	-	-	-	EPHA2	C.843del	P.F281Lfs*112
118L8029M1	1	27056238	27056238	Nonsense_Mutation	SNP	C	T	T	ARID1A	c.1234C>T	p.Q412*
118L8029M1		-	-	Frame_Shift_Del	INS	-	-	-	JAK1	C.2580del	P.K860Nfs*16
118L8029M1		-	-	Missense_Mutation	SNP	-	-	-	JAK1	C.2118_2120de	P.E706del
118L8029M1	1	241665738	241665738	Splice_Site	SNP	C	A	A	FH	c.1236+5G>T	-
118L8029M1		-	-	Frame_Shift_Del	INS	-	-	-	CHD4	C.218del	P.K73Rfs*129
118L8029M1		-	-	Frame_Shift_Del	INS	-	-	-	HNF1A	C.872del	P.P291Qfs*51
118L8029M1		-	-	Frame_Shift_Del	INS	-	-	-	BRCA2	C.1813del	P.I605Yfs*9
118L8029M1	14	36987159	36987159	Missense_Mutation	SNP	C	T	T	NKX2-1	c.530G>A	p.G177E
118L8029M1		-	-	Frame_Shift_Del	INS	-	-	-	B2M	C.43_44del	P.L15Ffs*41
118L8029M1	15	67477113	67477113	Missense_Mutation	SNP	G	A	A	SMAD3	c.920G>A	p.C307Y
118L8029M1	16	10274135	10274135	Missense_Mutation	SNP	T	C	C	GRIN2A	c.134A>G	p.D45G
118L8029M1	17	7578388	7578388	Missense_Mutation	SNP	C	T	T	TP53	c.542G>A	p.R181H
118L8029M1		-	-	Frame_Shift_Del	INS	-	-	-	RNF43	C.1943_1944de	P.S648Cfs*98
118L8029M1	19	15299971	15299971	Missense_Mutation	SNP	C	T	T	NOTCH3	c.1207G>A	p.E403K
118L8029M1	2	141122273	141122273	Missense_Mutation	SNP	C	A	A	LRP1B	c.11088G>T	p.E3696D
118L8029M1		-	-	Frame_Shift_Del	INS	-	-	-	ACVR2A	C.1310del	P.K437Rfs*5
118L8029M1		-	-	Splice_Site	SNP	-	-	-	CASP8	23-6_-123-3delT	-
118L8029M1		-	-	Frame_Shift_Ins	INS	-	-	-	ERBB4	C.1798dup	P.A600Gfs*10
118L8029M1		-	-	Frame_Shift_Del	INS	-	-	-	TGFBR2	C.383del	P.K128Sfs*35
118L8029M1		-	-	Frame_Shift_Del	INS	-	-	-	TGFBR2	C.1229_1230de	P.V410Gfs*2
118L8029M1	3	37090440	37090440	Nonsense_Mutation	SNP	G	T	T	MLH1	c.2035G>T	p.E679*
118L8029M1		-	-	In_Frame_Del	INS	-	-	-	PBRM1	C.159_160del	P.Y54*
118L8029M1	3	89456470	89456470	Missense_Mutation	SNP	C	T	T	EPHA3	c.1646C>T	p.A549V
118L8029M1	3	178936082	178936082	Missense_Mutation	SNP	G	A	A	PIK3CA	c.1624G>A	p.E542K
118L8029M1		-	-	Frame_Shift_Del	INS	-	-	-	SLIT2	C.2712del	P.K904Nfs*13
118L8029M1	4	126336687	126336687	Missense_Mutation	SNP	G	A	A	FAT4	c.6569G>A	p.R2190H
118L8029M1	4	153250856	153250856	Missense_Mutation	SNP	T	C	C	FBXW7	c.1204A>G	p.T402A
118L8029M1		-	-	Frame_Shift_Del	INS	-	-	-	RAD50	C.2165del	P.K722Rfs*14
118L8029M1	5	149497260	149497260	Missense_Mutation	SNP	C	T	T	PDGFRB	c.3058G>A	p.D1020N
118L8029M1	5	156671313	156671313	Missense_Mutation	SNP	G	A	A	ITK	c.1274G>A	p.C425Y
118L8029M1	5	176666797	176666797	Missense_Mutation	SNP	A	T	T	NSD1	c.4233A>T	p.K1411N
118L8029M1	5	180058718	180058718	Missense_Mutation	SNP	A	G	G	FLT4	c.119T>C	p.V40A
118L8029M1		-	-	Splice_Site	SNP	-	-	-	FYN	c.345-9delT	-
118L8029M1	8	32621591	32621591	Missense_Mutation	SNP	G	A	A	NRG1	c.1123G>A	p.E375K
118L8029M1		-	-	Splice_Site	SNP	-	-	-	NBN	c.1398-10delT	-
118L8029M1	X	76909699	76909699	Splice_Site	SNP	A	C	C	ATRX	c.4215-9T>G	-
118L8044A1	1	16258127	16258127	Nonsense_Mutation	SNP	C	T	T	SPEN	c.5392C>T	p.Q1798*
118L8044A1	1	32741204	32741204	Missense_Mutation	SNP	G	A	A	LCK	c.412G>A	p.E138K
118L8044A1	1	204506578	204506578	Missense_Mutation	SNP	C	G	G	MDM4	c.364C>G	p.L122V
118L8044A1	1	206648248	206648248	Missense_Mutation	SNP	C	G	G	IKBKE	c.269C>G	p.S90C
118L8044A1	11	22646489	22646489	Nonsense_Mutation	SNP	G	A	A	FANCF	c.868C>T	p.Q290*
118L8044A1	11	92564779	92564779	Splice_Site	SNP	C	T	T	FAT3	c.9482-9C>T	-
118L8044A1	12	46254583	46254583	Splice_Site	SNP	G	C	C	ARID2	c.4774-1G>C	-
118L8044A1	12	133233991	133233991	Missense_Mutation	SNP	C	G	G	POLE	c.3403G>C	p.E1135Q

118L8044A1	12	133244079	133244079	Splice_Site	SNP	C	T	T	POLE	c.2319+10G>A	-
118L8044A1	13	25072298	25072298	Missense_Mutation	SNP	G	T	T	PARP4	c.547C>A	p.P183T
118L8044A1	13	48934254	48934254	Missense_Mutation	SNP	G	C	C	RB1	c.709G>C	p.E237Q
118L8044A1	14	81574987	81574987	Missense_Mutation	SNP	G	A	A	TSHR	c.724G>A	p.E242K
118L8044A1	15	75092849	75092849	Splice_Site	SNP	G	C	C	CSK	c.556+3G>C	-
118L8044A1	15	88690645	88690645	Splice_Site	SNP	G	C	C	NTRK3	c.396-11C>G	-
118L8044A1	15	99465606	99465606	Missense_Mutation	SNP	G	A	A	IGF1R	c.2431G>A	p.E811K
118L8044A1	17	7578406	7578406	Missense_Mutation	SNP	C	T	T	TP53	c.524G>A	p.R175H
118L8044A1	18	739737	739737	Nonsense_Mutation	SNP	G	A	A	YES1	c.1135C>T	p.Q379*
118L8044A1	19	11097056	11097056	Nonsense_Mutation	SNP	C	T	T	SMARCA4	c.547C>T	p.Q183*
118L8044A1	2	11337318	11337318	Missense_Mutation	SNP	C	A	A	ROCK2	c.3436G>T	p.D1146Y
118L8044A1	2	47606929	47606929	Missense_Mutation	SNP	C	T	T	EPCAM	c.679C>T	p.H227Y
118L8044A1	3	47061326	47061326	Missense_Mutation	SNP	G	A	A	SETD2	c.7355C>T	p.S2452L
118L8044A1	4	55565800	55565800	Missense_Mutation	SNP	C	G	G	KIT	c.624C>G	p.F208L
118L8044A1	5	176638948	176638948	Missense_Mutation	SNP	C	T	T	NSD1	c.3548C>T	p.S1183F
118L8044A1	7	20199233	20199233	Missense_Mutation	SNP	G	C	C	MACC1	c.751C>G	p.L251V
118L8044A1	7	127544837	127544837	Nonsense_Mutation	SNP	G	T	T	SND1	c.1492G>T	p.E498*
118L8044A1	8	93107578	93107578	Missense_Mutation	SNP	C	T	T	RUNX1T1	c.118G>A	p.E40K
118L8044A1	9	27228199	27228199	Splice_Site	SNP	C	T	T	TEK	c.3201-5C>T	-
118L8044A1	X	15552336	15552336	Missense_Mutation	SNP	G	A	A	BMX	c.1021G>A	p.D341N
118L8044A1	X	70339981	70339981	Missense_Mutation	SNP	G	A	A	MED12	c.514G>A	p.E172K
118L8044A1	X	70643021	70643021	Missense_Mutation	SNP	G	T	T	TAF1	c.4567G>T	p.D1523Y
118L8044A1	1	16258127	16258127	Nonsense_Mutation	SNP	C	T	T	SPEN	c.5392C>T	p.Q1798*
118L8044A1		-	-	In_Frame_Del	INS	-	-	-	SPEN	c.5997_6004del	p.G2000*
118L8044A1	1	204506578	204506578	Missense_Mutation	SNP	C	G	G	MDM4	c.364C>G	p.L122V
118L8044A1	1	206648248	206648248	Missense_Mutation	SNP	C	G	G	IKBKE	c.269C>G	p.S90C
118L8044A1	10	114925435	114925435	Missense_Mutation	SNP	C	G	G	TCF7L2	c.1495C>G	p.R499G
118L8044A1	11	22646489	22646489	Nonsense_Mutation	SNP	G	A	A	FANCF	c.868C>T	p.Q290*
118L8044A1		-	-	Frame_Shift_Del	INS	-	-	-	MS4A1	c.478_487del	p.P160Tfs*34
118L8044A1	11	92564779	92564779	Splice_Site	SNP	C	T	T	FAT3	c.9482-9C>T	-
118L8044A1	12	46254583	46254583	Splice_Site	SNP	G	C	C	ARID2	c.4774-1G>C	-
118L8044A1	12	133244079	133244079	Splice_Site	SNP	C	T	T	POLE	c.2319+10G>A	-
118L8044A1	13	25072298	25072298	Missense_Mutation	SNP	G	T	T	PARP4	c.547C>A	p.P183T
118L8044A1	13	48934254	48934254	Missense_Mutation	SNP	G	C	C	RB1	c.709G>C	p.E237Q
118L8044A1	14	81574987	81574987	Missense_Mutation	SNP	G	A	A	TSHR	c.724G>A	p.E242K
118L8044A1	15	75092849	75092849	Splice_Site	SNP	G	C	C	CSK	c.556+3G>C	-
118L8044A1	15	88690645	88690645	Splice_Site	SNP	G	C	C	NTRK3	c.396-11C>G	-
118L8044A1	15	99465606	99465606	Missense_Mutation	SNP	G	A	A	IGF1R	c.2431G>A	p.E811K
118L8044A1	18	739737	739737	Nonsense_Mutation	SNP	G	A	A	YES1	c.1135C>T	p.Q379*
118L8044A1	19	11097056	11097056	Nonsense_Mutation	SNP	C	T	T	SMARCA4	c.547C>T	p.Q183*
118L8044A1	2	11337318	11337318	Missense_Mutation	SNP	C	A	A	ROCK2	c.3436G>T	p.D1146Y
118L8044A1	3	47061326	47061326	Missense_Mutation	SNP	G	A	A	SETD2	c.7355C>T	p.S2452L
118L8044A1	3	178936091	178936091	Missense_Mutation	SNP	G	A	A	PIK3CA	c.1633G>A	p.E545K
118L8044A1	4	55565800	55565800	Missense_Mutation	SNP	C	G	G	KIT	c.624C>G	p.F208L
118L8044A1	4	153268144	153268144	Nonsense_Mutation	SNP	G	A	A	FBXW7	c.664C>T	p.R222*
118L8044A1	5	176638948	176638948	Missense_Mutation	SNP	C	T	T	NSD1	c.3548C>T	p.S1183F
118L8044A1	7	20199233	20199233	Missense_Mutation	SNP	G	C	C	MACC1	c.751C>G	p.L251V
118L8044A1	7	127544837	127544837	Nonsense_Mutation	SNP	G	T	T	SND1	c.1492G>T	p.E498*
118L8044A1	9	5126756	5126756	Nonsense_Mutation	SNP	C	T	T	JAK2	c.3364C>T	p.R1122*
118L8044A1	X	15552336	15552336	Missense_Mutation	SNP	G	A	A	BMX	c.1021G>A	p.D341N
118L8044A1	X	63410021	63410021	Missense_Mutation	SNP	C	T	T	AMER1	c.3146G>A	p.R1049Q
118L8044A1	X	70339981	70339981	Missense_Mutation	SNP	G	A	A	MED12	c.514G>A	p.E172K
118L8044A1	X	70346933	70346933	Missense_Mutation	SNP	C	T	T	MED12	c.2800C>T	p.H934Y
118L8044A1	X	70643021	70643021	Missense_Mutation	SNP	G	T	T	TAF1	c.4567G>T	p.D1523Y
118L8083A1		-	-	Frame_Shift_Del	INS	-	-	-	KMT2D	C.5921del	P.T1974Rfs*73
118L8083A1	17	7577117	7577117	Missense_Mutation	SNP	A	C	C	TP53	c.821T>G	p.V274G
118L8083A1	3	119585439	119585439	Missense_Mutation	SNP	G	A	A	GSK3B	c.946C>T	p.R316W
118L8083A1	X	39914756	39914756	Missense_Mutation	SNP	C	T	T	BCOR	c.4504G>A	p.D1502N
118L8213A1		-	-	Frame_Shift_Del	INS	-	-	-	ARID1A	c.291_301del	p.P98Efs*9
118L8213A1	12	25398285	25398285	Missense_Mutation	SNP	C	A	A	KRAS	c.34G>T	p.G12C
118L8213A1		-	-	Frame_Shift_Ins	INS	-	-	-	LRP1	c.9496_9497ins[illegible]	p.D3166Vfs*36
118L8213A1	13	32910944	32910944	Missense_Mutation	SNP	A	T	T	BRCA2	c.2452A>T	p.N818Y
118L8213A1	16	341199	341199	Missense_Mutation	SNP	G	C	C	AXIN1	c.2285C>G	p.S762C
118L8213A1	17	7578413	7578413	Missense_Mutation	SNP	C	T	T	TP53	c.517G>A	p.V173M
118L8213A1		-	-	Frame_Shift_Ins	INS	-	-	-	RNF43	c.190dup	p.T64Nfs*11
118L8213A1	19	42796456	42796456	Missense_Mutation	SNP	A	T	T	CIC	c.3013A>T	p.T1005S
118L8213A1	5	1282656	1282656	Missense_Mutation	SNP	C	T	T	TERT	c.1657G>A	p.V553I
118L8213A1	5	38963091	38963091	Nonsense_Mutation	SNP	G	A	A	RICTOR	c.1453C>T	p.R485*
118L8213A1	6	32190870	32190870	Splice_Site	SNP	G	T	T	NOTCH4	c.74-7C>A	-
118L8213A1	X	47045475	47045475	Nonsense_Mutation	SNP	C	G	G	RBM10	c.2442C>G	p.Y814*
118L8367M1		-	-	Frame_Shift_Del	INS	-	-	-	ARID1A	2G>A;c.5944_59	1981Y;V1982Kfs*9]
118L8367M1	16	2126493	2126493	Missense_Mutation	SNP	G	T	T	TSC2	c.2744G>T	p.G915V
118L8367M1		-	-	In_Frame_Ins	INS	-	-	-	TP53	c.681dup	p.D228*
118L8367M1	17	11998897	11998897	Missense_Mutation	SNP	T	G	G	MAP2K4	c.399T>G	p.I133M
118L8367M1	19	1220698	1220698	Missense_Mutation	SNP	G	C	C	STK11	c.716G>C	p.W239S
118L8367M1	2	109368371	109368371	Missense_Mutation	SNP	T	A	A	RANBP2	c.1676T>A	p.I559K
118L8367M1	2	191898347	191898347	Missense_Mutation	SNP	A	C	C	STAT4	c.1721T>G	p.V574G
118L8367M1	3	41266097	41266097	Missense_Mutation	SNP	G	T	T	CTNNB1	c.94G>T	p.D32Y
118L8367M1	3	52437790	52437790	Missense_Mutation	SNP	C	G	G	BAP1	c.1371G>C	p.K457N
118L8367M1	3	130992335	130992335	Missense_Mutation	SNP	C	G	G	NEK11	c.1635C>G	p.I545M
118L8367M1	7	42005889	42005889	Missense_Mutation	SNP	G	A	A	GLI3	c.2782C>T	p.R928C
118L8367M1		-	-	In_Frame_Del	INS	-	-	-	CFTR	4230_4236delin	Q1411_Q1412del
118L8367M1	9	135786877	135786877	Missense_Mutation	SNP	C	A	A	TSC1	c.992G>T	p.S331I
118L8370M1	15	49716538	49716538	Missense_Mutation	SNP	A	G	G	FGF7	c.44A>G	p.Y15C
118L8370M1	17	56437605	56437605	Missense_Mutation	SNP	C	T	T	RNF43	c.857G>A	p.R286Q
118L8370M1	17	59878736	59878736	Missense_Mutation	SNP	G	A	A	BRIP1	c.1018C>T	p.L340F
118L8370M1	19	11152182	11152182	Missense_Mutation	SNP	A	G	G	SMARCA4	c.4466A>G	p.N1489S
118L8370M1	19	41759543	41759543	Missense_Mutation	SNP	A	G	G	AXL	c.1939A>G	p.I647V
118L8370M1	4	55136826	55136826	Missense_Mutation	SNP	G	A	A	PDGFRA	c.1148G>A	p.R383H
118L8370M1	4	66197706	66197706	Missense_Mutation	SNP	G	T	T	EPHA5	c.2993C>A	p.A998D
118L8370M1	5	121736798	121736798	Missense_Mutation	SNP	G	T	T	SNCAIP	c.175G>T	p.A59S
118L8370M1	6	117677785	117677785	Splice_Site	SNP	A	T	T	ROS1	c.4141+7T>A	-
118L8370M1	8	71036972	71036972	Missense_Mutation	SNP	C	T	T	NCOA2	c.4045G>A	p.A1349T
118L8457A1	14	105239924	105239924	Splice_Site	SNP	G	A	A	AKT1	c.703-7C>T	-
118L8457A1	17	7577120	7577120	Missense_Mutation	SNP	C	A	A	TP53	c.818G>T	p.R273L
118L8457A1	17	47677762	47677762	Missense_Mutation	SNP	C	T	T	SPOP	c.1103G>A	p.R368H
118L8457A1	2	29519851	29519851	Missense_Mutation	SNP	C	T	T	ALK	c.1720G>A	p.G574R
118L8457A1	2	141130655	141130655	Nonsense_Mutation	SNP	G	A	A	LRP1B	c.10690C>T	p.Q3564*

118L8457A1	2	141609252	141609252	Missense_Mutation	SNP	C	A	A	LRP1B	c.4680G>T	p.M1560I
118L8457A1	2	212289029	212289029	Splice_Site	SNP	A	G	G	ERBB4	c.2720-3T>C	-
118L8457A1	5	176619009	176619009	Nonsense_Mutation	SNP	C	G	G	NSD1	c.1052C>G	p.S351*
118L8457A1	7	141408647	141408647	Missense_Mutation	SNP	T	C	C	WEE2	c.89T>C	p.V30A
118L8475A1		-	-	Frame_Shift_Del	INS	-	-	-	ARID1A	c.3530del	p.P1177Qfs*3
118L8475A1	10	123325014	123325014	Missense_Mutation	SNP	T	C	C	FGFR2	c.314A>G	p.Y105C
118L8475A1	16	396149	396149	Missense_Mutation	SNP	T	G	G	AXIN1	c.877A>C	p.R293R
118L8475A1	17	29562641	29562641	Nonsense_Mutation	SNP	C	T	T	NF1	c.3721C>T	p.R1241*
118L8475A1	3	52620644	52620644	Missense_Mutation	SNP	G	C	C	PBRM1	c.3109C>G	p.R1037G
118L8475A1		-	-	Frame_Shift_Del	INS	-	-	-	IRF2	c.175del	p.A59Qfs*11
118L8475A1	9	139396719	139396719	Splice_Site	SNP	C	T	T	NOTCH1	c.5384+5G>A	-
118L8475A1	9	139413166	139413166	Missense_Mutation	SNP	C	T	T	NOTCH1	c.976G>A	p.G326S
118L8475A1	X	70344083	70344083	Missense_Mutation	SNP	G	A	A	MED12	c.1819G>A	p.D607N
118L8606A1		-	-	Frame_Shift_Del	INS	-	-	-	ARID1A	c.867del	p.T290Pfs*73
118L8606A1	17	7577094	7577094	Missense_Mutation	SNP	G	A	A	TP53	c.844C>T	p.R282W
118L8606A1	17	29553452	29553452	Splice_Site	SNP	G	T	T	NF1	c.2002-1G>T	-
118L8606A1	17	37619371	37619371	Splice_Site	SNP	G	A	A	CDK12	c.1046+1G>A	-
118L8606A1		-	-	Frame_Shift_Del	INS	-	-	-	RAD51C	c.896del	p.P299Lfs*3
118L8606A1	19	33792773	33792773	Missense_Mutation	SNP	G	C	C	CEBPA	c.548C>G	p.P183R
118L8606A1	2	25467120	25467120	Missense_Mutation	SNP	C	A	A	DNMT3A	c.1755G>T	p.M585I
118L8606A1	2	142004825	142004825	Missense_Mutation	SNP	G	C	C	LRP1B	c.562C>G	p.P188A
118L8606A1	2	191929601	191929601	Missense_Mutation	SNP	C	A	A	STAT4	c.714G>T	p.W238C
118L8606A1	20	39751925	39751925	Missense_Mutation	SNP	C	G	G	TOP1	c.2286C>G	p.D762E
118L8606A1	20	52194888	52194888	Missense_Mutation	SNP	G	T	T	ZNF217	c.1468C>A	p.L490I
118L8606A1	3	47162860	47162860	Missense_Mutation	SNP	C	T	T	SETD2	c.3266G>A	p.R1089Q
118L8606A1	3	70014398	70014398	Nonsense_Mutation	SNP	A	C	C	MITF	c.1259A>C	p.*420S
118L8606A1	3	169988318	169988318	Missense_Mutation	SNP	C	G	G	PRKCI	c.560C>G	p.T187R
118L8606A1	4	55980368	55980368	Missense_Mutation	SNP	C	A	A	KDR	c.723G>T	p.K241N
118L8606A1	5	112164551	112164551	Splice_Site	SNP	A	C	C	APC	c.1627-2A>C	-
118L8606A1	6	43749782	43749782	Missense_Mutation	SNP	G	T	T	VEGFA	c.1175G>T	p.R392L
118L8606A1	7	55249180	55249180	Splice_Site	SNP	G	T	T	EGFR	c.2469+9G>T	-
118L8606A1	8	48711936	48711936	Nonsense_Mutation	SNP				PRKDC	c.10131C>A	p.Y3377*
118L8606A1	9	139405215	139405215	Missense_Mutation	SNP	G	A	A	NOTCH1	c.2630C>T	p.P877L
118L8638M2		-	-	Splice_Site	SNP	-	-	-	FUBP1	.120+1_120+5de	-
118L8638M2	11	118362545	118362545	Nonsense_Mutation	SNP	C	T	T	KMT2A	c.4906C>T	p.R1636*
118L8638M2		-	-	Frame_Shift_Ins	INS	-	-	-	GATA6	C.581_582dup	P.G195Wfs*100
118L8638M2	3	178947852	178947852	Missense_Mutation	SNP	C	A	A	PIK3CA	c.2727C>A	p.F909L
118L8638M2	9	135786956	135786956	Splice_Site	SNP	C	T	T	TSC1	c.914-1G>A	-
118L8638M2	9	139399486	139399486	Missense_Mutation	SNP	C	T	T	NOTCH1	c.4657G>A	p.E1553K
118L8642M1		-	-	Frame_Shift_Ins	INS	-	-	-	ARID1A	c.3154dup	p.Y1052Lfs*53
118L8642M1		-	-	In_Frame_Del	INS	-	-	-	TBX3	C.1821del	P.Y607*
118L8642M1		-	-	Frame_Shift_Del	INS	-	-	-	IGF1R	c.1314_1323del	p.N438Kfs*25
118L8642M1		-	-	Frame_Shift_Del	INS	-	-	-	TP53	c.340_343del	p.L114Ifs*8
118L8642M1	2	11355098	11355098	Missense_Mutation	SNP	G	C	C	ROCK2	c.1804C>G	p.L602V
118L8642M1	2	141081457	141081457	Splice_Site	SNP	C	A	A	LRP1B	c.12514+5G>T	-
118L8642M1		-	-	Frame_Shift_Del	INS	-	-	-	APC	c.7929_7932del	p.Y2645Kfs*14
118L8642M1	5	147209156	147209156	Splice_Site	SNP	C	G	G	SPINK1	c.87+6G>C	-
118L8642M1	6	35423622	35423622	Missense_Mutation	SNP	C	G	G	FANCE	c.347C>G	p.S116C
118L8642M1	7	42116457	42116457	Splice_Site	SNP	C	T	T	GLI3	c.368-1G>A	-
118L8875M1		-	-	Frame_Shift_Del	INS	-	-	-	ARID1A	c.6711del	p.L2238Cfs*29
118L8875M1	11	532682	532682	Missense_Mutation	SNP	T	G	G	HRAS	c.524A>C	p.D175A
118L8875M1		-	-	Frame_Shift_Del	INS	-	-	-	FOXP1	c.1668del	p.N558Tfs*41
118L8884A1	12	25398284	25398284	Missense_Mutation	SNP	C	T	T	KRAS	c.35G>A	p.G12D
118L8884A1	14	45653093	45653093	Missense_Mutation	SNP	G	T	T	FANCM	c.4503G>T	p.Q1501H
118L8884A1	17	7576852	7576852	Splice_Site	SNP	C	T	T	TP53	c.993+1G>A	-
118L8884A1	3	37070324	37070324	Nonsense_Mutation	SNP	C	T	T	MLH1	c.1459C>T	p.R487*
118L8884A1		-	-	Frame_Shift_Ins	INS	-	-	-	PBRM1	c.310dup	p.E104Gfs*4
118L8884A1	7	117235045	117235045	Missense_Mutation	SNP	G	A	A	CFTR	c.2552G>A	p.R851Q
118L8913A1	1	27087868	27087868	Splice_Site	SNP	G	A	A	ARID1A	c.2162-7G>A	-
118L8913A1	1	243809206	243809206	Missense_Mutation	SNP	G	T	T	AKT3	c.418C>A	p.H140N
118L8913A1	11	92087187	92087187	Missense_Mutation	SNP	A	G	G	FAT3	c.1909A>G	p.K637E
118L8913A1	12	25398284	25398284	Missense_Mutation	SNP	C	A	A	KRAS	c.35G>T	p.G12V
118L8913A1	13	102521110	102521110	Missense_Mutation	SNP	T	G	G	FGF14	c.388A>C	p.I130L
118L8913A1	18	48604750	48604750	Missense_Mutation	SNP	G	T	T	SMAD4	c.1572G>T	p.W524C
118L8913A1	19	45532237	45532237	Splice_Site	SNP				RELB	c.986-12G>A	-
118L8913A1	3	187449633	187449633	Missense_Mutation	SNP	A	G	G	BCL6	c.247T>C	p.F83L
118L8913A1		-	-	In_Frame_Ins	INS	-	-	-	ARID1B	c.369_392dup	.Q124_Q131dup
118L8913A1	7	116415025	116415025	Missense_Mutation	SNP	C	G	G	MET	c.3119C>G	p.S1040C
118L8913A1	X	70348534	70348534	Missense_Mutation	SNP	T	C	C	MED12	.3441T>C;3443G	p.R1148P
118L8983A1	1	27058093	27058093	Nonsense_Mutation	SNP	C	T	T	ARID1A	c.1801C>T	p.Q601*
118L8983A1	1	27100919	27100919	Nonsense_Mutation	SNP	C	T	T	ARID1A	c.4201C>T	p.Q1401*
118L8983A1	12	6687306	6687306	Splice_Site	SNP	G	C	C	CHD4	c.5120-3C>G	-
118L8983A1	12	112888210	112888210	Missense_Mutation	SNP	G	A	A	PTPN11	c.226G>A	p.E76K
118L8983A1	16	68855957	68855957	Missense_Mutation	SNP	A	T	T	CDH1	c.1765A>T	p.N589Y
118L8983A1	16	81990330	81990330	Missense_Mutation	SNP	C	A	A	PLCG2	c.3601C>A	p.R1201S
118L8983A1		-	-	Missense_Mutation	SNP	-	-	-	TP53	c.526_528del	p.C176del
118L8983A1	17	37868208	37868208	Missense_Mutation	SNP	C	T	T	ERBB2	c.929C>T	p.S310F
118L8983A1	18	48575132	48575132	Missense_Mutation	SNP	T	G	G	SMAD4	c.326T>G	p.L109R
118L8983A1	18	48603041	48603041	Nonsense_Mutation	SNP	C	T	T	SMAD4	c.1342C>T	p.Q448*
118L8983A1	21	44524456	44524456	Missense_Mutation	SNP	G	A	A	U2AF1	c.101C>T	p.S34F
118L8983A1	5	149459731	149459731	Missense_Mutation	SNP	C	G	G	CSF1R	c.476G>C	p.W159S
118L8983A1	7	13935508	13935508	Missense_Mutation	SNP	C	T	T	ETV1	c.1417G>A	p.E473K
118L8983A1		-	-	Frame_Shift_Del	INS	-	-	-	CD36	c.483del	p.I162Lfs*14
118L8983A1	7	152009018	152009018	Nonsense_Mutation	SNP	G	A	A	KMT2C	c.604C>T	p.Q202*
118L8989A1	14	68963831	68963831	Splice_Site	SNP	C	T	T	RAD51B	c.1037-10C>T	-
118L8989A1	17	7577121	7577121	Missense_Mutation	SNP	G	A	A	TP53	c.817C>T	p.R273C
118L8989A1	2	170131604	170131604	Nonsense_Mutation	SNP	G	T	T	LRP2	c.1917C>A	p.Y639*
118L8989A1	3	52441192	52441192	Missense_Mutation	SNP	T	C	C	BAP1	c.578A>G	p.H193R
118L8989A1	4	153245515	153245515	Missense_Mutation	SNP	A	G	G	FBXW7	c.1676T>C	p.L559P
118L9109A2	12	25398284	25398284	Missense_Mutation	SNP	C	T	T	KRAS	c.35G>A	p.G12D
118L9109A2	14	69077785	69077785	Missense_Mutation	SNP	C	A	A	RAD51B	c.1099C>A	p.P367T
118L9109A2		-	-	Frame_Shift_Del	INS	-	-	-	TP53	194_197delinsA	p.R65Nfs*58
118L9109A2		-	-	In_Frame_Del	INS	-	-	-	FOXL2	.672_696delins	.A227_A234del
118L9110M1	17	7578461	7578461	Missense_Mutation	SNP	C	A	A	TP53	c.469G>T	p.V157F
118L9110M1	17	15995258	15995258	Nonsense_Mutation	SNP	T	A	A	NCOR1	c.2935A>T	p.R979*
118L9110M1	7	18687547	18687547	Missense_Mutation	SNP	A	G	G	HDAC9	c.1166A>G	p.N389S
119A1499J1	1	158582758	158582758	Splice_Site	SNP	G	A	A	SPTA1	c.6990-7C>T	-
119A1499J1		-	-	Frame_Shift_Ins	INS	-	-	-	PTEN	C.1028dup	P.K344Efs*17

119A1499J1	12	49445723	49445723	Missense_Mutation	SNP	C	A	A	KMT2D	c.1743G>T	p.M581I
119A1499J1	7	128846109	128846109	Missense_Mutation	SNP	G	C	C	SMO	c.1039G>C	p.G347R
119A1568M1	12	25398284	25398284	Missense_Mutation	SNP	C	T	T	KRAS	c.35G>A	p.G12D
119A1568M1	17	7578268	7578268	Missense_Mutation	SNP	A	C	C	TP53	c.581T>G	p.L194R
119A1568M1		-	-	Frame_Shift_Ins	INS	-	-	-	NF1	c.2596_2599dup	p.M867Tfs*7
119A1568M1	2	25463181	25463181	Missense_Mutation	SNP	C	T	T	DNMT3A	c.2312G>A	p.R771Q
119A1568M1	2	170012811	170012811	Missense_Mutation	SNP	G	A	A	LRP2	c.12124C>T	p.R4042C
119A1568M1	20	36028607	36028607	Missense_Mutation	SNP	A	T	T	SRC	c.949A>T	p.M317L
119A1621M1		-	-	Frame_Shift_Del	INS	-	-	-	EPHA2	c.1825del	p.H609Ifs*10
119A1621M1		-	-	Frame_Shift_Ins	INS	-	-	-	ARID1A	c.2343dup	p.H782Tfs*35
119A1621M1	2	141709477	141709477	Missense_Mutation	SNP	C	T	T	LRP1B	c.2920G>A	p.V974I
119A1621M1	22	23596126	23596126	Missense_Mutation	SNP	C	T	T	BCR	c.1420C>T	p.R474W
119A1621M1	4	126370060	126370060	Missense_Mutation	SNP	A	G	G	FAT4	c.7889A>G	p.E2630G
119A1621M1		-	-	Frame_Shift_Ins	INS	-	-	-	KDM6A	c.1918_1919dup	p.Q641Lfs*51
119A1621M1	X	70614053	70614053	Missense_Mutation	SNP	C	T	T	TAF1	c.3424C>T	p.R1142W
119A1715M1	10	89717720	89717720	Missense_Mutation	SNP	G	A	A	PTEN	c.745G>A	p.V249M
119A1715M1		-	-	Frame_Shift_Del	INS	-	-	-	ATM	c.8283_8284del	p.Q2762Afs*6
119A1715M1	12	25398284	25398284	Missense_Mutation	SNP	C	T	T	KRAS	c.35G>A	p.G12D
119A1715M1	17	74732555	74732555	Splice_Site	SNP	G	A	A	SRSF2	c.363-9C>T	-
119A1715M1	19	1220721	1220721	Splice_Site	SNP	G	A	A	STK11	c.734+5G>A	-
119A1715M1	4	66361184	66361184	Missense_Mutation	SNP	C	T	T	EPHA5	c.988G>A	p.E330K
119A1715M1	4	187549788	187549788	Nonsense_Mutation	SNP	C	A	A	FAT1	c.4453G>T	p.E1485*
119A1715M1		-	-	Frame_Shift_Del	INS	-	-	-	RBM10	2467_2504delin	p.E823Rfs*12
119A1725M1	12	25398281	25398281	Missense_Mutation	SNP	C	T	T	KRAS	c.38G>A	p.G13D
119A1725M1	12	56487277	56487277	Missense_Mutation	SNP	C	T	T	ERBB3	c.1423C>T	p.R475W
119A1725M1	17	7577565	7577565	Missense_Mutation	SNP	T	C	C	TP53	c.716A>G	p.N239S
119A1725M1	2	170112747	170112747	Splice_Site	SNP	C	A	A	LRP2	c.2640-1G>T	-
119A1725M1		-	-	Frame_Shift_Del	INS	-	-	-	CDKN2A	C.52_83del	P.T18Afs*15
119A1933A1	1	11316237	11316237	Missense_Mutation	SNP	G	A	A	MTOR	c.517C>T	p.R173C
119A1933A1	1	27099988	27099988	Splice_Site	SNP	G	A	A	ARID1A	c.3866+1G>A	-
119A1933A1	10	89692905	89692905	Missense_Mutation	SNP	G	A	A	PTEN	c.389G>A	p.R130Q
119A1933A1	12	49438646	49438646	Missense_Mutation	SNP	C	T	T	KMT2D	c.4844G>A	p.R1615Q
119A1933A1	19	11097280	11097280	Splice_Site	SNP	C	A	A	SMARCA4	c.760+11C>A	-
119A1933A1	2	61121627	61121627	Missense_Mutation	SNP	C	G	G	REL	c.249C>G	p.D83E
119A1933A1	3	41266097	41266097	Missense_Mutation	SNP	G	T	T	CTNNB1	c.94G>T	p.D32Y
119A1933A1	3	89259511	89259511	Missense_Mutation	SNP	G	T	T	EPHA3	c.655G>T	p.D219Y
119A1933A1	3	178936091	178936091	Missense_Mutation	SNP	G	A	A	PIK3CA	c.1633G>A	p.E545K
119A1933A1	3	195594719	195594719	Missense_Mutation	SNP	G	A	A	TNK2	c.2405C>T	p.P802L
119A1933A1	4	66535454	66535454	Missense_Mutation	SNP	C	T	T	EPHA5	c.7G>A	p.G3S
119A1933A1		-	-	In_Frame_Ins	INS	-	-	-	ARID1B	C.363_380dup	.Q126_Q131dup
119A1933A1	9	139401157	139401157	Splice_Site	SNP	G	A	A	NOTCH1	c.3901+11C>T	-
119A1949A1	12	25398284	25398284	Missense_Mutation	SNP	C	A	A	KRAS	c.35G>T	p.G12V
119A1949A1	18	48584708	48584708	Splice_Site	SNP	A	G	G	SMAD4	c.788-2A>G	-
119A1949A1	22	24129456	24129456	Splice_Site	SNP	C	T	T	SMARCB1	c.93+7C>T	-
119A1949A1	7	13946149	13946149	Missense_Mutation	SNP	T	C	C	ETV1	c.1016A>G	p.Q339R
119A9209D1	12	121434449	121434449	Missense_Mutation	SNP	A	G	G	HNF1A	c.1213A>G	p.M405V
119A9209D1	17	7578263	7578263	Nonsense_Mutation	SNP	G	A	A	TP53	c.586C>T	p.R196*
119A9336A1	17	7577105	7577105	Missense_Mutation	SNP	G	C	C	TP53	c.833C>G	p.P278R
119A9336A1	2	121746593	121746593	Missense_Mutation	SNP	G	A	A	GLI2	c.3103G>A	p.V1035M
119A9336A1	21	44524456	44524456	Missense_Mutation	SNP	G	A	A	U2AF1	c.101C>T	p.S34F
119A9336A1	3	130871239	130871239	Missense_Mutation	SNP	G	A	A	NEK11	c.655G>A	p.A219T
119A9336A1	6	43746274	43746274	Splice_Site	SNP	G	T	T	VEGFA	c.932+1G>T	-
119A9340A1	1	206665040	206665040	Missense_Mutation	SNP	A	G	G	IKBKE	c.1793A>G	p.Q598R
119A9340A1		-	-	Frame_Shift_Del	INS	-	-	-	BAP1	c.2034del	p.F678Lfs*14
119A9694A1	11	22646462	22646462	Missense_Mutation	SNP	A	G	G	FANCF	c.895T>C	p.S299P
119A9694A1	12	4488639	4488639	Missense_Mutation	SNP	C	G	G	FGF23	c.110G>C	p.G37A
119A9694A1		-	-	Frame_Shift_Ins	INS	-	-	-	DICER1	C.536_539dup	P.I181Cfs*15
119A9694A1	17	7578524	7578524	Nonsense_Mutation	SNP	G	A	A	TP53	c.406C>T	p.Q136*
119A9694A1	2	121742123	121742123	Missense_Mutation	SNP	C	T	T	GLI2	c.1760C>T	p.T587M
119A9694A1		-	-	Frame_Shift_Ins	INS	-	-	-	IRF2	C.102dup	P.Q35Sfs*9
119A9698A1	1	16462185	16462185	Nonsense_Mutation	SNP	G	A	A	EPHA2	c.1393C>T	p.R465*
119A9698A1		-	-	In_Frame_Del	INS	-	-	-	ARID1A	c.2667del	p.M890*
119A9698A1	1	27105581	27105581	Nonsense_Mutation	SNP	T	G	G	ARID1A	c.5192T>G	p.L1731*
119A9698A1	1	27105679	27105679	Nonsense_Mutation	SNP	G	T	T	ARID1A	c.5290G>T	p.E1764*
119A9698A1	11	92531644	92531644	Missense_Mutation	SNP	A	T	T	FAT3	c.5465A>T	p.K1822M
119A9698A1	11	108196806	108196806	Nonsense_Mutation	SNP	C	T	T	ATM	c.6829C>T	p.Q2277*
119A9698A1	11	108218086	108218086	Missense_Mutation	SNP	G	A	A	ATM	c.8665G>A	p.D2889N
119A9698A1	12	51213429	51213429	Missense_Mutation	SNP	G	C	C	ATF1	c.683G>C	p.R228P
119A9698A1	13	28623777	28623777	Missense_Mutation	SNP	C	G	G	FLT3	c.877G>C	p.E293Q
119A9698A1	15	66774131	66774131	Missense_Mutation	SNP	G	A	A	MAP2K1	c.607G>A	p.E203K
119A9698A1	17	7577141	7577141	Missense_Mutation	SNP	C	T	T	TP53	c.797G>A	p.G266E
119A9698A1	17	56436149	56436149	Nonsense_Mutation	SNP	G	A	A	RNF43	c.988C>T	p.R330*
119A9698A1	18	48581229	48581229	Nonsense_Mutation	SNP	C	G	G	SMAD4	c.533C>G	p.S178*
119A9698A1	19	11132522	11132522	Missense_Mutation	SNP	C	T	T	SMARCA4	c.2738C>T	p.P913L
119A9698A1	20	57415513	57415513	Missense_Mutation	SNP	G	A	A	GNAS	c.352G>A	p.E118K
119A9698A1	21	42870101	42870101	Missense_Mutation	SNP	G	T	T	TMPRSS2	c.71C>A	p.S24Y
119A9698A1	3	178936082	178936082	Missense_Mutation	SNP	G	A	A	PIK3CA	c.1624G>A	p.E542K
119A9698A1	4	55564449	55564449	Splice_Site	SNP	G	C	C	KIT	c.338-1G>C	-
119A9698A1	7	116412040	116412040	Missense_Mutation	SNP	G	A	A	MET	c.3025G>A	p.E1009K
119A9698A1	7	140453155	140453155	Missense_Mutation	SNP	C	T	T	BRAF	c.1780G>A	p.D594N
119A9698A1	8	69000039	69000039	Missense_Mutation	SNP	G	A	A	PREX2	c.2108G>A	p.G703E
119A9698A1	8	139164406	139164406	Missense_Mutation	SNP	A	G	G	FAM135B	c.2312T>C	p.V771A
119A9698A1		-	-	Frame_Shift_Del	INS	-	-	-	CDKN2A	c.324_346del	p.A109Mfs*16
119A9698A1	X	47044700	47044700	Splice_Site	SNP	G	A	A	RBM10	c.2101-1G>A	-
119A9698A1	X	66765547	66765547	Nonsense_Mutation	SNP	G	T	T	AR	c.559G>T	p.E187*
119A9702A1	12	25398284	25398284	Missense_Mutation	SNP	C	T	T	KRAS	c.35G>A	p.G12D
119A9702A1		-	-	Frame_Shift_Ins	INS	-	-	-	STK11	c.516dup	p.V173Cfs*93
119A9702A1	19	11107018	11107018	Nonsense_Mutation	SNP	C	T	T	SMARCA4	c.1723C>T	p.Q575*
119A9702A1	7	18688280	18688280	Missense_Mutation	SNP	T	C	C	HDAC9	c.1432T>C	p.Y478H
119A9806A1		-	-	Splice_Site	SNP	-	-	-	HTATIP2	c.82_101+25del	-
119A9806A1		-	-	Frame_Shift_Ins	INS	-	-	-	ARID2	C.341dup	P.P115Tfs*39
119A9806A1	17	38560458	38560458	Missense_Mutation	SNP	C	A	A	TOP2A	c.2229G>T	p.K743N
119A9806A1		-	-	Frame_Shift_Ins	INS	-	-	-	PBRM1	C.757_758dup	P.D253Efs*31
119A9806A1		-	-	Splice_Site	SNP	-	-	-	CD36	.701+5_701+8d	-
119A9806A1		-	-	Frame_Shift_Ins	INS	-	-	-	TGFBR1	C.1284_1285ins	P.Y429Lfs*2
119A9806A1		-	-	Frame_Shift_Del	INS	-	-	-	TFE3	C.669_672del	P.S224Pfs*118
119A9889A1	10	123310911	123310911	Missense_Mutation	SNP	T	A	A	FGFR2	c.517A>T	p.N173Y
119A9889A1	17	5076250	5076250	Missense_Mutation	SNP	G	A	A	USP6	c.4198G>A	p.E1400K

119A9889A1	17	7578442	7578442	Missense_Mutation	SNP	T	G	G	TP53	c.488A>C	p.Y163S
119A9889A1	3	130992328	130992328	Missense_Mutation	SNP	A	G	G	NEK11	c.1628A>G	p.K543R
119A9889A1	7	117232434	117232434	Nonsense_Mutation	SNP	T	A	A	CFTR	c.2213T>A	p.L738*
119A9889A1	8	93026977	93026977	Missense_Mutation	SNP	C	T	T	RUNX1T1	c.187G>A	p.G63R
119A9889A1	X	123220554	123220554	Missense_Mutation	SNP	C	G	G	STAG2	c.3211C>G	p.R1071G
216L0152A1		-	-	Frame_Shift_Ins	INS	-	-	-	EPHA2	.1141_1151dup	p.Y385Pfs*12
216L0152A1		-	-	Frame_Shift_Del	INS	-	-	-	TGFBR1	c.528_532del	p.L177Rfs*5
216L0152A1		-	-	Frame_Shift_Ins	INS	-	-	-	CDKN1B	c.356dup	p.G120Wfs*5
216L0152A1		-	-	Frame_Shift_Ins	INS	-	-	-	KEAP1	c.1637dup	p.V547Rfs*27
216L0152A1		-	-	Frame_Shift_Ins	INS	-	-	-	ARID1A	.5888_5889dup	p.E1964Mfs*52
216L0152A1	11	118344506	118344506	Missense_Mutation	SNP	C	T	T	KMT2A	c.2632C>T	p.R878W
216L0276A1	17	7578554	7578554	Missense_Mutation	SNP	A	T	T	TP53	c.376T>A	p.Y126N
216L0276A1	9	98239918	98239918	Missense_Mutation	SNP	C	T	T	PTCH1	c.1414G>A	p.A472T
216L0276A1	6	93956606	93956606	Missense_Mutation	SNP	C	T	T	EPHA7	c.2630G>A	p.R877H
216L0276A1	4	126372666	126372666	Missense_Mutation	SNP	A	C	C	FAT4	c.10495A>C	p.T3499P
217A0361A1	2	29474097	29474097	Missense_Mutation	SNP	G	A	A	ALK	c.2078C>T	p.P693L
217A0361A1	2	209113113	209113113	Missense_Mutation	SNP	G	C	C	IDH1	c.394C>G	p.R132G
217A0361A1		-	-	Frame_Shift_Ins	INS	-	-	-	EPHA2	c.1723dupT	p.Y575Lfs*21
217A0361A1		-	-	In_Frame_Del	INS	-	-	-	PBRM1	c.2994delC	p.Y998*
217B0420A1	21	44514790	44514790	Missense_Mutation	SNP	C	T	T	U2AF1	c.457G>A	p.A153T
217B0420A1	12	25398284	25398284	Missense_Mutation	SNP	C	T	T	KRAS	c.35G>A	p.G12D
217B0420A1	6	117708169	117708169	Splice_Site	SNP	A	G	G	ROS1	c.2015-7T>C	-
217B0420A1	17	7577539	7577539	Missense_Mutation	SNP	G	A	A	TP53	c.742C>T	p.R248W
217B0420A1	11	69514229	69514229	Missense_Mutation	SNP	C	G	G	FGF19	c.452G>C	p.R151P
217B0420A1	12	57574940	57574940	Missense_Mutation	SNP	A	G	G	LRP1	c.5528A>G	p.D1843G
217B0528A1	16	2137891	2137891	Missense_Mutation	SNP	G	C	C	TSC2	c.5017G>C	p.V1673L
217B0528A1		-	-	Frame_Shift_Ins	INS	-	-	-	STK11	C.361_362insTT	P.E121Vfs*9
217B0528A1	2	209108226	209108226	Missense_Mutation	SNP	T	C	C	IDH1	c.623A>G	p.Y208C
217B0528A1	7	151877980	151877980	Missense_Mutation	SNP	A	G	G	KMT2C	c.6965T>C	p.V2322A
217B0528A1	17	15952304	15952304	Splice_Site	SNP	T	C	C	NCOR1	c.6393-2A>G	-
217B0528A1	13	25052393	25052393	Missense_Mutation	SNP	G	T	T	PARP4	c.1470C>A	p.H490Q
217C0588A1	1	162746131	162746131	Missense_Mutation	SNP	C	T	T	DDR2	c.2254C>T	p.R752C
217C0588A1	7	140453136	140453136	Missense_Mutation	SNP	A	T	T	BRAF	c.1799T>A	p.V600E
217C0588A1	2	209113112	209113112	Missense_Mutation	SNP	C	A	A	IDH1	c.395G>T	p.R132L
217C0745A1	19	52715982	52715982	Missense_Mutation	SNP	C	T	T	PPP2R1A	c.547C>T	p.R183W
217C0745A1	3	178916930	178916930	Missense_Mutation	SNP	G	T	T	PIK3CA	c.317G>T	p.G106V
217D0937A1		-	-	Frame_Shift_Ins	INS	-	-	-	ARID1A	C.2624dup	P.M875Ifs*61
217D0937A1	20	57430394	57430394	Splice_Site	SNP	G	A	A	GNAS	c.2068+6G>A	-
217E1258A1	8	38272358	38272358	Missense_Mutation	SNP	A	G	G	FGFR1	c.1916T>C	p.I639T
217E1258A1	19	17955175	17955175	Missense_Mutation	SNP	G	A	A	JAK3	c.52C>T	p.L18F
217E1258A1	17	7577534	7577534	Missense_Mutation	SNP	C	A	A	TP53	c.747G>T	p.R249S
217E1258A1	1	204415111	204415111	Missense_Mutation	SNP	T	A	A	PIK3C2B	c.2651A>T	p.Q884L
217E1258A1	13	48881516	48881516	Nonsense_Mutation	SNP	A	T	T	RB1	c.238A>T	p.K80*
217E1258A1	20	31022879	31022879	Missense_Mutation	SNP	A	T	T	ASXL1	c.2364A>T	p.E788D
217E1258A1	X	76937219	76937219	Nonsense_Mutation	SNP	T	A	A	ATRX	c.3529A>T	p.K1177*
217E1258A1	2	25463271	25463271	Missense_Mutation	SNP	G	A	A	DNMT3A	c.2222C>T	p.A741V
217E1258A1	14	45644490	45644490	Missense_Mutation	SNP	A	G	G	FANCM	c.2533A>G	p.I845V
217E1258A1	6	394881	394881	Missense_Mutation	SNP	T	A	A	IRF4	c.277T>A	p.W93R
217E1258A1	7	151970849	151970849	Missense_Mutation	SNP	T	A	A	KMT2C	c.953A>T	p.Q318L
217E1258A1	12	49439944	49439944	Missense_Mutation	SNP	C	A	A	KMT2D	c.4597G>T	p.G1533C
217E1258A1	2	47709916	47709916	Splice_Site	SNP	A	T	T	MSH2	c.2635-2A>T	-
217E1258A1	X	110388152	110388152	Splice_Site	SNP	T	A	A	PAK3	c.339+2T>A	-
217E1258A1	19	14204552	14204552	Missense_Mutation	SNP	A	G	G	PRKACA	c.818T>C	p.L273P
217E1258A1	8	92999196	92999196	Missense_Mutation	SNP	C	G	G	RUNX1T1	c.885G>C	p.L295F
217E1258A1	22	38373903	38373903	Missense_Mutation	SNP	A	T	T	SOX10	c.668T>A	p.M223K
217E1258A1	1	16262741	16262741	Missense_Mutation	SNP	C	T	T	SPEN	c.10006C>T	p.R3336W
217E1258A1	10	104268931	104268931	Missense_Mutation	SNP	G	T	T	SUFU	c.188G>T	p.G63V
217E1387A1	17	37883956	37883956	Missense_Mutation	SNP	C	A	A	ERBB2	c.3427C>A	p.P1143T
217F1558A1	16	2138141	2138141	Splice_Site	SNP	G	C	C	TSC2	c.5160+1G>C	-
217F1558A1	15	90631839	90631839	Missense_Mutation	SNP	T	A	A	IDH2	c.514A>T	p.R172W
217F1558A1	17	7577514	7577514	Missense_Mutation	SNP	G	T	T	TP53	c.767C>A	p.T256K
217F1558A1		-	-	Frame_Shift_Ins	INS	-	-	-	ARID1A	c.5548dup	p.D1850Gfs*4
217F1558A1	7	151927097	151927097	Missense_Mutation	SNP	A	C	C	KMT2C	c.2887T>G	p.C963G
217F1599A1	12	25398284	25398284	Missense_Mutation	SNP	C	T	T	KRAS	c.35G>A	p.G12D
217F1599A1	18	48591888	48591888	Missense_Mutation	SNP	G	T	T	SMAD4	c.1051G>T	p.D351Y
217F1625A1		-	-	Frame_Shift_Ins	INS	-	-	-	ARID1A	c.5423dup	p.F1809Vfs*2
217F1625A1	3	52436390	52436390	Nonsense_Mutation	SNP	G	A	A	BAP1	c.2104C>T	p.Q702*
217F1625A1		-	-	In_Frame_Ins	INS	-	-	-	CFTR	c.3492dup	p.K1165*
217F1625A1	7	18687615	18687615	Missense_Mutation	SNP	G	T	T	HDAC9	c.1234G>T	p.V412L
217F1660A1	17	7577548	7577548	Missense_Mutation	SNP	C	T	T	TP53	c.733G>A	p.G245S
217F1660A1	5	149499084	149499084	Missense_Mutation	SNP	T	C	C	PDGFRB	c.2744A>G	p.N915S
217F1660A1	5	138266629	138266629	Splice_Site	SNP	G	A	A	CTNNA1	c.2298+5G>A	-
217F1870A1	X	63411227	63411227	Missense_Mutation	SNP	T	C	C	AMER1	c.1940A>G	p.Q647R
217F1870A1	12	46243818	46243818	Splice_Site	SNP	G	A	A	ARID2	c.1913-1G>A	-
217F1870A1	8	139155364	139155364	Splice_Site	SNP	G	C	C	FAM135B	c.3541-12C>G	-
217F1870A1	9	35078227	35078227	Missense_Mutation	SNP	G	A	A	FANCG	c.421C>T	p.R141C
217F1870A1		-	-	In_Frame_Del	INS	-	-	-	FAT1	c.3386_3391del	V1129_E1130del
217F1870A1	8	69002843	69002843	Missense_Mutation	SNP	C	A	A	PREX2	c.2143C>A	p.P715T
217F1870A1	X	47045667	47045667	Nonsense_Mutation	SNP	C	T	T	RBM10	c.2548C>T	p.Q850*
217F1870A1	11	65429463	65429463	Missense_Mutation	SNP	C	A	A	RELA	c.131G>T	p.G44V
217G1915A1	11	76169271	76169271	Missense_Mutation	SNP	G	T	T	EMSY	c.290G>T	p.R97L
217G2113A1	7	140453136	140453136	Missense_Mutation	SNP	A	T	T	BRAF	c.1799T>A	p.V600E
217G2113A1	17	7577497	7577497	Splice_Site	SNP	A	T	T	TP53	c.782+2T>A	-
217G2113A1		-	-	Frame_Shift_Del	INS	-	-	-	SMAD4	c.1607del	p.L536Qfs*16
217H2225A1		-	-	Frame_Shift_Ins	INS	-	-	-	ATM	1_7922insAGAG	p.K2643Sfs*6
217H2225A1	4	55603449	55603449	Splice_Site	SNP	G	A	A	KIT	c.2802+3G>A	-
217H2225A1	8	32585527	32585527	Missense_Mutation	SNP	C	A	A	NRG1	c.1106C>A	p.T369N
217H2225A1		-	-	Frame_Shift_Ins	INS	-	-	-	ARID1A	c.3732dup	p.F1245Lfs*14
217H2254A1	12	25398285	25398285	Missense_Mutation	SNP	C	T	T	KRAS	c.34G>A	p.G12S
217H2254A1	17	7578217	7578217	Missense_Mutation	SNP	G	A	A	TP53	c.632C>T	p.T211I
217H2400A1		-	-	Frame_Shift_Del	INS	-	-	-	ARID1A	C.3524del	P.P1175Hfs*5
217H2400A1		-	-	Frame_Shift_Del	INS	-	-	-	GATA3	C.708del	P.S237Afs*29
217H2400A1		-	-	Frame_Shift_Del	INS	-	-	-	KMT2D	C.7061del	P.P2354Lfs*30
217H2400A1		-	-	Frame_Shift_Del	INS	-	-	-	KMT2D	C.1940del	P.P647Hfs*283
217H2400A1	13	25029208	25029208	Missense_Mutation	SNP	G	A	A	PARP4	c.2705C>T	p.A902V
217H2400A1	13	25030478	25030478	Splice_Site	SNP	C	T	T	PARP4	c.2562+1G>A	-
217H2400A1	17	7578262	7578262	Missense_Mutation	SNP	C	T	T	TP53	c.587G>A	p.R196Q
217H2400A1	17	7578406	7578406	Missense_Mutation	SNP	C	T	T	TP53	c.524G>A	p.R175H

217H2400A1		-	-	Frame_Shift_Del	INS	-	-	-	NCOR1	C.1584del	P.E529Kfs*26
217H2400A1	18	18533624	18533624	Missense_Mutation	SNP	G	A	A	ROCK1	c.3976C>T	p.R1326C
217H2400A1		-	-	Frame_Shift_Del	INS	-	-	-	TGFBR2	C.1226_1229de	P.S409Wfs*21
217H2400A1	3	37053589	37053589	Nonsense_Mutation	SNP	C	T	T	MLH1	c.676C>T	p.R226*
217H2400A1		-	-	Frame_Shift_Del	INS	-	-	-	FAT1	C.7381del	P.Y2461Tfs*20
217H2400A1	6	35423676	35423676	Missense_Mutation	SNP	G	A	A	FANCE	c.401G>A	p.R134H
217H2400A1		-	-	Frame_Shift_Del	INS	-	-	-	ARID1B	C.3851del	P.G1284Efs*2
217H2400A1		-	-	Frame_Shift_Ins	INS	-	-	-	ARID1B	C.5870dup	P.V1958Rfs*5
217H2400A1		-	-	Frame_Shift_Del	INS	-	-	-	ARID1B	C.6216del	P.N2073Tfs*26
217H2400A1		-	-	Frame_Shift_Del	INS	-	-	-	QKI	C.401del	P.K134Rfs*14
217H2400A1	7	106508082	106508082	Missense_Mutation	SNP	C	T	T	PIK3CG	c.76C>T	p.R26C
217H2400A1		-	-	Frame_Shift_Del	INS	-	-	-	KMT2C	C.8390del	P.K2797Rfs*26
217H2400A1		-	-	Frame_Shift_Del	INS	-	-	-	AMER1	C.780del	P.K260Nfs*22
217H2423A1	19	1221336	1221336	Nonsense_Mutation	SNP	A	T	T	STK11	c.859A>T	p.K287*
217H2423A1	20	30659499	30659499	Missense_Mutation	SNP	G	A	A	HCK	c.34G>A	p.G12R
217H2487A1	12	56481622	56481622	Missense_Mutation	SNP	T	G	G	ERBB3	c.657T>G	p.F219L
217H2487A1	15	91303853	91303853	Missense_Mutation	SNP	A	G	G	BLM	c.1250A>G	p.N417S
217H2487A1	17	29528086	29528086	Nonsense_Mutation	SNP	C	G	G	NF1	c.1094C>G	p.S365*
217H2487A1	17	29553492	29553492	Nonsense_Mutation	SNP	C	T	T	NF1	c.2041C>T	p.R681*
217H2487A1	17	37868208	37868208	Missense_Mutation	SNP	C	T	T	ERBB2	c.929C>T	p.S310F
217H2487A1		-	-	Frame_Shift_Del	INS	-	-	-	SMAD4	c.767del	p.Q256Rfs*80
217H2487A1	X	44945115	44945115	Nonsense_Mutation	SNP	C	T	T	KDM6A	c.3595C>T	p.Q1199*
217I2570A1	1	158623223	158623223	Splice_Site	SNP	G	C	C	SPTA1	c.3037-8C>G	-
217I2570A1	16	396812	396812	Missense_Mutation	SNP	G	A	A	AXIN1	c.214C>T	p.P72S
217I2570A1		-	-	Frame_Shift_Del	INS	-	-	-	TSC2	c.729_730del	p.C244Sfs*93
217I2570A1	17	7577534	7577534	Missense_Mutation	SNP	C	A	A	TP53	c.747G>T	p.R249S
217I2570A1	2	121746789	121746789	Missense_Mutation	SNP	C	T	T	GLI2	c.3299C>T	p.P1100L
217I2570A1	4	55972925	55972925	Missense_Mutation	SNP	C	G	G	KDR	c.1465G>C	p.E489Q
217I2570A1		-	-	Frame_Shift_Del	INS	-	-	-	FAT4	c.4187_4190del	p.G1396Dfs*14
217I2570A1	5	180048866	180048866	Missense_Mutation	SNP	C	T	T	FLT4	c.1696G>A	p.E566K
217I2570A1		-	-	In_Frame_Del	INS	-	-	-	KMT2C	c.2448del	p.Y816*
217I2570A1	8	141716207	141716207	Splice_Site	SNP	G	A	A	PTK2	c.2295+11C>T	-
217I2570A1	X	70613164	70613164	Missense_Mutation	SNP	C	G	G	TAF1	c.3125C>G	p.S1042C
217I2920A1	1	27023663	27023663	Missense_Mutation	SNP	G	T	T	ARID1A	c.769G>T	p.A257S
217I2920A1	17	7577547	7577547	Missense_Mutation	SNP	C	T	T	TP53	c.734G>A	p.G245D
217I2920A1	2	212426722	212426722	Missense_Mutation	SNP	A	C	C	ERBB4	c.2393T>G	p.L798R
217I2920A1	7	151945112	151945112	Missense_Mutation	SNP	C	A	A	KMT2C	c.2407G>T	p.A803S
217I2921A1	1	158650531	158650531	Splice_Site	SNP	G	A	A	SPTA1	c.532-12C>T	-
217I2921A1	12	56482369	56482369	Missense_Mutation	SNP	C	G	G	ERBB3	c.917C>G	p.P306R
217I2921A1	17	7577538	7577538	Missense_Mutation	SNP	C	T	T	TP53	c.743G>A	p.R248Q
217I2921A1	17	37863344	37863344	Missense_Mutation	SNP	A	C	C	ERBB2	c.175A>C	p.N59H
217I2921A1		-	-	Frame_Shift_Del	INS	-	-	-	DNMT3A	708_1719delins	p.G570Qfs*78
217I2921A1	2	58390085	58390085	Splice_Site	SNP	G	A	A	FANCL	c.822-3C>T	-
217I2921A1	2	170063141	170063141	Missense_Mutation	SNP	C	A	A	LRP2	c.7089G>T	p.L2363F
217I2921A1	4	153247174	153247174	Missense_Mutation	SNP	C	G	G	FBXW7	c.1628G>C	p.R543T
217I2921A1	7	77648765	77648765	Missense_Mutation	SNP	G	A	A	MAGI2	c.4235C>T	p.P1412L
217J3201A1	1	27101242	27101242	Nonsense_Mutation	SNP	T	A	A	ARID1A	c.4524T>A	p.Y1508*
217J3201A1	2	209113112	209113112	Missense_Mutation	SNP	C	A	A	IDH1	c.395G>T	p.R132L
217J3201A1	3	12626437	12626437	Missense_Mutation	SNP	C	T	T	RAF1	c.1712G>A	p.S571N
217J3201A1		-	-	Frame_Shift_Ins	INS	-	-	-	PBRM1	c.773dup	p.N258Kfs*6
217J3201A1	4	110897341	110897341	Missense_Mutation	SNP	T	A	A	EGF	c.2003T>A	p.M668K
217K3355A1	12	25398284	25398284	Missense_Mutation	SNP	C	T	T	KRAS	c.35G>A	p.G12D
217K3355A1	17	7578271	7578271	Missense_Mutation	SNP	T	C	C	TP53	c.578A>G	p.H193R
217K3355A1	18	48604788	48604788	Missense_Mutation	SNP	A	G	G	SMAD4	c.1610A>G	p.D537G
217K3355A1	2	198266611	198266611	Missense_Mutation	SNP	C	T	T	SF3B1	c.2225G>A	p.G742D
217K3355A1		-	-	In_Frame_Del	INS	-	-	-	CDKN2A	c.349_353del	p.L117*
217K3692A1	1	158651311	158651311	Splice_Site	SNP	C	T	T	SPTA1	c.531+6G>A	-
217K3692A1	1	162724518	162724518	Missense_Mutation	SNP	T	C	C	DDR2	c.290T>C	p.I97T
217K3692A1	15	88727493	88727493	Missense_Mutation	SNP	C	T	T	NTRK3	c.286G>A	p.A96T
217K3692A1		-	-	In_Frame_Del	INS	-	-	-	TP53	c.852_884del	p.E285_P295del
217K3692A1	3	134880980	134880980	Missense_Mutation	SNP	G	A	A	EPHB1	c.1543G>A	p.G515S
217K3692A1	3	185181346	185181346	Nonsense_Mutation	SNP	G	A	A	MAP3K13	c.1287G>A	p.W429*
217K3692A1	7	73535232	73535232	Missense_Mutation	SNP	G	A	A	LIMK1	c.1634G>A	p.R545Q
217K3692A1	X	66937323	66937323	Missense_Mutation	SNP	T	C	C	AR	c.2177T>C	p.F726S
217K3729A1		-	-	Frame_Shift_Del	INS	-	-	-	ARID1A	5391_5392delin	p.S1798Qfs*8
217K3729A1	12	25398284	25398284	Missense_Mutation	SNP	C	A	A	KRAS	c.35G>T	p.G12V
217K3729A1	17	38560458	38560458	Missense_Mutation	SNP	C	A	A	TOP2A	c.2229G>T	p.K743N
217K3729A1		-	-	Frame_Shift_Ins	INS	-	-	-	RNF43	c.1371_1372dup	p.L458Pfs*45
217K3729A1		-	-	Splice_Site	SNP	-	-	-	LRP1B	2767_2770+10d	-
217K3729A1		-	-	Frame_Shift_Ins	INS	-	-	-	MACC1	c.1946_1949dup	p.S651Lfs*14
217K3729A1	7	42004656	42004656	Missense_Mutation	SNP	G	A	A	GLI3	c.4015C>T	p.R1339C
217K3730A1	12	49427693	49427693	Nonsense_Mutation	SNP	G	A	A	KMT2D	c.10795C>T	p.Q3599*
217K3730A1	4	20598069	20598069	Missense_Mutation	SNP	G	T	T	SLIT2	c.3352G>T	p.V1118F
217K3730A1	9	135777994	135777994	Nonsense_Mutation	SNP	G	A	A	TSC1	c.2389C>T	p.Q797*
217L3903A1	1	115256529	115256529	Missense_Mutation	SNP	T	A	A	NRAS	c.182A>T	p.Q61L
217L3903A1		-	-	Frame_Shift_Del	INS	-	-	-	TP53	c.64del	p.L22Yfs*22
217L3903A1	3	178952030	178952030	Missense_Mutation	SNP	G	C	C	PIK3CA	c.3085G>C	p.D1029H
217L3903A1	X	76940011	76940011	Missense_Mutation	SNP	C	T	T	ATRX	c.737G>A	p.R246H
218A4699A1	1	158605803	158605803	Missense_Mutation	SNP	T	C	C	SPTA1	c.5332A>G	p.K1778E
218A4699A1	1	179112155	179112155	Missense_Mutation	SNP	G	A	A	ABL2	c.25C>T	p.P9S
218A4699A1	12	25398284	25398284	Missense_Mutation	SNP	C	A	A	KRAS	c.35G>T	p.G12V
218A4699A1	14	45645043	45645043	Missense_Mutation	SNP	T	C	C	FANCM	c.3086T>C	p.L1029S
218A4699A1	17	7577570	7577570	Missense_Mutation	SNP	C	T	T	TP53	c.711G>A	p.M237I
218A4699A1	5	112043565	112043565	Missense_Mutation	SNP	G	A	A	APC	c.151G>A	p.A51T
218A4699A1	8	37696517	37696517	Missense_Mutation	SNP	G	A	A	ADGRA2	c.2303G>A	p.G768E
218A4699A1	X	47028867	47028867	Nonsense_Mutation	SNP	T	A	A	RBM10	c.171T>A	p.Y57*
218B4967A1	1	46738363	46738363	Missense_Mutation	SNP	G	C	C	RAD54L	c.1264G>C	p.E422Q
218B4967A1	10	89685313	89685313	Missense_Mutation	SNP	C	G	G	PTEN	c.208C>G	p.L70V
218B4967A1	11	92086395	92086395	Missense_Mutation	SNP	C	G	G	FAT3	c.1117C>G	p.P373A
218B4967A1	17	7578206	7578206	Missense_Mutation	SNP	T	C	C	TP53	c.643A>G	p.S215G
218B4967A1	3	49412973	49412973	Missense_Mutation	SNP	C	T	T	RHOA	c.50G>A	p.G17E
218B4967A1	7	18975551	18975551	Missense_Mutation	SNP	C	T	T	HDAC9	c.2914C>T	p.L972F
218B4967A1	8	69017409	69017409	Missense_Mutation	SNP	C	T	T	PREX2	c.2752C>T	p.R918W
218C5441A1	17	7577141	7577141	Missense_Mutation	SNP	C	T	T	TP53	c.797G>A	p.G266E
218C5441A1	17	37881440	37881440	Missense_Mutation	SNP	C	T	T	ERBB2	c.2632C>T	p.H878Y
218C5441A1	8	93003911	93003911	Missense_Mutation	SNP	C	T	T	RUNX1T1	c.836G>A	p.R279Q
218C5575A1	11	118359366	118359366	Missense_Mutation	SNP	A	T	T	KMT2A	c.4370A>T	p.K1457M
218C5575A1	14	20813099	20813099	Missense_Mutation	SNP	G	C	C	PARP2	c.55G>C	p.E19Q

218C5575A1	16	68867257	68867257	Missense_Mutation	SNP	A	G	G	CDH1	c.2504A>G	p.Y835C
218C5575A1	16	89836259	89836259	Missense_Mutation	SNP	C	A	A	FANCA	c.2490G>T	p.L830F
218C5575A1	17	7577559	7577559	Missense_Mutation	SNP	G	C	C	TP53	c.722C>G	p.S241C
218C5575A1	2	141460104	141460104	Missense_Mutation	SNP	T	A	A	LRP1B	c.6042A>T	p.E2014D
218C5575A1	3	52663008	52663008	Nonsense_Mutation	SNP	C	A	A	PBRM1	c.1345G>T	p.E449*
218C5588A2	1	158592947	158592947	Missense_Mutation	SNP	T	A	A	SPTA1	c.5946A>T	p.Q1982H
218C5588A2	12	57538862	57538862	Missense_Mutation	SNP	C	T	T	LRP1	c.556C>T	p.R186C
218C5588A2	17	7577507	7577507	Missense_Mutation	SNP	T	G	G	TP53	c.774A>C	p.E258D
218C5588A2	2	198267484	198267484	Missense_Mutation	SNP	G	A	A	SF3B1	c.1873C>T	p.R625C
218C5780A1	17	7578406	7578406	Missense_Mutation	SNP	C	T	T	TP53	c.524G>A	p.R175H
218C5780A1	17	29533379	29533379	Missense_Mutation	SNP	G	A	A	NF1	c.1382G>A	p.R461Q
218C5780A1	6	32166817	32166817	Missense_Mutation	SNP	C	T	T	NOTCH4	c.4421G>A	p.R1474Q
218D5915A1	17	7578431	7578431	Nonsense_Mutation	SNP	G	A	A	TP53	c.499C>T	p.Q167*
218D5915A1		-	-	Frame_Shift_Ins	INS	-	-	-	COL1A1	c.2069_2109dup	p.G704Pfs*76
218D5915A1	5	38950441	38950441	Missense_Mutation	SNP	T	C	C	RICTOR	c.3509A>G	p.E1170G
218D5915A1	6	93969182	93969182	Missense_Mutation	SNP	G	T	T	EPHA7	c.1814C>A	p.T605N
218D6413A1	1	158597523	158597523	Splice_Site	SNP	T	C	C	SPTA1	c.5566-10A>G	-
218D6413A1	10	70405010	70405010	Missense_Mutation	SNP	A	G	G	TET1	c.2524A>G	p.I842V
218D6413A1	12	1022603	1022603	Missense_Mutation	SNP	T	A	A	RAD52	c.1211A>T	p.H404L
218D6413A1	12	25398285	25398285	Missense_Mutation	SNP	C	A	A	KRAS	c.34G>T	p.G12C
218D6413A1	13	28896915	28896915	Splice_Site	SNP	A	G	G	FLT1	c.2953+12T>C	-
218D6413A1	16	2100429	2100429	Missense_Mutation	SNP	A	G	G	TSC2	c.167A>G	p.N56S
218D6413A1	17	7579722	7579722	Splice_Site	SNP	C	G	G	TP53	c.75-1G>C	-
218D6413A1	17	48270360	48270360	Missense_Mutation	SNP	C	T	T	COL1A1	c.1816G>A	p.A606T
218D6413A1	2	212812269	212812269	Missense_Mutation	SNP	G	A	A	ERBB4	c.307C>T	p.R103C
218D6413A1	7	20199671	20199671	Missense_Mutation	SNP	T	C	C	MACC1	c.313A>G	p.R105G
218D6413A1	7	41739882	41739882	Missense_Mutation	SNP	C	T	T	INHBA	c.91G>A	p.A31T
218D6413A1		-	-	Splice_Site	SNP	-	-	-	CFTR	1210-7_1210-6d	-
218D6413A1	8	139149456	139149456	Missense_Mutation	SNP	G	A	A	FAM135B	c.3949C>T	p.R1317C
218D6413A1		-	-	Frame_Shift_Del	INS	-	-	-	CDKN2A	c.332del	p.G111Afs*35
218D6471A1		-	-	Frame_Shift_Ins	INS	-	-	-	ARID1A	c.5548dup	p.D1850Gfs*4
218D6471A1		-	-	Frame_Shift_Del	INS	-	-	-	TGFBR2	c.1483del	p.R495Efs*23
218D6471A1	3	71064802	71064802	Nonsense_Mutation	SNP	A	T	T	FOXP1	c.872T>A	p.L291*
218D6471A1		-	-	Splice_Site	SNP	-	-	-	RICTOR	c.1152-5dup	-
218D6874A1	10	104356918	104356918	Missense_Mutation	SNP	G	A	A	SUFU	c.778G>A	p.E260K
218E7563A1	2	29443591	29443591	Missense_Mutation	SNP	C	T	T	ALK	c.3626G>A	p.R1209Q
218E7564A1	11	69625249	69625249	Missense_Mutation	SNP	C	T	T	FGF3	c.544G>A	p.V182M
218E7564A1		-	-	Frame_Shift_Ins	INS	-	-	-	ATM	c.2547_2592dup	p.S865Gfs*10
218E7564A1	12	25398284	25398284	Missense_Mutation	SNP	C	T	T	KRAS	c.35G>A	p.G12D
218E7564A1	19	1221340	1221340	Splice_Site	SNP	G	T	T	STK11	c.862+1G>T	-
218E7564A1		-	-	Frame_Shift_Del	INS	-	-	-	BARD1	c.631_632del	p.L211Sfs*2
218E7564A1	3	47144879	47144879	Missense_Mutation	SNP	C	T	T	SETD2	c.4874G>A	p.R1625H
218E7890A1	1	97547948	97547948	Missense_Mutation	SNP	C	T	T	DPYD	c.2845G>A	p.D949N
218E7890A1	11	92534419	92534419	Missense_Mutation	SNP	G	A	A	FAT3	c.8240G>A	p.R2747Q
218E7890A1	12	50481161	50481161	Nonsense_Mutation	SNP	C	T	T	SMARCD1	c.547C>T	p.R183*
218E7890A1	13	28597543	28597543	Missense_Mutation	SNP	G	C	C	FLT3	c.2362C>G	p.L788V
218F8298A1	12	25398284	25398284	Missense_Mutation	SNP	C	T	T	KRAS	c.35G>A	p.G12D
218F8400A1	4	55140693	55140693	Splice_Site	SNP	C	T	T	PDGFRA	c.1559-5C>T	-
218F8400A1	9	139412297	139412297	Nonsense_Mutation	SNP	C	A	A	NOTCH1	c.1348G>T	p.E450*
218F8651A1		-	-	In_Frame_Del	INS	-	-	-	TP53	C.754_759del	P.L252_T253del
218F8651A1	17	12028645	12028645	Missense_Mutation	SNP	G	A	A	MAP2K4	c.848G>A	p.G283E
218F8651A1	4	126239844	126239844	Nonsense_Mutation	SNP	C	T	T	FAT4	c.2278C>T	p.Q760*
218G8980A1	1	16261701	16261701	Missense_Mutation	SNP	C	A	A	SPEN	c.8966C>A	p.A2989D
218G8980A1	1	158612193	158612193	Splice_Site	SNP	A	C	C	SPTA1	c.4737+8T>G	-
218G8980A1	1	193117054	193117054	Missense_Mutation	SNP	C	T	T	CDC73	c.787C>T	p.R263C
218G8980A1	10	104592424	104592424	Missense_Mutation	SNP	A	C	C	CYP17A1	c.983T>G	p.L328R
218G8980A1	11	92565001	92565001	Missense_Mutation	SNP	A	C	C	FAT3	c.9695A>C	p.N3232T
218G8980A1	12	6690202	6690202	Splice_Site	SNP	A	G	G	CHD4	c.4909+8T>C	-
218G8980A1	12	25398284	25398284	Missense_Mutation	SNP	C	T	T	KRAS	c.35G>A	p.G12D
218G8980A1	12	57548458	57548458	Missense_Mutation	SNP	C	T	T	LRP1	c.1201C>T	p.R401C
218G8980A1	13	28609708	28609708	Missense_Mutation	SNP	T	C	C	FLT3	c.1521A>G	p.I507M
218G8980A1	13	110437616	110437616	Missense_Mutation	SNP	G	A	A	IRS2	c.785C>T	p.S262L
218G8980A1	15	75093390	75093390	Missense_Mutation	SNP	G	A	A	CSK	c.760G>A	p.V254M
218G8980A1	16	2105412	2105412	Missense_Mutation	SNP	T	A	A	TSC2	c.491T>A	p.V164D
218G8980A1	16	9916145	9916145	Missense_Mutation	SNP	T	C	C	GRIN2A	c.2144A>G	p.D715G
218G8980A1	17	41605981	41605981	Missense_Mutation	SNP	A	G	G	ETV4	c.1361T>C	p.V454A
218G8980A1		-	-	Frame_Shift_Ins	INS	-	-	-	RNF43	c.2094dup	p.I700Dfs*47
218G8980A1	17	63052458	63052458	Missense_Mutation	SNP	C	T	T	GNA13	c.254G>A	p.R85H
218G8980A1	17	78867626	78867626	Missense_Mutation	SNP	C	T	T	RPTOR	c.2362C>T	p.R788C
218G8980A1	19	14213683	14213683	Missense_Mutation	SNP	C	T	T	PRKACA	c.281G>A	p.R94H
218G8980A1	19	15298710	15298710	Nonsense_Mutation	SNP	C	A	A	NOTCH3	c.1588G>T	p.E530*
218G8980A1	2	25467502	25467502	Missense_Mutation	SNP	G	A	A	DNMT3A	c.1574C>T	p.A525V
218G8980A1	2	47630542	47630542	Splice_Site	SNP	G	T	T	MSH2	c.211+1G>T	-
218G8980A1		-	-	Frame_Shift_Del	INS	-	-	-	ACVR2A	c.1310del	p.K437Rfs*5
218G8980A1	2	198257804	198257804	Nonsense_Mutation	SNP	C	T	T	SF3B1	c.3648G>A	p.W1216*
218G8980A1	2	198267373	198267373	Missense_Mutation	SNP	G	C	C	SF3B1	c.1984C>G	p.H662D
218G8980A1	20	31020720	31020720	Missense_Mutation	SNP	G	T	T	ASXL1	c.1017G>T	p.Q339H
218G8980A1	4	126240490	126240490	Missense_Mutation	SNP	A	C	C	FAT4	c.2924A>C	p.Q975P
218G8980A1	4	126411235	126411235	Missense_Mutation	SNP	T	C	C	FAT4	c.13258T>C	p.Y4420H
218G8980A1	5	38962976	38962976	Splice_Site	SNP	A	G	G	RICTOR	c.1566+2T>C	-
218G8980A1	7	41729394	41729394	Missense_Mutation	SNP	G	A	A	INHBA	c.1135C>T	p.R379W
218G8980A1	8	37691318	37691318	Missense_Mutation	SNP	T	A	A	ADGRA2	c.1409T>A	p.I470N
218G8980A1	8	71041004	71041004	Missense_Mutation	SNP	G	A	A	NCOA2	c.3536C>T	p.T1179M
218G8980A1	9	5522590	5522590	Missense_Mutation	SNP	A	C	C	PDCD1LG2	c.44A>C	p.H15P
218G8980A1	9	27204927	27204927	Missense_Mutation	SNP	G	C	C	TEK	c.2228G>C	p.G743A
218G8980A1	X	76855026	76855026	Missense_Mutation	SNP	C	T	T	ATRX	c.5810G>A	p.G1937E
218G8980A1	X	100611203	100611203	Missense_Mutation	SNP	C	T	T	BTK	c.1403G>A	p.R468H
218G8980A1	X	123034349	123034349	Missense_Mutation	SNP	C	G	G	XIAP	c.1106C>G	p.T369S
218G8980A1	X	123205175	123205175	Splice_Site	SNP	T	C	C	STAG2	c.2533+2T>C	-
218G9808A1	10	89717661	89717661	Nonsense_Mutation	SNP	C	G	G	PTEN	c.686C>G	p.S229*
218G9808A1	2	111907671	111907671	Missense_Mutation	SNP	G	A	A	BCL2L11	c.265G>A	p.A89T
218G9808A1	2	198267491	198267491	Missense_Mutation	SNP	C	A	A	SF3B1	c.1866G>T	p.E622D
218G9808A1	3	52437267	52437267	Nonsense_Mutation	SNP	G	A	A	BAP1	c.1777C>T	p.Q593*
218G9808A1	3	142257358	142257358	Missense_Mutation	SNP	C	A	A	ATR	c.3691G>T	p.A1231S
218G9947A1	12	25380275	25380275	Missense_Mutation	SNP	T	G	G	KRAS	c.183A>C	p.Q61H
218G9952A1	12	25398284	25398284	Missense_Mutation	SNP	C	T	T	KRAS	c.35G>A	p.G12D
218G9952A1	17	29670025	29670025	Splice_Site	SNP	A	G	G	NF1	c.7063-2A>G	-
218G9952A1	21	44524456	44524456	Missense_Mutation	SNP	G	A	A	U2AF1	c.101C>T	p.S34F

218G9952A1	3	189604320	189604320	Missense_Mutation	SNP	C	T	T	TP63	c.1487C>T	p.P496L
218G9952A1		-	-	Frame_Shift_Del	INS	-	-	-	KMT2C	C.10545del	P.F3515Lfs*18
218G9952A1	9	21971028	21971028	Nonsense_Mutation	SNP	C	T	T	CDKN2A	c.330G>A	p.W110*
218H1383A1	1	43773507	43773507	Missense_Mutation	SNP	G	T	T	TIE1	c.954G>T	p.Q318H
218H1383A1	1	158617446	158617446	Missense_Mutation	SNP	A	G	G	SPTA1	c.3779T>C	p.L1260P
218H1383A1	11	69589800	69589800	Missense_Mutation	SNP	G	A	A	FGF4	c.53C>T	p.A18V
218H1383A1	11	108173611	108173611	Missense_Mutation	SNP	A	C	C	ATM	c.5351A>C	p.N1784T
218H1383A1	12	51189780	51189780	Missense_Mutation	SNP	G	A	A	ATF1	c.182G>A	p.R61H
218H1383A1	13	32972432	32972432	Missense_Mutation	SNP	A	G	G	BRCA2	c.9782A>G	p.D3261G
218H1383A1		-	-	Frame_Shift_Del	INS	-	-	-	B2M	c.43_44del	p.L15Ffs*41
218H1383A1	15	90631877	90631877	Missense_Mutation	SNP	C	T	T	IDH2	c.476G>A	p.R159H
218H1383A1	16	9858133	9858133	Missense_Mutation	SNP	C	T	T	GRIN2A	c.3268G>A	p.V1090M
218H1383A1	17	37687478	37687478	Missense_Mutation	SNP	G	C	C	CDK12	c.4355G>C	p.G1452A
218H1383A1	17	48271816	48271816	Splice_Site	SNP	G	T	T	COL1A1	c.1516-8C>A	-
218H1383A1	17	78811739	78811739	Missense_Mutation	SNP	C	T	T	RPTOR	c.1154C>T	p.A385V
218H1383A1	19	2226439	2226439	Missense_Mutation	SNP	A	G	G	DOT1L	c.3919A>G	p.S1307G
218H1383A1		-	-	Missense_Mutation	SNP	-	-	-	SMARCA4	c.3634_3636del	p.E1212del
218H1383A1	20	57415601	57415601	Missense_Mutation	SNP	C	T	T	GNAS	c.440C>T	p.P147L
218H1383A1	3	12641190	12641190	Missense_Mutation	SNP	C	T	T	RAF1	c.1108G>A	p.G370R
218H1383A1	3	37038192	37038192	Missense_Mutation	SNP	G	A	A	MLH1	c.199G>A	p.G67R
218H1383A1	3	70014295	70014295	Missense_Mutation	SNP	G	A	A	MITF	c.1156G>A	p.V386I
218H1383A1	3	128200008	128200008	Missense_Mutation	SNP	G	T	T	GATA2	c.1297C>A	p.L433M
218H1383A1		-	-	Frame_Shift_Del	INS	-	-	-	TET2	c.1243del	p.V415Ffs*12
218H1383A1	5	112116536	112116536	Missense_Mutation	SNP	G	T	T	APC	c.581G>T	p.R194M
218H1383A1	5	121776414	121776414	Missense_Mutation	SNP	G	A	A	SNCAIP	c.1387G>A	p.V463I
218H1383A1	6	117677874	117677874	Nonsense_Mutation	SNP	G	T	T	ROS1	c.4059C>A	p.Y1353*
218H1383A1	7	142459867	142459867	Missense_Mutation	SNP	C	T	T	PRSS1	c.443C>T	p.A148V
218H1383A1	8	48872556	48872556	Missense_Mutation	SNP	A	G	G	PRKDC	c.131T>C	p.L44P
218H1383A1	8	95470504	95470504	Missense_Mutation	SNP	G	T	T	RAD54B	c.296C>A	p.P99H
218H1383A1	X	53225149	53225149	Missense_Mutation	SNP	C	A	A	KDM5C	c.3069G>T	p.K1023N
218H1383A1		-	-	Frame_Shift_Ins	INS	-	-	-	ATRX	c.477dup	p.R160Tfs*29
218H1627A1		-	-	Frame_Shift_Ins	INS	-	-	-	ARID2	c.5309dup	p.T1771Nfs*11
218H1627A1	16	347779	347779	Missense_Mutation	SNP	C	T	T	AXIN1	c.1727G>A	p.R576Q
218H1627A1	17	7577105	7577105	Missense_Mutation	SNP	G	A	A	TP53	c.833C>T	p.P278L
218H1627A1	17	37868208	37868208	Missense_Mutation	SNP	C	T	T	ERBB2	c.929C>T	p.S310F
218H1627A1	17	37881440	37881440	Missense_Mutation	SNP	C	T	T	ERBB2	c.2632C>T	p.H878Y
218H1627A1		-	-	In_Frame_Del	INS	-	-	-	ERBB4	c.3579_3590del	N1193_P1196del
218H1627A1	3	142184013	142184013	Missense_Mutation	SNP	T	C	C	ATR	c.6967A>G	p.I2323V
218H1627A1		-	-	In_Frame_Del	INS	-	-	-	KMT2C	c.5427del	p.L1810*
218H1627A1	8	37555941	37555941	Missense_Mutation	SNP	G	T	T	ZNF703	c.1522G>T	p.A508S
218H1627A1	9	21971186	21971186	Nonsense_Mutation	SNP	G	A	A	CDKN2A	c.172C>T	p.R58*
218H1627A1	X	110439716	110439716	Nonsense_Mutation	SNP	C	T	T	PAK3	c.1255C>T	p.R419*
218H1955A1	17	7578449	7578449	Missense_Mutation	SNP	C	T	T	TP53	c.481G>A	p.A161T
218H1955A1		-	-	Frame_Shift_Del	INS	-	-	-	PBRM1	c.3197_3213del	p.P1066Rfs*10
218H1955A1	4	20569192	20569192	Missense_Mutation	SNP	C	T	T	SLIT2	c.2902C>T	p.H968Y
218H1955A1	7	42005901	42005901	Missense_Mutation	SNP	C	T	T	GLI3	c.2770G>A	p.A924T
218H2052A1	1	16462185	16462185	Nonsense_Mutation	SNP	G	A	A	EPHA2	c.1393C>T	p.R465*
218H2052A1		-	-	Frame_Shift_Ins	INS	-	-	-	TP53	c.902dup	p.G302Rfs*4
218H2052A1	17	37880998	37880998	Missense_Mutation	SNP	G	T	T	ERBB2	c.2327G>T	p.G776V
218H2052A1	17	41245075	41245075	Missense_Mutation	SNP	C	T	T	BRCA1	c.2473G>A	p.D825N
218H2159A1	12	25398284	25398284	Missense_Mutation	SNP	C	A	A	KRAS	c.35G>T	p.G12V
218H2159A1	17	7577108	7577108	Missense_Mutation	SNP	C	A	A	TP53	c.830G>T	p.C277F
218I2875A1	1	16256442	16256442	Missense_Mutation	SNP	A	T	T	SPEN	c.3707A>T	p.D1236V
218I2875A1	12	133218820	133218820	Missense_Mutation	SNP	T	C	C	POLE	c.5116A>G	p.M1706V
218I2875A1		-	-	Frame_Shift_Del	INS	-	-	-	CTCF	c.615_624del	p.K206Rfs*13
218I2875A1	17	7578203	7578203	Missense_Mutation	SNP	C	A	A	TP53	c.646G>T	p.V216L
218I2875A1	2	209113113	209113113	Missense_Mutation	SNP	G	A	A	IDH1	c.394C>T	p.R132C
218I2875A1	20	36012626	36012626	Missense_Mutation	SNP	G	A	A	SRC	c.70G>A	p.V24M
218I3043A1		-	-	Frame_Shift_Del	INS	-	-	-	TP53	c.626_627del	p.R209Kfs*6
218I3043A1	19	42796753	42796753	Missense_Mutation	SNP	G	A	A	CIC	c.3211G>A	p.G1071S
218I3043A1	2	140997050	140997050	Missense_Mutation	SNP	G	T	T	LRP1B	c.13376C>A	p.T4459N
218I3043A1	2	141598571	141598571	Missense_Mutation	SNP	A	G	G	LRP1B	c.5030T>C	p.V1677A
218I3043A1	8	90958514	90958514	Missense_Mutation	SNP	T	C	C	NBN	c.1924A>G	p.K642E
218I3048A1	1	32751309	32751309	Nonsense_Mutation	SNP	C	T	T	LCK	c.1522C>T	p.Q508*
218I3048A1	10	70426835	70426835	Missense_Mutation	SNP	C	G	G	TET1	c.4495C>G	p.L1499V
218I3048A1	17	7577099	7577099	Missense_Mutation	SNP	C	T	T	TP53	c.839G>A	p.R280K
218I3048A1	17	7577543	7577543	Missense_Mutation	SNP	C	T	T	TP53	c.738G>A	p.M246I
218I3048A1	3	142168452	142168452	Splice_Site	SNP	G	C	C	ATR	c.7762-8C>G	-
218I3048A1	3	142176554	142176554	Missense_Mutation	SNP	G	C	C	ATR	c.7547C>G	p.T2516S
218I3048A1	X	70612782	70612782	Missense_Mutation	SNP	C	T	T	TAF1	c.3049C>T	p.R1017C
218I3048A1		-	-	In_Frame_Ins	INS	-	-	-	STAG2	C.2133dup	P.N712*
218J4306A1		-	-	Frame_Shift_Ins	INS	-	-	-	EPHA2	c.1589_1590ins	p.E530Dfs*66
218J4306A1	1	158606472	158606472	Missense_Mutation	SNP	G	A	A	SPTA1	c.5269C>T	p.R1757C
218J4306A1	2	209113113	209113113	Missense_Mutation	SNP	G	A	A	IDH1	c.394C>T	p.R132C
218J4306A1		-	-	Frame_Shift_Ins	INS	-	-	-	NF2	c.186dup	p.G63Wfs*23
218J4306A1	8	68864674	68864674	Missense_Mutation	SNP	G	C	C	PREX2	c.45G>C	p.K15N
218J4308A1	13	32911410	32911410	Nonsense_Mutation	SNP	C	A	A	BRCA2	c.2918C>A	p.S973*
218J4308A1	16	89845208	89845208	Splice_Site	SNP	C	G	G	FANCA	c.1826+1G>C	-
218J4308A1		-	-	Frame_Shift_Ins	INS	-	-	-	STK11	c.1012dup	p.V338Gfs*22
218J4308A1	19	45916954	45916954	Missense_Mutation	SNP	G	C	C	ERCC1	c.824C>G	p.P275R
218J4308A1	2	121746495	121746495	Missense_Mutation	SNP	C	A	A	GLI2	c.3005C>A	p.A1002D
218J4308A1	20	57484464	57484464	Missense_Mutation	SNP	C	G	G	GNAS	c.645C>G	p.D215E
218J4308A1	3	195605917	195605917	Splice_Site	SNP	C	T	T	TNK2	c.1014+3G>A	-
218J4341A1	1	11187212	11187212	Splice_Site	SNP	A	C	C	MTOR	c.6217-11T>G	-
218J4341A1	12	46231201	46231201	Splice_Site	SNP	G	T	T	ARID2	c.1120+1G>T	-
218J4341A1		-	-	Frame_Shift_Del	INS	-	-	-	ARID2	c.1616_1632del	p.S539Yfs*4
218J4341A1	17	7573976	7573976	Nonsense_Mutation	SNP	T	A	A	TP53	c.1051A>T	p.K351*
218J4341A1	3	178952085	178952085	Missense_Mutation	SNP	A	G	G	PIK3CA	c.3140A>G	p.H1047R
218J4341A1	7	55211089	55211089	Missense_Mutation	SNP	T	A	A	EGFR	c.332T>A	p.M111K
218J4341A1	8	32613989	32613989	Missense_Mutation	SNP	A	G	G	NRG1	c.410A>G	p.N137S
218J4341A1	8	48792158	48792158	Nonsense_Mutation	SNP				PRKDC	c.5128A>T	p.R1710*
218J4343A1	12	25398284	25398284	Missense_Mutation	SNP	C	T	T	KRAS	c.35G>A	p.G12D
218J4343A1	18	48591895	48591895	Missense_Mutation	SNP	A	G	G	SMAD4	c.1058A>G	p.Y353C
218J4343A1	22	29669841	29669841	Nonsense_Mutation	SNP	C	T	T	EWSR1	c.214C>T	p.Q72*
218J4343A1	9	22008876	22008876	Missense_Mutation	SNP	A	G	G	CDKN2B	c.77T>C	p.L26P
218J4343A1		-	-	Splice_Site	SNP	-	-	-	RBM10	c.543_576+4del	-
218J4612A1	3	178952085	178952085	Missense_Mutation	SNP	A	G	G	PIK3CA	c.3140A>G	p.H1047R
218J4612A1	3	189584549	189584549	Missense_Mutation	SNP	G	A	A	TP63	c.845G>A	p.G282E

218J4612A1	6	30866999	30866999	Missense_Mutation	SNP	C	T	T	DDR1	c.2557C>T	p.R853W
218J4612A1	X	44949176	44949176	Splice_Site	SNP	G	C	C	KDM6A	c.3736+1G>C	-
218J4612A1	X	47426725	47426725	Missense_Mutation	SNP	G	A	A	ARAF	c.970G>A	p.V324M
218J5499A1	12	4554699	4554699	Missense_Mutation	SNP	C	T	T	FGF6	c.38G>A	p.R13Q
218J5499A1		-	-	Frame_Shift_Del	INS	-	-	-	NF1	c.4354_4358del	p.H1452Sfs*13
218J5499A1	19	52719151	52719151	Splice_Site	SNP	G	C	C	PPP2R1A	c.922+5G>C	-
218J5499A1	22	41513644	41513644	Missense_Mutation	SNP	G	T	T	EP300	c.548G>T	p.G183V
218J5499A1	7	2984069	2984069	Missense_Mutation	SNP	G	C	C	CARD11	c.461C>G	p.A154G
218K6790A1	11	118373323	118373323	Missense_Mutation	SNP	T	C	C	KMT2A	c.6716T>C	p.L2239P
218K6790A1	12	12870782	12870782	Missense_Mutation	SNP	C	A	A	CDKN1B	c.9C>A	p.N3K
218K6790A1	14	36986493	36986493	Missense_Mutation	SNP	C	G	G	NKX2-1	c.1196G>C	p.R399P
218K6790A1	15	75090959	75090959	Missense_Mutation	SNP	G	T	T	CSK	c.19G>T	p.A7S
218K6790A1		-	-	Splice_Site	SNP	-	-	-	TP53	3-1delinsACCAC	-
218K6790A1	17	7577565	7577565	Missense_Mutation	SNP	T	C	C	TP53	c.716A>G	p.N239S
218K6790A1	17	37671991	37671991	Missense_Mutation	SNP	C	A	A	CDK12	c.2776C>A	p.L926I
218K6790A1	2	198266609	198266609	Missense_Mutation	SNP	A	C	C	SF3B1	c.2227T>G	p.L743V
218K6790A1	6	138192350	138192350	Splice_Site	SNP				TNFAIP3	c.-15-5T>C	-
218K6790A1	7	81359066	81359066	Missense_Mutation	SNP	G	C	C	HGF	c.895C>G	p.P299A
218K6907A1	17	7578406	7578406	Missense_Mutation	SNP	C	T	T	TP53	c.524G>A	p.R175H
218K6907A1	2	141259449	141259449	Splice_Site	SNP	A	T	T	LRP1B	c.8663-6T>A	-
218K6907A1	6	32187467	32187467	Missense_Mutation	SNP	C	T	T	NOTCH4	c.1412G>A	p.R471H
218K6907A1	8	68968203	68968203	Missense_Mutation	SNP	T	C	C	PREX2	c.1232T>C	p.L411P
218K6907A1	X	70338696	70338696	Missense_Mutation	SNP	A	G	G	MED12	c.92A>G	p.Q31R
218L7136A1	12	58145430	58145430	Missense_Mutation	SNP	C	T	T	CDK4	c.71G>A	p.R24H
218L7136A1	16	81957117	81957117	Missense_Mutation	SNP	G	T	T	PLCG2	c.2335G>T	p.D779Y
218L7137A1	12	25398284	25398284	Missense_Mutation	SNP	C	A	A	KRAS	c.35G>T	p.G12V
218L7137A1	20	57484420	57484420	Missense_Mutation	SNP	C	G	G	GNAS	c.601C>G	p.R201G
218L7990A1	1	206649713	206649713	Splice_Site	SNP	A	T	T	IKBKE	c.540+8A>T	-
218L7990A1	17	7577114	7577114	Missense_Mutation	SNP	C	A	A	TP53	c.824G>T	p.C275F
218L7990A1	3	189607225	189607225	Missense_Mutation	SNP	C	G	G	TP63	c.1604C>G	p.S535C
218L7990A1	7	117144312	117144312	Missense_Mutation	SNP	C	G	G	CFTR	c.59C>G	p.T20S
218L9107A1	16	67654728	67654728	Splice_Site	SNP	T	G	G	CTCF	c.1207+8T>G	-
218L9107A1	17	7578370	7578370	Splice_Site	SNP	C	T	T	TP53	c.559+1G>A	-
218L9107A1	5	180047888	180047888	Missense_Mutation	SNP	C	T	T	FLT4	c.2287G>A	p.V763M
218L9107A1		-	-	In_Frame_Del	INS	-	-	-	WISP3	c.623_625del	K208_C209delinsS
219A1770D1	17	7578413	7578413	Missense_Mutation	SNP	C	T	T	TP53	c.517G>A	p.V173M
219A1770D1		-	-	Frame_Shift_Del	INS	-	-	-	SMAD4	C.1596_1603de	P.L533Pfs*41
219A1770D1	2	141819768	141819768	Missense_Mutation	SNP	C	T	T	LRP1B	c.1088G>A	p.R363K
219A1770D1	4	66230893	66230893	Missense_Mutation	SNP	C	T	T	EPHA5	c.2078G>A	p.R693H
219A1770D1	4	126239215	126239215	Missense_Mutation	SNP	T	G	G	FAT4	c.1649T>G	p.L550R
219A1770D1	X	100611040	100611040	Missense_Mutation	SNP	C	A	A	BTK	c.1566G>T	p.L522L
219A1770D1	X	123019573	123019573	Missense_Mutation	SNP	G	C	C	XIAP	c.61G>C	p.E21Q
219A9920A1	1	120484314	120484314	Missense_Mutation	SNP	G	A	A	NOTCH2	c.2816C>T	p.P939L
219A9920A1	17	7578392	7578392	Nonsense_Mutation	SNP	C	A	A	TP53	c.538G>T	p.E180*
219A9920A1	2	142004878	142004878	Missense_Mutation	SNP	C	G	G	LRP1B	c.509G>C	p.R170T
219A9920A1	7	106508720	106508720	Missense_Mutation	SNP	C	A	A	PIK3CG	c.714C>A	p.D238E
219A9920A1	9	133759526	133759526	Missense_Mutation	SNP	C	T	T	ABL1	c.1906C>T	p.R636C
219A9920A1	X	44921969	44921969	Missense_Mutation	SNP	G	T	T	KDM6A	c.1503G>T	p.Q501H
24059085A1	9	87570318	87570318	Missense_Mutation	SNP	G	C	C	NTRK2	c.2058G>C	p.Q686H
24059085A1	17	7577559	7577559	Missense_Mutation	SNP	G	C	C	TP53	c.722C>G	p.S241C
24059085A1	2	141108425	141108425	Missense_Mutation	SNP	C	T	T	LRP1B	c.11833G>A	p.G3945R
24059085A1	3	52441226	52441226	Nonsense_Mutation	SNP	C	A	A	BAP1	c.544G>T	p.E182*
618F8157A2	11	108128226	108128226	Nonsense_Mutation	SNP	G	T	T	ATM	c.2269G>T	p.G757*
618F8157A2	17	16004563	16004563	Splice_Site	SNP	C	A	A	NCOR1	c.2690+1G>T	-
618F8167A2	3	30713760	30713760	Missense_Mutation	SNP	A	G	G	TGFBR2	c.1085A>G	p.H362R
618F8167A2		-	-	Frame_Shift_Del	INS	-	-	-	BAP1	c.421del	p.H141Ifs*46
618F8167A2	7	77789586	77789586	Missense_Mutation	SNP	C	A	A	MAGI2	c.2601G>T	p.E867D
ASCO795410	3	142272103	142272103	Missense_Mutation	SNP	C	T	T	ATR	2771G>A;2777T	).[S924N;F926C]
ASCO795410		-	-	Frame_Shift_Del	INS	-	-	-	SETD2	C.5481del	P.W1827Cfs*11
ASCO795410	12	25398281	25398281	Missense_Mutation	SNP	C	T	T	KRAS	c.38G>A	p.G13D
CASE111111	1	27088755	27088755	Nonsense_Mutation	SNP	C	A	A	ARID1A	c.2364C>A	p.Y788*
CASE111111	1	202710591	202710591	Missense_Mutation	SNP	G	A	A	KDM5B	c.2957C>T	p.P986L
CASE111111	1	204437999	204437999	Missense_Mutation	SNP	G	A	A	PIK3C2B	c.932C>T	p.P311L
CASE111111	10	43601830	43601830	Missense_Mutation	SNP	G	A	A	RET	c.874G>A	p.V292M
CASE111111	11	9597523	9597523	Missense_Mutation	SNP	A	G	G	WEE1	c.665A>G	p.K222R
CASE111111	11	92088570	92088570	Missense_Mutation	SNP	G	A	A	FAT3	c.3292G>A	p.G1098R
CASE111111	12	25398284	25398284	Missense_Mutation	SNP	C	T	T	KRAS	c.35G>A	p.G12D
CASE111111		-	-	Splice_Site	SNP	-	-	-	RB1	c.1128-5delT	-
CASE111111	13	48947631	48947631	Splice_Site	SNP	A	G	G	RB1	c.1215+3A>G	-
CASE111111	16	81968061	81968061	Missense_Mutation	SNP	A	T	T	PLCG2	c.2767A>T	p.N923Y
CASE111111	16	81969933	81969933	Missense_Mutation	SNP	G	A	A	PLCG2	c.3002G>A	p.R1001H
CASE111111	17	7577085	7577085	Nonsense_Mutation	SNP	C	A	A	TP53	c.853G>T	p.E285*
CASE111111	17	7577609	7577609	Splice_Site	SNP	C	T	T	TP53	c.673-1G>A	-
CASE111111	17	15961283	15961283	Missense_Mutation	SNP	C	A	A	NCOR1	c.6106G>T	p.A2036S
CASE111111	17	15983379	15983379	Splice_Site	SNP	C	G	G	NCOR1	c.3401-1G>C	-
CASE111111	17	29527462	29527462	Missense_Mutation	SNP	G	A	A	NF1	c.911G>A	p.R304Q
CASE111111	17	37627362	37627362	Missense_Mutation	SNP	T	A	A	CDK12	c.1277T>A	p.F426Y
CASE111111	17	38510560	38510560	Missense_Mutation	SNP	C	T	T	RARA	c.814C>T	p.R272W
CASE111111	19	30314572	30314572	Missense_Mutation	SNP	G	A	A	CCNE1	c.1121G>A	p.R374Q
CASE111111	2	121708880	121708880	Missense_Mutation	SNP	C	T	T	GLI2	c.316C>T	p.H106Y
CASE111111	20	62173546	62173546	Missense_Mutation	SNP	G	T	T	SRMS	c.916C>A	p.R306S
CASE111111	22	41574907	41574907	Missense_Mutation	SNP	G	A	A	EP300	c.7192G>A	p.D2398N
CASE111111	3	38182748	38182748	Missense_Mutation	SNP	C	T	T	MYD88	c.925C>T	p.R309C
CASE111111	3	142268985	142268985	Missense_Mutation	SNP	G	A	A	ATR	c.2965C>T	p.R989C
CASE111111	4	55946257	55946257	Missense_Mutation	SNP	C	T	T	KDR	c.3922G>A	p.G1308R
CASE111111	4	187541228	187541228	Missense_Mutation	SNP	G	A	A	FAT1	c.6512C>T	p.P2171L
CASE111111	5	38975689	38975689	Missense_Mutation	SNP	C	T	T	RICTOR	c.839G>A	p.R280Q
CASE111111	5	176523067	176523067	Missense_Mutation	SNP	C	T	T	FGFR4	c.1831C>T	p.R611W
CASE111111	6	138201337	138201337	Missense_Mutation	SNP	T	C	C	TNFAIP3	c.2036T>C	p.I679T
CASE111111	7	2976817	2976817	Missense_Mutation	SNP	C	T	T	CARD11	c.1195G>A	p.E399K
CASE111111	7	116340214	116340214	Missense_Mutation	SNP	G	A	A	MET	c.1076G>A	p.R359Q
CASE111111	7	151874767	151874767	Missense_Mutation	SNP	C	T	T	KMT2C	c.7771G>A	p.G2591R
CASE111111	8	41836193	41836193	Missense_Mutation	SNP	C	T	T	KAT6A	c.1010G>A	p.R337H
CASE111111	9	98220390	98220390	Missense_Mutation	SNP	G	A	A	PTCH1	c.3073C>T	p.R1025C
CASE111111	X	129159352	129159352	Missense_Mutation	SNP	C	A	A	BCORL1	c.4076C>A	p.A1359E
CASE222222	1	45805988	45805988	Splice_Site	SNP	C	A	A	MUTYH	c.-62G>T	-
CASE222222	11	108117784	108117784	Missense_Mutation	SNP	A	G	G	ATM	c.995A>G	p.Y332C
CASE222222	11	118344839	118344839	Missense_Mutation	SNP	C	G	G	KMT2A	c.2965C>G	p.L989V

CASE222222	14	95566106	95566106	Splice_Site	SNP	C	A	A	DICER1	c.4206+11G>T	-
CASE222222	14	102549644	102549644	Splice_Site	SNP	G	T	T	HSP90AA1	c.1853-5C>A	-
CASE222222	16	3807850	3807850	Missense_Mutation	SNP	T	C	C	CREBBP	c.3569A>G	p.D1190G
CASE222222	16	23646324	23646324	Nonsense_Mutation	SNP	T	A	A	PALB2	c.1543A>T	p.K515*
CASE222222	17	7577509	7577509	Missense_Mutation	SNP	C	T	T	TP53	c.772G>A	p.E258K
CASE222222	2	121744143	121744143	Missense_Mutation	SNP	C	T	T	GLI2	c.2246C>T	p.P749L
CASE222222	21	44840885	44840885	Splice_Site	SNP	C	T	T	SIK1	c.748+5G>A	-
CASE222222		-	-	Frame_Shift_Del	INS	-	-	-	SETD2	C.5533del	P.S1845Vfs*20
CASE222222	3	49940126	49940126	Missense_Mutation	SNP	C	T	T	MST1R	c.917G>A	p.R306H
CASE222222	3	195593777	195593777	Nonsense_Mutation	SNP	C	T	T	TNK2	c.3093G>A	p.W1031*
CASE222222	4	1952795	1952795	Splice_Site	SNP	C	G	G	NSD2	c.1882-4C>G	-
CASE222222	4	126336878	126336878	Missense_Mutation	SNP	G	A	A	FAT4	c.6760G>A	p.D2254N
CASE222222	5	131927598	131927598	Missense_Mutation	SNP	A	G	G	RAD50	c.1665A>G	p.I555M
CASE222222	6	32165209	32165209	Missense_Mutation	SNP	A	T	T	NOTCH4	c.4919T>A	p.L1640Q
CASE222222	9	21971209	21971209	Splice_Site	SNP	T	C	C	CDKN2A	c.151-2A>G	-
CASE222222	X	76874314	76874314	Missense_Mutation	SNP	C	T	T	ATRX	c.5408G>A	p.R1803H
CASE222222	X	123185061	123185061	Missense_Mutation	SNP	C	T	T	STAG2	c.1108C>T	p.R370W
CASE333333	1	158632689	158632689	Missense_Mutation	SNP	A	G	G	SPTA1	c.2267T>C	p.I756T
CASE333333	10	70404467	70404467	Missense_Mutation	SNP	A	C	C	TET1	c.1981A>C	p.N661H
CASE333333	10	104595035	104595035	Missense_Mutation	SNP	C	T	T	CYP17A1	c.412G>A	p.G138S
CASE333333	10	123239457	123239457	Missense_Mutation	SNP	C	G	G	FGFR2	c.2380G>C	p.D794H
CASE333333	12	112939990	112939990	Missense_Mutation	SNP	T	C	C	PTPN11	c.1642T>C	p.S548P
CASE333333	17	38555017	38555017	Splice_Site	SNP	C	T	T	TOP2A	c.3453+8G>A	-
CASE333333	19	2216771	2216771	Splice_Site	SNP	G	A	A	DOT1L	c.2408+7G>A	-
CASE333333	19	10475580	10475580	Missense_Mutation	SNP	C	T	T	TYK2	c.1156G>A	p.V386M
CASE333333	19	15272347	15272347	Missense_Mutation	SNP	C	T	T	NOTCH3	c.6092G>A	p.R2031H
CASE333333	19	15303304	15303304	Missense_Mutation	SNP	C	T	T	NOTCH3	c.224G>A	p.R75Q
CASE333333	19	42776140	42776140	Missense_Mutation	SNP	C	T	T	CIC	c.205C>T	p.R69W
CASE333333	2	29416667	29416667	Missense_Mutation	SNP	T	C	C	ALK	c.4286A>G	p.Q1429R
CASE333333	2	29462691	29462691	Missense_Mutation	SNP	G	A	A	ALK	c.2210C>T	p.S737L
CASE333333	2	29543716	29543716	Missense_Mutation	SNP	C	T	T	ALK	c.1447G>A	p.E483K
CASE333333	4	175896743	175896743	Missense_Mutation	SNP	G	A	A	ADAM29	c.67G>A	p.E23K
CASE333333	5	131915115	131915115	Missense_Mutation	SNP	C	T	T	RAD50	c.472C>T	p.H158Y
CASE333333		-	-	Frame_Shift_Ins	INS	-	-	-	RAD50	C.2165dup	P.E723Gfs*5
CASE333333	8	48689409	48689409	Splice_Site	SNP				PRKDC	c.12180-3C>T	-
CASE333333	9	93626890	93626890	Missense_Mutation	SNP	A	G	G	SYK	c.737A>G	p.Y246C
CASE444444	1	16262537	16262537	Missense_Mutation	SNP	C	T	T	SPEN	c.9802C>T	p.R3268C
CASE444444	1	97981368	97981368	Missense_Mutation	SNP	T	G	G	DPYD	c.1654A>C	p.T552P
CASE444444	1	120491753	120491753	Splice_Site	SNP	G	C	C	NOTCH2	c.2480-4C>G	-
CASE444444	11	111965567	111965567	Missense_Mutation	SNP	A	G	G	SDHD	c.353A>G	p.D118G
CASE444444	12	1034604	1034604	Splice_Site	SNP	T	C	C	RAD52	c.543+12A>G	-
CASE444444	17	7578212	7578212	Nonsense_Mutation	SNP	G	A	A	TP53	c.637C>T	p.R213*
CASE444444	17	29654521	29654521	Missense_Mutation	SNP	G	T	T	NF1	c.5273G>T	p.G1758V
CASE444444	17	56448400	56448400	Splice_Site	SNP	G	A	A	RNF43	c.253-6C>T	-
CASE444444	18	60795959	60795959	Missense_Mutation	SNP	G	A	A	BCL2	c.619C>T	p.R207W
CASE444444	19	42794884	42794884	Missense_Mutation	SNP	T	C	C	CIC	c.1964T>C	p.L655P
CASE444444	4	20618743	20618743	Missense_Mutation	SNP	G	T	T	SLIT2	c.4058G>T	p.G1353V
CASE444444	X	48123213	48123213	Splice_Site	SNP	A	G	G	SSX1	c.331-4A>G	-
CASE555555	1	16262429	16262429	Missense_Mutation	SNP	G	A	A	SPEN	c.9694G>A	p.E3232K
CASE555555	1	204438084	204438084	Missense_Mutation	SNP	G	C	C	PIK3C2B	c.847C>G	p.Q283E
CASE555555	10	43606814	43606814	Missense_Mutation	SNP	C	T	T	RET	c.1423C>T	p.R475W
CASE555555	11	92085554	92085554	Missense_Mutation	SNP	C	G	G	FAT3	c.276C>G	p.F92L
CASE555555	11	92531376	92531376	Missense_Mutation	SNP	C	T	T	FAT3	c.5197C>T	p.R1733C
CASE555555	12	92538031	92538031	Missense_Mutation	SNP	C	A	A	BTG1	c.341G>T	p.R114I
CASE555555		-	-	Frame_Shift_Ins	INS	-	-	-	BRCA2	C.1813dup	P.I605Nfs*11
CASE555555	13	48936983	48936983	Nonsense_Mutation	SNP	C	T	T	RB1	c.751C>T	p.R251*
CASE555555	17	7577099	7577099	Missense_Mutation	SNP	C	G	G	TP53	c.839G>C	p.R280T
CASE555555	17	56448354	56448354	Missense_Mutation	SNP	T	C	C	RNF43	c.293A>G	p.N98S
CASE555555	2	112758843	112758843	Missense_Mutation	SNP	G	A	A	MERTK	c.1670G>A	p.R557Q
CASE555555	2	141283818	141283818	Nonsense_Mutation	SNP	C	A	A	LRP1B	c.7864G>T	p.E2622*
CASE555555	3	130799295	130799295	Missense_Mutation	SNP	G	A	A	NEK11	c.199G>A	p.E67K
CASE555555	3	142281429	142281429	Missense_Mutation	SNP	G	A	A	ATR	c.815C>T	p.S272L
CASE555555	4	126370901	126370901	Missense_Mutation	SNP	C	A	A	FAT4	c.8730C>A	p.F2910L
CASE555555	5	35876391	35876391	Missense_Mutation	SNP	A	C	C	IL7R	c.1183A>C	p.K395Q
CASE555555	6	162206948	162206948	Splice_Site	SNP	G	A	A	PRKN	c.735-8C>T	-
R18C5410R2	17	7577538	7577538	Missense_Mutation	SNP	C	T	T	TP53	c.743G>A	p.R248Q
R18C5410R2	20	31021211	31021211	Nonsense_Mutation	SNP	C	T	T	ASXL1	c.1210C>T	p.R404*
R18C5410R2	5	121758851	121758851	Missense_Mutation	SNP	T	A	A	SNCAIP	c.419T>A	p.L140Q
R18C5410R2	5	149497300	149497300	Missense_Mutation	SNP	G	A	A	PDGFRB	c.3018C>T	p.S1006S
R18C5410R2	8	71068858	71068858	Missense_Mutation	SNP	T	G	G	NCOA2	c.1742A>C	p.D581A
R18C5411R2	1	27056207	27056207	Nonsense_Mutation	SNP	C	G	G	ARID1A	c.1203C>G	p.Y401*
R18C5411R2	1	115256530	115256530	Missense_Mutation	SNP	G	T	T	NRAS	c.181C>A	p.Q61K
R18C5411R2		-	-	Frame_Shift_Ins	INS	-	-	-	SMAD4	C.153dup	P.D52Rfs*2
R18C5411R2	2	61726995	61726995	Missense_Mutation	SNP	G	T	T	XPO1	c.443C>A	p.T148N
R18C5411R2	X	44949082	44949082	Missense_Mutation	SNP	G	A	A	KDM6A	c.3643G>A	p.G1215R
R18D6313R1	9	135772702	135772702	Nonsense_Mutation	SNP	A	C	C	TSC1	c.2844T>G	p.Y948*
R18D6314R1		-	-	Frame_Shift_Del	INS	-	-	-	ARID1A	6_5327delinsTT	p.E1776Ffs*3
R18D6314R1		-	-	Frame_Shift_Del	INS	-	-	-	ARID2	c.4632_4651del	p.A1547Gfs*14
R18D6314R1	7	2974086	2974086	Splice_Site	SNP	C	A	A	CARD11	c.1518+1G>T	-
R18D6315R1	1	27106354	27106354	Nonsense_Mutation	SNP	C	T	T	ARID1A	c.5965C>T	p.R1989*
R18D6316R1		-	-	Frame_Shift_Del	INS	-	-	-	JAK1	c.472del	p.L158Cfs*10
R18D6316R1	15	93557954	93557954	Missense_Mutation	SNP	G	T	T	CHD2	c.4721G>T	p.G1574V
R18D6316R1	16	360008	360008	Nonsense_Mutation	SNP	G	A	A	AXIN1	c.1081C>T	p.Q361*
R18D6316R1	17	37868208	37868208	Missense_Mutation	SNP	C	T	T	ERBB2	c.929C>T	p.S310F
R18D6316R1	2	29419665	29419665	Missense_Mutation	SNP	T	C	C	ALK	c.4135A>G	p.I1379V
R18D6316R1	7	116371867	116371867	Missense_Mutation	SNP	A	G	G	MET	c.1346A>G	p.D449G
R18D6317R1		-	-	In_Frame_Ins	INS	-	-	-	EPHA2	c.713dup	p.E241*
R18D6317R1	17	7577120	7577120	Missense_Mutation	SNP	C	T	T	TP53	c.818G>A	p.R273H
R18D6317R1	X	47044850	47044850	Nonsense_Mutation	SNP	C	T	T	RBM10	c.2176C>T	p.R726*
R18D6417R1	1	9780797	9780797	Splice_Site	SNP	C	T	T	PIK3CD	c.1522-3C>T	-
R18D6417R1	11	108201099	108201099	Missense_Mutation	SNP	C	T	T	ATM	c.7466C>T	p.S2489F
R18D6417R1	12	6687638	6687638	Missense_Mutation	SNP	C	T	T	CHD4	c.5056G>A	p.E1686K
R18D6417R1	12	49425896	49425896	Nonsense_Mutation	SNP	G	A	A	KMT2D	c.12592C>T	p.R4198*
R18D6417R1	15	93543766	93543766	Missense_Mutation	SNP	C	T	T	CHD2	c.4033C>T	p.R1345W
R18D6417R1	17	16040727	16040727	Splice_Site	SNP	C	T	T	NCOR1	c.1408-1G>A	-
R18D6417R1	2	121745873	121745873	Missense_Mutation	SNP	G	A	A	GLI2	c.2383G>A	p.E795K
R18D6417R1	2	212488655	212488655	Missense_Mutation	SNP	C	A	A	ERBB4	c.2194G>T	p.V732F
R18D6417R1	3	178936082	178936082	Missense_Mutation	SNP	G	A	A	PIK3CA	c.1624G>A	p.E542K

R18D6417R1	3	178936091	178936091	Missense_Mutation	SNP	G	A	A	PIK3CA	c.1633G>A	p.E545K
R18D6417R1	6	32184839	32184839	Missense_Mutation	SNP	C	G	G	NOTCH4	c.1744G>C	p.E582Q
R18D6417R1	6	33286968	33286968	Missense_Mutation	SNP	C	G	G	DAXX	c.1969G>C	p.E657Q
R18D6417R1	6	33287293	33287293	Missense_Mutation	SNP	C	G	G	DAXX	c.1804G>C	p.E602Q
R18D6417R1	X	70608704	70608704	Missense_Mutation	SNP	G	C	C	TAF1	c.2746G>C	p.E916Q
R18D6463R1	1	16477446	16477446	Missense_Mutation	SNP	T	A	A	EPHA2	c.98A>T	p.D33V
R18D6463R1	13	48951052	48951052	Splice_Site	SNP	A	G	G	RB1	c.1216-2A>G	-
R18D6463R1	2	191929525	191929525	Splice_Site	SNP	C	G	G	STAT4	c.782+8G>C	-
R18D6463R1	3	178936082	178936082	Missense_Mutation	SNP	G	A	A	PIK3CA	c.1624G>A	p.E542K
R18D6470R1		-	-	Frame_Shift_Del	INS	-	-	-	RAD54L	c.488del	p.F163Sfs*25
R18D6470R1	10	90768757	90768757	Splice_Site	SNP	A	G	G	FAS	c.443+3A>G	-
R18D6470R1	11	108117783	108117783	Missense_Mutation	SNP	T	C	C	ATM	c.994T>C	p.Y332H
R18D6470R1	11	108178659	108178659	Nonsense_Mutation	SNP	A	T	T	ATM	c.5710A>T	p.K1904*
R18D6470R1	11	119169120	119169120	Missense_Mutation	SNP	A	T	T	CBL	c.2304A>T	p.E768D
R18D6470R1		-	-	Frame_Shift_Del	INS	-	-	-	ARID2	c.130del	p.E44Sfs*14
R18D6470R1	13	48934148	48934148	Splice_Site	SNP	T	C	C	RB1	c.608-5T>C	-
R18D6470R1	14	45620637	45620637	Missense_Mutation	SNP	A	G	G	FANCM	c.956A>G	p.N319S
R18D6470R1	14	95599677	95599677	Missense_Mutation	SNP	T	A	A	DICER1	c.119A>T	p.N40I
R18D6470R1		-	-	Frame_Shift_Del	INS	-	-	-	NUP93	c.880_892del	p.R294Tfs*24
R18D6470R1	16	67645024	67645024	Missense_Mutation	SNP	A	G	G	CTCF	c.289A>G	p.T97A
R18D6470R1	17	7579494	7579494	Nonsense_Mutation	SNP	T	A	A	TP53	c.193A>T	p.R65*
R18D6470R1	17	16012219	16012219	Missense_Mutation	SNP	C	T	T	NCOR1	c.2063G>A	p.R688Q
R18D6470R1	17	38555391	38555391	Splice_Site	SNP	T	A	A	TOP2A	c.3197-8A>T	-
R18D6470R1	17	59878832	59878832	Nonsense_Mutation	SNP	T	A	A	BRIP1	c.922A>T	p.K308*
R18D6470R1	18	45395607	45395607	Splice_Site	SNP	T	C	C	SMAD2	c.430+7A>G	-
R18D6470R1	2	61719504	61719504	Missense_Mutation	SNP	T	G	G	XPO1	c.1679A>C	p.K560T
R18D6470R1	4	143066982	143066982	Splice_Site	SNP	T	C	C	INPP4B	c.1720+11A>G	-
R18D6470R1	4	175898741	175898741	Missense_Mutation	SNP	A	G	G	ADAM29	c.2065A>G	p.I689V
R18D6470R1	5	38955718	38955718	Missense_Mutation	SNP	T	C	C	RICTOR	c.2588A>G	p.Y863C
R18D6470R1	8	31497909	31497909	Missense_Mutation	SNP	A	C	C	NRG1	c.409A>C	p.N137H
R18D6483R1	1	16262648	16262648	Missense_Mutation	SNP	G	C	C	SPEN	c.9913G>C	p.E3305Q
R18D6483R1	1	158650489	158650489	Missense_Mutation	SNP	C	T	T	SPTA1	c.562G>A	p.E188K
R18D6483R1	11	92532543	92532543	Missense_Mutation	SNP	G	T	T	FAT3	c.6364G>T	p.V2122F
R18D6483R1	11	119146713	119146713	Missense_Mutation	SNP	C	G	G	CBL	c.876C>G	p.I292M
R18D6483R1		-	-	Frame_Shift_Ins	INS	-	-	-	ARID2	c.4903_4904dup	p.W1636Cfs*66
R18D6483R1		-	-	Frame_Shift_Del	INS	-	-	-	ACVR1B	c.559_560del	p.S187Hfs*43
R18D6483R1	13	43155412	43155412	Missense_Mutation	SNP	C	G	G	TNFSF11	c.151C>G	p.Q51E
R18D6483R1	14	81422193	81422193	Missense_Mutation	SNP	C	G	G	TSHR	c.169C>G	p.L57V
R18D6483R1	17	7577099	7577099	Missense_Mutation	SNP	C	T	T	TP53	c.839G>A	p.R280K
R18D6483R1	17	12011144	12011144	Missense_Mutation	SNP	C	T	T	MAP2K4	c.551C>T	p.S184L
R18D6483R1	17	29663744	29663744	Missense_Mutation	SNP	T	A	A	NF1	c.6239T>A	p.I2080N
R18D6483R1	19	17945815	17945815	Splice_Site	SNP	G	A	A	JAK3	c.2048-3C>T	-
R18D6483R1	3	178936091	178936091	Missense_Mutation	SNP	G	A	A	PIK3CA	c.1633G>A	p.E545K
R18D6483R1	4	187539252	187539252	Nonsense_Mutation	SNP	G	A	A	FAT1	c.8488C>T	p.Q2830*
R18D6483R1	6	30865360	30865360	Missense_Mutation	SNP	G	C	C	DDR1	c.2091G>C	p.Q697H
R18D6483R1	7	127727059	127727059	Nonsense_Mutation	SNP	C	T	T	SND1	c.2374C>T	p.Q792*
R18D6483R1	7	148513873	148513873	Splice_Site	SNP	G	A	A	EZH2	c.1411-3C>T	-
R18D6483R1	8	68965459	68965459	Missense_Mutation	SNP	G	C	C	PREX2	c.1071G>C	p.L357F
R18D6483R1	9	135787737	135787737	Nonsense_Mutation	SNP	G	T	T	TSC1	c.845C>A	p.S282*
R18D6488R1	12	115109962	115109962	Missense_Mutation	SNP	C	T	T	TBX3	c.1916G>A	p.R639H
R18D6488R1	13	110437691	110437691	Missense_Mutation	SNP	G	A	A	IRS2	c.710C>T	p.S237L
R18D6488R1	17	7574021	7574021	Nonsense_Mutation	SNP	C	A	A	TP53	c.1006G>T	p.E336*
R18D6488R1	22	30069414	30069414	Nonsense_Mutation	SNP	G	T	T	NF2	c.1279G>T	p.E427*
R18D6489R1	16	50816345	50816345	Nonsense_Mutation	SNP	C	A	A	CYLD	c.1794C>A	p.Y598*
R18D6489R1	17	7578263	7578263	Nonsense_Mutation	SNP	G	A	A	TP53	c.586C>T	p.R196*
R18D6489R1	2	141004672	141004672	Missense_Mutation	SNP	G	A	A	LRP1B	c.13307C>T	p.S4436F
R18D6489R1	2	170070170	170070170	Missense_Mutation	SNP	G	A	A	LRP2	c.6037C>T	p.R2013C
R18D6489R1	3	134851591	134851591	Missense_Mutation	SNP	G	A	A	EPHB1	c.997G>A	p.V333I
R18D6489R1		-	-	Frame_Shift_Del	INS	-	-	-	PRKDC	c.12342del	p.N4115Tfs*48
R18D6500R1	1	204416690	204416690	Missense_Mutation	SNP	G	A	A	PIK3C2B	c.2363C>T	p.S788L
R18D6500R1	13	32893318	32893318	Missense_Mutation	SNP	G	A	A	BRCA2	c.172G>A	p.E58K
R18D6500R1	17	7578528	7578528	Missense_Mutation	SNP	A	T	T	TP53	c.402T>A	p.F134L
R18D6500R1	7	42005843	42005843	Missense_Mutation	SNP	G	A	A	GLI3	c.2828C>T	p.T943M
R18D6500R1	8	48826630	48826630	Splice_Site	SNP	A	G	G	PRKDC	c.2618-6T>C	-
R18D6500R1	X	63412907	63412907	Missense_Mutation	SNP	T	C	C	AMER1	c.260A>G	p.K87R
R18D6500R1	X	76938635	76938635	Missense_Mutation	SNP	C	T	T	ATRX	c.2113G>A	p.D705N
R18D6508R1	11	111957566	111957566	Splice_Site	SNP	C	T	T	SDHD	c.-66C>T	-
R18D6508R1	12	25398284	25398284	Missense_Mutation	SNP	C	G	G	KRAS	c.35G>C	p.G12A
R18D6508R1	16	23619201	23619201	Missense_Mutation	SNP	G	A	A	PALB2	c.3334C>T	p.P1112S
R18D6508R1	17	37868208	37868208	Missense_Mutation	SNP	C	A	A	ERBB2	c.929C>A	p.S310Y
R18D6508R1	18	48591918	48591918	Missense_Mutation	SNP	C	T	T	SMAD4	c.1081C>T	p.R361C
R18D6508R1	19	42776461	42776461	Nonsense_Mutation	SNP	G	T	T	CIC	c.526G>T	p.E176*
R18D6508R1	2	109388204	109388204	Missense_Mutation	SNP	C	A	A	RANBP2	c.7897C>A	p.L2633I
R18D6508R1	5	112176329	112176329	Nonsense_Mutation	SNP	C	T	T	APC	c.5038C>T	p.Q1680*
R18D6508R1	7	6026649	6026649	Missense_Mutation	SNP	C	T	T	PMS2	c.1747G>A	p.E583K
R18D6510R1	1	115256529	115256529	Missense_Mutation	SNP	T	A	A	NRAS	c.182A>T	p.Q61L
R18D6510R1	11	69514104	69514104	Missense_Mutation	SNP	C	T	T	FGF19	c.577G>A	p.E193K
R18D6510R1	12	46211585	46211585	Nonsense_Mutation	SNP	C	G	G	ARID2	c.551C>G	p.S184*
R18D6510R1	17	17124746	17124746	Missense_Mutation	SNP	G	C	C	FLCN	c.976C>G	p.L326V
R18D6510R1	17	56780658	56780658	Missense_Mutation	SNP	C	G	G	RAD51C	c.673C>G	p.L225V
R18D6510R1	19	15302774	15302774	Missense_Mutation	SNP	G	A	A	NOTCH3	c.676C>T	p.P226S
R18D6510R1	21	36164509	36164509	Missense_Mutation	SNP	C	T	T	RUNX1	c.1366G>A	p.E456K
R18D6510R1	22	41523488	41523488	Splice_Site	SNP	C	G	G	EP300	c.907-3C>G	-
R18D6510R1	3	178936091	178936091	Missense_Mutation	SNP	G	A	A	PIK3CA	c.1633G>A	p.E545K
R18D6510R1	3	178938934	178938934	Missense_Mutation	SNP	G	A	A	PIK3CA	c.2176G>A	p.E726K
R18D6510R1	3	185200173	185200173	Missense_Mutation	SNP	G	A	A	MAP3K13	c.2830G>A	p.D944N
R18D6510R1	4	187524726	187524726	Missense_Mutation	SNP	G	A	A	FAT1	c.10954C>T	p.P3652S
R18D6510R1	6	135521246	135521246	Missense_Mutation	SNP	G	A	A	MYB	c.1370G>A	p.R457K
R18D6510R1	7	81346668	81346668	Missense_Mutation	SNP	C	T	T	HGF	c.1285G>A	p.E429K
R18D6510R1	7	106513354	106513354	Missense_Mutation	SNP	C	T	T	PIK3CG	c.2258C>T	p.S753F
R18D6510R1	7	116339297	116339297	Missense_Mutation	SNP	G	C	C	MET	c.159G>C	p.Q53H
R18D6510R1	8	11615824	11615824	Missense_Mutation	SNP	C	G	G	GATA4	c.1169C>G	p.S390C
R18D6510R1	8	32620804	32620804	Nonsense_Mutation	SNP	C	G	G	NRG1	c.875C>G	p.S292*
R18D6510R1	8	48842522	48842522	Missense_Mutation	SNP	G	A	A	PRKDC	c.1943C>T	p.S648L
R18D6510R1	8	48847559	48847559	Splice_Site	SNP	C	G	G	PRKDC	c.1497+10G>C	-
R18D6510R1	9	80430659	80430659	Missense_Mutation	SNP	C	T	T	GNAQ	c.349G>A	p.D117N
R18D6510R1	X	70339981	70339981	Missense_Mutation	SNP	G	A	A	MED12	c.514G>A	p.E172K
R18D6510R1	X	70347799	70347799	Nonsense_Mutation	SNP	C	G	G	MED12	c.3038C>G	p.S1013*

R18D6521R1		-	-	Frame_Shift_Del	INS	-	-	-	ARID1A	c.6384_6406del	p.Q2128Hfs*14
R18D6521R1	13	26923240	26923240	Missense_Mutation	SNP	T	C	C	CDK8	c.236T>C	p.I79T
R18D6521R1	14	95578516	95578516	Missense_Mutation	SNP	G	T	T	DICER1	c.2109C>A	p.H703Q
R18D6521R1	16	9857538	9857538	Missense_Mutation	SNP	C	T	T	GRIN2A	c.3863G>A	p.R1288H
R18D6521R1		-	-	Frame_Shift_Del	INS	-	-	-	TP53	C.868del	P.R290Afs*55
R18D6521R1	19	10599915	10599915	Missense_Mutation	SNP	C	T	T	KEAP1	c.1661G>A	p.R554Q
R18D6521R1	19	17953875	17953875	Missense_Mutation	SNP	T	A	A	JAK3	c.527A>T	p.E176V
R18D6521R1	2	47693793	47693793	Splice_Site	SNP	C	G	G	MSH2	c.1511-4C>G	-
R18D6521R1	2	141079628	141079628	Missense_Mutation	SNP	C	T	T	LRP1B	c.12544G>A	p.E4182K
R18D6521R1	2	141260649	141260649	Nonsense_Mutation	SNP	C	A	A	LRP1B	c.8545G>T	p.E2849*
R18D6521R1	3	41266137	41266137	Missense_Mutation	SNP	C	T	T	CTNNB1	c.134C>T	p.S45F
R18D6521R1	4	187524422	187524422	Missense_Mutation	SNP	G	T	T	FAT1	c.11258C>A	p.S3753Y
R18D6521R1	6	157256633	157256633	Missense_Mutation	SNP	C	A	A	ARID1B	c.1960C>A	p.Q654K
R18D6521R1	7	81388068	81388068	Missense_Mutation	SNP	T	G	G	HGF	c.307A>C	p.S103R
R18D6523R1	17	7578403	7578403	Missense_Mutation	SNP	C	T	T	TP53	c.527G>A	p.C176Y
R18D6523R1	17	37868208	37868208	Missense_Mutation	SNP	C	T	T	ERBB2	c.929C>T	p.S310F
R18D6523R1	17	56492790	56492790	Missense_Mutation	SNP	A	C	C	RNF43	c.149T>G	p.V50G
R18D6523R1		-	-	Frame_Shift_Ins	INS	-	-	-	TET2	c.2520dup	p.V841Sfs*5
R18D6523R1	9	21971035	21971035	Missense_Mutation	SNP	T	C	C	CDKN2A	c.323A>G	p.D108G
R18D6529R1	10	89717607	89717607	Splice_Site	SNP	C	G	G	PTEN	c.635-3C>G	-
R18D6529R1	11	119167693	119167693	Missense_Mutation	SNP	T	G	G	CBL	c.2102T>G	p.M701R
R18D6529R1	12	4383376	4383376	Missense_Mutation	SNP	G	C	C	CCND2	c.170G>C	p.R57T
R18D6529R1	12	46245858	46245858	Nonsense_Mutation	SNP	C	T	T	ARID2	3952C>T;3959C	[Q1318*;S1320L]
R18D6529R1	12	133238115	133238115	Missense_Mutation	SNP	C	G	G	POLE	c.2862G>C	p.K954N
R18D6529R1	13	32914890	32914890	Nonsense_Mutation	SNP	C	G	G	BRCA2	6398C>G;6425C	[S2133*;S2142C]
R18D6529R1	14	81422193	81422193	Missense_Mutation	SNP	C	T	T	TSHR	c.169C>T	p.L57L
R18D6529R1	16	89839697	89839697	Missense_Mutation	SNP	C	G	G	FANCA	c.1996G>C	p.D666H
R18D6529R1	17	7577082	7577082	Missense_Mutation	SNP	C	T	T	TP53	c.856G>A	p.E286K
R18D6529R1		-	-	Frame_Shift_Del	INS	-	-	-	ROCK2	c.2855_2874del	p.E952Afs*14
R18D6529R1	3	30715684	30715684	Missense_Mutation	SNP	T	G	G	TGFBR2	c.1342T>G	p.Y448D
R18D6529R1	4	1805458	1805458	Missense_Mutation	SNP	C	G	G	FGFR3	c.970C>G	p.L324V
R18D6529R1	4	126373483	126373483	Missense_Mutation	SNP	A	T	T	FAT4	c.11312A>T	p.N3771I
R18D6529R1	4	153244293	153244293	Nonsense_Mutation	SNP	T	A	A	FBXW7	c.1864A>T	p.K622*
R18D6529R1	7	117199602	117199602	Missense_Mutation	SNP	C	G	G	CFTR	c.1477C>G	p.Q493E
R18D6529R1	7	141414215	141414215	Splice_Site	SNP	C	T	T	WEE2	c.539+10C>T	-
R18D6529R1	7	142651602	142651602	Missense_Mutation	SNP	C	G	G	KEL	c.685G>C	p.E229Q
R18D6531R1	14	45606403	45606403	Missense_Mutation	SNP	G	C	C	FANCM	c.640G>C	p.D214H
R18D6531R1	7	18688315	18688315	Splice_Site	SNP	C	T	T	HDAC9	c.1458+9C>T	-
R18D6531R1	7	42065851	42065851	Missense_Mutation	SNP	G	T	T	GLI3	c.1189C>A	p.P397T
R18D6531R1	X	66931262	66931262	Missense_Mutation	SNP	T	G	G	AR	c.1904T>G	p.L635R
R18D6542R1	1	16255703	16255703	Missense_Mutation	SNP	G	C	C	SPEN	c.2968G>C	p.D990H
R18D6542R1	1	16255796	16255796	Missense_Mutation	SNP	G	C	C	SPEN	c.3061G>C	p.D1021H
R18D6542R1	1	16255841	16255841	Missense_Mutation	SNP	G	C	C	SPEN	c.3106G>C	p.E1036Q
R18D6542R1	1	16458218	16458218	Missense_Mutation	SNP	C	T	T	EPHA2	c.2473G>A	p.E825K
R18D6542R1	11	108175420	108175420	Nonsense_Mutation	SNP	C	T	T	ATM	c.5515C>T	p.Q1839*
R18D6542R1	11	119170447	119170447	Missense_Mutation	SNP	C	T	T	CBL	c.2677C>T	p.R893W
R18D6542R1	17	7578191	7578191	Missense_Mutation	SNP	A	T	T	TP53	c.658T>A	p.Y220N
R18D6542R1	19	1220629	1220629	Missense_Mutation	SNP	C	T	T	STK11	c.647C>T	p.S216F
R18D6542R1	19	1220701	1220701	Missense_Mutation	SNP	C	T	T	STK11	c.719C>T	p.S240L
R18D6542R1	19	42795328	42795328	Missense_Mutation	SNP	A	G	G	CIC	c.2408A>G	p.N803S
R18D6542R1	2	178098960	178098960	Missense_Mutation	SNP	C	G	G	NFE2L2	c.85G>C	p.D29H
R18D6542R1	4	88957483	88957483	Missense_Mutation	SNP	C	T	T	PKD2	c.821C>T	p.S274F
R18D6542R1	4	187524189	187524189	Splice_Site	SNP	C	G	G	FAT1	c.11351-1G>C	-
R18D6542R1	7	140477831	140477831	Nonsense_Mutation	SNP	G	A	A	BRAF	c.1477C>T	p.Q493*
R18D6542R1	8	48856408	48856408	Splice_Site	SNP	C	G	G	PRKDC	c.808+5G>C	-
R18D6543R1	17	7574018	7574018	Missense_Mutation	SNP	G	A	A	TP53	c.1009C>T	p.R337C
R18D6543R1		-	-	Frame_Shift_Del	INS	-	-	-	KDM6A	c.2512_2515del	p.N839Vfs*27
R18D6543R1	X	66766006	66766006	Missense_Mutation	SNP	G	A	A	AR	c.1018G>A	p.E340K
R18D6640R1		-	-	Frame_Shift_Del	INS	-	-	-	TP53	c.870_876del	p.K291Gfs*52
R18D6640R1	18	48593391	48593391	Nonsense_Mutation	SNP	T	A	A	SMAD4	c.1142T>A	p.L381*
R18D6641R1	1	65312335	65312335	Missense_Mutation	SNP	C	G	G	JAK1	c.1984G>C	p.E662Q
R18D6641R1	10	114912095	114912095	Missense_Mutation	SNP	C	T	T	TCF7L2	c.1096C>T	p.H366Y
R18D6641R1	12	56491645	56491645	Missense_Mutation	SNP	G	T	T	ERBB3	c.2537G>T	p.S846I
R18D6641R1	17	7577085	7577085	Missense_Mutation	SNP	C	T	T	TP53	c.853G>A	p.E285K
R18D6641R1	17	41244447	41244447	Missense_Mutation	SNP	T	C	C	BRCA1	c.3101A>G	p.N1034S
R18D6641R1	18	48584497	48584497	Nonsense_Mutation	SNP	C	T	T	SMAD4	c.670C>T	p.Q224*
R18D6641R1	2	141143543	141143543	Missense_Mutation	SNP	T	C	C	LRP1B	c.10450A>G	p.K3484E
R18D6641R1	3	185183588	185183588	Missense_Mutation	SNP	A	G	G	MAP3K13	c.1442A>G	p.N481S
R18D6641R1	7	77764470	77764470	Missense_Mutation	SNP	C	T	T	MAGI2	c.2899G>A	p.A967T
R18D6641R1	7	142643377	142643377	Missense_Mutation	SNP	C	T	T	KEL	c.1231G>A	p.V411M
R18D6641R1	8	139153458	139153458	Missense_Mutation	SNP	C	A	A	FAM135B	c.3773G>T	p.S1258I
R18D6641R1	9	21971108	21971108	Missense_Mutation	SNP	C	T	T	CDKN2A	c.250G>A	p.D84N
R18D6641R1	X	66905905	66905905	Nonsense_Mutation	SNP	C	T	T	AR	c.1822C>T	p.R608*
R18D6641R1	X	70602478	70602478	Missense_Mutation	SNP	G	T	T	TAF1	c.1690G>T	p.G564W
R18D6643R1	11	108143267	108143267	Missense_Mutation	SNP	C	G	G	ATM	c.3086C>G	p.T1029R
R18D6643R1	12	25398281	25398281	Missense_Mutation	SNP	C	T	T	KRAS	c.38G>A	p.G13D
R18D6643R1	16	396649	396649	Missense_Mutation	SNP	T	G	G	AXIN1	c.377A>C	p.K126T
R18D6643R1	16	89837054	89837054	Splice_Site	SNP	C	G	G	FANCA	c.2152-12G>C	-
R18D6643R1	17	7578190	7578190	Missense_Mutation	SNP	T	C	C	TP53	c.659A>G	p.Y220C
R18D6643R1	18	48604821	48604821	Missense_Mutation	SNP	C	T	T	SMAD4	c.1643C>T	p.P548L
R18D6643R1	3	47164342	47164342	Nonsense_Mutation	SNP	G	C	C	SETD2	c.1784C>G	p.S595*
R18D6643R1	7	2984130	2984130	Missense_Mutation	SNP	C	T	T	CARD11	c.400G>A	p.E134K
R18D6643R1	7	86415650	86415650	Missense_Mutation	SNP	A	C	C	GRM3	c.542A>C	p.K181T
R18D6643R1	7	127714563	127714563	Missense_Mutation	SNP	G	A	A	SND1	c.1789G>A	p.E597K
R18D6643R1	9	21971111	21971111	Missense_Mutation	SNP	G	A	A	CDKN2A	c.247C>T	p.H83Y
R18D6713R1		-	-	In_Frame_Del	INS	-	-	-	ACVR1B	c.323_331del	p.108_111del
R18D6713R1	12	57578943	57578943	Missense_Mutation	SNP	G	A	A	LRP1	c.6418G>A	p.V2140I
R18D6713R1	17	7578266	7578266	Missense_Mutation	SNP	T	A	A	TP53	c.583A>T	p.I195F
R18D6713R1		-	-	Frame_Shift_Del	INS	-	-	-	TP53	C.277del	P.L93Cfs*30
R18D6713R1	2	141115559	141115559	Missense_Mutation	SNP	C	T	T	LRP1B	c.11384G>A	p.G3795E
R18D6713R1	20	57429235	57429235	Missense_Mutation	SNP	C	T	T	GNAS	c.728C>T	p.P243L
R18D6713R1	3	134670657	134670657	Missense_Mutation	SNP	C	T	T	EPHB1	c.568C>T	p.R190C
R18D6713R1	4	126370607	126370607	Missense_Mutation	SNP	G	A	A	FAT4	c.8436G>A	p.M2812I
R18D6713R1	4	187549458	187549458	Missense_Mutation	SNP	C	A	A	FAT1	c.4660G>T	p.D1554Y
R18D6713R1	6	36651988	36651988	Missense_Mutation	SNP	T	C	C	CDKN1A	c.110T>C	p.L37P
R18D6713R1	7	92354942	92354942	Missense_Mutation	SNP	C	A	A	CDK6	c.535G>T	p.V179L
R18D6713R1	7	141427152	141427152	Missense_Mutation	SNP	G	A	A	WEE2	c.1441G>A	p.A481T
R18D6713R1		-	-	Missense_Mutation	SNP	-	-	-	EZH2	024_1025delins	p.A342I

R18D6713R1	8	139380195	139380195	Missense_Mutation	SNP	G	A	A	FAM135B	c.32C>T	p.S11L
R18D6714R1	2	209113112	209113112	Missense_Mutation	SNP	C	A	A	IDH1	c.395G>T	p.R132L
R18D6714R1	3	52442594	52442594	Nonsense_Mutation	SNP	T	A	A	BAP1	c.151A>T	p.K51*
R18D6831R1	1	65330568	65330568	Missense_Mutation	SNP	G	A	A	JAK1	c.1078C>T	p.R360W
R18D6831R1	12	25398284	25398284	Missense_Mutation	SNP	C	T	T	KRAS	c.35G>A	p.G12D
R18D6831R1		-	-	In_Frame_Del	INS	-	-	-	TP53	c.844_849del	p.R282_R283del
R18D6831R1	3	185198046	185198046	Missense_Mutation	SNP	A	G	G	MAP3K13	c.2528A>G	p.Q843R
R18D6831R1	4	126372500	126372500	Missense_Mutation	SNP	C	A	A	FAT4	c.10329C>A	p.F3443L
R18D6831R1		-	-	Frame_Shift_Ins	INS	-	-	-	CDKN2A	c.225_243dup	p.V82Rfs*44
R18D6917S2	1	16202810	16202810	Missense_Mutation	SNP	C	T	T	SPEN	c.518C>T	p.P173L
R18D6917S2	10	43597849	43597849	Missense_Mutation	SNP	C	T	T	RET	c.397C>T	p.R133C
R18D6917S2	12	56482369	56482369	Missense_Mutation	SNP	C	G	G	ERBB3	c.917C>G	p.P306R
R18D6917S2	17	7577156	7577156	Splice_Site	SNP	C	A	A	TP53	c.783-1G>T	-
R18D6917S2	4	153247288	153247288	Missense_Mutation	SNP	C	T	T	FBXW7	c.1514G>A	p.R505H
R18D6917S2	8	68950401	68950401	Missense_Mutation	SNP	A	C	C	PREX2	c.713A>C	p.N238T
R18E7186R1	11	61562846	61562846	Missense_Mutation	SNP	G	A	A	FEN1	c.13G>A	p.G5S
R18E7186R1	17	7578542	7578542	Missense_Mutation	SNP	G	C	C	TP53	c.388C>G	p.L130V
R18E7186R1	2	141128790	141128790	Missense_Mutation	SNP	C	G	G	LRP1B	c.10833G>C	p.L3611F
R18E7186R1	3	89390090	89390090	Missense_Mutation	SNP	C	T	T	EPHA3	c.839C>T	p.A280V
R18E7186R1	4	153249385	153249385	Missense_Mutation	SNP	G	A	A	FBXW7	c.1393C>T	p.R465C
R18E7186R1	7	41739911	41739911	Missense_Mutation	SNP	G	T	T	INHBA	c.62C>A	p.S21Y
R18E7186R1	7	151873435	151873435	Nonsense_Mutation	SNP	G	A	A	KMT2C	c.9103C>T	p.Q3035*
R18F8364R1	13	28611311	28611311	Splice_Site	SNP	A	T	T	FLT3	c.1309+11T>A	-
R18F8364R1	16	68844245	68844245	Splice_Site	SNP	G	A	A	CDH1	c.832+1G>A	-
R18F8364R1	19	10602494	10602494	Missense_Mutation	SNP	G	A	A	KEAP1	c.1084C>T	p.R362W
R18F8364R1	3	178916890	178916890	Missense_Mutation	SNP	C	T	T	PIK3CA	c.277C>T	p.R93W
R18F8364R1	8	11615801	11615801	Splice_Site	SNP	G	T	T	GATA4	c.1147-1G>T	-
R18F8364R1	X	15574239	15574239	Missense_Mutation	SNP	T	C	C	BMX	c.1997T>C	p.I666T
R18F8364R1	X	47428427	47428427	Missense_Mutation	SNP	C	G	G	ARAF	c.1295C>G	p.S432C
R18F8368R1	1	8073694	8073694	Missense_Mutation	SNP	C	T	T	ERRFI1	c.965G>A	p.R322K
R18F8368R1	11	92531664	92531664	Missense_Mutation	SNP	A	T	T	FAT3	c.5485A>T	p.S1829C
R18F8368R1	15	93536086	93536086	Splice_Site	SNP	C	T	T	CHD2	c.3456-3C>T	-
R18F8368R1	16	3820729	3820729	Missense_Mutation	SNP	T	A	A	CREBBP	c.2722A>T	p.S908C
R18F8368R1	17	7577081	7577081	Missense_Mutation	SNP	T	A	A	TP53	c.857A>T	p.E286V
R18F8368R1	3	156396271	156396271	Missense_Mutation	SNP	C	G	G	TIPARP	c.785C>G	p.T262S
R18F8368R1	4	110897215	110897215	Missense_Mutation	SNP	C	G	G	EGF	c.1877C>G	p.S626C
R18F8368R1	4	143114256	143114256	Missense_Mutation	SNP	C	G	G	INPP4B	c.1165G>C	p.E389Q
R18F8368R1	6	117709028	117709028	Missense_Mutation	SNP	C	T	T	ROS1	c.1929G>A	p.M643I
R18F8368R1	7	127528043	127528043	Missense_Mutation	SNP	G	C	C	SND1	c.1432G>C	p.E478Q
R18F8368R1	7	128845240	128845240	Missense_Mutation	SNP	C	T	T	SMO	c.734C>T	p.T245M
R18F8368R1	7	151919153	151919153	Splice_Site	SNP	T	A	A	KMT2C	c.3434-2A>T	-
R18F8368R1	7	151960145	151960145	Nonsense_Mutation	SNP	G	A	A	KMT2C	c.1255C>T	p.Q419*
R18F8368R1	X	66943532	66943532	Missense_Mutation	SNP	C	T	T	AR	c.2612C>T	p.A871V
R18F8368R1	X	70602466	70602466	Missense_Mutation	SNP	C	G	G	TAF1	c.1678C>G	p.R560G
R18F8384R1	1	97564154	97564154	Missense_Mutation	SNP	C	T	T	DPYD	c.2657G>A	p.R886H
R18F8384R1		-	-	Frame_Shift_Del	INS	-	-	-	HRAS	C.169_188del	P.S57Mfs*2
R18F8384R1	12	52385761	52385761	Nonsense_Mutation	SNP	G	A	A	ACVR1B	c.1499G>A	p.W500*
R18F8384R1	17	7577523	7577523	Missense_Mutation	SNP	G	T	T	TP53	c.758C>A	p.T253N
R18F8384R1		-	-	Frame_Shift_Del	INS	-	-	-	TP53	c.726_730delins	p.M243Afs*3
R18F8384R1	2	109384335	109384335	Missense_Mutation	SNP	C	A	A	RANBP2	c.7340C>A	p.S2447Y
R18F8384R1	4	153259070	153259070	Missense_Mutation	SNP	T	C	C	FBXW7	c.745A>G	p.K249E
R18F8384R1	9	21971138	21971138	Missense_Mutation	SNP	C	T	T	CDKN2A	c.220G>A	p.D74N
R18F8385R1	7	151877808	151877808	Missense_Mutation	SNP	C	G	G	KMT2C	c.7137G>C	p.E2379D
R18F8390R1	1	11194405	11194405	Splice_Site	SNP	C	T	T	MTOR	c.5246+3G>A	-
R18F8390R1	1	158613182	158613182	Missense_Mutation	SNP	C	T	T	SPTA1	c.4372G>A	p.E1458K
R18F8390R1	12	46230533	46230533	Nonsense_Mutation	SNP	C	G	G	ARID2	c.782C>G	p.S261*
R18F8390R1	12	49420253	49420253	Missense_Mutation	SNP	C	A	A	KMT2D	c.15496G>T	p.A5166S
R18F8390R1	12	49422953	49422953	Missense_Mutation	SNP	G	C	C	KMT2D	c.14142C>G	p.I4714M
R18F8390R1	12	133252721	133252721	Missense_Mutation	SNP	C	T	T	POLE	c.979G>A	p.E327K
R18F8390R1	17	7577085	7577085	Missense_Mutation	SNP	C	T	T	TP53	c.853G>A	p.E285K
R18F8390R1	2	109347797	109347797	Missense_Mutation	SNP	C	G	G	RANBP2	c.272C>G	p.P91R
R18F8390R1	5	56111522	56111522	Missense_Mutation	SNP	C	T	T	MAP3K1	c.122C>T	p.A41V
R18F8390R1	7	142658153	142658153	Missense_Mutation	SNP	G	A	A	KEL	c.262C>T	p.H88Y
R18F8390R1	7	151919152	151919152	Splice_Site	SNP	C	G	G	KMT2C	c.3434-1G>C	-
R18F8390R1	8	37698769	37698769	Missense_Mutation	SNP	G	T	T	ADGRA2	c.2913G>T	p.E971D
R18F8390R1	9	133710845	133710845	Missense_Mutation	SNP	C	G	G	ABL1	c.12C>G	p.I4M
R18F8410R1	1	27092775	27092775	Missense_Mutation	SNP	A	T	T	ARID1A	c.2796A>T	p.Q932H
R18F8410R1	1	158647604	158647604	Missense_Mutation	SNP	T	A	A	SPTA1	c.833A>T	p.Q278L
R18F8410R1	10	84118611	84118611	Missense_Mutation	SNP	C	T	T	NRG3	c.940C>T	p.H314Y
R18F8410R1	16	56878455	56878455	Missense_Mutation	SNP	G	T	T	NUP93	c.2394G>T	p.M798I
R18F8410R1	17	7578416	7578416	Missense_Mutation	SNP	C	A	A	TP53	c.514G>T	p.V172F
R18F8410R1	3	195594503	195594503	Missense_Mutation	SNP	C	T	T	TNK2	c.2621G>A	p.R874H
R18F8410R1	7	81346572	81346572	Missense_Mutation	SNP	A	T	T	HGF	c.1381T>A	p.W461R
R18F8410R1	9	5066798	5066798	Splice_Site	SNP	G	A	A	JAK2	c.1326+9G>A	-
R18F8428R1	17	7577094	7577094	Missense_Mutation	SNP	G	A	A	TP53	c.844C>T	p.R282W
R18F8428R1	3	178921552	178921552	Missense_Mutation	SNP	A	C	C	PIK3CA	c.1034A>C	p.N345T
R18F8428R1	8	139323096	139323096	Missense_Mutation	SNP	C	T	T	FAM135B	c.145G>A	p.A49T
R18F8428R1	X	76778767	76778767	Missense_Mutation	SNP	C	G	G	ATRX	c.6812G>C	p.R2271T
R18F8429R1	1	27094291	27094291	Missense_Mutation	SNP	C	A	A	ARID1A	c.2999C>A	p.S1000Y
R18F8429R1	15	88727469	88727469	Missense_Mutation	SNP	C	T	T	NTRK3	c.310G>A	p.G104R
R18F8429R1	22	41566521	41566521	Missense_Mutation	SNP	G	C	C	EP300	c.4398G>C	p.W1466C
R18F8429R1	3	178952085	178952085	Missense_Mutation	SNP	A	G	G	PIK3CA	c.3140A>G	p.H1047R
R18F8432R1		-	-	Frame_Shift_Del	INS	-	-	-	PTEN	c.416_419del	p.L139Yfs*7
R18F8432R1	13	110437226	110437226	Missense_Mutation	SNP	G	C	C	IRS2	c.1175C>G	p.P392R
R18F8432R1	17	7578437	7578437	Nonsense_Mutation	SNP	G	A	A	TP53	c.493C>T	p.Q165*
R18F8432R1		-	-	In_Frame_Ins	INS	-	-	-	PREX2	c.2731dup	p.R911fKs*17
R18F8432R1	9	98209213	98209213	Missense_Mutation	SNP	C	A	A	PTCH1	c.4325G>T	p.R1442L
R18F8433R1	1	27106720	27106720	Missense_Mutation	SNP	G	A	A	ARID1A	c.6331G>A	p.V2111I
R18F8433R1	1	110459981	110459981	Missense_Mutation	SNP	C	T	T	CSF1	c.292C>T	p.R98C
R18F8433R1	14	105246551	105246551	Missense_Mutation	SNP	C	T	T	AKT1	c.49G>A	p.E17K
R18F8433R1	16	81969884	81969884	Missense_Mutation	SNP	G	A	A	PLCG2	c.2953G>A	p.V985I
R18F8433R1	16	89862356	89862356	Missense_Mutation	SNP	G	A	A	FANCA	c.964C>T	p.H322Y
R18F8433R1	17	7578443	7578443	Missense_Mutation	SNP	A	C	C	TP53	c.487T>G	p.Y163D
R18F8433R1	2	141093274	141093274	Missense_Mutation	SNP	T	G	G	LRP1B	c.12026A>C	p.N4009T
R18F8433R1	2	198265062	198265062	Missense_Mutation	SNP	G	A	A	SF3B1	c.2815C>T	p.R939C
R18F8433R1	3	70014337	70014337	Missense_Mutation	SNP	G	A	A	MITF	c.1198G>A	p.A400T
R18F8433R1	6	93979375	93979375	Nonsense_Mutation	SNP	G	A	A	EPHA7	c.1453C>T	p.Q485*
R18F8442R1	11	92616460	92616460	Missense_Mutation	SNP	G	A	A	FAT3	c.12838G>A	p.V4280M

R18F8442R1		-	-	Frame_Shift_Del	INS	-	-	-	TP53	C.267del	P.S90Pfs*33
R18F8442R1	2	178098957	178098957	Missense_Mutation	SNP	G	A	A	NFE2L2	c.88C>T	p.L30F
R18F8442R1	3	178927458	178927458	Missense_Mutation	SNP	C	G	G	PIK3CA	c.1221C>G	p.C407W
R18F8442R1	3	178951979	178951979	Missense_Mutation	SNP	G	C	C	PIK3CA	c.3034G>C	p.E1012Q
R18F8442R1	4	126241650	126241650	Missense_Mutation	SNP	A	G	G	FAT4	c.4084A>G	p.T1362A
R18F8442R1		-	-	Frame_Shift_Del	INS	-	-	-	RBM10	c.485del	p.M162Sfs*104
R18F8445R1	12	46243401	46243401	Missense_Mutation	SNP	A	G	G	ARID2	c.1754A>G	p.D585G
R18F8445R1	17	7578461	7578461	Missense_Mutation	SNP	C	A	A	TP53	c.469G>T	p.V157F
R18F8445R1	22	29696116	29696116	Nonsense_Mutation	SNP	G	T	T	EWSR1	c.1936G>T	p.E646*
R18F8636M2		-	-	In_Frame_Del	INS	-	-	-	EPHA2	c.1715_1738del	p.D573_E580del
R18F8636M2	1	16462239	16462239	Missense_Mutation	SNP	G	A	A	EPHA2	c.1339C>T	p.R447C
R18F8636M2		-	-	In_Frame_Del	INS	-	-	-	ARID1A	c.6177_6182del	2059_L2061delinsK
R18F8636M2		-	-	Frame_Shift_Ins	INS	-	-	-	KMT2D	c.4168dup	p.A1390Gfs*42
R18F8636M2	13	32914082	32914082	Missense_Mutation	SNP	G	A	A	BRCA2	c.5590G>A	p.D1864N
R18F8636M2	2	212812269	212812269	Missense_Mutation	SNP	G	A	A	ERBB4	c.307C>T	p.R103C
R18F8636M2	3	37067240	37067240	Missense_Mutation	SNP	T	A	A	MLH1	c.1151T>A	p.V384D
R18F8636M2	3	38182641	38182641	Missense_Mutation	SNP	T	C	C	MYD88	c.818T>C	p.L273P
R18F8636M2	4	187629096	187629096	Missense_Mutation	SNP	G	C	C	FAT1	c.1886C>G	p.S629W
R18F8636M2	7	18629980	18629980	Missense_Mutation	SNP	A	G	G	HDAC9	c.277A>G	p.I93V
R18F8636M2	7	140453154	140453154	Missense_Mutation	SNP	T	C	C	BRAF	c.1781A>G	p.D594G
R18F8636M2	7	141408627	141408627	Missense_Mutation	SNP	G	C	C	WEE2	c.69G>C	p.E23D
R18F8636M2	8	92982876	92982876	Splice_Site	SNP	G	C	C	RUNX1T1	c.1428+10C>G	-
R18F8636M2	9	5029776	5029776	Splice_Site	SNP	T	C	C	JAK2	c.227-7T>C	-
R18G9202R1	13	28602354	28602354	Missense_Mutation	SNP	C	T	T	FLT3	c.2014G>A	p.E672K
R18G9202R1	17	7577538	7577538	Missense_Mutation	SNP	C	T	T	TP53	c.743G>A	p.R248Q
R18G9202R1	17	41622708	41622708	Missense_Mutation	SNP	C	T	T	ETV4	c.88G>A	p.E30K
R18G9202R1	20	57484421	57484421	Missense_Mutation	SNP	G	A	A	GNAS	c.602G>A	p.R201H
R18G9202R1		-	-	Frame_Shift_Del	INS	-	-	-	CDKN2A	C.52_83del	P.T18Afs*15
R18G9202R1	X	47040995	47040995	Nonsense_Mutation	SNP	C	T	T	RBM10	c.1525C>T	p.Q509*
R18G9203R1	10	70406465	70406465	Nonsense_Mutation	SNP	G	T	T	TET1	c.3979G>T	p.E1327*
R18G9203R1	22	38379425	38379425	Missense_Mutation	SNP	C	T	T	SOX10	c.367G>A	p.A123T
R18G9203R1	4	187516839	187516839	Splice_Site	SNP	T	A	A	FAT1	c.13138+4A>T	-
R18G9203R1	9	139390917	139390917	Missense_Mutation	SNP	G	T	T	NOTCH1	c.7274C>A	p.A2425D
R18G9211R1	1	27106354	27106354	Nonsense_Mutation	SNP	C	T	T	ARID1A	c.5965C>T	p.R1989*
R18G9211R1	1	156844729	156844729	Missense_Mutation	SNP	C	T	T	NTRK1	c.1265C>T	p.A422V
R18G9211R1	10	123239466	123239466	Missense_Mutation	SNP	A	T	T	FGFR2	c.2371T>A	p.S791T
R18G9211R1	12	25398281	25398281	Missense_Mutation	SNP	C	T	T	KRAS	c.38G>A	p.G13D
R18G9211R1	17	7577124	7577124	Missense_Mutation	SNP	C	T	T	TP53	c.814G>A	p.V272M
R18G9211R1	17	41245859	41245859	Missense_Mutation	SNP	C	T	T	BRCA1	c.1689G>A	p.Q563Q
R18G9211R1		-	-	Frame_Shift_Ins	INS	-	-	-	SMAD4	c.692dup	p.S232Qfs*3
R18G9211R1	18	48593520	48593520	Missense_Mutation	SNP	A	T	T	SMAD4	c.1271A>T	p.D424V
R18G9211R1	19	33793251	33793251	Missense_Mutation	SNP	G	C	C	CEBPA	c.70C>G	p.H24D
R18G9211R1	7	151859696	151859696	Missense_Mutation	SNP	C	T	T	KMT2C	c.10966G>A	p.A3656T
R18G9211R1	X	123229283	123229283	Missense_Mutation	SNP	C	T	T	STAG2	c.3656C>T	p.S1219L
R18G9220R1	1	16475487	16475487	Missense_Mutation	SNP	C	T	T	EPHA2	c.209G>A	p.C70Y
R18G9220R1		-	-	Frame_Shift_Del	INS	-	-	-	ARID1A	c.836_873del	p.P279Hfs*108
R18G9220R1		-	-	Frame_Shift_Ins	INS	-	-	-	BRCA2	c.8021dup	p.I2675Dfs*6
R18G9220R1	17	78831689	78831689	Missense_Mutation	SNP	G	T	T	RPTOR	c.1498G>T	p.A500S
R18G9220R1	18	48591856	48591856	Missense_Mutation	SNP	A	C	C	SMAD4	c.1019A>C	p.K340T
R18G9220R1	3	191888367	191888367	Missense_Mutation	SNP	G	T	T	FGF12	c.307C>A	p.R103S
R18G9220R1	4	55972050	55972050	Missense_Mutation	SNP	C	T	T	KDR	c.1594G>A	p.A532T
R18G9220R1	X	47044604	47044604	Splice_Site	SNP	G	T	T	RBM10	c.2100+1G>T	-
R18G9241R1		-	-	In_Frame_Del	INS	-	-	-	ARID2	c.3437_3445del	p.S1146*
R18G9241R1	2	112722842	112722842	Missense_Mutation	SNP	A	T	T	MERTK	c.832A>T	p.I278F
R18G9241R1		-	-	Frame_Shift_Del	INS	-	-	-	ASXL1	c.1797del	p.T600Pfs*103
R18G9243R1		-	-	Frame_Shift_Ins	INS	-	-	-	SMARCD1	c.207_210dup	p.P71Dfs*61
R18G9243R1		-	-	In_Frame_Ins	INS	-	-	-	TP53	c.155_158dup	p.W53*
R18G9243R1	17	38559194	38559194	Missense_Mutation	SNP	T	C	C	TOP2A	c.2396A>G	p.D799G
R18G9244R1	12	115114110	115114110	Splice_Site	SNP	T	C	C	TBX3	c.1099+8A>G	-
R18G9244R1	2	74320789	74320789	Missense_Mutation	SNP	C	T	T	TET3	c.3263C>T	p.T1088I
R18G9244R1	2	170062706	170062706	Splice_Site	SNP	C	G	G	LRP2	c.7391-8G>C	-
R18G9244R1	7	77756686	77756686	Missense_Mutation	SNP	C	T	T	MAGI2	c.3251G>A	p.R1084Q
R18G9244R1	7	117254762	117254762	Missense_Mutation	SNP	A	T	T	CFTR	c.3463A>T	p.S1155C
R18G9244R1	8	37691533	37691533	Nonsense_Mutation	SNP	G	T	T	ADGRA2	c.1495G>T	p.E499*
R18G9244R1		-	-	Missense_Mutation	SNP	-	-	-	RUNX1T1	c.183_185del	p.V62del
R18G9244R1	9	5534820	5534820	Missense_Mutation	SNP	T	C	C	PDCD1LG2	c.131T>C	p.F44S
R18G9245R1	1	158605742	158605742	Missense_Mutation	SNP	A	G	G	SPTA1	c.5393T>C	p.V1798A
R18G9245R1	11	92534385	92534385	Nonsense_Mutation	SNP	C	T	T	FAT3	c.8206C>T	p.Q2736*
R18G9245R1	12	56481922	56481922	Missense_Mutation	SNP	G	A	A	ERBB3	c.850G>A	p.G284R
R18G9245R1	17	7577538	7577538	Missense_Mutation	SNP	C	T	T	TP53	c.743G>A	p.R248Q
R18G9245R1		-	-	Frame_Shift_Del	INS	-	-	-	MAP2K4	C.70_86del	P.V24Rfs*11
R18G9245R1	19	2226538	2226538	Missense_Mutation	SNP	A	C	C	DOT1L	c.4018A>C	p.K1340Q
R18G9245R1	9	5069196	5069196	Missense_Mutation	SNP	C	A	A	JAK2	c.1501C>A	p.P501T
R18G9245R1	X	44733234	44733234	Splice_Site	SNP	G	T	T	KDM6A	c.225+1G>T	-
R18G9249R1	1	32741220	32741220	Missense_Mutation	SNP	C	A	A	LCK	c.428C>A	p.A143E
R18G9249R1	13	28891687	28891687	Missense_Mutation	SNP	G	A	A	FLT1	c.3334C>T	p.R1112C
R18G9249R1	16	9862896	9862896	Nonsense_Mutation	SNP	C	A	A	GRIN2A	c.2407G>T	p.E803*
R18G9249R1		-	-	Frame_Shift_Del	INS	-	-	-	TP53	C.997del	P.R333Vfs*12
R18G9249R1	2	48030799	48030799	Missense_Mutation	SNP	G	A	A	MSH6	c.3413G>A	p.G1138E
R18G9249R1	7	127714681	127714681	Missense_Mutation	SNP	G	A	A	SND1	c.1907G>A	p.R636H
R18G9249R1	7	151880109	151880109	Missense_Mutation	SNP	G	T	T	KMT2C	c.5215C>A	p.P1739T
R18G9249R1	8	32463111	32463111	Missense_Mutation	SNP	C	T	T	NRG1	c.955C>T	p.L319L
R18G9250R1	1	51436086	51436086	Missense_Mutation	SNP	G	T	T	CDKN2C	c.46G>T	p.G16W
R18G9250R1	12	1039231	1039231	Missense_Mutation	SNP	G	A	A	RAD52	c.266C>T	p.T89M
R18G9250R1	12	56478854	56478854	Missense_Mutation	SNP	G	T	T	ERBB3	c.310G>T	p.V104L
R18G9250R1	17	7577121	7577121	Missense_Mutation	SNP	G	A	A	TP53	c.817C>T	p.R273C
R18G9250R1	17	37879658	37879658	Missense_Mutation	SNP	G	A	A	ERBB2	c.2033G>A	p.R678Q
R18G9250R1		-	-	Frame_Shift_Ins	INS	-	-	-	MSH6	c.2548dup	p.Y850Lfs*8
R18G9250R1	2	234668955	234668955	Missense_Mutation	SNP	G	A	A	UGT1A1	c.22G>A	p.G8R
R18G9250R1	3	52668619	52668619	Nonsense_Mutation	SNP	G	A	A	PBRM1	c.1300C>T	p.R434*
R18G9250R1	6	30866038	30866038	Missense_Mutation	SNP	G	A	A	DDR1	c.2294G>A	p.R765Q
R18G9250R1		-	-	Frame_Shift_Del	INS	-	-	-	MAGI2	c.1243del	p.R415Gfs*6
R18G9250R1		-	-	Frame_Shift_Del	INS	-	-	-	PRKDC	c.3179del	p.F1060Sfs*46
R18G9250R1	X	47044541	47044541	Nonsense_Mutation	SNP	C	T	T	RBM10	c.2038C>T	p.R680*
R18G9251R1		-	-	Frame_Shift_Del	INS	-	-	-	SRC	c.1560_1573del	p.E520Dfs*51
R18G9251R1		-	-	Frame_Shift_Del	INS	-	-	-	LZTR1	c.2412del	p.K805Sfs*8
R18G9255R1	11	92600168	92600168	Missense_Mutation	SNP	C	T	T	FAT3	c.11920C>T	p.R3974W
R18G9255R1	17	7578190	7578190	Missense_Mutation	SNP	T	C	C	TP53	c.659A>G	p.Y220C

R18G9255R1	17	7579359	7579359	Missense_Mutation	SNP	G	A	A	TP53	c.328C>T	p.R110C
R18G9255R1	17	37619346	37619346	Missense_Mutation	SNP	C	G	G	CDK12	c.1022C>G	p.S341C
R18G9255R1	18	42530035	42530035	Missense_Mutation	SNP	G	A	A	SETBP1	c.730G>A	p.E244K
R18G9255R1	20	31016158	31016158	Missense_Mutation	SNP	C	A	A	ASXL1	c.404C>A	p.S135Y
R18G9255R1	3	89259506	89259506	Missense_Mutation	SNP	C	G	G	EPHA3	c.650C>G	p.P217R
R18G9255R1	8	48748975	48748975	Nonsense_Mutation	SNP	G	C	C	PRKDC	c.7874C>G	p.S2625*
R18G9255R1	8	48749017	48749017	Missense_Mutation	SNP	G	A	A	PRKDC	c.7832C>T	p.S2611F
R18G9257R1	11	108153578	108153578	Missense_Mutation	SNP	A	C	C	ATM	c.3718A>C	p.N1240H
R18G9257R1	13	32968898	32968898	Missense_Mutation	SNP	A	T	T	BRCA2	c.9329A>T	p.N3110I
R18G9257R1	17	47699369	47699369	Missense_Mutation	SNP	C	T	T	SPOP	c.139G>A	p.E47K
R18G9257R1	19	1218486	1218486	Nonsense_Mutation	SNP	G	T	T	STK11	c.361G>T	p.E121*
R18G9257R1	22	21347167	21347167	Missense_Mutation	SNP	C	T	T	LZTR1	c.1234C>T	p.R412C
R18G9258R1	13	108922466	108922466	Missense_Mutation	SNP	G	A	A	TNFSF13B	c.223G>A	p.D75N
R18G9258R1		-	-	Frame_Shift_Del	INS	-	-	-	TP53	.420_428delins	p.C141Afs*5
R18G9258R1	6	157528883	157528883	Missense_Mutation	SNP	T	C	C	ARID1B	c.6608T>C	p.L2203P
R18G9258R1	9	101908864	101908864	Nonsense_Mutation	SNP	G	T	T	TGFBR1	c.1228G>T	p.E410*
R18G9465R1	11	108200967	108200967	Missense_Mutation	SNP	T	C	C	ATM	c.7334T>C	p.L2445P
R18G9465R1	12	56478854	56478854	Missense_Mutation	SNP	G	T	T	ERBB3	c.310G>T	p.V104L
R18G9465R1	12	56487218	56487218	Missense_Mutation	SNP	A	G	G	ERBB3	c.1364A>G	p.Y455C
R18G9465R1		-	-	In_Frame_Ins	INS	-	-	-	AXIN1	c.345_350dup	.L115_D116dup
R18G9465R1		-	-	Frame_Shift_Ins	INS	-	-	-	SMAD4	c.332dup	p.H111Qfs*3
R18G9465R1	19	11135058	11135058	Missense_Mutation	SNP	C	T	T	SMARCA4	c.3025C>T	p.R1009C
R18G9465R1	7	140453134	140453134	Missense_Mutation	SNP	T	C	C	BRAF	c.1801A>G	p.K601E
R18G9465R1	9	36846874	36846874	Missense_Mutation	SNP	G	A	A	PAX5	c.833C>T	p.T278M
R18G9465R1	9	87570217	87570217	Missense_Mutation	SNP	G	A	A	NTRK2	c.1957G>A	p.V653M
R18G9477R1	15	99192881	99192881	Missense_Mutation	SNP	C	T	T	IGF1R	c.71C>T	p.S24L
R18G9477R1	17	7579312	7579312	Missense_Mutation	SNP	C	A	A	TP53	c.375G>T	p.T125T
R18G9477R1	3	89521695	89521695	Missense_Mutation	SNP	G	T	T	EPHA3	c.2772G>T	p.W924C
R18G9477R1	3	178922324	178922324	Missense_Mutation	SNP	G	A	A	PIK3CA	c.1093G>A	p.E365K
R18G9477R1	6	33286547	33286547	Missense_Mutation	SNP	C	G	G	DAXX	c.2196G>C	p.E732D
R18G9477R1	X	53222304	53222304	Missense_Mutation	SNP	G	C	C	KDM5C	c.4528C>G	p.P1510A
R18G9484R1	17	7577153	7577153	Missense_Mutation	SNP	C	A	A	TP53	c.785G>T	p.G262V
R18G9499R1	11	118343871	118343871	Missense_Mutation	SNP	G	C	C	KMT2A	c.1997G>C	p.G666A
R18G9499R1	19	17954626	17954626	Missense_Mutation	SNP	C	T	T	JAK3	c.268G>A	p.V90M
R18G9499R1	3	142217458	142217458	Missense_Mutation	SNP	C	T	T	ATR	c.5539G>A	p.G1847R
R18G9499R1	5	156671391	156671391	Missense_Mutation	SNP	G	A	A	ITK	c.1352G>A	p.R451Q
R18G9499R1	6	36652246	36652246	Missense_Mutation	SNP	C	T	T	CDKN1A	c.368C>T	p.S123L
R18G9499R1	7	19035653	19035653	Missense_Mutation	SNP	G	T	T	HDAC9	c.3178G>T	p.G1060C
R18G9501R1		-	-	Frame_Shift_Ins	INS	-	-	-	ARID1A	c.6706dup	p.R2236Pfs*42
R18G9501R1	1	158637816	158637816	Missense_Mutation	SNP	G	C	C	SPTA1	c.1870C>G	p.Q624E
R18G9501R1		-	-	Missense_Mutation	SNP	-	-	-	CCND1	c.683_685del	p.R228del
R18G9501R1		-	-	Frame_Shift_Del	INS	-	-	-	CREBBP	c.3317_3329del	p.P1106Lfs*6
R18G9501R1		-	-	Splice_Site	SNP	-	-	-	NCOR1	242+5_242+6del	-
R18G9501R1	17	38560458	38560458	Missense_Mutation	SNP	C	A	A	TOP2A	c.2229G>T	p.K743N
R18G9501R1		-	-	Frame_Shift_Ins	INS	-	-	-	TOP2A	c.214dup	p.Y72Lfs*2
R18G9501R1	2	11389904	11389904	Missense_Mutation	SNP	C	G	G	ROCK2	c.345G>C	p.Q115H
R18G9501R1		-	-	Missense_Mutation	SNP	-	-	-	BCL2L1	c.351_353del	p.T118del
R18G9501R1		-	-	Frame_Shift_Ins	INS	-	-	-	ROS1	C.1720_1721dup	P.V575Lfs*11
R18G9501R1	7	151947971	151947971	Missense_Mutation	SNP	C	T	T	KMT2C	c.1702G>A	p.G568S
R18G9503R1	12	25398284	25398284	Missense_Mutation	SNP	C	G	G	KRAS	c.35G>C	p.G12A
R18G9503R1	17	7574017	7574017	Missense_Mutation	SNP	C	G	G	TP53	c.1010G>C	p.R337P
R18G9503R1		-	-	Frame_Shift_Del	INS	-	-	-	SOX9	C.724_740del	P.K242Gfs*4
R18G9503R1	9	101900191	101900191	Nonsense_Mutation	SNP	G	T	T	TGFBR1	c.625G>T	p.E209*
R18G9504R1		-	-	Frame_Shift_Ins	INS	-	-	-	JAK1	C.1289dup	P.L431Vfs*22
R18G9504R1	1	193119422	193119422	Splice_Site	SNP	A	T	T	CDC73	c.829-12A>T	-
R18G9504R1	10	70446225	70446225	Missense_Mutation	SNP	A	C	C	TET1	c.5165A>C	p.K1722T
R18G9504R1	12	52387796	52387796	Nonsense_Mutation	SNP	C	T	T	ACVR1B	c.1543C>T	p.R515*
R18G9504R1	14	23778068	23778068	Missense_Mutation	SNP	C	T	T	BCL2L2	c.476C>T	p.A159V
R18G9504R1	16	3777750	3777750	Missense_Mutation	SNP	T	C	C	CREBBP	c.7298A>G	p.D2433G
R18G9504R1	17	7577121	7577121	Missense_Mutation	SNP	G	A	A	TP53	c.817C>T	p.R273C
R18G9504R1	17	29670031	29670031	Missense_Mutation	SNP	C	A	A	NF1	c.7067C>A	p.P2356Q
R18G9504R1	19	10473296	10473296	Missense_Mutation	SNP	C	T	T	TYK2	c.1405G>A	p.G469S
R18G9504R1	19	40761125	40761125	Missense_Mutation	SNP	C	T	T	AKT2	c.227G>A	p.R76H
R18G9504R1	2	29473967	29473967	Splice_Site	SNP	G	A	A	ALK	c.2204+4C>T	-
R18G9504R1	2	141122224	141122224	Splice_Site	SNP	C	T	T	LRP1B	c.11131+6G>A	-
R18G9504R1		-	-	Splice_Site	SNP	-	-	-	LRP1B	c.9964-4dup	-
R18G9504R1	3	12650322	12650322	Missense_Mutation	SNP	T	C	C	RAF1	c.524A>G	p.H175R
R18G9504R1	3	37035145	37035145	Missense_Mutation	SNP	T	G	G	MLH1	c.107T>G	p.I36S
R18G9504R1	3	134644703	134644703	Missense_Mutation	SNP	C	T	T	EPHB1	c.104C>T	p.T35M
R18G9504R1		-	-	Splice_Site	SNP	-	-	-	SLIT2	c.1833-9dup	-
R18G9504R1	4	66280156	66280156	Missense_Mutation	SNP	T	G	G	EPHA5	c.1533A>C	p.Q511H
R18G9504R1	7	117307044	117307044	Missense_Mutation	SNP	G	T	T	CFTR	c.4325G>T	p.S1442I
R18G9504R1		-	-	Missense_Mutation	SNP	-	-	-	CDKN2A	C.192_194del	P.L65del
R18G9508R1	12	25398284	25398284	Missense_Mutation	SNP	C	G	G	KRAS	c.35G>C	p.G12A
R18G9509R1	1	27089676	27089676	Nonsense_Mutation	SNP	C	T	T	ARID1A	c.2632C>T	p.Q878*
R18G9509R1	17	7577099	7577099	Missense_Mutation	SNP	C	T	T	TP53	c.839G>A	p.R280K
R18G9509R1	9	87342612	87342612	Missense_Mutation	SNP	C	A	A	NTRK2	c.897C>A	p.H299Q
R18H2220M1	1	11186709	11186709	Nonsense_Mutation	SNP	T	A	A	MTOR	c.6496A>T	p.K2166*
R18H2220M1	17	7578202	7578202	Missense_Mutation	SNP	A	C	C	TP53	c.647T>G	p.V216G
R18H2220M1	18	48603032	48603032	Nonsense_Mutation	SNP	C	T	T	SMAD4	c.1333C>T	p.R445*
R18H2220M1	6	138196907	138196907	Missense_Mutation	SNP	C	T	T	TNFAIP3	c.569C>T	p.S190L
R18H2220M1	7	148514980	148514980	Missense_Mutation	SNP	G	A	A	EZH2	c.1229C>T	p.S410L
R18H2220M1	X	129149695	129149695	Nonsense_Mutation	SNP	A	T	T	BCORL1	c.2947A>T	p.K983*
SH_01	1	162725527	162725527	Silent	SNV	T	C	C	DDR2	c.639T>C	P.N213N
SH_01	1	186099838	186099838	Splice_Site	SNV	C	T	T	HMCN1	c.13230+9C>T	
SH_01	1	186862271	186862271	Missense_Mutation	SNV	A	T	T	PLA2G4A	c.244A>T	P.N82Y
SH_01	1	201060930	201060930	Splice_Site	SNV	C	A	A	CACNA1S	c.542-10G>T	
SH_01	1	248343682	248343682	Missense_Mutation	SNV	A	G	G	OR2M2	c.395A>G	P.Y132C
SH_01	1	27277241	27277241	Frame_Shift_Del	INDEL	G	-	-	KDF1	c.1046delC	P.T349fs
SH_01	11	621598	621598	Silent	SNV	G	A	A	CDHR5	c.471C>T	P.D157D
SH_01	12	58113926	58113926	Missense_Mutation	SNV	C	G	G	OS9	c.1645C>G	P.R549G
SH_01	13	100207882	100207882	Missense_Mutation	SNV	A	G	G	TM9SF2	c.1733A>G	P.Y578C
SH_01	14	90767602	90767602	Missense_Mutation	SNV	T	A	A	NRDE2	c.1526A>T	P.D509V
SH_01	15	85406805	85406805	Missense_Mutation	SNV	G	A	A	ALPK3	c.5039G>A	P.S1680N
SH_01	19	17085966	17085966	Missense_Mutation	SNV	G	A	A	CPAMD8	c.2152C>T	P.R718C
SH_01	19	55241136	55241136	Missense_Mutation	SNV	C	A	A	KIR3DL3	c.833C>A	P.A278D
SH_01	2	61441650	61441650	Missense_Mutation	SNV	C	G	G	USP34	c.8227G>C	P.D2743H
SH_01	3	143185925	143185925	Missense_Mutation	SNV	C	T	T	SLC9A9	c.1423G>A	P.V475I

SH_01	5	41067320	41067320	Missense_Mutation	SNV	C	G	G	MROH2B	c.91G>C	P.E31Q
SH_01	6	33288240	33288240	Missense_Mutation	SNV	C	T	T	DAXX	c.1168G>A	P.E390K
SH_01	7	38803062	38803062	Missense_Mutation	SNV	T	A	A	VPS41	c.1415A>T	P.D472V
SH_01	8	141262967	141262967	Frame_Shift_Ins	INDEL	-	A	A	TRAPPC9	c.2339dupT	P.L780fs
SH_02	19	10433378	10433378	Silent	SNV	A	G	G	RAVER1	c.1167T>C	P.N389N
SH_02	19	9069847	9069847	Missense_Mutation	SNV	G	A	A	MUC16	c.17599C>T	P.P5867S
SH_02	2	216286937	216286937	Missense_Mutation	SNV	C	A	A	FN1	c.1423G>T	P.G475W
SH_03	1	10471591	10471591	Silent	SNV	G	A	A	PGD	c.570G>A	P.A190A
SH_03	1	29344901	29344901	Silent	SNV	G	A	A	EPB41	c.1071G>A	P.P357P
SH_03	10	11527879	11527879	Silent	SNV	C	T	T	USP6NL	c.747G>A	P.L249L
SH_03	10	117093839	117093839	Missense_Mutation	SNV	G	A	A	ATRNL1	c.3085G>A	P.E1029K
SH_03	10	93252175	93252175	Missense_Mutation	SNV	G	A	A	HECTD2	c.1378G>A	P.D460N
SH_03	11	58275454	58275454	Missense_Mutation	SNV	C	T	T	OR5B21	c.125G>A	P.G42E
SH_03	12	21377711	21377711	Missense_Mutation	SNV	G	T	T	SLCO1B1	c.1803G>T	P.K601N
SH_03	12	25398284	25398284	Missense_Mutation	SNV	C	T	T	KRAS	c.35G>A	P.G12D
SH_03	13	103385224	103385224	Silent	SNV	G	A	A	CCDC168	c.17823C>T	P.D5941D
SH_03	18	48591930	48591930	Missense_Mutation	SNV	G	A	A	SMAD4	c.1093G>A	P.G365S
SH_03	4	8477600	8477600	Missense_Mutation	SNV	A	C	C	TRMT44	c.2146A>C	P.T716P
SH_03	5	13792115	13792115	Missense_Mutation	SNV	G	C	C	DNAH5	c.8436C>G	P.I2812M
SH_05	1	15970017	15970017	Missense_Mutation	SNV	G	T	T	DDI2	c.761G>T	P.G254V
SH_05	12	4870167	4870167	Missense_Mutation	SNV	G	A	A	GALNT8	c.1217G>A	P.R406Q
SH_05	12	7973877	7973877	Missense_Mutation	SNV	T	A	A	SLC2A14	c.978A>T	P.Q326H
SH_05	14	88654388	88654388	Missense_Mutation	SNV	C	T	T	KCNK10	c.919G>A	P.G307S
SH_05	15	24923954	24923954	Silent	SNV	T	A	A	NPAP1	c.2940T>A	P.S980S
SH_05	16	14947821	14947821	Missense_Mutation	SNV	A	G	G	NOMO1	c.901A>G	P.T301A
SH_05	17	39240887	39240887	Silent	SNV	C	G	G	KRTAP4-7	c.429C>G	P.T143T
SH_05	17	41907409	41907409	Missense_Mutation	SNV	T	G	G	MPP3	c.289A>C	P.T97P
SH_05	17	7577568	7577568	Missense_Mutation	SNV	C	A	A	TP53	c.713G>T	P.C238F
SH_05	19	51334742	51334742	Missense_Mutation	SNV	G	T	T	KLK15	c.7C>A	P.L3I
SH_05	7	20420460	20420460	Splice_Site	SNV	A	G	G	ITGB8	c.801+6A>G	
SH_05	7	57188582	57188582	Silent	SNV	A	T	T	ZNF479	c.540T>A	P.T180T
SH_05	7	80447713	80447713	Missense_Mutation	SNV	G	T	T	SEMA3C	c.349C>A	P.R117S
SH_05	8	26627987	26627987	Silent	SNV	C	A	A	ADRA1A	c.1080G>T	P.L360L
SH_05	8	59409434	59409434	Missense_Mutation	SNV	C	T	T	CYP7A1	c.637G>A	P.D213N
SH_05	X	129499646	129499646	Nonsense_Mutation	SNV	T	A	A	SLC25A14	c.851T>A	P.L284*
SH_05	X	74649819	74649819	Splice_Site	SNV	C	T	T	ZDHHC15	c.423-4G>A	
SH_05	X	91131848	91131848	Silent	SNV	T	A	A	PCDH11X	c.609T>A	P.V203V
SH_05	X	9866272	9866272	Nonsense_Mutation	SNV	G	T	T	SHROOM2	c.2833G>T	P.E945*
SH_06	1	5925295	5925295	Missense_Mutation	SNV	A	C	C	NPHP4	c.2144T>G	P.V715G
SH_06	18	12463443	12463443	Silent	SNV	C	T	T	SPIRE1	c.1545G>A	P.R515R
SH_06	19	43967775	43967775	Missense_Mutation	SNV	T	G	G	LYPD3	c.358A>C	P.T120P
SH_06	2	179517003	179517003	Missense_Mutation	SNV	G	A	A	TTN	c.34678C>T	P.P11560S
SH_07	12	25398284	25398284	Missense_Mutation	SNV	C	TRUE	TRUE	KRAS	c.35G>A	P.G12D
SH_08	1	248129634	248129634	Missense_Mutation	SNV	T	A	A	OR2AK2	c.1001T>A	P.L334Q
SH_08	1	26152814	26152814	Frame_Shift_Ins	INDEL	-	TG	TG	MTFR1L	:.155_156dupG1	P.L53fs
SH_08	1	34667819	34667819	Missense_Mutation	SNV	C	G	G	C1orf94	c.1405C>G	P.P469A
SH_08	1	68947861	68947861	Missense_Mutation	SNV	T	A	A	DEPDC1	c.1630A>T	P.S544C
SH_08	1	94940756	94940756	Nonsense_Mutation	SNV	C	T	T	ABCD3	c.463C>T	P.R155*
SH_08	11	26558957	26558957	Silent	SNV	C	T	T	ANO3	c.1164C>T	P.I388I
SH_08	11	4144502	4144502	Missense_Mutation	SNV	C	T	T	RRM1	c.1208C>T	P.P403L
SH_08	11	4790634	4790634	Missense_Mutation	SNV	T	C	C	OR51F1	c.514A>G	P.R172G
SH_08	11	49974335	49974335	Missense_Mutation	SNV	T	A	A	OR4C13	c.361T>A	P.Y121N
SH_08	11	5510223	5510223	Missense_Mutation	SNV	C	T	T	OR52D1	c.287C>T	P.S96F
SH_08	12	124358269	124358269	Splice_Site	SNV	C	A	A	DNAH10	c.7584+12C>A	
SH_08	12	62785633	62785633	Frame_Shift_Ins	INDEL	-	A	A	USP15	c.2278dupA	P.R760fs
SH_08	13	103395589	103395589	Silent	SNV	T	G	G	CCDC168	c.7458A>C	P.I2486I
SH_08	15	55929382	55929382	Missense_Mutation	SNV	A	C	C	PRTG	c.2609T>G	P.V870G
SH_08	18	2599075	2599075	Nonsense_Mutation	SNV	A	T	T	NDC80	c.1279A>T	P.K427*
SH_08	18	63476982	63476982	Missense_Mutation	SNV	A	T	T	CDH7	c.253A>T	P.I85F
SH_08	19	53086139	53086139	Missense_Mutation	SNV	G	T	T	ZNF701	c.1025G>T	P.G342V
SH_08	19	54760159	54760159	Silent	SNV	C	A	A	LILRB5	c.402G>T	P.V134V
SH_08	19	58198693	58198693	Silent	SNV	C	A	A	ZNF551	c.966C>A	P.L322L
SH_08	19	9068836	9068836	Missense_Mutation	SNV	G	A	A	MUC16	c.18610C>T	P.L6204F
SH_08	19	9091198	9091198	Missense_Mutation	SNV	G	A	A	MUC16	c.617C>T	P.A206V
SH_08	2	152426737	152426737	Missense_Mutation	SNV	T	A	A	NEB	c.17288A>T	P.D5763V
SH_08	2	182347092	182347092	Silent	SNV	T	C	C	ITGA4	c.846T>C	P.Y282Y
SH_08	2	207452041	207452041	Splice_Site	SNV	T	G	G	ADAM23	c.1738-8T>G	
SH_08	2	55812299	55812299	Missense_Mutation	SNV	A	G	G	PPP4R3B	c.1121T>C	P.M374T
SH_08	21	14983010	14983010	Missense_Mutation	SNV	G	C	C	POTED	c.461G>C	P.R154T
SH_08	21	17135205	17135205	Splice_Site	SNV	T	C	C	USP25	c.46-5T>C	
SH_08	3	121438521	121438521	Missense_Mutation	SNV	T	A	A	GOLGB1	c.743A>T	P.Q248L
SH_08	3	193130046	193130046	Missense_Mutation	SNV	G	C	C	ATP13A4	c.3129C>G	P.F1043L
SH_08	4	144618702	144618702	Missense_Mutation	SNV	A	T	T	FREM3	c.3127T>A	P.S1043T
SH_08	4	15826591	15826591	Missense_Mutation	SNV	G	T	T	CD38	c.451G>T	P.G151C
SH_08	4	165118501	165118501	Silent	SNV	T	G	G	ANP32C	c.363A>C	P.V121V
SH_08	5	180657785	180657785	Silent	SNV	G	T	T	TRIM41	c.855G>T	P.L285L
SH_08	6	147106755	147106755	Missense_Mutation	SNV	G	C	C	ADGB	c.4222G>C	P.A1408P
SH_08	7	102743935	102743935	Missense_Mutation	SNV	A	G	G	NAPEPLD	c.1123T>C	P.F375L
SH_08	7	117876942	117876942	Nonsense_Mutation	SNV	C	A	A	ANKRD7	c.674C>A	P.S225*
SH_08	7	143632711	143632711	Missense_Mutation	SNV	G	A	A	OR2F2	c.386G>A	P.R129H
SH_08	7	84628984	84628984	Silent	SNV	G	T	T	SEMA3D	c.2106C>A	P.V702V
SH_08	8	67067878	67067878	Silent	SNV	G	C	C	TRIM55	c.1545G>C	P.A515A
SH_08	8	71075095	71075095	Splice_Site	SNV	A	C	C	NCOA2	c.833-6T>G	
SH_08	X	101821163	101821163	Splice_Site	SNV	A	T	T	NXF4	n.1363A>T	
SH_08	X	105125700	105125700	Splice_Site	SNV	G	T	T	NRK	c.181-1G>T	
SH_09	1	120930078	120930078	Missense_Mutation	SNV	G	A	A	FCGR1B	c.247C>T	P.R83C
SH_09	1	24301546	24301546	Splice_Site	SNV	A	G	G	SRSF10	n.802-5T>C	
SH_09	1	33330385	33330385	Silent	SNV	G	C	C	FNDC5	c.513C>G	P.L171L
SH_09	1	75218038	75218038	Silent	SNV	A	G	G	TYW3	c.390A>G	P.T130T
SH_09	10	25861694	25861694	Nonsense_Mutation	SNV	G	A	A	GPR158	c.1631G>A	P.W544*
SH_09	11	4976165	4976165	Missense_Mutation	SNV	G	A	A	OR51A2	c.779C>T	P.A260V
SH_09	13	111077183	111077183	Frame_Shift_Del	INDEL	G	-	-	COL4A2	c.283delG	P.A95fs
SH_09	13	42877460	42877460	Silent	SNV	T	C	C	AKAP11	c.4578T>C	P.S1526S
SH_09	2	141250202	141250202	Missense_Mutation	SNV	C	A	A	LRP1B	c.9095G>T	P.G3032V
SH_09	20	34430626	34430626	Missense_Mutation	SNV	A	T	T	PHF20	c.215A>T	P.Q72L
SH_09	4	95191958	95191958	Missense_Mutation	SNV	G	A	A	SMARCAD1	c.1561G>A	P.A521T
SH_09	7	50444466	50444466	Silent	SNV	C	A	A	IKZF1	c.396C>A	P.L132L
SH_09	8	110980831	110980831	Splice_Site	SNV	G	T	T	KCNV1	c.992-3C>A	

SH_09	8	59515912	59515912	Missense_Mutation	SNV	T	C	C	NSMAF	c.995A>G	P.Y332C
SH_10	1	111216638	111216638	Missense_Mutation	SNV	T	G	G	KCNA3	c.794A>C	P.Y265S
SH_10	1	160106787	160106787	Missense_Mutation	SNV	A	C	C	ATP1A2	c.2806A>C	P.T936P
SH_10	1	197397096	197397096	Missense_Mutation	SNV	G	A	A	CRB1	c.2305G>A	P.V769I
SH_10	1	27057766	27057766	Nonsense_Mutation	SNV	C	T	T	ARID1A	c.1474C>T	P.Q492*
SH_10	1	50317156	50317156	Silent	SNV	C	T	T	AGBL4	c.69G>A	P.G23G
SH_10	15	54916034	54916034	Frame_Shift_Del	INDEL	T	-	-	UNC13C	c.6242delT	P.F2081fs
SH_10	15	65553304	65553304	Missense_Mutation	SNV	G	A	A	PARP16	c.752C>T	P.P251L
SH_10	15	93595630	93595630	Missense_Mutation	SNV	G	A	A	RGMA	c.262C>T	P.R88C
SH_10	15	94883491	94883491	Missense_Mutation	SNV	C	G	G	MCTP2	c.844C>G	P.L282V
SH_10	17	18188822	18188822	Missense_Mutation	SNV	G	A	A	TOP3A	c.1325C>T	P.T442I
SH_10	17	35548072	35548072	Missense_Mutation	SNV	C	T	T	ACACA	c.4561G>A	P.D1521N
SH_10	17	73239608	73239608	Missense_Mutation	SNV	A	C	C	GGA3	c.128T>G	P.I43S
SH_10	17	73887396	73887396	Missense_Mutation	SNV	C	T	T	TRIM65	c.952G>A	P.A318T
SH_10	19	50138891	50138891	Missense_Mutation	SNV	G	A	A	RRAS	c.599C>T	P.P200L
SH_10	19	54403899	54403899	Missense_Mutation	SNV	G	A	A	PRKCG	c.1471G>A	P.E491K
SH_10	2	183731159	183731159	Missense_Mutation	SNV	G	A	A	FRZB	c.122C>T	P.P41L
SH_10	2	242026810	242026810	Silent	SNV	T	C	C	SNED1	c.4125T>C	P.Y1375Y
SH_10	20	57485829	57485829	Missense_Mutation	SNV	A	G	G	GNAS	c.1130A>G	P.N377S
SH_10	21	31973533	31973533	Missense_Mutation	SNV	G	T	T	KRTAP22-1	c.94G>T	P.A32S
SH_10	3	12586867	12586867	Missense_Mutation	SNV	G	T	T	MKRN2OS	c.97C>A	P.Q33K
SH_10	3	186969515	186969521	Frame_Shift_Del	INDEL	AGGCTGT	-	-	MASP1	12_918delACAG	P.Q305fs
SH_10	3	42742237	42742237	Missense_Mutation	SNV	G	A	A	HHATL	c.82C>T	P.R28W
SH_10	7	150269953	150269953	Missense_Mutation	SNV	G	T	T	GIMAP4	c.795G>T	P.K265N
SH_10	7	158540897	158540897	Missense_Mutation	SNV	G	T	T	ESYT2	c.1713C>A	P.D571E
SH_10	7	96650088	96650088	Missense_Mutation	SNV	A	G	G	DLX5	c.830T>C	P.L277S
SH_10	8	59059801	59059801	Missense_Mutation	SNV	G	A	A	FAM110B	c.1012G>A	P.V338M
SH_10	9	37441440	37441440	Missense_Mutation	SNV	A	C	C	ZBTB5	c.1109T>G	P.L370R
SH_100	10	76781010	76781010	Silent	SNV	A	T	T	KAT6B	c.2439A>T	P.A813A
SH_100	14	42356383	42356383	Missense_Mutation	SNV	C	A	A	LRFN5	c.555C>A	P.N185K
SH_100	16	17211774	17211774	Silent	SNV	G	A	A	XYLT1	c.2286C>T	P.P762P
SH_100	16	568941	568941	Splice_Site	SNV	A	G	G	RAB11FIP3	c.753-2A>G	
SH_100	17	72366769	72366769	Silent	SNV	C	T	T	GPR142	c.468C>T	P.G156G
SH_100	2	118735598	118735598	Missense_Mutation	SNV	C	A	A	CCDC93	c.629G>T	P.G210V
SH_100	2	169791787	169791787	Missense_Mutation	SNV	G	A	A	ABCB11	c.2963C>T	P.A988V
SH_100	2	179426577	179426577	Silent	SNV	C	T	T	TTN	c.79359G>A	P.K26453K
SH_100	22	16266960	16266960	Missense_Mutation	SNV	A	G	G	POTEH	c.1489T>C	P.F497L
SH_100	4	145580881	145580881	Missense_Mutation	SNV	G	A	A	HHIP	c.722G>A	P.R241H
SH_100	4	2097630	2097630	Silent	SNV	G	A	A	POLN	c.2013C>T	P.H671H
SH_100	5	37153998	37153998	Silent	SNV	T	C	C	C5orf42	c.8055A>G	P.A2685A
SH_100	5	96315225	96315225	Missense_Mutation	SNV	A	T	T	LNPEP	c.403A>T	P.T135S
SH_100	6	138583961	138583961	Missense_Mutation	SNV	C	A	A	ARFGEF3	c.1341C>A	P.S447R
SH_100	6	24528252	24528252	Missense_Mutation	SNV	T	A	A	ALDH5A1	c.1201T>A	P.S401T
SH_100	7	26680305	26680305	Splice_Site	SNV	G	C	C	C7orf71	c.201-10G>C	
SH_100	7	99621357	99621357	Silent	SNV	G	T	T	ZKSCAN1	c.120G>T	P.R40R
SH_100	8	67831323	67831323	Silent	SNV	T	C	C	MCMDC2	c.1965T>C	P.Y655Y
SH_100	9	139357484	139357484	Missense_Mutation	SNV	G	A	A	SEC16A	c.4748C>T	P.T1583M
SH_100	X	123480532	123480532	Missense_Mutation	SNV	G	A	A	SH2D1A	c.40G>A	P.E14K
SH_101	1	173446546	173446546	Missense_Mutation	SNV	G	A	A	PRDX6	c.10G>A	P.G4S
SH_101	1	228362441	228362441	Missense_Mutation	SNV	C	A	A	IBA57	c.390C>A	P.S130R
SH_101	10	51579175	51579175	Missense_Mutation	SNV	A	C	C	NCOA4	c.82A>C	P.S28R
SH_101	13	99087969	99087969	Splice_Site	SNV	C	A	A	FARP1	c.2274+9C>A	
SH_101	14	75420612	75420612	Missense_Mutation	SNV	A	C	C	PGF	c.113T>G	P.V38G
SH_101	15	43571977	43571977	Silent	SNV	C	A	A	TGM7	c.1524G>T	P.L508L
SH_101	16	57945763	57945763	Missense_Mutation	SNV	C	A	A	CNGB1	c.2386G>T	P.A796S
SH_101	18	42532845	42532845	Silent	SNV	C	A	A	SETBP1	c.3540C>A	P.G1180G
SH_101	19	41119074	41119074	Silent	SNV	G	T	T	LTBP4	c.2604G>T	P.A868A
SH_101	19	4513287	4513287	Missense_Mutation	SNV	C	T	T	PLIN4	c.643G>A	P.A215T
SH_101	19	6396450	6396450	Silent	SNV	C	G	G	LOC390877	c.60C>G	P.G20G
SH_101	2	202252434	202252434	Missense_Mutation	SNV	G	T	T	TRAK2	c.1688C>A	P.P563H
SH_101	2	37152334	37152334	Silent	SNV	C	T	T	STRN	c.252G>A	P.L84L
SH_101	2	55825578	55825578	Missense_Mutation	SNV	T	C	C	PPP4R3B	c.895A>G	P.K299E
SH_101	21	45528860	45528860	Splice_Site	SNV	C	T	T	PWP2	c.19-5C>T	
SH_101	5	170323065	170323065	Silent	SNV	A	G	G	RANBP17	c.435A>G	P.E145E
SH_101	5	5464725	5464725	Missense_Mutation	SNV	T	C	C	ICE1	c.5278T>C	P.S1760P
SH_101	6	7558461	7558461	Missense_Mutation	SNV	G	A	A	DSP	c.386G>A	P.R129Q
SH_101	7	50455052	50455052	Missense_Mutation	SNV	C	A	A	IKZF1	c.599C>A	P.P200H
SH_101	7	55240762	55240762	Missense_Mutation	SNV	G	A	A	EGFR	c.2006G>A	P.R669Q
SH_107	1	205895738	205895738	Silent	SNV	G	A	A	SLC26A9	c.1314C>T	P.I438I
SH_107	10	135053221	135053221	Missense_Mutation	SNV	C	T	T	VENTX	c.283C>T	P.R95C
SH_107	10	71968993	71968993	Silent	SNV	C	A	A	PPA1	c.693G>T	P.V231V
SH_107	10	90497497	90497497	Silent	SNV	C	T	T	LIPK	c.775C>T	P.L259L
SH_107	10	98144423	98144423	Silent	SNV	C	T	T	TLL2	c.2115G>A	P.S705S
SH_107	11	22398184	22398184	Missense_Mutation	SNV	G	T	T	SLC17A6	c.1379G>T	P.C460F
SH_107	12	25398284	25398284	Missense_Mutation	SNV	C	A	A	KRAS	c.35G>T	P.G12V
SH_107	13	61141657	61141657	Splice_Site	SNV	A	G	G	TDRD3	c.2119-2A>G	
SH_107	15	24922841	24922841	Silent	SNV	A	C	C	NPAP1	c.1827A>C	P.A609A
SH_107	15	25223587	25223587	Missense_Mutation	SNV	C	G	G	SNRPN	c.719C>G	P.P240R
SH_107	16	81969881	81969881	Missense_Mutation	SNV	C	T	T	PLCG2	c.2950C>T	P.R984C
SH_107	17	12901792	12901792	Missense_Mutation	SNV	A	C	C	ELAC2	c.1337T>G	P.L446R
SH_107	17	79496251	79496251	Missense_Mutation	SNV	G	A	A	FSCN2	c.694G>A	P.V232M
SH_107	2	209113113	209113113	Missense_Mutation	SNV	G	A	A	IDH1	c.394C>T	P.R132C
SH_107	2	60995638	60995638	Missense_Mutation	SNV	G	T	T	PAPOLG	c.280G>T	P.G94C
SH_107	4	174253396	174253396	Splice_Site	SNV	T	C	C	HMGB2	c.472-7A>G	
SH_107	4	54424083	54424083	Frame_Shift_Del	INDEL	T	-	-	LNX1	c.50delA	P.Y17fs
SH_107	7	63720585	63720587	Splice_Site	INDEL	TTA	-	-	ZNF679	40-12_40-10delATT	
SH_107	8	106573623	106573623	Nonsense_Mutation	SNV	C	T	T	ZFPM2	c.334C>T	P.R112*
SH_107	X	114541292	114541292	Missense_Mutation	SNV	A	C	C	LUZP4	c.619A>C	P.N207H
SH_108	1	109369841	109369841	Nonsense_Mutation	SNV	G	T	T	AKNAD1	c.1922C>A	P.S641*
SH_108	1	152052228	152052228	Silent	SNV	C	T	T	LOC100131107	c.1056G>A	P.P352P
SH_108	1	153735305	153735305	Missense_Mutation	SNV	G	A	A	INTS3	c.1618G>A	P.E540K
SH_108	1	2700312	2700312	Missense_Mutation	SNV	C	T	T	TTC34	c.529G>A	P.A177T
SH_108	1	27100292	27100292	Splice_Site	SNV	G	C	C	ARID1A	c.4005-1G>C	
SH_108	1	3679830	3679830	Missense_Mutation	SNV	G	T	T	CCDC27	c.1113G>T	P.E371D
SH_108	1	67559216	67559216	Frame_Shift_Ins	INDEL	-	T	T	C1orf141	c.674dupA	P.N225fs
SH_108	11	111904162	111904162	Missense_Mutation	SNV	T	C	C	DLAT	c.695T>C	P.M232T
SH_108	11	118942532	118942532	Missense_Mutation	SNV	G	A	A	VPS11	c.830G>A	P.R277H
SH_108	11	494979	494979	Missense_Mutation	SNV	C	T	T	RNH1	c.1202G>A	P.R401H

SH_108	11	51412298	51412298	Missense_Mutation	SNV	T	C	C	OR4A5	c.98A>G	P.Y33C
SH_108	12	123103017	123103017	Splice_Site	INDEL	-	G	G	KNTC1	)86-12_6086-11insG	
SH_108	12	20885887	20885887	Missense_Mutation	SNV	G	A	A	SLCO1C1	c.877G>A	P.G293R
SH_108	12	25398284	25398284	Missense_Mutation	SNV	C	A	A	KRAS	c.35G>T	P.G12V
SH_108	12	41967137	41967137	Missense_Mutation	SNV	C	G	G	PDZRN4	c.2556C>G	P.S852R
SH_108	12	48596381	48596381	Missense_Mutation	SNV	G	A	A	OR10AD1	c.695C>T	P.S232F
SH_108	12	52864372	52864372	Missense_Mutation	SNV	G	C	C	KRT6C	c.1120C>G	P.L374V
SH_108	12	58131354	58131354	Missense_Mutation	SNV	T	A	A	AGAP2	c.676A>T	P.S226C
SH_108	12	6707550	6707550	Nonsense_Mutation	SNV	C	T	T	CHD4	c.1524G>A	P.W508*
SH_108	12	88589318	88589318	Silent	SNV	C	T	T	TMTC3	c.2637C>T	P.D879D
SH_108	13	110438120	110438120	Missense_Mutation	SNV	G	C	C	IRS2	c.281C>G	P.A94G
SH_108	14	21961267	21961267	Missense_Mutation	SNV	C	G	G	TOX4	c.1423C>G	P.L475V
SH_108	14	93412799	93412799	Missense_Mutation	SNV	C	T	T	ITPK1	c.778G>A	P.E260K
SH_108	15	29415586	29415586	Missense_Mutation	SNV	C	G	G	FAM189A1	c.1576G>C	P.E526Q
SH_108	15	34147045	34147045	Silent	SNV	T	C	C	RYR3	c.13939T>C	P.L4647L
SH_108	15	89391214	89391214	Silent	SNV	C	T	T	ACAN	c.1677C>T	P.G559G
SH_108	16	16313478	16313478	Missense_Mutation	SNV	A	G	G	ABCC6	c.407T>C	P.L136P
SH_108	16	23360010	23360010	Silent	SNV	C	T	T	SCNN1B	c.90C>T	P.C30C
SH_108	16	88951511	88951511	Nonsense_Mutation	SNV	G	A	A	CBFA2T3	c.1060C>T	P.R354*
SH_108	17	10222426	10222426	Missense_Mutation	SNV	C	T	T	MYH13	c.3419G>A	P.R1140H
SH_108	17	42152809	42152809	Missense_Mutation	SNV	G	C	C	G6PC3	c.548G>C	P.G183A
SH_108	17	7577559	7577559	Missense_Mutation	SNV	G	A	A	TP53	c.722C>T	P.S241F
SH_108	17	76122990	76122990	Splice_Site	SNV	G	A	A	TMC6	c.-74-3C>T	
SH_108	18	67614066	67614066	Missense_Mutation	SNV	G	A	A	CD226	c.286C>T	P.R96W
SH_108	19	45648183	45648183	Silent	SNV	C	G	G	PPP1R37	c.1059C>G	P.L353L
SH_108	19	52869634	52869634	Nonsense_Mutation	SNV	C	T	T	ZNF610	c.1003C>T	P.Q335*
SH_108	19	56274176	56274176	Missense_Mutation	SNV	G	A	A	RFPL4A	c.499G>A	P.V167M
SH_108	19	9057855	9057855	Missense_Mutation	SNV	G	T	T	MUC16	c.29591C>A	P.T9864N
SH_108	2	141625299	141625299	Missense_Mutation	SNV	C	T	T	LRP1B	c.4439G>A	P.S1480N
SH_108	2	174230331	174230331	Splice_Site	SNV	C	G	G	CDCA7	c.1035+11C>G	
SH_108	20	3146827	3146827	Silent	SNV	C	T	T	LZTS3	c.639G>A	P.G213G
SH_108	22	26176157	26176157	Nonsense_Mutation	SNV	C	T	T	MYO18B	c.2203C>T	P.Q735*
SH_108	22	39710716	39710716	Missense_Mutation	SNV	T	C	C	RPL3	c.824A>G	P.H275R
SH_108	3	178952085	178952085	Missense_Mutation	SNV	A	G	G	PIK3CA	c.3140A>G	P.H1047R
SH_108	3	182941958	182941958	Nonsense_Mutation	SNV	A	C	C	MCF2L2	c.2136T>G	P.Y712*
SH_108	3	52545756	52545756	Missense_Mutation	SNV	G	A	A	STAB1	c.2878G>A	P.G960S
SH_108	3	61548051	61548051	Splice_Site	SNV	G	T	T	PTPRG	c.85+5G>T	
SH_108	3	77607107	77607107	Missense_Mutation	SNV	G	T	T	ROBO2	c.1184G>T	P.R395I
SH_108	4	145038018	145038018	Nonsense_Mutation	SNV	G	A	A	GYPA	c.307C>T	P.R103*
SH_108	5	132160905	132160905	Missense_Mutation	SNV	C	T	T	SHROOM1	c.928G>A	P.E310K
SH_108	5	149431584	149431584	Silent	SNV	C	T	T	HMGXB3	c.3708C>T	P.D1236D
SH_108	5	43174972	43174972	Missense_Mutation	SNV	G	A	A	ZNF131	c.1609G>A	P.E537K
SH_108	6	152265535	152265535	Missense_Mutation	SNV	G	A	A	ESR1	c.988G>A	P.E330K
SH_108	6	26452100	26452100	Missense_Mutation	SNV	G	C	C	BTN3A3	c.586G>C	P.E196Q
SH_108	7	127954977	127954977	Missense_Mutation	SNV	C	T	T	RBM28	c.1462G>A	P.E488K
SH_108	7	158424395	158424395	Missense_Mutation	SNV	G	A	A	NCAPG2	c.3395C>T	P.S1132L
SH_108	7	29186261	29186261	Missense_Mutation	SNV	C	T	T	CHN2	c.62C>T	P.T21I
SH_108	7	30721577	30721577	Missense_Mutation	SNV	C	G	G	CRHR2	c.264G>C	P.E88D
SH_108	8	100654571	100654571	Missense_Mutation	SNV	G	A	A	VPS13B	c.5828G>A	P.R1943Q
SH_108	8	110416854	110416854	Missense_Mutation	SNV	G	A	A	PKHD1L1	c.1445G>A	P.R482Q
SH_108	8	145168736	145168736	Silent	SNV	G	A	A	WDR97	c.3360G>A	P.L1120L
SH_108	8	145692708	145692708	Silent	SNV	G	C	C	KIFC2	c.453G>C	P.R151R
SH_108	8	55542151	55542151	Missense_Mutation	SNV	A	T	T	RP1	c.5709A>T	P.E1903D
SH_108	8	93004034	93004034	Missense_Mutation	SNV	C	T	T	RUNX1T1	c.824G>A	P.R275Q
SH_108	9	125424476	125424476	Missense_Mutation	SNV	T	A	A	OR1L1	c.482T>A	P.L161H
SH_108	9	33030547	33030547	Silent	SNV	T	A	A	DNAJA1	c.54T>A	P.S18S
SH_108	9	87342596	87342596	Missense_Mutation	SNV	C	G	G	NTRK2	c.881C>G	P.S294C
SH_108	X	24844692	24844692	Missense_Mutation	SNV	A	T	T	POLA1	c.3692A>T	P.D1231V
SH_108	X	69263794	69263794	Silent	SNV	G	A	A	AWAT2	c.249C>T	P.S83S
SH_110	1	153642663	153642663	Missense_Mutation	SNV	C	T	T	ILF2	c.49G>A	P.G17R
SH_110	1	158913594	158913594	Missense_Mutation	SNV	G	T	T	PYHIN1	c.1017G>T	P.R339S
SH_110	1	160209613	160209613	Silent	SNV	C	A	A	DCAF8	c.597G>T	P.L199L
SH_110	1	175323551	175323551	Missense_Mutation	SNV	T	C	C	TNR	c.2359A>G	P.I787V
SH_110	1	39878310	39878310	Silent	SNV	A	T	T	KIAA0754	c.2373A>T	P.S791S
SH_110	10	5248281	5248281	Missense_Mutation	SNV	G	T	T	AKR1C4	c.491G>T	P.G164V
SH_110	10	5967365	5967365	Silent	SNV	C	T	T	FBXO18	c.2461C>T	P.L821L
SH_110	10	79616501	79616501	Missense_Mutation	SNV	C	A	A	DLG5	c.523G>T	P.A175S
SH_110	11	17596262	17596262	Splice_Site	SNV	T	A	A	OTOG	c.2330-5T>A	
SH_110	15	44093333	44093333	Missense_Mutation	SNV	A	G	G	HYPK	c.190A>G	P.M64V
SH_110	17	1704227	1704231	Frame_Shift_Del	INDEL	ACCAG	-	-	SMYD4	457_461delCTG(	P.L153fs
SH_110	17	7577535	7577535	Missense_Mutation	SNV	C	A	A	TP53	c.746G>T	P.R249M
SH_110	18	24917153	24917153	Splice_Site	SNV	T	C	C	LOC105372038	n.251-2A>G	
SH_110	19	42312940	42312940	Missense_Mutation	SNV	T	A	A	CEACAM3	c.514T>A	P.C172S
SH_110	19	58145986	58145986	Splice_Site	SNV	T	C	C	ZNF211	c.130-11T>C	
SH_110	2	216248111	216248112	Frame_Shift_Del	INDEL	TG	-	-	FN1	.4989_4990del(	P.Y1663fs
SH_110	2	219330857	219330857	Missense_Mutation	SNV	T	C	C	USP37	c.2342A>G	P.N781S
SH_110	20	23065896	23065896	Missense_Mutation	SNV	G	A	A	CD93	c.934C>T	P.R312C
SH_110	20	33077647	33077647	Silent	SNV	A	G	G	ITCH	c.2358A>G	P.Q786Q
SH_110	3	125701106	125701106	Splice_Site	SNV	T	A	A	ROPN1B	c.397-7T>A	
SH_110	5	161576158	161576158	Silent	SNV	C	A	A	GABRG2	c.967C>A	P.R323R
SH_110	6	43269002	43269002	Splice_Site	SNV	G	A	A	SLC22A7	c.945+6G>A	
SH_110	6	43603619	43603619	Missense_Mutation	SNV	G	C	C	MAD2L1BP	c.7G>C	P.A3P
SH_110	7	44097465	44097465	Splice_Site	SNV	G	T	T	DBNL	c.552+7G>T	
SH_110	8	13965710	13965710	Missense_Mutation	SNV	C	A	A	SGCZ	c.480G>T	P.E160D
SH_111	1	110581320	110581320	Missense_Mutation	SNV	C	T	T	STRIP1	c.266C>T	P.T89M
SH_111	1	121116750	121116750	Missense_Mutation	SNV	G	T	T	SRGAP2C	c.343G>T	P.A115S
SH_111	1	156449559	156449559	Missense_Mutation	SNV	C	A	A	MEF2D	c.426G>T	P.M142I
SH_111	1	17657625	17657625	Missense_Mutation	SNV	G	T	T	PADI4	c.254G>T	P.G85V
SH_111	1	236212233	236212233	Silent	SNV	G	A	A	NID1	c.282C>T	P.P94P
SH_111	1	27106504	27106504	Nonsense_Mutation	SNV	C	T	T	ARID1A	c.6115C>T	P.Q2039*
SH_111	1	68942629	68942629	Missense_Mutation	SNV	G	A	A	DEPDC1	c.2422C>T	P.R808C
SH_111	1	74507219	74507219	Frame_Shift_Ins	INDEL	-	T	T	LRRIQ3	c.1395dupA	P.Y466fs
SH_111	10	102275937	102275937	Missense_Mutation	SNV	T	C	C	SEC31B	c.119A>G	P.N40S
SH_111	10	108357142	108357142	Nonsense_Mutation	SNV	G	A	A	SORCS1	c.3232C>T	P.R1078*
SH_111	10	59959035	59959035	Silent	SNV	G	A	A	IPMK	c.594C>T	P.Y198Y
SH_111	10	71255367	71255367	Silent	SNV	C	T	T	TSPAN15	c.375C>T	P.N125N
SH_111	11	111848524	111848524	Splice_Site	SNV	G	T	T	DIXDC1	c.23+5G>T	
SH_111	11	119291030	119291030	Missense_Mutation	SNV	C	T	T	THY1	c.104G>A	P.R35H

SH_111	11	92715229	92715229	Missense_Mutation	SNV	A	T	T	MTNR1B	c.840A>T	P.E280D
SH_111	12	25398284	25398284	Missense_Mutation	SNV	C	T	T	KRAS	c.35G>A	P.G12D
SH_111	12	58013409	58013409	Splice_Site	SNV	C	A	A	LOC101927583	n.2120+5G>T	
SH_111	12	8375111	8375111	Silent	SNV	C	T	T	FAM90A1	c.702G>A	P.A234A
SH_111	14	93412735	93412735	Missense_Mutation	SNV	T	A	A	ITPK1	c.842A>T	P.D281V
SH_111	14	94088174	94088174	Missense_Mutation	SNV	C	T	T	UNC79	c.4064C>T	P.P1355L
SH_111	15	22383359	22383359	Missense_Mutation	SNV	C	G	G	OR4N4	c.887C>G	P.T296S
SH_111	15	99671001	99671001	Silent	SNV	G	A	A	SYNM	c.2433G>A	P.T811T
SH_111	16	19620328	19620328	Missense_Mutation	SNV	A	G	G	C16orf62	c.1181A>G	P.K394R
SH_111	16	20435280	20435280	Silent	SNV	G	A	A	ACSM5	c.810G>A	P.T270T
SH_111	17	7324624	7324624	Silent	SNV	A	C	C	SPEM1	c.630A>C	P.P210P
SH_111	2	113539278	113539278	Silent	SNV	T	C	C	IL1A	c.222A>G	P.V74V
SH_111	2	159033054	159033054	Frame_Shift_Ins	INDEL	-	A	A	CCDC148	c.1169dupT	P.L390fs
SH_111	2	27857793	27857793	Splice_Site	SNV	T	G	G	GPN1	c.393+2T>G	
SH_111	2	68882553	68882553	Missense_Mutation	SNV	G	A	A	PROKR1	c.1027G>A	P.V343M
SH_111	20	55777539	55777539	Missense_Mutation	SNV	G	A	A	BMP7	c.752C>T	P.T251M
SH_111	21	30699470	30699470	Frame_Shift_Del	INDEL	T	-	-	BACH1	c.1326delT	P.S443fs
SH_111	3	148896286	148896286	Missense_Mutation	SNV	C	G	G	CP	c.2794G>C	P.D932H
SH_111	3	30713853	30713853	Missense_Mutation	SNV	G	A	A	TGFBR2	c.1253G>A	P.C418Y
SH_111	3	54933813	54933813	Splice_Site	SNV	A	T	T	CACNA2D3	c.2369-2A>T	
SH_111	5	147051334	147051334	Silent	SNV	G	A	A	JAKMIP2	c.36C>T	P.P12P
SH_111	6	160174638	160174638	Missense_Mutation	SNV	G	A	A	WTAP	c.599G>A	P.S200N
SH_111	6	24875951	24875951	Silent	SNV	C	T	T	FAM65B	c.156G>A	P.A52A
SH_111	7	121965521	121965521	Splice_Site	SNV	T	C	C	CADPS2	c.3594+10A>G	
SH_111	7	144345911	144345911	Missense_Mutation	SNV	A	T	T	TPK1	c.247T>A	P.Y83N
SH_111	8	25199252	25199252	Splice_Site	SNV	C	T	T	DOCK5	c.2440-3C>T	
SH_111	8	89209488	89209488	Missense_Mutation	SNV	C	T	T	MMP16	c.180G>A	P.M60I
SH_111	9	125424800	125424800	Missense_Mutation	SNV	A	G	G	OR1L1	c.806A>G	P.K269R
SH_111	9	125424802	125424802	Missense_Mutation	SNV	G	A	A	OR1L1	c.808G>A	P.D270N
SH_111	9	130982480	130982480	Missense_Mutation	SNV	C	T	T	DNM1	c.709C>T	P.R237W
SH_111	9	86468653	86468653	Missense_Mutation	SNV	A	C	C	KIF27	c.3050T>G	P.L1017R
SH_111	X	37587545	37587545	Nonsense_Mutation	SNV	C	T	T	XK	c.1165C>T	P.Q389*
SH_111	X	47032597	47032597	Splice_Site	SNV	G	A	A	RBM10	c.502+1G>A	
SH_113	1	13183781	13183781	Missense_Mutation	SNV	G	T	T	HNRNPCL2	c.92C>A	P.S31Y
SH_113	1	39775375	39775375	Splice_Site	SNV	A	G	G	MACF1	c.2931+7A>G	
SH_113	1	43773103	43773103	Missense_Mutation	SNV	C	A	A	TIE1	c.773C>A	P.A258D
SH_113	10	119003551	119003551	Missense_Mutation	SNV	G	A	A	SLC18A2	c.191G>A	P.R64K
SH_113	10	132891516	132891516	Missense_Mutation	SNV	C	A	A	TCERG1L	c.1670G>T	P.R557I
SH_113	10	29769544	29769544	Missense_Mutation	SNV	G	T	T	SVIL	c.4369C>A	P.L1457I
SH_113	11	124180558	124180558	Silent	SNV	A	G	G	OR8D1	c.105T>C	P.Y35Y
SH_113	11	126080806	126080806	Missense_Mutation	SNV	G	A	A	RPUSD4	c.334C>T	P.P112S
SH_113	11	126391275	126391275	Missense_Mutation	SNV	G	A	A	KIRREL3	c.368C>T	P.A123V
SH_113	11	55872934	55872934	Missense_Mutation	SNV	G	T	T	OR8H2	c.416G>T	P.R139M
SH_113	11	62327300	62327300	Missense_Mutation	SNV	C	A	A	EEF1G	c.1165G>T	P.D389Y
SH_113	11	986887	986887	Silent	SNV	G	T	T	AP2A2	c.1068G>T	P.L356L
SH_113	12	129293321	129293321	Splice_Site	SNV	A	T	T	SLC15A4	c.1258+12T>A	
SH_113	12	9254111	9254114	Frame_Shift_Del	INDEL	CTGT	-	-	A2M	423_1426delAC	P.T475fs
SH_113	13	80911668	80911668	Missense_Mutation	SNV	C	G	G	SPRY2	c.173G>C	P.G58A
SH_113	14	21991956	21991956	Missense_Mutation	SNV	A	T	T	SALL2	c.1501T>A	P.C501S
SH_113	2	107002879	107002879	Splice_Site	SNV	C	A	A	PLGLA	n.244+8C>A	
SH_113	2	179605000	179605000	Silent	SNV	G	T	T	TTN	c.12009C>A	P.V4003V
SH_113	3	35835429	35835429	Silent	SNV	T	C	C	ARPP21	c.2418T>C	P.A806A
SH_113	3	56651150	56651150	Silent	SNV	T	C	C	CCDC66	c.1752T>C	P.N584N
SH_113	4	55973998	55973998	Missense_Mutation	SNV	T	C	C	KDR	c.1318A>G	P.T440A
SH_113	4	68380089	68380089	Missense_Mutation	SNV	T	C	C	CENPC	c.1147A>G	P.T383A
SH_113	4	956312	956312	Missense_Mutation	SNV	C	A	A	DGKQ	c.2125G>T	P.A709S
SH_113	5	37048753	37048753	Missense_Mutation	SNV	C	T	T	NIPBL	c.6739C>T	P.R2247C
SH_113	5	41149466	41149466	Missense_Mutation	SNV	T	C	C	C6	c.2500A>G	P.I834V
SH_113	6	33263862	33263862	Silent	SNV	C	A	A	RGL2	c.465G>T	P.T155T
SH_113	7	73462526	73462526	Missense_Mutation	SNV	G	T	T	ELN	c.740G>T	P.G247V
SH_113	7	82580151	82580151	Silent	SNV	G	A	A	PCLO	c.9753C>T	P.I3251I
SH_113	8	107773510	107773510	Missense_Mutation	SNV	G	A	A	ABRA	c.901C>T	P.R301C
SH_113	8	145947384	145947384	Missense_Mutation	SNV	A	G	G	ZNF251	c.1661T>C	P.I554T
SH_113	9	35802548	35802548	Missense_Mutation	SNV	A	G	G	NPR2	c.1759A>G	P.I587V
SH_115	1	152284210	152284210	Missense_Mutation	SNV	C	T	T	FLG	c.3152G>A	P.R1051H
SH_115	1	247062817	247062817	Splice_Site	SNV	A	C	C	AHCTF1	c.1433-3T>G	
SH_115	11	73022494	73022494	Missense_Mutation	SNV	C	A	A	ARHGEF17	c.2811C>A	P.D937E
SH_115	11	76371887	76371887	Silent	SNV	C	A	A	LRRC32	c.750G>T	P.L250L
SH_115	14	77751890	77751890	Missense_Mutation	SNV	C	T	T	POMT2	c.1418G>A	P.R473Q
SH_115	16	684601	684601	Missense_Mutation	SNV	T	C	C	METTL26	c.380A>G	P.N127S
SH_115	17	29622596	29622596	Missense_Mutation	SNV	T	C	C	OMG	c.754A>G	P.T252A
SH_115	17	30986316	30986316	Missense_Mutation	SNV	G	T	T	MYO1D	c.2162C>A	P.T721N
SH_115	17	48667880	48667880	Missense_Mutation	SNV	A	C	C	CACNA1G	c.2350A>C	P.S784R
SH_115	19	43259170	43259170	Missense_Mutation	SNV	G	A	A	PSG8	c.958C>T	P.R320C
SH_115	2	113785536	113785536	Missense_Mutation	SNV	T	A	A	IL36B	c.418A>T	P.T140S
SH_115	2	152289674	152289674	Missense_Mutation	SNV	A	G	G	RIF1	c.1009A>G	P.T337A
SH_115	2	203378462	203378462	Nonsense_Mutation	SNV	C	T	T	BMPR2	c.439C>T	P.R147*
SH_115	20	44036884	44036884	Missense_Mutation	SNV	G	A	A	DBNDD2	c.83G>A	P.S28N
SH_115	22	16287550	16287550	Silent	SNV	G	A	A	POTEH	c.336C>T	P.S112S
SH_115	3	178952085	178952085	Missense_Mutation	SNV	A	G	G	PIK3CA	c.3140A>G	P.H1047R
SH_115	4	56502258	56502258	Silent	SNV	G	T	T	NMU	c.102C>A	P.G34G
SH_115	5	37006698	37006698	Splice_Site	SNV	C	T	T	NIPBL	c.4087+8C>T	
SH_115	5	67591077	67591077	Missense_Mutation	SNV	G	A	A	PIK3R1	c.1670G>A	P.R557Q
SH_115	7	140453136	140453136	Missense_Mutation	SNV	A	T	T	BRAF	c.1799T>A	P.V600E
SH_115	7	148771598	148771598	Missense_Mutation	SNV	A	C	C	ZNF786	c.178T>G	P.W60G
SH_115	8	133882025	133882025	Silent	SNV	C	T	T	TG	c.228C>T	P.N76N
SH_115	8	42694370	42694370	Frame_Shift_Ins	INDEL	-	A	A	THAP1	c.225dupT	P.A76fs
SH_115	8	57026165	57026165	Missense_Mutation	SNV	C	T	T	MOS	c.377G>A	P.R126H
SH_115	8	92972548	92972548	Silent	SNV	C	T	T	RUNX1T1	c.1737G>A	P.P579P
SH_115	9	386353	386353	Missense_Mutation	SNV	G	A	A	DOCK8	c.2801G>A	P.R934Q
SH_118	1	176863905	176863905	Silent	SNV	T	A	A	ASTN1	c.2733A>T	P.P911P
SH_118	1	89732172	89732172	Missense_Mutation	SNV	T	C	C	GBP5	c.725A>G	P.Q242R
SH_118	10	112839054	112839054	Missense_Mutation	SNV	A	T	T	ADRA2A	c.1300A>T	P.S434C
SH_118	10	13164469	13164469	Missense_Mutation	SNV	A	T	T	OPTN	c.864A>T	P.E288D
SH_118	10	22675791	22675791	Missense_Mutation	SNV	T	C	C	SPAG6	c.506T>C	P.I169T
SH_118	11	122756913	122756913	Splice_Site	SNV	G	T	T	C11orf63	c.344+12G>T	
SH_118	11	6245708	6245708	Silent	SNV	C	T	T	FAM160A2	c.39G>A	P.R13R
SH_118	13	78235572	78235572	Missense_Mutation	SNV	C	G	G	LOC100129307	c.1190G>C	P.R397T

SH_118	17	48917669	48917669	Missense_Mutation	SNV	G	C	C	WFIKKN2	c.1020G>C	P.E340D
SH_118	19	19680276	19680276	Silent	SNV	G	A	A	PBX4	c.750C>T	P.G250G
SH_118	19	40834359	40834359	Missense_Mutation	SNV	C	T	T	C19orf47	c.511G>A	P.V171M
SH_118	19	4306232	4306234	Missense_Mutation	INDEL	AGT	-	-	FSD1	.149_151delAG	.E50_F51delinsV
SH_118	2	132021093	132021093	Missense_Mutation	SNV	A	G	G	POTEE	c.2065A>G	P.N689D
SH_118	2	166850884	166850884	Missense_Mutation	SNV	C	A	A	SCN1A	c.4624G>T	P.V1542F
SH_118	21	46687568	46687568	Missense_Mutation	SNV	T	A	A	POFUT2	c.1073A>T	P.E358V
SH_118	3	169497224	169497224	Missense_Mutation	SNV	C	T	T	MYNN	c.935C>T	P.S312L
SH_118	3	31725287	31725287	Missense_Mutation	SNV	T	A	A	OSBPL10	c.1373A>T	P.K458I
SH_118	3	97887561	97887561	Silent	SNV	A	T	T	OR5H15	c.18A>T	P.A6A
SH_118	5	140167794	140167794	Missense_Mutation	SNV	C	A	A	PCDHA1	c.1919C>A	P.A640D
SH_118	7	82531991	82531991	Missense_Mutation	SNV	C	T	T	PCLO	c.13504G>A	P.D4502N
SH_118	8	94767159	94767159	Missense_Mutation	SNV	G	C	C	TMEM67	c.17G>C	P.G6A
SH_118	9	133932471	133932471	Missense_Mutation	SNV	G	T	T	LAMC3	c.2095G>T	P.G699C
SH_118	X	50377499	50377499	Missense_Mutation	SNV	T	A	A	SHROOM4	c.1574A>T	P.Q525L
SH_119	1	103387119	103387119	Silent	SNV	A	T	T	COL11A1	c.3546T>A	P.P1182P
SH_119	1	109007883	109007883	Silent	SNV	T	A	A	NBPF6	c.1584T>A	P.I528I
SH_119	1	109803859	109803859	Missense_Mutation	SNV	A	T	T	CELSR2	c.4154A>T	P.Q1385L
SH_119	1	109823787	109823787	Missense_Mutation	SNV	T	A	A	PSRC1	c.606A>T	P.R202S
SH_119	1	110134771	110134771	Silent	SNV	A	T	T	GNAI3	c.981A>T	P.T327T
SH_119	1	112042988	112042988	Missense_Mutation	SNV	T	A	A	ADORA3	c.541A>T	P.S181C
SH_119	1	11827056	11827056	Missense_Mutation	SNV	G	A	A	C1orf167	c.1213G>A	P.G405R
SH_119	1	118514487	118514487	Missense_Mutation	SNV	T	A	A	SPAG17	c.6325A>T	P.R2109W
SH_119	1	12776220	12776220	Missense_Mutation	SNV	T	A	A	AADACL3	c.41T>A	P.V14E
SH_119	1	13330623	13330623	Nonsense_Mutation	SNV	A	T	T	PRAMEF22	c.663T>A	P.C221*
SH_119	1	144867918	144867918	Splice_Site	SNV	T	C	C	PDE4DIP	c.5518+3A>G	
SH_119	1	145558247	145558247	Missense_Mutation	SNV	A	T	T	ANKRD35	c.94A>T	P.S32C
SH_119	1	145597578	145597578	Splice_Site	SNV	T	C	C	POLR3C	c.1049-4A>G	
SH_119	1	149898602	149898602	Silent	SNV	C	A	A	SF3B4	c.372G>T	P.G124G
SH_119	1	151090447	151090447	Missense_Mutation	SNV	A	T	T	GABPB2	c.1062A>T	P.R354S
SH_119	1	151774931	151774931	Missense_Mutation	SNV	T	A	A	LINGO4	c.250A>T	P.S84C
SH_119	1	151787148	151787148	Nonsense_Mutation	SNV	T	A	A	RORC	c.772A>T	P.K258*
SH_119	1	152382333	152382333	Missense_Mutation	SNV	T	A	A	CRNN	c.1225A>T	P.S409C
SH_119	1	152882585	152882585	Silent	SNV	A	G	G	IVL	c.312A>G	P.P104P
SH_119	1	153043107	153043107	Missense_Mutation	SNV	T	A	A	SPRR2B	c.209A>T	P.K70M
SH_119	1	153580528	153580528	Missense_Mutation	SNV	T	A	A	S100A16	c.100A>T	P.S34C
SH_119	1	154234163	154234163	Silent	SNV	A	T	T	UBAP2L	c.2892A>T	P.G964G
SH_119	1	154295520	154295520	Nonsense_Mutation	SNV	A	T	T	AQP10	c.295A>T	P.K99*
SH_119	1	15429900	15429900	Missense_Mutation	SNV	C	T	T	KAZN	c.1825C>T	P.P609S
SH_119	1	154574588	154574588	Missense_Mutation	SNV	T	A	A	ADAR	c.530A>T	P.Y177F
SH_119	1	155230223	155230223	Missense_Mutation	SNV	T	A	A	SCAMP3	c.286A>T	P.T96S
SH_119	1	155448365	155448365	Missense_Mutation	SNV	T	A	A	ASH1L	c.4296A>T	P.L1432F
SH_119	1	155874533	155874533	Missense_Mutation	SNV	T	A	A	RIT1	c.226A>T	P.T76S
SH_119	1	156376964	156376964	Missense_Mutation	SNV	A	T	T	C1orf61	c.331T>A	P.C111S
SH_119	1	15684631	15684631	Missense_Mutation	SNV	A	T	T	FHAD1	c.2522A>T	P.E841V
SH_119	1	156882027	156882027	Splice_Site	SNV	T	A	A	PEAR1	c.1952-12T>A	
SH_119	1	157067737	157067737	Missense_Mutation	SNV	T	A	A	ETV3L	c.530A>T	P.Q177L
SH_119	1	157488251	157488251	Missense_Mutation	SNV	A	T	T	FCRL5	c.2770T>A	P.Y924N
SH_119	1	158389916	158389916	Silent	SNV	A	T	T	OR10K2	c.741T>A	P.I247I
SH_119	1	158576331	158576331	Missense_Mutation	SNV	T	C	C	OR10Z1	c.103T>C	P.Y35H
SH_119	1	159015243	159015243	Missense_Mutation	SNV	T	A	A	IFI16	c.1318T>A	P.F440I
SH_119	1	159842349	159842349	Missense_Mutation	SNV	T	A	A	CFAP45	c.1582A>T	P.T528S
SH_119	1	159856340	159856340	Missense_Mutation	SNV	T	A	A	CFAP45	c.728A>T	P.Q243L
SH_119	1	160523799	160523799	Missense_Mutation	SNV	T	A	A	CD84	c.526A>T	P.N176Y
SH_119	1	161024252	161024252	Missense_Mutation	SNV	A	T	T	ARHGAP30	c.440T>A	P.F147Y
SH_119	1	161165954	161165954	Splice_Site	SNV	A	T	T	ADAMTS4	c.1090+7T>A	
SH_119	1	161968022	161968022	Missense_Mutation	SNV	T	A	A	OLFML2B	c.1070A>T	P.H357L
SH_119	1	167096999	167096999	Missense_Mutation	SNV	T	A	A	DUSP27	c.2631T>A	P.D877E
SH_119	1	167487657	167487657	Missense_Mutation	SNV	A	T	T	CD247	c.46T>A	P.L16M
SH_119	1	168513158	168513158	Silent	SNV	A	T	T	XCL2	c.45T>A	P.T15T
SH_119	1	169519870	169519870	Splice_Site	SNV	A	G	G	F5	c.1396+8T>C	
SH_119	1	169564086	169564086	Nonsense_Mutation	SNV	T	A	A	SELP	c.2131A>T	P.K711*
SH_119	1	171504565	171504565	Splice_Site	SNV	A	T	T	PRRC2C	c.1868-2A>T	
SH_119	1	172417594	172417594	Missense_Mutation	SNV	T	A	A	C1orf105	c.121T>A	P.S41T
SH_119	1	175130037	175130037	Nonsense_Mutation	SNV	A	T	T	KIAA0040	c.113T>A	P.L38*
SH_119	1	175375420	175375420	Missense_Mutation	SNV	A	T	T	TNR	c.431T>A	P.L144Q
SH_119	1	176564581	176564581	Missense_Mutation	SNV	T	A	A	PAPPA2	c.1841T>A	P.L614Q
SH_119	1	179452349	179452349	Missense_Mutation	SNV	A	T	T	AXDND1	c.2084A>T	P.Q695L
SH_119	1	179815404	179815404	Missense_Mutation	SNV	T	A	A	TOR1AIP2	c.1215A>T	P.L405F
SH_119	1	181546907	181546907	Missense_Mutation	SNV	T	A	A	CACNA1E	c.518T>A	P.L173Q
SH_119	1	183201909	183201909	Nonsense_Mutation	SNV	C	T	T	LAMC2	c.2137C>T	P.Q713*
SH_119	1	183208627	183208627	Missense_Mutation	SNV	G	A	A	LAMC2	c.2998G>A	P.A1000T
SH_119	1	183885704	183885704	Nonsense_Mutation	SNV	A	T	T	RGL1	c.1867A>T	P.K623*
SH_119	1	185932990	185932990	Silent	SNV	T	A	A	HMCN1	c.2061T>A	P.A687A
SH_119	1	185951443	185951443	Silent	SNV	A	T	T	HMCN1	c.2712A>T	P.G904G
SH_119	1	185953438	185953438	Silent	SNV	T	A	A	HMCN1	c.2928T>A	P.V976V
SH_119	1	185964041	185964041	Silent	SNV	T	A	A	HMCN1	c.3600T>A	P.P1200P
SH_119	1	185966618	185966618	Missense_Mutation	SNV	T	A	A	HMCN1	c.3836T>A	P.V1279E
SH_119	1	186287674	186287674	Silent	SNV	T	A	A	TPR	c.6726A>T	P.P2242P
SH_119	1	186646003	186646003	Nonsense_Mutation	SNV	T	A	A	PTGS2	c.685A>T	P.K229*
SH_119	1	19018446	19018446	Missense_Mutation	SNV	A	T	T	PAX7	c.785A>T	P.Q262L
SH_119	1	192985494	192985494	Missense_Mutation	SNV	T	A	A	UCHL5	c.974A>T	P.Q325L
SH_119	1	197094228	197094228	Silent	SNV	G	A	A	ASPM	c.3030C>T	P.D1010D
SH_119	1	197890658	197890658	Missense_Mutation	SNV	A	T	T	LHX9	c.575A>T	P.Y192F
SH_119	1	200529913	200529913	Silent	SNV	T	A	A	KIF14	c.2694A>T	P.A898A
SH_119	1	201038314	201038314	Missense_Mutation	SNV	T	A	A	CACNA1S	c.2501A>T	P.H834L
SH_119	1	201181196	201181196	Missense_Mutation	SNV	A	G	G	IGFN1	c.7175A>G	P.Y2392C
SH_119	1	203466094	203466094	Splice_Site	SNV	T	A	A	OPTC	c.232-11T>A	
SH_119	1	203689873	203689873	Splice_Site	SNV	A	T	T	ATP2B4	c.2598+5A>T	
SH_119	1	204197873	204197873	Silent	SNV	T	A	A	PLEKHA6	c.2868A>T	P.T956T
SH_119	1	205899115	205899115	Missense_Mutation	SNV	T	A	A	SLC26A9	c.622A>T	P.M208L
SH_119	1	207252304	207252304	Silent	SNV	A	T	T	PFKFB2	c.1356A>T	P.A452A
SH_119	1	213139656	213139656	Missense_Mutation	SNV	A	T	T	VASH2	c.271A>T	P.I91F
SH_119	1	223176096	223176096	Missense_Mutation	SNV	A	T	T	DISP1	c.1357A>T	P.M453L
SH_119	1	223178411	223178411	Silent	SNV	A	T	T	DISP1	c.3672A>T	P.A1224A
SH_119	1	224495722	224495722	Missense_Mutation	SNV	T	A	A	NVL	c.268A>T	P.N90Y
SH_119	1	226420236	226420236	Missense_Mutation	SNV	T	A	A	LIN9	c.1538A>T	P.H513L
SH_119	1	228525739	228525739	Missense_Mutation	SNV	T	A	A	OBSCN	c.16895T>A	P.L5632Q

SH_119	1	22902927	22902927	Missense_Mutation	SNV	A	T	T	EPHA8	c.377A>T	P.Y126F
SH_119	1	22919920	22919920	Missense_Mutation	SNV	T	A	A	EPHA8	c.1417T>A	P.Y473N
SH_119	1	229763428	229763428	Silent	SNV	A	T	T	URB2	c.48A>T	P.T16T
SH_119	1	229768107	229768107	Missense_Mutation	SNV	A	T	T	URB2	c.218A>T	P.Y73F
SH_119	1	231344750	231344750	Missense_Mutation	SNV	A	T	T	TRIM67	c.1877A>T	P.D626V
SH_119	1	232650879	232650879	Silent	SNV	T	A	A	SIPA1L2	c.207A>T	P.P69P
SH_119	1	235409824	235409824	Splice_Site	SNV	T	C	C	ARID4B	c.357A>G	P.T119T
SH_119	1	236746148	236746148	Missense_Mutation	SNV	T	A	A	HEATR1	c.2450A>T	P.Q817L
SH_119	1	236760210	236760210	Missense_Mutation	SNV	T	A	A	HEATR1	c.670A>T	P.I224L
SH_119	1	236899908	236899908	Silent	SNV	A	T	T	ACTN2	c.99A>T	P.P33P
SH_119	1	238048720	238048720	Missense_Mutation	SNV	A	T	T	ZP4	c.1131T>A	P.S377R
SH_119	1	243328876	243328876	Missense_Mutation	SNV	T	A	A	CEP170	c.2092A>T	P.T698S
SH_119	1	243778465	243778465	Splice_Site	SNV	T	C	C	AKT3	c.562-2A>G	
SH_119	1	247264327	247264327	Silent	SNV	T	A	A	ZNF669	c.486A>T	P.P162P
SH_119	1	247875399	247875399	Missense_Mutation	SNV	T	C	C	OR6F1	c.659A>G	P.Y220C
SH_119	1	248903065	248903065	Silent	SNV	A	T	T	LYPD8	c.87T>A	P.T29T
SH_119	1	24981391	24981391	Silent	SNV	C	A	A	SRRM1	c.1086C>A	P.S362S
SH_119	1	26392813	26392813	Missense_Mutation	SNV	T	G	G	TRIM63	c.278A>C	P.Q93P
SH_119	1	26515998	26515998	Missense_Mutation	SNV	C	T	T	CNKSR1	c.2122C>T	P.P708S
SH_119	1	26694142	26694142	Silent	SNV	T	A	A	ZNF683	c.261A>T	P.P87P
SH_119	1	29070398	29070398	Missense_Mutation	SNV	A	T	T	YTHDF2	c.1466A>T	P.Q489L
SH_119	1	31230577	31230577	Missense_Mutation	SNV	A	T	T	LAPTM5	c.16T>A	P.S6T
SH_119	1	34673816	34673816	Missense_Mutation	SNV	T	A	A	C1orf94	c.1505T>A	P.L502Q
SH_119	1	35447612	35447612	Missense_Mutation	SNV	T	A	A	TMEM35B	c.398A>T	P.Q133L
SH_119	1	46472002	46472002	Silent	SNV	A	T	T	MAST2	c.837A>T	P.T279T
SH_119	1	47548087	47548087	Missense_Mutation	SNV	T	G	G	CYP4Z1	c.446T>G	P.L149R
SH_119	1	55119552	55119552	Missense_Mutation	SNV	A	T	T	MROH7	c.953A>T	P.N318I
SH_119	1	55152062	55152062	Silent	SNV	C	T	T	MROH7	c.2652C>T	P.C884C
SH_119	1	57349294	57349294	Silent	SNV	A	T	T	C8A	c.795A>T	P.V265V
SH_119	1	59922760	59922760	Splice_Site	SNV	A	T	T	FGGY	c.670+12A>T	
SH_119	1	6214762	6214762	Missense_Mutation	SNV	G	T	T	CHD5	c.703C>A	P.Q235K
SH_119	1	64643545	64643545	Silent	SNV	T	A	A	ROR1	c.1821T>A	P.S607S
SH_119	1	6522970	6522970	Missense_Mutation	SNV	A	T	T	TNFRSF25	c.659T>A	P.L220Q
SH_119	1	79383640	79383640	Silent	SNV	A	C	C	ADGRL4	c.1557T>G	P.V519V
SH_119	1	85570272	85570272	Silent	SNV	A	T	T	WDR63	c.1428A>T	P.S476S
SH_119	1	89659026	89659026	Missense_Mutation	SNV	T	A	A	GBP4	c.433A>T	P.S145C
SH_119	1	95294069	95294069	Missense_Mutation	SNV	A	T	T	SLC44A3	c.436A>T	P.S146C
SH_119	1	9992936	9992936	Silent	SNV	T	A	A	LZIC	c.357A>T	P.V119V
SH_119	10	100242464	100242464	Silent	SNV	A	T	T	HPSE2	c.1368T>A	P.A456A
SH_119	10	102088970	102088970	Missense_Mutation	SNV	A	T	T	PKD2L1	c.206T>A	P.L69Q
SH_119	10	102510522	102510522	Missense_Mutation	SNV	T	A	A	PAX2	c.284T>A	P.V95E
SH_119	10	105838347	105838347	Splice_Site	SNV	A	T	T	COL17A1	c.53-3T>A	
SH_119	10	112838295	112838295	Missense_Mutation	SNV	T	A	A	ADRA2A	c.541T>A	P.F181I
SH_119	10	120809466	120809466	Silent	SNV	T	A	A	EIF3A	c.2505A>T	P.A835A
SH_119	10	124399886	124399886	Missense_Mutation	SNV	A	T	T	DMBT1	c.6883A>T	P.T2295S
SH_119	10	126507985	126507985	Missense_Mutation	SNV	A	G	G	FAM175B	c.233A>G	P.E78G
SH_119	10	135027563	135027563	Silent	SNV	A	G	G	KNDC1	c.4614A>G	P.L1538L
SH_119	10	15014579	15014579	Missense_Mutation	SNV	A	T	T	MEIG1	c.206A>T	P.Y69F
SH_119	10	21962788	21962788	Missense_Mutation	SNV	A	T	T	MLLT10	c.1561A>T	P.T521S
SH_119	10	23326397	23326397	Nonsense_Mutation	SNV	A	T	T	ARMC3	c.2587A>T	P.R863*
SH_119	10	25885621	25885621	Missense_Mutation	SNV	A	G	G	GPR158	c.2048A>G	P.Y683C
SH_119	10	29812662	29812662	Missense_Mutation	SNV	T	A	A	SVIL	c.1951A>T	P.S651C
SH_119	10	43291974	43291974	Missense_Mutation	SNV	C	T	T	BMS1	c.1282C>T	P.R428C
SH_119	10	43656042	43656042	Missense_Mutation	SNV	A	T	T	CSGALNACT2	c.979A>T	P.S327C
SH_119	10	46272760	46272760	Missense_Mutation	SNV	A	T	T	FAM21C	c.2182A>T	P.S728C
SH_119	10	46968575	46968575	Splice_Site	SNV	A	T	T	SYT15	c.355+6T>A	
SH_119	10	51769531	51769531	Missense_Mutation	SNV	A	T	T	AGAP6	c.1646A>T	P.Q549L
SH_119	10	55626482	55626482	Missense_Mutation	SNV	T	A	A	PCDH15	c.3652A>T	P.M1218L
SH_119	10	74994592	74994592	Missense_Mutation	SNV	C	T	T	FAM149B1	c.1370C>T	P.S457L
SH_119	10	75407400	75407400	Silent	SNV	A	T	T	SYNPO2L	c.2010T>A	P.S670S
SH_119	10	7682717	7682717	Missense_Mutation	SNV	C	A	A	ITIH5	c.401G>T	P.G134V
SH_119	10	7763739	7763739	Missense_Mutation	SNV	A	T	T	ITIH2	c.866A>T	P.E289V
SH_119	10	90568136	90568136	Missense_Mutation	SNV	A	T	T	LIPM	c.229A>T	P.R77W
SH_119	11	100819186	100819186	Splice_Site	SNV	A	T	T	ARHGAP42	c.1044-2A>T	
SH_119	11	100836393	100836393	Splice_Site	SNV	A	T	T	ARHGAP42	c.1445-9A>T	
SH_119	11	100999800	100999800	Nonsense_Mutation	SNV	A	T	T	PGR	c.2T>A	P.M1?
SH_119	11	102593382	102593382	Missense_Mutation	SNV	T	A	A	MMP8	c.56A>T	P.Q19L
SH_119	11	103070122	103070122	Missense_Mutation	SNV	A	T	T	DYNC2H1	c.8005A>T	P.S2669C
SH_119	11	107965721	107965721	Splice_Site	SNV	A	T	T	CUL5	c.1743+7A>T	
SH_119	11	108536020	108536020	Missense_Mutation	SNV	A	T	T	DDX10	c.140A>T	P.Q47L
SH_119	11	108593923	108593923	Missense_Mutation	SNV	G	A	A	DDX10	c.1699G>A	P.E567K
SH_119	11	111368725	111368725	Nonsense_Mutation	SNV	A	T	T	BTG4	c.309T>A	P.C103*
SH_119	11	111915958	111915958	Splice_Site	SNV	A	T	T	DLAT	c.1290+4A>T	
SH_119	11	113628549	113628549	Missense_Mutation	SNV	T	A	A	ZW10	c.760A>T	P.R254W
SH_119	11	1151627	1151627	Missense_Mutation	SNV	A	T	T	MUC5AC	c.1A>T	P.M1?
SH_119	11	116633571	116633571	Missense_Mutation	SNV	T	A	A	BUD13	c.734A>T	P.H245L
SH_119	11	117329461	117329461	Splice_Site	SNV	T	A	A	DSCAML1	c.3748+9A>T	
SH_119	11	117866339	117866339	Missense_Mutation	SNV	T	A	A	IL10RA	c.724T>A	P.F242I
SH_119	11	119043108	119043108	Missense_Mutation	SNV	A	T	T	NLRX1	c.114A>T	P.Q38H
SH_119	11	124761432	124761432	Missense_Mutation	SNV	C	T	T	ROBO4	c.1276G>A	P.G426S
SH_119	11	128933805	128933805	Missense_Mutation	SNV	T	A	A	ARHGAP32	c.835A>T	P.T279S
SH_119	11	130777872	130777872	Missense_Mutation	SNV	T	A	A	SNX19	c.281A>T	P.Q94L
SH_119	11	134238601	134238601	Missense_Mutation	SNV	A	T	T	GLB1L2	c.953A>T	P.H318L
SH_119	11	18743106	18743106	Silent	SNV	G	A	A	IGSF22	c.354C>T	P.S118S
SH_119	11	1908759	1908759	Missense_Mutation	SNV	A	T	T	LSP1	c.800A>T	P.E267V
SH_119	11	20005673	20005673	Missense_Mutation	SNV	A	T	T	NAV2	c.2456A>T	P.Y819F
SH_119	11	2444225	2444225	Missense_Mutation	SNV	A	T	T	TRPM5	c.42T>A	P.D14E
SH_119	11	2593278	2593278	Missense_Mutation	SNV	A	T	T	KCNQ1	c.719A>T	P.H240L
SH_119	11	35453944	35453944	Missense_Mutation	SNV	A	T	T	PAMR1	c.2123T>A	P.V708E
SH_119	11	35453945	35453945	Missense_Mutation	SNV	C	T	T	PAMR1	c.2122G>A	P.V708M
SH_119	11	36397717	36397717	Splice_Site	SNV	A	T	T	PRR5L	c.2+9A>T	
SH_119	11	3714478	3714478	Missense_Mutation	SNV	T	A	A	NUP98	c.4295A>T	P.Y1432F
SH_119	11	43427103	43427103	Missense_Mutation	SNV	A	T	T	TTC17	c.1519A>T	P.T507S
SH_119	11	44939571	44939571	Missense_Mutation	SNV	A	T	T	TSPAN18	c.307A>T	P.I103F
SH_119	11	46392894	46392894	Missense_Mutation	SNV	A	T	T	DGKZ	c.1166A>T	P.K389M
SH_119	11	47609903	47609903	Missense_Mutation	SNV	T	A	A	FAM180B	c.469T>A	P.W157R
SH_119	11	5067880	5067880	Missense_Mutation	SNV	T	A	A	OR52J3	c.125T>A	P.L42Q
SH_119	11	51515748	51515748	Missense_Mutation	SNV	T	A	A	OR4C46	c.467T>A	P.I156K

SH_119	11	51515749	51515749	Silent	SNV	A	C	C	OR4C46	c.468A>C	P.I156I
SH_119	11	55659015	55659015	Missense_Mutation	SNV	T	A	A	TRIM51	c.1266T>A	P.F422L
SH_119	11	56000335	56000335	Missense_Mutation	SNV	A	C	C	OR5T2	c.327T>G	P.D109E
SH_119	11	61026245	61026245	Missense_Mutation	SNV	T	A	A	VWCE	c.2770A>T	P.T924S
SH_119	11	6261397	6261397	Missense_Mutation	SNV	A	T	T	CNGA4	c.373A>T	P.T125S
SH_119	11	64022882	64022882	Missense_Mutation	SNV	A	T	T	PLCB3	c.482A>T	P.K161M
SH_119	11	6477326	6477326	Silent	SNV	T	A	A	TRIM3	c.1509A>T	P.A503A
SH_119	11	66358745	66358745	Missense_Mutation	SNV	T	A	A	CCDC87	c.1742A>T	P.K581M
SH_119	11	68030220	68030220	Silent	SNV	T	A	A	C11orf24	c.243A>T	P.T81T
SH_119	11	68171171	68171171	Splice_Site	SNV	A	T	T	LRP5	c.-137+4A>T	
SH_119	11	73941339	73941339	Missense_Mutation	SNV	A	T	T	PPME1	c.448A>T	P.I150F
SH_119	11	76922906	76922906	Missense_Mutation	SNV	A	T	T	MYO7A	c.6278A>T	P.K2093M
SH_119	11	77702289	77702289	Silent	SNV	T	A	A	INTS4	c.111A>T	P.A37A
SH_119	11	8462246	8462246	Missense_Mutation	SNV	A	T	T	STK33	c.803T>A	P.I268K
SH_119	11	88045643	88045643	Missense_Mutation	SNV	T	A	A	CTSC	c.398A>T	P.N133I
SH_119	11	88911873	88911873	Missense_Mutation	SNV	A	T	T	TYR	c.752A>T	P.Y251F
SH_119	11	89911120	89911120	Missense_Mutation	SNV	T	A	A	NAALAD2	c.1594T>A	P.S532T
SH_119	11	92533570	92533570	Missense_Mutation	SNV	T	A	A	FAT3	c.7391T>A	P.L2464Q
SH_119	11	93528081	93528081	Silent	SNV	A	T	T	MED17	c.867A>T	P.P289P
SH_119	11	93819280	93819280	Nonsense_Mutation	SNV	C	T	T	HEPHL1	c.2005C>T	P.R669*
SH_119	11	94126683	94126683	Silent	SNV	A	T	T	GPR83	c.615T>A	P.A205A
SH_119	12	100904910	100904910	Missense_Mutation	SNV	A	T	T	NR1H4	c.434A>T	P.E145V
SH_119	12	104118860	104118860	Silent	SNV	C	A	A	STAB2	c.4791C>A	P.G1597G
SH_119	12	104118861	104118861	Missense_Mutation	SNV	A	G	G	STAB2	c.4792A>G	P.S1598G
SH_119	12	105322003	105322003	Silent	SNV	T	A	A	SLC41A2	c.303A>T	P.A101A
SH_119	12	10586504	10586504	Silent	SNV	T	C	C	KLRC2	c.369A>G	P.T123T
SH_119	12	10774505	10774505	Missense_Mutation	SNV	A	T	T	STYK1	c.1034T>A	P.M345K
SH_119	12	109046121	109046121	Missense_Mutation	SNV	T	A	A	CORO1C	c.1087A>T	P.S363C
SH_119	12	109870803	109870803	Missense_Mutation	SNV	A	T	T	MYO1H	c.2003A>T	P.Y668F
SH_119	12	109968357	109968357	Missense_Mutation	SNV	A	T	T	UBE3B	c.2816A>T	P.H939L
SH_119	12	112182565	112182565	Silent	SNV	A	T	T	ACAD10	c.1926A>T	P.T642T
SH_119	12	116408484	116408484	Silent	SNV	T	A	A	MED13L	c.5982A>T	P.T1994T
SH_119	12	120613934	120613934	Splice_Site	SNV	T	A	A	GCN1	c.913+12A>T	
SH_119	12	120613935	120613935	Splice_Site	SNV	G	T	T	GCN1	c.913+11C>A	
SH_119	12	122818637	122818637	Nonsense_Mutation	SNV	G	A	A	CLIP1	c.2647C>T	P.Q883*
SH_119	12	123472453	123472453	Splice_Site	SNV	G	A	A	PITPNM2	c.2980-5C>T	
SH_119	12	123651251	123651251	Missense_Mutation	SNV	T	A	A	MPHOSPH9	c.2542A>T	P.T848S
SH_119	12	124840022	124840022	Nonsense_Mutation	SNV	T	A	A	NCOR2	c.3307A>T	P.K1103*
SH_119	12	125441289	125441289	Missense_Mutation	SNV	T	A	A	DHX37	c.2401A>T	P.I801F
SH_119	12	133424751	133424751	Splice_Site	SNV	T	A	A	CHFR	c.1613-10A>T	
SH_119	12	14976470	14976470	Missense_Mutation	SNV	A	G	G	C12orf60	c.601A>G	P.K201E
SH_119	12	2027580	2027580	Silent	SNV	T	A	A	CACNA2D4	c.60A>T	P.A20A
SH_119	12	22627686	22627686	Missense_Mutation	SNV	T	A	A	C2CD5	c.2048A>T	P.E683V
SH_119	12	25157405	25157405	Missense_Mutation	SNV	A	T	T	LOC645177	c.130A>T	P.S44C
SH_119	12	25243079	25243079	Nonsense_Mutation	SNV	T	A	A	LRMP	c.554T>A	P.L185*
SH_119	12	2760876	2760876	Missense_Mutation	SNV	T	A	A	CACNA1C	c.4016T>A	P.L1339Q
SH_119	12	32134470	32134470	Missense_Mutation	SNV	A	T	T	KIAA1551	c.581A>T	P.Q194L
SH_119	12	3726177	3726177	Missense_Mutation	SNV	T	A	A	CRACR2A	c.2054A>T	P.Y685F
SH_119	12	40441900	40441900	Silent	SNV	T	A	A	SLC2A13	c.669A>T	P.A223A
SH_119	12	416623	416623	Missense_Mutation	SNV	T	A	A	KDM5A	c.3927A>T	P.L1309F
SH_119	12	45168561	45168561	Nonsense_Mutation	SNV	A	T	T	NELL2	c.1113T>A	P.Y371*
SH_119	12	46592528	46592528	Splice_Site	SNV	T	A	A	SLC38A1	c.1004-2A>T	
SH_119	12	4835747	4835747	Silent	SNV	A	T	T	GALNT8	c.261A>T	P.T87T
SH_119	12	48888578	48888578	Splice_Site	SNV	C	T	T	C12orf54	c.243-3C>T	
SH_119	12	49217252	49217252	Splice_Site	SNV	A	C	C	CACNB3	c.168+3A>C	
SH_119	12	49521873	49521873	Silent	SNV	G	A	A	TUBA1B	c.1224C>T	P.Y408Y
SH_119	12	49959442	49959442	Missense_Mutation	SNV	T	A	A	MCRS1	c.197A>T	P.K66M
SH_119	12	50757002	50757002	Missense_Mutation	SNV	A	G	G	FAM186A	c.338T>C	P.M113T
SH_119	12	52758778	52758779	Frame_Shift_Del	INDEL	AT	-	-	KRT85	c.596_597delAT	P.H199fs
SH_119	12	53007614	53007614	Missense_Mutation	SNV	T	A	A	KRT73	c.842A>T	P.H281L
SH_119	12	53412590	53412590	Missense_Mutation	SNV	A	T	T	EIF4B	c.160A>T	P.T54S
SH_119	12	53686114	53686114	Missense_Mutation	SNV	A	G	G	ESPL1	c.5849A>G	P.Y1950C
SH_119	12	53722674	53722674	Silent	SNV	T	A	A	SP7	c.552A>T	P.T184T
SH_119	12	53946413	53946413	Silent	SNV	T	A	A	ATF7	c.57A>T	P.T19T
SH_119	12	54905728	54905728	Splice_Site	SNV	T	A	A	NCKAP1L	c.635-5T>A	
SH_119	12	55846616	55846616	Missense_Mutation	SNV	A	T	T	OR6C2	c.619A>T	P.I207F
SH_119	12	57009268	57009268	Missense_Mutation	SNV	T	A	A	BAZ2A	c.260A>T	P.Y87F
SH_119	12	57397765	57397765	Missense_Mutation	SNV	T	A	A	ZBTB39	c.937A>T	P.I313L
SH_119	12	57600268	57600268	Missense_Mutation	SNV	A	T	T	LRP1	c.11603A>T	P.Q3868L
SH_119	12	57677748	57677748	Missense_Mutation	SNV	T	A	A	R3HDM2	c.988A>T	P.S330C
SH_119	12	57882876	57882876	Missense_Mutation	SNV	A	T	T	MARS	c.184A>T	P.T62S
SH_119	12	58024298	58024298	Missense_Mutation	SNV	A	T	T	B4GALNT1	c.329T>A	P.L110Q
SH_119	12	5841688	5841688	Nonsense_Mutation	SNV	C	A	A	ANO2	c.1558G>T	P.E520*
SH_119	12	66345189	66345189	Silent	SNV	T	A	A	HMGA2	c.276T>A	P.P92P
SH_119	12	6666280	6666280	Missense_Mutation	SNV	T	A	A	NOP2	c.2306A>T	P.Q769L
SH_119	12	66715758	66715758	Missense_Mutation	SNV	A	T	T	HELB	c.2186A>T	P.Q729L
SH_119	12	70048843	70048843	Silent	SNV	A	T	T	BEST3	c.1533T>A	P.L511L
SH_119	12	7047640	7047640	Splice_Site	SNV	A	T	T	ATN1	c.2518-4A>T	
SH_119	12	70928618	70928618	Missense_Mutation	SNV	A	T	T	PTPRB	c.6199T>A	P.C2067S
SH_119	12	7970499	7970499	Silent	SNV	A	T	T	SLC2A14	c.1272T>A	P.A424A
SH_119	12	85446008	85446008	Missense_Mutation	SNV	A	T	T	LRRIQ1	c.732A>T	P.K244N
SH_119	12	85466830	85466830	Silent	SNV	A	T	T	LRRIQ1	c.2841A>T	P.T947T
SH_119	12	85547816	85547816	Missense_Mutation	SNV	A	C	C	LRRIQ1	c.4664A>C	P.Q1555P
SH_119	12	89917772	89917772	Missense_Mutation	SNV	T	A	A	POC1B-GALNT4	c.546A>T	P.Q182H
SH_119	12	91348357	91348357	Missense_Mutation	SNV	T	A	A	CCER1	c.163A>T	P.S55C
SH_119	12	95443013	95443013	Splice_Site	SNV	T	A	A	NR2C1	c.966-4A>T	
SH_119	12	95926982	95926982	Missense_Mutation	SNV	T	A	A	USP44	c.1051A>T	P.S351C
SH_119	12	96974418	96974418	Missense_Mutation	SNV	A	T	T	CFAP54	c.2840A>T	P.Y947F
SH_119	12	970386	970386	Missense_Mutation	SNV	A	T	T	WNK1	c.1828A>T	P.S610C
SH_119	12	99638169	99638169	Missense_Mutation	SNV	T	C	C	ANKS1B	c.2370A>G	P.I790M
SH_119	13	103389773	103389773	Missense_Mutation	SNV	T	A	A	CCDC168	c.13274A>T	P.K4425I
SH_119	13	107209408	107209408	Silent	SNV	T	A	A	ARGLU1	c.645A>T	P.A215A
SH_119	13	107822921	107822921	Nonsense_Mutation	SNV	G	T	T	FAM155A	c.1301C>A	P.S434*
SH_119	13	108861215	108861215	Missense_Mutation	SNV	T	C	C	LIG4	c.2402A>G	P.Y801C
SH_119	13	111102119	111102119	Missense_Mutation	SNV	T	A	A	COL4A2	c.1172T>A	P.L391H
SH_119	13	21417069	21417069	Missense_Mutation	SNV	T	A	A	XPO4	c.692A>T	P.N231I
SH_119	13	23929590	23929590	Silent	SNV	T	A	A	SACS	c.720A>T	P.A240A
SH_119	13	25008540	25008540	Missense_Mutation	SNV	T	A	A	PARP4	c.4739A>T	P.Q1580L

SH_119	13	25281243	25281243	Missense_Mutation	SNV	T	A	A	ATP12A	c.2270T>A	P.I757K
SH_119	13	25399879	25399879	Missense_Mutation	SNV	A	T	T	RNF17	c.2214A>T	P.E738D
SH_119	13	25671186	25671186	Missense_Mutation	SNV	A	C	C	PABPC3	c.850A>C	P.K284Q
SH_119	13	25671389	25671389	Silent	SNV	A	T	T	PABPC3	c.1053A>T	P.T351T
SH_119	13	28623522	28623522	Splice_Site	SNV	T	A	A	FLT3	c.1035A>T	P.V345V
SH_119	13	30346253	30346253	Missense_Mutation	SNV	T	A	A	UBL3	c.219A>T	P.L73F
SH_119	13	33347443	33347443	Missense_Mutation	SNV	A	T	T	PDS5B	c.4289A>T	P.E1430V
SH_119	13	39262527	39262527	Missense_Mutation	SNV	A	G	G	FREM2	c.1046A>G	P.E349G
SH_119	13	39265875	39265875	Missense_Mutation	SNV	T	A	A	FREM2	c.4394T>A	P.M1465K
SH_119	13	46170688	46170688	Nonsense_Mutation	SNV	A	T	T	ERICH6B	c.453T>A	P.Y151*
SH_119	13	52293340	52293340	Missense_Mutation	SNV	A	T	T	WDFY2	c.341A>T	P.Q114L
SH_119	13	95899894	95899894	Splice_Site	SNV	T	A	A	ABCC4	c.185+3A>T	
SH_119	14	100002353	100002353	Missense_Mutation	SNV	T	A	A	CCDC85C	c.866A>T	P.Q289L
SH_119	14	100317234	100317234	Missense_Mutation	SNV	A	G	G	EML1	c.112A>G	P.I38V
SH_119	14	100808791	100808791	Missense_Mutation	SNV	T	A	A	WARS	c.1057A>T	P.S353C
SH_119	14	100950389	100950389	Silent	SNV	A	G	G	WDR25	c.1029A>G	P.P343P
SH_119	14	102568340	102568340	Missense_Mutation	SNV	A	T	T	HSP90AA1	c.238T>A	P.C80S
SH_119	14	104470604	104470604	Missense_Mutation	SNV	T	A	A	TDRD9	c.1513T>A	P.C505S
SH_119	14	19571376	19571376	Missense_Mutation	SNV	A	T	T	POTEG	c.1155A>T	P.E385D
SH_119	14	19574260	19574260	Silent	SNV	T	A	A	POTEG	c.1317T>A	P.A439A
SH_119	14	20837522	20837522	Missense_Mutation	SNV	T	A	A	TEP1	c.7313A>T	P.K2438M
SH_119	14	21215769	21215769	Silent	SNV	A	T	T	EDDM3A	c.30A>T	P.I10I
SH_119	14	21547147	21547147	Missense_Mutation	SNV	A	T	T	ARHGEF40	c.209A>T	P.Q70L
SH_119	14	23898291	23898291	Missense_Mutation	SNV	A	T	T	MYH7	c.1280T>A	P.L427Q
SH_119	14	24614592	24614592	Missense_Mutation	SNV	T	A	A	PSME2	c.227A>T	P.D76V
SH_119	14	24884675	24884675	Silent	SNV	G	A	A	NYNRIN	c.3720G>A	P.A1240A
SH_119	14	31388185	31388185	Silent	SNV	T	A	A	STRN3	c.1227A>T	P.A409A
SH_119	14	36337141	36337141	Missense_Mutation	SNV	A	T	T	BRMS1L	c.757A>T	P.S253C
SH_119	14	52520946	52520946	Silent	SNV	A	T	T	NID2	c.861T>A	P.L287L
SH_119	14	58605793	58605793	Missense_Mutation	SNV	T	A	A	C14orf37	c.284A>T	P.E95V
SH_119	14	64542703	64542703	Missense_Mutation	SNV	A	T	T	SYNE2	c.10907A>T	P.E3636V
SH_119	14	64634290	64634290	Missense_Mutation	SNV	A	T	T	SYNE2	c.16838A>T	P.E5613V
SH_119	14	64898591	64898591	Splice_Site	SNV	T	G	G	MTHFD1	c.1494+12T>G	
SH_119	14	65208994	65208994	Missense_Mutation	SNV	A	T	T	PLEKHG3	c.2759A>T	P.E920V
SH_119	14	74824304	74824304	Missense_Mutation	SNV	T	A	A	VRTN	c.818T>A	P.L273Q
SH_119	14	76644365	76644365	Missense_Mutation	SNV	A	T	T	GPATCH2L	c.1183A>T	P.I395F
SH_119	14	79181183	79181183	Missense_Mutation	SNV	T	A	A	NRXN3	c.626T>A	P.L209Q
SH_119	14	81965866	81965866	Splice_Site	SNV	T	A	A	SEL1L	c.832-4A>T	
SH_119	14	86089332	86089332	Missense_Mutation	SNV	T	A	A	FLRT2	c.1474T>A	P.C492S
SH_119	14	88693819	88693819	Missense_Mutation	SNV	A	T	T	KCNK10	c.566T>A	P.I189N
SH_119	14	89154632	89154632	Missense_Mutation	SNV	T	A	A	EML5	c.2725A>T	P.M909L
SH_119	14	90429693	90429693	Missense_Mutation	SNV	A	T	T	TDP1	c.235A>T	P.S79C
SH_119	14	91633583	91633583	Missense_Mutation	SNV	A	T	T	C14orf159	c.118A>T	P.S40C
SH_119	14	95669729	95669729	Nonsense_Mutation	SNV	T	A	A	CLMN	c.1957A>T	P.K653*
SH_119	15	23283157	23283157	Splice_Site	SNV	T	A	A	HERC2P2	n.5187-4A>T	
SH_119	15	33443550	33443550	Missense_Mutation	SNV	T	A	A	FMN1	c.1898A>T	P.Q633L
SH_119	15	33835859	33835859	Missense_Mutation	SNV	A	T	T	RYR3	c.683A>T	P.H228L
SH_119	15	33905481	33905481	Silent	SNV	A	T	T	RYR3	c.2262A>T	P.S754S
SH_119	15	35084333	35084333	Missense_Mutation	SNV	G	T	T	ACTC1	c.766C>A	P.R256S
SH_119	15	42162648	42162648	Missense_Mutation	SNV	A	T	T	SPTBN5	c.5558T>A	P.V1853D
SH_119	15	42167176	42167176	Missense_Mutation	SNV	T	A	A	SPTBN5	c.4366A>T	P.R1456W
SH_119	15	43307939	43307940	Frame_Shift_Del	INDEL	AT	-	-	UBR1	.3155_3156delA	P.Y1052fs
SH_119	15	43446997	43446997	Missense_Mutation	SNV	A	T	T	TMEM62	c.1150A>T	P.S384C
SH_119	15	43724410	43724410	Silent	SNV	T	A	A	TP53BP1	c.3657A>T	P.T1219T
SH_119	15	43827419	43827419	Missense_Mutation	SNV	T	A	A	PPIP5K1	c.3755A>T	P.E1252V
SH_119	15	44913948	44913948	Splice_Site	SNV	C	A	A	SPG11	c.2620+9G>T	
SH_119	15	44943729	44943729	Silent	SNV	T	A	A	SPG11	c.1416A>T	P.V472V
SH_119	15	45399031	45399031	Missense_Mutation	SNV	T	A	A	DUOX2	c.1830A>T	P.L610F
SH_119	15	49301469	49301469	Silent	SNV	T	A	A	SECISBP2L	c.1971A>T	P.I657I
SH_119	15	50154546	50154546	Nonsense_Mutation	SNV	T	A	A	ATP8B4	c.3193A>T	P.K1065*
SH_119	15	52529710	52529710	Missense_Mutation	SNV	T	A	A	MYO5C	c.2837A>T	P.K946M
SH_119	15	52849387	52849387	Missense_Mutation	SNV	T	A	A	ARPP19	c.135A>T	P.K45N
SH_119	15	54307454	54307454	Missense_Mutation	SNV	T	A	A	UNC13C	c.2354T>A	P.I785N
SH_119	15	54527312	54527312	Splice_Site	SNV	T	A	A	UNC13C	c.3154+2T>A	
SH_119	15	56388429	56388429	Silent	SNV	T	A	A	RFX7	c.1497A>T	P.T499T
SH_119	15	59528811	59528811	Missense_Mutation	SNV	T	A	A	MYO1E	c.393A>T	P.R131S
SH_119	15	62147130	62147130	Missense_Mutation	SNV	T	A	A	VPS13C	c.11100A>T	P.K3700N
SH_119	15	63954122	63954122	Silent	SNV	T	A	A	HERC1	c.9000A>T	P.P3000P
SH_119	15	64592676	64592676	Missense_Mutation	SNV	T	A	A	CSNK1G1	c.23A>T	P.K8M
SH_119	15	65553347	65553347	Missense_Mutation	SNV	G	A	A	PARP16	c.709C>T	P.R237C
SH_119	15	65799697	65799697	Splice_Site	SNV	T	A	A	DPP8	c.308-4A>T	
SH_119	15	72292276	72292276	Missense_Mutation	SNV	T	A	A	MYO9A	c.1386A>T	P.K462N
SH_119	15	74474721	74474721	Missense_Mutation	SNV	T	A	A	STRA6	c.1381A>T	P.R461W
SH_119	15	78841176	78841176	Nonsense_Mutation	SNV	A	T	T	PSMA4	c.676A>T	P.R226*
SH_119	15	78921777	78921777	Silent	SNV	G	T	T	CHRNB4	c.870C>A	P.L290L
SH_119	15	79183829	79183829	Missense_Mutation	SNV	C	A	A	MORF4L1	c.89C>A	P.P30H
SH_119	15	79288145	79288145	Splice_Site	SNV	A	T	T	RASGRF1	c.2967-3T>A	
SH_119	15	83725173	83725173	Missense_Mutation	SNV	T	A	A	BTBD1	c.526A>T	P.R176W
SH_119	15	83935660	83935660	Missense_Mutation	SNV	T	A	A	BNC1	c.342A>T	P.Q114H
SH_119	15	86686984	86686984	Missense_Mutation	SNV	T	A	A	AGBL1	c.170T>A	P.L57Q
SH_119	15	89381952	89381952	Silent	SNV	G	A	A	ACAN	c.129G>A	P.L43L
SH_119	15	89424819	89424819	Missense_Mutation	SNV	A	G	G	HAPLN3	c.448T>C	P.W150R
SH_119	15	92987891	92987891	Missense_Mutation	SNV	T	A	A	ST8SIA2	c.574T>A	P.Y192N
SH_119	15	99478246	99478246	Missense_Mutation	SNV	A	T	T	IGF1R	c.3150A>T	P.E1050D
SH_119	16	1262530	1262530	Splice_Site	SNV	T	A	A	CACNA1H	c.4777+2T>A	
SH_119	16	1485086	1485086	Splice_Site	SNV	T	A	A	CCDC154	c.1692+4A>T	
SH_119	16	21123137	21123137	Splice_Site	SNV	A	G	G	DNAH3	c.1921-12T>C	
SH_119	16	21262133	21262133	Missense_Mutation	SNV	A	T	T	ANKS4B	c.1246A>T	P.S416C
SH_119	16	22926576	22926576	Missense_Mutation	SNV	T	A	A	HS3ST2	c.797T>A	P.L266Q
SH_119	16	24135213	24135213	Missense_Mutation	SNV	T	A	A	PRKCB	c.976T>A	P.S326T
SH_119	16	27483142	27483142	Missense_Mutation	SNV	T	A	A	GTF3C1	c.4453A>T	P.T1485S
SH_119	16	29909302	29909302	Missense_Mutation	SNV	A	T	T	SEZ6L2	c.83T>A	P.L28Q
SH_119	16	32182377	32182377	Splice_Site	SNV	T	A	A	HERC2P4	n.1890+4A>T	
SH_119	16	396776	396776	Nonsense_Mutation	SNV	T	A	A	AXIN1	c.250A>T	P.K84*
SH_119	16	46508243	46508243	Splice_Site	SNV	T	A	A	ANKRD26P1	n.3451-2A>T	
SH_119	16	46771662	46771662	Missense_Mutation	SNV	T	A	A	MYLK3	c.962A>T	P.Q321L
SH_119	16	48167719	48167719	Missense_Mutation	SNV	A	T	T	ABCC12	c.1007T>A	P.L336Q
SH_119	16	48304150	48304150	Silent	SNV	A	T	T	LONP2	c.1074A>T	P.G358G

SH_119	16	50745688	50745688	Silent	SNV	A	T	T	NOD2	c.1785A>T	P.P595P
SH_119	16	52473506	52473506	Missense_Mutation	SNV	T	A	A	TOX3	c.1362A>T	P.Q454H
SH_119	16	55725927	55725927	Missense_Mutation	SNV	A	T	T	SLC6A2	c.881A>T	P.Y294F
SH_119	16	56443361	56443361	Missense_Mutation	SNV	T	A	A	AMFR	c.488A>T	P.Q163L
SH_119	16	56531746	56531746	Missense_Mutation	SNV	T	A	A	BBS2	c.1706A>T	P.Q569L
SH_119	16	56704477	56704477	Nonsense_Mutation	SNV	A	T	T	MT1H	c.88A>T	P.K30*
SH_119	16	56938317	56938317	Missense_Mutation	SNV	A	T	T	SLC12A3	c.2894A>T	P.Q965L
SH_119	16	57685157	57685157	Missense_Mutation	SNV	A	T	T	ADGRG1	c.110A>T	P.Q37L
SH_119	16	57758742	57758742	Missense_Mutation	SNV	A	T	T	DRC7	c.1753A>T	P.I585F
SH_119	16	67229816	67229816	Missense_Mutation	SNV	A	T	T	E2F4	c.940A>T	P.S314C
SH_119	16	67267986	67267986	Silent	SNV	A	T	T	FHOD1	c.1698T>A	P.S566S
SH_119	16	67404918	67404918	Missense_Mutation	SNV	A	T	T	LRRC36	c.904A>T	P.S302C
SH_119	16	69602396	69602396	Splice_Site	SNV	A	T	T	NFAT5	c.74-2A>T	
SH_119	16	69680984	69680984	Missense_Mutation	SNV	A	T	T	NFAT5	c.307A>T	P.T103S
SH_119	16	70998672	70998672	Missense_Mutation	SNV	T	A	A	HYDIN	c.5747A>T	P.Q1916L
SH_119	16	71009073	71009073	Missense_Mutation	SNV	A	T	T	HYDIN	c.4738T>A	P.C1580S
SH_119	16	71054141	71054141	Missense_Mutation	SNV	A	T	T	HYDIN	c.3266T>A	P.L1089Q
SH_119	16	71509706	71509706	Silent	SNV	T	A	A	ZNF19	c.744A>T	P.T248T
SH_119	16	72159863	72159863	Splice_Site	SNV	T	A	A	PMFBP1	c.1818+4A>T	
SH_119	16	72984390	72984390	Missense_Mutation	SNV	T	A	A	ZFHX3	c.3194A>T	P.E1065V
SH_119	16	72992423	72992423	Missense_Mutation	SNV	T	A	A	ZFHX3	c.1622A>T	P.Q541L
SH_119	16	75276951	75276951	Missense_Mutation	SNV	T	A	A	BCAR1	c.188A>T	P.E63V
SH_119	16	7759084	7759084	Missense_Mutation	SNV	C	A	A	RBFOX1	c.941C>A	P.P314H
SH_119	16	84516298	84516298	Missense_Mutation	SNV	A	T	T	TLDC1	c.977T>A	P.L326Q
SH_119	16	86566057	86566057	Missense_Mutation	SNV	T	A	A	MTHFSD	c.712A>T	P.I238L
SH_119	16	88502420	88502420	Missense_Mutation	SNV	G	T	T	ZNF469	c.8458G>T	P.D2820Y
SH_119	16	89713675	89713675	Missense_Mutation	SNV	T	A	A	CHMP1A	c.317A>T	P.Q106L
SH_119	16	89849452	89849452	Missense_Mutation	SNV	T	A	A	FANCA	c.1529A>T	P.Y510F
SH_119	17	10544603	10544603	Splice_Site	SNV	T	A	A	MYH3	c.2046A>T	P.P682P
SH_119	17	11786894	11786894	Splice_Site	SNV	T	A	A	DNAH9	c.10807-9T>A	
SH_119	17	16068419	16068419	Silent	SNV	T	A	A	NCOR1	c.492A>T	P.P164P
SH_119	17	17699107	17699107	Missense_Mutation	SNV	A	T	T	RAI1	c.2845A>T	P.M949L
SH_119	17	18007965	18007965	Silent	SNV	T	A	A	DRG2	c.924T>A	P.I308I
SH_119	17	20135564	20135564	Missense_Mutation	SNV	G	T	T	SPECC1	c.2197G>T	P.V733L
SH_119	17	26085914	26085914	Missense_Mutation	SNV	T	A	A	NOS2	c.3347A>T	P.Q1116L
SH_119	17	26109071	26109071	Missense_Mutation	SNV	A	G	G	NOS2	c.692T>C	P.V231A
SH_119	17	26668457	26668457	Missense_Mutation	SNV	A	T	T	TNFAIP1	c.493A>T	P.S165C
SH_119	17	26887125	26887125	Missense_Mutation	SNV	T	A	A	PIGS	c.761A>T	P.Y254F
SH_119	17	29281490	29281490	Splice_Site	SNV	T	A	A	ADAP2	c.805-6T>A	
SH_119	17	33259383	33259383	Splice_Site	SNV	T	A	A	CCT6B	c.1236+3A>T	
SH_119	17	33806597	33806597	Missense_Mutation	SNV	T	A	A	SLFN12L	c.632A>T	P.K211I
SH_119	17	33806598	33806598	Nonsense_Mutation	SNV	T	A	A	SLFN12L	c.631A>T	P.K211*
SH_119	17	34067491	34067491	Missense_Mutation	SNV	A	T	T	RASL10B	c.280A>T	P.I94F
SH_119	17	37356606	37356606	Splice_Site	SNV	A	T	T	RPL19	c.5+4A>T	
SH_119	17	39086223	39086223	Missense_Mutation	SNV	T	A	A	KRT23	c.194A>T	P.E65V
SH_119	17	39137384	39137384	Missense_Mutation	SNV	T	A	A	KRT40	c.707A>T	P.E236V
SH_119	17	39412055	39412055	Missense_Mutation	SNV	T	A	A	KRTAP9-9	c.433T>A	P.C145S
SH_119	17	39619260	39619260	Missense_Mutation	SNV	A	G	G	KRT32	c.1039T>C	P.Y347H
SH_119	17	39661506	39661506	Silent	SNV	G	A	A	KRT13	c.297C>T	P.L99L
SH_119	17	39778605	39778605	Splice_Site	SNV	A	T	T	KRT17	c.672+2T>A	
SH_119	17	39888954	39888954	Missense_Mutation	SNV	A	T	T	HAP1	c.566T>A	P.L189Q
SH_119	17	40054028	40054028	Missense_Mutation	SNV	T	A	A	ACLY	c.1403A>T	P.K468M
SH_119	17	40142271	40142271	Splice_Site	SNV	T	A	A	DNAJC7	c.431+11A>T	
SH_119	17	40344300	40344300	Missense_Mutation	SNV	T	A	A	GHDC	c.848A>T	P.Q283L
SH_119	17	42338101	42338101	Missense_Mutation	SNV	A	T	T	SLC4A1	c.251T>A	P.L84Q
SH_119	17	42739769	42739769	Missense_Mutation	SNV	T	A	A	MEIOC	c.289T>A	P.C97S
SH_119	17	43247046	43247046	Missense_Mutation	SNV	A	T	T	HEXIM2	c.797A>T	P.Q266L
SH_119	17	48243426	48243426	Missense_Mutation	SNV	C	T	T	SGCA	c.25C>T	P.P9S
SH_119	17	48646546	48646546	Missense_Mutation	SNV	T	A	A	CACNA1G	c.375T>A	P.F125L
SH_119	17	48694820	48694820	Silent	SNV	C	T	T	CACNA1G	c.4989C>T	P.A1663A
SH_119	17	49067881	49067881	Silent	SNV	T	A	A	SPAG9	c.2517A>T	P.A839A
SH_119	17	54933869	54933869	Splice_Site	SNV	A	T	T	DGKE	c.1099-4A>T	
SH_119	17	55028025	55028025	Missense_Mutation	SNV	T	A	A	COIL	c.578A>T	P.Y193F
SH_119	17	57290403	57290403	Missense_Mutation	SNV	A	T	T	SMG8	c.2219A>T	P.Q740L
SH_119	17	60768066	60768066	Missense_Mutation	SNV	A	T	T	MRC2	c.3956A>T	P.Q1319L
SH_119	17	61558959	61558959	Silent	SNV	A	T	T	ACE	c.978A>T	P.A326A
SH_119	17	61950652	61950652	Missense_Mutation	SNV	A	T	T	CSH2	c.58T>A	P.W20R
SH_119	17	62291499	62291499	Missense_Mutation	SNV	T	A	A	TEX2	c.79A>T	P.R27W
SH_119	17	63898403	63898403	Missense_Mutation	SNV	T	A	A	CEP112	c.2030A>T	P.Q677L
SH_119	17	6493836	6493836	Missense_Mutation	SNV	T	A	A	KIAA0753	c.2555A>T	P.N852I
SH_119	17	6661443	6661443	Missense_Mutation	SNV	A	T	T	XAF1	c.68A>T	P.H23L
SH_119	17	6663876	6663876	Missense_Mutation	SNV	A	T	T	XAF1	c.377A>T	P.Q126L
SH_119	17	67305544	67305544	Missense_Mutation	SNV	T	A	A	ABCA5	c.328A>T	P.T110S
SH_119	17	72356294	72356294	Missense_Mutation	SNV	T	A	A	BTBD17	c.176A>T	P.Q59L
SH_119	17	72443011	72443011	Silent	SNV	C	T	T	GPRC5C	c.1206C>T	P.I402I
SH_119	17	7246865	7246865	Missense_Mutation	SNV	T	A	A	ACAP1	c.512T>A	P.L171Q
SH_119	17	74289407	74289407	Silent	SNV	T	A	A	QRICH2	c.903A>T	P.S301S
SH_119	17	7578455	7578455	Missense_Mutation	SNV	C	G	G	TP53	c.475G>C	P.A159P
SH_119	17	76118840	76118840	Splice_Site	SNV	A	T	T	TMC6	c.1083-10T>A	
SH_119	17	76446414	76446414	Silent	SNV	A	T	T	DNAH17	c.10962T>A	P.A3654A
SH_119	17	76449447	76449447	Missense_Mutation	SNV	T	A	A	DNAH17	c.10507A>T	P.R3503W
SH_119	17	76526588	76526588	Missense_Mutation	SNV	A	T	T	DNAH17	c.3121T>A	P.S1041T
SH_119	17	77102803	77102803	Missense_Mutation	SNV	T	A	A	RBFOX3	c.290A>T	P.Q97L
SH_119	17	77984068	77984068	Missense_Mutation	SNV	T	A	A	TBC1D16	c.670A>T	P.S224C
SH_119	17	78168994	78168994	Missense_Mutation	SNV	A	T	T	CARD14	c.1361A>T	P.Q454L
SH_119	17	78178881	78178881	Missense_Mutation	SNV	T	A	A	CARD14	c.2446T>A	P.Y816N
SH_119	17	79655738	79655738	Missense_Mutation	SNV	A	C	C	HGS	c.296A>C	P.Q99P
SH_119	17	80038319	80038319	Missense_Mutation	SNV	T	A	A	FASN	c.6974A>T	P.Q2325L
SH_119	17	80048258	80048258	Silent	SNV	T	A	A	FASN	c.1863A>T	P.A621A
SH_119	17	8015465	8015465	Nonsense_Mutation	SNV	T	A	A	ALOXE3	c.1126A>T	P.K376*
SH_119	17	80521337	80521337	Missense_Mutation	SNV	A	T	T	FOXK2	c.527A>T	P.Q176L
SH_119	17	9570027	9570027	Silent	SNV	A	T	T	USP43	c.699A>T	P.L233L
SH_119	18	12122314	12122314	Missense_Mutation	SNV	T	A	A	ANKRD62	c.1253T>A	P.L418Q
SH_119	18	12351196	12351196	Silent	SNV	T	A	A	AFG3L2	c.1440A>T	P.I480I
SH_119	18	14537923	14537923	Frame_Shift_Del	INDEL	G	-	-	POTEC	c.687delC	P.A230fs
SH_119	18	20572948	20572948	Silent	SNV	A	C	C	RBBP8	c.1158A>C	P.T386T
SH_119	18	20596798	20596798	Missense_Mutation	SNV	A	T	T	RBBP8	c.2365A>T	P.S789C
SH_119	18	29432410	29432410	Missense_Mutation	SNV	T	A	A	TRAPPC8	c.3560A>T	P.Q1187L

SH_119	18	2951094	2951094	Silent	SNV	T	A	A	LPIN2	c.549A>T	P.V183V
SH_119	18	51731502	51731502	Nonsense_Mutation	SNV	T	A	A	MBD2	c.568A>T	P.K190*
SH_119	18	59895685	59895685	Silent	SNV	A	T	T	KIAA1468	c.1302A>T	P.T434T
SH_119	18	60241696	60241696	Silent	SNV	T	G	G	ZCCHC2	c.2382T>G	P.P794P
SH_119	18	6948522	6948522	Missense_Mutation	SNV	C	A	A	LAMA1	c.8590G>T	P.V2864L
SH_119	18	6985374	6985374	Missense_Mutation	SNV	A	T	T	LAMA1	c.5522T>A	P.L1841Q
SH_119	18	7009239	7009239	Nonsense_Mutation	SNV	T	A	A	LAMA1	c.4000A>T	P.R1334*
SH_119	18	9954125	9954125	Missense_Mutation	SNV	A	T	T	VAPA	c.802A>T	P.N268Y
SH_119	19	10262205	10262205	Missense_Mutation	SNV	T	A	A	DNMT1	c.2134A>T	P.M712L
SH_119	19	10798253	10798253	Missense_Mutation	SNV	A	T	T	ILF3	c.2303A>T	P.Y768F
SH_119	19	11892571	11892571	Missense_Mutation	SNV	A	T	T	ZNF441	c.1932A>T	P.K644N
SH_119	19	12830084	12830084	Nonsense_Mutation	SNV	T	A	A	TNPO2	c.166A>T	P.K56*
SH_119	19	14028916	14028916	Missense_Mutation	SNV	A	T	T	CC2D1A	c.782A>T	P.Q261L
SH_119	19	14703169	14703169	Splice_Site	SNV	A	T	T	CLEC17A	c.276A>T	P.P92P
SH_119	19	14910228	14910228	Missense_Mutation	SNV	A	T	T	OR7C1	c.721T>A	P.C241S
SH_119	19	14938140	14938140	Nonsense_Mutation	SNV	A	T	T	OR7A5	c.914T>A	P.L305*
SH_119	19	14952199	14952199	Nonsense_Mutation	SNV	A	T	T	OR7A10	c.491T>A	P.L164*
SH_119	19	15350788	15350788	Missense_Mutation	SNV	T	A	A	BRD4	c.3215A>T	P.Q1072L
SH_119	19	16855259	16855259	Missense_Mutation	SNV	T	A	A	NWD1	c.226T>A	P.C76S
SH_119	19	17487911	17487911	Missense_Mutation	SNV	T	A	A	PLVAP	c.187A>T	P.T63S
SH_119	19	19825167	19825167	Splice_Site	SNV	T	A	A	ZNF14	c.130+3A>T	
SH_119	19	23328191	23328191	Missense_Mutation	SNV	A	T	T	ZNF730	c.345A>T	P.K115N
SH_119	19	30017525	30017525	Missense_Mutation	SNV	A	T	T	VSTM2B	c.35A>T	P.Y12F
SH_119	19	33092917	33092917	Splice_Site	SNV	T	G	G	ANKRD27	c.2767+4A>C	
SH_119	19	33355184	33355184	Missense_Mutation	SNV	T	A	A	SLC7A9	c.296A>T	P.Y99F
SH_119	19	35420778	35420778	Splice_Site	SNV	G	T	T	ZNF30	c.-64-10G>T	
SH_119	19	35719225	35719225	Missense_Mutation	SNV	T	A	A	FAM187B	c.359A>T	P.H120L
SH_119	19	35940991	35940991	Frame_Shift_Del	INDEL	T	-	-	FFAR2	c.375delT	P.Y125fs
SH_119	19	35940992	35940992	Missense_Mutation	SNV	G	A	A	FFAR2	c.376G>A	P.G126R
SH_119	19	37488058	37488058	Missense_Mutation	SNV	A	T	T	ZNF568	c.1273A>T	P.S425C
SH_119	19	37677174	37677174	Missense_Mutation	SNV	T	A	A	ZNF585B	c.1265A>T	P.K422M
SH_119	19	3831810	3831810	Missense_Mutation	SNV	T	A	A	ZFR2	c.446A>T	P.Q149L
SH_119	19	3852539	3852539	Missense_Mutation	SNV	T	A	A	ZFR2	c.127A>T	P.S43C
SH_119	19	38689034	38689034	Missense_Mutation	SNV	A	T	T	SIPA1L3	c.4846A>T	P.I1616F
SH_119	19	38946293	38946293	Missense_Mutation	SNV	T	A	A	RYR1	c.1693T>A	P.C565S
SH_119	19	38989769	38989769	Missense_Mutation	SNV	T	A	A	RYR1	c.6913T>A	P.C2305S
SH_119	19	38996483	38996483	Missense_Mutation	SNV	T	A	A	RYR1	c.8438T>A	P.L2813Q
SH_119	19	3908286	3908286	Missense_Mutation	SNV	A	T	T	ATCAY	c.565A>T	P.N189Y
SH_119	19	39734311	39734311	Silent	SNV	T	A	A	IFNL3	c.552A>T	P.R184R
SH_119	19	39735447	39735447	Missense_Mutation	SNV	T	A	A	IFNL3	c.161A>T	P.K54M
SH_119	19	39976413	39976413	Missense_Mutation	SNV	A	T	T	TIMM50	c.767A>T	P.E256V
SH_119	19	40408377	40408377	Missense_Mutation	SNV	T	C	C	FCGBP	c.4462A>G	P.I1488V
SH_119	19	40761086	40761086	Missense_Mutation	SNV	T	A	A	AKT2	c.266A>T	P.H89L
SH_119	19	42125779	42125779	Splice_Site	SNV	A	T	T	CEACAM4	c.670-11T>A	
SH_119	19	43243004	43243004	Missense_Mutation	SNV	A	T	T	PSG3	c.302T>A	P.V101E
SH_119	19	43382400	43382400	Missense_Mutation	SNV	G	A	A	PSG1	c.95C>T	P.T32I
SH_119	19	44580556	44580556	Missense_Mutation	SNV	A	T	T	ZNF284	c.14A>T	P.K5M
SH_119	19	45296740	45296740	Missense_Mutation	SNV	A	T	T	CBLC	c.1009A>T	P.S337C
SH_119	19	45315801	45315801	Missense_Mutation	SNV	A	T	T	BCAM	c.500A>T	P.Q167L
SH_119	19	46321288	46321288	Nonsense_Mutation	SNV	G	A	A	SYMPK	c.3010C>T	P.Q1004*
SH_119	19	46664070	46664070	Silent	SNV	T	A	A	IGFL2	c.306T>A	P.V102V
SH_119	19	46974046	46974046	Missense_Mutation	SNV	G	T	T	PNMAL1	c.247C>A	P.P83T
SH_119	19	49692314	49692314	Missense_Mutation	SNV	A	T	T	TRPM4	c.1985A>T	P.D662V
SH_119	19	50154500	50154500	Missense_Mutation	SNV	A	C	C	SCAF1	c.854A>C	P.E285A
SH_119	19	50195571	50195571	Missense_Mutation	SNV	T	A	A	CPT1C	c.62T>A	P.V21E
SH_119	19	50461894	50461894	Splice_Site	SNV	A	T	T	SIGLEC11	c.1363+6T>A	
SH_119	19	51362778	51362778	Splice_Site	SNV	A	T	T	KLK3	c.631-8A>T	
SH_119	19	51470515	51470515	Missense_Mutation	SNV	T	A	A	KLK6	c.107A>T	P.Q36L
SH_119	19	51526433	51526433	Missense_Mutation	SNV	T	A	A	KLK11	c.515A>T	P.Q172L
SH_119	19	52034690	52034690	Missense_Mutation	SNV	A	T	T	SIGLEC6	c.151T>A	P.C51S
SH_119	19	52942137	52942137	Missense_Mutation	SNV	T	A	A	ZNF534	c.1424T>A	P.F475Y
SH_119	19	53007923	53007923	Missense_Mutation	SNV	A	T	T	ZNF578	c.79A>T	P.R27W
SH_119	19	53208806	53208806	Missense_Mutation	SNV	T	A	A	ZNF611	c.1502A>T	P.K501M
SH_119	19	53282109	53282109	Missense_Mutation	SNV	T	A	A	ZNF600	c.33A>T	P.K11N
SH_119	19	53453502	53453502	Missense_Mutation	SNV	T	A	A	ZNF816	c.1526A>T	P.Y509F
SH_119	19	53667895	53667895	Missense_Mutation	SNV	A	T	T	ZNF665	c.1848T>A	P.H616Q
SH_119	19	53747053	53747053	Nonsense_Mutation	SNV	A	T	T	ZNF677	c.113T>A	P.L38*
SH_119	19	55175453	55175453	Silent	SNV	A	T	T	LILRB4	c.312A>T	P.V104V
SH_119	19	55363541	55363541	Silent	SNV	T	A	A	KIR3DL2	c.159T>A	P.R53R
SH_119	19	55832369	55832369	Silent	SNV	T	A	A	TMEM150B	c.36A>T	P.L12L
SH_119	19	56283365	56283365	Frame_Shift_Ins	INDEL	-	A	A	RFPL4AL1	c.197dupA	P.L67fs
SH_119	19	56329362	56329362	Missense_Mutation	SNV	T	A	A	NLRP11	c.179A>T	P.Y60F
SH_119	19	56413491	56413491	Missense_Mutation	SNV	A	T	T	NLRP13	c.2699T>A	P.L900Q
SH_119	19	56423919	56423919	Missense_Mutation	SNV	A	G	G	NLRP13	c.1264T>C	P.C422R
SH_119	19	56569698	56569698	Missense_Mutation	SNV	A	T	T	NLRP5	c.3392A>T	P.Q1131L
SH_119	19	57840540	57840540	Missense_Mutation	SNV	A	T	T	ZNF543	c.1710A>T	P.R570S
SH_119	19	58118585	58118585	Missense_Mutation	SNV	A	T	T	ZNF530	c.1593A>T	P.Q531H
SH_119	19	58420718	58420718	Missense_Mutation	SNV	T	A	A	ZNF417	c.925A>T	P.S309C
SH_119	19	59068063	59068063	Missense_Mutation	SNV	T	A	A	UBE2M	c.338A>T	P.N113I
SH_119	19	6714059	6714059	Silent	SNV	T	A	A	C3	c.717A>T	P.T239T
SH_119	19	6916299	6916299	Missense_Mutation	SNV	A	T	T	ADGRE1	c.1184A>T	P.E395V
SH_119	19	9048696	9048696	Missense_Mutation	SNV	T	A	A	MUC16	c.32935A>T	P.T10979S
SH_119	19	9049462	9049462	Silent	SNV	A	T	T	MUC16	c.32169T>A	P.A10723A
SH_119	19	9064052	9064052	Nonsense_Mutation	SNV	A	T	T	MUC16	c.23394T>A	P.C7798*
SH_119	19	9071660	9071660	Silent	SNV	T	A	A	MUC16	c.15786A>T	P.T5262T
SH_119	19	9075153	9075153	Missense_Mutation	SNV	C	A	A	MUC16	c.12293G>T	P.S4098I
SH_119	19	9075971	9075971	Silent	SNV	T	A	A	MUC16	c.11475A>T	P.P3825P
SH_119	19	9577862	9577862	Silent	SNV	A	G	G	ZNF560	c.1761T>C	P.L587L
SH_119	19	9721235	9721235	Nonsense_Mutation	SNV	T	A	A	ZNF561	c.1102A>T	P.K368*
SH_119	2	100623906	100623906	Missense_Mutation	SNV	T	A	A	AFF3	c.191A>T	P.E64V
SH_119	2	101182980	101182980	Missense_Mutation	SNV	A	T	T	PDCL3	c.22A>T	P.T8S
SH_119	2	102781563	102781563	Splice_Site	SNV	A	T	T	IL1R1	c.297-12A>T	
SH_119	2	103136421	103136421	Splice_Site	SNV	A	G	G	SLC9A4	c.1818+7A>G	
SH_119	2	105961828	105961828	Nonstop_Mutation	SNV	A	C	C	C2orf49	c.573A>C	P.Ter191Cext*?
SH_119	2	108477368	108477368	Missense_Mutation	SNV	T	A	A	RGPD4	c.1905T>A	P.H635Q
SH_119	2	112786192	112786192	Silent	SNV	A	T	T	MERTK	c.2751A>T	P.A917A
SH_119	2	113780356	113780356	Splice_Site	SNV	T	A	A	IL36B	c.392-2A>T	
SH_119	2	11767202	11767202	Missense_Mutation	SNV	A	T	T	GREB1	c.4421A>T	P.Q1474L

SH_119	2	120640187	120640187	Missense_Mutation	SNV	A	T	T	PTPN4	c.575A>T	P.H192L
SH_119	2	128855116	128855116	Missense_Mutation	SNV	A	T	T	UGGT1	c.172A>T	P.T58S
SH_119	2	131103934	131103934	Missense_Mutation	SNV	A	C	C	IMP4	c.763A>C	P.M255L
SH_119	2	136569919	136569919	Missense_Mutation	SNV	A	T	T	LCT	c.2315T>A	P.V772E
SH_119	2	141143540	141143540	Missense_Mutation	SNV	T	A	A	LRP1B	c.10453A>T	P.N3485Y
SH_119	2	159537134	159537134	Missense_Mutation	SNV	A	T	T	PKP4	c.3395A>T	P.K1132I
SH_119	2	159537135	159537135	Missense_Mutation	SNV	A	T	T	PKP4	c.3396A>T	P.K1132N
SH_119	2	163072416	163072416	Silent	SNV	T	A	A	FAP	c.783A>T	P.I261I
SH_119	2	165552311	165552311	Missense_Mutation	SNV	T	A	A	COBLL1	c.1906A>T	P.T636S
SH_119	2	166183470	166183470	Missense_Mutation	SNV	A	T	T	SCN2A	c.2125A>T	P.S709C
SH_119	2	166764192	166764192	Missense_Mutation	SNV	T	A	A	TTC21B	c.2564A>T	P.Q855L
SH_119	2	168103327	168103327	Missense_Mutation	SNV	A	T	T	XIRP2	c.5425A>T	P.I1809F
SH_119	2	170063206	170063206	Missense_Mutation	SNV	T	A	A	LRP2	c.7024A>T	P.N2342Y
SH_119	2	171070912	171070912	Silent	SNV	T	A	A	MYO3B	c.345T>A	P.T115T
SH_119	2	175326222	175326222	Splice_Site	SNV	T	C	C	GPR155	c.1470-2A>G	
SH_119	2	175619008	175619008	Missense_Mutation	SNV	T	A	A	CHRNA1	c.479A>T	P.Q160L
SH_119	2	179413835	179413835	Missense_Mutation	SNV	A	T	T	TTN	c.87595T>A	P.L29199I
SH_119	2	179417635	179417635	Missense_Mutation	SNV	A	T	T	TTN	c.85069T>A	P.S28357T
SH_119	2	179417927	179417927	Silent	SNV	A	T	T	TTN	c.84777T>A	P.P28259P
SH_119	2	179437100	179437100	Missense_Mutation	SNV	A	T	T	TTN	c.68836T>A	P.C22946S
SH_119	2	18113513	18113513	Missense_Mutation	SNV	A	T	T	KCNS3	c.1238A>T	P.Y413F
SH_119	2	186654677	186654677	Silent	SNV	T	A	A	FSIP2	c.2814T>A	P.L938L
SH_119	2	189599392	189599392	Missense_Mutation	SNV	G	T	T	DIRC1	c.256C>A	P.L86I
SH_119	2	189867050	189867050	Silent	SNV	A	T	T	COL3A1	c.2418A>T	P.P806P
SH_119	2	202358262	202358262	Silent	SNV	A	T	T	ALS2CR11	c.2802T>A	P.P934P
SH_119	2	206659775	206659775	Missense_Mutation	SNV	A	T	T	NRP2	c.2774A>T	P.E925V
SH_119	2	208425086	208425086	Missense_Mutation	SNV	A	T	T	CREB1	c.251A>T	P.Q84L
SH_119	2	209201588	209201588	Missense_Mutation	SNV	A	T	T	PIKFYVE	c.4547A>T	P.K1516M
SH_119	2	209357998	209357998	Nonsense_Mutation	SNV	G	T	T	PTH2R	c.934G>T	P.E312*
SH_119	2	214878801	214878801	Splice_Site	SNV	A	T	T	SPAG16	n.1807A>T	
SH_119	2	220348946	220348946	Missense_Mutation	SNV	T	A	A	SPEG	c.6761T>A	P.L2254Q
SH_119	2	220353787	220353787	Missense_Mutation	SNV	T	A	A	SPEG	c.8161T>A	P.W2721R
SH_119	2	220478470	220478470	Splice_Site	SNV	A	T	T	STK11IP	c.2538-4A>T	
SH_119	2	220493244	220493244	Missense_Mutation	SNV	T	A	A	SLC4A3	c.169T>A	P.W57R
SH_119	2	226447431	226447431	Missense_Mutation	SNV	A	T	T	NYAP2	c.1298A>T	P.H433L
SH_119	2	230312255	230312255	Splice_Site	SNV	T	C	C	DNER	c.1263A>G	P.G421G
SH_119	2	234627755	234627755	Missense_Mutation	SNV	A	T	T	UGT1A4	c.289A>T	P.T97S
SH_119	2	235951376	235951376	Missense_Mutation	SNV	A	T	T	SH3BP4	c.1963A>T	P.S655C
SH_119	2	237395519	237395519	Nonsense_Mutation	SNV	T	A	A	IQCA1	c.673A>T	P.K225*
SH_119	2	238257010	238257010	Silent	SNV	A	T	T	COL6A3	c.6948T>A	P.P2316P
SH_119	2	241700261	241700261	Splice_Site	SNV	C	T	T	KIF1A	c.2266-1G>A	
SH_119	2	242066314	242066314	Silent	SNV	T	A	A	PASK	c.2016A>T	P.A672A
SH_119	2	25754369	25754369	Missense_Mutation	SNV	T	A	A	DTNB	c.974A>T	P.E325V
SH_119	2	26667182	26667182	Missense_Mutation	SNV	A	T	T	DRC1	c.1121A>T	P.K374I
SH_119	2	29356607	29356607	Missense_Mutation	SNV	A	T	T	CLIP4	c.454A>T	P.N152Y
SH_119	2	31410672	31410672	Missense_Mutation	SNV	T	A	A	CAPN14	c.1539A>T	P.E513D
SH_119	2	31600003	31600003	Missense_Mutation	SNV	T	A	A	XDH	c.1343A>T	P.E448V
SH_119	2	32474989	32474989	Silent	SNV	A	T	T	NLRC4	c.1944T>A	P.A648A
SH_119	2	32668631	32668631	Silent	SNV	A	G	G	BIRC6	c.4311A>G	P.S1437S
SH_119	2	32730271	32730271	Splice_Site	SNV	A	T	T	BIRC6	c.9699A>T	P.A3233A
SH_119	2	33572536	33572536	Missense_Mutation	SNV	A	T	T	LTBP1	c.2981A>T	P.Y994F
SH_119	2	42720536	42720536	Missense_Mutation	SNV	T	A	A	KCNG3	c.106A>T	P.S36C
SH_119	2	46605841	46605841	Nonsense_Mutation	SNV	A	T	T	EPAS1	c.1489A>T	P.K497*
SH_119	2	55791498	55791498	Silent	SNV	T	A	A	PPP4R3B	c.2211A>T	P.P737P
SH_119	2	61483549	61483549	Missense_Mutation	SNV	T	A	A	USP34	c.6191A>T	P.H2064L
SH_119	2	64685489	64685489	Missense_Mutation	SNV	A	G	G	LGALSL	c.446A>G	P.H149R
SH_119	2	66667702	66667702	Silent	SNV	A	T	T	MEIS1	c.390A>T	P.A130A
SH_119	2	69204616	69204616	Splice_Site	SNV	T	A	A	GKN1	c.55-11T>A	
SH_119	2	71816758	71816758	Missense_Mutation	SNV	G	A	A	DYSF	c.3387G>A	P.M1129I
SH_119	2	73996404	73996404	Missense_Mutation	SNV	T	A	A	DUSP11	c.623A>T	P.Y208F
SH_119	2	74135834	74135834	Missense_Mutation	SNV	T	A	A	ACTG2	c.161T>A	P.V54E
SH_119	2	74808801	74808801	Missense_Mutation	SNV	T	A	A	M1AP	c.769A>T	P.M257L
SH_119	2	80529404	80529404	Missense_Mutation	SNV	T	A	A	LRRTM1	c.1541A>T	P.Q514L
SH_119	2	9048826	9048826	Missense_Mutation	SNV	A	T	T	MBOAT2	c.53T>A	P.L18Q
SH_119	2	96076652	96076652	Missense_Mutation	SNV	C	T	T	FAHD2A	c.563C>T	P.A188V
SH_119	2	9668039	9668039	Missense_Mutation	SNV	T	A	A	ADAM17	c.495A>T	P.L165F
SH_119	2	97026404	97026404	Missense_Mutation	SNV	A	T	T	NCAPH	c.1510A>T	P.N504Y
SH_119	2	97530828	97530828	Missense_Mutation	SNV	T	A	A	SEMA4C	c.659A>T	P.Y220F
SH_119	20	1616954	1616954	Missense_Mutation	SNV	T	A	A	SIRPG	c.628A>T	P.T210S
SH_119	20	19665861	19665861	Missense_Mutation	SNV	G	A	A	SLC24A3	c.1180G>A	P.G394S
SH_119	20	20033147	20033147	Missense_Mutation	SNV	A	G	G	CRNKL1	c.287T>C	P.V96A
SH_119	20	21687214	21687214	Missense_Mutation	SNV	T	A	A	PAX1	c.425T>A	P.V142E
SH_119	20	23028754	23028754	Missense_Mutation	SNV	T	A	A	THBD	c.1388A>T	P.E463V
SH_119	20	23966809	23966809	Missense_Mutation	SNV	C	T	T	GGTLC1	c.208G>A	P.G70R
SH_119	20	25261031	25261031	Missense_Mutation	SNV	A	T	T	PYGB	c.1222A>T	P.N408Y
SH_119	20	30527026	30527026	Silent	SNV	T	A	A	TTLL9	c.1200T>A	P.P400P
SH_119	20	30556175	30556175	Missense_Mutation	SNV	T	A	A	XKR7	c.197T>A	P.V66E
SH_119	20	31692643	31692643	Nonsense_Mutation	SNV	A	T	T	BPIFB4	c.1708A>T	P.K570*
SH_119	20	33703495	33703495	Missense_Mutation	SNV	T	A	A	EDEM2	c.1367A>T	P.E456V
SH_119	20	34022535	34022535	Silent	SNV	A	G	G	GDF5	c.678T>C	P.I226I
SH_119	20	35668692	35668692	Splice_Site	SNV	T	A	A	RBL1	c.490-4A>T	
SH_119	20	36406054	36406054	Splice_Site	SNV	A	T	T	CTNNBL1	c.743-2A>T	
SH_119	20	36641090	36641090	Missense_Mutation	SNV	T	A	A	TTI1	c.1129A>T	P.S377C
SH_119	20	37174977	37174977	Missense_Mutation	SNV	A	T	T	RALGAPB	c.2806A>T	P.R936W
SH_119	20	37581107	37581107	Missense_Mutation	SNV	A	T	T	FAM83D	c.1702A>T	P.S568C
SH_119	20	3765945	3765945	Missense_Mutation	SNV	T	A	A	CENPB	c.1186A>T	P.T396S
SH_119	20	42694395	42694395	Missense_Mutation	SNV	C	T	T	TOX2	c.878C>T	P.P293L
SH_119	20	47258735	47258735	Missense_Mutation	SNV	T	A	A	PREX1	c.3746A>T	P.K1249M
SH_119	20	47695092	47695092	Splice_Site	INDEL	A	-	-	CSE1L	c.1253-6delA	
SH_119	20	47733764	47733764	Nonsense_Mutation	SNV	T	A	A	STAU1	c.1306A>T	P.K436*
SH_119	20	50407599	50407599	Nonsense_Mutation	SNV	T	A	A	SALL4	c.1423A>T	P.K475*
SH_119	20	57480539	57480539	Splice_Site	SNV	A	T	T	GNAS	c.530+4A>T	
SH_119	20	58453085	58453085	Missense_Mutation	SNV	T	A	A	SYCP2	c.2957A>T	P.K986M
SH_119	20	62737376	62737376	Missense_Mutation	SNV	A	T	T	NPBWR2	c.809T>A	P.L270H
SH_119	21	16338771	16338771	Frame_Shift_Del	INDEL	T	-	-	NRIP1	c.1743delA	P.Y582fs
SH_119	21	16338772	16338772	Missense_Mutation	SNV	G	A	A	NRIP1	c.1742C>T	P.P581L
SH_119	21	19629064	19629064	Silent	SNV	T	C	C	CHODL	c.261T>C	P.N87N
SH_119	21	27348267	27348267	Missense_Mutation	SNV	C	A	A	APP	c.1299G>T	P.Q433H

SH_119	21	41446962	41446962	Splice_Site	SNV	A	T	T	DSCAM	c.4888+2T>A	
SH_119	21	43321802	43321802	Missense_Mutation	SNV	T	A	A	C2CD2	c.1489A>T	P.S497C
SH_119	21	43413159	43413159	Missense_Mutation	SNV	T	A	A	ZBTB21	c.1046A>T	P.Q349L
SH_119	21	44841919	44841919	Splice_Site	SNV	T	A	A	SIK1	c.274-2A>T	
SH_119	21	45774583	45774583	Missense_Mutation	SNV	T	A	A	TRPM2	c.218T>A	P.V73E
SH_119	21	46066653	46066653	Frame_Shift_Del	INDEL	C	-	-	KRTAP10-11	c.282delC	P.C95fs
SH_119	21	46086493	46086493	Missense_Mutation	SNV	A	T	T	KRTAP12-2	c.311T>A	P.L104Q
SH_119	22	16277843	16277843	Silent	SNV	T	A	A	POTEH	c.1071A>T	P.I357I
SH_119	22	17976530	17976530	Missense_Mutation	SNV	A	T	T	CECR2	c.191A>T	P.Q64L
SH_119	22	19223255	19223255	Silent	SNV	T	A	A	CLTCL1	c.933A>T	P.P311P
SH_119	22	20024365	20024365	Missense_Mutation	SNV	A	T	T	TANGO2	c.44A>T	P.K15I
SH_119	22	21065029	21065029	Missense_Mutation	SNV	T	A	A	PI4KA	c.6041A>T	P.K2014M
SH_119	22	21385366	21385366	Missense_Mutation	SNV	A	G	G	SLC7A4	c.736T>C	P.Y246H
SH_119	22	22892611	22892611	Missense_Mutation	SNV	A	T	T	PRAME	c.490T>A	P.L164M
SH_119	22	24086414	24086414	Missense_Mutation	SNV	T	A	A	ZNF70	c.914A>T	P.K305M
SH_119	22	26860783	26860783	Silent	SNV	T	A	A	HPS4	c.813A>T	P.A271A
SH_119	22	28492398	28492398	Splice_Site	SNV	T	A	A	TTC28	c.3548-2A>T	
SH_119	22	29669815	29669815	Missense_Mutation	SNV	A	T	T	EWSR1	c.188A>T	P.Q63L
SH_119	22	31013440	31013440	Missense_Mutation	SNV	T	A	A	TCN2	c.1064T>A	P.V355E
SH_119	22	32269261	32269261	Missense_Mutation	SNV	G	A	A	DEPDC5	c.3479G>A	P.S1160N
SH_119	22	32754173	32754173	Missense_Mutation	SNV	A	T	T	RFPL3	c.115A>T	P.S39C
SH_119	22	35811931	35811931	Missense_Mutation	SNV	A	T	T	MCM5	c.1313A>T	P.D438V
SH_119	22	38273931	38273931	Missense_Mutation	SNV	A	T	T	EIF3L	c.1184A>T	P.Q395L
SH_119	22	38882323	38882323	Missense_Mutation	SNV	G	A	A	DDX17	c.1813C>T	P.R605W
SH_119	22	40055777	40055777	Missense_Mutation	SNV	T	A	A	CACNA1I	c.2419T>A	P.F807I
SH_119	22	40365493	40365493	Missense_Mutation	SNV	A	T	T	GRAP2	c.769A>T	P.M257L
SH_119	22	44589713	44589713	Splice_Site	SNV	T	A	A	PARVG	c.583+10T>A	
SH_119	22	45958887	45958887	Missense_Mutation	SNV	A	C	C	FBLN1	c.1793A>C	P.Q598P
SH_119	3	10413649	10413649	Silent	SNV	T	A	A	ATP2B2	c.1503A>T	P.T501T
SH_119	3	111988887	111988887	Silent	SNV	T	A	A	SLC9C1	c.651A>T	P.I217I
SH_119	3	124103755	124103755	Missense_Mutation	SNV	T	A	A	KALRN	c.1828T>A	P.C610S
SH_119	3	126229580	126229580	Missense_Mutation	SNV	G	A	A	UROC1	c.184C>T	P.P62S
SH_119	3	127390284	127390284	Missense_Mutation	SNV	T	A	A	PODXL2	c.1433T>A	P.I478N
SH_119	3	128077070	128077070	Missense_Mutation	SNV	A	T	T	EEFSEC	c.1454A>T	P.D485V
SH_119	3	128623244	128623244	Missense_Mutation	SNV	A	T	T	ACAD9	c.1045A>T	P.M349L
SH_119	3	12976961	12976961	Missense_Mutation	SNV	T	A	A	IQSEC1	c.1555A>T	P.N519Y
SH_119	3	131268871	131268871	Missense_Mutation	SNV	A	T	T	CPNE4	c.1276T>A	P.Y426N
SH_119	3	132075694	132075694	Missense_Mutation	SNV	A	T	T	ACPP	c.1133A>T	P.H378L
SH_119	3	132337466	132337466	Splice_Site	SNV	T	A	A	ACAD11	c.1414+12A>T	
SH_119	3	132360859	132360859	Missense_Mutation	SNV	T	A	A	ACAD11	c.494A>T	P.Q165L
SH_119	3	138121086	138121086	Missense_Mutation	SNV	A	T	T	MRAS	c.602A>T	P.H201L
SH_119	3	13916487	13916487	Silent	SNV	A	T	T	WNT7A	c.255T>A	P.S85S
SH_119	3	1414093	1414093	Missense_Mutation	SNV	T	A	A	CNTN6	c.1603T>A	P.W535R
SH_119	3	148458820	148458820	Nonsense_Mutation	SNV	A	T	T	AGTR1	c.85A>T	P.K29*
SH_119	3	150911419	150911419	Missense_Mutation	SNV	A	T	T	MED12L	c.2111A>T	P.H704L
SH_119	3	150916900	150916900	Missense_Mutation	SNV	A	T	T	GPR171	c.274T>A	P.C92S
SH_119	3	15119651	15119651	Missense_Mutation	SNV	T	C	C	RBSN	c.929A>G	P.Y310C
SH_119	3	155210644	155210644	Splice_Site	SNV	T	A	A	PLCH1	c.2147-2A>T	
SH_119	3	155286055	155286055	Missense_Mutation	SNV	T	A	A	PLCH1	c.794A>T	P.E265V
SH_119	3	158384162	158384162	Missense_Mutation	SNV	A	T	T	GFM1	c.1645A>T	P.T549S
SH_119	3	158523163	158523163	Missense_Mutation	SNV	A	G	G	MFSD1	c.10A>G	P.N4D
SH_119	3	168845708	168845708	Missense_Mutation	SNV	T	A	A	MECOM	c.382A>T	P.S128C
SH_119	3	17052911	17052911	Silent	SNV	T	C	C	PLCL2	c.2061T>C	P.D687D
SH_119	3	172766763	172766763	Missense_Mutation	SNV	T	A	A	SPATA16	c.734A>T	P.D245V
SH_119	3	178745227	178745227	Silent	SNV	T	A	A	ZMAT3	c.642A>T	P.T214T
SH_119	3	179076760	179076760	Silent	SNV	A	T	T	MFN1	c.381A>T	P.T127T
SH_119	3	183823640	183823640	Missense_Mutation	SNV	T	A	A	HTR3E	c.808T>A	P.Y270N
SH_119	3	183954141	183954141	Missense_Mutation	SNV	A	T	T	VWA5B2	c.566A>T	P.Q189L
SH_119	3	183976257	183976257	Missense_Mutation	SNV	A	T	T	ECE2	c.662A>T	P.Q221L
SH_119	3	185304301	185304301	Missense_Mutation	SNV	A	T	T	SENP2	c.100A>T	P.S34C
SH_119	3	185542560	185542560	Splice_Site	SNV	T	A	A	IGF2BP2	c.178+11A>T	
SH_119	3	185990076	185990076	Nonsense_Mutation	SNV	T	A	A	DGKG	c.967A>T	P.K323*
SH_119	3	187388070	187388070	Missense_Mutation	SNV	A	T	T	SST	c.10T>A	P.C4S
SH_119	3	189706687	189706687	Splice_Site	SNV	A	T	T	P3H2	c.955+9T>A	
SH_119	3	193029642	193029642	Missense_Mutation	SNV	T	A	A	ATP13A5	c.2408A>T	P.Q803L
SH_119	3	195518151	195518151	Silent	SNV	A	T	T	MUC4	c.300T>A	P.T100T
SH_119	3	21552416	21552416	Missense_Mutation	SNV	T	A	A	ZNF385D	c.376A>T	P.S126C
SH_119	3	31621525	31621525	Silent	SNV	A	T	T	STT3B	c.648A>T	P.V216V
SH_119	3	32932313	32932313	Silent	SNV	G	A	A	TRIM71	c.1617G>A	P.E539E
SH_119	3	37535989	37535989	Splice_Site	SNV	T	A	A	ITGA9	c.545-3T>A	
SH_119	3	38622494	38622494	Silent	SNV	G	C	C	SCN5A	c.3156C>G	P.A1052A
SH_119	3	44328923	44328923	Missense_Mutation	SNV	T	A	A	TOPAZ1	c.3479T>A	P.V1160E
SH_119	3	44867945	44867945	Missense_Mutation	SNV	A	T	T	KIF15	c.2779A>T	P.S927C
SH_119	3	45965182	45965182	Missense_Mutation	SNV	T	A	A	FYCO1	c.4327A>T	P.I1443F
SH_119	3	48607617	48607617	Splice_Site	SNV	T	A	A	COL7A1	c.7441-6A>T	
SH_119	3	48629432	48629432	Missense_Mutation	SNV	T	A	A	COL7A1	c.1256A>T	P.Q419L
SH_119	3	49055043	49055043	Splice_Site	SNV	T	A	A	DALRD3	c.718+3A>T	
SH_119	3	49570051	49570051	Missense_Mutation	SNV	A	T	T	DAG1	c.2107A>T	P.T703S
SH_119	3	51929251	51929251	Silent	SNV	T	A	A	IQCF1	c.273A>T	P.A91A
SH_119	3	52027799	52027799	Missense_Mutation	SNV	T	A	A	RPL29	c.446A>T	P.K149I
SH_119	3	52485850	52485850	Missense_Mutation	SNV	T	A	A	TNNC1	c.227A>T	P.E76V
SH_119	3	53217135	53217135	Splice_Site	SNV	A	T	T	PRKCD	c.572-2A>T	
SH_119	3	54661900	54661900	Missense_Mutation	SNV	T	A	A	CACNA2D3	c.1050T>A	P.S350R
SH_119	3	62543149	62543149	Missense_Mutation	SNV	T	C	C	CADPS	c.1684A>G	P.K562E
SH_119	3	87039772	87039772	Missense_Mutation	SNV	T	A	A	VGLL3	c.122A>T	P.Q41L
SH_119	3	97595483	97595483	Silent	SNV	A	G	G	CRYBG3	c.5445A>G	P.A1815A
SH_119	3	97686259	97686259	Missense_Mutation	SNV	T	A	A	MINA	c.179A>T	P.E60V
SH_119	3	98073430	98073430	Missense_Mutation	SNV	T	A	A	OR5K4	c.733T>A	P.F245I
SH_119	4	10083026	10083026	Silent	SNV	T	A	A	WDR1	c.819A>T	P.V273V
SH_119	4	106576777	106576777	Silent	SNV	T	A	A	ARHGEF38	c.1131T>A	P.A377A
SH_119	4	108940731	108940731	Missense_Mutation	SNV	A	T	T	HADH	c.455A>T	P.Q152L
SH_119	4	114199612	114199612	Splice_Site	INDEL	A	-	-	ANK2	c.1720-4delA	
SH_119	4	123132087	123132087	Missense_Mutation	SNV	A	T	T	KIAA1109	c.2084A>T	P.Q695L
SH_119	4	123246471	123246471	Missense_Mutation	SNV	A	T	T	KIAA1109	c.10991A>T	P.D3664V
SH_119	4	129121747	129121747	Nonsense_Mutation	SNV	A	T	T	LARP1B	c.2236A>T	P.R746*
SH_119	4	134084217	134084217	Silent	SNV	T	C	C	PCDH10	c.2883T>C	P.S961S
SH_119	4	13589365	13589365	Missense_Mutation	SNV	T	C	C	BOD1L1	c.8311A>G	P.R2771G
SH_119	4	141868808	141868808	Missense_Mutation	SNV	C	T	T	RNF150	c.890G>A	P.R297Q

SH_119	4	145640104	145640104	Nonsense_Mutation	SNV	A	T	T	HHIP	c.1756A>T	P.K586*
SH_119	4	148984337	148984337	Missense_Mutation	SNV	A	T	T	ARHGAP10	c.2066A>T	P.N689I
SH_119	4	152498952	152498952	Silent	SNV	A	C	C	FAM160A1	c.456A>C	P.G152G
SH_119	4	153883988	153883988	Silent	SNV	A	G	G	FHDC1	c.873A>G	P.L291L
SH_119	4	154702659	154702659	Nonsense_Mutation	SNV	T	A	A	SFRP2	c.832A>T	P.K278*
SH_119	4	159076755	159076755	Splice_Site	SNV	A	T	T	FAM198B	c.1149+8T>A	
SH_119	4	15937806	15937806	Silent	SNV	T	A	A	FGFBP1	c.450A>T	P.L150L
SH_119	4	164394412	164394412	Missense_Mutation	SNV	A	C	C	TKTL2	c.475T>G	P.S159A
SH_119	4	164394868	164394868	Nonsense_Mutation	SNV	T	A	A	TKTL2	c.19A>T	P.K7*
SH_119	4	174169288	174169288	Missense_Mutation	SNV	A	T	T	GALNT7	c.284A>T	P.Q95L
SH_119	4	175898417	175898417	Missense_Mutation	SNV	T	A	A	ADAM29	c.1741T>A	P.C581S
SH_119	4	186209239	186209239	Splice_Site	SNV	A	T	T	SNX25	c.670+3A>T	
SH_119	4	187510324	187510324	Missense_Mutation	SNV	T	A	A	FAT1	c.13189A>T	P.I4397L
SH_119	4	25253938	25253938	Splice_Site	SNV	T	C	C	PI4K2B	c.269-5T>C	
SH_119	4	25675937	25675937	Silent	SNV	A	G	G	SLC34A2	c.1236A>G	P.A412A
SH_119	4	25677761	25677761	Missense_Mutation	SNV	C	T	T	SLC34A2	c.1463C>T	P.A488V
SH_119	4	2835429	2835429	Silent	SNV	T	A	A	SH3BP2	c.1554T>A	P.S518S
SH_119	4	30723300	30723300	Missense_Mutation	SNV	A	G	G	PCDH7	c.256A>G	P.T86A
SH_119	4	30725916	30725916	Nonsense_Mutation	SNV	A	T	T	PCDH7	c.2872A>T	P.K958*
SH_119	4	36286088	36286088	Silent	SNV	A	T	T	DTHD1	c.387A>T	P.S129S
SH_119	4	38126728	38126728	Splice_Site	SNV	A	T	T	TBC1D1	c.3085-4A>T	
SH_119	4	39255564	39255564	Missense_Mutation	SNV	A	T	T	WDR19	c.2435A>T	P.Q812L
SH_119	4	40351163	40351163	Silent	SNV	T	A	A	CHRNA9	c.630T>A	P.A210A
SH_119	4	42122136	42122136	Missense_Mutation	SNV	A	T	T	BEND4	c.1322T>A	P.V441E
SH_119	4	42592867	42592867	Missense_Mutation	SNV	C	T	T	ATP8A1	c.485G>A	P.G162E
SH_119	4	46966983	46966983	Splice_Site	SNV	A	T	T	GABRA4	c.1134+4T>A	
SH_119	4	47597807	47597807	Silent	SNV	A	G	G	CORIN	c.2748T>C	P.Y916Y
SH_119	4	56448298	56448298	Missense_Mutation	SNV	T	A	A	PDCL2	c.113A>T	P.Q38L
SH_119	4	57215634	57215634	Silent	SNV	T	A	A	AASDH	c.1983A>T	P.S661S
SH_119	4	6382725	6382725	Missense_Mutation	SNV	T	A	A	PPP2R2C	c.146A>T	P.E49V
SH_119	4	66189875	66189875	Missense_Mutation	SNV	T	A	A	EPHA5	c.3071A>T	P.Q1024L
SH_119	4	72313400	72313400	Nonsense_Mutation	SNV	A	T	T	SLC4A4	c.1003A>T	P.K335*
SH_119	4	76530824	76530824	Splice_Site	SNV	A	T	T	CDKL2	c.543-6T>A	
SH_119	4	84191035	84191035	Missense_Mutation	SNV	T	A	A	COQ2	c.890A>T	P.Y297F
SH_119	5	100147637	100147637	Silent	SNV	T	G	G	ST8SIA4	c.994A>C	P.R332R
SH_119	5	112116599	112116599	Missense_Mutation	SNV	A	T	T	APC	c.644A>T	P.Q215L
SH_119	5	121409725	121409725	Missense_Mutation	SNV	A	G	G	LOX	c.328T>C	P.C110R
SH_119	5	126738327	126738327	Missense_Mutation	SNV	T	A	A	MEGF10	c.870T>A	P.D290E
SH_119	5	126771071	126771071	Missense_Mutation	SNV	T	A	A	MEGF10	c.1994T>A	P.F665Y
SH_119	5	128326089	128326089	Nonsense_Mutation	SNV	A	T	T	SLC27A6	c.901A>T	P.K301*
SH_119	5	137243561	137243561	Missense_Mutation	SNV	A	G	G	PKD2L2	c.1040A>G	P.N347S
SH_119	5	138163242	138163242	Missense_Mutation	SNV	G	T	T	CTNNA1	c.897G>T	P.E299D
SH_119	5	140167186	140167186	Silent	SNV	C	T	T	PCDHA1	c.1311C>T	P.A437A
SH_119	5	140215249	140215249	Silent	SNV	G	A	A	PCDHA7	c.1281G>A	P.A427A
SH_119	5	140255343	140255343	Missense_Mutation	SNV	T	A	A	PCDHA12	c.286T>A	P.C96S
SH_119	5	140474424	140474424	Missense_Mutation	SNV	T	C	C	PCDHB2	c.50T>C	P.L17P
SH_119	5	140482146	140482146	Missense_Mutation	SNV	A	T	T	PCDHB3	c.1913A>T	P.H638L
SH_119	5	140567770	140567770	Missense_Mutation	SNV	A	T	T	PCDHB9	c.878A>T	P.Q293L
SH_119	5	140594491	140594491	Missense_Mutation	SNV	T	C	C	PCDHB13	c.796T>C	P.S266P
SH_119	5	140778452	140778452	Missense_Mutation	SNV	A	T	T	PCDHGB5	c.758A>T	P.E253V
SH_119	5	141041681	141041681	Missense_Mutation	SNV	T	A	A	ARAP3	c.2942A>T	P.K981I
SH_119	5	141244731	141244731	Missense_Mutation	SNV	T	A	A	PCDH1	c.1213A>T	P.I405F
SH_119	5	149581924	149581924	Silent	SNV	T	A	A	SLC6A7	c.873T>A	P.A291A
SH_119	5	151043792	151043792	Missense_Mutation	SNV	G	A	A	SPARC	c.736C>T	P.L246F
SH_119	5	154315430	154315430	Silent	SNV	T	A	A	GEMIN5	c.480A>T	P.S160S
SH_119	5	156753269	156753269	Missense_Mutation	SNV	A	T	T	CYFIP2	c.2073A>T	P.E691D
SH_119	5	16764414	16764414	Missense_Mutation	SNV	T	A	A	MYO10	c.1271A>T	P.N424I
SH_119	5	178139478	178139478	Missense_Mutation	SNV	T	A	A	ZNF354A	c.1275A>T	P.K425N
SH_119	5	178563024	178563024	Silent	SNV	C	T	T	ADAMTS2	c.1971G>A	P.L657L
SH_119	5	21975294	21975294	Silent	SNV	T	A	A	CDH12	c.432A>T	P.S144S
SH_119	5	225645	225645	Missense_Mutation	SNV	A	T	T	SDHA	c.424A>T	P.M142L
SH_119	5	26902708	26902708	Missense_Mutation	SNV	A	T	T	CDH9	c.1130T>A	P.I377K
SH_119	5	26906171	26906171	Silent	SNV	T	A	A	CDH9	c.708A>T	P.I236I
SH_119	5	32037363	32037363	Missense_Mutation	SNV	T	A	A	PDZD2	c.1434T>A	P.D478E
SH_119	5	32783020	32783020	Nonsense_Mutation	SNV	A	T	T	NPR3	c.1312A>T	P.K438*
SH_119	5	35010157	35010157	Missense_Mutation	SNV	C	G	G	AGXT2	c.1061G>C	P.G354A
SH_119	5	37000636	37000636	Missense_Mutation	SNV	A	G	G	NIPBL	c.3466A>G	P.M1156V
SH_119	5	37815958	37815958	Missense_Mutation	SNV	T	A	A	GDNF	c.431A>T	P.Y144F
SH_119	5	39130690	39130690	Splice_Site	SNV	A	T	T	FYB	c.1870+2T>A	
SH_119	5	66449352	66449352	Missense_Mutation	SNV	A	T	T	MAST4	c.3592A>T	P.N1198Y
SH_119	5	72853467	72853467	Splice_Site	SNV	T	A	A	ANKRA2	c.449-2A>T	
SH_119	5	81608541	81608541	Silent	SNV	A	T	T	ATP6AP1L	c.243A>T	P.V81V
SH_119	5	82837882	82837882	Missense_Mutation	SNV	A	T	T	VCAN	c.9060A>T	P.R3020S
SH_119	5	93989002	93989002	Missense_Mutation	SNV	A	T	T	SLF1	c.908A>T	P.D303V
SH_119	6	108499292	108499292	Splice_Site	SNV	T	A	A	NR2E1	c.607-7T>A	
SH_119	6	109935621	109935621	Missense_Mutation	SNV	T	A	A	AK9	c.1462A>T	P.M488L
SH_119	6	110763638	110763638	Missense_Mutation	SNV	T	A	A	SLC22A16	c.992A>T	P.Q331L
SH_119	6	117662737	117662737	Missense_Mutation	SNV	T	C	C	ROS1	c.4728A>G	P.I1576M
SH_119	6	125569430	125569430	Missense_Mutation	SNV	A	C	C	TPD52L1	c.200A>C	P.Y67S
SH_119	6	136599326	136599326	Silent	SNV	A	T	T	BCLAF1	c.687T>A	P.P229P
SH_119	6	137519389	137519389	Missense_Mutation	SNV	T	A	A	IFNGR1	c.1249A>T	P.S417C
SH_119	6	138576598	138576598	Missense_Mutation	SNV	G	C	C	ARFGEF3	c.796G>C	P.V266L
SH_119	6	138628511	138628511	Missense_Mutation	SNV	A	T	T	ARFGEF3	c.3950A>T	P.Y1317F
SH_119	6	13977775	13977775	Missense_Mutation	SNV	C	T	T	RNF182	c.425C>T	P.P142L
SH_119	6	144814510	144814510	Missense_Mutation	SNV	A	T	T	UTRN	c.4511A>T	P.Q1504L
SH_119	6	149833385	149833385	Missense_Mutation	SNV	T	A	A	PPIL4	c.1133A>T	P.H378L
SH_119	6	152554911	152554911	Splice_Site	SNV	T	A	A	SYNE1	c.20499+5A>T	
SH_119	6	160479077	160479077	Missense_Mutation	SNV	A	T	T	IGF2R	c.2819A>T	P.N940I
SH_119	6	165712993	165712993	Splice_Site	SNV	T	A	A	C6orf118	c.877-2A>T	
SH_119	6	24777537	24777537	Splice_Site	SNV	A	G	G	GMNN	c.51+12A>G	
SH_119	6	26446013	26446013	Missense_Mutation	SNV	A	T	T	BTN3A3	c.515A>T	P.Y172F
SH_119	6	27101157	27101157	Missense_Mutation	SNV	A	T	T	HIST1H2AG	c.307A>T	P.I103F
SH_119	6	28239825	28239825	Missense_Mutation	SNV	A	T	T	ZSCAN26	c.128A>T	P.K43I
SH_119	6	28402285	28402285	Missense_Mutation	SNV	A	T	T	ZSCAN23	c.1127T>A	P.L376Q
SH_119	6	28500177	28500177	Nonsense_Mutation	SNV	A	T	T	GPX5	c.439A>T	P.K147*
SH_119	6	30546166	30546166	Splice_Site	SNV	A	T	T	ABCF1	c.381+4A>T	
SH_119	6	30671957	30671957	Missense_Mutation	SNV	T	A	A	MDC1	c.5003A>T	P.Q1668L
SH_119	6	32008187	32008187	Missense_Mutation	SNV	A	T	T	CYP21A2	c.944A>T	P.Q315L

SH_119	6	33406350	33406350	Splice_Site	SNV	A	T	T	SYNGAP1	c.1531+10A>T	
SH_119	6	33543695	33543695	Silent	SNV	T	A	A	BAK1	c.81A>T	P.V27V
SH_119	6	35096967	35096967	Splice_Site	SNV	A	T	T	TCP11	c.276-8T>A	
SH_119	6	36672184	36672184	Nonsense_Mutation	SNV	A	T	T	RAB44	c.172A>T	P.K58*
SH_119	6	38994446	38994446	Splice_Site	SNV	A	T	T	DNAH8	c.13836+3A>T	
SH_119	6	42034075	42034075	Missense_Mutation	SNV	A	T	T	TAF8	c.515A>T	P.Y172F
SH_119	6	42619808	42619808	Splice_Site	SNV	A	T	T	UBR2	c.2613A>T	P.T871T
SH_119	6	42830284	42830284	Missense_Mutation	SNV	A	G	G	GLTSCR1L	c.2386A>G	P.R796G
SH_119	6	49574885	49574885	Silent	SNV	T	C	C	RHAG	c.1116A>G	P.A372A
SH_119	6	4996215	4996215	Missense_Mutation	SNV	T	A	A	RPP40	c.794A>T	P.E265V
SH_119	6	49976862	49976862	Missense_Mutation	SNV	A	G	G	DEFB110	c.178T>C	P.C60R
SH_119	6	51712640	51712640	Silent	SNV	T	A	A	PKHD1	c.8040A>T	P.P2680P
SH_119	6	52761649	52761649	Silent	SNV	T	A	A	GSTA3	c.624A>T	P.A208A
SH_119	6	55216285	55216285	Missense_Mutation	SNV	A	G	G	GFRAL	c.605A>G	P.K202R
SH_119	6	55264003	55264003	Missense_Mutation	SNV	A	T	T	GFRAL	c.978A>T	P.K326N
SH_119	6	57467155	57467155	Missense_Mutation	SNV	A	T	T	PRIM2	c.1096A>T	P.S366C
SH_119	6	82935195	82935195	Missense_Mutation	SNV	T	A	A	IBTK	c.824A>T	P.Q275L
SH_119	6	84859375	84859375	Missense_Mutation	SNV	T	A	A	CEP162	c.3449A>T	P.H1150L
SH_119	6	85447044	85447044	Missense_Mutation	SNV	A	T	T	TBX18	c.1183T>A	P.C395S
SH_119	6	89816936	89816936	Missense_Mutation	SNV	A	G	G	SRSF12	c.107T>C	P.I36T
SH_119	7	100636024	100636024	Missense_Mutation	SNV	C	T	T	MUC12	c.2180C>T	P.S727L
SH_119	7	100646531	100646531	Silent	SNV	A	T	T	MUC12	c.12687A>T	P.T4229T
SH_119	7	100675016	100675016	Missense_Mutation	SNV	A	T	T	MUC17	c.319A>T	P.S107C
SH_119	7	100687033	100687033	Silent	SNV	T	A	A	MUC17	c.12336T>A	P.A4112A
SH_119	7	102108744	102108744	Missense_Mutation	SNV	A	T	T	LRWD1	c.383A>T	P.Q128L
SH_119	7	103131192	103131192	Silent	SNV	T	C	C	RELN	c.9528A>G	P.E3176E
SH_119	7	105909612	105909612	Silent	SNV	G	C	C	NAMPT	c.594C>G	P.V198V
SH_119	7	107431656	107431656	Missense_Mutation	SNV	A	G	G	SLC26A3	c.407T>C	P.M136T
SH_119	7	120979077	120979077	Missense_Mutation	SNV	T	A	A	WNT16	c.746T>A	P.I249K
SH_119	7	123150025	123150025	Silent	SNV	A	T	T	IQUB	c.462T>A	P.L154L
SH_119	7	123455084	123455084	Splice_Site	SNV	A	T	T	HYALP1	n.892A>T	
SH_119	7	126544162	126544162	Silent	SNV	T	A	A	GRM8	c.882A>T	P.A294A
SH_119	7	127014164	127014164	Missense_Mutation	SNV	T	A	A	ZNF800	c.1226A>T	P.K409I
SH_119	7	127235515	127235515	Missense_Mutation	SNV	A	T	T	FSCN3	c.299A>T	P.H100L
SH_119	7	127544860	127544860	Frame_Shift_Del	INDEL	A	-	-	SND1	c.1515delA	P.D506fs
SH_119	7	130368449	130368449	Frame_Shift_Del	INDEL	C	-	-	TSGA13	c.85delG	P.V29fs
SH_119	7	131864596	131864596	Missense_Mutation	SNV	T	A	A	PLXNA4	c.3724A>T	P.I1242F
SH_119	7	136699651	136699651	Silent	SNV	T	A	A	CHRM2	c.39T>A	P.A13A
SH_119	7	138732341	138732341	Nonstop_Mutation	SNV	T	C	C	ZC3HAV1	c.2708A>G	P.Ter903Wext*?
SH_119	7	139741562	139741562	Missense_Mutation	SNV	T	A	A	PARP12	c.1064A>T	P.Q355L
SH_119	7	141450153	141450153	Nonstop_Mutation	SNV	A	T	T	SSBP1	c.446A>T	P.Ter149Lext*?
SH_119	7	141643769	141643769	Splice_Site	SNV	A	T	T	CLEC5A	c.140-5T>A	
SH_119	7	141705384	141705384	Silent	SNV	T	C	C	MGAM	c.54T>C	P.V18V
SH_119	7	141705385	141705385	Missense_Mutation	SNV	C	A	A	MGAM	c.55C>A	P.L19I
SH_119	7	142568157	142568157	Missense_Mutation	SNV	A	G	G	EPHB6	c.1922A>G	P.E641G
SH_119	7	149522043	149522043	Splice_Site	SNV	A	T	T	SSPO	c.13841-11A>T	
SH_119	7	150691046	150691046	Missense_Mutation	SNV	A	T	T	NOS3	c.155A>T	P.H52L
SH_119	7	150716334	150716334	Missense_Mutation	SNV	T	A	A	ATG9B	c.1091A>T	P.Y364F
SH_119	7	150767992	150767992	Silent	SNV	A	T	T	SLC4A2	c.1743A>T	P.S581S
SH_119	7	150884051	150884051	Missense_Mutation	SNV	A	T	T	ASB10	c.167T>A	P.L56H
SH_119	7	150901674	150901674	Missense_Mutation	SNV	A	T	T	IQCA1L	c.260T>A	P.L87H
SH_119	7	157903543	157903543	Missense_Mutation	SNV	T	A	A	PTPRN2	c.1507A>T	P.S503C
SH_119	7	158715200	158715200	Missense_Mutation	SNV	A	T	T	WDR60	c.2054A>T	P.Q685L
SH_119	7	18066597	18066597	Missense_Mutation	SNV	A	T	T	PRPS1L1	c.809T>A	P.V270E
SH_119	7	19738071	19738071	Missense_Mutation	SNV	T	A	A	TWISTNB	c.885A>T	P.Q295H
SH_119	7	20418782	20418782	Nonsense_Mutation	SNV	T	A	A	ITGB8	c.497T>A	P.L166*
SH_119	7	2974137	2974137	Missense_Mutation	SNV	A	T	T	CARD11	c.1468T>A	P.F490I
SH_119	7	31126076	31126076	Missense_Mutation	SNV	T	A	A	ADCYAP1R1	c.748T>A	P.Y250N
SH_119	7	35733830	35733830	Silent	SNV	T	C	C	HERPUD2	c.111A>G	P.L37L
SH_119	7	38305149	38305149	Missense_Mutation	SNV	T	A	A	TARP	c.119A>T	P.Y40F
SH_119	7	4198152	4198152	Silent	SNV	A	G	G	SDK1	c.159A>G	P.S53S
SH_119	7	42006128	42006128	Missense_Mutation	SNV	T	C	C	GLI3	c.2543A>G	P.D848G
SH_119	7	45123806	45123806	Missense_Mutation	SNV	T	A	A	NACAD	c.1973A>T	P.K658M
SH_119	7	45701681	45701681	Silent	SNV	A	T	T	ADCY1	c.798A>T	P.S266S
SH_119	7	47925305	47925305	Missense_Mutation	SNV	T	A	A	PKD1L1	c.3184A>T	P.S1062C
SH_119	7	4796793	4796793	Missense_Mutation	SNV	A	T	T	FOXK1	c.1219A>T	P.T407S
SH_119	7	48431656	48431656	Silent	SNV	C	G	G	ABCA13	c.11793C>G	P.T3931T
SH_119	7	50057825	50057825	Splice_Site	SNV	A	G	G	ZPBP	c.780+11T>C	
SH_119	7	5529802	5529802	Missense_Mutation	SNV	T	A	A	FBXL18	c.2042A>T	P.E681V
SH_119	7	5568230	5568230	Missense_Mutation	SNV	T	A	A	ACTB	c.484A>T	P.T162S
SH_119	7	56142414	56142414	Nonsense_Mutation	SNV	C	T	T	SUMF2	c.532C>T	P.R178*
SH_119	7	63680478	63680478	Missense_Mutation	SNV	A	T	T	ZNF735	c.1049A>T	P.E350V
SH_119	7	72159711	72159711	Missense_Mutation	SNV	T	A	A	TYW1B	c.1472A>T	P.Q491L
SH_119	7	7622860	7622860	Missense_Mutation	SNV	A	G	G	MIOS	c.1505A>G	P.K502R
SH_119	7	8099799	8099799	Missense_Mutation	SNV	C	T	T	GLCCI1	c.887C>T	P.P296L
SH_119	7	81642783	81642783	Silent	SNV	A	G	G	CACNA2D1	c.1266T>C	P.N422N
SH_119	7	82545753	82545753	Missense_Mutation	SNV	T	A	A	PCLO	c.11549A>T	P.Q3850L
SH_119	7	82583237	82583237	Silent	SNV	A	T	T	PCLO	c.7032T>A	P.P2344P
SH_119	7	82595339	82595339	Silent	SNV	T	A	A	PCLO	c.3765A>T	P.S1255S
SH_119	7	83689823	83689823	Missense_Mutation	SNV	T	A	A	SEMA3A	c.505A>T	P.S169C
SH_119	7	90528627	90528627	Missense_Mutation	SNV	A	T	T	CDK14	c.661A>T	P.M221L
SH_119	7	94539923	94539923	Silent	SNV	T	A	A	PPP1R9A	c.498T>A	P.A166A
SH_119	7	97493810	97493810	Splice_Site	SNV	T	A	A	ASNS	c.187-2A>T	
SH_119	7	98507696	98507696	Frame_Shift_Del	INDEL	C	-	-	TRRAP	c.1369delC	P.H457fs
SH_119	7	99631564	99631564	Missense_Mutation	SNV	A	T	T	ZKSCAN1	c.1328A>T	P.H443L
SH_119	8	100729472	100729472	Silent	SNV	C	T	T	VPS13B	c.6603C>T	P.S2201S
SH_119	8	100836129	100836129	Missense_Mutation	SNV	A	T	T	VPS13B	c.9328A>T	P.T3110S
SH_119	8	101075705	101075705	Missense_Mutation	SNV	A	T	T	RGS22	c.1255T>A	P.W419R
SH_119	8	101721781	101721781	Missense_Mutation	SNV	T	A	A	PABPC1	c.1151A>T	P.Q384L
SH_119	8	104394777	104394777	Silent	SNV	T	A	A	CTHRC1	c.639T>A	P.S213S
SH_119	8	10464617	10464617	Missense_Mutation	SNV	T	A	A	RP1L1	c.6991A>T	P.T2331S
SH_119	8	108509638	108509638	Missense_Mutation	SNV	T	A	A	ANGPT1	c.149A>T	P.H50L
SH_119	8	110287574	110287574	Splice_Site	SNV	T	A	A	NUDCD1	c.1086+7A>T	
SH_119	8	110302071	110302071	Silent	SNV	T	A	A	NUDCD1	c.645A>T	P.L215L
SH_119	8	110980551	110980551	Silent	SNV	A	T	T	KCNV1	c.1269T>A	P.P423P
SH_119	8	11687801	11687801	Missense_Mutation	SNV	A	T	T	FDFT1	c.751A>T	P.N251Y
SH_119	8	12250795	12250795	Splice_Site	SNV	A	T	T	DEFB109P1	n.187T>A	
SH_119	8	125082827	125082827	Silent	SNV	A	T	T	FER1L6	c.3954A>T	P.I1318I

SH_119	8	131896862	131896862	Missense_Mutation	SNV	T	A	A	ADCY8	c.2057A>T	P.E686V
SH_119	8	139164223	139164223	Missense_Mutation	SNV	A	G	G	FAM135B	c.2495T>C	P.I832T
SH_119	8	139715588	139715588	Splice_Site	SNV	A	T	T	COL22A1	c.2520T>A	P.G840G
SH_119	8	143846567	143846567	Splice_Site	SNV	A	T	T	LYNX1	c.155-9T>A	
SH_119	8	145278066	145278066	Missense_Mutation	SNV	A	T	T	MROH1	c.1271A>T	P.E424V
SH_119	8	1513975	1513975	Missense_Mutation	SNV	A	T	T	DLGAP2	c.1117A>T	P.S373C
SH_119	8	19315934	19315934	Splice_Site	SNV	A	T	T	CSGALNACT1	c.851+3T>A	
SH_119	8	20107310	20107310	Missense_Mutation	SNV	T	A	A	LZTS1	c.1714A>T	P.S572C
SH_119	8	23712004	23712004	Silent	SNV	C	T	T	STC1	c.33G>A	P.L11L
SH_119	8	27287675	27287675	Splice_Site	SNV	A	T	T	PTK2B	c.614+3A>T	
SH_119	8	30704323	30704323	Silent	SNV	T	A	A	TEX15	c.2211A>T	P.T737T
SH_119	8	31004626	31004626	Silent	SNV	A	G	G	WRN	c.3441A>G	P.A1147A
SH_119	8	35624472	35624472	Missense_Mutation	SNV	C	T	T	UNC5D	c.2351C>T	P.A784V
SH_119	8	37696501	37696501	Missense_Mutation	SNV	G	A	A	ADGRA2	c.2287G>A	P.G763R
SH_119	8	37792013	37792013	Splice_Site	SNV	A	T	T	GOT1L1	c.1073-9T>A	
SH_119	8	38008322	38008322	Silent	SNV	T	C	C	STAR	c.15A>G	P.T5T
SH_119	8	49832965	49832965	Missense_Mutation	SNV	T	A	A	SNAI2	c.115A>T	P.M39L
SH_119	8	55541716	55541716	Silent	SNV	T	A	A	RP1	c.5274T>A	P.S1758S
SH_119	8	66605875	66605875	Splice_Site	SNV	A	T	T	MTFR1	c.67-4A>T	
SH_119	8	68934262	68934262	Splice_Site	SNV	A	T	T	PREX2	c.337-9A>T	
SH_119	8	69688676	69688676	Missense_Mutation	SNV	A	G	G	C8orf34	c.1447A>G	P.M483V
SH_119	8	7190825	7190825	Missense_Mutation	SNV	A	T	T	USP17L1	c.917A>T	P.Y306F
SH_119	8	72959392	72959392	Silent	SNV	T	A	A	TRPA1	c.1956A>T	P.R652R
SH_119	8	75233195	75233195	Missense_Mutation	SNV	A	T	T	JPH1	c.328T>A	P.W110R
SH_119	8	75263618	75263618	Missense_Mutation	SNV	A	T	T	GDAP1	c.23A>T	P.E8V
SH_119	8	77745615	77745615	Missense_Mutation	SNV	A	T	T	ZFHX4	c.3367A>T	P.T1123S
SH_119	8	79601546	79601546	Missense_Mutation	SNV	A	G	G	ZC2HC1A	c.442A>G	P.I148V
SH_119	8	80950452	80950452	Nonsense_Mutation	SNV	T	A	A	TPD52	c.574A>T	P.K192*
SH_119	8	90965555	90965555	Missense_Mutation	SNV	T	A	A	NBN	c.1762A>T	P.N588Y
SH_119	8	90995053	90995053	Missense_Mutation	SNV	T	A	A	NBN	c.68A>T	P.E23V
SH_119	8	9592454	9592454	Missense_Mutation	SNV	A	T	T	TNKS	c.2393A>T	P.Q798L
SH_119	9	100692896	100692896	Missense_Mutation	SNV	A	T	T	HEMGN	c.781T>A	P.Y261N
SH_119	9	101784386	101784386	Missense_Mutation	SNV	A	T	T	COL15A1	c.1715A>T	P.E572V
SH_119	9	101814676	101814676	Missense_Mutation	SNV	A	T	T	COL15A1	c.2911A>T	P.T971S
SH_119	9	107367401	107367401	Missense_Mutation	SNV	T	A	A	OR13C2	c.508A>T	P.R170W
SH_119	9	111903647	111903647	Missense_Mutation	SNV	A	G	G	FRRS1L	c.838T>C	P.F280L
SH_119	9	113189902	113189902	Missense_Mutation	SNV	T	A	A	SVEP1	c.5944A>T	P.R1982W
SH_119	9	113538138	113538138	Nonsense_Mutation	SNV	A	T	T	MUSK	c.1021A>T	P.R341*
SH_119	9	114486151	114486151	Silent	SNV	A	T	T	C9orf84	c.1566T>A	P.P522P
SH_119	9	115945043	115945043	Splice_Site	SNV	T	C	C	FKBP15	c.1914+3A>G	
SH_119	9	116060057	116060057	Silent	SNV	A	T	T	RNF183	c.408T>A	P.S136S
SH_119	9	125748501	125748501	Missense_Mutation	SNV	A	T	T	RABGAP1	c.393A>T	P.E131D
SH_119	9	12704631	12704631	Missense_Mutation	SNV	A	T	T	TYRP1	c.1187A>T	P.N396I
SH_119	9	129453183	129453183	Missense_Mutation	SNV	T	A	A	LMX1B	c.395T>A	P.L132Q
SH_119	9	131186439	131186439	Missense_Mutation	SNV	T	A	A	CERCAM	c.215T>A	P.L72Q
SH_119	9	131347058	131347058	Missense_Mutation	SNV	A	T	T	SPTAN1	c.2496A>T	P.E832D
SH_119	9	131398640	131398640	Missense_Mutation	SNV	T	A	A	WDR34	c.623A>T	P.Q208L
SH_119	9	132593216	132593216	Missense_Mutation	SNV	T	A	A	C9orf78	c.476A>T	P.K159M
SH_119	9	133258883	133258883	Splice_Site	SNV	A	G	G	HMCN2	c.7060-8A>G	
SH_119	9	133951258	133951258	Missense_Mutation	SNV	A	T	T	LAMC3	c.3535A>T	P.R1179W
SH_119	9	135772727	135772727	Missense_Mutation	SNV	T	A	A	TSC1	c.2819A>T	P.Q940L
SH_119	9	135864542	135864542	Missense_Mutation	SNV	G	A	A	GFI1B	c.605G>A	P.G202D
SH_119	9	137645753	137645753	Splice_Site	SNV	T	A	A	COL5A1	c.1773+4T>A	
SH_119	9	138516290	138516290	Missense_Mutation	SNV	T	A	A	GLT6D1	c.484A>T	P.I162F
SH_119	9	21187053	21187053	Missense_Mutation	SNV	T	A	A	IFNA4	c.478A>T	P.S160C
SH_119	9	26996313	26996313	Missense_Mutation	SNV	A	T	T	LRRC19	c.780T>A	P.N260K
SH_119	9	32630687	32630687	Missense_Mutation	SNV	T	A	A	TAF1L	c.4891A>T	P.T1631S
SH_119	9	33364085	33364085	Missense_Mutation	SNV	A	T	T	NFX1	c.2954A>T	P.D985V
SH_119	9	33548561	33548561	Missense_Mutation	SNV	T	A	A	ANKRD18B	c.1586T>A	P.M529K
SH_119	9	35060790	35060790	Splice_Site	SNV	T	A	A	VCP	c.1482+8A>T	
SH_119	9	36356404	36356404	Missense_Mutation	SNV	T	A	A	RNF38	c.805A>T	P.S269C
SH_119	9	36665353	36665353	Nonsense_Mutation	SNV	A	T	T	MELK	c.1060A>T	P.K354*
SH_119	9	36665561	36665561	Missense_Mutation	SNV	A	G	G	MELK	c.1268A>G	P.Y423C
SH_119	9	38596345	38596345	Missense_Mutation	SNV	T	A	A	ANKRD18A	c.992A>T	P.K331M
SH_119	9	38620267	38620267	Missense_Mutation	SNV	T	A	A	ANKRD18A	c.16A>T	P.S6C
SH_119	9	43920246	43920246	Splice_Site	SNV	T	A	A	CNTNAP3P2	n.3845-3T>A	
SH_119	9	72520864	72520864	Splice_Site	SNV	C	A	A	C9orf135	c.508-6C>A	
SH_119	9	74675410	74675410	Splice_Site	SNV	T	A	A	C9orf57	c.13+3A>T	
SH_119	9	95012487	95012487	Silent	SNV	T	A	A	IARS	c.2514A>T	P.R838R
SH_119	9	96416737	96416737	Missense_Mutation	SNV	A	T	T	PHF2	c.832A>T	P.I278F
SH_119	9	98669432	98669432	Nonsense_Mutation	SNV	A	T	T	ERCC6L2	c.700A>T	P.R234*
SH_119	X	100809225	100809225	Missense_Mutation	SNV	A	C	C	ARMCX1	c.1312A>C	P.N438H
SH_119	X	101096635	101096635	Splice_Site	SNV	T	A	A	NXF5	c.240+11A>T	
SH_119	X	10180534	10180534	Frame_Shift_Ins	INDEL	-	T	T	CLCN4	c.1136dupT	P.M379fs
SH_119	X	107920727	107920727	Splice_Site	SNV	A	T	T	COL4A5	c.3791-3A>T	
SH_119	X	108906543	108906543	Missense_Mutation	SNV	T	A	A	ACSL4	c.1602A>T	P.E534D
SH_119	X	118219478	118219478	Silent	SNV	A	T	T	KIAA1210	c.4716T>A	P.A1572A
SH_119	X	118223259	118223259	Missense_Mutation	SNV	A	T	T	KIAA1210	c.1934T>A	P.M645K
SH_119	X	12720135	12720135	Splice_Site	SNV	T	A	A	FRMPD4	c.1070+6T>A	
SH_119	X	129354456	129354456	Splice_Site	SNV	T	A	A	ZNF280C	c.1396-2A>T	
SH_119	X	12938956	12938956	Silent	SNV	A	G	G	TLR8	c.1851A>G	P.L617L
SH_119	X	133119345	133119345	Missense_Mutation	SNV	T	A	A	GPC3	c.132A>T	P.R44S
SH_119	X	133919983	133919983	Splice_Site	SNV	T	A	A	FAM122B	c.441-6A>T	
SH_119	X	135126797	135126797	Missense_Mutation	SNV	A	T	T	SLC9A6	c.2020A>T	P.I674F
SH_119	X	135430026	135430026	Silent	SNV	A	T	T	ADGRG4	c.4161A>T	P.P1387P
SH_119	X	135431670	135431670	Frame_Shift_Del	INDEL	G	-	-	ADGRG4	c.5806delG	P.A1936fs
SH_119	X	13621529	13621529	Missense_Mutation	SNV	T	A	A	EGFL6	c.494T>A	P.L165Q
SH_119	X	138667339	138667339	Splice_Site	SNV	T	A	A	MCF2	c.2813-2A>T	
SH_119	X	140969477	140969477	Silent	SNV	G	A	A	MAGEC3	c.804G>A	P.Q268Q
SH_119	X	148062323	148062323	Splice_Site	SNV	A	T	T	AFF2	c.3518+3A>T	
SH_119	X	41003771	41003771	Splice_Site	SNV	A	G	G	USP9X	c.1315-4A>G	
SH_119	X	47060933	47060933	Silent	SNV	G	C	C	UBA1	c.735G>C	P.G245G
SH_119	X	47272277	47272277	Missense_Mutation	SNV	A	T	T	ZNF157	c.805A>T	P.T269S
SH_119	X	47426129	47426129	Missense_Mutation	SNV	A	T	T	ARAF	c.649A>T	P.N217Y
SH_119	X	54048758	54048758	Missense_Mutation	SNV	T	A	A	PHF8	c.335A>T	P.K112M
SH_119	X	65409598	65409598	Nonsense_Mutation	SNV	T	A	A	HEPH	c.890T>A	P.L297*
SH_119	X	65413410	65413410	Nonsense_Mutation	SNV	T	A	A	HEPH	c.1308T>A	P.Y436*
SH_119	X	70331299	70331299	Missense_Mutation	SNV	T	A	A	IL2RG	c.91A>T	P.N31Y

SH_119	X	70361081	70361081	Missense_Mutation	SNV	A	T	T	MED12	c.6269A>T	P.Q2090L
SH_119	X	78010453	78010453	Silent	SNV	T	C	C	LPAR4	c.87T>C	P.N29N
SH_119	X	99848912	99848912	Silent	SNV	T	A	A	TNMD	c.201T>A	P.T67T
SH_119	Y	14952176	14952176	Silent	SNV	A	T	T	USP9Y	c.5724A>T	P.T1908T
SH_119	Y	21883052	21883052	Missense_Mutation	SNV	T	A	A	KDM5D	c.1610A>T	P.E537V
SH_120	1	200377084	200377084	Missense_Mutation	SNV	T	G	G	ZNF281	c.1750A>C	P.I584L
SH_120	1	215807811	215807811	Frame_Shift_Ins	INDEL	-	C	C	USH2A	c.15286dupG	P.E5096fs
SH_120	12	25398285	25398285	Missense_Mutation	SNV	C	A	A	KRAS	c.34G>T	P.G12C
SH_120	12	6566581	6566581	Missense_Mutation	SNV	A	G	G	TAPBPL	c.575A>G	P.Q192R
SH_120	13	33629207	33629207	Missense_Mutation	SNV	G	T	T	KL	c.1354G>T	P.V452F
SH_120	16	58287944	58287944	Missense_Mutation	SNV	C	T	T	CCDC113	c.271C>T	P.R91C
SH_120	2	137928486	137928486	Missense_Mutation	SNV	G	T	T	THSD7B	c.1701G>T	P.K567N
SH_120	3	105400433	105400433	Missense_Mutation	SNV	G	C	C	CBLB	c.2402C>G	P.S801C
SH_120	4	23816104	23816104	Missense_Mutation	SNV	A	C	C	PPARGC1A	c.1002T>G	P.S334R
SH_120	8	143993984	143993984	Missense_Mutation	SNV	G	A	A	CYP11B2	c.1360C>T	P.R454C
SH_120	8	2038013	2038013	Splice_Site	SNV	C	T	T	MYOM2	c.1827C>T	P.T609T
SH_120	9	114820772	114820772	Missense_Mutation	SNV	T	A	A	SUSD1	c.2045A>T	P.N682I
SH_120	9	128347883	128347883	Missense_Mutation	SNV	C	A	A	MAPKAP1	c.622G>T	P.G208W
SH_121	1	156177730	156177730	Missense_Mutation	SNV	G	C	C	SLC25A44	c.703G>C	P.V235L
SH_121	1	156202181	156202181	Missense_Mutation	SNV	G	A	A	PMF1-BGLAP	c.232G>A	P.D78N
SH_121	1	161059016	161059016	Nonsense_Mutation	SNV	G	T	T	NECTIN4	c.71C>A	P.S24*
SH_121	1	204588236	204588236	Missense_Mutation	SNV	C	T	T	LRRN2	c.885G>A	P.M295I
SH_121	1	99753679	99753679	Silent	SNV	C	T	T	PLPPR4	c.381C>T	P.S127S
SH_121	10	37508335	37508335	Missense_Mutation	SNV	A	G	G	ANKRD30A	c.3527A>G	P.Q1176R
SH_121	11	17582329	17582329	Silent	SNV	A	T	T	OTOG	c.1452A>T	P.P484P
SH_121	11	4790976	4790976	Missense_Mutation	SNV	G	T	T	OR51F1	c.172C>A	P.H58N
SH_121	11	60073583	60073583	Missense_Mutation	SNV	G	C	C	MS4A4A	c.398G>C	P.G133A
SH_121	12	112227632	112227632	Missense_Mutation	SNV	A	G	G	ALDH2	c.446A>G	P.Y149C
SH_121	12	53186577	53186577	Missense_Mutation	SNV	G	T	T	KRT3	c.941C>A	P.A314D
SH_121	12	60169172	60169172	Missense_Mutation	SNV	G	A	A	SLC16A7	c.1096G>A	P.V366M
SH_121	12	63989812	63989812	Silent	SNV	C	A	A	DPY19L2	c.1467G>T	P.P489P
SH_121	12	65232608	65232608	Silent	SNV	G	T	T	TBC1D30	c.909G>T	P.L303L
SH_121	12	65232609	65232609	Missense_Mutation	SNV	G	T	T	TBC1D30	c.910G>T	P.A304S
SH_121	13	48953730	48953730	Nonsense_Mutation	SNV	C	T	T	RB1	c.1333C>T	P.R445*
SH_121	14	31626160	31626160	Silent	SNV	T	C	C	HECTD1	c.1818A>G	P.K606K
SH_121	16	19722744	19722744	Missense_Mutation	SNV	C	G	G	KNOP1	c.937G>C	P.E313Q
SH_121	16	70590870	70590870	Missense_Mutation	SNV	A	G	G	SF3B3	c.1948A>G	P.K650E
SH_121	2	188228181	188228181	Silent	SNV	T	C	C	CALCRL	c.549A>G	P.S183S
SH_121	2	189434045	189434045	Missense_Mutation	SNV	T	C	C	GULP1	c.479T>C	P.V160A
SH_121	3	186395235	186395249	Missense_Mutation	INDEL	CATGGACAC	-	-	HRG	69delCCCCATC	P.P386_H390del
SH_121	3	45988342	45988342	Silent	SNV	C	T	T	CXCR6	c.369C>T	P.I123I
SH_121	3	57131806	57131806	Missense_Mutation	SNV	G	T	T	IL17RD	c.1493C>A	P.A498D
SH_121	3	9828890	9828890	Silent	SNV	G	A	A	TADA3	c.699C>T	P.D233D
SH_121	4	37831635	37831635	Missense_Mutation	SNV	A	C	C	PGM2	c.131A>C	P.K44T
SH_121	5	127637068	127637068	Splice_Site	SNV	A	G	G	FBN2	c.6046+6T>C	
SH_121	5	136964010	136964010	Missense_Mutation	SNV	T	C	C	KLHL3	c.1471A>G	P.M491V
SH_121	5	162910182	162910182	Splice_Site	SNV	A	G	G	HMMR	c.1685+4A>G	
SH_121	6	129621954	129621954	Silent	SNV	T	C	C	LAMA2	c.3111T>C	P.I1037I
SH_121	6	24843737	24843737	Nonsense_Mutation	SNV	C	A	A	FAM65B	c.1210G>T	P.E404*
SH_121	6	46135309	46135309	Missense_Mutation	SNV	G	T	T	ENPP5	c.691C>A	P.L231M
SH_121	7	16745698	16745698	Missense_Mutation	SNV	A	C	C	BZW2	c.1245A>C	P.E415D
SH_121	8	130789776	130789776	Missense_Mutation	SNV	C	A	A	GSDMC	c.58G>T	P.D20Y
SH_121	8	75925113	75925113	Splice_Site	SNV	T	C	C	CRISPLD1	c.-65-12T>C	
SH_121	9	113166647	113166647	Missense_Mutation	SNV	T	C	C	SVEP1	c.9626A>G	P.E3209G
SH_122	1	115217380	115217380	Missense_Mutation	SNV	A	G	G	AMPD1	c.1892T>C	P.L631S
SH_122	1	151142989	151142992	Splice_Site	INDEL	ACTC	-	-	TMOD4	5+3_1015+6delGAGT	
SH_122	1	167088659	167088659	Missense_Mutation	SNV	G	A	A	DUSP27	c.611G>A	P.R204Q
SH_122	1	214542866	214542866	Missense_Mutation	SNV	C	A	A	PTPN14	c.3205G>T	P.V1069L
SH_122	11	118065080	118065080	Missense_Mutation	SNV	T	G	G	JAML	c.1145A>C	P.K382T
SH_122	11	56000281	56000281	Missense_Mutation	SNV	C	A	A	OR5T2	c.381G>T	P.K127N
SH_122	11	57296320	57296320	Missense_Mutation	SNV	G	A	A	TIMM10	c.143C>T	P.S48F
SH_122	11	67191096	67191096	Missense_Mutation	SNV	A	G	G	CARNS1	c.1877A>G	P.Q626R
SH_122	11	82705139	82705139	Missense_Mutation	SNV	G	T	T	RAB30	c.119C>A	P.A40D
SH_122	11	9770684	9770684	Silent	SNV	G	A	A	SWAP70	c.1431G>A	P.K477K
SH_122	12	113728805	113728805	Splice_Site	SNV	T	C	C	TPCN1	c.2175+2T>C	
SH_122	12	89819052	89819052	Silent	SNV	C	T	T	POC1B	c.1092G>A	P.T364T
SH_122	15	33928584	33928584	Missense_Mutation	SNV	G	A	A	RYR3	c.3389G>A	P.R1130H
SH_122	15	91025246	91025246	Silent	SNV	G	A	A	IQGAP1	c.3384G>A	P.K1128K
SH_122	17	17380384	17380384	Missense_Mutation	SNV	G	A	A	MED9	c.29G>A	P.R10Q
SH_122	17	29685499	29685499	Missense_Mutation	SNV	C	T	T	NF1	c.7972C>T	P.H2658Y
SH_122	17	37903122	37903122	Missense_Mutation	SNV	G	A	A	GRB7	c.1571G>A	P.R524H
SH_122	19	36293942	36293942	Missense_Mutation	SNV	G	A	A	PRODH2	c.1268C>T	P.A423V
SH_122	19	38382377	38382377	Missense_Mutation	SNV	T	G	G	WDR87	c.3209A>C	P.E1070A
SH_122	19	55435147	55435147	Silent	SNV	G	A	A	NLRP7	c.3075C>T	P.S1025S
SH_122	19	55857643	55857643	Silent	SNV	C	T	T	KMT5C	c.633C>T	P.D211D
SH_122	2	209113112	209113112	Missense_Mutation	SNV	C	A	A	IDH1	c.395G>T	P.R132L
SH_122	3	108780897	108780897	Missense_Mutation	SNV	T	A	A	MORC1	c.904A>T	P.I302L
SH_122	4	70346634	70346634	Splice_Site	SNV	C	G	G	UGT2B4	c.1091-6G>C	
SH_122	5	121356358	121356358	Missense_Mutation	SNV	C	G	G	SRFBP1	c.928C>G	P.L310V
SH_122	5	140167440	140167440	Missense_Mutation	SNV	C	A	A	PCDHA1	c.1565C>A	P.P522H
SH_122	5	64748604	64748604	Missense_Mutation	SNV	G	A	A	ADAMTS6	c.773C>T	P.A258V
SH_122	6	51720720	51720720	Missense_Mutation	SNV	C	T	T	PKHD1	c.7882G>A	P.E2628K
SH_122	7	100606261	100606261	Missense_Mutation	SNV	G	A	A	MUC3A	c.3607G>A	P.D1203N
SH_122	7	47842947	47842947	Splice_Site	SNV	G	C	C	PKD1L1	c.7828-5C>G	
SH_122	7	6062981	6062981	Missense_Mutation	SNV	G	A	A	AIMP2	c.502G>A	P.E168K
SH_122	8	1719266	1719266	Silent	SNV	C	T	T	CLN8	c.46C>T	P.L16L
SH_122	X	12736297	12736297	Missense_Mutation	SNV	G	T	T	FRMPD4	c.3352G>T	P.A1118S
SH_122	X	138633418	138633418	Missense_Mutation	SNV	T	C	C	F9	c.718T>C	P.W240R
SH_122	X	149678287	149678287	Missense_Mutation	SNV	A	G	G	MAMLD1	c.1898A>G	P.Y633C
SH_123	1	100155038	100155041	Frame_Shift_Del	INDEL	AATG	-	-	PALMD	223_1226delAT	P.N408fs
SH_123	1	110022069	110022069	Missense_Mutation	SNV	C	T	T	SYPL2	c.718C>T	P.H240Y
SH_123	1	160326909	160326909	Missense_Mutation	SNV	G	T	T	NCSTN	c.1813G>T	P.A605S
SH_123	1	196274360	196274360	Splice_Site	SNV	T	C	C	KCNT2	c.2523+4A>G	
SH_123	1	19981627	19981627	Missense_Mutation	SNV	G	A	A	NBL1	c.104G>A	P.C35Y
SH_123	1	6314094	6314094	Missense_Mutation	SNV	T	C	C	GPR153	c.470A>G	P.Y157C
SH_123	1	78433858	78433858	Missense_Mutation	SNV	G	C	C	FUBP1	c.241C>G	P.Q81E
SH_123	10	115981204	115981204	Missense_Mutation	SNV	C	G	G	TDRD1	c.2859C>G	P.N953K
SH_123	10	82331298	82331298	Silent	SNV	C	T	T	SH2D4B	c.312C>T	P.A104A

SH_123	11	1016371	1016373	Missense_Mutation	INDEL	AAG	-	-	MUC6	6428_6430delC	P.S2143del
SH_123	11	32118719	32118719	Missense_Mutation	SNV	G	C	C	RCN1	c.284G>C	P.G95A
SH_123	11	4608632	4608632	Missense_Mutation	SNV	C	A	A	OR52I2	c.590C>A	P.P197H
SH_123	11	5799345	5799345	Missense_Mutation	SNV	G	A	A	OR52N5	c.520C>T	P.R174C
SH_123	11	60496979	60496979	Missense_Mutation	SNV	T	A	A	MS4A18	c.191T>A	P.V64E
SH_123	11	687999	687999	Nonsense_Mutation	SNV	G	C	C	DEAF1	c.576C>G	P.Y192*
SH_123	11	77594962	77594962	Missense_Mutation	SNV	G	A	A	INTS4	c.2629C>T	P.P877S
SH_123	12	109182506	109182506	Missense_Mutation	SNV	T	C	C	SSH1	c.2408A>G	P.N803S
SH_123	12	15262150	15262150	Missense_Mutation	SNV	C	T	T	RERG	c.437G>A	P.R146Q
SH_123	12	52627311	52627311	Silent	SNV	G	A	A	KRT7	c.231G>A	P.R77R
SH_123	12	56568456	56568456	Missense_Mutation	SNV	C	T	T	SMARCC2	c.1475G>A	P.G492D
SH_123	12	62929421	62929421	Missense_Mutation	SNV	G	A	A	MON2	c.1832G>A	P.G611D
SH_123	12	69085850	69085850	Missense_Mutation	SNV	A	G	G	NUP107	c.319A>G	P.I107V
SH_123	13	101936280	101936280	Splice_Site	SNV	G	T	T	NALCN	c.1134+4C>A	
SH_123	13	46099156	46099156	Missense_Mutation	SNV	A	G	G	COG3	c.2200A>G	P.T734A
SH_123	14	95669906	95669906	Missense_Mutation	SNV	C	T	T	CLMN	c.1780G>A	P.D594N
SH_123	14	96768405	96768405	Missense_Mutation	SNV	C	T	T	ATG2B	c.5078G>A	P.R1693K
SH_123	15	45386807	45386807	Missense_Mutation	SNV	G	C	C	DUOX2	c.4478C>G	P.P1493R
SH_123	16	28913639	28913639	Frame_Shift_Ins	INDEL	-	C	C	ATP2A1	c.2089dupC	P.R697fs
SH_123	16	31435158	31435158	Splice_Site	INDEL	-	C	C	ITGAD	c.3049-3dupC	
SH_123	16	70996011	70996011	Missense_Mutation	SNV	C	T	T	HYDIN	c.5819G>A	P.G1940E
SH_123	16	86601102	86601102	Missense_Mutation	SNV	G	A	A	FOXC2	c.161G>A	P.R54H
SH_123	17	29483057	29483071	Missense_Mutation	INDEL	GGAATGTCT	-	-	NF1	4delGGAATGTC	P.K40_I44del
SH_123	17	38079462	38079462	Missense_Mutation	SNV	G	C	C	ORMDL3	c.229C>G	P.Q77E
SH_123	17	39777943	39777943	Missense_Mutation	SNV	C	T	T	KRT17	c.736G>A	P.G246S
SH_123	17	61621609	61621609	Splice_Site	SNV	G	A	A	KCNH6	c.2234-1G>A	
SH_123	17	7573976	7573976	Missense_Mutation	SNV	T	C	C	TP53	c.1051A>G	P.K351E
SH_123	17	79654108	79654108	Silent	SNV	C	T	T	HGS	c.274C>T	P.L92L
SH_123	18	76757181	76757181	Silent	SNV	C	A	A	SALL3	c.3762C>A	P.G1254G
SH_123	19	10624875	10624875	Silent	SNV	C	T	T	S1PR5	c.813G>A	P.L271L
SH_123	19	19762593	19762593	Missense_Mutation	SNV	G	A	A	ATP13A1	c.2240C>T	P.T747M
SH_123	19	22586244	22586244	Missense_Mutation	SNV	T	C	C	ZNF98	c.101A>G	P.Q34R
SH_123	19	33089162	33089162	Silent	SNV	T	C	C	ANKRD27	c.3042A>G	P.G1014G
SH_123	19	35800921	35800921	Missense_Mutation	SNV	A	G	G	MAG	c.1301A>G	P.E434G
SH_123	19	36564435	36564435	Splice_Site	SNV	T	G	G	WDR62	c.1233+2T>G	
SH_123	19	41269577	41269577	Missense_Mutation	SNV	A	G	G	SNRPA	c.686A>G	P.N229S
SH_123	19	41509955	41509955	Missense_Mutation	SNV	C	A	A	CYP2B6	c.221C>A	P.P74H
SH_123	19	44513074	44513074	Splice_Site	SNV	C	T	T	ZNF230	c.142+6C>T	
SH_123	19	46375654	46375654	Nonsense_Mutation	SNV	C	T	T	FOXA3	c.391C>T	P.Q131*
SH_123	19	46388166	46388166	Silent	SNV	G	A	A	IRF2BP1	c.867C>T	P.V289V
SH_123	19	47126919	47126919	Silent	SNV	G	A	A	PTGIR	c.564C>T	P.Y188Y
SH_123	19	48832748	48832748	Splice_Site	SNV	C	G	G	EMP3	c.321C>G	P.T107T
SH_123	19	49392831	49392831	Missense_Mutation	SNV	G	A	A	TULP2	c.572C>T	P.P191L
SH_123	19	50826910	50826910	Missense_Mutation	SNV	G	T	T	KCNC3	c.1300C>A	P.L434M
SH_123	19	52471891	52471891	Silent	SNV	A	G	G	ZNF350	c.178T>C	P.L60L
SH_123	19	59027759	59027759	Splice_Site	SNV	C	T	T	ZBTB45	c.1279+3G>A	
SH_123	2	135884178	135884178	Missense_Mutation	SNV	C	G	G	RAB3GAP1	c.925C>G	P.H309D
SH_123	2	234681191	234681191	Frame_Shift_Del	INDEL	T	-	-	UGT1A6	c.1585delT	P.S529fs
SH_123	2	58311207	58311207	Splice_Site	INDEL	-	T	T	VRK2	c.137-9dupT	
SH_123	20	40733366	40733366	Splice_Site	SNV	G	T	T	PTPRT	c.3448-8C>A	
SH_123	20	56234706	56234706	Frame_Shift_Ins	INDEL	-	CATCATCAA	CATCATCAA	PMEPA1	2_73insTTGATG	M24_V25insLMM
SH_123	21	10910352	10910352	Silent	SNV	G	A	A	TPTE	c.990C>T	P.D330D
SH_123	21	35468920	35468920	Missense_Mutation	SNV	A	G	G	SLC5A3	c.1423A>G	P.M475V
SH_123	22	18371802	18371802	Missense_Mutation	SNV	C	G	G	MICAL3	c.1889G>C	P.S630T
SH_123	22	40760365	40760365	Missense_Mutation	SNV	G	A	A	ADSL	c.1187G>A	P.R396H
SH_123	22	46611071	46611071	Missense_Mutation	SNV	C	A	A	PPARA	c.210C>A	P.D70E
SH_123	3	183209922	183209922	Silent	SNV	G	A	A	KLHL6	c.1659C>T	P.I553I
SH_123	3	38156521	38156521	Missense_Mutation	SNV	G	A	A	DLEC1	c.3842G>A	P.R1281H
SH_123	4	100273815	100273815	Frame_Shift_Del	INDEL	T	-	-	ADH1C	c.18delA	P.V7fs
SH_123	4	52895026	52895026	Missense_Mutation	SNV	C	T	T	SGCB	c.491G>A	P.S164N
SH_123	4	5418608	5418608	Missense_Mutation	SNV	T	C	C	STK32B	c.368T>C	P.V123A
SH_123	4	5633750	5633750	Missense_Mutation	SNV	C	G	G	EVC2	c.1240G>C	P.E414Q
SH_123	5	150663622	150663622	Silent	SNV	G	T	T	SLC36A3	c.1080C>A	P.L360L
SH_123	5	26885718	26885718	Splice_Site	SNV	C	T	T	CDH9	c.1882+5G>A	
SH_123	6	128135034	128135034	Missense_Mutation	SNV	T	C	C	THEMIS	c.752A>G	P.E251G
SH_123	6	69666596	69666596	Missense_Mutation	SNV	G	A	A	ADGRB3	c.1420G>A	P.G474S
SH_123	6	90660516	90660516	Missense_Mutation	SNV	C	T	T	BACH2	c.1309G>A	P.A437T
SH_123	7	100490833	100490833	Missense_Mutation	SNV	C	G	G	ACHE	c.1021G>C	P.D341H
SH_123	7	103969292	103969306	Missense_Mutation	INDEL	AGCTGTACC	-	-	LHFPL3	delGCTGTACCA	P.K23_T27del
SH_123	7	121943770	121943770	Frame_Shift_Ins	INDEL	-	T	T	FEZF1	c.721dupA	P.I241fs
SH_123	7	138863321	138863321	Missense_Mutation	SNV	G	A	A	TTC26	c.1219G>A	P.G407S
SH_123	7	141795424	141795424	Silent	SNV	G	A	A	MGAM	c.4830G>A	P.Q1610Q
SH_123	8	57079730	57079730	Missense_Mutation	SNV	C	T	T	PLAG1	c.575G>A	P.R192H
SH_123	9	13176348	13176348	Missense_Mutation	SNV	C	G	G	MPDZ	c.2718G>C	P.Q906H
SH_123	9	86421324	86421324	Nonsense_Mutation	SNV	G	A	A	GKAP1	c.109C>T	P.R37*
SH_123	X	107381045	107381045	Missense_Mutation	SNV	C	T	T	ATG4A	c.328C>T	P.R110C
SH_123	X	3241027	3241027	Missense_Mutation	SNV	G	T	T	MXRA5	c.2699C>A	P.S900Y
SH_123	X	7175381	7175381	Missense_Mutation	SNV	C	T	T	STS	c.251C>T	P.T84I
SH_125	1	205273506	205273506	Missense_Mutation	SNV	C	A	A	NUAK2	c.1091G>T	P.G364V
SH_125	1	233121966	233121966	Missense_Mutation	SNV	A	T	T	PCNX2	c.6112T>A	P.F2038I
SH_125	1	245530375	245530375	Silent	SNV	C	T	T	KIF26B	c.705C>T	P.H235H
SH_125	1	39920706	39920706	Missense_Mutation	SNV	G	T	T	MACF1	c.14835G>T	P.Q4945H
SH_125	10	125516776	125516776	Nonsense_Mutation	SNV	C	A	A	CPXM2	c.1870G>T	P.E624*
SH_125	10	27040647	27040647	Missense_Mutation	SNV	G	A	A	ABI1	c.1150C>T	P.P384S
SH_125	10	49203417	49203417	Silent	SNV	T	C	C	FAM25G	c.264A>G	P.G88G
SH_125	10	61552723	61552723	Silent	SNV	C	G	G	CCDC6	c.1377G>C	P.S459S
SH_125	12	122748705	122748705	Silent	SNV	C	A	A	VPS33A	c.144G>T	P.L48L
SH_125	12	56481655	56481655	Silent	SNV	G	A	A	ERBB3	c.690G>A	P.E230E
SH_125	14	21109124	21109124	Missense_Mutation	SNV	T	C	C	OR6S1	c.727A>G	P.T243A
SH_125	15	48052552	48052552	Missense_Mutation	SNV	A	T	T	SEMA6D	c.161A>T	P.Q54L
SH_125	15	81638761	81638761	Frame_Shift_Del	INDEL	T	-	-	TMC3	c.1217delA	P.N406fs
SH_125	15	89836213	89836213	Frame_Shift_Ins	INDEL	-	A	A	FANCI	c.2216dupA	P.N739fs
SH_125	17	20361556	20361556	Missense_Mutation	SNV	C	T	T	LGALS9B	c.273G>A	P.M91I
SH_125	17	7577534	7577534	Missense_Mutation	SNV	C	A	A	TP53	c.747G>T	P.R249S
SH_125	17	77755496	77755496	Nonsense_Mutation	SNV	G	T	T	CBX2	c.184G>T	P.E62*
SH_125	18	678707	678707	Missense_Mutation	SNV	T	G	G	ENOSF1	c.661A>C	P.T221P
SH_125	19	40704404	40704404	Missense_Mutation	SNV	T	A	A	MAP3K10	c.805T>A	P.W269R
SH_125	19	53612744	53612744	Missense_Mutation	SNV	A	G	G	ZNF415	c.554T>C	P.I185T

SH_125	19	55284899	55284899	Missense_Mutation	SNV	G	A	A	KIR2DL1	c.185G>A	P.R62K
SH_125	19	58153221	58153221	Missense_Mutation	SNV	G	T	T	ZNF211	c.1562G>T	P.G521V
SH_125	2	55470681	55470681	Missense_Mutation	SNV	C	G	G	MTIF2	c.1435G>C	P.E479Q
SH_125	20	3759058	3759058	Splice_Site	SNV	G	C	C	SPEF1	c.603+10C>G	
SH_125	20	62597586	62597586	Silent	SNV	G	T	T	ZNF512B	c.942C>A	P.I314I
SH_125	21	32007769	32007769	Missense_Mutation	SNV	G	T	T	KRTAP20-2	c.187G>T	P.G63C
SH_125	21	36259172	36259172	Missense_Mutation	SNV	G	A	A	RUNX1	c.238C>T	P.R80C
SH_125	21	47965164	47965164	Missense_Mutation	SNV	G	T	T	DIP2A	c.2162G>T	P.G721V
SH_125	22	18299497	18299497	Missense_Mutation	SNV	C	T	T	MICAL3	c.5404G>A	P.D1802N
SH_125	22	19209622	19209622	Splice_Site	SNV	G	T	T	CLTCL1	c.2419-6C>A	
SH_125	22	20230163	20230163	Missense_Mutation	SNV	C	T	T	RTN4R	c.493G>A	P.A165T
SH_125	22	50968978	50968978	Silent	SNV	G	T	T	ODF3B	c.693C>A	P.P231P
SH_125	3	142277598	142277598	Missense_Mutation	SNV	G	T	T	ATR	c.1753C>A	P.H585N
SH_125	3	194152498	194152498	Missense_Mutation	SNV	T	G	G	ATP13A3	c.2369A>C	P.H790P
SH_125	3	45077180	45077180	Missense_Mutation	SNV	G	A	A	CLEC3B	c.247G>A	P.V83M
SH_125	4	17634226	17634226	Nonsense_Mutation	SNV	G	A	A	FAM184B	c.3112C>T	P.Q1038*
SH_125	4	75959104	75959104	Missense_Mutation	SNV	G	C	C	PARM1	c.781G>C	P.A261P
SH_125	5	125819209	125819209	Missense_Mutation	SNV	G	T	T	GRAMD3	c.835G>T	P.D279Y
SH_125	5	139661100	139661100	Silent	SNV	C	T	T	PFDN1	c.219G>A	P.K73K
SH_125	5	32059467	32059467	Splice_Site	SNV	G	T	T	PDZD2	c.2318+5G>T	
SH_125	5	37138912	37138912	Missense_Mutation	SNV	T	C	C	C5orf42	c.8540A>G	P.D2847G
SH_125	6	107372386	107372386	Silent	SNV	G	A	A	C6orf203	c.669G>A	P.V223V
SH_125	6	108214859	108214859	Nonsense_Mutation	SNV	G	A	A	SEC63	c.1501C>T	P.Q501*
SH_125	6	138584341	138584341	Missense_Mutation	SNV	T	A	A	ARFGEF3	c.1721T>A	P.I574K
SH_125	6	155571044	155571044	Nonsense_Mutation	SNV	G	T	T	TIAM2	c.667G>T	P.E223*
SH_125	6	168273023	168273023	Missense_Mutation	SNV	G	T	T	AFDN	c.544G>T	P.D182Y
SH_125	6	46847555	46847555	Missense_Mutation	SNV	C	T	T	ADGRF5	c.1036G>A	P.G346S
SH_125	6	65301096	65301096	Missense_Mutation	SNV	G	A	A	EYS	c.4664C>T	P.T1555I
SH_125	6	75844446	75844446	Silent	SNV	C	T	T	COL12A1	c.5520G>A	P.K1840K
SH_125	6	83949327	83949327	Silent	SNV	G	C	C	ME1	c.843C>G	P.L281L
SH_125	7	100549646	100549646	Missense_Mutation	SNV	T	C	C	MUC3A	c.227T>C	P.M76T
SH_125	7	104751254	104751254	Missense_Mutation	SNV	A	C	C	KMT2E	c.4007A>C	P.N1336T
SH_125	7	138719380	138719380	Missense_Mutation	SNV	A	T	T	ZC3HAV1L	c.410T>A	P.M137K
SH_125	7	143747693	143747693	Missense_Mutation	SNV	A	G	G	OR2A5	c.199A>G	P.I67V
SH_125	8	142450415	142450415	Missense_Mutation	SNV	C	T	T	MROH5	c.3133G>A	P.V1045M
SH_125	9	6498096	6498096	Frame_Shift_Del	INDEL	G	-	-	UHRF2	c.1846delG	P.E616fs
SH_125	9	77362834	77362834	Splice_Site	SNV	T	C	C	TRPM6	c.5043-4A>G	
SH_125	X	100601518	100601518	Missense_Mutation	SNV	T	A	A	TIMM8A	c.263A>T	P.K88M
SH_125	X	11215073	11215073	Silent	SNV	T	C	C	ARHGAP6	c.792A>G	P.S264S
SH_125	X	127185710	127185710	Missense_Mutation	SNV	C	G	G	ACTRT1	c.476G>C	P.G159A
SH_127	1	2421257	2421257	Missense_Mutation	SNV	A	T	T	PLCH2	c.1385A>T	P.E462V
SH_127	10	69700720	69700720	Missense_Mutation	SNV	T	C	C	HERC4	c.2480A>G	P.K827R
SH_127	10	89264720	89264736	Frame_Shift_Del	INDEL	GCCCTGGCT	-	-	MINPP1	elGGCCCTGGCT	P.A20fs
SH_127	10	96269940	96269940	Splice_Site	SNV	A	T	T	TBC1D12	c.1689+4A>T	
SH_127	11	56258310	56258310	Silent	SNV	C	A	A	OR5M8	c.537G>T	P.A179A
SH_127	11	60711227	60711244	Missense_Mutation	INDEL	TTGGCGATG	-	-	SLC15A3	delGAGGACATC	P.E305_F310del
SH_127	11	73471602	73471602	Splice_Site	SNV	C	T	T	RAB6A	c.70+9G>A	
SH_127	12	54394068	54394068	Silent	SNV	G	C	C	HOXC9	c.96G>C	P.G32G
SH_127	12	6554406	6554406	Splice_Site	SNV	G	C	C	CD27	c.136+9G>C	
SH_127	14	52985913	52985913	Missense_Mutation	SNV	T	A	A	TXNDC16	c.476A>T	P.Y159F
SH_127	15	74564066	74564066	Missense_Mutation	SNV	A	T	T	CCDC33	c.569A>T	P.E190V
SH_127	15	76494607	76494609	Splice_Site	INDEL	TAA	-	-	TMEM266	21+2_1021+4delTAA	
SH_127	2	162805701	162805701	Missense_Mutation	SNV	T	G	G	SLC4A10	c.2309T>G	P.L770R
SH_127	3	161214760	161214760	Missense_Mutation	SNV	A	T	T	OTOL1	c.165A>T	P.E55D
SH_127	3	168802733	168802733	Silent	SNV	C	T	T	MECOM	c.3315G>A	P.A1105A
SH_127	3	44948517	44948517	Missense_Mutation	SNV	C	A	A	TGM4	c.1152C>A	P.F384L
SH_127	3	45988819	45988819	Nonsense_Mutation	SNV	C	A	A	CXCR6	c.846C>A	P.C282*
SH_127	4	155411772	155411772	Silent	SNV	G	A	A	DCHS2	c.736C>T	P.L246L
SH_127	5	140744890	140744890	Silent	SNV	T	A	A	PCDHGA5	c.993T>A	P.A331A
SH_127	6	154735543	154735543	Silent	SNV	G	C	C	CNKSR3	c.960C>G	P.P320P
SH_127	6	27277644	27277644	Missense_Mutation	SNV	C	G	G	POM121L2	c.2306G>C	P.G769A
SH_127	6	3140625	3140625	Missense_Mutation	SNV	A	T	T	BPHL	c.619A>T	P.I207F
SH_127	6	7230336	7230336	Missense_Mutation	SNV	C	G	G	RREB1	c.2004C>G	P.I668M
SH_127	7	104782874	104782874	Missense_Mutation	SNV	T	A	A	SRPK2	c.1091A>T	P.D364V
SH_127	7	151699844	151699844	Missense_Mutation	SNV	T	A	A	GALNTL5	c.704T>A	P.V235E
SH_127	7	30893042	30893042	Silent	SNV	C	T	T	FAM188B	c.1644C>T	P.D548D
SH_127	7	34851445	34851445	Missense_Mutation	SNV	A	T	T	NPSR1	c.415A>T	P.I139F
SH_127	7	36446098	36446098	Missense_Mutation	SNV	G	T	T	ANLN	c.796G>T	P.D266Y
SH_128	1	109649750	109649750	Splice_Site	SNV	G	T	T	C1orf194	c.163-6C>A	
SH_128	1	109649751	109649751	Splice_Site	SNV	A	T	T	C1orf194	c.163-7T>A	
SH_128	1	110737263	110737263	Silent	SNV	C	A	A	SLC6A17	c.1362C>A	P.A454A
SH_128	1	151867527	151867527	Silent	SNV	A	C	C	THEM4	c.243T>G	P.R81R
SH_128	1	152081126	152081126	Missense_Mutation	SNV	G	T	T	TCHH	c.4567C>A	P.Q1523K
SH_128	1	158724748	158724748	Missense_Mutation	SNV	C	T	T	OR6K6	c.143C>T	P.A48V
SH_128	1	186008949	186008949	Missense_Mutation	SNV	G	T	T	HMCN1	c.6118G>T	P.A2040S
SH_128	1	1956375	1956375	Splice_Site	SNV	C	T	T	GABRD	c.69-6C>T	
SH_128	1	201061111	201061111	Missense_Mutation	SNV	G	C	C	CACNA1S	c.530C>G	P.S177W
SH_128	1	215813980	215813980	Frame_Shift_Ins	INDEL	-	C	C	USH2A	c.14887dupG	P.E4963fs
SH_128	1	243319677	243319677	Nonsense_Mutation	SNV	T	A	A	CEP170	c.3463A>T	P.K1155*
SH_128	1	2576820	2576820	Silent	SNV	G	T	T	TTC34	c.870C>A	P.G290G
SH_128	1	37948355	37948355	Missense_Mutation	SNV	G	A	A	ZC3H12A	c.943G>A	P.G315R
SH_128	1	40872474	40872474	Missense_Mutation	SNV	A	G	G	SMAP2	c.80A>G	P.N27S
SH_128	1	7798224	7798224	Missense_Mutation	SNV	C	A	A	CAMTA1	c.3864C>A	P.S1288R
SH_128	10	129904172	129904172	Missense_Mutation	SNV	C	T	T	MKI67	c.4852G>A	P.E1618K
SH_128	10	43615037	43615037	Silent	SNV	C	T	T	RET	c.2451C>T	P.R817R
SH_128	10	75830581	75830581	Splice_Site	SNV	A	T	T	VCL	c.390+3A>T	
SH_128	10	89622183	89622183	Missense_Mutation	SNV	C	T	T	KLLN	c.62G>A	P.R21Q
SH_128	11	124487256	124487256	Missense_Mutation	SNV	C	A	A	PANX3	c.411C>A	P.S137R
SH_128	11	13438723	13438723	Silent	SNV	A	G	G	BTBD10	c.525T>C	P.T175T
SH_128	11	17122828	17122828	Silent	SNV	T	A	A	PIK3C2A	c.4005A>T	P.S1335S
SH_128	11	21250965	21250965	Missense_Mutation	SNV	A	T	T	NELL1	c.1598A>T	P.K533I
SH_128	11	34938235	34938235	Silent	SNV	G	A	A	PDHX	c.33G>A	P.P11P
SH_128	11	47333296	47333296	Missense_Mutation	SNV	A	G	G	MADD	c.3863A>G	P.D1288G
SH_128	11	62288510	62288510	Missense_Mutation	SNV	G	C	C	AHNAK	c.13379C>G	P.S4460C
SH_128	11	68700868	68700868	Missense_Mutation	SNV	A	T	T	IGHMBP2	c.1337A>T	P.Q446L
SH_128	11	68854064	68854064	Missense_Mutation	SNV	A	G	G	TPCN2	c.2077A>G	P.I693V
SH_128	11	76825287	76825287	Splice_Site	SNV	G	T	T	CAPN5	c.507-1G>T	
SH_128	12	123678991	123678991	Missense_Mutation	SNV	T	A	A	MPHOSPH9	c.2183A>T	P.Y728F

SH_128	12	124399435	124399435	Silent	SNV	C	T	T	DNAH10	c.10257C>T	P.S3419S
SH_128	12	133502748	133502748	Missense_Mutation	SNV	C	G	G	ZNF605	c.1230G>C	P.Q410H
SH_128	12	51236851	51236851	Missense_Mutation	SNV	C	T	T	TMPRSS12	c.104C>T	P.P35L
SH_128	12	54379492	54379492	Missense_Mutation	SNV	G	T	T	HOXC10	c.449G>T	P.R150L
SH_128	12	661265	661265	Missense_Mutation	SNV	C	A	A	B4GALNT3	c.1144C>A	P.R382S
SH_128	12	9070332	9070332	Missense_Mutation	SNV	G	T	T	PHC1	c.59G>T	P.G20V
SH_128	13	114062306	114062306	Missense_Mutation	SNV	G	T	T	LOC101928841	c.199C>A	P.L67I
SH_128	13	114152743	114152743	Missense_Mutation	SNV	A	T	T	TMCO3	c.531A>T	P.L177F
SH_128	13	99947324	99947324	Frame_Shift_Del	INDEL	T	-	-	GPR183	c.1076delA	P.N359fs
SH_128	14	102904496	102904496	Missense_Mutation	SNV	G	T	T	TECPR2	c.2532G>T	P.W844C
SH_128	14	70245218	70245218	Missense_Mutation	SNV	C	T	T	SLC10A1	c.775G>A	P.G259R
SH_128	14	77951074	77951074	Silent	SNV	C	T	T	ISM2	c.330G>A	P.Q110Q
SH_128	14	94039067	94039067	Missense_Mutation	SNV	C	G	G	UNC79	c.1444C>G	P.L482V
SH_128	14	99969294	99969294	Missense_Mutation	SNV	T	A	A	CCNK	c.984T>A	P.S328R
SH_128	15	63882902	63882902	Silent	SNV	C	T	T	USP3	c.1308C>T	P.G436G
SH_128	15	74003483	74003483	Missense_Mutation	SNV	G	T	T	CD276	c.1554G>T	P.Q518H
SH_128	15	74481552	74481552	Frame_Shift_Del	INDEL	C	-	-	STRA6	c.994delG	P.D332fs
SH_128	15	78780991	78780991	Missense_Mutation	SNV	A	T	T	IREB2	c.1204A>T	P.T402S
SH_128	16	28328771	28328771	Missense_Mutation	SNV	G	A	A	SBK1	c.59G>A	P.G20E
SH_128	16	48226422	48226422	Splice_Site	SNV	G	T	T	ABCC11	c.2706+9C>A	
SH_128	17	36622676	36622676	Missense_Mutation	SNV	G	A	A	ARHGAP23	c.752G>A	P.R251H
SH_128	17	55183902	55183902	Silent	SNV	A	G	G	AKAP1	c.1077A>G	P.A359A
SH_128	17	57741313	57741313	Silent	SNV	A	G	G	CLTC	c.1491A>G	P.A497A
SH_128	17	72937869	72937869	Missense_Mutation	SNV	A	G	G	OTOP3	c.401A>G	P.Y134C
SH_128	17	73835918	73835918	Splice_Site	SNV	A	T	T	UNC13D	c.1055+2T>A	
SH_128	17	74774288	74774288	Missense_Mutation	SNV	G	T	T	MFSD11	c.1204G>T	P.A402S
SH_128	17	7573996	7573996	Missense_Mutation	SNV	A	C	C	TP53	c.1031T>G	P.L344R
SH_128	17	77811696	77811696	Frame_Shift_Ins	INDEL	-	TA	TA	CBX4	c.117_118dupTA	P.N40fs
SH_128	18	14105152	14105152	Nonsense_Mutation	SNV	C	A	A	ZNF519	c.1387G>T	P.E463*
SH_128	18	44284595	44284595	Missense_Mutation	SNV	T	C	C	ST8SIA5	c.272A>G	P.Y91C
SH_128	18	77037163	77037163	Missense_Mutation	SNV	G	A	A	ATP9B	c.1378G>A	P.G460R
SH_128	19	13910666	13910666	Missense_Mutation	SNV	G	T	T	ZSWIM4	c.286G>T	P.A96S
SH_128	19	13919736	13919736	Missense_Mutation	SNV	C	A	A	ZSWIM4	c.799C>A	P.Q267K
SH_128	19	1826866	1826866	Splice_Site	SNV	G	A	A	REXO1	c.1911+11C>T	
SH_128	19	2988081	2988081	Splice_Site	SNV	G	A	A	TLE6	c.703-8G>A	
SH_128	19	42473645	42473645	Missense_Mutation	SNV	C	T	T	ATP1A3	c.2663G>A	P.R888Q
SH_128	19	42911380	42911380	Silent	SNV	G	A	A	LIPE	c.2083C>T	P.L695L
SH_128	19	49100007	49100007	Silent	SNV	C	A	A	SULT2B1	c.612C>A	P.G204G
SH_128	19	50118217	50118217	Missense_Mutation	SNV	G	T	T	PRR12	c.4975G>T	P.V1659L
SH_128	19	54485526	54485526	Missense_Mutation	SNV	C	T	T	CACNG8	c.701C>T	P.A234V
SH_128	19	55592170	55592170	Missense_Mutation	SNV	G	A	A	EPS8L1	c.79G>A	P.A27T
SH_128	19	56206342	56206342	Silent	SNV	G	T	T	EPN1	c.1773G>T	P.L591L
SH_128	19	57327999	57327999	Missense_Mutation	SNV	C	T	T	PEG3	c.1811G>A	P.R604H
SH_128	2	109384034	109384034	Missense_Mutation	SNV	G	A	A	RANBP2	c.7039G>A	P.G2347S
SH_128	2	152496949	152496949	Missense_Mutation	SNV	T	G	G	NEB	c.8605A>C	P.N2869H
SH_128	2	162251685	162251685	Missense_Mutation	SNV	A	G	G	PSMD14	c.742A>G	P.M248V
SH_128	2	1664682	1664682	Missense_Mutation	SNV	G	T	T	PXDN	c.1808C>A	P.A603D
SH_128	2	17699586	17699586	Missense_Mutation	SNV	T	G	G	RAD51AP2	c.97A>C	P.S33R
SH_128	2	183731007	183731007	Missense_Mutation	SNV	A	G	G	FRZB	c.274T>C	P.Y92H
SH_128	2	202558641	202558641	Missense_Mutation	SNV	T	C	C	MPP4	c.37A>G	P.K13E
SH_128	2	207171802	207171802	Missense_Mutation	SNV	A	T	T	ZDBF2	c.2544A>T	P.E848D
SH_128	2	207636665	207636665	Silent	SNV	A	G	G	FASTKD2	c.1038A>G	P.Q346Q
SH_128	2	215890488	215890488	Missense_Mutation	SNV	A	C	C	ABCA12	c.242T>G	P.L81R
SH_128	2	231775073	231775073	Missense_Mutation	SNV	G	A	A	GPR55	c.605C>T	P.S202F
SH_128	2	241715334	241715334	Missense_Mutation	SNV	T	C	C	KIF1A	c.892A>G	P.K298E
SH_128	2	63272303	63272303	Missense_Mutation	SNV	C	T	T	EHBP1	c.3488C>T	P.A1163V
SH_128	20	20020291	20020291	Nonsense_Mutation	SNV	C	A	A	CRNKL1	c.1954G>T	P.E652*
SH_128	3	101578168	101578168	Missense_Mutation	SNV	C	G	G	NFKBIZ	c.1810C>G	P.R604G
SH_128	3	128758582	128758582	Missense_Mutation	SNV	T	A	A	EFCC1	c.1688T>A	P.L563Q
SH_128	3	129252533	129252533	Missense_Mutation	SNV	C	G	G	RHO	c.1019C>G	P.T340R
SH_128	3	150931202	150931202	Silent	SNV	C	T	T	P2RY14	c.903G>A	P.P301P
SH_128	3	151166575	151166575	Silent	SNV	C	A	A	IGSF10	c.1194G>T	P.P398P
SH_128	3	154145382	154145382	Missense_Mutation	SNV	C	G	G	GPR149	c.1097G>C	P.R366P
SH_128	3	154834261	154834261	Splice_Site	SNV	A	G	G	MME	c.359-7A>G	
SH_128	3	170585827	170585827	Missense_Mutation	SNV	C	A	A	RPL22L1	c.199G>T	P.V67F
SH_128	3	175455171	175455171	Silent	SNV	T	A	A	NAALADL2	c.1974T>A	P.V658V
SH_128	3	196449409	196449409	Silent	SNV	A	T	T	PIGX	c.300A>T	P.R100R
SH_128	3	25665866	25665866	Missense_Mutation	SNV	T	A	A	TOP2B	c.2402A>T	P.Q801L
SH_128	3	47165258	47165258	Nonsense_Mutation	SNV	C	A	A	SETD2	c.868G>T	P.E290*
SH_128	3	87294880	87294880	Missense_Mutation	SNV	A	C	C	CHMP2B	c.20A>C	P.K7T
SH_128	3	89259185	89259185	Missense_Mutation	SNV	C	T	T	EPHA3	c.329C>T	P.P110L
SH_128	4	144618046	144618046	Silent	SNV	C	T	T	FREM3	c.3783G>A	P.Q1261Q
SH_128	4	5731114	5731114	Silent	SNV	C	T	T	EVC	c.381C>T	P.F127F
SH_128	4	667183	667183	Silent	SNV	T	C	C	ATP5I	c.99A>G	P.L33L
SH_128	4	68441149	68441149	Missense_Mutation	SNV	A	T	T	STAP1	c.239A>T	P.Q80L
SH_128	4	79400873	79400873	Splice_Site	SNV	G	T	T	FRAS1	c.8443+1G>T	
SH_128	4	89589096	89589096	Silent	SNV	A	G	G	HERC3	c.1143A>G	P.P381P
SH_128	5	132087754	132087754	Missense_Mutation	SNV	C	A	A	CCNI2	c.871C>A	P.Q291K
SH_128	5	13751352	13751352	Missense_Mutation	SNV	C	A	A	DNAH5	c.11046G>T	P.K3682N
SH_128	5	138773156	138773156	Silent	SNV	T	G	G	DNAJC18	c.132A>C	P.A44A
SH_128	5	140230276	140230276	Silent	SNV	C	T	T	PCDHA9	c.2196C>T	P.C732C
SH_128	5	140870391	140870391	Missense_Mutation	SNV	G	C	C	PCDHGC5	c.1584G>C	P.Q528H
SH_128	5	14280489	14280489	Missense_Mutation	SNV	C	G	G	TRIO	c.291C>G	P.D97E
SH_128	5	31267811	31267811	Splice_Site	SNV	A	G	G	CDH6	c.228+3A>G	
SH_128	5	74651259	74651259	Missense_Mutation	SNV	C	T	T	HMGCR	c.1792C>T	P.R598C
SH_128	6	133100511	133100511	Missense_Mutation	SNV	G	C	C	SLC18B1	c.691C>G	P.L231V
SH_128	6	138726283	138726283	Missense_Mutation	SNV	C	G	G	HEBP2	c.137C>G	P.P46R
SH_128	6	17788054	17788054	Missense_Mutation	SNV	C	T	T	KIF13A	c.3314G>A	P.R1105Q
SH_128	6	32798107	32798107	Silent	SNV	C	A	A	TAP2	c.1572G>T	P.G524G
SH_128	6	33400547	33400547	Missense_Mutation	SNV	A	T	T	SYNGAP1	c.473A>T	P.Q158L
SH_128	6	35467937	35467937	Splice_Site	SNV	G	T	T	TULP1	c.1165-8C>A	
SH_128	6	41059275	41059275	Missense_Mutation	SNV	C	A	A	NFYA	c.556C>A	P.P186T
SH_128	6	42713053	42713053	Silent	SNV	C	G	G	TBCC	c.759G>C	P.V253V
SH_128	6	64404493	64404493	Nonsense_Mutation	SNV	C	A	A	PHF3	c.2255C>A	P.S752*
SH_128	6	75912486	75912486	Missense_Mutation	SNV	G	T	T	COL12A1	c.23C>A	P.A8E
SH_128	6	84303452	84303452	Missense_Mutation	SNV	C	A	A	SNAP91	c.1441G>T	P.A481S
SH_128	6	90573128	90573128	Missense_Mutation	SNV	C	T	T	CASP8AP2	c.1700C>T	P.S567F
SH_128	7	106710735	106710735	Missense_Mutation	SNV	T	C	C	PRKAR2B	c.317T>C	P.I106T

SH_128	7	141423844	141423844	Silent	SNV	A	G	G	WEE2	c.1131A>G	P.K377K
SH_128	7	149513059	149513059	Nonsense_Mutation	SNV	G	A	A	SSPO	c.10962G>A	P.W3654*
SH_128	8	48647870	48647870	Missense_Mutation	SNV	G	T	T	SPIDR	c.2606G>T	P.S869I
SH_128	9	101751487	101751487	Missense_Mutation	SNV	G	A	A	COL15A1	c.751G>A	P.E251K
SH_128	9	114804403	114804403	Missense_Mutation	SNV	C	G	G	SUSD1	c.2192G>C	P.R731T
SH_128	9	116224120	116224120	Frame_Shift_Del	INDEL	C	-	-	RGS3	c.216delC	P.W73fs
SH_128	9	123780066	123780066	Missense_Mutation	SNV	T	C	C	C5	c.1589A>G	P.Q530R
SH_128	9	134151534	134151534	Silent	SNV	G	C	C	FAM78A	c.33C>G	P.S11S
SH_128	9	34724376	34724376	Missense_Mutation	SNV	G	A	A	FAM205A	c.2861C>T	P.P954L
SH_128	9	67930495	67930495	Missense_Mutation	SNV	G	C	C	ANKRD20A3	c.312G>C	P.L104F
SH_128	9	99521410	99521410	Nonsense_Mutation	SNV	C	A	A	ZNF510	c.1702G>T	P.G568*
SH_128	X	115588781	115588781	Missense_Mutation	SNV	T	G	G	SLC6A14	c.1621T>G	P.F541V
SH_128	X	129519343	129519343	Missense_Mutation	SNV	C	T	T	GPR119	c.79G>A	P.V27M
SH_128	X	23410817	23410817	Silent	SNV	G	A	A	PTCHD1	c.1182G>A	P.T394T
SH_128	X	24078209	24078209	Missense_Mutation	SNV	G	T	T	EIF2S3	c.388G>T	P.V130F
SH_128	X	47062621	47062621	Splice_Site	INDEL	-	T	T	UBA1	c.1419+9dupT	
SH_129	1	158911819	158911819	Missense_Mutation	SNV	G	A	A	PYHIN1	c.632G>A	P.R211Q
SH_129	1	185931768	185931768	Missense_Mutation	SNV	T	A	A	HMCN1	c.1947T>A	P.D649E
SH_129	1	227834307	227834307	Missense_Mutation	SNV	C	A	A	ZNF678	c.186C>A	P.D62E
SH_129	1	25228934	25228934	Silent	SNV	C	T	T	RUNX3	c.969G>A	P.P323P
SH_129	1	46874779	46874779	Missense_Mutation	SNV	C	T	T	FAAH	c.1084C>T	P.P362S
SH_129	1	53543382	53543382	Frame_Shift_Ins	INDEL	-	G	G	PODN	c.856dupG	P.A286fs
SH_129	10	101961894	101961894	Frame_Shift_Ins	INDEL	-	T	T	CHUK	c.1519dupA	P.M507fs
SH_129	11	116798030	116798030	Missense_Mutation	SNV	G	A	A	SIK3	c.44C>T	P.T15I
SH_129	11	120008562	120008562	Silent	SNV	G	A	A	TRIM29	c.178C>T	P.L60L
SH_129	11	59225149	59225149	Missense_Mutation	SNV	C	T	T	OR4D6	c.716C>T	P.T239M
SH_129	11	62380940	62380940	Missense_Mutation	SNV	C	T	T	ROM1	c.187C>T	P.P63S
SH_129	11	62931321	62931321	Missense_Mutation	SNV	G	A	A	SLC22A25	c.1619C>T	P.A540V
SH_129	11	92523203	92523203	Missense_Mutation	SNV	C	A	A	FAT3	c.4430C>A	P.T1477K
SH_129	12	114841613	114841613	Missense_Mutation	SNV	C	T	T	TBX5	c.91G>A	P.A31T
SH_129	12	123276583	123276583	Missense_Mutation	SNV	G	T	T	CCDC62	c.687G>T	P.K229N
SH_129	12	15130986	15130986	Nonsense_Mutation	SNV	C	T	T	PDE6H	c.40C>T	P.Q14*
SH_129	12	25398284	25398284	Missense_Mutation	SNV	C	T	T	KRAS	c.35G>A	P.G12D
SH_129	12	54394208	54394208	Missense_Mutation	SNV	C	T	T	HOXC9	c.236C>T	P.P79L
SH_129	12	70975011	70975011	Nonsense_Mutation	SNV	G	A	A	PTPRB	c.2383C>T	P.Q795*
SH_129	13	50285084	50285084	Silent	SNV	A	G	G	KPNA3	c.846T>C	P.D282D
SH_129	14	105413795	105413795	Missense_Mutation	SNV	C	T	T	AHNAK2	c.7993G>A	P.E2665K
SH_129	14	23549113	23549113	Missense_Mutation	SNV	T	A	A	ACIN1	c.1605A>T	P.E535D
SH_129	15	64422138	64422138	Missense_Mutation	SNV	G	T	T	SNX1	c.831G>T	P.Q277H
SH_129	15	79227355	79227355	Missense_Mutation	SNV	T	C	C	CTSH	c.370A>G	P.K124E
SH_129	16	24135257	24135257	Silent	SNV	C	T	T	PRKCB	c.1020C>T	P.T340T
SH_129	16	24581635	24581635	Missense_Mutation	SNV	A	C	C	RBBP6	c.3624A>C	P.K1208N
SH_129	17	15532029	15532029	Missense_Mutation	SNV	G	A	A	TRIM16	c.1595C>T	P.A532V
SH_129	17	34171885	34171885	Frame_Shift_Ins	INDEL	-	G	G	TAF15	c.1580dupG	P.Y528fs
SH_129	17	39643395	39643395	Missense_Mutation	SNV	C	T	T	KRT36	c.1015G>A	P.E339K
SH_129	17	66039357	66039357	Missense_Mutation	SNV	G	T	T	KPNA2	c.808G>T	P.D270Y
SH_129	17	7578406	7578406	Missense_Mutation	SNV	C	T	T	TP53	c.524G>A	P.R175H
SH_129	19	11833852	11833852	Missense_Mutation	SNV	C	A	A	ZNF823	c.497G>T	P.C166F
SH_129	19	40411763	40411763	Frame_Shift_Ins	INDEL	-	T	T	FCGBP	c.3864_3865ins	P.H1289fs
SH_129	19	41289949	41289949	Missense_Mutation	SNV	C	G	G	RAB4B	c.399C>G	P.F133L
SH_129	19	50187307	50187307	Missense_Mutation	SNV	A	G	G	PRMT1	c.482A>G	P.Y161C
SH_129	19	54140051	54140051	Missense_Mutation	SNV	T	A	A	DPRX	c.385T>A	P.F129I
SH_129	19	7119591	7119591	Silent	SNV	G	A	A	INSR	c.3663C>T	P.S1221S
SH_129	2	152135364	152135364	Silent	SNV	A	G	G	NMI	c.318T>C	P.L106L
SH_129	2	179534998	179534998	Missense_Mutation	SNV	A	C	C	TTN	c.34211T>G	P.V11404G
SH_129	2	206036979	206036979	Silent	SNV	G	A	A	PARD3B	c.1665G>A	P.G555G
SH_129	2	233899020	233899020	Silent	SNV	C	T	T	NEU2	c.396C>T	P.H132H
SH_129	2	39222277	39222277	Silent	SNV	G	A	A	SOS1	c.3333C>T	P.S1111S
SH_129	2	73679160	73679160	Missense_Mutation	SNV	C	A	A	ALMS1	c.5503C>A	P.P1835T
SH_129	20	3686472	3686472	Missense_Mutation	SNV	G	T	T	SIGLEC1	c.625C>A	P.Q209K
SH_129	20	36868058	36868058	Missense_Mutation	SNV	G	T	T	KIAA1755	c.1619C>A	P.A540D
SH_129	3	121345679	121345679	Missense_Mutation	SNV	G	A	A	FBXO40	c.2052G>A	P.M684I
SH_129	3	158254895	158254895	Missense_Mutation	SNV	G	A	A	RSRC1	c.493G>A	P.V165I
SH_129	3	97194294	97194294	Missense_Mutation	SNV	A	T	T	EPHA6	c.169A>T	P.I57F
SH_129	4	154626161	154626161	Missense_Mutation	SNV	T	G	G	TLR2	c.2102T>G	P.F701C
SH_129	4	169315746	169315746	Missense_Mutation	SNV	C	T	T	DDX60L	c.3680G>A	P.R1227Q
SH_129	4	68449286	68449286	Splice_Site	SNV	C	T	T	STAP1	c.531-6C>T	
SH_129	4	94006296	94006296	Missense_Mutation	SNV	G	A	A	GRID2	c.395G>A	P.G132E
SH_129	5	132534816	132534816	Missense_Mutation	SNV	T	C	C	FSTL4	c.2500A>G	P.T834A
SH_129	5	140475961	140475961	Silent	SNV	C	T	T	PCDHB2	c.1587C>T	P.F529F
SH_129	5	41058274	41058274	Missense_Mutation	SNV	G	T	T	MROH2B	c.647C>A	P.T216K
SH_129	6	111983103	111983116	Frame_Shift_Del	INDEL	CTGTAGCCTC	-	-	FYN	53delGCGAGGC	P.E480fs
SH_129	6	33284572	33284572	Missense_Mutation	SNV	C	T	T	ZBTB22	c.122G>A	P.S41N
SH_129	7	76126759	76126759	Missense_Mutation	SNV	G	A	A	DTX2	c.1115G>A	P.G372D
SH_129	7	77797412	77797412	Missense_Mutation	SNV	G	A	A	MAGI2	c.2417C>T	P.A806V
SH_129	8	24207486	24207486	Splice_Site	SNV	G	A	A	ADAM28	c.2099+1G>A	
SH_129	8	91013798	91013798	Splice_Site	SNV	G	T	T	DECR1	c.69+9G>T	
SH_129	9	118949636	118949636	Missense_Mutation	SNV	T	C	C	PAPPA	c.619T>C	P.F207L
SH_129	9	123152034	123152034	Silent	SNV	G	A	A	CDK5RAP2	c.5373C>T	P.A1791A
SH_129	9	130504196	130504196	Silent	SNV	G	T	T	SH2D3C	c.897C>A	P.G299G
SH_129	9	34658538	34658538	Missense_Mutation	SNV	G	T	T	IL11RA	c.668G>T	P.G223V
SH_129	X	118771070	118771070	Silent	SNV	G	A	A	SEPT6	c.876C>T	P.T292T
SH_129	X	129173244	129173244	Silent	SNV	G	A	A	BCORL1	c.4605G>A	P.K1535K
SH_129	X	132161989	132161989	Missense_Mutation	SNV	G	A	A	USP26	c.260C>T	P.S87F
SH_129	X	41333619	41333619	Missense_Mutation	SNV	C	T	T	NYX	c.913C>T	P.L305F
SH_129	X	70823875	70823875	Missense_Mutation	SNV	G	A	A	ACRC	c.748G>A	P.D250N
SH_13	1	103352460	103352460	Silent	SNV	T	C	C	COL11A1	c.4644A>G	P.K1548K
SH_13	1	108724567	108724567	Missense_Mutation	SNV	G	T	T	SLC25A24	c.394C>A	P.Q132K
SH_13	1	114680475	114680475	Missense_Mutation	SNV	C	A	A	SYT6	c.458G>T	P.R153L
SH_13	1	117146380	117146380	Missense_Mutation	SNV	C	A	A	IGSF3	c.1490G>T	P.G497V
SH_13	1	152193463	152193463	Missense_Mutation	SNV	C	A	A	HRNR	c.642G>T	P.Q214H
SH_13	1	155874285	155874285	Missense_Mutation	SNV	A	T	T	RIT1	c.246T>A	P.F82L
SH_13	1	159802968	159802968	Missense_Mutation	SNV	G	A	A	SLAMF8	c.670G>A	P.A224T
SH_13	1	228407193	228407193	Missense_Mutation	SNV	T	A	A	OBSCN	c.2822T>A	P.V941E
SH_13	1	34070903	34070903	Missense_Mutation	SNV	G	T	T	CSMD2	c.6511C>A	P.H2171N
SH_13	1	97915674	97915674	Nonsense_Mutation	SNV	T	A	A	DPYD	c.1846A>T	P.K616*
SH_13	10	27405029	27405075	Frame_Shift_Del	INDEL	AATCACTGG	-	-	YME1L1	AGGTGCTTCCAC	P.G583fs
SH_13	10	49917882	49917882	Silent	SNV	G	T	T	WDFY4	c.105G>T	P.G35G

SH_13	10	50533075	50533075	Nonsense_Mutation	SNV	G	T	T	C10orf71	c.2485G>T	P.E829*
SH_13	10	89653867	89653867	Splice_Site	SNV	G	A	A	PTEN	c.164+1G>A	
SH_13	11	111228162	111228162	Splice_Site	SNV	T	A	A	POU2AF1	c.456+8A>T	
SH_13	11	113221975	113221975	Missense_Mutation	SNV	T	G	G	TTC12	c.1280T>G	P.F427C
SH_13	11	114270739	114270739	Silent	SNV	C	A	A	C11orf71	c.315G>T	P.V105V
SH_13	11	4976182	4976182	Silent	SNV	C	A	A	OR51A2	c.762G>T	P.L254L
SH_13	11	5020384	5020384	Missense_Mutation	SNV	C	T	T	OR51L1	c.172C>T	P.H58Y
SH_13	11	59564765	59564765	Missense_Mutation	SNV	A	G	G	STX3	c.796A>G	P.I266V
SH_13	12	112614475	112614475	Missense_Mutation	SNV	C	T	T	HECTD4	c.10993G>A	P.V3665M
SH_13	12	6960098	6960098	Missense_Mutation	SNV	C	T	T	CDCA3	c.19G>A	P.V7I
SH_13	12	80732888	80732888	Missense_Mutation	SNV	T	G	G	OTOGL	c.4867T>G	P.F1623V
SH_13	14	103969471	103969471	Silent	SNV	G	T	T	MARK3	c.2169G>T	P.L723L
SH_13	14	105420665	105420665	Missense_Mutation	SNV	T	A	A	AHNAK2	c.1123A>T	P.S375C
SH_13	14	23299285	23299285	Missense_Mutation	SNV	G	T	T	MRPL52	c.52G>T	P.V18L
SH_13	14	23826721	23826721	Missense_Mutation	SNV	G	A	A	EFS	c.893C>T	P.P298L
SH_13	14	23946563	23946563	Nonsense_Mutation	SNV	G	T	T	NGDN	c.868G>T	P.E290*
SH_13	16	68057083	68057083	Missense_Mutation	SNV	C	A	A	DDX28	c.23G>T	P.R8L
SH_13	16	68248230	68248230	Splice_Site	SNV	A	C	C	NFATC3	c.3107-2A>C	
SH_13	16	70925750	70925750	Missense_Mutation	SNV	A	G	G	HYDIN	c.9455T>C	P.I3152T
SH_13	16	70969907	70969907	Missense_Mutation	SNV	C	A	A	HYDIN	c.7106G>T	P.G2369V
SH_13	16	85834894	85834894	Splice_Site	SNV	A	T	T	COX4I1	c.73+10A>T	
SH_13	17	3301642	3301642	Silent	SNV	T	A	A	OR1E1	c.63A>T	P.P21P
SH_13	17	34074884	34074884	Nonsense_Mutation	SNV	G	T	T	GAS2L2	c.816C>A	P.C272*
SH_13	17	40966589	40966589	Silent	SNV	C	A	A	BECN1	c.933G>T	P.V311V
SH_13	17	45777066	45777066	Splice_Site	SNV	A	T	T	TBKBP1	c.872+3A>T	
SH_13	17	74625520	74625520	Silent	SNV	G	A	A	ST6GALNAC1	c.9C>T	P.N3N
SH_13	17	7577534	7577534	Missense_Mutation	SNV	C	A	A	TP53	c.747G>T	P.R249S
SH_13	17	76134461	76134461	Missense_Mutation	SNV	C	G	G	TMC8	c.1565C>G	P.S522W
SH_13	18	22806113	22806113	Missense_Mutation	SNV	A	G	G	ZNF521	c.1109T>C	P.L370S
SH_13	18	29171935	29171935	Splice_Site	SNV	G	T	T	TTR	c.69+1G>T	
SH_13	18	33744438	33744438	Missense_Mutation	SNV	G	A	A	ELP2	c.1957G>A	P.A653T
SH_13	18	42532203	42532203	Silent	SNV	G	T	T	SETBP1	c.2898G>T	P.V966V
SH_13	18	52896252	52896252	Silent	SNV	G	T	T	TCF4	c.1705C>A	P.R569R
SH_13	19	40411969	40411969	Missense_Mutation	SNV	C	A	A	FCGBP	c.3659G>T	P.G1220V
SH_13	19	42482130	42482130	Missense_Mutation	SNV	A	T	T	ATP1A3	c.1934T>A	P.I645N
SH_13	19	55317435	55317435	Silent	SNV	C	A	A	KIR2DL4	c.391C>A	P.R131R
SH_13	19	56223303	56223303	Missense_Mutation	SNV	G	C	C	NLRP9	c.2706C>G	P.C902W
SH_13	19	57647140	57647140	Missense_Mutation	SNV	G	A	A	ZIM3	c.565C>T	P.H189Y
SH_13	19	577939	577939	Missense_Mutation	SNV	G	T	T	BSG	c.233G>T	P.R78L
SH_13	2	179443615	179443615	Silent	SNV	A	G	G	TTN	c.63219T>C	P.Y21073Y
SH_13	2	189925466	189925466	Frame_Shift_Del	INDEL	G	-	-	COL5A2	c.2075delC	P.P692fs
SH_13	2	71360152	71360152	Missense_Mutation	SNV	G	A	A	MPHOSPH10	c.214G>A	P.D72N
SH_13	2	72725596	72725596	Missense_Mutation	SNV	C	A	A	EXOC6B	c.1324G>T	P.A442S
SH_13	2	80529586	80529586	Missense_Mutation	SNV	G	T	T	LRRTM1	c.1359C>A	P.F453L
SH_13	20	3211715	3211715	Splice_Site	SNV	G	C	C	SLC4A11	c.1043-11C>G	
SH_13	20	48744532	48744532	Missense_Mutation	SNV	G	T	T	TMEM189	c.662C>A	P.T221N
SH_13	20	61542712	61542712	Missense_Mutation	SNV	C	A	A	DIDO1	c.253G>T	P.G85C
SH_13	20	61982181	61982181	Missense_Mutation	SNV	C	A	A	CHRNA4	c.582G>T	P.M194I
SH_13	20	62124632	62124632	Nonsense_Mutation	SNV	C	T	T	EEF1A2	c.630G>A	P.W210*
SH_13	21	35468082	35468082	Silent	SNV	G	T	T	SLC5A3	c.585G>T	P.G195G
SH_13	21	45502786	45502786	Missense_Mutation	SNV	G	A	A	TRAPPC10	c.1841G>A	P.S614N
SH_13	22	20907437	20907437	Missense_Mutation	SNV	A	G	G	MED15	c.214A>G	P.K72E
SH_13	22	37471174	37471174	Splice_Site	SNV	C	T	T	TMPRSS6	c.1342+1G>A	
SH_13	22	40054956	40054956	Missense_Mutation	SNV	A	G	G	CACNA1I	c.2060A>G	P.Q687R
SH_13	3	111793176	111793176	Missense_Mutation	SNV	G	T	T	TMPRSS7	c.1322G>T	P.G441V
SH_13	3	11300845	11300845	Missense_Mutation	SNV	C	A	A	HRH1	c.122C>A	P.T41K
SH_13	3	124732832	124732832	Missense_Mutation	SNV	C	A	A	HEG1	c.1591G>T	P.A531S
SH_13	3	193082072	193082072	Splice_Site	SNV	G	C	C	ATP13A5	c.64-3C>G	
SH_13	3	38797459	38797459	Splice_Site	SNV	G	T	T	SCN10A	c.1291-10C>A	
SH_13	3	50412208	50412208	Nonsense_Mutation	SNV	G	T	T	CACNA2D2	c.1944C>A	P.Y648*
SH_13	3	57817197	57817197	Nonsense_Mutation	SNV	G	T	T	SLMAP	c.286G>T	P.E96*
SH_13	4	135121249	135121249	Missense_Mutation	SNV	A	C	C	PABPC4L	c.1100T>G	P.L367R
SH_13	5	131398462	131398462	Missense_Mutation	SNV	C	A	A	IL3	c.437C>A	P.T146N
SH_13	5	31995683	31995683	Missense_Mutation	SNV	A	T	T	PDZD2	c.980A>T	P.E327V
SH_13	5	35697790	35697790	Splice_Site	SNV	A	G	G	SPEF2	c.2038-2A>G	
SH_13	5	70751716	70751716	Missense_Mutation	SNV	G	T	T	BDP1	c.12G>T	P.R4S
SH_13	6	102266269	102266269	Missense_Mutation	SNV	G	T	T	GRIK2	c.1228G>T	P.G410C
SH_13	6	132892350	132892350	Missense_Mutation	SNV	G	A	A	TAAR6	c.890G>A	P.C297Y
SH_13	6	28473524	28473524	Missense_Mutation	SNV	C	A	A	GPX6	c.415G>T	P.D139Y
SH_13	6	30698749	30698749	Silent	SNV	C	A	A	FLOT1	c.708G>T	P.R236R
SH_13	6	42612073	42612073	Missense_Mutation	SNV	A	C	C	UBR2	c.2219A>C	P.K740T
SH_13	6	46047610	46047610	Missense_Mutation	SNV	G	A	A	CLIC5	c.370C>T	P.L124F
SH_13	6	65301109	65301109	Nonsense_Mutation	SNV	C	A	A	EYS	c.4651G>T	P.E1551*
SH_13	7	107696124	107696124	Missense_Mutation	SNV	G	T	T	LAMB4	c.3708C>A	P.F1236L
SH_13	7	123092932	123092932	Silent	SNV	C	G	G	IQUB	c.2241G>C	P.L747L
SH_13	7	141618961	141618961	Missense_Mutation	SNV	G	T	T	OR9A4	c.286G>T	P.A96S
SH_13	7	31912917	31912917	Silent	SNV	G	T	T	PDE1C	c.597C>A	P.I199I
SH_13	7	81579718	81579718	Missense_Mutation	SNV	C	T	T	CACNA2D1	c.3266G>A	P.R1089H
SH_13	7	99032823	99032823	Missense_Mutation	SNV	G	T	T	ATP5J2-PTCD1	c.190C>A	P.P64T
SH_13	8	2976107	2976107	Missense_Mutation	SNV	A	T	T	CSMD1	c.6244T>A	P.Y2082N
SH_13	8	75156754	75156754	Splice_Site	SNV	C	A	A	JPH1	c.1905+10G>T	
SH_13	8	95508665	95508665	Missense_Mutation	SNV	G	A	A	KIAA1429	c.4274C>T	P.A1425V
SH_13	9	101748165	101748165	Missense_Mutation	SNV	A	G	G	COL15A1	c.419A>G	P.E140G
SH_13	9	139368612	139368612	Silent	SNV	C	A	A	SEC16A	c.3456G>T	P.P1152P
SH_13	9	5919945	5919945	Silent	SNV	T	C	C	KIAA2026	c.6051A>G	P.T2017T
SH_13	9	77436710	77436710	Silent	SNV	G	A	A	TRPM6	c.870C>T	P.G290G
SH_13	9	96030136	96030136	Missense_Mutation	SNV	C	G	G	WNK2	c.3805C>G	P.R1269G
SH_13	X	114418979	114418979	Nonsense_Mutation	SNV	C	A	A	LRCH2	c.616G>T	P.E206*
SH_13	X	49066496	49066496	Missense_Mutation	SNV	A	G	G	CACNA1F	c.4595T>C	P.L1532P
SH_130	1	1421986	1421986	Missense_Mutation	SNV	G	T	T	ATAD3B	c.1014G>T	P.M338I
SH_130	1	1421987	1421987	Missense_Mutation	SNV	G	T	T	ATAD3B	c.1015G>T	P.G339W
SH_130	1	15428051	15428051	Silent	SNV	T	A	A	KAZN	c.1560T>A	P.A520A
SH_130	1	155927566	155927566	Silent	SNV	C	T	T	ARHGEF2	c.1653G>A	P.V551V
SH_130	1	161160769	161160769	Missense_Mutation	SNV	C	A	A	ADAMTS4	c.2512G>T	P.A838S
SH_130	1	180044231	180044231	Missense_Mutation	SNV	G	T	T	CEP350	c.5642G>T	P.R1881L
SH_130	1	198682206	198682206	Splice_Site	SNV	T	A	A	PTPRC	c.808+5T>A	
SH_130	1	21191018	21191018	Missense_Mutation	SNV	C	A	A	EIF4G3	c.2897G>T	P.C966F
SH_130	1	237824130	237824130	Missense_Mutation	SNV	G	T	T	RYR2	c.8319G>T	P.W2773C

SH_130	1	247978864	247978864	Silent	SNV	G	A	A	OR14A16	c.168C>T	P.P56P
SH_130	1	248224164	248224164	Missense_Mutation	SNV	C	A	A	OR2L3	c.181C>A	P.L61I
SH_130	1	248224885	248224885	Missense_Mutation	SNV	C	A	A	OR2L3	c.902C>A	P.T301K
SH_130	1	45473170	45473170	Missense_Mutation	SNV	C	A	A	HECTD3	c.1418G>T	P.R473L
SH_130	10	101590191	101590191	Splice_Site	SNV	G	T	T	ABCC2	c.2747+1G>T	
SH_130	10	103789389	103789389	Splice_Site	SNV	C	A	A	C10orf76	c.414+6G>T	
SH_130	10	115357747	115357747	Missense_Mutation	SNV	G	A	A	NRAP	c.4313C>T	P.A1438V
SH_130	10	125805589	125805589	Missense_Mutation	SNV	A	T	T	CHST15	c.140T>A	P.V47E
SH_130	10	135126297	135126297	Missense_Mutation	SNV	C	A	A	ZNF511	c.686C>A	P.P229H
SH_130	10	18266928	18266928	Silent	SNV	C	G	G	SLC39A12	c.849C>G	P.T283T
SH_130	10	226061	226061	Missense_Mutation	SNV	A	G	G	ZMYND11	c.109A>G	P.I37V
SH_130	10	49635192	49635192	Splice_Site	SNV	T	G	G	MAPK8	c.1060+6T>G	
SH_130	10	50666905	50666905	Missense_Mutation	SNV	A	G	G	ERCC6	c.4438T>C	P.S1480P
SH_130	10	52588005	52588005	Nonsense_Mutation	SNV	C	A	A	A1CF	c.655G>T	P.E219*
SH_130	10	52834576	52834576	Silent	SNV	C	A	A	PRKG1	c.226C>A	P.R76R
SH_130	10	70764800	70764800	Splice_Site	SNV	A	G	G	KIAA1279	c.526-2A>G	
SH_130	11	102736636	102736636	Missense_Mutation	SNV	C	G	G	MMP12	c.1073G>C	P.R358T
SH_130	11	123479437	123479437	Silent	SNV	G	T	T	GRAMD1B	c.1176G>T	P.V392V
SH_130	11	123909020	123909020	Missense_Mutation	SNV	G	A	A	OR10G7	c.689C>T	P.S230L
SH_130	11	5776918	5776918	Silent	SNV	C	A	A	OR52N4	c.948C>A	P.T316T
SH_130	11	64323775	64323775	Missense_Mutation	SNV	G	A	A	SLC22A11	c.304G>A	P.A102T
SH_130	11	65618597	65618597	Silent	SNV	C	T	T	SNX32	c.675C>T	P.C225C
SH_130	12	104461804	104461804	Missense_Mutation	SNV	G	T	T	HCFC2	c.392G>T	P.S131I
SH_130	12	3149329	3149329	Silent	SNV	G	T	T	TEAD4	c.1095G>T	P.P365P
SH_130	12	4628057	4628057	Splice_Site	SNV	T	C	C	C12orf4	c.729-11A>G	
SH_130	12	57828687	57828687	Silent	SNV	T	A	A	INHBC	c.18T>A	P.L6L
SH_130	12	7069873	7069873	Missense_Mutation	SNV	G	C	C	PTPN6	c.1721G>C	P.R574P
SH_130	12	88568497	88568497	Missense_Mutation	SNV	C	T	T	TMTC3	c.1313C>T	P.A438V
SH_130	13	114061329	114061329	Missense_Mutation	SNV	C	A	A	LOC101928841	c.1176G>T	P.Q392H
SH_130	13	48947539	48947539	Splice_Site	SNV	A	G	G	RB1	c.1128-2A>G	
SH_130	13	61103226	61103226	Missense_Mutation	SNV	G	T	T	TDRD3	c.1867G>T	P.G623W
SH_130	13	61103227	61103227	Missense_Mutation	SNV	G	T	T	TDRD3	c.1868G>T	P.G623V
SH_130	14	20181623	20181623	Missense_Mutation	SNV	C	G	G	OR11H2	c.453G>C	P.M151I
SH_130	14	24040569	24040569	Silent	SNV	G	T	T	JPH4	c.1371C>A	P.S457S
SH_130	14	53619451	53619451	Missense_Mutation	SNV	C	A	A	DDHD1	c.366G>T	P.Q122H
SH_130	15	40708323	40708323	Missense_Mutation	SNV	G	T	T	IVD	c.926G>T	P.C309F
SH_130	15	50763999	50763999	Splice_Site	SNV	A	G	G	USP8	c.849+7A>G	
SH_130	15	57837794	57837794	Missense_Mutation	SNV	C	G	G	CGNL1	c.3505C>G	P.R1169G
SH_130	15	72454657	72454657	Missense_Mutation	SNV	G	C	C	GRAMD2	c.1018C>G	P.Q340E
SH_130	16	11369763	11369763	Missense_Mutation	SNV	C	A	A	PRM2	c.347G>T	P.G116V
SH_130	16	3781828	3781828	Silent	SNV	C	A	A	CREBBP	c.4839G>T	P.V1613V
SH_130	16	50816304	50816304	Nonsense_Mutation	SNV	G	T	T	CYLD	c.1753G>T	P.E585*
SH_130	16	53501036	53501036	Nonsense_Mutation	SNV	A	T	T	RBL2	c.1930A>T	P.R644*
SH_130	16	89789814	89789814	Missense_Mutation	SNV	G	T	T	ZNF276	c.703G>T	P.D235Y
SH_130	2	220246123	220246123	Silent	SNV	A	G	G	DNPEP	c.1197T>C	P.Y399Y
SH_130	2	49195955	49195955	Missense_Mutation	SNV	C	A	A	FSHR	c.736G>T	P.A246S
SH_130	2	64863208	64863208	Silent	SNV	C	T	T	SERTAD2	c.798G>A	P.G266G
SH_130	2	9000856	9000856	Silent	SNV	G	T	T	MBOAT2	c.957C>A	P.S319S
SH_130	3	133495987	133495987	Missense_Mutation	SNV	G	T	T	TF	c.1967G>T	P.C656F
SH_130	3	133547664	133547664	Missense_Mutation	SNV	G	T	T	RAB6B	c.595C>A	P.P199T
SH_130	3	137717926	137717926	Silent	SNV	G	A	A	CLDN18	c.216G>A	P.L72L
SH_130	3	168833418	168833418	Silent	SNV	G	T	T	MECOM	c.1873C>A	P.R625R
SH_130	3	186331011	186331011	Silent	SNV	T	C	C	AHSG	c.81T>C	P.Y27Y
SH_130	3	49936290	49936290	Splice_Site	SNV	C	A	A	MST1R	c.1548+10G>T	
SH_130	4	13371542	13371542	Silent	SNV	T	C	C	RAB28	c.621A>G	P.Q207Q
SH_130	4	183836170	183836170	Silent	SNV	C	A	A	DCTD	c.183G>T	P.G61G
SH_130	4	30724764	30724764	Silent	SNV	C	T	T	PCDH7	c.1720C>T	P.L574L
SH_130	4	44638032	44638032	Missense_Mutation	SNV	C	T	T	YIPF7	c.259G>A	P.A87T
SH_130	4	87968323	87968323	Silent	SNV	T	A	A	AFF1	c.636T>A	P.P212P
SH_130	4	92520063	92520063	Missense_Mutation	SNV	C	G	G	CCSER1	c.2558C>G	P.A853G
SH_130	5	138758421	138758421	Missense_Mutation	SNV	G	T	T	DNAJC18	c.755C>A	P.P252H
SH_130	5	140263706	140263706	Missense_Mutation	SNV	C	T	T	PCDHA13	c.1853C>T	P.A618V
SH_130	5	140554686	140554686	Missense_Mutation	SNV	C	A	A	PCDHB7	c.2270C>A	P.S757Y
SH_130	5	39135107	39135107	Missense_Mutation	SNV	G	T	T	FYB	c.1555C>A	P.P519T
SH_130	5	39382768	39382768	Missense_Mutation	SNV	G	C	C	DAB2	c.1230C>G	P.I410M
SH_130	6	146673473	146673473	Missense_Mutation	SNV	A	G	G	GRM1	c.1274A>G	P.Q425R
SH_130	6	150211941	150211941	Splice_Site	SNV	C	T	T	RAET1E	c.85+12G>A	
SH_130	6	160968906	160968906	Missense_Mutation	SNV	G	T	T	LPA	c.5219C>A	P.P1740Q
SH_130	6	31504460	31504460	Missense_Mutation	SNV	C	A	A	DDX39B	c.433G>T	P.V145F
SH_130	6	42713784	42713784	Missense_Mutation	SNV	C	T	T	TBCC	c.28G>A	P.A10T
SH_130	6	74072590	74072590	Silent	SNV	C	A	A	KHDC3L	c.138C>A	P.R46R
SH_130	6	78172048	78172048	Missense_Mutation	SNV	C	A	A	HTR1B	c.1073G>T	P.G358V
SH_130	6	83075166	83075166	Nonsense_Mutation	SNV	C	A	A	TPBG	c.488C>A	P.S163*
SH_130	6	84765103	84765103	Silent	SNV	C	T	T	MRAP2	c.66C>T	P.T22T
SH_130	7	100635286	100635286	Missense_Mutation	SNV	C	A	A	MUC12	c.1442C>A	P.P481H
SH_130	7	131883300	131883300	Silent	SNV	G	T	T	PLXNA4	c.2682C>A	P.V894V
SH_130	7	150763726	150763726	Missense_Mutation	SNV	A	T	T	SLC4A2	c.701A>T	P.N234I
SH_130	7	156799271	156799271	Nonsense_Mutation	SNV	G	A	A	MNX1	c.118C>T	P.Q40*
SH_130	7	38502670	38502670	Missense_Mutation	SNV	C	T	T	AMPH	c.793G>A	P.E265K
SH_130	7	5347779	5347779	Silent	SNV	C	A	A	TNRC18	c.8865G>T	P.T2955T
SH_130	7	55229263	55229263	Missense_Mutation	SNV	G	T	T	EGFR	c.1570G>T	P.V524F
SH_130	7	99260471	99260471	Missense_Mutation	SNV	G	T	T	CYP3A5	c.833C>A	P.S278Y
SH_130	8	104427611	104427611	Missense_Mutation	SNV	G	T	T	DCAF13	c.393G>T	P.R131S
SH_130	8	105361442	105361442	Nonsense_Mutation	SNV	C	A	A	DCSTAMP	c.662C>A	P.S221*
SH_130	8	24346664	24346664	Splice_Site	SNV	A	G	G	ADAM7	c.1092-8A>G	
SH_130	8	28573681	28573681	Silent	SNV	G	C	C	EXTL3	c.105G>C	P.T35T
SH_130	8	665995	665995	Missense_Mutation	SNV	T	G	G	ERICH1	c.35A>C	P.K12T
SH_130	8	72756027	72756027	Missense_Mutation	SNV	C	A	A	MSC	c.387G>T	P.R129S
SH_130	9	136654384	136654384	Missense_Mutation	SNV	C	T	T	VAV2	c.1270G>A	P.D424N
SH_130	9	140948389	140948389	Missense_Mutation	SNV	C	A	A	CACNA1B	c.3899C>A	P.A1300E
SH_130	9	26842467	26842467	Silent	SNV	T	C	C	CAAP1	c.483A>G	P.L161L
SH_130	9	74344996	74344996	Silent	SNV	T	A	A	TMEM2	c.1758A>T	P.G586G
SH_130	9	90535372	90535372	Missense_Mutation	SNV	G	T	T	SPATA31C1	c.550G>T	P.G184C
SH_131	1	12460335	12460335	Missense_Mutation	SNV	A	T	T	VPS13D	c.11732A>T	P.Q3911L
SH_131	1	148017622	148017622	Missense_Mutation	SNV	A	T	T	NBPF8	c.1681T>A	P.W561R
SH_131	1	155207274	155207274	Missense_Mutation	SNV	T	A	A	GBA	c.857A>T	P.Q286L
SH_131	1	156351299	156351299	Missense_Mutation	SNV	G	C	C	RHBG	c.628G>C	P.D210H
SH_131	1	156554689	156554689	Silent	SNV	C	G	G	TTC24	c.1272C>G	P.L424L

SH_131	1	158590176	158590176	Silent	SNV	T	A	A	SPTA1	c.6201A>T	P.S2067S
SH_131	1	159846499	159846499	Frame_Shift_Ins	INDEL	-	T	T	CFAP45	c.1198dupA	P.R400fs
SH_131	1	160604625	160604625	Missense_Mutation	SNV	A	T	T	SLAMF1	c.478T>A	P.L160M
SH_131	1	169673698	169673698	Splice_Site	SNV	A	T	T	SELL	c.805+11T>A	
SH_131	1	179623487	179623487	Missense_Mutation	SNV	A	T	T	TDRD5	c.2312A>T	P.E771V
SH_131	1	196392233	196392233	Missense_Mutation	SNV	C	A	A	KCNT2	c.1132G>T	P.A378S
SH_131	1	203830821	203830821	Nonsense_Mutation	SNV	A	T	T	SNRPE	c.34A>T	P.K12*
SH_131	1	215820908	215820908	Missense_Mutation	SNV	C	T	T	USH2A	c.14747G>A	P.G4916D
SH_131	1	215901593	215901593	Missense_Mutation	SNV	C	T	T	USH2A	c.11845G>A	P.G3949S
SH_131	1	229567738	229567738	Splice_Site	SNV	C	A	A	ACTA1	c.808+3G>T	
SH_131	1	243716053	243716053	Missense_Mutation	SNV	A	T	T	AKT3	c.1141T>A	P.L381M
SH_131	1	247654472	247654472	Missense_Mutation	SNV	G	T	T	OR2W5	c.43G>T	P.V15L
SH_131	1	248524891	248524891	Silent	SNV	C	T	T	OR2T4	c.9C>T	P.N3N
SH_131	1	26515327	26515327	Silent	SNV	A	T	T	CNKSR1	c.1776A>T	P.L592L
SH_131	1	27621003	27621003	Splice_Site	SNV	A	T	T	WDTC1	c.758-2A>T	
SH_131	1	38488401	38488401	Missense_Mutation	SNV	A	T	T	UTP11	c.598A>T	P.N200Y
SH_131	1	67356867	67356867	Missense_Mutation	SNV	A	T	T	WDR78	c.613T>A	P.Y205N
SH_131	10	108466295	108466295	Splice_Site	INDEL	G	-	-	SORCS1	c.1233+8delC	
SH_131	10	1245940	1245940	Silent	SNV	G	A	A	ADARB2	c.1830C>T	P.P610P
SH_131	10	134722047	134722047	Missense_Mutation	SNV	C	A	A	CFAP46	c.2849G>T	P.R950L
SH_131	10	16877003	16877003	Splice_Site	SNV	T	C	C	CUBN	c.10362+10A>G	
SH_131	10	27826809	27826809	Missense_Mutation	SNV	A	T	T	RAB18	c.383A>T	P.Q128L
SH_131	10	51130638	51130638	Silent	SNV	A	T	T	PARG	c.1287T>A	P.V429V
SH_131	11	102487587	102487587	Silent	SNV	G	T	T	MMP20	c.330C>A	P.L110L
SH_131	11	102667820	102667820	Missense_Mutation	SNV	T	A	A	MMP1	c.226A>T	P.S76C
SH_131	11	10821805	10821805	Missense_Mutation	SNV	T	C	C	EIF4G2	c.1837A>G	P.I613V
SH_131	11	132305072	132305072	Missense_Mutation	SNV	G	T	T	OPCML	c.945C>A	P.S315R
SH_131	11	16117579	16117579	Missense_Mutation	SNV	T	C	C	SOX6	c.1064A>G	P.H355R
SH_131	11	45971785	45971785	Silent	SNV	T	A	A	PHF21A	c.1116A>T	P.L372L
SH_131	11	57561528	57561528	Missense_Mutation	SNV	C	T	T	CTNND1	c.242C>T	P.S81L
SH_131	11	6806814	6806814	Silent	SNV	A	T	T	OR2AG1	c.546A>T	P.P182P
SH_131	11	85968550	85968550	Splice_Site	INDEL	A	-	-	EED	c.553-7delA	
SH_131	11	9101037	9101037	Missense_Mutation	SNV	A	T	T	SCUBE2	c.276T>A	P.N92K
SH_131	12	107080718	107080718	Missense_Mutation	SNV	A	T	T	RFX4	c.461A>T	P.Y154F
SH_131	12	111342549	111342549	Frame_Shift_Del	INDEL	C	-	-	CCDC63	c.1385delC	P.P462fs
SH_131	12	112668590	112668615	Frame_Shift_Del	INDEL	CACTGATGAA	-	-	HECTD4	GCGTCCCTTCAT	P.V1937fs
SH_131	12	11506787	11506787	Missense_Mutation	SNV	C	T	T	PRB1	c.250G>A	P.G84R
SH_131	12	21788503	21788503	Silent	SNV	G	A	A	LDHB	c.978C>T	P.D326D
SH_131	12	29648281	29648281	Missense_Mutation	SNV	T	A	A	OVCH1	c.391A>T	P.N131Y
SH_131	12	2994567	2994567	Missense_Mutation	SNV	A	T	T	RHNO1	c.35A>T	P.Q12L
SH_131	12	39967595	39967595	Missense_Mutation	SNV	A	T	T	ABCD2	c.1926T>A	P.D642E
SH_131	12	48145291	48145291	Silent	SNV	A	T	T	RAPGEF3	c.393T>A	P.S131S
SH_131	12	50452758	50452758	Missense_Mutation	SNV	A	T	T	ASIC1	c.209A>T	P.H70L
SH_131	12	52845352	52845352	Missense_Mutation	SNV	T	A	A	KRT6B	c.511A>T	P.N171Y
SH_131	12	53237959	53237959	Missense_Mutation	SNV	A	T	T	KRT78	c.635T>A	P.M212K
SH_131	12	59272763	59272763	Silent	SNV	T	A	A	LRIG3	c.1746A>T	P.T582T
SH_131	12	7848149	7848149	Missense_Mutation	SNV	T	A	A	GDF3	c.176A>T	P.E59V
SH_131	12	86377356	86377356	Silent	SNV	T	A	A	MGAT4C	c.240A>T	P.S80S
SH_131	12	95479603	95479603	Missense_Mutation	SNV	A	G	G	FGD6	c.4060T>C	P.W1354R
SH_131	13	108518427	108518427	Missense_Mutation	SNV	G	T	T	FAM155A	c.518C>A	P.P173H
SH_131	13	21562330	21562330	Missense_Mutation	SNV	T	A	A	LATS2	c.1589A>T	P.Q530L
SH_131	13	24797806	24797806	Missense_Mutation	SNV	A	T	T	SPATA13	c.739A>T	P.M247L
SH_131	13	53035553	53035553	Missense_Mutation	SNV	G	C	C	CKAP2	c.595G>C	P.D199H
SH_131	13	98673301	98673301	Missense_Mutation	SNV	A	G	G	IPO5	c.3175A>G	P.I1059V
SH_131	14	21789464	21789464	Missense_Mutation	SNV	T	A	A	RPGRIP1	c.1514T>A	P.L505Q
SH_131	14	31420109	31420109	Missense_Mutation	SNV	T	A	A	STRN3	c.502A>T	P.S168C
SH_131	14	69814679	69814679	Missense_Mutation	SNV	C	A	A	GALNT16	c.1499C>A	P.P500Q
SH_131	14	94395463	94395463	Nonsense_Mutation	SNV	A	T	T	FAM181A	c.832A>T	P.K278*
SH_131	15	100801716	100801716	Silent	SNV	C	T	T	ADAMTS17	c.999G>A	P.P333P
SH_131	15	25963545	25963545	Splice_Site	SNV	C	T	T	ATP10A	c.1365G>A	P.A455A
SH_131	15	31339447	31339447	Missense_Mutation	SNV	A	G	G	TRPM1	c.1682T>C	P.L561P
SH_131	15	42129008	42129008	Silent	SNV	G	T	T	JMJD7	c.732G>T	P.A244A
SH_131	15	49776595	49776595	Missense_Mutation	SNV	G	T	T	FGF7	c.479G>T	P.G160V
SH_131	15	55664086	55664086	Missense_Mutation	SNV	T	A	A	CCPG1	c.611A>T	P.K204M
SH_131	15	73635865	73635865	Missense_Mutation	SNV	A	T	T	HCN4	c.1070T>A	P.L357H
SH_131	15	74166078	74166078	Missense_Mutation	SNV	C	G	G	TBC1D21	c.28C>G	P.L10V
SH_131	16	22114849	22114849	Missense_Mutation	SNV	A	T	T	VWA3A	c.482A>T	P.K161M
SH_131	16	23208733	23208733	Silent	SNV	T	A	A	SCNN1G	c.1062T>A	P.S354S
SH_131	17	15609773	15609773	Missense_Mutation	SNV	A	T	T	ZNF286A	c.199A>T	P.R67W
SH_131	17	26861836	26861836	Missense_Mutation	SNV	C	A	A	FOXN1	c.1247C>A	P.P416Q
SH_131	17	38451717	38451717	Splice_Site	SNV	C	T	T	CDC6	c.1184+9C>T	
SH_131	17	39471690	39471690	Nonsense_Mutation	SNV	G	T	T	KRTAP17-1	c.213C>A	P.C71*
SH_131	17	39882134	39882134	Silent	SNV	G	A	A	HAP1	c.1332C>T	P.P444P
SH_131	17	41886396	41886396	Silent	SNV	T	C	C	MPP3	c.1509A>G	P.V503V
SH_131	17	42165866	42165866	Silent	SNV	A	C	C	HDAC5	c.1491T>G	P.T497T
SH_131	17	56060741	56060741	Missense_Mutation	SNV	G	A	A	VEZF1	c.47C>T	P.S16F
SH_131	17	7578239	7578239	Nonsense_Mutation	SNV	C	A	A	TP53	c.610G>T	P.E204*
SH_131	18	28934553	28934553	Silent	SNV	A	T	T	DSG1	c.2394A>T	P.P798P
SH_131	19	12014711	12014711	Splice_Site	SNV	A	G	G	ZNF69	c.191-9A>G	
SH_131	19	2834510	2834510	Missense_Mutation	SNV	A	T	T	ZNF554	c.1277A>T	P.H426L
SH_131	19	36213507	36213507	Missense_Mutation	SNV	A	T	T	KMT2B	c.2609A>T	P.H870L
SH_131	19	37117073	37117073	Nonsense_Mutation	SNV	G	T	T	ZNF382	c.271G>T	P.E91*
SH_131	19	38160851	38160851	Missense_Mutation	SNV	C	T	T	ZNF781	c.199G>A	P.G67R
SH_131	19	45296780	45296780	Missense_Mutation	SNV	G	T	T	CBLC	c.1049G>T	P.W350L
SH_131	19	5456494	5456494	Missense_Mutation	SNV	A	T	T	ZNRF4	c.992A>T	P.Y331F
SH_131	19	56244066	56244066	Missense_Mutation	SNV	T	A	A	NLRP9	c.1131A>T	P.K377N
SH_131	19	57036779	57036779	Missense_Mutation	SNV	A	T	T	ZNF471	c.1121A>T	P.Q374L
SH_131	19	58100036	58100036	Splice_Site	SNV	A	T	T	ZIK1	c.199+3A>T	
SH_131	19	58199438	58199438	Missense_Mutation	SNV	A	T	T	ZNF551	c.1711A>T	P.S571C
SH_131	19	964249	964249	Splice_Site	SNV	G	A	A	ARID3A	c.768G>A	P.G256G
SH_131	2	166165177	166165177	Missense_Mutation	SNV	T	C	C	SCN2A	c.478T>C	P.Y160H
SH_131	2	179247810	179247810	Missense_Mutation	SNV	G	A	A	OSBPL6	c.1756G>A	P.A586T
SH_131	2	179400006	179400006	Missense_Mutation	SNV	G	A	A	TTN	c.96413C>T	P.P32138L
SH_131	2	179594929	179594929	Silent	SNV	A	T	T	TTN	c.17247T>A	P.S5749S
SH_131	2	179612601	179612601	Silent	SNV	A	G	G	TTN	c.14526T>C	P.N4842N
SH_131	2	179640512	179640512	Missense_Mutation	SNV	A	T	T	TTN	c.6079T>A	P.Y2027N
SH_131	2	202245857	202245857	Missense_Mutation	SNV	T	A	A	TRAK2	c.2154A>T	P.R718S
SH_131	2	209198037	209198037	Splice_Site	SNV	A	G	G	PIKFYVE	c.3964-2A>G	

SH_131	2	219486194	219486194	Missense_Mutation	SNV	T	A	A	PLCD4	c.412T>A	P.W138R
SH_131	2	236659018	236659018	Missense_Mutation	SNV	C	T	T	AGAP1	c.559C>T	P.P187S
SH_131	2	47273501	47273501	Missense_Mutation	SNV	A	T	T	TTC7A	c.1850A>T	P.E617V
SH_131	2	74641666	74641666	Silent	SNV	T	A	A	C2orf81	c.1557A>T	P.T519T
SH_131	20	31619555	31619555	Splice_Site	SNV	T	A	A	BPIFB6	c.97+5T>A	
SH_131	20	36789850	36789850	Silent	SNV	T	A	A	TGM2	c.162A>T	P.V54V
SH_131	20	4705585	4705585	Missense_Mutation	SNV	C	A	A	PRND	c.388C>A	P.Q130K
SH_131	20	47297852	47297852	Silent	SNV	T	C	C	PREX1	c.1356A>G	P.L452L
SH_131	22	25282591	25282591	Missense_Mutation	SNV	C	T	T	SGSM1	c.1831C>T	P.R611C
SH_131	22	32482268	32482268	Nonsense_Mutation	SNV	T	A	A	SLC5A1	c.1083T>A	P.C361*
SH_131	22	50502577	50502577	Missense_Mutation	SNV	C	A	A	MLC1	c.945G>T	P.Q315H
SH_131	3	115395178	115395178	Frame_Shift_Del	INDEL	G	-	-	GAP43	c.457delG	P.D153fs
SH_131	3	123010209	123010209	Missense_Mutation	SNV	T	A	A	ADCY5	c.2028A>T	P.K676N
SH_131	3	129023648	129023648	Nonsense_Mutation	SNV	A	T	T	HMCES	c.1045A>T	P.K349*
SH_131	3	139088125	139088125	Missense_Mutation	SNV	C	A	A	COPB2	c.1379G>T	P.R460I
SH_131	3	139088127	139088127	Silent	SNV	T	C	C	COPB2	c.1377A>G	P.R459R
SH_131	3	170625534	170625534	Missense_Mutation	SNV	T	A	A	EIF5A2	c.62A>T	P.Q21L
SH_131	3	176755903	176755903	Missense_Mutation	SNV	C	A	A	TBL1XR1	c.1105G>T	P.D369Y
SH_131	3	182590191	182590191	Silent	SNV	A	G	G	ATP11B	c.1914A>G	P.K638K
SH_131	3	383699	383699	Missense_Mutation	SNV	T	A	A	CHL1	c.613T>A	P.F205I
SH_131	3	38593058	38593058	Splice_Site	SNV	A	T	T	SCN5A	c.4811-9T>A	
SH_131	3	38946723	38946723	Missense_Mutation	SNV	T	A	A	SCN11A	c.1563A>T	P.R521S
SH_131	3	51749744	51749744	Missense_Mutation	SNV	C	A	A	GRM2	c.1955C>A	P.T652N
SH_131	3	51750100	51750100	Missense_Mutation	SNV	A	G	G	GRM2	c.2311A>G	P.I771V
SH_131	4	37447057	37447057	Silent	SNV	T	A	A	NWD2	c.3447T>A	P.L1149L
SH_131	4	48993572	48993572	Missense_Mutation	SNV	T	A	A	CWH43	c.256T>A	P.S86T
SH_131	5	132535320	132535320	Missense_Mutation	SNV	C	A	A	FSTL4	c.1996G>T	P.A666S
SH_131	5	133643968	133643968	Missense_Mutation	SNV	T	C	C	CDKL3	c.1225A>G	P.I409V
SH_131	5	137420325	137420325	Missense_Mutation	SNV	G	A	A	WNT8A	c.295G>A	P.A99T
SH_131	5	178311177	178311177	Missense_Mutation	SNV	A	T	T	ZNF354B	c.1724A>T	P.E575V
SH_131	5	24487809	24487809	Missense_Mutation	SNV	T	C	C	CDH10	c.548A>G	P.E183G
SH_131	5	70351201	70351201	Missense_Mutation	SNV	T	A	A	GTF2H2	c.448A>T	P.S150C
SH_131	6	109980447	109980447	Missense_Mutation	SNV	T	A	A	AK9	c.614A>T	P.E205V
SH_131	6	118813033	118813033	Splice_Site	SNV	T	A	A	CEP85L	c.1258-5A>T	
SH_131	6	130370426	130370426	Splice_Site	SNV	G	T	T	L3MBTL3	c.103-1G>T	
SH_131	6	131574200	131574200	Missense_Mutation	SNV	G	A	A	AKAP7	c.73G>A	P.A25T
SH_131	6	136709537	136709537	Missense_Mutation	SNV	T	A	A	MAP7	c.586A>T	P.S196C
SH_131	6	152532640	152532640	Missense_Mutation	SNV	T	A	A	SYNE1	c.22365A>T	P.Q7455H
SH_131	6	159657345	159657345	Missense_Mutation	SNV	T	A	A	FNDC1	c.4066T>A	P.W1356R
SH_131	6	161807841	161807841	Silent	SNV	T	A	A	PARK2	c.1152A>T	P.S384S
SH_131	6	30883177	30883177	Missense_Mutation	SNV	A	T	T	VARS2	c.329A>T	P.E110V
SH_131	6	52943682	52943682	Silent	SNV	C	T	T	FBXO9	c.423C>T	P.G141G
SH_131	6	74121997	74121997	Silent	SNV	T	A	A	DDX43	c.1350T>A	P.V450V
SH_131	6	85457815	85457815	Splice_Site	SNV	A	T	T	TBX18	c.772-10T>A	
SH_131	6	88120348	88120348	Missense_Mutation	SNV	A	G	G	CFAP206	c.154A>G	P.R52G
SH_131	6	88125402	88125402	Splice_Site	SNV	A	T	T	CFAP206	c.284-2A>T	
SH_131	7	100087151	100087151	Missense_Mutation	SNV	A	T	T	NYAP1	c.1807A>T	P.I603F
SH_131	7	103243800	103243800	Missense_Mutation	SNV	T	A	A	RELN	c.3284A>T	P.E1095V
SH_131	7	127724820	127724820	Missense_Mutation	SNV	G	T	T	SND1	c.2155G>T	P.A719S
SH_131	7	140374503	140374503	Missense_Mutation	SNV	G	T	T	ADCK2	c.1026G>T	P.K342N
SH_131	7	142571345	142571345	Missense_Mutation	SNV	G	T	T	TRPV6	c.1764C>A	P.N588K
SH_131	7	143979885	143979885	Splice_Site	SNV	T	A	A	ARHGEF34P	n.2250-2A>T	
SH_131	7	147815235	147815235	Frame_Shift_Del	INDEL	C	-	-	CNTNAP2	c.2411delC	P.P804fs
SH_131	7	150831646	150831654	Frame_Shift_Del	INDEL	GGGGGCACA	-	-	AGAP3	1495+1delGGG	P.G497fs
SH_131	7	2617917	2617917	Silent	SNV	G	T	T	IQCE	c.507G>T	P.T169T
SH_131	7	2629736	2629736	Nonsense_Mutation	SNV	A	T	T	IQCE	c.1240A>T	P.R414*
SH_131	7	27148292	27148292	Nonsense_Mutation	SNV	T	A	A	HOXA3	c.574A>T	P.K192*
SH_131	7	38457432	38457432	Missense_Mutation	SNV	T	A	A	AMPH	c.1391A>T	P.E464V
SH_131	7	42005003	42005003	Missense_Mutation	SNV	T	A	A	GLI3	c.3668A>T	P.Y1223F
SH_131	7	44279206	44279206	Missense_Mutation	SNV	T	A	A	CAMK2B	c.1003A>T	P.M335L
SH_131	7	45115444	45115444	Nonsense_Mutation	SNV	A	T	T	CCM2	c.1186A>T	P.K396*
SH_131	7	6022520	6022520	Silent	SNV	C	A	A	PMS2	c.2109G>T	P.T703T
SH_131	7	6565851	6565851	Splice_Site	SNV	T	A	A	GRID2IP	c.919+4A>T	
SH_131	7	97822611	97822611	Missense_Mutation	SNV	A	T	T	LMTK2	c.2834A>T	P.E945V
SH_131	7	97863051	97863051	Missense_Mutation	SNV	T	A	A	TECPR1	c.1354A>T	P.R452W
SH_131	8	100973434	100973434	Splice_Site	SNV	A	G	G	RGS22	c.*46-10T>C	
SH_131	8	116616395	116616395	Nonsense_Mutation	SNV	T	A	A	TRPS1	c.1774A>T	P.R592*
SH_131	8	119936895	119936895	Silent	SNV	T	A	A	TNFRSF11B	c.924A>T	P.G308G
SH_131	8	2832079	2832079	Silent	SNV	G	C	C	CSMD1	c.8634C>G	P.G2878G
SH_131	8	57228583	57228583	Silent	SNV	T	A	A	SDR16C5	c.324A>T	P.V108V
SH_131	9	138660691	138660691	Missense_Mutation	SNV	T	A	A	KCNT1	c.1283T>A	P.L428Q
SH_131	X	110951488	110951488	Missense_Mutation	SNV	A	T	T	ALG13	c.617A>T	P.K206I
SH_131	X	49088392	49088392	Splice_Site	SNV	A	T	T	CACNA1F	c.26-3T>A	
SH_131	X	70341616	70341616	Missense_Mutation	SNV	A	T	T	MED12	c.1051A>T	P.M351L
SH_131	Y	14832516	14832516	Splice_Site	SNV	A	T	T	USP9Y	c.97-6A>T	
SH_132	1	247836117	247836117	Missense_Mutation	SNV	G	A	A	OR13G1	c.227C>T	P.P76L
SH_132	1	248487170	248487170	Missense_Mutation	SNV	C	T	T	OR2M7	c.701G>A	P.R234H
SH_132	1	33960027	33960027	Missense_Mutation	SNV	G	A	A	ZSCAN20	c.2083G>A	P.E695K
SH_132	1	92508402	92508402	Nonsense_Mutation	SNV	A	T	T	EPHX4	c.340A>T	P.R114*
SH_132	10	125804227	125804227	Missense_Mutation	SNV	C	A	A	CHST15	c.755G>T	P.C252F
SH_132	11	3060460	3060460	Silent	SNV	C	T	T	CARS	c.615G>A	P.A205A
SH_132	11	89450816	89450816	Missense_Mutation	SNV	C	G	G	TRIM77	c.1129C>G	P.L377V
SH_132	12	29608179	29608179	Splice_Site	SNV	T	A	A	OVCH1	c.2437+3A>T	
SH_132	12	51127954	51127954	Missense_Mutation	SNV	G	A	A	DIP2B	c.4018G>A	P.D1340N
SH_132	16	87637894	87637911	Missense_Mutation	INDEL	TGCTGCTGCT	-	-	JPH3	delCTGCTGCTGC	P.A152_A157del
SH_132	17	53068211	53068211	Missense_Mutation	SNV	A	G	G	STXBP4	c.73A>G	P.I25V
SH_132	17	76117181	76117181	Missense_Mutation	SNV	T	C	C	TMC6	c.1448A>G	P.N483S
SH_132	19	39125730	39125730	Missense_Mutation	SNV	G	C	C	EIF3K	c.519G>C	P.K173N
SH_132	2	207407998	207407998	Silent	SNV	C	G	G	ADAM23	c.678C>G	P.T226T
SH_132	2	209113113	209113113	Missense_Mutation	SNV	G	A	A	IDH1	c.394C>T	P.R132C
SH_132	3	119222460	119222460	Missense_Mutation	SNV	A	G	G	TIMMDC1	c.442A>G	P.I148V
SH_132	3	124527879	124527879	Missense_Mutation	SNV	A	G	G	ITGB5	c.1253T>C	P.I418T
SH_132	3	183994711	183994711	Missense_Mutation	SNV	T	C	C	ECE2	c.71T>C	P.L24S
SH_132	6	150005276	150005276	Nonsense_Mutation	SNV	G	A	A	LATS1	c.949C>T	P.Q317*
SH_132	7	2565091	2565091	Missense_Mutation	SNV	G	T	T	LFNG	c.625G>T	P.A209S
SH_132	X	148062316	148062316	Missense_Mutation	SNV	G	A	A	AFF2	c.3514G>A	P.G1172R
SH_132	X	3238828	3238828	Missense_Mutation	SNV	A	T	T	MXRA5	c.4898T>A	P.F1633Y
SH_133	1	152187174	152187174	Missense_Mutation	SNV	G	T	T	HRNR	c.6931C>A	P.H2311N

SH_133	1	160192555	160192555	Missense_Mutation	SNV	G	C	C	DCAF8	c.1326C>G	P.F442L
SH_133	1	173526502	173526502	Nonsense_Mutation	SNV	G	A	A	SLC9C2	c.1192C>T	P.R398*
SH_133	1	216850804	216850804	Missense_Mutation	SNV	C	G	G	ESRRG	c.17G>C	P.R6P
SH_133	1	248185835	248185835	Missense_Mutation	SNV	T	A	A	OR2L5	c.586T>A	P.Y196N
SH_133	1	248487703	248487703	Missense_Mutation	SNV	G	T	T	OR2M7	c.168C>A	P.H56Q
SH_133	1	26230211	26230211	Missense_Mutation	SNV	G	A	A	STMN1	c.107C>T	P.P36L
SH_133	1	29606044	29606044	Missense_Mutation	SNV	G	A	A	PTPRU	c.1640G>A	P.R547H
SH_133	1	39823347	39823347	Missense_Mutation	SNV	A	G	G	MACF1	c.5539A>G	P.S1847G
SH_133	1	42049239	42049239	Silent	SNV	G	A	A	HIVEP3	c.1230C>T	P.N410N
SH_133	1	49193587	49193587	Nonsense_Mutation	SNV	G	A	A	BEND5	c.730C>T	P.R244*
SH_133	1	55559732	55559732	Missense_Mutation	SNV	G	A	A	USP24	c.6232C>T	P.P2078S
SH_133	1	874822	874822	Missense_Mutation	SNV	C	A	A	SAMD11	c.688C>A	P.H230N
SH_133	1	91182581	91182581	Missense_Mutation	SNV	C	T	T	BARHL2	c.172G>A	P.V58I
SH_133	1	92944306	92944306	Missense_Mutation	SNV	C	T	T	GFI1	c.929G>A	P.R310Q
SH_133	10	129536928	129536928	Missense_Mutation	SNV	C	A	A	FOXI2	c.656C>A	P.A219E
SH_133	10	50594558	50594558	Silent	SNV	G	A	A	DRGX	c.492C>T	P.Y164Y
SH_133	10	64136198	64136198	Silent	SNV	G	A	A	ZNF365	c.246G>A	P.P82P
SH_133	10	72513565	72513565	Silent	SNV	G	A	A	ADAMTS14	c.2739G>A	P.T913T
SH_133	11	124489462	124489462	Missense_Mutation	SNV	G	C	C	PANX3	c.810G>C	P.Q270H
SH_133	11	124495747	124495747	Missense_Mutation	SNV	G	T	T	TBRG1	c.402G>T	P.K134N
SH_133	11	1977498	1977498	Missense_Mutation	SNV	A	G	G	MRPL23	c.310A>G	P.T104A
SH_133	11	33565454	33565454	Missense_Mutation	SNV	G	T	T	KIAA1549L	c.1454G>T	P.S485I
SH_133	11	46800054	46800054	Silent	SNV	G	A	A	CKAP5	c.2529C>T	P.D843D
SH_133	11	755975	755975	Missense_Mutation	SNV	C	T	T	TALDO1	c.194C>T	P.A65V
SH_133	12	109637326	109637326	Missense_Mutation	SNV	T	A	A	ACACB	c.2747T>A	P.V916D
SH_133	12	132837669	132837669	Missense_Mutation	SNV	C	T	T	GALNT9	c.626G>A	P.R209Q
SH_133	12	18234370	18234370	Nonsense_Mutation	SNV	G	A	A	RERGL	c.370C>T	P.R124*
SH_133	12	25398285	25398285	Missense_Mutation	SNV	C	A	A	KRAS	c.34G>T	P.G12C
SH_133	12	27126968	27126968	Missense_Mutation	SNV	C	T	T	TM7SF3	c.1643G>A	P.R548Q
SH_133	12	56481627	56481627	Missense_Mutation	SNV	C	A	A	ERBB3	c.662C>A	P.P221H
SH_133	12	81647354	81647354	Nonsense_Mutation	SNV	C	T	T	ACSS3	c.1900C>T	P.R634*
SH_133	12	85460577	85460577	Missense_Mutation	SNV	G	T	T	LRRIQ1	c.2596G>T	P.V866F
SH_133	13	101890116	101890116	Missense_Mutation	SNV	G	A	A	NALCN	c.1424C>T	P.T475M
SH_133	13	103400936	103400936	Missense_Mutation	SNV	G	T	T	CCDC168	c.2111C>A	P.S704Y
SH_133	13	49281758	49281758	Missense_Mutation	SNV	G	A	A	CYSLTR2	c.805G>A	P.V269I
SH_133	13	52523858	52523858	Silent	SNV	C	T	T	ATP7B	c.2805G>A	P.T935T
SH_133	14	19563432	19563432	Nonsense_Mutation	SNV	G	T	T	POTEG	c.946G>T	P.G316*
SH_133	14	65009472	65009472	Silent	SNV	C	T	T	HSPA2	c.1905C>T	P.I635I
SH_133	15	45398328	45398328	Missense_Mutation	SNV	C	T	T	DUOX2	c.2143G>A	P.D715N
SH_133	15	75975220	75975220	Nonsense_Mutation	SNV	G	A	A	CSPG4	c.4612C>T	P.Q1538*
SH_133	15	79265694	79265694	Missense_Mutation	SNV	G	A	A	RASGRF1	c.3563C>T	P.T1188M
SH_133	15	86259119	86259119	Silent	SNV	G	A	A	AKAP13	c.1563G>A	P.S521S
SH_133	15	88576096	88576096	Missense_Mutation	SNV	G	A	A	NTRK3	c.1577C>T	P.P526L
SH_133	16	32264962	32264962	Silent	SNV	T	C	C	TP53TG3D	c.288T>C	P.H96H
SH_133	16	75665747	75665747	Frame_Shift_Del	INDEL	C	-	-	KARS	c.1006delG	P.V336fs
SH_133	17	10543537	10543537	Missense_Mutation	SNV	G	A	A	MYH3	c.2458C>T	P.R820C
SH_133	17	10549104	10549104	Missense_Mutation	SNV	G	A	A	MYH3	c.1061C>T	P.T354M
SH_133	17	33267585	33267585	Missense_Mutation	SNV	C	T	T	CCT6B	c.830G>A	P.R277H
SH_133	17	73723903	73723903	Silent	SNV	C	T	T	ITGB4	c.436C>T	P.L146L
SH_133	18	48591919	48591919	Missense_Mutation	SNV	G	A	A	SMAD4	c.1082G>A	P.R361H
SH_133	18	59166595	59166595	Silent	SNV	G	A	A	CDH20	c.423G>A	P.T141T
SH_133	19	11327936	11327936	Missense_Mutation	SNV	G	A	A	DOCK6	c.3682C>T	P.R1228W
SH_133	19	18707544	18707544	Silent	SNV	G	T	T	CRLF1	c.912C>A	P.G304G
SH_133	19	2339972	2339972	Splice_Site	SNV	G	A	A	SPPL2B	c.742+7G>A	
SH_133	19	31768611	31768611	Silent	SNV	C	T	T	TSHZ3	c.2088G>A	P.K696K
SH_133	19	37382567	37382567	Missense_Mutation	SNV	C	A	A	ZNF829	c.1126G>T	P.D376Y
SH_133	19	3964908	3964908	Silent	SNV	G	A	A	DAPK3	c.144C>T	P.R48R
SH_133	19	46733629	46733629	Missense_Mutation	SNV	G	A	A	IGFL1	c.178G>A	P.V60I
SH_133	19	47228739	47228739	Missense_Mutation	SNV	G	A	A	STRN4	c.1436C>T	P.T479M
SH_133	19	47844845	47844845	Silent	SNV	C	T	T	C5AR2	c.789C>T	P.S263S
SH_133	19	48305953	48305953	Silent	SNV	G	T	T	TPRX1	c.315C>A	P.G105G
SH_133	19	50243064	50243064	Frame_Shift_Del	INDEL	G	-	-	TSKS	c.1748delC	P.P583fs
SH_133	19	50476388	50476388	Splice_Site	SNV	C	T	T	SIGLEC16	n.1551+9C>T	
SH_133	19	52942411	52942411	Silent	SNV	G	A	A	ZNF534	c.1698G>A	P.A566A
SH_133	19	5455995	5455995	Missense_Mutation	SNV	G	A	A	ZNRF4	c.493G>A	P.D165N
SH_133	19	55753733	55753733	Splice_Site	SNV	C	T	T	PPP6R1	c.731+11G>A	
SH_133	19	57286598	57286598	Missense_Mutation	SNV	G	A	A	ZIM2	c.1042C>T	P.R348W
SH_133	19	9062407	9062407	Missense_Mutation	SNV	G	T	T	MUC16	c.25039C>A	P.P8347T
SH_133	2	136873083	136873083	Missense_Mutation	SNV	C	T	T	CXCR4	c.427G>A	P.V143I
SH_133	2	160043530	160043530	Missense_Mutation	SNV	G	A	A	TANC1	c.2713G>A	P.V905M
SH_133	2	166895977	166895977	Missense_Mutation	SNV	G	T	T	SCN1A	c.2545C>A	P.L849I
SH_133	2	166898844	166898844	Nonsense_Mutation	SNV	G	A	A	SCN1A	c.2134C>T	P.R712*
SH_133	2	179260240	179260240	Missense_Mutation	SNV	G	A	A	OSBPL6	c.2834G>A	P.R945Q
SH_133	2	179522883	179522883	Splice_Site	SNV	C	A	A	TTN	c.37713G>T	P.V12571V
SH_133	2	189904068	189904068	Silent	SNV	G	A	A	COL5A2	c.3855C>T	P.P1285P
SH_133	2	200137047	200137047	Missense_Mutation	SNV	C	T	T	SATB2	c.2089G>A	P.E697K
SH_133	2	210642204	210642204	Missense_Mutation	SNV	G	A	A	UNC80	c.521G>A	P.R174Q
SH_133	2	220088899	220088899	Silent	SNV	G	A	A	ATG9A	c.1194C>T	P.D398D
SH_133	2	228769723	228769723	Missense_Mutation	SNV	G	A	A	DAW1	c.682G>A	P.V228I
SH_133	2	27601343	27601343	Nonsense_Mutation	SNV	G	A	A	ZNF513	c.604C>T	P.R202*
SH_133	2	43452831	43452831	Frame_Shift_Ins	INDEL	-	C	C	ZFP36L2	c.111dupG	P.T38fs
SH_133	2	84777081	84777081	Missense_Mutation	SNV	C	T	T	DNAH6	c.1385C>T	P.S462L
SH_133	2	98273619	98273619	Missense_Mutation	SNV	T	A	A	ACTR1B	c.1024A>T	P.I342F
SH_133	22	43603587	43603587	Missense_Mutation	SNV	G	A	A	SCUBE1	c.2767C>T	P.R923C
SH_133	22	43831108	43831108	Missense_Mutation	SNV	G	A	A	MPPED1	c.379G>A	P.E127K
SH_133	22	43933407	43933407	Missense_Mutation	SNV	C	T	T	EFCAB6	c.3898G>A	P.V1300M
SH_133	3	12224924	12224924	Missense_Mutation	SNV	A	C	C	SYN2	c.1418A>C	P.Q473P
SH_133	3	148894115	148894115	Missense_Mutation	SNV	T	G	G	CP	c.3103A>C	P.I1035L
SH_133	3	178921553	178921553	Missense_Mutation	SNV	T	A	A	PIK3CA	c.1035T>A	P.N345K
SH_133	3	36763045	36763045	Missense_Mutation	SNV	C	T	T	DCLK3	c.1558G>A	P.V520I
SH_133	3	49698778	49698778	Missense_Mutation	SNV	C	A	A	BSN	c.9500C>A	P.P3167H
SH_133	3	49831352	49831352	Missense_Mutation	SNV	G	A	A	CDHR4	c.1367C>T	P.A456V
SH_133	3	67411060	67411060	Frame_Shift_Del	INDEL	A	-	-	SUCLG2	c.1316delT	P.L439fs
SH_133	4	144545333	144545333	Missense_Mutation	SNV	G	T	T	FREM3	c.5581C>A	P.L1861M
SH_133	4	183267884	183267884	Missense_Mutation	SNV	C	A	A	TENM3	c.313C>A	P.L105I
SH_133	4	187554989	187554989	Splice_Site	SNV	A	G	G	FAT1	c.4184-12T>C	
SH_133	4	40099049	40099049	Missense_Mutation	SNV	G	A	A	N4BP2	c.89G>A	P.R30H
SH_133	4	57522119	57522119	Silent	SNV	G	A	A	HOPX	c.48C>T	P.I16I

SH_133	4	77324344	77324344	Missense_Mutation	SNV	C	A	A	CCDC158	c.17G>T	P.W6L
SH_133	5	112175752	112175752	Frame_Shift_Del	INDEL	T	-	-	APC	c.4463delT	P.L1488fs
SH_133	5	118969858	118969858	Missense_Mutation	SNV	G	A	A	FAM170A	c.274G>A	P.V92M
SH_133	5	140215137	140215137	Missense_Mutation	SNV	C	T	T	PCDHA7	c.1169C>T	P.T390M
SH_133	5	140431404	140431404	Missense_Mutation	SNV	T	C	C	PCDHB1	c.349T>C	P.F117L
SH_133	5	140764210	140764210	Missense_Mutation	SNV	G	A	A	PCDHGA7	c.1744G>A	P.A582T
SH_133	5	140773552	140773552	Missense_Mutation	SNV	C	T	T	PCDHGA8	c.1172C>T	P.P391L
SH_133	5	33577076	33577076	Missense_Mutation	SNV	G	C	C	ADAMTS12	c.3055C>G	P.L1019V
SH_133	5	34796132	34796132	Missense_Mutation	SNV	G	A	A	RAI14	c.256G>A	P.G86R
SH_133	6	152674520	152674520	Nonsense_Mutation	SNV	C	A	A	SYNE1	c.11086G>T	P.E3696*
SH_133	6	26123757	26123757	Frame_Shift_Ins	INDEL	-	G	G	HIST1H2BC	c.375dupC	P.K126fs
SH_133	6	4943906	4943906	Nonsense_Mutation	SNV	G	A	A	CDYL	c.852G>A	P.W284*
SH_133	6	83905381	83905381	Missense_Mutation	SNV	G	A	A	RWDD2A	c.44G>A	P.R15Q
SH_133	6	88853781	88853781	Nonsense_Mutation	SNV	G	A	A	CNR1	c.1213C>T	P.R405*
SH_133	7	126173278	126173278	Missense_Mutation	SNV	G	T	T	GRM8	c.2158C>A	P.H720N
SH_133	7	140048419	140048419	Splice_Site	SNV	G	T	T	SLC37A3	c.1024+7C>A	
SH_133	7	156752835	156752835	Silent	SNV	C	T	T	NOM1	c.1599C>T	P.S533S
SH_133	7	28848874	28848874	Missense_Mutation	SNV	G	A	A	CREB5	c.680G>A	P.R227Q
SH_133	7	30962227	30962227	Missense_Mutation	SNV	G	A	A	AQP1	c.598G>A	P.A200T
SH_133	7	69064885	69064885	Silent	SNV	G	A	A	AUTS2	c.246G>A	P.S82S
SH_133	7	73752935	73752935	Silent	SNV	C	T	T	CLIP2	c.279C>T	P.G93G
SH_133	7	75694258	75694258	Missense_Mutation	SNV	C	T	T	MDH2	c.746C>T	P.P249L
SH_133	7	99802682	99802682	Silent	SNV	G	A	A	STAG3	c.3006G>A	P.Q1002Q
SH_133	8	104928737	104928737	Missense_Mutation	SNV	G	A	A	RIMS2	c.2008G>A	P.V670I
SH_133	8	125080023	125080023	Missense_Mutation	SNV	C	A	A	FER1L6	c.3694C>A	P.P1232T
SH_133	8	134034311	134034311	Missense_Mutation	SNV	G	A	A	TG	c.6952G>A	P.D2318N
SH_133	8	24811765	24811765	Nonsense_Mutation	SNV	G	A	A	NEFL	c.1099C>T	P.R367*
SH_133	8	32614052	32614052	Splice_Site	SNV	G	A	A	NRG1	c.921+5G>A	
SH_133	8	41122786	41122786	Missense_Mutation	SNV	G	A	A	SFRP1	c.845C>T	P.T282M
SH_133	8	66659975	66659975	Missense_Mutation	SNV	C	T	T	PDE7A	c.347G>A	P.R116Q
SH_133	9	108297336	108297336	Splice_Site	SNV	G	C	C	FSD1L	c.1127-12G>C	
SH_133	X	138643671	138643671	Splice_Site	SNV	T	A	A	F9	c.839-12T>A	
SH_133	X	140785800	140785800	Missense_Mutation	SNV	G	T	T	SPANXC	c.116C>A	P.P39Q
SH_134	1	111957781	111957781	Missense_Mutation	SNV	C	T	T	OVGP1	c.1342G>A	P.G448S
SH_134	1	32381586	32381586	Splice_Site	SNV	T	C	C	PTP4A2	c.99A>G	P.E33E
SH_134	1	3784569	3784569	Silent	SNV	C	G	G	DFFB	c.534C>G	P.G178G
SH_134	10	102739956	102739958	Missense_Mutation	INDEL	GTT	-	-	SEMA4G	1207_1209delT	P.L403del
SH_134	10	43606853	43606853	Missense_Mutation	SNV	A	T	T	RET	c.1462A>T	P.T488S
SH_134	10	89711966	89711966	Missense_Mutation	SNV	T	C	C	PTEN	c.584T>C	P.F195S
SH_134	11	57886041	57886041	Silent	SNV	T	C	C	OR9I1	c.876A>G	P.L292L
SH_134	11	66033568	66033568	Missense_Mutation	SNV	G	A	A	KLC2	c.1376G>A	P.G459D
SH_134	11	71208554	71208554	Missense_Mutation	SNV	A	G	G	NADSYN1	c.1790A>G	P.E597G
SH_134	11	78177001	78177001	Missense_Mutation	SNV	A	T	T	NARS2	c.404T>A	P.I135K
SH_134	12	108985455	108985455	Silent	SNV	C	T	T	TMEM119	c.705G>A	P.P235P
SH_134	12	25398284	25398284	Missense_Mutation	SNV	C	G	G	KRAS	c.35G>C	P.G12A
SH_134	12	58014749	58014749	Silent	SNV	C	T	T	SLC26A10	c.264C>T	P.G88G
SH_134	12	72022795	72022795	Missense_Mutation	SNV	T	G	G	ZFC3H1	c.3849A>C	P.K1283N
SH_134	12	80770982	80770982	Splice_Site	SNV	T	A	A	OTOGL	c.6834T>A	P.P2278P
SH_134	13	24871809	24871809	Frame_Shift_Ins	INDEL	-	T	T	SPATA13	c.3519_3520ins	P.K1174fs
SH_134	13	96438330	96438330	Splice_Site	SNV	A	T	T	DNAJC3	c.1208+5A>T	
SH_134	14	75330206	75330206	Missense_Mutation	SNV	G	A	A	PROX2	c.332C>T	P.P111L
SH_134	15	32460267	32460267	Missense_Mutation	SNV	G	A	A	CHRNA7	c.1117G>A	P.A373T
SH_134	15	33955053	33955053	Silent	SNV	G	C	C	RYR3	c.5322G>C	P.V1774V
SH_134	15	43512935	43512935	Missense_Mutation	SNV	C	A	A	EPB42	c.89G>T	P.S30I
SH_134	15	52506866	52506866	Missense_Mutation	SNV	T	C	C	MYO5C	c.3976A>G	P.M1326V
SH_134	15	90125922	90125922	Nonsense_Mutation	SNV	G	A	A	TICRR	c.660G>A	P.W220*
SH_134	15	90347207	90347207	Missense_Mutation	SNV	C	A	A	ANPEP	c.1206G>T	P.E402D
SH_134	16	20944736	20944736	Frame_Shift_Del	INDEL	T	-	-	DNAH3	c.12091delA	P.T4031fs
SH_134	16	28499036	28499036	Silent	SNV	G	C	C	CLN3	c.321C>G	P.P107P
SH_134	16	4458241	4458241	Missense_Mutation	SNV	C	T	T	CORO7	c.244G>A	P.D82N
SH_134	16	731334	731334	Silent	SNV	C	T	T	STUB1	c.126C>T	P.I42I
SH_134	17	17928015	17928015	Nonsense_Mutation	SNV	G	C	C	ATPAF2	c.426C>G	P.Y142*
SH_134	17	2604454	2604454	Silent	SNV	G	A	A	CLUH	c.891C>T	P.F297F
SH_134	17	40950129	40950129	Missense_Mutation	SNV	C	T	T	COA3	c.271G>A	P.E91K
SH_134	17	7574017	7574017	Missense_Mutation	SNV	C	A	A	TP53	c.1010G>T	P.R337L
SH_134	17	7577547	7577547	Missense_Mutation	SNV	C	T	T	TP53	c.734G>A	P.G245D
SH_134	19	17756595	17756595	Missense_Mutation	SNV	G	C	C	UNC13A	c.2244C>G	P.D748E
SH_134	19	35758719	35758719	Splice_Site	SNV	C	A	A	LSR	c.1855-8C>A	
SH_134	19	53619609	53619609	Nonsense_Mutation	SNV	G	C	C	ZNF415	c.93C>G	P.Y31*
SH_134	19	54802050	54802050	Missense_Mutation	SNV	C	T	T	LILRA3	c.946G>A	P.A316T
SH_134	19	56009241	56009241	Splice_Site	SNV	C	T	T	SSC5D	c.1388-6C>T	
SH_134	19	984437	984437	Silent	SNV	C	T	T	WDR18	c.84C>T	P.G28G
SH_134	2	118771520	118771520	Splice_Site	SNV	G	A	A	CCDC93	c.42+10C>T	
SH_134	2	131130445	131130445	Splice_Site	SNV	G	A	A	PTPN18	c.920-1G>A	
SH_134	20	40877391	40877391	Missense_Mutation	SNV	C	T	T	PTPRT	c.2305G>A	P.V769M
SH_134	5	140755446	140755446	Missense_Mutation	SNV	G	T	T	PCDHGA6	c.1796G>T	P.G599V
SH_134	5	33576822	33576822	Missense_Mutation	SNV	C	G	G	ADAMTS12	c.3054G>C	P.E1018D
SH_134	6	26205128	26205128	Missense_Mutation	SNV	G	A	A	HIST1H4E	c.256G>A	P.D86N
SH_134	6	30708545	30708545	Missense_Mutation	SNV	C	T	T	FLOT1	c.241G>A	P.E81K
SH_134	6	46659271	46659271	Missense_Mutation	SNV	A	G	G	TDRD6	c.3406A>G	P.T1136A
SH_134	6	73332234	73332234	Missense_Mutation	SNV	T	C	C	KCNQ5	c.317T>C	P.V106A
SH_134	7	131913188	131913188	Missense_Mutation	SNV	G	A	A	PLXNA4	c.1645C>T	P.R549C
SH_134	7	142612744	142612744	Splice_Site	SNV	T	A	A	TRPV5	c.1123-6A>T	
SH_134	7	23293812	23293812	Missense_Mutation	SNV	C	G	G	GPNMB	c.248C>G	P.T83S
SH_134	9	104171708	104171708	Missense_Mutation	SNV	C	T	T	ZNF189	c.1616C>T	P.S539L
SH_134	9	107599377	107599377	Missense_Mutation	SNV	C	T	T	ABCA1	c.1195G>A	P.V399M
SH_134	X	100747980	100747980	Silent	SNV	G	A	A	ARMCX4	c.4404G>A	P.Q1468Q
SH_134	X	114400852	114400852	Missense_Mutation	SNV	C	A	A	LRCH2	c.1052G>T	P.S351I
SH_134	X	129299554	129299554	Missense_Mutation	SNV	C	T	T	AIFM1	c.77G>A	P.R26Q
SH_134	X	51151318	51151318	Missense_Mutation	SNV	C	T	T	CXorf67	c.1450C>T	P.P484S
SH_134	X	69366580	69366580	Missense_Mutation	SNV	C	T	T	IGBP1	c.580C>T	P.L194F
SH_135	1	118491040	118491040	Nonsense_Mutation	SNV	C	T	T	WDR3	c.1435C>T	P.Q479*
SH_135	1	11883785	11883785	Missense_Mutation	SNV	A	G	G	CLCN6	c.409A>G	P.I137V
SH_135	1	155264362	155264362	Silent	SNV	G	A	A	PKLR	c.876C>T	P.S292S
SH_135	1	156018394	156018394	Missense_Mutation	SNV	G	T	T	UBQLN4	c.738C>A	P.D246E
SH_135	1	159002369	159002369	Frame_Shift_Del	INDEL	A	-	-	IFI16	c.1049delA	P.Q350fs
SH_135	1	176671803	176671803	Silent	SNV	G	T	T	PAPPA2	c.3297G>T	P.L1099L
SH_135	1	186120313	186120313	Splice_Site	INDEL	T	-	-	HMCN1	c.14609-11delT	

SH_135	1	196303097	196303097	Missense_Mutation	SNV	C	G	G	KCNT2	c.1877G>C	P.R626T
SH_135	1	196759303	196759303	Missense_Mutation	SNV	T	A	A	CFHR3	c.559T>A	P.S187T
SH_135	1	206328960	206328960	Splice_Site	SNV	A	C	C	CTSE	c.561-2A>C	
SH_135	1	21900187	21900187	Missense_Mutation	SNV	G	A	A	ALPL	c.892G>A	P.E298K
SH_135	1	223976872	223976872	Missense_Mutation	SNV	G	C	C	TP53BP2	c.3001C>G	P.P1001A
SH_135	1	231830517	231830517	Missense_Mutation	SNV	G	C	C	DISC1	c.1013G>C	P.R338P
SH_135	1	237774219	237774219	Missense_Mutation	SNV	G	A	A	RYR2	c.4841G>A	P.R1614H
SH_135	1	247492768	247492768	Missense_Mutation	SNV	G	T	T	ZNF496	c.113C>A	P.P38H
SH_135	1	47610279	47610279	Missense_Mutation	SNV	A	G	G	CYP4A22	c.955A>G	P.M319V
SH_135	1	52264057	52264057	Missense_Mutation	SNV	T	C	C	NRDC	c.2468A>G	P.Y823C
SH_135	1	54607096	54607096	Silent	SNV	G	A	A	CDCP2	c.438C>T	P.G146G
SH_135	1	76384798	76384798	Missense_Mutation	SNV	C	G	G	ASB17	c.727G>C	P.D243H
SH_135	1	7731083	7731083	Missense_Mutation	SNV	G	A	A	CAMTA1	c.2765G>A	P.R922H
SH_135	1	7731084	7731084	Silent	SNV	C	A	A	CAMTA1	c.2766C>A	P.R922R
SH_135	1	86622024	86622024	Missense_Mutation	SNV	G	C	C	COL24A1	c.56C>G	P.T19R
SH_135	10	6521061	6521061	Missense_Mutation	SNV	C	G	G	PRKCQ	c.1246G>C	P.V416L
SH_135	11	102733870	102733870	Silent	SNV	G	A	A	MMP12	c.1371C>T	P.I457I
SH_135	11	113264223	113264223	Missense_Mutation	SNV	T	A	A	ANKK1	c.206T>A	P.I69N
SH_135	11	118955769	118955769	Missense_Mutation	SNV	C	T	T	HMBS	c.26C>T	P.A9V
SH_135	11	17653705	17653705	Frame_Shift_Del	INDEL	T	-	-	OTOG	c.7040delT	P.L2347fs
SH_135	11	20805362	20805362	Silent	SNV	A	T	T	NELL1	c.405A>T	P.R135R
SH_135	11	71698065	71698065	Missense_Mutation	SNV	G	C	C	RNF121	c.318G>C	P.W106C
SH_135	11	96092240	96092240	Missense_Mutation	SNV	T	C	C	CCDC82	c.1483A>G	P.T495A
SH_135	12	16035656	16035656	Silent	SNV	G	A	A	STRAP	c.15G>A	P.Q5Q
SH_135	12	25173843	25173843	Splice_Site	SNV	T	A	A	LOC645177	c.1305+2T>A	
SH_135	12	2742879	2742882	Splice_Site	INDEL	GTGA	-	-	CACNA1C	2+3_3972+6delGAGT	
SH_135	12	6692459	6692459	Missense_Mutation	SNV	T	C	C	CHD4	c.3965A>G	P.Y1322C
SH_135	12	97151272	97151272	Splice_Site	SNV	G	T	T	CFAP54	c.7947-1G>T	
SH_135	12	99640648	99640648	Splice_Site	SNV	A	G	G	ANKS1B	c.1757-6T>C	
SH_135	13	114088137	114088137	Missense_Mutation	SNV	C	T	T	ADPRHL1	c.425G>A	P.R142Q
SH_135	13	114782808	114782808	Nonsense_Mutation	SNV	G	A	A	RASA3	c.1015C>T	P.R339*
SH_135	13	28794407	28794407	Nonsense_Mutation	SNV	G	T	T	PAN3	c.892G>T	P.G298*
SH_135	13	38266351	38266351	Missense_Mutation	SNV	A	T	T	TRPC4	c.1019T>A	P.F340Y
SH_135	13	99948153	99948153	Missense_Mutation	SNV	C	T	T	GPR183	c.247G>A	P.A83T
SH_135	14	20924154	20924154	Frame_Shift_Ins	INDEL	-	C	C	APEX1	c.146dupC	P.D50fs
SH_135	14	21681156	21681156	Silent	SNV	A	C	C	HNRNPC	c.525T>G	P.S175S
SH_135	14	37737996	37737996	Nonsense_Mutation	SNV	A	T	T	MIPOL1	c.385A>T	P.K129*
SH_135	14	74988750	74988750	Splice_Site	SNV	G	A	A	LTBP2	c.2660-8C>T	
SH_135	14	81950704	81950704	Missense_Mutation	SNV	A	C	C	SEL1L	c.1911T>G	P.H637Q
SH_135	15	25220642	25220642	Missense_Mutation	SNV	G	T	T	SNRPN	c.141G>T	P.E47D
SH_135	15	41071483	41071483	Missense_Mutation	SNV	G	A	A	DNAJC17	c.233C>T	P.A78V
SH_135	15	45427385	45427385	Missense_Mutation	SNV	G	A	A	DUOX1	c.391G>A	P.D131N
SH_135	16	11940401	11940401	Missense_Mutation	SNV	T	C	C	RSL1D1	c.592A>G	P.I198V
SH_135	16	29997924	29997924	Silent	SNV	A	C	C	TAOK2	c.2331A>C	P.S777S
SH_135	16	68011904	68011904	Silent	SNV	C	T	T	DPEP3	c.768G>A	P.E256E
SH_135	16	89764086	89764086	Frame_Shift_Ins	INDEL	-	AG	AG	SPATA2L	c.930_931insCT	P.S311fs
SH_135	16	89764088	89764088	Missense_Mutation	SNV	A	C	C	SPATA2L	c.929T>G	P.L310R
SH_135	17	12917796	12917796	Silent	SNV	T	G	G	ELAC2	c.450A>C	P.A150A
SH_135	17	19286158	19286158	Silent	SNV	C	T	T	MAPK7	c.2196C>T	P.G732G
SH_135	17	19611607	19611607	Splice_Site	SNV	G	A	A	SLC47A2	c.629+8C>T	
SH_135	17	34746986	34746986	Silent	SNV	C	A	A	TBC1D3F	c.1098G>T	P.G366G
SH_135	17	38289323	38289323	Missense_Mutation	SNV	G	A	A	MSL1	c.728G>A	P.R243Q
SH_135	17	65732794	65732794	Missense_Mutation	SNV	T	C	C	NOL11	c.521T>C	P.V174A
SH_135	17	73664598	73664598	Missense_Mutation	SNV	G	C	C	SAP30BP	c.109G>C	P.E37Q
SH_135	17	7577018	7577018	Splice_Site	SNV	C	A	A	TP53	c.919+1G>T	
SH_135	17	76411040	76411040	Missense_Mutation	SNV	C	G	G	PGS1	c.1483C>G	P.H495D
SH_135	17	7912916	7912916	Splice_Site	SNV	G	A	A	GUCY2D	c.1749+12G>A	
SH_135	18	31709893	31709893	Missense_Mutation	SNV	G	A	A	NOL4	c.356C>T	P.T119M
SH_135	18	48591888	48591888	Missense_Mutation	SNV	G	T	T	SMAD4	c.1051G>T	P.D351Y
SH_135	18	61306469	61306469	Missense_Mutation	SNV	C	A	A	SERPINB4	c.718G>T	P.D240Y
SH_135	18	658196	658196	Missense_Mutation	SNV	G	C	C	TYMSOS	c.52C>G	P.P18A
SH_135	18	9337317	9337317	Silent	SNV	C	G	G	TWSG1	c.90C>G	P.L30L
SH_135	19	10463703	10463703	Missense_Mutation	SNV	G	C	C	TYK2	c.3099C>G	P.N1033K
SH_135	19	11979192	11979192	Missense_Mutation	SNV	A	T	T	ZNF439	c.1308A>T	P.K436N
SH_135	19	14717866	14717866	Missense_Mutation	SNV	C	A	A	CLEC17A	c.956C>A	P.A319D
SH_135	19	35613764	35613764	Missense_Mutation	SNV	G	A	A	FXYD3	c.193G>A	P.E65K
SH_135	19	44934469	44934469	Missense_Mutation	SNV	C	G	G	ZNF229	c.469G>C	P.D157H
SH_135	19	50399165	50399165	Silent	SNV	G	A	A	IL4I1	c.225C>T	P.L75L
SH_135	19	50399300	50399300	Silent	SNV	G	A	A	IL4I1	c.90C>T	P.L30L
SH_135	19	57641791	57641791	Missense_Mutation	SNV	T	G	G	USP29	c.1748T>G	P.L583R
SH_135	2	101624549	101624549	Missense_Mutation	SNV	G	A	A	TBC1D8	c.3157C>T	P.P1053S
SH_135	2	102984428	102984428	Missense_Mutation	SNV	C	A	A	IL18R1	c.202C>A	P.L68M
SH_135	2	107460197	107460197	Silent	SNV	C	T	T	ST6GAL2	c.237G>A	P.A79A
SH_135	2	179597289	179597289	Missense_Mutation	SNV	T	G	G	TTN	c.15548A>C	P.K5183T
SH_135	2	187533560	187533560	Silent	SNV	T	C	C	ITGAV	c.2367T>C	P.N789N
SH_135	2	198363515	198363515	Missense_Mutation	SNV	G	A	A	HSPD1	c.58C>T	P.P20S
SH_135	2	242373687	242373687	Missense_Mutation	SNV	C	T	T	FARP2	c.982C>T	P.P328S
SH_135	2	37455310	37455310	Silent	SNV	T	C	C	CEBPZ	c.1026A>G	P.L342L
SH_135	2	63058148	63058148	Splice_Site	SNV	A	C	C	EHBP1	c.495-6A>C	
SH_135	2	63223821	63223821	Missense_Mutation	SNV	C	A	A	EHBP1	c.3131C>A	P.A1044E
SH_135	2	69771687	69771687	Splice_Site	SNV	G	C	C	AAK1	c.283-11C>G	
SH_135	20	35414983	35414983	Missense_Mutation	SNV	G	A	A	SOGA1	c.4891C>T	P.R1631C
SH_135	20	62366761	62366761	Silent	SNV	G	A	A	ZGPAT	c.1242G>A	P.A414A
SH_135	21	27326987	27326987	Missense_Mutation	SNV	C	A	A	APP	c.1604G>T	P.R535L
SH_135	21	31802902	31802902	Nonsense_Mutation	SNV	C	G	G	KRTAP13-4	c.309C>G	P.Y103*
SH_135	21	31971156	31971156	Missense_Mutation	SNV	T	G	G	KRTAP6-2	c.38A>C	P.H13P
SH_135	21	34954302	34954302	Missense_Mutation	SNV	C	T	T	DONSON	c.1106G>A	P.G369D
SH_135	21	45821606	45821606	Silent	SNV	C	G	G	TRPM2	c.2364C>G	P.A788A
SH_135	21	46066966	46066966	Silent	SNV	C	T	T	KRTAP10-11	c.591C>T	P.V197V
SH_135	22	38897182	38897182	Frame_Shift_Ins	INDEL	-	A	A	DDX17	c.390dupT	P.E131fs
SH_135	22	40661931	40661931	Missense_Mutation	SNV	G	T	T	TNRC6B	c.1697G>T	P.G566V
SH_135	22	46658521	46658521	Silent	SNV	C	G	G	PKDREJ	c.699G>C	P.L233L
SH_135	3	101284835	101284835	Nonstop_Mutation	SNV	T	G	G	TRMT10C	c.1210T>G	P.Ter404Eext*?
SH_135	3	122646768	122646768	Missense_Mutation	SNV	A	G	G	SEMA5B	c.719T>C	P.V240A
SH_135	3	123456371	123456371	Missense_Mutation	SNV	G	A	A	MYLK	c.80C>T	P.P27L
SH_135	3	157820626	157820626	Missense_Mutation	SNV	G	C	C	SHOX2	c.396C>G	P.D132E
SH_135	3	165548484	165548484	Missense_Mutation	SNV	T	G	G	BCHE	c.338A>C	P.N113T
SH_135	3	173998882	173998882	Missense_Mutation	SNV	T	C	C	NLGN1	c.2261T>C	P.L754P

SH_135	3	184119478	184119478	Splice_Site	SNV	G	C	C	LOC105374250	c.-199+5G>C	
SH_135	3	46728593	46728593	Silent	SNV	T	A	A	ALS2CL	c.414A>T	P.S138S
SH_135	3	47307213	47307213	Splice_Site	SNV	C	G	G	KIF9	c.916+7G>C	
SH_135	3	58092555	58092555	Silent	SNV	C	T	T	FLNB	c.1896C>T	P.Y632Y
SH_135	3	63594966	63594966	Missense_Mutation	SNV	G	C	C	SYNPR	c.574G>C	P.V192L
SH_135	3	63824177	63824177	Splice_Site	SNV	T	A	A	THOC7	c.-19-2A>T	
SH_135	3	7456852	7456852	Splice_Site	SNV	T	A	A	GRM7	c.1174+2T>A	
SH_135	3	77638054	77638054	Missense_Mutation	SNV	A	C	C	ROBO2	c.2593A>C	P.K865Q
SH_135	4	118005950	118005950	Silent	SNV	A	T	T	TRAM1L1	c.600T>A	P.I200I
SH_135	4	15539586	15539586	Missense_Mutation	SNV	C	T	T	CC2D2A	c.1829C>T	P.P610L
SH_135	4	26483689	26483689	Silent	SNV	G	A	A	CCKAR	c.858C>T	P.S286S
SH_135	4	68527936	68527936	Missense_Mutation	SNV	T	C	C	UBA6	c.1075A>G	P.T359A
SH_135	4	68703995	68703995	Missense_Mutation	SNV	T	C	C	TMPRSS11D	c.370A>G	P.R124G
SH_135	4	86938433	86938433	Missense_Mutation	SNV	T	A	A	MAPK10	c.1343A>T	P.D448V
SH_135	4	94032012	94032012	Missense_Mutation	SNV	A	T	T	GRID2	c.358A>T	P.M120L
SH_135	5	121786780	121786780	Missense_Mutation	SNV	C	A	A	SNCAIP	c.2238C>A	P.H746Q
SH_135	5	137626366	137626366	Missense_Mutation	SNV	C	T	T	CDC25C	c.826G>A	P.D276N
SH_135	5	1394869	1394869	Missense_Mutation	SNV	C	T	T	SLC6A3	c.1844G>A	P.R615H
SH_135	5	140530319	140530319	Missense_Mutation	SNV	A	G	G	PCDHB6	c.73A>G	P.T25A
SH_135	5	159437658	159437658	Silent	SNV	C	T	T	TTC1	c.123C>T	P.S41S
SH_135	5	38962588	38962588	Missense_Mutation	SNV	T	G	G	RICTOR	c.1667A>C	P.K556T
SH_135	5	5303745	5303751	Frame_Shift_Del	INDEL	CCCTCGG	-	-	ADAMTS16	54_3060delCTC(	P.S1019fs
SH_135	5	53751807	53751807	Missense_Mutation	SNV	C	T	T	HSPB3	c.188C>T	P.P63L
SH_135	5	80409724	80409724	Nonsense_Mutation	SNV	C	T	T	RASGRF2	c.2455C>T	P.R819*
SH_135	6	109767486	109767486	Missense_Mutation	SNV	C	T	T	MICAL1	c.2176G>A	P.E726K
SH_135	6	13327129	13327129	Frame_Shift_Del	INDEL	A	-	-	TBC1D7	c.2delT	P.M1fs
SH_135	6	158900821	158900821	Silent	SNV	G	A	A	TULP4	c.1065G>A	P.S355S
SH_135	6	161032713	161032713	Silent	SNV	G	A	A	LPA	c.2484C>T	P.H828H
SH_135	6	26033440	26033440	Silent	SNV	C	T	T	HIST1H2AB	c.357G>A	P.K119K
SH_135	6	29911975	29911975	Missense_Mutation	SNV	C	G	G	HLA-A	c.696C>G	P.F232L
SH_135	6	32021156	32021156	Splice_Site	SNV	T	C	C	TNXB	c.8785+9A>G	
SH_135	6	69348639	69348639	Silent	SNV	T	G	G	ADGRB3	c.72T>G	P.A24A
SH_135	6	69646425	69646425	Missense_Mutation	SNV	G	C	C	ADGRB3	c.883G>C	P.E295Q
SH_135	6	71186851	71186851	Splice_Site	INDEL	C	-	-	FAM135A	c.369-10delC	
SH_135	6	75892822	75892822	Missense_Mutation	SNV	A	C	C	COL12A1	c.1835T>G	P.L612R
SH_135	6	7845531	7845531	Missense_Mutation	SNV	A	C	C	BMP6	c.823A>C	P.S275R
SH_135	6	83767734	83767734	Nonsense_Mutation	SNV	T	A	A	UBE3D	c.85A>T	P.K29*
SH_135	6	99374622	99374622	Silent	SNV	T	G	G	FBXL4	c.243A>C	P.V81V
SH_135	7	116937823	116937823	Missense_Mutation	SNV	A	C	C	WNT2	c.696T>G	P.D232E
SH_135	7	150068604	150068604	Missense_Mutation	SNV	C	G	G	REPIN1	c.445C>G	P.R149G
SH_135	7	150878359	150878359	Silent	SNV	G	A	A	ASB10	c.771C>T	P.D257D
SH_135	7	27187159	27187159	Silent	SNV	C	T	T	HOXA6	c.210G>A	P.A70A
SH_135	7	36895324	36895324	Silent	SNV	T	C	C	ELMO1	c.576A>G	P.L192L
SH_135	7	4167123	4167123	Missense_Mutation	SNV	T	G	G	SDK1	c.3934T>G	P.Y1312D
SH_135	7	47342638	47342638	Missense_Mutation	SNV	T	A	A	TNS3	c.3367A>T	P.S1123C
SH_135	7	89790632	89790633	Splice_Site	INDEL	GT	-	-	STEAP1	597+1_597+2delGT	
SH_135	7	94039078	94039078	Missense_Mutation	SNV	G	A	A	COL1A2	c.980G>A	P.R327H
SH_135	8	110535458	110535458	Splice_Site	SNV	T	A	A	PKHD1L1	c.12331-4T>A	
SH_135	8	114111149	114111149	Silent	SNV	G	T	T	CSMD3	c.753C>A	P.I251I
SH_135	8	136619211	136619211	Missense_Mutation	SNV	G	A	A	KHDRBS3	c.821G>A	P.G274E
SH_135	8	136619212	136619212	Silent	SNV	A	T	T	KHDRBS3	c.822A>T	P.G274G
SH_135	8	139701164	139701164	Missense_Mutation	SNV	T	G	G	COL22A1	c.2858A>C	P.E953A
SH_135	8	143602256	143602256	Silent	SNV	C	T	T	ADGRB1	c.2994C>T	P.I998I
SH_135	8	88365898	88365898	Missense_Mutation	SNV	T	G	G	CNBD1	c.1187T>G	P.L396R
SH_135	8	92145440	92145440	Silent	SNV	C	T	T	LRRC69	c.486C>T	P.Y162Y
SH_135	9	101824260	101824260	Missense_Mutation	SNV	C	A	A	COL15A1	c.3410C>A	P.A1137E
SH_135	9	113169297	113169297	Silent	SNV	T	C	C	SVEP1	c.8583A>G	P.E2861E
SH_135	9	131255075	131255075	Missense_Mutation	SNV	G	C	C	ODF2	c.1728G>C	P.E576D
SH_135	9	132481528	132481528	Missense_Mutation	SNV	G	A	A	PRRX2	c.278G>A	P.G93E
SH_135	9	136277538	136277538	Missense_Mutation	SNV	C	T	T	REXO4	c.791G>A	P.R264H
SH_135	9	137591754	137591754	Splice_Site	SNV	G	A	A	COL5A1	c.278-1G>A	
SH_135	9	137990304	137990304	Missense_Mutation	SNV	G	C	C	OLFM1	c.629G>C	P.R210T
SH_135	9	140110380	140110380	Missense_Mutation	SNV	C	T	T	NDOR1	c.1465C>T	P.R489W
SH_135	9	140387525	140387525	Missense_Mutation	SNV	G	C	C	PNPLA7	c.2137C>G	P.R713G
SH_135	9	16437085	16437085	Silent	SNV	G	A	A	BNC2	c.981C>T	P.S327S
SH_135	9	17464512	17464512	Missense_Mutation	SNV	G	T	T	CNTLN	c.3422G>T	P.R1141M
SH_135	9	40706066	40706066	Silent	SNV	C	T	T	SPATA31A3	c.3723C>T	P.A1241A
SH_135	9	43625463	43625463	Missense_Mutation	SNV	C	T	T	SPATA31A6	c.3224G>A	P.R1075K
SH_135	9	82333783	82333783	Missense_Mutation	SNV	G	A	A	TLE4	c.1583G>A	P.S528N
SH_135	9	84610048	84610048	Missense_Mutation	SNV	C	G	G	SPATA31D1	c.4663C>G	P.P1555A
SH_135	9	90745831	90745831	Silent	SNV	A	G	G	SPATA31C2	c.2121T>C	P.L707L
SH_135	9	91991854	91991854	Silent	SNV	C	T	T	SEMA4D	c.1776G>A	P.A592A
SH_135	9	93375582	93375582	Silent	SNV	G	A	A	DIRAS2	c.528C>T	P.L176L
SH_135	X	125686088	125686088	Silent	SNV	G	C	C	DCAF12L1	c.504C>G	P.G168G
SH_135	X	140994553	140994553	Silent	SNV	T	C	C	MAGEC1	c.1363T>C	P.L455L
SH_135	X	47044992	47044992	Missense_Mutation	SNV	C	T	T	RBM10	c.2318C>T	P.A773V
SH_135	X	69502174	69502174	Missense_Mutation	SNV	T	G	G	RAB41	c.107T>G	P.F36C
SH_135	X	71495426	71495426	Missense_Mutation	SNV	C	G	G	RPS4X	c.230G>C	P.R77P
SH_137	1	241901790	241901790	Splice_Site	SNV	G	A	A	WDR64	c.1290G>A	P.T430T
SH_137	11	108203492	108203492	Nonsense_Mutation	SNV	C	T	T	ATM	c.7792C>T	P.R2598*
SH_137	11	68029844	68029844	Missense_Mutation	SNV	G	C	C	C11orf24	c.619C>G	P.L207V
SH_137	12	20769211	20769211	Silent	SNV	C	T	T	PDE3A	c.1317C>T	P.S439S
SH_137	12	25398284	25398284	Missense_Mutation	SNV	C	A	A	KRAS	c.35G>T	P.G12V
SH_137	12	5154061	5154061	Missense_Mutation	SNV	C	T	T	KCNA5	c.748C>T	P.R250W
SH_137	13	43500560	43500560	Missense_Mutation	SNV	G	T	T	EPSTI1	c.569C>A	P.T190N
SH_137	15	43571977	43571977	Silent	SNV	C	A	A	TGM7	c.1524G>T	P.L508L
SH_137	16	24166004	24166004	Splice_Site	SNV	G	T	T	PRKCB	c.1066-1G>T	
SH_137	16	66839741	66839741	Splice_Site	SNV	C	T	T	NAE1	c.1428-12G>A	
SH_137	17	38152515	38152515	Missense_Mutation	SNV	A	G	G	PSMD3	c.1400A>G	P.Y467C
SH_137	19	36034295	36034295	Missense_Mutation	SNV	G	T	T	GAPDHS	c.795G>T	P.R265S
SH_137	19	40363914	40363914	Missense_Mutation	SNV	G	A	A	FCGBP	c.14728C>T	P.L4910F
SH_137	20	44048180	44048180	Missense_Mutation	SNV	T	C	C	PIGT	c.463T>C	P.F155L
SH_137	3	170908566	170908575	Frame_Shift_Del	INDEL	TGACTCAGC(	-	-	TNIK	_428delGGCTGA	P.G140fs
SH_137	5	33576343	33576343	Missense_Mutation	SNV	G	A	A	ADAMTS12	c.3533C>T	P.T1178M
SH_137	5	63991419	63991419	Silent	SNV	C	T	T	FAM159B	c.277C>T	P.L93L
SH_137	6	76731925	76731925	Missense_Mutation	SNV	C	T	T	IMPG1	c.340G>A	P.V114I
SH_137	6	83847259	83847259	Silent	SNV	G	A	A	DOPEY1	c.3471G>A	P.V1157V
SH_137	7	557051	557051	Missense_Mutation	SNV	A	T	T	PDGFA	c.140T>A	P.L47H

SH_137	7	74197350	74197350	Missense_Mutation	SNV	G	A	A	NCF1	c.520G>A	P.E174K
SH_137	8	131149206	131149206	Silent	SNV	G	A	A	ASAP1	c.1138C>T	P.L380L
SH_137	9	34658538	34658538	Missense_Mutation	SNV	G	T	T	IL11RA	c.668G>T	P.G223V
SH_137	X	48558900	48558900	Missense_Mutation	SNV	G	T	T	SUV39H1	c.617G>T	P.C206F
SH_137	X	71424900	71424900	Frame_Shift_Ins	INDEL	-	T	T	ERCC6L	:.3716_3717ins/	P.T1240fs
SH_139	1	118461210	118461210	Splice_Site	SNV	T	C	C	GDAP2	c.316+9A>G	
SH_139	1	152282017	152282017	Missense_Mutation	SNV	G	T	T	FLG	c.5345C>A	P.T1782K
SH_139	1	182354621	182354621	Nonsense_Mutation	SNV	A	T	T	GLUL	c.674T>A	P.L225*
SH_139	1	19528290	19528290	Missense_Mutation	SNV	G	A	A	UBR4	c.196C>T	P.H66Y
SH_139	12	106708150	106708150	Silent	SNV	A	C	C	TCP11L2	c.172A>C	P.R58R
SH_139	12	52946471	52946471	Nonsense_Mutation	SNV	G	A	A	KRT71	c.391C>T	P.R131*
SH_139	13	95114363	95114363	Missense_Mutation	SNV	T	C	C	DCT	c.944A>G	P.N315S
SH_139	15	59064273	59064273	Missense_Mutation	SNV	G	T	T	FAM63B	c.679G>T	P.A227S
SH_139	15	99482579	99482579	Silent	SNV	C	A	A	IGF1R	c.3447C>A	P.V1149V
SH_139	16	86601038	86601038	Missense_Mutation	SNV	G	A	A	FOXC2	c.97G>A	P.G33S
SH_139	19	30020720	30020720	Splice_Site	SNV	T	C	C	VSTM2B	c.298-6T>C	
SH_139	19	42461207	42461207	Nonsense_Mutation	SNV	C	T	T	RABAC1	c.432G>A	P.W144*
SH_139	2	137814268	137814268	Missense_Mutation	SNV	C	A	A	THSD7B	c.418C>A	P.H140N
SH_139	2	207804397	207804397	Splice_Site	SNV	T	C	C	CPO	c.68+6T>C	
SH_139	3	100287677	100287677	Silent	SNV	C	T	T	TMEM45A	c.600C>T	P.V200V
SH_139	5	149669160	149669160	Missense_Mutation	SNV	G	A	A	CAMK2A	c.29C>T	P.T10M
SH_139	5	150506042	150506042	Splice_Site	SNV	C	A	A	ANXA6	c.978-1G>T	
SH_139	6	112015613	112015613	Missense_Mutation	SNV	C	T	T	FYN	c.1238G>A	P.R413Q
SH_139	6	12136101	12136101	Missense_Mutation	SNV	G	C	C	HIVEP1	c.6463G>C	P.D2155H
SH_139	7	129929507	129929507	Missense_Mutation	SNV	C	T	T	CPA2	c.1180C>T	P.R394C
SH_139	7	81593617	81593617	Nonsense_Mutation	SNV	G	T	T	CACNA2D1	c.2633C>A	P.S878*
SH_139	8	114388963	114388963	Splice_Site	SNV	C	A	A	CSMD3	c.58+4G>T	
SH_139	8	17043889	17043889	Silent	SNV	A	G	G	ZDHHC2	c.207A>G	P.S69S
SH_139	9	34724008	34724008	Missense_Mutation	SNV	G	A	A	FAM205A	c.3229C>T	P.R1077C
SH_139	X	47466554	47466554	Missense_Mutation	SNV	T	C	C	SYN1	c.421A>G	P.I141V
SH_139	X	69282394	69282394	Missense_Mutation	SNV	A	G	G	OTUD6A	c.20A>G	P.E7G
SH_140	1	12321179	12321179	Missense_Mutation	SNV	G	A	A	VPS13D	c.1387G>A	P.E463K
SH_140	1	152195670	152195670	Silent	SNV	G	C	C	HRNR	c.60C>G	P.T20T
SH_140	1	172411244	172411244	Silent	SNV	T	C	C	PIGC	c.519A>G	P.V173V
SH_140	1	184679567	184679567	Silent	SNV	G	A	A	EDEM3	c.1806C>T	P.L602L
SH_140	1	202992017	202992017	Missense_Mutation	SNV	A	T	T	TMEM183A	c.980A>T	P.H327L
SH_140	1	203194885	203194885	Missense_Mutation	SNV	C	T	T	CHIT1	c.169G>A	P.A57T
SH_140	1	232002303	232002303	Missense_Mutation	SNV	G	T	T	DISC1	c.2016G>T	P.Q672H
SH_140	1	36212367	36212367	Missense_Mutation	SNV	C	G	G	CLSPN	c.2662G>C	P.D888H
SH_140	1	74929218	74929218	Missense_Mutation	SNV	G	T	T	FPGT-TNNI3K	c.2447G>T	P.W816L
SH_140	1	74929219	74929219	Nonsense_Mutation	SNV	G	A	A	FPGT-TNNI3K	c.2448G>A	P.W816*
SH_140	10	105903325	105903325	Missense_Mutation	SNV	C	A	A	CFAP43	c.4017G>T	P.K1339N
SH_140	10	13702476	13702476	Missense_Mutation	SNV	T	C	C	FRMD4A	c.1786A>G	P.N596D
SH_140	10	25885616	25885616	Missense_Mutation	SNV	A	T	T	GPR158	c.2043A>T	P.E681D
SH_140	10	32580127	32580142	Frame_Shift_Del	INDEL	GTGTTTAAAT	-	-	EPC1	9delTCAATTTAA	P.S308fs
SH_140	10	47086937	47086937	Missense_Mutation	SNV	A	G	G	NPY4R	c.154A>G	P.T52A
SH_140	10	51866195	51866195	Silent	SNV	A	G	G	FAM21A	c.1839A>G	P.A613A
SH_140	11	122929771	122929771	Missense_Mutation	SNV	A	G	G	HSPA8	c.1319T>C	P.I440T
SH_140	11	133814242	133814242	Missense_Mutation	SNV	A	T	T	IGSF9B	c.282T>A	P.D94E
SH_140	11	4080616	4080616	Silent	SNV	G	C	C	STIM1	c.603G>C	P.G201G
SH_140	11	56345101	56345101	Missense_Mutation	SNV	C	A	A	OR5M10	c.97G>T	P.A33S
SH_140	11	56345102	56345102	Silent	SNV	C	T	T	OR5M10	c.96G>A	P.L32L
SH_140	11	57995536	57995536	Missense_Mutation	SNV	G	T	T	OR10Q1	c.812C>A	P.T271N
SH_140	11	62290722	62290722	Missense_Mutation	SNV	C	G	G	AHNAK	c.11167G>C	P.G3723R
SH_140	12	132393680	132393680	Splice_Site	SNV	G	T	T	ULK1	c.565-1G>T	
SH_140	12	29502037	29502037	Silent	SNV	A	G	G	ERGIC2	c.702T>C	P.D234D
SH_140	12	30877346	30877346	Missense_Mutation	SNV	G	C	C	CAPRIN2	c.1945C>G	P.P649A
SH_140	12	52567428	52567428	Missense_Mutation	SNV	C	T	T	KRT80	c.787G>A	P.A263T
SH_140	12	58014112	58014112	Missense_Mutation	SNV	A	G	G	SLC26A10	c.109A>G	P.T37A
SH_140	12	6730336	6730336	Missense_Mutation	SNV	C	G	G	LPAR5	c.79G>C	P.V27L
SH_140	13	103390153	103390153	Silent	SNV	A	T	T	CCDC168	c.12894T>A	P.P4298P
SH_140	13	19751595	19751595	Missense_Mutation	SNV	C	A	A	TUBA3C	c.528G>T	P.Q176H
SH_140	13	32954039	32954039	Nonsense_Mutation	SNV	C	T	T	BRCA2	c.9106C>T	P.Q3036*
SH_140	13	48942667	48942667	Nonsense_Mutation	SNV	G	T	T	RB1	c.1054G>T	P.E352*
SH_140	14	37641547	37641547	Silent	SNV	G	T	T	SLC25A21	c.9C>A	P.A3A
SH_140	15	88690602	88690602	Nonsense_Mutation	SNV	G	T	T	NTRK3	c.428C>A	P.S143*
SH_140	15	94841562	94841562	Nonsense_Mutation	SNV	T	A	A	MCTP2	c.68T>A	P.L23*
SH_140	16	10864187	10864187	Missense_Mutation	SNV	G	T	T	TVP23A	c.584C>A	P.A195D
SH_140	16	4908007	4908007	Missense_Mutation	SNV	A	G	G	UBN1	c.266A>G	P.D89G
SH_140	16	82891983	82891983	Missense_Mutation	SNV	C	T	T	CDH13	c.203C>T	P.S68F
SH_140	16	85699957	85699957	Splice_Site	SNV	A	G	G	GSE1	c.2818+4A>G	
SH_140	16	88691630	88691630	Missense_Mutation	SNV	G	A	A	ZC3H18	c.2135G>A	P.G712D
SH_140	16	90025448	90025448	Silent	SNV	C	A	A	DEF8	c.369C>A	P.I123I
SH_140	17	30376157	30376157	Missense_Mutation	SNV	G	T	T	LRRC37B	c.2174G>T	P.R725L
SH_140	17	41566881	41566881	Frame_Shift_Ins	INDEL	-	A	A	DHX8	c.218dupA	P.N73fs
SH_140	17	55062796	55062796	Missense_Mutation	SNV	G	T	T	SCPEP1	c.283G>T	P.D95Y
SH_140	17	56572598	56572598	Missense_Mutation	SNV	T	G	G	MTMR4	c.2905A>C	P.N969H
SH_140	17	7579311	7579311	Splice_Site	SNV	C	A	A	TP53	c.375+1G>T	
SH_140	19	10107105	10107105	Splice_Site	SNV	T	A	A	COL5A3	c.1422+4A>T	
SH_140	19	11553282	11553282	Missense_Mutation	SNV	G	C	C	PRKCSH	c.550G>C	P.E184Q
SH_140	19	18896845	18896845	Silent	SNV	G	A	A	COMP	c.1419C>T	P.D473D
SH_140	19	41105308	41105308	Missense_Mutation	SNV	T	A	A	LTBP4	c.76T>A	P.S26T
SH_140	19	43268270	43268270	Nonsense_Mutation	SNV	G	T	T	PSG8	c.228C>A	P.Y76*
SH_140	19	46886706	46886706	Missense_Mutation	SNV	C	A	A	PPP5C	c.672C>A	P.S224R
SH_140	19	49657228	49657228	Missense_Mutation	SNV	G	A	A	HRC	c.1267C>T	P.P423S
SH_140	19	51022639	51022639	Missense_Mutation	SNV	G	T	T	LRRC4B	c.331C>A	P.H111N
SH_140	19	54632428	54632428	Splice_Site	SNV	C	G	G	PRPF31	c.1147-4C>G	
SH_140	19	54746415	54746415	Splice_Site	SNV	G	C	C	LILRA6	c.33-7C>G	
SH_140	19	57334150	57334150	Missense_Mutation	SNV	C	T	T	PEG3	c.536G>A	P.R179H
SH_140	19	806448	806448	Silent	SNV	G	A	A	PTBP1	c.1011G>A	P.L337L
SH_140	19	8435960	8435960	Missense_Mutation	SNV	C	G	G	ANGPTL4	c.568C>G	P.Q190E
SH_140	2	10008515	10008515	Missense_Mutation	SNV	C	G	G	TAF1B	c.510C>G	P.I170M
SH_140	2	141762979	141762979	Silent	SNV	A	G	G	LRP1B	c.2428T>C	P.L810L
SH_140	2	153533164	153533164	Silent	SNV	T	C	C	PRPF40A	c.786A>G	P.A262A
SH_140	2	161052067	161052067	Missense_Mutation	SNV	A	G	G	ITGB6	c.406T>C	P.Y136H
SH_140	2	220480797	220480797	Missense_Mutation	SNV	G	T	T	STK11IP	c.3149G>T	P.R1050L
SH_140	20	31954677	31954677	Silent	SNV	C	T	T	CDK5RAP1	c.1527G>A	P.V509V
SH_140	20	43251494	43251494	Silent	SNV	C	T	T	ADA	c.756G>A	P.L252L

SH_140	20	60888859	60888859	Splice_Site	SNV	C	A	A	LAMA5	c.8505-1G>T	
SH_140	20	60888861	60888861	Splice_Site	SNV	G	A	A	LAMA5	c.8505-3C>T	
SH_140	21	15537680	15537680	Missense_Mutation	SNV	T	A	A	LIPI	c.765A>T	P.R255S
SH_140	21	16337081	16337081	Nonsense_Mutation	SNV	C	A	A	NRIP1	c.3433G>T	P.G1145*
SH_140	22	30975186	30975186	Missense_Mutation	SNV	C	A	A	PES1	c.1444G>T	P.G482C
SH_140	22	50967557	50967557	Splice_Site	SNV	T	C	C	TYMP	c.417+8A>G	
SH_140	3	148563264	148563264	Missense_Mutation	SNV	G	A	A	CPB1	c.832G>A	P.E278K
SH_140	3	32030675	32030675	Missense_Mutation	SNV	T	G	G	ZNF860	c.104T>G	P.L35W
SH_140	3	73433915	73433915	Missense_Mutation	SNV	G	T	T	PDZRN3	c.896C>A	P.A299E
SH_140	3	73433916	73433916	Missense_Mutation	SNV	C	T	T	PDZRN3	c.895G>A	P.A299T
SH_140	4	155241686	155241686	Frame_Shift_Del	INDEL	G	-	-	DCHS2	c.3500delC	P.S1167fs
SH_140	4	25146671	25146671	Missense_Mutation	SNV	C	A	A	SEPSECS	c.889G>T	P.G297C
SH_140	5	150897269	150897269	Missense_Mutation	SNV	C	T	T	FAT2	c.11375G>A	P.R3792Q
SH_140	5	37169448	37169448	Silent	SNV	A	C	C	C5orf42	c.6678T>G	P.P2226P
SH_140	6	155749923	155749923	Splice_Site	SNV	C	T	T	NOX3	c.1145+5G>A	
SH_140	6	31608924	31608924	Missense_Mutation	SNV	C	T	T	BAG6	c.2636G>A	P.G879E
SH_140	6	41303637	41303637	Missense_Mutation	SNV	C	A	A	NCR2	c.23C>A	P.P8Q
SH_140	6	43415495	43415495	Missense_Mutation	SNV	C	T	T	ABCC10	c.3779C>T	P.P1260L
SH_140	6	619531	619531	Silent	SNV	C	A	A	EXOC2	c.435G>T	P.S145S
SH_140	7	112423847	112423847	Missense_Mutation	SNV	G	T	T	TMEM168	c.1034C>A	P.A345E
SH_140	7	131870125	131870125	Missense_Mutation	SNV	C	G	G	PLXNA4	c.3091G>C	P.D1031H
SH_140	7	134223038	134223038	Splice_Site	SNV	C	A	A	AKR1B10	c.825+9C>A	
SH_140	7	134223039	134223039	Splice_Site	SNV	C	T	T	AKR1B10	c.825+10C>T	
SH_140	7	35288451	35288451	Missense_Mutation	SNV	C	A	A	TBX20	c.383G>T	P.R128M
SH_140	7	4249721	4249721	Missense_Mutation	SNV	G	T	T	SDK1	c.927G>T	P.W309C
SH_140	7	48564012	48564012	Silent	SNV	T	A	A	ABCA13	c.14220T>A	P.I4740I
SH_140	8	8176074	8176074	Missense_Mutation	SNV	C	A	A	SGK223	c.3811G>T	P.A1271S
SH_140	8	97614760	97614760	Splice_Site	SNV	C	T	T	SDC2	c.306+4C>T	
SH_140	9	101156534	101156534	Missense_Mutation	SNV	C	T	T	GABBR2	c.1301G>A	P.S434N
SH_140	9	101784396	101784396	Silent	SNV	T	A	A	COL15A1	c.1725T>A	P.P575P
SH_140	9	104312898	104312898	Missense_Mutation	SNV	G	T	T	RNF20	c.1103G>T	P.R368L
SH_140	9	130495572	130495572	Missense_Mutation	SNV	C	G	G	TOR2A	c.685G>C	P.V229L
SH_140	9	35077350	35077350	Missense_Mutation	SNV	G	A	A	FANCG	c.557C>T	P.P186L
SH_140	X	109693883	109693883	Missense_Mutation	SNV	C	A	A	RGAG1	c.38C>A	P.T13K
SH_140	X	135593723	135593827	Missense_Mutation	INDEL	GATTCAGAT	-	-	HTATSF1	c.1824_1928del	P.E608_S642del
SH_140	X	151900607	151900607	Missense_Mutation	SNV	G	T	T	MAGEA12	c.194C>A	P.P65H
SH_140	X	2945349	2945349	Splice_Site	SNV	T	G	G	ARSH	c.1037-5T>G	
SH_140	X	31645909	31645909	Nonsense_Mutation	SNV	T	A	A	DMD	c.8074A>T	P.K2692*
SH_140	X	43655116	43655116	Missense_Mutation	SNV	C	A	A	MAOB	c.638G>T	P.G213V
SH_140	X	48341392	48341392	Missense_Mutation	SNV	A	T	T	FTSJ1	c.565A>T	P.S189C
SH_140	X	83129053	83129053	Missense_Mutation	SNV	C	A	A	CYLC1	c.1337C>A	P.A446D
SH_141	1	104120437	104120437	Missense_Mutation	SNV	G	C	C	AMY2B	c.1316G>C	P.R439T
SH_141	1	10431286	10431286	Missense_Mutation	SNV	C	G	G	KIF1B	c.4774C>G	P.L1592V
SH_141	1	114340440	114340440	Missense_Mutation	SNV	A	C	C	RSBN1	c.922T>G	P.S308A
SH_141	1	169828347	169828347	Silent	SNV	G	A	A	SCYL3	c.1147C>T	P.L383L
SH_141	1	169828348	169828348	Missense_Mutation	SNV	C	A	A	SCYL3	c.1146G>T	P.L382F
SH_141	1	17350557	17350557	Nonsense_Mutation	SNV	C	A	A	SDHB	c.553G>T	P.E185*
SH_141	1	202278196	202278196	Missense_Mutation	SNV	A	C	C	LGR6	c.1298A>C	P.Q433P
SH_141	1	2130383	2130383	Silent	SNV	C	T	T	FAAP20	c.45G>A	P.A15A
SH_141	1	26212309	26212309	Nonsense_Mutation	SNV	C	A	A	STMN1	c.430G>T	P.E144*
SH_141	1	26629322	26629322	Missense_Mutation	SNV	G	A	A	UBXN11	c.53C>T	P.S18L
SH_141	1	38034653	38034653	Missense_Mutation	SNV	T	G	G	GNL2	c.1871A>C	P.E624A
SH_141	1	38341323	38341323	Missense_Mutation	SNV	C	G	G	INPP5B	c.1251G>C	P.K417N
SH_141	1	95609561	95609561	Missense_Mutation	SNV	C	T	T	TMEM56	c.104C>T	P.S35F
SH_141	1	981124	981124	Missense_Mutation	SNV	G	T	T	AGRN	c.2548G>T	P.D850Y
SH_141	10	135020810	135020810	Missense_Mutation	SNV	T	A	A	KNDC1	c.3749T>A	P.L1250Q
SH_141	10	16806507	16806507	Missense_Mutation	SNV	C	T	T	RSU1	c.163G>A	P.V55M
SH_141	10	38121463	38121463	Missense_Mutation	SNV	T	C	C	ZNF248	c.820A>G	P.I274V
SH_141	10	73434879	73434879	Missense_Mutation	SNV	A	G	G	CDH23	c.1460A>G	P.N487S
SH_141	10	75673516	75673516	Missense_Mutation	SNV	T	A	A	PLAU	c.629T>A	P.I210N
SH_141	10	79785496	79785496	Missense_Mutation	SNV	G	T	T	POLR3A	c.202C>A	P.P68T
SH_141	11	125542520	125542520	Missense_Mutation	SNV	A	C	C	ACRV1	c.766T>G	P.C256G
SH_141	11	128360402	128360402	Missense_Mutation	SNV	G	A	A	ETS1	c.284C>T	P.A95V
SH_141	11	17333656	17333656	Missense_Mutation	SNV	G	A	A	NUCB2	c.901G>A	P.V301I
SH_141	11	56409209	56409209	Missense_Mutation	SNV	G	A	A	OR5AP2	c.707C>T	P.S236L
SH_141	11	58190258	58190258	Silent	SNV	C	G	G	OR5B2	c.477G>C	P.G159G
SH_141	12	100453202	100453202	Missense_Mutation	SNV	C	A	A	UHRF1BP1L	c.1853G>T	P.C618F
SH_141	12	100691850	100691850	Missense_Mutation	SNV	T	C	C	SCYL2	c.377T>C	P.L126S
SH_141	12	11463289	11463289	Nonsense_Mutation	SNV	G	C	C	PRB4	c.44C>G	P.S15*
SH_141	12	122657079	122657079	Silent	SNV	A	C	C	IL31	c.375T>G	P.S125S
SH_141	12	133635293	133635293	Silent	SNV	C	T	T	ZNF84	c.1992C>T	P.F664F
SH_141	12	51453144	51453144	Missense_Mutation	SNV	A	T	T	LETMD1	c.1052A>T	P.E351V
SH_141	12	56384564	56384564	Silent	SNV	C	T	T	RAB5B	c.414C>T	P.A138A
SH_141	12	70352246	70352246	Missense_Mutation	SNV	G	A	A	MYRFL	c.2668G>A	P.D890N
SH_141	13	23928670	23928670	Missense_Mutation	SNV	G	A	A	SACS	c.1640C>T	P.A547V
SH_141	13	39265120	39265120	Silent	SNV	C	G	G	FREM2	c.3639C>G	P.L1213L
SH_141	14	90450886	90450886	Missense_Mutation	SNV	G	T	T	TDP1	c.911G>T	P.R304L
SH_141	14	95570125	95570125	Missense_Mutation	SNV	T	C	C	DICER1	c.3608A>G	P.N1203S
SH_141	15	65450936	65450936	Nonsense_Mutation	SNV	A	T	T	CLPX	c.869T>A	P.L290*
SH_141	15	74032879	74032879	Silent	SNV	C	A	A	C15orf59	c.261G>T	P.A87A
SH_141	16	11076740	11076740	Silent	SNV	C	T	T	CLEC16A	c.957C>T	P.F319F
SH_141	16	29828302	29828302	Silent	SNV	G	C	C	PAGR1	c.456G>C	P.P152P
SH_141	16	30748790	30748790	Missense_Mutation	SNV	C	G	G	SRCAP	c.7429C>G	P.L2477V
SH_141	16	72110310	72110310	Missense_Mutation	SNV	A	T	T	HPR	c.377A>T	P.N126I
SH_141	16	89293624	89293624	Missense_Mutation	SNV	G	A	A	ZNF778	c.928G>A	P.E310K
SH_141	16	89349639	89349639	Missense_Mutation	SNV	T	C	C	ANKRD11	c.3311A>G	P.D1104G
SH_141	17	37604108	37604108	Silent	SNV	T	C	C	MED1	c.75A>G	P.A25A
SH_141	17	39186251	39186251	Missense_Mutation	SNV	G	T	T	KRTAP1-4	c.80C>A	P.P27Q
SH_141	17	61958860	61958860	Silent	SNV	G	A	A	GH2	c.30C>T	P.L10L
SH_141	17	72939718	72939718	Missense_Mutation	SNV	C	A	A	OTOP3	c.650C>A	P.T217N
SH_141	17	7577534	7577534	Missense_Mutation	SNV	C	A	A	TP53	c.747G>T	P.R249S
SH_141	18	3534337	3534337	Missense_Mutation	SNV	T	G	G	DLGAP1	c.1428A>C	P.E476D
SH_141	18	5406824	5406824	Silent	SNV	G	A	A	EPB41L3	c.1794C>T	P.A598A
SH_141	19	1220391	1220391	Missense_Mutation	SNV	G	T	T	STK11	c.484G>T	P.D162Y
SH_141	19	38989882	38989882	Splice_Site	SNV	C	T	T	RYR1	c.7026C>T	P.N2342N
SH_141	19	4013176	4013176	Missense_Mutation	SNV	G	T	T	PIAS4	c.283G>T	P.A95S
SH_141	19	48945409	48945409	Missense_Mutation	SNV	G	A	A	GRIN2D	c.2443G>A	P.E815K
SH_141	19	54314454	54314454	Silent	SNV	G	A	A	NLRP12	c.459C>T	P.L153L

SH_141	19	9049125	9049125	Missense_Mutation	SNV	G	T	T	MUC16	c.32506C>A	P.P10836T
SH_141	19	9325136	9325136	Silent	SNV	G	A	A	OR7D4	c.378C>T	P.I126I
SH_141	2	178482221	178482221	Silent	SNV	G	A	A	TTC30A	c.1209C>T	P.N403N
SH_141	2	179396768	179396768	Missense_Mutation	SNV	G	C	C	TTN	c.99651C>G	P.I33217M
SH_141	2	203914526	203914526	Splice_Site	SNV	T	C	C	NBEAL1	c.144-11T>C	
SH_141	2	230875533	230875533	Missense_Mutation	SNV	T	A	A	FBXO36	c.500T>A	P.L167H
SH_141	2	231264962	231264962	Splice_Site	SNV	G	T	T	SP140L	c.1238+5G>T	
SH_141	2	28828736	28828736	Missense_Mutation	SNV	C	T	T	PLB1	c.2945C>T	P.T982M
SH_141	2	74184338	74184338	Missense_Mutation	SNV	C	G	G	DGUOK	c.369C>G	P.H123Q
SH_141	20	1223449	1223449	Missense_Mutation	SNV	C	T	T	RAD21L1	c.1043C>T	P.S348L
SH_141	20	23731467	23731467	Missense_Mutation	SNV	C	T	T	CST1	c.37G>A	P.A13T
SH_141	20	25656194	25656194	Missense_Mutation	SNV	C	T	T	ZNF337	c.1730G>A	P.C577Y
SH_141	20	39794955	39794955	Missense_Mutation	SNV	C	A	A	PLCG1	c.1921C>A	P.Q641K
SH_141	20	44531175	44531175	Silent	SNV	G	A	A	PLTP	c.726C>T	P.I242I
SH_141	21	17246830	17246830	Silent	SNV	A	G	G	USP25	c.2994A>G	P.R998R
SH_141	22	17601380	17601380	Missense_Mutation	SNV	C	A	A	CECR6	c.638G>T	P.G213V
SH_141	22	32545746	32545746	Missense_Mutation	SNV	A	T	T	C22orf42	c.676T>A	P.Y226N
SH_141	3	189612142	189612142	Missense_Mutation	SNV	G	A	A	TP63	c.1894G>A	P.E632K
SH_141	3	53707056	53707056	Missense_Mutation	SNV	G	A	A	CACNA1D	c.1123G>A	P.D375N
SH_141	4	106638858	106638858	Missense_Mutation	SNV	C	T	T	GSTCD	c.88C>T	P.H30Y
SH_141	4	20543217	20543217	Silent	SNV	C	T	T	SLIT2	c.2118C>T	P.A706A
SH_141	5	140730577	140730577	Silent	SNV	A	G	G	PCDHGB1	c.750A>G	P.E250E
SH_141	5	141243441	141243441	Missense_Mutation	SNV	G	A	A	PCDH1	c.2503C>T	P.R835C
SH_141	5	41018003	41018003	Missense_Mutation	SNV	T	A	A	MROH2B	c.2833A>T	P.T945S
SH_141	6	100965917	100965917	Silent	SNV	A	C	C	ASCC3	c.5877T>G	P.S1959S
SH_141	6	165832122	165832122	Splice_Site	SNV	C	T	T	PDE10A	c.998+1G>A	
SH_141	6	36076212	36076212	Missense_Mutation	SNV	G	C	C	MAPK14	c.1071G>C	P.E357D
SH_141	6	90397840	90397840	Missense_Mutation	SNV	G	A	A	MDN1	c.11312C>T	P.S3771L
SH_141	7	102604079	102604079	Missense_Mutation	SNV	C	G	G	FBXL13	c.625G>C	P.D209H
SH_141	7	140082295	140082295	Missense_Mutation	SNV	G	A	A	SLC37A3	c.32C>T	P.S11F
SH_141	7	146825878	146825878	Missense_Mutation	SNV	G	T	T	CNTNAP2	c.1033G>T	P.V345F
SH_141	7	30825394	30825394	Missense_Mutation	SNV	G	A	A	FAM188B	c.449G>A	P.G150D
SH_141	7	32582833	32582833	Silent	SNV	C	T	T	AVL9	c.174C>T	P.P58P
SH_141	8	11189627	11189627	Nonsense_Mutation	SNV	G	T	T	SLC35G5	c.1012G>T	P.E338*
SH_141	8	113323333	113323333	Missense_Mutation	SNV	G	T	T	CSMD3	c.7447C>A	P.Q2483K
SH_141	8	24192998	24192998	Missense_Mutation	SNV	G	A	A	ADAM28	c.1411G>A	P.E471K
SH_141	8	48767815	48767815	Silent	SNV	C	T	T	PRKDC	c.6726G>A	P.E2242E
SH_141	9	33548311	33548311	Nonsense_Mutation	SNV	G	T	T	ANKRD18B	c.1336G>T	P.E446*
SH_141	9	86530464	86530464	Silent	SNV	G	A	A	KIF27	c.43C>T	P.L15L
SH_141	9	96019387	96019387	Missense_Mutation	SNV	C	T	T	WNK2	c.2348C>T	P.A783V
SH_141	X	154009536	154009536	Missense_Mutation	SNV	G	A	A	MPP1	c.1151C>T	P.A384V
SH_142	1	152328545	152328545	Missense_Mutation	SNV	C	T	T	FLG2	c.1717G>A	P.G573S
SH_142	1	160128882	160128882	Nonsense_Mutation	SNV	C	T	T	ATP1A4	c.616C>T	P.R206*
SH_142	1	169521867	169521867	Silent	SNV	T	A	A	F5	c.1224A>T	P.T408T
SH_142	11	1015833	1015833	Missense_Mutation	SNV	A	T	T	MUC6	c.6968T>A	P.L2323H
SH_142	11	60511334	60511334	Missense_Mutation	SNV	A	T	T	MS4A18	c.950A>T	P.N317I
SH_142	13	46549875	46549875	Missense_Mutation	SNV	T	A	A	ZC3H13	c.2011A>T	P.R671W
SH_142	16	88889111	88889111	Missense_Mutation	SNV	T	A	A	GALNS	c.1250A>T	P.D417V
SH_142	17	40066498	40066498	Nonsense_Mutation	SNV	C	A	A	ACLY	c.322G>T	P.E108*
SH_142	17	7577534	7577534	Missense_Mutation	SNV	C	A	A	TP53	c.747G>T	P.R249S
SH_142	18	12840737	12840737	Missense_Mutation	SNV	T	C	C	PTPN2	c.49A>G	P.M17V
SH_142	19	51381807	51381807	Missense_Mutation	SNV	A	G	G	KLK2	c.778A>G	P.N260D
SH_142	19	53855810	53855810	Missense_Mutation	SNV	A	T	T	ZNF845	c.1882A>T	P.T628S
SH_142	19	55495073	55495073	Silent	SNV	T	A	A	NLRP2	c.2007T>A	P.S669S
SH_142	2	152584345	152584345	Missense_Mutation	SNV	G	C	C	NEB	c.154C>G	P.L52V
SH_142	2	95818945	95818945	Silent	SNV	C	T	T	ZNF514	c.273G>A	P.L91L
SH_142	21	47981673	47981673	Silent	SNV	A	T	T	DIP2A	c.4032A>T	P.V1344V
SH_142	22	30064329	30064329	Missense_Mutation	SNV	A	T	T	NF2	c.893A>T	P.Q298L
SH_142	5	160042933	160042933	Missense_Mutation	SNV	G	T	T	ATP10B	c.2565C>A	P.F855L
SH_142	6	30864789	30864789	Splice_Site	SNV	A	T	T	DDR1	c.1759-2A>T	
SH_142	7	98460938	98460938	Missense_Mutation	SNV	G	T	T	TMEM130	c.171C>A	P.D57E
SH_142	7	98581022	98581022	Missense_Mutation	SNV	G	A	A	TRRAP	c.8941G>A	P.V2981M
SH_142	7	99757853	99757853	Missense_Mutation	SNV	C	T	T	GAL3ST4	c.1159G>A	P.G387S
SH_142	9	116050472	116050472	Missense_Mutation	SNV	A	T	T	PRPF4	c.950A>T	P.D317V
SH_143	1	158517848	158517848	Silent	SNV	A	G	G	OR6Y1	c.48T>C	P.R16R
SH_143	1	158906853	158906853	Missense_Mutation	SNV	G	C	C	PYHIN1	c.153G>C	P.L51F
SH_143	1	168054847	168054847	Silent	SNV	G	A	A	GPR161	c.1572C>T	P.G524G
SH_143	11	123676773	123676773	Silent	SNV	G	A	A	OR6M1	c.285C>T	P.C95C
SH_143	11	64390360	64390377	Missense_Mutation	INDEL	GACGGCCCC	-	-	NRXN2	delGGGGAGGG	G1341_V1346del
SH_143	11	65810698	65810698	Silent	SNV	G	A	A	GAL3ST3	c.576C>T	P.R192R
SH_143	11	66114676	66114676	Missense_Mutation	SNV	T	A	A	B4GAT1	c.341A>T	P.E114V
SH_143	12	103696114	103696114	Silent	SNV	G	T	T	C12orf42	c.855C>A	P.L285L
SH_143	12	12871186	12871186	Nonsense_Mutation	SNV	C	A	A	CDKN1B	c.413C>A	P.S138*
SH_143	12	77236716	77236716	Silent	SNV	A	G	G	ZDHHC17	c.1284A>G	P.A428A
SH_143	13	26620769	26620769	Missense_Mutation	SNV	A	G	G	SHISA2	c.770T>C	P.V257A
SH_143	15	102358648	102358648	Missense_Mutation	SNV	A	G	G	OR4F15	c.259A>G	P.K87E
SH_143	15	22382718	22382718	Silent	SNV	G	A	A	OR4N4	c.246G>A	P.L82L
SH_143	15	23812278	23812278	Missense_Mutation	SNV	G	T	T	MKRN3	c.1349G>T	P.R450L
SH_143	15	41554970	41554970	Missense_Mutation	SNV	T	G	G	CHP1	c.238T>G	P.F80V
SH_143	15	43495413	43495413	Missense_Mutation	SNV	G	A	A	EPB42	c.1769C>T	P.T590I
SH_143	16	1258196	1258196	Missense_Mutation	SNV	C	T	T	CACNA1H	c.3338C>T	P.P1113L
SH_143	16	3339800	3339817	Missense_Mutation	INDEL	CCCACAAGT	-	-	ZNF263	delGGCCCACAA	P.A433_E438del
SH_143	17	56247732	56247732	Missense_Mutation	SNV	C	T	T	OR4D2	c.716C>T	P.T239I
SH_143	17	72287244	72287244	Silent	SNV	C	G	G	DNAI2	c.696C>G	P.L232L
SH_143	17	72576200	72576200	Missense_Mutation	SNV	G	C	C	CD300LD	c.526C>G	P.L176V
SH_143	17	7578530	7578530	Missense_Mutation	SNV	A	G	G	TP53	c.400T>C	P.F134L
SH_143	18	47500762	47500762	Missense_Mutation	SNV	G	A	A	MYO5B	c.1280C>T	P.S427F
SH_143	2	131976469	131976469	Missense_Mutation	SNV	A	G	G	POTEE	c.494A>G	P.D165G
SH_143	2	75280721	75280721	Splice_Site	SNV	C	T	T	TACR1	c.735+11G>A	
SH_143	2	86255777	86255777	Missense_Mutation	SNV	C	T	T	POLR1A	c.4837G>A	P.G1613S
SH_143	20	20144789	20144789	Silent	SNV	C	T	T	CFAP61	c.1122C>T	P.P374P
SH_143	21	39817354	39817354	Missense_Mutation	SNV	C	A	A	ERG	c.230G>T	P.C77F
SH_143	21	43959744	43959744	Missense_Mutation	SNV	A	C	C	SLC37A1	c.473A>C	P.Y158S
SH_143	22	18171920	18171920	Splice_Site	SNV	T	A	A	BCL2L13	c.458+12T>A	
SH_143	3	148727077	148727077	Missense_Mutation	SNV	A	G	G	GYG1	c.496A>G	P.I166V
SH_143	3	158520026	158520026	Missense_Mutation	SNV	G	T	T	MFSD1	c.232G>T	P.A78S
SH_143	3	38138125	38138125	Missense_Mutation	SNV	A	C	C	DLEC1	c.2237A>C	P.E746A
SH_143	3	42742268	42742268	Silent	SNV	C	G	G	HHATL	c.51G>C	P.L17L

SH_143	3	89521634	89521634	Missense_Mutation	SNV	A	G	G	EPHA3	c.2711A>G	P.D904G
SH_143	4	5633724	5633724	Silent	SNV	G	A	A	EVC2	c.1266C>T	P.L422L
SH_143	5	140720334	140720334	Missense_Mutation	SNV	G	T	T	PCDHGA2	c.1796G>T	P.G599V
SH_143	5	71490897	71490897	Missense_Mutation	SNV	A	G	G	MAP1B	c.1337A>G	P.N446S
SH_143	5	7707830	7707830	Frame_Shift_Del	INDEL	T	-	-	ADCY2	c.1282delT	P.S428fs
SH_143	6	46846033	46846033	Nonsense_Mutation	SNV	G	T	T	ADGRF5	c.1146C>A	P.C382*
SH_143	6	64694495	64694495	Missense_Mutation	SNV	G	T	T	EYS	c.6836C>A	P.A2279E
SH_143	7	27868383	27868383	Missense_Mutation	SNV	G	T	T	TAX1BP1	c.2179G>T	P.D727Y
SH_143	7	43503278	43503278	Missense_Mutation	SNV	A	G	G	HECW1	c.2569A>G	P.T857A
SH_143	8	119122426	119122426	Missense_Mutation	SNV	T	C	C	EXT1	c.860A>G	P.H287R
SH_143	8	144895892	144895892	Missense_Mutation	SNV	G	C	C	SCRIB	c.361C>G	P.P121A
SH_143	8	145657749	145657749	Silent	SNV	G	A	A	TONSL	c.3654C>T	P.L1218L
SH_143	8	146076454	146076454	Missense_Mutation	SNV	G	C	C	COMMD5	c.270C>G	P.H90Q
SH_143	8	32616818	32616818	Splice_Site	SNV	A	G	G	NRG1	c.922-6A>G	
SH_143	8	87683256	87683256	Nonsense_Mutation	SNV	T	A	A	CNGB3	c.409A>T	P.K137*
SH_143	9	97321230	97321230	Missense_Mutation	SNV	G	A	A	FBP2	c.1010C>T	P.A337V
SH_143	X	128701285	128701285	Missense_Mutation	SNV	A	G	G	OCRL	c.1411A>G	P.I471V
SH_143	X	151869767	151869767	Missense_Mutation	SNV	A	G	G	MAGEA6	c.457A>G	P.K153E
SH_143	X	69353826	69353826	Missense_Mutation	SNV	C	T	T	IGBP1	c.29C>T	P.P10L
SH_143	X	77224545	77224545	Silent	SNV	C	T	T	PGAM4	c.591G>A	P.V197V
SH_144	1	152883791	152883791	Missense_Mutation	SNV	G	T	T	IVL	c.1518G>T	P.Q506H
SH_144	1	158670122	158670122	Silent	SNV	G	A	A	OR6K2	c.321C>T	P.G107G
SH_144	10	101165535	101165535	Missense_Mutation	SNV	T	A	A	GOT1	c.620A>T	P.K207M
SH_144	11	5632106	5632106	Missense_Mutation	SNV	C	T	T	TRIM6	c.1085C>T	P.S362F
SH_144	12	25398284	25398284	Missense_Mutation	SNV	C	A	A	KRAS	c.35G>T	P.G12V
SH_144	12	2675598	2675598	Missense_Mutation	SNV	C	T	T	CACNA1C	c.1519C>T	P.R507C
SH_144	12	95927629	95927629	Missense_Mutation	SNV	C	T	T	USP44	c.404G>A	P.G135D
SH_144	13	99036977	99036977	Splice_Site	SNV	T	A	A	FARP1	c.497-7T>A	
SH_144	15	29346119	29346119	Missense_Mutation	SNV	G	A	A	APBA2	c.32G>A	P.S11N
SH_144	15	68617541	68617541	Silent	SNV	G	A	A	ITGA11	c.2250C>T	P.V750V
SH_144	15	86076959	86076959	Missense_Mutation	SNV	A	G	G	AKAP13	c.326A>G	P.N109S
SH_144	16	56969335	56969335	Missense_Mutation	SNV	A	G	G	HERPUD1	c.245A>G	P.H82R
SH_144	16	9857975	9857975	Silent	SNV	G	A	A	GRIN2A	c.3426C>T	P.P1142P
SH_144	17	3916801	3916801	Missense_Mutation	SNV	G	A	A	ZZEF1	c.8521C>T	P.H2841Y
SH_144	17	48605584	48605584	Missense_Mutation	SNV	G	A	A	MYCBPAP	c.2489G>A	P.G830D
SH_144	19	40433036	40433036	Silent	SNV	G	A	A	FCGBP	c.1233C>T	P.T411T
SH_144	19	9067768	9067768	Missense_Mutation	SNV	C	G	G	MUC16	c.19678G>C	P.A6560P
SH_144	20	1616925	1616925	Silent	SNV	G	A	A	SIRPG	c.657C>T	P.D219D
SH_144	21	34950679	34950679	Silent	SNV	C	T	T	DONSON	c.1635G>A	P.P545P
SH_144	3	164905979	164905979	Silent	SNV	A	G	G	SLITRK3	c.2640T>C	P.S880S
SH_144	3	5024843	5024843	Silent	SNV	C	T	T	BHLHE40	c.705C>T	P.S235S
SH_144	3	78663961	78663961	Splice_Site	SNV	A	G	G	ROBO1	c.3983-11T>C	
SH_144	4	177058738	177058738	Frame_Shift_Del	INDEL	C	-	-	WDR17	c.1407delC	P.I471fs
SH_144	4	177649010	177649010	Silent	SNV	G	A	A	VEGFC	c.474C>T	P.S158S
SH_144	4	90170826	90170826	Missense_Mutation	SNV	T	C	C	GPRIN3	c.436A>G	P.I146V
SH_144	5	140182649	140182649	Missense_Mutation	SNV	C	T	T	PCDHA3	c.1867C>T	P.R623C
SH_144	6	101173474	101173474	Missense_Mutation	SNV	A	C	C	ASCC3	c.1843T>G	P.L615V
SH_144	6	46977694	46977694	Missense_Mutation	SNV	C	T	T	ADGRF1	c.1477G>A	P.V493I
SH_144	X	27998532	27998532	Missense_Mutation	SNV	T	C	C	DCAF8L1	c.920A>G	P.E307G
SH_144	X	79985517	79985517	Missense_Mutation	SNV	A	G	G	BRWD3	c.1130T>C	P.L377S
SH_146	3	47165534	47165534	Nonsense_Mutation	SNV	G	A	A	SETD2	c.592C>T	P.Q198*
SH_146	5	140774176	140774176	Missense_Mutation	SNV	G	T	T	PCDHGA8	c.1796G>T	P.G599V
SH_147	1	53723145	53723145	Splice_Site	SNV	G	T	T	LRP8	c.2210-9C>A	
SH_147	10	33200967	33200967	Nonsense_Mutation	SNV	C	A	A	ITGB1	c.1555G>T	P.E519*
SH_147	11	55433390	55433390	Missense_Mutation	SNV	G	T	T	OR4C6	c.748G>T	P.V250F
SH_147	11	60105210	60105210	Splice_Site	SNV	T	G	G	MS4A6E	c.148-4T>G	
SH_147	12	100166768	100166768	Missense_Mutation	SNV	C	G	G	ANKS1B	c.1060G>C	P.D354H
SH_147	12	31944829	31944829	Missense_Mutation	SNV	G	T	T	H3F3C	c.272C>A	P.A91E
SH_147	12	94580225	94580225	Missense_Mutation	SNV	G	A	A	PLXNC1	c.1415G>A	P.C472Y
SH_147	16	49671974	49671974	Missense_Mutation	SNV	G	T	T	ZNF423	c.909C>A	P.S303R
SH_147	17	3438998	3438998	Missense_Mutation	SNV	G	T	T	TRPV3	c.653C>A	P.A218E
SH_147	17	7577046	7577046	Nonsense_Mutation	SNV	C	A	A	TP53	c.892G>T	P.E298*
SH_147	2	128387313	128387313	Missense_Mutation	SNV	A	G	G	MYO7B	c.4640A>G	P.Q1547R
SH_147	3	52637689	52637689	Missense_Mutation	SNV	C	A	A	PBRM1	c.2627G>T	P.R876L
SH_147	5	140768936	140768936	Missense_Mutation	SNV	G	T	T	PCDHGB4	c.1485G>T	P.E495D
SH_147	6	52878499	52878499	Silent	SNV	C	A	A	ICK	c.1113G>T	P.P371P
SH_147	6	7583200	7583200	Missense_Mutation	SNV	T	G	G	DSP	c.3908T>G	P.L1303R
SH_147	8	81430634	81430634	Splice_Site	SNV	C	G	G	ZBTB10	c.1961-4C>G	
SH_147	8	92007983	92007983	Silent	SNV	G	T	T	TMEM55A	c.696C>A	P.L232L
SH_147	8	92007984	92007984	Missense_Mutation	SNV	A	T	T	TMEM55A	c.695T>A	P.L232H
SH_147	9	32634328	32634328	Missense_Mutation	SNV	G	T	T	TAF1L	c.1250C>A	P.A417D
SH_147	X	147919231	147919231	Missense_Mutation	SNV	C	A	A	AFF2	c.1147C>A	P.Q383K
SH_147	X	27766382	27766382	Missense_Mutation	SNV	C	T	T	DCAF8L2	c.1370C>T	P.A457V
SH_16	1	148888233	148888233	Splice_Site	SNV	G	T	T	LOC101927429	n.540+8G>T	
SH_16	1	151681548	151681548	Missense_Mutation	SNV	C	A	A	CELF3	c.271G>T	P.A91S
SH_16	1	201180607	201180607	Missense_Mutation	SNV	T	A	A	IGFN1	c.6586T>A	P.F2196I
SH_16	1	24076378	24076378	Missense_Mutation	SNV	A	G	G	TCEB3	c.257A>G	P.H86R
SH_16	1	39768605	39768605	Missense_Mutation	SNV	T	A	A	MACF1	c.2659T>A	P.C887S
SH_16	10	107005371	107005371	Missense_Mutation	SNV	G	T	T	SORCS3	c.2940G>T	P.Q980H
SH_16	11	95712566	95712566	Missense_Mutation	SNV	C	T	T	MAML2	c.3017G>A	P.S1006N
SH_16	12	46125092	46125092	Frame_Shift_Del	INDEL	C	-	-	ARID2	c.279delC	P.L94fs
SH_16	12	7252302	7252302	Missense_Mutation	SNV	T	C	C	C1RL	c.545A>G	P.E182G
SH_16	14	56104500	56104500	Missense_Mutation	SNV	A	G	G	KTN1	c.1720A>G	P.I574V
SH_16	15	28451412	28451412	Missense_Mutation	SNV	C	A	A	HERC2	c.7186G>T	P.A2396S
SH_16	16	71509288	71509288	Missense_Mutation	SNV	C	T	T	ZNF19	c.1162G>A	P.D388N
SH_16	16	74425621	74425621	Silent	SNV	C	G	G	NPIPB15	c.975C>G	P.V325V
SH_16	16	79245590	79245590	Missense_Mutation	SNV	G	T	T	WWOX	c.803G>T	P.R268L
SH_16	17	10408714	10408714	Silent	SNV	A	G	G	MYH1	c.2289T>C	P.G763G
SH_16	17	7577534	7577534	Missense_Mutation	SNV	C	A	A	TP53	c.747G>T	P.R249S
SH_16	19	8997470	8997470	Missense_Mutation	SNV	T	C	C	MUC16	c.40952A>G	P.N13651S
SH_16	2	231065609	231065609	Missense_Mutation	SNV	A	G	G	SP110	c.1139T>C	P.V380A
SH_16	2	26437363	26437363	Missense_Mutation	SNV	T	G	G	HADHA	c.867A>C	P.K289N
SH_16	20	9353722	9353722	Missense_Mutation	SNV	G	T	T	PLCB4	c.715G>T	P.V239L
SH_16	3	111870794	111870794	Missense_Mutation	SNV	G	T	T	SLC9C1	c.3290C>A	P.A1097D
SH_16	3	178937402	178937402	Missense_Mutation	SNV	A	C	C	PIK3CA	c.1790A>C	P.Q597P
SH_16	6	112397659	112397659	Missense_Mutation	SNV	G	T	T	TUBE1	c.502C>A	P.P168T
SH_16	6	143605321	143605321	Silent	SNV	A	T	T	AIG1	c.444A>T	P.G148G
SH_16	6	28365896	28365896	Missense_Mutation	SNV	G	A	A	ZSCAN12	c.287C>T	P.T96I

SH_16	6	3137682	3137682	Missense_Mutation	SNV	G	A	A	BPHL	c.568G>A	P.E190K
SH_16	6	5613523	5613523	Frame_Shift_Del	INDEL	T	-	-	FARS2	c.1188delT	P.D397fs
SH_16	7	11514106	11514106	Missense_Mutation	SNV	G	A	A	THSD7A	c.2107C>T	P.P703S
SH_16	7	148904439	148904439	Splice_Site	SNV	C	A	A	ZNF282	c.713-4C>A	
SH_16	8	35624493	35624493	Missense_Mutation	SNV	C	A	A	UNC5D	c.2372C>A	P.T791K
SH_16	9	18635934	18635934	Splice_Site	SNV	T	A	A	ADAMTSL1	c.602-7T>A	
SH_16	9	27172744	27172744	Missense_Mutation	SNV	G	C	C	TEK	c.759G>C	P.K253N
SH_16	9	33467459	33467459	Frame_Shift_Del	INDEL	C	-	-	NOL6	c.1658delG	P.G553fs
SH_17	1	160105259	160105259	Silent	SNV	C	T	T	ATP1A2	c.2151C>T	P.N717N
SH_17	1	186084057	186084057	Nonsense_Mutation	SNV	C	T	T	HMCN1	c.11383C>T	P.R3795*
SH_17	11	6291978	6291978	Silent	SNV	C	T	T	CCKBR	c.504C>T	P.D168D
SH_17	11	7110527	7110527	Missense_Mutation	SNV	C	T	T	RBMXL2	c.176C>T	P.P59L
SH_17	12	25398284	25398284	Missense_Mutation	SNV	C	T	T	KRAS	c.35G>A	P.G12D
SH_17	17	7578404	7578404	Missense_Mutation	SNV	A	T	T	TP53	c.526T>A	P.C176S
SH_17	20	39800857	39800857	Missense_Mutation	SNV	C	T	T	PLCG1	c.2833C>T	P.R945W
SH_17	6	152673313	152673313	Missense_Mutation	SNV	G	A	A	SYNE1	c.11384C>T	P.T3795M
SH_17	6	25726439	25726439	Missense_Mutation	SNV	C	T	T	HIST1H2AA	c.317G>A	P.G106D
SH_17	7	129350276	129350276	Silent	SNV	A	G	G	NRF1	c.828A>G	P.E276E
SH_17	X	53619395	53619395	Missense_Mutation	SNV	C	T	T	HUWE1	c.3935G>A	P.R1312H
SH_18	1	158054241	158054241	Missense_Mutation	SNV	G	T	T	KIRREL	c.82G>T	P.G28C
SH_18	1	230468694	230468694	Missense_Mutation	SNV	C	T	T	PGBD5	c.1169G>A	P.R390Q
SH_18	11	12020291	12020291	Silent	SNV	T	C	C	DKK3	c.387A>G	P.S129S
SH_18	11	5254266	5254266	Silent	SNV	G	T	T	HBD	c.372C>A	P.T124T
SH_18	12	26277461	26277461	Silent	SNV	G	A	A	BHLHE41	c.117C>T	P.D39D
SH_18	13	46726927	46726927	Missense_Mutation	SNV	T	G	G	LCP1	c.727A>C	P.S243R
SH_18	15	48533766	48533766	Missense_Mutation	SNV	G	T	T	SLC12A1	c.1270G>T	P.V424F
SH_18	17	48677006	48677006	Missense_Mutation	SNV	G	T	T	CACNA1G	c.3476G>T	P.R1159L
SH_18	19	58153454	58153454	Missense_Mutation	SNV	A	G	G	ZNF211	c.1795A>G	P.K599E
SH_18	19	9076224	9076224	Missense_Mutation	SNV	C	A	A	MUC16	c.11222G>T	P.G3741V
SH_18	2	20405124	20405124	Missense_Mutation	SNV	T	C	C	SDC1	c.128A>G	P.N43S
SH_18	3	110912063	110912063	Missense_Mutation	SNV	G	A	A	NECTIN3	c.1301G>A	P.R434K
SH_18	4	26640392	26640392	Splice_Site	SNV	G	T	T	TBC1D19	c.175-1G>T	
SH_18	5	169533358	169533358	Missense_Mutation	SNV	G	A	A	FOXI1	c.397G>A	P.A133T
SH_19	1	160788066	160788066	Missense_Mutation	SNV	C	G	G	LY9	c.1131C>G	P.C377W
SH_19	1	85736473	85736473	Silent	SNV	T	G	G	BCL10	c.174A>C	P.R58R
SH_19	10	127524684	127524684	Frame_Shift_Ins	INDEL	-	T	T	BCCIP	c.786_787insT	P.K263fs
SH_19	10	49447708	49447708	Missense_Mutation	SNV	C	A	A	FRMPD2	c.728G>T	P.S243I
SH_19	10	93601080	93601080	Missense_Mutation	SNV	T	C	C	TNKS2	c.1714T>C	P.Y572H
SH_19	11	8661866	8661866	Nonsense_Mutation	SNV	G	A	A	TRIM66	c.1621C>T	P.Q541*
SH_19	13	101997759	101997759	Nonsense_Mutation	SNV	C	T	T	NALCN	c.657G>A	P.W219*
SH_19	16	1306632	1306632	Silent	SNV	C	T	T	TPSD1	c.198C>T	P.C66C
SH_19	4	110454797	110454797	Missense_Mutation	SNV	G	A	A	SEC24B	c.3439G>A	P.D1147N
SH_19	6	30313242	30313242	Splice_Site	SNV	A	T	T	RPP21	c.159-2A>T	
SH_19	7	48413961	48413961	Silent	SNV	C	T	T	ABCA13	c.11151C>T	P.T3717T
SH_19	7	8198260	8198260	Missense_Mutation	SNV	G	A	A	ICA1	c.602C>T	P.A201V
SH_19	9	101900207	101900207	Missense_Mutation	SNV	G	T	T	TGFBR1	c.641G>T	P.G214V
SH_20	1	248343535	248343535	Missense_Mutation	SNV	T	C	C	OR2M2	c.248T>C	P.F83S
SH_20	1	53728185	53728185	Silent	SNV	C	A	A	LRP8	c.1707G>T	P.G569G
SH_20	1	75708631	75708631	Missense_Mutation	SNV	T	A	A	SLC44A5	c.411A>T	P.K137N
SH_20	10	33218859	33218859	Missense_Mutation	SNV	T	C	C	ITGB1	c.267A>G	P.I89M
SH_20	10	6527155	6527155	Missense_Mutation	SNV	T	A	A	PRKCQ	c.977A>T	P.N326I
SH_20	11	61732277	61732277	Silent	SNV	C	T	T	FTH1	c.474G>A	P.K158K
SH_20	12	41337930	41337930	Frame_Shift_Ins	INDEL	-	GA	GA	CNTN1	1642_1643dup(	P.D548fs
SH_20	12	66622112	66622112	Silent	SNV	C	T	T	IRAK3	c.666C>T	P.N222N
SH_20	12	93245003	93245003	Missense_Mutation	SNV	T	C	C	EEA1	c.682A>G	P.K228E
SH_20	13	28964068	28964068	Missense_Mutation	SNV	T	C	C	FLT1	c.1834A>G	P.T612A
SH_20	17	7577534	7577534	Missense_Mutation	SNV	C	A	A	TP53	c.747G>T	P.R249S
SH_20	19	55363450	55363450	Splice_Site	SNV	C	A	A	KIR3DL2	c.71-3C>A	
SH_20	19	56423938	56423938	Missense_Mutation	SNV	G	T	T	NLRP13	c.1245C>A	P.N415K
SH_20	3	111923246	111923246	Missense_Mutation	SNV	G	A	A	SLC9C1	c.1847C>T	P.A616V
SH_20	3	146312817	146312817	Splice_Site	SNV	A	T	T	PLSCR5	c.232+11T>A	
SH_20	3	58084471	58084471	Missense_Mutation	SNV	A	T	T	FLNB	c.1181A>T	P.E394V
SH_20	3	74350606	74350606	Nonsense_Mutation	SNV	T	A	A	CNTN3	c.2038A>T	P.K680*
SH_20	4	107845802	107845802	Missense_Mutation	SNV	A	T	T	DKK2	c.429T>A	P.D143E
SH_20	5	140236939	140236939	Missense_Mutation	SNV	G	A	A	PCDHA10	c.1306G>A	P.A436T
SH_20	6	26091275	26091275	Missense_Mutation	SNV	G	A	A	HFE	c.283G>A	P.D95N
SH_20	7	131829986	131829986	Missense_Mutation	SNV	C	A	A	PLXNA4	c.5117G>T	P.G1706V
SH_20	7	54610501	54610501	Missense_Mutation	SNV	A	T	T	VSTM2A	c.78A>T	P.Q26H
SH_20	8	87423940	87423940	Missense_Mutation	SNV	A	T	T	WWP1	c.898A>T	P.S300C
SH_20	X	147018025	147018025	Missense_Mutation	SNV	A	G	G	FMR1	c.883A>G	P.K295E
SH_24	18	48604743	48604780	Frame_Shift_Del	INDEL	ATTCACTTAC	-	-	SMAD4	TTGAAATTCACT	P.C523fs
SH_24	6	27925499	27925499	Missense_Mutation	SNV	A	C	C	OR2B6	c.481A>C	P.T161P
SH_25	1	151789714	151789714	Missense_Mutation	SNV	G	A	A	RORC	c.50C>T	P.S17L
SH_25	1	155207299	155207299	Missense_Mutation	SNV	C	T	T	GBA	c.832G>A	P.G278R
SH_25	1	158614179	158614179	Missense_Mutation	SNV	T	C	C	SPTA1	c.4202A>G	P.Q1401R
SH_25	1	180155280	180155280	Missense_Mutation	SNV	T	A	A	QSOX1	c.980T>A	P.L327Q
SH_25	1	225510522	225510522	Missense_Mutation	SNV	T	A	A	DNAH14	c.9213T>A	P.D3071E
SH_25	1	24696306	24696306	Missense_Mutation	SNV	G	T	T	STPG1	c.595C>A	P.L199I
SH_25	10	17026108	17026108	Silent	SNV	A	T	T	CUBN	c.4521T>A	P.T1507T
SH_25	10	27323935	27323935	Missense_Mutation	SNV	T	A	A	ANKRD26	c.3441A>T	P.Q1147H
SH_25	10	29822291	29822291	Silent	SNV	C	A	A	SVIL	c.1005G>T	P.A335A
SH_25	11	1099786	1099786	Frame_Shift_Del	INDEL	G	-	-	MUC2	c.7371delG	P.R2459fs
SH_25	11	56258299	56258299	Missense_Mutation	SNV	A	T	T	OR5M8	c.548T>A	P.L183Q
SH_25	11	56756576	56756576	Missense_Mutation	SNV	T	G	G	OR5AK2	c.188T>G	P.L63R
SH_25	12	1702845	1702845	Missense_Mutation	SNV	T	C	C	FBXL14	c.388A>G	P.I130V
SH_25	12	31236909	31236909	Missense_Mutation	SNV	G	A	A	DDX11	c.307G>A	P.G103S
SH_25	12	63974547	63974547	Missense_Mutation	SNV	T	A	A	DPY19L2	c.1795A>T	P.I599L
SH_25	12	78582472	78582472	Silent	SNV	T	A	A	NAV3	c.5970T>A	P.I1990I
SH_25	13	103389927	103389927	Missense_Mutation	SNV	T	C	C	CCDC168	c.13120A>G	P.K4374E
SH_25	13	76374968	76374968	Missense_Mutation	SNV	G	C	C	LMO7	c.611G>C	P.R204T
SH_25	14	64892794	64892794	Missense_Mutation	SNV	A	T	T	MTHFD1	c.1011A>T	P.E337D
SH_25	15	31294159	31294159	Missense_Mutation	SNV	C	A	A	TRPM1	c.4795G>T	P.V1599L
SH_25	16	60392609	60392609	Missense_Mutation	SNV	A	T	T	LOC729159	c.998T>A	P.L333Q
SH_25	17	41852171	41852171	Silent	SNV	G	A	A	DUSP3	c.261C>T	P.G87G
SH_25	19	33953923	33953923	Missense_Mutation	SNV	T	C	C	PEPD	c.649A>G	P.M217V
SH_25	19	58017748	58017748	Missense_Mutation	SNV	T	A	A	ZNF773	c.278T>A	P.L93Q
SH_25	2	108455427	108455427	Splice_Site	SNV	A	G	G	RGPD4	c.405+7A>G	
SH_25	2	109382945	109382945	Missense_Mutation	SNV	G	A	A	RANBP2	c.5950G>A	P.G1984S

SH_25	2	131220756	131220756	Missense_Mutation	SNV	C	A	A	POTEI	c.2861G>T	P.W954L
SH_25	2	179480059	179480059	Missense_Mutation	SNV	A	T	T	TTN	c.43690T>A	P.C14564S
SH_25	2	222428585	222428585	Missense_Mutation	SNV	C	T	T	EPHA4	c.689G>A	P.R230Q
SH_25	3	100489594	100489594	Silent	SNV	A	T	T	ABI3BP	c.2601T>A	P.T867T
SH_25	3	56694964	56694964	Missense_Mutation	SNV	T	A	A	FAM208A	c.1242A>T	P.E414D
SH_25	4	187455400	187455400	Missense_Mutation	SNV	C	A	A	MTNR1A	c.496G>T	P.G166W
SH_25	4	38696488	38696488	Missense_Mutation	SNV	A	G	G	KLF3	c.817A>G	P.K273E
SH_25	4	52743962	52743962	Silent	SNV	G	A	A	DCUN1D4	c.285G>A	P.K95K
SH_25	6	102250231	102250231	Missense_Mutation	SNV	G	C	C	GRIK2	c.1121G>C	P.R374T
SH_25	6	150240933	150240933	Silent	SNV	A	G	G	RAET1G	c.105T>C	P.Y35Y
SH_25	6	41711073	41711073	Missense_Mutation	SNV	T	A	A	PGC	c.383A>T	P.Q128L
SH_25	6	43333120	43333120	Frame_Shift_Del	INDEL	C	-	-	ZNF318	c.458delG	P.G153fs
SH_25	6	49754093	49754093	Missense_Mutation	SNV	T	A	A	PGK2	c.808A>T	P.M270L
SH_25	7	100636133	100636133	Silent	SNV	T	C	C	MUC12	c.2289T>C	P.R763R
SH_25	7	80091863	80091863	Silent	SNV	A	T	T	GNAT3	c.675T>A	P.A225A
SH_25	8	142487587	142487587	Splice_Site	SNV	A	G	G	MROH5	c.1366-5T>C	
SH_25	8	61178570	61178570	Missense_Mutation	SNV	C	T	T	CA8	c.331G>A	P.E111K
SH_25	9	113168980	113168980	Missense_Mutation	SNV	T	C	C	SVEP1	c.8900A>G	P.H2967R
SH_25	X	122755252	122755252	Silent	SNV	A	T	T	THOC2	c.3972T>A	P.S1324S
SH_26	1	103491886	103491886	Nonsense_Mutation	SNV	A	T	T	COL11A1	c.783T>A	P.Y261*
SH_26	1	145456723	145456723	Frame_Shift_Del	INDEL	C	-	-	POLR3GL	c.571delG	P.E191fs
SH_26	1	151001429	151001429	Splice_Site	SNV	G	T	T	PRUNE1	c.387+9G>T	
SH_26	1	151789184	151789184	Missense_Mutation	SNV	T	C	C	RORC	c.191A>G	P.H64R
SH_26	1	152383072	152383072	Silent	SNV	C	A	A	CRNN	c.486G>T	P.A162A
SH_26	1	155931448	155931448	Splice_Site	SNV	T	A	A	ARHGEF2	c.1468+4A>T	
SH_26	1	161559531	161559531	Missense_Mutation	SNV	A	T	T	FCGR2C	c.313A>T	P.N105Y
SH_26	1	162335213	162335213	Missense_Mutation	SNV	A	G	G	NOS1AP	c.74A>G	P.Q25R
SH_26	1	171753573	171753573	Missense_Mutation	SNV	A	C	C	METTL13	c.589A>C	P.T197P
SH_26	1	186045576	186045576	Missense_Mutation	SNV	G	T	T	HMCN1	c.8307G>T	P.Q2769H
SH_26	1	201194977	201194977	Missense_Mutation	SNV	C	G	G	IGFN1	c.10512C>G	P.I3504M
SH_26	1	222920395	222920395	Silent	SNV	A	T	T	FAM177B	c.213A>T	P.A71A
SH_26	1	43784956	43784956	Silent	SNV	A	T	T	TIE1	c.2973A>T	P.L991L
SH_26	1	45253107	45253107	Missense_Mutation	SNV	A	T	T	BEST4	c.184T>A	P.Y62N
SH_26	1	47610033	47610033	Missense_Mutation	SNV	A	T	T	CYP4A22	c.795A>T	P.Q265H
SH_26	10	105200499	105200499	Silent	SNV	G	T	T	PDCD11	c.4449G>T	P.V1483V
SH_26	10	134691545	134691545	Missense_Mutation	SNV	A	T	T	CFAP46	c.4052T>A	P.L1351Q
SH_26	10	61443912	61443912	Missense_Mutation	SNV	T	A	A	SLC16A9	c.138A>T	P.E46D
SH_26	10	98303889	98303889	Missense_Mutation	SNV	T	C	C	TM9SF3	c.1129A>G	P.I377V
SH_26	11	1020229	1020229	Missense_Mutation	SNV	C	T	T	MUC6	c.3669G>A	P.M1223I
SH_26	11	125448157	125448157	Splice_Site	SNV	G	T	T	EI24	c.399+4G>T	
SH_26	11	16847727	16847727	Missense_Mutation	SNV	C	G	G	PLEKHA7	c.1283G>C	P.R428T
SH_26	11	59604645	59604645	Splice_Site	SNV	A	T	T	GIF	c.871+2T>A	
SH_26	12	104715085	104715085	Silent	SNV	A	T	T	TXNRD1	c.1206A>T	P.V402V
SH_26	12	109964161	109964161	Splice_Site	SNV	A	T	T	UBE3B	c.2569-2A>T	
SH_26	12	118848896	118848896	Silent	SNV	C	G	G	SUDS3	c.822C>G	P.A274A
SH_26	12	54447850	54447850	Silent	SNV	C	T	T	HOXC4	c.144C>T	P.H48H
SH_26	12	7064439	7064439	Splice_Site	SNV	T	G	G	PTPN6	c.633+9T>G	
SH_26	12	70980902	70980902	Silent	SNV	G	A	A	PTPRB	c.2196C>T	P.S732S
SH_26	12	80660340	80660340	Silent	SNV	C	T	T	OTOGL	c.2307C>T	P.G769G
SH_26	12	99065362	99065362	Missense_Mutation	SNV	A	T	T	APAF1	c.1625A>T	P.H542L
SH_26	13	103387815	103387815	Frame_Shift_Ins	INDEL	-	A	A	CCDC168	15231_15232in	P.L5078fs
SH_26	13	103387816	103387816	Missense_Mutation	SNV	T	A	A	CCDC168	c.15231A>T	P.L5077F
SH_26	13	28136914	28136914	Missense_Mutation	SNV	T	A	A	LNX2	c.860A>T	P.N287I
SH_26	13	41706613	41706613	Missense_Mutation	SNV	C	T	T	KBTBD6	c.35G>A	P.R12H
SH_26	13	48934155	48934155	Nonsense_Mutation	SNV	G	T	T	RB1	c.610G>T	P.E204*
SH_26	14	21024751	21024751	Missense_Mutation	SNV	T	A	A	RNASE9	c.478A>T	P.R160W
SH_26	14	34400402	34400402	Missense_Mutation	SNV	G	A	A	EGLN3	c.95C>T	P.P32L
SH_26	15	41961511	41961511	Missense_Mutation	SNV	G	A	A	MGA	c.419G>A	P.R140H
SH_26	15	43352244	43352244	Splice_Site	SNV	A	C	C	UBR1	c.861+6T>G	
SH_26	15	48559862	48559862	Silent	SNV	A	T	T	SLC12A1	c.2259A>T	P.A753A
SH_26	15	52540980	52540980	Missense_Mutation	SNV	C	T	T	MYO5C	c.1663G>A	P.V555I
SH_26	17	27286237	27286237	Missense_Mutation	SNV	A	T	T	SEZ6	c.1913T>A	P.L638Q
SH_26	17	36485747	36485747	Silent	SNV	G	A	A	GPR179	c.3705C>T	P.S1235S
SH_26	17	42744211	42744211	Missense_Mutation	SNV	T	A	A	MEIOC	c.932T>A	P.M311K
SH_26	17	55187384	55187384	Splice_Site	SNV	A	T	T	AKAP1	c.1715-2A>T	
SH_26	17	7578177	7578177	Missense_Mutation	SNV	C	A	A	TP53	c.672G>T	P.E224D
SH_26	18	56008937	56008937	Missense_Mutation	SNV	G	T	T	NEDD4L	c.922G>T	P.A308S
SH_26	18	57103235	57103235	Missense_Mutation	SNV	T	A	A	CCBE1	c.1126A>T	P.S376C
SH_26	18	8114778	8114778	Splice_Site	SNV	A	G	G	PTPRM	c.2131-11A>G	
SH_26	19	18324189	18324189	Missense_Mutation	SNV	C	T	T	PDE4C	c.1597G>A	P.D533N
SH_26	19	22847939	22847939	Missense_Mutation	SNV	T	A	A	ZNF492	c.1468T>A	P.S490T
SH_26	19	34929592	34929592	Nonsense_Mutation	SNV	A	T	T	UBA2	c.502A>T	P.R168*
SH_26	19	41600269	41600269	Missense_Mutation	SNV	A	T	T	CYP2A13	c.1093A>T	P.M365L
SH_26	19	43420615	43420615	Missense_Mutation	SNV	A	C	C	PSG6	c.89T>G	P.L30R
SH_26	19	45397225	45397225	Missense_Mutation	SNV	A	T	T	TOMM40	c.545A>T	P.Q182L
SH_26	19	48305649	48305649	Missense_Mutation	SNV	T	A	A	TPRX1	c.619A>T	P.I207F
SH_26	19	9677401	9677401	Missense_Mutation	SNV	T	C	C	ZNF121	c.388A>G	P.S130G
SH_26	2	107075753	107075753	Nonsense_Mutation	SNV	A	C	C	RGPD3	c.108T>G	P.Y36*
SH_26	2	133075566	133075566	Missense_Mutation	SNV	T	A	A	ZNF806	c.1027T>A	P.Y343N
SH_26	2	197987363	197987363	Splice_Site	SNV	T	A	A	ANKRD44	c.693+11A>T	
SH_26	2	207615711	207615711	Frame_Shift_Del	INDEL	G	-	-	MDH1B	c.999delC	P.I334fs
SH_26	20	44279191	44279191	Missense_Mutation	SNV	A	T	T	WFDC11	c.49T>A	P.C17S
SH_26	20	45004377	45004377	Missense_Mutation	SNV	T	C	C	ELMO2	c.596A>G	P.Y199C
SH_26	20	61391368	61391368	Splice_Site	SNV	A	T	T	NTSR1	c.1008-2A>T	
SH_26	22	26343745	26343745	Missense_Mutation	SNV	A	T	T	MYO18B	c.5702A>T	P.Q1901L
SH_26	22	30518101	30518101	Silent	SNV	A	T	T	HORMAD2	c.717A>T	P.T239T
SH_26	22	44583718	44583718	Silent	SNV	G	A	A	PARVG	c.207G>A	P.E69E
SH_26	3	105243336	105243336	Silent	SNV	A	T	T	ALCAM	c.378A>T	P.T126T
SH_26	3	113378388	113378388	Missense_Mutation	SNV	A	G	G	USF3	c.2141T>C	P.L714P
SH_26	3	130311438	130311438	Silent	SNV	T	C	C	COL6A6	c.4326T>C	P.Y1442Y
SH_26	4	108614942	108614942	Missense_Mutation	SNV	T	C	C	PAPSS1	c.396A>G	P.I132M
SH_26	4	126412071	126412071	Silent	SNV	G	A	A	FAT4	c.14094G>A	P.L4698L
SH_26	4	15560785	15560785	Splice_Site	SNV	T	A	A	CC2D2A	c.2830-3T>A	
SH_26	4	165118701	165118701	Missense_Mutation	SNV	T	A	A	ANP32C	c.163A>T	P.T55S
SH_26	4	55976869	55976869	Missense_Mutation	SNV	A	T	T	KDR	c.1043T>A	P.V348D
SH_26	4	80905082	80905082	Missense_Mutation	SNV	C	A	A	ANTXR2	c.1129G>T	P.D377Y
SH_26	4	96199465	96199465	Nonsense_Mutation	SNV	A	T	T	UNC5C	c.539T>A	P.L180*
SH_26	5	132228797	132228797	Missense_Mutation	SNV	T	G	G	AFF4	c.2321A>C	P.N774T

SH_26	5	140788050	140788050	Missense_Mutation	SNV	A	T	T	PCDHGB6	c.281A>T	P.Q94L	
SH_26	5	156514136	156514136	Missense_Mutation	SNV	C	A	A	HAVCR2	c.883G>T	P.G295C	
SH_26	5	19483540	19483540	Nonsense_Mutation	SNV	G	T	T	CDH18	c.1643C>A	P.S548*	
SH_26	5	19839092	19839092	Missense_Mutation	SNV	T	C	C	CDH18	c.4A>G	P.K2E	
SH_26	5	89918411	89918411	Splice_Site	SNV	T	C	C	ADGRV1	c.454-3T>C		
SH_26	6	155761094	155761094	Missense_Mutation	SNV	T	A	A	NOX3	c.664A>T	P.T222S	
SH_26	6	26107950	26107950	Silent	SNV	A	T	T	HIST1H1T	c.372T>A	P.A124A	
SH_26	6	29342352	29342352	Missense_Mutation	SNV	G	T	T	OR12D3	c.713C>A	P.S238Y	
SH_26	6	29574706	29574706	Missense_Mutation	SNV	G	T	T	GABBR1	c.1654C>A	P.Q552K	
SH_26	6	32797229	32797229	Missense_Mutation	SNV	A	C	C	TAP2	c.1880T>G	P.V627G	
SH_26	6	33371764	33371764	Missense_Mutation	SNV	A	T	T	KIFC1	c.614A>T	P.E205V	
SH_26	7	138258349	138258349	Missense_Mutation	SNV	A	T	T	TRIM24	c.1874A>T	P.Q625L	
SH_26	7	141755491	141755491	Missense_Mutation	SNV	A	T	T	MGAM	c.3448A>T	P.T1150S	
SH_26	7	141763320	141763320	Missense_Mutation	SNV	T	A	A	MGAM	c.4279T>A	P.F1427I	
SH_26	7	144060399	144060399	Nonsense_Mutation	SNV	G	T	T	ARHGEF5	c.637G>T	P.E213*	
SH_26	7	151945241	151945241	Missense_Mutation	SNV	T	A	A	KMT2C	c.2278A>T	P.I760L	
SH_26	7	56052539	56052539	Splice_Site	SNV	G	T	T	GBAS	c.469-1G>T		
SH_26	7	99221754	99221754	Silent	SNV	A	T	T	ZSCAN25	c.756A>T	P.I252I	
SH_26	8	100733119	100733119	Missense_Mutation	SNV	A	T	T	VPS13B	c.6969A>T	P.E2323D	
SH_26	8	105509634	105509634	Silent	SNV	A	G	G	LRP12	c.1089T>C	P.C363C	
SH_26	8	113655811	113655811	Splice_Site	SNV	A	C	C	MIR2053	n.90A>C		
SH_26	8	133141758	133141758	Silent	SNV	C	T	T	KCNQ3	c.2010G>A	P.L670L	
SH_26	8	16850679	16850679	Missense_Mutation	SNV	T	A	A	FGF20	c.538A>T	P.R180W	
SH_26	8	56435975	56435975	Missense_Mutation	SNV	T	C	C	XKR4	c.1142T>C	P.F381S	
SH_26	8	56708615	56708615	Silent	SNV	A	T	T	TGS1	c.1167A>T	P.L389L	
SH_26	8	86250689	86250689	Splice_Site	SNV	A	G	G	CA1	c.38-11T>C		
SH_26	9	117692411	117692411	Missense_Mutation	SNV	A	T	T	TNFSF8	c.173T>A	P.M58K	
SH_26	9	118666932	118666932	Splice_Site	SNV	A	T	T	LINC00474	n.77-11T>A		
SH_26	9	125486740	125486740	Missense_Mutation	SNV	C	A	A	OR1L4	c.472C>A	P.L158M	
SH_26	9	39118130	39118130	Missense_Mutation	SNV	T	G	G	CNTNAP3	c.2207A>C	P.Y736S	
SH_26	9	40705661	40705663	Missense_Mutation	INDEL	TAT	-	-	SPATA31A3	3319_3321delA	P.I1107del	
SH_26	X	104512082	104512082	Silent	SNV	A	T	T	IL1RAPL2	c.555A>T	P.P185P	
SH_26	X	36317165	36317165	Missense_Mutation	SNV	G	T	T	CFAP47	c.7760G>T	P.S2587I	
SH_26	X	47308003	47308003	Missense_Mutation	SNV	C	A	A	ZNF41	c.908G>T	P.G303V	
SH_26	X	50134504	50134504	Nonsense_Mutation	SNV	C	T	T	DGKK	c.1775G>A	P.W592*	
SH_28	1	111957125	111957125	Frame_Shift_Del	INDEL	T	-	-	OVGP1	c.1998delA	P.E667fs	
SH_28	1	186092174	186092174	Silent	SNV	G	A	A	HMCN1	c.12321G>A	P.P4107P	
SH_28	1	207245668	207245668	Missense_Mutation	SNV	G	T	T	PFKFB2	c.1470G>T	P.K490N	
SH_28	1	248343439	248343439	Missense_Mutation	SNV	T	C	C	OR2M2	c.152T>C	P.L51P	
SH_28	1	52878217	52878217	Missense_Mutation	SNV	T	A	A	PRPF38A	c.530T>A	P.L177Q	
SH_28	1	53682484	53682484	Silent	SNV	C	T	T	C1orf123	c.252G>A	P.R84R	
SH_28	1	63299696	63299696	Splice_Site	INDEL	T	-	-	ATG4C	c.934-6delT		
SH_28	1	70504220	70504220	Missense_Mutation	SNV	A	G	G	LRRC7	c.2599A>G	P.T867A	
SH_28	1	86426921	86426921	Splice_Site	SNV	A	T	T	COL24A1	c.2562+11T>A		
SH_28	10	17032333	17032333	Splice_Site	SNV	C	T	T	CUBN	c.4350G>A	P.E1450E	
SH_28	10	6259131	6259131	Missense_Mutation	SNV	A	G	G	PFKFB3	c.415A>G	P.T139A	
SH_28	10	78761223	78761223	Missense_Mutation	SNV	C	A	A	KCNMA1	c.2208G>T	P.E736D	
SH_28	10	89692847	89692847	Missense_Mutation	SNV	T	C	C	PTEN	c.331T>C	P.W111R	
SH_28	11	117303080	117303080	Missense_Mutation	SNV	G	A	A	DSCAML1	c.5347C>T	P.R1783W	
SH_28	11	55653610	55653610	Frame_Shift_Del	INDEL	A	-	-	TRIM51	c.430delA	P.M144fs	
SH_28	12	110815425	110815425	Splice_Site	INDEL	-	A	A	ANAPC7	c.1235-4dupT		
SH_28	12	123801862	123801862	Silent	SNV	T	C	C	SBNO1	c.2841A>G	P.S947S	
SH_28	12	48465478	48465478	Missense_Mutation	SNV	C	T	T	SENP1	c.967G>A	P.V323M	
SH_28	12	52758834	52758834	Missense_Mutation	SNV	G	A	A	KRT85	c.541C>T	P.R181W	
SH_28	12	54063678	54063678	Nonsense_Mutation	SNV	G	A	A	ATP5G2	c.142C>T	P.R48*	
SH_28	12	6125705	6125705	Missense_Mutation	SNV	C	A	A	VWF	c.5288G>T	P.R1763L	
SH_28	12	8085704	8085704	Missense_Mutation	SNV	C	T	T	SLC2A3	c.148G>A	P.G50R	
SH_28	12	90028984	90028984	Frame_Shift_Del	INDEL	T	-	-	ATP2B1	c.451delA	P.T151fs	
SH_28	13	114469224	114469224	Silent	SNV	G	T	T	TMEM255B	c.183G>T	P.G61G	
SH_28	14	102349849	102349849	Silent	SNV	C	T	T	PPP2R5C	c.672C>T	P.F224F	
SH_28	14	23893245	23893245	Silent	SNV	C	T	T	MYH7	c.2793G>A	P.E931E	
SH_28	14	88940113	88940113	Frame_Shift_Del	INDEL	T	-	-	PTPN21	c.2545delA	P.I849fs	
SH_28	15	30382058	30382058	Silent	SNV	C	A	A	GOLGA8J	c.1030C>A	P.R344R	
SH_28	15	55613506	55613506	Missense_Mutation	SNV	T	C	C	PIGB	c.335T>C	P.L112P	
SH_28	15	74640322	74640322	Missense_Mutation	SNV	C	G	G	CYP11A1	c.344G>C	P.G115A	
SH_28	17	7577534	7577534	Missense_Mutation	SNV	C	A	A	TP53	c.747G>T	P.R249S	
SH_28	17	8171937	8171937	Missense_Mutation	SNV	G	A	A	PFAS	c.3469G>A	P.V1157M	
SH_28	17	8659736	8659736	Missense_Mutation	SNV	T	A	A	SPDYE4	c.359A>T	P.K120I	
SH_28	19	11123688	11123688	Missense_Mutation	SNV	G	A	A	SMARCA4	c.2338G>A	P.E780K	
SH_28	19	41307092	41307092	Silent	SNV	C	T	T	EGLN2	c.615C>T	P.R205R	
SH_28	19	42914611	42914611	Missense_Mutation	SNV	G	A	A	LIPE	c.1267C>T	P.R423C	
SH_28	19	51165297	51165297	Silent	SNV	G	A	A	SHANK1	c.6411C>T	P.Y2137Y	
SH_28	19	53385106	53385106	Missense_Mutation	SNV	C	G	G	ZNF320	c.273G>C	P.E91D	
SH_28	19	6719407	6719407	Missense_Mutation	SNV	T	C	C	C3	c.82A>G	P.I28V	
SH_28	2	125405360	125405360	Silent	SNV	G	A	A	CNTNAP5	c.1899G>A	P.Q633Q	
SH_28	2	128467270	128467270	Nonsense_Mutation	SNV	G	A	A	WDR33	c.3469C>T	P.R1157*	
SH_28	2	196689003	196689003	Silent	SNV	A	G	G	DNAH7	c.9267T>C	P.H3089H	
SH_28	2	210685243	210685243	Missense_Mutation	SNV	G	T	T	UNC80	c.2171G>T	P.S724I	
SH_28	20	57009718	57009718	Missense_Mutation	SNV	A	C	C	VAPB	c.272A>C	P.Q91P	
SH_28	20	61597917	61597917	Missense_Mutation	SNV	G	T	T	SLC17A9	c.1084G>T	P.A362S	
SH_28	21	35154395	35154395	Silent	SNV	A	G	G	ITSN1	c.1782A>G	P.R594R	
SH_28	3	119336894	119336894	Missense_Mutation	SNV	G	A	A	PLA1A	c.735G>A	P.M245I	
SH_28	3	164785144	164785144	Frame_Shift_Del	INDEL	T	-	-	SI	c.619delA	P.S207fs	
SH_28	3	168833263	168833263	Silent	SNV	T	C	C	MECOM	c.2028A>G	P.G676G	
SH_28	4	111543439	111543439	Missense_Mutation	SNV	A	T	T	PITX2	c.178T>A	P.S60T	
SH_28	5	129243945	129243945	Silent	SNV	G	A	A	CHSY3	c.978G>A	P.V326V	
SH_28	5	139231295	139231295	Silent	SNV	G	T	T	NRG2	c.1468C>A	P.R490R	
SH_28	5	140625308	140625308	Silent	SNV	G	T	T	PCDHB15	c.162G>T	P.L54L	
SH_28	5	159840963	159840963	Silent	SNV	G	A	A	SLU7	c.348C>T	P.R116R	
SH_28	6	117888071	117888071	Missense_Mutation	SNV	C	T	T	GOPC	c.1180G>A	P.G394S	
SH_28	6	165827144	165827144	Silent	SNV	G	T	T	PDE10A	c.1123C>A	P.R375R	
SH_28	6	46801209	46801209	Missense_Mutation	SNV	C	A	A	MEP1A	c.1543C>A	P.P515T	
SH_28	7	107542817	107542817	Missense_Mutation	SNV	T	A	A	DLD	c.246T>A	P.N82K	
SH_28	7	40899911	40899911	Splice_Site	SNV	G	T	T	SUGCT	c.1253-4G>T		
SH_28	7	70881056	70881056	Splice_Site	SNV	C	T	T	WBSCR17	c.764+7C>T		
SH_28	X	53603909	53603909	Silent	SNV	C	T	T	HUWE1	c.5835G>A	P.V1945V	
SH_29	10	71678036	71678036	Splice_Site	SNV	G	T	T	COL13A1	c.997-5G>T		
SH_29	12	12038893	12038893	Missense_Mutation	SNV	A	G	G	ETV6	c.1186A>G	P.R396G	

SH_29	12	25398284	25398284	Missense_Mutation	SNV	C	A	A	KRAS	c.35G>T	P.G12V
SH_29	15	66782901	66782901	Missense_Mutation	SNV	C	T	T	MAP2K1	c.1130C>T	P.S377F
SH_29	17	7578395	7578395	Missense_Mutation	SNV	G	A	A	TP53	c.535C>T	P.H179Y
SH_29	19	14094065	14094065	Silent	SNV	C	T	T	RFX1	c.447G>A	P.T149T
SH_29	22	32754310	32754310	Silent	SNV	C	T	T	RFPL3	c.252C>T	P.V84V
SH_29	3	32398937	32398937	Missense_Mutation	SNV	A	G	G	CMTM8	c.220A>G	P.M74V
SH_29	7	105255059	105255059	Silent	SNV	C	T	T	ATXN7L1	c.1074G>A	P.P358P
SH_29	8	145114792	145114792	Silent	SNV	G	A	A	OPLAH	c.144C>T	P.T48T
SH_29	9	112153424	112153424	Silent	SNV	G	A	A	PTPN3	c.1965C>T	P.Y655Y
SH_33	1	153905343	153905343	Missense_Mutation	SNV	C	T	T	DENND4B	c.3617G>A	P.R1206Q
SH_33	10	112838102	112838102	Missense_Mutation	SNV	C	A	A	ADRA2A	c.348C>A	P.F116L
SH_33	10	68940163	68940163	Missense_Mutation	SNV	G	A	A	CTNNA3	c.959C>T	P.T320M
SH_33	11	121430301	121430301	Missense_Mutation	SNV	A	C	C	SORL1	c.2984A>C	P.E995A
SH_33	12	113726026	113726026	Missense_Mutation	SNV	C	T	T	TPCN1	c.1909C>T	P.P637S
SH_33	12	25398284	25398284	Missense_Mutation	SNV	C	A	A	KRAS	c.35G>T	P.G12V
SH_33	15	23690558	23690558	Missense_Mutation	SNV	C	T	T	GOLGA6L2	c.112G>A	P.A38T
SH_33	16	449461	449461	Missense_Mutation	SNV	C	G	G	NME4	c.308C>G	P.S103C
SH_33	17	7578513	7578525	Frame_Shift_Del	INDEL	TGGCCAGTT	-	-	TP53	17delCCAACTG	P.C135fs
SH_33	2	125192246	125192246	Missense_Mutation	SNV	G	T	T	CNTNAP5	c.715G>T	P.A239S
SH_33	2	220505520	220505520	Missense_Mutation	SNV	T	C	C	SLC4A3	c.3538T>C	P.W1180R
SH_33	20	62227109	62227109	Missense_Mutation	SNV	T	A	A	GMEB2	c.473A>T	P.D158V
SH_33	3	137787000	137787000	Missense_Mutation	SNV	C	T	T	DZIP1L	c.1825G>A	P.G609R
SH_33	4	187530361	187530361	Silent	SNV	C	A	A	FAT1	c.10182G>T	P.V3394V
SH_33	5	176522365	176522365	Silent	SNV	C	A	A	FGFR4	c.1554C>A	P.V518V
SH_33	6	166873029	166873029	Missense_Mutation	SNV	C	T	T	RPS6KA2	c.1007G>A	P.R336Q
SH_33	7	146805424	146805424	Missense_Mutation	SNV	G	T	T	CNTNAP2	c.736G>T	P.V246F
SH_33	7	27238939	27238939	Missense_Mutation	SNV	G	A	A	HOXA13	c.758C>T	P.P253L
SH_33	9	35802213	35802213	Missense_Mutation	SNV	T	C	C	NPR2	c.1643T>C	P.V548A
SH_33	9	38396888	38396888	Silent	SNV	C	T	T	ALDH1B1	c.1143C>T	P.G381G
SH_33	X	100745656	100745656	Frame_Shift_Del	INDEL	G	-	-	ARMCX4	c.2080delG	P.A694fs
SH_33	X	100745657	100745657	Missense_Mutation	SNV	C	A	A	ARMCX4	c.2081C>A	P.A694D
SH_33	X	132161384	132161384	Missense_Mutation	SNV	A	G	G	USP26	c.865T>C	P.F289L
SH_33	X	18602479	18602479	Splice_Site	SNV	T	C	C	CDKL5	c.554+6T>C	
SH_33	X	49114840	49114840	Frame_Shift_Del	INDEL	T	-	-	FOXP3	c.123delA	P.G43fs
SH_33	X	76856044	76856044	Splice_Site	SNV	G	T	T	ATRX	c.5567-11C>A	
SH_34	1	11014114	11014114	Missense_Mutation	SNV	G	A	A	C1orf127	c.1061C>T	P.P354L
SH_34	1	1153964	1153964	Silent	SNV	G	A	A	SDF4	c.786C>T	P.P262P
SH_34	1	1248918	1248918	Missense_Mutation	SNV	G	A	A	CPSF3L	c.1030C>T	P.R344W
SH_34	1	16378220	16378220	Missense_Mutation	SNV	G	A	A	CLCNKB	c.1313G>A	P.R438H
SH_34	10	65357815	65357815	In_Frame_Ins	INDEL	-	AAAGTTAAATGG	AAAGTTAAATGG	REEP3	AAGTTAAAGTTA	nsTerGFRKLKLNGYWCLNI
SH_34	11	32118715	32118715	Missense_Mutation	SNV	G	A	A	RCN1	c.280G>A	P.D94N
SH_34	11	5221093	5221093	Missense_Mutation	SNV	C	T	T	OR51V1	c.838G>A	P.V280M
SH_34	11	64004663	64004663	Frame_Shift_Del	INDEL	A	-	-	VEGFB	c.386delA	P.K129fs
SH_34	12	9075393	9075393	Splice_Site	SNV	C	T	T	PHC1	c.612+9C>T	
SH_34	13	29001983	29001983	Silent	SNV	G	A	A	FLT1	c.1182C>T	P.D394D
SH_34	16	89777069	89777069	Missense_Mutation	SNV	G	A	A	VPS9D1	c.1183C>T	P.R395W
SH_34	17	7577509	7577509	Missense_Mutation	SNV	C	G	G	TP53	c.772G>C	P.E258Q
SH_34	19	57133360	57133360	Silent	SNV	C	T	T	ZNF71	c.705C>T	P.I235I
SH_34	2	100167958	100167958	Missense_Mutation	SNV	C	T	T	AFF3	c.3659G>A	P.R1220Q
SH_34	20	1552646	1552646	Silent	SNV	C	A	A	SIRPB1	c.471G>T	P.V157V
SH_34	20	61461004	61461004	Missense_Mutation	SNV	G	T	T	COL9A3	c.1078G>T	P.A360S
SH_34	20	62076690	62076690	Missense_Mutation	SNV	C	T	T	KCNQ2	c.415G>A	P.V139M
SH_34	21	27840860	27840860	Missense_Mutation	SNV	C	T	T	CYYR1	c.752G>A	P.R251H
SH_34	22	30000101	30000101	Splice_Site	INDEL	-	GGACCTCTTTGA	GGACCTCTTTGA	NF2	AGGGAAGGACCTCTTTGATTTGGTGTGCCGGACTCT	
SH_34	22	32588955	32588955	Missense_Mutation	SNV	T	C	C	RFPL2	c.490A>G	P.I164V
SH_34	3	134920360	134920360	Silent	SNV	C	A	A	EPHB1	c.2175C>A	P.L725L
SH_34	3	39108051	39108051	Frame_Shift_Ins	INDEL	-	T	T	WDR48	c.37dupT	P.S13fs
SH_34	6	6654824	6654824	Silent	SNV	C	T	T	LY86	c.453C>T	P.T151T
SH_34	7	140453154	140453154	Missense_Mutation	SNV	T	C	C	BRAF	c.1781A>G	P.D594G
SH_34	9	26109943	26109943	Splice_Site	SNV	T	A	A	LOC100506422	c.34-8T>A	
SH_35	1	144882622	144882622	Nonsense_Mutation	SNV	T	A	A	PDE4DIP	c.3397A>T	P.K1133*
SH_35	1	158435940	158435940	Silent	SNV	C	T	T	OR10K1	c.589C>T	P.L197L
SH_35	1	228468251	228468251	Missense_Mutation	SNV	A	C	C	OBSCN	c.7951A>C	P.K2651Q
SH_35	10	134464009	134464009	Splice_Site	SNV	G	A	A	INPP5A	c.306G>A	P.T102T
SH_35	12	104378698	104378698	Splice_Site	INDEL	-	AGGATGCAAA	AGGATGCAAA	TDG	964+1insAGGATGCAAA	
SH_35	12	129822359	129822359	Silent	SNV	C	T	T	TMEM132D	c.1119G>A	P.A373A
SH_35	12	57567046	57567046	Missense_Mutation	SNV	G	C	C	LRP1	c.3259G>C	P.D1087H
SH_35	12	58240172	58240172	Missense_Mutation	SNV	A	T	T	CTDSP2	c.47T>A	P.L16Q
SH_35	12	72665953	72665953	Splice_Site	SNV	G	C	C	TRHDE-AS1	n.746-11C>G	
SH_35	14	61924300	61924300	Missense_Mutation	SNV	A	T	T	PRKCH	c.1181A>T	P.D394V
SH_35	14	69795268	69795268	Missense_Mutation	SNV	A	C	C	GALNT16	c.670A>C	P.M224L
SH_35	15	49325204	49325204	Nonsense_Mutation	SNV	G	A	A	SECISBP2L	c.622C>T	P.R208*
SH_35	16	17234999	17234999	Splice_Site	SNV	C	T	T	XYLT1	c.1587+11G>A	
SH_35	17	48773448	48773448	Missense_Mutation	SNV	T	C	C	ANKRD40	c.1017A>G	P.I339M
SH_35	2	102818104	102818104	Missense_Mutation	SNV	A	G	G	IL1RL2	c.578A>G	P.Y193C
SH_35	2	136873013	136873013	Missense_Mutation	SNV	A	G	G	CXCR4	c.497T>C	P.I166T
SH_35	2	204009852	204009852	Missense_Mutation	SNV	G	A	A	NBEAL1	c.5186G>A	P.R1729Q
SH_35	2	241685218	241685218	Missense_Mutation	SNV	C	G	G	KIF1A	c.3311G>C	P.R1104P
SH_35	20	1961294	1961294	Missense_Mutation	SNV	C	T	T	PDYN	c.440G>A	P.R147K
SH_35	22	30057328	30057328	Splice_Site	INDEL	-	CTGGATAAGAA	CTGGATAAGAA	NF2	AAACCACTGGATAAGAAAATTGATGTCTTCAA	
SH_35	22	30064436	30064436	Splice_Site	SNV	G	A	A	NF2	c.999+1G>A	
SH_35	22	30064437	30064437	Splice_Site	INDEL	-	TCGCTCGAGAGA	TCGCTCGAGAGA	NF2	GCGCCTCGCTCGAGAGAAGCAGATGAGGGAGG	
SH_35	3	12616309	12616309	Missense_Mutation	SNV	G	A	A	MKRN2	c.532G>A	P.E178K
SH_35	3	129262117	129262117	Missense_Mutation	SNV	G	T	T	H1FOO	c.56G>T	P.G19V
SH_35	3	183953050	183953050	Missense_Mutation	SNV	C	T	T	VWA5B2	c.398C>T	P.T133I
SH_35	3	38674672	38674672	Nonsense_Mutation	SNV	G	A	A	SCN5A	c.127C>T	P.R43*
SH_35	3	44945459	44945459	Missense_Mutation	SNV	C	T	T	TGM4	c.1055C>T	P.T352M
SH_35	4	170983036	170983036	Splice_Site	SNV	C	A	A	AADAT	c.1248+7G>T	
SH_35	4	87696336	87696336	Missense_Mutation	SNV	G	A	A	PTPN13	c.5464G>A	P.D1822N
SH_35	5	110712615	110712615	Missense_Mutation	SNV	G	A	A	CAMK4	c.361G>A	P.V121I
SH_35	5	140573833	140573833	Missense_Mutation	SNV	G	A	A	PCDHB10	c.1708G>A	P.A570T
SH_35	5	7784532	7784532	Missense_Mutation	SNV	C	A	A	ADCY2	c.2439C>A	P.F813L
SH_35	6	26205091	26205091	Silent	SNV	C	T	T	HIST1H4E	c.219C>T	P.Y73Y
SH_35	6	74528152	74528152	Missense_Mutation	SNV	T	C	C	CD109	c.3902T>C	P.V1301A
SH_41	1	157660082	157660082	Silent	SNV	C	T	T	FCRL3	c.1653G>A	P.G551G
SH_41	1	208062174	208062174	Silent	SNV	G	A	A	CD34	c.825C>T	P.F275F
SH_41	1	247061569	247061569	Missense_Mutation	SNV	G	T	T	AHCTF1	c.1564C>A	P.L522I
SH_41	10	89717672	89717672	Nonsense_Mutation	SNV	C	T	T	PTEN	c.697C>T	P.R233*

SH_41	11	31312387	31312387	Frame_Shift_Del	INDEL	A	-	-	DCDC1	c.767delT	P.L256fs
SH_41	11	55653674	55653674	Missense_Mutation	SNV	A	C	C	TRIM51	c.487A>C	P.T163P
SH_41	11	55735542	55735542	Missense_Mutation	SNV	A	T	T	OR10AG1	c.398T>A	P.I133N
SH_41	11	60541301	60541301	Splice_Site	SNV	G	T	T	MS4A15	c.499-9G>T	
SH_41	11	8720838	8720838	Silent	SNV	G	A	A	ST5	c.2820C>T	P.I940I
SH_41	12	51584328	51584328	Missense_Mutation	SNV	G	A	A	POU6F1	c.608C>T	P.P203L
SH_41	12	57596242	57596242	Frame_Shift_Del	INDEL	A	-	-	LRP1	c.10634delA	P.N3545fs
SH_41	12	58190116	58190116	Missense_Mutation	SNV	C	A	A	TSFM	c.728C>A	P.S243Y
SH_41	12	6729407	6729407	Silent	SNV	G	A	A	LPAR5	c.1008C>T	P.A336A
SH_41	12	78574763	78574763	Missense_Mutation	SNV	C	T	T	NAV3	c.5630C>T	P.S1877F
SH_41	13	21563060	21563060	Missense_Mutation	SNV	C	A	A	LATS2	c.859G>T	P.A287S
SH_41	13	49050852	49050852	Nonsense_Mutation	SNV	C	T	T	RB1	c.2536C>T	P.Q846*
SH_41	14	105406645	105406645	Missense_Mutation	SNV	G	A	A	AHNAK2	c.15143C>T	P.S5048F
SH_41	14	53130556	53130556	Silent	SNV	T	A	A	ERO1A	c.639A>T	P.T213T
SH_41	15	28483324	28483324	Missense_Mutation	SNV	G	T	T	HERC2	c.3788C>A	P.A1263D
SH_41	15	66727451	66727451	Missense_Mutation	SNV	A	C	C	MAP2K1	c.167A>C	P.Q56P
SH_41	17	48753285	48753285	Silent	SNV	G	T	T	ABCC3	c.2901G>T	P.G967G
SH_41	17	51901132	51901132	Silent	SNV	G	T	T	KIF2B	c.738G>T	P.V246V
SH_41	17	7577534	7577534	Missense_Mutation	SNV	C	A	A	TP53	c.747G>T	P.R249S
SH_41	18	5395656	5395656	Silent	SNV	C	T	T	EPB41L3	c.2517G>A	P.T839T
SH_41	19	17547396	17547396	Missense_Mutation	SNV	G	T	T	TMEM221	c.747C>A	P.S249R
SH_41	2	143742720	143742720	Missense_Mutation	SNV	G	T	T	KYNU	c.797G>T	P.G266V
SH_41	2	150327236	150327236	Missense_Mutation	SNV	C	A	A	LYPD6	c.388C>A	P.P130T
SH_41	2	179553444	179553444	Silent	SNV	G	T	T	TTN	c.31206C>A	P.S10402S
SH_41	2	189867729	189867729	Missense_Mutation	SNV	G	A	A	COL3A1	c.2494G>A	P.E832K
SH_41	2	228881815	228881815	Missense_Mutation	SNV	T	C	C	SPHKAP	c.3755A>G	P.N1252S
SH_41	20	47990211	47990211	Missense_Mutation	SNV	G	A	A	KCNB1	c.1886C>T	P.A629V
SH_41	3	142422812	142422812	Missense_Mutation	SNV	A	T	T	PLS1	c.1474A>T	P.T492S
SH_41	3	167254745	167254745	Missense_Mutation	SNV	T	C	C	WDR49	c.811A>G	P.I271V
SH_41	3	183975398	183975398	Missense_Mutation	SNV	A	G	G	ECE2	c.334A>G	P.M112V
SH_41	5	127610310	127610310	Missense_Mutation	SNV	A	C	C	FBN2	c.7660T>G	P.F2554V
SH_41	5	149927871	149927871	Missense_Mutation	SNV	C	T	T	NDST1	c.2237C>T	P.A746V
SH_41	5	45645361	45645361	Missense_Mutation	SNV	A	T	T	HCN1	c.775T>A	P.F259I
SH_41	6	58287587	58287587	Splice_Site	SNV	C	T	T	LINC00680	n.126+12G>A	
SH_41	7	5553282	5553282	Splice_Site	SNV	C	A	A	FBXL18	c.18+6G>T	
SH_41	7	5643166	5643166	Silent	SNV	G	A	A	FSCN1	c.1029G>A	P.R343R
SH_41	8	145140994	145140994	Missense_Mutation	SNV	G	T	T	GPAA1	c.1832G>T	P.W611L
SH_41	8	22009360	22009360	Silent	SNV	G	A	A	LGI3	c.648C>T	P.F216F
SH_41	8	41575200	41575200	Silent	SNV	G	A	A	ANK1	c.1227C>T	P.H409H
SH_41	9	70918720	70918720	Missense_Mutation	SNV	G	T	T	FOXD4L3	c.853G>T	P.A285S
SH_41	X	47429396	47429396	Silent	SNV	T	G	G	ARAF	c.1524T>G	P.P508P
SH_41	X	54955222	54955222	Missense_Mutation	SNV	A	C	C	TRO	c.2065A>C	P.T689P
SH_42	1	9778973	9778973	Splice_Site	SNV	G	A	A	PIK3CD	c.1242G>A	P.A414A
SH_42	12	46246569	46246569	Missense_Mutation	SNV	G	T	T	ARID2	c.4663G>T	P.A1555S
SH_42	12	57397394	57397394	Silent	SNV	C	A	A	ZBTB39	c.1308G>T	P.L436L
SH_42	15	81631832	81631832	Missense_Mutation	SNV	A	T	T	TMC3	c.1858T>A	P.Y620N
SH_42	17	78914348	78914348	Missense_Mutation	SNV	G	A	A	RPTOR	c.2972G>A	P.R991Q
SH_42	19	44470902	44470902	Missense_Mutation	SNV	C	A	A	ZNF221	c.1248C>A	P.H416Q
SH_42	2	209113113	209113113	Missense_Mutation	SNV	G	A	A	IDH1	c.394C>T	P.R132C
SH_42	2	21231975	21231975	Missense_Mutation	SNV	C	T	T	APOB	c.7765G>A	P.E2589K
SH_42	3	127323538	127323538	Missense_Mutation	SNV	T	A	A	MCM2	c.324T>A	P.S108R
SH_42	4	165118559	165118559	Missense_Mutation	SNV	A	T	T	ANP32C	c.305T>A	P.I102K
SH_42	5	176301435	176301435	Missense_Mutation	SNV	A	C	C	UNC5A	c.1246A>C	P.T416P
SH_42	7	100187952	100187967	Frame_Shift_Del	INDEL	CTGGAAGAG	-	-	FBXO24	5delCTGGAAGAG	P.W88fs
SH_42	X	129168470	129168470	Missense_Mutation	SNV	A	G	G	BCORL1	c.4346A>G	P.K1449R
SH_44	1	152785137	152785137	Missense_Mutation	SNV	G	T	T	LCE1B	c.215G>T	P.G72V
SH_44	1	182523777	182523777	Missense_Mutation	SNV	T	G	G	RGSL1	c.3146T>G	P.L1049R
SH_44	1	201709159	201709159	Missense_Mutation	SNV	A	G	G	NAV1	c.8A>G	P.H3R
SH_44	1	201981144	201981144	Missense_Mutation	SNV	T	C	C	ELF3	c.223T>C	P.W75R
SH_44	1	231411243	231411243	Missense_Mutation	SNV	A	G	G	GNPAT	c.1753A>G	P.I585V
SH_44	1	34083096	34083096	Splice_Site	SNV	T	C	C	CSMD2	c.5946+3A>G	
SH_44	1	3599664	3599664	Missense_Mutation	SNV	C	T	T	TP73	c.106C>T	P.R36W
SH_44	1	37282808	37282808	Silent	SNV	G	A	A	GRIK3	c.1944C>T	P.I648I
SH_44	1	45268672	45268672	Silent	SNV	G	A	A	PLK3	c.795G>A	P.L265L
SH_44	1	46509357	46509357	Silent	SNV	C	T	T	PIK3R3	c.1374G>A	P.S458S
SH_44	10	6520975	6520975	Missense_Mutation	SNV	C	A	A	PRKCQ	c.1332G>T	P.M444I
SH_44	10	89690847	89690847	Splice_Site	SNV	G	A	A	PTEN	c.253+1G>A	
SH_44	11	133816111	133816111	Missense_Mutation	SNV	C	G	G	IGSF9B	c.107G>C	P.R36T
SH_44	11	69588259	69588259	Splice_Site	SNV	G	T	T	FGF4	c.445-6C>A	
SH_44	12	56482340	56482340	Silent	SNV	G	T	T	ERBB3	c.888G>T	P.V296V
SH_44	12	56482341	56482341	Missense_Mutation	SNV	G	T	T	ERBB3	c.889G>T	P.D297Y
SH_44	14	102816331	102816331	Missense_Mutation	SNV	C	T	T	CINP	c.361G>A	P.E121K
SH_44	14	39648393	39648393	Silent	SNV	G	A	A	PNN	c.597G>A	P.R199R
SH_44	15	22382597	22382597	Missense_Mutation	SNV	A	G	G	OR4N4	c.125A>G	P.N42S
SH_44	15	45007706	45007728	Frame_Shift_Del	INDEL	GACATTGAA	-	-	B2M	CCATCCGACATT	P.P52fs
SH_44	15	83331571	83331571	Missense_Mutation	SNV	C	A	A	AP3B2	c.2555G>T	P.G852V
SH_44	15	88423670	88423670	Splice_Site	INDEL	T	-	-	NTRK3	c.2134-11delA	
SH_44	16	30776762	30776762	Missense_Mutation	SNV	A	G	G	RNF40	c.935A>G	P.Y312C
SH_44	16	67911571	67911571	Splice_Site	SNV	G	A	A	EDC4	c.789+12G>A	
SH_44	16	70989370	70989370	Missense_Mutation	SNV	A	T	T	HYDIN	c.6224T>A	P.V2075E
SH_44	17	40043958	40043958	Splice_Site	SNV	T	C	C	ACLY	c.1771-2A>G	
SH_44	17	40937447	40937447	Missense_Mutation	SNV	G	A	A	WNK4	c.415G>A	P.E139K
SH_44	17	41180652	41180652	Missense_Mutation	SNV	G	T	T	RND2	c.639G>T	P.E213D
SH_44	17	4907333	4907333	Missense_Mutation	SNV	A	C	C	KIF1C	c.905A>C	P.D302A
SH_44	17	7578177	7578177	Splice_Site	SNV	C	T	T	TP53	c.672G>A	P.E224E
SH_44	19	1220449	1220449	Missense_Mutation	SNV	A	G	G	STK11	c.542A>G	P.N181S
SH_44	19	48052644	48052644	Missense_Mutation	SNV	G	C	C	ZNF541	c.406C>G	P.Q136E
SH_44	19	50910345	50910345	Nonsense_Mutation	SNV	G	T	T	POLD1	c.1600G>T	P.E534*
SH_44	19	55317424	55317424	Missense_Mutation	SNV	C	T	T	KIR2DL4	c.380C>T	P.S127L
SH_44	19	56703300	56703300	Silent	SNV	C	T	T	ZSCAN5B	c.507G>A	P.V169V
SH_44	19	7976372	7976372	Missense_Mutation	SNV	G	A	A	MAP2K7	c.1036G>A	P.E346K
SH_44	2	113007828	113007828	Silent	SNV	T	C	C	ZC3H8	c.96A>G	P.T32T
SH_44	2	179433009	179433009	Silent	SNV	G	T	T	TTN	c.72927C>A	P.L24309L
SH_44	2	207634902	207634902	Missense_Mutation	SNV	C	G	G	FASTKD2	c.865C>G	P.L289V
SH_44	2	61413571	61413571	Splice_Site	SNV	C	T	T	AHSA2	c.193-10C>T	
SH_44	2	69759291	69759291	Missense_Mutation	SNV	C	G	G	AAK1	c.538G>C	P.E180Q
SH_44	20	40877335	40877335	Missense_Mutation	SNV	G	C	C	PTPRT	c.2361C>G	P.I787M
SH_44	20	60419757	60419757	Missense_Mutation	SNV	C	T	T	CDH4	c.499C>T	P.R167W

SH_44	22	18064136	18064136	Silent	SNV	G	A	A	SLC25A18	c.156G>A	P.L52L
SH_44	22	36962434	36962434	Silent	SNV	G	A	A	CACNG2	c.402C>T	P.I134I
SH_44	22	41360075	41360075	Missense_Mutation	SNV	C	T	T	RBX1	c.182C>T	P.A61V
SH_44	3	130871234	130871234	Nonsense_Mutation	SNV	C	A	A	NEK11	c.650C>A	P.S217*
SH_44	3	13649565	13649565	Missense_Mutation	SNV	C	T	T	FBLN2	c.1310C>T	P.S437F
SH_44	3	4703915	4703915	Missense_Mutation	SNV	G	C	C	ITPR1	c.1401G>C	P.Q467H
SH_44	3	56597721	56597721	Missense_Mutation	SNV	A	G	G	CCDC66	c.10A>G	P.K4E
SH_44	4	123267913	123267913	Missense_Mutation	SNV	C	T	T	KIAA1109	c.12869C>T	P.P4290L
SH_44	4	169182051	169182051	Silent	SNV	T	C	C	DDX60	c.3363A>G	P.L1121L
SH_44	4	169325132	169325132	Frame_Shift_Ins	INDEL	-	A	A	DDX60L	c.3338dupT	P.L1113fs
SH_44	4	66242784	66242784	Splice_Site	SNV	G	A	A	EPHA5	c.1791-3C>T	
SH_44	5	140567922	140567922	Missense_Mutation	SNV	C	A	A	PCDHB9	c.1030C>A	P.P344T
SH_44	5	156770275	156770275	Silent	SNV	G	T	T	FNDC9	c.270C>A	P.T90T
SH_44	5	31294219	31294219	Nonsense_Mutation	SNV	C	T	T	CDH6	c.379C>T	P.R127*
SH_44	5	31532521	31532521	Missense_Mutation	SNV	C	T	T	C5orf22	c.22C>T	P.R8C
SH_44	5	41917285	41917285	Missense_Mutation	SNV	G	A	A	C5orf51	c.769G>A	P.G257R
SH_44	6	13053760	13053760	Frame_Shift_Del	INDEL	A	-	-	PHACTR1	c.414delA	P.A139fs
SH_44	6	28359202	28359202	Missense_Mutation	SNV	G	C	C	ZSCAN12	c.865C>G	P.L289V
SH_44	6	35754907	35754907	Splice_Site	SNV	G	A	A	CLPSL1	c.222+10G>A	
SH_44	7	1489909	1489909	Silent	SNV	G	C	C	MICALL2	c.159C>G	P.L53L
SH_44	7	37355563	37355563	Missense_Mutation	SNV	T	G	G	ELMO1	c.80A>C	P.K27T
SH_44	7	3990590	3990590	Missense_Mutation	SNV	A	G	G	SDK1	c.883A>G	P.I295V
SH_44	7	74197947	74197947	Missense_Mutation	SNV	G	T	T	NCF1	c.654G>T	P.E218D
SH_44	8	11991418	11991418	Missense_Mutation	SNV	C	T	T	USP17L7	c.101G>A	P.R34Q
SH_44	8	144940597	144940597	Silent	SNV	G	A	A	EPPK1	c.6825C>T	P.P2275P
SH_44	8	28360629	28360629	Missense_Mutation	SNV	G	C	C	FZD3	c.99G>C	P.L33F
SH_44	8	41792315	41792315	Missense_Mutation	SNV	A	C	C	KAT6A	c.3423T>G	P.D1141E
SH_44	9	112189399	112189399	Missense_Mutation	SNV	C	T	T	PTPN3	c.832G>A	P.E278K
SH_44	9	117092830	117092830	Silent	SNV	G	T	T	ORM2	c.231G>T	P.T77T
SH_44	9	119449033	119449033	Missense_Mutation	SNV	C	T	T	ASTN2	c.52G>A	P.D18N
SH_44	9	71555718	71555718	Splice_Site	SNV	T	C	C	PIP5K1B	c.1502+12T>C	
SH_44	X	153042370	153042370	Missense_Mutation	SNV	T	G	G	PLXNB3	c.4931T>G	P.V1644G
SH_44	X	47429025	47429025	Missense_Mutation	SNV	T	C	C	ARAF	c.1388T>C	P.L463S
SH_44	X	53106662	53106662	Missense_Mutation	SNV	A	G	G	GPR173	c.859A>G	P.M287V
SH_44	X	79698068	79698068	Silent	SNV	T	G	G	FAM46D	c.30T>G	P.T10T
SH_44	X	82764359	82764359	Missense_Mutation	SNV	C	A	A	POU3F4	c.1027C>A	P.Q343K
SH_44	X	9673033	9673033	Missense_Mutation	SNV	C	T	T	TBL1X	c.1115C>T	P.A372V
SH_46	12	11992223	11992223	Missense_Mutation	SNV	C	G	G	ETV6	c.313C>G	P.R105G
SH_46	12	25398284	25398284	Missense_Mutation	SNV	C	T	T	KRAS	c.35G>A	P.G12D
SH_46	14	96807908	96807908	Frame_Shift_Del	INDEL	A	-	-	ATG2B	c.875delT	P.L292fs
SH_46	2	209113113	209113113	Missense_Mutation	SNV	G	A	A	IDH1	c.394C>T	P.R132C
SH_46	22	30057328	30057328	Splice_Site	INDEL	-	ACCACTGGATAA	ACCACTGGATAA	NF2	TATTAAACCACTGGATAAGAAAATTGAT	
SH_46	3	52668666	52668666	Frame_Shift_Del	INDEL	G	-	-	PBRM1	c.1253delC	P.P418fs
SH_46	4	62849178	62849178	Silent	SNV	G	T	T	ADGRL3	c.2889G>T	P.V963V
SH_46	5	140564296	140564296	Missense_Mutation	SNV	C	T	T	PCDHB16	c.2162C>T	P.S721L
SH_46	6	30075934	30075934	Missense_Mutation	SNV	A	C	C	TRIM31	c.779T>G	P.F260C
SH_46	7	149484874	149484874	Silent	SNV	C	T	T	SSPO	c.3696C>T	P.D1232D
SH_46	X	37431496	37431496	Missense_Mutation	SNV	G	A	A	LANCL3	c.373G>A	P.A125T
SH_48	1	150039960	150039960	Missense_Mutation	SNV	G	A	A	VPS45	c.46G>A	P.E16K
SH_48	1	155448157	155448157	Missense_Mutation	SNV	T	C	C	ASH1L	c.4504A>G	P.M1502V
SH_48	1	156264146	156264146	Splice_Site	SNV	C	G	G	GLMP	c.579+10G>C	
SH_48	1	169509601	169509601	Missense_Mutation	SNV	T	C	C	F5	c.4727A>G	P.N1576S
SH_48	1	224318274	224318274	Missense_Mutation	SNV	A	C	C	FBXO28	c.368A>C	P.Q123P
SH_48	1	225195220	225195220	Missense_Mutation	SNV	T	G	G	DNAH14	c.1081T>G	P.S361A
SH_48	1	228464928	228464928	Missense_Mutation	SNV	C	A	A	OBSCN	c.6668C>A	P.T2223N
SH_48	1	228464929	228464929	Silent	SNV	C	A	A	OBSCN	c.6669C>A	P.T2223T
SH_48	1	231471924	231471924	Missense_Mutation	SNV	T	G	G	EXOC8	c.1568A>C	P.K523T
SH_48	1	247875463	247875463	Missense_Mutation	SNV	C	G	G	OR6F1	c.595G>C	P.A199P
SH_48	1	248128836	248128836	Missense_Mutation	SNV	C	T	T	OR2AK2	c.203C>T	P.T68I
SH_48	10	20106112	20106112	Missense_Mutation	SNV	A	G	G	PLXDC2	c.104A>G	P.Q35R
SH_48	10	267258	267258	Nonsense_Mutation	SNV	A	T	T	ZMYND11	c.400A>T	P.R134*
SH_48	10	27702354	27702354	Missense_Mutation	SNV	T	C	C	PTCHD3	c.826A>G	P.K276E
SH_48	10	28970333	28970333	Missense_Mutation	SNV	A	G	G	BAMBI	c.223A>G	P.S75G
SH_48	10	93619301	93619301	Missense_Mutation	SNV	T	G	G	TNKS2	c.3177T>G	P.I1059M
SH_48	11	1075781	1075781	Silent	SNV	C	A	A	MUC2	c.207C>A	P.G69G
SH_48	11	6636205	6636205	Silent	SNV	A	G	G	TPP1	c.1443T>C	P.F481F
SH_48	11	67401718	67401718	Missense_Mutation	SNV	T	G	G	TBX10	c.491A>C	P.Q164P
SH_48	11	72316235	72316235	Silent	SNV	G	A	A	PDE2A	c.249C>T	P.S83S
SH_48	11	72316236	72316236	Missense_Mutation	SNV	G	C	C	PDE2A	c.248C>G	P.S83C
SH_48	11	85692231	85692231	Missense_Mutation	SNV	C	T	T	PICALM	c.1570G>A	P.G524R
SH_48	11	893426	893426	Splice_Site	SNV	C	T	T	CHID1	c.701+1G>A	
SH_48	12	110940153	110940153	Missense_Mutation	SNV	T	G	G	RAD9B	c.11T>G	P.M4R
SH_48	12	26784800	26784800	Missense_Mutation	SNV	T	C	C	ITPR2	c.2933A>G	P.Q978R
SH_48	13	86369778	86369778	Missense_Mutation	SNV	G	T	T	SLITRK6	c.866C>A	P.S289Y
SH_48	13	99457388	99457388	Missense_Mutation	SNV	C	T	T	DOCK9	c.5626G>A	P.E1876K
SH_48	14	94741772	94741772	Silent	SNV	C	T	T	PPP4R4	c.2511C>T	P.P837P
SH_48	15	40468676	40468676	Splice_Site	SNV	A	C	C	BUB1B	c.385-2A>C	
SH_48	15	45404807	45404807	Silent	SNV	C	A	A	DUOX2	c.270G>T	P.R90R
SH_48	16	30748863	30748863	Missense_Mutation	SNV	C	G	G	SRCAP	c.7502C>G	P.P2501R
SH_48	16	8875190	8875190	Missense_Mutation	SNV	A	G	G	ABAT	c.1406A>G	P.D469G
SH_48	17	2281170	2281170	Missense_Mutation	SNV	T	G	G	SGSM2	c.2687T>G	P.F896C
SH_48	17	73847671	73847671	Missense_Mutation	SNV	G	A	A	WBP2	c.146C>T	P.T49I
SH_48	17	8349126	8349126	Missense_Mutation	SNV	A	C	C	NDEL1	c.184A>C	P.N62H
SH_48	18	14851748	14851748	Missense_Mutation	SNV	G	A	A	ANKRD30B	c.3448G>A	P.V1150I
SH_48	19	21607171	21607171	Missense_Mutation	SNV	C	G	G	ZNF493	c.1710C>G	P.H570Q
SH_48	19	32968420	32968420	Splice_Site	SNV	T	C	C	DPY19L3	c.1698-8T>C	
SH_48	19	33424486	33424486	Missense_Mutation	SNV	T	C	C	CEP89	c.757A>G	P.M253V
SH_48	19	39908540	39908540	Splice_Site	SNV	G	A	A	PLEKHG2	c.883-5G>A	
SH_48	19	42126042	42126042	Splice_Site	SNV	C	T	T	CEACAM4	c.669+1G>A	
SH_48	19	42911506	42911506	Missense_Mutation	SNV	C	T	T	LIPE	c.1957G>A	P.G653S
SH_48	19	45867689	45867689	Missense_Mutation	SNV	G	C	C	ERCC2	c.711C>G	P.H237Q
SH_48	19	46144940	46144940	Silent	SNV	C	A	A	EML2	c.582G>T	P.R194R
SH_48	19	46144941	46144941	Missense_Mutation	SNV	C	A	A	EML2	c.581G>T	P.R194L
SH_48	19	52569023	52569023	Silent	SNV	A	G	G	ZNF841	c.2112T>C	P.T704T
SH_48	19	5265071	5265071	Missense_Mutation	SNV	G	C	C	PTPRS	c.516C>G	P.F172L
SH_48	2	128941363	128941363	Splice_Site	SNV	A	G	G	UGGT1	c.4359A>G	P.Q1453Q
SH_48	2	1695711	1695711	Missense_Mutation	SNV	A	C	C	PXDN	c.333T>G	P.N111K
SH_48	20	40162094	40162094	Missense_Mutation	SNV	T	C	C	CHD6	c.149A>G	P.D50G

SH_48	22	20100738	20100738	Silent	SNV	G	A	A	TRMT2A	c.1452C>T	P.F484F
SH_48	3	15731711	15731711	Missense_Mutation	SNV	T	C	C	ANKRD28	c.1226A>G	P.H409R
SH_48	3	41942261	41942261	Missense_Mutation	SNV	T	G	G	ULK4	c.1243A>C	P.T415P
SH_48	5	10748320	10748320	Missense_Mutation	SNV	T	G	G	DAP	c.119A>C	P.K40T
SH_48	5	65455039	65455039	Splice_Site	SNV	C	G	G	SREK1	c.296-8C>G	
SH_48	5	66391445	66391445	Missense_Mutation	SNV	C	G	G	MAST4	c.854C>G	P.S285W
SH_48	5	68840897	68840897	Missense_Mutation	SNV	T	C	C	OCLN	c.1204T>C	P.Y402H
SH_48	6	18207631	18207631	Splice_Site	SNV	A	C	C	KDM1B	c.966A>C	P.V322V
SH_48	7	129098559	129098559	Missense_Mutation	SNV	G	T	T	STRIP2	c.1281G>T	P.K427N
SH_48	7	51150927	51150927	Splice_Site	SNV	T	C	C	COBL	c.1097-2A>G	
SH_48	7	75610920	75610920	Missense_Mutation	SNV	T	C	C	POR	c.727T>C	P.S243P
SH_48	7	896012	896012	Silent	SNV	A	G	G	SUN1	c.1368A>G	P.L456L
SH_48	8	142185524	142185524	Missense_Mutation	SNV	G	T	T	DENND3	c.2261G>T	P.G754V
SH_48	8	145678610	145678610	Silent	SNV	C	T	T	CYHR1	c.480G>A	P.Q160Q
SH_48	8	2823298	2823298	Missense_Mutation	SNV	T	A	A	CSMD1	c.9279A>T	P.K3093N
SH_48	8	2823300	2823300	Missense_Mutation	SNV	T	G	G	CSMD1	c.9277A>C	P.K3093Q
SH_48	8	48879955	48879955	Missense_Mutation	SNV	C	T	T	MCM4	c.1084C>T	P.P362S
SH_48	9	4849475	4849475	Missense_Mutation	SNV	G	A	A	RCL1	c.422G>A	P.S141N
SH_48	X	70443741	70443741	Missense_Mutation	SNV	A	T	T	GJB1	c.184A>T	P.S62C
SH_48	X	7889816	7889816	Missense_Mutation	SNV	T	A	A	PNPLA4	c.349A>T	P.N117Y
SH_50	1	100345543	100345543	Missense_Mutation	SNV	G	T	T	AGL	c.1676G>T	P.G559V
SH_50	1	164761941	164761941	Missense_Mutation	SNV	A	G	G	PBX1	c.476A>G	P.Q159R
SH_50	1	236433273	236433273	Missense_Mutation	SNV	A	G	G	ERO1B	c.124T>C	P.C42R
SH_50	1	2408030	2408030	Nonsense_Mutation	SNV	G	T	T	PLCH2	c.3G>T	P.M1?
SH_50	10	71142485	71142485	Missense_Mutation	SNV	C	T	T	HK1	c.1508C>T	P.T503M
SH_50	12	58121327	58121327	Missense_Mutation	SNV	A	G	G	AGAP2	c.2896T>C	P.C966R
SH_50	13	107220055	107220055	Silent	SNV	G	A	A	ARGLU1	c.213C>T	P.D71D
SH_50	13	99555311	99555311	Missense_Mutation	SNV	C	T	T	DOCK9	c.1091G>A	P.G364E
SH_50	17	10442778	10442778	Missense_Mutation	SNV	C	T	T	MYH2	c.1249G>A	P.G417S
SH_50	17	41959644	41959644	Splice_Site	SNV	G	A	A	MPP2	c.816+8C>T	
SH_50	17	62525490	62525490	Missense_Mutation	SNV	A	C	C	CEP95	c.899A>C	P.E300A
SH_50	17	7577498	7577498	Splice_Site	SNV	C	A	A	TP53	c.782+1G>T	
SH_50	2	10059883	10059883	Missense_Mutation	SNV	A	G	G	TAF1B	c.734A>G	P.E245G
SH_50	2	177036509	177036509	Missense_Mutation	SNV	G	T	T	HOXD3	c.806G>T	P.R269L
SH_50	20	61527574	61527574	Splice_Site	SNV	C	T	T	DIDO1	c.2214+11G>A	
SH_50	21	15599454	15599454	Frame_Shift_Del	INDEL	A	-	-	RBM11	c.707delA	P.Y236fs
SH_50	22	21133604	21133604	Nonsense_Mutation	SNV	A	T	T	SERPIND1	c.4A>T	P.K2*
SH_50	3	120123719	120123719	Missense_Mutation	SNV	C	G	G	FSTL1	c.562G>C	P.E188Q
SH_50	3	56026112	56026112	Missense_Mutation	SNV	T	C	C	ERC2	c.2228A>G	P.K743R
SH_50	5	1294023	1294023	Silent	SNV	G	T	T	TERT	c.978C>A	P.A326A
SH_50	8	113323360	113323360	Nonsense_Mutation	SNV	C	A	A	CSMD3	c.7420G>T	P.G2474*
SH_50	8	120598546	120598546	Missense_Mutation	SNV	T	A	A	ENPP2	c.1247A>T	P.K416I
SH_50	8	141745351	141745351	Missense_Mutation	SNV	T	A	A	PTK2	c.2029A>T	P.S677C
SH_50	9	140777321	140777321	Silent	SNV	G	T	T	CACNA1B	c.516G>T	P.V172V
SH_50	X	100748157	100748157	Silent	SNV	T	C	C	ARMCX4	c.4581T>C	P.S1527S
SH_50	X	17819198	17819198	Silent	SNV	T	A	A	RAI2	c.783A>T	P.G261G
SH_50	X	3228746	3228746	Missense_Mutation	SNV	T	G	G	MXRA5	c.7498A>C	P.N2500H
SH_50	X	70361781	70361781	Missense_Mutation	SNV	G	A	A	MED12	c.6457G>A	P.A2153T
SH_51	1	157509008	157509008	Missense_Mutation	SNV	T	A	A	FCRL5	c.1270A>T	P.R424W
SH_51	1	171688329	171688329	Missense_Mutation	SNV	C	G	G	VAMP4	c.143G>C	P.R48T
SH_51	1	186008081	186008081	Missense_Mutation	SNV	G	T	T	HMCN1	c.5972G>T	P.C1991F
SH_51	1	186141211	186141211	Silent	SNV	C	T	T	HMCN1	c.15762C>T	P.C5254C
SH_51	1	241667429	241667429	Missense_Mutation	SNV	C	A	A	FH	c.1021G>T	P.D341Y
SH_51	1	3643789	3643789	Splice_Site	SNV	G	T	T	TP73	c.695+1G>T	
SH_51	1	47075713	47075713	Silent	SNV	G	A	A	MOB3C	c.738C>T	P.R246R
SH_51	1	55509596	55509596	Silent	SNV	C	T	T	PCSK9	c.288C>T	P.R96R
SH_51	1	77510056	77510056	Silent	SNV	C	A	A	ST6GALNAC5	c.429C>A	P.V143V
SH_51	10	114057904	114057904	Missense_Mutation	SNV	G	A	A	TECTB	c.749G>A	P.R250Q
SH_51	11	122944224	122944224	Silent	SNV	G	T	T	CLMP	c.1080C>A	P.P360P
SH_51	11	5841654	5841654	Missense_Mutation	SNV	C	A	A	OR52N2	c.89C>A	P.S30Y
SH_51	11	61917614	61917614	Missense_Mutation	SNV	T	G	G	INCENP	c.2477T>G	P.L826R
SH_51	11	67352618	67352618	Missense_Mutation	SNV	G	A	A	GSTP1	c.242G>A	P.G81E
SH_51	12	105557961	105557961	Missense_Mutation	SNV	G	C	C	KIAA1033	c.3233G>C	P.R1078T
SH_51	12	21997857	21997857	Splice_Site	SNV	G	A	A	ABCC9	c.3097-8C>T	
SH_51	13	36158140	36158140	Missense_Mutation	SNV	A	G	G	NBEA	c.520A>G	P.T174A
SH_51	14	75590874	75590874	Missense_Mutation	SNV	T	C	C	NEK9	c.272A>G	P.K91R
SH_51	15	56736697	56736697	Missense_Mutation	SNV	T	C	C	MNS1	c.631A>G	P.K211E
SH_51	15	80452129	80452129	Missense_Mutation	SNV	A	T	T	FAH	c.224A>T	P.Q75L
SH_51	15	85165000	85165000	Missense_Mutation	SNV	C	A	A	ZSCAN2	c.1574C>A	P.T525K
SH_51	16	2263877	2263877	Missense_Mutation	SNV	C	A	A	PGP	c.818G>T	P.G273V
SH_51	16	309997	309997	Missense_Mutation	SNV	G	T	T	FAM234A	c.415G>T	P.A139S
SH_51	17	67252332	67252332	Missense_Mutation	SNV	A	T	T	ABCA5	c.3824T>A	P.V1275D
SH_51	17	74474996	74474996	Silent	SNV	C	T	T	RHBDF2	c.651G>A	P.L217L
SH_51	17	7577534	7577534	Missense_Mutation	SNV	C	A	A	TP53	c.747G>T	P.R249S
SH_51	18	44121743	44121743	Silent	SNV	T	C	C	LOXHD1	c.576A>G	P.T192T
SH_51	18	5419865	5419865	Missense_Mutation	SNV	C	A	A	EPB41L3	c.1405G>T	P.G469C
SH_51	19	13445238	13445238	Missense_Mutation	SNV	C	G	G	CACNA1A	c.1152G>C	P.Q384H
SH_51	19	24115260	24115260	Frame_Shift_Del	INDEL	A	-	-	ZNF726	c.345delA	P.G116fs
SH_51	2	230724031	230724031	Nonsense_Mutation	SNV	G	A	A	TRIP12	c.484C>T	P.Q162*
SH_51	20	39802378	39802378	Missense_Mutation	SNV	G	T	T	PLCG1	c.3481G>T	P.V1161L
SH_51	20	53208187	53208187	Missense_Mutation	SNV	A	C	C	DOK5	c.118A>C	P.N40H
SH_51	20	62323101	62323101	Missense_Mutation	SNV	A	T	T	RTEL1	c.2563A>T	P.S855C
SH_51	22	20780311	20780311	Missense_Mutation	SNV	C	G	G	SCARF2	c.1967G>C	P.R656P
SH_51	3	49845239	49845239	Missense_Mutation	SNV	C	T	T	UBA7	c.2645G>A	P.R882H
SH_51	4	10083071	10083082	Splice_Site	INDEL	TTAGAGAGA	-	-	WDR1	_777-3delTTTCTCTCTAAC	
SH_51	4	155489625	155489625	Missense_Mutation	SNV	G	A	A	FGB	c.634G>A	P.D212N
SH_51	4	190947588	190947588	Silent	SNV	G	A	A	FRG2	c.282C>T	P.N94N
SH_51	4	48073630	48073630	Missense_Mutation	SNV	C	A	A	TXK	c.1419G>T	P.L473F
SH_51	6	158873184	158873184	Missense_Mutation	SNV	C	G	G	TULP4	c.743C>G	P.P248R
SH_51	6	33245650	33245655	Missense_Mutation	INDEL	CTAAAG	-	-	B3GALT4	54_459delCTAA	P.L152_K153del
SH_51	7	146825887	146825887	Missense_Mutation	SNV	A	G	G	CNTNAP2	c.1042A>G	P.T348A
SH_51	7	151904477	151904477	Missense_Mutation	SNV	G	C	C	KMT2C	c.3749C>G	P.S1250C
SH_51	7	44797630	44797630	Missense_Mutation	SNV	C	A	A	ZMIZ2	c.640C>A	P.L214M
SH_51	7	69599520	69599520	Splice_Site	SNV	A	G	G	AUTS2	c.625-2A>G	
SH_51	8	101178075	101178075	Silent	SNV	A	G	G	SPAG1	c.174A>G	P.T58T
SH_51	8	121301897	121301897	Missense_Mutation	SNV	G	C	C	COL14A1	c.4128G>C	P.K1376N
SH_51	8	143624772	143624772	Silent	SNV	G	A	A	ADGRB1	c.4422G>A	P.K1474K
SH_51	8	1497807	1497807	Missense_Mutation	SNV	C	A	A	DLGAP2	c.948C>A	P.H316Q

SH_51	8	25220572	25220572	Missense_Mutation	SNV	A	G	G	DOCK5	c.2959A>G	P.M987V
SH_51	8	67488461	67488461	Silent	SNV	C	T	T	MYBL1	c.1251G>A	P.G417G
SH_51	9	138712974	138712974	Missense_Mutation	SNV	C	A	A	CAMSAP1	c.3533G>T	P.R1178L
SH_51	X	19398339	19398339	Missense_Mutation	SNV	C	G	G	MAP3K15	c.2488G>C	P.A830P
SH_52	1	150310780	150310780	Missense_Mutation	SNV	A	G	G	PRPF3	c.1180A>G	P.I394V
SH_52	1	226026499	226026499	Missense_Mutation	SNV	A	G	G	EPHX1	c.509A>G	P.H170R
SH_52	1	248813579	248813579	Missense_Mutation	SNV	A	T	T	OR2T27	c.607T>A	P.C203S
SH_52	10	27964267	27964267	Missense_Mutation	SNV	A	T	T	MKX	c.950T>A	P.L317H
SH_52	11	64398026	64398026	Missense_Mutation	SNV	A	T	T	NRXN2	c.3605T>A	P.V1202E
SH_52	12	22354673	22354673	Missense_Mutation	SNV	A	G	G	ST8SIA1	c.455T>C	P.F152S
SH_52	12	96912713	96912713	Missense_Mutation	SNV	A	T	T	CFAP54	c.806A>T	P.E269V
SH_52	14	23896835	23896835	Missense_Mutation	SNV	A	T	T	MYH7	c.1847T>A	P.L616Q
SH_52	14	75329483	75329483	Missense_Mutation	SNV	T	A	A	PROX2	c.1055A>T	P.H352L
SH_52	15	73996270	73996270	Missense_Mutation	SNV	A	T	T	CD276	c.1004A>T	P.E335V
SH_52	15	99874205	99874205	Missense_Mutation	SNV	A	T	T	LRRC28	c.463A>T	P.M155L
SH_52	2	130930883	130930883	Missense_Mutation	SNV	T	C	C	SMPD4	c.431A>G	P.Y144C
SH_52	2	168726411	168726411	Missense_Mutation	SNV	T	A	A	B3GALT1	c.862T>A	P.W288R
SH_52	2	206884550	206884550	Missense_Mutation	SNV	T	A	A	INO80D	c.1318A>T	P.R440W
SH_52	2	212248411	212248411	Missense_Mutation	SNV	A	G	G	ERBB4	c.3856T>C	P.S1286P
SH_52	2	86831392	86831392	Silent	SNV	T	G	G	RNF103	c.1620A>C	P.T540T
SH_52	3	155205805	155205805	Splice_Site	SNV	G	A	A	PLCH1	c.2583+12C>T	
SH_52	3	16635220	16635220	Missense_Mutation	SNV	A	T	T	DAZL	c.737T>A	P.V246E
SH_52	3	167189420	167189420	Missense_Mutation	SNV	T	A	A	SERPINI2	c.233A>T	P.Q78L
SH_52	4	146807071	146807071	Missense_Mutation	SNV	C	T	T	ZNF827	c.1506G>A	P.M502I
SH_52	5	13721242	13721242	Missense_Mutation	SNV	A	T	T	DNAH5	c.12146T>A	P.L4049Q
SH_52	5	140215289	140215289	Missense_Mutation	SNV	G	A	A	PCDHA7	c.1321G>A	P.V441M
SH_52	5	140574441	140574441	Silent	SNV	T	A	A	PCDHB10	c.2316T>A	P.V772V
SH_52	5	140751778	140751778	Missense_Mutation	SNV	A	T	T	PCDHGB3	c.1817A>T	P.Y606F
SH_52	5	140774176	140774176	Missense_Mutation	SNV	G	T	T	PCDHGA8	c.1796G>T	P.G599V
SH_52	5	16676268	16676268	Splice_Site	SNV	A	T	T	MYO10	c.4543-5T>A	
SH_52	5	33984538	33984538	Missense_Mutation	SNV	C	A	A	SLC45A2	c.151G>T	P.V51L
SH_52	5	7893003	7893003	Missense_Mutation	SNV	A	T	T	MTRR	c.1534A>T	P.S512C
SH_52	6	34825597	34825597	Missense_Mutation	SNV	A	T	T	UHRF1BP1	c.1670A>T	P.Y557F
SH_52	6	38548105	38548105	Missense_Mutation	SNV	C	A	A	BTBD9	c.923G>T	P.G308V
SH_52	6	38862563	38862563	Silent	SNV	A	T	T	DNAH8	c.8670A>T	P.I2890I
SH_52	6	45882026	45882026	Missense_Mutation	SNV	A	T	T	CLIC5	c.1004T>A	P.L335Q
SH_52	7	94139506	94139506	Missense_Mutation	SNV	A	C	C	CASD1	c.110A>C	P.H37P
SH_52	7	97873920	97873920	Missense_Mutation	SNV	C	A	A	TECPR1	c.494G>T	P.R165L
SH_52	8	104922697	104922697	Silent	SNV	C	T	T	RIMS2	c.1197C>T	P.D399D
SH_52	8	29195990	29195990	Missense_Mutation	SNV	A	T	T	DUSP4	c.608T>A	P.L203H
SH_52	8	82356823	82356823	Missense_Mutation	SNV	A	T	T	PMP2	c.260T>A	P.L87Q
SH_52	9	133230220	133230220	Silent	SNV	G	T	T	HMCN2	c.3261G>T	P.A1087A
SH_52	9	136405769	136405769	Silent	SNV	C	T	T	ADAMTSL2	c.462C>T	P.T154T
SH_53	1	156498302	156498302	Missense_Mutation	SNV	G	A	A	IQGAP3	c.4672C>T	P.H1558Y
SH_53	1	16464659	16464659	Frame_Shift_Ins	INDEL	-	A	A	EPHA2	c.1000dupT	P.Y334fs
SH_53	1	197111506	197111506	Missense_Mutation	SNV	T	C	C	ASPM	c.1876A>G	P.K626E
SH_53	1	27105550	27105550	Nonsense_Mutation	SNV	C	T	T	ARID1A	c.5161C>T	P.R1721*
SH_53	10	13656040	13656040	Missense_Mutation	SNV	C	T	T	PRPF18	c.746C>T	P.S249L
SH_53	12	112318261	112318261	Missense_Mutation	SNV	C	T	T	MAPKAPK5	c.590C>T	P.A197V
SH_53	12	113533132	113533132	Splice_Site	SNV	C	T	T	DTX1	c.1551C>T	P.G517G
SH_53	12	41967566	41967566	Missense_Mutation	SNV	G	A	A	PDZRN4	c.2985G>A	P.M995I
SH_53	12	71003122	71003122	Splice_Site	SNV	G	A	A	PTPRB	c.709-3C>T	
SH_53	14	21897454	21897454	Missense_Mutation	SNV	T	G	G	CHD8	c.884A>C	P.Q295P
SH_53	16	16291888	16291888	Missense_Mutation	SNV	T	C	C	ABCC6	c.1328A>G	P.Y443C
SH_53	16	67696157	67696157	Silent	SNV	C	A	A	PARD6A	c.645C>A	P.L215L
SH_53	16	71127847	71127847	Splice_Site	SNV	A	G	G	HYDIN	c.1409-9T>C	
SH_53	17	17700957	17700957	Silent	SNV	G	T	T	RAI1	c.4695G>T	P.V1565V
SH_53	17	5436190	5436190	Missense_Mutation	SNV	G	A	A	NLRP1	c.3260C>T	P.T1087M
SH_53	17	54969228	54969228	Missense_Mutation	SNV	G	A	A	TRIM25	c.1726C>T	P.R576W
SH_53	17	57951937	57951937	Missense_Mutation	SNV	C	G	G	TUBD1	c.249G>C	P.L83F
SH_53	19	11258684	11258684	Splice_Site	SNV	C	A	A	SPC24	c.410+5G>T	
SH_53	19	14066781	14066781	Missense_Mutation	SNV	G	T	T	DCAF15	c.424G>T	P.V142L
SH_53	19	36360590	36360590	Missense_Mutation	SNV	G	T	T	APLP1	c.169G>T	P.A57S
SH_53	19	46886660	46886660	Splice_Site	SNV	C	T	T	PPP5C	c.634-8C>T	
SH_53	19	57286803	57286803	Silent	SNV	C	T	T	ZIM2	c.837G>A	P.P279P
SH_53	2	226378249	226378249	Missense_Mutation	SNV	C	A	A	NYAP2	c.384C>A	P.D128E
SH_53	2	75118019	75118019	Missense_Mutation	SNV	C	T	T	HK2	c.2705C>T	P.A902V
SH_53	22	38895436	38895436	Silent	SNV	G	A	A	DDX17	c.507C>T	P.P169P
SH_53	3	75788489	75788489	Silent	SNV	C	T	T	ZNF717	c.285G>A	P.Q95Q
SH_53	4	1806099	1806099	Missense_Mutation	SNV	A	G	G	FGFR3	c.1118A>G	P.Y373C
SH_53	5	173426809	173426809	Splice_Site	SNV	T	C	C	C5orf47	c.511+7T>C	
SH_53	5	36679852	36679852	Silent	SNV	C	T	T	SLC1A3	c.984C>T	P.H328H
SH_53	6	10695230	10695230	Silent	SNV	C	G	G	PAK1IP1	c.12C>G	P.V4V
SH_53	6	78632903	78632903	Missense_Mutation	SNV	C	T	T	MEI4	c.998C>T	P.T333I
SH_53	7	149503850	149503850	Missense_Mutation	SNV	G	A	A	SSPO	c.8678G>A	P.R2893H
SH_53	7	57522840	57522840	Silent	SNV	T	C	C	ZNF716	c.228T>C	P.N76N
SH_53	8	65527782	65527782	Silent	SNV	A	G	G	CYP7B1	c.858T>C	P.H286H
SH_53	9	18777780	18777780	Silent	SNV	C	T	T	ADAMTSL1	c.3553C>T	P.L1185L
SH_56	1	175323607	175323607	Missense_Mutation	SNV	T	C	C	TNR	c.2303A>G	P.E768G
SH_56	1	27092812	27092812	Nonsense_Mutation	SNV	G	T	T	ARID1A	c.2833G>T	P.G945*
SH_56	1	64515434	64515434	Missense_Mutation	SNV	T	C	C	ROR1	c.235T>C	P.C79R
SH_56	10	54031200	54031200	Missense_Mutation	SNV	C	T	T	PRKG1	c.1219C>T	P.R407C
SH_56	11	16362672	16362672	Silent	SNV	C	T	T	SOX6	c.123G>A	P.L41L
SH_56	11	26660711	26660711	Missense_Mutation	SNV	G	C	C	ANO3	c.2247G>C	P.L749F
SH_56	12	6972503	6972503	Missense_Mutation	SNV	A	G	G	USP5	c.1916A>G	P.E639G
SH_56	13	24874584	24874584	Missense_Mutation	SNV	C	T	T	SPATA13	c.3629C>T	P.A1210V
SH_56	13	75886884	75886884	Missense_Mutation	SNV	C	A	A	TBC1D4	c.2349G>T	P.M783I
SH_56	13	90883530	90883530	Splice_Site	SNV	C	A	A	MIR622	n.95C>A	
SH_56	16	57778317	57778317	Silent	SNV	C	T	T	KATNB1	c.183C>T	P.G61G
SH_56	17	30202297	30202297	Missense_Mutation	SNV	T	C	C	UTP6	c.1261A>G	P.I421V
SH_56	17	7579485	7579485	Nonsense_Mutation	SNV	C	A	A	TP53	c.202G>T	P.E68*
SH_56	2	162875305	162875305	Nonsense_Mutation	SNV	C	A	A	DPP4	c.1354G>T	P.E452*
SH_56	20	45865227	45865227	Missense_Mutation	SNV	G	C	C	ZMYND8	c.2486C>G	P.S829C
SH_56	22	18609267	18609267	Silent	SNV	C	T	T	TUBA8	c.324C>T	P.A108A
SH_56	22	29138118	29138118	Missense_Mutation	SNV	T	G	G	HSCB	c.35T>G	P.V12G
SH_56	3	122869138	122869138	Missense_Mutation	SNV	G	T	T	PDIA5	c.1200G>T	P.L400F
SH_56	6	29275027	29275027	Silent	SNV	T	C	C	OR14J1	c.561T>C	P.C187C
SH_56	7	129037061	129037061	Splice_Site	SNV	A	T	T	AHCYL2	c.718-2A>T	

SH_56	8	3226842	3226842	Missense_Mutation	SNV	C	A	A	CSMD1	c.2833G>T	P.G945W
SH_56	8	77766065	77766065	Missense_Mutation	SNV	G	A	A	ZFHX4	c.6908G>A	P.R2303Q
SH_56	X	69385729	69385729	Silent	SNV	G	A	A	IGBP1	c.918G>A	P.K306K
SH_58	1	110587495	110587495	Missense_Mutation	SNV	C	A	A	STRIP1	c.1035C>A	P.S345R
SH_58	1	145562056	145562056	Missense_Mutation	SNV	G	A	A	ANKRD35	c.1474G>A	P.E492K
SH_58	1	167409971	167409971	Missense_Mutation	SNV	A	T	T	CD247	c.92T>A	P.L31H
SH_58	1	17698833	17698833	Silent	SNV	A	T	T	PADI6	c.93A>T	P.T31T
SH_58	1	1854415	1854415	Splice_Site	SNV	T	A	A	CFAP74	c.4683+9A>T	
SH_58	1	207072737	207072737	Silent	SNV	C	A	A	IL24	c.120C>A	P.T40T
SH_58	1	22033256	22033256	Missense_Mutation	SNV	T	C	C	USP48	c.2069A>G	P.E690G
SH_58	1	226175617	226175617	Nonsense_Mutation	SNV	C	A	A	SDE2	c.1114G>T	P.E372*
SH_58	1	230907846	230907846	Splice_Site	SNV	G	T	T	CAPN9	c.686+1G>T	
SH_58	1	248343929	248343929	Silent	SNV	C	T	T	OR2M2	c.642C>T	P.I214I
SH_58	1	248436229	248436229	Silent	SNV	C	T	T	OR2T33	c.888G>A	P.K296K
SH_58	1	37271916	37271916	Silent	SNV	G	A	A	GRIK3	c.2103C>T	P.I701I
SH_58	1	41094539	41094539	Silent	SNV	A	G	G	RIMS3	c.657T>C	P.D219D
SH_58	1	52947569	52947569	Missense_Mutation	SNV	A	T	T	ZCCHC11	c.1542T>A	P.D514E
SH_58	1	53544202	53544202	Silent	SNV	G	A	A	PODN	c.1107G>A	P.L369L
SH_58	10	125528200	125528200	Missense_Mutation	SNV	T	C	C	CPXM2	c.1141A>G	P.N381D
SH_58	10	81319120	81319120	Silent	SNV	G	C	C	SFTPA2	c.120C>G	P.G40G
SH_58	11	119206919	119206919	Missense_Mutation	SNV	G	A	A	RNF26	c.1087G>A	P.G363S
SH_58	11	123988417	123988417	Silent	SNV	C	T	T	VWA5A	c.81C>T	P.Y27Y
SH_58	11	28116225	28116225	Nonsense_Mutation	SNV	C	A	A	KIF18A	c.448G>T	P.E150*
SH_58	11	36614684	36614684	Missense_Mutation	SNV	G	C	C	RAG2	c.1035C>G	P.F345L
SH_58	11	56237071	56237071	Silent	SNV	T	C	C	OR5M3	c.903A>G	P.K301K
SH_58	11	66838987	66838987	Missense_Mutation	SNV	T	A	A	RHOD	c.349T>A	P.F117I
SH_58	12	11149831	11149831	Missense_Mutation	SNV	C	T	T	TAS2R20	c.644G>A	P.G215E
SH_58	12	124840118	124840118	Splice_Site	SNV	C	A	A	NCOR2	c.3212-1G>T	
SH_58	12	49745126	49745127	Frame_Shift_Del	INDEL	AA	-	-	DNAJC22	c.868_869delAA	P.N290fs
SH_58	12	52886456	52886456	Nonsense_Mutation	SNV	T	A	A	KRT6A	c.517A>T	P.K173*
SH_58	12	57422907	57422907	Splice_Site	SNV	T	A	A	MYO1A	c.3005+9A>T	
SH_58	12	58089821	58089821	Splice_Site	SNV	G	A	A	OS9	c.480G>A	P.K160K
SH_58	14	105416790	105416790	Frame_Shift_Del	INDEL	T	-	-	AHNAK2	c.4998delA	P.S1667fs
SH_58	14	44975880	44975880	Missense_Mutation	SNV	G	A	A	FSCB	c.311C>T	P.A104V
SH_58	15	25953373	25953373	Missense_Mutation	SNV	C	A	A	ATP10A	c.2419G>T	P.G807C
SH_58	15	31229467	31229467	Splice_Site	SNV	G	A	A	FAN1	c.*3+5G>A	
SH_58	15	43308034	43308034	Missense_Mutation	SNV	T	C	C	UBR1	c.3061A>G	P.R1021G
SH_58	15	48527135	48527135	Silent	SNV	C	A	A	SLC12A1	c.1149C>A	P.V383V
SH_58	15	91035806	91035806	Silent	SNV	G	A	A	IQGAP1	c.4491G>A	P.Q1497Q
SH_58	16	28113251	28113251	Missense_Mutation	SNV	A	C	C	XPO6	c.2920T>G	P.F974V
SH_58	16	50744570	50744570	Missense_Mutation	SNV	G	T	T	NOD2	c.667G>T	P.D223Y
SH_58	16	50744571	50744571	Missense_Mutation	SNV	A	T	T	NOD2	c.668A>T	P.D223V
SH_58	16	74425905	74425905	Missense_Mutation	SNV	G	C	C	NPIPB15	c.1259G>C	P.R420T
SH_58	17	10363305	10363305	Silent	SNV	G	A	A	MYH4	c.1380C>T	P.I460I
SH_58	17	26938585	26938585	Missense_Mutation	SNV	C	A	A	SGK494	c.811G>T	P.A271S
SH_58	17	27001380	27001380	Missense_Mutation	SNV	T	A	A	SUPT6H	c.189T>A	P.D63E
SH_58	17	27936012	27936012	Missense_Mutation	SNV	G	T	T	ANKRD13B	c.565G>T	P.D189Y
SH_58	17	36622418	36622418	Missense_Mutation	SNV	A	T	T	ARHGAP23	c.494A>T	P.Q165L
SH_58	17	40061912	40061912	Splice_Site	SNV	C	T	T	ACLY	c.867-1G>A	
SH_58	17	61511848	61511848	Nonsense_Mutation	SNV	C	T	T	CYB561	c.671G>A	P.W224*
SH_58	17	7578224	7578224	Nonsense_Mutation	SNV	T	A	A	TP53	c.625A>T	P.R209*
SH_58	17	76456070	76456070	Missense_Mutation	SNV	T	A	A	DNAH17	c.9449A>T	P.E3150V
SH_58	18	55319709	55319709	Splice_Site	SNV	C	A	A	ATP8B1	c.3261+7G>T	
SH_58	18	9887580	9887580	Silent	SNV	G	A	A	TXNDC2	c.1104G>A	P.Q368Q
SH_58	19	19035081	19035081	Missense_Mutation	SNV	C	G	G	DDX49	c.816C>G	P.S272R
SH_58	19	36394686	36394686	Missense_Mutation	SNV	G	T	T	HCST	c.122G>T	P.G41V
SH_58	19	4164504	4164504	Missense_Mutation	SNV	A	T	T	CREB3L3	c.578A>T	P.Q193L
SH_58	19	44981055	44981055	Missense_Mutation	SNV	G	A	A	ZNF180	c.1568C>T	P.P523L
SH_58	19	7620180	7620180	Frame_Shift_Del	INDEL	A	-	-	PNPLA6	c.2893delA	P.S965fs
SH_58	2	109383358	109383358	Silent	SNV	G	A	A	RANBP2	c.6363G>A	P.Q2121Q
SH_58	2	160139338	160139338	Frame_Shift_Del	INDEL	C	-	-	WDSUB1	c.243delG	P.W81fs
SH_58	2	179427165	179427165	Nonsense_Mutation	SNV	A	T	T	TTN	c.78771T>A	P.Y26257*
SH_58	2	179498064	179498064	Splice_Site	SNV	A	T	T	TTN	c.38024-11T>A	
SH_58	2	179542355	179542355	Silent	SNV	A	T	T	TTN	c.33333T>A	P.P11111P
SH_58	2	179611206	179611206	Silent	SNV	A	T	T	TTN	c.15921T>A	P.P5307P
SH_58	2	179997145	179997145	Missense_Mutation	SNV	G	T	T	SESTD1	c.858C>A	P.N286K
SH_58	2	220333672	220333672	Silent	SNV	G	A	A	SPEG	c.3393G>A	P.E1131E
SH_58	2	228104959	228104959	Splice_Site	SNV	C	T	T	COL4A3	c.234+11C>T	
SH_58	2	95513760	95513760	Splice_Site	SNV	A	G	G	ANKRD20A8P	n.643+2T>C	
SH_58	2	96781498	96781498	Missense_Mutation	SNV	A	T	T	ADRA2B	c.391T>A	P.C131S
SH_58	2	98458319	98458319	Missense_Mutation	SNV	G	T	T	TMEM131	c.682C>A	P.P228T
SH_58	20	3760330	3760330	Missense_Mutation	SNV	T	A	A	SPEF1	c.202A>T	P.N68Y
SH_58	20	60991294	60991294	Splice_Site	SNV	T	A	A	RBBP8NL	c.314-2A>T	
SH_58	20	9624935	9624935	Silent	SNV	C	T	T	PAK5	c.42G>A	P.P14P
SH_58	21	33065634	33065634	Missense_Mutation	SNV	G	C	C	SCAF4	c.1441C>G	P.P481A
SH_58	22	30189610	30189610	Frame_Shift_Del	INDEL	C	-	-	ASCC2	c.1516delG	P.A506fs
SH_58	22	35480473	35480473	Missense_Mutation	SNV	C	A	A	ISX	c.479C>A	P.P160H
SH_58	3	130187749	130187749	Nonsense_Mutation	SNV	A	T	T	COL6A5	c.6901A>T	P.R2301*
SH_58	3	184586829	184586829	Splice_Site	SNV	G	T	T	VPS8	c.1597+5G>T	
SH_58	3	184910636	184910636	Missense_Mutation	SNV	C	A	A	EHHADH	c.1262G>T	P.G421V
SH_58	3	185232321	185232321	Splice_Site	SNV	A	T	T	LIPH	c.983-12T>A	
SH_58	3	48680384	48680389	Splice_Site	INDEL	CCCCAT	-	-	CELSR3	11_8406+16delATGGGG	
SH_58	3	50417151	50417151	Splice_Site	SNV	C	T	T	CACNA2D2	c.993+5G>A	
SH_58	3	77651371	77651371	Silent	SNV	A	T	T	ROBO2	c.2805A>T	P.P935P
SH_58	4	108574771	108574771	Silent	SNV	T	C	C	PAPSS1	c.1113A>G	P.E371E
SH_58	4	5630395	5630395	Missense_Mutation	SNV	C	T	T	EVC2	c.1537G>A	P.G513S
SH_58	5	127686642	127686642	Silent	SNV	A	G	G	FBN2	c.2730T>C	P.N910N
SH_58	5	149361085	149361085	Silent	SNV	C	T	T	SLC26A2	c.1929C>T	P.P643P
SH_58	5	180477298	180477298	Missense_Mutation	SNV	T	A	A	BTNL9	c.665T>A	P.L222H
SH_58	5	60067866	60067866	Missense_Mutation	SNV	A	T	T	ELOVL7	c.119T>A	P.L40H
SH_58	5	60067867	60067867	Missense_Mutation	SNV	G	T	T	ELOVL7	c.118C>A	P.L40I
SH_58	5	71479618	71479618	Missense_Mutation	SNV	T	A	A	MAP1B	c.335T>A	P.L112Q
SH_58	6	159166544	159166544	Silent	SNV	A	T	T	SYTL3	c.684A>T	P.T228T
SH_58	7	115580744	115580744	Missense_Mutation	SNV	T	A	A	TFEC	c.818A>T	P.Q273L
SH_58	7	20406662	20406662	Nonsense_Mutation	SNV	G	T	T	ITGB8	c.241G>T	P.E81*
SH_58	7	93519584	93519584	Silent	SNV	G	T	T	TFPI2	c.103C>A	P.R35R
SH_58	8	61654710	61654710	Missense_Mutation	SNV	A	T	T	CHD7	c.719A>T	P.Q240L
SH_58	9	19516234	19516234	Missense_Mutation	SNV	T	A	A	SLC24A2	c.1852A>T	P.I618F

SH_58	X	144905208	144905208	Missense_Mutation	SNV	T	A	A	SLITRK2	c.1265T>A	P.L422Q
SH_58	X	99849248	99849248	Splice_Site	SNV	T	A	A	TNMD	c.322-10T>A	
SH_59	1	186303541	186303541	Missense_Mutation	SNV	T	C	C	TPR	c.5098A>G	P.M1700V
SH_59	1	222802659	222802659	Silent	SNV	C	A	A	MIA3	c.2097C>A	P.G699G
SH_59	1	247586609	247586609	Missense_Mutation	SNV	C	A	A	NLRP3	c.361C>A	P.L121M
SH_59	12	25398284	25398284	Missense_Mutation	SNV	C	T	T	KRAS	c.35G>A	P.G12D
SH_59	12	48919813	48919813	Silent	SNV	A	T	T	OR8S1	c.399A>T	P.G133G
SH_59	12	53207801	53207801	Silent	SNV	G	A	A	KRT4	c.42C>T	P.G14G
SH_59	12	6080796	6080796	Missense_Mutation	SNV	G	A	A	VWF	c.7517C>T	P.P2506L
SH_59	13	78335108	78335108	Silent	SNV	G	A	A	SLAIN1	c.1068G>A	P.L356L
SH_59	14	105418360	105418360	Missense_Mutation	SNV	G	A	A	AHNAK2	c.3428C>T	P.A1143V
SH_59	15	33992040	33992040	Splice_Site	SNV	T	G	G	RYR3	c.6379+6T>G	
SH_59	15	68609602	68609602	Splice_Site	SNV	C	T	T	ITGA11	c.2715+1G>A	
SH_59	15	75684622	75684622	Missense_Mutation	SNV	T	C	C	SIN3A	c.2812A>G	P.S938G
SH_59	16	24268113	24268113	Missense_Mutation	SNV	C	A	A	CACNG3	c.38C>A	P.T13N
SH_59	16	70926383	70926383	Missense_Mutation	SNV	T	A	A	HYDIN	c.9298A>T	P.I3100F
SH_59	17	36508652	36508652	Silent	SNV	G	A	A	SOCS7	c.525G>A	P.L175L
SH_59	17	7578555	7578555	Splice_Site	SNV	C	T	T	TP53	c.376-1G>A	
SH_59	18	21044196	21044196	Missense_Mutation	SNV	A	G	G	RIOK3	c.352A>G	P.K118E
SH_59	19	36592216	36592216	Missense_Mutation	SNV	G	C	C	WDR62	c.2968G>C	P.E990Q
SH_59	19	45556377	45556377	Silent	SNV	C	T	T	CLASRP	c.135C>T	P.D45D
SH_59	19	4682840	4682840	Missense_Mutation	SNV	G	A	A	DPP9	c.2342C>T	P.A781V
SH_59	2	172950481	172950481	Missense_Mutation	SNV	C	G	G	DLX1	c.76C>G	P.P26A
SH_59	2	220038876	220038876	Silent	SNV	C	T	T	CNPPD1	c.564G>A	P.L188L
SH_59	2	242189388	242189388	Missense_Mutation	SNV	T	A	A	HDLBP	c.1281A>T	P.R427S
SH_59	3	193855521	193855521	Silent	SNV	C	T	T	HES1	c.342C>T	P.F114F
SH_59	3	47035468	47035468	Frame_Shift_Ins	INDEL	-	AA	AA	NBEAL2	.1156_1157insA	P.R386fs
SH_59	3	53684912	53684912	Nonsense_Mutation	SNV	G	A	A	CACNA1D	c.590G>A	P.W197*
SH_59	4	39077684	39077684	Frame_Shift_Ins	INDEL	-	A	A	KLHL5	c.490dupA	P.M164fs
SH_59	7	100635176	100635176	Silent	SNV	A	G	G	MUC12	c.1332A>G	P.P444P
SH_59	7	124387076	124387076	Missense_Mutation	SNV	A	T	T	GPR37	c.1345T>A	P.C449S
SH_59	7	42085104	42085104	Silent	SNV	T	A	A	GLI3	c.705A>T	P.A235A
SH_59	8	141669637	141669637	Silent	SNV	C	A	A	PTK2	c.3126G>T	P.V1042V
SH_59	9	116779055	116779055	Missense_Mutation	SNV	G	A	A	ZNF618	c.739G>A	P.A247T
SH_59	9	134323164	134323164	Missense_Mutation	SNV	G	T	T	PRRC2B	c.1093G>T	P.D365Y
SH_59	X	47045027	47045027	Nonsense_Mutation	SNV	A	T	T	RBM10	c.2353A>T	P.K785*
SH_59	X	70349261	70349261	Missense_Mutation	SNV	A	C	C	MED12	c.3673A>C	P.K1225Q
SH_61	11	46894714	46894714	Missense_Mutation	SNV	T	G	G	LRP4	c.4520A>C	P.K1507T
SH_61	8	144992723	144992723	Missense_Mutation	SNV	C	T	T	PLEC	c.11347G>A	P.E3783K
SH_61	X	15262714	15262714	Missense_Mutation	SNV	G	A	A	ASB9	c.799C>T	P.R267W
SH_61	X	47424204	47424204	Missense_Mutation	SNV	C	T	T	ARAF	c.209C>T	P.T70M
SH_63	1	152279994	152279994	Silent	SNV	T	C	C	FLG	c.7368A>G	P.Q2456Q
SH_63	1	152280133	152280133	Missense_Mutation	SNV	C	T	T	FLG	c.7229G>A	P.R2410H
SH_63	1	152280540	152280540	Missense_Mutation	SNV	C	G	G	FLG	c.6822G>C	P.Q2274H
SH_63	1	154543934	154543934	Missense_Mutation	SNV	G	A	A	CHRNB2	c.635G>A	P.R212H
SH_63	1	67194943	67194943	Splice_Site	SNV	C	T	T	SGIP1	c.1152-4C>T	
SH_63	1	89521775	89521775	Missense_Mutation	SNV	C	T	T	GBP1	c.1292G>A	P.G431E
SH_63	10	104268946	104268946	Missense_Mutation	SNV	T	C	C	SUFU	c.203T>C	P.L68S
SH_63	10	127349670	127349670	Missense_Mutation	SNV	G	T	T	TEX36	c.184C>A	P.Q62K
SH_63	10	73544798	73544798	Missense_Mutation	SNV	C	T	T	CDH23	c.5653C>T	P.R1885C
SH_63	11	118764729	118764729	Missense_Mutation	SNV	G	A	A	CXCR5	c.476G>A	P.R159H
SH_63	11	17653759	17653759	Missense_Mutation	SNV	G	A	A	OTOG	c.7094G>A	P.R2365H
SH_63	11	56043599	56043599	Frame_Shift_Del	INDEL	T	-	-	OR5T1	c.486delT	P.A163fs
SH_63	11	62474578	62474578	Splice_Site	SNV	T	C	C	BSCL2	c.87+3A>G	
SH_63	12	3659248	3659248	Silent	SNV	G	A	A	PRMT8	c.381G>A	P.K127K
SH_63	12	48737167	48737167	Silent	SNV	C	T	T	ZNF641	c.864G>A	P.G288G
SH_63	12	7241272	7241272	Silent	SNV	C	A	A	C1R	c.972G>T	P.L324L
SH_63	13	102568894	102568894	Missense_Mutation	SNV	G	T	T	FGF14	c.102C>A	P.S34R
SH_63	13	20797280	20797280	Missense_Mutation	SNV	C	T	T	GJB6	c.340G>A	P.D114N
SH_63	15	33833073	33833073	Nonsense_Mutation	SNV	G	T	T	RYR3	c.628G>T	P.G210*
SH_63	16	8997165	8997165	Missense_Mutation	SNV	G	C	C	USP7	c.1751C>G	P.S584C
SH_63	17	29314958	29314958	Splice_Site	SNV	A	G	G	RNF135	c.517-4A>G	
SH_63	17	29585521	29585521	Splice_Site	SNV	G	C	C	NF1	c.4332+1G>C	
SH_63	17	31618659	31618659	Missense_Mutation	SNV	C	G	G	ASIC2	c.475G>C	P.G159R
SH_63	17	45291195	45291195	Splice_Site	SNV	G	A	A	MYL4	c.163+3G>A	
SH_63	17	7577538	7577538	Missense_Mutation	SNV	C	T	T	TP53	c.743G>A	P.R248Q
SH_63	18	29340285	29340285	Missense_Mutation	SNV	G	A	A	SLC25A52	c.370C>T	P.R124C
SH_63	18	77227587	77227587	Splice_Site	SNV	C	T	T	NFATC1	c.2092+5C>T	
SH_63	19	38860665	38860665	Missense_Mutation	SNV	T	C	C	CATSPERG	c.3074T>C	P.F1025S
SH_63	2	165986510	165986510	Silent	SNV	G	A	A	SCN3A	c.2715C>T	P.V905V
SH_63	2	167060926	167060926	Missense_Mutation	SNV	T	G	G	SCN9A	c.4414A>C	P.N1472H
SH_63	2	190530907	190530907	Missense_Mutation	SNV	C	G	G	ASNSD1	c.49C>G	P.Q17E
SH_63	2	85262234	85262234	Missense_Mutation	SNV	C	G	G	KCMF1	c.280C>G	P.Q94E
SH_63	20	18461131	18461131	Missense_Mutation	SNV	T	C	C	POLR3F	c.536T>C	P.I179T
SH_63	22	37771192	37771192	Missense_Mutation	SNV	C	T	T	ELFN2	c.383G>A	P.R128H
SH_63	3	164907236	164907236	Silent	SNV	G	A	A	SLITRK3	c.1383C>T	P.L461L
SH_63	3	183957181	183957181	Splice_Site	SNV	G	A	A	VWA5B2	c.1549-1G>A	
SH_63	3	47898936	47898936	Missense_Mutation	SNV	T	A	A	MAP4	c.3133A>T	P.I1045F
SH_63	5	140209476	140209476	Missense_Mutation	SNV	C	A	A	PCDHA6	c.1800C>A	P.D600E
SH_63	5	140720660	140720660	Missense_Mutation	SNV	G	A	A	PCDHGA2	c.2122G>A	P.V708I
SH_63	5	65458332	65458332	Missense_Mutation	SNV	C	T	T	SREK1	c.667C>T	P.R223W
SH_63	5	6737691	6737691	Missense_Mutation	SNV	G	A	A	PAPD7	c.35G>A	P.R12K
SH_63	5	82940474	82940474	Missense_Mutation	SNV	G	T	T	HAPLN1	c.483C>A	P.F161L
SH_63	5	90001230	90001230	Silent	SNV	C	T	T	ADGRV1	c.8400C>T	P.A2800A
SH_63	5	92923847	92923847	Missense_Mutation	SNV	G	A	A	NR2F1	c.688G>A	P.A230T
SH_63	6	112041037	112041037	Missense_Mutation	SNV	G	A	A	FYN	c.218C>T	P.T73M
SH_63	6	33143401	33143401	Missense_Mutation	SNV	G	A	A	COL11A2	c.2068C>T	P.R690C
SH_63	6	46135305	46135305	Missense_Mutation	SNV	T	C	C	ENPP5	c.695A>G	P.N232S
SH_63	6	47681774	47681774	Missense_Mutation	SNV	A	T	T	ADGRF4	c.793A>T	P.T265S
SH_63	6	64472524	64472524	Missense_Mutation	SNV	C	T	T	EYS	c.7901G>A	P.C2634Y
SH_63	7	150901820	150901820	Silent	SNV	G	A	A	IQCA1L	c.114C>T	P.S38S
SH_63	7	34125504	34125504	Missense_Mutation	SNV	C	G	G	BMPER	c.1545C>G	P.F515L
SH_63	7	57187736	57187736	Missense_Mutation	SNV	C	G	G	ZNF479	c.1386G>C	P.E462D
SH_63	8	113599310	113599310	Silent	SNV	T	C	C	CSMD3	c.3558A>G	P.Q1186Q
SH_63	8	121344387	121344387	Missense_Mutation	SNV	C	T	T	COL14A1	c.4667C>T	P.P1556L
SH_63	8	126044542	126044542	Silent	SNV	G	A	A	KIAA0196	c.3276C>T	P.T1092T
SH_63	9	103035322	103035322	Missense_Mutation	SNV	T	A	A	INVS	c.1460T>A	P.M487K
SH_63	9	136379827	136379827	Missense_Mutation	SNV	G	C	C	TMEM8C	c.597C>G	P.N199K

SH_63	9	96021359	96021359	Silent	SNV	C	T	T	WNK2	c.2529C>T	P.L843L
SH_63	X	106888567	106888567	Missense_Mutation	SNV	C	A	A	PRPS1	c.79C>A	P.H27N
SH_63	X	11187706	11187706	Silent	SNV	C	T	T	ARHGAP6	c.1728G>A	P.Q576Q
SH_63	X	21875409	21875409	Silent	SNV	C	T	T	YY2	c.807C>T	P.A269A
SH_63	X	35820977	35820977	Nonsense_Mutation	SNV	G	T	T	MAGEB16	c.664G>T	P.E222*
SH_63	X	8763404	8763404	Silent	SNV	G	A	A	FAM9A	c.546C>T	P.I182I
SH_64	1	151679994	151679994	Missense_Mutation	SNV	G	A	A	CELF3	c.620C>T	P.T207I
SH_64	1	152324169	152324169	Missense_Mutation	SNV	A	T	T	FLG2	c.6093T>A	P.S2031R
SH_64	1	156255485	156255485	Silent	SNV	C	T	T	TMEM79	c.468C>T	P.C156C
SH_64	1	156641644	156641644	Missense_Mutation	SNV	G	T	T	NES	c.2336C>A	P.P779H
SH_64	1	204118896	204118896	Silent	SNV	C	A	A	ETNK2	c.453G>T	P.L151L
SH_64	1	209788657	209788657	Missense_Mutation	SNV	G	A	A	LAMB3	c.3478C>T	P.H1160Y
SH_64	1	39775993	39775993	Missense_Mutation	SNV	A	G	G	MACF1	c.3008A>G	P.D1003G
SH_64	1	48231758	48231758	Missense_Mutation	SNV	G	A	A	TRABD2B	c.1370C>T	P.P457L
SH_64	10	134004294	134004294	Missense_Mutation	SNV	A	G	G	DPYSL4	c.83A>G	P.D28G
SH_64	11	104912392	104912392	Missense_Mutation	SNV	C	G	G	CARD16	c.329G>C	P.G110A
SH_64	11	55587681	55587681	Silent	SNV	T	C	C	OR5D18	c.576T>C	P.D192D
SH_64	11	62296777	62296777	Missense_Mutation	SNV	G	C	C	AHNAK	c.5112C>G	P.H1704Q
SH_64	12	15073918	15073918	Missense_Mutation	SNV	C	A	A	ERP27	c.95G>T	P.S32I
SH_64	12	2702394	2702394	Missense_Mutation	SNV	A	T	T	CACNA1C	c.2546A>T	P.E849V
SH_64	12	65232577	65232577	Missense_Mutation	SNV	A	T	T	TBC1D30	c.878A>T	P.E293V
SH_64	12	7647755	7647755	Missense_Mutation	SNV	A	T	T	CD163	c.1342T>A	P.S448T
SH_64	13	99853802	99853802	Missense_Mutation	SNV	G	T	T	UBAC2	c.137G>T	P.R46L
SH_64	15	41865614	41865614	Missense_Mutation	SNV	G	T	T	TYRO3	c.2094G>T	P.W698C
SH_64	15	45456966	45456966	Splice_Site	SNV	T	A	A	DUOX1	c.4534-11T>A	
SH_64	17	3101040	3101040	Silent	SNV	A	T	T	OR1A2	c.228A>T	P.V76V
SH_64	17	7577534	7577534	Missense_Mutation	SNV	C	A	A	TP53	c.747G>T	P.R249S
SH_64	2	128334281	128334281	Missense_Mutation	SNV	A	T	T	MYO7B	c.847A>T	P.M283L
SH_64	2	200804867	200804867	Splice_Site	SNV	C	A	A	TYW5	c.304-11G>T	
SH_64	2	212576833	212576833	Missense_Mutation	SNV	A	G	G	ERBB4	c.1066T>C	P.F356L
SH_64	2	234627496	234627496	Silent	SNV	G	T	T	UGT1A4	c.30G>T	P.P10P
SH_64	2	88407989	88407989	Missense_Mutation	SNV	G	T	T	SMYD1	c.1245G>T	P.M415I
SH_64	22	46733701	46733701	Missense_Mutation	SNV	G	T	T	TRMU	c.108G>T	P.M36I
SH_64	22	46785261	46785261	Missense_Mutation	SNV	C	A	A	CELSR1	c.6481G>T	P.G2161C
SH_64	4	119659414	119659414	Splice_Site	SNV	A	T	T	SEC24D	c.2499+2T>A	
SH_64	4	121958488	121958488	Missense_Mutation	SNV	A	G	G	NDNF	c.638T>C	P.V213A
SH_64	4	130023842	130023842	Missense_Mutation	SNV	G	T	T	C4orf33	c.77G>T	P.G26V
SH_64	4	183815639	183815639	Splice_Site	SNV	T	C	C	DCTD	c.394+3A>G	
SH_64	5	140256509	140256509	Silent	SNV	G	A	A	PCDHA12	c.1452G>A	P.A484A
SH_64	5	161576191	161576191	Missense_Mutation	SNV	G	A	A	GABRG2	c.1000G>A	P.A334T
SH_64	5	60825973	60825973	Missense_Mutation	SNV	G	T	T	ZSWIM6	c.1932G>T	P.M644I
SH_64	6	26598557	26598557	Missense_Mutation	SNV	G	T	T	ABT1	c.503G>T	P.R168M
SH_64	6	31912624	31912624	Missense_Mutation	SNV	T	A	A	C2	c.2023T>A	P.C675S
SH_64	7	103180757	103180757	Missense_Mutation	SNV	C	T	T	RELN	c.6817G>A	P.G2273S
SH_64	7	107738918	107738918	Missense_Mutation	SNV	T	A	A	LAMB4	c.1290A>T	P.E430D
SH_64	7	116423410	116423410	Missense_Mutation	SNV	A	T	T	MET	c.3685A>T	P.M1229L
SH_64	7	141920332	141920332	Missense_Mutation	SNV	T	A	A	MGAM2	c.6021T>A	P.S2007R
SH_64	7	55750530	55750530	Splice_Site	INDEL	G	-	-	FKBP9P1	n.686-12delC	
SH_64	7	82581706	82581706	Missense_Mutation	SNV	A	T	T	PCLO	c.8563T>A	P.L2855I
SH_64	8	113246675	113246675	Missense_Mutation	SNV	C	T	T	CSMD3	c.10152G>A	P.M3384I
SH_64	8	133041356	133041356	Missense_Mutation	SNV	A	T	T	OC90	c.1102T>A	P.W368R
SH_64	8	42045020	42045020	Silent	SNV	G	T	T	PLAT	c.435C>A	P.G145G
SH_64	8	72981427	72981427	Missense_Mutation	SNV	T	A	A	TRPA1	c.275A>T	P.H92L
SH_64	9	125239745	125239745	Missense_Mutation	SNV	G	T	T	OR1J1	c.461C>A	P.A154E
SH_64	9	21217180	21217180	Missense_Mutation	SNV	G	A	A	IFNA16	c.125C>T	P.A42V
SH_66	1	2105415	2105415	Silent	SNV	C	T	T	PRKCZ	c.816C>T	P.T272T
SH_66	12	25398284	25398284	Missense_Mutation	SNV	C	G	G	KRAS	c.35G>C	P.G12A
SH_66	16	2283025	2283025	Silent	SNV	A	C	C	E4F1	c.897A>C	P.G299G
SH_66	16	24886938	24886938	Splice_Site	SNV	T	C	C	SLC5A11	c.181-10T>C	
SH_66	22	19124841	19124841	Missense_Mutation	SNV	A	C	C	DGCR14	c.1030T>G	P.F344V
SH_66	22	43986044	43986044	Missense_Mutation	SNV	G	C	C	EFCAB6	c.2942C>G	P.T981S
SH_66	5	153175078	153175078	Silent	SNV	C	T	T	GRIA1	c.2313C>T	P.G771G
SH_66	9	140043509	140043509	Silent	SNV	C	T	T	GRIN1	c.619C>T	P.L207L
SH_66	9	35061070	35061070	Missense_Mutation	SNV	T	C	C	VCP	c.1301A>G	P.D434G
SH_67	1	148341939	148341939	Missense_Mutation	SNV	A	C	C	NBPF9	c.634T>G	P.S212A
SH_67	10	72633267	72633267	Missense_Mutation	SNV	G	T	T	SGPL1	c.1219G>T	P.A407S
SH_67	11	44286426	44286426	Missense_Mutation	SNV	G	A	A	ALX4	c.1214C>T	P.A405V
SH_67	14	21161742	21161742	Missense_Mutation	SNV	G	A	A	ANG	c.19G>A	P.V7I
SH_69	1	145299871	145299871	Missense_Mutation	SNV	G	T	T	NBPF10	c.920G>T	P.R307I
SH_69	1	228459745	228459745	Missense_Mutation	SNV	C	A	A	OBSCN	c.5809C>A	P.Q1937K
SH_69	1	228487233	228487233	Splice_Site	SNV	C	T	T	OBSCN	c.13474+10C>T	
SH_69	1	65309803	65309803	Missense_Mutation	SNV	G	A	A	JAK1	c.2347C>T	P.L783F
SH_69	14	20216204	20216204	Silent	SNV	G	C	C	OR4Q3	c.618G>C	P.L206L
SH_69	16	11362921	11362921	Missense_Mutation	SNV	C	T	T	TNP2	c.199G>A	P.G67S
SH_69	16	50741788	50741788	Missense_Mutation	SNV	C	A	A	NOD2	c.482C>A	P.A161D
SH_69	19	43689246	43689246	Missense_Mutation	SNV	C	T	T	PSG5	c.118G>A	P.A40T
SH_69	2	108487603	108487603	Missense_Mutation	SNV	T	C	C	RGPD4	c.3143T>C	P.V1048A
SH_69	2	96546346	96546346	Missense_Mutation	SNV	C	G	G	ANKRD36C	c.4488G>C	P.L1496F
SH_69	5	159854783	159854783	Silent	SNV	G	T	T	PTTG1	c.432G>T	P.V144V
SH_69	8	73848937	73848937	Silent	SNV	C	A	A	KCNB2	c.1347C>A	P.I449I
SH_70	1	197479681	197479681	Missense_Mutation	SNV	A	G	G	DENND1B	c.2237T>C	P.L746P
SH_70	1	200008926	200008926	Splice_Site	SNV	A	G	G	NR5A2	c.202+3A>G	
SH_70	1	226574083	226574083	Missense_Mutation	SNV	C	T	T	PARP1	c.778G>A	P.D260N
SH_70	1	235345367	235345367	Missense_Mutation	SNV	G	A	A	ARID4B	c.2867C>T	P.P956L
SH_70	11	7642253	7642253	Missense_Mutation	SNV	A	G	G	PPFIBP2	c.370A>G	P.T124A
SH_70	12	104048404	104048404	Silent	SNV	T	A	A	STAB2	c.1479T>A	P.A493A
SH_70	12	79689895	79689895	Missense_Mutation	SNV	T	G	G	SYT1	c.521T>G	P.M174R
SH_70	13	49684548	49684548	Splice_Site	SNV	A	C	C	FNDC3A	c.7+6A>C	
SH_70	14	101349285	101349285	Missense_Mutation	SNV	G	A	A	RTL1	c.1841C>T	P.A614V
SH_70	15	48776091	48776091	Silent	SNV	G	A	A	FBN1	c.3762C>T	P.C1254C
SH_70	15	59500001	59500001	Missense_Mutation	SNV	T	A	A	LDHAL6B	c.862T>A	P.Y288N
SH_70	16	48175175	48175175	Missense_Mutation	SNV	C	T	T	ABCC12	c.365G>A	P.R122H
SH_70	16	69170708	69170708	Missense_Mutation	SNV	C	T	T	UTP4	c.20C>T	P.A7V
SH_70	17	27861114	27861117	Splice_Site	INDEL	TCTC	-	-	TAOK1	52-11_2362-8delCTCT	
SH_70	2	24283674	24283674	Splice_Site	SNV	G	A	A	FKBP1B	c.-2-10G>A	
SH_70	2	71576596	71576596	Missense_Mutation	SNV	C	T	T	ZNF638	c.512C>T	P.P171L
SH_70	20	47688902	47688903	Frame_Shift_Del	INDEL	AT	-	-	CSE1L	c.848_849delAT	P.D283fs
SH_70	20	5933137	5933137	Silent	SNV	C	T	T	MCM8	c.216C>T	P.F72F

SH_70	20	62168506	62168506	Silent	SNV	A	C	C	PTK6	c.162T>G	P.G54G
SH_70	22	19344435	19344435	Missense_Mutation	SNV	C	A	A	HIRA	c.2374G>T	P.A792S
SH_70	22	38204131	38204131	Missense_Mutation	SNV	C	T	T	GCAT	c.157C>T	P.R53C
SH_70	3	45972616	45972616	Missense_Mutation	SNV	A	T	T	FYCO1	c.4198T>A	P.S1400T
SH_70	4	151849656	151849658	Splice_Site	INDEL	AAG	-	-	LRBA	9+10_549+12delCTT	
SH_70	5	140595018	140595018	Silent	SNV	C	T	T	PCDHB13	c.1323C>T	P.I441I
SH_70	5	140712198	140712198	Silent	SNV	C	T	T	PCDHGA1	c.1947C>T	P.H649H
SH_70	5	154396819	154396819	Missense_Mutation	SNV	C	A	A	KIF4B	c.3400C>A	P.Q1134K
SH_70	5	169101307	169101307	Frame_Shift_Del	INDEL	A	-	-	DOCK2	c.332delA	P.K111fs
SH_70	5	176004723	176004723	Missense_Mutation	SNV	C	T	T	CDHR2	c.1436C>T	P.T479M
SH_70	6	25653642	25653642	Missense_Mutation	SNV	G	A	A	SCGN	c.115G>A	P.A39T
SH_70	6	26199965	26199965	Missense_Mutation	SNV	T	G	G	HIST1H2BF	c.179T>G	P.M60R
SH_70	6	27100374	27100374	Missense_Mutation	SNV	G	T	T	HIST1H2BJ	c.156C>A	P.D52E
SH_70	6	31324628	31324628	Missense_Mutation	SNV	G	T	T	HLA-B	c.180C>A	P.F60L
SH_70	7	127014776	127014776	Missense_Mutation	SNV	G	C	C	ZNF800	c.614C>G	P.P205R
SH_70	8	113529433	113529433	Missense_Mutation	SNV	C	T	T	CSMD3	c.4274G>A	P.R1425H
SH_70	X	105011452	105011452	Missense_Mutation	SNV	G	C	C	IL1RAPL2	c.1859G>C	P.G620A
SH_70	X	105970265	105970265	Missense_Mutation	SNV	C	G	G	RNF128	c.122C>G	P.A41G
SH_70	X	53112240	53112240	Missense_Mutation	SNV	G	C	C	TSPYL2	c.560G>C	P.R187T
SH_70	X	70523144	70523144	Missense_Mutation	SNV	A	C	C	ITGB1BP2	c.350A>C	P.N117T
SH_70	X	90690678	90690678	Silent	SNV	C	A	A	PABPC5	c.102C>A	P.L34L
SH_71	1	12726141	12726141	Missense_Mutation	SNV	G	A	A	AADACL4	c.619G>A	P.V207M
SH_71	1	145281968	145281968	Missense_Mutation	SNV	A	T	T	NOTCH2NL	c.648A>T	P.R216S
SH_71	1	152086470	152086470	Silent	SNV	A	T	T	TCHH	c.87T>A	P.T29T
SH_71	1	159828008	159828008	Missense_Mutation	SNV	G	A	A	VSIG8	c.302C>T	P.A101V
SH_71	1	160147404	160147404	Missense_Mutation	SNV	T	A	A	ATP1A4	c.94T>A	P.W32R
SH_71	1	161476312	161476312	Missense_Mutation	SNV	A	T	T	FCGR2A	c.295A>T	P.S99C
SH_71	1	181479644	181479644	Missense_Mutation	SNV	A	T	T	CACNA1E	c.298A>T	P.I100F
SH_71	1	19181133	19181133	Silent	SNV	G	A	A	TAS1R2	c.831C>T	P.P277P
SH_71	1	196309613	196309613	Silent	SNV	T	A	A	KCNT2	c.1641A>T	P.T547T
SH_71	1	201177789	201177789	Silent	SNV	A	T	T	IGFN1	c.3768A>T	P.S1256S
SH_71	1	201195010	201195010	Silent	SNV	G	A	A	IGFN1	c.10545G>A	P.T3515T
SH_71	1	20412610	20412610	Silent	SNV	A	T	T	PLA2G5	c.75A>T	P.L25L
SH_71	1	20656805	20656805	Missense_Mutation	SNV	A	T	T	VWA5B1	c.1387A>T	P.I463F
SH_71	1	209974729	209974729	Silent	SNV	T	C	C	IRF6	c.30A>G	P.L10L
SH_71	1	21795269	21795269	Silent	SNV	A	T	T	NBPF3	c.222A>T	P.L74L
SH_71	1	223116553	223116553	Missense_Mutation	SNV	A	T	T	DISP1	c.388A>T	P.N130Y
SH_71	1	231401865	231401865	Missense_Mutation	SNV	A	G	G	GNPAT	c.695A>G	P.Y232C
SH_71	1	24022297	24022297	Missense_Mutation	SNV	A	T	T	RPL11	c.406A>T	P.R136W
SH_71	1	247031021	247031021	Missense_Mutation	SNV	T	C	C	AHCTF1	c.3181A>G	P.K1061E
SH_71	1	248031306	248031306	Missense_Mutation	SNV	T	A	A	TRIM58	c.812T>A	P.M271K
SH_71	1	28354311	28354311	Missense_Mutation	SNV	T	A	A	EYA3	c.329A>T	P.Y110F
SH_71	1	3102742	3102742	Missense_Mutation	SNV	A	T	T	PRDM16	c.91A>T	P.S31C
SH_71	1	90484250	90484250	Missense_Mutation	SNV	A	T	T	ZNF326	c.814A>T	P.M272L
SH_71	10	105889993	105889993	Silent	SNV	T	C	C	CFAP43	c.4902A>G	P.T1634T
SH_71	10	18169595	18169595	Missense_Mutation	SNV	A	T	T	MRC1	c.2861A>T	P.N954I
SH_71	10	55943333	55943333	Silent	SNV	T	A	A	PCDH15	c.1476A>T	P.V492V
SH_71	11	121481850	121481850	Missense_Mutation	SNV	A	T	T	SORL1	c.5305A>T	P.M1769L
SH_71	11	1260724	1260724	Missense_Mutation	SNV	T	G	G	MUC5B	c.3511T>G	P.C1171G
SH_71	11	18108760	18108760	Silent	SNV	T	A	A	SAAL1	c.783A>T	P.P261P
SH_71	11	75115772	75115772	Missense_Mutation	SNV	G	T	T	RPS3	c.595G>T	P.G199C
SH_71	12	12870990	12870990	Nonsense_Mutation	SNV	A	T	T	CDKN1B	c.217A>T	P.K73*
SH_71	12	132685736	132685736	Missense_Mutation	SNV	A	T	T	GALNT9	c.1334T>A	P.F445Y
SH_71	12	15654750	15654750	Missense_Mutation	SNV	T	A	A	PTPRO	c.858T>A	P.F286L
SH_71	12	4705744	4705744	Splice_Site	SNV	A	T	T	DYRK4	n.962-11A>T	
SH_71	12	4870133	4870133	Missense_Mutation	SNV	T	A	A	GALNT8	c.1183T>A	P.C395S
SH_71	12	52992743	52992743	Missense_Mutation	SNV	C	T	T	KRT72	c.580G>A	P.G194R
SH_71	12	85255635	85255635	Missense_Mutation	SNV	A	T	T	SLC6A15	c.1648T>A	P.Y550N
SH_71	12	91502374	91502374	Missense_Mutation	SNV	A	T	T	LUM	c.383T>A	P.L128Q
SH_71	12	97084952	97084952	Missense_Mutation	SNV	A	T	T	CFAP54	c.6128A>T	P.Y2043F
SH_71	13	45149121	45149121	Missense_Mutation	SNV	A	T	T	TSC22D1	c.1090T>A	P.L364M
SH_71	13	46065104	46065104	Splice_Site	SNV	A	T	T	COG3	c.968+11A>T	
SH_71	13	76301222	76301222	Silent	SNV	A	T	T	LMO7	c.198A>T	P.P66P
SH_71	14	20404317	20404317	Silent	SNV	G	T	T	OR4K1	c.492G>T	P.V164V
SH_71	15	30900711	30900711	Missense_Mutation	SNV	T	A	A	GOLGA8H	c.438T>A	P.N146K
SH_71	15	33954893	33954893	Missense_Mutation	SNV	A	T	T	RYR3	c.5162A>T	P.Q1721L
SH_71	15	41120653	41120653	Silent	SNV	G	T	T	PPP1R14D	c.187C>A	P.R63R
SH_71	15	42342792	42342792	Missense_Mutation	SNV	T	A	A	PLA2G4E	c.110A>T	P.E37V
SH_71	15	49037129	49037129	Frame_Shift_Del	INDEL	A	-	-	CEP152	c.3695delT	P.L1232fs
SH_71	15	55783366	55783366	Silent	SNV	T	A	A	DYX1C1	c.360A>T	P.A120A
SH_71	15	55972250	55972250	Splice_Site	SNV	A	T	T	PRTG	c.973+2T>A	
SH_71	15	56387737	56387737	Missense_Mutation	SNV	T	A	A	RFX7	c.2189A>T	P.Q730L
SH_71	15	64680075	64680075	Missense_Mutation	SNV	G	T	T	TRIP4	c.13G>T	P.G5W
SH_71	15	64680076	64680076	Missense_Mutation	SNV	G	T	T	TRIP4	c.14G>T	P.G5V
SH_71	15	92705976	92705976	Splice_Site	SNV	C	G	G	SLCO3A1	c.1754-10C>G	
SH_71	16	12093152	12093152	Splice_Site	SNV	A	T	T	SNX29	c.8-2A>T	
SH_71	16	3283657	3283657	Silent	SNV	T	A	A	ZNF200	c.99A>T	P.R33R
SH_71	16	48396197	48396197	Missense_Mutation	SNV	A	T	T	SIAH1	c.236T>A	P.V79E
SH_71	16	67911472	67911472	Missense_Mutation	SNV	G	T	T	EDC4	c.702G>T	P.R234S
SH_71	16	69727852	69727852	Missense_Mutation	SNV	A	T	T	NFAT5	c.4121A>T	P.Q1374L
SH_71	16	71007873	71007873	Silent	SNV	A	T	T	HYDIN	c.5088T>A	P.I1696I
SH_71	17	10362659	10362659	Missense_Mutation	SNV	A	T	T	MYH4	c.1496T>A	P.L499Q
SH_71	17	16021198	16021198	Splice_Site	SNV	T	A	A	NCOR1	c.2055+4A>T	
SH_71	17	39120715	39120715	Silent	SNV	T	G	G	KRT39	c.534A>C	P.A178A
SH_71	17	48191581	48191581	Silent	SNV	G	A	A	SAMD14	c.912C>T	P.P304P
SH_71	17	7577568	7577568	Missense_Mutation	SNV	C	A	A	TP53	c.713G>T	P.C238F
SH_71	18	30913191	30913191	Missense_Mutation	SNV	T	G	G	CCDC178	c.826A>C	P.K276Q
SH_71	18	50866104	50866104	Missense_Mutation	SNV	C	G	G	DCC	c.2186C>G	P.P729R
SH_71	19	31769226	31769226	Silent	SNV	A	T	T	TSHZ3	c.1473T>A	P.P491P
SH_71	19	35836550	35836550	Missense_Mutation	SNV	T	A	A	CD22	c.2254T>A	P.Y752N
SH_71	19	55107274	55107274	Missense_Mutation	SNV	T	A	A	LILRA1	c.832T>A	P.S278T
SH_71	19	57051027	57051027	Missense_Mutation	SNV	A	G	G	ZFP28	c.242A>G	P.E81G
SH_71	19	57327876	57327876	Missense_Mutation	SNV	T	A	A	PEG3	c.1934A>T	P.H645L
SH_71	19	9056879	9056879	Silent	SNV	A	T	T	MUC16	c.30567T>A	P.S10189S
SH_71	2	113081919	113081919	Missense_Mutation	SNV	C	T	T	ZC3H6	c.1531C>T	P.P511S
SH_71	2	113082677	113082677	Silent	SNV	AA	T	T	ZC3H6	c.1989A>T	P.A663A
SH_71	2	113537149	113537149	Silent	SNV	G	A	A	IL1A	c.414C>T	P.D138D
SH_71	2	125555776	125555776	Silent	SNV	A	T	T	CNTNAP5	c.3093A>T	P.T1031T

SH_71	2	149679720	149679720	Nonsense_Mutation	SNV	T	A	A	KIF5C	c.141T>A	P.Y47*
SH_71	2	167297907	167297907	Missense_Mutation	SNV	A	T	T	SCN7A	c.2156T>A	P.L719Q
SH_71	2	189850408	189850408	Silent	SNV	T	A	A	COL3A1	c.351T>A	P.P117P
SH_71	2	192194682	192194682	Silent	SNV	A	T	T	MYO1B	c.273A>T	P.A91A
SH_71	2	209113365	209113365	Missense_Mutation	SNV	T	A	A	IDH1	c.142A>T	P.N48Y
SH_71	2	21232828	21232828	Silent	SNV	T	A	A	APOB	c.6912A>T	P.T2304T
SH_71	2	239003074	239003074	Missense_Mutation	SNV	A	T	T	SCLY	c.1043A>T	P.Q348L
SH_71	2	242076617	242076617	Missense_Mutation	SNV	T	A	A	PASK	c.939A>T	P.L313F
SH_71	2	44528130	44528130	Splice_Site	SNV	T	A	A	SLC3A1	c.1012-12T>A	
SH_71	2	47630423	47630423	Silent	SNV	C	A	A	MSH2	c.93C>A	P.T31T
SH_71	2	74074873	74074873	Silent	SNV	A	T	T	STAMBP	c.735A>T	P.S245S
SH_71	20	3102162	3102162	Missense_Mutation	SNV	T	A	A	UBOX5	c.1123A>T	P.S375C
SH_71	20	44669966	44669966	Splice_Site	SNV	A	T	T	SLC12A5	c.924-2A>T	
SH_71	20	45891038	45891038	Missense_Mutation	SNV	T	A	A	ZMYND8	c.1615A>T	P.N539Y
SH_71	20	55748285	55748285	Missense_Mutation	SNV	C	T	T	BMP7	c.1117G>A	P.A373T
SH_71	21	10916449	10916449	Missense_Mutation	SNV	T	A	A	TPTE	c.783A>T	P.Q261H
SH_71	21	10951307	10951307	Missense_Mutation	SNV	T	A	A	TPTE	c.291A>T	P.L97F
SH_71	3	10089723	10089723	Silent	SNV	G	T	T	FANCD2	c.1401G>T	P.T467T
SH_71	3	111317017	111317017	Silent	SNV	A	T	T	CD96	c.858A>T	P.T286T
SH_71	3	125179630	125179630	Missense_Mutation	SNV	T	A	A	SNX4	c.929A>T	P.Y310F
SH_71	3	132036312	132036312	Silent	SNV	A	T	T	ACPP	c.12A>T	P.A4A
SH_71	3	160395290	160395290	Silent	SNV	C	A	A	ARL14	c.156C>A	P.I52I
SH_71	3	18083033	18083033	Missense_Mutation	SNV	G	A	A	LOC339862	c.316G>A	P.G106S
SH_71	3	47651717	47651717	Missense_Mutation	SNV	T	A	A	SMARCC1	c.2882A>T	P.Q961L
SH_71	4	106510453	106510453	Missense_Mutation	SNV	C	T	T	ARHGEF38	c.245C>T	P.P82L
SH_71	4	143181694	143181694	Silent	SNV	T	A	A	INPP4B	c.639A>T	P.T213T
SH_71	4	148834219	148834219	Splice_Site	SNV	T	A	A	ARHGAP10	c.1163-6T>A	
SH_71	4	159756593	159756593	Missense_Mutation	SNV	A	T	T	FNIP2	c.761A>T	P.Q254L
SH_71	4	437355	437355	Nonsense_Mutation	SNV	C	A	A	ZNF721	c.901G>T	P.G301*
SH_71	5	140751633	140751633	Missense_Mutation	SNV	A	T	T	PCDHGB3	c.1672A>T	P.N558Y
SH_71	5	168201298	168201298	Missense_Mutation	SNV	T	A	A	SLIT3	c.1237A>T	P.N413Y
SH_71	5	176318173	176318173	Silent	SNV	C	A	A	HK3	c.279G>T	P.V93V
SH_71	5	52942144	52942144	Missense_Mutation	SNV	A	T	T	NDUFS4	c.259A>T	P.M87L
SH_71	5	90024644	90024644	Missense_Mutation	SNV	T	A	A	ADGRV1	c.10320T>A	P.S3440R
SH_71	6	114264511	114264511	Splice_Site	SNV	T	A	A	HDAC2	c.1378+4A>T	
SH_71	6	137815010	137815010	Missense_Mutation	SNV	T	A	A	OLIG3	c.298A>T	P.N100Y
SH_71	6	152722287	152722287	Missense_Mutation	SNV	A	T	T	SYNE1	c.7036T>A	P.L2346M
SH_71	6	155750005	155750005	Silent	SNV	T	A	A	NOX3	c.1068A>T	P.A356A
SH_71	6	24447101	24447101	Silent	SNV	C	A	A	GPLD1	c.1785G>T	P.L595L
SH_71	6	36104687	36104687	Silent	SNV	A	T	T	MAPK13	c.642A>T	P.A214A
SH_71	6	88769271	88769271	Missense_Mutation	SNV	T	A	A	SPACA1	c.575T>A	P.V192E
SH_71	6	89977435	89977435	Missense_Mutation	SNV	A	T	T	GABRR2	c.699T>A	P.F233L
SH_71	7	100683484	100683484	Silent	SNV	A	T	T	MUC17	c.8787A>T	P.P2929P
SH_71	7	146829371	146829371	Missense_Mutation	SNV	C	T	T	CNTNAP2	c.1118C>T	P.T373M
SH_71	7	36461510	36461510	Missense_Mutation	SNV	A	T	T	ANLN	c.2097A>T	P.Q699H
SH_71	7	45124415	45124439	Frame_Shift_Del	INDEL	TGAGGTCTG/	-	-	NACAD	TGCTCCTCAGAC	P.A447fs
SH_71	7	82580537	82580537	Missense_Mutation	SNV	T	A	A	PCLO	c.9367A>T	P.T3123S
SH_71	8	110587828	110587828	Missense_Mutation	SNV	A	T	T	SYBU	c.1296T>A	P.D432E
SH_71	8	135612685	135612685	Silent	SNV	T	A	A	ZFAT	c.2433A>T	P.T811T
SH_71	8	36766896	36766896	Missense_Mutation	SNV	A	T	T	KCNU1	c.2174A>T	P.H725L
SH_71	8	48827961	48827961	Missense_Mutation	SNV	T	A	A	PRKDC	c.2544A>T	P.L848F
SH_71	8	69552677	69552677	Missense_Mutation	SNV	A	T	T	C8orf34	c.1172A>T	P.Q391L
SH_71	8	70967630	70967630	Missense_Mutation	SNV	T	A	A	PRDM14	c.1393A>T	P.T465S
SH_71	8	91937869	91937869	Missense_Mutation	SNV	A	T	T	NECAB1	c.601A>T	P.N201Y
SH_71	8	94772166	94772166	Silent	SNV	G	A	A	TMEM67	c.108G>A	P.P36P
SH_71	9	101810231	101810231	Silent	SNV	A	T	T	COL15A1	c.2742A>T	P.P914P
SH_71	9	116259668	116259668	Silent	SNV	A	G	G	RGS3	c.825A>G	P.R275R
SH_71	9	21333634	21333634	Missense_Mutation	SNV	C	A	A	KLHL9	c.1225G>T	P.A409S
SH_71	9	33338526	33338526	Missense_Mutation	SNV	A	T	T	NFX1	c.2057A>T	P.D686V
SH_71	9	6014278	6014278	Nonsense_Mutation	SNV	T	A	A	RANBP6	c.274A>T	P.K92*
SH_71	9	84605951	84605951	Missense_Mutation	SNV	G	A	A	SPATA31D1	c.566G>A	P.R189Q
SH_71	9	84609680	84609680	Missense_Mutation	SNV	T	A	A	SPATA31D1	c.4295T>A	P.L1432H
SH_71	X	117676887	117676887	Splice_Site	SNV	A	T	T	DOCK11	c.220-2A>T	
SH_71	X	124455317	124455317	Missense_Mutation	SNV	A	T	T	TEX13C	c.1349A>T	P.Q450L
SH_71	X	131219616	131219616	Missense_Mutation	SNV	A	T	T	FRMD7	c.593T>A	P.V198E
SH_71	X	154319075	154319075	Missense_Mutation	SNV	A	T	T	BRCC3	c.509A>T	P.Y170F
SH_71	X	24380967	24380967	Silent	SNV	T	A	A	SUPT20HL1	c.90T>A	P.P30P
SH_71	X	34148324	34148324	Missense_Mutation	SNV	A	T	T	FAM47A	c.2072T>A	P.V691E
SH_71	X	53430585	53430585	Missense_Mutation	SNV	T	A	A	SMC1A	c.2267A>T	P.E756V
SH_74	10	115336953	115336953	Missense_Mutation	SNV	C	T	T	HABP2	c.298C>T	P.L100F
SH_74	10	135233650	135233650	Missense_Mutation	SNV	C	T	T	MTG1	c.986C>T	P.P329L
SH_74	11	113269863	113269863	Missense_Mutation	SNV	A	T	T	ANKK1	c.1172A>T	P.Q391L
SH_74	11	5411452	5411452	Missense_Mutation	SNV	G	A	A	OR51M1	c.824G>A	P.R275H
SH_74	11	55406246	55406246	Missense_Mutation	SNV	A	G	G	OR4P4	c.413A>G	P.K138R
SH_74	12	57556141	57556141	Silent	SNV	G	T	T	LRP1	c.2244G>T	P.L748L
SH_74	12	7459223	7459223	Missense_Mutation	SNV	G	A	A	ACSM4	c.296G>A	P.R99Q
SH_74	14	20611945	20611945	Silent	SNV	G	A	A	OR4N5	c.51G>A	P.L17L
SH_74	14	65252602	65252602	Missense_Mutation	SNV	A	G	G	SPTB	c.3629T>C	P.F1210S
SH_74	17	35836890	35836890	Splice_Site	SNV	C	A	A	TADA2A	c.1147-12C>A	
SH_74	19	13211506	13211506	Missense_Mutation	SNV	C	T	T	LYL1	c.392G>A	P.R131Q
SH_74	19	19576376	19576376	Silent	SNV	C	T	T	GATAD2A	c.222C>T	P.G74G
SH_74	19	50298881	50298881	Splice_Site	SNV	C	T	T	AP2A1	c.706-6C>T	
SH_74	19	50549938	50549938	Silent	SNV	C	T	T	ZNF473	c.2238C>T	P.V746V
SH_74	2	138414474	138414474	Missense_Mutation	SNV	T	A	A	THSD7B	c.4214T>A	P.I1405K
SH_74	2	177017521	177017521	Missense_Mutation	SNV	A	G	G	HOXD4	c.619A>G	P.M207V
SH_74	2	65541233	65541233	Missense_Mutation	SNV	C	T	T	SPRED2	c.650G>A	P.R217Q
SH_74	3	4687363	4687363	Missense_Mutation	SNV	G	A	A	ITPR1	c.806G>A	P.R269Q
SH_74	3	52443565	52443565	Splice_Site	SNV	C	T	T	BAP1	c.122+5G>A	
SH_74	3	97251308	97251308	Missense_Mutation	SNV	A	T	T	EPHA6	c.483A>T	P.R161S
SH_74	4	30724929	30724929	Missense_Mutation	SNV	G	A	A	PCDH7	c.1885G>A	P.V629M
SH_74	5	161520898	161520898	Missense_Mutation	SNV	A	G	G	GABRG2	c.172A>G	P.T58A
SH_74	5	37179516	37179516	Missense_Mutation	SNV	T	C	C	C5orf42	c.5767A>G	P.R1923G
SH_74	5	40955662	40955662	Splice_Site	SNV	C	T	T	C7	c.1260+7C>T	
SH_74	6	25770603	25770603	Splice_Site	SNV	T	C	C	SLC17A4	c.370-9T>C	
SH_74	6	31630061	31630061	Silent	SNV	G	A	A	GPANK1	c.1053C>T	P.Y351Y
SH_74	6	96971097	96971097	Silent	SNV	C	G	G	UFL1	c.153C>G	P.L51L
SH_74	7	151879655	151879655	Nonsense_Mutation	SNV	G	A	A	KMT2C	c.5290C>T	P.Q1764*
SH_74	9	133730260	133730260	Missense_Mutation	SNV	G	T	T	ABL1	c.326G>T	P.G109V

SH_74	9	43627372	43627372	Missense_Mutation	SNV	T	C	C	SPATA31A6	c.1315A>G	P.T439A
SH_74	9	97555152	97555152	Missense_Mutation	SNV	A	G	G	C9orf3	c.1070A>G	P.N357S
SH_74	X	129058934	129058934	Missense_Mutation	SNV	A	T	T	UTP14A	c.1356A>T	P.R452S
SH_79	1	152286108	152286108	Silent	SNV	G	A	A	FLG	c.1254C>T	P.S418S
SH_79	1	230883258	230883258	Missense_Mutation	SNV	C	T	T	CAPN9	c.16C>T	P.R6W
SH_79	1	55181597	55181597	Nonsense_Mutation	SNV	C	T	T	TTC4	c.16C>T	P.Q6*
SH_79	1	86934709	86934709	Missense_Mutation	SNV	G	A	A	CLCA1	c.55G>A	P.A19T
SH_79	12	49361759	49361759	Missense_Mutation	SNV	C	A	A	WNT10B	c.681G>T	P.M227I
SH_79	12	57872896	57872896	Silent	SNV	G	A	A	ARHGAP9	c.294C>T	P.P98P
SH_79	15	55932003	55932003	Missense_Mutation	SNV	G	A	A	PRTG	c.2161C>T	P.P721S
SH_79	18	10789289	10789289	Nonsense_Mutation	SNV	G	A	A	PIEZO2	c.1957C>T	P.Q653*
SH_79	2	113514408	113514408	Silent	SNV	T	C	C	CKAP2L	c.45A>G	P.L15L
SH_79	2	28739716	28739716	Nonsense_Mutation	SNV	G	T	T	PLB1	c.97G>T	P.E33*
SH_79	3	135789393	135789393	Missense_Mutation	SNV	C	T	T	PPP2R3A	c.2543C>T	P.T848M
SH_79	5	176887561	176887561	Splice_Site	SNV	G	A	A	DBN1	c.832-5C>T	
SH_79	8	142151322	142151322	Silent	SNV	T	C	C	DENND3	c.282T>C	P.N94N
SH_79	8	36790435	36790435	Splice_Site	SNV	C	T	T	KCNU1	c.2932-3C>T	
SH_79	9	117849127	117849127	Missense_Mutation	SNV	C	T	T	TNC	c.883G>A	P.V295M
SH_79	9	35609179	35609179	Missense_Mutation	SNV	T	C	C	TESK1	c.841T>C	P.S281P
SH_79	X	9866263	9866263	Missense_Mutation	SNV	G	A	A	SHROOM2	c.2824G>A	P.D942N
SH_80	1	110051093	110051093	Missense_Mutation	SNV	C	T	T	AMIGO1	c.442G>A	P.A148T
SH_80	1	113933701	113933701	Nonsense_Mutation	SNV	C	T	T	MAGI3	c.46C>T	P.Q16*
SH_80	1	115256529	115256529	Missense_Mutation	SNV	T	C	C	NRAS	c.182A>G	P.Q61R
SH_80	1	115256530	115256530	Missense_Mutation	SNV	G	T	T	NRAS	c.181C>A	P.Q61K
SH_80	1	158263012	158263012	Missense_Mutation	SNV	T	A	A	CD1C	c.900T>A	P.F300L
SH_80	1	161495275	161495275	Missense_Mutation	SNV	T	C	C	HSPA6	c.827T>C	P.L276P
SH_80	1	174987615	174987615	Missense_Mutation	SNV	G	C	C	MRPS14	c.143C>G	P.A48G
SH_80	1	186106036	186106036	Missense_Mutation	SNV	T	A	A	HMCN1	c.13549T>A	P.S4517T
SH_80	1	186281392	186281392	Nonsense_Mutation	SNV	T	A	A	PRG4	c.3756T>A	P.Y1252*
SH_80	1	232940856	232940856	Silent	SNV	G	A	A	MAP10	c.87G>A	P.R29R
SH_80	1	234556514	234556514	Silent	SNV	A	C	C	TARBP1	c.3489T>G	P.S1163S
SH_80	10	105233320	105233320	Missense_Mutation	SNV	C	T	T	CALHM3	c.685G>A	P.E229K
SH_80	10	16893363	16893363	Missense_Mutation	SNV	C	T	T	CUBN	c.9534G>A	P.M3178I
SH_80	10	49986752	49986752	Missense_Mutation	SNV	G	A	A	WDFY4	c.3272G>A	P.R1091H
SH_80	11	106680764	106680764	Silent	SNV	C	A	A	GUCY1A2	c.1647G>T	P.L549L
SH_80	11	1220154	1220154	Missense_Mutation	SNV	G	T	T	MUC5AC	c.4702G>T	P.G1568C
SH_80	11	85397172	85397172	Nonsense_Mutation	SNV	A	G	G	CCDC89	c.2T>C	P.M1?
SH_80	12	57389405	57389405	Missense_Mutation	SNV	T	C	C	GPR182	c.412T>C	P.Y138H
SH_80	12	75804410	75804410	Missense_Mutation	SNV	A	G	G	GLIPR1L2	c.431A>G	P.Y144C
SH_80	12	80714376	80714376	Missense_Mutation	SNV	A	T	T	OTOGL	c.3950A>T	P.Y1317F
SH_80	14	93723599	93723599	Missense_Mutation	SNV	A	G	G	BTBD7	c.1550T>C	P.L517S
SH_80	16	30616450	30616450	Missense_Mutation	SNV	C	T	T	ZNF689	c.638G>A	P.R213H
SH_80	16	84516259	84516259	Missense_Mutation	SNV	G	A	A	TLDC1	c.1016C>T	P.T339M
SH_80	19	10421576	10421576	Silent	SNV	C	A	A	FDX1L	c.357G>T	P.V119V
SH_80	19	38673170	38673170	Missense_Mutation	SNV	C	T	T	SIPA1L3	c.4220C>T	P.S1407L
SH_80	19	44531609	44531609	Missense_Mutation	SNV	A	T	T	ZNF222	c.229A>T	P.M77L
SH_80	19	4902616	4902616	Silent	SNV	G	A	A	ARRDC5	c.264C>T	P.Y88Y
SH_80	19	55143558	55143558	Silent	SNV	G	A	A	LILRB1	c.531G>A	P.S177S
SH_80	2	166848129	166848129	Nonsense_Mutation	SNV	G	A	A	SCN1A	c.5656C>T	P.R1886*
SH_80	2	219320415	219320415	Missense_Mutation	SNV	T	C	C	USP37	c.2740A>G	P.I914V
SH_80	2	226447616	226447616	Missense_Mutation	SNV	T	A	A	NYAP2	c.1483T>A	P.Y495N
SH_80	20	53205321	53205321	Missense_Mutation	SNV	A	G	G	DOK5	c.61A>G	P.T21A
SH_80	22	46773045	46773045	Missense_Mutation	SNV	G	C	C	CELSR1	c.7497C>G	P.N2499K
SH_80	3	134670397	134670397	Missense_Mutation	SNV	C	T	T	EPHB1	c.308C>T	P.P103L
SH_80	3	52443757	52443757	Frame_Shift_Del	INDEL	G	-	-	BAP1	c.40delC	P.L14fs
SH_80	3	97983712	97983712	Missense_Mutation	SNV	G	A	A	OR5H6	c.584G>A	P.C195Y
SH_80	4	79500256	79500256	Missense_Mutation	SNV	A	G	G	ANXA3	c.179A>G	P.Y60C
SH_80	5	155756543	155756543	Splice_Site	SNV	G	T	T	SGCD	c.-43-1G>T	
SH_80	5	72383891	72383903	Frame_Shift_Del	INDEL	AGGACGATA	-	-	FCHO2	2321delCAGGA(	P.Y773fs
SH_80	6	35773583	35773583	Nonsense_Mutation	SNV	C	T	T	LHFPL5	c.136C>T	P.Q46*
SH_80	6	40399853	40399853	Missense_Mutation	SNV	A	G	G	LRFN2	c.1000T>C	P.S334P
SH_80	7	135421920	135421920	Missense_Mutation	SNV	G	T	T	FAM180A	c.104C>A	P.P35Q
SH_80	7	140159588	140159588	Missense_Mutation	SNV	T	C	C	MKRN1	c.463A>G	P.N155D
SH_80	7	150900168	150900168	Missense_Mutation	SNV	C	A	A	IQCA1L	c.474G>T	P.M158I
SH_80	8	110510963	110510963	Missense_Mutation	SNV	G	T	T	PKHD1L1	c.10791G>T	P.M3597I
SH_80	9	124532835	124532835	Missense_Mutation	SNV	C	A	A	DAB2IP	c.1826C>A	P.S609Y
SH_80	9	140136272	140136272	Nonsense_Mutation	SNV	G	T	T	TUBB4B	c.157G>T	P.E53*
SH_80	X	100645492	100645492	Missense_Mutation	SNV	A	T	T	BTK	c.46T>A	P.C16S
SH_80	X	124454177	124454177	Missense_Mutation	SNV	G	A	A	TEX13C	c.209G>A	P.S70N
SH_80	X	13753378	13753378	Missense_Mutation	SNV	T	G	G	OFD1	c.24T>G	P.F8L
SH_80	X	15403215	15403215	Missense_Mutation	SNV	C	T	T	PIR	c.784G>A	P.E262K
SH_80	X	23019839	23019839	Silent	SNV	A	G	G	DDX53	c.1665A>G	P.V555V
SH_81	1	149785218	149785218	Missense_Mutation	SNV	T	C	C	HIST2H3D	c.19A>G	P.T7A
SH_81	1	233802459	233802459	Silent	SNV	G	A	A	KCNK1	c.474G>A	P.V158V
SH_81	1	241797231	241797231	Missense_Mutation	SNV	G	T	T	CHML	c.1838C>A	P.P613Q
SH_81	11	47193243	47193243	Missense_Mutation	SNV	C	T	T	ARFGAP2	c.697G>A	P.D233N
SH_81	12	51754557	51754557	Missense_Mutation	SNV	T	G	G	GALNT6	c.1115A>C	P.Y372S
SH_81	12	76752543	76752543	Missense_Mutation	SNV	C	A	A	OSBPL8	c.2251G>T	P.V751L
SH_81	14	102805285	102805285	Missense_Mutation	SNV	C	T	T	ZNF839	c.1444C>T	P.R482C
SH_81	14	88983510	88983510	Silent	SNV	T	C	C	PTPN21	c.276A>G	P.A92A
SH_81	16	57508784	57508784	Missense_Mutation	SNV	C	T	T	DOK4	c.520G>A	P.V174M
SH_81	17	74053597	74053597	Nonsense_Mutation	SNV	G	A	A	SRP68	c.751C>T	P.Q251*
SH_81	2	125555700	125555700	Missense_Mutation	SNV	C	T	T	CNTNAP5	c.3017C>T	P.T1006M
SH_81	2	185803163	185803163	Missense_Mutation	SNV	G	T	T	ZNF804A	c.3040G>T	P.G1014C
SH_81	2	198266713	198266713	Missense_Mutation	SNV	C	T	T	SF3B1	c.2219G>A	P.G740E
SH_81	2	80085156	80085156	Missense_Mutation	SNV	G	A	A	CTNNA2	c.316G>A	P.A106T
SH_81	3	52831902	52831902	Missense_Mutation	SNV	G	A	A	ITIH3	c.619G>A	P.D207N
SH_81	5	10397404	10397404	Splice_Site	SNV	G	A	A	MARCH6	c.718-1G>A	
SH_81	5	127493768	127493768	Missense_Mutation	SNV	G	A	A	SLC12A2	c.2387G>A	P.G796D
SH_81	5	156589869	156589869	Silent	SNV	G	A	A	FAM71B	c.1407C>T	P.S469S
SH_81	6	137519730	137519730	Missense_Mutation	SNV	T	G	G	IFNGR1	c.908A>C	P.K303T
SH_81	6	74073373	74073373	Silent	SNV	G	A	A	KHDC3L	c.444G>A	P.S148S
SH_81	7	140453149	140453149	Missense_Mutation	SNV	C	G	G	BRAF	c.1786G>C	P.G596R
SH_81	9	101891223	101891226	Frame_Shift_Del	INDEL	ACAG	-	-	TGFBR1	187_190delGA(	P.D63fs
SH_82	1	161276581	161276581	Missense_Mutation	SNV	T	A	A	MPZ	c.365A>T	P.N122I
SH_82	1	208219299	208219299	Missense_Mutation	SNV	T	C	C	PLXNA2	c.3419A>G	P.Y1140C
SH_82	18	57136753	57136753	Nonsense_Mutation	SNV	G	A	A	CCBE1	c.352C>T	P.R118*
SH_82	7	100680600	100680600	Missense_Mutation	SNV	C	A	A	MUC17	c.5903C>A	P.T1968K

SH_82	8	114111096	114111096	Missense_Mutation	SNV	C	G	G	CSMD3	c.806G>C	P.W269S
SH_82	X	77224504	77224504	Missense_Mutation	SNV	G	T	T	PGAM4	c.632C>A	P.P211Q
SH_85	1	151027510	151027510	Silent	SNV	G	A	A	CDC42SE1	c.147C>T	P.A49A
SH_85	1	158299402	158299402	Missense_Mutation	SNV	G	A	A	CD1B	c.644C>T	P.P215L
SH_85	1	158746718	158746718	Missense_Mutation	SNV	C	A	A	OR6N2	c.708G>T	P.K236N
SH_85	1	75097523	75097523	Missense_Mutation	SNV	C	A	A	ERICH3	c.693G>T	P.M231I
SH_85	10	50819847	50819847	Missense_Mutation	SNV	A	G	G	SLC18A3	c.1061A>G	P.H354R
SH_85	10	99656674	99656674	Missense_Mutation	SNV	C	T	T	CRTAC1	c.1208G>A	P.R403Q
SH_85	12	49165607	49165607	Silent	SNV	A	G	G	ADCY6	c.2937T>C	P.A979A
SH_85	14	24706305	24706305	Missense_Mutation	SNV	T	C	C	GMPR2	c.494T>C	P.V165A
SH_85	14	47324319	47324322	Frame_Shift_Del	INDEL	TTGT	-	-	MDGA2	581_2584delAC	P.T861fs
SH_85	15	24921631	24921631	Missense_Mutation	SNV	G	C	C	NPAP1	c.617G>C	P.G206A
SH_85	15	41819471	41819471	Missense_Mutation	SNV	C	T	T	RPAP1	c.1640G>A	P.R547Q
SH_85	15	48722919	48722919	Missense_Mutation	SNV	A	G	G	FBN1	c.6820T>C	P.C2274R
SH_85	16	71022312	71022312	Missense_Mutation	SNV	G	A	A	HYDIN	c.3968C>T	P.S1323F
SH_85	17	29667567	29667567	Silent	SNV	G	A	A	NF1	c.6966G>A	P.V2322V
SH_85	17	7577121	7577121	Missense_Mutation	SNV	G	T	T	TP53	c.817C>A	P.R273S
SH_85	18	29340201	29340201	Nonsense_Mutation	SNV	G	A	A	SLC25A52	c.454C>T	P.Q152*
SH_85	19	14071278	14071278	Missense_Mutation	SNV	G	A	A	DCAF15	c.1633G>A	P.G545S
SH_85	19	18700265	18700265	Silent	SNV	C	T	T	C19orf60	c.225C>T	P.G75G
SH_85	19	47572471	47572471	Missense_Mutation	SNV	G	C	C	ZC3H4	c.2276C>G	P.P759R
SH_85	2	181925392	181925392	Missense_Mutation	SNV	G	A	A	UBE2E3	c.379G>A	P.V127I
SH_85	2	239056572	239056572	Silent	SNV	G	C	C	KLHL30	c.1248G>C	P.S416S
SH_85	22	28397455	28397455	Silent	SNV	G	A	A	TTC28	c.4305C>T	P.A1435A
SH_85	3	188327596	188327596	Silent	SNV	T	C	C	LPP	c.1077T>C	P.P359P
SH_85	4	126239141	126239141	Silent	SNV	C	T	T	FAT4	c.1575C>T	P.I525I
SH_85	5	1221295	1221295	Missense_Mutation	SNV	G	A	A	SLC6A19	c.1568G>A	P.G523D
SH_85	5	433010	433010	Missense_Mutation	SNV	C	A	A	AHRR	c.1072C>A	P.L358M
SH_85	5	76249961	76249961	Missense_Mutation	SNV	A	G	G	CRHBP	c.283A>G	P.I95V
SH_85	6	165752824	165752824	Silent	SNV	T	C	C	PDE10A	c.2121A>G	P.G707G
SH_85	6	18171617	18171617	Silent	SNV	T	C	C	KDM1B	c.441T>C	P.C147C
SH_85	6	24278368	24278368	Silent	SNV	T	C	C	DCDC2	c.831A>G	P.K277K
SH_85	6	41655744	41655744	Missense_Mutation	SNV	A	G	G	TFEB	c.572T>C	P.L191P
SH_85	6	52617677	52617677	Missense_Mutation	SNV	T	C	C	GSTA2	c.389A>G	P.N130S
SH_85	7	73938437	73938437	Missense_Mutation	SNV	T	C	C	GTF2IRD1	c.1139T>C	P.I380T
SH_85	9	93375718	93375718	Missense_Mutation	SNV	A	G	G	DIRAS2	c.392T>C	P.V131A
SH_85	X	102586465	102586465	Frame_Shift_Del	INDEL	T	-	-	TCEAL7	c.134delT	P.L45fs
SH_86	1	159912834	159912834	Missense_Mutation	SNV	C	T	T	IGSF9	c.166G>A	P.E56K
SH_86	1	16475486	16475486	Missense_Mutation	SNV	G	C	C	EPHA2	c.210C>G	P.C70W
SH_86	1	175334260	175334260	Missense_Mutation	SNV	T	A	A	TNR	c.1474A>T	P.T492S
SH_86	1	27276585	27276585	Frame_Shift_Ins	INDEL	-	C	C	KDF1	c.1171dupG	P.A391fs
SH_86	1	94370099	94370099	Missense_Mutation	SNV	T	C	C	GCLM	c.172A>G	P.I58V
SH_86	10	96602680	96602680	Missense_Mutation	SNV	G	A	A	CYP2C19	c.1048G>A	P.A350T
SH_86	16	29923194	29923194	Splice_Site	SNV	G	A	A	KCTD13	c.505-6C>T	
SH_86	16	88501585	88501585	Silent	SNV	C	T	T	ZNF469	c.7623C>T	P.H2541H
SH_86	2	179421718	179421718	Missense_Mutation	SNV	G	T	T	TTN	c.83240C>A	P.T27747N
SH_86	2	27522156	27522156	Missense_Mutation	SNV	C	T	T	TRIM54	c.385C>T	P.H129Y
SH_86	3	148559623	148559623	Missense_Mutation	SNV	G	A	A	CPB1	c.488G>A	P.G163E
SH_86	5	139244708	139244708	Missense_Mutation	SNV	T	C	C	NRG2	c.1163A>G	P.N388S
SH_86	5	161118994	161118994	Missense_Mutation	SNV	C	A	A	GABRA6	c.874C>A	P.H292N
SH_86	6	146480713	146480713	Silent	SNV	C	T	T	GRM1	c.930C>T	P.G310G
SH_86	6	30858846	30858861	Frame_Shift_Del	INDEL	GTCATGAGC	-	-	DDR1	9delCGGGTCATG	P.R172fs
SH_86	6	7413144	7413144	Missense_Mutation	SNV	G	A	A	RIOK1	c.1412G>A	P.G471E
SH_86	7	146536869	146536869	Missense_Mutation	SNV	G	A	A	CNTNAP2	c.275G>A	P.R92Q
SH_87	1	26671702	26671702	Missense_Mutation	SNV	C	G	G	AIM1L	c.1447G>C	P.G483R
SH_87	10	129868571	129868571	Missense_Mutation	SNV	T	A	A	PTPRE	c.1183T>A	P.Y395N
SH_87	11	118392772	118392772	Missense_Mutation	SNV	G	T	T	KMT2A	c.11795G>T	P.R3932L
SH_87	11	1252747	1252747	Missense_Mutation	SNV	G	T	T	MUC5B	c.1635G>T	P.Q545H
SH_87	11	134237144	134237144	Splice_Site	SNV	T	C	C	GLB1L2	c.805-7T>C	
SH_87	11	407887	407887	Silent	SNV	C	T	T	SIGIRR	c.411G>A	P.L137L
SH_87	12	46245900	46245916	Frame_Shift_Del	INDEL	ATGGAGCAT	-	-	ARID2	2delGAATGGAG	P.N1333fs
SH_87	12	52822234	52822234	Silent	SNV	A	T	T	KRT75	c.1188T>A	P.A396A
SH_87	13	109704736	109704736	Missense_Mutation	SNV	C	G	G	MYO16	c.2961C>G	P.F987L
SH_87	15	64017691	64017691	Missense_Mutation	SNV	G	T	T	HERC1	c.3368C>A	P.P1123H
SH_87	15	76183325	76183325	Silent	SNV	C	G	G	UBE2Q2	c.951C>G	P.G317G
SH_87	16	30663177	30663177	Silent	SNV	G	A	A	PRR14	c.84G>A	P.R28R
SH_87	16	70883779	70883779	Silent	SNV	C	T	T	HYDIN	c.12723G>A	P.Q4241Q
SH_87	17	72301531	72301531	Silent	SNV	A	T	T	DNAI2	c.1161A>T	P.T387T
SH_87	17	7578226	7578226	Missense_Mutation	SNV	T	A	A	TP53	c.623A>T	P.D208V
SH_87	2	179419416	179419416	Missense_Mutation	SNV	A	T	T	TTN	c.83735T>A	P.L27912H
SH_87	2	217543719	217543719	Frame_Shift_Ins	INDEL	-	T	T	IGFBP5	c.420dupA	P.H141fs
SH_87	2	32602801	32602801	Missense_Mutation	SNV	G	T	T	BIRC6	c.471G>T	P.Q157H
SH_87	22	19839462	19839462	Missense_Mutation	SNV	T	C	C	C22orf29	c.323A>G	P.D108G
SH_87	3	9146461	9146461	Missense_Mutation	SNV	T	C	C	SRGAP3	c.326A>G	P.E109G
SH_87	4	3449285	3449285	Silent	SNV	G	T	T	HGFAC	c.1443G>T	P.T481T
SH_87	4	4204226	4204226	Missense_Mutation	SNV	T	A	A	OTOP1	c.679A>T	P.N227Y
SH_87	5	163276	163276	Missense_Mutation	SNV	T	A	A	PLEKHG4B	c.2021T>A	P.L674Q
SH_87	6	129835749	129835749	Splice_Site	SNV	T	C	C	LAMA2	c.9211+9T>C	
SH_87	7	138546006	138546006	Missense_Mutation	SNV	C	T	T	KIAA1549	c.5126G>A	P.G1709D
SH_87	7	37956008	37956008	Silent	SNV	G	A	A	SFRP4	c.132C>T	P.N44N
SH_87	8	145745694	145745694	Missense_Mutation	SNV	G	A	A	LRRC14	c.402G>A	P.M134I
SH_87	8	48880035	48880035	Silent	SNV	G	A	A	MCM4	c.1164G>A	P.V388V
SH_87	9	135798860	135798862	Missense_Mutation	INDEL	ACA	-	-	TSC1	c.381_383delTG	P.V128del
SH_87	9	33794883	33794883	Splice_Site	SNV	C	G	G	PRSS3	c.82+12C>G	
SH_87	X	122336563	122336563	Missense_Mutation	SNV	A	C	C	GRIA3	c.344A>C	P.K115T
SH_87	X	152959638	152959638	Silent	SNV	C	T	T	SLC6A8	c.1278C>T	P.S426S
SH_88	1	43885836	43885836	Missense_Mutation	SNV	G	A	A	SZT2	c.1103G>A	P.R368H
SH_88	1	94930315	94930315	Splice_Site	INDEL	-	T	T	ABCD3	c.148-8dupT	
SH_88	11	64701305	64701305	Missense_Mutation	SNV	G	A	A	PPP2R5B	c.1483G>A	P.G495S
SH_88	11	82877707	82877707	Missense_Mutation	SNV	A	T	T	PCF11	c.1768A>T	P.S590C
SH_88	13	113770009	113770009	Missense_Mutation	SNV	G	A	A	F7	c.466G>A	P.G156S
SH_88	14	23540747	23540747	Missense_Mutation	SNV	G	A	A	ACIN1	c.5C>T	P.S2F
SH_88	17	45421670	45421670	Splice_Site	SNV	C	T	T	EFCAB13	c.434+12C>T	
SH_88	17	76456275	76456275	Missense_Mutation	SNV	G	T	T	DNAH17	c.9404C>A	P.A3135E
SH_88	18	30913366	30913366	Splice_Site	SNV	T	G	G	CCDC178	c.659-8A>C	
SH_88	19	57327976	57327976	Missense_Mutation	SNV	G	A	A	PEG3	c.1834C>T	P.P612S
SH_88	2	40656342	40656342	Frame_Shift_Ins	INDEL	-	T	T	SLC8A1	c.1078dupA	P.S360fs
SH_88	20	4776504	4776504	Missense_Mutation	SNV	A	G	G	RASSF2	c.244T>C	P.S82P

SH_88	21	31869297	31869297	Silent	SNV	G	A	A	KRTAP19-4	c.132C>T	P.N44N
SH_88	3	14206941	14206941	Missense_Mutation	SNV	T	C	C	XPC	c.766A>G	P.N256D
SH_88	4	74279300	74279300	Frame_Shift_Del	INDEL	G	-	-	ALB	c.1007delG	P.S336fs
SH_88	5	137756620	137756620	Missense_Mutation	SNV	C	T	T	KDM3B	c.3941C>T	P.P1314L
SH_88	6	116441262	116441262	Missense_Mutation	SNV	A	C	C	COL10A1	c.2017T>G	P.S673A
SH_88	6	44988339	44988339	Missense_Mutation	SNV	G	A	A	SUPT3H	c.217C>T	P.R73W
SH_88	6	75865411	75865411	Missense_Mutation	SNV	G	A	A	COL12A1	c.3410C>T	P.P1137L
SH_88	7	141722246	141722246	Splice_Site	INDEL	T	-	-	MGAM	c.882+9delT	
SH_88	8	27296873	27296873	Missense_Mutation	SNV	A	C	C	PTK2B	c.1792A>C	P.N598H
SH_88	9	5713917	5713917	Silent	SNV	G	A	A	RIC1	c.354G>A	P.G118G
SH_88	X	111020157	111020157	Missense_Mutation	SNV	C	T	T	TRPC5	c.2306G>A	P.S769N
SH_88	X	151138721	151138721	Missense_Mutation	SNV	T	G	G	GABRE	c.210A>C	P.E70D
SH_89	10	119805477	119805477	Missense_Mutation	SNV	G	C	C	RAB11FIP2	c.198C>G	P.F66L
SH_89	15	66729175	66729175	Missense_Mutation	SNV	G	A	A	MAP2K1	c.383G>A	P.G128D
SH_89	18	31323712	31323712	Silent	SNV	T	A	A	ASXL3	c.3900T>A	P.I1300I
SH_89	18	5145596	5145596	Missense_Mutation	SNV	C	T	T	AKAIN1	c.175G>A	P.V59I
SH_89	19	15754772	15754772	Missense_Mutation	SNV	C	T	T	CYP4F3	c.254C>T	P.P85L
SH_89	5	121759202	121759202	Missense_Mutation	SNV	A	G	G	SNCAIP	c.770A>G	P.D257G
SH_89	5	140031727	140031727	Silent	SNV	G	A	A	IK	c.231G>A	P.K77K
SH_90	1	109198321	109198321	Splice_Site	SNV	C	A	A	HENMT1	c.151-1G>T	
SH_90	1	109810543	109810543	Missense_Mutation	SNV	G	T	T	CELSR2	c.6179G>T	P.R2060L
SH_90	1	109953700	109953700	Missense_Mutation	SNV	C	T	T	PSMA5	c.320G>A	P.C107Y
SH_90	1	110085864	110085864	Nonsense_Mutation	SNV	C	T	T	GPR61	c.220C>T	P.R74*
SH_90	1	12907955	12907955	Missense_Mutation	SNV	G	T	T	HNRNPCL1	c.188C>A	P.A63D
SH_90	1	13839850	13839850	Missense_Mutation	SNV	C	A	A	LRRC38	c.239G>T	P.G80V
SH_90	1	144868013	144868013	Missense_Mutation	SNV	C	T	T	PDE4DIP	c.5108G>A	P.G1703D
SH_90	1	155796708	155796708	Missense_Mutation	SNV	G	C	C	GON4L	c.617C>G	P.S206C
SH_90	1	157068490	157068490	Splice_Site	SNV	G	T	T	ETV3L	c.486+8C>A	
SH_90	1	161277173	161277173	Missense_Mutation	SNV	C	G	G	MPZ	c.109G>C	P.E37Q
SH_90	1	163138132	163138132	Missense_Mutation	SNV	C	A	A	RGS5	c.71G>T	P.G24V
SH_90	1	175046848	175046848	Missense_Mutation	SNV	C	A	A	TNN	c.294C>A	P.D98E
SH_90	1	179035966	179035966	Missense_Mutation	SNV	A	T	T	FAM20B	c.970A>T	P.I324F
SH_90	1	179562594	179562594	Splice_Site	SNV	G	T	T	TDRD5	c.233-1G>T	
SH_90	1	181688996	181688996	Missense_Mutation	SNV	C	T	T	CACNA1E	c.1748C>T	P.T583I
SH_90	1	182617404	182617404	Silent	SNV	C	T	T	RGS8	c.228G>A	P.T76T
SH_90	1	190067439	190067439	Silent	SNV	G	A	A	BRINP3	c.1704C>T	P.G568G
SH_90	1	201838826	201838826	Missense_Mutation	SNV	G	T	T	IPO9	c.2113G>T	P.D705Y
SH_90	1	22165342	22165342	Missense_Mutation	SNV	C	A	A	HSPG2	c.10129G>T	P.A3377S
SH_90	1	221912939	221912939	Missense_Mutation	SNV	C	A	A	DUSP10	c.148G>T	P.V50F
SH_90	1	228210370	228210370	Missense_Mutation	SNV	C	T	T	WNT3A	c.74C>T	P.S25L
SH_90	1	228482105	228482105	Missense_Mutation	SNV	T	A	A	OBSCN	c.11384T>A	P.L3795H
SH_90	1	231472123	231472123	Missense_Mutation	SNV	G	C	C	EXOC8	c.1369C>G	P.H457D
SH_90	1	232596667	232596667	Missense_Mutation	SNV	T	C	C	SIPA1L2	c.3061A>G	P.I1021V
SH_90	1	232650572	232650572	Nonsense_Mutation	SNV	C	A	A	SIPA1L2	c.514G>T	P.E172*
SH_90	1	237729945	237729945	Missense_Mutation	SNV	C	A	A	RYR2	c.3293C>A	P.A1098D
SH_90	1	240370867	240370867	Missense_Mutation	SNV	G	T	T	FMN2	c.2767G>T	P.G923C
SH_90	1	26316019	26316019	Missense_Mutation	SNV	C	A	A	PAFAH2	c.164G>T	P.R55L
SH_90	1	29639164	29639164	Missense_Mutation	SNV	G	T	T	PTPRU	c.3164G>T	P.R1055L
SH_90	1	40228862	40228862	Missense_Mutation	SNV	C	A	A	BMP8B	c.961G>T	P.A321S
SH_90	1	43022042	43022042	Missense_Mutation	SNV	G	T	T	CCDC30	c.641G>T	P.S214I
SH_90	1	45166656	45166656	Silent	SNV	G	A	A	C1orf228	c.504G>A	P.V168V
SH_90	1	897290	897290	Missense_Mutation	SNV	T	G	G	KLHL17	c.574T>G	P.S192A
SH_90	10	13152488	13152488	Splice_Site	SNV	G	T	T	OPTN	c.369+12G>T	
SH_90	10	32311157	32311160	Frame_Shift_Del	INDEL	TCAG	-	-	KIF5B	940_1943delCT	P.T647fs
SH_90	10	50339858	50339858	Missense_Mutation	SNV	C	A	A	FAM170B	c.652G>T	P.A218S
SH_90	10	61828560	61828560	Missense_Mutation	SNV	C	T	T	ANK3	c.12079G>A	P.D4027N
SH_90	11	102206691	102206691	Splice_Site	SNV	A	G	G	BIRC3	c.1325-6A>G	
SH_90	11	10477956	10477956	Splice_Site	SNV	G	T	T	AMPD3	c.16+7G>T	
SH_90	11	118359339	118359339	Missense_Mutation	SNV	G	T	T	KMT2A	c.4343G>T	P.C1448F
SH_90	11	123477491	123477491	Missense_Mutation	SNV	G	T	T	GRAMD1B	c.1090G>T	P.D364Y
SH_90	11	130275690	130275690	Missense_Mutation	SNV	G	T	T	ADAMTS8	c.2433C>A	P.D811E
SH_90	11	16847695	16847695	Missense_Mutation	SNV	C	A	A	PLEKHA7	c.1315G>T	P.A439S
SH_90	11	17633783	17633783	Missense_Mutation	SNV	C	A	A	OTOG	c.6234C>A	P.H2078Q
SH_90	11	20658776	20658776	Missense_Mutation	SNV	G	A	A	SLC6A5	c.1094G>A	P.R365H
SH_90	11	46910976	46910976	Missense_Mutation	SNV	T	C	C	LRP4	c.2201A>G	P.H734R
SH_90	11	4976182	4976182	Silent	SNV	C	T	T	OR51A2	c.762G>A	P.L254L
SH_90	11	551827	551827	Missense_Mutation	SNV	G	T	T	LRRC56	c.973G>T	P.G325C
SH_90	11	55587398	55587398	Missense_Mutation	SNV	G	T	T	OR5D18	c.293G>T	P.C98F
SH_90	11	61563713	61563713	Missense_Mutation	SNV	G	A	A	FEN1	c.880G>A	P.V294M
SH_90	11	63683082	63683082	Missense_Mutation	SNV	G	C	C	RCOR2	c.129C>G	P.D43E
SH_90	11	66278161	66278161	Missense_Mutation	SNV	G	T	T	BBS1	c.31G>T	P.A11S
SH_90	11	71939475	71939475	Silent	SNV	G	C	C	INPPL1	c.330G>C	P.V110V
SH_90	11	7817647	7817647	Missense_Mutation	SNV	C	A	A	OR5P2	c.843G>T	P.L281F
SH_90	11	78602346	78602346	Missense_Mutation	SNV	T	G	G	TENM4	c.785A>C	P.Q262P
SH_90	11	87908378	87908378	Missense_Mutation	SNV	C	A	A	RAB38	c.175G>T	P.V59L
SH_90	12	104054121	104054121	Missense_Mutation	SNV	C	T	T	STAB2	c.1747C>T	P.L583F
SH_90	12	117768772	117768772	Silent	SNV	G	T	T	NOS1	c.103C>A	P.R35R
SH_90	12	118821811	118821811	Splice_Site	SNV	T	A	A	SUDS3	c.213-6T>A	
SH_90	12	2558139	2558139	Splice_Site	SNV	T	A	A	CACNA1C	c.478-3T>A	
SH_90	12	49167362	49167362	Silent	SNV	C	A	A	ADCY6	c.2511G>T	P.G837G
SH_90	12	52579220	52579220	Missense_Mutation	SNV	C	T	T	KRT80	c.452G>A	P.G151E
SH_90	12	52985302	52985302	Nonsense_Mutation	SNV	G	T	T	KRT72	c.909C>A	P.Y303*
SH_90	12	55688438	55688438	Silent	SNV	T	A	A	OR6C6	c.579A>T	P.L193L
SH_90	12	55820602	55820602	Missense_Mutation	SNV	A	T	T	OR6C76	c.565A>T	P.T189S
SH_90	12	66232350	66232350	Splice_Site	SNV	G	A	A	HMGA2	c.249+1G>A	
SH_90	12	6965218	6965218	Missense_Mutation	SNV	T	A	A	USP5	c.342T>A	P.F114L
SH_90	12	70929938	70929938	Missense_Mutation	SNV	C	T	T	PTPRB	c.5948G>A	P.G1983D
SH_90	12	9994993	9994993	Missense_Mutation	SNV	C	A	A	KLRF1	c.401C>A	P.T134K
SH_90	13	103391271	103391271	Missense_Mutation	SNV	C	A	A	CCDC168	c.11776G>T	P.A3926S
SH_90	13	20233359	20233359	Missense_Mutation	SNV	T	C	C	MPHOSPH8	c.1721T>C	P.V574A
SH_90	13	36367616	36367616	Missense_Mutation	SNV	C	A	A	DCLK1	c.1024G>T	P.D342Y
SH_90	13	38320109	38320109	Missense_Mutation	SNV	T	C	C	TRPC4	c.862A>G	P.R288G
SH_90	13	52325468	52325468	Missense_Mutation	SNV	T	G	G	WDFY2	c.748T>G	P.Y250D
SH_90	13	88329796	88329796	Missense_Mutation	SNV	G	A	A	SLITRK5	c.2153G>A	P.G718D
SH_90	14	105405796	105405796	Missense_Mutation	SNV	G	T	T	AHNAK2	c.15992C>A	P.S5331Y
SH_90	14	22102091	22102091	Missense_Mutation	SNV	G	T	T	OR10G2	c.908C>A	P.A303D
SH_90	14	24632636	24632636	Silent	SNV	G	T	T	IRF9	c.414G>T	P.V138V
SH_90	14	24649736	24649736	Missense_Mutation	SNV	T	G	G	IPO4	c.3158A>C	P.K1053T

SH_90	14	80669199	80669199	Missense_Mutation	SNV	C	T	T	DIO2	c.655G>A	P.A219T
SH_90	15	33928648	33928648	Missense_Mutation	SNV	G	T	T	RYR3	c.3453G>T	P.M1151I
SH_90	15	48441237	48441237	Missense_Mutation	SNV	C	G	G	MYEF2	c.1559G>C	P.S520T
SH_90	15	48888545	48888545	Missense_Mutation	SNV	C	A	A	FBN1	c.473G>T	P.G158V
SH_90	15	60639885	60639885	Missense_Mutation	SNV	C	A	A	ANXA2	c.964G>T	P.D322Y
SH_90	15	78836555	78836555	Missense_Mutation	SNV	G	T	T	PSMA4	c.233G>T	P.G78V
SH_90	15	83332351	83332351	Missense_Mutation	SNV	G	T	T	AP3B2	c.2305C>A	P.P769T
SH_90	15	91021124	91021124	Splice_Site	SNV	A	G	G	IQGAP1	c.3329+3A>G	
SH_90	16	1505182	1505182	Missense_Mutation	SNV	C	A	A	CLCN7	c.979G>T	P.D327Y
SH_90	16	3490822	3490822	Missense_Mutation	SNV	C	A	A	ZNF597	c.145G>T	P.D49Y
SH_90	16	4650138	4650138	Silent	SNV	G	T	T	C16orf96	c.3246G>T	P.S1082S
SH_90	16	47533729	47533729	Missense_Mutation	SNV	G	A	A	PHKB	c.229G>A	P.G77S
SH_90	16	70916555	70916555	Splice_Site	SNV	C	A	A	HYDIN	c.10215+8G>T	
SH_90	16	86601020	86601020	Missense_Mutation	SNV	G	T	T	FOXC2	c.79G>T	P.A27S
SH_90	17	39458727	39458727	Missense_Mutation	SNV	C	A	A	KRTAP29-1	c.377G>T	P.C126F
SH_90	17	39553443	39553443	Splice_Site	SNV	C	A	A	KRT31	c.348+1G>T	
SH_90	17	39646022	39646022	Missense_Mutation	SNV	C	A	A	KRT36	c.95G>T	P.R32L
SH_90	17	40257105	40257105	Silent	SNV	C	A	A	DHX58	c.1332G>T	P.G444G
SH_90	17	43923828	43923828	Missense_Mutation	SNV	G	T	T	SPPL2C	c.1556G>T	P.R519L
SH_90	17	46928502	46928502	Missense_Mutation	SNV	A	G	G	CALCOCO2	c.667A>G	P.K223E
SH_90	17	61498172	61498172	Missense_Mutation	SNV	C	T	T	TANC2	c.4829C>T	P.A1610V
SH_90	17	61557709	61557709	Missense_Mutation	SNV	A	G	G	ACE	c.667A>G	P.T223A
SH_90	17	66914585	66914585	Missense_Mutation	SNV	T	C	C	ABCA8	c.1891A>G	P.I631V
SH_90	17	71238441	71238441	Silent	SNV	T	A	A	C17orf80	c.1572T>A	P.L524L
SH_90	17	72538991	72538991	Splice_Site	SNV	G	T	T	CD300C	c.527+9C>A	
SH_90	17	7577534	7577534	Missense_Mutation	SNV	C	A	A	TP53	c.747G>T	P.R249S
SH_90	17	78268730	78268730	Missense_Mutation	SNV	T	A	A	RNF213	c.1683T>A	P.F561L
SH_90	18	24127375	24127375	Missense_Mutation	SNV	G	C	C	KCTD1	c.1126C>G	P.P376A
SH_90	18	7002376	7002376	Silent	SNV	G	T	T	LAMA1	c.4269C>A	P.G1423G
SH_90	18	72343937	72343937	Missense_Mutation	SNV	G	A	A	ZNF407	c.962G>A	P.R321Q
SH_90	19	12937655	12937655	Nonsense_Mutation	SNV	G	T	T	RTBDN	c.396C>A	P.C132*
SH_90	19	36370299	36370299	Missense_Mutation	SNV	G	T	T	APLP1	c.1912G>T	P.G638C
SH_90	19	5131936	5131936	Missense_Mutation	SNV	G	T	T	KDM4B	c.1824G>T	P.K608N
SH_90	19	57868600	57868600	Missense_Mutation	SNV	G	T	T	ZNF304	c.1504G>T	P.A502S
SH_90	19	9049310	9049310	Missense_Mutation	SNV	G	T	T	MUC16	c.32321C>A	P.A10774E
SH_90	2	120734565	120734565	Silent	SNV	A	G	G	PTPN4	c.2700A>G	P.Q900Q
SH_90	2	128322829	128322829	Missense_Mutation	SNV	G	A	A	MYO7B	c.154G>A	P.D52N
SH_90	2	160843807	160843807	Missense_Mutation	SNV	G	T	T	PLA2R1	c.1897C>A	P.H633N
SH_90	2	166211091	166211091	Silent	SNV	G	C	C	SCN2A	c.3309G>C	P.V1103V
SH_90	2	166848676	166848676	Missense_Mutation	SNV	G	C	C	SCN1A	c.5109C>G	P.D1703E
SH_90	2	170662182	170662240	Frame_Shift_Del	INDEL	TTGTGGAAG	-	-	SSB	TGTAATTGTGGA	P.R60fs
SH_90	2	171092540	171092540	Missense_Mutation	SNV	G	T	T	MYO3B	c.643G>T	P.A215S
SH_90	2	179436743	179436743	Missense_Mutation	SNV	G	A	A	TTN	c.69193C>T	P.P23065S
SH_90	2	18112837	18112837	Missense_Mutation	SNV	G	T	T	KCNS3	c.562G>T	P.A188S
SH_90	2	209308253	209308253	Missense_Mutation	SNV	A	C	C	PTH2R	c.357A>C	P.K119N
SH_90	2	220331955	220331955	Missense_Mutation	SNV	G	T	T	SPEG	c.2941G>T	P.A981S
SH_90	2	220371069	220371069	Missense_Mutation	SNV	A	T	T	GMPPA	c.1087A>T	P.N363Y
SH_90	2	220494354	220494354	Missense_Mutation	SNV	C	T	T	SLC4A3	c.548C>T	P.A183V
SH_90	2	222298873	222298874	Frame_Shift_Del	INDEL	AC	-	-	EPHA4	2484_2485delG	P.M828fs
SH_90	2	223160365	223160365	Silent	SNV	C	G	G	PAX3	c.333G>C	P.T111T
SH_90	2	232389978	232389978	Missense_Mutation	SNV	G	T	T	NMUR1	c.1057C>A	P.P353T
SH_90	2	234967610	234967610	Splice_Site	SNV	G	C	C	SPP2	c.333+8G>C	
SH_90	2	240036802	240036802	Nonsense_Mutation	SNV	C	A	A	HDAC4	c.1723G>T	P.E575*
SH_90	2	24524114	24524114	Splice_Site	SNV	A	C	C	ITSN2	c.996-6T>G	
SH_90	2	26677581	26677581	Nonsense_Mutation	SNV	G	A	A	DRC1	c.1986G>A	P.W662*
SH_90	2	37516651	37516651	Missense_Mutation	SNV	C	T	T	PRKD3	c.565G>A	P.G189R
SH_90	2	43015711	43015711	Silent	SNV	G	T	T	HAAO	c.117C>A	P.G39G
SH_90	2	55155791	55155791	Missense_Mutation	SNV	G	T	T	EML6	c.3907G>T	P.A1303S
SH_90	2	71304731	71304731	Missense_Mutation	SNV	G	T	T	NAGK	c.947G>T	P.G316V
SH_90	2	71375187	71375187	Frame_Shift_Del	INDEL	C	-	-	MPHOSPH10	c.1619delC	P.P540fs
SH_90	2	74783894	74783894	Missense_Mutation	SNV	G	T	T	DOK1	c.682G>T	P.G228C
SH_90	2	86297387	86297387	Silent	SNV	C	A	A	POLR1A	c.1620G>T	P.R540R
SH_90	2	96795692	96795692	Missense_Mutation	SNV	G	T	T	ASTL	c.745C>A	P.P249T
SH_90	20	30584986	30584986	Missense_Mutation	SNV	C	A	A	XKR7	c.1466C>A	P.A489E
SH_90	20	33501306	33501306	Splice_Site	SNV	G	T	T	ACSS2	c.570+7G>T	
SH_90	20	33711738	33711738	Nonsense_Mutation	SNV	G	A	A	EDEM2	c.958C>T	P.Q320*
SH_90	20	33868547	33868547	Missense_Mutation	SNV	C	A	A	EIF6	c.279G>T	P.Q93H
SH_90	20	35443597	35443597	Missense_Mutation	SNV	G	A	A	SOGA1	c.2248C>T	P.P750S
SH_90	20	40727180	40727180	Missense_Mutation	SNV	G	T	T	PTPRT	c.3784C>A	P.P1262T
SH_90	20	50140058	50140058	Missense_Mutation	SNV	G	A	A	NFATC2	c.662C>T	P.P221L
SH_90	20	57268916	57268916	Missense_Mutation	SNV	G	T	T	NPEPL1	c.190G>T	P.A64S
SH_90	20	57766452	57766452	Silent	SNV	G	T	T	ZNF831	c.378G>T	P.P126P
SH_90	20	61477089	61477089	Missense_Mutation	SNV	C	T	T	DPH3P1	c.74C>T	P.P25L
SH_90	20	62679615	62679615	Silent	SNV	G	T	T	SOX18	c.1059C>A	P.G353G
SH_90	21	31661769	31661769	Missense_Mutation	SNV	G	T	T	KRTAP25-1	c.40C>A	P.H14N
SH_90	21	31869280	31869280	Missense_Mutation	SNV	C	A	A	KRTAP19-4	c.149G>T	P.R50L
SH_90	21	33716979	33716979	Missense_Mutation	SNV	C	T	T	URB1	c.4157G>A	P.C1386Y
SH_90	21	47954002	47954002	Silent	SNV	A	G	G	DIP2A	c.1386A>G	P.T462T
SH_90	22	18379039	18379039	Missense_Mutation	SNV	G	C	C	MICAL3	c.1295C>G	P.T432R
SH_90	22	25425493	25425493	Silent	SNV	G	T	T	KIAA1671	c.1527G>T	P.A509A
SH_90	22	32200902	32200902	Splice_Site	SNV	G	A	A	DEPDC5	c.1217+1G>A	
SH_90	22	44602285	44602285	Silent	SNV	G	T	T	PARVG	c.975G>T	P.T325T
SH_90	3	113380252	113380252	Missense_Mutation	SNV	G	A	A	USF3	c.277C>T	P.R93W
SH_90	3	128853696	128853696	Missense_Mutation	SNV	C	G	G	ISY1	c.586G>C	P.E196Q
SH_90	3	13670711	13670711	Missense_Mutation	SNV	G	A	A	FBLN2	c.2761G>A	P.V921M
SH_90	3	138474692	138474692	Missense_Mutation	SNV	C	A	A	PIK3CB	c.301G>T	P.D101Y
SH_90	3	182615091	182615091	Missense_Mutation	SNV	A	G	G	ATP11B	c.3049A>G	P.M1017V
SH_90	3	184700404	184700404	Silent	SNV	C	T	T	VPS8	c.3471C>T	P.S1157S
SH_90	3	194081566	194081566	Missense_Mutation	SNV	A	T	T	LRRC15	c.225T>A	P.N75K
SH_90	3	35750466	35750466	Missense_Mutation	SNV	C	G	G	ARPP21	c.801C>G	P.N267K
SH_90	4	113435851	113435851	Missense_Mutation	SNV	A	T	T	NEUROG2	c.781T>A	P.Y261N
SH_90	4	138452122	138452122	Missense_Mutation	SNV	G	A	A	PCDH18	c.1121C>T	P.P374L
SH_90	4	38022260	38022260	Missense_Mutation	SNV	G	A	A	TBC1D1	c.1021G>A	P.G341R
SH_90	4	78815299	78815299	Missense_Mutation	SNV	G	T	T	MRPL1	c.565G>T	P.D189Y
SH_90	4	78987134	78987134	Splice_Site	SNV	C	A	A	FRAS1	c.77-5C>A	
SH_90	4	8293228	8293228	Silent	SNV	G	A	A	HTRA3	c.840G>A	P.E280E
SH_90	4	8584268	8584268	Missense_Mutation	SNV	C	A	A	GPR78	c.679C>A	P.R227S
SH_90	4	89649749	89649749	Missense_Mutation	SNV	T	G	G	FAM13A	c.2029A>C	P.K677Q

SH_90	5	121758812	121758812	Missense_Mutation	SNV	G	A	A	SNCAIP	c.380G>A	P.G127D
SH_90	5	138456824	138456824	Silent	SNV	G	T	T	SIL1	c.144C>A	P.T48T
SH_90	5	140264201	140264201	Missense_Mutation	SNV	C	A	A	PCDHA13	c.2348C>A	P.A783E
SH_90	5	140625227	140625227	Silent	SNV	C	A	A	PCDHB15	c.81C>A	P.G27G
SH_90	5	140720777	140720777	Missense_Mutation	SNV	C	T	T	PCDHGA2	c.2239C>T	P.R747W
SH_90	5	146798103	146798103	Missense_Mutation	SNV	T	C	C	DPYSL3	c.562A>G	P.T188A
SH_90	5	160071232	160071232	Missense_Mutation	SNV	T	C	C	ATP10B	c.781A>G	P.R261G
SH_90	5	40853925	40853925	Nonsense_Mutation	SNV	G	T	T	CARD6	c.2491G>T	P.E831*
SH_90	5	55081594	55081594	Missense_Mutation	SNV	G	T	T	DDX4	c.657G>T	P.E219D
SH_90	5	64558701	64558701	Missense_Mutation	SNV	C	A	A	ADAMTS6	c.1709G>T	P.C570F
SH_90	6	106555106	106555106	Silent	SNV	G	T	T	PRDM1	c.2223G>T	P.R741R
SH_90	6	144508366	144508366	Missense_Mutation	SNV	G	T	T	STX11	c.602G>T	P.R201L
SH_90	6	149997839	149997839	Missense_Mutation	SNV	A	T	T	LATS1	c.2628T>A	P.S876R
SH_90	6	159618553	159618553	Missense_Mutation	SNV	G	T	T	FNDC1	c.200G>T	P.S67I
SH_90	6	168352294	168352294	Frame_Shift_Ins	INDEL	-	G	G	AFDN	c.4240dupG	P.E1414fs
SH_90	6	26022174	26022174	Missense_Mutation	SNV	G	T	T	HIST1H4A	c.268G>T	P.A90S
SH_90	6	26217347	26217347	Missense_Mutation	SNV	C	G	G	HIST1H2AE	c.145C>G	P.P49A
SH_90	6	28327532	28327532	Missense_Mutation	SNV	G	T	T	ZKSCAN3	c.169G>T	P.A57S
SH_90	6	29454993	29454993	Silent	SNV	C	A	A	MAS1L	c.687G>T	P.G229G
SH_90	6	33635036	33635036	Missense_Mutation	SNV	G	C	C	ITPR3	c.1682G>C	P.R561P
SH_90	6	50683324	50683324	Missense_Mutation	SNV	C	G	G	TFAP2D	c.535C>G	P.Q179E
SH_90	6	71567899	71567899	Silent	SNV	G	T	T	SMAP1	c.1236G>T	P.P412P
SH_90	6	83767661	83767661	Missense_Mutation	SNV	G	A	A	UBE3D	c.62C>T	P.T21I
SH_90	6	86282021	86282031	Frame_Shift_Del	INDEL	TCGTTTACAT	-	-	SNX14	333delAATGTA/	P.K108fs
SH_90	6	90581050	90581050	Silent	SNV	G	T	T	CASP8AP2	c.5835G>T	P.G1945G
SH_90	7	111368600	111368600	Silent	SNV	C	A	A	DOCK4	c.5631G>T	P.P1877P
SH_90	7	128454716	128454716	Silent	SNV	C	A	A	CCDC136	c.2788C>A	P.R930R
SH_90	7	138711555	138711555	Missense_Mutation	SNV	T	C	C	ZC3HAV1L	c.785A>G	P.H262R
SH_90	7	142658054	142658054	Missense_Mutation	SNV	G	T	T	KEL	c.361C>A	P.L121I
SH_90	7	149430774	149430774	Missense_Mutation	SNV	G	T	T	KRBA1	c.2830G>T	P.A944S
SH_90	7	149511875	149511875	Nonsense_Mutation	SNV	G	A	A	SSPO	c.10425G>A	P.W3475*
SH_90	7	150269312	150269312	Missense_Mutation	SNV	G	T	T	GIMAP4	c.154G>T	P.G52C
SH_90	7	150648887	150648887	Missense_Mutation	SNV	G	T	T	KCNH2	c.1594C>A	P.L532M
SH_90	7	21599350	21599350	Silent	SNV	T	C	C	DNAH11	c.822T>C	P.P274P
SH_90	7	21789410	21789410	Silent	SNV	C	T	T	DNAH11	c.8788C>T	P.L2930L
SH_90	7	32535362	32535362	Missense_Mutation	SNV	G	T	T	AVL9	c.41G>T	P.G14V
SH_90	7	47920360	47920360	Missense_Mutation	SNV	G	T	T	PKD1L1	c.3486C>A	P.S1162R
SH_90	7	47944053	47944053	Missense_Mutation	SNV	G	T	T	PKD1L1	c.1853C>A	P.T618K
SH_90	7	6731631	6731631	Silent	SNV	C	G	G	ZNF12	c.828G>C	P.V276V
SH_90	7	82595337	82595337	Missense_Mutation	SNV	G	T	T	PCLO	c.3767C>A	P.A1256D
SH_90	8	10468264	10468264	Missense_Mutation	SNV	G	A	A	RP1L1	c.3344C>T	P.A1115V
SH_90	8	119936964	119936964	Silent	SNV	A	G	G	TNFRSF11B	c.855T>C	P.I285I
SH_90	8	135600604	135600604	Silent	SNV	C	A	A	ZFAT	c.2562G>T	P.G854G
SH_90	8	142488730	142488730	Missense_Mutation	SNV	A	G	G	MROH5	c.1231T>C	P.Y411H
SH_90	8	144942421	144942421	Missense_Mutation	SNV	C	T	T	EPPK1	c.5001G>A	P.M1667I
SH_90	8	56015729	56015729	Silent	SNV	C	A	A	XKR4	c.681C>A	P.A227A
SH_90	8	85441565	85441565	Silent	SNV	C	T	T	RALYL	c.48C>T	P.G16G
SH_90	8	87683304	87683304	Missense_Mutation	SNV	C	A	A	CNGB3	c.361G>T	P.A121S
SH_90	8	93029523	93029523	Missense_Mutation	SNV	G	A	A	RUNX1T1	c.157C>T	P.P53S
SH_90	8	97157059	97157059	Missense_Mutation	SNV	C	A	A	GDF6	c.1100G>T	P.G367V
SH_90	9	109701346	109701346	Missense_Mutation	SNV	T	G	G	ZNF462	c.6385T>G	P.S2129A
SH_90	9	109701347	109701347	Missense_Mutation	SNV	C	A	A	ZNF462	c.6386C>A	P.S2129Y
SH_90	9	127765388	127765388	Splice_Site	SNV	C	T	T	SCAI	c.1065+5G>A	
SH_90	9	130272502	130272502	Silent	SNV	G	T	T	FAM129B	c.1045C>A	P.R349R
SH_90	9	138439749	138439749	Missense_Mutation	SNV	C	T	T	OBP2A	c.176C>T	P.A59V
SH_90	9	138651570	138651577	Frame_Shift_Del	INDEL	CCTCCTGA	-	-	KCNT1	1_778delGACCT	P.T258fs
SH_90	9	140919585	140919585	Missense_Mutation	SNV	A	G	G	CACNA1B	c.3247A>G	P.M1083V
SH_90	9	36882080	36882080	Silent	SNV	G	T	T	PAX5	c.933C>A	P.T311T
SH_90	9	71628567	71628567	Missense_Mutation	SNV	C	A	A	PRKACG	c.442G>T	P.A148S
SH_90	9	88650301	88650301	Nonsense_Mutation	SNV	G	A	A	GOLM1	c.997C>T	P.Q333*
SH_90	9	92220635	92220635	Missense_Mutation	SNV	G	T	T	GADD45G	c.209G>T	P.G70V
SH_90	X	106844339	106844339	Missense_Mutation	SNV	C	A	A	FRMPD3	c.3169C>A	P.H1057N
SH_90	X	110463640	110463640	Missense_Mutation	SNV	G	A	A	PAK3	c.1600G>A	P.A534T
SH_90	X	130220331	130220331	Missense_Mutation	SNV	C	T	T	ARHGAP36	c.1274C>T	P.A425V
SH_90	X	130408836	130408836	Missense_Mutation	SNV	G	T	T	IGSF1	c.3503C>A	P.P1168H
SH_90	X	24382330	24382330	Missense_Mutation	SNV	G	T	T	SUPT20HL1	c.1453G>T	P.A485S
SH_90	X	3241813	3241813	Missense_Mutation	SNV	A	G	G	MXRA5	c.1913T>C	P.I638T
SH_90	X	51150018	51150018	Silent	SNV	C	T	T	CXorf67	c.150C>T	P.S50S
SH_90	X	73961449	73961449	Silent	SNV	C	T	T	KIAA2022	c.2943G>A	P.G981G
SH_91	1	119466161	119466161	Silent	SNV	A	G	G	TBX15	c.441T>C	P.S147S
SH_91	1	204168364	204168364	Missense_Mutation	SNV	C	T	T	GOLT1A	c.319G>A	P.G107S
SH_91	1	3753967	3753967	Silent	SNV	A	G	G	CEP104	c.1008T>C	P.F336F
SH_91	11	6453208	6453208	Nonsense_Mutation	SNV	C	T	T	HPX	c.875G>A	P.W292*
SH_91	11	71259797	71259797	Missense_Mutation	SNV	G	A	A	KRTAP5-9	c.94G>A	P.G32S
SH_91	12	72388266	72388266	Missense_Mutation	SNV	C	T	T	TPH2	c.989C>T	P.P330L
SH_91	17	63193302	63193302	Missense_Mutation	SNV	A	G	G	RGS9	c.910A>G	P.I304V
SH_91	19	54135383	54135383	Missense_Mutation	SNV	G	A	A	DPRX	c.23G>A	P.R8H
SH_91	19	58757694	58757694	Missense_Mutation	SNV	A	G	G	ZNF544	c.61A>G	P.M21V
SH_91	2	153435446	153435446	Missense_Mutation	SNV	G	T	T	FMNL2	c.750G>T	P.E250D
SH_91	2	209113113	209113113	Missense_Mutation	SNV	G	A	A	IDH1	c.394C>T	P.R132C
SH_91	X	106356707	106356707	Splice_Site	SNV	G	A	A	RBM41	n.672-9C>T	
SH_91	X	120182341	120182341	Missense_Mutation	SNV	G	A	A	GLUD2	c.803G>A	P.R268H
SH_94	1	152732155	152732155	Missense_Mutation	SNV	G	A	A	KPRP	c.91G>A	P.A31T
SH_94	1	156909500	156909500	Silent	SNV	C	A	A	ARHGEF11	c.3816G>T	P.G1272G
SH_94	1	158533025	158533025	Silent	SNV	G	A	A	OR6P1	c.370C>T	P.L124L
SH_94	1	20097963	20097963	Silent	SNV	T	A	A	TMCO4	c.192A>T	P.T64T
SH_94	1	222825366	222825366	Silent	SNV	A	C	C	MIA3	c.591A>C	P.S197S
SH_94	1	32936476	32936476	Missense_Mutation	SNV	A	T	T	ZBTB8B	c.251A>T	P.Y84F
SH_94	1	39768645	39768645	Missense_Mutation	SNV	G	A	A	MACF1	c.2699G>A	P.R900Q
SH_94	1	72241959	72241959	Missense_Mutation	SNV	A	T	T	NEGR1	c.431T>A	P.I144N
SH_94	1	94654872	94654872	Silent	SNV	T	C	C	ARHGAP29	c.1476A>G	P.S492S
SH_94	10	135345210	135345210	Frame_Shift_Del	INDEL	C	-	-	CYP2E1	c.460delC	P.L154fs
SH_94	10	23319572	23319572	Missense_Mutation	SNV	A	T	T	ARMC3	c.2072A>T	P.E691V
SH_94	10	75434614	75434614	Missense_Mutation	SNV	T	A	A	AGAP5	c.1804A>T	P.T602S
SH_94	10	89690843	89690843	Nonsense_Mutation	SNV	A	T	T	PTEN	c.250A>T	P.R84*
SH_94	11	118959406	118959406	Missense_Mutation	SNV	A	C	C	HMBS	c.149A>C	P.Q50P
SH_94	11	62367715	62367715	Missense_Mutation	SNV	A	G	G	MTA2	c.113T>C	P.V38A
SH_94	11	94353020	94353020	Missense_Mutation	SNV	G	T	T	PIWIL4	c.2263G>T	P.V755F

SH_94	12	72956755	72956755	Silent	SNV	T	A	A	TRHDE	c.1842T>A	P.I614I
SH_94	12	99640197	99640197	Missense_Mutation	SNV	A	T	T	ANKS1B	c.2202T>A	P.S734R
SH_94	13	103392851	103392851	Missense_Mutation	SNV	T	G	G	CCDC168	c.10196A>C	P.Y3399S
SH_94	13	49039389	49039389	Frame_Shift_Del	INDEL	T	-	-	RB1	c.2376delT	P.P793fs
SH_94	15	45814375	45814375	Missense_Mutation	SNV	T	C	C	SLC30A4	c.178A>G	P.R60G
SH_94	15	49254658	49254658	Silent	SNV	C	A	A	SHC4	c.555G>T	P.L185L
SH_94	15	56132699	56132699	Silent	SNV	T	C	C	NEDD4	c.3234A>G	P.P1078P
SH_94	15	70961218	70961218	Missense_Mutation	SNV	C	T	T	UACA	c.1766G>A	P.R589Q
SH_94	16	1449434	1449434	Silent	SNV	C	A	A	UNKL	c.675G>T	P.A225A
SH_94	16	2812720	2812720	Frame_Shift_Del	INDEL	A	-	-	SRRM2	c.2195delA	P.N732fs
SH_94	16	89967103	89967103	Silent	SNV	C	T	T	TCF25	c.1282C>T	P.L428L
SH_94	17	20135522	20135522	Missense_Mutation	SNV	G	A	A	SPECC1	c.2155G>A	P.E719K
SH_94	17	27903575	27903575	Missense_Mutation	SNV	T	C	C	GIT1	c.1372A>G	P.S458G
SH_94	17	33520403	33520403	Silent	SNV	A	T	T	SLC35G3	c.924T>A	P.S308S
SH_94	17	68171535	68171535	Missense_Mutation	SNV	G	A	A	KCNJ2	c.355G>A	P.G119S
SH_94	17	7578168	7578190	Frame_Shift_Del	INDEL	GACCTCAGGC	-	-	TP53	ATGAGCCGCCT	P.Y220fs
SH_94	18	19088328	19088328	Splice_Site	INDEL	-	T	T	GREB1L	c.4531-13dupT	
SH_94	19	19136593	19136593	Missense_Mutation	SNV	A	C	C	SUGP2	c.564T>G	P.S188R
SH_94	19	22498378	22498378	Missense_Mutation	SNV	C	G	G	ZNF729	c.2159C>G	P.A720G
SH_94	19	44237030	44237030	Silent	SNV	C	A	A	SMG9	c.1464G>T	P.T488T
SH_94	2	103335600	103335600	Missense_Mutation	SNV	C	T	T	MFSD9	c.521G>A	P.R174Q
SH_94	2	15307245	15307247	Missense_Mutation	INDEL	AGA	-	-	NBAS	7041_7043delT	P.L2348del
SH_94	2	179638773	179638773	Missense_Mutation	SNV	C	A	A	TTN	c.7122G>T	P.Q2374H
SH_94	2	18745093	18745093	Missense_Mutation	SNV	G	T	T	NT5C1B	c.1802C>A	P.A601D
SH_94	2	80831222	80831222	Missense_Mutation	SNV	C	A	A	CTNNA2	c.2213C>A	P.T738K
SH_94	20	1532412	1532412	Missense_Mutation	SNV	A	T	T	SIRPD	c.346T>A	P.Y116N
SH_94	20	30418646	30418646	Missense_Mutation	SNV	A	C	C	MYLK2	c.1249A>C	P.T417P
SH_94	20	49552780	49552780	Missense_Mutation	SNV	G	T	T	DPM1	c.688C>A	P.L230I
SH_94	21	30909664	30909664	Missense_Mutation	SNV	G	T	T	GRIK1	c.2233C>A	P.L745M
SH_94	22	17128681	17128681	Splice_Site	SNV	T	A	A	TPTEP1	n.1126+6T>A	
SH_94	22	18185059	18185059	Missense_Mutation	SNV	A	T	T	BCL2L13	c.579A>T	P.R193S
SH_94	22	30682080	30682080	Missense_Mutation	SNV	T	A	A	GATSL3	c.751A>T	P.S251C
SH_94	3	137483868	137483868	Missense_Mutation	SNV	G	T	T	SOX14	c.242G>T	P.R81L
SH_94	3	149459450	149459450	Missense_Mutation	SNV	T	A	A	COMMD2	c.458A>T	P.H153L
SH_94	3	179050845	179050845	Nonsense_Mutation	SNV	A	T	T	ZNF639	c.238A>T	P.R80*
SH_94	3	196730771	196730771	Splice_Site	SNV	C	T	T	MELTF	c.2137+1G>A	
SH_94	3	2787274	2787274	Nonsense_Mutation	SNV	T	A	A	CNTN4	c.251T>A	P.L84*
SH_94	3	38781183	38781183	Splice_Site	SNV	G	A	A	SCN10A	c.2107-4C>T	
SH_94	3	45869950	45869950	Missense_Mutation	SNV	T	A	A	LZTFL1	c.707A>T	P.Q236L
SH_94	3	48728864	48728864	Nonsense_Mutation	SNV	G	C	C	IP6K2	c.480C>G	P.Y160*
SH_94	3	54919573	54919573	Silent	SNV	G	T	T	CACNA2D3	c.2016G>T	P.A672A
SH_94	4	122825507	122825507	Silent	SNV	A	T	T	TRPC3	c.2223T>A	P.A741A
SH_94	4	40356119	40356119	Missense_Mutation	SNV	T	A	A	CHRNA9	c.1022T>A	P.I341N
SH_94	4	46930264	46930264	Missense_Mutation	SNV	T	G	G	GABRA4	c.1643A>C	P.E548A
SH_94	4	55964424	55964424	Missense_Mutation	SNV	G	T	T	KDR	c.2389C>A	P.L797M
SH_94	4	74283828	74283828	Silent	SNV	A	G	G	ALB	c.1452A>G	P.L484L
SH_94	4	81967509	81967509	Missense_Mutation	SNV	G	T	T	BMP3	c.934G>T	P.D312Y
SH_94	5	137496591	137496591	Nonsense_Mutation	SNV	C	A	A	BRD8	c.2425G>T	P.E809*
SH_94	5	140480240	140480240	Missense_Mutation	SNV	G	T	T	PCDHB3	c.7G>T	P.A3S
SH_94	5	169693807	169693807	Splice_Site	SNV	C	A	A	LCP2	c.772+5G>T	
SH_94	5	169697839	169697839	Missense_Mutation	SNV	T	A	A	LCP2	c.407A>T	P.E136V
SH_94	5	38949465	38949465	Frame_Shift_Del	INDEL	G	-	-	RICTOR	c.4201delC	P.L1401fs
SH_94	5	70806005	70806005	Missense_Mutation	SNV	T	G	G	BDP1	c.3086T>G	P.I1029S
SH_94	5	74101066	74101066	Silent	SNV	G	T	T	FAM169A	c.714C>A	P.L238L
SH_94	6	43488766	43488766	Missense_Mutation	SNV	G	T	T	POLR1C	c.902G>T	P.R301L
SH_94	6	57498995	57498995	Missense_Mutation	SNV	A	T	T	PRIM2	c.1259A>T	P.Y420F
SH_94	7	126173556	126173556	Missense_Mutation	SNV	G	T	T	GRM8	c.1880C>A	P.T627K
SH_94	7	143097027	143097027	Silent	SNV	A	G	G	EPHA1	c.552T>C	P.A184A
SH_94	7	36336642	36336642	Silent	SNV	G	T	T	EEPD1	c.1356G>T	P.G452G
SH_94	7	63796736	63796736	Missense_Mutation	SNV	G	A	A	ZNF736	c.103G>A	P.E35K
SH_94	7	82451998	82451998	Silent	SNV	C	T	T	PCLO	c.14604G>A	P.Q4868Q
SH_94	7	99017783	99017783	Splice_Site	SNV	A	G	G	ATP5J2-PTCD1	c.2068-11T>C	
SH_94	8	113516187	113516187	Missense_Mutation	SNV	A	T	T	CSMD3	c.4603T>A	P.Y1535N
SH_94	8	128752857	128752857	Missense_Mutation	SNV	G	C	C	MYC	c.1018G>C	P.V340L
SH_94	8	73993274	73993274	Missense_Mutation	SNV	T	A	A	SBSPON	c.389A>T	P.Q130L
SH_94	9	107595019	107595019	Frame_Shift_Del	INDEL	A	-	-	ABCA1	c.1345delT	P.W449fs
SH_94	9	135777083	135777083	Nonsense_Mutation	SNV	T	A	A	TSC1	c.2395A>T	P.K799*
SH_94	9	14759790	14759790	Missense_Mutation	SNV	A	T	T	FREM1	c.922T>A	P.S308T
SH_94	9	16436624	16436624	Missense_Mutation	SNV	G	T	T	BNC2	c.1442C>A	P.A481E
SH_94	9	17786417	17786417	Nonsense_Mutation	SNV	A	T	T	SH3GL2	c.226A>T	P.K76*
SH_94	9	96054925	96054925	Missense_Mutation	SNV	G	T	T	WNK2	c.5289G>T	P.L1763F
SH_94	X	77284795	77284795	Missense_Mutation	SNV	T	A	A	ATP7A	c.2965T>A	P.F989I
SH_94	X	77284796	77284796	Missense_Mutation	SNV	T	G	G	ATP7A	c.2966T>G	P.F989C
SH_94	X	78618526	78618526	Silent	SNV	A	T	T	ITM2A	c.222T>A	P.R74R
SH_95	1	149783852	149783852	Silent	SNV	A	G	G	HIST2H2BF	c.27T>C	P.P9P
SH_95	1	159410218	159410218	Missense_Mutation	SNV	G	T	T	OR10J1	c.670G>T	P.V224F
SH_95	1	214814366	214814366	Silent	SNV	C	T	T	CENPF	c.2685C>T	P.H895H
SH_95	1	55224012	55224012	Missense_Mutation	SNV	G	A	A	PARS2	c.823C>T	P.R275C
SH_95	11	67789219	67789219	Splice_Site	SNV	C	T	T	ALDH3B1	c.478-4C>T	
SH_95	12	85264405	85264405	Silent	SNV	C	T	T	SLC6A15	c.1026G>A	P.A342A
SH_95	13	21557710	21557710	Missense_Mutation	SNV	T	A	A	LATS2	c.2135A>T	P.H712L
SH_95	15	58465397	58465397	Missense_Mutation	SNV	T	G	G	AQP9	c.369T>G	P.I123M
SH_95	15	83481947	83481947	Silent	SNV	G	A	A	WHAMM	c.702G>A	P.V234V
SH_95	17	11659887	11659887	Splice_Site	SNV	G	T	T	DNAH9	c.6741G>T	P.V2247V
SH_95	17	18233896	18233896	Missense_Mutation	SNV	T	C	C	SHMT1	c.730A>G	P.I244V
SH_95	17	5072078	5072078	Missense_Mutation	SNV	G	A	A	USP6	c.3245G>A	P.R1082Q
SH_95	17	61902624	61902633	Frame_Shift_Del	INDEL	TGCAGATTCA	-	-	FTSJ3	_573delTGAATC	P.H188fs
SH_95	17	7576927	7576927	Splice_Site	SNV	C	A	A	TP53	c.920-1G>T	
SH_95	17	7578419	7578419	Nonsense_Mutation	SNV	C	A	A	TP53	c.511G>T	P.E171*
SH_95	19	42854300	42854300	Missense_Mutation	SNV	G	A	A	MEGF8	c.2500G>A	P.E834K
SH_95	19	43373002	43373002	Silent	SNV	G	T	T	PSG1	c.894C>A	P.P298P
SH_95	19	436191	436191	Silent	SNV	C	A	A	SHC2	c.927G>T	P.P309P
SH_95	19	43773563	43773563	Silent	SNV	A	G	G	PSG9	c.21T>C	P.P7P
SH_95	2	20990038	20990038	Missense_Mutation	SNV	T	A	A	LDAH	c.275A>T	P.K92M
SH_95	2	99232702	99232702	Missense_Mutation	SNV	A	G	G	UNC50	c.434A>G	P.Q145R
SH_95	20	20071526	20071526	Missense_Mutation	SNV	G	T	T	CFAP61	c.605G>T	P.R202L
SH_95	20	23016479	23016479	Missense_Mutation	SNV	G	T	T	SSTR4	c.359G>T	P.R120L
SH_95	20	23584331	23584331	Missense_Mutation	SNV	C	T	T	CST9	c.296G>A	P.R99H

SH_95	3	112648154	112648154	Missense_Mutation	SNV	C	A	A	CD200R1	c.403G>T	P.D135Y
SH_95	3	142119349	142119349	Missense_Mutation	SNV	A	T	T	XRN1	c.2144T>A	P.V715D
SH_95	3	158317966	158317966	Splice_Site	SNV	A	T	T	MLF1	c.493+4A>T	
SH_95	3	36931370	36931370	Missense_Mutation	SNV	C	T	T	TRANK1	c.857G>A	P.C286Y
SH_95	3	39436033	39436033	Missense_Mutation	SNV	A	T	T	SLC25A38	c.758A>T	P.Q253L
SH_95	3	71101704	71101704	Missense_Mutation	SNV	C	A	A	FOXP1	c.494G>T	P.G165V
SH_95	5	140209436	140209436	Missense_Mutation	SNV	C	T	T	PCDHA6	c.1760C>T	P.A587V
SH_95	5	90079728	90079728	Missense_Mutation	SNV	A	G	G	ADGRV1	c.13507A>G	P.S4503G
SH_95	6	159653744	159653744	Missense_Mutation	SNV	C	T	T	FNDC1	c.2200C>T	P.P734S
SH_95	6	53761699	53761699	Splice_Site	SNV	G	T	T	LRRC1	c.567+9G>T	
SH_95	6	78172786	78172786	Missense_Mutation	SNV	G	A	A	HTR1B	c.335C>T	P.T112I
SH_95	7	151945330	151945330	Missense_Mutation	SNV	G	T	T	KMT2C	c.2189C>A	P.S730Y
SH_95	7	91503086	91503086	Missense_Mutation	SNV	C	T	T	MTERF1	c.962G>A	P.S321N
SH_95	8	11189000	11189000	Frame_Shift_Del	INDEL	A	-	-	SLC35G5	c.386delA	P.N129fs
SH_95	8	120431397	120431397	Missense_Mutation	SNV	G	A	A	NOV	c.589G>A	P.V197I
SH_95	8	144896218	144896218	Missense_Mutation	SNV	T	A	A	SCRIB	c.230A>T	P.E77V
SH_95	8	73117533	73117533	Splice_Site	SNV	C	G	G	LOC392232	n.2513+1G>C	
SH_95	9	19276317	19276317	Missense_Mutation	SNV	A	G	G	DENND4C	c.145A>G	P.I49V
SH_95	X	49033526	49033526	Silent	SNV	G	A	A	PRICKLE3	c.777C>T	P.Y259Y
SH_98	1	145650534	145650534	Silent	SNV	T	C	C	RNF115	c.213T>C	P.F71F
SH_98	1	171752872	171752872	Splice_Site	SNV	T	G	G	METTL13	c.154-8T>G	
SH_98	10	105889958	105889958	Missense_Mutation	SNV	A	G	G	CFAP43	c.4937T>C	P.I1646T
SH_98	10	128147638	128147638	Missense_Mutation	SNV	T	C	C	C10orf90	c.1868A>G	P.K623R
SH_98	12	11174371	11174371	Missense_Mutation	SNV	A	G	G	TAS2R19	c.800T>C	P.V267A
SH_98	12	126068387	126068387	Splice_Site	SNV	T	C	C	TMEM132B	c.1279-10T>C	
SH_98	13	20637113	20637113	Splice_Site	SNV	T	C	C	ZMYM2	c.3037+2T>C	
SH_98	15	81221384	81221384	Silent	SNV	C	T	T	CEMIP	c.2481C>T	P.D827D
SH_98	16	19083402	19083402	Silent	SNV	C	A	A	COQ7	c.112C>A	P.R38R
SH_98	16	30745237	30745237	Nonsense_Mutation	SNV	G	T	T	SRCAP	c.6517G>T	P.E2173*
SH_98	17	26686410	26686410	Missense_Mutation	SNV	C	T	T	TMEM199	c.358C>T	P.R120C
SH_98	17	26902473	26902473	Silent	SNV	C	T	T	ALDOC	c.78G>A	P.P26P
SH_98	17	33690347	33690347	Silent	SNV	G	A	A	SLFN11	c.480C>T	P.I160I
SH_98	17	35549227	35549227	Missense_Mutation	SNV	T	C	C	ACACA	c.4220A>G	P.Y1407C
SH_98	17	36625495	36625495	Missense_Mutation	SNV	C	A	A	ARHGAP23	c.1759C>A	P.P587T
SH_98	17	39274122	39274122	Missense_Mutation	SNV	C	G	G	KRTAP4-11	c.446G>C	P.C149S
SH_98	17	66246484	66246484	Silent	SNV	C	T	T	AMZ2	c.156C>T	P.T52T
SH_98	17	79478964	79478964	Frame_Shift_Del	INDEL	G	-	-	ACTG1	c.328delC	P.L110fs
SH_98	3	112648004	112648004	Missense_Mutation	SNV	A	T	T	CD200R1	c.553T>A	P.S185T
SH_98	5	130517915	130517915	Splice_Site	SNV	T	A	A	LYRM7	c.92-7T>A	
SH_98	5	140762685	140762685	Missense_Mutation	SNV	G	T	T	PCDHGA7	c.219G>T	P.Q73H
SH_98	6	32411602	32411602	Missense_Mutation	SNV	G	C	C	HLA-DRA	c.680G>C	P.G227A
SH_98	7	158705673	158705673	Splice_Site	SNV	A	G	G	WDR60	c.1591-3A>G	
SH_98	7	47344561	47344561	Silent	SNV	C	A	A	TNS3	c.2691G>T	P.G897G
SH_98	7	90225967	90225967	Missense_Mutation	SNV	C	A	A	CDK14	c.26C>A	P.P9Q
SH_98	8	101721692	101721692	Missense_Mutation	SNV	G	A	A	PABPC1	c.1240C>T	P.P414S
SH_98	8	17089978	17089978	Silent	SNV	A	C	C	CNOT7	c.687T>G	P.S229S
SH_98	8	95508590	95508590	Missense_Mutation	SNV	T	C	C	KIAA1429	c.4349A>G	P.E1450G
SH_98	9	107367427	107367427	Missense_Mutation	SNV	A	C	C	OR13C2	c.482T>G	P.V161G
SH_98	X	117677486	117677486	Missense_Mutation	SNV	T	C	C	DOCK11	c.322T>C	P.Y108H
SH_98	X	5810898	5810898	Missense_Mutation	SNV	C	G	G	NLGN4X	c.2411G>C	P.S804T
SH_99	1	117509574	117509574	Missense_Mutation	SNV	T	C	C	PTGFRN	c.1681T>C	P.F561L
SH_99	1	149291202	149291202	Splice_Site	SNV	A	T	T	FAM231D	n.1447-2A>T	
SH_99	1	151263518	151263518	Missense_Mutation	SNV	A	T	T	ZNF687	c.3547A>T	P.R1183W
SH_99	1	159828021	159828021	Missense_Mutation	SNV	C	T	T	VSIG8	c.289G>A	P.V97I
SH_99	1	166927098	166927098	Missense_Mutation	SNV	C	T	T	ILDR2	c.287G>A	P.S96N
SH_99	1	183109595	183109595	Silent	SNV	T	A	A	LAMC1	c.4530T>A	P.V1510V
SH_99	1	203466135	203466135	Missense_Mutation	SNV	A	T	T	OPTC	c.262A>T	P.S88C
SH_99	1	206648264	206648264	Silent	SNV	G	T	T	IKBKE	c.285G>T	P.L95L
SH_99	1	22150826	22150826	Missense_Mutation	SNV	C	T	T	HSPG2	c.12791G>A	P.G4264E
SH_99	1	22927164	22927164	Missense_Mutation	SNV	T	A	A	EPHA8	c.2399T>A	P.I800N
SH_99	1	229568405	229568405	Missense_Mutation	SNV	G	T	T	ACTA1	c.352C>A	P.R118S
SH_99	1	245862253	245862253	Missense_Mutation	SNV	T	A	A	KIF26B	c.6092T>A	P.V2031D
SH_99	1	245927411	245927411	Missense_Mutation	SNV	C	T	T	SMYD3	c.1117G>A	P.V373M
SH_99	1	247319994	247319994	Missense_Mutation	SNV	C	G	G	ZNF124	c.744G>C	P.R248S
SH_99	1	248004936	248004936	Missense_Mutation	SNV	C	A	A	OR11L1	c.263G>T	P.W88L
SH_99	1	248309355	248309355	Missense_Mutation	SNV	G	T	T	OR2M5	c.906G>T	P.R302S
SH_99	1	40097227	40097227	Missense_Mutation	SNV	G	A	A	HEYL	c.172C>T	P.R58C
SH_99	1	43649426	43649426	Silent	SNV	T	A	A	CFAP57	c.639T>A	P.V213V
SH_99	1	52924039	52924039	Missense_Mutation	SNV	C	G	G	ZCCHC11	c.3404G>C	P.R1135T
SH_99	1	72241850	72241850	Splice_Site	SNV	T	A	A	NEGR1	c.535+5A>T	
SH_99	10	115973299	115973299	Missense_Mutation	SNV	G	A	A	TDRD1	c.2026G>A	P.E676K
SH_99	10	115987852	115987852	Missense_Mutation	SNV	A	T	T	TDRD1	c.3506A>T	P.N1169I
SH_99	10	119100618	119100618	Splice_Site	SNV	C	A	A	PDZD8	c.873-5G>T	
SH_99	10	12123583	12123583	Silent	SNV	C	T	T	DHTKD1	c.267C>T	P.I89I
SH_99	10	134162503	134162503	Silent	SNV	A	T	T	LRRC27	c.963A>T	P.L321L
SH_99	10	22676827	22676827	Missense_Mutation	SNV	A	C	C	SPAG6	c.679A>C	P.I227L
SH_99	10	29762890	29762890	Silent	SNV	C	G	G	SVIL	c.4476G>C	P.S1492S
SH_99	10	63976895	63976895	Missense_Mutation	SNV	C	A	A	RTKN2	c.1002G>T	P.L334F
SH_99	10	70723092	70723092	Missense_Mutation	SNV	A	G	G	DDX21	c.449A>G	P.H150R
SH_99	11	105929699	105929699	Silent	SNV	T	C	C	KBTBD3	c.126A>G	P.L42L
SH_99	11	14856619	14856619	Silent	SNV	T	C	C	PDE3B	c.2298T>C	P.G766G
SH_99	11	18723250	18723250	Silent	SNV	C	T	T	TMEM86A	c.417C>T	P.L139L
SH_99	11	20122660	20122660	Missense_Mutation	SNV	G	A	A	NAV2	c.6167G>A	P.G2056E
SH_99	11	27114793	27114793	Missense_Mutation	SNV	A	T	T	BBOX1	c.413A>T	P.Y138F
SH_99	11	27406880	27406880	Silent	SNV	T	A	A	LGR4	c.537A>T	P.L179L
SH_99	11	48327792	48327792	Silent	SNV	T	C	C	OR4S1	c.18T>C	P.N6N
SH_99	11	57102046	57102046	Missense_Mutation	SNV	T	C	C	SSRP1	c.131A>G	P.Q44R
SH_99	11	5799397	5799397	Silent	SNV	G	A	A	OR52N5	c.468C>T	P.A156A
SH_99	11	61630785	61630785	Missense_Mutation	SNV	A	T	T	FADS2	c.946A>T	P.T316S
SH_99	11	62298592	62298592	Silent	SNV	T	G	G	AHNAK	c.3297A>C	P.P1099P
SH_99	11	63665721	63665721	Silent	SNV	A	G	G	MARK2	c.306A>G	P.R102R
SH_99	11	6964436	6964436	Silent	SNV	A	T	T	ZNF215	c.606A>T	P.S202S
SH_99	11	73074427	73074427	Missense_Mutation	SNV	A	T	T	ARHGEF17	c.5173A>T	P.S1725C
SH_99	11	8646530	8646530	Silent	SNV	C	A	A	TRIM66	c.2121G>T	P.V707V
SH_99	11	93435990	93435990	Missense_Mutation	SNV	T	C	C	CEP295	c.5501T>C	P.V1834A
SH_99	12	100441357	100441357	Frame_Shift_Del	INDEL	A	-	-	UHRF1BP1L	c.4032delT	P.I1344fs
SH_99	12	109998867	109998867	Missense_Mutation	SNV	C	A	A	MMAB	c.562G>T	P.V188L
SH_99	12	112888278	112888278	Silent	SNV	T	G	G	PTPN11	c.294T>G	P.L98L

SH_99	12	124298384	124298384	Missense_Mutation	SNV	T	A	A	DNAH10	c.3351T>A	P.S1117R
SH_99	12	124352133	124352133	Silent	SNV	G	A	A	DNAH10	c.6933G>A	P.Q2311Q
SH_99	12	132426326	132426326	Missense_Mutation	SNV	A	T	T	PUS1	c.950A>T	P.Q317L
SH_99	12	15800073	15800073	Missense_Mutation	SNV	C	T	T	EPS8	c.1556G>A	P.R519H
SH_99	12	43821205	43821205	Missense_Mutation	SNV	C	T	T	ADAMTS20	c.4013G>A	P.S1338N
SH_99	12	53073903	53073903	Missense_Mutation	SNV	G	T	T	KRT1	c.230C>A	P.S77Y
SH_99	12	56619414	56619414	Silent	SNV	T	C	C	NABP2	c.225T>C	P.A75A
SH_99	12	64472797	64472797	Silent	SNV	A	T	T	SRGAP1	c.1224A>T	P.T408T
SH_99	12	6710638	6710638	Missense_Mutation	SNV	C	A	A	CHD4	c.616G>T	P.G206C
SH_99	12	72343413	72343413	Missense_Mutation	SNV	G	T	T	TPH2	c.586G>T	P.D196Y
SH_99	12	85449971	85449971	Missense_Mutation	SNV	A	G	G	LRRIQ1	c.1400A>G	P.E467G
SH_99	13	66879141	66879141	Silent	SNV	T	A	A	PCDH9	c.3234A>T	P.R1078R
SH_99	15	29428579	29428579	Missense_Mutation	SNV	G	C	C	FAM189A1	c.917C>G	P.P306R
SH_99	15	45983167	45983167	Splice_Site	SNV	A	T	T	SQRDL	c.1296-4A>T	
SH_99	15	50494759	50494759	Missense_Mutation	SNV	A	T	T	SLC27A2	c.764A>T	P.K255M
SH_99	15	70960775	70960775	Frame_Shift_Del	INDEL	T	-	-	UACA	c.2209delA	P.S737fs
SH_99	15	74240248	74240248	Splice_Site	SNV	G	A	A	LOXL1	c.1602+5G>A	
SH_99	15	92988061	92988061	Silent	SNV	C	A	A	ST8SIA2	c.744C>A	P.G248G
SH_99	16	11862261	11862261	Missense_Mutation	SNV	T	A	A	ZC3H7A	c.1270A>T	P.T424S
SH_99	16	23116833	23116833	Frame_Shift_Del	INDEL	C	-	-	USP31	c.1018delG	P.V340fs
SH_99	16	23390074	23390074	Silent	SNV	T	C	C	SCNN1B	c.1452T>C	P.N484N
SH_99	16	23392021	23392021	Nonsense_Mutation	SNV	C	T	T	SCNN1B	c.1822C>T	P.Q608*
SH_99	16	29874177	29874177	Nonsense_Mutation	SNV	A	T	T	CDIPT	c.2T>A	P.M1?
SH_99	16	60392721	60392721	Missense_Mutation	SNV	C	T	T	LOC729159	c.886G>A	P.E296K
SH_99	16	65032724	65032724	Silent	SNV	A	G	G	CDH11	c.264T>C	P.I88I
SH_99	17	35641738	35641738	Splice_Site	INDEL	C	-	-	ACACA	c.471+1delG	
SH_99	17	37234265	37234265	Missense_Mutation	SNV	C	G	G	PLXDC1	c.1087G>C	P.D363H
SH_99	17	42745523	42745523	Missense_Mutation	SNV	T	A	A	MEIOC	c.2244T>A	P.S748R
SH_99	17	43906946	43906946	Missense_Mutation	SNV	G	C	C	CRHR1	c.454G>C	P.G152R
SH_99	17	44109017	44109017	Missense_Mutation	SNV	G	T	T	KANSL1	c.3140C>A	P.A1047E
SH_99	17	56277584	56277584	Splice_Site	SNV	A	G	G	EPX	c.1538-2A>G	
SH_99	17	58136784	58136784	Splice_Site	SNV	T	C	C	HEATR6	c.1722A>G	P.K574K
SH_99	17	7577120	7577120	Missense_Mutation	SNV	C	T	T	TP53	c.818G>A	P.R273H
SH_99	17	7684027	7684027	Missense_Mutation	SNV	T	A	A	DNAH2	c.5930T>A	P.L1977Q
SH_99	17	79916821	79916821	Missense_Mutation	SNV	C	T	T	NOTUM	c.523G>A	P.A175T
SH_99	18	14791470	14791470	Missense_Mutation	SNV	C	G	G	ANKRD30B	c.1805C>G	P.T602S
SH_99	18	18690847	18690847	Missense_Mutation	SNV	T	C	C	ROCK1	c.25A>G	P.T9A
SH_99	18	22806182	22806182	Missense_Mutation	SNV	T	C	C	ZNF521	c.1040A>G	P.N347S
SH_99	18	23658119	23658119	Missense_Mutation	SNV	T	A	A	SS18	c.152A>T	P.Q51L
SH_99	18	2705796	2705796	Silent	SNV	A	G	G	SMCHD1	c.1947A>G	P.Q649Q
SH_99	18	5291250	5291250	Missense_Mutation	SNV	G	C	C	ZBTB14	c.957C>G	P.H319Q
SH_99	18	54362409	54362409	Missense_Mutation	SNV	G	C	C	WDR7	c.1337G>C	P.G446A
SH_99	19	10428248	10428248	Missense_Mutation	SNV	A	T	T	RAVER1	c.2153T>A	P.F718Y
SH_99	19	12163615	12163615	Nonsense_Mutation	SNV	A	T	T	ZNF878	c.2T>A	P.M1?
SH_99	19	15569464	15569464	Missense_Mutation	SNV	G	A	A	RASAL3	c.665C>T	P.P222L
SH_99	19	18901419	18901419	Missense_Mutation	SNV	T	A	A	COMP	c.169A>T	P.R57W
SH_99	19	21991512	21991512	Nonsense_Mutation	SNV	T	A	A	ZNF43	c.1309A>T	P.K437*
SH_99	19	22498424	22498424	Silent	SNV	T	A	A	ZNF729	c.2205T>A	P.T735T
SH_99	19	33321530	33321530	Missense_Mutation	SNV	T	A	A	SLC7A9	c.1460A>T	P.E487V
SH_99	19	37734526	37734526	Missense_Mutation	SNV	C	T	T	ZNF383	c.1388C>T	P.S463L
SH_99	19	38385310	38385310	Missense_Mutation	SNV	T	A	A	WDR87	c.1033A>T	P.S345C
SH_99	19	39056410	39056410	Missense_Mutation	SNV	G	A	A	RYR1	c.13436G>A	P.G4479E
SH_99	19	39409078	39409078	Silent	SNV	C	T	T	SARS2	c.906G>A	P.A302A
SH_99	19	49551638	49551638	Splice_Site	SNV	A	T	T	CGB8	c.16-9T>A	
SH_99	19	49573943	49573943	Missense_Mutation	SNV	T	A	A	KCNA7	c.748A>T	P.I250F
SH_99	19	50949187	50949187	Missense_Mutation	SNV	A	T	T	MYBPC2	c.1184A>T	P.Y395F
SH_99	19	55872239	55872239	Missense_Mutation	SNV	T	A	A	FAM71E2	c.793A>T	P.T265S
SH_99	19	58453032	58453032	Missense_Mutation	SNV	T	A	A	ZNF256	c.1144A>T	P.S382C
SH_99	19	5915128	5915128	Missense_Mutation	SNV	A	C	C	CAPS	c.697A>C	P.N233H
SH_99	19	6586369	6586369	Missense_Mutation	SNV	C	T	T	CD70	c.244G>A	P.G82S
SH_99	19	7755298	7755298	Silent	SNV	C	T	T	FCER2	c.612G>A	P.E204E
SH_99	2	137928508	137928508	Missense_Mutation	SNV	G	A	A	THSD7B	c.1723G>A	P.G575R
SH_99	2	162696317	162696317	Missense_Mutation	SNV	T	C	C	SLC4A10	c.296T>C	P.V99A
SH_99	2	175337958	175337958	Missense_Mutation	SNV	C	A	A	GPR155	c.595G>T	P.A199S
SH_99	2	198367935	198367935	Missense_Mutation	SNV	T	G	G	HSPE1	c.261T>G	P.D87E
SH_99	2	210843267	210843267	Missense_Mutation	SNV	A	G	G	UNC80	c.8755A>G	P.T2919A
SH_99	2	242195596	242195596	Splice_Site	INDEL	-	AG	AG	HDLBP	981+2_981+3insCT	
SH_99	20	23016847	23016847	Missense_Mutation	SNV	C	A	A	SSTR4	c.727C>A	P.L243M
SH_99	20	30436225	30436225	Silent	SNV	G	A	A	DUSP15	c.870C>T	P.S290S
SH_99	20	34241735	34241735	Missense_Mutation	SNV	G	A	A	RBM12	c.1510C>T	P.P504S
SH_99	20	43600795	43600795	Nonsense_Mutation	SNV	G	T	T	STK4	c.112G>T	P.E38*
SH_99	21	28211978	28211978	Silent	SNV	G	T	T	ADAMTS1	c.1956C>A	P.V652V
SH_99	22	19808743	19808743	Splice_Site	SNV	C	A	A	GNB1L	c.128+8G>T	
SH_99	22	29966476	29966476	Frame_Shift_Del	INDEL	A	-	-	NIPSNAP1	c.84delT	P.F28fs
SH_99	22	38039793	38039793	Missense_Mutation	SNV	A	G	G	SH3BP1	c.616A>G	P.R206G
SH_99	22	42180353	42180353	Missense_Mutation	SNV	C	T	T	MEI1	c.3098C>T	P.T1033I
SH_99	22	51048663	51048663	Missense_Mutation	SNV	A	C	C	MAPK8IP2	c.2324A>C	P.H775P
SH_99	3	113179525	113179525	Missense_Mutation	SNV	G	C	C	SPICE1	c.1374C>G	P.N458K
SH_99	3	132180013	132180013	Silent	SNV	A	G	G	DNAJC13	c.1677A>G	P.V559V
SH_99	3	184003327	184003327	Missense_Mutation	SNV	C	T	T	ECE2	c.1123C>T	P.R375C
SH_99	3	190158052	190158052	Silent	SNV	T	A	A	TMEM207	c.285A>T	P.G95G
SH_99	3	3139934	3139934	Silent	SNV	T	G	G	IL5RA	c.408A>C	P.T136T
SH_99	3	48510523	48510523	Missense_Mutation	SNV	G	A	A	SHISA5	c.685C>T	P.P229S
SH_99	3	64607992	64607992	Silent	SNV	C	A	A	ADAMTS9	c.2484G>T	P.V828V
SH_99	4	10567698	10567698	Missense_Mutation	SNV	A	T	T	CLNK	c.227T>A	P.M76K
SH_99	4	1379675	1379675	Missense_Mutation	SNV	G	A	A	UVSSA	c.2056G>A	P.V686M
SH_99	4	140441478	140441478	Missense_Mutation	SNV	T	A	A	SETD7	c.715A>T	P.N239Y
SH_99	4	74275104	74275116	Frame_Shift_Del	INDEL	CTTTTATGCC	-	-	ALB	527delACTTTTAT	P.Y172fs
SH_99	4	7726879	7726879	Splice_Site	SNV	A	T	T	SORCS2	c.2612-2A>T	
SH_99	5	111540117	111540117	Missense_Mutation	SNV	G	A	A	EPB41L4A	c.1331C>T	P.P444L
SH_99	5	134782493	134782493	Silent	SNV	T	C	C	DCANP1	c.306A>G	P.A102A
SH_99	5	140073865	140073883	Frame_Shift_Del	INDEL	TCCTTTTGAG	-	-	HARS2	8delTGTATCCTT	P.V109fs
SH_99	5	145969766	145969766	Missense_Mutation	SNV	T	C	C	PPP2R2B	c.1094A>G	P.N365S
SH_99	5	160761852	160761852	Silent	SNV	G	A	A	GABRB2	c.739C>T	P.L247L
SH_99	5	161580363	161580363	Missense_Mutation	SNV	C	T	T	GABRG2	c.1393C>T	P.L465F
SH_99	5	167929115	167929115	Splice_Site	SNV	G	A	A	RARS	c.1057+5G>A	
SH_99	5	26915890	26915890	Missense_Mutation	SNV	T	A	A	CDH9	c.371A>T	P.Y124F
SH_99	5	32010481	32010481	Missense_Mutation	SNV	C	A	A	PDZD2	c.1300C>A	P.P434T

SH_99	5	38338834	38338834	Missense_Mutation	SNV	T	A	A	EGFLAM	c.242T>A	P.L81Q
SH_99	5	38412726	38412726	Silent	SNV	C	A	A	EGFLAM	c.1470C>A	P.G490G
SH_99	5	38506152	38506152	Silent	SNV	A	G	G	LIFR	c.1146T>C	P.L382L
SH_99	5	488440	488440	Silent	SNV	T	A	A	SLC9A3	c.666A>T	P.A222A
SH_99	5	54404055	54404055	Missense_Mutation	SNV	G	A	A	GZMA	c.460G>A	P.G154S
SH_99	6	109802547	109802547	Missense_Mutation	SNV	T	A	A	ZBTB24	c.683A>T	P.E228V
SH_99	6	111587678	111587678	Missense_Mutation	SNV	A	T	T	MFSD4B	c.913A>T	P.S305C
SH_99	6	112462042	112462042	Missense_Mutation	SNV	A	C	C	LAMA4	c.2896T>G	P.F966V
SH_99	6	151672199	151672199	Missense_Mutation	SNV	G	C	C	AKAP12	c.2673G>C	P.M891I
SH_99	6	31712030	31712030	Missense_Mutation	SNV	G	C	C	MSH5	c.601G>C	P.D201H
SH_99	6	33168241	33168241	Missense_Mutation	SNV	C	A	A	RXRB	c.13G>T	P.A5S
SH_99	6	43323428	43323428	Silent	SNV	T	C	C	ZNF318	c.1644A>G	P.A548A
SH_99	7	122261550	122261550	Silent	SNV	C	T	T	CADPS2	c.1089G>A	P.L363L
SH_99	7	134800350	134800350	Nonsense_Mutation	SNV	C	T	T	AGBL3	c.2329C>T	P.Q777*
SH_99	7	142574150	142574150	Splice_Site	SNV	C	A	A	TRPV6	c.882+11G>T	
SH_99	7	143748025	143748025	Missense_Mutation	SNV	C	G	G	OR2A5	c.531C>G	P.F177L
SH_99	7	27195946	27195946	Silent	SNV	G	T	T	HOXA7	c.219C>A	P.G73G
SH_99	7	44747549	44747549	Missense_Mutation	SNV	G	A	A	OGDH	c.3011G>A	P.R1004H
SH_99	7	48311617	48311617	Missense_Mutation	SNV	C	G	G	ABCA13	c.2354C>G	P.P785R
SH_99	7	56945249	56945249	Splice_Site	SNV	A	T	T	LOC100130849	n.195-6T>A	
SH_99	8	133961016	133961016	Splice_Site	SNV	C	A	A	TG	c.5234-5C>A	
SH_99	8	142458115	142458115	Missense_Mutation	SNV	A	G	G	MROH5	c.2711T>C	P.L904P
SH_99	8	143958306	143958306	Splice_Site	INDEL	G	-	-	CYP11B1	c.596-5delC	
SH_99	8	21560317	21560317	Splice_Site	SNV	C	T	T	GFRA2	c.903G>A	P.Q301Q
SH_99	9	111741564	111741564	Missense_Mutation	SNV	T	A	A	CTNNAL1	c.1098A>T	P.Q366H
SH_99	9	130482367	130482367	Missense_Mutation	SNV	T	C	C	TTC16	c.398T>C	P.L133P
SH_99	9	79318286	79318286	Missense_Mutation	SNV	A	G	G	PRUNE2	c.8243T>C	P.L2748P
SH_99	X	100808778	100808778	Missense_Mutation	SNV	A	T	T	ARMCX1	c.865A>T	P.S289C
SH_99	X	129349898	129349898	Missense_Mutation	SNV	T	A	A	ZNF280C	c.1705A>T	P.T569S
SH_99	X	12939574	12939574	Silent	SNV	T	C	C	TLR8	c.2469T>C	P.S823S
SH_99	X	13337934	13337934	Silent	SNV	T	A	A	ATXN3L	c.120A>T	P.L40L
SH_99	X	37664381	37664381	Missense_Mutation	SNV	A	G	G	CYBB	c.1274A>G	P.Y425C
XH_P01	1	149859194	149859194	Missense_Mutation	SNV	G	C	C	HIST2H2AB	c.273C>G	P.D91E
XH_P01	1	222838724	222838724	Silent	SNV	T	C	C	MIA3	c.2121T>C	P.P707P
XH_P01	1	228475953	228475953	Missense_Mutation	SNV	G	T	T	OBSCN	c.10003G>T	P.A3335S
XH_P01	1	229738394	229738394	Missense_Mutation	SNV	A	G	G	TAF5L	c.520T>C	P.Y174H
XH_P01	1	237664085	237664085	Missense_Mutation	SNV	G	A	A	RYR2	c.2278G>A	P.D760N
XH_P01	1	67672667	67672667	Missense_Mutation	SNV	G	A	A	IL23R	c.727G>A	P.D243N
XH_P01	11	118235819	118235819	Missense_Mutation	SNV	C	G	G	UBE4A	c.24C>G	P.N8K
XH_P01	11	26587304	26587304	Silent	SNV	G	A	A	MUC15	c.183C>T	P.N61N
XH_P01	11	71714951	71714951	Silent	SNV	G	C	C	NUMA1	c.6276C>G	P.T2092T
XH_P01	11	93862514	93862514	Silent	SNV	C	T	T	PANX1	c.36C>T	P.F12F
XH_P01	12	10213820	10213820	Silent	SNV	T	C	C	CLEC9A	c.267T>C	P.C89C
XH_P01	12	107371696	107371696	Missense_Mutation	SNV	C	A	A	MTERF2	c.797G>T	P.S266I
XH_P01	12	26648182	26648182	Silent	SNV	T	C	C	ITPR2	c.5085A>G	P.L1695L
XH_P01	12	57573673	57573673	Missense_Mutation	SNV	C	G	G	LRP1	c.5075C>G	P.S1692C
XH_P01	12	8667850	8667850	Missense_Mutation	SNV	C	T	T	CLEC4D	c.47C>T	P.P16L
XH_P01	12	88500811	88500811	Missense_Mutation	SNV	G	C	C	CEP290	c.2548C>G	P.Q850E
XH_P01	13	110959359	110959359	Missense_Mutation	SNV	T	G	G	COL4A1	c.16A>C	P.S6R
XH_P01	13	111955360	111955360	Missense_Mutation	SNV	C	T	T	ARHGEF7	c.2066C>T	P.S689F
XH_P01	13	43566239	43566239	Silent	SNV	G	C	C	EPSTI1	c.63C>G	P.T21T
XH_P01	15	73660199	73660199	Missense_Mutation	SNV	G	T	T	HCN4	c.413C>A	P.S138Y
XH_P01	16	24583242	24583242	Missense_Mutation	SNV	A	C	C	RBBP6	c.4855A>C	P.S1619R
XH_P01	16	88599909	88599909	Missense_Mutation	SNV	C	T	T	ZFPM1	c.1543C>T	P.P515S
XH_P01	17	18539782	18539782	Missense_Mutation	SNV	G	A	A	TBC1D28	c.626C>T	P.T209I
XH_P01	17	5991412	5991412	Missense_Mutation	SNV	C	T	T	WSCD1	c.530C>T	P.A177V
XH_P01	17	79518111	79518111	Missense_Mutation	SNV	C	A	A	FAAP100	c.409G>T	P.D137Y
XH_P01	18	66506122	66506122	Silent	SNV	G	T	T	CCDC102B	c.786G>T	P.V262V
XH_P01	19	10602341	10602341	Missense_Mutation	SNV	G	A	A	KEAP1	c.1237C>T	P.R413C
XH_P01	19	43093599	43093599	Splice_Site	SNV	A	G	G	CEACAM8	c.703+10T>C	
XH_P01	19	52091424	52091424	Missense_Mutation	SNV	C	T	T	ZNF175	c.1840C>T	P.P614S
XH_P01	19	53014288	53014288	Silent	SNV	C	T	T	ZNF578	c.654C>T	P.L218L
XH_P01	19	54632474	54632474	Missense_Mutation	SNV	G	A	A	PRPF31	c.1189G>A	P.G397S
XH_P01	2	202134249	202134249	Missense_Mutation	SNV	A	G	G	CASP8	c.322A>G	P.M108V
XH_P01	2	210558106	210558106	Missense_Mutation	SNV	G	A	A	MAP2	c.1212G>A	P.M404I
XH_P01	2	215910727	215910727	Missense_Mutation	SNV	G	T	T	ABCA12	c.706C>A	P.P236T
XH_P01	20	36769757	36769757	Missense_Mutation	SNV	C	T	T	TGM2	c.1026G>A	P.M342I
XH_P01	22	38234520	38234520	Silent	SNV	G	T	T	ANKRD54	c.462C>A	P.G154G
XH_P01	22	45750972	45750972	Silent	SNV	G	A	A	SMC1B	c.2985C>T	P.I995I
XH_P01	3	121134796	121134804	Missense_Mutation	INDEL	ATTTGACAG	-	-	STXBP5L	3_3216delTTTGA	F1070_R1072del
XH_P01	3	46775282	46775282	Missense_Mutation	SNV	T	G	G	PRSS46	c.364A>C	P.S122R
XH_P01	4	3449695	3449695	Silent	SNV	C	T	T	HGFAC	c.1590C>T	P.P530P
XH_P01	4	99407920	99407920	Missense_Mutation	SNV	G	A	A	TSPAN5	c.248C>T	P.A83V
XH_P01	5	11346513	11346513	Silent	SNV	C	T	T	CTNND2	c.1326G>A	P.P442P
XH_P01	5	125939460	125939460	Missense_Mutation	SNV	T	G	G	PHAX	c.295T>G	P.F99V
XH_P01	5	140214119	140214119	Missense_Mutation	SNV	G	A	A	PCDHA7	c.151G>A	P.D51N
XH_P01	5	176721441	176721441	Missense_Mutation	SNV	G	A	A	NSD1	c.7072G>A	P.D2358N
XH_P01	5	94764383	94764383	Missense_Mutation	SNV	A	T	T	FAM81B	c.733A>T	P.I245F
XH_P01	7	100684905	100684905	Missense_Mutation	SNV	G	A	A	MUC17	c.10208G>A	P.S3403N
XH_P01	7	105255226	105255226	Missense_Mutation	SNV	G	T	T	ATXN7L1	c.907C>A	P.P303T
XH_P01	7	150696136	150696136	Missense_Mutation	SNV	C	A	A	NOS3	c.919C>A	P.P307T
XH_P01	8	142486189	142486189	Missense_Mutation	SNV	G	A	A	MROH5	c.1504C>T	P.R502C
XH_P01	8	146068297	146068297	Missense_Mutation	SNV	T	C	C	ZNF7	c.1838T>C	P.I613T
XH_P01	8	68423880	68423880	Missense_Mutation	SNV	C	G	G	CPA6	c.328G>C	P.E110Q
XH_P01	8	94738721	94738721	Missense_Mutation	SNV	T	C	C	FAM92A1	c.757T>C	P.C253R
XH_P01	9	113096539	113096539	Missense_Mutation	SNV	G	A	A	TXNDC8	c.86C>T	P.S29L
XH_P01	9	140057773	140057773	Missense_Mutation	SNV	C	A	A	GRIN1	c.2324C>A	P.S775Y
XH_P01	9	86616477	86616477	Silent	SNV	T	A	A	RMI1	c.576T>A	P.G192G
XH_P01	X	8553320	8553320	Missense_Mutation	SNV	G	A	A	ANOS1	c.844C>T	P.R282C
XH_P01	Y	16942259	16942259	Missense_Mutation	SNV	G	A	A	NLGN4Y	c.1461G>A	P.S487S
XH_P02	1	150915032	150915032	Splice_Site	SNV	T	G	G	SETDB1	c.548-7T>G	
XH_P02	1	173493988	173493988	Missense_Mutation	SNV	G	T	T	SLC9C2	c.2444C>A	P.A815D
XH_P02	1	26691694	26691694	Missense_Mutation	SNV	C	A	A	ZNF683	c.343G>T	P.A115S
XH_P02	1	26691695	26691695	Missense_Mutation	SNV	C	A	A	ZNF683	c.342G>T	P.Q114H
XH_P02	1	85656188	85656188	Silent	SNV	G	A	A	SYDE2	c.993C>T	P.L331L
XH_P02	10	105176422	105176422	Missense_Mutation	SNV	G	T	T	PDCD11	c.1693G>T	P.V565L
XH_P02	10	128147615	128147615	Silent	SNV	G	A	A	C10orf90	c.1891C>T	P.L631L
XH_P02	10	49935617	49935617	Missense_Mutation	SNV	T	C	C	WDFY4	c.884T>C	P.V295A

XH_P02	10	97373840	97373840	Missense_Mutation	SNV	G	T	T	ALDH18A1	c.1678C>A	P.Q560K
XH_P02	11	111715447	111715447	Splice_Site	SNV	C	T	T	ALG9	c.896-1G>A	
XH_P02	11	117306527	117306527	Splice_Site	SNV	C	A	A	DSCAML1	c.4901-12G>T	
XH_P02	11	122852165	122852165	Missense_Mutation	SNV	G	T	T	BSX	c.215C>A	P.P72H
XH_P02	11	17575067	17575067	Splice_Site	SNV	G	C	C	OTOG	c.576+1G>C	
XH_P02	11	32954189	32954189	Missense_Mutation	SNV	C	G	G	QSER1	c.998C>G	P.P333R
XH_P02	11	46406842	46406877	Missense_Mutation	INDEL	ACGTTGTAGC	-	-	CHRM4	TGGACGCCCTAC	P.L411_V422del
XH_P02	11	55419162	55419162	Silent	SNV	G	A	A	OR4S2	c.783G>A	P.T261T
XH_P02	11	58190486	58190486	Silent	SNV	G	A	A	OR5B2	c.249C>T	P.F83F
XH_P02	11	63993277	63993277	Missense_Mutation	SNV	G	A	A	TRPT1	c.56C>T	P.A19V
XH_P02	12	114804154	114804154	Missense_Mutation	SNV	T	A	A	TBX5	c.798A>T	P.K266N
XH_P02	12	124319945	124319945	Splice_Site	SNV	A	C	C	DNAH10	c.4426-8A>C	
XH_P02	12	54903516	54903516	Silent	SNV	G	C	C	NCKAP1L	c.420G>C	P.L140L
XH_P02	13	27246022	27246029	Frame_Shift_Del	INDEL	GATGGGCT	-	-	WASF3	8_445delTGGG	P.D146fs
XH_P02	13	42263587	42263624	Frame_Shift_Del	INDEL	TAGTTGATCA	-	-	VWA8	ATTTCCAGTGATC	P.H1333fs
XH_P02	13	99036996	99036996	Missense_Mutation	SNV	A	G	G	FARP1	c.509A>G	P.D170G
XH_P02	14	52481797	52481797	Silent	SNV	G	T	T	NID2	c.3225C>A	P.R1075R
XH_P02	14	58714498	58714498	Missense_Mutation	SNV	G	A	A	PSMA3	c.52G>A	P.D18N
XH_P02	14	64407358	64407358	Missense_Mutation	SNV	G	T	T	SYNE2	c.106G>T	P.A36S
XH_P02	14	78709655	78709655	Silent	SNV	C	T	T	NRXN3	c.219C>T	P.C73C
XH_P02	15	35202495	35202495	Missense_Mutation	SNV	C	T	T	AQR	c.1504G>A	P.V502I
XH_P02	15	89060216	89060216	Splice_Site	SNV	G	C	C	DET1	c.1272-12C>G	
XH_P02	15	99434840	99434840	Silent	SNV	G	A	A	IGF1R	c.927G>A	P.S309S
XH_P02	16	1259233	1259233	Missense_Mutation	SNV	C	A	A	CACNA1H	c.3565C>A	P.R1189S
XH_P02	16	24573001	24573001	Missense_Mutation	SNV	C	G	G	RBBP6	c.916C>G	P.P306A
XH_P02	16	74415476	74415476	Silent	SNV	C	T	T	NPIPB15	c.129C>T	P.D43D
XH_P02	16	75665348	75665348	Missense_Mutation	SNV	C	A	A	KARS	c.1302G>T	P.M434I
XH_P02	17	1401300	1401300	Missense_Mutation	SNV	C	T	T	INPP5K	c.665G>A	P.R222K
XH_P02	17	29548945	29548945	Frame_Shift_Ins	INDEL	-	AG	AG	NF1	1720_1721dupA	P.Y575fs
XH_P02	17	32957157	32957157	Missense_Mutation	SNV	A	T	T	TMEM132E	c.1469A>T	P.E490V
XH_P02	17	46669797	46669797	Missense_Mutation	SNV	T	C	C	HOXB5	c.584A>G	P.K195R
XH_P02	17	54939559	54939559	Missense_Mutation	SNV	A	G	G	DGKE	c.1471A>G	P.I491V
XH_P02	17	58136863	58136863	Nonsense_Mutation	SNV	G	C	C	HEATR6	c.1643C>G	P.S548*
XH_P02	17	60642376	60642376	Missense_Mutation	SNV	G	C	C	TLK2	c.846G>C	P.K282N
XH_P02	17	71427811	71427811	Splice_Site	SNV	G	T	T	SDK2	c.1313-3C>A	
XH_P02	17	78073445	78073445	Silent	SNV	G	C	C	CCDC40	c.3300G>C	P.L1100L
XH_P02	19	19369352	19369352	Missense_Mutation	SNV	C	T	T	HAPLN4	c.797G>A	P.C266Y
XH_P02	19	3600621	3600621	Silent	SNV	G	A	A	TBXA2R	c.12C>T	P.N4N
XH_P02	19	41239209	41239209	Missense_Mutation	SNV	G	A	A	ITPKC	c.1614G>A	P.M538I
XH_P02	19	44082863	44082863	Splice_Site	SNV	G	C	C	PINLYP	c.259+5G>C	
XH_P02	19	44681311	44681311	Silent	SNV	C	T	T	ZNF226	c.1896C>T	P.A632A
XH_P02	19	5221145	5221145	Silent	SNV	G	A	A	PTPRS	c.3321C>T	P.G1107G
XH_P02	19	52876405	52876405	Missense_Mutation	SNV	G	C	C	ZNF880	c.54G>C	P.Q18H
XH_P02	19	53304356	53304356	Missense_Mutation	SNV	A	G	G	ZNF28	c.742T>C	P.Y248H
XH_P02	19	56090932	56090932	Missense_Mutation	SNV	C	T	T	ZNF579	c.74G>A	P.G25D
XH_P02	19	581560	581560	Silent	SNV	G	A	A	BSG	c.690G>A	P.E230E
XH_P02	19	8373094	8373094	Silent	SNV	G	T	T	CD320	c.81C>A	P.L27L
XH_P02	2	100203615	100203615	Splice_Site	SNV	C	A	A	AFF3	c.2591+1G>T	
XH_P02	2	171243770	171243770	Missense_Mutation	SNV	G	A	A	MYO3B	c.1529G>A	P.R510K
XH_P02	2	186670236	186670236	Missense_Mutation	SNV	C	A	A	FSIP2	c.16203C>A	P.D5401E
XH_P02	2	201862262	201862262	Missense_Mutation	SNV	A	G	G	FAM126B	c.532T>C	P.F178L
XH_P02	2	20251182	20251182	Missense_Mutation	SNV	T	A	A	LAPTM4A	c.100A>T	P.T34S
XH_P02	2	55106756	55106756	Missense_Mutation	SNV	G	C	C	EML6	c.2417G>C	P.G806A
XH_P02	21	43846809	43846809	Missense_Mutation	SNV	G	A	A	UBASH3A	c.936G>A	P.M312I
XH_P02	3	27363704	27363704	Missense_Mutation	SNV	G	C	C	NEK10	c.411C>G	P.I137M
XH_P02	3	47618948	47618948	Missense_Mutation	SNV	C	G	G	CSPG5	c.154G>C	P.E52Q
XH_P02	4	13603624	13603624	Frame_Shift_Ins	INDEL	-	CA	CA	BOD1L1	4898_4899dupT	P.H1634fs
XH_P02	4	157782554	157782569	Splice_Site	INDEL	AATAAAAATA	-	-	PDGFC	70+27delGTATATTTTTATTTAC	
XH_P02	4	52861722	52861722	Missense_Mutation	SNV	C	A	A	LRRC66	c.1466G>T	P.G489V
XH_P02	4	95202724	95202724	Missense_Mutation	SNV	G	C	C	SMARCAD1	c.2715G>C	P.K905N
XH_P02	5	140044539	140044539	Missense_Mutation	SNV	C	A	A	WDR55	c.42C>A	P.S14R
XH_P02	5	140784744	140784744	Missense_Mutation	SNV	G	T	T	PCDHGA9	c.2225G>T	P.G742V
XH_P02	6	100055062	100055062	Splice_Site	SNV	C	T	T	PRDM13	c.144+8C>T	
XH_P02	6	136787905	136787905	Missense_Mutation	SNV	G	C	C	MAP7	c.16C>G	P.L6V
XH_P02	6	168927051	168927051	Missense_Mutation	SNV	G	C	C	SMOC2	c.282G>C	P.R94S
XH_P02	6	17601146	17601146	Missense_Mutation	SNV	T	G	G	FAM8A1	c.506T>G	P.L169R
XH_P02	6	99283623	99283623	Missense_Mutation	SNV	G	C	C	POU3F2	c.874G>C	P.G292R
XH_P02	7	120496816	120496816	Nonsense_Mutation	SNV	A	T	T	TSPAN12	c.2T>A	P.M1?
XH_P02	7	121944378	121944378	Silent	SNV	C	T	T	FEZF1	c.114G>A	P.A38A
XH_P02	7	127326763	127326763	Missense_Mutation	SNV	C	T	T	SND1	c.175C>T	P.L59F
XH_P02	7	13946129	13946129	Missense_Mutation	SNV	C	A	A	ETV1	c.1036G>T	P.D346Y
XH_P02	7	42088288	42088288	Missense_Mutation	SNV	C	T	T	GLI3	c.481G>A	P.A161T
XH_P02	7	5270481	5270481	Missense_Mutation	SNV	T	G	G	WIPI2	c.1235T>G	P.L412R
XH_P02	7	65547932	65547932	Splice_Site	SNV	G	T	T	ASL	c.348+9G>T	
XH_P02	7	99914732	99914732	Missense_Mutation	SNV	G	A	A	SPDYE3	c.1300G>A	P.G434S
XH_P02	8	113267550	113267550	Silent	SNV	T	C	C	CSMD3	c.9462A>G	P.L3154L
XH_P02	8	144990529	144990529	Missense_Mutation	SNV	G	C	C	PLEC	c.13541C>G	P.A4514G
XH_P02	8	25136112	25136112	Missense_Mutation	SNV	G	T	T	DOCK5	c.252G>T	P.E84D
XH_P02	9	124544696	124544704	Missense_Mutation	INDEL	CCGTAACGG	-	-	DAB2IP	3_3126delCGTAA	R1040_G1042del
XH_P02	9	131233655	131233655	Silent	SNV	G	A	A	ODF2	c.474G>A	P.E158E
XH_P02	9	137326016	137326016	Missense_Mutation	SNV	G	A	A	RXRA	c.1204G>A	P.A402T
XH_P02	9	140218174	140218174	Splice_Site	SNV	C	G	G	EXD3	c.2184+3G>C	
XH_P02	9	35650527	35650527	Missense_Mutation	SNV	G	A	A	SIT1	c.208C>T	P.R70W
XH_P02	9	73168099	73168099	Silent	SNV	G	A	A	TRPM3	c.2862C>T	P.V954V
XH_P02	9	95481094	95481094	Silent	SNV	G	T	T	BICD2	c.1833C>A	P.S611S
XH_P02	X	44703479	44703479	Missense_Mutation	SNV	G	C	C	DUSP21	c.101G>C	P.G34A
XH_P02	X	76872153	76872153	Missense_Mutation	SNV	C	T	T	ATRX	c.5494G>A	P.E1832K
XH_P02	X	9622356	9622356	Missense_Mutation	SNV	G	C	C	TBL1X	c.205G>C	P.E69Q
XH_P03	1	15495211	15495211	Missense_Mutation	SNV	A	C	C	C1orf195	c.161T>G	P.L54R
XH_P03	1	155291136	155291136	Silent	SNV	G	A	A	RUSC1-AS1	c.144C>T	P.S48S
XH_P03	1	159907625	159907625	Missense_Mutation	SNV	C	A	A	IGSF9	c.251G>T	P.R84L
XH_P03	1	161967977	161967977	Missense_Mutation	SNV	C	A	A	OLFML2B	c.1115G>T	P.W372L
XH_P03	1	215408203	215408203	Nonsense_Mutation	SNV	G	A	A	KCNK2	c.984G>A	P.W328*
XH_P03	1	53732201	53732201	Silent	SNV	C	T	T	LRP8	c.1371G>A	P.V457V
XH_P03	10	27499836	27499836	Missense_Mutation	SNV	C	T	T	ACBD5	c.1006G>A	P.G336R
XH_P03	11	118362629	118362629	Silent	SNV	C	T	T	KMT2A	c.4981C>T	P.L1661L
XH_P03	11	20065830	20065830	Splice_Site	SNV	G	C	C	NAV2	c.3007+12G>C	
XH_P03	11	77412557	77412557	Missense_Mutation	SNV	T	A	A	RSF1	c.1717A>T	P.T573S

XH_P03	12	10961913	10961913	Silent	SNV	G	A	A	TAS2R9	c.762C>T	P.S254S
XH_P03	12	112471137	112471141	Frame_Shift_Del	INDEL	GTCTG	-	-	NAA25	592_2696delCA(	P.Q898fs
XH_P03	12	130926460	130926460	Silent	SNV	C	T	T	RIMBP2	c.1386G>A	P.P462P
XH_P03	12	32137217	32137217	Missense_Mutation	SNV	A	C	C	KIAA1551	c.3328A>C	P.S1110R
XH_P03	12	40882992	40882992	Missense_Mutation	SNV	G	T	T	MUC19	c.16238G>T	P.W5413L
XH_P03	13	73345038	73345038	Splice_Site	SNV	G	A	A	DIS3	c.1755+4C>T	
XH_P03	14	45599894	45599894	Splice_Site	SNV	A	G	G	FKBP3	c.210+8T>C	
XH_P03	15	65622694	65622694	Nonsense_Mutation	SNV	G	A	A	IGDCC3	c.1795C>T	P.Q599*
XH_P03	15	83499668	83499668	Missense_Mutation	INDEL	-	CCCCCA	CCCCCA	WHAMM	65_1970dupAC(	P.P656_P657dup
XH_P03	16	30018509	30018509	Silent	SNV	G	A	A	DOC2A	c.639C>T	P.L213L
XH_P03	17	11686963	11686963	Nonsense_Mutation	SNV	G	A	A	DNAH9	c.7719G>A	P.W2573*
XH_P03	17	29684099	29684101	Missense_Mutation	INDEL	TAC	-	-	NF1	7861_7863delA(	P.T2621del
XH_P03	17	36483753	36483753	Missense_Mutation	SNV	C	T	T	GPR179	c.5699G>A	P.S1900N
XH_P03	17	48941207	48941207	Missense_Mutation	SNV	T	G	G	TOB1	c.172A>C	P.I58L
XH_P03	17	77080716	77080716	Silent	SNV	G	T	T	ENGASE	c.1539G>T	P.L513L
XH_P03	17	79671676	79671676	Missense_Mutation	SNV	T	A	A	MRPL12	c.275T>A	P.I92N
XH_P03	19	44377599	44377599	Missense_Mutation	SNV	C	T	T	ZNF404	c.758G>A	P.R253Q
XH_P03	19	4546298	4546298	Splice_Site	SNV	A	T	T	SEMA6B	c.1680-12T>A	
XH_P03	19	4817728	4817728	Missense_Mutation	SNV	T	G	G	TICAM1	c.662A>C	P.H221P
XH_P03	2	163059643	163059643	Missense_Mutation	SNV	T	C	C	FAP	c.985A>G	P.T329A
XH_P03	2	181848774	181848774	Missense_Mutation	SNV	C	G	G	UBE2E3	c.199C>G	P.Q67E
XH_P03	2	185803076	185803076	Missense_Mutation	SNV	A	T	T	ZNF804A	c.2953A>T	P.N985Y
XH_P03	2	96521150	96521150	Missense_Mutation	SNV	G	A	A	ANKRD36C	c.5957C>T	P.A1986V
XH_P03	20	42789050	42789050	Splice_Site	SNV	G	T	T	JPH2	c.380-3C>A	
XH_P03	20	42789051	42789051	Splice_Site	SNV	C	T	T	JPH2	c.380-4G>A	
XH_P03	3	14964543	14964543	Splice_Site	SNV	G	A	A	FGD5	c.3808-10G>A	
XH_P03	3	178936091	178936091	Missense_Mutation	SNV	G	A	A	PIK3CA	c.1633G>A	P.E545K
XH_P03	3	45134810	45134810	Missense_Mutation	SNV	G	A	A	CDCP1	c.1586C>T	P.S529F
XH_P03	3	50143140	50143140	Missense_Mutation	SNV	A	G	G	RBM5	c.853A>G	P.M285V
XH_P03	4	100824906	100824906	Missense_Mutation	SNV	T	C	C	DNAJB14	c.992A>G	P.N331S
XH_P03	4	17887763	17887763	Missense_Mutation	SNV	G	A	A	LCORL	c.704C>T	P.S235F
XH_P03	5	113740517	113740517	Missense_Mutation	SNV	G	T	T	KCNN2	c.965G>T	P.W322L
XH_P03	5	113740518	113740518	Missense_Mutation	SNV	G	T	T	KCNN2	c.966G>T	P.W322C
XH_P03	5	140202741	140202741	Missense_Mutation	SNV	G	A	A	PCDHA5	c.1381G>A	P.V461M
XH_P03	5	92929283	92929283	Missense_Mutation	SNV	C	T	T	NR2F1	c.1007C>T	P.S336L
XH_P03	5	95234171	95234171	Missense_Mutation	SNV	G	T	T	ELL2	c.1298C>A	P.T433N
XH_P03	6	161006150	161006150	Missense_Mutation	SNV	C	G	G	LPA	c.4217G>C	P.G1406A
XH_P03	6	42225920	42225920	Silent	SNV	G	A	A	TRERF1	c.2349C>T	P.N783N
XH_P03	7	135433253	135433253	Missense_Mutation	SNV	C	A	A	FAM180A	c.76G>T	P.A26S
XH_P03	7	143043691	143043691	Silent	SNV	C	T	T	CLCN1	c.2304C>T	P.F768F
XH_P03	7	148506444	148506444	Missense_Mutation	SNV	G	A	A	EZH2	c.2068C>T	P.R690C
XH_P03	8	146032778	146032778	Silent	SNV	C	G	G	ZNF517	c.456C>G	P.S152S
XH_P03	9	125589002	125589002	Missense_Mutation	SNV	T	C	C	PDCL	c.65A>G	P.D22G
XH_P03	X	147003491	147003491	Missense_Mutation	SNV	C	T	T	FMR1	c.92C>T	P.A31V
XH_P03	X	41088615	41088615	Missense_Mutation	SNV	A	G	G	USP9X	c.7171A>G	P.M2391V
XH_P03	X	53563452	53563452	Missense_Mutation	SNV	C	A	A	HUWE1	c.12314G>T	P.S4105I
XH_P03	X	53615355	53615355	Missense_Mutation	SNV	G	T	T	HUWE1	c.4601C>A	P.T1534K
XH_P04	1	1653154	1653155	Splice_Site	INDEL	AG	-	-	CDK11A	112-5_112-4delCT	
XH_P04	1	66075632	66075632	Missense_Mutation	SNV	G	A	A	LEPR	c.1755G>A	P.M585I
XH_P04	1	68905317	68905320	Frame_Shift_Del	INDEL	CTTC	-	-	RPE65	649_652delGAA	P.E217fs
XH_P04	1	91980363	91980364	Splice_Site	INDEL	TT	-	-	CDC7	19-11_919-10delTT	
XH_P04	11	380337	380337	Missense_Mutation	SNV	C	G	G	B4GALNT4	c.2761C>G	P.P921A
XH_P04	11	46717223	46717223	Missense_Mutation	SNV	G	C	C	ARHGAP1	c.216C>G	P.I72M
XH_P04	11	5462320	5462320	Missense_Mutation	SNV	C	T	T	OR51I1	c.425G>A	P.R142H
XH_P04	11	6220784	6220784	Missense_Mutation	SNV	G	A	A	OR52W1	c.331G>A	P.A111T
XH_P04	11	797770	797772	Missense_Mutation	INDEL	AAG	-	-	PANO1	.145_147delAA(	P.K49del
XH_P04	12	111779848	111779850	Missense_Mutation	INDEL	ACA	-	-	CUX2	3652_3654delA	P.N1218del
XH_P04	12	57485551	57485551	Missense_Mutation	SNV	C	T	T	NAB2	c.727C>T	P.R243C
XH_P04	12	78400963	78400964	Frame_Shift_Del	INDEL	AA	-	-	NAV3	.1646_1647delA	P.K549fs
XH_P04	14	39555003	39555003	Frame_Shift_Del	INDEL	G	-	-	SEC23A	c.791delC	P.S264fs
XH_P04	14	51088550	51088550	Splice_Site	INDEL	T	-	-	ATL1	c.991-3delT	
XH_P04	15	53992072	53992073	Frame_Shift_Del	INDEL	TT	-	-	WDR72	.1639_1640delA	P.K547fs
XH_P04	15	62266480	62266483	Splice_Site	INDEL	TATT	-	-	VPS13C	38+4_2538+7delAATA	
XH_P04	16	4723530	4723530	Missense_Mutation	SNV	A	G	G	MGRN1	c.827A>G	P.N276S
XH_P04	16	58540824	58540824	Frame_Shift_Del	INDEL	G	-	-	NDRG4	c.682delG	P.V228fs
XH_P04	16	66762968	66762970	Missense_Mutation	INDEL	TCA	-	-	DYNC1LI2	.843_845delTG	P.D281del
XH_P04	17	33771703	33771703	Missense_Mutation	SNV	C	T	T	SLFN13	c.997G>A	P.V333M
XH_P04	17	38711735	38711735	Silent	SNV	G	A	A	CCR7	c.207C>T	P.I69I
XH_P04	18	21112253	21112256	Splice_Site	INDEL	GAGA	-	-	NPC1	55-8_3755-5delTCTC	
XH_P04	18	2739508	2739510	Missense_Mutation	INDEL	AGG	-	-	SMCHD1	3506_3508delG	P.G1169del
XH_P04	18	61602442	61602442	Missense_Mutation	SNV	C	G	G	SERPINB10	c.1160C>G	P.T387S
XH_P04	19	22157509	22157509	Missense_Mutation	SNV	C	A	A	ZNF208	c.327G>T	P.E109D
XH_P04	19	52569420	52569420	Missense_Mutation	SNV	T	C	C	ZNF841	c.1715A>G	P.N572S
XH_P04	2	155711682	155711682	Missense_Mutation	SNV	A	G	G	KCNJ3	c.1363A>G	P.T455A
XH_P04	2	1796166	1796166	Missense_Mutation	SNV	G	T	T	MYT1L	c.3347C>A	P.S1116Y
XH_P04	2	55402952	55402955	Frame_Shift_Del	INDEL	TGAC	-	-	CLHC1	366_1369delGT	P.V456fs
XH_P04	2	74688843	74688844	Frame_Shift_Del	INDEL	AT	-	-	MOGS	.1754_1755delA	P.Y585fs
XH_P04	20	35532493	35532567	Frame_Shift_Del	INDEL	TATTCACTCA	-	-	SAMHD1	GTAATAAACTGA	P.I499fs
XH_P04	20	60989462	60989462	Silent	SNV	G	A	A	RBBP8NL	c.945C>T	P.S315S
XH_P04	3	52437595	52437596	Frame_Shift_Del	INDEL	AG	-	-	BAP1	.1565_1566del(	P.P522fs
XH_P04	4	106180824	106180826	Missense_Mutation	INDEL	CTT	-	-	TET2	3854_3856delT	P.F1285del
XH_P04	4	13604703	13604703	Missense_Mutation	SNV	T	A	A	BOD1L1	c.3821A>T	P.H1274L
XH_P04	4	153259068	153259068	Silent	SNV	T	C	C	FBXW7	c.747A>G	P.K249K
XH_P04	4	187524609	187524609	Missense_Mutation	SNV	G	A	A	FAT1	c.11071C>T	P.H3691Y
XH_P04	5	176518670	176518673	Splice_Site	INDEL	CTCT	-	-	FGFR4	)4-12_604-9delCTCT	
XH_P04	6	144869774	144869774	Splice_Site	SNV	A	T	T	UTRN	c.6596-2A>T	
XH_P04	6	152668244	152668244	Missense_Mutation	SNV	G	T	T	SYNE1	c.11815C>A	P.L3939M
XH_P04	6	25435814	25435814	Missense_Mutation	SNV	T	A	A	CARMIL1	c.353T>A	P.F118Y
XH_P04	6	35392280	35392280	Missense_Mutation	SNV	C	T	T	PPARD	c.802C>T	P.P268S
XH_P04	6	46656550	46656550	Nonsense_Mutation	SNV	C	T	T	TDRD6	c.685C>T	P.R229*
XH_P04	7	122338518	122338520	Missense_Mutation	INDEL	ACA	-	-	RNF133	.453_455delTG	P.V152del
XH_P04	7	134891878	134891878	Silent	SNV	A	C	C	WDR91	c.588T>G	P.R196R
XH_P04	7	40535940	40535940	Silent	SNV	G	A	A	SUGCT	c.1065G>A	P.P355P
XH_P04	7	47832299	47832299	Missense_Mutation	SNV	T	C	C	PKD1L1	c.8452A>G	P.T2818A
XH_P04	7	99489911	99489913	Missense_Mutation	INDEL	AGA	-	-	TRIM4	1376_1378delT(	P.F459del
XH_P04	8	145170488	145170488	Missense_Mutation	SNV	C	T	T	WDR97	c.4322C>T	P.T1441M
XH_P04	8	37555654	37555654	Missense_Mutation	SNV	G	A	A	ZNF703	c.1235G>A	P.G412E
XH_P04	8	53062362	53062362	Missense_Mutation	SNV	C	T	T	ST18	c.1982G>A	P.C661Y

XH_P04	9	127262988	127262988	Missense_Mutation	SNV	C	T	T	NR5A1	c.251G>A	P.R84H
XH_P04	9	35684796	35684798	Missense_Mutation	INDEL	CCA	-	-	TPM2	.570_572delTGC	190_G191delinsW
XH_P04	X	105875919	105875919	Silent	SNV	A	G	G	CXorf57	c.1044A>G	P.K348K
XH_P04	X	37655319	37655322	Frame_Shift_Del	INDEL	CTTA	-	-	CYBB	.602_605delACT	P.Y201fs
XH_P05	1	101196794	101196794	Silent	SNV	C	T	T	VCAM1	c.1245C>T	P.L415L
XH_P05	1	158435621	158435621	Silent	SNV	G	A	A	OR10K1	c.270G>A	P.K90K
XH_P05	1	197147562	197147562	Missense_Mutation	SNV	T	C	C	ZBTB41	c.1757A>G	P.H586R
XH_P05	1	204889759	204889759	Splice_Site	SNV	G	A	A	NFASC	c.-199-1G>A	
XH_P05	1	248112802	248112802	Missense_Mutation	SNV	T	C	C	OR2L8	c.643T>C	P.C215R
XH_P05	1	248185319	248185319	Missense_Mutation	SNV	C	A	A	OR2L5	c.70C>A	P.L24I
XH_P05	10	112764539	112764539	Missense_Mutation	SNV	A	G	G	SHOC2	c.1148A>G	P.K383R
XH_P05	10	61834319	61834319	Missense_Mutation	SNV	G	T	T	ANK3	c.6320C>A	P.T2107N
XH_P05	11	108043227	108043227	Missense_Mutation	SNV	C	G	G	NPAT	c.2484G>C	P.Q828H
XH_P05	11	14380346	14380346	Missense_Mutation	SNV	C	T	T	RRAS2	c.71G>A	P.G24D
XH_P05	11	65824404	65824404	Silent	SNV	G	A	A	SF3B2	c.645G>A	P.V215V
XH_P05	11	68846406	68846406	Missense_Mutation	SNV	T	C	C	TPCN2	c.1457T>C	P.L486P
XH_P05	11	95825221	95825221	Silent	SNV	C	T	T	MAML2	c.1974G>A	P.Q658Q
XH_P05	12	111348909	111348909	Missense_Mutation	SNV	A	T	T	MYL2	c.473T>A	P.I158N
XH_P05	12	133208980	133208980	Nonsense_Mutation	SNV	G	T	T	POLE	c.6251C>A	P.S2084*
XH_P05	13	103390725	103390725	Missense_Mutation	SNV	A	G	G	CCDC168	c.12322T>C	P.Y4108H
XH_P05	13	103391555	103391555	Missense_Mutation	SNV	C	T	T	CCDC168	c.11492G>A	P.R3831Q
XH_P05	14	20404251	20404251	Silent	SNV	A	T	T	OR4K1	c.426A>T	P.V142V
XH_P05	15	41099840	41099840	Missense_Mutation	SNV	G	T	T	ZFYVE19	c.53G>T	P.C18F
XH_P05	15	41857274	41857274	Missense_Mutation	SNV	A	T	T	TYRO3	c.718A>T	P.N240Y
XH_P05	15	42930926	42930926	Missense_Mutation	SNV	C	T	T	STARD9	c.475C>T	P.R159W
XH_P05	15	51397322	51397322	Missense_Mutation	SNV	C	A	A	TNFAIP8L3	c.52G>T	P.A18S
XH_P05	15	72432064	72432064	Missense_Mutation	SNV	T	C	C	SENP8	c.100T>C	P.F34L
XH_P05	16	81295871	81295871	Missense_Mutation	SNV	C	A	A	BCO1	c.454C>A	P.L152M
XH_P05	17	39464717	39464717	Silent	SNV	G	A	A	KRTAP16-1	c.789C>T	P.I263I
XH_P05	17	43195485	43195485	Missense_Mutation	SNV	C	A	A	PLCD3	c.1136G>T	P.R379L
XH_P05	17	7577541	7577541	Missense_Mutation	SNV	T	A	A	TP53	c.740A>T	P.N247I
XH_P05	18	29972863	29972863	Missense_Mutation	SNV	T	C	C	GAREM1	c.257A>G	P.Y86C
XH_P05	18	76757121	76757121	Silent	SNV	G	A	A	SALL3	c.3702G>A	P.Q1234Q
XH_P05	19	10610202	10610202	Missense_Mutation	SNV	C	G	G	KEAP1	c.508G>C	P.A170P
XH_P05	19	41858919	41858921	Missense_Mutation	INDEL	GCG	-	-	TGFB1	c.29_31delCGC	P.P10del
XH_P05	19	42703686	42703686	Silent	SNV	C	T	T	DEDD2	c.885G>A	P.L295L
XH_P05	19	47258831	47258831	Missense_Mutation	SNV	G	A	A	FKRP	c.124G>A	P.A42T
XH_P05	19	49514534	49514534	Missense_Mutation	SNV	T	C	C	RUVBL2	c.866T>C	P.V289A
XH_P05	19	53015366	53015366	Missense_Mutation	SNV	C	A	A	ZNF578	c.1732C>A	P.H578N
XH_P05	2	133075404	133075404	Frame_Shift_Del	INDEL	T	-	-	ZNF806	c.866delT	P.F289fs
XH_P05	2	191235749	191235749	Missense_Mutation	SNV	C	T	T	INPP1	c.821C>T	P.A274V
XH_P05	2	232457934	232457934	Missense_Mutation	SNV	G	A	A	C2orf57	c.272G>A	P.S91N
XH_P05	20	55093211	55093211	Missense_Mutation	SNV	G	T	T	RTFDC1	c.901G>T	P.D301Y
XH_P05	20	58440877	58440877	Missense_Mutation	SNV	T	C	C	SYCP2	c.4364A>G	P.K1455R
XH_P05	21	18981391	18981391	Silent	SNV	T	C	C	BTG3	c.72A>G	P.K24K
XH_P05	22	16287712	16287712	Missense_Mutation	SNV	C	A	A	POTEH	c.174G>T	P.R58S
XH_P05	22	16287713	16287713	Missense_Mutation	SNV	C	A	A	POTEH	c.173G>T	P.R58M
XH_P05	3	99513635	99513635	Missense_Mutation	SNV	C	A	A	COL8A1	c.890C>A	P.P297Q
XH_P05	4	6302503	6302503	Silent	SNV	C	T	T	WFS1	c.981C>T	P.L327L
XH_P05	4	72316208	72316208	Missense_Mutation	SNV	G	T	T	SLC4A4	c.1156G>T	P.D386Y
XH_P05	5	1210580	1210580	Missense_Mutation	SNV	C	A	A	SLC6A19	c.365C>A	P.S122Y
XH_P05	5	149375561	149375561	Frame_Shift_Del	INDEL	A	-	-	TIGD6	c.351delT	P.Q118fs
XH_P05	5	31302318	31302318	Missense_Mutation	SNV	G	T	T	CDH6	c.912G>T	P.E304D
XH_P05	5	73165977	73165977	Missense_Mutation	SNV	C	T	T	ARHGEF28	c.2509C>T	P.P837S
XH_P05	5	82834438	82834438	Silent	SNV	G	A	A	VCAN	c.5616G>A	P.P1872P
XH_P05	6	117046997	117046997	Missense_Mutation	SNV	G	T	T	KPNA5	c.1083G>T	P.W361C
XH_P05	6	117923259	117923259	Missense_Mutation	SNV	C	T	T	GOPC	c.193G>A	P.E65K
XH_P05	6	152651958	152651958	Missense_Mutation	SNV	G	T	T	SYNE1	c.13649C>A	P.T4550K
XH_P05	6	32609940	32609940	Missense_Mutation	SNV	A	T	T	HLA-DQA1	c.523A>T	P.S175C
XH_P05	7	149467533	149467533	Nonsense_Mutation	SNV	G	T	T	ZNF467	c.147C>A	P.C49*
XH_P05	7	87080969	87080969	Silent	SNV	A	G	G	ABCB4	c.678T>C	P.I226I
XH_P05	8	116616447	116616447	Silent	SNV	T	A	A	TRPS1	c.1722A>T	P.G574G
XH_P05	8	19679938	19679938	Splice_Site	SNV	G	A	A	INTS10	c.442-12G>A	
XH_P05	8	9437709	9437709	Missense_Mutation	SNV	G	A	A	TNKS	c.714G>A	P.M238I
XH_P05	9	135073987	135073987	Missense_Mutation	SNV	T	C	C	NTNG2	c.848T>C	P.V283A
XH_P05	9	37126350	37126350	Missense_Mutation	SNV	G	T	T	ZCCHC7	c.21G>T	P.E7D
XH_P05	X	150869486	150869486	Missense_Mutation	SNV	A	G	G	PRRG3	c.677A>G	P.N226S
XH_P05	X	18220019	18220019	Nonsense_Mutation	SNV	C	A	A	BEND2	c.949G>T	P.E317*
XH_P05	X	46917987	46917987	Missense_Mutation	SNV	C	A	A	JADE3	c.1980C>A	P.N660K
XH_P05	X	9673133	9673133	Silent	SNV	C	G	G	TBL1X	c.1215C>G	P.V405V
XH_P06	1	203154322	203154322	Missense_Mutation	SNV	G	A	A	CHI3L1	c.247C>T	P.L83F
XH_P06	1	237774081	237774081	Missense_Mutation	SNV	C	T	T	RYR2	c.4703C>T	P.A1568V
XH_P06	10	123279556	123279591	Missense_Mutation	INDEL	ATGTGGGGCT	-	-	FGFR2	ATGCCCAGCCCC	P.Y281_K292del
XH_P06	11	103866971	103866971	Missense_Mutation	SNV	T	A	A	PDGFD	c.332A>T	P.Y111F
XH_P06	11	60668333	60668333	Silent	SNV	G	A	A	PRPF19	c.711C>T	P.I237I
XH_P06	11	62489400	62489400	Silent	SNV	C	G	G	HNRNPUL2	c.1374G>C	P.V458V
XH_P06	11	970204	970204	Missense_Mutation	SNV	T	A	A	AP2A2	c.172T>A	P.Y58N
XH_P06	13	37393812	37393812	Silent	SNV	T	C	C	RFXAP	c.318T>C	P.A106A
XH_P06	13	50587139	50587139	Missense_Mutation	SNV	A	G	G	TRIM13	c.1072A>G	P.S358G
XH_P06	13	52598520	52598520	Silent	SNV	T	C	C	ALG11	c.654T>C	P.N218N
XH_P06	14	24040526	24040526	Nonsense_Mutation	SNV	G	A	A	JPH4	c.1414C>T	P.R472*
XH_P06	17	7605866	7605866	Missense_Mutation	SNV	G	A	A	WRAP53	c.1160G>A	P.R387H
XH_P06	19	39898700	39898700	Missense_Mutation	SNV	G	T	T	ZFP36	c.360G>T	P.E120D
XH_P06	19	56320517	56320517	Missense_Mutation	SNV	C	A	A	NLRP11	c.1162G>T	P.D388Y
XH_P06	20	34828358	34828358	Missense_Mutation	SNV	G	A	A	AAR2	c.568G>A	P.E190K
XH_P06	3	52441246	52441246	Missense_Mutation	SNV	G	A	A	BAP1	c.524C>T	P.P175L
XH_P06	4	70462079	70462079	Missense_Mutation	SNV	C	G	G	UGT2A1	c.912G>C	P.Q304H
XH_P06	5	140744110	140744110	Missense_Mutation	SNV	G	T	T	PCDHGA5	c.213G>T	P.R71S
XH_P06	5	140779426	140779426	Missense_Mutation	SNV	G	A	A	PCDHGB5	c.1732G>A	P.A578T
XH_P06	6	20124649	20124649	Silent	SNV	T	C	C	MBOAT1	c.897A>G	P.A299A
XH_P06	6	55684654	55684654	Splice_Site	SNV	A	G	G	BMP5	c.491-9T>C	
XH_P06	6	65622479	65622479	Missense_Mutation	SNV	G	C	C	EYS	c.2539C>G	P.H847D
XH_P06	9	135779187	135779187	Nonsense_Mutation	SNV	C	A	A	TSC1	c.2059G>T	P.E687*
XH_P06	9	139744560	139744560	Frame_Shift_Ins	INDEL	-	AG	AG	PHPT1	c.258_259dupGA	P.K87fs
XH_P06	X	109697368	109697368	Missense_Mutation	SNV	C	A	A	RGAG1	c.3523C>A	P.Q1175K
XH_P06	X	125685479	125685479	Silent	SNV	G	A	A	DCAF12L1	c.1113C>T	P.S371S
XH_P06	X	83389787	83389787	Splice_Site	SNV	T	C	C	RPS6KA6	c.646+3A>G	
XH_P07	1	16474948	16474948	Frame_Shift_Ins	INDEL	-	AGTG	AGTG	EPHA2	.747_748insCAC	P.D250fs

XH_P07	1	24447730	24447730	Silent	SNV	C	T	T	IL22RA1	c.1290G>A	P.Q430Q
XH_P07	1	35580033	35580033	Missense_Mutation	SNV	G	T	T	ZMYM1	c.2602G>T	P.V868L
XH_P07	1	8074115	8074115	Frame_Shift_Del	INDEL	G	-	-	ERRFI1	c.544delC	P.L182fs
XH_P07	10	118306822	118306822	Nonsense_Mutation	SNV	C	A	A	PNLIP	c.63C>A	P.Y21*
XH_P07	11	32635525	32635525	Missense_Mutation	SNV	T	C	C	CCDC73	c.2339A>G	P.N780S
XH_P07	11	61498848	61498848	Silent	SNV	C	T	T	DAGLA	c.909C>T	P.Y303Y
XH_P07	13	28124466	28124474	Frame_Shift_Del	INDEL	TCACTTTAA	-	-	LNX2	_1937+4delTTAA	P.L645fs
XH_P07	16	88706365	88706365	Missense_Mutation	SNV	G	T	T	IL17C	c.479G>T	P.R160L
XH_P07	17	76704300	76704300	Frame_Shift_Del	INDEL	T	-	-	CYTH1	c.149delA	P.N50fs
XH_P07	17	76993313	76993313	Missense_Mutation	SNV	T	C	C	CANT1	c.392A>G	P.K131R
XH_P07	17	78617539	78617539	Missense_Mutation	SNV	A	T	T	RPTOR	c.277A>T	P.M93L
XH_P07	19	54672371	54672371	Silent	SNV	G	A	A	TMC4	c.496C>T	P.L166L
XH_P07	19	8167683	8167683	Missense_Mutation	SNV	T	C	C	FBN3	c.5014A>G	P.K1672E
XH_P07	2	166535267	166535267	Silent	SNV	A	G	G	CSRNP3	c.762A>G	P.A254A
XH_P07	2	1926644	1926644	Silent	SNV	G	A	A	MYT1L	c.897C>T	P.Y299Y
XH_P07	2	209113113	209113113	Missense_Mutation	SNV	G	A	A	IDH1	c.394C>T	P.R132C
XH_P07	3	13417789	13417789	Splice_Site	SNV	G	C	C	NUP210	c.1293+2C>G	
XH_P07	3	183962425	183962425	Silent	SNV	C	A	A	ALG3	c.546G>T	P.G182G
XH_P07	3	52588739	52588739	Splice_Site	INDEL	C	-	-	PBRM1	c.4288+1delG	
XH_P07	6	36070442	36070442	Missense_Mutation	SNV	A	C	C	MAPK14	c.857A>C	P.Y286S
XH_P07	6	53387336	53387336	Splice_Site	INDEL	-	A	A	GCLC	c.151-12dupT	
XH_P07	7	45701692	45701692	Missense_Mutation	SNV	C	T	T	ADCY1	c.809C>T	P.P270L
XH_P07	7	73449745	73449745	Splice_Site	SNV	G	T	T	ELN	c.133+1G>T	
XH_P07	7	74105444	74105444	Splice_Site	INDEL	G	-	-	GTF2I	c.238+1delG	
XH_P07	9	32974548	32974548	Missense_Mutation	SNV	A	T	T	APTX	c.824T>A	P.L275H
XH_P07	X	107400378	107400378	Missense_Mutation	SNV	C	A	A	COL4A6	c.4976G>T	P.C1659F
XH_P08	1	10238810	10238810	Nonsense_Mutation	SNV	G	T	T	UBE4B	c.3634G>T	P.E1212*
XH_P08	1	115256529	115256529	Missense_Mutation	SNV	T	C	C	NRAS	c.182A>G	P.Q61R
XH_P08	1	150199097	150199097	Missense_Mutation	SNV	T	A	A	ANP32E	c.401A>T	P.E134V
XH_P08	1	247013661	247013661	Missense_Mutation	SNV	C	T	T	AHCTF1	c.5647G>A	P.E1883K
XH_P08	1	27100927	27100927	Missense_Mutation	SNV	G	T	T	ARID1A	c.4209G>T	P.Q1403H
XH_P08	10	14885378	14885378	Missense_Mutation	SNV	C	T	T	HSPA14	c.230C>T	P.S77L
XH_P08	11	10792125	10792125	Missense_Mutation	SNV	A	G	G	CTR9	c.2318A>G	P.K773R
XH_P08	11	726294	726294	Splice_Site	INDEL	-	C	C	EPS8L2	c.1754-5dupC	
XH_P08	12	416963	416963	Missense_Mutation	SNV	G	A	A	KDM5A	c.3587C>T	P.S1196F
XH_P08	14	24601533	24601533	Missense_Mutation	SNV	G	A	A	FITM1	c.380G>A	P.R127Q
XH_P08	16	2162340	2162340	Splice_Site	SNV	C	A	A	PKD1	c.3295+1G>T	
XH_P08	16	30724886	30724886	Missense_Mutation	SNV	A	T	T	SRCAP	c.2347A>T	P.M783L
XH_P08	18	52928759	52928759	Missense_Mutation	SNV	T	C	C	TCF4	c.928A>G	P.R310G
XH_P08	19	15852665	15852665	Missense_Mutation	SNV	G	A	A	OR10H3	c.463G>A	P.V155I
XH_P08	2	158115066	158115066	Missense_Mutation	SNV	G	T	T	GALNT5	c.472G>T	P.G158W
XH_P08	2	1652604	1652604	Missense_Mutation	SNV	C	T	T	PXDN	c.2948G>A	P.G983D
XH_P08	2	29234310	29234310	Splice_Site	SNV	G	A	A	FAM179A	c.831-11G>A	
XH_P08	20	45242138	45242138	Missense_Mutation	SNV	G	A	A	SLC13A3	c.197C>T	P.A66V
XH_P08	3	121207626	121207626	Silent	SNV	C	T	T	POLQ	c.4152G>A	P.A1384A
XH_P08	3	140678331	140678331	Missense_Mutation	SNV	G	T	T	SLC25A36	c.233G>T	P.R78L
XH_P08	4	25418083	25418083	Missense_Mutation	SNV	T	G	G	ANAPC4	c.1941T>G	P.D647E
XH_P08	5	67576346	67576346	Splice_Site	SNV	A	T	T	PIK3R1	c.635-10A>T	
XH_P08	6	143081215	143081215	Silent	SNV	T	C	C	HIVEP2	c.6210A>G	P.L2070L
XH_P08	7	116380114	116380114	Nonsense_Mutation	SNV	C	A	A	MET	c.1503C>A	P.Y501*
XH_P08	8	145739921	145739921	Splice_Site	SNV	G	A	A	RECQL4	c.1621-12C>T	
XH_P08	8	55538282	55538282	Missense_Mutation	SNV	C	A	A	RP1	c.1840C>A	P.H614N
XH_P08	9	2039777	2039782	Missense_Mutation	INDEL	CAGCAG	-	-	SMARCA2	02_707delGCAG	.Q235_Q236del
XH_P08	X	75649221	75649221	Missense_Mutation	SNV	G	A	A	MAGEE1	c.898G>A	P.A300T
XH_P08	X	79932611	79932611	Missense_Mutation	SNV	C	T	T	BRWD3	c.4906G>A	P.V1636I
XH_P09	1	115218197	115218197	Missense_Mutation	SNV	C	T	T	AMPD1	c.1732G>A	P.A578T
XH_P09	1	16458604	16458637	Frame_Shift_Del	INDEL	AGACACCTTG	-	-	EPHA2	TGGTCTGCAAGG	P.N750fs
XH_P09	10	95518532	95518532	Silent	SNV	T	C	C	LGI1	c.231T>C	P.S77S
XH_P09	11	34680381	34680381	Missense_Mutation	SNV	A	G	G	EHF	c.737A>G	P.Y246C
XH_P09	13	103301837	103301837	Splice_Site	INDEL	G	-	-	TPP2	c.2952+1delG	
XH_P09	13	61985922	61985922	Silent	SNV	G	A	A	PCDH20	c.2310C>T	P.V770V
XH_P09	14	21359882	21359882	Missense_Mutation	SNV	C	T	T	RNASE3	c.37C>T	P.L13F
XH_P09	14	36988440	36988440	Nonsense_Mutation	SNV	G	T	T	NKX2-1	c.123C>A	P.Y41*
XH_P09	14	74407706	74407706	Silent	SNV	G	A	A	FAM161B	c.1512C>T	P.S504S
XH_P09	17	26732433	26732433	Silent	SNV	G	A	A	SLC46A1	c.282C>T	P.F94F
XH_P09	17	42949912	42949912	Missense_Mutation	SNV	A	G	G	EFTUD2	c.791T>C	P.I264T
XH_P09	17	7906389	7906389	Silent	SNV	G	A	A	GUCY2D	c.24G>A	P.A8A
XH_P09	17	80210924	80210924	Missense_Mutation	SNV	C	T	T	CSNK1D	c.533G>A	P.R178Q
XH_P09	18	67614640	67614640	Nonsense_Mutation	SNV	C	A	A	CD226	c.3G>T	P.M1?
XH_P09	19	6678196	6678196	Missense_Mutation	SNV	C	A	A	C3	c.4817G>T	P.G1606V
XH_P09	19	7976189	7976189	Missense_Mutation	SNV	G	A	A	MAP2K7	c.958G>A	P.D320N
XH_P09	2	138413101	138413101	Missense_Mutation	SNV	C	T	T	THSD7B	c.3976C>T	P.R1326C
XH_P09	2	15618381	15618381	Missense_Mutation	SNV	C	G	G	NBAS	c.1116G>C	P.R372S
XH_P09	21	19713841	19713841	Missense_Mutation	SNV	T	C	C	TMPRSS15	c.1453A>G	P.K485E
XH_P09	22	21340177	21340177	Missense_Mutation	SNV	C	A	A	LZTR1	c.311C>A	P.S104Y
XH_P09	3	10357042	10357042	Missense_Mutation	SNV	G	A	A	SEC13	c.265C>T	P.R89C
XH_P09	3	156396253	156396253	Frame_Shift_Del	INDEL	A	-	-	TIPARP	c.767delA	P.D256fs
XH_P09	3	49739838	49739838	Splice_Site	SNV	G	C	C	RNF123	c.1629G>C	P.G543G
XH_P09	3	66436487	66436487	Silent	SNV	G	A	A	LRIG1	c.1707C>T	P.F569F
XH_P09	5	37057308	37057308	Silent	SNV	C	A	A	NIPBL	c.7284C>A	P.L2428L
XH_P09	6	105609626	105609626	Silent	SNV	G	T	T	POPDC3	c.159C>A	P.V53V
XH_P09	6	129465137	129465137	Missense_Mutation	SNV	G	A	A	LAMA2	c.731G>A	P.R244K
XH_P09	6	131211522	131211522	Silent	SNV	C	T	T	EPB41L2	c.1572G>A	P.Q524Q
XH_P09	6	151130273	151130273	Frame_Shift_Ins	INDEL	-	CCAGA	CCAGA	PLEKHG1	948_952dupAGA	P.L318fs
XH_P09	6	34857302	34857302	Frame_Shift_Ins	INDEL	-	GGCGGC	GGCGGC	ANKS1A	33_138dupGGC	P.G45_G46dup
XH_P09	6	3751754	3751754	Silent	SNV	C	A	A	PXDC1	c.12G>T	P.A4A
XH_P09	6	47846407	47846407	Missense_Mutation	SNV	C	T	T	PTCHD4	c.2173G>A	P.A725T
XH_P09	6	6152165	6152165	Silent	SNV	T	C	C	F13A1	c.1926A>G	P.V642V
XH_P09	7	117365321	117365321	Splice_Site	SNV	G	A	A	CTTNBP2	c.4056-10C>T	
XH_P09	8	113392682	113392682	Missense_Mutation	SNV	G	T	T	CSMD3	c.5723C>A	P.T1908K
XH_P09	8	133899201	133899201	Silent	SNV	A	G	G	TG	c.1584A>G	P.S528S
XH_P09	8	145154262	145154262	Missense_Mutation	SNV	C	G	G	SHARPIN	c.840G>C	P.E280D
XH_P09	X	38262901	38262901	Missense_Mutation	SNV	C	T	T	OTC	c.571C>T	P.L191F
XH_P10	1	110085668	110085668	Missense_Mutation	SNV	G	C	C	GPR61	c.24G>C	P.Q8H
XH_P10	1	115456674	115456674	Splice_Site	SNV	T	A	A	SYCP1	c.1718+8T>A	
XH_P10	1	120384101	120384101	Missense_Mutation	SNV	T	C	C	NBPF7	c.461A>G	P.D154G
XH_P10	1	152276716	152276716	Nonsense_Mutation	SNV	G	T	T	FLG	c.10646C>A	P.S3549*
XH_P10	1	153614832	153614832	Missense_Mutation	SNV	A	T	T	CHTOP	c.333A>T	P.R111S

XH_P10	1	161693401	161693401	Silent	SNV	T	A	A	FCRLB	c.297T>A	P.S99S
XH_P10	1	182636106	182636106	Missense_Mutation	SNV	T	A	A	RGS8	c.29A>T	P.N10I
XH_P10	1	184701982	184701982	Missense_Mutation	SNV	G	C	C	EDEM3	c.601C>G	P.P201A
XH_P10	1	201983028	201983028	Missense_Mutation	SNV	A	T	T	ELF3	c.877A>T	P.M293L
XH_P10	1	205132039	205132039	Splice_Site	SNV	A	G	G	DSTYK	c.1641+12T>C	
XH_P10	1	215802332	215802332	Missense_Mutation	SNV	T	C	C	USH2A	c.15343A>G	P.I5115V
XH_P10	1	223284802	223284802	Missense_Mutation	SNV	A	T	T	TLR5	c.1572T>A	P.H524Q
XH_P10	1	227170471	227170471	Splice_Site	SNV	A	G	G	COQ8A	c.939+7A>G	
XH_P10	1	236371346	236371346	Splice_Site	SNV	T	C	C	GPR137B	c.1092-4T>C	
XH_P10	1	236966910	236966910	Missense_Mutation	SNV	A	T	T	MTR	c.217A>T	P.T73S
XH_P10	1	248112618	248112618	Silent	SNV	C	A	A	OR2L8	c.459C>A	P.I153I
XH_P10	1	2529644	2529644	Splice_Site	SNV	A	G	G	MMEL1	c.1272+2T>C	
XH_P10	1	4832433	4832433	Silent	SNV	G	A	A	AJAP1	c.1011G>A	P.S337S
XH_P10	1	89426907	89426907	Missense_Mutation	SNV	T	C	C	KYAT3	c.730A>G	P.I244V
XH_P10	1	9323763	9323763	Missense_Mutation	SNV	A	G	G	H6PD	c.1244A>G	P.H415R
XH_P10	10	103899315	103899315	Silent	SNV	G	T	T	PPRC1	c.1050G>T	P.G350G
XH_P10	10	120445656	120445656	Missense_Mutation	SNV	G	A	A	CACUL1	c.1094C>T	P.S365F
XH_P10	10	15614215	15614215	Missense_Mutation	SNV	T	A	A	ITGA8	c.2587A>T	P.I863L
XH_P10	10	16960680	16960680	Missense_Mutation	SNV	A	G	G	CUBN	c.6941T>C	P.L2314S
XH_P10	10	17087135	17087135	Silent	SNV	G	A	A	CUBN	c.3543C>T	P.Y1181Y
XH_P10	11	113092057	113092057	Splice_Site	SNV	A	G	G	NCAM1	c.1089+10A>G	
XH_P10	11	14515222	14515222	Silent	SNV	T	G	G	COPB1	c.457A>C	P.R153R
XH_P10	11	57995931	57995931	Silent	SNV	G	A	A	OR10Q1	c.417C>T	P.I139I
XH_P10	11	68696655	68696655	Silent	SNV	G	T	T	IGHMBP2	c.1065G>T	P.A355A
XH_P10	11	8476297	8476297	Missense_Mutation	SNV	G	C	C	STK33	c.657C>G	P.N219K
XH_P10	11	94704658	94704658	Splice_Site	SNV	T	C	C	CWC15	c.132-7A>G	
XH_P10	12	119594505	119594505	Missense_Mutation	SNV	C	T	T	SRRM4	c.1738C>T	P.R580C
XH_P10	12	120574327	120574327	Silent	SNV	T	C	C	GCN1	c.6987A>G	P.T2329T
XH_P10	12	124887114	124887114	Splice_Site	SNV	G	C	C	NCOR2	c.1480-7C>G	
XH_P10	12	133254232	133254232	Missense_Mutation	SNV	T	A	A	POLE	c.652A>T	P.I218F
XH_P10	12	14591213	14591213	Splice_Site	SNV	G	A	A	ATF7IP	c.1926+12G>A	
XH_P10	12	20893226	20893226	Missense_Mutation	SNV	T	C	C	SLCO1C1	c.1303T>C	P.Y435H
XH_P10	12	250375	250375	Silent	SNV	C	A	A	IQSEC3	c.2077C>A	P.R693R
XH_P10	12	48737351	48737351	Frame_Shift_Del	INDEL	G	-	-	ZNF641	c.680delC	P.P227fs
XH_P10	12	54932678	54932678	Missense_Mutation	SNV	T	A	A	NCKAP1L	c.3044T>A	P.V1015D
XH_P10	12	5674825	5674825	Nonsense_Mutation	SNV	T	A	A	ANO2	c.2641A>T	P.K881*
XH_P10	12	62932235	62932235	Missense_Mutation	SNV	C	T	T	MON2	c.2314C>T	P.L772F
XH_P10	12	6946165	6946165	Missense_Mutation	SNV	G	A	A	P3H3	c.1472G>A	P.G491E
XH_P10	12	69759596	69759596	Missense_Mutation	SNV	A	G	G	YEATS4	c.265A>G	P.T89A
XH_P10	13	33333804	33333804	Missense_Mutation	SNV	G	C	C	PDS5B	c.3348G>C	P.M1116I
XH_P10	13	38213401	38213401	Missense_Mutation	SNV	T	A	A	TRPC4	c.2115A>T	P.Q705H
XH_P10	14	105174894	105174894	Silent	SNV	G	T	T	INF2	c.1857G>T	P.V619V
XH_P10	14	105615377	105615377	Silent	SNV	T	A	A	JAG2	c.1803A>T	P.A601A
XH_P10	14	31819042	31819042	Silent	SNV	T	A	A	HEATR5A	c.2661A>T	P.V887V
XH_P10	14	55462343	55462343	Silent	SNV	T	A	A	WDHD1	c.762A>T	P.L254L
XH_P10	14	64194406	64194406	Missense_Mutation	SNV	G	A	A	SGPP1	c.257C>T	P.P86L
XH_P10	14	78365505	78365505	Missense_Mutation	SNV	A	C	C	ADCK1	c.441A>C	P.E147D
XH_P10	14	94756345	94756345	Missense_Mutation	SNV	G	A	A	SERPINA10	c.586C>T	P.R196C
XH_P10	15	31362097	31362097	Missense_Mutation	SNV	T	C	C	TRPM1	c.350A>G	P.Q117R
XH_P10	15	43269043	43269043	Missense_Mutation	SNV	G	A	A	UBR1	c.4241C>T	P.P1414L
XH_P10	15	51687131	51687131	Missense_Mutation	SNV	G	C	C	GLDN	c.641G>C	P.G214A
XH_P10	15	64015465	64015465	Missense_Mutation	SNV	C	G	G	HERC1	c.3658G>C	P.V1220L
XH_P10	15	68599767	68599767	Frame_Shift_Del	INDEL	G	-	-	ITGA11	c.3300delC	P.M1101fs
XH_P10	15	73075906	73075906	Missense_Mutation	SNV	G	A	A	ADPGK	c.127C>T	P.P43S
XH_P10	15	74327873	74327873	Missense_Mutation	SNV	G	A	A	PML	c.2071G>A	P.V691I
XH_P10	15	92459396	92459396	Frame_Shift_Del	INDEL	G	-	-	SLCO3A1	c.356delG	P.G119fs
XH_P10	16	25251928	25251928	Nonsense_Mutation	SNV	T	A	A	ZKSCAN2	c.2113A>T	P.K705*
XH_P10	16	31447482	31447482	Missense_Mutation	SNV	T	A	A	ZNF843	c.689A>T	P.Q230L
XH_P10	16	47497784	47497784	Splice_Site	SNV	A	G	G	PHKB	c.-57-9A>G	
XH_P10	16	57690174	57690174	Missense_Mutation	SNV	G	T	T	ADGRG1	c.1054G>T	P.D352Y
XH_P10	16	62055223	62055223	Missense_Mutation	SNV	T	A	A	CDH8	c.85A>T	P.M29L
XH_P10	16	84188387	84188387	Silent	SNV	G	A	A	DNAAF1	c.558G>A	P.K186K
XH_P10	17	10210383	10210383	Splice_Site	SNV	T	A	A	MYH13	c.5170-2A>T	
XH_P10	17	11845672	11845672	Missense_Mutation	SNV	T	A	A	DNAH9	c.12713T>A	P.I4238N
XH_P10	17	29622292	29622292	Missense_Mutation	SNV	A	G	G	OMG	c.1058T>C	P.L353P
XH_P10	17	35736272	35736272	Silent	SNV	C	T	T	C17orf78	c.343C>T	P.L115L
XH_P10	17	42432372	42432372	Missense_Mutation	SNV	T	A	A	FAM171A2	c.1210A>T	P.S404C
XH_P10	17	45043999	45043999	Missense_Mutation	SNV	G	T	T	GOSR2	c.683G>T	P.S228I
XH_P10	17	56296840	56296840	Silent	SNV	G	A	A	MKS1	c.33C>T	P.R11R
XH_P10	17	62499104	62499104	Missense_Mutation	SNV	C	T	T	DDX5	c.923G>A	P.S308N
XH_P10	17	66347754	66347754	Missense_Mutation	SNV	A	T	T	ARSG	c.493A>T	P.M165L
XH_P10	17	7578176	7578176	Splice_Site	SNV	C	T	T	TP53	c.672+1G>A	
XH_P10	17	76498683	76498683	Missense_Mutation	SNV	T	C	C	DNAH17	c.5180A>G	P.N1727S
XH_P10	17	79254487	79254487	Missense_Mutation	SNV	A	C	C	SLC38A10	c.548T>G	P.L183R
XH_P10	17	79254488	79254488	Missense_Mutation	SNV	G	C	C	SLC38A10	c.547C>G	P.L183V
XH_P10	18	10770301	10770301	Missense_Mutation	SNV	T	A	A	PIEZO2	c.2716A>T	P.R906W
XH_P10	18	30257200	30257200	Missense_Mutation	SNV	C	A	A	KLHL14	c.1682G>T	P.S561I
XH_P10	18	47390570	47390570	Missense_Mutation	SNV	G	C	C	MYO5B	c.3784C>G	P.L1262V
XH_P10	18	48255980	48255980	Missense_Mutation	SNV	C	G	G	MAPK4	c.887C>G	P.A296G
XH_P10	18	67741193	67741193	Silent	SNV	A	T	T	RTTN	c.1866T>A	P.S622S
XH_P10	18	77728085	77728085	Splice_Site	SNV	G	T	T	HSBP1L1	c.119-4G>T	
XH_P10	19	10397892	10397892	Silent	SNV	T	C	C	ICAM4	c.204T>C	P.N68N
XH_P10	19	12978664	12978664	Silent	SNV	C	T	T	MAST1	c.2439C>T	P.D813D
XH_P10	19	16003156	16003156	Missense_Mutation	SNV	T	A	A	CYP4F2	c.488A>T	P.Y163F
XH_P10	19	35434352	35434352	Missense_Mutation	SNV	A	G	G	ZNF30	c.485A>G	P.H162R
XH_P10	19	36505084	36505084	Missense_Mutation	SNV	C	A	A	ALKBH6	c.52G>T	P.G18C
XH_P10	19	36507099	36507099	Nonsense_Mutation	SNV	G	T	T	CLIP3	c.1605C>A	P.C535*
XH_P10	19	3831759	3831759	Missense_Mutation	SNV	T	A	A	ZFR2	c.497A>T	P.Y166F
XH_P10	19	4409468	4409468	Silent	SNV	T	C	C	CHAF1A	c.672T>C	P.S224S
XH_P10	19	45667416	45667416	Splice_Site	SNV	C	A	A	TRAPPC6A	c.354+6G>T	
XH_P10	19	50209346	50209346	Missense_Mutation	SNV	T	G	G	CPT1C	c.1112T>G	P.L371R
XH_P10	19	55340876	55340876	Missense_Mutation	SNV	T	A	A	KIR3DL1	c.1061T>A	P.L354H
XH_P10	19	56203380	56203380	Silent	SNV	A	T	T	EPN1	c.1281A>T	P.A427A
XH_P10	19	58500056	58500056	Missense_Mutation	SNV	A	T	T	ZNF606	c.211T>A	P.F71I
XH_P10	2	122106163	122106163	Silent	SNV	C	T	T	CLASP1	c.4161G>A	P.Q1387Q
XH_P10	2	128522477	128522477	Missense_Mutation	SNV	T	A	A	WDR33	c.551A>T	P.Y184F
XH_P10	2	157186393	157186393	Silent	SNV	T	C	C	NR4A2	c.306A>G	P.Q102Q
XH_P10	2	175292572	175292572	Missense_Mutation	SNV	A	C	C	SCRN3	c.1203A>C	P.K401N

XH_P10	2	196825613	196825613	Silent	SNV	A	T	T	DNAH7	c.2262T>A	P.P754P
XH_P10	2	20840859	20840859	Missense_Mutation	SNV	G	C	C	HS1BP3	c.280C>G	P.P94A
XH_P10	2	238267714	238267714	Silent	SNV	A	T	T	COL6A3	c.6372T>A	P.P2124P
XH_P10	2	39931321	39931321	Missense_Mutation	SNV	G	C	C	TMEM178A	c.501G>C	P.E167D
XH_P10	2	46739679	46739679	Missense_Mutation	SNV	C	G	G	ATP6V1E2	c.172G>C	P.E58Q
XH_P10	2	61605547	61605547	Missense_Mutation	SNV	T	C	C	USP34	c.1027A>G	P.T343A
XH_P10	2	98906978	98906978	Missense_Mutation	SNV	A	T	T	VWA3B	c.3050A>T	P.Q1017L
XH_P10	20	20505548	20505548	Splice_Site	SNV	T	A	A	RALGAPA2	c.3694-6A>T	
XH_P10	20	36784376	36784376	Silent	SNV	C	A	A	TGM2	c.306G>T	P.L102L
XH_P10	20	57599042	57599042	Missense_Mutation	SNV	T	A	A	TUBB1	c.560T>A	P.L187Q
XH_P10	21	11049611	11049611	Missense_Mutation	SNV	A	G	G	BAGE2	c.290T>C	P.M97T
XH_P10	22	18095623	18095623	Silent	SNV	A	G	G	ATP6V1E1	c.165T>C	P.N55N
XH_P10	22	18387485	18387485	Missense_Mutation	SNV	G	C	C	MICAL3	c.385C>G	P.L129V
XH_P10	22	19484972	19484972	Splice_Site	SNV	T	C	C	CDC45	c.749+2T>C	
XH_P10	22	19484974	19484974	Splice_Site	SNV	C	T	T	CDC45	c.749+4C>T	
XH_P10	22	33245528	33245528	Splice_Site	INDEL	A	-	-	TIMP3	c.204+8delA	
XH_P10	22	36902196	36902196	Missense_Mutation	SNV	C	T	T	FOXRED2	c.274G>A	P.E92K
XH_P10	22	45972975	45972975	Missense_Mutation	SNV	C	G	G	FBLN1	c.1959C>G	P.D653E
XH_P10	3	145791075	145791075	Missense_Mutation	SNV	T	A	A	PLOD2	c.1679A>T	P.Q560L
XH_P10	3	14964552	14964552	Splice_Site	SNV	G	A	A	FGD5	c.3808-1G>A	
XH_P10	3	164786622	164786622	Splice_Site	SNV	T	A	A	SI	c.374-3A>T	
XH_P10	3	180324162	180324162	Splice_Site	SNV	T	C	C	TTC14	c.1049+2T>C	
XH_P10	3	189691695	189691695	Splice_Site	SNV	T	A	A	P3H2	c.1548+3A>T	
XH_P10	3	195497151	195497151	Missense_Mutation	SNV	G	A	A	MUC4	c.11117C>T	P.A3706V
XH_P10	3	38833611	38833611	Missense_Mutation	SNV	T	A	A	SCN10A	c.319A>T	P.T107S
XH_P10	3	42261087	42261087	Missense_Mutation	SNV	A	C	C	TRAK1	c.2065A>C	P.S689R
XH_P10	3	44402962	44402962	Missense_Mutation	SNV	G	A	A	TCAIM	c.271G>A	P.E91K
XH_P10	3	44879864	44879864	Missense_Mutation	SNV	A	T	T	KIF15	c.3269A>T	P.Q1090L
XH_P10	3	58398686	58398686	Silent	SNV	A	G	G	PXK	c.1470A>G	P.T490T
XH_P10	3	71823611	71823611	Silent	SNV	G	A	A	PROK2	c.270C>T	P.S90S
XH_P10	4	38830955	38830955	Frame_Shift_Del	INDEL	G	-	-	TLR6	c.140delC	P.P47fs
XH_P10	4	668024	668024	Missense_Mutation	SNV	C	T	T	ATP5I	c.13G>A	P.V5M
XH_P10	4	72994513	72994513	Missense_Mutation	SNV	A	G	G	NPFFR2	c.214A>G	P.I72V
XH_P10	4	80328611	80328611	Silent	SNV	C	A	A	GK2	c.744G>T	P.L248L
XH_P10	5	11199654	11199654	Missense_Mutation	SNV	A	T	T	CTNND2	c.1608T>A	P.D536E
XH_P10	5	113740320	113740320	Silent	SNV	T	C	C	KCNN2	c.768T>C	P.I256I
XH_P10	5	140175455	140175455	Silent	SNV	C	T	T	PCDHA2	c.906C>T	P.I302I
XH_P10	5	140777949	140777949	Silent	SNV	T	A	A	PCDHGB5	c.255T>A	P.L85L
XH_P10	5	156907988	156907988	Missense_Mutation	SNV	C	G	G	ADAM19	c.2726G>C	P.R909T
XH_P10	5	156929868	156929868	Silent	SNV	C	G	G	ADAM19	c.1251G>C	P.R417R
XH_P10	5	23523476	23523476	Splice_Site	SNV	A	G	G	PRDM9	c.950+9A>G	
XH_P10	5	95084136	95084136	Missense_Mutation	SNV	A	T	T	RHOBTB3	c.515A>T	P.Y172F
XH_P10	6	108882753	108882753	Silent	SNV	C	G	G	FOXO3	c.342C>G	P.T114T
XH_P10	6	117700231	117700231	Missense_Mutation	SNV	C	A	A	ROS1	c.2588G>T	P.R863L
XH_P10	6	12125566	12125566	Missense_Mutation	SNV	T	G	G	HIVEP1	c.5538T>G	P.F1846L
XH_P10	6	130497044	130497044	Missense_Mutation	SNV	T	C	C	SAMD3	c.764A>G	P.Q255R
XH_P10	6	132910082	132910082	Missense_Mutation	SNV	T	A	A	TAAR5	c.744A>T	P.R248S
XH_P10	6	161653213	161653213	Silent	SNV	G	A	A	AGPAT4	c.33C>T	P.F11F
XH_P10	6	166720756	166720756	Missense_Mutation	SNV	A	C	C	PRR18	c.875T>G	P.L292R
XH_P10	6	25862217	25862217	Missense_Mutation	SNV	A	G	G	SLC17A3	c.344T>C	P.I115T
XH_P10	6	43030851	43030851	Missense_Mutation	SNV	A	T	T	KLC4	c.455A>T	P.Q152L
XH_P10	6	4999080	4999080	Splice_Site	SNV	A	G	G	RPP40	c.365-5T>C	
XH_P10	6	97001277	97001277	Silent	SNV	A	G	G	UFL1	c.2283A>G	P.Q761Q
XH_P10	6	99365567	99365567	Missense_Mutation	SNV	T	C	C	FBXL4	c.541A>G	P.T181A
XH_P10	7	102016347	102016347	Silent	SNV	A	C	C	LOC100289561	c.93A>C	P.I31I
XH_P10	7	104749493	104749493	Missense_Mutation	SNV	C	G	G	KMT2E	c.3573C>G	P.S1191R
XH_P10	7	107735780	107735780	Missense_Mutation	SNV	A	T	T	LAMB4	c.1363T>A	P.C455S
XH_P10	7	114655782	114655782	Missense_Mutation	SNV	A	T	T	MDFIC	c.534A>T	P.E178D
XH_P10	7	129474788	129474788	Missense_Mutation	SNV	T	C	C	UBE2H	c.331A>G	P.M111V
XH_P10	7	143792541	143792541	Nonsense_Mutation	SNV	T	A	A	OR2A12	c.341T>A	P.L114*
XH_P10	7	149506250	149506250	Missense_Mutation	SNV	G	T	T	SSPO	c.9240G>T	P.Q3080H
XH_P10	7	1785003	1785003	Silent	SNV	T	A	A	ELFN1	c.771T>A	P.A257A
XH_P10	7	5372637	5372637	Silent	SNV	C	A	A	TNRC18	c.5763G>T	P.V1921V
XH_P10	7	5983029	5983029	Missense_Mutation	SNV	C	T	T	RSPH10B	c.1684G>A	P.A562T
XH_P10	7	82586052	82586052	Missense_Mutation	SNV	G	C	C	PCLO	c.4217C>G	P.P1406R
XH_P10	7	99688069	99688069	Frame_Shift_Del	INDEL	G	-	-	COPS6	c.387delG	P.P130fs
XH_P10	7	99696202	99696202	Missense_Mutation	SNV	T	C	C	MCM7	c.191A>G	P.H64R
XH_P10	8	103324475	103324475	Splice_Site	SNV	T	C	C	UBR5	c.2152-2A>G	
XH_P10	8	10465144	10465144	Missense_Mutation	SNV	C	G	G	RP1L1	c.6464G>C	P.G2155A
XH_P10	8	110452977	110452977	Missense_Mutation	SNV	T	A	A	PKHD1L1	c.3995T>A	P.V1332E
XH_P10	8	110587217	110587217	Missense_Mutation	SNV	T	C	C	SYBU	c.1907A>G	P.Y636C
XH_P10	8	57219372	57219372	Nonsense_Mutation	SNV	A	T	T	SDR16C5	c.573T>A	P.C191*
XH_P10	8	70650421	70650421	Missense_Mutation	SNV	A	G	G	SLCO5A1	c.1277T>C	P.V426A
XH_P10	8	9473163	9473163	Missense_Mutation	SNV	C	T	T	TNKS	c.970C>T	P.P324S
XH_P10	9	104432937	104432937	Missense_Mutation	SNV	T	A	A	GRIN3A	c.1757A>T	P.K586M
XH_P10	9	111745457	111745457	Missense_Mutation	SNV	T	C	C	CTNNAL1	c.868A>G	P.I290V
XH_P10	9	111909481	111909481	Splice_Site	SNV	A	T	T	FRRS1L	c.477-12T>A	
XH_P10	9	114820713	114820713	Missense_Mutation	SNV	T	C	C	SUSD1	c.2104A>G	P.N702D
XH_P10	9	131825873	131825873	Missense_Mutation	SNV	G	A	A	MIGA2	c.1133G>A	P.R378Q
XH_P10	9	133235846	133235846	Silent	SNV	C	G	G	HMCN2	c.4131C>G	P.V1377V
XH_P10	9	136131555	136131567	Frame_Shift_Del	INDEL	GCGCATGGA	-	-	ABO	60delTGTCCATC	P.V183fs
XH_P10	9	137694778	137694778	Silent	SNV	T	A	A	COL5A1	c.3051T>A	P.P1017P
XH_P10	9	21971209	21971209	Splice_Site	SNV	T	A	A	CDKN2A	c.151-2A>T	
XH_P10	9	72000810	72000810	Missense_Mutation	SNV	C	A	A	FAM189A2	c.803C>A	P.P268H
XH_P10	9	847135	847135	Missense_Mutation	SNV	C	T	T	DMRT1	c.530C>T	P.A177V
XH_P10	X	117707935	117707935	Missense_Mutation	SNV	C	A	A	DOCK11	c.1343C>A	P.S448Y
XH_P10	X	118679419	118679419	Splice_Site	SNV	T	A	A	CXorf56	c.137+7A>T	
XH_P10	X	118759344	118759344	Splice_Site	SNV	T	C	C	SEPT6	c.1281-2A>G	
XH_P10	X	14868754	14868754	Missense_Mutation	SNV	C	A	A	FANCB	c.1369G>T	P.V457F
XH_P10	X	152686563	152686563	Missense_Mutation	SNV	A	C	C	ZFP92	c.728A>C	P.K243T
XH_P10	X	153209551	153209551	Missense_Mutation	SNV	A	G	G	RENBP	c.194T>C	P.V65A
XH_P10	X	49075334	49075334	Splice_Site	SNV	T	C	C	CACNA1F	c.2733+7A>G	
XH_P10	X	6452079	6452079	Missense_Mutation	SNV	G	C	C	VCX3A	c.268C>G	P.P90A
XH_P11	1	10327436	10327436	Splice_Site	SNV	A	T	T	KIF1B	c.430-2A>T	
XH_P11	1	103404615	103404615	Silent	SNV	G	C	C	COL11A1	c.3297C>G	P.G1099G
XH_P11	1	108145666	108145666	Splice_Site	SNV	T	A	A	VAV3	c.452+3A>T	
XH_P11	1	11008434	11008434	Silent	SNV	C	T	T	C1orf127	c.1758G>A	P.E586E
XH_P11	1	110466115	110466115	Missense_Mutation	SNV	T	A	A	CSF1	c.872T>A	P.M291K

XH_P11	1	110580561	110580561	Nonsense_Mutation	SNV	A	T	T	STRIP1	c.229A>T	P.K77*
XH_P11	1	110655275	110655275	Missense_Mutation	SNV	A	T	T	UBL4B	c.119A>T	P.Q40L
XH_P11	1	110925532	110925532	Missense_Mutation	SNV	A	T	T	SLC16A4	c.144T>A	P.F48L
XH_P11	1	114184577	114184577	Missense_Mutation	SNV	A	T	T	MAGI3	c.1405A>T	P.T469S
XH_P11	1	118533485	118533485	Missense_Mutation	SNV	T	A	A	SPAG17	c.5520A>T	P.E1840D
XH_P11	1	118596689	118596689	Missense_Mutation	SNV	A	T	T	SPAG17	c.2750T>A	P.I917K
XH_P11	1	120168570	120168570	Missense_Mutation	SNV	G	A	A	ZNF697	c.154C>T	P.H52Y
XH_P11	1	12032972	12032972	Missense_Mutation	SNV	A	T	T	PLOD1	c.1946A>T	P.E649V
XH_P11	1	12089129	12089129	Missense_Mutation	SNV	A	T	T	MIIP	c.587A>T	P.E196V
XH_P11	1	145304601	145304601	Missense_Mutation	SNV	A	T	T	NBPF10	c.1534A>T	P.I512F
XH_P11	1	147381324	147381324	Missense_Mutation	SNV	A	T	T	GJA8	c.1242A>T	P.R414S
XH_P11	1	152283052	152283052	Missense_Mutation	SNV	C	T	T	FLG	c.4310G>A	P.R1437H
XH_P11	1	153662818	153662818	Missense_Mutation	SNV	T	A	A	NPR1	c.2991T>A	P.D997E
XH_P11	1	158906786	158906786	Missense_Mutation	SNV	T	A	A	PYHIN1	c.86T>A	P.L29Q
XH_P11	1	159023358	159023358	Silent	SNV	A	T	T	IFI16	c.1953A>T	P.I651I
XH_P11	1	161277194	161277194	Missense_Mutation	SNV	T	A	A	MPZ	c.88A>T	P.I30F
XH_P11	1	162731053	162731053	Missense_Mutation	SNV	A	T	T	DDR2	c.908A>T	P.Q303L
XH_P11	1	166987164	166987164	Nonsense_Mutation	SNV	A	T	T	MAEL	c.916A>T	P.K306*
XH_P11	1	167992297	167992297	Splice_Site	SNV	A	T	T	DCAF6	c.1516+12A>T	
XH_P11	1	171763626	171763626	Missense_Mutation	SNV	A	T	T	METTL13	c.1316A>T	P.Q439L
XH_P11	1	176934382	176934382	Splice_Site	SNV	A	C	C	ASTN1	c.1524-9T>G	
XH_P11	1	179815653	179815653	Silent	SNV	G	A	A	TOR1AIP2	c.966C>T	P.S322S
XH_P11	1	179966255	179966255	Silent	SNV	A	C	C	CEP350	c.963A>C	P.S321S
XH_P11	1	186876132	186876132	Splice_Site	SNV	T	A	A	PLA2G4A	c.414T>A	P.V138V
XH_P11	1	192321220	192321220	Missense_Mutation	SNV	T	A	A	RGS21	c.132T>A	P.S44R
XH_P11	1	198713229	198713229	Missense_Mutation	SNV	T	A	A	PTPRC	c.2261T>A	P.F754Y
XH_P11	1	20097951	20097951	Silent	SNV	T	A	A	TMCO4	c.204A>T	P.A68A
XH_P11	1	201054691	201054691	Silent	SNV	C	A	A	CACNA1S	c.1023G>T	P.R341R
XH_P11	1	203144581	203144581	Missense_Mutation	SNV	T	A	A	MYBPH	c.214A>T	P.S72C
XH_P11	1	204985459	204985459	Missense_Mutation	SNV	A	T	T	NFASC	c.3515A>T	P.Q1172L
XH_P11	1	206649519	206649519	Splice_Site	SNV	C	T	T	IKBKE	c.359-5C>T	
XH_P11	1	206772913	206772913	Missense_Mutation	SNV	T	A	A	EIF2D	c.734A>T	P.E245V
XH_P11	1	207134144	207134144	Silent	SNV	T	A	A	FCAMR	c.1077A>T	P.I359I
XH_P11	1	207697162	207697162	Missense_Mutation	SNV	T	G	G	CR1	c.694T>G	P.C232G
XH_P11	1	21903087	21903087	Missense_Mutation	SNV	A	C	C	ALPL	c.1262A>C	P.Y421S
XH_P11	1	223567317	223567317	Missense_Mutation	SNV	A	T	T	CCDC185	c.500A>T	P.Q167L
XH_P11	1	223984215	223984215	Nonsense_Mutation	SNV	T	A	A	TP53BP2	c.2026A>T	P.K676*
XH_P11	1	223986038	223986038	Silent	SNV	G	A	A	TP53BP2	c.1827C>T	P.T609T
XH_P11	1	223991121	223991121	Missense_Mutation	SNV	T	A	A	TP53BP2	c.683A>T	P.Q228L
XH_P11	1	225305581	225305581	Splice_Site	SNV	A	T	T	DNAH14	c.3973-2A>T	
XH_P11	1	225533711	225533711	Nonsense_Mutation	SNV	A	T	T	DNAH14	c.10447A>T	P.K3483*
XH_P11	1	228412198	228412198	Silent	SNV	A	C	C	OBSCN	c.2692A>C	P.R898R
XH_P11	1	228431079	228431079	Missense_Mutation	SNV	A	T	T	OBSCN	c.3125A>T	P.Q1042L
XH_P11	1	228494079	228494079	Missense_Mutation	SNV	A	T	T	OBSCN	c.11666A>T	P.Q3889L
XH_P11	1	22852727	22852727	Silent	SNV	A	T	T	ZBTB40	c.3558A>T	P.T1186T
XH_P11	1	228563859	228563859	Missense_Mutation	SNV	T	A	A	OBSCN	c.22937T>A	P.L7646Q
XH_P11	1	228646135	228646135	Missense_Mutation	SNV	T	A	A	HIST3H2BB	c.305T>A	P.L102Q
XH_P11	1	237550652	237550652	Silent	SNV	A	T	T	RYR2	c.648A>T	P.P216P
XH_P11	1	237789049	237789049	Silent	SNV	A	G	G	RYR2	c.6111A>G	P.T2037T
XH_P11	1	243229377	243229377	Splice_Site	SNV	T	A	A	LINC01347	n.2507A>T	
XH_P11	1	243329015	243329015	Missense_Mutation	SNV	T	A	A	CEP170	c.1953A>T	P.Q651H
XH_P11	1	24448020	24448020	Missense_Mutation	SNV	T	A	A	IL22RA1	c.1000A>T	P.I334F
XH_P11	1	245851517	245851517	Missense_Mutation	SNV	A	T	T	KIF26B	c.5232A>T	P.R1744S
XH_P11	1	246797883	246797883	Missense_Mutation	SNV	A	T	T	CNST	c.812A>T	P.H271L
XH_P11	1	248309085	248309085	Silent	SNV	A	T	T	OR2M5	c.636A>T	P.A212A
XH_P11	1	248343805	248343805	Missense_Mutation	SNV	A	T	T	OR2M2	c.518A>T	P.E173V
XH_P11	1	248636880	248636880	Missense_Mutation	SNV	A	T	T	OR2T3	c.229A>T	P.M77L
XH_P11	1	26526403	26526403	Missense_Mutation	SNV	A	T	T	CATSPER4	c.841A>T	P.I281F
XH_P11	1	26672425	26672425	Missense_Mutation	SNV	C	A	A	AIM1L	c.724G>T	P.V242L
XH_P11	1	26688660	26688660	Missense_Mutation	SNV	A	T	T	ZNF683	c.1150T>A	P.W384R
XH_P11	1	33060697	33060697	Missense_Mutation	SNV	A	T	T	ZBTB8A	c.866A>T	P.H289L
XH_P11	1	3334551	3334551	Missense_Mutation	SNV	T	A	A	PRDM16	c.2851T>A	P.Y951N
XH_P11	1	3425710	3425710	Missense_Mutation	SNV	T	A	A	MEGF6	c.1457A>T	P.Q486L
XH_P11	1	37325569	37325569	Missense_Mutation	SNV	A	T	T	GRIK3	c.836T>A	P.L279Q
XH_P11	1	40236825	40236825	Missense_Mutation	SNV	T	A	A	OXCT2	c.103A>T	P.S35C
XH_P11	1	40756575	40756575	Missense_Mutation	SNV	A	T	T	ZMPSTE24	c.1109A>T	P.K370M
XH_P11	1	44595497	44595497	Missense_Mutation	SNV	T	A	A	KLF17	c.554T>A	P.V185E
XH_P11	1	45469920	45469920	Missense_Mutation	SNV	T	A	A	HECTD3	c.2272A>T	P.T758S
XH_P11	1	4772061	4772061	Missense_Mutation	SNV	T	A	A	AJAP1	c.131T>A	P.L44Q
XH_P11	1	54262715	54262715	Missense_Mutation	SNV	T	A	A	NDC1	c.1205A>T	P.D402V
XH_P11	1	59125755	59125755	Missense_Mutation	SNV	T	C	C	MYSM1	c.2401A>G	P.I801V
XH_P11	1	65858280	65858280	Silent	SNV	A	T	T	DNAJC6	c.1635A>T	P.P545P
XH_P11	1	67313226	67313226	Missense_Mutation	SNV	A	T	T	WDR78	c.1232T>A	P.L411Q
XH_P11	1	67560969	67560969	Silent	SNV	T	A	A	C1orf141	c.552A>T	P.P184P
XH_P11	1	74832999	74832999	Missense_Mutation	SNV	T	A	A	FPGT-TNNI3K	c.1579T>A	P.C527S
XH_P11	1	75108743	75108743	Missense_Mutation	SNV	A	C	C	ERICH3	c.283T>G	P.L95V
XH_P11	1	85583420	85583420	Missense_Mutation	SNV	T	A	A	WDR63	c.1678T>A	P.L560I
XH_P11	1	87045812	87045812	Silent	SNV	A	T	T	CLCA4	c.2544A>T	P.I848I
XH_P11	1	91733321	91733321	Silent	SNV	A	T	T	HFM1	c.3864T>A	P.T1288T
XH_P11	1	92646194	92646194	Missense_Mutation	SNV	A	T	T	KIAA1107	c.1415A>T	P.Q472L
XH_P11	10	100150374	100150374	Missense_Mutation	SNV	C	T	T	PYROXD2	c.1273G>A	P.D425N
XH_P11	10	102056727	102056727	Splice_Site	SNV	T	A	A	PKD2L1	c.1044+10A>T	
XH_P11	10	112581358	112581358	Missense_Mutation	SNV	A	T	T	RBM20	c.2981A>T	P.D994V
XH_P11	10	118364969	118364969	Missense_Mutation	SNV	A	T	T	PNLIPRP1	c.1244A>T	P.K415I
XH_P11	10	121335228	121335228	Silent	SNV	T	A	A	TIAL1	c.1128A>T	P.I376I
XH_P11	10	121411297	121411297	Missense_Mutation	SNV	C	A	A	BAG3	c.110C>A	P.P37H
XH_P11	10	123847226	123847226	Silent	SNV	A	T	T	TACC2	c.5211A>T	P.A1737A
XH_P11	10	129904062	129904062	Silent	SNV	T	A	A	MKI67	c.4962A>T	P.P1654P
XH_P11	10	135015264	135015264	Silent	SNV	A	T	T	KNDC1	c.3249A>T	P.P1083P
XH_P11	10	24873923	24873923	Silent	SNV	T	A	A	ARHGAP21	c.5295A>T	P.I1765I
XH_P11	10	25284682	25284682	Missense_Mutation	SNV	T	A	A	ENKUR	c.154A>T	P.I52F
XH_P11	10	31810285	31810285	Silent	SNV	A	T	T	ZEB1	c.1974A>T	P.T658T
XH_P11	10	32580144	32580144	Missense_Mutation	SNV	T	A	A	EPC1	c.922A>T	P.S308C
XH_P11	10	34630694	34630694	Missense_Mutation	SNV	T	G	G	PARD3	c.2269A>C	P.I757L
XH_P11	10	43292911	43292911	Missense_Mutation	SNV	G	C	C	BMS1	c.2029G>C	P.E677Q
XH_P11	10	45956744	45956744	Missense_Mutation	SNV	T	A	A	MARCH8	c.358A>T	P.T120S
XH_P11	10	50151412	50151412	Missense_Mutation	SNV	T	A	A	WDFY4	c.7667T>A	P.L2556H
XH_P11	10	51769402	51769402	Missense_Mutation	SNV	A	T	T	AGAP6	c.1517A>T	P.H506L

XH_P11	10	63983070	63983070	Silent	SNV	T	A	A	RTKN2	c.708A>T	P.L236L
XH_P11	10	69750720	69750720	Missense_Mutation	SNV	C	T	T	HERC4	c.1384G>A	P.A462T
XH_P11	10	69991121	69991121	Missense_Mutation	SNV	T	A	A	ATOH7	c.314A>T	P.E105V
XH_P11	10	70141012	70141012	Missense_Mutation	SNV	T	A	A	RUFY2	c.982A>T	P.I328L
XH_P11	10	70863725	70863725	Missense_Mutation	SNV	G	T	T	SRGN	c.326G>T	P.G109V
XH_P11	10	78669774	78669774	Missense_Mutation	SNV	C	T	T	KCNMA1	c.2935G>A	P.A979T
XH_P11	10	78761335	78761335	Missense_Mutation	SNV	T	A	A	KCNMA1	c.2096A>T	P.K699M
XH_P11	10	85992497	85992497	Missense_Mutation	SNV	T	A	A	LRIT1	c.1058A>T	P.E353V
XH_P11	10	88418450	88418450	Splice_Site	SNV	T	A	A	OPN4	c.661+6T>A	
XH_P11	10	90486632	90486632	Silent	SNV	T	C	C	LIPK	c.186T>C	P.Y62Y
XH_P11	10	94230081	94230081	Missense_Mutation	SNV	T	G	G	IDE	c.473A>C	P.K158T
XH_P11	11	102100651	102100651	Missense_Mutation	SNV	A	T	T	YAP1	c.1333A>T	P.S445C
XH_P11	11	1078540	1078540	Missense_Mutation	SNV	T	A	A	MUC2	c.748T>A	P.C250S
XH_P11	11	118038791	118038791	Splice_Site	SNV	A	T	T	SCN2B	c.448+9T>A	
XH_P11	11	118509703	118509703	Nonsense_Mutation	SNV	T	A	A	PHLDB1	c.2630T>A	P.L877*
XH_P11	11	123479435	123479435	Missense_Mutation	SNV	G	T	T	GRAMD1B	c.1174G>T	P.V392L
XH_P11	11	123894400	123894400	Missense_Mutation	SNV	C	G	G	OR10G9	c.681C>G	P.I227M
XH_P11	11	124551301	124551301	Silent	SNV	A	G	G	SPA17	c.171A>G	P.P57P
XH_P11	11	17653625	17653625	Splice_Site	SNV	A	T	T	OTOG	c.6970-10A>T	
XH_P11	11	30358143	30358143	Missense_Mutation	SNV	A	C	C	ARL14EP	c.584A>C	P.Y195S
XH_P11	11	376697	376697	Missense_Mutation	SNV	T	A	A	B4GALNT4	c.1574T>A	P.V525E
XH_P11	11	49974217	49974217	Silent	SNV	A	T	T	OR4C13	c.243A>T	P.T81T
XH_P11	11	55541348	55541348	Silent	SNV	T	A	A	OR5D13	c.435T>A	P.L145L
XH_P11	11	5626603	5626603	Missense_Mutation	SNV	G	C	C	TRIM6	c.640G>C	P.E214Q
XH_P11	11	56467921	56467921	Missense_Mutation	SNV	C	T	T	OR9G9	c.58C>T	P.P20S
XH_P11	11	57099227	57099227	Missense_Mutation	SNV	C	T	T	SSRP1	c.1138G>A	P.A380T
XH_P11	11	5878235	5878235	Missense_Mutation	SNV	A	T	T	OR52E8	c.698T>A	P.L233Q
XH_P11	11	63283171	63283171	Nonsense_Mutation	SNV	C	T	T	LGALS12	c.853C>T	P.Q285*
XH_P11	11	65325201	65325201	Missense_Mutation	SNV	T	A	A	LTBP3	c.230A>T	P.K77M
XH_P11	11	65350003	65350003	Silent	SNV	A	T	T	EHBP1L1	c.1860A>T	P.S620S
XH_P11	11	74952918	74952918	Missense_Mutation	SNV	T	A	A	TPBGL	c.824T>A	P.L275Q
XH_P11	11	75378511	75378511	Missense_Mutation	SNV	T	A	A	MAP6	c.904A>T	P.R302W
XH_P11	11	76371120	76371120	Missense_Mutation	SNV	A	T	T	LRRC32	c.1517T>A	P.L506Q
XH_P11	11	82880256	82880256	Missense_Mutation	SNV	A	T	T	PCF11	c.2879A>T	P.H960L
XH_P11	11	93913103	93913103	Missense_Mutation	SNV	C	T	T	PANX1	c.881C>T	P.T294M
XH_P11	12	101689317	101689317	Silent	SNV	A	T	T	UTP20	c.1311A>T	P.V437V
XH_P11	12	102122917	102122917	Silent	SNV	A	C	C	SYCP3	c.627T>G	P.A209A
XH_P11	12	105519895	105519895	Missense_Mutation	SNV	A	T	T	KIAA1033	c.900A>T	P.E300D
XH_P11	12	108642066	108642066	Silent	SNV	A	T	T	WSCD2	c.1644A>T	P.A548A
XH_P11	12	109858782	109858782	Splice_Site	SNV	A	T	T	MYO1H	c.1550-4A>T	
XH_P11	12	113645809	113645809	Missense_Mutation	SNV	T	A	A	IQCD	c.163A>T	P.I55F
XH_P11	12	117191863	117191863	Nonsense_Mutation	SNV	A	T	T	RNFT2	c.625A>T	P.K209*
XH_P11	12	118610442	118610442	Silent	SNV	T	A	A	TAOK3	c.1719A>T	P.T573T
XH_P11	12	122396931	122396931	Silent	SNV	T	A	A	WDR66	c.2064T>A	P.P688P
XH_P11	12	122409475	122409475	Missense_Mutation	SNV	G	T	T	WDR66	c.2783G>T	P.S928I
XH_P11	12	124332644	124332644	Splice_Site	SNV	T	A	A	DNAH10	c.5595+2T>A	
XH_P11	12	132562031	132562031	Missense_Mutation	SNV	A	T	T	EP400	c.9185A>T	P.Q3062L
XH_P11	12	21196456	21196456	Missense_Mutation	SNV	A	T	T	SLCO1B7	c.775A>T	P.N259Y
XH_P11	12	26348644	26348644	Missense_Mutation	SNV	G	T	T	SSPN	c.39G>T	P.Q13H
XH_P11	12	2760891	2760891	Missense_Mutation	SNV	A	T	T	CACNA1C	c.4031A>T	P.E1344V
XH_P11	12	32136405	32136405	Missense_Mutation	SNV	A	T	T	KIAA1551	c.2516A>T	P.K839M
XH_P11	12	40441985	40441985	Missense_Mutation	SNV	T	A	A	SLC2A13	c.584A>T	P.Y195F
XH_P11	12	46322603	46322603	Missense_Mutation	SNV	T	A	A	SCAF11	c.881A>T	P.E294V
XH_P11	12	48962871	48962871	Missense_Mutation	SNV	A	T	T	LALBA	c.286T>A	P.C96S
XH_P11	12	51054047	51054047	Silent	SNV	A	T	T	DIP2B	c.372A>T	P.P124P
XH_P11	12	53201609	53201609	Nonsense_Mutation	SNV	G	A	A	KRT4	c.1165C>T	P.R389*
XH_P11	12	53415595	53415595	Silent	SNV	A	T	T	EIF4B	c.483A>T	P.L161L
XH_P11	12	57593803	57593803	Missense_Mutation	SNV	A	T	T	LRP1	c.10009A>T	P.S3337C
XH_P11	12	57602502	57602502	Missense_Mutation	SNV	A	T	T	LRP1	c.12047A>T	P.Y4016F
XH_P11	12	57858966	57858966	Nonsense_Mutation	SNV	T	A	A	GLI1	c.462T>A	P.C154*
XH_P11	12	57873068	57873068	Missense_Mutation	SNV	T	A	A	ARHGAP9	c.122A>T	P.Q41L
XH_P11	12	6554316	6554316	Missense_Mutation	SNV	T	A	A	CD27	c.55T>A	P.S19T
XH_P11	12	69783940	69783940	Missense_Mutation	SNV	A	T	T	YEATS4	c.366A>T	P.E122D
XH_P11	12	70989989	70989989	Missense_Mutation	SNV	A	T	T	PTPRB	c.1098T>A	P.D366E
XH_P11	12	72017923	72017923	Missense_Mutation	SNV	T	C	C	ZFC3H1	c.4467A>G	P.I1489M
XH_P11	12	78570945	78570945	Missense_Mutation	SNV	T	A	A	NAV3	c.5149T>A	P.F1717I
XH_P11	12	80747121	80747121	Frame_Shift_Del	INDEL	G	-	-	OTOGL	c.5400delG	P.K1801fs
XH_P11	12	81063191	81063191	Missense_Mutation	SNV	T	A	A	PTPRQ	c.5885T>A	P.L1962Q
XH_P11	12	81101647	81101647	Missense_Mutation	SNV	C	T	T	MYF6	c.149C>T	P.P50L
XH_P11	12	86229969	86229969	Missense_Mutation	SNV	A	T	T	RASSF9	c.11T>A	P.F4Y
XH_P11	12	8667914	8667914	Silent	SNV	A	T	T	CLEC4D	c.111A>T	P.A37A
XH_P11	13	111090347	111090347	Splice_Site	SNV	A	T	T	COL4A2	c.826-2A>T	
XH_P11	13	25008845	25008845	Silent	SNV	T	A	A	PARP4	c.4434A>T	P.P1478P
XH_P11	13	58207642	58207642	Missense_Mutation	SNV	T	A	A	PCDH17	c.962T>A	P.V321E
XH_P11	13	77756024	77756024	Missense_Mutation	SNV	T	A	A	MYCBP2	c.4753A>T	P.S1585C
XH_P11	13	99083293	99083293	Splice_Site	SNV	C	T	T	FARP1	c.1906-4C>T	
XH_P11	14	100792568	100792568	Splice_Site	SNV	A	T	T	SLC25A47	c.144+3A>T	
XH_P11	14	102500476	102500476	Missense_Mutation	SNV	A	T	T	DYNC1H1	c.10577A>T	P.Q3526L
XH_P11	14	102718293	102718293	Missense_Mutation	SNV	T	C	C	MOK	c.233A>G	P.Y78C
XH_P11	14	104492006	104492006	Missense_Mutation	SNV	C	T	T	TDRD9	c.2824C>T	P.P942S
XH_P11	14	20182033	20182033	Missense_Mutation	SNV	A	T	T	OR11H2	c.43T>A	P.S15T
XH_P11	14	20345285	20345285	Missense_Mutation	SNV	T	A	A	OR4K2	c.859T>A	P.Y287N
XH_P11	14	21991690	21991690	Silent	SNV	T	A	A	SALL2	c.1767A>T	P.A589A
XH_P11	14	23871992	23871992	Missense_Mutation	SNV	T	A	A	MYH6	c.916A>T	P.N306Y
XH_P11	14	24613173	24613173	Splice_Site	SNV	T	A	A	PSME2	c.552+11A>T	
XH_P11	14	38222435	38222435	Missense_Mutation	SNV	A	T	T	TTC6	c.3309A>T	P.Q1103H
XH_P11	14	54946533	54946533	Nonsense_Mutation	SNV	T	A	A	GMFB	c.328A>T	P.K110*
XH_P11	14	58605536	58605536	Missense_Mutation	SNV	T	C	C	C14orf37	c.541A>G	P.K181E
XH_P11	14	65041181	65041181	Missense_Mutation	SNV	T	A	A	PPP1R36	c.542T>A	P.V181E
XH_P11	14	68219062	68219062	Missense_Mutation	SNV	T	A	A	ZFYVE26	c.7370A>T	P.Q2457L
XH_P11	14	73720624	73720624	Nonsense_Mutation	SNV	T	A	A	PAPLN	c.1176T>A	P.C392*
XH_P11	14	74823599	74823599	Missense_Mutation	SNV	T	A	A	VRTN	c.113T>A	P.L38Q
XH_P11	14	92136196	92136196	Missense_Mutation	SNV	G	A	A	CATSPERB	c.1249C>T	P.H417Y
XH_P11	14	92441585	92441585	Missense_Mutation	SNV	T	A	A	TRIP11	c.5385A>T	P.K1795N
XH_P11	14	92470389	92470389	Missense_Mutation	SNV	T	C	C	TRIP11	c.3928A>G	P.I1310V
XH_P11	14	94041486	94041486	Missense_Mutation	SNV	T	A	A	UNC79	c.1622T>A	P.L541Q
XH_P11	14	94420755	94420755	Missense_Mutation	SNV	T	A	A	ASB2	c.386A>T	P.K129M
XH_P11	14	99640913	99640913	Missense_Mutation	SNV	T	A	A	BCL11B	c.2257A>T	P.T753S

XH_P11	14	99697763	99697763	Missense_Mutation	SNV	T	A	A	BCL11B	c.556A>T	P.S186C
XH_P11	15	101562114	101562114	Missense_Mutation	SNV	A	T	T	LRRK1	c.1804A>T	P.T602S
XH_P11	15	22369062	22369062	Missense_Mutation	SNV	A	C	C	OR4M2	c.487A>C	P.I163L
XH_P11	15	23811694	23811694	Silent	SNV	G	A	A	MKRN3	c.765G>A	P.G255G
XH_P11	15	33091112	33091112	Silent	SNV	A	T	T	FMN1	c.3354T>A	P.S1118S
XH_P11	15	42054382	42054382	Missense_Mutation	SNV	A	T	T	MGA	c.7566A>T	P.Q2522H
XH_P11	15	51792010	51792010	Silent	SNV	T	A	A	DMXL2	c.3411A>T	P.S1137S
XH_P11	15	52263903	52263903	Missense_Mutation	SNV	T	A	A	LEO1	c.40A>T	P.S14C
XH_P11	15	52338748	52338748	Missense_Mutation	SNV	A	T	T	MAPK6	c.91A>T	P.N31Y
XH_P11	15	57213245	57213245	Missense_Mutation	SNV	A	G	G	TCF12	c.97A>G	P.S33G
XH_P11	15	59179748	59179748	Splice_Site	SNV	T	A	A	SLTM	c.2322-9A>T	
XH_P11	15	66828351	66828351	Missense_Mutation	SNV	A	T	T	ZWILCH	c.1080A>T	P.L360F
XH_P11	15	74427083	74427083	Missense_Mutation	SNV	A	T	T	ISLR2	c.1988A>T	P.E663V
XH_P11	15	75015158	75015158	Missense_Mutation	SNV	T	A	A	CYP1A1	c.281A>T	P.Q94L
XH_P11	15	81199019	81199019	Missense_Mutation	SNV	T	A	A	CEMIP	c.1427T>A	P.L476Q
XH_P11	15	90992870	90992870	Missense_Mutation	SNV	A	T	T	IQGAP1	c.1157A>T	P.Q386L
XH_P11	16	11852279	11852279	Splice_Site	SNV	T	A	A	ZC3H7A	c.2428+8A>T	
XH_P11	16	12142268	12142268	Missense_Mutation	SNV	A	T	T	SNX29	c.539A>T	P.K180M
XH_P11	16	1486441	1486441	Splice_Site	SNV	T	A	A	CCDC154	c.1487+5A>T	
XH_P11	16	1552645	1552645	Splice_Site	SNV	G	T	T	TELO2	c.1654-1G>T	
XH_P11	16	17294396	17294396	Missense_Mutation	SNV	C	A	A	XYLT1	c.1029G>T	P.Q343H
XH_P11	16	1735447	1735447	Nonsense_Mutation	SNV	A	T	T	HN1L	c.52A>T	P.K18*
XH_P11	16	19451366	19451366	Silent	SNV	T	A	A	TMC5	c.6T>A	P.S2S
XH_P11	16	23486290	23486290	Missense_Mutation	SNV	G	C	C	GGA2	c.1385C>G	P.P462R
XH_P11	16	2980644	2980644	Missense_Mutation	SNV	A	T	T	FLYWCH1	c.559A>T	P.R187W
XH_P11	16	31046327	31046327	Missense_Mutation	SNV	A	G	G	STX4	c.110A>G	P.Y37C
XH_P11	16	31288067	31288067	Splice_Site	SNV	T	A	A	ITGAM	c.1083+2T>A	
XH_P11	16	31374649	31374649	Missense_Mutation	SNV	T	A	A	ITGAX	c.1664T>A	P.L555Q
XH_P11	16	31393213	31393213	Silent	SNV	G	A	A	ITGAX	c.3477G>A	P.P1159P
XH_P11	16	3254365	3254365	Missense_Mutation	SNV	T	A	A	OR1F1	c.119T>A	P.L40Q
XH_P11	16	32890861	32890861	Splice_Site	SNV	A	T	T	SLC6A10P	n.3029-9T>A	
XH_P11	16	4438057	4438064	Frame_Shift_Del	INDEL	CGCGCTCA	-	-	CORO7	4_661delTGAGC	P.E219fs
XH_P11	16	47117433	47117433	Missense_Mutation	SNV	C	T	T	NETO2	c.1256G>A	P.R419Q
XH_P11	16	49670335	49670335	Missense_Mutation	SNV	T	A	A	ZNF423	c.2548A>T	P.N850Y
XH_P11	16	50188158	50188158	Missense_Mutation	SNV	A	T	T	PAPD5	c.581A>T	P.Q194L
XH_P11	16	50340952	50340952	Splice_Site	SNV	T	A	A	ADCY7	c.1753-9T>A	
XH_P11	16	57110738	57110738	Missense_Mutation	SNV	A	G	G	NLRC5	c.4759A>G	P.R1587G
XH_P11	16	57597029	57597029	Missense_Mutation	SNV	T	A	A	ADGRG5	c.167T>A	P.L56Q
XH_P11	16	57689897	57689897	Missense_Mutation	SNV	T	A	A	ADGRG1	c.1010T>A	P.L337Q
XH_P11	16	57805253	57805253	Nonsense_Mutation	SNV	T	A	A	KIFC3	c.205A>T	P.K69*
XH_P11	16	57994796	57994796	Nonsense_Mutation	SNV	C	T	T	CNGB1	c.482G>A	P.W161*
XH_P11	16	65025681	65025681	Missense_Mutation	SNV	C	G	G	CDH11	c.801G>C	P.K267N
XH_P11	16	67354676	67354676	Missense_Mutation	SNV	A	T	T	KCTD19	c.116T>A	P.L39Q
XH_P11	16	68405275	68405275	Silent	SNV	T	A	A	SMPD3	c.810A>T	P.G270G
XH_P11	16	70843761	70843761	Silent	SNV	A	T	T	HYDIN	c.14808T>A	P.T4936T
XH_P11	16	81157318	81157318	Missense_Mutation	SNV	A	G	G	PKD1L2	c.4364T>C	P.V1455A
XH_P11	16	81946207	81946207	Missense_Mutation	SNV	A	T	T	PLCG2	c.1940A>T	P.Y647F
XH_P11	16	87417139	87417139	Missense_Mutation	SNV	G	A	A	FBXO31	c.212C>T	P.P71L
XH_P11	16	87795598	87795598	Missense_Mutation	SNV	T	A	A	KLHDC4	c.148A>T	P.R50W
XH_P11	16	88501076	88501076	Missense_Mutation	SNV	T	A	A	ZNF469	c.7114T>A	P.S2372T
XH_P11	17	10309651	10309651	Silent	SNV	A	T	T	MYH8	c.2235T>A	P.S745S
XH_P11	17	10419328	10419328	Silent	SNV	C	T	T	MYH1	c.420G>A	P.V140V
XH_P11	17	1173936	1173936	Missense_Mutation	SNV	T	A	A	BHLHA9	c.79T>A	P.C27S
XH_P11	17	12656355	12656355	Missense_Mutation	SNV	C	A	A	MYOCD	c.1750C>A	P.Q584K
XH_P11	17	15406345	15406345	Missense_Mutation	SNV	T	A	A	TVP23C	c.664A>T	P.S222C
XH_P11	17	16456008	16456008	Nonsense_Mutation	SNV	A	T	T	ZNF287	c.1448T>A	P.L483*
XH_P11	17	18671881	18671881	Missense_Mutation	SNV	T	A	A	FBXW10	c.1739T>A	P.L580Q
XH_P11	17	28405286	28405286	Missense_Mutation	SNV	T	C	C	EFCAB5	c.2791T>C	P.S931P
XH_P11	17	36622886	36622886	Missense_Mutation	SNV	A	T	T	ARHGAP23	c.962A>T	P.E321V
XH_P11	17	37374196	37374196	Silent	SNV	T	A	A	STAC2	c.321A>T	P.P107P
XH_P11	17	39724760	39724760	Missense_Mutation	SNV	C	A	A	KRT9	c.1170G>T	P.K390N
XH_P11	17	39741272	39741272	Missense_Mutation	SNV	A	T	T	KRT14	c.563T>A	P.L188Q
XH_P11	17	40332941	40332941	Missense_Mutation	SNV	A	T	T	KCNH4	c.23T>A	P.L8Q
XH_P11	17	48680533	48680533	Missense_Mutation	SNV	G	A	A	CACNA1G	c.4142G>A	P.R1381Q
XH_P11	17	4995277	4995277	Missense_Mutation	SNV	A	T	T	ZFP3	c.478A>T	P.M160L
XH_P11	17	5037241	5037241	Silent	SNV	G	A	A	USP6	c.444G>A	P.V148V
XH_P11	17	59667963	59667963	Silent	SNV	T	A	A	NACA2	c.579A>T	P.A193A
XH_P11	17	61561900	61561900	Missense_Mutation	SNV	T	G	G	ACE	c.1919T>G	P.I640R
XH_P11	17	62265728	62265728	Missense_Mutation	SNV	T	A	A	TEX2	c.2224A>T	P.T742S
XH_P11	17	66268769	66268769	Splice_Site	SNV	A	T	T	SLC16A6	c.505+11T>A	
XH_P11	17	70120481	70120481	Missense_Mutation	SNV	C	A	A	SOX9	c.1483C>A	P.P495T
XH_P11	17	72246161	72246161	Silent	SNV	T	A	A	TTYH2	c.1017T>A	P.T339T
XH_P11	17	72522045	72522045	Missense_Mutation	SNV	T	A	A	CD300LB	c.212A>T	P.E71V
XH_P11	17	72932032	72932032	Missense_Mutation	SNV	T	A	A	OTOP3	c.16T>A	P.S6T
XH_P11	17	72949646	72949646	Silent	SNV	A	T	T	HID1	c.1965T>A	P.A655A
XH_P11	17	7320990	7320990	Missense_Mutation	SNV	G	T	T	NLGN2	c.2380G>T	P.G794C
XH_P11	17	7507153	7507153	Missense_Mutation	SNV	G	A	A	FXR2	c.371C>T	P.P124L
XH_P11	17	7558003	7558004	Splice_Site	INDEL	GT	-	-	ATP1B2	363+2_363+3delTG	
XH_P11	17	7577587	7577587	Missense_Mutation	SNV	T	A	A	TP53	c.694A>T	P.I232F
XH_P11	17	7578226	7578226	Missense_Mutation	SNV	T	A	A	TP53	c.623A>T	P.D208V
XH_P11	17	76540027	76540027	Missense_Mutation	SNV	A	T	T	DNAH17	c.2444T>A	P.L815Q
XH_P11	17	80543794	80543794	Missense_Mutation	SNV	A	T	T	FOXK2	c.1294A>T	P.S432C
XH_P11	17	9676188	9676188	Missense_Mutation	SNV	T	A	A	DHRS7C	c.623A>T	P.E208V
XH_P11	18	11689607	11689607	Silent	SNV	A	T	T	GNAL	c.45A>T	P.P15P
XH_P11	18	13038575	13038575	Missense_Mutation	SNV	A	T	T	CEP192	c.1806A>T	P.K602N
XH_P11	18	21474994	21474994	Missense_Mutation	SNV	A	T	T	LAMA3	c.758A>T	P.H253L
XH_P11	18	2722522	2722522	Missense_Mutation	SNV	A	C	C	SMCHD1	c.2464A>C	P.K822Q
XH_P11	18	5423365	5423365	Splice_Site	SNV	A	T	T	EPB41L3	c.1339+12T>A	
XH_P11	18	64211317	64211317	Missense_Mutation	SNV	G	C	C	CDH19	c.1105C>G	P.L369V
XH_P11	18	72343308	72343308	Silent	SNV	A	T	T	ZNF407	c.333A>T	P.T111T
XH_P11	18	74672633	74672633	Splice_Site	SNV	A	T	T	ZNF236	c.5243-2A>T	
XH_P11	19	1042135	1042135	Silent	SNV	A	C	C	ABCA7	c.375A>C	P.L125L
XH_P11	19	11728111	11728111	Missense_Mutation	SNV	T	C	C	ZNF627	c.697T>C	P.Y233H
XH_P11	19	13054581	13054581	Nonsense_Mutation	SNV	G	T	T	CALR	c.1108G>T	P.E370*
XH_P11	19	1399572	1399572	Missense_Mutation	SNV	T	A	A	GAMT	c.342A>T	P.K114N
XH_P11	19	14091471	14091471	Missense_Mutation	SNV	T	A	A	RFX1	c.652A>T	P.S218C
XH_P11	19	1418659	1418659	Splice_Site	SNV	C	T	T	DAZAP1	c.238-6C>T	
XH_P11	19	14938527	14938527	Missense_Mutation	SNV	T	A	A	OR7A5	c.527A>T	P.H176L

XH_P11	19	15730539	15730539	Nonstop_Mutation	SNV	A	T	T	CYP4F8	c.491A>T	P.Ter164Lext*?
XH_P11	19	17010292	17010292	Missense_Mutation	SNV	A	T	T	CPAMD8	c.4983T>A	P.F1661L
XH_P11	19	17296790	17296790	Missense_Mutation	SNV	A	T	T	MYO9B	c.2557A>T	P.I853F
XH_P11	19	17471364	17471364	Frame_Shift_Del	INDEL	G	-	-	PLVAP	c.1295delC	P.P432fs
XH_P11	19	17918872	17918872	Missense_Mutation	SNV	T	A	A	B3GNT3	c.256T>A	P.Y86N
XH_P11	19	1800363	1800363	Missense_Mutation	SNV	T	A	A	ATP8B3	c.1097A>T	P.Y366F
XH_P11	19	1827407	1827407	Missense_Mutation	SNV	T	A	A	REXO1	c.1381A>T	P.T461S
XH_P11	19	19136612	19136612	Missense_Mutation	SNV	T	A	A	SUGP2	c.545A>T	P.E182V
XH_P11	19	19349151	19349151	Nonsense_Mutation	SNV	A	T	T	NCAN	c.3340A>T	P.K1114*
XH_P11	19	20727849	20727849	Missense_Mutation	SNV	T	C	C	ZNF737	c.1160A>G	P.H387R
XH_P11	19	22001965	22001965	Missense_Mutation	SNV	A	T	T	ZNF43	c.44T>A	P.L15Q
XH_P11	19	31040399	31040399	Missense_Mutation	SNV	T	G	G	ZNF536	c.3873T>G	P.C1291W
XH_P11	19	31040400	31040400	Missense_Mutation	SNV	A	T	T	ZNF536	c.3874A>T	P.I1292F
XH_P11	19	33095278	33095278	Missense_Mutation	SNV	T	A	A	ANKRD27	c.2546A>T	P.E849V
XH_P11	19	33698025	33698025	Silent	SNV	A	T	T	LRP3	c.1857A>T	P.R619R
XH_P11	19	36049497	36049497	Missense_Mutation	SNV	A	T	T	ATP4A	c.1347T>A	P.D449E
XH_P11	19	36340136	36340136	Splice_Site	SNV	A	T	T	NPHS1	c.840+2T>A	
XH_P11	19	36342421	36342421	Missense_Mutation	SNV	A	T	T	NPHS1	c.212T>A	P.L71Q
XH_P11	19	37853035	37853035	Missense_Mutation	SNV	G	C	C	HKR1	c.284G>C	P.S95T
XH_P11	19	38877661	38877661	Missense_Mutation	SNV	A	T	T	GGN	c.241T>A	P.S81T
XH_P11	19	39026650	39026650	Missense_Mutation	SNV	A	T	T	RYR1	c.11530A>T	P.N3844Y
XH_P11	19	39061251	39061251	Missense_Mutation	SNV	A	T	T	RYR1	c.13664A>T	P.Y4555F
XH_P11	19	39088026	39088026	Splice_Site	SNV	T	A	A	MAP4K1	c.1779-2A>T	
XH_P11	19	39329231	39329231	Missense_Mutation	SNV	T	C	C	HNRNPL	c.964A>G	P.K322E
XH_P11	19	39370142	39370142	Splice_Site	SNV	T	A	A	SIRT2	c.685-2A>T	
XH_P11	19	41008183	41008183	Missense_Mutation	SNV	A	T	T	SPTBN4	c.1046A>T	P.Q349L
XH_P11	19	41263279	41263279	Missense_Mutation	SNV	A	T	T	SNRPA	c.116A>T	P.Q39L
XH_P11	19	4216921	4216921	Silent	SNV	A	T	T	ANKRD24	c.1764A>T	P.G588G
XH_P11	19	44352308	44352391	Missense_Mutation	INDEL	TTCAACATGA	-	-	ZNF283	GAATCCATACAG	P.N386_F413del
XH_P11	19	45368854	45368854	Missense_Mutation	SNV	A	T	T	NECTIN2	c.415A>T	P.T139S
XH_P11	19	4544319	4544319	Missense_Mutation	SNV	A	T	T	SEMA6B	c.1961T>A	P.L654Q
XH_P11	19	46262753	46262753	Splice_Site	SNV	A	T	T	BHMG1	c.1245+5A>T	
XH_P11	19	47259495	47259495	Missense_Mutation	SNV	C	T	T	FKRP	c.788C>T	P.A263V
XH_P11	19	47424658	47424658	Missense_Mutation	SNV	A	C	C	ARHGAP35	c.2726A>C	P.E909A
XH_P11	19	4816360	4816360	Missense_Mutation	SNV	T	A	A	TICAM1	c.2030A>T	P.Q677L
XH_P11	19	49001914	49001914	Silent	SNV	C	T	T	LMTK3	c.2499G>A	P.E833E
XH_P11	19	49388703	49388703	Splice_Site	SNV	A	T	T	TULP2	c.1061+2T>A	
XH_P11	19	49510613	49510613	Silent	SNV	A	T	T	RUVBL2	c.348A>T	P.P116P
XH_P11	19	50128360	50128360	Splice_Site	SNV	A	T	T	PRR12	c.5869-2A>T	
XH_P11	19	51410215	51410215	Missense_Mutation	SNV	A	T	T	KLK4	c.455T>A	P.I152K
XH_P11	19	51650074	51650074	Missense_Mutation	SNV	T	A	A	SIGLEC7	c.1091T>A	P.L364Q
XH_P11	19	51957571	51957571	Splice_Site	SNV	T	A	A	SIGLEC8	c.1149-2A>T	
XH_P11	19	5212443	5212443	Missense_Mutation	SNV	A	T	T	PTPRS	c.4674T>A	P.H1558Q
XH_P11	19	52619682	52619682	Silent	SNV	T	A	A	ZNF616	c.735A>T	P.V245V
XH_P11	19	53644475	53644475	Missense_Mutation	SNV	T	A	A	ZNF347	c.1609A>T	P.T537S
XH_P11	19	54502990	54502990	Missense_Mutation	SNV	T	A	A	CACNG6	c.509T>A	P.L170Q
XH_P11	19	55423540	55423540	Missense_Mutation	SNV	C	G	G	NCR1	c.684C>G	P.D228E
XH_P11	19	56156320	56156320	Missense_Mutation	SNV	G	A	A	ZNF581	c.383G>A	P.S128N
XH_P11	19	58189659	58189659	Missense_Mutation	SNV	C	A	A	ZSCAN4	c.688C>A	P.P230T
XH_P11	19	7762190	7762190	Splice_Site	SNV	T	A	A	FCER2	c.251-6A>T	
XH_P11	19	7830719	7830719	Missense_Mutation	SNV	A	T	T	CLEC4M	c.410A>T	P.Q137L
XH_P11	19	7927358	7927358	Missense_Mutation	SNV	A	T	T	EVI5L	c.1885A>T	P.N629Y
XH_P11	19	902047	902047	Missense_Mutation	SNV	T	A	A	R3HDM4	c.155A>T	P.N52I
XH_P11	19	9028402	9028402	Splice_Site	SNV	T	A	A	MUC16	c.36396-6A>T	
XH_P11	19	9057035	9057035	Silent	SNV	A	T	T	MUC16	c.30411T>A	P.S10137S
XH_P11	19	9065786	9065786	Frame_Shift_Ins	INDEL	-	G	G	MUC16	21659_21660ins	P.T7221fs
XH_P11	19	9204736	9204736	Missense_Mutation	SNV	A	T	T	OR1M1	c.816A>T	P.K272N
XH_P11	2	100623239	100623239	Missense_Mutation	SNV	T	A	A	AFF3	c.728A>T	P.Q243L
XH_P11	2	10192447	10192447	Missense_Mutation	SNV	A	T	T	KLF11	c.1301A>T	P.E434V
XH_P11	2	102440494	102440494	Silent	SNV	A	T	T	MAP4K4	c.285A>T	P.P95P
XH_P11	2	105859191	105859191	Missense_Mutation	SNV	T	A	A	GPR45	c.876T>A	P.F292L
XH_P11	2	107459548	107459548	Missense_Mutation	SNV	A	T	T	ST6GAL2	c.886T>A	P.C296S
XH_P11	2	109383541	109383541	Silent	SNV	A	T	T	RANBP2	c.6546A>T	P.T2182T
XH_P11	2	120438813	120438813	Silent	SNV	A	T	T	TMEM177	c.384A>T	P.T128T
XH_P11	2	121742321	121742321	Splice_Site	SNV	T	A	A	GLI2	c.1956+2T>A	
XH_P11	2	128377990	128377990	Silent	SNV	C	T	T	MYO7B	c.3396C>T	P.I1132I
XH_P11	2	131674965	131674965	Splice_Site	SNV	T	A	A	ARHGEF4	c.-108-5T>A	
XH_P11	2	133076099	133076099	Silent	SNV	A	T	T	ZNF806	c.1560A>T	P.S520S
XH_P11	2	141806736	141806736	Missense_Mutation	SNV	T	A	A	LRP1B	c.1608A>T	P.R536S
XH_P11	2	144765091	144765091	Missense_Mutation	SNV	T	A	A	GTDC1	c.533A>T	P.K178M
XH_P11	2	1481312	1481312	Missense_Mutation	SNV	A	T	T	TPO	c.1274A>T	P.N425I
XH_P11	2	159035463	159035463	Silent	SNV	T	C	C	CCDC148	c.978A>G	P.K326K
XH_P11	2	162760524	162760524	Nonsense_Mutation	SNV	G	T	T	SLC4A10	c.1453G>T	P.G485*
XH_P11	2	165997248	165997248	Missense_Mutation	SNV	C	A	A	SCN3A	c.1932G>T	P.M644I
XH_P11	2	166613679	166613679	Missense_Mutation	SNV	T	G	G	GALNT3	c.1270A>C	P.S424R
XH_P11	2	168101702	168101702	Missense_Mutation	SNV	T	A	A	XIRP2	c.3134T>A	P.I1045K
XH_P11	2	168105365	168105365	Missense_Mutation	SNV	A	T	T	XIRP2	c.7463A>T	P.E2488V
XH_P11	2	168821245	168821245	Splice_Site	SNV	T	C	C	STK39	c.1499-9A>G	
XH_P11	2	169707374	169707374	Missense_Mutation	SNV	T	A	A	NOSTRIN	c.659T>A	P.F220Y
XH_P11	2	170063022	170063022	Nonsense_Mutation	SNV	A	T	T	LRP2	c.7208T>A	P.L2403*
XH_P11	2	175087791	175087791	Missense_Mutation	SNV	T	A	A	OLA1	c.343A>T	P.S115C
XH_P11	2	179412323	179412323	Missense_Mutation	SNV	A	T	T	TTN	c.89107T>A	P.Y29703N
XH_P11	2	179477760	179477760	Missense_Mutation	SNV	A	T	T	TTN	c.44765T>A	P.V14922E
XH_P11	2	179590334	179590334	Missense_Mutation	SNV	A	T	T	TTN	c.19646T>A	P.V6549E
XH_P11	2	179733959	179733959	Missense_Mutation	SNV	T	A	A	CCDC141	c.2279A>T	P.E760V
XH_P11	2	18112546	18112546	Missense_Mutation	SNV	A	T	T	KCNS3	c.271A>T	P.M91L
XH_P11	2	185801729	185801729	Missense_Mutation	SNV	T	A	A	ZNF804A	c.1606T>A	P.C536S
XH_P11	2	198495806	198495806	Missense_Mutation	SNV	T	A	A	RFTN2	c.857A>T	P.E286V
XH_P11	2	201469525	201469525	Missense_Mutation	SNV	A	T	T	AOX1	c.776A>T	P.Y259F
XH_P11	2	202068479	202068479	Silent	SNV	A	T	T	CASP10	c.711A>T	P.A237A
XH_P11	2	204036749	204036749	Splice_Site	SNV	A	T	T	NBEAL1	c.6111+3A>T	
XH_P11	2	207007452	207007452	Missense_Mutation	SNV	T	A	A	NDUFS1	c.983A>T	P.D328V
XH_P11	2	211163253	211163253	Silent	SNV	T	A	A	MYL1	c.195A>T	P.T65T
XH_P11	2	211465293	211465293	Nonsense_Mutation	SNV	A	T	T	CPS1	c.1582A>T	P.K528*
XH_P11	2	217559329	217559329	Missense_Mutation	SNV	A	T	T	IGFBP5	c.170T>A	P.M57K
XH_P11	2	219090676	219090676	Splice_Site	SNV	A	T	T	ARPC2	c.108A>T	P.A36A
XH_P11	2	233244233	233244233	Missense_Mutation	SNV	T	A	A	ALPP	c.320T>A	P.V107E
XH_P11	2	242794453	242794453	Silent	SNV	G	T	T	PDCD1	c.489C>A	P.A163A

XH_P11	2	31178842	31178842	Splice_Site	SNV	A	T	T	GALNT14	c.483T>A	P.P161P
XH_P11	2	3750010	3750010	Missense_Mutation	SNV	A	T	T	ALLC	c.1033A>T	P.I345F
XH_P11	2	48602480	48602480	Silent	SNV	A	G	G	FOXN2	c.1194A>G	P.K398K
XH_P11	2	50282144	50282144	Nonsense_Mutation	SNV	G	A	A	NRXN1	c.3877C>T	P.R1293*
XH_P11	2	69046378	69046378	Missense_Mutation	SNV	T	A	A	ARHGAP25	c.1127T>A	P.V376E
XH_P11	2	69372485	69372485	Missense_Mutation	SNV	A	T	T	ANTXR1	c.979A>T	P.T327S
XH_P11	2	73800483	73800483	Missense_Mutation	SNV	A	T	T	ALMS1	c.11476A>T	P.S3826C
XH_P11	2	74752852	74752852	Missense_Mutation	SNV	G	A	A	DQX1	c.5C>T	P.T2I
XH_P11	2	79384810	79384810	Missense_Mutation	SNV	A	T	T	REG3A	c.348T>A	P.N116K
XH_P11	2	85785593	85785593	Missense_Mutation	SNV	T	A	A	GGCX	c.509A>T	P.Q170L
XH_P11	2	88396102	88396102	Splice_Site	SNV	A	T	T	SMYD1	c.699-12A>T	
XH_P11	2	95942340	95942340	Splice_Site	SNV	A	G	G	PROM2	c.619-2A>G	
XH_P11	2	97327961	97327961	Missense_Mutation	SNV	A	T	T	FER1L5	c.1028A>T	P.Q343L
XH_P11	2	97845512	97845512	Splice_Site	SNV	A	T	T	ANKRD36	c.1662+9A>T	
XH_P11	20	13029591	13029591	Splice_Site	SNV	A	T	T	SPTLC3	c.118-2A>T	
XH_P11	20	18122902	18122902	Missense_Mutation	SNV	A	T	T	PET117	c.147A>T	P.K49N
XH_P11	20	23017157	23017157	Missense_Mutation	SNV	A	T	T	SSTR4	c.1037A>T	P.Y346F
XH_P11	20	2636125	2636125	Frame_Shift_Del	INDEL	A	-	-	NOP56	c.725delA	P.K242fs
XH_P11	20	33364210	33364210	Missense_Mutation	SNV	T	A	A	NCOA6	c.277A>T	P.S93C
XH_P11	20	34090273	34090273	Missense_Mutation	SNV	A	T	T	CEP250	c.2180A>T	P.H727L
XH_P11	20	35547789	35547789	Missense_Mutation	SNV	T	A	A	SAMHD1	c.830A>T	P.E277V
XH_P11	20	35579920	35579920	Nonsense_Mutation	SNV	T	A	A	SAMHD1	c.127A>T	P.K43*
XH_P11	20	35882691	35882691	Missense_Mutation	SNV	C	T	T	GHRH	c.230G>A	P.R77H
XH_P11	20	3686576	3686576	Missense_Mutation	SNV	T	A	A	SIGLEC1	c.521A>T	P.Q174L
XH_P11	20	43615891	43615891	Missense_Mutation	SNV	A	T	T	STK4	c.479A>T	P.E160V
XH_P11	20	44674996	44674996	Missense_Mutation	SNV	A	G	G	SLC12A5	c.1777A>G	P.M593V
XH_P11	20	47569272	47569272	Missense_Mutation	SNV	A	T	T	ARFGEF2	c.454A>T	P.I152F
XH_P11	20	47689178	47689178	Missense_Mutation	SNV	A	T	T	CSE1L	c.842A>T	P.Q281L
XH_P11	20	58581847	58581847	Splice_Site	SNV	G	A	A	CDH26	c.294+5G>A	
XH_P11	20	61880250	61880250	Splice_Site	SNV	A	T	T	NKAIN4	c.193-3T>A	
XH_P11	20	62195325	62195325	Missense_Mutation	SNV	T	A	A	HELZ2	c.4850A>T	P.Q1617L
XH_P11	21	33717819	33717819	Missense_Mutation	SNV	T	A	A	URB1	c.3934A>T	P.S1312C
XH_P11	21	37418294	37418294	Silent	SNV	T	A	A	SETD4	c.312A>T	P.P104P
XH_P11	21	38511013	38511013	Missense_Mutation	SNV	A	T	T	TTC3	c.1658A>T	P.E553V
XH_P11	21	45825020	45825020	Splice_Site	SNV	A	T	T	TRPM2	c.2539-5A>T	
XH_P11	21	46020705	46020705	Missense_Mutation	SNV	T	A	A	KRTAP10-7	c.169T>A	P.C57S
XH_P11	21	48022264	48022264	Missense_Mutation	SNV	T	A	A	S100B	c.65A>T	P.E22V
XH_P11	22	17621989	17621989	Missense_Mutation	SNV	T	A	A	CECR5	c.616A>T	P.N206Y
XH_P11	22	26291143	26291143	Missense_Mutation	SNV	T	A	A	MYO18B	c.4567T>A	P.F1523I
XH_P11	22	35719787	35719787	Silent	SNV	A	T	T	TOM1	c.429A>T	P.S143S
XH_P11	22	37584212	37584212	Silent	SNV	T	A	A	C1QTNF6	c.42A>T	P.T14T
XH_P11	22	37769948	37769948	Missense_Mutation	SNV	T	G	G	ELFN2	c.1627A>C	P.K543Q
XH_P11	22	39267556	39267556	Splice_Site	SNV	G	A	A	CBX6	c.180-5C>T	
XH_P11	22	40037046	40037046	Silent	SNV	G	A	A	CACNA1I	c.915G>A	P.A305A
XH_P11	22	40140175	40140175	Missense_Mutation	SNV	A	T	T	ENTHD1	c.1333T>A	P.S445T
XH_P11	22	43218426	43218426	Splice_Site	SNV	T	A	A	ARFGAP3	c.541-11A>T	
XH_P11	22	45182343	45182343	Missense_Mutation	SNV	A	T	T	PRR5-ARHGAP8	c.329A>T	P.Q110L
XH_P11	22	46653706	46653706	Silent	SNV	A	T	T	PKDREJ	c.5514T>A	P.S1838S
XH_P11	3	121441230	121441230	Splice_Site	SNV	T	A	A	GOLGB1	c.532-5A>T	
XH_P11	3	124732556	124732556	Missense_Mutation	SNV	T	A	A	HEG1	c.1867A>T	P.T623S
XH_P11	3	128348790	128348790	Splice_Site	SNV	T	A	A	RPN1	c.1036+4A>T	
XH_P11	3	128749534	128749534	Splice_Site	SNV	C	A	A	EFCC1	c.981-12C>A	
XH_P11	3	130107796	130107796	Missense_Mutation	SNV	A	T	T	COL6A5	c.2235A>T	P.R745S
XH_P11	3	133347527	133347527	Missense_Mutation	SNV	C	A	A	TOPBP1	c.2571G>T	P.R857S
XH_P11	3	13381535	13381535	Splice_Site	SNV	T	A	A	NUP210	c.3292-2A>T	
XH_P11	3	142274729	142274729	Silent	SNV	T	A	A	ATR	c.2331A>T	P.P777P
XH_P11	3	154146959	154146959	Missense_Mutation	SNV	T	A	A	GPR149	c.446A>T	P.Q149L
XH_P11	3	156183448	156183448	Missense_Mutation	SNV	G	T	T	KCNAB1	c.544G>T	P.G182W
XH_P11	3	159605645	159605645	Missense_Mutation	SNV	A	T	T	SCHIP1	c.1106A>T	P.Q369L
XH_P11	3	164725748	164725748	Silent	SNV	A	T	T	SI	c.4218T>A	P.T1406T
XH_P11	3	170732381	170732381	Missense_Mutation	SNV	T	A	A	SLC2A2	c.248A>T	P.E83V
XH_P11	3	184009005	184009005	Missense_Mutation	SNV	A	T	T	ECE2	c.1925A>T	P.K642M
XH_P11	3	184099591	184099591	Missense_Mutation	SNV	T	G	G	CHRD	c.599T>G	P.I200S
XH_P11	3	185867920	185867920	Missense_Mutation	SNV	T	A	A	DGKG	c.2260A>T	P.S754C
XH_P11	3	192517238	192517238	Missense_Mutation	SNV	A	T	T	MB21D2	c.413T>A	P.M138K
XH_P11	3	193220392	193220392	Missense_Mutation	SNV	A	T	T	ATP13A4	c.271T>A	P.W91R
XH_P11	3	196626808	196626808	Missense_Mutation	SNV	A	T	T	SENP5	c.1633A>T	P.N545Y
XH_P11	3	36779838	36779838	Missense_Mutation	SNV	T	A	A	DCLK3	c.313A>T	P.S105C
XH_P11	3	46755854	46755854	Missense_Mutation	SNV	T	A	A	PRSS50	c.608A>T	P.Q203L
XH_P11	3	48501901	48501901	Missense_Mutation	SNV	A	T	T	ATRIP	c.1067A>T	P.Q356L
XH_P11	3	49011252	49011252	Splice_Site	SNV	A	T	T	ARIH2	c.888+3A>T	
XH_P11	3	52536089	52536089	Silent	SNV	T	C	C	STAB1	c.399T>C	P.N133N
XH_P11	3	52840986	52840986	Missense_Mutation	SNV	A	T	T	ITIH3	c.2126A>T	P.Y709F
XH_P11	3	68934324	68934324	Splice_Site	SNV	A	T	T	FAM19A4	c.14+2T>A	
XH_P11	3	72428408	72428408	Missense_Mutation	SNV	T	A	A	RYBP	c.299A>T	P.K100I
XH_P11	3	73432931	73432931	Missense_Mutation	SNV	C	T	T	PDZRN3	c.1880G>A	P.G627E
XH_P11	4	100208008	100208008	Splice_Site	SNV	T	A	A	ADH1A	c.258A>T	P.P86P
XH_P11	4	128554246	128554246	Missense_Mutation	SNV	C	A	A	INTU	c.57C>A	P.D19E
XH_P11	4	151142863	151142863	Silent	SNV	T	A	A	DCLK2	c.1167T>A	P.T389T
XH_P11	4	155411997	155411997	Missense_Mutation	SNV	A	T	T	DCHS2	c.511T>A	P.S171T
XH_P11	4	185694238	185694238	Silent	SNV	T	A	A	ACSL1	c.912A>T	P.T304T
XH_P11	4	26483699	26483699	Missense_Mutation	SNV	C	T	T	CCKAR	c.848G>A	P.R283Q
XH_P11	4	42403143	42403143	Missense_Mutation	SNV	A	T	T	SHISA3	c.392A>T	P.Q131L
XH_P11	4	42454003	42454003	Missense_Mutation	SNV	T	A	A	ATP8A1	c.2846A>T	P.Q949L
XH_P11	4	47655624	47655624	Nonsense_Mutation	SNV	T	A	A	CORIN	c.1477A>T	P.R493*
XH_P11	4	5586546	5586546	Missense_Mutation	SNV	T	A	A	EVC2	c.2621A>T	P.Q874L
XH_P11	4	57180394	57180394	Silent	SNV	A	T	T	KIAA1211	c.726A>T	P.A242A
XH_P11	4	66217260	66217260	Silent	SNV	A	T	T	EPHA5	c.2355T>A	P.S785S
XH_P11	4	70823137	70823137	Missense_Mutation	SNV	A	T	T	CSN2	c.527T>A	P.L176Q
XH_P11	4	71844923	71844923	Missense_Mutation	SNV	A	T	T	MOB1B	c.503A>T	P.Y168F
XH_P11	4	77677974	77677974	Silent	SNV	A	T	T	SHROOM3	c.5082A>T	P.T1694T
XH_P11	4	88536818	88536818	Missense_Mutation	SNV	A	T	T	DSPP	c.3004A>T	P.N1002Y
XH_P11	4	89020494	89020494	Missense_Mutation	SNV	T	C	C	ABCG2	c.1474A>G	P.I492V
XH_P11	5	115346583	115346583	Missense_Mutation	SNV	T	G	G	LVRN	c.2239T>G	P.L747V
XH_P11	5	118503451	118503451	Missense_Mutation	SNV	A	T	T	DMXL1	c.5290A>T	P.I1764L
XH_P11	5	121759360	121759360	Missense_Mutation	SNV	G	C	C	SNCAIP	c.928G>C	P.E310Q
XH_P11	5	127681252	127681252	Nonsense_Mutation	SNV	A	T	T	FBN2	c.3014T>A	P.L1005*
XH_P11	5	131994015	131994015	Missense_Mutation	SNV	T	A	A	IL13	c.137T>A	P.L46H

XH_P11	5	136320846	136320846	Missense_Mutation	SNV	T	A	A	SPOCK1	c.974A>T	P.K325M
XH_P11	5	137243545	137243545	Missense_Mutation	SNV	T	A	A	PKD2L2	c.1024T>A	P.W342R
XH_P11	5	13862801	13862801	Missense_Mutation	SNV	A	T	T	DNAH5	c.4652T>A	P.V1551E
XH_P11	5	138661321	138661321	Missense_Mutation	SNV	A	G	G	MATR3	c.2341A>G	P.I781V
XH_P11	5	139227555	139227555	Nonsense_Mutation	SNV	T	A	A	NRG2	c.2302A>T	P.R768*
XH_P11	5	139231183	139231183	Splice_Site	SNV	A	T	T	NRG2	c.1574+6T>A	
XH_P11	5	140255653	140255653	Nonsense_Mutation	SNV	T	A	A	PCDHA12	c.596T>A	P.L199*
XH_P11	5	140263733	140263733	Missense_Mutation	SNV	A	T	T	PCDHA13	c.1880A>T	P.Y627F
XH_P11	5	140347116	140347116	Silent	SNV	T	A	A	PCDHAC2	c.765T>A	P.P255P
XH_P11	5	140865147	140865147	Missense_Mutation	SNV	A	T	T	PCDHGC4	c.407A>T	P.Q136L
XH_P11	5	141036345	141036345	Missense_Mutation	SNV	A	T	T	ARAP3	c.3595T>A	P.C1199S
XH_P11	5	141051138	141051138	Missense_Mutation	SNV	T	A	A	ARAP3	c.1853A>T	P.Q618L
XH_P11	5	145883002	145883002	Missense_Mutation	SNV	A	T	T	TCERG1	c.2384A>T	P.K795I
XH_P11	5	145887511	145887511	Missense_Mutation	SNV	A	T	T	TCERG1	c.2923A>T	P.S975C
XH_P11	5	149677801	149677801	Missense_Mutation	SNV	A	T	T	ARSI	c.686T>A	P.L229H
XH_P11	5	150923958	150923958	Nonsense_Mutation	SNV	T	A	A	FAT2	c.6730A>T	P.R2244*
XH_P11	5	151178857	151178857	Missense_Mutation	SNV	A	G	G	G3BP1	c.826A>G	P.K276E
XH_P11	5	151784182	151784182	Missense_Mutation	SNV	T	A	A	NMUR2	c.493A>T	P.R165W
XH_P11	5	156887267	156887267	Missense_Mutation	SNV	C	G	G	NIPAL4	c.125C>G	P.A42G
XH_P11	5	156899467	156899467	Silent	SNV	T	A	A	NIPAL4	c.900T>A	P.S300S
XH_P11	5	157053510	157053510	Missense_Mutation	SNV	A	T	T	SOX30	c.1185T>A	P.H395Q
XH_P11	5	157053564	157053564	Missense_Mutation	SNV	A	T	T	SOX30	c.1131T>A	P.S377R
XH_P11	5	169472877	169472877	Missense_Mutation	SNV	A	T	T	DOCK2	c.3934A>T	P.I1312F
XH_P11	5	169679439	169679439	Missense_Mutation	SNV	T	A	A	LCP2	c.1322A>T	P.Q441L
XH_P11	5	170323057	170323057	Missense_Mutation	SNV	A	G	G	RANBP17	c.427A>G	P.T143A
XH_P11	5	170669805	170669805	Silent	SNV	A	T	T	RANBP17	c.2757A>T	P.S919S
XH_P11	5	171789808	171789808	Nonsense_Mutation	SNV	T	A	A	SH3PXD2B	c.493A>T	P.K165*
XH_P11	5	180056746	180056746	Missense_Mutation	SNV	C	A	A	FLT4	c.766G>T	P.A256S
XH_P11	5	180056747	180056747	Missense_Mutation	SNV	C	A	A	FLT4	c.765G>T	P.W255C
XH_P11	5	3600293	3600293	Missense_Mutation	SNV	C	A	A	IRX1	c.1231C>A	P.P411T
XH_P11	5	3600294	3600294	Missense_Mutation	SNV	C	A	A	IRX1	c.1232C>A	P.P411Q
XH_P11	5	38489334	38489334	Silent	SNV	T	A	A	LIFR	c.2181A>T	P.A727A
XH_P11	5	38885449	38885449	Splice_Site	SNV	A	T	T	OSMR	c.704-2A>T	
XH_P11	5	39311276	39311276	Silent	SNV	T	A	A	C9	c.1074A>T	P.L358L
XH_P11	5	59284496	59284496	Missense_Mutation	SNV	G	A	A	PDE4D	c.91C>T	P.R31C
XH_P11	5	64521985	64521985	Silent	SNV	A	T	T	ADAMTS6	c.1995T>A	P.R665R
XH_P11	5	68661453	68661453	Nonsense_Mutation	SNV	G	A	A	TAF9	c.112C>T	P.Q38*
XH_P11	5	82808022	82808022	Silent	SNV	C	G	G	VCAN	c.849C>G	P.L283L
XH_P11	5	90670801	90670801	Missense_Mutation	SNV	T	C	C	ARRDC3	c.148A>G	P.I50V
XH_P11	5	95751755	95751755	Missense_Mutation	SNV	A	T	T	PCSK1	c.691T>A	P.Y231N
XH_P11	5	98224856	98224856	Missense_Mutation	SNV	T	C	C	CHD1	c.2267A>G	P.K756R
XH_P11	6	109286307	109286307	Missense_Mutation	SNV	A	T	T	ARMC2	c.1915A>T	P.T639S
XH_P11	6	10930657	10930657	Missense_Mutation	SNV	A	T	T	SYCP2L	c.1543A>T	P.S515C
XH_P11	6	109311861	109311861	Missense_Mutation	SNV	T	A	A	SESN1	c.1234A>T	P.M412L
XH_P11	6	12122866	12122866	Silent	SNV	A	T	T	HIVEP1	c.2838A>T	P.I946I
XH_P11	6	131903778	131903778	Silent	SNV	A	G	G	ARG1	c.483A>G	P.G161G
XH_P11	6	136667043	136667043	Silent	SNV	T	A	A	MAP7	c.2256A>T	P.T752T
XH_P11	6	160328317	160328317	Silent	SNV	G	T	T	MAS1	c.330G>T	P.V110V
XH_P11	6	160494893	160494893	Silent	SNV	C	A	A	IGF2R	c.5052C>A	P.S1684S
XH_P11	6	160668208	160668208	Splice_Site	SNV	T	A	A	SLC22A2	c.957+8A>T	
XH_P11	6	170034587	170034587	Missense_Mutation	SNV	T	A	A	WDR27	c.1657A>T	P.R553W
XH_P11	6	170604533	170604533	Missense_Mutation	SNV	A	T	T	FAM120B	c.40A>T	P.R14W
XH_P11	6	24528345	24528345	Missense_Mutation	SNV	A	T	T	ALDH5A1	c.1294A>T	P.M432L
XH_P11	6	26384085	26384085	Silent	SNV	A	T	T	BTN2A2	c.36A>T	P.P12P
XH_P11	6	26501919	26501919	Missense_Mutation	SNV	T	A	A	BTN1A1	c.181T>A	P.L61M
XH_P11	6	34789692	34789692	Splice_Site	SNV	A	T	T	UHRF1BP1	c.208-2A>T	
XH_P11	6	35810331	35810331	Missense_Mutation	SNV	T	A	A	SRPK1	c.1671A>T	P.E557D
XH_P11	6	37140784	37140784	Missense_Mutation	SNV	A	G	G	PIM1	c.620A>G	P.Y207C
XH_P11	6	42072464	42072464	Silent	SNV	T	A	A	C6orf132	c.3186A>T	P.I1062I
XH_P11	6	42930845	42930845	Missense_Mutation	SNV	A	C	C	CNPY3-GNMT	c.289A>C	P.I97L
XH_P11	6	44241086	44241086	Missense_Mutation	SNV	T	A	A	TMEM151B	c.419T>A	P.L140Q
XH_P11	6	55922643	55922643	Splice_Site	SNV	C	A	A	COL21A1	c.2687-1G>T	
XH_P11	6	56918377	56918377	Frame_Shift_Del	INDEL	G	-	-	KIAA1586	c.999delG	P.E333fs
XH_P11	6	57244790	57244790	Missense_Mutation	SNV	A	G	G	PRIM2	c.551A>G	P.Y184C
XH_P11	6	65531582	65531582	Missense_Mutation	SNV	A	T	T	EYS	c.3199T>A	P.C1067S
XH_P11	6	66115217	66115217	Silent	SNV	A	G	G	EYS	c.906T>C	P.L302L
XH_P11	6	69349336	69349336	Splice_Site	SNV	A	T	T	ADGRB3	c.757+12A>T	
XH_P11	6	71003995	71003995	Missense_Mutation	SNV	T	A	A	COL9A1	c.571A>T	P.S191C
XH_P11	6	7365738	7365738	Missense_Mutation	SNV	T	A	A	CAGE1	c.2156A>T	P.D719V
XH_P11	6	83845497	83845497	Silent	SNV	A	T	T	DOPEY1	c.3003A>T	P.S1001S
XH_P11	6	8413810	8413810	Missense_Mutation	SNV	T	A	A	SLC35B3	c.1178A>T	P.K393M
XH_P11	6	90457105	90457105	Nonsense_Mutation	SNV	T	A	A	MDN1	c.3847A>T	P.R1283*
XH_P11	7	102856898	102856898	Splice_Site	SNV	A	T	T	DPY19L2P2	n.2980+12T>A	
XH_P11	7	103162486	103162486	Missense_Mutation	SNV	C	T	T	RELN	c.7651G>A	P.A2551T
XH_P11	7	106508991	106508991	Missense_Mutation	SNV	T	A	A	PIK3CG	c.985T>A	P.C329S
XH_P11	7	106520073	106520073	Missense_Mutation	SNV	A	T	T	PIK3CG	c.2501A>T	P.H834L
XH_P11	7	117232155	117232155	Missense_Mutation	SNV	T	A	A	CFTR	c.1934T>A	P.M645K
XH_P11	7	120965532	120965532	Splice_Site	SNV	A	T	T	WNT16	c.63A>T	P.L21L
XH_P11	7	121652098	121652098	Missense_Mutation	SNV	T	A	A	PTPRZ1	c.2998T>A	P.S1000T
XH_P11	7	12383835	12383835	Missense_Mutation	SNV	G	T	T	VWDE	c.4064C>A	P.A1355E
XH_P11	7	127013894	127013894	Missense_Mutation	SNV	T	A	A	ZNF800	c.1496A>T	P.Q499L
XH_P11	7	134650060	134650064	Frame_Shift_Del	INDEL	TTGAG	-	-	CALD1	534_1538delTTG	P.L512fs
XH_P11	7	141464013	141464013	Missense_Mutation	SNV	C	G	G	TAS2R3	c.55C>G	P.L19V
XH_P11	7	142574237	142574237	Missense_Mutation	SNV	A	T	T	TRPV6	c.806T>A	P.L269Q
XH_P11	7	143792609	143792609	Missense_Mutation	SNV	T	A	A	OR2A12	c.409T>A	P.W137R
XH_P11	7	146825887	146825887	Missense_Mutation	SNV	A	T	T	CNTNAP2	c.1042A>T	P.T348S
XH_P11	7	1484749	1484749	Silent	SNV	T	A	A	MICALL2	c.957A>T	P.P319P
XH_P11	7	150932468	150932468	Missense_Mutation	SNV	A	T	T	CHPF2	c.574A>T	P.S192C
XH_P11	7	150935660	150935660	Missense_Mutation	SNV	A	T	T	CHPF2	c.2188A>T	P.S730C
XH_P11	7	17869650	17869650	Missense_Mutation	SNV	T	A	A	SNX13	c.1618A>T	P.T540S
XH_P11	7	20441375	20441375	Missense_Mutation	SNV	A	T	T	ITGB8	c.1313A>T	P.K438I
XH_P11	7	23385576	23385576	Silent	SNV	A	T	T	IGF2BP3	c.924T>A	P.T308T
XH_P11	7	24708197	24708197	Silent	SNV	A	T	T	MPP6	c.1032A>T	P.I344I
XH_P11	7	2692550	2692550	Splice_Site	SNV	A	T	T	TTYH3	c.928-2A>T	
XH_P11	7	48559691	48559691	Missense_Mutation	SNV	T	G	G	ABCA13	c.13852T>G	P.F4618V
XH_P11	7	55492962	55492962	Nonsense_Mutation	SNV	A	T	T	LANCL2	c.1024A>T	P.K342*
XH_P11	7	6075189	6075189	Silent	SNV	T	C	C	ANKRD61	c.429T>C	P.N143N
XH_P11	7	6692663	6692663	Missense_Mutation	SNV	A	T	T	ZNF316	c.1436A>T	P.H479L

XH_P11	7	72732888	72732888	Missense_Mutation	SNV	T	A	A	TRIM50	c.659A>T	P.E220V
XH_P11	7	76984861	76984861	Splice_Site	SNV	T	A	A	GSAP	c.1120+11A>T	
XH_P11	7	82583921	82583921	Silent	SNV	A	T	T	PCLO	c.6348T>A	P.L2116L
XH_P11	7	83640552	83640552	Missense_Mutation	SNV	A	T	T	SEMA3A	c.872T>A	P.L291Q
XH_P11	7	87536623	87536623	Silent	SNV	A	T	T	DBF4	c.471A>T	P.T157T
XH_P11	7	91631099	91631099	Missense_Mutation	SNV	A	T	T	AKAP9	c.1868A>T	P.E623V
XH_P11	8	105361105	105361105	Missense_Mutation	SNV	G	A	A	DCSTAMP	c.325G>A	P.G109R
XH_P11	8	124975603	124975603	Silent	SNV	T	A	A	FER1L6	c.162T>A	P.S54S
XH_P11	8	133900474	133900474	Missense_Mutation	SNV	A	T	T	TG	c.2422A>T	P.S808C
XH_P11	8	143866665	143866665	Missense_Mutation	SNV	A	T	T	LY6D	c.359T>A	P.L120Q
XH_P11	8	1626739	1626739	Missense_Mutation	SNV	A	T	T	DLGAP2	c.2366A>T	P.E789V
XH_P11	8	20112463	20112463	Missense_Mutation	SNV	G	C	C	LZTS1	c.230C>G	P.S77C
XH_P11	8	24775411	24775411	Silent	SNV	T	A	A	NEFM	c.915T>A	P.P305P
XH_P11	8	29207762	29207762	Missense_Mutation	SNV	A	T	T	DUSP4	c.34T>A	P.C12S
XH_P11	8	3063060	3063060	Silent	SNV	T	A	A	CSMD1	c.4950A>T	P.I1650I
XH_P11	8	42179909	42179909	Missense_Mutation	SNV	A	T	T	IKBKB	c.1877A>T	P.K626M
XH_P11	8	49644026	49644026	Missense_Mutation	SNV	A	C	C	EFCAB1	c.95T>G	P.F32C
XH_P11	8	52287162	52287162	Silent	SNV	T	A	A	PXDNL	c.3687A>T	P.G1229G
XH_P11	8	72211921	72211921	Silent	SNV	T	A	A	EYA1	c.591A>T	P.T197T
XH_P11	8	77754968	77754968	Nonsense_Mutation	SNV	A	T	T	ZFHX4	c.3472A>T	P.R1158*
XH_P11	8	80677605	80677605	Missense_Mutation	SNV	T	A	A	HEY1	c.745A>T	P.T249S
XH_P11	9	113168934	113168934	Missense_Mutation	SNV	A	T	T	SVEP1	c.8946T>A	P.H2982Q
XH_P11	9	12698449	12698449	Splice_Site	SNV	A	T	T	TYRP1	c.709-2A>T	
XH_P11	9	13206036	13206036	Silent	SNV	T	A	A	MPDZ	c.1353A>T	P.T451T
XH_P11	9	133948110	133948110	Missense_Mutation	SNV	G	A	A	LAMC3	c.3305G>A	P.R1102H
XH_P11	9	133951254	133951254	Silent	SNV	T	A	A	LAMC3	c.3531T>A	P.A1177A
XH_P11	9	139976496	139976496	Missense_Mutation	SNV	T	A	A	UAP1L1	c.1411T>A	P.L471M
XH_P11	9	140082221	140082221	Missense_Mutation	SNV	T	A	A	ANAPC2	c.452A>T	P.E151V
XH_P11	9	140108411	140108411	Splice_Site	SNV	C	T	T	NDOR1	c.411-8C>T	
XH_P11	9	17330679	17330679	Missense_Mutation	SNV	A	T	T	CNTLN	c.1391A>T	P.Q464L
XH_P11	9	21141223	21141223	Missense_Mutation	SNV	A	T	T	IFNW1	c.347T>A	P.L116Q
XH_P11	9	21481239	21481239	Missense_Mutation	SNV	T	A	A	IFNE	c.455A>T	P.H152L
XH_P11	9	33533514	33533514	Silent	SNV	A	T	T	ANKRD18B	c.573A>T	P.A191A
XH_P11	9	38411463	38411463	Silent	SNV	T	A	A	IGFBPL1	c.771A>T	P.T257T
XH_P11	9	79634898	79634898	Nonsense_Mutation	SNV	A	T	T	FOXB2	c.328A>T	P.K110*
XH_P11	9	91159284	91159284	Splice_Site	SNV	T	A	A	NXNL2	c.303-10T>A	
XH_P11	9	93606602	93606602	Splice_Site	SNV	G	A	A	SYK	c.417+5G>A	
XH_P11	9	95778017	95778017	Splice_Site	SNV	A	T	T	FGD3	c.1183-2A>T	
XH_P11	9	990823	990823	Missense_Mutation	SNV	A	T	T	DMRT3	c.1237A>T	P.S413C
XH_P11	X	100613596	100613596	Splice_Site	SNV	A	T	T	BTK	c.974+9T>A	
XH_P11	X	100747535	100747535	Missense_Mutation	SNV	A	T	T	ARMCX4	c.3959A>T	P.E1320V
XH_P11	X	101909212	101909303	Frame_Shift_Del	INDEL	ATAGAGAAC	-	-	GPRASP1	TGAGGATAGAG	P.D125fs
XH_P11	X	102632577	102632577	Missense_Mutation	SNV	A	T	T	BEX3	c.398A>T	P.N133I
XH_P11	X	110391038	110391038	Missense_Mutation	SNV	A	T	T	PAK3	c.458A>T	P.Q153L
XH_P11	X	110973782	110973782	Missense_Mutation	SNV	A	T	T	ALG13	c.1945A>T	P.S649C
XH_P11	X	114425982	114425982	Missense_Mutation	SNV	A	T	T	RBMXL3	c.1978A>T	P.S660C
XH_P11	X	117770326	117770326	Missense_Mutation	SNV	T	A	A	DOCK11	c.3904T>A	P.W1302R
XH_P11	X	118223089	118223089	Missense_Mutation	SNV	A	T	T	KIAA1210	c.2104T>A	P.L702I
XH_P11	X	125954989	125954989	Missense_Mutation	SNV	A	T	T	PRR32	c.368A>T	P.Q123L
XH_P11	X	134156367	134156367	Silent	SNV	C	T	T	FAM127C	c.123G>A	P.P41P
XH_P11	X	135106540	135106540	Missense_Mutation	SNV	A	T	T	SLC9A6	c.1514A>T	P.Y505F
XH_P11	X	135572524	135572524	Missense_Mutation	SNV	T	A	A	BRS3	c.667T>A	P.L223I
XH_P11	X	141291237	141291237	Silent	SNV	A	T	T	MAGEC2	c.537T>A	P.P179P
XH_P11	X	148037128	148037128	Splice_Site	SNV	T	A	A	AFF2	c.1459-5T>A	
XH_P11	X	151815491	151815491	Missense_Mutation	SNV	T	A	A	GABRQ	c.389T>A	P.L130Q
XH_P11	X	152686475	152686475	Missense_Mutation	SNV	A	T	T	ZFP92	c.640A>T	P.S214C
XH_P11	X	152807125	152807125	Splice_Site	SNV	A	T	T	ATP2B3	c.407-2A>T	
XH_P11	X	153416401	153416401	Missense_Mutation	SNV	A	T	T	OPN1LW	c.386A>T	P.E129V
XH_P11	X	18221726	18221726	Missense_Mutation	SNV	T	A	A	BEND2	c.802A>T	P.S268C
XH_P11	X	23751309	23751309	Silent	SNV	T	A	A	ACOT9	c.144A>T	P.V48V
XH_P11	X	24006594	24006594	Missense_Mutation	SNV	T	A	A	KLHL15	c.1259A>T	P.E420V
XH_P11	X	24006595	24006595	Nonsense_Mutation	SNV	C	A	A	KLHL15	c.1258G>T	P.E420*
XH_P11	X	24521566	24521566	Missense_Mutation	SNV	A	T	T	PDK3	c.443A>T	P.Q148L
XH_P12	1	120341157	120341157	Silent	SNV	A	T	T	REG4	c.372T>A	P.G124G
XH_P12	1	149916917	149916917	Silent	SNV	C	T	T	OTUD7B	c.1371G>A	P.E457E
XH_P12	1	159683396	159683396	Splice_Site	SNV	G	A	A	CRP	c.198-3C>T	
XH_P12	1	174973834	174973834	Missense_Mutation	SNV	T	C	C	CACYBP	c.100T>C	P.S34P
XH_P12	1	176564590	176564590	Missense_Mutation	SNV	G	A	A	PAPPA2	c.1850G>A	P.R617H
XH_P12	1	43317025	43317025	Silent	SNV	G	T	T	ZNF691	c.489G>T	P.R163R
XH_P12	10	105750590	105750590	Missense_Mutation	SNV	A	G	G	SLK	c.308A>G	P.N103S
XH_P12	10	48413992	48413992	Silent	SNV	G	A	A	GDF2	c.876C>T	P.S292S
XH_P12	10	97920441	97920441	Silent	SNV	G	T	T	ZNF518A	c.4362G>T	P.V1454V
XH_P12	11	134028234	134028234	Missense_Mutation	SNV	G	A	A	NCAPD3	c.4024C>T	P.P1342S
XH_P12	12	108919308	108919308	Missense_Mutation	SNV	G	C	C	SART3	c.2449C>G	P.L817V
XH_P12	12	14766195	14766195	Missense_Mutation	SNV	A	T	T	GUCY2C	c.3078T>A	P.F1026L
XH_P12	12	40914221	40914221	Splice_Site	SNV	A	G	G	MUC19	c.20531-8A>G	
XH_P12	12	53189718	53189718	Missense_Mutation	SNV	C	T	T	KRT3	c.109G>A	P.G37R
XH_P12	13	20716365	20716365	Missense_Mutation	SNV	C	T	T	GJA3	c.1063G>A	P.V355I
XH_P12	13	25281315	25281315	Missense_Mutation	SNV	C	T	T	ATP12A	c.2342C>T	P.T781I
XH_P12	13	25745485	25745485	Missense_Mutation	SNV	G	T	T	AMER2	c.273C>A	P.D91E
XH_P12	13	48563108	48563108	Missense_Mutation	SNV	C	A	A	SUCLA2	c.280G>T	P.D94Y
XH_P12	13	97485160	97485160	Missense_Mutation	SNV	C	A	A	HS6ST3	c.1124C>A	P.P375H
XH_P12	14	77872347	77872347	Missense_Mutation	SNV	C	G	G	NOXRED1	c.814G>C	P.E272Q
XH_P12	15	42740297	42740297	Missense_Mutation	SNV	A	T	T	ZNF106	c.594T>A	P.S198R
XH_P12	18	24442406	24442406	Nonsense_Mutation	SNV	C	A	A	AQP4	c.187G>T	P.E63*
XH_P12	18	43526704	43526704	Silent	SNV	C	T	T	EPG5	c.1602G>A	P.K534K
XH_P12	19	17435731	17435731	Missense_Mutation	SNV	G	C	C	ANO8	c.3126C>G	P.S1042R
XH_P12	19	20307863	20307863	Missense_Mutation	SNV	G	A	A	ZNF486	c.344G>A	P.C115Y
XH_P12	19	46294273	46294273	Missense_Mutation	SNV	G	T	T	DMWD	c.514C>A	P.Q172K
XH_P12	19	49604664	49604664	Frame_Shift_Ins	INDEL	-	A	A	SNRNP70	c.413dupA	P.R139fs
XH_P12	19	57840518	57840518	Missense_Mutation	SNV	C	T	T	ZNF543	c.1688C>T	P.T563I
XH_P12	19	619773	619773	Splice_Site	SNV	G	A	A	POLRMT	c.2887-8C>T	
XH_P12	2	62100411	62100411	Missense_Mutation	SNV	A	T	T	CCT4	c.845T>A	P.L282H
XH_P12	2	79349129	79349129	Missense_Mutation	SNV	A	T	T	REG1A	c.199A>T	P.M67L
XH_P12	20	15210595	15210595	Missense_Mutation	SNV	A	G	G	MACROD2	c.428A>G	P.H143R
XH_P12	22	41077833	41077833	Silent	SNV	C	T	T	MCHR1	c.1170C>T	P.R390R
XH_P12	3	140406700	140406700	Missense_Mutation	SNV	G	T	T	TRIM42	c.1176G>T	P.K392N
XH_P12	3	180369939	180369939	Splice_Site	INDEL	T	-	-	CCDC39	c.1034+12delA	

XH_P12	3	183226120	183226120	Missense_Mutation	SNV	G	T	T	KLHL6	c.636C>A	P.D212E
XH_P12	4	101344506	101344506	Missense_Mutation	SNV	T	C	C	EMCN	c.432A>G	P.I144M
XH_P12	4	1165095	1165095	Missense_Mutation	SNV	C	T	T	SPON2	c.400G>A	P.G134R
XH_P12	4	126372696	126372696	Missense_Mutation	SNV	A	G	G	FAT4	c.10525A>G	P.M3509V
XH_P12	4	177083301	177083301	Silent	SNV	C	T	T	WDR17	c.2898C>T	P.C966C
XH_P12	4	8229831	8229831	Missense_Mutation	SNV	C	T	T	SH3TC1	c.2182C>T	P.P728S
XH_P12	5	11346648	11346648	Missense_Mutation	SNV	C	A	A	CTNND2	c.1191G>T	P.R397S
XH_P12	6	35211400	35211400	Missense_Mutation	SNV	G	A	A	SCUBE3	c.1936G>A	P.G646S
XH_P12	7	150027864	150027864	Missense_Mutation	SNV	A	G	G	ZBED6CL	c.371A>G	P.Q124R
XH_P12	7	45753535	45753535	Missense_Mutation	SNV	C	T	T	ADCY1	c.3301C>T	P.L1101F
XH_P12	7	4839054	4839054	Silent	SNV	G	A	A	RADIL	c.3183C>T	P.D1061D
XH_P12	7	50057879	50057879	Missense_Mutation	SNV	C	T	T	ZPBP	c.737G>A	P.R246Q
XH_P12	7	64168245	64168245	Missense_Mutation	SNV	G	C	C	ZNF107	c.1563G>C	P.E521D
XH_P12	7	64292175	64292175	Frame_Shift_Ins	INDEL	-	A	A	ZNF138	c.466dupA	P.I156fs
XH_P12	7	92848737	92848737	Missense_Mutation	SNV	A	G	G	HEPACAM2	c.107T>C	P.V36A
XH_P12	8	113317056	113317056	Missense_Mutation	SNV	T	A	A	CSMD3	c.8040A>T	P.K2680N
XH_P12	8	69028159	69028159	Silent	SNV	C	T	T	PREX2	c.3318C>T	P.D1106D
XH_P12	X	12904891	12904891	Missense_Mutation	SNV	A	G	G	TLR7	c.1264A>G	P.R422G
XH_P12	X	77224854	77224854	Silent	SNV	A	G	G	PGAM4	c.282T>C	P.G94G
XH_P13	1	152275306	152275306	Missense_Mutation	SNV	G	A	A	FLG	c.12056C>T	P.A4019V
XH_P13	1	152785216	152785216	Silent	SNV	G	A	A	LCE1B	c.294G>A	P.Q98Q
XH_P13	1	158390649	158390649	Missense_Mutation	SNV	C	T	T	OR10K2	c.8G>A	P.R3Q
XH_P13	1	17085998	17085998	Missense_Mutation	SNV	C	A	A	MST1L	c.899G>T	P.G300V
XH_P13	1	93341931	93341931	Silent	SNV	A	G	G	FAM69A	c.111T>C	P.F37F
XH_P13	10	55582706	55582706	Missense_Mutation	SNV	T	C	C	PCDH15	c.4801A>G	P.I1601V
XH_P13	10	70700833	70700833	Silent	SNV	T	C	C	DDX50	c.1773T>C	P.T591T
XH_P13	11	125848197	125848197	Splice_Site	SNV	A	G	G	CDON	c.3356+2T>C	
XH_P13	11	18313169	18313169	Missense_Mutation	SNV	T	C	C	HPS5	c.1918A>G	P.I640V
XH_P13	11	46709804	46709804	Missense_Mutation	SNV	T	C	C	ARHGAP1	c.236A>G	P.D79G
XH_P13	11	58379120	58379120	Missense_Mutation	SNV	G	C	C	ZFP91	c.763G>C	P.E255Q
XH_P13	11	92600134	92600150	Frame_Shift_Del	INDEL	.GCGGATAA(	-	-	FAT3	05delAGCGGAT	P.A3964fs
XH_P13	12	115110047	115110047	Missense_Mutation	SNV	C	T	T	TBX3	c.1831G>A	P.A611T
XH_P13	12	133367816	133367816	Missense_Mutation	SNV	A	G	G	GOLGA3	c.2533T>C	P.F845L
XH_P13	12	1702388	1702392	Frame_Shift_Del	INDEL	GAGAG	-	-	FBXL14	841_845delCTC	P.L281fs
XH_P13	12	25398284	25398284	Missense_Mutation	SNV	C	T	T	KRAS	c.35G>A	P.G12D
XH_P13	12	57569323	57569334	Missense_Mutation	INDEL	'GGGCATGG/	-	-	LRP1	3644delCATGGA	M1212_G1215del
XH_P13	12	62929428	62929428	Splice_Site	SNV	G	A	A	MON2	c.1831+8G>A	
XH_P13	12	8990075	8990075	Silent	SNV	A	C	C	A2ML1	c.768A>C	P.V256V
XH_P13	13	48939029	48939029	Splice_Site	SNV	G	C	C	RB1	c.862-1G>C	
XH_P13	15	64427018	64427018	Splice_Site	SNV	G	A	A	SNX1	c.1365+12G>A	
XH_P13	16	56901024	56901024	Frame_Shift_Del	INDEL	C	-	-	SLC12A3	c.327delC	P.S110fs
XH_P13	17	7578382	7578382	Nonsense_Mutation	SNV	G	T	T	TP53	c.548C>A	P.S183*
XH_P13	18	32407609	32407609	Missense_Mutation	SNV	C	A	A	DTNA	c.1054C>A	P.P352T
XH_P13	18	44585965	44585965	Silent	SNV	C	T	T	KATNAL2	c.273C>T	P.F91F
XH_P13	19	1507331	1507331	Silent	SNV	C	T	T	ADAMTSL5	c.762G>A	P.A254A
XH_P13	19	30164799	30164799	Missense_Mutation	SNV	T	G	G	PLEKHF1	c.53T>G	P.V18G
XH_P13	19	39522547	39522547	Silent	SNV	G	A	A	FBXO27	c.321C>T	P.R107R
XH_P13	19	4702093	4702093	Missense_Mutation	SNV	G	C	C	DPP9	c.958C>G	P.P320A
XH_P13	2	113588157	113588157	Splice_Site	SNV	C	T	T	IL1B	c.598-7G>A	
XH_P13	2	180348009	180348009	Silent	SNV	A	T	T	ZNF385B	c.432T>A	P.S144S
XH_P13	2	198266834	198266834	Missense_Mutation	SNV	T	C	C	SF3B1	c.2098A>G	P.K700E
XH_P13	2	241684943	241684943	Splice_Site	SNV	C	A	A	KIF1A	c.3375-9G>T	
XH_P13	2	98408917	98408917	Missense_Mutation	SNV	T	A	A	TMEM131	c.4076A>T	P.D1359V
XH_P13	20	31383295	31383295	Missense_Mutation	SNV	T	G	G	DNMT3B	c.1207T>G	P.C403G
XH_P13	20	33337761	33337761	Frame_Shift_Del	INDEL	C	-	-	NCOA6	c.2237delG	P.G746fs
XH_P13	21	45713715	45713715	Missense_Mutation	SNV	C	T	T	AIRE	c.1322C>T	P.T441M
XH_P13	3	124732416	124732416	Missense_Mutation	INDEL	-	AGAGGAGGAGG	AGAGGAGGAGG	HEG1	)06dupCTCCTCC	).S665_S669dup
XH_P13	3	127396614	127396614	Silent	SNV	C	T	T	ABTB1	c.531C>T	P.A177A
XH_P13	3	49753557	49753557	Missense_Mutation	SNV	G	T	T	RNF123	c.3362G>T	P.R1121L
XH_P13	3	7620616	7620616	Missense_Mutation	SNV	A	T	T	GRM7	c.2023A>T	P.T675S
XH_P13	4	119219892	119219892	Silent	SNV	A	G	G	PRSS12	c.1833T>C	P.N611N
XH_P13	4	146059690	146059690	Missense_Mutation	SNV	T	C	C	OTUD4	c.2042A>G	P.N681S
XH_P13	5	150656998	150656998	Missense_Mutation	SNV	G	A	A	SLC36A3	c.1492C>T	P.P498S
XH_P13	6	143784108	143784108	Missense_Mutation	SNV	G	C	C	PEX3	c.261G>C	P.E87D
XH_P13	7	148487454	148487454	Missense_Mutation	SNV	G	A	A	CUL1	c.1727G>A	P.G576D
XH_P13	7	18801895	18801895	Missense_Mutation	SNV	T	C	C	HDAC9	c.2159T>C	P.L720P
XH_P13	7	99129330	99129330	Missense_Mutation	SNV	T	C	C	ZKSCAN5	c.1978T>C	P.S660P
XH_P13	8	125463583	125463583	Missense_Mutation	SNV	A	C	C	TRMT12	c.415A>C	P.N139H
XH_P13	8	135524749	135524749	Silent	SNV	C	T	T	ZFAT	c.3294G>A	P.A1098A
XH_P13	X	149867658	149867658	Splice_Site	INDEL	T	-	-	MTMR1	c.147-3delT	
XH_P13	X	37312611	37312611	Frame_Shift_Del	INDEL	C	-	-	PRRG1	c.401delC	P.P134fs
XH_P13	X	47045136	47045136	Nonsense_Mutation	SNV	C	T	T	RBM10	c.2377C>T	P.R793*
XH_P15	1	110591793	110591793	Frame_Shift_Del	INDEL	T	-	-	STRIP1	c.1437delT	P.I479fs
XH_P15	1	1226722	1226722	Missense_Mutation	INDEL	-	GCA	GCA	SCNN1D	2143_2144insA(	).L714_L715insQ
XH_P15	1	1875802	1875802	Frame_Shift_Ins	INDEL	-	CGGT	CGGT	CFAP74	450_2453dupA(	P.L819fs
XH_P15	1	26509741	26509741	Frame_Shift_Del	INDEL	C	-	-	CNKSR1	c.678delC	P.D226fs
XH_P15	1	46685774	46685774	Missense_Mutation	SNV	C	T	T	LURAP1	c.602C>T	P.P201L
XH_P15	1	6647539	6647539	Frame_Shift_Ins	INDEL	-	C	C	ZBTB48	c.1230dupC	P.T411fs
XH_P15	1	877515	877516	Frame_Shift_Del	INDEL	GG	-	-	SAMD11	c.870_871delGG	P.E291fs
XH_P15	1	93299181	93299181	Frame_Shift_Ins	INDEL	-	AT	AT	RPL5	c.155_156dupT/	P.V53fs
XH_P15	10	45939270	45939270	Missense_Mutation	SNV	C	A	A	ALOX5	c.1668C>A	P.F556L
XH_P15	10	89711879	89711882	Frame_Shift_Del	INDEL	TAAC	-	-	PTEN	499_502delACT	P.T167fs
XH_P15	10	97444362	97444362	Frame_Shift_Ins	INDEL	-	TC	TC	TCTN3	.987_988dupG/	P.T330fs
XH_P15	10	99531082	99531108	Missense_Mutation	INDEL	AACTGCACG	-	-	SFRP5	CATCGCCGTGC	P.C162_L170del
XH_P15	11	102714170	102714171	Splice_Site	INDEL	TA	-	-	MMP3	.05+2_105+3delTA	
XH_P15	11	118897733	118897733	Frame_Shift_Ins	INDEL	-	A	A	SLC37A4	c.697dupT	P.Y233fs
XH_P15	11	18723515	18723515	Frame_Shift_Ins	INDEL	-	G	G	TMEM86A	c.685dupG	P.E229fs
XH_P15	11	1905812	1905812	Splice_Site	INDEL	-	TA	TA	LSP1	31+3_531+4dupAT	
XH_P15	11	28104442	28104442	Missense_Mutation	SNV	T	A	A	KIF18A	c.1223A>T	P.K408M
XH_P15	11	34654158	34654158	Frame_Shift_Ins	INDEL	-	GATT	GATT	EHF	:.24_27dupATT(	P.V10fs
XH_P15	11	36310918	36310918	Frame_Shift_Ins	INDEL	-	GCCT	GCCT	COMMD9	:.44_45insAGG(	P.L16fs
XH_P15	11	63586402	63586402	Missense_Mutation	SNV	G	A	A	C11orf84	c.862G>A	P.D288N
XH_P15	11	64543964	64543964	Splice_Site	INDEL	-	T	T	SF1	c.535+5dupA	
XH_P15	11	67410314	67410314	Frame_Shift_Ins	INDEL	-	GG	GG	ACY3	:.839_840dupC(	P.V281fs
XH_P15	11	71955041	71955041	Frame_Shift_Ins	INDEL	-	GAC	GAC	PHOX2A	c.7_8insGTC	P.Y3delinsCH
XH_P15	11	75153602	75153602	Frame_Shift_Ins	INDEL	-	GCT	GCT	GDPD5	.970_972dupAG	P.S324dup
XH_P15	11	76922242	76922242	Missense_Mutation	INDEL	-	ACC	ACC	MYO7A	6098_6099insC(	).E2033delinsDQ
XH_P15	12	11061483	11061483	Frame_Shift_Ins	INDEL	-	CAA	CAA	TAS2R13	.412_414dupTT	P.L138dup

XH_P15	12	30872054	30872054	Frame_Shift_Ins	INDEL	-	ACT	ACT	CAPRIN2	2251_2253dupA	P.S751dup
XH_P15	12	48173947	48173947	Frame_Shift_Ins	INDEL	-	TACT	TACT	SLC48A1	325_328dupTA	P.Y110fs
XH_P15	12	4920326	4920326	Missense_Mutation	SNV	G	C	C	KCNA6	c.1119G>C	P.M373I
XH_P15	12	57039021	57039021	Frame_Shift_Ins	INDEL	-	TGG	TGG	ATP5B	.241_243dupCC	P.P81dup
XH_P15	12	92537947	92537947	Frame_Shift_Ins	INDEL	-	T	T	BTG1	c.424dupA	P.M142fs
XH_P15	13	23911365	23911365	Missense_Mutation	SNV	G	A	A	SACS	c.6209C>T	P.P2070L
XH_P15	13	49050843	49050843	Nonsense_Mutation	SNV	G	T	T	RB1	c.2527G>T	P.E843*
XH_P15	13	76055493	76055504	Missense_Mutation	INDEL	GTGAGGTCGT	-	-	TBC1D4	411delCACGAC	P.H134_T137del
XH_P15	13	78492216	78492216	Splice_Site	SNV	C	T	T	EDNRB	c.483+10G>A	
XH_P15	14	104387272	104387272	Splice_Site	INDEL	-	T	T	C14orf2	c.51+4dupA	
XH_P15	14	20849516	20849516	Nonsense_Mutation	SNV	G	A	A	TEP1	c.4279C>T	P.R1427*
XH_P15	14	21541273	21541273	Frame_Shift_Ins	INDEL	-	C	C	ARHGEF40	c.78dupC	P.T27fs
XH_P15	14	24528128	24528128	Frame_Shift_Ins	INDEL	-	CT	CT	CARMIL3	1505_1506dup	P.A503fs
XH_P15	14	24802202	24802202	Splice_Site	INDEL	-	AGGG	AGGG	ADCY4	0-12_160-9dupCCCT	
XH_P15	14	31358824	31358824	Frame_Shift_Ins	INDEL	-	TCAC	TCAC	COCH	482_1485dupC	P.I496fs
XH_P15	14	31790758	31790758	Frame_Shift_Ins	INDEL	-	AACT	AACT	HEATR5A	3915_3916insAG	P.V1306fs
XH_P15	14	50053052	50053053	Frame_Shift_Del	INDEL	GC	-	-	RPS29	c.12_13delGC	P.Q5fs
XH_P15	14	50300137	50300137	Splice_Site	INDEL	-	T	T	NEMF	c.735+3dupA	
XH_P15	14	50555913	50555913	Splice_Site	SNV	A	G	G	LINC01599	n.48T>C	
XH_P15	14	50952325	50952325	Frame_Shift_Ins	INDEL	-	CA	CA	MAP4K5	c.291_292dupT	P.G98fs
XH_P15	14	55636160	55636162	Missense_Mutation	INDEL	GTA	-	-	DLGAP5	1503_1505delT	P.T502del
XH_P15	14	92409302	92409303	Frame_Shift_Del	INDEL	TA	-	-	FBLN5	c.20_21delTA	P.I7fs
XH_P15	15	101833228	101833228	Splice_Site	INDEL	-	C	C	SNRPA1	c.230+1dupG	
XH_P15	15	23812308	23812308	Missense_Mutation	SNV	G	T	T	MKRN3	c.1379G>T	P.S460I
XH_P15	15	38235601	38235601	Splice_Site	INDEL	-	A	A	TMCO5A	c.627+5dupA	
XH_P15	15	62237949	62237949	Missense_Mutation	SNV	T	C	C	VPS13C	c.5113A>G	P.I1705V
XH_P15	15	62299587	62299587	Silent	SNV	A	G	G	VPS13C	c.1210T>C	P.L404L
XH_P15	15	62299588	62299588	Silent	SNV	C	T	T	VPS13C	c.1209G>A	P.Q403Q
XH_P15	15	63414237	63414237	Missense_Mutation	INDEL	-	GGC	GGC	LACTB	.168_170dupGG	P.A57dup
XH_P15	15	68501975	68501975	Frame_Shift_Ins	INDEL	-	A	A	CLN6	c.664dupT	P.W222fs
XH_P15	15	74494557	74494557	Frame_Shift_Del	INDEL	A	-	-	STRA6	c.52delT	P.Y18fs
XH_P15	15	89443007	89443007	Frame_Shift_Del	INDEL	G	-	-	MFGE8	c.774delC	P.I259fs
XH_P15	16	2358443	2358443	Splice_Site	INDEL	-	CACT	CACT	ABCA3	5+4_1285+7dupAGTG	
XH_P15	16	68867322	68867322	Frame_Shift_Ins	INDEL	-	AGGA	AGGA	CDH1	570_2573dupAG	P.D858fs
XH_P15	16	71706204	71706204	Frame_Shift_Ins	INDEL	-	A	A	PHLPP2	c.1492dupT	P.Y498fs
XH_P15	17	15848840	15848842	Missense_Mutation	INDEL	TCT	-	-	ADORA2B	c.280_282delTT	P.F94del
XH_P15	17	16075109	16075114	Splice_Site	INDEL	ATATTT	-	-	NCOR1	+3_435+8delAAATAT	
XH_P15	17	38560458	38560458	Missense_Mutation	SNV	C	A	A	TOP2A	c.2229G>T	P.K743N
XH_P15	17	40336409	40336409	Frame_Shift_Ins	INDEL	-	AGCA	AGCA	HCRT	155_158dupTG	P.H54fs
XH_P15	17	43215332	43215332	Frame_Shift_Ins	INDEL	-	C	C	ACBD4	c.630dupC	P.V211fs
XH_P15	17	43552749	43552749	Frame_Shift_Ins	INDEL	-	T	T	PLEKHM1	c.639dupA	P.L214fs
XH_P15	17	5071260	5071260	Missense_Mutation	SNV	C	T	T	USP6	c.3070C>T	P.R1024W
XH_P15	17	5418828	5418828	Missense_Mutation	SNV	A	G	G	NLRP1	c.3845T>C	P.M1282T
XH_P15	17	62045456	62045456	Silent	SNV	G	A	A	SCN4A	c.963C>T	P.N321N
XH_P15	17	72301563	72301563	Missense_Mutation	SNV	C	T	T	DNAI2	c.1193C>T	P.S398L
XH_P15	17	73102118	73102119	Frame_Shift_Del	INDEL	GC	-	-	SLC16A5	.1508_1509delG	P.S503fs
XH_P15	17	73235158	73235158	Frame_Shift_Ins	INDEL	-	CACT	CACT	GGA3	567_1570dupA	P.A524fs
XH_P15	17	79418847	79418847	Missense_Mutation	SNV	G	A	A	BAHCC1	c.4055G>A	P.R1352H
XH_P15	17	80656339	80656339	Frame_Shift_Ins	INDEL	-	GGCC	GGCC	RAB40B	130_133dupGG	P.H45fs
XH_P15	18	12014299	12014301	Missense_Mutation	INDEL	GCG	-	-	IMPA2	.419_421delGG	P.R140del
XH_P15	18	44559687	44559687	Missense_Mutation	SNV	G	A	A	TCEB3B	c.1949C>T	P.T650M
XH_P15	18	51888540	51888540	Frame_Shift_Ins	INDEL	-	T	T	C18orf54	c.812dupT	P.P272fs
XH_P15	19	10400734	10400759	Frame_Shift_Del	INDEL	TGCGCCGGG	-	-	ICAM5	GCTGCGCCGGG	P.G7fs
XH_P15	19	10935797	10935797	Frame_Shift_Ins	INDEL	-	G	G	DNM2	c.1960dupG	P.E654fs
XH_P15	19	11485584	11485584	Frame_Shift_Ins	INDEL	-	CTA	CTA	SWSAP1	.166_168dupCT	P.L56dup
XH_P15	19	12958818	12958818	Frame_Shift_Ins	INDEL	-	CC	CC	MAST1	c.722_723dupC	P.T242fs
XH_P15	19	15536187	15536189	Missense_Mutation	INDEL	AGC	-	-	WIZ	1472_1474delG	G491_Y492delinsD
XH_P15	19	18643010	18643024	Missense_Mutation	INDEL	CACACCCCC	-	-	FKBP8	08delCTTGGGG	P.L399_A403del
XH_P15	19	22379432	22379432	Silent	SNV	A	G	G	ZNF676	c.4T>C	P.L2L
XH_P15	19	30106230	30106230	Silent	SNV	T	G	G	POP4	c.606T>G	P.L202L
XH_P15	19	35715965	35715965	Frame_Shift_Ins	INDEL	-	TCCT	TCCT	FAM187B	869_872dupAG	P.L292fs
XH_P15	19	36017858	36017867	Frame_Shift_Del	INDEL	ATGAACTGCT	-	-	SBSN	_1326delAGCA	P.I439fs
XH_P15	19	36486332	36486332	Frame_Shift_Ins	INDEL	-	CT	CT	SDHAF1	c.157_158dupCT	P.Y54fs
XH_P15	19	36501809	36501809	Missense_Mutation	SNV	C	T	T	ALKBH6	c.323G>A	P.G108E
XH_P15	19	39361489	39361489	Missense_Mutation	SNV	C	T	T	RINL	c.745G>A	P.D249N
XH_P15	19	43679507	43679507	Frame_Shift_Ins	INDEL	-	T	T	PSG5	c.823dupA	P.I275fs
XH_P15	19	47192929	47192933	Frame_Shift_Del	INDEL	GTTCA	-	-	PRKD2	832_1836delTG	P.V611fs
XH_P15	19	48945578	48945578	Frame_Shift_Del	INDEL	G	-	-	GRIN2D	c.2613delG	P.W871fs
XH_P15	19	49951302	49951302	Frame_Shift_Ins	INDEL	-	GGTA	GGTA	PIH1D1	351_354dupTA	P.A119fs
XH_P15	19	50919981	50919982	Splice_Site	INDEL	GT	-	-	POLD1	067+2_3067+3delTG	
XH_P15	19	58420589	58420589	Frame_Shift_Ins	INDEL	-	TT	TT	ZNF417	.1053_1054insA	P.H352fs
XH_P15	19	59065565	59065572	Frame_Shift_Del	INDEL	GAACAATA	-	-	CHMP2A	8_15delTATTGT	P.L3fs
XH_P15	2	101670669	101670670	Frame_Shift_Del	INDEL	AG	-	-	TBC1D8	c.486_487delCT	P.Y162fs
XH_P15	2	113332737	113332737	Frame_Shift_Ins	INDEL	-	CTAC	CTAC	POLR1B	672_2675dupCT	P.P893fs
XH_P15	2	145157709	145157709	Nonsense_Mutation	SNV	T	A	A	ZEB2	c.973A>T	P.K325*
XH_P15	2	160879231	160879233	Missense_Mutation	INDEL	TAA	-	-	PLA2R1	1237_1239delT	P.L413del
XH_P15	2	191845336	191845336	Splice_Site	INDEL	C	-	-	STAT1	c.1632+10delG	
XH_P15	2	197738399	197738399	Splice_Site	INDEL	-	ATTA	ATTA	PGAP1	8+6_978+9dupTAAT	
XH_P15	2	242814426	242814428	Missense_Mutation	INDEL	TCA	-	-	RTP5	c.721_723delAT	P.I241del
XH_P15	2	48698513	48698518	Splice_Site	INDEL	ACCCCC	-	-	PPP1R21	+7_1088+12delACCCCC	
XH_P15	2	73635784	73635784	Frame_Shift_Ins	INDEL	-	A	A	ALMS1	c.360dupA	P.V121fs
XH_P15	2	9637293	9637293	Missense_Mutation	SNV	C	T	T	ADAM17	c.1733G>A	P.C578Y
XH_P15	2	97427091	97427091	Frame_Shift_Ins	INDEL	-	AGCT	AGCT	CNNM4	356_359dupAG	P.V121fs
XH_P15	20	10256220	10256220	Splice_Site	INDEL	-	C	C	SNAP25	.72+9_72+10insC	
XH_P15	20	10654168	10654169	Frame_Shift_Del	INDEL	GG	-	-	JAG1	c.10_11delCC	P.P4fs
XH_P15	20	21367645	21367645	Splice_Site	INDEL	-	TAAA	TAAA	XRN2	1+3_3021+6dupAAAT	
XH_P15	20	33077677	33077677	Frame_Shift_Ins	INDEL	-	GCC	GCC	ITCH	2389_2391dupG	P.A797dup
XH_P15	20	43552884	43552885	Frame_Shift_Del	INDEL	TT	-	-	PABPC1L	c.959_960delTT	P.I320fs
XH_P15	20	44598226	44598226	Frame_Shift_Ins	INDEL	-	C	C	ZNF335	c.305dupG	P.P103fs
XH_P15	20	46262946	46262946	Splice_Site	INDEL	A	-	-	NCOA3	c.1112+7delA	
XH_P15	20	47739677	47739677	Frame_Shift_Ins	INDEL	-	GT	GT	STAU1	c.691_692dupA	P.L232fs
XH_P15	20	60902411	60902411	Frame_Shift_Ins	INDEL	-	CAC	CAC	LAMA5	4987_4989dupG	P.V1663dup
XH_P15	20	62520589	62520593	Frame_Shift_Del	INDEL	AAGGT	-	-	TPD52L2	50_450+4delGG	P.K150fs
XH_P15	21	37417936	37417936	Frame_Shift_Ins	INDEL	-	CTGA	CTGA	SETD4	.669_670insTCA	P.D224fs
XH_P15	21	38114037	38114038	Frame_Shift_Del	INDEL	CA	-	-	SIM2	c.871_872delA	P.T291fs
XH_P15	22	20307492	20307524	In_Frame_Del	INDEL	GCGCTCCATG	-	-	DGCR6L	CCGCGCCATGGA	P.M1_A7del
XH_P15	22	26181425	26181425	Frame_Shift_Ins	INDEL	-	AGC	AGC	MYO18B	2347_2349dupA	P.S784dup
XH_P15	22	38083967	38083967	Missense_Mutation	SNV	C	A	A	NOL12	c.134C>A	P.A45D

XH_P15	22	46932215	46932229	Missense_Mutation	INDEL	ATGTAATAGC	-	-	CELSR1	3delTGAGCTAT	P.V280_M284del
XH_P15	3	101474341	101474341	Missense_Mutation	SNV	C	G	G	CEP97	c.796C>G	P.L266V
XH_P15	3	113594396	113594396	Frame_Shift_Ins	INDEL	-	TATG	TATG	GRAMD1C	327_330dupATC	P.F111fs
XH_P15	3	179298703	179298708	Missense_Mutation	INDEL	TGTTCA	-	-	ACTL6A	353_858delGTTC	P.V285_H286del
XH_P15	3	180320212	180320212	Splice_Site	INDEL	-	AA	AA	TTC14	161+3_161+4dupAA	
XH_P15	3	40352489	40352489	Frame_Shift_Ins	INDEL	-	T	T	EIF1B	c.138dupT	P.V47fs
XH_P15	3	42727133	42727133	Missense_Mutation	SNV	C	T	T	KLHL40	c.23C>T	P.A8V
XH_P15	3	45823712	45823712	Frame_Shift_Ins	INDEL	-	T	T	SLC6A20	c.124dupA	P.S42fs
XH_P15	3	47084059	47084059	Frame_Shift_Del	INDEL	C	-	-	SETD2	c.7230delG	P.I2411fs
XH_P15	3	47374726	47374726	Frame_Shift_Ins	INDEL	-	C	C	KLHL18	c.682dupC	P.R228fs
XH_P15	3	58094969	58094969	Frame_Shift_Del	INDEL	T	-	-	FLNB	c.2119delT	P.S707fs
XH_P15	3	58154312	58154315	Frame_Shift_Del	INDEL	TAAC	-	-	FLNB	7439_7442delAC	P.N2480fs
XH_P15	4	106190765	106190765	Splice_Site	SNV	A	G	G	TET2	c.4045-2A>G	
XH_P15	4	138450913	138450913	Missense_Mutation	SNV	G	C	C	PCDH18	c.2330C>G	P.S777W
XH_P15	4	139980616	139980622	Frame_Shift_Del	INDEL	TGACTAC	-	-	ELF2	30_1036delGTAC	P.V344fs
XH_P15	4	184367966	184367981	Frame_Shift_Del	INDEL	CAGACCAGTC	-	-	CDKN2AIP	44delTCCCAGAC	P.S377fs
XH_P15	4	959035	959035	Frame_Shift_Ins	INDEL	-	CC	CC	DGKQ	1661_1662dupG	P.L555fs
XH_P15	5	127710331	127710331	Silent	SNV	A	T	T	FBN2	c.2085T>A	P.R695R
XH_P15	5	33944831	33944831	Silent	SNV	G	A	A	SLC45A2	c.1515C>T	P.V505V
XH_P15	5	53814873	53814880	Frame_Shift_Del	INDEL	CCAGCCAC	-	-	SNX18	5_1102delCCAC	P.H366fs
XH_P15	6	116381223	116381223	Missense_Mutation	SNV	G	C	C	FRK	c.252C>G	P.H84Q
XH_P15	6	121427164	121427164	Splice_Site	INDEL	-	T	T	TBC1D32	c.3465+4dupA	
XH_P15	6	130762171	130762197	Missense_Mutation	INDEL	ATTGGCAGCA	-	-	TMEM200A	GATTGGCAGCAA	P.R204_S212del
XH_P15	6	134493912	134493912	Splice_Site	INDEL	-	CT	CT	SGK1	835-2_835-1dupAG	
XH_P15	6	136476805	136476808	Frame_Shift_Del	INDEL	TGCT	-	-	PDE7B	620_623delTGC	P.L207fs
XH_P15	6	162683710	162683710	Missense_Mutation	SNV	C	T	T	PARK2	c.259G>A	P.D87N
XH_P15	6	17794483	17794483	Frame_Shift_Ins	INDEL	-	TAGT	TAGT	KIF13A	3218_3219insAC	P.Y1073fs
XH_P15	6	24495516	24495516	Missense_Mutation	SNV	G	A	A	ALDH5A1	c.292G>A	P.A98T
XH_P15	6	26022047	26022047	Frame_Shift_Ins	INDEL	-	TA	TA	HIST1H4A	c.142_143insAT	P.S48fs
XH_P15	6	28333382	28333382	Frame_Shift_Ins	INDEL	-	GGCA	GGCA	ZKSCAN3	938_941dupGGC	P.H314fs
XH_P15	6	43400951	43400951	Frame_Shift_Ins	INDEL	-	CT	CT	ABCC10	1236_1237dupC	P.Y413fs
XH_P15	6	44253899	44253899	Silent	SNV	G	A	A	TCTE1	c.648C>T	P.S216S
XH_P15	7	128482269	128482269	Splice_Site	INDEL	-	G	G	FLNC	c.2122-13dupG	
XH_P15	7	138713681	138713681	Frame_Shift_Ins	INDEL	-	C	C	ZC3HAV1L	c.526dupG	P.E176fs
XH_P15	7	13971133	13971133	Missense_Mutation	SNV	C	T	T	ETV1	c.796G>A	P.D266N
XH_P15	7	150164095	150164095	Silent	SNV	C	T	T	GIMAP8	c.309C>T	P.I103I
XH_P15	7	30068407	30068407	Frame_Shift_Ins	INDEL	-	CT	CT	PLEKHA8	c.30_31dupCT	P.Y11fs
XH_P15	7	44104864	44104864	Nonsense_Mutation	SNV	C	A	A	PGAM2	c.265G>T	P.E89*
XH_P15	7	73118738	73118738	Frame_Shift_Ins	INDEL	-	TGAG	TGAG	STX1A	.298_299insCTC	P.I100fs
XH_P15	7	89856600	89856600	Frame_Shift_Ins	INDEL	-	TA	TA	STEAP2	c.811_812dupTA	P.L272fs
XH_P15	7	92761723	92761723	Nonsense_Mutation	SNV	G	A	A	SAMD9L	c.3562C>T	P.R1188*
XH_P15	8	104312436	104312436	Missense_Mutation	SNV	A	C	C	FZD6	c.101A>C	P.K34T
XH_P15	8	144944735	144944742	Frame_Shift_Del	INDEL	CCGGCCTC	-	-	EPPK1	0_2687delGAGG	P.E894fs
XH_P15	8	27145644	27145644	Frame_Shift_Del	INDEL	A	-	-	TRIM35	c.786delT	P.C262fs
XH_P15	8	27145645	27145645	Splice_Site	SNV	C	T	T	TRIM35	c.786-1G>A	
XH_P15	8	30585153	30585153	Frame_Shift_Ins	INDEL	-	GA	GA	GSR	c.199_200insTC	P.Y67fs
XH_P15	8	53045681	53045681	Missense_Mutation	SNV	G	A	A	ST18	c.2380C>T	P.R794C
XH_P15	8	72756341	72756341	Missense_Mutation	SNV	C	T	T	MSC	c.73G>A	P.A25T
XH_P15	8	90965619	90965622	Frame_Shift_Del	INDEL	TAAC	-	-	NBN	1695_1698delGT	P.Q565fs
XH_P15	9	129376788	129376788	Frame_Shift_Ins	INDEL	-	CG	CG	LMX1B	c.60_61insCG	P.C21fs
XH_P15	9	135979141	135979141	Frame_Shift_Ins	INDEL	-	AGTC	AGTC	RALGDS	582_1585dupGA	P.Y529fs
XH_P15	9	139686484	139686484	Splice_Site	INDEL	-	GG	GG	TMEM141	05+4_205+5dupGG	
XH_P15	9	139835501	139835506	Missense_Mutation	INDEL	AGCAGC	-	-	FBXW5	75_1580delGCT	P.L526_L527del
XH_P15	9	140331030	140331032	Missense_Mutation	INDEL	GCT	-	-	ENTPD8	.727_729delAG	P.S243del
XH_P15	9	19126149	19126150	Frame_Shift_Del	INDEL	GG	-	-	PLIN2	c.188_189delCC	P.T63fs
XH_P15	9	37800650	37800650	Splice_Site	INDEL	-	TTG	TTG	DCAF10	20+6_20+7insTTG	
XH_P15	9	37800650	37800650	Splice_Site	INDEL	T	-	-	DCAF10	c.20+6delT	
XH_P15	9	86571117	86571117	Frame_Shift_Ins	INDEL	-	AGT	AGT	C9orf64	.296_298dupAC	P.Y99dup
XH_P15	9	97844955	97844955	Frame_Shift_Ins	INDEL	-	GAT	GAT	C9orf3	2419_2421dupG	P.D807dup
XH_P15	X	41204684	41204684	Missense_Mutation	INDEL	-	ATA	ATA	DDX3X	1199_1201dupA	P.Y400_I401insN
XH_P15	X	57021122	57021126	Frame_Shift_Del	INDEL	ATTTG	-	-	SPIN3	255_259delCAA	P.K86fs
XH_P15	X	68058601	68058601	Frame_Shift_Ins	INDEL	-	ACAT	ACAT	EFNB1	.273_274insTAC	P.V92fs
XH_P16	1	198697459	198697459	Splice_Site	SNV	T	A	A	PTPRC	c.1721-10T>A	
XH_P16	1	54509117	54509117	Missense_Mutation	SNV	C	T	T	TMEM59	c.472G>A	P.A158T
XH_P16	1	8397927	8397927	Missense_Mutation	SNV	A	G	G	SLC45A1	c.1751A>G	P.E584G
XH_P16	10	103900368	103900368	Silent	SNV	C	A	A	PPRC1	c.2103C>A	P.A701A
XH_P16	10	82267045	82267045	Missense_Mutation	SNV	T	G	G	TSPAN14	c.194T>G	P.V65G
XH_P16	10	88203077	88203077	Silent	SNV	G	C	C	WAPL	c.3348C>G	P.A1116A
XH_P16	10	88768297	88768297	Silent	SNV	G	A	A	AGAP11	c.288G>A	P.E96E
XH_P16	11	50003751	50003751	Missense_Mutation	SNV	A	C	C	OR4C12	c.287T>G	P.M96R
XH_P16	12	8375278	8375278	Missense_Mutation	SNV	C	T	T	FAM90A1	c.535G>A	P.V179I
XH_P16	13	20637066	20637066	Missense_Mutation	SNV	G	A	A	ZMYM2	c.2992G>A	P.V998I
XH_P16	13	53421212	53421212	Missense_Mutation	SNV	A	T	T	PCDH8	c.1360T>A	P.S454T
XH_P16	14	23447665	23447665	Splice_Site	SNV	A	G	G	AJUBA	c.1007-11T>C	
XH_P16	14	25100338	25100338	Missense_Mutation	SNV	C	G	G	GZMB	c.683G>C	P.R228P
XH_P16	15	74468454	74468454	Missense_Mutation	SNV	C	T	T	ISLR	c.1255C>T	P.L419F
XH_P16	16	31382991	31382991	Frame_Shift_Ins	INDEL	-	T	T	ITGAX	c.2046_2047insT	P.P683fs
XH_P16	16	31382992	31382992	Frame_Shift_Ins	INDEL	-	TG	TG	ITGAX	.2047_2048insT	P.P683fs
XH_P16	16	89347823	89347823	Silent	SNV	C	A	A	ANKRD11	c.5127G>T	P.V1709V
XH_P16	17	10297566	10297566	Splice_Site	SNV	C	T	T	MYH8	c.5166G>A	P.Q1722Q
XH_P16	17	18700880	18700880	Splice_Site	SNV	A	T	T	TVP23B	c.49-12A>T	
XH_P16	19	32083744	32083744	Silent	SNV	G	A	A	THEG5	c.246G>A	P.L82L
XH_P16	19	3633460	3633460	Frame_Shift_Del	INDEL	G	-	-	PIP5K1C	c.1979delC	P.P660fs
XH_P16	19	44680888	44680888	Silent	SNV	A	G	G	ZNF226	c.1473A>G	P.Q491Q
XH_P16	19	50213590	50213590	Missense_Mutation	SNV	T	A	A	CPT1C	c.1547T>A	P.I516N
XH_P16	19	6743270	6743270	Splice_Site	SNV	G	T	T	TRIP10	c.408+3G>T	
XH_P16	2	17913067	17913067	Missense_Mutation	SNV	A	G	G	SMC6	c.422T>C	P.L141P
XH_P16	2	231134655	231134655	Silent	SNV	G	A	A	SP140	c.1251G>A	P.A417A
XH_P16	2	242163117	242163117	Missense_Mutation	SNV	T	C	C	ANO7	c.2615T>C	P.V872A
XH_P16	2	28634869	28634869	Frame_Shift_Del	INDEL	A	-	-	FOSL2	c.536delA	P.K179fs
XH_P16	2	97833499	97833499	Missense_Mutation	SNV	G	C	C	ANKRD36	c.1628G>C	P.G543A
XH_P16	20	62195645	62195645	Silent	SNV	C	T	T	HELZ2	c.4530G>A	P.P1510P
XH_P16	20	9510313	9510313	Missense_Mutation	SNV	G	A	A	LAMP5	c.557G>A	P.R186Q
XH_P16	21	47648346	47648346	Splice_Site	SNV	A	G	G	LSS	c.180+2T>C	
XH_P16	22	36177688	36177688	Missense_Mutation	SNV	G	T	T	RBFOX2	c.355C>A	P.P119T
XH_P16	3	168806806	168806806	Missense_Mutation	SNV	G	T	T	MECOM	c.3198C>A	P.H1066Q
XH_P16	3	178917478	178917478	Missense_Mutation	SNV	G	A	A	PIK3CA	c.353G>A	P.G118D
XH_P16	3	52437845	52437848	Frame_Shift_Del	INDEL	ACTG	-	-	BAP1	1313_1316delCA	P.S438fs

XH_P16	4	160274909	160274909	Silent	SNV	C	T	T	RAPGEF2	c.3879C>T	P.S1293S
XH_P16	5	136324278	136324278	Missense_Mutation	SNV	T	C	C	SPOCK1	c.761A>G	P.N254S
XH_P16	5	148407689	148407689	Missense_Mutation	SNV	G	A	A	SH3TC2	c.1606C>T	P.R536W
XH_P16	5	38407201	38407201	Missense_Mutation	SNV	C	T	T	EGFLAM	c.1100C>T	P.S367L
XH_P16	5	98221357	98221357	Splice_Site	INDEL	-	A	A	CHD1	c.2497-5dupT	
XH_P16	6	136590653	136590653	Missense_Mutation	SNV	G	A	A	BCLAF1	c.2135C>T	P.S712F
XH_P16	6	44392313	44392313	Missense_Mutation	SNV	G	T	T	CDC5L	c.1562G>T	P.R521L
XH_P16	6	56418088	56418088	Frame_Shift_Ins	INDEL	-	T	T	DST	c.9144dupA	P.A3049fs
XH_P16	7	111976201	111976201	Silent	SNV	C	A	A	ZNF277	c.844C>A	P.R282R
XH_P16	7	121616296	121616296	Frame_Shift_Del	INDEL	T	-	-	PTPRZ1	c.527delT	P.L176fs
XH_P16	7	146818146	146818146	Missense_Mutation	SNV	A	G	G	CNTNAP2	c.830A>G	P.H277R
XH_P16	7	18688241	18688241	Missense_Mutation	SNV	C	G	G	HDAC9	c.1402C>G	P.Q468E
XH_P16	8	134062215	134062215	Missense_Mutation	SNV	T	G	G	SLA	c.180A>C	P.K60N
XH_P16	8	145060131	145060131	Missense_Mutation	SNV	A	C	C	PARP10	c.230T>G	P.V77G
XH_P16	9	136599206	136599206	Silent	SNV	G	A	A	SARDH	c.90C>T	P.S30S
XH_P16	X	16887325	16887325	Missense_Mutation	SNV	T	G	G	RBBP7	c.167A>C	P.E56A
XH_P17	1	150116893	150116893	Missense_Mutation	SNV	A	G	G	VPS45	c.1411A>G	P.R471G
XH_P17	1	151681773	151681773	Missense_Mutation	SNV	T	C	C	CELF3	c.188A>G	P.D63G
XH_P17	1	17727720	17727720	Missense_Mutation	SNV	G	A	A	PADI6	c.1871G>A	P.G624D
XH_P17	1	197313501	197313501	Missense_Mutation	SNV	G	A	A	CRB1	c.743G>A	P.C248Y
XH_P17	10	104356900	104356900	Nonsense_Mutation	SNV	A	T	T	SUFU	c.760A>T	P.R254*
XH_P17	10	75560464	75560464	Frame_Shift_Del	INDEL	C	-	-	ZSWIM8	c.5085delC	P.Y1696fs
XH_P17	11	34680218	34680218	Nonsense_Mutation	SNV	G	A	A	EHF	c.677G>A	P.W226*
XH_P17	11	64564284	64564284	Silent	SNV	G	T	T	MAP4K2	c.1465C>A	P.R489R
XH_P17	13	111274693	111274693	Silent	SNV	C	T	T	NAXD	c.231C>T	P.G77G
XH_P17	14	105417034	105417034	Missense_Mutation	SNV	T	C	C	AHNAK2	c.4754A>G	P.K1585R
XH_P17	14	91356823	91356823	Splice_Site	SNV	G	A	A	RPS6KA5	c.993+8C>T	
XH_P17	16	15790606	15790606	Missense_Mutation	SNV	A	G	G	NDE1	c.836A>G	P.Y279C
XH_P17	16	31539967	31539967	Silent	SNV	C	T	T	AHSP	c.264C>T	P.F88F
XH_P17	16	53256655	53256655	Silent	SNV	T	C	C	CHD9	c.1884T>C	P.D628D
XH_P17	19	43237120	43237120	Silent	SNV	G	A	A	PSG3	c.525C>T	P.D175D
XH_P17	19	47200450	47200450	Silent	SNV	C	T	T	PRKD2	c.1281G>A	P.T427T
XH_P17	19	8154821	8154821	Silent	SNV	G	A	A	FBN3	c.6207C>T	P.P2069P
XH_P17	2	179981410	179981410	Missense_Mutation	SNV	A	T	T	SESTD1	c.1632T>A	P.F544L
XH_P17	2	234627609	234627609	Missense_Mutation	SNV	T	C	C	UGT1A4	c.143T>C	P.L48S
XH_P17	20	16486474	16486474	Missense_Mutation	SNV	T	C	C	KIF16B	c.893A>G	P.N298S
XH_P17	20	47274740	47274740	Silent	SNV	G	A	A	PREX1	c.1908C>T	P.G636G
XH_P17	21	45844768	45844768	Missense_Mutation	SNV	G	A	A	TRPM2	c.3733G>A	P.V1245M
XH_P17	21	47704502	47704502	Silent	SNV	T	C	C	MCM3AP	c.699A>G	P.E233E
XH_P17	22	21330964	21330964	Missense_Mutation	SNV	C	T	T	AIFM3	c.1055C>T	P.T352M
XH_P17	22	51159965	51159965	Missense_Mutation	SNV	C	T	T	SHANK3	c.3662C>T	P.P1221L
XH_P17	3	52539331	52539331	Splice_Site	SNV	A	T	T	STAB1	c.1519-4A>T	
XH_P17	3	97596432	97596432	Nonsense_Mutation	SNV	G	T	T	CRYBG3	c.6394G>T	P.E2132*
XH_P17	4	88055769	88055769	Missense_Mutation	SNV	A	T	T	AFF1	c.3458A>T	P.Y1153F
XH_P17	5	149512405	149512405	Silent	SNV	C	T	T	PDGFRB	c.1035G>A	P.P345P
XH_P17	6	24839358	24839358	Missense_Mutation	SNV	G	C	C	FAM65B	c.2000C>G	P.A667G
XH_P17	6	44117662	44117662	Missense_Mutation	SNV	A	C	C	TMEM63B	c.1480A>C	P.I494L
XH_P17	7	120704280	120704280	Splice_Site	SNV	C	T	T	CPED1	c.541-12C>T	
XH_P17	7	140481411	140481411	Missense_Mutation	SNV	C	A	A	BRAF	c.1397G>T	P.G466V
XH_P17	8	144789836	144789836	Splice_Site	SNV	C	T	T	CCDC166	c.444G>A	P.K148K
XH_P17	8	25890659	25890659	Missense_Mutation	SNV	C	G	G	EBF2	c.493G>C	P.E165Q
XH_P17	8	57128950	57128950	Splice_Site	SNV	C	T	T	CHCHD7	c.132C>T	P.I44I
XH_P17	9	114986141	114986141	Missense_Mutation	SNV	G	C	C	PTBP3	c.1565C>G	P.S522C
XH_P17	9	134362687	134362687	Splice_Site	SNV	C	T	T	PRRC2B	c.5984+6C>T	
XH_P17	9	38068518	38068518	Frame_Shift_Ins	INDEL	-	G	G	SHB	c.124dupC	P.Q42fs
XH_P17	X	107924981	107924981	Splice_Site	SNV	G	T	T	COL4A5	c.4070-9G>T	
XH_P17	X	32407785	32407785	Frame_Shift_Ins	INDEL	-	T	T	DMD	c.4326dupA	P.L1443fs
XH_P18	1	115251238	115251238	Frame_Shift_Del	INDEL	A	-	-	NRAS	c.488delT	P.I163fs
XH_P18	1	158450049	158450049	Missense_Mutation	SNV	G	A	A	OR10R2	c.382G>A	P.A128T
XH_P18	10	118443293	118443293	Splice_Site	SNV	C	G	G	HSPA12A	c.886+9G>C	
XH_P18	10	50028381	50028381	Missense_Mutation	SNV	G	A	A	WDFY4	c.5608G>A	P.A1870T
XH_P18	11	56058264	56058264	Missense_Mutation	SNV	G	A	A	OR8H1	c.275C>T	P.S92F
XH_P18	11	612757	612757	Missense_Mutation	SNV	C	T	T	IRF7	c.1400G>A	P.R467H
XH_P18	11	64110663	64110663	Missense_Mutation	SNV	C	T	T	CCDC88B	c.1075C>T	P.L359F
XH_P18	11	6554289	6554289	Missense_Mutation	SNV	C	G	G	DNHD1	c.2380C>G	P.L794V
XH_P18	11	67811294	67811294	Missense_Mutation	SNV	G	A	A	TCIRG1	c.727G>A	P.V243I
XH_P18	11	68475782	68475782	Missense_Mutation	SNV	C	A	A	TESMIN	c.1521G>T	P.M507I
XH_P18	11	77590088	77590088	Missense_Mutation	SNV	C	G	G	INTS4	c.2799G>C	P.E933D
XH_P18	12	123200497	123200497	Missense_Mutation	SNV	G	A	A	HCAR3	c.788C>T	P.T263M
XH_P18	12	25398285	25398285	Missense_Mutation	SNV	C	A	A	KRAS	c.34G>T	P.G12C
XH_P18	12	40940944	40940944	Silent	SNV	C	T	T	MUC19	c.23139C>T	P.T7713T
XH_P18	12	52865464	52865464	Missense_Mutation	SNV	G	C	C	KRT6C	c.808C>G	P.L270V
XH_P18	13	113803460	113803460	Missense_Mutation	SNV	C	A	A	F10	c.1096C>A	P.R366S
XH_P18	13	36686200	36686200	Nonsense_Mutation	SNV	G	A	A	DCLK1	c.529C>T	P.R177*
XH_P18	14	20847178	20847178	Silent	SNV	G	A	A	TEP1	c.4890C>T	P.D1630D
XH_P18	14	35592700	35592700	Frame_Shift_Del	INDEL	T	-	-	KIAA0391	c.257delT	P.L86fs
XH_P18	14	77685214	77685214	Missense_Mutation	SNV	G	C	C	TMEM63C	c.58G>C	P.D20H
XH_P18	15	43023935	43023935	Missense_Mutation	SNV	C	T	T	CDAN1	c.1622G>A	P.R541Q
XH_P18	15	48786440	48786440	Missense_Mutation	SNV	C	T	T	FBN1	c.2689G>A	P.G897S
XH_P18	15	65491108	65491108	Missense_Mutation	SNV	G	A	A	CILP	c.1516C>T	P.R506C
XH_P18	15	79603645	79603645	Silent	SNV	G	A	A	TMED3	c.54G>A	P.L18L
XH_P18	16	1709952	1709952	Silent	SNV	G	A	A	CRAMP1	c.2301G>A	P.T767T
XH_P18	16	22926676	22926676	Silent	SNV	G	A	A	HS3ST2	c.897G>A	P.T299T
XH_P18	16	682597	682597	Nonsense_Mutation	SNV	G	T	T	WFIKKN1	c.187G>T	P.E63*
XH_P18	16	84179152	84179152	Missense_Mutation	SNV	G	C	C	DNAAF1	c.107G>C	P.R36P
XH_P18	17	40028336	40028336	Silent	SNV	C	T	T	ACLY	c.2742G>A	P.A914A
XH_P18	17	46930276	46930276	Splice_Site	SNV	C	G	G	CALCOCO2	c.898-12C>G	
XH_P18	17	55822346	55822346	Silent	SNV	G	A	A	CCDC182	c.288C>T	P.R96R
XH_P18	18	12699131	12699131	Nonsense_Mutation	SNV	G	A	A	CEP76	c.367C>T	P.Q123*
XH_P18	18	32825531	32825531	Missense_Mutation	SNV	G	A	A	ZNF397	c.862G>A	P.D288N
XH_P18	18	42530447	42530447	Missense_Mutation	SNV	C	T	T	SETBP1	c.1142C>T	P.S381L
XH_P18	18	48591933	48591933	Nonsense_Mutation	SNV	C	T	T	SMAD4	c.1096C>T	P.Q366*
XH_P18	18	7080391	7080391	Missense_Mutation	SNV	C	T	T	LAMA1	c.127G>A	P.E43K
XH_P18	19	17648265	17648265	Missense_Mutation	SNV	C	T	T	FAM129C	c.601C>T	P.R201W
XH_P18	2	114257213	114257213	Missense_Mutation	SNV	G	A	A	FOXD4L1	c.380G>A	P.R127H
XH_P18	2	114355053	114355053	Splice_Site	SNV	C	T	T	WASH2P	n.1307-10C>T	
XH_P18	2	120223398	120223398	Missense_Mutation	SNV	A	T	T	SCTR	c.470T>A	P.L157H
XH_P18	2	165947654	165947654	Missense_Mutation	SNV	G	C	C	SCN3A	c.4862C>G	P.A1621G

XH_P18	20	30436343	30436343	Missense_Mutation	SNV	C	T	T	DUSP15	c.752G>A	P.S251N
XH_P18	20	45021755	45021755	Missense_Mutation	SNV	T	C	C	ELMO2	c.220A>G	P.I74V
XH_P18	20	55033716	55033716	Frame_Shift_Del	INDEL	C	-	-	CASS4	c.2115delC	P.S706fs
XH_P18	20	57766219	57766219	Frame_Shift_Ins	INDEL	-	C	C	ZNF831	c.153dupC	P.T52fs
XH_P18	22	30064391	30064391	Nonsense_Mutation	SNV	C	T	T	NF2	c.955C>T	P.Q319*
XH_P18	3	130095279	130095279	Silent	SNV	C	A	A	COL6A5	c.267C>A	P.G89G
XH_P18	3	134348527	134348527	Silent	SNV	C	T	T	KY	c.273G>A	P.L91L
XH_P18	3	192516769	192516769	Silent	SNV	G	A	A	MB21D2	c.882C>T	P.S294S
XH_P18	4	78079683	78079683	Splice_Site	SNV	C	T	T	CCNG2	c.1-3C>T	
XH_P18	5	128932256	128932256	Missense_Mutation	SNV	A	G	G	ADAMTS19	c.1377A>G	P.I459M
XH_P18	5	150051388	150051388	Silent	SNV	G	A	A	MYOZ3	c.342G>A	P.G114G
XH_P18	6	167754781	167754781	Missense_Mutation	SNV	T	C	C	TTLL2	c.1393T>C	P.Y465H
XH_P18	6	26598218	26598218	Silent	SNV	C	A	A	ABT1	c.318C>A	P.T106T
XH_P18	7	154876053	154876053	Silent	SNV	C	G	G	HTR5A	c.930C>G	P.L310L
XH_P18	7	31144526	31144526	Splice_Site	SNV	A	G	G	ADCYAP1R1	c.1218+3A>G	
XH_P18	7	4107515	4107515	Missense_Mutation	SNV	C	T	T	SDK1	c.2957C>T	P.T986I
XH_P18	9	113217858	113217858	Splice_Site	SNV	C	T	T	SVEP1	c.3787+12G>A	
XH_P18	9	117749	117749	Missense_Mutation	SNV	C	T	T	FOXD4	c.371G>A	P.R124H
XH_P18	9	117840316	117840316	Silent	SNV	G	A	A	TNC	c.2580C>T	P.I860I
XH_P18	9	123935683	123935683	Missense_Mutation	SNV	G	A	A	CNTRL	c.6580G>A	P.E2194K
XH_P18	9	127549470	127549470	Missense_Mutation	SNV	G	A	A	OLFML2A	c.307G>A	P.E103K
XH_P18	9	32573010	32573010	Missense_Mutation	SNV	C	T	T	NDUFB6	c.49G>A	P.E17K
XH_P18	X	120009158	120009158	Missense_Mutation	SNV	C	G	G	CT47B1	c.367G>C	P.V123L
XH_P18	X	30327273	30327273	Missense_Mutation	SNV	C	T	T	NR0B1	c.208G>A	P.G70S
XH_P18	X	41043854	41043854	Missense_Mutation	SNV	A	T	T	USP9X	c.3484A>T	P.I1162F
XH_P18	X	47028898	47028898	Splice_Site	SNV	G	A	A	RBM10	c.201+1G>A	
XH_P18	X	64738312	64738312	Silent	SNV	G	A	A	LAS1L	c.1431C>T	P.D477D
XH_P18	X	92927194	92927194	Missense_Mutation	SNV	A	C	C	NAP1L3	c.1110T>G	P.N370K
XH_P20	1	104166510	104166510	Missense_Mutation	SNV	C	T	T	AMY2A	c.1124C>T	P.P375L
XH_P20	1	248004654	248004654	Missense_Mutation	SNV	G	A	A	OR11L1	c.545C>T	P.P182L
XH_P20	1	42660569	42660569	Missense_Mutation	SNV	C	G	G	FOXJ3	c.926G>C	P.S309T
XH_P20	1	44128657	44128657	Silent	SNV	A	C	C	KDM4A	c.522A>C	P.P174P
XH_P20	1	86047648	86047648	Silent	SNV	A	G	G	CYR61	c.315A>G	P.R105R
XH_P20	1	87043754	87043754	Splice_Site	SNV	C	T	T	CLCA4	c.2121C>T	P.N707N
XH_P20	10	113913376	113913376	Missense_Mutation	SNV	T	G	G	GPAM	c.2419A>C	P.S807R
XH_P20	11	117309618	117309618	Missense_Mutation	SNV	C	A	A	DSCAML1	c.4406G>T	P.G1469V
XH_P20	11	62287822	62287822	Silent	SNV	G	A	A	AHNAK	c.14067C>T	P.D4689D
XH_P20	11	64029057	64029057	Missense_Mutation	SNV	G	T	T	PLCB3	c.1833G>T	P.R611S
XH_P20	12	46123836	46123836	Frame_Shift_Ins	INDEL	-	A	A	ARID2	c.109dupA	P.I37fs
XH_P20	12	7277231	7277231	Silent	SNV	C	A	A	RBP5	c.201G>T	P.L67L
XH_P20	16	20749214	20749214	Frame_Shift_Del	INDEL	T	-	-	THUMPD1	c.471delA	P.V158fs
XH_P20	16	3169341	3169341	Missense_Mutation	SNV	G	T	T	ZNF205	c.680G>T	P.R227I
XH_P20	16	3169342	3169342	Missense_Mutation	SNV	A	T	T	ZNF205	c.681A>T	P.R227S
XH_P20	17	19700909	19700909	Nonsense_Mutation	SNV	G	A	A	ULK2	c.1609C>T	P.Q537*
XH_P20	17	32906211	32906211	Missense_Mutation	SNV	T	C	C	C17orf102	c.89A>G	P.Q30R
XH_P20	17	3558527	3558527	Frame_Shift_Ins	INDEL	-	T	T	CTNS	c.345dupT	P.L116fs
XH_P20	17	46675333	46675333	Silent	SNV	C	T	T	HOXB6	c.180G>A	P.P60P
XH_P20	17	47295128	47295128	Missense_Mutation	SNV	G	T	T	ABI3	c.295G>T	P.A99S
XH_P20	17	61833623	61833623	Missense_Mutation	SNV	T	A	A	CCDC47	c.925A>T	P.T309S
XH_P20	17	62499430	62499430	Missense_Mutation	SNV	A	C	C	DDX5	c.686T>G	P.I229S
XH_P20	17	67151195	67151195	Missense_Mutation	SNV	A	G	G	ABCA10	c.3659T>C	P.I1220T
XH_P20	19	17448421	17448421	Missense_Mutation	SNV	A	T	T	GTPBP3	c.1A>T	P.M1?
XH_P20	19	287603	287603	Frame_Shift_Ins	INDEL	-	A	A	PLPP2	c.352dupT	P.Y118fs
XH_P20	19	33716448	33716448	Splice_Site	SNV	G	A	A	SLC7A10	c.151+11C>T	
XH_P20	19	40322434	40322434	Splice_Site	SNV	T	G	G	DYRK1B	c.63+11A>C	
XH_P20	19	4525772	4525772	Missense_Mutation	SNV	A	G	G	PLIN5	c.593T>C	P.F198S
XH_P20	19	46915241	46915241	Missense_Mutation	SNV	C	T	T	CCDC8	c.827G>A	P.R276H
XH_P20	19	55812869	55812869	Splice_Site	SNV	C	A	A	BRSK1	c.679-11C>A	
XH_P20	2	170025100	170025100	Missense_Mutation	SNV	C	T	T	LRP2	c.11584G>A	P.D3862N
XH_P20	2	209113113	209113113	Missense_Mutation	SNV	G	A	A	IDH1	c.394C>T	P.R132C
XH_P20	2	219688611	219688611	Splice_Site	SNV	C	T	T	PRKAG3	c.1354-10G>A	
XH_P20	2	241696735	241696735	Splice_Site	SNV	C	T	T	KIF1A	c.2858+1G>A	
XH_P20	2	242590732	242590732	Frame_Shift_Del	INDEL	A	-	-	ATG4B	c.170delA	P.N57fs
XH_P20	2	46231746	46231746	Silent	SNV	C	T	T	PRKCE	c.1032C>T	P.S344S
XH_P20	20	25596768	25596768	Silent	SNV	T	C	C	NANP	c.540A>G	P.Q180Q
XH_P20	20	37464638	37464638	Missense_Mutation	SNV	C	T	T	PPP1R16B	c.70C>T	P.R24W
XH_P20	20	56139592	56139592	Missense_Mutation	SNV	C	T	T	PCK1	c.1241C>T	P.P414L
XH_P20	22	36661955	36661955	Missense_Mutation	SNV	A	C	C	APOL1	c.1073A>C	P.H358P
XH_P20	22	39132380	39132380	Silent	SNV	A	C	C	SUN2	c.2109T>G	P.P703P
XH_P20	3	30713759	30713759	Missense_Mutation	SNV	C	T	T	TGFBR2	c.1159C>T	P.H387Y
XH_P20	3	46759092	46759092	Missense_Mutation	SNV	A	G	G	PRSS50	c.142T>C	P.S48P
XH_P20	4	99997531	99997531	Missense_Mutation	SNV	A	C	C	ADH5	c.737T>G	P.F246C
XH_P20	5	130775337	130775337	Missense_Mutation	SNV	T	C	C	RAPGEF6	c.3706A>G	P.I1236V
XH_P20	5	140798530	140798530	Silent	SNV	C	T	T	PCDHGB7	c.1104C>T	P.L368L
XH_P20	5	179302035	179302035	Missense_Mutation	SNV	C	T	T	TBC1D9B	c.2053G>A	P.V685M
XH_P20	5	90671344	90671344	Missense_Mutation	SNV	C	A	A	ARRDC3	c.597G>T	P.R199S
XH_P20	6	144783784	144783784	Splice_Site	SNV	T	G	G	UTRN	c.2854-6T>G	
XH_P20	6	52380816	52380816	Silent	SNV	G	A	A	TRAM2	c.399C>T	P.Y133Y
XH_P20	7	126409909	126409909	Splice_Site	SNV	T	C	C	GRM8	c.1357+10A>G	
XH_P20	7	157959907	157959907	Missense_Mutation	SNV	C	T	T	PTPRN2	c.512G>A	P.R171Q
XH_P20	7	158566022	158566022	Splice_Site	SNV	C	T	T	ESYT2	c.947+10G>A	
XH_P20	7	29126126	29126126	Missense_Mutation	SNV	T	C	C	CPVL	c.583A>G	P.N195D
XH_P20	9	107556705	107556727	Frame_Shift_Del	INDEL	TCGATGAGC	-	-	ABCA1	GACGAGGGCTC	P.G1816fs
XH_P20	9	35698173	35698173	Splice_Site	SNV	G	A	A	TLN1	c.7372-4C>T	
XH_P20	X	110494522	110494522	Splice_Site	SNV	T	C	C	CAPN6	c.894-8A>G	
XH_P21	1	16475402	16475418	Frame_Shift_Del	INDEL	TCAATGAAG	-	-	EPHA2	delGTATCTTCA	P.R93fs
XH_P21	1	27089779	27089779	Splice_Site	SNV	A	T	T	ARID1A	c.2732+3A>T	
XH_P21	1	48698073	48698073	Missense_Mutation	SNV	T	C	C	SLC5A9	c.932T>C	P.L311P
XH_P21	11	113639609	113639609	Silent	SNV	C	T	T	ZW10	c.186G>A	P.Q62Q
XH_P21	11	117074070	117074070	Silent	SNV	G	A	A	TAGLN	c.228G>A	P.P76P
XH_P21	11	4095774	4095774	Silent	SNV	G	A	A	STIM1	c.834G>A	P.E278E
XH_P21	11	60705508	60705508	Splice_Site	SNV	G	T	T	SLC15A3	c.1436-11C>A	
XH_P21	12	32908789	32908789	Missense_Mutation	SNV	C	T	T	YARS2	c.20G>A	P.R7Q
XH_P21	12	56094189	56094189	Splice_Site	SNV	G	A	A	ITGA7	c.671-12C>T	
XH_P21	14	21831293	21831293	Missense_Mutation	SNV	C	T	T	SUPT16H	c.1411G>A	P.E471K
XH_P21	14	53347983	53347983	Missense_Mutation	SNV	T	G	G	FERMT2	c.731A>C	P.D244A
XH_P21	14	93290937	93290937	Missense_Mutation	SNV	T	C	C	GOLGA5	c.1667T>C	P.L556S
XH_P21	15	40094264	40094264	Missense_Mutation	SNV	A	C	C	GPR176	c.614T>G	P.V205G

XH_P21	16	11785284	11785284	Missense_Mutation	SNV	T	C	C	TXNDC11	c.1843A>G	P.N615D
XH_P21	17	56060555	56060555	Missense_Mutation	SNV	T	A	A	VEZF1	c.233A>T	P.Y78F
XH_P21	18	76752084	76752084	Silent	SNV	G	C	C	SALL3	c.93G>C	P.G31G
XH_P21	19	3110297	3110297	Missense_Mutation	SNV	C	T	T	GNA11	c.287C>T	P.T96M
XH_P21	19	44792033	44792033	Missense_Mutation	SNV	C	T	T	ZNF235	c.1555G>A	P.V519M
XH_P21	19	8551102	8551102	Missense_Mutation	SNV	G	T	T	HNRNPM	c.1385G>T	P.G462V
XH_P21	2	170012809	170012809	Silent	SNV	G	A	A	LRP2	c.12126C>T	P.R4042R
XH_P21	2	171863153	171863153	Splice_Site	SNV	C	T	T	TLK1	c.1455G>A	P.T485T
XH_P21	2	179472377	179472377	Missense_Mutation	SNV	C	G	G	TTN	c.48115G>C	P.G16039R
XH_P21	20	60884451	60884451	Missense_Mutation	SNV	T	C	C	LAMA5	c.11029A>G	P.M3677V
XH_P21	22	17445688	17445688	Missense_Mutation	SNV	A	T	T	GAB4	c.1444T>A	P.S482T
XH_P21	3	52649430	52649430	Nonsense_Mutation	SNV	T	A	A	PBRM1	c.1861A>T	P.K621*
XH_P21	5	122718697	122718697	Missense_Mutation	SNV	T	C	C	CEP120	c.1765A>G	P.I589V
XH_P21	5	149918784	149918784	Splice_Site	SNV	C	T	T	NDST1	c.1438-6C>T	
XH_P21	5	176522651	176522651	Missense_Mutation	SNV	C	T	T	FGFR4	c.1748C>T	P.P583L
XH_P21	5	77334917	77334917	Missense_Mutation	SNV	C	T	T	AP3B1	c.2612G>A	P.G871E
XH_P21	5	7897231	7897231	Missense_Mutation	SNV	C	A	A	MTRR	c.1823C>A	P.S608Y
XH_P21	6	123369766	123369766	Splice_Site	SNV	G	A	A	CLVS2	c.565-1G>A	
XH_P21	6	38690689	38690689	Missense_Mutation	SNV	G	A	A	DNAH8	c.104G>A	P.R35H
XH_P21	6	43167899	43167899	Splice_Site	SNV	G	A	A	CUL9	c.3384+5G>A	
XH_P21	7	105305727	105305727	Missense_Mutation	SNV	G	C	C	ATXN7L1	c.364C>G	P.H122D
XH_P21	7	149129166	149129166	Missense_Mutation	SNV	C	G	G	ZNF777	c.2197G>C	P.E733Q
XH_P21	7	35242154	35242154	Missense_Mutation	SNV	G	T	T	TBX20	c.1232C>A	P.P411H
XH_P21	7	70598012	70598012	Missense_Mutation	SNV	A	T	T	WBSCR17	c.224A>T	P.Y75F
XH_P21	X	154493522	154493522	Missense_Mutation	SNV	T	G	G	RAB39B	c.52A>C	P.T18P
XH_P24	1	248814142	248814142	Frame_Shift_Ins	INDEL	-	TGTACTTCCT	TGTACTTCCT	OR2T27	_44insAGGAAGT	P.L15fs
XH_P24	18	48575131	48575131	Frame_Shift_Ins	INDEL	-	GACCATTTATTCA	GACCATTTATTCA	SMAD4	326insGACCATT	P.L109fs
XH_P24	22	30077521	30077521	Frame_Shift_Ins	INDEL	-	CG	CG	NF2	.1668_1669insC	P.A557fs
XH_P24	6	28097573	28097573	Missense_Mutation	SNV	G	C	C	ZSCAN16	c.892G>C	P.G298R
XH_P24	7	138732549	138732549	Frame_Shift_Ins	INDEL	-	TTTCAGAAAG	TTTCAGAAAG	ZC3HAV1	_2500insCTTTCT	P.D834fs
XH_P25	1	170521361	170521361	Nonsense_Mutation	SNV	G	T	T	GORAB	c.403G>T	P.E135*
XH_P25	1	173930226	173930226	Missense_Mutation	SNV	G	A	A	RC3H1	c.2359C>T	P.H787Y
XH_P25	1	196577385	196577385	Missense_Mutation	SNV	C	T	T	KCNT2	c.55G>A	P.D19N
XH_P25	1	223989973	223989973	Missense_Mutation	SNV	A	C	C	TP53BP2	c.1070T>G	P.I357S
XH_P25	1	240370732	240370732	Missense_Mutation	SNV	G	T	T	FMN2	c.2632G>T	P.G878C
XH_P25	11	124543592	124543592	Missense_Mutation	SNV	C	A	A	SIAE	c.13G>T	P.G5W
XH_P25	11	124543593	124543593	Silent	SNV	C	A	A	SIAE	c.12G>T	P.P4P
XH_P25	11	1578670	1578670	Missense_Mutation	SNV	G	A	A	DUSP8	c.956C>T	P.P319L
XH_P25	11	373469	373469	Silent	SNV	G	T	T	B4GALNT4	c.657G>T	P.A219A
XH_P25	11	78467903	78467903	Silent	SNV	G	A	A	TENM4	c.2703C>T	P.L901L
XH_P25	12	113549986	113549986	Missense_Mutation	SNV	C	A	A	RASAL1	c.1279G>T	P.V427L
XH_P25	12	130833889	130833889	Silent	SNV	G	A	A	PIWIL1	c.840G>A	P.L280L
XH_P25	12	41900447	41900447	Missense_Mutation	SNV	G	A	A	PDZRN4	c.1033G>A	P.A345T
XH_P25	12	50358787	50358787	Missense_Mutation	SNV	G	A	A	AQP5	c.625G>A	P.G209R
XH_P25	12	7355295	7355295	Missense_Mutation	SNV	A	G	G	PEX5	c.841A>G	P.I281V
XH_P25	13	114059226	114059226	Silent	SNV	C	T	T	LOC101928841	c.3279G>A	P.T1093T
XH_P25	13	53313269	53313269	Missense_Mutation	SNV	G	T	T	LECT1	c.110C>A	P.A37E
XH_P25	13	73636749	73636749	Missense_Mutation	SNV	A	G	G	KLF5	c.1012A>G	P.I338V
XH_P25	13	84453670	84453670	Missense_Mutation	SNV	G	A	A	SLITRK1	c.1973C>T	P.A658V
XH_P25	15	25223443	25223443	Frame_Shift_Ins	INDEL	-	C	C	SNRPN	c.668dupC	P.P224fs
XH_P25	15	62948259	62948259	Missense_Mutation	SNV	G	T	T	TLN2	c.634G>T	P.V212L
XH_P25	15	68492004	68492005	Frame_Shift_Del	INDEL	AA	-	-	CALML4	c.178_179delTT	P.F60fs
XH_P25	16	1306884	1306884	Missense_Mutation	SNV	T	A	A	TPSD1	c.341T>A	P.I114N
XH_P25	16	22343426	22343426	Missense_Mutation	SNV	C	T	T	POLR3E	c.1882C>T	P.R628C
XH_P25	17	12852965	12852965	Splice_Site	SNV	G	A	A	ARHGAP44	c.974-1G>A	
XH_P25	17	15539578	15539578	Silent	SNV	C	A	A	TRIM16	c.621G>T	P.S207S
XH_P25	17	30980907	30980907	Missense_Mutation	SNV	C	T	T	MYO1D	c.2549G>A	P.R850Q
XH_P25	17	43923696	43923696	Missense_Mutation	SNV	C	A	A	SPPL2C	c.1424C>A	P.A475D
XH_P25	17	43923697	43923697	Silent	SNV	C	A	A	SPPL2C	c.1425C>A	P.A475A
XH_P25	17	47218678	47218678	Silent	SNV	C	T	T	B4GALNT2	c.84C>T	P.F28F
XH_P25	17	7577070	7577074	Frame_Shift_Del	INDEL	GGAGA	-	-	TP53	864_868delTCTC	P.N288fs
XH_P25	17	76100671	76100672	Frame_Shift_Del	INDEL	GT	-	-	TNRC6C	.4926_4927delG	P.Q1642fs
XH_P25	17	7906637	7906637	Missense_Mutation	SNV	C	A	A	GUCY2D	c.272C>A	P.A91E
XH_P25	18	13885084	13885084	Missense_Mutation	SNV	C	T	T	MC2R	c.434G>A	P.R145H
XH_P25	18	8783914	8783914	Silent	SNV	G	T	T	MTCL1	c.804G>T	P.L268L
XH_P25	18	8783915	8783915	Nonsense_Mutation	SNV	G	T	T	MTCL1	c.805G>T	P.E269*
XH_P25	19	11559980	11559980	Missense_Mutation	SNV	G	T	T	PRKCSH	c.1421G>T	P.R474L
XH_P25	19	21240796	21240796	Missense_Mutation	SNV	A	G	G	ZNF430	c.1679A>G	P.N560S
XH_P25	19	39018362	39018362	Missense_Mutation	SNV	G	T	T	RYR1	c.10762G>T	P.D3588Y
XH_P25	19	4523635	4523635	Missense_Mutation	SNV	C	A	A	PLIN5	c.1297G>T	P.D433Y
XH_P25	19	4523636	4523636	Silent	SNV	C	A	A	PLIN5	c.1296G>T	P.G432G
XH_P25	19	5957919	5957919	Splice_Site	SNV	G	A	A	RANBP3	c.78+10C>T	
XH_P25	19	7998877	7998877	Silent	SNV	G	A	A	TIMM44	c.555C>T	P.S185S
XH_P25	2	110881564	110881564	Missense_Mutation	SNV	C	A	A	NPHP1	c.2006G>T	P.R669L
XH_P25	2	113817047	113817047	Splice_Site	SNV	G	A	A	IL36RN	c.29+3G>A	
XH_P25	2	160245943	160245943	Missense_Mutation	SNV	C	G	G	BAZ2B	c.3021G>C	P.E1007D
XH_P25	2	228884239	228884239	Missense_Mutation	SNV	C	A	A	SPHKAP	c.1331G>T	P.W444L
XH_P25	2	233986944	233986944	Missense_Mutation	SNV	G	A	A	INPP5D	c.326G>A	P.G109D
XH_P25	2	234463031	234463031	Missense_Mutation	SNV	C	G	G	USP40	c.724G>C	P.A242P
XH_P25	2	25976397	25976397	Splice_Site	SNV	T	C	C	ASXL2	c.1142+6A>G	
XH_P25	2	98354504	98354504	Missense_Mutation	SNV	G	A	A	ZAP70	c.1670G>A	P.R557Q
XH_P25	22	28378504	28378504	Missense_Mutation	SNV	G	A	A	TTC28	c.7151C>T	P.T2384M
XH_P25	22	40140184	40140184	Missense_Mutation	SNV	C	A	A	ENTHD1	c.1324G>T	P.A442S
XH_P25	3	11402185	11402185	Nonsense_Mutation	SNV	C	G	G	ATG7	c.1610C>G	P.S537*
XH_P25	3	129247918	129247918	Silent	SNV	C	T	T	RHO	c.342C>T	P.G114G
XH_P25	3	141305476	141305476	Splice_Site	SNV	T	C	C	RASA2	c.1827-12T>C	
XH_P25	3	155654143	155654143	Silent	SNV	C	T	T	GMPS	c.1824C>T	P.I608I
XH_P25	3	164907582	164907582	Missense_Mutation	SNV	G	A	A	SLITRK3	c.1037C>T	P.S346F
XH_P25	3	190158126	190158126	Missense_Mutation	SNV	G	T	T	TMEM207	c.211C>A	P.L71I
XH_P25	3	190158127	190158127	Silent	SNV	G	T	T	TMEM207	c.210C>A	P.V70V
XH_P25	3	193355029	193355029	Missense_Mutation	SNV	G	T	T	OPA1	c.829G>T	P.D277Y
XH_P25	3	52620655	52620655	Missense_Mutation	SNV	A	T	T	PBRM1	c.3098T>A	P.V1033D
XH_P25	3	58486842	58486842	Missense_Mutation	SNV	G	T	T	KCTD6	c.197G>T	P.R66L
XH_P25	4	121616342	121616342	Missense_Mutation	SNV	A	G	G	PRDM5	c.1724T>C	P.F575S
XH_P25	5	17275669	17275669	Missense_Mutation	SNV	C	T	T	BASP1	c.344C>T	P.A115V
XH_P25	5	70945025	70945025	Frame_Shift_Del	INDEL	A	-	-	MCCC2	c.1318delA	P.I440fs
XH_P25	5	7867230	7867230	Missense_Mutation	SNV	C	A	A	FASTKD3	c.967G>T	P.V323L
XH_P25	5	89938743	89938743	Missense_Mutation	SNV	G	C	C	ADGRV1	c.2438G>C	P.R813P

XH_P25	6	106555213	106555213	Missense_Mutation	SNV	A	T	T	PRDM1	c.2330A>T	P.Q777L
XH_P25	6	152652741	152652741	Frame_Shift_Ins	INDEL	-	C	C	SYNE1	c.12865dupG	P.A4289fs
XH_P25	7	100161551	100161551	Frame_Shift_Del	INDEL	C	-	-	AGFG2	c.1271delC	P.P424fs
XH_P25	7	43918743	43918743	Missense_Mutation	SNV	C	G	G	URGCP	c.319G>C	P.A107P
XH_P25	7	44806172	44806172	Silent	SNV	T	G	G	ZMIZ2	c.2391T>G	P.P797P
XH_P25	7	81346681	81346681	Splice_Site	SNV	A	G	G	HGF	c.1272T>C	P.R424R
XH_P25	8	133953677	133953677	Missense_Mutation	SNV	T	C	C	TG	c.5123T>C	P.I1708T
XH_P25	9	117536	117536	Missense_Mutation	SNV	C	T	T	FOXD4	c.584G>A	P.R195Q
XH_P25	9	32631851	32631851	Silent	SNV	G	T	T	TAF1L	c.3727C>A	P.R1243R
XH_P25	9	36169791	36169791	Missense_Mutation	SNV	G	A	A	CCIN	c.292G>A	P.E98K
XH_P25	X	125299685	125299685	Missense_Mutation	SNV	G	T	T	DCAF12L2	c.223C>A	P.L75M
XH_P27	1	179414125	179414125	Silent	SNV	A	G	G	AXDND1	c.1584A>G	P.E528E
XH_P27	1	18808631	18808631	Missense_Mutation	SNV	G	A	A	KLHDC7A	c.1156G>A	P.A386T
XH_P27	1	34329964	34329964	Missense_Mutation	SNV	G	A	A	HMGB4	c.172G>A	P.A58T
XH_P27	1	35370539	35370539	Missense_Mutation	SNV	C	A	A	DLGAP3	c.446G>T	P.G149V
XH_P27	1	8422759	8422772	Frame_Shift_Del	INDEL	TGTCTCTGCT	-	-	RERE	386delAAAGCAG	P.K625fs
XH_P27	10	124742302	124742302	Silent	SNV	C	T	T	PSTK	c.270C>T	P.F90F
XH_P27	11	103055658	103055658	Missense_Mutation	SNV	A	T	T	DYNC2H1	c.6511A>T	P.T2171S
XH_P27	11	124749086	124749086	Missense_Mutation	SNV	G	C	C	ROBO3	c.3534G>C	P.R1178S
XH_P27	12	121442106	121442106	Silent	SNV	G	C	C	C12orf43	c.732C>G	P.T244T
XH_P27	12	12277603	12277603	Splice_Site	INDEL	-	A	A	LRP6	c.4450-8dupT	
XH_P27	12	40884463	40884463	Silent	SNV	C	G	G	MUC19	c.17709C>G	P.T5903T
XH_P27	13	108882125	108882125	Missense_Mutation	SNV	A	T	T	ABHD13	c.559A>T	P.I187F
XH_P27	15	42285075	42285075	Missense_Mutation	SNV	C	T	T	PLA2G4E	c.1330G>A	P.V444I
XH_P27	16	28116004	28116004	Nonsense_Mutation	SNV	C	A	A	XPO6	c.2767G>T	P.E923*
XH_P27	17	10219334	10219334	Missense_Mutation	SNV	T	C	C	MYH13	c.3747A>G	P.I1249M
XH_P27	17	33884249	33884249	Missense_Mutation	SNV	A	G	G	SLFN14	c.833T>C	P.L278S
XH_P27	17	38179531	38179531	Silent	SNV	C	T	T	MED24	c.2064G>A	P.L688L
XH_P27	17	77073866	77073866	Silent	SNV	G	A	A	ENGASE	c.336G>A	P.A112A
XH_P27	18	29110998	29110998	Missense_Mutation	SNV	G	A	A	DSG2	c.1063G>A	P.A355T
XH_P27	19	40316855	40316855	Missense_Mutation	SNV	G	A	A	DYRK1B	c.1483C>T	P.P495S
XH_P27	19	57133313	57133313	Frame_Shift_Del	INDEL	G	-	-	ZNF71	c.659delG	P.G220fs
XH_P27	2	108488685	108488685	Missense_Mutation	SNV	C	A	A	RGPD4	c.4225C>A	P.H1409N
XH_P27	2	165997191	165997191	Silent	SNV	T	C	C	SCN3A	c.1989A>G	P.L663L
XH_P27	2	225706624	225706624	Splice_Site	INDEL	A	-	-	DOCK10	c.2551-11delT	
XH_P27	20	43926873	43926873	Missense_Mutation	SNV	C	G	G	MATN4	c.1363G>C	P.V455L
XH_P27	21	41668064	41668064	Silent	SNV	G	A	A	DSCAM	c.2100C>T	P.D700D
XH_P27	3	20212651	20212651	Silent	SNV	A	T	T	SGO1	c.1356T>A	P.V452V
XH_P27	3	52437289	52437289	Frame_Shift_Ins	INDEL	-	A	A	BAP1	c.1754_1755ins	P.I586fs
XH_P27	4	57871762	57871762	Splice_Site	SNV	A	T	T	POLR2B	c.1098-4A>T	
XH_P27	7	133943088	133943088	Missense_Mutation	SNV	C	T	T	LRGUK	c.2278C>T	P.P760S
XH_P27	7	81381436	81381436	Missense_Mutation	SNV	C	G	G	HGF	c.625G>C	P.V209L
XH_P27	8	22995468	22995468	Missense_Mutation	SNV	T	C	C	TNFRSF10D	c.1076A>G	P.K359R
XH_P27	9	37441257	37441257	Missense_Mutation	SNV	A	G	G	ZBTB5	c.1292T>C	P.I431T
XH_P27	9	40704387	40704387	Silent	SNV	C	T	T	SPATA31A3	c.2044C>T	P.L682L
XH_P28	1	1296629	1296629	Silent	SNV	C	T	T	MXRA8	c.15G>A	P.A5A
XH_P28	1	145487324	145487324	Missense_Mutation	SNV	C	A	A	LIX1L	c.313C>A	P.Q105K
XH_P28	1	154562702	154562702	Silent	SNV	A	G	G	ADAR	c.1569T>C	P.T523T
XH_P28	1	16460396	16460396	Missense_Mutation	SNV	C	T	T	EPHA2	c.1697G>A	P.R566H
XH_P28	1	1904424	1904424	Missense_Mutation	SNV	C	T	T	CFAP74	c.737G>A	P.R246Q
XH_P28	1	19212008	19212008	Missense_Mutation	SNV	G	A	A	ALDH4A1	c.232C>T	P.P78S
XH_P28	1	201282405	201282405	Missense_Mutation	SNV	G	A	A	PKP1	c.418G>A	P.A140T
XH_P28	1	201984399	201984399	Missense_Mutation	SNV	G	A	A	ELF3	c.1064G>A	P.G355D
XH_P28	1	203798691	203798691	Silent	SNV	C	T	T	ZC3H11A	c.411C>T	P.S137S
XH_P28	1	235323959	235323959	Missense_Mutation	SNV	T	C	C	RBM34	c.232A>G	P.T78A
XH_P28	1	248570275	248570275	Missense_Mutation	SNV	T	C	C	OR2T1	c.980T>C	P.F327S
XH_P28	1	3328624	3328624	Silent	SNV	G	A	A	PRDM16	c.1863G>A	P.T621T
XH_P28	1	35334506	35334506	Missense_Mutation	SNV	G	A	A	DLGAP3	c.2185C>T	P.R729C
XH_P28	1	43232368	43232368	Missense_Mutation	SNV	G	A	A	P3H1	c.275C>T	P.S92F
XH_P28	1	63790160	63790160	Silent	SNV	G	A	A	FOXD3	c.1431G>A	P.A477A
XH_P28	1	6533472	6533472	Frame_Shift_Del	INDEL	C	-	-	PLEKHG5	c.802delG	P.E268fs
XH_P28	1	7724165	7724165	Missense_Mutation	SNV	C	T	T	CAMTA1	c.1558C>T	P.R520W
XH_P28	1	94522178	94522178	Silent	SNV	G	A	A	ABCA4	c.2361C>T	P.T787T
XH_P28	1	980820	980820	Frame_Shift_Ins	INDEL	-	G	G	AGRN	c.2459dupG	P.L821fs
XH_P28	10	100177413	100177413	Missense_Mutation	SNV	G	A	A	HPS1	c.2011C>T	P.H671Y
XH_P28	10	105238607	105238607	Silent	SNV	G	A	A	CALHM3	c.183C>T	P.L61L
XH_P28	10	105807524	105807524	Nonsense_Mutation	SNV	C	A	A	COL17A1	c.2308G>T	P.G770*
XH_P28	10	135085339	135085339	Silent	SNV	G	A	A	ADAM8	c.1077C>T	P.A359A
XH_P28	10	21806265	21806265	Missense_Mutation	SNV	C	T	T	SKIDA1	c.487G>A	P.A163T
XH_P28	10	76790232	76790232	Silent	SNV	C	T	T	KAT6B	c.5101C>T	P.L1701L
XH_P28	10	85956347	85956347	Missense_Mutation	SNV	G	A	A	CDHR1	c.238G>A	P.V80I
XH_P28	10	94450019	94450019	Silent	SNV	A	G	G	HHEX	c.276A>G	P.G92G
XH_P28	10	94712026	94712026	Splice_Site	SNV	A	G	G	EXOC6	c.1402-4A>G	
XH_P28	11	116717260	116717260	Missense_Mutation	SNV	G	A	A	SIK3	c.3161C>T	P.A1054V
XH_P28	11	121466482	121466482	Splice_Site	SNV	G	A	A	SORL1	c.4519+1G>A	
XH_P28	11	122805215	122805215	Missense_Mutation	SNV	C	G	G	C11orf63	c.1066C>G	P.Q356E
XH_P28	11	123908980	123908980	Silent	SNV	G	A	A	OR10G7	c.729C>T	P.H243H
XH_P28	11	128391866	128391866	Silent	SNV	C	T	T	ETS1	c.24G>A	P.K8K
XH_P28	11	133790624	133790624	Missense_Mutation	SNV	G	A	A	IGSF9B	c.2996C>T	P.P999L
XH_P28	11	44069683	44069683	Missense_Mutation	SNV	A	G	G	ACCSL	c.97A>G	P.T33A
XH_P28	11	6239872	6239872	Missense_Mutation	SNV	C	T	T	FAM160A2	c.1393G>A	P.A465T
XH_P28	11	63999443	63999443	Splice_Site	SNV	G	A	A	DNAJC4	c.180+7G>A	
XH_P28	11	6561152	6561152	Missense_Mutation	SNV	G	A	A	DNHD1	c.3467G>A	P.R1156Q
XH_P28	11	71729269	71729269	Frame_Shift_Ins	INDEL	-	T	T	NUMA1	c.937dupA	P.I313fs
XH_P28	11	73021808	73021808	Nonsense_Mutation	SNV	C	T	T	ARHGEF17	c.2125C>T	P.R709*
XH_P28	11	8118251	8118251	Frame_Shift_Ins	INDEL	-	T	T	TUB	c.753dupT	P.D252fs
XH_P28	11	94800405	94800405	Frame_Shift_Del	INDEL	C	-	-	SRSF8	c.20delC	P.P7fs
XH_P28	11	9754192	9754192	Nonsense_Mutation	SNV	C	T	T	SWAP70	c.841C>T	P.R281*
XH_P28	12	119540104	119540104	Silent	SNV	G	A	A	SRRM4	c.195G>A	P.Q65Q
XH_P28	12	120210692	120210692	Missense_Mutation	SNV	G	A	A	CIT	c.1964C>T	P.A655V
XH_P28	12	122674857	122674857	Silent	SNV	C	T	T	LRRC43	c.843C>T	P.D281D
XH_P28	12	129566528	129566528	Missense_Mutation	SNV	C	T	T	TMEM132D	c.1699G>A	P.G567S
XH_P28	12	133810910	133810910	Silent	SNV	A	G	G	ANHX	c.33T>C	P.H11H
XH_P28	12	2102299	2102299	Silent	SNV	A	G	G	DCP1B	c.450T>C	P.N150N
XH_P28	12	21615043	21615043	Missense_Mutation	SNV	C	T	T	PYROXD1	c.982C>T	P.H328Y
XH_P28	12	54757554	54757554	Frame_Shift_Del	INDEL	C	-	-	GPR84	c.82delG	P.V28fs
XH_P28	12	57396918	57396918	Missense_Mutation	SNV	G	A	A	ZBTB39	c.1784C>T	P.A595V
XH_P28	12	57858985	57858985	Missense_Mutation	SNV	C	T	T	GLI1	c.481C>T	P.R161W

XH_P28	12	6637841	6637841	Splice_Site	SNV	C	A	A	NCAPD2	c.3300-4C>A	
XH_P28	13	103387482	103387482	Missense_Mutation	SNV	T	C	C	CCDC168	c.15565A>G	P.K5189E
XH_P28	13	110835395	110835395	Silent	SNV	C	T	T	COL4A1	c.2040G>A	P.E680E
XH_P28	13	28959036	28959036	Missense_Mutation	SNV	A	T	T	FLT1	c.2102T>A	P.I701K
XH_P28	13	31232944	31232944	Silent	SNV	G	A	A	USPL1	c.2187G>A	P.P729P
XH_P28	14	100615721	100615721	Missense_Mutation	SNV	C	T	T	DEGS2	c.409G>A	P.G137S
XH_P28	14	103568923	103568923	Missense_Mutation	SNV	G	A	A	EXOC3L4	c.863G>A	P.R288H
XH_P28	14	103931985	103931985	Missense_Mutation	SNV	C	T	T	MARK3	c.632C>T	P.T211M
XH_P28	14	103932414	103932414	Missense_Mutation	SNV	C	T	T	MARK3	c.883C>T	P.R295C
XH_P28	14	104633185	104633185	Splice_Site	SNV	C	T	T	KIF26A	c.924-11C>T	
XH_P28	14	105688102	105688102	Missense_Mutation	SNV	C	T	T	BRF1	c.853G>A	P.E285K
XH_P28	14	23817149	23817149	Missense_Mutation	SNV	G	T	T	SLC22A17	c.241C>A	P.L81I
XH_P28	14	23865570	23865570	Silent	SNV	G	A	A	MYH6	c.2352C>T	P.S784S
XH_P28	14	44975941	44975941	Frame_Shift_Ins	INDEL	-	T	T	FSCB	c.249dupA	P.E84fs
XH_P28	14	55241717	55241717	Missense_Mutation	SNV	G	A	A	SAMD4A	c.1517G>A	P.R506Q
XH_P28	14	55818138	55818138	Missense_Mutation	SNV	G	T	T	FBXO34	c.1030G>T	P.G344W
XH_P28	14	62200939	62200939	Missense_Mutation	SNV	A	T	T	HIF1A	c.1036A>T	P.T346S
XH_P28	14	65209863	65209863	Silent	SNV	C	T	T	PLEKHG3	c.3102C>T	P.S1034S
XH_P28	14	70040029	70040029	Missense_Mutation	SNV	G	A	A	CCDC177	c.311C>T	P.A104V
XH_P28	14	71444720	71444720	Missense_Mutation	SNV	G	A	A	PCNX1	c.1666G>A	P.A556T
XH_P28	14	73614807	73614807	Missense_Mutation	SNV	G	A	A	PSEN1	c.80G>A	P.R27H
XH_P28	14	76147940	76147940	Silent	SNV	C	T	T	TTLL5	c.234C>T	P.R78R
XH_P28	14	76241937	76241937	Silent	SNV	C	T	T	TTLL5	c.2247C>T	P.A749A
XH_P28	14	86088574	86088574	Missense_Mutation	SNV	G	A	A	FLRT2	c.716G>A	P.R239H
XH_P28	14	90752749	90752749	Silent	SNV	G	A	A	NRDE2	c.3246C>T	P.S1082S
XH_P28	14	99641398	99641398	Missense_Mutation	SNV	G	A	A	BCL11B	c.1772C>T	P.A591V
XH_P28	14	99976715	99976715	Missense_Mutation	SNV	C	A	A	CCNK	c.1339C>A	P.L447M
XH_P28	15	35189856	35189856	Missense_Mutation	SNV	T	C	C	AQR	c.2294A>G	P.D765G
XH_P28	15	42700450	42700450	Missense_Mutation	SNV	G	T	T	CAPN3	c.1842G>T	P.E614D
XH_P28	15	43821376	43821376	Missense_Mutation	SNV	C	T	T	MAP1A	c.7705C>T	P.R2569C
XH_P28	15	44216401	44216401	Splice_Site	SNV	A	G	G	FRMD5	c.-61+2T>C	
XH_P28	15	84611424	84611424	Missense_Mutation	SNV	C	T	T	ADAMTSL3	c.2194C>T	P.H732Y
XH_P28	15	93588329	93588329	Missense_Mutation	SNV	G	A	A	RGMA	c.1276C>T	P.R426W
XH_P28	15	93595343	93595343	Silent	SNV	G	A	A	RGMA	c.549C>T	P.T183T
XH_P28	16	11370114	11370114	Silent	SNV	C	T	T	PRM2	c.114G>A	P.P38P
XH_P28	16	11773145	11773145	Missense_Mutation	SNV	G	A	A	TXNDC11	c.2864C>T	P.S955L
XH_P28	16	1417273	1417273	Silent	SNV	C	T	T	UNKL	c.354G>A	P.A118A
XH_P28	16	1706892	1706892	Missense_Mutation	SNV	C	T	T	CRAMP1	c.2134C>T	P.R712C
XH_P28	16	21726417	21726417	Missense_Mutation	SNV	G	A	A	OTOA	c.1195G>A	P.V399I
XH_P28	16	23544090	23544090	Splice_Site	SNV	C	T	T	EARS2	c.959-4G>A	
XH_P28	16	23598596	23598596	Missense_Mutation	SNV	C	T	T	NDUFAB1	c.213G>A	P.M71I
XH_P28	16	27481536	27481536	Silent	SNV	C	A	A	GTF3C1	c.4707G>T	P.V1569V
XH_P28	16	27709660	27709660	Missense_Mutation	SNV	C	T	T	KIAA0556	c.952C>T	P.R318W
XH_P28	16	2806550	2806550	Missense_Mutation	SNV	G	A	A	SRRM2	c.185G>A	P.R62H
XH_P28	16	2815737	2815737	Silent	SNV	C	T	T	SRRM2	c.5208C>T	P.A1736A
XH_P28	16	3076774	3076774	Missense_Mutation	SNV	G	A	A	THOC6	c.578G>A	P.R193Q
XH_P28	16	30999077	30999077	Splice_Site	SNV	C	T	T	HSD3B7	c.532-12C>T	
XH_P28	16	48149326	48149326	Missense_Mutation	SNV	C	A	A	ABCC12	c.1989G>T	P.Q663H
XH_P28	16	53342661	53342661	Missense_Mutation	SNV	G	A	A	CHD9	c.7117G>A	P.E2373K
XH_P28	16	55522566	55522566	Missense_Mutation	SNV	G	A	A	MMP2	c.794G>A	P.R265H
XH_P28	16	67198839	67198839	Splice_Site	SNV	T	C	C	HSF4	c.123+2T>C	
XH_P28	16	70292001	70292001	Silent	SNV	G	A	A	AARS	c.2112C>T	P.S704S
XH_P28	16	8722915	8722915	Silent	SNV	C	T	T	METTL22	c.462C>T	P.D154D
XH_P28	16	88008733	88008733	Silent	SNV	A	G	G	BANP	c.12A>G	P.E4E
XH_P28	16	8941640	8941640	Silent	SNV	G	A	A	PMM2	c.699G>A	P.A233A
XH_P28	16	89763967	89763967	Silent	SNV	C	T	T	SPATA2L	c.1050G>A	P.S350S
XH_P28	16	9858581	9858581	Missense_Mutation	SNV	A	T	T	GRIN2A	c.2820T>A	P.N940K
XH_P28	17	1631740	1631740	Missense_Mutation	SNV	G	A	A	WDR81	c.334G>A	P.V112M
XH_P28	17	17398719	17398719	Missense_Mutation	SNV	C	T	T	RASD1	c.566G>A	P.R189H
XH_P28	17	18022900	18022900	Silent	SNV	G	A	A	MYO15A	c.786G>A	P.A262A
XH_P28	17	21087119	21087119	Silent	SNV	G	A	A	DHRS7B	c.477G>A	P.T159T
XH_P28	17	26086032	26086032	Missense_Mutation	SNV	C	T	T	NOS2	c.3229G>A	P.G1077S
XH_P28	17	27068496	27068496	Missense_Mutation	SNV	G	A	A	NEK8	c.1957G>A	P.G653R
XH_P28	17	28544188	28544188	Missense_Mutation	SNV	C	T	T	SLC6A4	c.833G>A	P.G278D
XH_P28	17	32965105	32965105	Missense_Mutation	SNV	G	A	A	TMEM132E	c.3079G>A	P.A1027T
XH_P28	17	33738570	33738570	Silent	SNV	G	A	A	SLFN12	c.1524C>T	P.C508C
XH_P28	17	37879658	37879658	Missense_Mutation	SNV	G	A	A	ERBB2	c.2033G>A	P.R678Q
XH_P28	17	40500444	40500444	Missense_Mutation	SNV	G	A	A	STAT3	c.91C>T	P.R31W
XH_P28	17	4462215	4462215	Missense_Mutation	SNV	A	C	C	GGT6	c.577T>G	P.F193V
XH_P28	17	46655662	46655662	Frame_Shift_Del	INDEL	A	-	-	HOXB4	c.20delT	P.L7fs
XH_P28	17	5347661	5347661	Missense_Mutation	SNV	G	A	A	DHX33	c.1469C>T	P.P490L
XH_P28	17	61622511	61622511	Silent	SNV	C	T	T	KCNH6	c.2469C>T	P.H823H
XH_P28	17	62290817	62290817	Missense_Mutation	SNV	C	T	T	TEX2	c.761G>A	P.R254Q
XH_P28	17	6703429	6703429	Nonsense_Mutation	SNV	G	A	A	TEKT1	c.1174C>T	P.Q392*
XH_P28	17	7094084	7094084	Silent	SNV	A	G	G	DLG4	c.2109T>C	P.R703R
XH_P28	17	71387631	71387631	Silent	SNV	C	T	T	SDK2	c.3945G>A	P.T1315T
XH_P28	17	72843517	72843517	Missense_Mutation	SNV	G	A	A	GRIN2C	c.1931C>T	P.T644M
XH_P28	17	72956286	72956286	Silent	SNV	C	T	T	HID1	c.786G>A	P.V262V
XH_P28	17	73517808	73517808	Missense_Mutation	SNV	G	A	A	TSEN54	c.646G>A	P.A216T
XH_P28	17	74003351	74003351	Missense_Mutation	SNV	C	T	T	EVPL	c.6001G>A	P.E2001K
XH_P28	17	7401124	7401124	Silent	SNV	C	T	T	POLR2A	c.1137C>T	P.G379G
XH_P28	17	74163791	74163791	Silent	SNV	A	C	C	RNF157	c.384T>G	P.A128A
XH_P28	17	76130996	76130996	Missense_Mutation	SNV	G	A	A	TMC8	c.1033G>A	P.A345T
XH_P28	17	76137108	76137108	Missense_Mutation	SNV	C	T	T	TMC8	c.2096C>T	P.A699V
XH_P28	17	7754477	7754477	Missense_Mutation	SNV	C	G	G	KDM6B	c.3812C>G	P.S1271C
XH_P28	17	77808469	77808469	Silent	SNV	C	T	T	CBX4	c.972G>A	P.P324P
XH_P28	17	78081349	78081349	Splice_Site	SNV	C	T	T	GAA	c.693-7C>T	
XH_P28	17	78172202	78172202	Missense_Mutation	SNV	G	A	A	CARD14	c.1663G>A	P.V555I
XH_P28	17	80159666	80159666	Missense_Mutation	SNV	C	T	T	CCDC57	c.155G>A	P.C52Y
XH_P28	17	8926332	8926332	Silent	SNV	G	A	A	NTN1	c.642G>A	P.S214S
XH_P28	18	24035760	24035760	Missense_Mutation	SNV	G	A	A	KCTD1	c.721C>T	P.R241W
XH_P28	18	3879988	3879988	Silent	SNV	G	A	A	DLGAP1	c.81C>T	P.S27S
XH_P28	18	60241823	60241823	Missense_Mutation	SNV	G	A	A	ZCCHC2	c.2509G>A	P.G837R
XH_P28	18	7015799	7015799	Silent	SNV	G	A	A	LAMA1	c.3048C>T	P.C1016C
XH_P28	18	7023335	7023335	Silent	SNV	G	A	A	LAMA1	c.2529C>T	P.G843G
XH_P28	18	74208486	74208487	Frame_Shift_Del	INDEL	GG	-	-	C18orf65	c.336_337delGG	P.A113fs
XH_P28	19	10610139	10610139	Missense_Mutation	SNV	C	T	T	KEAP1	c.571G>A	P.A191T
XH_P28	19	15122052	15122052	Missense_Mutation	SNV	C	T	T	CCDC105	c.415C>T	P.R139C

XH_P28	19	16466591	16466591	Silent	SNV	C	T	T	EPS15L1	c.2658G>A	P.A886A
XH_P28	19	17392616	17392616	Missense_Mutation	SNV	G	A	A	ANKLE1	c.49G>A	P.G17S
XH_P28	19	17450246	17450246	Missense_Mutation	SNV	G	A	A	GTPBP3	c.812G>A	P.R271Q
XH_P28	19	20228702	20228702	Frame_Shift_Del	INDEL	A	-	-	ZNF90	c.345delA	P.G116fs
XH_P28	19	30164847	30164847	Missense_Mutation	SNV	G	A	A	PLEKHF1	c.101G>A	P.R34Q
XH_P28	19	30935520	30935520	Missense_Mutation	SNV	G	A	A	ZNF536	c.1051G>A	P.G351S
XH_P28	19	31770647	31770647	Missense_Mutation	SNV	C	T	T	TSHZ3	c.52G>A	P.E18K
XH_P28	19	35758381	35758381	Missense_Mutation	SNV	A	C	C	LSR	c.1598A>C	P.D533A
XH_P28	19	36221512	36221512	Silent	SNV	C	T	T	KMT2B	c.5271C>T	P.G1757G
XH_P28	19	38055624	38055624	Missense_Mutation	SNV	C	T	T	ZNF571	c.1706G>A	P.R569H
XH_P28	19	38609985	38609985	Frame_Shift_Del	INDEL	C	-	-	SIPA1L3	c.2337delC	P.I780fs
XH_P28	19	39669208	39669208	Missense_Mutation	SNV	C	T	T	PAK4	c.1765C>T	P.R589C
XH_P28	19	3977539	3977539	Missense_Mutation	SNV	C	T	T	EEF2	c.2137G>A	P.A713T
XH_P28	19	39799069	39799069	Missense_Mutation	SNV	G	A	A	LRFN1	c.1520C>T	P.T507M
XH_P28	19	39888220	39888220	Missense_Mutation	SNV	G	T	T	MED29	c.477G>T	P.W159C
XH_P28	19	40587790	40587790	Silent	SNV	C	T	T	ZNF780A	c.168G>A	P.T56T
XH_P28	19	41113263	41113263	Splice_Site	SNV	C	T	T	LTBP4	c.1193-8C>T	
XH_P28	19	41173899	41173907	Missense_Mutation	INDEL	TGCTGTTGC	-	-	NUMBL	3_1181delGCAA	P.Q392_Q394del
XH_P28	19	41351919	41351919	Silent	SNV	G	C	C	CYP2A6	c.915C>G	P.T305T
XH_P28	19	42569497	42569497	Missense_Mutation	SNV	C	T	T	GRIK5	c.122G>A	P.R41H
XH_P28	19	44612194	44612194	Silent	SNV	G	A	A	ZNF224	c.1881G>A	P.E627E
XH_P28	19	45974060	45974060	Silent	SNV	G	A	A	FOSB	c.300G>A	P.P100P
XH_P28	19	47341769	47341769	Missense_Mutation	SNV	C	T	T	AP2S1	c.412G>A	P.E138K
XH_P28	19	47570024	47570024	Silent	SNV	C	T	T	ZC3H4	c.3501G>A	P.T1167T
XH_P28	19	47935484	47935484	Missense_Mutation	SNV	C	T	T	SLC8A2	c.2329G>A	P.V777I
XH_P28	19	48955975	48955975	Missense_Mutation	SNV	C	T	T	GRWD1	c.1034C>T	P.P345L
XH_P28	19	50100978	50100978	Missense_Mutation	SNV	G	A	A	PRR12	c.3386G>A	P.R1129H
XH_P28	19	50165782	50165782	Silent	SNV	G	A	A	IRF3	c.501C>T	P.C167C
XH_P28	19	50266381	50266381	Missense_Mutation	SNV	G	A	A	TSKS	c.124C>T	P.R42W
XH_P28	19	50334697	50334697	Silent	SNV	C	T	T	MED25	c.1209C>T	P.S403S
XH_P28	19	50338373	50338373	Missense_Mutation	SNV	G	A	A	MED25	c.1613G>A	P.R538Q
XH_P28	19	50917032	50917032	Nonsense_Mutation	SNV	C	T	T	POLD1	c.2284C>T	P.R762*
XH_P28	19	51455893	51455893	Frame_Shift_Del	INDEL	G	-	-	KLK5	c.21delC	P.W8fs
XH_P28	19	55665463	55665463	Missense_Mutation	SNV	G	A	A	TNNI3	c.481C>T	P.R161W
XH_P28	19	55708681	55708681	Nonsense_Mutation	SNV	G	C	C	PTPRH	c.1260C>G	P.Y420*
XH_P28	19	55993414	55993414	Missense_Mutation	SNV	C	T	T	ZNF628	c.854C>T	P.A285V
XH_P28	19	56090637	56090637	Silent	SNV	G	A	A	ZNF579	c.369C>T	P.H123H
XH_P28	19	56156372	56156372	Silent	SNV	C	T	T	ZNF581	c.435C>T	P.H145H
XH_P28	19	58991281	58991281	Splice_Site	INDEL	C	-	-	ZNF446	c.713-11delC	
XH_P28	19	59028908	59028908	Missense_Mutation	SNV	G	A	A	ZBTB45	c.133C>T	P.R45C
XH_P28	19	6185636	6185636	Silent	SNV	T	G	G	ACSBG2	c.1512T>G	P.G504G
XH_P28	19	815231	815231	Missense_Mutation	SNV	C	T	T	PLPPR3	c.358G>A	P.G120S
XH_P28	2	160229572	160229572	Silent	SNV	G	A	A	BAZ2B	c.4089C>T	P.G1363G
XH_P28	2	17692078	17692078	Missense_Mutation	SNV	T	A	A	RAD51AP2	c.3473A>T	P.N1158I
XH_P28	2	179441464	179441464	Silent	SNV	G	A	A	TTN	c.64584C>T	P.S21528S
XH_P28	2	179616465	179616465	Silent	SNV	A	G	G	TTN	c.10662T>C	P.A3554A
XH_P28	2	189873814	189873814	Silent	SNV	C	T	T	COL3A1	c.3690C>T	P.N1230N
XH_P28	2	202614445	202614445	Missense_Mutation	SNV	C	T	T	ALS2	c.1805G>A	P.R602H
XH_P28	2	218675013	218675013	Missense_Mutation	SNV	C	T	T	TNS1	c.4708G>A	P.G1570S
XH_P28	2	222428935	222428935	Silent	SNV	G	A	A	EPHA4	c.339C>T	P.G113G
XH_P28	2	232392908	232392908	Missense_Mutation	SNV	G	A	A	NMUR1	c.824C>T	P.A275V
XH_P28	2	233684582	233684582	Nonsense_Mutation	SNV	C	T	T	GIGYF2	c.2416C>T	P.R806*
XH_P28	2	241405579	241405579	Missense_Mutation	SNV	G	A	A	GPC1	c.1549G>A	P.A517T
XH_P28	2	241689855	241689855	Missense_Mutation	SNV	C	A	A	KIF1A	c.2968G>T	P.A990S
XH_P28	2	32800277	32800277	Missense_Mutation	SNV	G	A	A	BIRC6	c.13199G>A	P.R4400Q
XH_P28	2	48026540	48026540	Missense_Mutation	SNV	T	C	C	MSH6	c.1418T>C	P.L473P
XH_P28	2	69049599	69049599	Missense_Mutation	SNV	C	T	T	ARHGAP25	c.1328C>T	P.T443I
XH_P28	2	85786207	85786207	Splice_Site	SNV	G	C	C	GGCX	c.215-9C>G	
XH_P28	2	9484764	9484764	Missense_Mutation	SNV	G	A	A	ASAP2	c.953G>A	P.G318E
XH_P28	20	21687066	21687066	Splice_Site	SNV	G	A	A	PAX1	c.287-10G>A	
XH_P28	20	259914	259914	Missense_Mutation	SNV	G	T	T	C20orf96	c.361C>A	P.L121I
XH_P28	20	31044128	31044128	Silent	SNV	C	T	T	NOL4L	c.912G>A	P.T304T
XH_P28	20	32666332	32666332	Missense_Mutation	SNV	G	A	A	RALY	c.850G>A	P.A284T
XH_P28	20	33588465	33588465	Silent	SNV	C	T	T	MYH7B	c.5277C>T	P.T1759T
XH_P28	20	35060130	35060130	Missense_Mutation	SNV	C	A	A	DLGAP4	c.10C>A	P.L4I
XH_P28	20	42142604	42142604	Missense_Mutation	SNV	C	T	T	L3MBTL1	c.197C>T	P.P66L
XH_P28	20	42744935	42744935	Silent	SNV	C	T	T	JPH2	c.1380G>A	P.A460A
XH_P28	20	44577619	44577619	Silent	SNV	G	A	A	ZNF335	c.4002C>T	P.Y1334Y
XH_P28	20	44987147	44987147	Missense_Mutation	SNV	C	T	T	SLC35C2	c.86G>A	P.R29Q
XH_P28	20	48522829	48522829	Missense_Mutation	SNV	G	A	A	SPATA2	c.890C>T	P.S297L
XH_P28	20	48760152	48760152	Missense_Mutation	SNV	C	T	T	TMEM189	c.128G>A	P.R43H
XH_P28	20	50406788	50406788	Missense_Mutation	SNV	C	T	T	SALL4	c.2234G>A	P.R745H
XH_P28	20	51870090	51870090	Missense_Mutation	SNV	G	T	T	TSHZ2	c.84G>T	P.E28D
XH_P28	20	51871597	51871597	Missense_Mutation	SNV	G	A	A	TSHZ2	c.1591G>A	P.G531R
XH_P28	20	54972283	54972283	Missense_Mutation	SNV	G	A	A	CSTF1	c.190G>A	P.A64T
XH_P28	20	55758870	55758870	Missense_Mutation	SNV	C	T	T	BMP7	c.866G>A	P.R289H
XH_P28	20	60895821	60895821	Silent	SNV	G	A	A	LAMA5	c.6622C>T	P.L2208L
XH_P28	20	60913389	60913389	Missense_Mutation	SNV	C	T	T	LAMA5	c.1655G>A	P.R552H
XH_P28	20	60966461	60966461	Silent	SNV	G	A	A	CABLES2	c.1140C>T	P.P380P
XH_P28	20	61288203	61288203	Missense_Mutation	SNV	C	T	T	SLCO4A1	c.397C>T	P.R133C
XH_P28	20	62577812	62577812	Missense_Mutation	SNV	C	T	T	UCKL1	c.253G>A	P.A85T
XH_P28	20	62701931	62701931	Silent	SNV	G	A	A	TCEA2	c.762G>A	P.T254T
XH_P28	20	7962997	7962997	Silent	SNV	A	G	G	TMX4	c.951T>C	P.A317A
XH_P28	20	9404515	9404515	Nonsense_Mutation	SNV	C	T	T	PLCB4	c.2404C>T	P.R802*
XH_P28	21	45522737	45522737	Missense_Mutation	SNV	C	A	A	TRAPPC10	c.3425C>A	P.T1142N
XH_P28	22	17619172	17619172	Silent	SNV	C	T	T	CECR5	c.921G>A	P.A307A
XH_P28	22	18300666	18300666	Silent	SNV	C	T	T	MICAL3	c.4761G>A	P.A1587A
XH_P28	22	24622121	24622121	Silent	SNV	G	A	A	GGT5	c.1152C>T	P.H384H
XH_P28	22	29445904	29445904	Missense_Mutation	SNV	G	A	A	ZNRF3	c.1735G>A	P.V579M
XH_P28	22	29735018	29735018	Silent	SNV	G	A	A	AP1B1	c.2124C>T	P.G708G
XH_P28	22	40417522	40417522	Silent	SNV	G	A	A	FAM83F	c.1008G>A	P.P336P
XH_P28	22	47057349	47057349	Splice_Site	SNV	G	A	A	GRAMD4	c.466+10G>A	
XH_P28	3	125298752	125298752	Silent	SNV	G	T	T	OSBPL11	c.366C>A	P.T122T
XH_P28	3	132280031	132280031	Missense_Mutation	SNV	G	A	A	ACAD11	c.2032C>T	P.R678C
XH_P28	3	138738766	138738766	Silent	SNV	C	T	T	PRR23B	c.738G>A	P.A246A
XH_P28	3	151163149	151163149	Silent	SNV	G	A	A	IGSF10	c.4620C>T	P.H1540H
XH_P28	3	15116352	15116352	Missense_Mutation	SNV	C	T	T	RBSN	c.1292G>A	P.R431H
XH_P28	3	178916936	178916936	Missense_Mutation	SNV	G	A	A	PIK3CA	c.323G>A	P.R108H

XH_P28	3	179437801	179437801	Silent	SNV	T	C	C	USP13	c.879T>C	P.I293I
XH_P28	3	183975265	183975265	Silent	SNV	C	T	T	ECE2	c.201C>T	P.C67C
XH_P28	3	184429232	184429232	Silent	SNV	C	T	T	MAGEF1	c.378G>A	P.R126R
XH_P28	3	26751633	26751633	Missense_Mutation	SNV	T	C	C	LRRC3B	c.470T>C	P.L157P
XH_P28	3	42236377	42236377	Missense_Mutation	SNV	A	C	C	TRAK1	c.1057A>C	P.K353Q
XH_P28	3	52712520	52712520	Nonsense_Mutation	SNV	G	A	A	PBRM1	c.232C>T	P.R78*
XH_P28	4	144616958	144616958	Missense_Mutation	SNV	A	G	G	FREM3	c.4871T>C	P.V1624A
XH_P28	4	153303455	153303455	Silent	SNV	A	G	G	FBXW7	c.33T>C	P.H11H
XH_P28	4	15937774	15937774	Missense_Mutation	SNV	G	A	A	FGFBP1	c.482C>T	P.P161L
XH_P28	4	169196555	169196555	Nonsense_Mutation	SNV	G	A	A	DDX60	c.2245C>T	P.R749*
XH_P28	4	184932524	184932524	Missense_Mutation	SNV	G	A	A	STOX2	c.2533G>A	P.D845N
XH_P28	4	2460957	2460957	Missense_Mutation	SNV	G	A	A	CFAP99	c.1427G>A	P.R476H
XH_P28	4	3240637	3240637	Silent	SNV	C	T	T	HTT	c.9147C>T	P.V3049V
XH_P28	4	47408822	47408822	Missense_Mutation	SNV	C	T	T	GABRB1	c.959C>T	P.A320V
XH_P28	4	52861210	52861210	Missense_Mutation	SNV	C	T	T	LRRC66	c.1978G>A	P.D660N
XH_P28	4	55956164	55956164	Missense_Mutation	SNV	G	A	A	KDR	c.3151C>T	P.R1051W
XH_P28	4	66197723	66197723	Silent	SNV	A	G	G	EPHA5	c.2976T>C	P.S992S
XH_P28	4	83785565	83785565	Frame_Shift_Del	INDEL	T	-	-	SEC31A	c.1384delA	P.I462fs
XH_P28	4	88047293	88047293	Frame_Shift_Del	INDEL	C	-	-	AFF1	c.2621delC	P.P874fs
XH_P28	5	134364584	134364584	Missense_Mutation	SNV	G	A	A	PITX1	c.830C>T	P.T277M
XH_P28	5	134782185	134782185	Missense_Mutation	SNV	G	A	A	DCANP1	c.614C>T	P.S205L
XH_P28	5	138730227	138730227	Missense_Mutation	SNV	C	T	T	PROB1	c.544G>A	P.A182T
XH_P28	5	140222691	140222691	Silent	SNV	C	T	T	PCDHA8	c.1785C>T	P.R595R
XH_P28	5	148748030	148748030	Missense_Mutation	SNV	C	T	T	PCYOX1L	c.1247C>T	P.T416M
XH_P28	5	16699587	16699587	Silent	SNV	C	T	T	MYO10	c.3528G>A	P.P1176P
XH_P28	5	16701243	16701243	Silent	SNV	G	A	A	MYO10	c.3261C>T	P.G1087G
XH_P28	5	175957711	175957711	Missense_Mutation	SNV	G	A	A	RNF44	c.673C>T	P.R225C
XH_P28	5	178549673	178549673	Silent	SNV	C	T	T	ADAMTS2	c.3060G>A	P.A1020A
XH_P28	5	178581175	178581175	Silent	SNV	G	A	A	ADAMTS2	c.1257C>T	P.D419D
XH_P28	5	180036991	180036991	Missense_Mutation	SNV	C	T	T	FLT4	c.3721G>A	P.A1241T
XH_P28	5	31294176	31294176	Silent	SNV	C	A	A	CDH6	c.336C>A	P.A112A
XH_P28	5	35667295	35667295	Missense_Mutation	SNV	C	T	T	SPEF2	c.1289C>T	P.A430V
XH_P28	5	40692086	40692086	Missense_Mutation	SNV	G	A	A	PTGER4	c.1073G>A	P.R358H
XH_P28	5	92929447	92929447	Missense_Mutation	SNV	G	A	A	NR2F1	c.1171G>A	P.V391I
XH_P28	6	12120605	12120605	Missense_Mutation	SNV	A	G	G	HIVEP1	c.577A>G	P.N193D
XH_P28	6	143755092	143755092	Silent	SNV	G	T	T	ADAT2	c.87C>A	P.A29A
XH_P28	6	161771192	161771192	Missense_Mutation	SNV	C	T	T	PARK2	c.1337G>A	P.C446Y
XH_P28	6	47522495	47522495	Silent	SNV	C	T	T	CD2AP	c.534C>T	P.D178D
XH_P28	6	7247252	7247252	Silent	SNV	G	A	A	RREB1	c.4404G>A	P.A1468A
XH_P28	7	102962480	102962480	Missense_Mutation	SNV	C	T	T	DNAJC2	c.982G>A	P.E328K
XH_P28	7	107596022	107596022	Missense_Mutation	SNV	C	T	T	LAMB1	c.2744G>A	P.R915H
XH_P28	7	117865007	117865007	Silent	SNV	G	A	A	ANKRD7	c.123G>A	P.L41L
XH_P28	7	121650957	121650957	Silent	SNV	T	C	C	PTPRZ1	c.1857T>C	P.Y619Y
XH_P28	7	148463663	148463663	Missense_Mutation	SNV	G	A	A	CUL1	c.800G>A	P.R267H
XH_P28	7	149946093	149946093	Silent	SNV	C	T	T	ACTR3C	c.615G>A	P.L205L
XH_P28	7	150767819	150767819	Missense_Mutation	SNV	G	A	A	SLC4A2	c.1570G>A	P.V524M
XH_P28	7	157333462	157333462	Silent	SNV	C	T	T	PTPRN2	c.2880G>A	P.A960A
XH_P28	7	20687580	20687580	Splice_Site	INDEL	-	T	T	ABCB5	c.1096-6dupT	
XH_P28	7	50450356	50450356	Silent	SNV	C	T	T	IKZF1	c.540C>T	P.Y180Y
XH_P28	7	65551627	65551627	Missense_Mutation	SNV	C	T	T	ASL	c.502C>T	P.R168C
XH_P28	7	73790458	73790458	Missense_Mutation	SNV	C	T	T	CLIP2	c.1727C>T	P.A576V
XH_P28	7	98524852	98524852	Missense_Mutation	SNV	C	T	T	TRRAP	c.3038C>T	P.P1013L
XH_P28	7	98547209	98547209	Splice_Site	SNV	C	T	T	TRRAP	c.4932+5C>T	
XH_P28	7	98552822	98552822	Silent	SNV	C	T	T	TRRAP	c.5811C>T	P.D1937D
XH_P28	8	100654627	100654627	Missense_Mutation	SNV	C	T	T	VPS13B	c.5884C>T	P.P1962S
XH_P28	8	123965564	123965564	Missense_Mutation	SNV	G	A	A	ZHX2	c.1814G>A	P.G605E
XH_P28	8	124658147	124658147	Silent	SNV	C	T	T	KLHL38	c.1578G>A	P.T526T
XH_P28	8	125570011	125570011	Missense_Mutation	SNV	G	A	A	MTSS1	c.1153C>T	P.R385W
XH_P28	8	135621035	135621035	Missense_Mutation	SNV	C	T	T	ZFAT	c.686G>A	P.R229Q
XH_P28	8	14181673	14181673	Missense_Mutation	SNV	C	T	T	SGCZ	c.53G>A	P.R18Q
XH_P28	8	144809687	144809687	Silent	SNV	G	A	A	FAM83H	c.1944C>T	P.G648G
XH_P28	8	145541365	145541365	Missense_Mutation	SNV	G	T	T	DGAT1	c.905C>A	P.P302H
XH_P28	8	22021465	22021465	Missense_Mutation	SNV	G	A	A	SFTPC	c.487G>A	P.A163T
XH_P28	8	22085787	22085787	Silent	SNV	G	A	A	PHYHIP	c.84C>T	P.D28D
XH_P28	8	23106880	23106880	Missense_Mutation	SNV	G	A	A	CHMP7	c.127G>A	P.V43M
XH_P28	8	73848281	73848281	Missense_Mutation	SNV	C	T	T	KCNB2	c.691C>T	P.R231C
XH_P28	8	87076326	87076326	Silent	SNV	G	T	T	PSKH2	c.720C>A	P.T240T
XH_P28	8	98656857	98656857	Silent	SNV	G	A	A	MTDH	c.123G>A	P.G41G
XH_P28	9	108424982	108424982	Missense_Mutation	SNV	G	A	A	TAL2	c.205G>A	P.A69T
XH_P28	9	115652533	115652533	Silent	SNV	G	A	A	SLC46A2	c.429C>T	P.N143N
XH_P28	9	117063343	117063343	Splice_Site	SNV	C	A	A	COL27A1	c.4702-9C>A	
XH_P28	9	117826999	117826999	Silent	SNV	C	T	T	TNC	c.3414G>A	P.T1138T
XH_P28	9	123205916	123205916	Missense_Mutation	SNV	G	T	T	CDK5RAP2	c.3130C>A	P.Q1044K
XH_P28	9	127253433	127253433	Silent	SNV	C	T	T	NR5A1	c.1065G>A	P.V355V
XH_P28	9	130886841	130886841	Splice_Site	SNV	A	G	G	PTGES2	c.-92-12T>C	
XH_P28	9	133257693	133257693	Splice_Site	SNV	C	T	T	HMCN2	c.6871+9C>T	
XH_P28	9	133738371	133738371	Silent	SNV	C	T	T	ABL1	c.771C>T	P.Y257Y
XH_P28	9	136298805	136298805	Missense_Mutation	SNV	C	T	T	ADAMTS13	c.1289C>T	P.A430V
XH_P28	9	136310047	136310047	Silent	SNV	C	T	T	ADAMTS13	c.2484C>T	P.N828N
XH_P28	9	139818433	139818433	Missense_Mutation	SNV	G	A	A	TRAF2	c.1268G>A	P.R423Q
XH_P28	9	139909376	139909376	Missense_Mutation	SNV	G	A	A	ABCA2	c.3871C>T	P.R1291C
XH_P28	9	5044464	5044464	Nonsense_Mutation	SNV	C	T	T	JAK2	c.412C>T	P.R138*
XH_P28	X	100088318	100088318	Missense_Mutation	SNV	C	T	T	CSTF2	c.1417C>T	P.R473C
XH_P28	X	103268037	103268037	Missense_Mutation	SNV	G	A	A	H2BFWT	c.196C>T	P.R66C
XH_P28	X	104465052	104465052	Silent	SNV	G	A	A	TEX13A	c.30C>T	P.S10S
XH_P28	X	141291651	141291651	Silent	SNV	G	A	A	MAGEC2	c.123C>T	P.S41S
XH_P28	X	152612997	152612997	Missense_Mutation	SNV	G	A	A	ZNF275	c.854G>A	P.R285H
XH_P28	X	153039681	153039681	Missense_Mutation	SNV	G	A	A	PLXNB3	c.3629G>A	P.R1210H
XH_P28	X	153041522	153041522	Missense_Mutation	SNV	G	A	A	PLXNB3	c.4651G>A	P.G1551R
XH_P28	X	153132320	153132320	Missense_Mutation	SNV	G	A	A	L1CAM	c.2215C>T	P.R739W
XH_P28	X	153135113	153135113	Missense_Mutation	SNV	C	T	T	L1CAM	c.1129G>A	P.A377T
XH_P28	X	153591108	153591108	Silent	SNV	G	A	A	FLNA	c.2325C>T	P.Y775Y
XH_P28	X	38144935	38144935	Frame_Shift_Del	INDEL	T	-	-	RPGR	c.3317delA	P.K1106fs
XH_P28	X	44918338	44918338	Nonsense_Mutation	SNV	G	A	A	KDM6A	c.963G>A	P.W321*
XH_P28	X	47034427	47034427	Missense_Mutation	SNV	G	A	A	RBM10	c.512G>A	P.R171Q
XH_P28	X	48674984	48674984	Missense_Mutation	SNV	G	A	A	HDAC6	c.1777G>A	P.A593T
XH_P28	X	49032283	49032283	Silent	SNV	A	G	G	PRICKLE3	c.1383T>C	P.R461R
XH_P28	X	50378589	50378589	Missense_Mutation	SNV	G	T	T	SHROOM4	c.484C>A	P.L162M

XH_P28	X	51076108	51076108	Silent	SNV	A	G	G	NUDT10	c.291A>G	P.V97V
XH_P28	X	53240966	53240966	Splice_Site	SNV	T	C	C	KDM5C	c.1242+3A>G	
XH_P28	X	53966652	53966652	Missense_Mutation	SNV	G	A	A	PHF8	c.3055C>T	P.R1019C
XH_P28	X	54783390	54783390	Splice_Site	SNV	G	T	T	ITIH6	c.3109+8C>A	
XH_P28	X	5810919	5810919	Missense_Mutation	SNV	G	T	T	NLGN4X	c.2390C>A	P.T797N
XH_P28	X	67731806	67731806	Missense_Mutation	SNV	T	C	C	YIPF6	c.173T>C	P.V58A
XH_P28	X	69255333	69255333	Silent	SNV	C	T	T	EDA	c.1044C>T	P.G348G
XH_P28	X	70347916	70347916	Missense_Mutation	SNV	G	T	T	MED12	c.3155G>T	P.S1052I
XH_P28	X	9863793	9863793	Silent	SNV	C	T	T	SHROOM2	c.1845C>T	P.H615H
XH_P29	1	201181334	201181334	Missense_Mutation	SNV	A	G	G	IGFN1	c.7313A>G	P.H2438R
XH_P29	1	6523161	6523161	Missense_Mutation	SNV	C	G	G	TNFRSF25	c.589G>C	P.A197P
XH_P29	11	65487511	65487511	Splice_Site	SNV	C	T	T	RNASEH2C	c.468+5G>A	
XH_P29	11	71671823	71671823	Silent	SNV	G	A	A	RNF121	c.33G>A	P.K11K
XH_P29	12	100784828	100784828	Frame_Shift_Ins	INDEL	-	T	T	SLC17A8	c.410dupT	P.W138fs
XH_P29	12	25398284	25398284	Missense_Mutation	SNV	C	T	T	KRAS	c.35G>A	P.G12D
XH_P29	12	94673379	94673379	Frame_Shift_Ins	INDEL	-	A	A	PLXNC1	c.3734dupA	P.N1245fs
XH_P29	17	34958339	34958339	Missense_Mutation	SNV	G	A	A	MRM1	c.100G>A	P.E34K
XH_P29	17	80788076	80788076	Missense_Mutation	SNV	G	A	A	ZNF750	c.2114C>T	P.A705V
XH_P29	18	60230287	60230287	Silent	SNV	A	G	G	ZCCHC2	c.1596A>G	P.Q532Q
XH_P29	19	36205949	36205949	Nonsense_Mutation	SNV	G	T	T	ZBTB32	c.421G>T	P.E141*
XH_P29	19	54632467	54632467	Missense_Mutation	SNV	C	G	G	PRPF31	c.1182C>G	P.F394L
XH_P29	19	5587916	5587916	Silent	SNV	G	A	A	SAFB2	c.2601C>T	P.D867D
XH_P29	19	8807973	8807973	Missense_Mutation	SNV	C	T	T	ACTL9	c.1079G>A	P.R360H
XH_P29	3	112267425	112267425	Missense_Mutation	SNV	C	G	G	ATG3	c.298G>C	P.E100Q
XH_P29	3	178952085	178952085	Missense_Mutation	SNV	A	G	G	PIK3CA	c.3140A>G	P.H1047R
XH_P29	4	68528872	68528872	Missense_Mutation	SNV	C	T	T	UBA6	c.1022G>A	P.R341H
XH_P29	4	83552526	83552526	Missense_Mutation	SNV	G	T	T	SCD5	c.947C>A	P.P316Q
XH_P29	4	982996	982996	Silent	SNV	G	A	A	SLC26A1	c.1731C>T	P.G577G
XH_P29	5	180036033	180036033	Silent	SNV	C	T	T	FLT4	c.3828G>A	P.G1276G
XH_P29	5	23521146	23521146	Silent	SNV	G	A	A	PRDM9	c.366G>A	P.A122A
XH_P29	6	108067995	108067995	Missense_Mutation	SNV	C	T	T	SCML4	c.211G>A	P.A71T
XH_P29	7	154767930	154767930	Missense_Mutation	SNV	G	A	A	PAXIP1	c.550C>T	P.R184C
XH_P29	7	71036298	71036298	Frame_Shift_Ins	INDEL	-	T	T	WBSCR17	c.992dupT	P.V332fs
XH_P29	8	101195601	101195601	Splice_Site	SNV	A	C	C	SPAG1	c.427-2A>C	
XH_P29	8	90921791	90921791	Splice_Site	SNV	A	T	T	OSGIN2	c.45-4A>T	
XH_P29	9	133281519	133281519	Missense_Mutation	SNV	C	T	T	HMCN2	c.10843C>T	P.R3615C
XH_P29	9	34658622	34658622	Frame_Shift_Ins	INDEL	-	G	G	IL11RA	c.752_753insG	P.H251fs
XH_P29	9	34658623	34658623	Frame_Shift_Ins	INDEL	-	CCCAG	CCCAG	IL11RA	753_754insCCC	P.F252fs
XH_P29	X	27839966	27839966	Silent	SNV	C	T	T	MAGEB10	c.543C>T	P.V181V
XH_P30	1	173769667	173769667	Splice_Site	SNV	G	T	T	CENPL	c.1102-10C>A	
XH_P30	1	248570073	248570073	Missense_Mutation	SNV	C	T	T	OR2T1	c.778C>T	P.P260S
XH_P30	11	94331083	94331083	Splice_Site	SNV	T	G	G	PIWIL4	c.1380+2T>G	
XH_P30	12	107486581	107486581	Splice_Site	SNV	C	T	T	CRY1	c.158+1G>A	
XH_P30	12	53410303	53410303	Missense_Mutation	SNV	T	G	G	EIF4B	c.60T>G	P.F20L
XH_P30	15	66727457	66727457	Missense_Mutation	SNV	A	C	C	MAP2K1	c.173A>C	P.Q58P
XH_P30	17	42290196	42290196	Silent	SNV	C	T	T	UBTF	c.651G>A	P.V217V
XH_P30	18	21111668	21111668	Nonstop_Mutation	SNV	A	G	G	C18orf8	c.1974A>G	P.Ter658Wext*?
XH_P30	19	18109195	18109195	Missense_Mutation	SNV	G	A	A	KCNN1	c.1612G>A	P.A538T
XH_P30	19	58005441	58005441	Missense_Mutation	SNV	A	G	G	ZNF419	c.1519A>G	P.T507A
XH_P30	2	128263138	128263138	Missense_Mutation	SNV	T	C	C	IWS1	c.341A>G	P.N114S
XH_P30	2	86258708	86258708	Silent	SNV	G	A	A	POLR1A	c.4323C>T	P.D1441D
XH_P30	20	10626616	10626616	Splice_Site	INDEL	-	A	A	JAG1	c.1999+2dupT	
XH_P30	20	33875303	33875303	Missense_Mutation	SNV	G	A	A	FAM83C	c.1279C>T	P.P427S
XH_P30	3	142841044	142841044	Silent	SNV	G	A	A	CHST2	c.1386G>A	P.S462S
XH_P30	3	164751200	164751200	Missense_Mutation	SNV	T	A	A	SI	c.2548A>T	P.T850S
XH_P30	3	45817409	45817409	Silent	SNV	C	T	T	SLC6A20	c.426G>A	P.A142A
XH_P30	3	73114721	73114721	Missense_Mutation	SNV	G	A	A	PPP4R2	c.931G>A	P.G311S
XH_P30	4	153247373	153247373	Missense_Mutation	SNV	C	T	T	FBXW7	c.1429G>A	P.G477S
XH_P30	5	153065788	153065788	Nonsense_Mutation	SNV	C	T	T	GRIA1	c.1033C>T	P.R345*
XH_P30	7	43679239	43679239	Missense_Mutation	SNV	T	A	A	COA1	c.383A>T	P.Q128L
XH_P30	9	137593143	137593143	Silent	SNV	G	A	A	COL5A1	c.618G>A	P.V206V
XH_P30	X	101096518	101096518	Missense_Mutation	SNV	C	G	G	NXF5	c.253G>C	P.V85L
XH_P31	1	151773897	151773897	Silent	SNV	G	T	T	LINGO4	c.1284C>A	P.G428G
XH_P31	1	169366603	169366603	Silent	SNV	A	T	T	CCDC181	c.1239T>A	P.A413A
XH_P31	10	95162004	95162004	Silent	SNV	G	A	A	MYOF	c.975C>T	P.T325T
XH_P31	11	46332740	46332740	Missense_Mutation	SNV	C	A	A	CREB3L1	c.753C>A	P.H251Q
XH_P31	11	5411379	5411379	Nonsense_Mutation	SNV	C	T	T	OR51M1	c.751C>T	P.Q251*
XH_P31	11	7111056	7111056	Silent	SNV	C	T	T	RBMXL2	c.705C>T	P.P235P
XH_P31	12	12940193	12940193	Silent	SNV	G	A	A	APOLD1	c.447G>A	P.R149R
XH_P31	12	176434	176434	Missense_Mutation	SNV	G	T	T	IQSEC3	c.386G>T	P.G129V
XH_P31	12	56481922	56481922	Missense_Mutation	SNV	G	A	A	ERBB3	c.850G>A	P.G284R
XH_P31	13	78171660	78171660	Splice_Site	INDEL	-	T	T	SCEL	c.687-13dupT	
XH_P31	14	21541219	21541219	Missense_Mutation	SNV	G	A	A	ARHGEF40	c.19G>A	P.E7K
XH_P31	14	24885799	24885799	Missense_Mutation	SNV	C	T	T	NYNRIN	c.4844C>T	P.S1615L
XH_P31	14	51237115	51237115	Silent	SNV	G	C	C	NIN	c.1425C>G	P.L475L
XH_P31	14	64989660	64989660	Missense_Mutation	SNV	G	A	A	ZBTB1	c.1438G>A	P.G480R
XH_P31	15	78911014	78911014	Splice_Site	SNV	G	A	A	CHRNA3	c.223-11C>T	
XH_P31	16	58031004	58031004	Missense_Mutation	SNV	T	A	A	ZNF319	c.1166A>T	P.H389L
XH_P31	17	11593565	11593565	Missense_Mutation	SNV	C	G	G	DNAH9	c.4426C>G	P.Q1476E
XH_P31	17	2268303	2268303	Silent	SNV	G	A	A	SGSM2	c.1113G>A	P.L371L
XH_P31	17	3417883	3417883	Splice_Site	SNV	G	C	C	TRPV3	c.2278+4C>G	
XH_P31	17	36243573	36243573	Splice_Site	SNV	G	A	A	YWHAEP7	n.108+8C>T	
XH_P31	17	8020082	8020082	Splice_Site	SNV	T	C	C	ALOXE3	c.748+12A>G	
XH_P31	19	19207020	19207020	Splice_Site	SNV	T	C	C	SLC25A42	c.81+6T>C	
XH_P31	19	46196073	46196073	Silent	SNV	C	T	T	QPCTL	c.112C>T	P.L38L
XH_P31	19	50498470	50498470	Missense_Mutation	SNV	C	T	T	VRK3	c.592G>A	P.G198S
XH_P31	19	50763926	50763926	Missense_Mutation	SNV	C	G	G	MYH14	c.2279C>G	P.A760G
XH_P31	19	51633249	51633249	Silent	SNV	C	A	A	SIGLEC9	c.1305C>A	P.S435S
XH_P31	19	56244764	56244764	Nonsense_Mutation	SNV	G	A	A	NLRP9	c.433C>T	P.R145*
XH_P31	19	58083602	58083602	Missense_Mutation	SNV	T	C	C	ZNF416	c.1670A>G	P.K557R
XH_P31	19	58565348	58565348	Missense_Mutation	SNV	G	A	A	ZSCAN1	c.1156G>A	P.G386R
XH_P31	2	175624357	175624357	Silent	SNV	G	A	A	CHRNA1	c.48C>T	P.G16G
XH_P31	2	24949517	24949517	Missense_Mutation	SNV	G	A	A	NCOA1	c.2659G>A	P.D887N
XH_P31	2	74317082	74317082	Missense_Mutation	SNV	G	A	A	TET3	c.2947G>A	P.G983S
XH_P31	21	47704090	47704090	Missense_Mutation	SNV	C	T	T	MCM3AP	c.1111G>A	P.E371K
XH_P31	21	47918732	47918732	Missense_Mutation	SNV	C	T	T	DIP2A	c.641C>T	P.A214V
XH_P31	22	29694815	29694815	Missense_Mutation	SNV	G	T	T	EWSR1	c.1510G>T	P.G504C
XH_P31	3	124174038	124174038	Silent	SNV	G	A	A	KALRN	c.3561G>A	P.K1187K

XH_P31	3	128205017	128205017	Missense_Mutation	SNV	G	A	A	GATA2	c.424C>T	P.P142S
XH_P31	3	52439876	52439876	Nonsense_Mutation	SNV	G	C	C	BAP1	c.836C>G	P.S279*
XH_P31	3	56667888	56667888	Silent	SNV	T	C	C	FAM208A	c.2931A>G	P.P977P
XH_P31	3	98110193	98110193	Silent	SNV	C	A	A	OR5K3	c.684C>A	P.S228S
XH_P31	3	9945092	9945092	Missense_Mutation	SNV	C	G	G	IL17RE	c.144C>G	P.F48L
XH_P31	4	72363373	72363373	Silent	SNV	G	A	A	SLC4A4	c.2130G>A	P.L710L
XH_P31	6	107390590	107390590	Missense_Mutation	SNV	C	A	A	BEND3	c.1805G>T	P.G602V
XH_P31	6	13486970	13486970	Silent	SNV	G	A	A	GFOD1	c.153C>T	P.Y51Y
XH_P31	6	146480500	146480500	Silent	SNV	C	T	T	GRM1	c.717C>T	P.S239S
XH_P31	6	78173104	78173104	Missense_Mutation	SNV	G	A	A	HTR1B	c.17C>T	P.A6V
XH_P31	7	48407455	48407455	Missense_Mutation	SNV	G	A	A	ABCA13	c.10753G>A	P.A3585T
XH_P31	9	136029080	136029080	Missense_Mutation	SNV	G	A	A	GBGT1	c.877C>T	P.P293S
XH_P31	9	33973173	33973173	Splice_Site	SNV	T	C	C	UBAP2	c.575+8A>G	
XH_P31	9	87317138	87317138	Silent	SNV	C	T	T	NTRK2	c.277C>T	P.L93L
XH_P31	X	103349764	103349764	Silent	SNV	G	C	C	SLC25A53	c.177C>G	P.A59A
XH_P31	X	134680316	134680316	Frame_Shift_Ins	INDEL	-	T	T	INTS6L	c.359dupT	P.L120fs
XH_P31	X	50653946	50653946	Missense_Mutation	SNV	G	A	A	BMP15	c.163G>A	P.E55K
XH_P32	1	1163875	1163885	Frame_Shift_Del	DEL	GGCTCCGCC	-	CGGCTCCGCCG	SDF4	c.289_299del	p.R97fs
XH_P32	1	59248400	59248411	In_Frame_Del	DEL	GAAGCCCTC(	-	CGAAGCCCTCGG	JUN	c.332_343del	p.111_115del
XH_P32	1	169696611	169696611	Silent	SNP	C	T	C	SELE	c.G1524A	p.G508G
XH_P32	1	228473981	228473981	Silent	SNP	G	T	G	OBSCN	c.G9207T	p.A3069A
XH_P32	2	96691726	96691726	Missense_Mutation	SNP	C	T	C	GPAT2	c.G977A	p.G326D
XH_P32	2	187511467	187511468	Frame_Shift_Del	DEL	TC	-	TC	ITGAV	c.1106_1107del	p.I369fs
XH_P32	2	202356237	202356237	Silent	SNP	C	T	C	C2CD6	c.G4827A	p.K1609K
XH_P32	3	42251577	42251577	Missense_Mutation	SNP	C	A	C	TRAK1	c.C1841A	p.T614K
XH_P32	3	52429416	52429416	Silent	SNP	C	G	C	DNAH1	c.C11061G	p.L3687L
XH_P32	3	98001922	98001922	Missense_Mutation	SNP	T	A	T	OR5H2	c.T191A	p.M64K
XH_P32	3	98073305	98073305	Nonsense_Mutation	SNP	C	A	C	OR5K4	c.C608A	p.S203X
XH_P32	3	126915884	126915884	Missense_Mutation	SNP	A	T	A	C3orf56	c.A356T	p.Y119F
XH_P32	3	150421527	150421527	In_Frame_Ins	INS	-	CTCCTCCTCCACC	-	ERICH6	sAGAGGTGGAG	E53delinsEEVEEEE
XH_P32	3	178951957	178951957	Missense_Mutation	SNP	G	A	G	PIK3CA	c.G3012A	p.M1004I
XH_P32	3	195507242	195507242	In_Frame_Ins	INS	-	AGATACTGAGGA	-	MUC4	CAGCACTTCCTC	IATPLPVTSTSSVSTGHVTPLHVTSPSSA
XH_P32	4	4239589	4239589	Missense_Mutation	SNP	C	T	C	TMEM128	c.G472A	p.V158I
XH_P32	4	25005736	25005736	Silent	SNP	C	T	C	LGI2	c.G975A	p.K325K
XH_P32	5	23527389	23527472	In_Frame_Del	DEL	GGGGAGAAC	-	CAGGGGAGAAGC	PRDM9	c.2192_2275del	p.731_759del
XH_P32	5	54529084	54529084	Missense_Mutation	SNP	C	G	C	CCNO	c.G268C	p.V90L
XH_P32	5	95730588	95730588	Missense_Mutation	SNP	T	C	T	PCSK1	c.A1864G	p.K622E
XH_P32	5	139781730	139781730	Missense_Mutation	SNP	G	A	G	ANKHD1	c.G178A	p.G60S
XH_P32	5	150512737	150512737	Missense_Mutation	SNP	C	G	C	ANXA6	c.G462C	p.E154D
XH_P32	5	170148829	170148829	Silent	SNP	C	T	C	KCNIP1	c.C324T	p.C108C
XH_P32	6	160525994	160525994	Missense_Mutation	SNP	G	A	G	IGF2R	c.G7354A	p.G2452R
XH_P32	6	170627695	170627695	In_Frame_Ins	INS	-	GCAAGAAGTTCC	-	FAM120B	ACCCAGGCAAGA	delinsDPEPRQEVPMCTG
XH_P32	7	38256827	38256827	Silent	SNP	C	A	C	STARD3NL	c.C474A	p.I158I
XH_P32	7	57522802	57522802	Missense_Mutation	SNP	T	G	T	ZNF716	c.T190G	p.L64V
XH_P32	7	91709094	91709094	Missense_Mutation	SNP	A	C	A	AKAP9	c.A7647C	p.E2549D
XH_P32	7	123302689	123302689	Missense_Mutation	SNP	G	T	G	LMOD2	c.G1049T	p.R350I
XH_P32	7	150783902	150783903	Frame_Shift_Del	DEL	CT	-	CT	AGAP3	c.74_75del	p.A25fs
XH_P32	8	101721812	101721812	Missense_Mutation	SNP	G	C	G	PABPC1	c.C1120G	p.R374G
XH_P32	9	33030613	33030613	Silent	SNP	C	T	C	DNAJA1	c.C120T	p.N40N
XH_P32	9	104499983	104499983	Silent	SNP	C	T	C	GRIN3A	c.G279A	p.P93P
XH_P32	9	131588839	131588839	Silent	SNP	C	T	C	SPOUT1	c.G438A	p.L146L
XH_P32	10	48390752	48390752	Silent	SNP	C	T	C	RBP3	c.G126A	p.P42P
XH_P32	10	95185893	95185893	Missense_Mutation	SNP	C	A	C	MYOF	c.G368T	p.G123V
XH_P32	11	1018419	1018419	Missense_Mutation	SNP	G	A	G	MUC6	c.C4382T	p.T1461I
XH_P32	11	6411931	6411948	In_Frame_Del	DEL	TGCTGGCGCT	-	GTGCTGGCGCTG	SMPD1	c.103_120del	p.35_40del
XH_P32	11	30926660	30926660	Missense_Mutation	SNP	G	A	G	DCDC1	c.C1477T	p.R493C
XH_P32	11	30938455	30938455	Silent	SNP	T	C	T	DCDC1	c.A735G	p.K245K
XH_P32	11	55658976	55658976	Silent	SNP	A	G	A	TRIM51	c.A1227G	p.V409V
XH_P32	11	62300443	62300443	Missense_Mutation	SNP	C	A	C	AHNAK	c.G1446T	p.K482N
XH_P32	11	67132692	67132692	Missense_Mutation	SNP	G	A	G	CLCF1	c.C563T	p.S188L
XH_P32	11	67200271	67200271	Silent	SNP	G	A	G	RPS6KB2	c.G579A	p.R193R
XH_P32	11	118890121	118890121	Missense_Mutation	SNP	G	A	G	TRAPPC4	c.G22A	p.D8N
XH_P32	12	52284540	52284551	In_Frame_Del	DEL	GCCCAGGCC	-	GGCCCAGGCCCA	ANKRD33	c.228_239del	p.76_80del
XH_P32	12	67692735	67692735	Missense_Mutation	SNP	C	T	C	CAND1	c.C860T	p.P287L
XH_P32	13	20025324	20025324	Silent	SNP	A	G	A	TPTE2	c.T552C	p.Y184Y
XH_P32	14	96157138	96157138	Silent	SNP	C	T	C	TCL1B	c.C228T	p.S76S
XH_P32	14	103987608	103987608	Silent	SNP	G	A	G	CKB	c.C645T	p.R215R
XH_P32	14	105211231	105211231	Missense_Mutation	SNP	A	C	A	ADSSL1	c.A541C	p.I181L
XH_P32	15	75650833	75650833	Silent	SNP	A	C	A	MAN2C1	c.T2076G	p.V692V
XH_P32	16	24574971	24574971	Silent	SNP	A	T	A	RBBP6	c.A1506T	p.R502R
XH_P32	16	57546777	57546777	Missense_Mutation	SNP	C	T	C	CCDC102A	c.G1529A	p.R510H
XH_P32	16	70292007	70292007	Silent	SNP	C	T	C	AARS	c.G2106A	p.P702P
XH_P32	17	6929775	6929775	Splice_Site	SNP	G	A	G	BCL6B	.	.
XH_P32	17	18927651	18927651	Silent	SNP	C	A	C	GRAP	c.G345T	p.S115S
XH_P32	17	42982204	42982204	Missense_Mutation	SNP	A	G	A	FAM187A	c.A1007G	p.Q336R
XH_P32	18	33795610	33795610	Silent	SNP	C	A	C	MOCOS	c.C1467A	p.R489R
XH_P32	18	67673641	67673641	Missense_Mutation	SNP	C	G	C	RTTN	c.G3765C	p.W1255C
XH_P32	19	4682879	4682882	Frame_Shift_Del	DEL	AGAG	-	AGAG	DPP9-AS1	c.9_12del	p.A3fs
XH_P32	19	10468453	10468453	Missense_Mutation	SNP	C	T	C	TYK2	c.G2453A	p.R818H
XH_P32	19	30500119	30500124	In_Frame_Del	DEL	TGATGA	-	TGATGA	URI1	c.840_845del	p.280_282del
XH_P32	19	32844003	32844003	Silent	SNP	G	A	G	ZNF507	c.G267A	p.P89P
XH_P32	19	40902229	40902229	Missense_Mutation	SNP	T	C	T	PRX	c.A2030G	p.E677G
XH_P32	19	46299167	46299167	In_Frame_Ins	INS	-	CTCCTCCTCCTCG	-	RSPH6A	14insGCGAGGAC	E705delinsGEEEEE
XH_P32	19	52888079	52888079	In_Frame_Ins	INS	-	GTGAAA	-	ZNF880	46_1247insGTG	).A416delinsGET
XH_P32	19	53911570	53911570	Silent	SNP	G	A	G	ZNF765	c.G603A	p.S201S
XH_P32	19	54744357	54744357	Frame_Shift_Ins	INS	-	CTGCCACC	-	LILRA6	0_1051insGGTG	p.F351fs
XH_P32	19	58864385	58864385	Silent	SNP	C	A	C	A1BG	c.G249T	p.L83L
XH_P32	20	1294128	1294128	Silent	SNP	G	T	G	SDCBP2	c.C240A	p.G80G
XH_P32	20	1294129	1294129	Missense_Mutation	SNP	C	T	C	SDCBP2	c.G239A	p.G80D
XH_P32	20	3767122	3767184	Frame_Shift_Del	DEL	CCGGGGCCC	-	CCCCGGGGCCCGG	CENPB	NM_001810	wholegene
XH_P32	20	61441003	61441003	Missense_Mutation	SNP	C	T	C	OGFR	c.C386T	p.S129F
XH_P32	21	33641384	33641384	Silent	SNP	G	A	G	MIS18A	c.C666T	p.A222A
XH_P32	22	18300128	18300128	Missense_Mutation	SNP	C	T	C	MICAL3	c.G5299A	p.G1767R
XH_P32	X	40506603	40506603	Missense_Mutation	SNP	C	T	C	CXorf38	c.G170A	p.R57H
XH_P32	X	106069422	106069422	Silent	SNP	T	A	T	TBC1D8B	c.T990A	p.A330A
XH_P32	X	142718309	142718309	Missense_Mutation	SNP	C	T	C	SLITRK4	c.G616A	p.V206I
XH_P32	1	228473981	228473981	Silent	SNV	G	T	T	OBSCN	c.9207G>T	P.A3069A
XH_P32	10	48390752	48390752	Silent	SNV	C	T	T	RBP3	c.126G>A	P.P42P

XH_P32	10	95185893	95185893	Missense_Mutation	SNV	C	A	A	MYOF	c.368G>T	P.G123V
XH_P32	11	118890121	118890121	Missense_Mutation	SNV	G	A	A	TRAPPC4	c.22G>A	P.D8N
XH_P32	11	30926660	30926660	Missense_Mutation	SNV	G	A	A	DCDC5	c.1477C>T	P.R493C
XH_P32	12	67692735	67692735	Missense_Mutation	SNV	C	T	T	CAND1	c.788C>T	P.P263L
XH_P32	14	103987608	103987608	Silent	SNV	G	A	A	CKB	c.645C>T	P.R215R
XH_P32	14	105211231	105211231	Missense_Mutation	SNV	A	C	C	ADSSL1	c.541A>C	P.I181L
XH_P32	15	75650833	75650833	Silent	SNV	A	C	C	MAN2C1	c.2424T>G	P.V808V
XH_P32	16	24574971	24574971	Silent	SNV	A	T	T	RBBP6	c.1506A>T	P.R502R
XH_P32	17	18927651	18927651	Silent	SNV	C	A	A	GRAP	c.345G>T	P.S115S
XH_P32	17	6929775	6929775	Splice_Site	SNV	G	A	A	BCL6B	c.890-1G>A	
XH_P32	19	58864385	58864385	Silent	SNV	C	A	A	A1BG	c.249G>T	P.L83L
XH_P32	2	187511467	187511468	Frame_Shift_Del	INDEL	TC	-	-	ITGAV	.1077_1078delC	P.F360fs
XH_P32	2	202356237	202356237	Silent	SNV	C	T	T	ALS2CR11	c.4827G>A	P.K1609K
XH_P32	20	1294129	1294129	Missense_Mutation	SNV	C	T	T	SDCBP2	c.239G>A	P.G80D
XH_P32	20	61441003	61441003	Missense_Mutation	SNV	C	T	T	OGFR	c.386C>T	P.S129F
XH_P32	3	178951957	178951957	Missense_Mutation	SNV	G	A	A	PIK3CA	c.3012G>A	P.M1004I
XH_P32	3	52429416	52429416	Silent	SNV	C	G	G	DNAH1	c.11061C>G	P.L3687L
XH_P32	3	98073305	98073305	Nonsense_Mutation	SNV	C	A	A	OR5K4	c.608C>A	P.S203*
XH_P32	4	4239589	4239589	Missense_Mutation	SNV	C	T	T	TMEM128	c.472G>A	P.V158I
XH_P32	5	150512737	150512737	Missense_Mutation	SNV	C	G	G	ANXA6	c.558G>C	P.E186D
XH_P32	5	23527389	23527472	Missense_Mutation	INDEL	GGGGAGAAC	-	-	PRDM9	GGACACACACAC	P.R758_F785del
XH_P32	5	54529084	54529084	Missense_Mutation	SNV	C	G	G	CCNO	c.268G>C	P.V90L
XH_P32	5	95730588	95730588	Missense_Mutation	SNV	T	C	C	PCSK1	c.1864A>G	P.K622E
XH_P32	7	38256827	38256827	Silent	SNV	C	A	A	STARD3NL	c.474C>A	P.I158I
XH_P32	7	57522802	57522802	Missense_Mutation	SNV	T	G	G	ZNF716	c.190T>G	P.L64V
XH_P32	7	91709094	91709094	Missense_Mutation	SNV	A	C	C	AKAP9	c.7647A>C	P.E2549D
XH_P32	9	104499983	104499983	Silent	SNV	C	T	T	GRIN3A	c.279G>A	P.P93P
XH_P32	9	131588839	131588839	Silent	SNV	C	T	T	SPOUT1	c.438G>A	P.L146L
XH_P32	9	33030613	33030613	Silent	SNV	C	T	T	DNAJA1	c.120C>T	P.N40N
XH_P32	X	106069422	106069422	Silent	SNV	T	A	A	TBC1D8B	c.990T>A	P.A330A
XH_P32	X	142718309	142718309	Missense_Mutation	SNV	C	T	T	SLITRK4	c.616G>A	P.V206I
XH_P32	X	40506603	40506603	Missense_Mutation	SNV	C	T	T	CXorf38	c.170G>A	P.R57H
XH_P33	14	45653104	45653104	Missense_Mutation	SNP	A	T	T	FANCM	c.4514A>T	p.K1505M
XH_P33	1	3649562	3649562	Silent	SNP	G	A	G	TP73	c.G1440A	p.A480A
XH_P33	1	16345867	16345867	Missense_Mutation	SNP	G	A	G	HSPB7	c.C98T	p.S33L
XH_P33	1	18808711	18808711	Silent	SNP	G	C	G	KLHDC7A	c.G1236C	p.G412G
XH_P33	1	19181393	19181393	Missense_Mutation	SNP	T	C	T	TAS1R2	c.A571G	p.I191V
XH_P33	1	19992513	19992513	Silent	SNP	C	T	C	HTR6	c.C267T	p.Y89Y
XH_P33	1	20009737	20009737	Silent	SNP	G	A	G	TMCO4	c.C1701T	p.S567S
XH_P33	1	20141528	20141528	Missense_Mutation	SNP	C	T	C	RNF186	c.G67A	p.A23T
XH_P33	1	26487940	26487940	Missense_Mutation	SNP	A	G	A	FAM110D	c.A158G	p.H53R
XH_P33	1	26609259	26609259	Silent	SNP	C	T	C	UBXN11	c.G813A	p.T271T
XH_P33	1	27107135	27107135	Frame_Shift_Ins	INS	-	A	-	ARID1A	c.6747dupA	p.S2249fs
XH_P33	1	33292533	33292533	Splice_Site	INS	-	TTGCTTTC	-	S100PBP	.	.
XH_P33	1	33646950	33646950	Silent	SNP	A	G	A	TRIM62	c.T84C	p.H28H
XH_P33	1	36807481	36807481	Missense_Mutation	SNP	C	T	C	STK40	c.G1198A	p.A400T
XH_P33	1	38023324	38023324	Frame_Shift_Ins	INS	-	T	-	DNALI1	c.269dupT	p.I90fs
XH_P33	1	38023326	38023326	Missense_Mutation	SNP	C	G	C	DNALI1	c.C270G	p.I90M
XH_P33	1	38155914	38155914	Missense_Mutation	SNP	T	C	T	C1orf109	c.A157G	p.K53E
XH_P33	1	43804285	43804285	Frame_Shift_Ins	INS	-	TCTTCTCA	-	MPL	35_286insTCTTC	p.F95fs
XH_P33	1	45476663	45476663	Silent	SNP	G	A	G	HECTD3	c.C267T	p.L89L
XH_P33	1	53600078	53600078	Silent	SNP	A	G	A	SLC1A7	c.T159C	p.P53P
XH_P33	1	66723505	66723505	Missense_Mutation	SNP	C	A	C	PDE4B	c.C498A	p.N166K
XH_P33	1	66723507	66723507	Frame_Shift_Ins	INS	-	AGAAC	-	PDE4B	500_501insAGA	p.N167fs
XH_P33	1	70694110	70694110	Missense_Mutation	SNP	C	T	C	SRSF11	c.C209T	p.S70L
XH_P33	1	75055571	75055571	Missense_Mutation	SNP	T	A	T	ERICH3	c.A1920T	p.L640F
XH_P33	1	78383958	78383958	In_Frame_Ins	INS	-	TTCCTAAAA	-	NEXN	5_256insTTCCTA	.Q85delinsQFLK
XH_P33	1	78383959	78383959	Splice_Site	SNP	G	A	G	NEXN	.	.
XH_P33	1	91182240	91182240	Silent	SNP	C	A	C	BARHL2	c.G513T	p.P171P
XH_P33	1	94342443	94342443	In_Frame_Ins	INS	-	AGTTATAAG	-	DNTTIP2	7_1048insCTTAT	.V350delinsLITV
XH_P33	1	110768764	110768764	Frame_Shift_Ins	INS	-	CTCCCCACCTGT	-	KCNC4	GGGGGCTCCCC	p.G595fs
XH_P33	1	152128110	152128110	In_Frame_Ins	INS	-	AACTCTGGCCTTC	-	RPTN	GACAGACAAGGC	delinsQTDRQGQSSHYGK
XH_P33	1	152129066	152129101	In_Frame_Del	DEL	TGTCTTGTTT	-	TCTGTCTTGTTTC	RPTN	c.474_509del	p.158_170del
XH_P33	1	152187389	152187427	In_Frame_Del	DEL	GGAATGTCCT	-	AGGAATGTCCTG	HRNR	c.6678_6716del	p.2226_2239del
XH_P33	1	152282032	152282032	Missense_Mutation	SNP	G	C	G	FLG	c.C5330G	p.S1777C
XH_P33	1	152732302	152732340	In_Frame_Del	DEL	GGCTCAATGT	-	AGGCTCAATGTC	KPRP	c.238_276del	p.80_92del
XH_P33	1	153317801	153317801	Missense_Mutation	SNP	A	G	A	PGLYRP4	c.T197C	p.V66A
XH_P33	1	159002411	159002411	Frame_Shift_Ins	INS	-	AT	-	IFI16	.1091_1092insA	p.T364fs
XH_P33	1	159002413	159002413	Frame_Shift_Ins	INS	-	TTTCA	-	IFI16	093_1094insTTT	p.F365fs
XH_P33	1	160302249	160302249	Missense_Mutation	SNP	C	A	C	COPA	c.G485T	p.W162L
XH_P33	1	161483723	161483723	Splice_Site	SNP	G	A	G	FCGR2A	.	.
XH_P33	1	161721745	161721745	Missense_Mutation	SNP	G	A	G	DUSP12	c.G548A	p.R183H
XH_P33	1	176709186	176709186	Silent	SNP	C	T	C	PAPPA2	c.C4005T	p.F1335F
XH_P33	1	176709187	176709187	Missense_Mutation	SNP	C	T	C	PAPPA2	c.C4006T	p.H1336Y
XH_P33	1	185109277	185109277	Frame_Shift_Ins	INS	-	TTTCTAA	-	TRMT1L	68_469insTTAGA	p.V157fs
XH_P33	1	186273365	186273365	Frame_Shift_Ins	INS	-	ACATCTTGTTTAT	-	PRG4	23insACATCTTG	p.I108fs
XH_P33	1	186280635	186280635	Nonsense_Mutation	SNP	A	T	A	PRG4	c.A3298T	p.K1100X
XH_P33	1	186280637	186280638	Frame_Shift_Del	DEL	AG	-	AG	PRG4	c.3300_3301del	p.K1100fs
XH_P33	1	200539091	200539091	Missense_Mutation	SNP	T	A	T	KIF14	c.A2136T	p.L712F
XH_P33	1	202418191	202418191	Nonsense_Mutation	INS	-	ATT	-	PPP1R12B	1742_1743insAT	p.C581delinsX
XH_P33	1	205290794	205290794	Missense_Mutation	SNP	G	A	G	NUAK2	c.C95T	p.T32I
XH_P33	1	207890918	207890918	Frame_Shift_Ins	INS	-	TTCCTGTGTGTC	-	CR1L	GGGAGTTCCTG	p.P508fs
XH_P33	1	222876553	222876554	Frame_Shift_Del	DEL	TG	-	TG	AIDA	c.116_117del	p.A39fs
XH_P33	1	228509886	228509886	Missense_Mutation	SNP	C	G	C	OBSCN	c.C15344G	p.P5115R
XH_P33	1	228509888	228509888	Nonsense_Mutation	INS	-	GAGAGGAAGCGC	-	OBSCN	AGCCTTAGAGAG	p.L5116delinsX
XH_P33	1	228564884	228564884	Missense_Mutation	SNP	G	A	G	OBSCN	c.G23171A	p.R7724H
XH_P33	1	228588680	228588680	Silent	SNP	A	C	A	TRIM11	c.T720G	p.A240A
XH_P33	1	236557771	236557771	Missense_Mutation	SNP	G	A	G	EDARADD	c.G27A	p.M9I
XH_P33	1	243329398	243329398	In_Frame_Ins	INS	-	GACCCTTGAGTA	-	CEP170	TCATTTTACTCA	sLHLLPCSFYSRVCQHLVYLSG
XH_P33	2	20205848	20205848	Silent	SNP	G	A	G	MATN3	c.C447T	p.A149A
XH_P33	2	26689660	26689660	Silent	SNP	G	A	G	OTOF	c.C2352T	p.Y784Y
XH_P33	2	27165492	27165492	Silent	SNP	C	A	C	DPYSL5	c.C1314A	p.V438V
XH_P33	2	29150453	29150453	Missense_Mutation	SNP	A	G	A	WDR43	c.A1192G	p.N398D
XH_P33	2	32605282	32605282	Frame_Shift_Ins	INS	-	CAGGTAC	-	BIRC6	69_570insCAGGT	p.D190fs
XH_P33	2	33568029	33568029	Frame_Shift_Ins	INS	-	AT	-	LTBP1	.2877_2878insA	p.V959fs
XH_P33	2	33568031	33568031	Splice_Site	SNP	G	A	G	LTBP1	.	.
XH_P33	2	36691676	36691676	Splice_Site	SNP	G	A	G	CRIM1	.	.
XH_P33	2	36691678	36691678	Nonsense_Mutation	INS	-	AACTAAG	-	CRIM1	71_872insAACTA	C291_E292delinsX

XH_P33	2	55436809	55436809	Nonsense_Mutation	INS	-	TTTTTTATCT	-	CLHC1	_292insAGATAA	R98_R99delinsRX
XH_P33	2	64322025	64322025	Silent	SNP	G	A	G	PELI1	c.C1068T	p.D356D
XH_P33	2	65217089	65217089	Silent	SNP	G	C	G	SLC1A4	c.G312C	p.S104S
XH_P33	2	86705748	86705748	Nonsense_Mutation	INS	-	GAATTATCTA	-	KDM3A	_2207insGAATT	736_I737delinsGIIX
XH_P33	2	88125215	88125215	Missense_Mutation	SNP	G	C	G	RGPD2	c.C34G	p.L12V
XH_P33	2	97166296	97166296	Missense_Mutation	SNP	A	G	A	NEURL3	c.T394C	p.C132R
XH_P33	2	97818241	97818241	Missense_Mutation	SNP	T	G	T	ANKRD36	c.T1163G	p.V388G
XH_P33	2	98732147	98732147	Missense_Mutation	SNP	T	C	T	VWA3B	c.T229C	p.S77P
XH_P33	2	99978030	99978030	Frame_Shift_Ins	INS	-	CACTTCTTATTTG	-	EIF5B	67insCACTTCTT	p.S222fs
XH_P33	2	113145814	113145814	Missense_Mutation	SNP	C	T	C	RGPD5	c.G4708A	p.G1570R
XH_P33	2	115822383	115822383	Splice_Site	SNP	G	A	G	DPP10	.	.
XH_P33	2	121736132	121736132	Frame_Shift_Ins	INS	-	CTATT	-	GLI2	491_1492insCT/	p.H497fs
XH_P33	2	131802045	131802045	Silent	SNP	C	T	C	ARHGEF4	c.C1773T	p.P591P
XH_P33	2	136396509	136396553	In_Frame_Del	DEL	GTTGAAGTAC	-	AGTTGAAGTACT(	R3HDM1	c.868_912del	p.290_304del
XH_P33	2	152487273	152487273	Nonsense_Mutation	INS	-	TTAAATATTACA	-	NEB	914insATGTAAT	972_E2973delinsMX
XH_P33	2	152487275	152487275	Missense_Mutation	SNP	G	T	G	NEB	c.C8912A	p.T2971K
XH_P33	2	153575161	153575161	Nonsense_Mutation	SNP	G	A	G	ARL6IP6	c.G23A	p.W8X
XH_P33	2	163039951	163039951	In_Frame_Ins	INS	-	ATCTTT	-	FAP	66_1767insAAA	p.D589delinsEKI
XH_P33	2	165578785	165578785	Frame_Shift_Ins	INS	-	TA	-	COBLL1	c.795_796insTA	p.H266fs
XH_P33	2	165578786	165578786	Frame_Shift_Ins	INS	-	CA	-	COBLL1	c.794_795insTG	p.K265fs
XH_P33	2	170493837	170493837	Frame_Shift_Ins	INS	-	CTCCT	-	PPIG	069_2070insCT(	p.S690fs
XH_P33	2	173338810	173338810	Frame_Shift_Ins	INS	-	CACA	-	ITGA6	.803_804insCAC	p.I268fs
XH_P33	2	175614857	175614857	Missense_Mutation	SNP	C	A	C	CHRNA1	c.G819T	p.L273F
XH_P33	2	179638614	179638614	Missense_Mutation	SNP	G	T	G	TTN	c.C7143A	p.F2381L
XH_P33	2	216002903	216002903	Frame_Shift_Ins	INS	-	AGTAAGATAT	-	ABCA12	3_29insATATCTT	p.I10fs
XH_P33	2	223554167	223554167	Nonsense_Mutation	SNP	C	T	C	MOGAT1	c.C457T	p.R153X
XH_P33	2	227915832	227915832	Missense_Mutation	SNP	G	A	G	COL4A4	c.C3011T	p.P1004L
XH_P33	2	231624800	231624877	Frame_Shift_Del	DEL	ATGGATTTG(	-	TATGGATTTGG(	CAB39	c.84_114del	p.K28fs
XH_P33	2	233498669	233498669	Silent	SNP	G	T	G	EFHD1	c.G255T	p.P85P
XH_P33	2	239229372	239229372	Silent	SNP	C	A	C	TRAF3IP1	c.C69A	p.T23T
XH_P33	2	241404499	241404499	Silent	SNP	C	T	C	GPC1	c.C1140T	p.S380S
XH_P33	2	241621869	241621869	Missense_Mutation	SNP	G	A	G	AQP12B	c.C386T	p.T129M
XH_P33	2	241827865	241827865	Silent	SNP	G	A	G	C2orf54	c.C1095T	p.F365F
XH_P33	2	242312576	242312576	Missense_Mutation	SNP	G	T	G	FARP2	c.G54T	p.L18F
XH_P33	2	242312577	242312577	Missense_Mutation	SNP	G	T	G	FARP2	c.G55T	p.G19C
XH_P33	2	242648677	242648677	Silent	SNP	G	A	G	ING5	c.G156A	p.T52T
XH_P33	2	242758179	242758179	Silent	SNP	C	T	C	NEU4	c.C1299T	p.S433S
XH_P33	3	39118670	39118670	Nonsense_Mutation	SNP	G	A	G	WDR48	c.G692A	p.W231X
XH_P33	3	52540773	52540773	Silent	SNP	T	C	T	STAB1	c.T1896C	p.G632G
XH_P33	3	70014263	70014263	Frame_Shift_Ins	INS	-	GAGC	-	MITF	124_1125insGA	p.E375fs
XH_P33	3	89445074	89445074	Frame_Shift_Ins	INS	-	A	-	EPHA3	:.1394_1395ins/	p.G465fs
XH_P33	3	89445075	89445075	In_Frame_Ins	INS	-	TCT	-	EPHA3	1395_1396insT(	p.G465delinsGS
XH_P33	3	128532244	128532244	Silent	SNP	G	A	G	RAB7A	c.G603A	p.S201S
XH_P33	3	130383927	130383927	Nonsense_Mutation	SNP	C	T	C	COL6A6	c.C6583T	p.R2195X
XH_P33	3	133302808	133302888	Frame_Shift_Del	DEL	GCAGTGAAA	-	AGCAGTGAAAA(	CDV3	c.318_360del	p.S106fs
XH_P33	3	136323310	136323310	Frame_Shift_Ins	INS	-	CACGTATAAA	-	STAG1	_138insTTTATA	p.T46fs
XH_P33	3	138724858	138724858	Missense_Mutation	SNP	T	C	T	PRR23A	c.A253G	p.T85A
XH_P33	3	142681657	142681686	In_Frame_Del	DEL	GCCACCGTG(	-	GGCCACCGTGCT	PAQR9	c.493_522del	p.165_174del
XH_P33	3	151451961	151451961	Silent	SNP	G	A	G	AADACL2	c.G138A	p.T46T
XH_P33	3	152018125	152018125	Missense_Mutation	SNP	G	T	G	MBNL1	c.G143T	p.G48V
XH_P33	3	152018126	152018126	Silent	SNP	A	C	A	MBNL1	c.A144C	p.G48G
XH_P33	3	154033019	154033019	Frame_Shift_Ins	INS	-	AAAAT	-	DHX36	418_419insATT	p.L140fs
XH_P33	3	154033020	154033020	Missense_Mutation	SNP	G	A	G	DHX36	c.C418T	p.L140F
XH_P33	3	186358297	186358297	Silent	SNP	C	T	C	FETUB	c.C48T	p.C16C
XH_P33	3	195506023	195506023	Missense_Mutation	SNP	G	A	G	MUC4	c.C12428T	p.T4143M
XH_P33	3	195517242	195517242	Frame_Shift_Ins	INS	-	AATATTTACT	-	MUC4	_1209insAGTAA	p.T403fs
XH_P33	3	195517243	195517243	Missense_Mutation	SNP	G	C	G	MUC4	c.C1208G	p.T403S
XH_P33	4	1018271	1018271	Silent	SNP	T	C	T	FGFRL1	c.T891C	p.D297D
XH_P33	4	1729765	1729800	In_Frame_Del	DEL	CGCACGGA(	-	TCCGCACGGAG(	TACC3	c.636_671del	p.212_224del
XH_P33	4	3446091	3446091	Missense_Mutation	SNP	G	T	G	HGFAC	c.G652T	p.A218S
XH_P33	4	3446682	3446682	Silent	SNP	C	T	C	HGFAC	c.C978T	p.A326A
XH_P33	4	9270563	9270563	Frame_Shift_Ins	INS	-	A	-	USP17L20	c.1220dupA	p.E407fs
XH_P33	4	9270565	9270565	Missense_Mutation	SNP	G	T	G	USP17L20	c.G1221T	p.E407D
XH_P33	4	24578236	24578236	Frame_Shift_Ins	INS	-	GATACTATGA	-	DHX15	_137insTCATAG	p.R46fs
XH_P33	4	37962354	37962354	Nonsense_Mutation	INS	-	TTGAGAT	-	PTTG2	99_300insTTGA(	100_K101delinsTX
XH_P33	4	39329290	39329290	Nonsense_Mutation	INS	-	TTAATAATTAAA/	-	RFC1	418insTTTTAATT	140_M141delinsFX
XH_P33	4	39329292	39329292	Missense_Mutation	SNP	G	A	G	RFC1	c.C416T	p.S139L
XH_P33	4	40434705	40434725	In_Frame_Del	DEL	GCGGCGGCT	-	CTGCGGCGGCTG	RBM47	c.1278_1298del	p.426_433del
XH_P33	4	55968137	55968137	Silent	SNP	G	A	G	KDR	c.C2193T	p.D731D
XH_P33	4	56744168	56744168	Missense_Mutation	SNP	G	A	G	EXOC1	c.G1160A	p.R387Q
XH_P33	4	73968236	73968236	Nonsense_Mutation	INS	-	TCCCATTTGTTTT	-	ANKRD17	677insAAAACAA	1226_A1227delinsX
XH_P33	4	74270085	74270085	Missense_Mutation	SNP	G	T	G	ALB	c.G41T	p.S14I
XH_P33	4	76928525	76928550	In_Frame_Del	DEL	CCTTACCTTG	-	CCCCTTACCTTGC	CXCL9	c.53_64del	p.18_22del
XH_P33	4	77230677	77230677	Missense_Mutation	SNP	G	A	G	STBD1	c.G601A	p.E201K
XH_P33	4	88278465	88278465	Missense_Mutation	SNP	C	T	C	HSD17B11	c.G661A	p.V221I
XH_P33	4	101947064	101947068	Frame_Shift_Del	DEL	GTTAG	-	GTTAG	PPP3CA	c.1364_1368del	p.T455fs
XH_P33	4	107156509	107156509	Frame_Shift_Ins	INS	-	C	-	TBCK	:.1176_1177ins(	p.K393fs
XH_P33	4	107156510	107156510	Frame_Shift_Ins	INS	-	AG	-	TBCK	.1175_1176ins(	p.K392fs
XH_P33	4	129959267	129959267	Missense_Mutation	SNP	A	T	A	SCLT1	c.T305A	p.L102H
XH_P33	4	141403540	141403540	Frame_Shift_Ins	INS	-	CTATATCAGTACT	-	MGAT4D	insCCAGTACTG.	p.K65fs
XH_P33	4	146575269	146575280	In_Frame_Del	DEL	AACGTTCAC/	-	AAACGTTCACAA	MMAA	c.943_954del	p.315_318del
XH_P33	4	148984372	148984372	Frame_Shift_Ins	INS	-	ATGA	-	ARHGAP10	2101_2102insAT	p.A701fs
XH_P33	4	148984374	148984374	Frame_Shift_Ins	INS	-	AG	-	ARHGAP10	2103_2104insA	p.A701fs
XH_P33	4	154502589	154502589	Missense_Mutation	SNP	C	T	C	TMEM131L	c.C769T	p.R257C
XH_P33	4	154502590	154502590	Frame_Shift_Ins	INS	-	TTTTATT	-	TMEM131L	70_771insTTTT/	p.R257fs
XH_P33	4	162841654	162841654	Missense_Mutation	SNP	T	A	T	FSTL5	c.A308T	p.D103V
XH_P33	4	166235300	166235300	Nonsense_Mutation	INS	-	ATTTATATCATAT	-	KLHL2	nsAGAGATTTAT/	I365delinsKRFISYX
XH_P33	4	183652079	183652079	Frame_Shift_Ins	INS	-	ATATTT	-	TENM3	54_2755insATA'	p.E918fs
XH_P33	4	183652080	183652080	Frame_Shift_Ins	INS	-	A	-	TENM3	:.2755_2756ins/	p.R919fs
XH_P33	4	186544619	186544619	Missense_Mutation	SNP	C	T	C	SORBS2	c.G1664A	p.R555H
XH_P33	5	878551	878551	Missense_Mutation	SNP	A	G	A	BRD9	c.T1031C	p.V344A
XH_P33	5	7789866	7789869	Frame_Shift_Del	DEL	CCCG	-	CCCG	ADCY2	c.2581_2584del	p.P861fs
XH_P33	5	32403346	32403346	Missense_Mutation	SNP	C	T	C	ZFR	c.G1381A	p.V461I
XH_P33	5	35867486	35867486	Frame_Shift_Ins	INS	-	TGGCTTT	-	IL7R	00_301insTGGC	p.L100fs
XH_P33	5	37148323	37148323	In_Frame_Ins	INS	-	AGATTAGGATTA	-	C5orf42	8259insTAATCC	2753delinsNNPNL
XH_P33	5	40853480	40853480	Missense_Mutation	SNP	G	T	G	CARD6	c.G2046T	p.E682D
XH_P33	5	40853481	40853481	Missense_Mutation	SNP	G	T	G	CARD6	c.G2047T	p.G683C
XH_P33	5	53813830	53813830	Silent	SNP	A	C	A	SNX18	c.A48C	p.P16P

XH_P33	5	58272278	58272278	Frame_Shift_Ins	INS	-	CTTCC	-	PDE4D	056_1057insGG	p.K353fs
XH_P33	5	64273018	64273018	Silent	SNP	G	A	G	CWC27	c.G1209A	p.T403T
XH_P33	5	72858502	72858525	In_Frame_Del	DEL	AGAACACAC	-	GAGAACACACAT	ANKRA2	c.182_205del	p.61_69del
XH_P33	5	79950724	79950724	In_Frame_Ins	INS	-	CCGCAGCGC	-	MSH3	3_179insCCGCA	.A60delinsAAAP
XH_P33	5	82817887	82817887	Silent	SNP	A	G	A	VCAN	c.A3762G	p.S1254S
XH_P33	5	90281284	90281284	Nonsense_Mutation	INS	-	TTTTTAAGTTAGT	-	ADGRV1	098insTTTTTTTA	33_G6034delinsIFLSX
XH_P33	5	95091218	95091279	Frame_Shift_Del	DEL	CTGTAAGCC	-	CGCTGTAAGCCAT	RHOBTB3	c.801_862del	p.V267fs
XH_P33	5	110836783	110836783	Frame_Shift_Ins	INS	-	TCTGTAAT	-	STARD4	9_20insATTACA	p.G7fs
XH_P33	5	131281106	131281106	Silent	SNP	G	C	G	MEIKIN	c.C93G	p.A31A
XH_P33	5	138356939	138356939	Nonsense_Mutation	INS	-	CCCTACCGTTTTT	-	SIL1	AAAAAAAAAACG	0_V231delinsKKKKRX
XH_P33	5	138730407	138730407	Missense_Mutation	SNP	C	T	C	PROB1	c.G364A	p.G122S
XH_P33	5	140516786	140516786	Silent	SNP	G	A	G	PCDHB5	c.G1770A	p.A590A
XH_P33	5	140563579	140563579	Missense_Mutation	SNP	C	T	C	PCDHB16	c.C1445T	p.T482I
XH_P33	5	140605030	140605030	Silent	SNP	T	G	T	PCDHB14	c.T1953G	p.P651P
XH_P33	5	140731740	140731740	Missense_Mutation	SNP	T	C	T	PCDHGB1	c.T1913C	p.L638P
XH_P33	5	140763194	140763194	Frame_Shift_Ins	INS	-	TATTTTTACATTT	-	PCDHGA7	insCATTATTTTTA	p.S243fs
XH_P33	5	141337170	141337171	Frame_Shift_Del	DEL	CC	-	CC	PCDH12	c.246_247del	p.E82fs
XH_P33	5	141337171	141337171	Frame_Shift_Ins	INS	-	GGATG	-	PCDH12	245_246insCAT	p.E82fs
XH_P33	5	145665571	145665571	Frame_Shift_Ins	INS	-	ATTC	-	RBM27	3161_3162insAT	p.E1054fs
XH_P33	5	145665572	145665572	Missense_Mutation	SNP	G	T	G	RBM27	c.G3162T	p.E1054D
XH_P33	5	148996328	148996328	Silent	SNP	G	A	G	ARHGEF37	c.G657A	p.V219V
XH_P33	5	176025160	176025231	In_Frame_Del	DEL	CACCGCGAG	-	CCCACCGCGAGG	GPRIN1	c.1605_1676del	p.535_559del
XH_P33	6	10557280	10557280	Missense_Mutation	SNP	T	A	T	GCNT2	c.T624A	p.F208L
XH_P33	6	10587038	10587038	Missense_Mutation	SNP	C	G	C	GCNT2	c.C816G	p.D272E
XH_P33	6	15513482	15513482	Silent	SNP	G	A	G	JARID2	c.G2763A	p.L921L
XH_P33	6	26463739	26463739	Missense_Mutation	SNP	T	C	T	BTN2A1	c.T515C	p.V172A
XH_P33	6	26463741	26463741	Missense_Mutation	SNP	A	T	A	BTN2A1	c.A517T	p.I173F
XH_P33	6	27806601	27806601	Silent	SNP	T	C	T	HIST1H2BN	c.T162C	p.G54G
XH_P33	6	34850732	34850767	In_Frame_Del	DEL	CTTTTTCTCTT	-	GCTTTTTCTCTTT	TAF11	c.262_297del	p.88_99del
XH_P33	6	36178202	36178202	Silent	SNP	C	T	C	BRPF3	c.C2076T	p.H692H
XH_P33	6	40360071	40360071	Missense_Mutation	SNP	C	T	C	LRFN2	c.G1981A	p.A661T
XH_P33	6	40360465	40360465	Silent	SNP	A	G	A	LRFN2	c.T1587C	p.I529I
XH_P33	6	43106987	43106987	Silent	SNP	G	A	G	PTK7	c.G1500A	p.A500A
XH_P33	6	44243559	44243559	Silent	SNP	A	G	A	TMEM151B	c.A996G	p.L332L
XH_P33	6	71665986	71665986	Silent	SNP	G	A	G	B3GAT2	c.C147T	p.G49G
XH_P33	6	85473758	85473758	Missense_Mutation	SNP	C	T	C	TBX18	c.G142A	p.G48R
XH_P33	6	111583624	111583624	Frame_Shift_Ins	INS	-	AATCATTAAAAT	-	MFSD4B	3insATAATCATT	p.L64fs
XH_P33	6	133078831	133078831	In_Frame_Ins	INS	-	TACACGAGGGGG	-	VNN2	CCCATGCCCCTC	4delinsAPMPLVYIM
XH_P33	6	135787437	135787437	Frame_Shift_Ins	INS	-	A	-	AHI1	c.263_264insT	p.N88fs
XH_P33	6	135787439	135787439	Frame_Shift_Ins	INS	-	CTAC	-	AHI1	261_262insGTA	p.N88fs
XH_P33	6	136582430	136582430	Frame_Shift_Ins	INS	-	AATTTTTTCTCTC	-	BCLAF1	577insGAGAGA	p.E859fs
XH_P33	6	144184370	144184370	Frame_Shift_Ins	INS	-	GTACTAGAGTT	-	LTV1	_728insGTACTA	p.E243fs
XH_P33	6	146056499	146056499	Missense_Mutation	SNP	C	G	C	EPM2A	c.G136C	p.A46P
XH_P33	6	146262862	146262862	Missense_Mutation	SNP	C	T	C	SHPRH	c.G2387A	p.R796Q
XH_P33	6	160679400	160679400	Silent	SNP	C	A	C	SLC22A2	c.G390T	p.T130T
XH_P33	6	160769811	160769811	Silent	SNP	C	T	C	SLC22A3	c.C360T	p.R120R
XH_P33	6	165844913	165844913	Nonsense_Mutation	INS	-	CTTAA	-	PDE10A	740_741insTTA	247_Q248delinsVX
XH_P33	7	1528979	1529005	Frame_Shift_Del	DEL	TCACCATCTC	-	ATCACCATCTCCA	INTS1	c.2293_2318del	p.M765fs
XH_P33	7	1532649	1532649	Missense_Mutation	SNP	G	A	G	INTS1	c.C2162T	p.P721L
XH_P33	7	2578434	2578434	Missense_Mutation	SNP	G	T	G	BRAT1	c.C1915A	p.P639T
XH_P33	7	6043658	6043658	Silent	SNP	A	G	A	PMS2	c.T195C	p.L65L
XH_P33	7	8791095	8791095	Missense_Mutation	SNP	C	G	C	NXPH1	c.C512G	p.T171R
XH_P33	7	31887668	31887668	Frame_Shift_Ins	INS	-	GTGTTGCT	-	PDE1C	3_894insAGCAA	p.E298fs
XH_P33	7	37934169	37934169	Missense_Mutation	SNP	A	C	A	NME8	c.A1501C	p.T501P
XH_P33	7	42005678	42005678	Missense_Mutation	SNP	G	A	G	GLI3	c.C2993T	p.P998L
XH_P33	7	43496040	43496040	Missense_Mutation	SNP	A	C	A	HECW1	c.A2543C	p.N848T
XH_P33	7	43496043	43496047	Frame_Shift_Del	DEL	GGCGG	-	GGCGG	HECW1	c.2546_2549del	p.R849fs
XH_P33	7	56170560	56170560	Missense_Mutation	SNP	C	T	C	CHCHD2	c.G445A	p.G149S
XH_P33	7	63537941	63537941	Missense_Mutation	SNP	T	C	T	ZNF727	c.T514C	p.C172R
XH_P33	7	63537942	63537942	Missense_Mutation	SNP	G	C	G	ZNF727	c.G515C	p.C172S
XH_P33	7	64168416	64168416	Missense_Mutation	SNP	C	A	C	ZNF107	c.C1845A	p.N615K
XH_P33	7	64168418	64168418	Missense_Mutation	SNP	T	C	T	ZNF107	c.T1847C	p.L616P
XH_P33	7	64169007	64169083	Frame_Shift_Del	DEL	CTACAAGCT	-	TCCTACAAGCTTA	ZNF107	c.2436_2512del	p.P812fs
XH_P33	7	73753250	73753250	Silent	SNP	T	C	T	CLIP2	c.T594C	p.T198T
XH_P33	7	76871086	76871086	Silent	SNP	G	A	G	CCDC146	c.G318A	p.L106L
XH_P33	7	82545635	82545635	Nonsense_Mutation	INS	-	ATTATATT	-	PCLO	6_11667insAAT	889_Y3890delinsQIX
XH_P33	7	87079391	87079391	Frame_Shift_Ins	INS	-	GACT	-	ABCB4	.725_726insAGT	p.S242fs
XH_P33	7	91775459	91775459	Missense_Mutation	SNP	A	C	A	LRRD1	c.T2334G	p.N778K
XH_P33	7	95739378	95739378	Missense_Mutation	SNP	A	G	A	DYNC1I1	c.A1804G	p.T602A
XH_P33	7	99017680	99017680	Silent	SNP	C	T	C	ATP5J2-PTCD1	c.G2013A	p.P671P
XH_P33	7	100549540	100549540	Missense_Mutation	SNP	A	T	A	MUC3A	c.A121T	p.S41C
XH_P33	7	100642572	100642572	Missense_Mutation	SNP	C	T	C	MUC12	c.C8728T	p.P2910S
XH_P33	7	100679209	100679209	Missense_Mutation	SNP	A	G	A	MUC17	c.A4512G	p.I1504M
XH_P33	7	100679210	100679210	Missense_Mutation	SNP	G	C	G	MUC17	c.G4513C	p.A1505P
XH_P33	7	100683474	100683474	Missense_Mutation	SNP	T	G	T	MUC17	c.T8777G	p.V2926G
XH_P33	7	101272355	101272355	Missense_Mutation	SNP	A	T	A	MYL10	c.T44A	p.L15H
XH_P33	7	102087202	102087202	Silent	SNP	T	C	T	ORAI2	c.T237C	p.L79L
XH_P33	7	102584754	102584754	Frame_Shift_Del	DEL	G	-	G	LRRC17	c.1026delG	p.L342fs
XH_P33	7	102584755	102584755	Nonsense_Mutation	SNP	A	T	A	LRRC17	c.A1027T	p.K343X
XH_P33	7	103162576	103162576	Frame_Shift_Ins	INS	-	CCTTCCAAATA	-	RELN	7561insTATTTG	p.N2521fs
XH_P33	7	104787030	104787030	In_Frame_Ins	INS	-	AAG	-	SRPK2	c.663_664insCT	.M222delinsLM
XH_P33	7	104787031	104787031	Missense_Mutation	SNP	C	G	C	SRPK2	c.G663C	p.L221F
XH_P33	7	105254929	105254929	Missense_Mutation	SNP	G	C	G	ATXN7L1	c.C1480G	p.P494A
XH_P33	7	105254930	105254930	Frame_Shift_Ins	INS	-	AG	-	ATXN7L1	.1478_1479insC	p.K493fs
XH_P33	7	105255190	105255190	Frame_Shift_Ins	INS	-	CAAAACCA	-	ATXN7L1	8_1219insTGGT	p.V407fs
XH_P33	7	107580520	107580520	Silent	SNP	C	T	C	LAMB1	c.G3675A	p.R1225R
XH_P33	7	107580521	107580521	Missense_Mutation	SNP	C	T	C	LAMB1	c.G3674A	p.R1225K
XH_P33	7	116339598	116339598	Frame_Shift_Ins	INS	-	T	-	MET	c.461dupT	p.I154fs
XH_P33	7	117250708	117250708	Frame_Shift_Ins	INS	-	TTTTTTTT	-	CFTR	24_3125insTTTT	p.Q1042fs
XH_P33	7	117250709	117250709	Frame_Shift_Ins	INS	-	TTTTTTTTATTAT	-	CFTR	3126insTTTTTTT	p.Q1042fs
XH_P33	7	129152259	129152259	Nonsense_Mutation	INS	-	ACAAATA	-	SMKR1	75_176insACAA	59_K60delinsDKX
XH_P33	7	129152260	129152260	Missense_Mutation	SNP	G	T	G	SMKR1	c.G176T	p.G59V
XH_P33	7	141921566	141921566	In_Frame_Ins	INS	-	TCTTTGGTG	-	MGAM2	5_7256insTCTTT	T2419delinsIFGA
XH_P33	7	142723693	142723693	In_Frame_Ins	INS	-	CAGCCATCACTC	-	OR9A2	ACTTGGAGTGAT	GTMEFALLGVMAVDRYVAVCN
XH_P33	7	150094853	150094853	Silent	SNP	G	A	G	ZNF775	c.G1284A	p.T428T
XH_P33	7	150878260	150878260	Silent	SNP	C	G	C	ASB10	c.G870C	p.A290A
XH_P33	7	151127240	151127240	In_Frame_Ins	INS	-	TTTTCATTT	-	CRYGN	2_443insAAATG	A148delinsEMKT
XH_P33	7	158697941	158697941	Missense_Mutation	SNP	T	C	T	WDR60	c.T76C	p.S26P

XH_P33	8	494659	494664	In_Frame_Del	DEL	GGCGGC	-	GGCGGC	TDRP	c.94_99del	p.32_33del
XH_P33	8	8560602	8560602	Missense_Mutation	SNP	C	T	C	CLDN23	c.C694T	p.P232S
XH_P33	8	11995019	11995019	Silent	SNP	T	G	T	USP17L2	c.A1251C	p.A417A
XH_P33	8	12957657	12957657	Missense_Mutation	SNP	C	T	C	DLC1	c.G656A	p.R219Q
XH_P33	8	22273397	22273397	Frame_Shift_Ins	INS	-	TCAGAGTAGCG(	-	SLC39A14	CGATATCAGAGT	p.H289fs
XH_P33	8	38285486	38285486	Nonsense_Mutation	INS	-	TAAGTAATACTT	-	FGFR1	)7insGAAAGTAT	.K103delinsESITX
XH_P33	8	38386106	38386106	Frame_Shift_Ins	INS	-	AAATCAT	-	C8orf86	49_50insATGAT	p.S17fs
XH_P33	8	38677932	38677932	Silent	SNP	A	G	A	TACC1	c.A585G	p.E195E
XH_P33	8	53569969	53569969	Nonsense_Mutation	INS	-	TAGTATG	-	RB1CC1	19_2420insCAT/	807_S808delinsAYX
XH_P33	8	68028309	68028309	Frame_Shift_Ins	INS	-	AAAAAAATCTG	-	CSPP1	_552insAAAAAA	p.D184fs
XH_P33	8	75233186	75233186	Missense_Mutation	SNP	C	T	C	JPH1	c.G337A	p.G113R
XH_P33	8	94807654	94807654	Silent	SNP	G	A	G	TMEM67	c.G1692A	p.L564L
XH_P33	8	105478933	105478933	Silent	SNP	G	A	G	DPYS	c.C216T	p.F72F
XH_P33	8	122627272	122627272	Frame_Shift_Ins	INS	-	GCATGAG	-	HAS2	35_736insCTCA	p.N246fs
XH_P33	8	126163472	126163472	Missense_Mutation	SNP	C	T	C	NSMCE2	c.C217T	p.R73W
XH_P33	8	133984912	133984912	Missense_Mutation	SNP	C	T	C	TG	c.C6125T	p.A2042V
XH_P33	8	133984913	133984913	Nonsense_Mutation	INS	-	TAATAAA	-	TG	26_6127insTAA	042_W2043delinsAX
XH_P33	8	134144102	134144102	Frame_Shift_Ins	INS	-	TTATATC	-	TG	09_7910insTTA	p.Y2637fs
XH_P33	8	143763490	143763490	Silent	SNP	T	C	T	PSCA	c.T285C	p.A95A
XH_P33	8	145004639	145004639	Silent	SNP	G	A	G	PLEC	c.C2328T	p.A776A
XH_P33	9	2717905	2717905	Missense_Mutation	SNP	G	A	G	KCNV2	c.G166A	p.E56K
XH_P33	9	5774170	5774170	Missense_Mutation	SNP	G	A	G	RIC1	c.G4085A	p.R1362Q
XH_P33	9	13150667	13150667	Frame_Shift_Ins	INS	-	TTTT	-	MPDZ	472_3473insAA	p.P1158fs
XH_P33	9	13150668	13150668	Nonsense_Mutation	INS	-	TTTTATTTATTTA	-	MPDZ	472insATAAATA	.58_S1159delinsINKX
XH_P33	9	15474136	15474136	Silent	SNP	C	T	C	PSIP1	c.G729A	p.K243K
XH_P33	9	94172745	94172745	Missense_Mutation	SNP	C	T	C	NFIL3	c.G272A	p.R91H
XH_P33	9	96097747	96097747	Missense_Mutation	SNP	C	T	C	C9orf129	c.G274A	p.G92S
XH_P33	9	104432683	104432683	Missense_Mutation	SNP	A	T	A	GRIN3A	c.T2011A	p.W671R
XH_P33	9	114454175	114454175	Missense_Mutation	SNP	T	A	T	C9orf84	c.A3773T	p.K1258M
XH_P33	9	125512340	125512340	In_Frame_Ins	INS	-	GGG	-	OR1L6	:.214_215insGG(	p.I72delinsRV
XH_P33	9	128692068	128692068	Silent	SNP	C	T	C	PBX3	c.C426T	p.L142L
XH_P33	9	130151230	130151304	In_Frame_Del	DEL	ACCCTTAGT(	-	TCACCCTTAGTCT	GARNL3	c.2575_2649del	p.859_883del
XH_P33	9	131285026	131285026	Missense_Mutation	SNP	G	A	G	GLE1	c.G512A	p.R171Q
XH_P33	9	131804785	131804785	Missense_Mutation	SNP	T	C	T	MIGA2	c.T299C	p.V100A
XH_P33	9	133911706	133911706	Silent	SNP	C	T	C	LAMC3	c.C963T	p.A321A
XH_P33	9	135946599	135946599	Silent	SNP	C	T	C	CEL	c.C1719T	p.P573P
XH_P33	9	138379059	138379059	Silent	SNP	G	T	G	PPP1R26	c.G2703T	p.L901L
XH_P33	9	138903671	138903671	Silent	SNP	A	G	A	NACC2	c.T1455C	p.P485P
XH_P33	9	140173413	140173413	Missense_Mutation	SNP	G	A	G	TOR4A	c.G272A	p.R91H
XH_P33	10	1042083	1042083	Missense_Mutation	SNP	G	A	G	GTPBP4	c.G361A	p.D121N
XH_P33	10	12077162	12077162	Silent	SNP	C	T	C	UPF2	c.G261A	p.K87K
XH_P33	10	22830930	22830930	In_Frame_Ins	INS	-	AGTAAG	-	PIP4K2A	561_662insCTTA	p.I221delinsTYF
XH_P33	10	43318594	43318596	In_Frame_Del	DEL	CCA	-	CCA	BMS1	c.3161_3163del	p.1054_1055del
XH_P33	10	43883134	43883134	Missense_Mutation	SNP	C	T	C	HNRNPF	c.G199A	p.V67I
XH_P33	10	50678459	50678459	Frame_Shift_Ins	INS	-	AAAG	-	ERCC6	3546_3547insCT	p.S1183fs
XH_P33	10	50819182	50819182	Silent	SNP	T	C	T	SLC18A3	c.T396C	p.A132A
XH_P33	10	52601649	52601649	Missense_Mutation	SNP	A	G	A	A1CF	c.T338C	p.I113T
XH_P33	10	55587197	55587197	In_Frame_Ins	INS	-	GGTGGC	-	PCDH15	09_4110insGC(	.P1370delinsPPP
XH_P33	10	61412631	61412631	Missense_Mutation	SNP	T	C	T	SLC16A9	c.A1168G	p.I390V
XH_P33	10	61665886	61665886	Silent	SNP	C	A	C	CCDC6	c.G297T	p.V99V
XH_P33	10	75155863	75155863	Nonsense_Mutation	INS	-	CTATTTTATCTTT(	-	ANXA7	20insCAAAGATA	07_T108delinsTKIKX
XH_P33	10	75301492	75301492	Missense_Mutation	SNP	G	T	G	USP54	c.C577A	p.Q193K
XH_P33	10	75301494	75301494	In_Frame_Ins	INS	-	ATA	-	USP54	c.574_575insTA	p.N192delinsIY
XH_P33	10	88702390	88702390	Silent	SNP	G	A	G	MMRN2	c.C2151T	p.A717A
XH_P33	10	89720828	89720828	Frame_Shift_Ins	INS	-	TATTT	-	PTEN	979_980insTAT	p.K327fs
XH_P33	10	89720830	89720830	Frame_Shift_Ins	INS	-	TTAATGAT	-	PTEN	1_982insTTAAT	p.K327fs
XH_P33	10	91163408	91163408	Missense_Mutation	SNP	A	T	A	IFIT1	c.A1376T	p.Y459F
XH_P33	10	94669344	94669344	Nonsense_Mutation	INS	-	ATCTAAATAATC/	-	EXOC6	20insAATCTAAA	).I207delinsKSKX
XH_P33	10	98079147	98079147	Silent	SNP	G	A	G	DNTT	c.G507A	p.T169T
XH_P33	10	99332379	99332381	In_Frame_Del	DEL	GGC	-	GGC	ANKRD2	c.173_175del	p.58_59del
XH_P33	10	99439541	99439541	Missense_Mutation	SNP	G	C	G	AVPI1	c.C122G	p.A41G
XH_P33	10	105362335	105362335	Frame_Shift_Ins	INS	-	TCAGGCACTCCA	-	SH3PXD2A	ACCGCTGGAGT(	p.V852fs
XH_P33	10	124399679	124399699	In_Frame_Del	DEL	CATGTACATT	-	CCATGTACATTG(	DMBT1	c.4795_4815del	p.1599_1605del
XH_P33	10	124740079	124740079	Silent	SNP	G	A	G	PSTK	c.G84A	p.A28A
XH_P33	10	129904590	129904590	Frame_Shift_Ins	INS	-	CTAACACC	-	MKI67	3_4434insGGTG	p.L1478fs
XH_P33	10	129921430	129921430	Silent	SNP	A	T	A	MKI67	c.T96A	p.G32G
XH_P33	11	903709	903709	Missense_Mutation	SNP	T	A	T	CHID1	c.A181T	p.T61S
XH_P33	11	993907	993907	Silent	SNP	C	G	C	AP2A2	c.C1707G	p.L569L
XH_P33	11	1858572	1858572	Missense_Mutation	SNP	C	T	C	SYT8	c.C1120T	p.R374W
XH_P33	11	1977552	1977552	Missense_Mutation	SNP	G	A	G	MRPL23	c.G364A	p.A122T
XH_P33	11	5510811	5510825	In_Frame_Del	DEL	CCTATTCTCT/	-	TCCTATTCTCTAT	OR52D1	c.875_889del	p.292_297del
XH_P33	11	7110363	7110363	Silent	SNP	G	A	G	RBMXL2	c.G12A	p.A4A
XH_P33	11	18111005	18111005	Frame_Shift_Ins	INS	-	GATATAAAAGTC	-	SAAL1	642insAGACTTT	p.D214fs
XH_P33	11	18194964	18194964	Missense_Mutation	SNP	A	G	A	MRGPRX4	c.A161G	p.Y54C
XH_P33	11	20623023	20623023	Silent	SNP	C	T	C	SLC6A5	c.C352T	p.L118L
XH_P33	11	31703413	31703413	Silent	SNP	C	T	C	ELP4	c.C1222T	p.L408L
XH_P33	11	31703414	31703414	Missense_Mutation	SNP	T	G	T	ELP4	c.T1223G	p.L408R
XH_P33	11	32456694	32456694	Silent	SNP	C	A	C	WT1	c.G198T	p.P66P
XH_P33	11	46369371	46369371	Splice_Site	INS	-	AAAGCCA	-	DGKZ	.	.
XH_P33	11	61914260	61914260	Missense_Mutation	SNP	G	A	G	INCENP	c.G2078A	p.R693Q
XH_P33	11	63403040	63403040	Missense_Mutation	SNP	C	T	C	ATL3	c.G947A	p.G316E
XH_P33	11	63403041	63403041	Frame_Shift_Ins	INS	-	TTTTTTTTTTTTTT	-	ATL3	5insAAAAAAAAA	p.G316fs
XH_P33	11	64027648	64027648	Frame_Shift_Ins	INS	-	GGTGGTGTCCA(	-	PLCB3	GAGCTGGGTGG	p.E491fs
XH_P33	11	64029457	64029457	Silent	SNP	C	G	C	PLCB3	c.C1746G	p.P582P
XH_P33	11	64645871	64645871	Silent	SNP	A	G	A	EHD1	c.T66C	p.A22A
XH_P33	11	71948483	71948483	Frame_Shift_Ins	INS	-	C	-	INPPL1	c.3196dupC	p.L1065fs
XH_P33	11	73534065	73534065	Missense_Mutation	SNP	T	C	T	MRPL48	c.T8C	p.L3P
XH_P33	11	73534066	73534066	Silent	SNP	G	A	G	MRPL48	c.G9A	p.L3L
XH_P33	11	75277992	75277992	Silent	SNP	C	T	C	SERPINH1	c.C598T	p.L200L
XH_P33	11	82880240	82880317	In_Frame_Del	DEL	GTCTGAGAT	-	GGTCTGAGATTT	PCF11	c.3256_3333del	p.1086_1111del
XH_P33	11	89177304	89177304	Missense_Mutation	SNP	T	A	T	NOX4	c.A371T	p.E124V
XH_P33	11	107799426	107799426	Silent	SNP	C	A	C	RAB39A	c.C132A	p.T44T
XH_P33	11	117034567	117034567	In_Frame_Ins	INS	-	GATGAAAATCTT	-	PAFAH1B2	ATTTAGATGAAA	76delinsHLDENLFFF
XH_P33	11	117329592	117329592	Missense_Mutation	SNP	C	T	C	DSCAML1	c.G3626A	p.R1209Q
XH_P33	11	128781758	128781758	Missense_Mutation	SNP	C	T	C	KCNJ5	c.C590T	p.A197V
XH_P33	11	132399080	132399080	Missense_Mutation	SNP	A	G	A	OPCML	c.T401C	p.V134A
XH_P33	12	248155	248155	Silent	SNP	C	T	C	IQSEC3	c.C717T	p.A239A
XH_P33	12	319125	319125	Missense_Mutation	SNP	A	G	A	SLC6A12	c.T28C	p.C10R

XH_P33	12	7636167	7636228	Frame_Shift_Del	DEL	ACTGTCCCC(	-	ACACTGTCCCCCA	CD163	c.2823_2884del	p.R941fs
XH_P33	12	7923090	7923090	Missense_Mutation	SNP	G	C	G	NANOGNB	c.G491C	p.R164T
XH_P33	12	10570989	10570989	Nonsense_Mutation	SNP	G	T	G	KLRC3	c.C440A	p.S147X
XH_P33	12	26275555	26275555	Missense_Mutation	SNP	G	A	G	BHLHE41	c.C893T	p.A298V
XH_P33	12	32481295	32481295	Frame_Shift_Ins	INS	-	AGTCC	-	BICD1	906_1907insAG	p.I636fs
XH_P33	12	49420871	49420871	Nonsense_Mutation	SNP	G	A	G	KMT2D	c.C14878T	p.R4960X
XH_P33	12	50571176	50571176	Frame_Shift_Ins	INS	-	GGAAC	-	LIMA1	044_1045insGT	p.T349fs
XH_P33	12	52139801	52139801	In_Frame_Ins	INS	-	AGT	-	SCN8A	2113_2114insA(	p.T705delinsKS
XH_P33	12	52139802	52139802	In_Frame_Ins	INS	-	TATAAAGGT	-	SCN8A	4_2115insTATA	.T705delinsTIKV
XH_P33	12	52284500	52284500	Missense_Mutation	SNP	A	G	A	ANKRD33	c.A188G	p.Q63R
XH_P33	12	53012149	53012149	Missense_Mutation	SNP	C	A	C	KRT73	c.G160T	p.A54S
XH_P33	12	55420611	55420653	Frame_Shift_Del	DEL	ATATTTGGG(	-	TATATTTGGGCT	NEUROD4	c.388_430del	p.R130fs
XH_P33	12	56194672	56194672	In_Frame_Ins	INS	-	ATAGATATTTAT	-	SARNP	149insATAAATA	.A50delinsDKYLS
XH_P33	12	56194673	56194673	Missense_Mutation	SNP	C	T	C	SARNP	c.G148A	p.A50T
XH_P33	12	56814867	56814867	Nonsense_Mutation	INS	-	A	-	TIMELESS	c.2916_2917ins	E973_E974delinsX
XH_P33	12	56814869	56814869	Nonsense_Mutation	INS	-	TCCTTAGG	-	TIMELESS	4_2915insCCTA	972_E973delinsAX
XH_P33	12	57587453	57587453	Missense_Mutation	SNP	G	A	G	LRP1	c.G7789A	p.E2597K
XH_P33	12	57629558	57629558	Missense_Mutation	SNP	G	A	G	NDUFA4L2	c.C181T	p.R61C
XH_P33	12	57648716	57648716	Missense_Mutation	SNP	G	A	G	R3HDM2	c.C2717T	p.S906L
XH_P33	12	64011087	64011087	Silent	SNP	C	T	C	DPY19L2	c.G1215A	p.T405T
XH_P33	12	81009984	81009984	Silent	SNP	C	T	C	PTPRQ	c.C4653T	p.Y1551Y
XH_P33	12	93772199	93772199	Splice_Site	INS	-	GCTGCTGGTG	-	NUDT4	.	.
XH_P33	12	102589861	102589861	Frame_Shift_Ins	INS	-	ACATGATATTGA	-	PARPBP	90insTTACATGA	p.K430fs
XH_P33	12	118681297	118681297	Missense_Mutation	SNP	C	T	C	TAOK3	c.G217A	p.V73I
XH_P33	12	118681299	118681299	Nonsense_Mutation	INS	-	AGTATTTAACATT	-	TAOK3	AACAAATGTTAA	V73delinsVLTNVKYX
XH_P33	12	121174899	121174899	Silent	SNP	T	C	T	ACADS	c.T321C	p.R107R
XH_P33	12	121605355	121605355	Missense_Mutation	SNP	G	A	G	P2RX7	c.G809A	p.R270H
XH_P33	12	124821696	124821696	Silent	SNP	C	T	C	NCOR2	c.G5688A	p.P1896P
XH_P33	12	124826598	124826598	Silent	SNP	T	C	T	NCOR2	c.A4929G	p.R1643R
XH_P33	12	132688137	132688137	Silent	SNP	A	G	A	GALNT9	c.T78C	p.Y26Y
XH_P33	13	23929696	23929696	Frame_Shift_Ins	INS	-	ATCATTG	-	SACS	13_614insCAAT(	p.L205fs
XH_P33	13	24410388	24410388	Frame_Shift_Ins	INS	-	TATTAAA	-	MIPEP	43_1644insTTTA	p.Q548fs
XH_P33	13	24410390	24410390	Missense_Mutation	SNP	G	C	G	MIPEP	c.C1642G	p.Q548E
XH_P33	13	49039488	49039488	Nonsense_Mutation	INS	-	AGTAGATTAT	-	RB1	_2474insAGTAC	825_T826delinsKX
XH_P33	13	49039490	49039490	Missense_Mutation	SNP	G	T	G	RB1	c.G2475T	p.M825I
XH_P33	13	53217270	53217270	Missense_Mutation	SNP	A	G	A	HNRNPA1L2	c.A643G	p.N215D
XH_P33	13	53421432	53421432	Silent	SNP	C	A	C	PCDH8	c.G1140T	p.G380G
XH_P33	13	76447992	76447992	Missense_Mutation	SNP	A	T	A	LMO7DN	c.A200T	p.H67L
XH_P33	13	99907895	99907895	Nonsense_Mutation	SNP	G	A	G	GPR18	c.C232T	p.R78X
XH_P33	13	101277913	101277913	Missense_Mutation	SNP	G	A	G	TMTC4	c.C1322T	p.A441V
XH_P33	13	103399910	103399910	Frame_Shift_Del	DEL	A	-	A	CCDC168	c.3137delT	p.V1046fs
XH_P33	13	103399911	103399911	Missense_Mutation	SNP	C	T	C	CCDC168	c.G3136A	p.V1046I
XH_P33	13	103483942	103483942	Missense_Mutation	SNP	G	T	G	BIVM	c.G242T	p.R81L
XH_P33	14	24458283	24458283	Missense_Mutation	SNP	G	T	G	DHRS4L2	c.G127T	p.G43W
XH_P33	14	29237757	29237757	Silent	SNP	G	A	G	FOXG1	c.G1272A	p.P424P
XH_P33	14	35483097	35483097	In_Frame_Ins	INS	-	ACAAAGGTA	-	SRP54	_729insACAAA(	S243delinsRQRY
XH_P33	14	45433307	45433383	Frame_Shift_Del	DEL	ATTGGCTAG(	-	GATTGGCTAGGA	TOGARAM1	c.1683_1759del	p.N561fs
XH_P33	14	45716108	45716108	In_Frame_Ins	INS	-	ACTAAGAGG	-	MIS18BP1	1_382insCCTCT	.E128delinsPLSE
XH_P33	14	70039587	70039587	Silent	SNP	C	T	C	CCDC177	c.G753A	p.S251S
XH_P33	14	75276122	75276122	Missense_Mutation	SNP	C	T	C	YLPM1	c.C4561T	p.P1521S
XH_P33	14	75276123	75276123	Frame_Shift_Ins	INS	-	TTAT	-	YLPM1	4562_4563insTT	p.P1521fs
XH_P33	14	77873900	77873900	Silent	SNP	C	T	C	NOXRED1	c.G438A	p.P146P
XH_P33	14	94545760	94545760	Missense_Mutation	SNP	T	G	T	DDX24	c.A329C	p.E110A
XH_P33	14	94545761	94545761	Nonsense_Mutation	SNP	C	A	C	DDX24	c.G328T	p.E110X
XH_P33	14	100070030	100070030	Silent	SNP	G	A	G	CCDC85C	c.C267T	p.C89C
XH_P33	14	101200780	101200780	Silent	SNP	T	C	T	DLK1	c.T699C	p.C233C
XH_P33	14	103998985	103998985	Missense_Mutation	SNP	C	T	C	TRMT61A	c.C398T	p.T133M
XH_P33	14	105268104	105268104	Silent	SNP	G	A	G	ZBTB42	c.G570A	p.L190L
XH_P33	14	105412827	105412827	Silent	SNP	C	T	C	AHNAK2	c.G8661A	p.S2887S
XH_P33	14	105417614	105417614	Missense_Mutation	SNP	A	C	A	AHNAK2	c.T3874G	p.L1292V
XH_P33	15	22368790	22368790	Frame_Shift_Ins	INS	-	CA	-	OR4M2	c.215_216insCA	p.W72fs
XH_P33	15	22369132	22369132	Missense_Mutation	SNP	G	A	G	OR4M2	c.G557A	p.R186Q
XH_P33	15	40903046	40903046	Silent	SNP	T	C	T	KNL1	c.T300C	p.F100F
XH_P33	15	50782542	50782542	In_Frame_Ins	INS	-	AAAGTA	-	USP8	36_1737insAAA	.P579delinsPKY
XH_P33	15	55710606	55710606	Frame_Shift_Ins	INS	-	GTACT	-	C15orf65	204_205insGTA(	p.E68fs
XH_P33	15	56924259	56924259	Frame_Shift_Ins	INS	-	AAAG	-	ZNF280D	2337_2338insCT	p.G780fs
XH_P33	15	59981491	59981491	Missense_Mutation	SNP	T	G	T	BNIP2	c.A149C	p.D50A
XH_P33	15	63433719	63433719	Silent	SNP	T	C	T	LACTB	c.T1359C	p.S453S
XH_P33	15	70959540	70959540	Missense_Mutation	SNP	G	C	G	UACA	c.C3444G	p.N1148K
XH_P33	15	74219546	74219546	Missense_Mutation	SNP	G	T	G	LOXL1	c.G422T	p.R141L
XH_P33	15	101815520	101815520	Frame_Shift_Ins	INS	-	TC	-	SELENOS	c.257_258insGA	p.R86fs
XH_P33	16	334580	334580	Silent	SNP	G	C	G	PDIA2	c.G393C	p.P131P
XH_P33	16	775240	775240	Silent	SNP	G	A	G	CCDC78	c.C489T	p.S163S
XH_P33	16	904551	904551	Missense_Mutation	SNP	G	C	G	LMF1	c.C1685G	p.P562R
XH_P33	16	1268494	1268494	Silent	SNP	C	T	C	CACNA1H	c.C5712T	p.D1904D
XH_P33	16	1270162	1270162	Missense_Mutation	SNP	G	A	G	CACNA1H	c.G6212A	p.R2071H
XH_P33	16	1279623	1279623	Silent	SNP	G	A	G	TPSB2	c.C177T	p.C59C
XH_P33	16	1827836	1827836	Silent	SNP	T	C	T	SPSB3	c.A633G	p.S211S
XH_P33	16	1838037	1838037	Silent	SNP	C	G	C	NUBP2	c.C162G	p.T54T
XH_P33	16	1842209	1842209	Silent	SNP	A	G	A	IGFALS	c.T324C	p.D108D
XH_P33	16	3340524	3340524	Missense_Mutation	SNP	G	A	G	ZNF263	c.G2018A	p.R673H
XH_P33	16	3632477	3632477	Missense_Mutation	SNP	G	A	G	SLX4	c.C5371T	p.R1791C
XH_P33	16	4511849	4511878	In_Frame_Del	DEL	CAGCTCGAT(	-	TCAGCTCGATGT	NMRAL1	c.803_832del	p.268_278del
XH_P33	16	8736341	8736341	Frame_Shift_Ins	INS	-	T	-	METTL22	c.930dupT	p.T310fs
XH_P33	16	8736343	8736343	Missense_Mutation	SNP	G	C	G	METTL22	c.G931C	p.D311H
XH_P33	16	19519072	19519072	Nonsense_Mutation	INS	-	TCTTTTAA	-	GDE1	2_243insTTAAA	M81_E82delinsIX
XH_P33	16	19639032	19639032	Nonsense_Mutation	INS	-	AA	-	C16orf62	.1354_1355insA	452_V453delinsX
XH_P33	16	20360359	20360359	Silent	SNP	G	A	G	UMOD	c.C264T	p.G88G
XH_P33	16	22826339	22826339	Silent	SNP	A	G	A	HS3ST2	c.A408G	p.V136V
XH_P33	16	30364880	30364880	Missense_Mutation	SNP	G	A	G	CD2BP2	c.C617T	p.A206V
XH_P33	16	30793330	30793332	In_Frame_Del	DEL	GCA	-	GCA	ZNF629	c.2317_2319del	p.773_773del
XH_P33	16	72139936	72139936	Silent	SNP	G	A	G	DHX38	c.G2520A	p.L840L
XH_P33	16	72139937	72139937	Nonsense_Mutation	INS	-	TGTTAAATT	-	DHX38	1_2522insTGTT	.Q841delinsLLNX
XH_P33	16	75563746	75563746	Silent	SNP	T	G	T	CHST5	c.A537C	p.P179P
XH_P33	16	81129759	81129759	Missense_Mutation	SNP	C	A	C	GCSH	c.G125T	p.R42L
XH_P33	17	1637102	1637102	Missense_Mutation	SNP	G	A	G	WDR81	c.G1162A	p.G388R
XH_P33	17	4461748	4461748	Nonsense_Mutation	SNP	G	A	G	GGT6	c.C358T	p.R120X
XH_P33	17	5085389	5085389	Silent	SNP	C	T	C	ZNF594	c.G2163A	p.T721T

XH_P33	17	5087013	5087096	In_Frame_Del	DEL	AACTTTGATT	-	TGAACTTTGATTA	ZNF594	c.456_539del	p.152_180del
XH_P33	17	8273524	8273524	Missense_Mutation	SNP	A	T	A	KRBA2	c.T161A	p.F54Y
XH_P33	17	8273526	8273526	Nonsense_Mutation	SNP	G	T	G	KRBA2	c.C159A	p.Y53X
XH_P33	17	11513803	11513803	Silent	SNP	G	A	G	DNAH9	c.G705A	p.E235E
XH_P33	17	11513805	11513805	Frame_Shift_Ins	INS	-	CCAAA	-	DNAH9	707_708insCCA	p.S236fs
XH_P33	17	15458614	15458614	In_Frame_Ins	INS	-	TGAATAATT	-	TVP23C	6_77insAATTAT	p.P26delinsQLFT
XH_P33	17	15458616	15458616	Missense_Mutation	SNP	T	G	T	TVP23C	c.A75C	p.R25S
XH_P33	17	17697094	17697108	In_Frame_Del	DEL	CAGCAGCAG	-	AGCAGCAGCAGC	RAI1	c.832_846del	p.278_282del
XH_P33	17	18023643	18023643	Missense_Mutation	SNP	C	T	C	MYO15A	c.C1529T	p.A510V
XH_P33	17	18167307	18167307	Missense_Mutation	SNP	G	A	G	MIEF2	c.G520A	p.A174T
XH_P33	17	18881381	18881381	Nonsense_Mutation	INS	-	CTGCCAGGCTC	-	FAM83G	AGACCCGGAGC	3_A534delinsGQHVX
XH_P33	17	18881572	18881572	Silent	SNP	C	T	C	FAM83G	c.G1407A	p.R469R
XH_P33	17	27052320	27052320	Splice_Site	INS	-	ATCTCCACTAACA	-	TLCD1	.	.
XH_P33	17	28380656	28380656	Nonsense_Mutation	INS	-	GTTGA	-	EFCAB5	516_1517insGT	506_L507delinsCX
XH_P33	17	32908139	32908139	Missense_Mutation	SNP	G	T	G	TMEM132E	c.G44T	p.C15F
XH_P33	17	35733079	35733079	Silent	SNP	T	C	T	C17orf78	c.T45C	p.D15D
XH_P33	17	36508385	36508385	Silent	SNP	T	C	T	SOCS7	c.T258C	p.P86P
XH_P33	17	37657596	37657596	Nonsense_Mutation	INS	-	CTAATATA	-	CDK12	13_2514insCTAA	838_F839delinsSX
XH_P33	17	37657597	37657597	Silent	SNP	G	A	G	CDK12	c.G2514A	p.S838S
XH_P33	17	38421318	38421318	Missense_Mutation	SNP	C	A	C	WIPF2	c.C890A	p.P297H
XH_P33	17	39580310	39580310	Missense_Mutation	SNP	G	A	G	KRT37	c.C466T	p.R156C
XH_P33	17	39890876	39890876	Missense_Mutation	SNP	T	C	T	HAP1	c.A11G	p.K4R
XH_P33	17	42475096	42475096	Frame_Shift_Ins	INS	-	ATTAATA	-	GPATCH8	14_4115insTAT	p.G1372fs
XH_P33	17	45235583	45235583	Nonsense_Mutation	SNP	A	C	A	CDC27	c.T281G	p.L94X
XH_P33	17	55028298	55028298	Frame_Shift_Ins	INS	-	AGCAACC	-	COIL	04_305insGGTT	p.I102fs
XH_P33	17	57157227	57157227	In_Frame_Ins	INS	-	TTG	-	TRIM37	c.401_402insCA	p.V134delinsVK
XH_P33	17	57157229	57157229	Missense_Mutation	SNP	C	G	C	TRIM37	c.G400C	p.V134L
XH_P33	17	62034818	62034818	Missense_Mutation	SNP	C	A	C	SCN4A	c.G2080T	p.G694C
XH_P33	17	62543873	62543873	Frame_Shift_Ins	INS	-	GAGAT	-	SMURF2	915_1916insAT	p.V639fs
XH_P33	17	62543874	62543874	Missense_Mutation	SNP	C	T	C	SMURF2	c.G1915A	p.V639I
XH_P33	17	62856239	62856239	Missense_Mutation	SNP	A	G	A	LRRC37A3	c.T1379C	p.L460P
XH_P33	17	66985961	66985961	Silent	SNP	A	T	A	ABCA9	c.T3948A	p.V1316V
XH_P33	17	71197860	71197860	Missense_Mutation	SNP	A	G	A	COG1	c.A1894G	p.I632V
XH_P33	17	71232018	71232018	Frame_Shift_Ins	INS	-	GCTTTAATTAAAT	-	C17orf80	CTTTAGCTTTAAT	p.Q133fs
XH_P33	17	74733099	74733099	Silent	SNP	G	A	G	SRSF2	c.C144T	p.D48D
XH_P33	17	76968110	76968110	Missense_Mutation	SNP	G	A	G	LGALS3BP	c.C1306T	p.R436W
XH_P33	17	79770740	79770740	Silent	SNP	C	T	C	GCGR	c.C1095T	p.F365F
XH_P33	18	3729175	3729175	Silent	SNP	C	T	C	DLGAP1	c.G645A	p.P215P
XH_P33	18	12454468	12454468	Frame_Shift_Ins	INS	-	AGAGTTA	-	SPIRE1	50_1251insTAA	p.Y417fs
XH_P33	18	18547885	18547885	In_Frame_Ins	INS	-	TATATA	-	ROCK1	019_3020insTAT	.A1007delinsVYT
XH_P33	18	50705483	50705483	Nonsense_Mutation	SNP	G	T	G	DCC	c.G1570T	p.E524X
XH_P33	18	55226383	55226383	Silent	SNP	G	C	G	FECH	c.C798G	p.P266P
XH_P33	18	56247180	56247180	Silent	SNP	T	C	T	ALPK2	c.A828G	p.L276L
XH_P33	18	56586657	56586657	In_Frame_Ins	INS	-	ATGGTT	-	ZNF532	38_1139insATG	.V380delinsDGL
XH_P33	18	59483514	59483514	Silent	SNP	A	G	A	RNF152	c.T183C	p.P61P
XH_P33	18	77475187	77475187	Missense_Mutation	SNP	T	A	T	CTDP1	c.T1370A	p.M457K
XH_P33	19	1003439	1003439	Missense_Mutation	SNP	G	A	G	GRIN3B	c.G737A	p.R246H
XH_P33	19	1004808	1004808	Silent	SNP	C	T	C	GRIN3B	c.C1308T	p.D436D
XH_P33	19	1049269	1049269	Silent	SNP	G	A	G	ABCA7	c.G2385A	p.L795L
XH_P33	19	1110829	1110829	Silent	SNP	G	A	G	SBNO2	c.C2772T	p.N924N
XH_P33	19	1978978	1978978	Missense_Mutation	SNP	G	A	G	CSNK1G2	c.G568A	p.G190R
XH_P33	19	3611972	3611972	Silent	SNP	G	A	G	CACTIN	c.C2226T	p.N742N
XH_P33	19	5110794	5110794	Silent	SNP	A	G	A	KDM4B	c.A1080G	p.A360A
XH_P33	19	5828064	5828064	Silent	SNP	G	C	G	NRTN	c.G474C	p.R158R
XH_P33	19	8196499	8196499	Silent	SNP	G	A	G	FBN3	c.C1929T	p.C643C
XH_P33	19	9072637	9072637	Nonsense_Mutation	INS	-	CA	-	MUC16	14808_14809ins	4937_T4938delinsX
XH_P33	19	9362031	9362031	Silent	SNP	A	G	A	OR7E24	c.A312G	p.Q104Q
XH_P33	19	9362032	9362032	Missense_Mutation	SNP	A	T	A	OR7E24	c.A313T	p.T105S
XH_P33	19	9452665	9452665	Frame_Shift_Ins	INS	-	GGTTTTCTTT	-	ZNF559	)_731insGGTTTT	p.T244fs
XH_P33	19	11464539	11464539	Missense_Mutation	SNP	C	T	C	CCDC159	c.C761T	p.S254L
XH_P33	19	11917672	11917755	In_Frame_Del	DEL	TCAATATCAT	-	TTTCAATATCATG	ZNF491	c.904_987del	p.302_329del
XH_P33	19	12791901	12791901	Silent	SNP	T	C	T	DHPS	c.A27G	p.E9E
XH_P33	19	15659002	15659002	Missense_Mutation	SNP	G	T	G	CYP4F22	c.G1220T	p.R407L
XH_P33	19	15659003	15659003	Frame_Shift_Ins	INS	-	TCTCTCTCTCT	-	CYP4F22	_1222insTCTCT	p.R407fs
XH_P33	19	17000632	17000632	Missense_Mutation	SNP	G	A	G	F2RL3	c.G358A	p.A120T
XH_P33	19	17283657	17283657	Silent	SNP	C	T	C	MYO9B	c.C2025T	p.I675I
XH_P33	19	17435884	17435884	Silent	SNP	T	C	T	ANO8	c.A2973G	p.S991S
XH_P33	19	19791092	19791092	Silent	SNP	C	T	C	ZNF101	c.C934T	p.L312L
XH_P33	19	19905508	19905508	Silent	SNP	C	G	C	ZNF506	c.G1092C	p.P364P
XH_P33	19	20044369	20044369	Missense_Mutation	SNP	T	A	T	ZNF93	c.T605A	p.I202N
XH_P33	19	20044370	20044370	Silent	SNP	T	A	T	ZNF93	c.T606A	p.I202I
XH_P33	19	20229706	20229706	Missense_Mutation	SNP	G	C	G	ZNF90	c.G1343C	p.S448T
XH_P33	19	20727402	20727402	In_Frame_Ins	INS	-	GGGTTGAGGAC	-	ZNF737	ATAAGTGGTCCT	EECGKAYKWSSTLTTHKVIHTGEKL
XH_P33	19	20727918	20727918	Missense_Mutation	SNP	C	G	C	ZNF737	c.G1091C	p.S364T
XH_P33	19	21477016	21477016	Missense_Mutation	SNP	A	G	A	ZNF708	c.T560C	p.L187P
XH_P33	19	21719097	21719097	Frame_Shift_Ins	INS	-	TTTCTGGCTTCTA	-	ZNF429	TTTATTTCTGGCT	p.F17fs
XH_P33	19	21720463	21720463	Silent	SNP	T	C	T	ZNF429	c.T1416C	p.P472P
XH_P33	19	22154102	22154102	Missense_Mutation	SNP	A	C	A	ZNF208	c.T3734G	p.V1245G
XH_P33	19	22154103	22154103	Missense_Mutation	SNP	C	T	C	ZNF208	c.G3733A	p.V1245I
XH_P33	19	22155615	22155615	Missense_Mutation	SNP	C	T	C	ZNF208	c.G2221A	p.V741I
XH_P33	19	22497267	22497267	Missense_Mutation	SNP	C	T	C	ZNF729	c.C1048T	p.R350C
XH_P33	19	23159061	23159061	Missense_Mutation	SNP	C	T	C	ZNF728	c.G1078A	p.V360I
XH_P33	19	23159522	23159522	Missense_Mutation	SNP	T	C	T	ZNF728	c.A617G	p.H206R
XH_P33	19	23159523	23159523	Missense_Mutation	SNP	G	A	G	ZNF728	c.C616T	p.H206Y
XH_P33	19	23542215	23542215	Missense_Mutation	SNP	A	T	A	ZNF91	c.T3470A	p.L1157H
XH_P33	19	23543464	23543464	Missense_Mutation	SNP	T	C	T	ZNF91	c.A2221G	p.K741E
XH_P33	19	31769763	31769763	Silent	SNP	G	A	G	TSHZ3	c.C936T	p.V312V
XH_P33	19	33424507	33424507	Nonsense_Mutation	SNP	C	A	C	CEP89	c.G736T	p.E246X
XH_P33	19	33424508	33424508	Frame_Shift_Ins	INS	-	GTTGTGATATTT	-	CEP89	735insCAAATAT	p.E245fs
XH_P33	19	33792631	33792631	Silent	SNP	C	A	C	CEBPA	c.G333T	p.T111T
XH_P33	19	34832656	34832656	Nonsense_Mutation	INS	-	GCAGAAGACATT	-	KIAA0355	GGCCTGCAGAA	delinsSAQNSSNTVACRRHSPNX
XH_P33	19	37240878	37240961	In_Frame_Del	DEL	CACATTCCT	-	TCCACATTCCTTA	ZNF850	c.885_968del	p.295_323del
XH_P33	19	38188974	38188988	In_Frame_Del	DEL	ATCTAAGCCT	-	GATCTAAGCCTA	ZNF607	c.2041_2055del	p.681_685del
XH_P33	19	38893856	38893856	Silent	SNP	G	C	G	FAM98C	c.G63C	p.L21L
XH_P33	19	39915758	39915758	Missense_Mutation	SNP	C	G	C	PLEKHG2	c.C3985G	p.P1329A
XH_P33	19	41307170	41307170	Silent	SNP	G	A	G	EGLN2	c.G693A	p.P231P
XH_P33	19	43234147	43234147	Silent	SNP	G	T	G	PSG3	c.C771A	p.V257V
XH_P33	19	44223113	44223113	Missense_Mutation	SNP	G	A	G	IRGC	c.G403A	p.D135N

XH_P33	19	44351733	44351733	Nonsense_Mutation	INS	-	ACTTAAATAATT	-	ZNF283	564insACTTAAA	.A188delinsALKX
XH_P33	19	45395699	45395699	Silent	SNP	G	A	G	TOMM40	c.G324A	p.G108G
XH_P33	19	45395700	45395700	In_Frame_Ins	INS	-	TCCACC	-	TOMM40	325_326insTCCA	p.L109delinsFHL
XH_P33	19	46887120	46887120	Silent	SNP	G	A	G	PPP5C	c.G717A	p.S239S
XH_P33	19	48182787	48182787	Silent	SNP	C	T	C	BICRA	c.C360T	p.A120A
XH_P33	19	48183771	48183771	Silent	SNP	C	T	C	BICRA	c.C1344T	p.S448S
XH_P33	19	48685877	48685877	Silent	SNP	G	A	G	C19orf68	c.G441A	p.P147P
XH_P33	19	49218562	49218602	Frame_Shift_Del	DEL	GGCGGACCC	-	AGGGCGGACCCC	MAMSTR	c.33_73del	p.A11fs
XH_P33	19	49964977	49964977	Missense_Mutation	SNP	C	G	C	ALDH16A1	c.C679G	p.L227V
XH_P33	19	50881840	50881840	Silent	SNP	G	A	G	NR1H2	c.G243A	p.Q81Q
XH_P33	19	51227707	51227707	Silent	SNP	G	C	G	CLEC11A	c.G375C	p.L125L
XH_P33	19	51519346	51519346	Silent	SNP	G	C	G	KLK10	c.C336G	p.T112T
XH_P33	19	53453982	53454065	In_Frame_Del	DEL	AGTATGAAG	-	CCAGTATGAAGT	ZNF816	c.963_1046del	p.321_349del
XH_P33	19	53958652	53958652	Silent	SNP	T	C	T	ZNF761	c.T891C	p.P297P
XH_P33	19	55143156	55143156	Silent	SNP	T	C	T	LILRB1	c.T276C	p.H92H
XH_P33	19	55143157	55143157	Missense_Mutation	SNP	G	A	G	LILRB1	c.G277A	p.A93T
XH_P33	19	55351039	55351039	Missense_Mutation	SNP	C	T	C	KIR2DS4	c.C527T	p.P176L
XH_P33	19	55450843	55450843	Missense_Mutation	SNP	G	C	G	NLRP7	c.C1344G	p.F448L
XH_P33	19	56156380	56156380	Missense_Mutation	SNP	C	T	C	ZNF581	c.C443T	p.P148L
XH_P33	19	56443519	56443519	Silent	SNP	C	T	C	NLRP13	c.G159A	p.P53P
XH_P33	19	57743562	57743562	Missense_Mutation	SNP	G	A	G	AURKC	c.G266A	p.R89H
XH_P33	19	58598709	58598709	Missense_Mutation	SNP	C	T	C	ZSCAN18	c.G211A	p.E71K
XH_P33	20	2187943	2187943	Silent	SNP	G	T	G	LOC388780	c.G153T	p.P51P
XH_P33	20	2187944	2187944	Nonsense_Mutation	SNP	C	T	C	LOC388780	c.C154T	p.Q52X
XH_P33	20	5953254	5953254	Missense_Mutation	SNP	A	G	A	MCM8	c.A1256G	p.Y419C
XH_P33	20	21689974	21689974	Missense_Mutation	SNP	C	T	C	PAX1	c.C1174T	p.P392S
XH_P33	20	23016340	23016340	Missense_Mutation	SNP	C	T	C	SSTR4	c.C220T	p.R74C
XH_P33	20	30142623	30142623	Missense_Mutation	SNP	G	A	G	HM13	c.G799A	p.V267I
XH_P33	20	34220149	34220149	Frame_Shift_Ins	INS	-	TCTTGTAA	-	CPNE1	1_392insTTACA	p.A131fs
XH_P33	20	35690652	35690652	Nonsense_Mutation	INS	-	TTAAATCTTGAG	-	RBL1	8insAGCTCAAGA	Y306_E307delinsX
XH_P33	20	42333925	42333925	Missense_Mutation	SNP	G	A	G	MYBL2	c.G1360A	p.V454M
XH_P33	20	43043159	43043159	Missense_Mutation	SNP	G	A	G	HNF4A	c.G505A	p.V169I
XH_P33	20	44038658	44038658	Missense_Mutation	SNP	G	T	G	DBNDD2	c.G670T	p.D224Y
XH_P33	20	44038659	44038659	Missense_Mutation	SNP	A	C	A	DBNDD2	c.A671C	p.D224A
XH_P33	20	49509359	49509359	Missense_Mutation	SNP	A	T	A	ADNP	c.T1892A	p.L631Q
XH_P33	20	49509360	49509360	Missense_Mutation	SNP	G	T	G	ADNP	c.C1891A	p.L631I
XH_P33	20	57268867	57268867	Silent	SNP	C	A	C	NPEPL1	c.C225A	p.T75T
XH_P33	20	60866809	60866809	Missense_Mutation	SNP	C	T	C	OSBPL2	c.C1264T	p.R422W
XH_P33	20	60893912	60893912	Silent	SNP	A	C	A	LAMA5	c.T7029G	p.A2343A
XH_P33	20	61162267	61162267	Missense_Mutation	SNP	T	C	T	MIR1-1HG	c.T80C	p.V27A
XH_P33	20	61288038	61288038	Missense_Mutation	SNP	G	A	G	SLCO4A1	c.G232A	p.V78I
XH_P33	20	62221766	62221766	Silent	SNP	C	T	C	GMEB2	c.G1269A	p.P423P
XH_P33	20	62328267	62328267	Silent	SNP	C	T	C	TNFRSF6B	c.C147T	p.C49C
XH_P33	21	34399401	34399401	Silent	SNP	A	G	A	OLIG2	c.A231G	p.S77S
XH_P33	21	43161022	43161022	Silent	SNP	C	T	C	RIPK4	c.G2331A	p.T777T
XH_P33	21	43254638	43254638	Missense_Mutation	SNP	C	T	C	PRDM15	c.G1513A	p.G505S
XH_P33	21	45978090	45978090	Missense_Mutation	SNP	C	T	C	KRTAP10-3	c.G509A	p.C170Y
XH_P33	21	47855876	47855876	Silent	SNP	A	G	A	PCNT	c.A8220G	p.T2740T
XH_P33	22	17589794	17589794	Missense_Mutation	SNP	C	A	C	IL17RA	c.C1583A	p.P528Q
XH_P33	22	18301862	18301862	Frame_Shift_Ins	INS	-	AGGCTGGAAGC	-	MICAL3	CCCATACGCTTCC	p.Q1189fs
XH_P33	22	22599739	22599739	Missense_Mutation	SNP	G	A	G	VPREB1	c.G425A	p.R142H
XH_P33	22	22599741	22599741	Missense_Mutation	SNP	G	C	G	VPREB1	c.G427C	p.V143L
XH_P33	22	30776095	30776095	Missense_Mutation	SNP	C	T	C	RNF215	c.G964A	p.A322T
XH_P33	22	30951956	30951956	Missense_Mutation	SNP	T	C	T	GAL3ST1	c.A256G	p.T86A
XH_P33	22	36537893	36537893	Silent	SNP	A	G	A	APOL3	c.T564C	p.N188N
XH_P33	22	39883736	39883736	Silent	SNP	G	C	G	MGAT3	c.G384C	p.P128P
XH_P33	22	45792336	45792336	Missense_Mutation	SNP	C	A	C	SMC1B	c.G1144T	p.V382L
XH_P33	22	45792337	45792337	In_Frame_Ins	INS	-	GAAATTTTA	-	SMC1B	2_1143insTAAA	.Q381delinsHKIS
XH_P33	22	46859815	46859815	Silent	SNP	G	A	G	CELSR1	c.C3972T	p.F1324F
XH_P33	22	50684415	50684415	Missense_Mutation	SNP	T	C	T	HDAC10	c.A1697G	p.Q566R
XH_P33	22	50686830	50686830	Silent	SNP	G	A	G	HDAC10	c.C918T	p.A306A
XH_P33	X	3021841	3021841	Missense_Mutation	SNP	G	A	G	ARSF	c.G1141A	p.V381I
XH_P33	X	13337438	13337438	Frame_Shift_Ins	INS	-	GAAAAGC	-	ATXN3L	15_616insGCTT	p.K206fs
XH_P33	X	15332558	15332558	Silent	SNP	A	G	A	ASB11	c.T51C	p.R17R
XH_P33	X	40495846	40495846	Missense_Mutation	SNP	A	T	A	CXorf38	c.T383A	p.L128Q
XH_P33	X	48690749	48690749	Silent	SNP	C	T	C	PCSK1N	c.G117A	p.E39E
XH_P33	X	69828949	69828949	Missense_Mutation	SNP	C	A	C	TEX11	c.G1871T	p.R624L
XH_P33	X	76778843	76778843	Missense_Mutation	SNP	C	T	C	ATRX	c.G6622A	p.E2208K
XH_P33	X	76778844	76778844	Frame_Shift_Ins	INS	-	TGTTTTTG	-	ATRX	0_6621insCAAA	p.K2207fs
XH_P33	X	135427087	135427087	Frame_Shift_Ins	INS	-	GT	-	ADGRG4	.1222_1223insG	p.E408fs
XH_P33	X	148037594	148037594	Frame_Shift_Ins	INS	-	TTTGTTTCTGG	-	AFF2	_943insTTTGTTT	p.A314fs
XH_P33	X	148037596	148037596	Missense_Mutation	SNP	C	A	C	AFF2	c.C944A	p.T315N
XH_P33	X	151337014	151337014	Frame_Shift_Ins	INS	-	CATA	-	GABRA3	.164_1165insTA	p.A389fs
XH_P33	X	153706739	153706739	Nonsense_Mutation	INS	-	ATGGAGACATGG	-	LAGE3	GTCCCCCATGTC	/67_P68delinsAPX
XH_P33	1	152282032	152282032	Missense_Mutation	SNV	G	C	C	FLG	c.5330C>G	P.S1777C
XH_P33	1	153317801	153317801	Missense_Mutation	SNV	A	G	G	PGLYRP4	c.197T>C	P.V66A
XH_P33	1	161483723	161483723	Splice_Site	SNV	G	A	A	FCGR2A	c.780+1G>A	
XH_P33	1	75055571	75055571	Missense_Mutation	SNV	T	A	A	ERICH3	c.1920A>T	P.L640F
XH_P33	1	91182240	91182240	Silent	SNV	C	A	A	BARHL2	c.513G>T	P.P171P
XH_P33	10	52601649	52601649	Missense_Mutation	SNV	A	G	G	A1CF	c.338T>C	P.I113T
XH_P33	10	61412631	61412631	Missense_Mutation	SNV	T	C	C	SLC16A9	c.1168A>G	P.I390V
XH_P33	11	128781758	128781758	Missense_Mutation	SNV	C	T	T	KCNJ5	c.590C>T	P.A197V
XH_P33	11	71948483	71948483	Frame_Shift_Ins	INDEL	-	C	C	INPPL1	c.3201dupC	P.S1068fs
XH_P33	12	49420871	49420871	Nonsense_Mutation	SNV	G	A	A	KMT2D	c.14878C>T	P.R4960*
XH_P33	12	53012149	53012149	Missense_Mutation	SNV	C	A	A	KRT73	c.160G>T	P.A54S
XH_P33	12	57648716	57648716	Missense_Mutation	SNV	G	A	A	R3HDM2	c.2873C>T	P.S958L
XH_P33	13	103483942	103483942	Missense_Mutation	SNV	G	T	T	BIVM	c.242G>T	P.R81L
XH_P33	14	29237757	29237757	Silent	SNV	G	A	A	FOXG1	c.1272G>A	P.P424P
XH_P33	14	77873900	77873900	Silent	SNV	C	T	T	NOXRED1	c.438G>A	P.P146P
XH_P33	15	63433719	63433719	Silent	SNV	T	C	C	LACTB	c.1359T>C	P.S453S
XH_P33	17	32908139	32908139	Missense_Mutation	SNV	G	T	T	TMEM132E	c.44G>T	P.C15F
XH_P33	17	62034818	62034818	Missense_Mutation	SNV	C	A	A	SCN4A	c.2080G>T	P.G694C
XH_P33	17	71197860	71197860	Missense_Mutation	SNV	A	G	G	COG1	c.1894A>G	P.I632V
XH_P33	19	21720463	21720463	Silent	SNV	T	C	C	ZNF429	c.1608T>C	P.P536P
XH_P33	19	38188974	38188988	Missense_Mutation	INDEL	ATCTAAGCCT	-	-	ZNF607	055delTTTAGGC	P.F681_S685del
XH_P33	19	57743562	57743562	Missense_Mutation	SNV	G	A	A	AURKC	c.266G>A	P.R89H
XH_P33	2	113942657	113942657	Splice_Site	SNV	C	T	T	PSD4	c.1173+7C>T	
XH_P33	2	153575161	153575161	Nonsense_Mutation	SNV	G	A	A	ARL6IP6	c.23G>A	P.W8*

XH_P33	2	179638614	179638614	Missense_Mutation	SNV	G	T	T	TTN	c.7281C>A	P.F2427L
XH_P33	2	27165492	27165492	Silent	SNV	C	A	A	DPYSL5	c.1314C>A	P.V438V
XH_P33	20	21689974	21689974	Missense_Mutation	SNV	C	T	T	PAX1	c.1174C>T	P.P392S
XH_P33	20	60866809	60866809	Missense_Mutation	SNV	C	T	T	OSBPL2	c.1264C>T	P.R422W
XH_P33	3	151451961	151451961	Splice_Site	SNV	G	A	A	AADACL2	c.138G>A	P.T46T
XH_P33	3	39118670	39118670	Nonsense_Mutation	SNV	G	A	A	WDR48	c.692G>A	P.W231*
XH_P33	3	44816738	44816738	Splice_Site	SNV	G	T	T	KIF15	c.63-8G>T	
XH_P33	4	162841654	162841654	Missense_Mutation	SNV	T	A	A	FSTL5	c.308A>T	P.D103V
XH_P33	4	55968137	55968137	Silent	SNV	G	A	A	KDR	c.2193C>T	P.D731D
XH_P33	5	40853480	40853480	Missense_Mutation	SNV	G	T	T	CARD6	c.2046G>T	P.E682D
XH_P33	5	40853481	40853481	Missense_Mutation	SNV	G	T	T	CARD6	c.2047G>T	P.G683C
XH_P33	5	82817887	82817887	Silent	SNV	A	G	G	VCAN	c.3762A>G	P.S1254S
XH_P33	6	10557280	10557280	Missense_Mutation	SNV	T	A	A	GCNT2	c.624T>A	P.F208L
XH_P33	6	40360071	40360071	Missense_Mutation	SNV	C	T	T	LRFN2	c.1981G>A	P.A661T
XH_P33	7	37934169	37934169	Missense_Mutation	SNV	A	C	C	NME8	c.1501A>C	P.T501P
XH_P33	7	39243828	39243828	Splice_Site	SNV	T	C	C	POU6F2	c.191-6T>C	
XH_P33	7	91775459	91775459	Missense_Mutation	SNV	A	C	C	LRRD1	c.2334T>G	P.N778K
XH_P33	8	75233186	75233186	Missense_Mutation	SNV	C	T	T	JPH1	c.337G>A	P.G113R
XH_P33	8	94807654	94807654	Silent	SNV	G	A	A	TMEM67	c.1449G>A	P.L483L
XH_P33	9	104432683	104432683	Missense_Mutation	SNV	A	T	T	GRIN3A	c.2011T>A	P.W671R
XH_P33	9	114454175	114454175	Missense_Mutation	SNV	T	A	A	C9orf84	c.3773A>T	P.K1258M
XH_P34	1	159847257	159847257	Splice_Site	SNV	C	T	T	CFAP45	c.1045-5G>A	
XH_P34	1	222721291	222721291	Missense_Mutation	SNV	C	A	A	HHIPL2	c.96G>T	P.L32F
XH_P34	1	235335950	235335950	Missense_Mutation	SNV	T	A	A	ARID4B	c.3794A>T	P.K1265M
XH_P34	1	236917388	236917388	Splice_Site	SNV	C	T	T	ACTN2	c.1974+7C>T	
XH_P34	1	35365685	35365685	Missense_Mutation	SNV	C	T	T	DLGAP3	c.1297G>A	P.D433N
XH_P34	1	40033476	40033476	Missense_Mutation	SNV	C	T	T	PABPC4	c.919G>A	P.D307N
XH_P34	10	50340346	50340346	Missense_Mutation	SNV	C	T	T	FAM170B	c.164G>A	P.R55Q
XH_P34	10	54053555	54053555	Splice_Site	SNV	T	A	A	PRKG1	c.1918-7T>A	
XH_P34	10	63760086	63760089	Splice_Site	INDEL	TCTT	-	-	ARID5B	3+7_733+10delCTTT	
XH_P34	11	1087943	1087943	Missense_Mutation	SNV	C	T	T	MUC2	c.3418C>T	P.R1140W
XH_P34	11	113650977	113650977	Missense_Mutation	SNV	C	T	T	CLDN25	c.460C>T	P.P154S
XH_P34	11	130784669	130784669	Missense_Mutation	SNV	G	C	C	SNX19	c.1166C>G	P.T389S
XH_P34	11	55432646	55432646	Missense_Mutation	SNV	G	C	C	OR4C6	c.4G>C	P.E2Q
XH_P34	11	61533447	61533447	Missense_Mutation	SNV	C	G	G	MYRF	c.152C>G	P.S51C
XH_P34	11	62520254	62520254	Missense_Mutation	SNV	C	A	A	ZBTB3	c.1033G>T	P.A345S
XH_P34	12	103352426	103352426	Missense_Mutation	SNV	A	G	G	ASCL1	c.404A>G	P.N135S
XH_P34	12	124832428	124832428	Nonsense_Mutation	SNV	G	A	A	NCOR2	c.3985C>T	P.R1329*
XH_P34	12	3649792	3649792	Missense_Mutation	SNV	G	C	C	PRMT8	c.69G>C	P.Q23H
XH_P34	12	45270449	45270449	Silent	SNV	G	A	A	NELL2	c.60C>T	P.S20S
XH_P34	12	54645964	54645964	Missense_Mutation	SNV	A	G	G	CBX5	c.185T>C	P.L62P
XH_P34	12	54645965	54645965	Frame_Shift_Ins	INDEL	-	CTCTTGTTACTT	CTCTTGTTACTT	CBX5	TCAGAAAGTAAC	P.L62fs
XH_P34	13	20056699	20056699	Splice_Site	INDEL	A	-	-	TPTE2	c.120-12delT	
XH_P34	14	34263088	34263088	Splice_Site	INDEL	-	T	T	NPAS3	c.1154-7dupT	
XH_P34	14	76123755	76123755	Missense_Mutation	SNV	T	C	C	C14orf1	c.91A>G	P.T31A
XH_P34	16	67861432	67861457	Frame_Shift_Del	INDEL	ACGAGTACT	-	-	TSNAXIP1	AGAAGGACGAG	P.E617fs
XH_P34	17	19454777	19454777	Missense_Mutation	SNV	A	G	G	SLC47A1	c.539A>G	P.N180S
XH_P34	17	4926940	4926940	Missense_Mutation	SNV	C	T	T	KIF1C	c.2806C>T	P.R936C
XH_P34	17	72999582	72999582	Missense_Mutation	SNV	C	G	G	CDR2L	c.811C>G	P.L271V
XH_P34	18	14542693	14542693	Silent	SNV	C	T	T	POTEC	c.453G>A	P.K151K
XH_P34	19	2276136	2276136	Missense_Mutation	SNV	C	T	T	C19orf35	c.965G>A	P.G322E
XH_P34	19	56284099	56284099	Missense_Mutation	SNV	G	A	A	RFPL4AL1	c.418G>A	P.V140I
XH_P34	19	9071439	9071439	Missense_Mutation	SNV	G	A	A	MUC16	c.16007C>T	P.S5336F
XH_P34	2	160741721	160741721	Nonsense_Mutation	SNV	C	A	A	LY75-CD302	c.997G>T	P.E333*
XH_P34	2	186671135	186671135	Missense_Mutation	SNV	C	T	T	FSIP2	c.17102C>T	P.S5701L
XH_P34	2	203075510	203075510	Missense_Mutation	SNV	A	G	G	SUMO1	c.185T>C	P.L62P
XH_P34	22	20097626	20097626	Missense_Mutation	SNV	G	A	A	DGCR8	c.2218G>A	P.V740M
XH_P34	22	25023406	25023406	Missense_Mutation	SNV	G	A	A	GGT1	c.1028G>A	P.R343H
XH_P34	3	126226930	126226930	Silent	SNV	C	T	T	UROC1	c.420G>A	P.L140L
XH_P34	3	134086356	134086362	Frame_Shift_Del	INDEL	CCTTCTC	-	-	AMOTL2	2_1198delGAG	P.E398fs
XH_P34	3	160141374	160141374	Silent	SNV	A	G	G	SMC4	c.2181A>G	P.V727V
XH_P34	3	194118088	194118088	Silent	SNV	G	A	A	GP5	c.924C>T	P.A308A
XH_P34	3	195936387	195936387	Splice_Site	SNV	C	T	T	ZDHHC19	c.269-1G>A	
XH_P34	3	49395261	49395261	Nonstop_Mutation	SNV	A	C	C	GPX1	c.313T>G	P.Ter105Eext*?
XH_P34	3	52441257	52441257	Silent	SNV	G	T	T	BAP1	c.513C>A	P.V171V
XH_P34	3	69168484	69168484	Missense_Mutation	SNV	G	T	T	LMOD3	c.1022C>A	P.T341N
XH_P34	4	110772933	110772933	Silent	SNV	T	G	G	LRIT3	c.390T>G	P.L130L
XH_P34	4	141483539	141483539	Splice_Site	SNV	C	T	T	UCP1	c.629-12G>A	
XH_P34	4	77100735	77100735	Nonsense_Mutation	SNV	C	A	A	SCARB2	c.547G>T	P.E183*
XH_P34	5	127685687	127685687	Silent	SNV	G	A	A	FBN2	c.2823C>T	P.C941C
XH_P34	5	128351733	128351733	Silent	SNV	G	A	A	SLC27A6	c.1125G>A	P.G375G
XH_P34	5	76607816	76607816	Splice_Site	SNV	T	C	C	PDE8B	c.340-3T>C	
XH_P34	6	110112635	110112635	Missense_Mutation	SNV	C	T	T	FIG4	c.2237C>T	P.P746L
XH_P34	6	40360377	40360377	Missense_Mutation	SNV	C	T	T	LRFN2	c.1675G>A	P.V559I
XH_P34	7	100349776	100349776	Missense_Mutation	SNV	G	A	A	ZAN	c.2048G>A	P.S683N
XH_P34	7	149519632	149519632	Nonsense_Mutation	SNV	G	A	A	SSPO	c.13122G>A	P.W4374*
XH_P34	7	151074246	151074246	Nonsense_Mutation	SNV	G	T	T	NUB1	c.1855G>T	P.E619*
XH_P34	8	30701243	30701243	Missense_Mutation	SNV	T	C	C	TEX15	c.5291A>G	P.D1764G
XH_P34	9	131370273	131370273	Missense_Mutation	SNV	T	G	G	SPTAN1	c.4289T>G	P.L1430R
XH_P34	9	134103641	134103641	Silent	SNV	C	T	T	NUP214	c.5967C>T	P.A1989A
XH_P34	X	123034518	123034518	Silent	SNV	G	A	A	XIAP	c.1275G>A	P.E425E
XH_P34	X	138856897	138856897	Missense_Mutation	SNV	C	T	T	ATP11C	c.2177G>A	P.R726H
XH_P34	X	2773183	2773183	Silent	SNV	C	T	T	GYG2	c.474C>T	P.H158H
XH_P34	X	47489199	47489199	Silent	SNV	C	T	T	CFP	c.45G>A	P.P15P
XH_P34	X	74280168	74280168	Splice_Site	SNV	G	T	T	ABCB7	c.1936-3C>A	
XH_P35	1	1670472	1670472	Missense_Mutation	SNP	G	A	G	SLC35E2	c.C347T	p.T116M
XH_P35	1	6188946	6188946	Missense_Mutation	SNP	C	T	C	CHD5	c.G3571A	p.D1191N
XH_P35	1	10364110	10364110	Missense_Mutation	SNP	G	A	G	KIF1B	c.G2867A	p.R956Q
XH_P35	1	11199403	11199403	Silent	SNP	C	A	C	MTOR	c.G5088T	p.V1696V
XH_P35	1	17550872	17550872	Silent	SNP	T	G	T	PADI1	c.T357G	p.L119L
XH_P35	1	27105745	27105745	Nonsense_Mutation	SNP	G	T	G	ARID1A	c.G5356T	p.E1786X
XH_P35	1	36025984	36025984	Missense_Mutation	SNP	G	A	G	NCDN	c.G181A	p.D61N
XH_P35	1	38305824	38305824	Missense_Mutation	SNP	G	A	G	MTF1	c.C415T	p.R139W
XH_P35	1	40214638	40214638	Missense_Mutation	SNP	G	A	G	PPIE	c.G572A	p.R191H
XH_P35	1	46184960	46184960	Nonsense_Mutation	INS	-	TATTTTTAATTTT	-	IPP	AAAATGAAAATTA	delinsTYYVSTKMKIKNTX
XH_P35	1	46463416	46463416	Missense_Mutation	SNP	C	T	C	MAST2	c.C130T	p.P44S
XH_P35	1	62910675	62910675	Frame_Shift_Ins	INS	-	TTTTACTTCTTCT	-	USP1	insATTTTTTACT	p.T275fs
XH_P35	1	100614640	100614640	Silent	SNP	G	A	G	LRRC39	c.C966T	p.N322N

XH_P35	1	109849583	109849583	Missense_Mutation	SNP	C	T	C	MYBPHL	c.G31A	p.A11T
XH_P35	1	110581915	110581915	Frame_Shift_Ins	INS	-	T	-	STRIP1	c.163dupT	p.L54fs
XH_P35	1	117617495	117617495	Missense_Mutation	SNP	C	T	C	TTF2	c.C289T	p.P97S
XH_P35	1	120436630	120436630	Missense_Mutation	SNP	C	T	C	ADAM30	c.G2330A	p.S777N
XH_P35	1	120436632	120436632	Nonsense_Mutation	INS	-	AACCAGCTAA	-	ADAM30	_2328insTTAGC	776_S777delinsDX
XH_P35	1	120466386	120466386	Missense_Mutation	SNP	C	T	C	NOTCH2	c.G4733A	p.R1578H
XH_P35	1	151204824	151204824	Missense_Mutation	SNP	C	T	C	PIP5K1A	c.C433T	p.R145W
XH_P35	1	152759927	152759950	In_Frame_Del	DEL	CTCCAGCTCT	-	GCTCCAGCTCTG	LCE1E	c.152_175del	p.51_59del
XH_P35	1	153333264	153333264	Missense_Mutation	SNP	G	A	G	S100A9	c.G295A	p.E99K
XH_P35	1	155011989	155011989	Missense_Mutation	SNP	T	A	T	DCST1	c.T298A	p.L100M
XH_P35	1	156894457	156894457	Silent	SNP	T	A	T	LRRC71	c.T462A	p.I154I
XH_P35	1	160340780	160340780	Missense_Mutation	SNP	C	T	C	NHLH1	c.C259T	p.R87C
XH_P35	1	160811186	160811186	Missense_Mutation	SNP	C	T	C	CD244	c.G484A	p.D162N
XH_P35	1	201181672	201181672	Missense_Mutation	SNP	C	T	C	IGFN1	c.C7651T	p.R2551W
XH_P35	1	201185848	201185848	Missense_Mutation	SNP	C	A	C	IGFN1	c.C9562A	p.L3188M
XH_P35	1	204379981	204379981	Missense_Mutation	SNP	G	A	G	PPP1R15B	c.C559T	p.P187S
XH_P35	1	210010427	210010427	Silent	SNP	C	T	C	DIEXF	c.C933T	p.C311C
XH_P35	1	220191803	220191803	Silent	SNP	C	T	C	EPRS	c.G1482A	p.A494A
XH_P35	1	236306134	236306134	Missense_Mutation	SNP	G	A	G	GPR137B	c.G212A	p.R71H
XH_P35	1	248685312	248685312	Missense_Mutation	SNP	G	A	G	OR2G6	c.G365A	p.R122H
XH_P35	1	248801977	248801977	Missense_Mutation	SNP	A	T	A	OR2T35	c.T583A	p.Y195N
XH_P35	2	17876434	17876434	Missense_Mutation	SNP	C	T	C	SMC6	c.G2663A	p.R888Q
XH_P35	2	17902510	17902510	Missense_Mutation	SNP	G	A	G	SMC6	c.C745T	p.R249C
XH_P35	2	37267597	37267597	Missense_Mutation	SNP	A	G	A	HEATR5B	c.T2921C	p.V974A
XH_P35	2	37267599	37267599	Nonsense_Mutation	SNP	A	C	A	HEATR5B	c.T2919G	p.Y973X
XH_P35	2	54176302	54176302	Missense_Mutation	SNP	G	A	G	PSME4	c.C361T	p.R121C
XH_P35	2	68415816	68415816	Missense_Mutation	SNP	G	A	G	PPP3R1	c.C50T	p.A17V
XH_P35	2	73675421	73675421	Missense_Mutation	SNP	G	T	G	ALMS1	c.G1764T	p.L588F
XH_P35	2	88879049	88879049	Missense_Mutation	SNP	G	A	G	EIF2AK3	c.C1420T	p.R474C
XH_P35	2	97818241	97818241	Missense_Mutation	SNP	T	G	T	ANKRD36	c.T1163G	p.V388G
XH_P35	2	100022844	100022844	Missense_Mutation	SNP	G	A	G	REV1	c.C2554T	p.R852C
XH_P35	2	110849289	110849289	Missense_Mutation	SNP	A	G	A	MALL	c.T164C	p.L55P
XH_P35	2	114713283	114713283	Silent	SNP	G	A	G	ACTR3	c.G1008A	p.T336T
XH_P35	2	133075118	133075118	Missense_Mutation	SNP	G	T	G	ZNF806	c.G579T	p.E193D
XH_P35	2	133075120	133075120	Missense_Mutation	SNP	C	T	C	ZNF806	c.C581T	p.S194F
XH_P35	2	160709792	160709792	Nonsense_Mutation	SNP	C	T	C	LY75	c.G2735A	p.W912X
XH_P35	2	179447755	179447755	Silent	SNP	G	A	G	TTN	c.C38580T	p.S12860S
XH_P35	2	190428890	190428890	Missense_Mutation	SNP	A	C	A	SLC40A1	c.T822G	p.H274Q
XH_P35	2	202207098	202207098	Silent	SNP	C	T	C	ALS2CR12	c.G519A	p.K173K
XH_P35	2	209113113	209113113	Missense_Mutation	SNP	G	C	G	IDH1	c.C394G	p.R132G
XH_P35	2	217498317	217498317	Frame_Shift_Ins	INS	-	GC	-	IGFBP2	c.71_72insGC	p.L24fs
XH_P35	2	217498318	217498318	Frame_Shift_Ins	INS	-	TGCTGCTGCTGC	-	IGFBP2	CTCTTGCTGCTG	p.L24fs
XH_P35	2	232262967	232262967	Silent	SNP	G	A	G	B3GNT7	c.G537A	p.T179T
XH_P35	2	242009366	242009366	Silent	SNP	C	T	C	SNED1	c.C3339T	p.A1113A
XH_P35	2	242757562	242757562	Missense_Mutation	SNP	G	A	G	NEU4	c.G682A	p.V228M
XH_P35	3	30686325	30686325	Missense_Mutation	SNP	T	C	T	TGFBR2	c.T181C	p.C61R
XH_P35	3	36756856	36756856	Missense_Mutation	SNP	C	T	C	DCLK3	c.G1910A	p.R637Q
XH_P35	3	39453515	39453515	Silent	SNP	A	G	A	RPSA	c.A771G	p.V257V
XH_P35	3	42906774	42906774	Silent	SNP	C	T	C	ACKR2	c.C780T	p.F260F
XH_P35	3	50683187	50683187	Missense_Mutation	SNP	G	T	G	MAPKAPK3	c.G675T	p.M225I
XH_P35	3	52550245	52550245	Missense_Mutation	SNP	T	C	T	STAB1	c.T4135C	p.C1379R
XH_P35	3	69244235	69244235	Missense_Mutation	SNP	G	C	G	FRMD4B	c.C1426G	p.Q476E
XH_P35	3	108776269	108776269	Missense_Mutation	SNP	C	T	C	MORC1	c.G1096A	p.A366T
XH_P35	3	121351315	121351315	In_Frame_Ins	INS	-	GGCTCAGGCTCA	-	HCLS1	993insTGAGCCT	P331delinsPEPEP
XH_P35	3	129251554	129251554	Missense_Mutation	SNP	C	T	C	RHO	c.C875T	p.A292V
XH_P35	3	137880741	137880743	In_Frame_Del	DEL	TCG	-	TCG	DBR1	c.1623_1625del	p.541_542del
XH_P35	3	178936083	178936083	Missense_Mutation	SNP	A	T	A	PIK3CA	c.A1625T	p.E542V
XH_P35	3	179046111	179046111	Silent	SNP	G	A	G	ZNF639	c.G30A	p.R10R
XH_P35	3	195507173	195507173	In_Frame_Ins	INS	-	AAGAGAGGTGGC	-	MUC4	AGGTCACGCCAC	DTSSVSTGHATSLPVTDTSSVSTGHATP
XH_P35	4	2176479	2176479	Splice_Site	SNP	C	T	C	POLN	.	.
XH_P35	4	9784705	9784705	Missense_Mutation	SNP	A	C	A	DRD5	c.A1052C	p.N351T
XH_P35	4	15689835	15689835	Missense_Mutation	SNP	A	C	A	FAM200B	c.A1235C	p.K412T
XH_P35	4	36317975	36317975	Missense_Mutation	SNP	C	T	C	DTHD1	c.C1337T	p.P446L
XH_P35	4	40434705	40434725	In_Frame_Del	DEL	GCGGCGGCT	-	CTGCGGCGGCTG	RBM47	c.1278_1298del	p.426_433del
XH_P35	4	41748011	41748028	In_Frame_Del	DEL	CCGCCGCTGC	-	GCCGCCGCTGCC	PHOX2B	c.741_758del	p.247_253del
XH_P35	4	56225642	56225642	Silent	SNP	G	A	G	SRD5A3	c.G351A	p.A117A
XH_P35	4	79308695	79308695	Missense_Mutation	SNP	C	T	C	FRAS1	c.C3815T	p.P1272L
XH_P35	4	88359522	88359522	Silent	SNP	G	A	G	NUDT9	c.G441A	p.P147P
XH_P35	4	89950618	89950618	Silent	SNP	C	T	C	FAM13A	c.G210A	p.T70T
XH_P35	4	106196618	106196618	Frame_Shift_Ins	INS	-	TACAGATCCATC	-	TET2	TACCTATACAGA	p.P1651fs
XH_P35	4	166946559	166946559	Frame_Shift_Ins	INS	-	TCTACAAGAATC	-	TLL1	insAACTCTACAA	p.R378fs
XH_P35	5	7866894	7866894	Missense_Mutation	SNP	C	T	C	FASTKD3	c.G1303A	p.V435M
XH_P35	5	32712262	32712262	Missense_Mutation	SNP	A	T	A	NPR3	c.A380T	p.K127M
XH_P35	5	65105431	65105431	Silent	SNP	C	T	C	NLN	c.C1617T	p.V539V
XH_P35	5	76734154	76734154	Silent	SNP	G	A	G	WDR41	c.C915T	p.Y305Y
XH_P35	5	90124829	90124829	Nonsense_Mutation	INS	-	TATCTGTAGATA	-	ADGRV1	TATATTTCTATCT	N5479delinsNYYX
XH_P35	5	122161755	122161755	Missense_Mutation	SNP	A	G	A	SNX2	c.A872G	p.D291G
XH_P35	5	140346712	140346712	Silent	SNP	T	C	T	PCDHAC2	c.T361C	p.L121L
XH_P35	5	149588999	149588999	Missense_Mutation	SNP	G	A	G	SLC6A7	c.G1732A	p.D578N
XH_P35	5	178045557	178045557	Silent	SNP	C	T	C	CLK4	c.G384A	p.S128S
XH_P35	6	7602864	7602864	Nonsense_Mutation	SNP	C	T	C	SNRNP48	c.C604T	p.R202X
XH_P35	6	25495416	25495416	Missense_Mutation	SNP	G	A	G	CARMIL1	c.G1298A	p.G433D
XH_P35	6	40999846	40999846	Missense_Mutation	SNP	T	A	T	UNC5CL	c.A980T	p.Q327L
XH_P35	6	49412434	49412434	Missense_Mutation	SNP	G	A	G	MUT	c.C1594T	p.R532C
XH_P35	6	52850316	52850316	Nonsense_Mutation	SNP	G	A	G	GSTA4	c.C205T	p.R69X
XH_P35	6	63995494	63995494	Frame_Shift_Ins	INS	-	A	-	LGSN	c.327dupT	p.Q110fs
XH_P35	6	74124361	74124361	Missense_Mutation	SNP	G	A	G	DDX43	c.G1697A	p.R566Q
XH_P35	6	90578368	90578368	Nonsense_Mutation	INS	-	AATTATCTTCATC	-	CASP8AP2	CCTTACAATTATC	IVCHLCLFFLTIIFIYLYHPTSQVFHSX
XH_P35	6	121768925	121768925	Frame_Shift_Del	DEL	C	-	C	GJA1	c.932delC	p.A311fs
XH_P35	6	135264980	135264980	Missense_Mutation	SNP	G	A	G	ALDH8A1	c.C263T	p.A88V
XH_P35	6	165703524	165703524	Nonsense_Mutation	SNP	G	A	G	C6orf118	c.C1153T	p.R385X
XH_P35	7	14017062	14017062	Frame_Shift_Ins	INS	-	AAGATGATTTGA	-	ETV1	5insAATCAAATC	p.Q35fs
XH_P35	7	38304989	38304989	Missense_Mutation	SNP	C	T	C	TARP	c.G106A	p.G36R
XH_P35	7	55496101	55496101	Missense_Mutation	SNP	G	A	G	LANCL2	c.G1219A	p.G407R
XH_P35	7	86813934	86813934	Missense_Mutation	SNP	A	C	A	DMTF1	c.A778C	p.I260L
XH_P35	7	92732231	92732231	Missense_Mutation	SNP	T	A	T	SAMD9	c.A3180T	p.K1060N
XH_P35	7	93065455	93065455	Missense_Mutation	SNP	C	T	C	CALCR	c.G958A	p.V320I
XH_P35	7	103030944	103030944	Missense_Mutation	SNP	A	G	A	SLC26A5	c.T1243C	p.C415R

XH_P35	7	104752364	104752364	Silent	SNP	C	T	C	KMT2E	c.C4161T	p.S1387S
XH_P35	7	104809669	104809669	Silent	SNP	C	T	C	SRPK2	c.G240A	p.R80R
XH_P35	7	121653375	121653380	In_Frame_Del	DEL	TGATGA	-	TGATGA	PTPRZ1	c.4275_4280del	p.1425_1427del
XH_P35	7	151164222	151164222	Missense_Mutation	SNP	A	C	A	RHEB	c.T538G	p.S180A
XH_P35	8	3076776	3076776	Splice_Site	DEL	C	-	C	CSMD1	.	.
XH_P35	8	27516792	27516792	Missense_Mutation	SNP	A	T	A	SCARA3	c.A1105T	p.N369Y
XH_P35	8	30643779	30643779	Frame_Shift_Del	DEL	G	-	G	PPP2CB	c.902delC	p.T301fs
XH_P35	8	69129947	69129947	Silent	SNP	G	A	G	PREX2	c.G4701A	p.R1567R
XH_P35	8	74464496	74464496	Silent	SNP	G	C	G	STAU2	c.C765G	p.R255R
XH_P35	8	94747621	94747621	Missense_Mutation	SNP	C	T	C	RBM12B	c.G1018A	p.A340T
XH_P35	8	103573011	103573037	In_Frame_Del	DEL	TGCAGCCCC	-	CCTGCAGCCCCTG	ODF1	c.652_678del	p.218_226del
XH_P35	8	110257638	110257638	Missense_Mutation	SNP	G	A	G	NUDCD1	c.C1285T	p.R429C
XH_P35	8	124714887	124714887	Missense_Mutation	SNP	C	T	C	ANXA13	c.G181A	p.G61S
XH_P35	8	142160950	142160950	Silent	SNP	G	A	G	DENND3	c.G513A	p.S171S
XH_P35	8	144681585	144681585	Silent	SNP	C	T	C	TIGD5	c.C1512T	p.A504A
XH_P35	8	145948513	145948513	Missense_Mutation	SNP	C	T	C	ZNF251	c.G532A	p.E178K
XH_P35	9	14823321	14823321	Missense_Mutation	SNP	G	A	G	FREM1	c.C2174T	p.A725V
XH_P35	9	26920224	26920224	Splice_Site	SNP	C	T	C	PLAA	.	.
XH_P35	9	69421983	69421983	Missense_Mutation	SNP	G	C	G	ANKRD20A4	c.G1447C	p.V483L
XH_P35	9	84199393	84199393	Nonsense_Mutation	SNP	C	T	C	TLE1	c.G2205A	p.W735X
XH_P35	9	107593319	107593319	Silent	SNP	G	A	G	ABCA1	c.C1779T	p.F593F
XH_P35	9	120474711	120474711	Missense_Mutation	SNP	G	T	G	TLR4	c.G305T	p.S102I
XH_P35	9	125486479	125486479	Missense_Mutation	SNP	G	A	G	OR1L4	c.G211A	p.D71N
XH_P35	9	140120136	140120136	Silent	SNP	C	T	C	CYSRT1	c.C183T	p.H61H
XH_P35	10	13178833	13178833	Silent	SNP	G	A	G	OPTN	c.G1701A	p.T567T
XH_P35	10	13213025	13213025	Silent	SNP	C	T	C	MCM10	c.C111T	p.G37G
XH_P35	10	14976739	14976739	Missense_Mutation	SNP	G	A	G	DCLRE1C	c.C155T	p.T52M
XH_P35	10	63851876	63851876	Missense_Mutation	SNP	C	T	C	ARID5B	c.C1925T	p.T642M
XH_P35	10	71129079	71129079	Silent	SNP	C	T	C	HK1	c.C582T	p.V194V
XH_P35	10	73553306	73553306	Silent	SNP	A	G	A	CDH23	c.A6621G	p.L2207L
XH_P35	10	75397633	75397633	Missense_Mutation	SNP	G	A	G	MYOZ1	c.C121T	p.P41S
XH_P35	10	78647070	78647070	Nonsense_Mutation	INS	-	TTTTACACATATT	-	KCNMA1	ATGCAAATATGT	4_E1115delinsICKYVX
XH_P35	10	88485912	88485912	Missense_Mutation	SNP	G	A	G	LDB3	c.G1667A	p.S556N
XH_P35	10	90768673	90768673	Missense_Mutation	SNP	G	A	G	FAS	c.G362A	p.R121Q
XH_P35	10	105218254	105218254	Silent	SNP	C	T	C	CALHM1	c.G255A	p.P85P
XH_P35	10	105971864	105971864	Silent	SNP	G	A	G	CFAP43	c.C636T	p.V212V
XH_P35	10	120488899	120488899	Frame_Shift_Ins	INS	-	CTTGTTAATTTG	-	CACUL1	CATAATCAAATT	p.K169fs
XH_P35	10	124159904	124159904	Missense_Mutation	SNP	G	A	G	PLEKHA1	c.G244A	p.V82I
XH_P35	11	320606	320606	Missense_Mutation	SNP	G	T	G	IFITM3	c.C208A	p.P70T
XH_P35	11	320649	320649	Silent	SNP	G	A	G	IFITM3	c.C165T	p.P55P
XH_P35	11	824776	824776	Missense_Mutation	SNP	C	T	C	PNPLA2	c.C1429T	p.P477S
XH_P35	11	1009180	1009180	Missense_Mutation	SNP	C	G	C	AP2A2	c.C2504G	p.S835C
XH_P35	11	1016649	1016649	Missense_Mutation	SNP	G	A	G	MUC6	c.C6152T	p.T2051M
XH_P35	11	1268481	1268481	Silent	SNP	G	A	G	MUC5B	c.G10371A	p.R3457R
XH_P35	11	6587042	6587042	In_Frame_Ins	INS	-	GCTCTTCTT	-	DNHD1	4_10875insGCT	.E3625delinsELFL
XH_P35	11	6587044	6587044	Missense_Mutation	SNP	A	G	A	DNHD1	c.A10876G	p.R3626G
XH_P35	11	10794111	10794111	Missense_Mutation	SNP	G	A	G	CTR9	c.G2417A	p.R806Q
XH_P35	11	33370147	33370147	Missense_Mutation	SNP	G	A	G	HIPK3	c.G2386A	p.E796K
XH_P35	11	59982088	59982088	Splice_Site	INS	-	TGATTAATCCTT	-	MS4A4E	.	.
XH_P35	11	62558120	62558120	Missense_Mutation	SNP	G	A	G	TMEM223	c.C584T	p.T195I
XH_P35	11	62644260	62644260	Silent	SNP	A	G	A	SLC3A2	c.A210G	p.E70E
XH_P35	11	63964966	63964966	Silent	SNP	G	A	G	STIP1	c.G942A	p.A314A
XH_P35	11	66235641	66235641	Silent	SNP	C	T	C	PELI3	c.C42T	p.T14T
XH_P35	11	66294233	66294233	Missense_Mutation	SNP	C	T	C	BBS1	c.C1294T	p.R432W
XH_P35	11	66807407	66807407	Silent	SNP	G	A	G	SYT12	c.G9A	p.R3R
XH_P35	11	69462910	69462910	Silent	SNP	G	A	G	CCND1	c.G723A	p.P241P
XH_P35	11	102398377	102398377	Missense_Mutation	SNP	G	A	G	MMP7	c.C362T	p.P121L
XH_P35	11	118827703	118827703	Missense_Mutation	SNP	C	T	C	UPK2	c.C187T	p.R63W
XH_P35	12	266790	266790	Silent	SNP	C	T	C	IQSEC3	c.C1464T	p.T488T
XH_P35	12	8374812	8374812	Missense_Mutation	SNP	G	A	G	FAM90A1	c.C1001T	p.P334L
XH_P35	12	20852524	20852524	Missense_Mutation	SNP	C	T	C	SLCO1C1	c.C14T	p.S5F
XH_P35	12	40808847	40808847	Splice_Site	SNP	G	A	G	MUC19	.	.
XH_P35	12	50036051	50036051	Missense_Mutation	SNP	C	T	C	PRPF40B	c.C1813T	p.R605W
XH_P35	12	54645964	54645964	Missense_Mutation	SNP	A	G	A	CBX5	c.T185C	p.L62P
XH_P35	12	54645965	54645965	Frame_Shift_Ins	INS	-	CTCTTGTTACTT	-	CBX5	TCAGAAAGTAAC	p.L62fs
XH_P35	12	76424934	76424934	Frame_Shift_Del	DEL	C	-	C	PHLDA1	c.588delG	p.Q196fs
XH_P35	12	76424936	76424940	Frame_Shift_Del	DEL	GTTGC	-	GTTGC	PHLDA1	c.582_586del	p.Q194fs
XH_P35	12	77244716	77244716	Nonsense_Mutation	INS	-	CAAATGATT	-	ZDHHC17	0_1851insCAAA	p.Y617delinsYKX
XH_P35	12	77244718	77244718	Nonsense_Mutation	INS	-	ATACATGAATT	-	ZDHHC17	_1853insATACA	618_I619delinsNTX
XH_P35	12	99117495	99117495	Missense_Mutation	SNP	C	A	C	APAF1	c.C3121A	p.H1041N
XH_P35	12	132474616	132474616	Silent	SNP	G	A	G	EP400	c.G2517A	p.R839R
XH_P35	13	33110750	33110750	Missense_Mutation	SNP	C	G	C	N4BP2L2	c.G415C	p.G139R
XH_P35	13	39448643	39448643	Missense_Mutation	SNP	G	A	G	FREM2	c.G8201A	p.R2734H
XH_P35	13	58208588	58208588	Missense_Mutation	SNP	T	G	T	PCDH17	c.T1908G	p.F636L
XH_P35	13	113201936	113201936	Missense_Mutation	SNP	T	C	T	TUBGCP3	c.A1136G	p.Q379R
XH_P35	14	19553724	19553724	Missense_Mutation	SNP	G	C	G	POTEG	c.G308C	p.C103S
XH_P35	14	20019912	20019912	Silent	SNP	G	A	G	POTEM	c.C309T	p.C103C
XH_P35	14	20019913	20019913	Missense_Mutation	SNP	C	G	C	POTEM	c.G308C	p.C103S
XH_P35	14	23612372	23612372	Missense_Mutation	SNP	T	G	T	SLC7A8	c.A235C	p.T79P
XH_P35	14	24774897	24774897	Missense_Mutation	SNP	C	T	C	CIDEB	c.G602A	p.R201H
XH_P35	14	65415128	65415128	Missense_Mutation	SNP	C	T	C	RAB15	c.G583A	p.E195K
XH_P35	14	72818827	72818827	Missense_Mutation	SNP	C	A	C	RGS6	c.C4A	p.Q2K
XH_P35	14	88477678	88477678	Missense_Mutation	SNP	G	A	G	GPR65	c.G487A	p.E163K
XH_P35	15	28951789	28951789	Missense_Mutation	SNP	C	G	C	GOLGA8M	c.G648C	p.E216D
XH_P35	15	29346574	29346574	Missense_Mutation	SNP	G	A	G	APBA2	c.G487A	p.G163S
XH_P35	15	37188865	37188865	Missense_Mutation	SNP	T	A	T	MEIS2	c.A961T	p.I321L
XH_P35	15	40702993	40702993	Silent	SNP	G	A	G	IVD	c.G372A	p.P124P
XH_P35	15	58936158	58936158	Missense_Mutation	SNP	G	A	G	ADAM10	c.C755T	p.A252V
XH_P35	15	59963488	59963488	In_Frame_Ins	INS	-	ATCATGTAGTTT	-	BNIP2	GCAGAAAACTA	delinsLVAENYMIVFCL
XH_P35	15	64388289	64388289	Frame_Shift_Ins	INS	-	G	-	SNX1	c.78dupG	p.E26fs
XH_P35	15	66786812	66786812	Missense_Mutation	SNP	C	T	C	SNAPC5	c.G169A	p.E57K
XH_P35	15	70979983	70979983	Silent	SNP	G	A	G	UACA	c.C459T	p.D153D
XH_P35	15	73615878	73615878	Silent	SNP	C	T	C	HCN4	c.G2556A	p.P852P
XH_P35	15	75809701	75809701	In_Frame_Ins	INS	-	CTTGGGATATAT	-	PTPN9	AAATATATATAT	43delinsKKIYISQALKE
XH_P35	15	101109820	101109820	Missense_Mutation	SNP	C	T	C	LINS1	c.G1897A	p.D633N
XH_P35	16	396485	396485	Missense_Mutation	SNP	T	A	T	AXIN1	c.A541T	p.M181L
XH_P35	16	2287505	2287505	Missense_Mutation	SNP	G	C	G	DNASE1L2	c.G446C	p.R149P
XH_P35	16	12061495	12061495	Frame_Shift_Del	DEL	C	-	C	TNFRSF17	c.346delC	p.P116fs

XH_P35	16	15045759	15045829	Frame_Shift_Del	DEL	CCACCCTCAG	-	CTCCACCCTCAGT	NPIPA1	c.930_1000del	p.E310fs
XH_P35	16	31089768	31089768	Missense_Mutation	SNP	C	T	C	ZNF646	c.C2123T	p.P708L
XH_P35	16	56388958	56388958	Frame_Shift_Ins	INS	-	CATAG	-	GNAO1	058_1059insCAT	p.L353fs
XH_P35	16	57468022	57468022	Missense_Mutation	SNP	G	T	G	CIAPIN1	c.C451A	p.P151T
XH_P35	16	67289072	67289072	Silent	SNP	C	T	C	SLC9A5	c.C336T	p.N112N
XH_P35	16	70154480	70154480	Missense_Mutation	SNP	A	G	A	PDPR	c.A85G	p.T29A
XH_P35	16	70154534	70154534	Missense_Mutation	SNP	A	G	A	PDPR	c.A139G	p.I47V
XH_P35	16	72142809	72142809	Silent	SNP	G	A	G	DHX38	c.G3366A	p.L1122L
XH_P35	16	74919684	74919684	Silent	SNP	G	A	G	WDR59	c.C2556T	p.D852D
XH_P35	16	84118718	84118718	Missense_Mutation	SNP	G	A	G	MBTPS1	c.C1156T	p.R386C
XH_P35	16	87637894	87637902	In_Frame_Del	DEL	CTGCTGCTG	-	CTGCTGCTG	JPH3	c.431_439del	p.144_147del
XH_P35	17	7578490	7578490	Missense_Mutation	SNP	A	C	A	TP53	c.T44G	p.V15G
XH_P35	17	29664876	29664876	Nonsense_Mutation	SNP	C	T	C	NF1	c.C6619T	p.Q2207X
XH_P35	17	33481662	33481662	Missense_Mutation	SNP	G	A	G	UNC45B	c.G541A	p.V181I
XH_P35	17	34797610	34797610	Frame_Shift_Ins	INS	-	GGGAATTCCATG	-	TBC1D3B	CGTGGGCATGG	p.P509fs
XH_P35	17	35734873	35734873	Missense_Mutation	SNP	G	A	G	C17orf78	c.G115A	p.V39M
XH_P35	17	40673079	40673079	Missense_Mutation	SNP	G	A	G	ATP6V0A1	c.G2458A	p.G820S
XH_P35	17	40818192	40818192	Missense_Mutation	SNP	C	T	C	TUBG2	c.C931T	p.R311C
XH_P35	17	41959742	41959742	Silent	SNP	G	A	G	MPP2	c.C558T	p.P186P
XH_P35	17	42476377	42476377	Missense_Mutation	SNP	C	T	C	GPATCH8	c.G2834A	p.R945Q
XH_P35	17	48545887	48545887	Silent	SNP	G	A	G	CHAD	c.C288T	p.R96R
XH_P35	17	55918306	55918306	Missense_Mutation	SNP	C	T	C	MRPS23	c.G401A	p.R134Q
XH_P35	17	62018433	62018433	In_Frame_Ins	INS	-	GATGGCGCACAC	-	SCN4A	AGGAGGTGTGC	7delinsKHEEVCAIKIQRA
XH_P35	17	62892168	62892168	Missense_Mutation	SNP	T	G	T	LRRC37A3	c.A1208C	p.H403P
XH_P35	17	72999576	72999576	Missense_Mutation	SNP	G	A	G	CDR2L	c.G805A	p.D269N
XH_P35	18	13731869	13731869	Frame_Shift_Ins	INS	-	AAACTCTTTCTC	-	RNMT	354insAAACTCTT	p.S118fs
XH_P35	18	13731871	13731871	Missense_Mutation	SNP	A	T	A	RNMT	c.A355T	p.T119S
XH_P35	18	19345793	19345793	Missense_Mutation	SNP	G	A	G	MIB1	c.G290A	p.R97Q
XH_P35	18	77703333	77703333	Silent	SNP	A	G	A	PQLC1	c.T333C	p.F111F
XH_P35	19	746301	746301	Silent	SNP	C	T	C	PALM	c.C519T	p.D173D
XH_P35	19	3204593	3204593	Missense_Mutation	SNP	A	T	A	NCLN	c.A1052T	p.E351V
XH_P35	19	4511622	4511622	Missense_Mutation	SNP	C	T	C	PLIN4	c.G2308A	p.G770S
XH_P35	19	8038746	8038746	Missense_Mutation	SNP	G	A	G	ELAVL1	c.C293T	p.P98L
XH_P35	19	9020005	9020005	Missense_Mutation	SNP	C	T	C	MUC16	c.G37490A	p.R12497K
XH_P35	19	9063095	9063095	Silent	SNP	G	A	G	MUC16	c.C24351T	p.S8117S
XH_P35	19	10503997	10503997	Missense_Mutation	SNP	G	A	G	CDC37	c.C847T	p.R283C
XH_P35	19	19243565	19243565	Splice_Site	SNP	T	A	T	TMEM161A	.	.
XH_P35	19	19391098	19391098	Nonsense_Mutation	SNP	G	A	G	SUGP1	c.C1246T	p.Q416X
XH_P35	19	22155423	22155423	Missense_Mutation	SNP	A	T	A	ZNF208	c.T2413A	p.Y805N
XH_P35	19	22836805	22836805	Missense_Mutation	SNP	G	A	G	ZNF492	c.G118A	p.A40T
XH_P35	19	33140695	33140695	Missense_Mutation	SNP	A	C	A	ANKRD27	c.T106G	p.L36V
XH_P35	19	38573264	38573264	Silent	SNP	C	T	C	SIPA1L3	c.C1059T	p.N353N
XH_P35	19	40368493	40368493	Silent	SNP	A	G	A	FCGBP	c.T12855C	p.S4285S
XH_P35	19	42754692	42754692	Silent	SNP	G	A	G	ERF	c.C48T	p.Y16Y
XH_P35	19	49878239	49878239	Missense_Mutation	SNP	G	A	G	DKKL1	c.G590A	p.R197Q
XH_P35	19	49926531	49926536	In_Frame_Del	DEL	CAGCAG	-	CAGCAG	PTH2	c.61_66del	p.21_22del
XH_P35	19	55020285	55020285	Silent	SNP	C	A	C	LAIR2	c.C405A	p.G135G
XH_P35	19	55106288	55106288	Missense_Mutation	SNP	A	C	A	LILRA1	c.A229C	p.I77L
XH_P35	19	57868377	57868377	Silent	SNP	G	A	G	ZNF304	c.G1140A	p.Q380Q
XH_P35	20	16730578	16730578	Missense_Mutation	SNP	G	A	G	OTOR	c.G286A	p.V96I
XH_P35	20	25273118	25273118	Silent	SNP	C	T	C	PYGB	c.C2046T	p.F682F
XH_P35	20	49492613	49492613	Missense_Mutation	SNP	A	G	A	BCAS4	c.A569G	p.Q190R
XH_P35	21	43161669	43161669	Missense_Mutation	SNP	C	A	C	RIPK4	c.G1684T	p.D562Y
XH_P35	21	45795846	45795846	Missense_Mutation	SNP	T	C	T	TRPM2	c.T902C	p.L301P
XH_P35	21	45833958	45833958	Splice_Site	SNP	G	A	G	TRPM2	.	.
XH_P35	22	18655973	18655973	Silent	SNP	C	T	C	USP18	c.C948T	p.S316S
XH_P35	22	18655974	18655974	Frame_Shift_Ins	INS	-	GGACTTTA	-	USP18	9_950insGGACT	p.G317fs
XH_P35	22	26829896	26829896	Silent	SNP	C	T	C	ASPHD2	c.C315T	p.Y105Y
XH_P35	22	32628915	32628915	Missense_Mutation	SNP	G	A	G	SLC5A4	c.C992T	p.P331L
XH_P35	22	39267544	39267544	Missense_Mutation	SNP	C	T	C	CBX6	c.G187A	p.E63K
XH_P35	22	40718888	40718888	Silent	SNP	T	G	T	TNRC6B	c.T4815G	p.A1605A
XH_P35	22	50616069	50616069	Missense_Mutation	SNP	A	C	A	PANX2	c.A928C	p.I310L
XH_P35	X	69828947	69828947	Nonsense_Mutation	INS	-	AAAATGGAAAT	-	TEX11	TATAACTATTTC	5_T626delinsYITISILX
XH_P35	X	129299559	129299559	Silent	SNP	G	A	G	AIFM1	c.C72T	p.C24C
XH_P35	X	153296440	153296440	Missense_Mutation	SNP	G	A	G	MECP2	c.C875T	p.A292V
XH_P35	1	155011989	155011989	Missense_Mutation	SNV	T	A	A	DCST1	c.298T>A	P.L100M
XH_P35	1	156894457	156894457	Silent	SNV	T	A	A	LRRC71	c.462T>A	P.I154I
XH_P35	1	17550872	17550872	Silent	SNV	T	G	G	PADI1	c.357T>G	P.L119L
XH_P35	1	201181672	201181672	Missense_Mutation	SNV	C	T	T	IGFN1	c.7651C>T	P.R2551W
XH_P35	1	201185848	201185848	Missense_Mutation	SNV	C	A	A	IGFN1	c.9562C>A	P.L3188M
XH_P35	1	248801977	248801977	Missense_Mutation	SNV	A	T	T	OR2T35	c.583T>A	P.Y195N
XH_P35	1	27105745	27105745	Nonsense_Mutation	SNV	G	T	T	ARID1A	c.5356G>T	P.E1786*
XH_P35	10	124159904	124159904	Missense_Mutation	SNV	G	A	A	PLEKHA1	c.244G>A	P.V82I
XH_P35	10	63851876	63851876	Missense_Mutation	SNV	C	T	T	ARID5B	c.1925C>T	P.T642M
XH_P35	10	73553306	73553306	Silent	SNV	A	G	G	CDH23	c.6621A>G	P.L2207L
XH_P35	11	1009180	1009180	Missense_Mutation	SNV	C	G	G	AP2A2	c.2504C>G	P.S835C
XH_P35	11	10794111	10794111	Missense_Mutation	SNV	G	A	A	CTR9	c.2489G>A	P.R830Q
XH_P35	11	124792272	124792272	Splice_Site	SNV	A	T	T	HEPACAM	c.948+12T>A	
XH_P35	12	124414204	124414204	Splice_Site	SNV	T	C	C	DNAH10	c.12163-7T>C	
XH_P35	12	40808847	40808847	Splice_Site	SNV	G	A	A	MUC19	c.914-1G>A	
XH_P35	12	50036051	50036051	Missense_Mutation	SNV	C	T	T	PRPF40B	c.1918C>T	P.R640W
XH_P35	12	76424936	76424940	Frame_Shift_Del	INDEL	GTTGC	-	-	PHLDA1	582_586delGCA	P.Q195fs
XH_P35	12	9230290	9230290	Splice_Site	SNV	G	A	A	A2M	c.3276+7C>T	
XH_P35	12	99117495	99117495	Missense_Mutation	SNV	C	A	A	APAF1	c.3121C>A	P.H1041N
XH_P35	13	33110750	33110750	Missense_Mutation	SNV	C	G	G	N4BP2L2	c.415G>C	P.G139R
XH_P35	13	39448643	39448643	Missense_Mutation	SNV	G	A	A	FREM2	c.8201G>A	P.R2734H
XH_P35	13	58208588	58208588	Missense_Mutation	SNV	T	G	G	PCDH17	c.1908T>G	P.F636L
XH_P35	14	23612372	23612372	Missense_Mutation	SNV	T	G	G	SLC7A8	c.235A>C	P.T79P
XH_P35	14	24774897	24774897	Missense_Mutation	SNV	C	T	T	CIDEB	c.602G>A	P.R201H
XH_P35	14	65415128	65415128	Missense_Mutation	SNV	C	T	T	RAB15	c.583G>A	P.E195K
XH_P35	14	72818827	72818827	Missense_Mutation	SNV	C	A	A	RGS6	c.109C>A	P.Q37K
XH_P35	14	73822324	73822324	Splice_Site	SNV	C	T	T	NUMB	c.126+10G>A	
XH_P35	14	88477678	88477678	Missense_Mutation	SNV	G	A	A	GPR65	c.487G>A	P.E163K
XH_P35	15	101109820	101109820	Missense_Mutation	SNV	C	T	T	LINS1	c.1897G>A	P.D633N
XH_P35	15	28951789	28951789	Missense_Mutation	SNV	C	G	G	GOLGA8M	c.648G>C	P.E216D
XH_P35	15	37188865	37188865	Missense_Mutation	SNV	T	A	A	MEIS2	c.1000A>T	P.I334L
XH_P35	15	64388289	64388289	Frame_Shift_Ins	INDEL	-	G	G	SNX1	c.82dupG	P.A28fs
XH_P35	16	12061495	12061495	Frame_Shift_Del	INDEL	C	-	-	TNFRSF17	c.347delC	P.P116fs

XH_P35	16	396485	396485	Missense_Mutation	SNV	T	A	A	AXIN1	c.541A>T	P.M181L
XH_P35	16	57468022	57468022	Missense_Mutation	SNV	G	T	T	CIAPIN1	c.451C>A	P.P151T
XH_P35	16	72142809	72142809	Silent	SNV	G	A	A	DHX38	c.3366G>A	P.L1122L
XH_P35	16	84118718	84118718	Missense_Mutation	SNV	G	A	A	MBTPS1	c.1156C>T	P.R386C
XH_P35	17	29664876	29664876	Nonsense_Mutation	SNV	C	T	T	NF1	c.6682C>T	P.Q2228*
XH_P35	17	41959742	41959742	Silent	SNV	G	A	A	MPP2	c.726C>T	P.P242P
XH_P35	17	62892168	62892168	Missense_Mutation	SNV	T	G	G	LRRC37A3	c.1208A>C	P.H403P
XH_P35	17	7578490	7578490	Missense_Mutation	SNV	A	C	C	TP53	c.440T>G	P.V147G
XH_P35	18	77703333	77703333	Silent	SNV	A	G	G	PQLC1	c.333T>C	P.F111F
XH_P35	19	1074092	1074092	Splice_Site	SNV	G	A	A	ARHGAP45	c.839-11G>A	
XH_P35	19	19243565	19243565	Splice_Site	SNV	T	A	A	TMEM161A	c.189-2A>T	
XH_P35	19	19391098	19391098	Nonsense_Mutation	SNV	G	A	A	SUGP1	c.1246C>T	P.Q416*
XH_P35	19	3204593	3204593	Missense_Mutation	SNV	A	T	T	NCLN	c.1052A>T	P.E351V
XH_P35	19	55020285	55020285	Silent	SNV	C	A	A	LAIR2	c.405C>A	P.G135G
XH_P35	2	110849289	110849289	Missense_Mutation	SNV	A	G	G	MALL	c.164T>C	P.L55P
XH_P35	2	114713283	114713283	Splice_Site	SNV	G	A	A	ACTR3	c.1008G>A	P.T336T
XH_P35	2	160709792	160709792	Nonsense_Mutation	SNV	C	T	T	LY75-CD302	c.2735G>A	P.W912*
XH_P35	2	178762781	178762781	Splice_Site	SNV	T	A	A	PDE11A	c.1302+4A>T	
XH_P35	2	190428890	190428890	Missense_Mutation	SNV	A	C	C	SLC40A1	c.822T>G	P.H274Q
XH_P35	2	209113113	209113113	Missense_Mutation	SNV	G	C	C	IDH1	c.394C>G	P.R132G
XH_P35	2	242009366	242009366	Silent	SNV	C	T	T	SNED1	c.3339C>T	P.A1113A
XH_P35	2	55214622	55214622	Splice_Site	SNV	C	A	A	RTN4	c.2603+5G>T	
XH_P35	2	73675421	73675421	Missense_Mutation	SNV	G	T	T	ALMS1	c.1764G>T	P.L588F
XH_P35	2	97818241	97818241	Missense_Mutation	SNV	T	G	G	ANKRD36	c.1163T>G	P.V388G
XH_P35	20	5169706	5169706	Splice_Site	SNV	T	G	G	CDS2	c.982-7T>G	
XH_P35	21	43161669	43161669	Missense_Mutation	SNV	C	A	A	RIPK4	c.1684G>T	P.D562Y
XH_P35	21	45795846	45795846	Missense_Mutation	SNV	T	C	C	TRPM2	c.902T>C	P.L301P
XH_P35	21	45833958	45833958	Splice_Site	SNV	G	A	A	TRPM2	c.3146+1G>A	
XH_P35	22	39267544	39267544	Missense_Mutation	SNV	C	T	T	CBX6	c.187G>A	P.E63K
XH_P35	22	40718888	40718888	Silent	SNV	T	G	G	TNRC6B	c.2733T>G	P.A911A
XH_P35	22	50616069	50616069	Missense_Mutation	SNV	A	C	C	PANX2	c.928A>C	P.I310L
XH_P35	3	108776269	108776269	Missense_Mutation	SNV	C	T	T	MORC1	c.1096G>A	P.A366T
XH_P35	3	138189876	138189876	Splice_Site	SNV	T	C	C	ESYT3	c.1740+8T>C	
XH_P35	3	172064234	172064234	Splice_Site	SNV	A	C	C	FNDC3B	c.2364+4A>C	
XH_P35	3	178936083	178936083	Missense_Mutation	SNV	A	T	T	PIK3CA	c.1625A>T	P.E542V
XH_P35	3	179046111	179046111	Silent	SNV	G	A	A	ZNF639	c.30G>A	P.R10R
XH_P35	3	30686325	30686325	Missense_Mutation	SNV	T	C	C	TGFBR2	c.256T>C	P.C86R
XH_P35	3	50683187	50683187	Missense_Mutation	SNV	G	T	T	MAPKAPK3	c.675G>T	P.M225I
XH_P35	3	52325942	52325942	Splice_Site	SNV	T	G	G	GLYCTK	n.801+4T>G	
XH_P35	3	69244235	69244235	Missense_Mutation	SNV	G	C	C	FRMD4B	c.1426C>G	P.Q476E
XH_P35	4	154330177	154330177	Splice_Site	SNV	A	T	T	MND1	c.*27+10A>T	
XH_P35	4	15689835	15689835	Missense_Mutation	SNV	A	C	C	FAM200B	c.1235A>C	P.K412T
XH_P35	4	2176479	2176479	Splice_Site	SNV	C	T	T	POLN	c.1249-1G>A	
XH_P35	4	9784705	9784705	Missense_Mutation	SNV	A	C	C	DRD5	c.1052A>C	P.N351T
XH_P35	5	122161755	122161755	Missense_Mutation	SNV	A	G	G	SNX2	c.872A>G	P.D291G
XH_P35	5	149588999	149588999	Missense_Mutation	SNV	G	A	A	SLC6A7	c.1732G>A	P.D578N
XH_P35	5	32712262	32712262	Missense_Mutation	SNV	A	T	T	NPR3	c.380A>T	P.K127M
XH_P35	5	65105431	65105431	Silent	SNV	C	T	T	NLN	c.1617C>T	P.V539V
XH_P35	5	7866894	7866894	Missense_Mutation	SNV	C	T	T	FASTKD3	c.1303G>A	P.V435M
XH_P35	5	78985887	78985887	Splice_Site	SNV	G	A	A	CMYA5	c.149+8G>A	
XH_P35	6	40999846	40999846	Missense_Mutation	SNV	T	A	A	UNC5CL	c.980A>T	P.Q327L
XH_P35	7	103030944	103030944	Missense_Mutation	SNV	A	G	G	SLC26A5	c.1243T>C	P.C415R
XH_P35	7	104752364	104752364	Silent	SNV	C	T	T	KMT2E	c.4161C>T	P.S1387S
XH_P35	7	151164222	151164222	Missense_Mutation	SNV	A	C	C	RHEB	c.538T>G	P.S180A
XH_P35	7	86813934	86813934	Missense_Mutation	SNV	A	C	C	DMTF1	c.778A>C	P.I260L
XH_P35	7	93065455	93065455	Missense_Mutation	SNV	C	T	T	CALCR	c.1060G>A	P.V354I
XH_P35	8	144681585	144681585	Silent	SNV	C	T	T	TIGD5	c.1512C>T	P.A504A
XH_P35	8	144695915	144695915	Splice_Site	SNV	A	G	G	TSTA3	c.748+6T>C	
XH_P35	8	27516792	27516792	Missense_Mutation	SNV	A	T	T	SCARA3	c.1105A>T	P.N369Y
XH_P35	8	3076776	3076776	Splice_Site	INDEL	C	-	-	CSMD1	c.4672+1delG	
XH_P35	8	74464496	74464496	Silent	SNV	G	C	C	STAU2	c.1281C>G	P.R427R
XH_P35	9	120474711	120474711	Missense_Mutation	SNV	G	T	T	TLR4	c.185G>T	P.S62I
XH_P35	9	125486479	125486479	Missense_Mutation	SNV	G	A	A	OR1L4	c.211G>A	P.D71N
XH_P35	9	131767814	131767814	Splice_Site	SNV	T	G	G	NUP188	c.4737+5T>G	
XH_P35	9	140120136	140120136	Silent	SNV	C	T	T	CYSRT1	c.183C>T	P.H61H
XH_P35	9	79234308	79234308	Splice_Site	SNV	G	C	C	PRUNE2	c.936-5C>G	
XH_P35	9	84199393	84199393	Nonsense_Mutation	SNV	C	T	T	TLE1	c.2205G>A	P.W735*
XH_P36	1	874456	874456	Missense_Mutation	SNP	G	A	G	SAMD11	c.G467A	p.R156H
XH_P36	1	900461	900461	Missense_Mutation	SNP	G	A	G	KLHL17	c.G1819A	p.A607T
XH_P36	1	1168240	1168240	Frame_Shift_Del	DEL	G	-	G	B3GALT6	c.582delG	p.P194fs
XH_P36	1	1268888	1268888	Missense_Mutation	SNP	G	A	G	TAS1R3	c.G1603A	p.D535N
XH_P36	1	1534720	1534720	Missense_Mutation	SNP	G	A	G	FNDC10	c.C676T	p.P226S
XH_P36	1	1684346	1684346	In_Frame_Ins	INS	-	CCC	-	NADK	1241_1242insG(	p.G414delinsGG
XH_P36	1	2238077	2238077	Missense_Mutation	SNP	A	G	A	SKI	c.A2060G	p.D687G
XH_P36	1	2290124	2290124	Missense_Mutation	SNP	G	A	G	MORN1	c.C776T	p.A259V
XH_P36	1	2572878	2572878	Missense_Mutation	SNP	C	T	C	TTC34	c.G1630A	p.D544N
XH_P36	1	3541774	3541774	Silent	SNP	C	T	C	TPRG1L	c.C138T	p.L46L
XH_P36	1	3742991	3742991	Nonsense_Mutation	INS	-	TAACTTAAAATA	-	CEP104	AAAGGATATTTT	G733delinsAERIFX
XH_P36	1	5937158	5937158	Missense_Mutation	SNP	C	T	C	NPHP4	c.G1276A	p.V426M
XH_P36	1	11082583	11082583	Frame_Shift_Ins	INS	-	CTATTGCTATTG	-	TARDBP	18insGTCTATTG	p.S373fs
XH_P36	1	12919891	12919891	Missense_Mutation	SNP	G	C	G	PRAMEF2	c.G631C	p.E211Q
XH_P36	1	17085996	17085996	Frame_Shift_Del	DEL	C	-	C	MST1L	c.901delG	p.A301fs
XH_P36	1	19180874	19180874	Missense_Mutation	SNP	C	T	C	TAS1R2	c.G1090A	p.D364N
XH_P36	1	19183876	19183876	Silent	SNP	G	T	G	TAS1R2	c.C432A	p.S144S
XH_P36	1	19566360	19566360	Silent	SNP	C	T	C	EMC1	c.G840A	p.L280L
XH_P36	1	20233009	20233009	Frame_Shift_Ins	INS	-	TACCTGGGTCC	-	OTUD3	_921insTACCTG(	p.I307fs
XH_P36	1	21267982	21267982	Splice_Site	SNP	A	G	A	EIF4G3	.	.
XH_P36	1	21806606	21806606	Missense_Mutation	SNP	T	G	T	NBPF3	c.T1061G	p.F354C
XH_P36	1	22923907	22923907	Missense_Mutation	SNP	G	A	G	EPHA8	c.G1868A	p.G623D
XH_P36	1	23240246	23240246	Frame_Shift_Del	DEL	A	-	A	EPHB2	c.3051delA	p.G1017fs
XH_P36	1	25889626	25889626	Frame_Shift_Ins	INS	-	C	-	LDLRAP1	c.599dupC	p.T200fs
XH_P36	1	27105931	27105931	Frame_Shift_Del	DEL	G	-	G	ARID1A	c.5542delG	p.G1848fs
XH_P36	1	27878528	27878528	Frame_Shift_Del	DEL	G	-	G	AHDC1	c.99delC	p.P33fs
XH_P36	1	28785730	28785730	Frame_Shift_Del	DEL	A	-	A	PHACTR4	c.181delA	p.K61fs
XH_P36	1	32208473	32208473	Silent	SNP	C	T	C	ADGRB2	c.G1218A	p.L406L
XH_P36	1	32800540	32800540	Frame_Shift_Del	DEL	G	-	G	MARCKSL1	c.246delC	p.P82fs
XH_P36	1	33059325	33059325	Frame_Shift_Ins	INS	-	GGTTGTT	-	ZBTB8A	93_794insGGTT(	p.G265fs
XH_P36	1	33059327	33059327	Frame_Shift_Ins	INS	-	GTATTCT	-	ZBTB8A	95_796insGTAT	p.G265fs

XH_P36	1	33060810	33060810	Nonsense_Mutation	INS	-	AATTTTATTAAGA	-	ZBTB8A	AAGAAATTTTAT	_H328delinsKRRKKFYX
XH_P36	1	33354802	33354802	Frame_Shift_Ins	INS	-	TG	-	HPCA	c.303_304insTG	p.L101fs
XH_P36	1	33354803	33354803	In_Frame_Ins	INS	-	GAACTC	-	HPCA	04_305insGAAC	.M102delinsRTL
XH_P36	1	34666397	34666397	Frame_Shift_Ins	INS	-	A	-	C1orf94	c.1035dupA	p.P345fs
XH_P36	1	35226919	35226919	Missense_Mutation	SNP	C	T	C	GJB4	c.C64T	p.R22C
XH_P36	1	35334661	35334661	Missense_Mutation	SNP	A	G	A	DLGAP3	c.T2030C	p.V677A
XH_P36	1	35370730	35370730	Frame_Shift_Ins	INS	-	C	-	DLGAP3	c.254dupG	p.G85fs
XH_P36	1	36030900	36030900	Missense_Mutation	SNP	C	T	C	NCDN	c.C1775T	p.A592V
XH_P36	1	36367904	36367904	Missense_Mutation	SNP	G	A	G	AGO1	c.G1138A	p.A380T
XH_P36	1	36380979	36380979	Nonsense_Mutation	SNP	C	T	C	AGO1	c.C1639T	p.R547X
XH_P36	1	36769566	36769566	In_Frame_Ins	INS	-	TTCACTCTGGCA	-	THRAP3	ATCTTGTTCACTC	elinsSSGHILFTLALMAIFF
XH_P36	1	38265467	38265467	Missense_Mutation	SNP	G	A	G	MANEAL	c.G300A	p.M100I
XH_P36	1	39893937	39893937	Nonsense_Mutation	INS	-	TGAGTCTATCTT	-	MACF1	0596insATGAGT	.H3532delinsQX
XH_P36	1	40775989	40775989	In_Frame_Ins	INS	-	TGGATT	-	COL9A2	84_685insAATC	.G229delinsNPG
XH_P36	1	42047087	42047087	Missense_Mutation	SNP	C	T	C	HIVEP3	c.G3382A	p.V1128I
XH_P36	1	43895417	43895417	Missense_Mutation	SNP	C	T	C	SZT2	c.C4039T	p.R1347C
XH_P36	1	44466534	44466534	Missense_Mutation	SNP	G	A	G	SLC6A9	c.C1246T	p.R416W
XH_P36	1	45267397	45267397	Missense_Mutation	SNP	G	A	G	PLK3	c.G539A	p.G180D
XH_P36	1	45407182	45407182	Frame_Shift_Del	DEL	T	-	T	EIF2B3	c.450delA	p.K150fs
XH_P36	1	46812736	46812736	In_Frame_Ins	INS	-	TTATTCACTTATT	-	NSUN4	CTTACTTATTCA	5delinsTHTYLFTYSFIY
XH_P36	1	47748086	47748086	Silent	SNP	G	A	G	STIL	c.C1179T	p.H393H
XH_P36	1	48917261	48917261	Silent	SNP	G	A	G	SPATA6	c.C201T	p.D67D
XH_P36	1	50666631	50666631	Silent	SNP	C	T	C	ELAVL4	c.C858T	p.S286S
XH_P36	1	50941243	50941243	Missense_Mutation	SNP	G	A	G	FAF1	c.C1762T	p.R588W
XH_P36	1	52825774	52825774	Missense_Mutation	SNP	G	C	G	CC2D1B	c.C735G	p.D245E
XH_P36	1	52891139	52891139	Frame_Shift_Ins	INS	-	AAAACAAAATC	-	ZCCHC11	_4752insGATTT	p.E1584fs
XH_P36	1	53446142	53446142	Nonsense_Mutation	INS	-	CATATATTAAATT	-	SCP2	insCACATATATT	D256delinsDHIYX
XH_P36	1	55080513	55080513	Frame_Shift_Ins	INS	-	CCTCTTCCTCCA	-	FAM151A	35insTTGGAGG	p.K145fs
XH_P36	1	55614239	55614239	Nonsense_Mutation	INS	-	TTT	-	USP24	1864_1865insA	p.S622delinsX
XH_P36	1	55614240	55614240	Frame_Shift_Ins	INS	-	TCACTATCTTTGT	-	USP24	nsATAAACAAAG	p.S622fs
XH_P36	1	57351827	57351827	Frame_Shift_Ins	INS	-	AGAGAGCATATA	-	C8A	AACAAAGAGAG	p.K361fs
XH_P36	1	57378171	57378171	Missense_Mutation	SNP	G	T	G	C8A	c.G1476T	p.M492I
XH_P36	1	57602268	57602268	Frame_Shift_Ins	INS	-	T	-	DAB1	c.253dupA	p.I85fs
XH_P36	1	62675863	62675863	In_Frame_Ins	INS	-	AGCAACTTTTAC	-	L1TD1	nsGCTTAGCAAC	473delinsGLATFTSL
XH_P36	1	62676780	62676780	Frame_Shift_Ins	INS	-	CTCTTTGCCTTTC	-	L1TD1	CTATATCTCTTTC	p.R778fs
XH_P36	1	63876949	63876949	Frame_Shift_Ins	INS	-	T	-	ALG6	c.628dupT	p.P209fs
XH_P36	1	65147002	65147002	Silent	SNP	C	T	C	CACHD1	c.C2580T	p.H860H
XH_P36	1	66833535	66833535	Splice_Site	SNP	T	C	T	PDE4B	.	.
XH_P36	1	67147843	67147843	Missense_Mutation	SNP	A	G	A	SGIP1	c.A1118G	p.N373S
XH_P36	1	67292634	67292634	Nonsense_Mutation	INS	-	AGAGGGTATTGT	-	WDR78	GTAACTACAATA	p.N736delinsKX
XH_P36	1	68624857	68624857	In_Frame_Ins	INS	-	TTTCCT	-	WLS	79_180insAGGA	p.M60delinsIGK
XH_P36	1	68624858	68624858	Frame_Shift_Ins	INS	-	ACTTTGCT	-	WLS	8_179insAGCAA	p.M60fs
XH_P36	1	68659747	68659747	Silent	SNP	G	A	G	WLS	c.C264T	p.I88I
XH_P36	1	70715677	70715677	Frame_Shift_Ins	INS	-	A	-	SRSF11	c.1066dupA	p.P355fs
XH_P36	1	75065510	75065510	Frame_Shift_Ins	INS	-	GTATGTTTTCTAA	-	ERICH3	595insTTAGAA	p.D532fs
XH_P36	1	75693468	75693468	Missense_Mutation	SNP	C	A	C	SLC44A5	c.G910T	p.G304W
XH_P36	1	78207315	78207315	Frame_Shift_Ins	INS	-	ATTAAACTTGCT	-	USP33	8insAAGCAAGT	p.I23fs
XH_P36	1	78207317	78207317	Silent	SNP	C	T	C	USP33	c.G66A	p.L22L
XH_P36	1	78309060	78309060	Missense_Mutation	SNP	G	A	G	MIGA1	c.G964A	p.A322T
XH_P36	1	78433883	78433883	Silent	SNP	T	C	T	FUBP1	c.A216G	p.Q72Q
XH_P36	1	79095563	79095563	Missense_Mutation	SNP	C	T	C	IFI44L	c.C686T	p.A229V
XH_P36	1	79116059	79116059	Frame_Shift_Ins	INS	-	ATTAAATATTAA	-	IFI44	TTTTCCATTAAAT	p.I60fs
XH_P36	1	79470864	79470864	In_Frame_Ins	INS	-	TATTAAAGATTT	-	ADGRL4	insGGAAATCTT	.N21delinsKEIFNI
XH_P36	1	86047153	86047153	Missense_Mutation	SNP	G	T	G	CYR61	c.G169T	p.A57S
XH_P36	1	86591275	86591275	Silent	SNP	G	A	G	COL24A1	c.C744T	p.Y248Y
XH_P36	1	90179646	90179646	Frame_Shift_Ins	INS	-	C	-	LRRC8C	c.1518dupC	p.L506fs
XH_P36	1	91967357	91967357	Frame_Shift_Del	DEL	A	-	A	CDC7	c.84delA	p.L28fs
XH_P36	1	91978751	91978751	Nonsense_Mutation	INS	-	GATCAATTAGGA	-	CDC7	TTGGGATCAATT	P237delinsLFGINX
XH_P36	1	92642755	92642755	Frame_Shift_Ins	INS	-	TTTATTTATACTA	-	KIAA1107	insAGTTTTATTTA	p.A156fs
XH_P36	1	92642757	92642757	Silent	SNP	A	T	A	KIAA1107	c.A468T	p.A156A
XH_P36	1	93306137	93306137	Silent	SNP	T	G	T	RPL5	c.T735G	p.A245A
XH_P36	1	93736257	93736257	Silent	SNP	T	C	T	CCDC18	c.T4236C	p.A1412A
XH_P36	1	94140186	94140186	Missense_Mutation	SNP	C	T	C	BCAR3	c.G301A	p.E101K
XH_P36	1	94502756	94502756	Missense_Mutation	SNP	G	A	G	ABCA4	c.C3758T	p.T1253M
XH_P36	1	99772074	99772074	Silent	SNP	G	A	G	PLPPR4	c.G1626A	p.T542T
XH_P36	1	101339807	101339807	Silent	SNP	C	T	C	EXTL2	c.G684A	p.R228R
XH_P36	1	109276077	109276077	Nonsense_Mutation	INS	-	AATATAAGCAAA	-	FNDC7	insAAAAATATAA	688_V689delinsEKIX
XH_P36	1	109735326	109735326	Missense_Mutation	SNP	C	A	C	KIAA1324	c.C763A	p.L255I
XH_P36	1	112269924	112269924	Frame_Shift_Ins	INS	-	C	-	FAM212B	c.559dupG	p.A187fs
XH_P36	1	115258747	115258747	Missense_Mutation	SNP	C	T	C	NRAS	c.G35A	p.G12D
XH_P36	1	117297419	117297419	In_Frame_Ins	INS	-	TTACTGAGAGTC	-	CD2	229insTTACTGA	.E76delinsELLRV
XH_P36	1	117504106	117504106	Frame_Shift_Ins	INS	-	A	-	PTGFRN	c.1455_1456insA	p.D485fs
XH_P36	1	118596651	118596651	Missense_Mutation	SNP	G	T	G	SPAG17	c.C2788A	p.P930T
XH_P36	1	144922225	144922225	Nonsense_Mutation	SNP	G	A	G	PDE4DIP	c.C1432T	p.R478X
XH_P36	1	145360624	145360624	Silent	SNP	G	A	G	NBPF19	c.G8430A	p.S2810S
XH_P36	1	148341948	148341948	Missense_Mutation	SNP	T	C	T	NBPF14	c.A625G	p.I209V
XH_P36	1	149895835	149895835	Missense_Mutation	SNP	A	T	A	SF3B4	c.T985A	p.S329T
XH_P36	1	151372604	151372604	Frame_Shift_Del	DEL	G	-	G	PSMB4	c.288delG	p.L96fs
XH_P36	1	151681548	151681548	Missense_Mutation	SNP	C	T	C	CELF3	c.G271A	p.A91T
XH_P36	1	151804203	151804203	Missense_Mutation	SNP	C	T	C	RORC	c.G38A	p.R13Q
XH_P36	1	152128948	152128948	Frame_Shift_Del	DEL	T	-	T	RPTN	c.627delA	p.K209fs
XH_P36	1	152129152	152129152	Silent	SNP	G	A	G	RPTN	c.C423T	p.D141D
XH_P36	1	152275289	152275289	Missense_Mutation	SNP	G	A	G	FLG	c.C12073T	p.P4025S
XH_P36	1	152279065	152279065	Missense_Mutation	SNP	C	T	C	FLG	c.G8297A	p.R2766H
XH_P36	1	152280274	152280274	Missense_Mutation	SNP	G	T	G	FLG	c.C7088A	p.S2363Y
XH_P36	1	152283324	152283324	Missense_Mutation	SNP	A	C	A	FLG	c.T4038G	p.H1346Q
XH_P36	1	152285188	152285188	Missense_Mutation	SNP	G	A	G	FLG	c.C2174T	p.T725I
XH_P36	1	152325759	152325759	Silent	SNP	G	A	G	FLG2	c.C4503T	p.H1501H
XH_P36	1	152327942	152327942	Missense_Mutation	SNP	T	C	T	FLG2	c.A2320G	p.S774G
XH_P36	1	152327943	152327943	Silent	SNP	G	A	G	FLG2	c.C2319T	p.H773H
XH_P36	1	152328398	152328398	Missense_Mutation	SNP	T	C	T	FLG2	c.A1864G	p.S622G
XH_P36	1	152328399	152328399	Silent	SNP	G	A	G	FLG2	c.C1863T	p.H621H
XH_P36	1	152770502	152770502	Missense_Mutation	SNP	C	T	C	LCE1D	c.C232T	p.R78C
XH_P36	1	154246391	154246391	Frame_Shift_Del	DEL	C	-	C	HAX1	c.314delC	p.S105fs
XH_P36	1	155148566	155148566	Frame_Shift_Ins	INS	-	C	-	TRIM46	c.151dupC	p.K50fs
XH_P36	1	155580268	155580268	Frame_Shift_Ins	INS	-	C	-	MSTO1	c.138dupC	p.C46fs
XH_P36	1	155638564	155638564	Nonsense_Mutation	INS	-	ACTTATATACTAG	-	YY1AP1	TAATCTAGTATA	34_A235delinsELIX
XH_P36	1	155722346	155722346	Nonsense_Mutation	SNP	G	A	G	GON4L	c.C5878T	p.R1960X

XH_P36	1	156642804	156642804	Frame_Shift_Del	DEL	G	-	G	NES	c.1176delC	p.P392fs
XH_P36	1	157095725	157095725	Nonsense_Mutation	INS	-	ACTACCTTCATTC	-	ETV3	47insAGAATGAA	149_F150delinsREX
XH_P36	1	158325766	158325766	Missense_Mutation	SNP	G	A	G	CD1E	c.G208A	p.V70I
XH_P36	1	158533299	158533299	Frame_Shift_Del	DEL	A	-	A	OR6P1	c.96delT	p.F32fs
XH_P36	1	158735706	158735706	Missense_Mutation	SNP	A	G	A	OR6N1	c.T767C	p.L256P
XH_P36	1	158985772	158985772	Missense_Mutation	SNP	G	C	G	IFI16	c.G376C	p.A126P
XH_P36	1	159284421	159284421	Nonsense_Mutation	INS	-	CTCTTCTTCTATC	-	OR10J3	9insGATAGAAG	T10_E11delinsRX
XH_P36	1	159779252	159779252	Missense_Mutation	SNP	A	G	A	FCRL6	c.A665G	p.D222G
XH_P36	1	161518234	161518234	Missense_Mutation	SNP	G	A	G	FCGR3A	c.C611T	p.P204L
XH_P36	1	161953527	161953527	Missense_Mutation	SNP	G	A	G	OLFML2B	c.C2194T	p.R732C
XH_P36	1	162731166	162731166	Nonsense_Mutation	SNP	C	T	C	DDR2	c.C1021T	p.R341X
XH_P36	1	162766447	162766447	Silent	SNP	T	G	T	HSD17B7	c.T312G	p.L104L
XH_P36	1	164769110	164769110	Nonsense_Mutation	SNP	C	T	C	PBX1	c.C685T	p.R229X
XH_P36	1	166896414	166896414	Missense_Mutation	SNP	C	T	C	ILDR2	c.G884A	p.R295H
XH_P36	1	171553156	171553156	Missense_Mutation	SNP	G	A	G	PRRC2C	c.G7465A	p.A2489T
XH_P36	1	173772575	173772575	Silent	SNP	G	A	G	CENPL	c.C489T	p.C163C
XH_P36	1	173930334	173930334	Frame_Shift_Ins	INS	-	A	-	RC3H1	c.2250dupT	p.L751fs
XH_P36	1	173930336	173930336	In_Frame_Ins	INS	-	TATGTATGTAAT	-	RC3H1	CATTACATTACA	0delinsNALHYIHTKIS
XH_P36	1	174200408	174200408	Missense_Mutation	SNP	C	T	C	RABGAP1L	c.C457T	p.R153W
XH_P36	1	175304899	175304899	Frame_Shift_Ins	INS	-	TCATTAATAGTC	-	TNR	80insGAGACTAT	p.K860fs
XH_P36	1	176659361	176659361	Silent	SNP	C	T	C	PAPPA2	c.C2226T	p.Y742Y
XH_P36	1	179887233	179887233	In_Frame_Ins	INS	-	ATTGCTCAGTTC	-	TOR1AIP1	5insTCCATTGCT	538delinsKSIAQFL
XH_P36	1	182550367	182550367	Missense_Mutation	SNP	G	A	G	RNASEL	c.C1898T	p.T633M
XH_P36	1	185278146	185278146	Nonsense_Mutation	INS	-	ATAAAATCATTT	-	IVNS1ABP	CTCCATAAATGA	90_Y91delinsAKX
XH_P36	1	197091723	197091723	Frame_Shift_Ins	INS	-	TTTCCTCCTGCAT	-	ASPM	393insATGCAGC	p.V1131fs
XH_P36	1	197128938	197128938	Nonsense_Mutation	INS	-	AAAAATATACTC	-	ZBTB41	1insTAAGAGTAT	p.E761delinsX
XH_P36	1	200569550	200569550	Missense_Mutation	SNP	C	T	C	KIF14	c.G761A	p.R254Q
XH_P36	1	201177935	201177935	Missense_Mutation	SNP	A	G	A	IGFN1	c.A3914G	p.D1305G
XH_P36	1	201178708	201178708	Missense_Mutation	SNP	A	G	A	IGFN1	c.A4687G	p.K1563E
XH_P36	1	202725607	202725607	Missense_Mutation	SNP	C	T	C	KDM5B	c.G1100A	p.R367K
XH_P36	1	202887497	202887497	Nonsense_Mutation	SNP	A	T	A	KLHL12	c.T483A	p.C161X
XH_P36	1	203668744	203668744	Missense_Mutation	SNP	G	A	G	ATP2B4	c.G548A	p.R183H
XH_P36	1	205241128	205241128	Missense_Mutation	SNP	C	T	C	TMCC2	c.C1331T	p.T444M
XH_P36	1	205589712	205589712	In_Frame_Ins	INS	-	ACTGCCTTCCCT	-	ELK4	462insAGGGAAC	S154delinsSGKAV
XH_P36	1	205696901	205696901	Nonsense_Mutation	INS	-	AACCATATTACAA	-	NUCKS1	TTATTGTAATAT	LISTSSHLFENYCNMVILQTFX
XH_P36	1	206758543	206758543	Frame_Shift_Del	DEL	C	-	C	RASSF5	c.561delC	p.R187fs
XH_P36	1	206821938	206821938	Frame_Shift_Del	DEL	G	-	G	DYRK3	c.1395delG	p.V465fs
XH_P36	1	207198372	207198372	In_Frame_Ins	INS	-	CACCAC	-	C1orf116	42_143insGTGG	p.K48delinsSGE
XH_P36	1	209807808	209807808	Missense_Mutation	SNP	C	T	C	LAMB3	c.G548A	p.R183H
XH_P36	1	214820283	214820283	In_Frame_Ins	INS	-	ATTTTTGCTTTTA	-	CENPF	1insTCTATTTTT	2457delinsLLFLLLG
XH_P36	1	214830519	214830519	Missense_Mutation	SNP	C	T	C	CENPF	c.C8729T	p.P2910L
XH_P36	1	220152861	220152861	Nonsense_Mutation	INS	-	TGTGTGTATTATT	-	EPRS	sAAATAAATAAT	270_N1271delinsKX
XH_P36	1	222798193	222798193	Frame_Shift_Ins	INS	-	ACTAGTGACACT	-	MIA3	2insTCACTAGTG	p.T117fs
XH_P36	1	222903458	222903458	Nonsense_Mutation	INS	-	TACCAATAATAAT	-	BROX	GCAATACCAATA	p.R278delinsTX
XH_P36	1	225490915	225490915	Missense_Mutation	SNP	C	T	C	DNAH14	c.C8410T	p.R2804C
XH_P36	1	226340081	226340081	Missense_Mutation	SNP	T	C	T	ACBD3	c.A1330G	p.S444G
XH_P36	1	226923872	226923872	Frame_Shift_Del	DEL	C	-	C	ITPKB	c.1288delG	p.A430fs
XH_P36	1	227843200	227843200	Nonsense_Mutation	INS	-	TCTTTTATGACG	-	ZNF678	ATGAATTCTTTTA	472delinsYEFFYDEX
XH_P36	1	228399732	228399732	Missense_Mutation	SNP	G	A	G	OBSCN	c.G248A	p.R83H
XH_P36	1	228506757	228506757	Silent	SNP	G	A	G	OBSCN	c.G14304A	p.S4768S
XH_P36	1	228522241	228522241	Silent	SNP	A	G	A	OBSCN	c.A16083G	p.A5361A
XH_P36	1	233464042	233464042	Missense_Mutation	SNP	G	T	G	MAP3K21	c.G268T	p.G90C
XH_P36	1	235658047	235658047	Silent	SNP	G	A	G	B3GALNT2	c.C204T	p.N68N
XH_P36	1	235940392	235940392	Missense_Mutation	SNP	T	A	T	LYST	c.A5431T	p.T1811S
XH_P36	1	237948089	237948089	Silent	SNP	C	A	C	RYR2	c.C13077A	p.P4359P
XH_P36	1	242042491	242042491	Missense_Mutation	SNP	C	T	C	EXO1	c.C1955T	p.S652L
XH_P36	1	243333027	243333027	Silent	SNP	A	G	A	CEP170	c.T1452C	p.R484R
XH_P36	1	243507615	243507615	Nonsense_Mutation	INS	-	ATTGATTCTAACA	-	SDCCAG8	162insTATTGAT	p.D387delinsDYX
XH_P36	1	245927412	245927412	Silent	SNP	T	C	T	SMYD3	c.A1116G	p.Q372Q
XH_P36	1	247007131	247007131	Frame_Shift_Del	DEL	T	-	T	AHCTF1	c.6491delA	p.N2164fs
XH_P36	1	247013524	247013524	Missense_Mutation	SNP	C	A	C	AHCTF1	c.G5784T	p.K1928N
XH_P36	1	248028086	248028086	Missense_Mutation	SNP	G	A	G	TRIM58	c.G596A	p.R199Q
XH_P36	1	248436586	248436586	Silent	SNP	G	A	G	OR2T33	c.C531T	p.C177C
XH_P36	1	248436712	248436712	Missense_Mutation	SNP	G	T	G	OR2T33	c.C405A	p.S135R
XH_P36	1	248737734	248737734	Missense_Mutation	SNP	G	A	G	OR2T34	c.C325T	p.H109Y
XH_P36	2	3750103	3750103	Missense_Mutation	SNP	C	T	C	ALLC	c.C1126T	p.R376W
XH_P36	2	10269359	10269359	Splice_Site	INS	-	TCACCCACTTTAC	-	RRM2	.	.
XH_P36	2	10269360	10269360	Splice_Site	SNP	G	T	G	RRM2	.	.
XH_P36	2	11774324	11774324	Missense_Mutation	SNP	G	A	G	GREB1	c.G5059A	p.D1687N
XH_P36	2	17698432	17698432	Frame_Shift_Ins	INS	-	CATGTGAACAAC	-	RAD51AP2	insAAGGGTTGT	p.K417fs
XH_P36	2	20234799	20234799	Frame_Shift_Ins	INS	-	ATTGAGGATTT	-	LAPTM4A	_457insAAATCC	p.L153fs
XH_P36	2	20478579	20478579	Frame_Shift_Ins	INS	-	TCACTTTCATC	-	PUM2	1554insGATGA	p.V518fs
XH_P36	2	20870507	20870507	Frame_Shift_Del	DEL	C	-	C	GDF7	c.675delC	p.R225fs
XH_P36	2	23865497	23865497	Silent	SNP	C	T	C	KLHL29	c.C717T	p.P239P
XH_P36	2	26507756	26507756	Missense_Mutation	SNP	G	T	G	HADHB	c.G1110T	p.Q370H
XH_P36	2	26705384	26705384	Frame_Shift_Del	DEL	G	-	G	OTOF	c.1469delC	p.P490fs
XH_P36	2	26951074	26951074	Frame_Shift_Del	DEL	G	-	G	KCNK3	c.823delG	p.G275fs
XH_P36	2	27248517	27248517	Frame_Shift_Del	DEL	C	-	C	MAPRE3	c.536delC	p.A179fs
XH_P36	2	29016762	29016762	Nonsense_Mutation	SNP	C	T	C	PPP1CB	c.C778T	p.R260X
XH_P36	2	29455174	29455174	Silent	SNP	G	A	G	ALK	c.C2628T	p.A876A
XH_P36	2	31010045	31010045	Missense_Mutation	SNP	C	A	C	CAPN13	c.G147T	p.Q49H
XH_P36	2	36704119	36704119	Frame_Shift_Del	DEL	A	-	A	CRIM1	c.1079delA	p.Q360fs
XH_P36	2	37235963	37235963	Nonsense_Mutation	INS	-	GATGGTGAGTAT	-	HEATR5B	insTGTAATACTC	438_E1439delinsMX
XH_P36	2	37347123	37347123	Frame_Shift_Del	DEL	T	-	T	EIF2AK2	c.1104delA	p.K368fs
XH_P36	2	42720141	42720141	Silent	SNP	C	T	C	KCNG3	c.G501A	p.S167S
XH_P36	2	44190786	44190786	Frame_Shift_Ins	INS	-	CAATTTTCTTTTT	-	LRPPRC	429insAAAAAAG	p.Y477fs
XH_P36	2	44190788	44190788	Missense_Mutation	SNP	G	C	G	LRPPRC	c.C1427G	p.T476R
XH_P36	2	46574080	46574080	Missense_Mutation	SNP	A	G	A	EPAS1	c.A95G	p.E32G
XH_P36	2	46842236	46842236	Missense_Mutation	SNP	C	A	C	PIGF	c.G68T	p.S23I
XH_P36	2	48602207	48602207	Silent	SNP	T	C	T	FOXN2	c.T921C	p.S307S
XH_P36	2	50765682	50765682	Frame_Shift_Ins	INS	-	C	-	NRXN1	c.1767dupG	p.L590fs
XH_P36	2	60987361	60987361	Missense_Mutation	SNP	A	G	A	PAPOLG	c.A110G	p.Y37C
XH_P36	2	61753609	61753609	Frame_Shift_Ins	INS	-	TATAATTAAT	-	XPO1	_174insATTAAT	p.D58fs
XH_P36	2	61753611	61753611	Nonsense_Mutation	INS	-	TGCAAATAGTCC	-	XPO1	72insTAGGACTA	p.D58delinsX
XH_P36	2	70408460	70408460	Missense_Mutation	SNP	G	A	G	C2orf42	c.C658T	p.R220C
XH_P36	2	71046551	71046551	Silent	SNP	C	T	C	CLEC4F	c.G204A	p.P68P
XH_P36	2	71650264	71650264	Frame_Shift_Ins	INS	-	GACTATCAGTTGC	-	ZNF638	nsAGGGACTATC	p.S1207fs
XH_P36	2	73518631	73518631	Missense_Mutation	SNP	T	C	T	EGR4	c.A1724G	p.K575R

XH_P36	2	73799626	73799626	Frame_Shift_Ins	INS	-	ACGGG	-	ALMS1	519_10620insAC	p.D3540fs
XH_P36	2	73799627	73799627	Frame_Shift_Ins	INS	-	AG	-	ALMS1	0620_10621ins	p.D3540fs
XH_P36	2	73828531	73828531	In_Frame_Ins	INS	-	ACAGGG	-	ALMS1	79_12080insAC	.L4027delinsHRV
XH_P36	2	74326498	74326498	Silent	SNP	G	T	G	TET3	c.G3363T	p.T1121T
XH_P36	2	74687550	74687550	Frame_Shift_Del	DEL	T	-	T	WBP1	c.552delT	p.P184fs
XH_P36	2	74742324	74742324	Missense_Mutation	SNP	C	T	C	TLX2	c.C391T	p.R131W
XH_P36	2	74900900	74900900	Missense_Mutation	SNP	G	A	G	SEMA4F	c.G668A	p.R223Q
XH_P36	2	85043102	85043102	Missense_Mutation	SNP	C	T	C	DNAH6	c.C12268T	p.P4090S
XH_P36	2	96521783	96521783	In_Frame_Ins	INS	-	GGCACAGTAGAT	-	ANKRD36C	4insTTTATCTAC	1775delinsIYLLCPG
XH_P36	2	97475131	97475131	Silent	SNP	G	A	G	CNNM4	c.G2205A	p.G735G
XH_P36	2	97638040	97638040	Silent	SNP	G	A	G	FAM178B	c.C162T	p.T54T
XH_P36	2	97827852	97827852	Missense_Mutation	SNP	C	T	C	ANKRD36	c.C1406T	p.P469L
XH_P36	2	99182127	99182127	Missense_Mutation	SNP	A	G	A	INPP4A	c.A2027G	p.D676G
XH_P36	2	102482928	102482928	Missense_Mutation	SNP	A	C	A	MAP4K4	c.A1754C	p.K585T
XH_P36	2	110301828	110301828	Frame_Shift_Del	DEL	T	-	T	SEPT10	c.1506delA	p.K502fs
XH_P36	2	112705139	112705139	Missense_Mutation	SNP	T	C	T	MERTK	c.T752C	p.V251A
XH_P36	2	112942828	112942828	Missense_Mutation	SNP	T	C	T	FBLN7	c.T721C	p.C241R
XH_P36	2	120303807	120303807	Frame_Shift_Del	DEL	A	-	A	CFAP221	c.100delA	p.K34fs
XH_P36	2	122205058	122205058	Silent	SNP	C	T	C	CLASP1	c.G1767A	p.V589V
XH_P36	2	128467115	128467115	Missense_Mutation	SNP	G	A	G	WDR33	c.C3514T	p.R1172C
XH_P36	2	128477850	128477850	Silent	SNP	G	A	G	WDR33	c.C1749T	p.L583L
XH_P36	2	128522398	128522398	Frame_Shift_Ins	INS	-	CGTT	-	WDR33	629_630insAAC	p.F210fs
XH_P36	2	128522399	128522399	Nonsense_Mutation	INS	-	TCCTTTTATTAT	-	WDR33	)insAGATAATAA	F210_I211delinsX
XH_P36	2	130897920	130897920	Silent	SNP	G	A	G	CCDC74B	c.C510T	p.N170N
XH_P36	2	131803707	131803707	Missense_Mutation	SNP	G	A	G	ARHGEF4	c.G2018A	p.R673H
XH_P36	2	131976173	131976283	In_Frame_Del	DEL	TGGAGACCA	-	TCTGGAGACCAC	POTEE	c.198_308del	p.66_103del
XH_P36	2	132290242	132290242	Missense_Mutation	SNP	G	C	G	CCDC74A	c.G566C	p.G189A
XH_P36	2	135745134	135745134	In_Frame_Ins	INS	-	ATGACAAGCATA	-	MAP3K19	TCATCTTATGCT	23delinsEFILCLSFSW
XH_P36	2	135893484	135893484	Frame_Shift_Ins	INS	-	TTATCTGTTTCTT	-	RAB3GAP1	TTTTCCTTATCT	p.L635fs
XH_P36	2	141115575	141115575	Missense_Mutation	SNP	C	A	C	LRP1B	c.G11368T	p.G3790C
XH_P36	2	141135790	141135790	Frame_Shift_Del	DEL	A	-	A	LRP1B	c.10597delT	p.W3533fs
XH_P36	2	143798030	143798030	Frame_Shift_Del	DEL	A	-	A	KYNU	c.1075delA	p.K359fs
XH_P36	2	148657041	148657041	Frame_Shift_Del	DEL	A	-	A	ACVR2A	c.278delA	p.E93fs
XH_P36	2	148683686	148683686	Frame_Shift_Del	DEL	A	-	A	ACVR2A	c.979delA	p.K327fs
XH_P36	2	152320446	152320446	In_Frame_Ins	INS	-	TACTACTGGGGG	-	RIF1	3insTATTACTACT	L471delinsDITTGGV
XH_P36	2	152548835	152548835	Silent	SNP	A	G	A	NEB	c.T1938C	p.S646S
XH_P36	2	160075810	160075810	Missense_Mutation	SNP	C	T	C	TANC1	c.C2837T	p.T946M
XH_P36	2	163291933	163291933	Frame_Shift_Ins	INS	-	TGTCTATTAGGA	-	KCNH7	ATTCTATCCTAAT	p.E570fs
XH_P36	2	166785799	166785799	Missense_Mutation	SNP	C	T	C	TTC21B	c.G1232A	p.R411Q
XH_P36	2	167262863	167262863	Frame_Shift_Ins	INS	-	ACCTTGAGTGAA	-	SCN7A	76insATCTTCACT	p.C1426fs
XH_P36	2	167298238	167298238	Missense_Mutation	SNP	A	C	A	SCN7A	c.T1825G	p.Y609D
XH_P36	2	167298240	167298240	Frame_Shift_Ins	INS	-	GGTGTATTCC	-	SCN7A	_1823insGGAAT	p.K608fs
XH_P36	2	168105837	168105837	Nonsense_Mutation	INS	-	TTGATTTTGGCT	-	XIRP2	sATGTGTTGATT	23_L2424delinsVMCX
XH_P36	2	168106512	168106512	Frame_Shift_Ins	INS	-	TTTTTGGTAAAG	-	XIRP2	TATTGTTTTTG	p.Q2648fs
XH_P36	2	170038761	170038761	Missense_Mutation	SNP	C	T	C	LRP2	c.G9914A	p.R3305H
XH_P36	2	170366973	170366973	Frame_Shift_Ins	INS	-	A	-	KLHL41	c.686dupA	p.E229fs
XH_P36	2	170460581	170460581	Frame_Shift_Del	DEL	T	-	T	PPIG	c.30delT	p.C10fs
XH_P36	2	170606130	170606130	Frame_Shift_Ins	INS	-	T	-	KLHL23	c.1565_1566ins	p.T522fs
XH_P36	2	170606131	170606131	Frame_Shift_Ins	INS	-	AGCACAATCCA	-	KLHL23	_1567insAGCAC	p.T522fs
XH_P36	2	172549339	172549339	Frame_Shift_Del	DEL	A	-	A	DYNC1I2	c.161delA	p.E54fs
XH_P36	2	173855617	173855617	Nonsense_Mutation	INS	-	ATGAGGTTTGA	-	RAPGEF4	795insATGAGG	265_A266delinsRX
XH_P36	2	173883474	173883474	Missense_Mutation	SNP	C	T	C	RAPGEF4	c.C1439T	p.A480V
XH_P36	2	175436887	175436887	Missense_Mutation	SNP	C	T	C	WIPF1	c.G646A	p.G216R
XH_P36	2	176995146	176995151	In_Frame_Del	DEL	GCGGCG	-	GCGGCG	HOXD8	c.52_57del	p.18_19del
XH_P36	2	177036404	177036404	Missense_Mutation	SNP	C	T	C	HOXD3	c.C701T	p.T234M
XH_P36	2	179318291	179318291	Nonsense_Mutation	INS	-	AAAATACAAAG	-	DFNB59	ATCTTAAAAATA	T52_S53delinsTSX
XH_P36	2	179478981	179478981	Silent	SNP	A	G	A	TTN	c.T21948C	p.S7316S
XH_P36	2	179483496	179483496	Missense_Mutation	SNP	T	C	T	TTN	c.A19586G	p.Y6529C
XH_P36	2	179536874	179536874	Nonsense_Mutation	INS	-	TAGATGATAGTA	-	TTN	TCAAATATTACT	0383_H10384delinsX
XH_P36	2	179549465	179549465	Nonsense_Mutation	INS	-	GTAGAGTATTA	-	TTN	_28834insTAAT	9612_K9613delinsX
XH_P36	2	179574422	179574422	In_Frame_Ins	INS	-	TGAGCATTTCTT	-	TTN	ATATCATAAGA	98delinsDIIRNAHGYI
XH_P36	2	179575449	179575449	Missense_Mutation	SNP	C	T	C	TTN	c.G24643A	p.V8215I
XH_P36	2	179578885	179578885	Nonsense_Mutation	INS	-	TAATAATGTAGT	-	TTN	ATCAGGAACTA	7590_K7591delinsX
XH_P36	2	179587901	179587901	Missense_Mutation	SNP	A	G	A	TTN	c.T18101C	p.I6034T
XH_P36	2	179587903	179587903	Frame_Shift_Ins	INS	-	AGCCTTTC	-	TTN	8_18099insGAA	p.N6033fs
XH_P36	2	179621158	179621158	Missense_Mutation	SNP	A	G	A	TTN	c.T10532C	p.V3511A
XH_P36	2	182763804	182763804	Silent	SNP	C	A	C	SSFA2	c.C9A	p.S3S
XH_P36	2	186662103	186662103	Frame_Shift_Del	DEL	A	-	A	FSIP2	c.10240delA	p.K3414fs
XH_P36	2	187615880	187615880	Silent	SNP	G	A	G	FAM171B	c.G744A	p.L248L
XH_P36	2	189866275	189866275	Frame_Shift_Del	DEL	C	-	C	COL3A1	c.2351delC	p.A784fs
XH_P36	2	189868745	189868745	Missense_Mutation	SNP	G	A	G	COL3A1	c.G2699A	p.G900D
XH_P36	2	191381108	191381108	Nonsense_Mutation	INS	-	AATAATATTTTG	-	NEMP2	GATAAACAAAAT	Y205_S206delinsX
XH_P36	2	200213696	200213696	In_Frame_Ins	INS	-	TCTTTGCAA	-	SATB2	)_901insTTGCA	Q301delinsLQRQ
XH_P36	2	202149545	202149545	Missense_Mutation	SNP	T	C	T	CASP8	c.T764C	p.L255S
XH_P36	2	203807528	203807528	Silent	SNP	C	A	C	CARF	c.C108A	p.P36P
XH_P36	2	207169621	207169621	Frame_Shift_Ins	INS	-	ATATTCTAATGTC	-	ZDBF2	0insCATATTCTA	p.K123fs
XH_P36	2	207171368	207171368	Frame_Shift_Ins	INS	-	CTTCAGAAGA	-	ZDBF2	_2117insCTTCA	p.L706fs
XH_P36	2	207172364	207172364	Missense_Mutation	SNP	G	A	G	ZDBF2	c.G3112A	p.G1038S
XH_P36	2	207174428	207174428	Frame_Shift_Del	DEL	A	-	A	ZDBF2	c.5176delA	p.K1726fs
XH_P36	2	207175098	207175098	Nonsense_Mutation	INS	-	GTAACCTT	-	ZDBF2	6_5847insGTAA	949_C1950delinsRX
XH_P36	2	207527743	207527743	Missense_Mutation	SNP	G	T	G	DYTN	c.C1517A	p.A506D
XH_P36	2	210840978	210840978	Missense_Mutation	SNP	G	A	G	UNC80	c.G8525A	p.R2842Q
XH_P36	2	217124353	217124353	Nonsense_Mutation	INS	-	ACATCCTGAGT	-	MARCH4	915insACTCAG	V305_Q306delinsX
XH_P36	2	218674638	218674638	Nonsense_Mutation	INS	-	TCTACCTTCTCAT	-	TNS1	06insGATGAGA	2_E1603delinsSMRRX
XH_P36	2	218674639	218674639	Missense_Mutation	SNP	G	C	G	TNS1	c.C4805G	p.S1602C
XH_P36	2	219892362	219892362	Missense_Mutation	SNP	G	A	G	CFAP65	c.C2026T	p.R676C
XH_P36	2	219896326	219896326	Missense_Mutation	SNP	C	T	C	CFAP65	c.G505A	p.G169S
XH_P36	2	220083200	220083200	Frame_Shift_Del	DEL	C	-	C	ABCB6	c.196delG	p.A66fs
XH_P36	2	220116337	220116337	Missense_Mutation	SNP	T	C	T	TUBA4A	c.A280G	p.T94A
XH_P36	2	223917773	223917773	Silent	SNP	C	T	C	KCNE4	c.C378T	p.D126D
XH_P36	2	233323757	233323757	Silent	SNP	G	A	G	ALPI	c.G1488A	p.A496A
XH_P36	2	233410400	233410400	Missense_Mutation	SNP	C	T	C	CHRNG	c.C1528T	p.R510C
XH_P36	2	234365952	234365952	Frame_Shift_Del	DEL	G	-	G	DGKD	c.2426delG	p.W809fs
XH_P36	2	239057734	239057734	Missense_Mutation	SNP	A	G	A	KLHL30	c.A1426G	p.I476V
XH_P36	2	239258015	239258015	Missense_Mutation	SNP	C	T	C	TRAF3IP1	c.C1219T	p.R407W
XH_P36	2	241885639	241885639	Missense_Mutation	SNP	C	T	C	CROCC2	c.C2332T	p.R778W
XH_P36	2	242179074	242179074	Silent	SNP	G	A	G	HDLBP	c.C2454T	p.G818G
XH_P36	3	367670	367670	Silent	SNP	G	A	G	CHL1	c.G120A	p.Q40Q

XH_P36	3	1371501	1371501	Frame_Shift_Del	DEL	A	-	A	CNTN6	c.934delA	p.K312fs
XH_P36	3	5025215	5025215	In_Frame_Ins	INS	-	CAT	-	BHLHE40	1077_1078insC/	p.A359delinsAH
XH_P36	3	5025217	5025217	In_Frame_Ins	INS	-	GAGAAGTGCTG	-	BHLHE40	80insTGGAGAA	360delinsAGEVLV
XH_P36	3	5252820	5252820	Silent	SNP	G	A	G	EDEM1	c.G1599A	p.T533T
XH_P36	3	10382200	10382200	Missense_Mutation	SNP	C	T	C	ATP2B2	c.G2971A	p.V991M
XH_P36	3	12641888	12641888	Frame_Shift_Ins	INS	-	CTGCCAATTAG	-	RAF1	61insCCTAATTG	p.S287fs
XH_P36	3	12942841	12942841	Frame_Shift_Del	DEL	G	-	G	IQSEC1	c.2986delC	p.H996fs
XH_P36	3	25654189	25654189	Silent	SNP	A	G	A	TOP2B	c.T3588C	p.S1196S
XH_P36	3	25805772	25805772	Frame_Shift_Ins	INS	-	T	-	NGLY1	c.276dupA	p.A93fs
XH_P36	3	27233665	27233665	Frame_Shift_Ins	INS	-	AATAGTTACATC	-	NEK10	ATATTGCATGTA	p.M99fs
XH_P36	3	30691872	30691873	Frame_Shift_Del	DEL	AA	-	AA	TGFBR2	c.374_375del	p.E125fs
XH_P36	3	31574563	31574563	Missense_Mutation	SNP	G	A	G	STT3B	c.G73A	p.A25T
XH_P36	3	31663787	31663787	Nonsense_Mutation	INS	-	CTGAACTAGATA	-	STT3B	527insCTGAACT	509_L510delinsNX
XH_P36	3	36897022	36897022	Silent	SNP	C	T	C	TRANK1	c.G4059A	p.L1353L
XH_P36	3	38889230	38889230	Nonsense_Mutation	INS	-	CTCTTTCTGATT/	-	SCN11A	AAACATAATCAG	444_M1445delinsRX
XH_P36	3	40528589	40528589	Silent	SNP	T	G	T	ZNF619	c.T456G	p.G152G
XH_P36	3	41275758	41275758	Silent	SNP	G	T	G	CTNNB1	c.G1653T	p.T551T
XH_P36	3	41275760	41275760	Frame_Shift_Ins	INS	-	CTAAGGAATAC/	-	CTNNB1	TACTTACTAAGG	p.S552fs
XH_P36	3	42680096	42680096	Frame_Shift_Ins	INS	-	AGATTCTCTTTC	-	NKTR	TCAGAAAGATT	p.S967fs
XH_P36	3	44636110	44636110	In_Frame_Ins	INS	-	TTTTCCACA	-	ZNF660	5_426insTTTTC	.A142delinsAFPH
XH_P36	3	44636112	44636112	Frame_Shift_Ins	INS	-	CCTTACAC	-	ZNF660	27_428insCCTTA	p.F143fs
XH_P36	3	44692631	44692631	Silent	SNP	A	G	A	ZNF35	c.A72G	p.E24E
XH_P36	3	45943194	45943194	Missense_Mutation	SNP	T	C	T	CCR9	c.T878C	p.I293T
XH_P36	3	46875151	46875151	Frame_Shift_Del	DEL	G	-	G	PRSS42	c.230delC	p.P77fs
XH_P36	3	47891256	47891256	Missense_Mutation	SNP	C	T	C	DHX30	c.C3328T	p.R1110C
XH_P36	3	48461122	48461122	Frame_Shift_Del	DEL	C	-	C	PLXNB1	c.2573delG	p.G858fs
XH_P36	3	48694444	48694444	Silent	SNP	C	T	C	CELSR3	c.G4086A	p.A1362A
XH_P36	3	49210370	49210370	Silent	SNP	G	A	G	KLHDC8B	c.G168A	p.S56S
XH_P36	3	50005145	50005145	Frame_Shift_Del	DEL	G	-	G	RBM6	c.287delG	p.R96fs
XH_P36	3	50155888	50155891	Frame_Shift_Del	DEL	GAGA	-	GAGA	RBM5	c.2447_2450del	p.*816fs
XH_P36	3	50324240	50324240	Missense_Mutation	SNP	C	T	C	LSMEM2	c.C305T	p.A102V
XH_P36	3	50385747	50385747	Splice_Site	SNP	C	T	C	NPRL2	.	.
XH_P36	3	52437315	52437315	Splice_Site	SNP	C	T	C	BAP1	.	.
XH_P36	3	53263342	53263342	Silent	SNP	G	A	G	TKT	c.C1218T	p.S406S
XH_P36	3	77623837	77623837	Frame_Shift_Ins	INS	-	TCCTCATAACTAT	-	ROBO2	TTTCTGTCCTCAT	p.Q720fs
XH_P36	3	77657037	77657037	Frame_Shift_Ins	INS	-	C	-	ROBO2	c.3226dupC	p.P1075fs
XH_P36	3	108139971	108139971	Silent	SNP	G	A	G	MYH15	c.C3853T	p.L1285L
XH_P36	3	108229398	108229398	Frame_Shift_Del	DEL	A	-	A	MYH15	c.40delT	p.W14fs
XH_P36	3	111828413	111828413	Silent	SNP	G	A	G	C3orf52	c.G420A	p.S140S
XH_P36	3	112538716	112538716	Silent	SNP	A	G	A	CD200R1L	c.T706C	p.L236L
XH_P36	3	112648129	112648129	Missense_Mutation	SNP	C	T	C	CD200R1	c.G359A	p.R120H
XH_P36	3	112989730	112989730	Silent	SNP	C	A	C	BOC	c.C606A	p.A202A
XH_P36	3	113377483	113377483	Missense_Mutation	SNP	T	G	T	USF3	c.A3046C	p.N1016H
XH_P36	3	113850142	113850142	Missense_Mutation	SNP	T	C	T	DRD3	c.A829G	p.K277E
XH_P36	3	119962621	119962621	Nonsense_Mutation	INS	-	CTAG	-	GPR156	c.98_99insCTAG	T33_S34delinsTX
XH_P36	3	119962623	119962623	Nonsense_Mutation	INS	-	GTATTGTGCTAA	-	GPR156	nsTAGTTAGCAC/	.T33_S34delinsX
XH_P36	3	121208048	121208048	Frame_Shift_Ins	INS	-	AAGATAGTTTCT	-	POLQ	CAGATAGAAAC	p.T1244fs
XH_P36	3	121208707	121208707	Frame_Shift_Ins	INS	-	CTAAAAATGTAT	-	POLQ	071insGATACAT	p.K1024fs
XH_P36	3	121208709	121208709	In_Frame_Ins	INS	-	TGAGTG	-	POLQ	068_3069insCAC	.T1023delinsTTQ
XH_P36	3	122943972	122943972	Missense_Mutation	SNP	G	T	G	SEC22A	c.G369T	p.K123N
XH_P36	3	126707536	126707536	Missense_Mutation	SNP	G	T	G	PLXNA1	c.G100T	p.G34C
XH_P36	3	130361855	130361855	Nonsense_Mutation	SNP	C	T	C	COL6A6	c.C5215T	p.R1739X
XH_P36	3	132051057	132051057	Missense_Mutation	SNP	G	A	G	ACPP	c.G325A	p.V109I
XH_P36	3	132319688	132319688	Frame_Shift_Ins	INS	-	TGTACACATCT	-	ACKR4	448insTGTACA	p.G149fs
XH_P36	3	133666246	133666246	Frame_Shift_Ins	INS	-	CTATTCTTTGCT	-	SLCO2A1	sTGTACAGCAAA	p.G383fs
XH_P36	3	136019877	136019877	Missense_Mutation	SNP	G	A	G	PCCB	c.G890A	p.R297H
XH_P36	3	136240241	136240241	Nonsense_Mutation	INS	-	CTTTTCATTTAG	-	STAG1	CAAAACTAAATG	o.L164delinsKTX
XH_P36	3	138739338	138739338	Missense_Mutation	SNP	C	T	C	PRR23B	c.G166A	p.A56T
XH_P36	3	142261555	142261555	Frame_Shift_Del	DEL	A	-	A	ATR	c.3402delT	p.F1134fs
XH_P36	3	142269109	142269109	In_Frame_Ins	INS	-	TTAAGTTTAAGT	-	ATR	CAAATCCACTTA/	47delinsTQIHLNLSFT
XH_P36	3	143214258	143214258	Frame_Shift_Ins	INS	-	AATGACTTACT	-	SLC9A9	1122insAGTAA	p.N374fs
XH_P36	3	148459721	148459721	Frame_Shift_Del	DEL	T	-	T	AGTR1	c.899delT	p.L300fs
XH_P36	3	149485156	149485156	Frame_Shift_Ins	INS	-	AG	-	ANKUB1	.1292_1293insC	p.T431fs
XH_P36	3	153842199	153842199	Frame_Shift_Del	DEL	A	-	A	ARHGEF26	c.1084delA	p.K362fs
XH_P36	3	157816092	157816092	Silent	SNP	C	T	C	SHOX2	c.G720A	p.V240V
XH_P36	3	160231939	160231939	Missense_Mutation	SNP	C	T	C	KPNA4	c.G1063A	p.A355T
XH_P36	3	161221015	161221015	Missense_Mutation	SNP	A	G	A	OTOL1	c.A719G	p.D240G
XH_P36	3	167507164	167507164	Nonsense_Mutation	INS	-	TTACATATTAAT	-	SERPINI1	ACAATTTACATA	G84delinsNNNLHINYX
XH_P36	3	170856027	170856027	Missense_Mutation	SNP	A	G	A	TNIK	c.T1474C	p.Y492H
XH_P36	3	170856028	170856028	Frame_Shift_Ins	INS	-	TTTTGTTTTTGTT	-	TNIK	insAAAAAACAA/	p.H491fs
XH_P36	3	175042056	175042056	Frame_Shift_Ins	INS	-	T	-	NAALADL2	c.1033dupT	p.T344fs
XH_P36	3	178952085	178952085	Missense_Mutation	SNP	A	G	A	PIK3CA	c.A3140G	p.H1047R
XH_P36	3	179690970	179690970	Missense_Mutation	SNP	G	A	G	PEX5L	c.C70T	p.R24W
XH_P36	3	183368801	183368801	Frame_Shift_Ins	INS	-	CTTCACCTTTCC/	-	KLHL24	658insCTTCACC	p.D219fs
XH_P36	3	183368803	183368803	Missense_Mutation	SNP	A	T	A	KLHL24	c.A659T	p.E220V
XH_P36	3	183888195	183888195	Missense_Mutation	SNP	C	G	C	DVL3	c.C1803G	p.S601R
XH_P36	3	193854823	193854823	Missense_Mutation	SNP	G	T	G	HES1	c.G278T	p.R93L
XH_P36	3	194081622	194081622	Nonsense_Mutation	INS	-	CAACCTCA	-	LRRC15	0_151insTGAGG	.L51_P52delinsX
XH_P36	3	195295892	195295892	Missense_Mutation	SNP	G	A	G	APOD	c.C449T	p.A150V
XH_P36	3	195508661	195508661	Missense_Mutation	SNP	A	G	A	MUC4	c.T9790C	p.S3264P
XH_P36	4	154798	154798	Nonsense_Mutation	INS	-	ATCTCCAGTATA	-	ZNF718	GTTTTATCTCCA	08_E109delinsHILYIX
XH_P36	4	437209	437209	Silent	SNP	G	A	G	ZNF721	c.C1047T	p.Y349Y
XH_P36	4	515806	515806	In_Frame_Ins	INS	-	TGCGCA	-	PIGG	23_1424insTGC	p.R475delinsLRS
XH_P36	4	961739	961739	Missense_Mutation	SNP	G	A	G	DGKQ	c.C740T	p.A247V
XH_P36	4	1719361	1719361	Missense_Mutation	SNP	C	T	C	TMEM129	c.G722A	p.R241Q
XH_P36	4	4304807	4304807	Missense_Mutation	SNP	G	A	G	ZBTB49	c.G1244A	p.R415Q
XH_P36	4	5018651	5018651	Missense_Mutation	SNP	G	A	G	CYTL1	c.C239T	p.S80L
XH_P36	4	8375308	8375308	Missense_Mutation	SNP	C	T	C	ACOX3	c.G1876A	p.V626I
XH_P36	4	20706377	20706377	Frame_Shift_Ins	INS	-	CCCTCTGATT	-	PACRGL	7_148insCCCTCT	p.E49fs
XH_P36	4	25278714	25278714	Missense_Mutation	SNP	C	T	C	PI4K2B	c.C1351T	p.R451C
XH_P36	4	39458114	39458114	Nonsense_Mutation	INS	-	ACTTAAGGGTTT	-	RPL9	GATATAAACCCT	01_N102delinsMIX
XH_P36	4	39864524	39864524	Missense_Mutation	SNP	C	T	C	PDS5A	c.G2936A	p.R979H
XH_P36	4	41748011	41748028	In_Frame_Del	DEL	CCGCCGCTGC	-	GCCGCCGCTGCC	PHOX2B	c.741_758del	p.247_253del
XH_P36	4	47560133	47560133	Silent	SNP	G	A	G	ATP10D	c.G2277A	p.L759L
XH_P36	4	48622707	48622707	Missense_Mutation	SNP	G	A	G	FRYL	c.C263T	p.T88M
XH_P36	4	57179502	57179502	Frame_Shift_Ins	INS	-	A	-	KIAA1211	c.495dupA	p.P165fs
XH_P36	4	57889645	57889645	Frame_Shift_Ins	INS	-	CACCCTTATTCCA	-	POLR2B	AATCACACCCTT	p.K814fs
XH_P36	4	68488908	68488908	Nonsense_Mutation	SNP	C	T	C	UBA6	c.G2873A	p.W958X

XH_P36	4	70146409	70146409	Frame_Shift_Ins	INS	-	CAAG	-	UGT2B28	.191_192insCAA	p.F64fs
XH_P36	4	70146410	70146410	Frame_Shift_Ins	INS	-	TCTTTCTCATACT	-	UGT2B28	CTGTTTCTTTCTC	p.F64fs
XH_P36	4	70720027	70720027	Nonsense_Mutation	INS	-	A	-	SULT1E1	c.276_277insT	.K93_Q94delinsX
XH_P36	4	70720028	70720028	Frame_Shift_Ins	INS	-	CTTTCATTGAATC	-	SULT1E1	CTATAGATTCAAT	p.V92fs
XH_P36	4	71232641	71232641	In_Frame_Ins	INS	-	TGG	-	SMR3A	c.335_336insTG	p.Y112delinsYG
XH_P36	4	71232643	71232643	Frame_Shift_Ins	INS	-	GGAGAATTCAA	-	SMR3A	insCAGGGAGAA	p.P113fs
XH_P36	4	74021478	74021478	Nonsense_Mutation	INS	-	AAAGTGATGTTT	-	ANKRD17	1insCTAAACATC	177_A178delinsHX
XH_P36	4	77065318	77065318	Silent	SNP	C	T	C	NUP54	c.G279A	p.Q93Q
XH_P36	4	80905990	80905990	Frame_Shift_Del	DEL	C	-	C	ANTXR2	c.1069delG	p.A357fs
XH_P36	4	81791254	81791254	Frame_Shift_Del	DEL	A	-	A	C4orf22	c.441delA	p.G147fs
XH_P36	4	83989669	83989669	Frame_Shift_Ins	INS	-	TAAGTTAACTGG	-	COPS4	insATTTTAAGTT	p.F361fs
XH_P36	4	88537426	88537426	Silent	SNP	T	C	T	DSPP	c.T3612C	p.S1204S
XH_P36	4	89653284	89653284	Silent	SNP	G	A	G	FAM13A	c.C1650T	p.T550T
XH_P36	4	90857025	90857025	Frame_Shift_Ins	INS	-	ATCATTTAGT	-	MMRN1	_2195insATCAT	p.M732fs
XH_P36	4	90857027	90857027	In_Frame_Ins	INS	-	CTC	-	MMRN1	2196_2197insC	.M732delinsML
XH_P36	4	95173909	95173909	Frame_Shift_Ins	INS	-	A	-	SMARCAD1	c.1033dupA	p.R344fs
XH_P36	4	95191919	95191919	Silent	SNP	C	T	C	SMARCAD1	c.C232T	p.L78L
XH_P36	4	100478535	100478535	Silent	SNP	G	A	G	TRMT10A	c.C387T	p.Y129Y
XH_P36	4	107156505	107156505	Frame_Shift_Del	DEL	T	-	T	TBCK	c.1181delA	p.N394fs
XH_P36	4	110972797	110972797	Nonsense_Mutation	INS	-	CT	-	ELOVL6	c.494_495insAG	V165_F166delinsX
XH_P36	4	110972799	110972799	In_Frame_Ins	INS	-	GCT	-	ELOVL6	c.492_493insAG	.W165delinsSW
XH_P36	4	114275990	114275990	Silent	SNP	C	A	C	ANK2	c.C6216A	p.S2072S
XH_P36	4	114275991	114275991	Frame_Shift_Ins	INS	-	TCCTCTCTGT	-	ANK2	7_6218insTCCTC	p.I2073fs
XH_P36	4	114286281	114286281	Missense_Mutation	SNP	C	T	C	ANK2	c.C4720T	p.R1574C
XH_P36	4	119148049	119148049	Silent	SNP	C	T	C	NDST3	c.C1731T	p.C577C
XH_P36	4	120189503	120189503	Missense_Mutation	SNP	C	A	C	USP53	c.C1216A	p.Q406K
XH_P36	4	134071649	134071649	Frame_Shift_Del	DEL	C	-	C	PCDH10	c.354delC	p.N118fs
XH_P36	4	134072199	134072199	Missense_Mutation	SNP	G	A	G	PCDH10	c.G904A	p.G302R
XH_P36	4	134073341	134073341	Silent	SNP	C	A	C	PCDH10	c.C2046A	p.A682A
XH_P36	4	138442370	138442370	Missense_Mutation	SNP	C	T	C	PCDH18	c.G3218A	p.G1073E
XH_P36	4	138452170	138452170	Missense_Mutation	SNP	A	C	A	PCDH18	c.T1073G	p.L358R
XH_P36	4	145658978	145658978	Frame_Shift_Del	DEL	A	-	A	HHIP	c.1972delA	p.K658fs
XH_P36	4	153268210	153268210	Nonsense_Mutation	INS	-	GAAATGCCTCAC	-	FBXW7	4insAAGTGAGG	V82_P83delinsKX
XH_P36	4	156643278	156643278	Frame_Shift_Ins	INS	-	TCTTAACTCTTTT	-	GUCY1A3	6insACTTCTTAA	p.T602fs
XH_P36	4	159573185	159573185	Missense_Mutation	SNP	C	T	C	RXFP1	c.C2108T	p.T703M
XH_P36	4	166946559	166946559	Frame_Shift_Ins	INS	-	CTACAAGAATCC	-	TLL1	nsAACTCTACAA	p.R378fs
XH_P36	4	183696222	183696222	Silent	SNP	C	T	C	TENM3	c.C5220T	p.G1740G
XH_P36	4	189022306	189022306	Missense_Mutation	SNP	C	A	C	TRIML2	c.G384T	p.Q128H
XH_P36	5	482774	482774	Silent	SNP	G	A	G	SLC9A3	c.C1245T	p.R415R
XH_P36	5	1212555	1212555	Missense_Mutation	SNP	G	A	G	SLC6A19	c.G619A	p.V207I
XH_P36	5	1882750	1882750	Silent	SNP	C	T	C	IRX4	c.G12A	p.P4P
XH_P36	5	5464054	5464054	Nonsense_Mutation	INS	-	TCTGACACTGAA	-	ICE1	08insTATCTGAC	536_S1537delinsAIX
XH_P36	5	9190436	9190436	Missense_Mutation	SNP	C	T	C	SEMA5A	c.G1216A	p.A406T
XH_P36	5	15928091	15928091	Missense_Mutation	SNP	T	C	T	FBXL7	c.T79C	p.S27P
XH_P36	5	16179661	16179661	Silent	SNP	G	A	G	MARCH11	c.C24T	p.G8G
XH_P36	5	23526788	23526788	Frame_Shift_Ins	INS	-	TTCTTTGATCTCT	-	PRDM9	92insCTTTCTTTG	p.Q531fs
XH_P36	5	24492973	24492973	Frame_Shift_Del	DEL	A	-	A	CDH10	c.1577delT	p.F526fs
XH_P36	5	26906213	26906213	In_Frame_Ins	INS	-	ACTTCTCAAATAC	-	CDH9	sTAGTGTATTTGA	222delinsPSVFEKFL
XH_P36	5	32091037	32091037	Missense_Mutation	SNP	A	G	A	PDZD2	c.A7483G	p.K2495E
XH_P36	5	32780891	32780891	Nonsense_Mutation	INS	-	TTCCTAAATTCTT	-	NPR3	60insATTCCTAA	0_M421delinsAFLNSX
XH_P36	5	36976160	36976160	In_Frame_Ins	INS	-	ATCTGGTGGAAC	-	NIPBL	2insGATATCTGG	I384delinsKISGGTS
XH_P36	5	36985241	36985241	Frame_Shift_Ins	INS	-	GTGC	-	NIPBL	1959_1960insGT	p.Q653fs
XH_P36	5	36985242	36985242	In_Frame_Ins	INS	-	CATTACATTGTTT	-	NIPBL	961insTCATTACA	N654delinsIITLFD
XH_P36	5	37438050	37438050	Silent	SNP	G	A	G	WDR70	c.G453A	p.S151S
XH_P36	5	55237462	55237462	In_Frame_Ins	INS	-	CTACAATTAGTA	-	IL6ST	2022insTACTAA	S674delinsSTNCR
XH_P36	5	55237464	55237464	Nonsense_Mutation	INS	-	TCATGTAGATGG	-	IL6ST	2020insCCATCT	.S674delinsPSTX
XH_P36	5	56171053	56171053	Nonsense_Mutation	INS	-	ACACACACGATG	-	MAP3K1	2insAAACACACA	7_S628delinsIKHTRX
XH_P36	5	61847115	61847115	Frame_Shift_Del	DEL	T	-	T	IPO11	c.2532delT	p.S844fs
XH_P36	5	64867698	64867698	Missense_Mutation	SNP	G	A	G	PPWD1	c.G86A	p.C29Y
XH_P36	5	67589266	67589266	Nonsense_Mutation	INS	-	TACCTTAGTGTA	-	PIK3R1	TGGCTTACCTTA	_K56delinsPWLTLVX
XH_P36	5	68651571	68651571	In_Frame_Ins	INS	-	TGTTGTTGTTTGT	-	AK6	ACAAAACAAACA	74delinsETKQTTTIF
XH_P36	5	68716041	68716041	Frame_Shift_Ins	INS	-	CGAC	-	MARVELD2	.829_830insCGA	p.M277fs
XH_P36	5	68716042	68716042	Frame_Shift_Ins	INS	-	TCATGTAGGA	-	MARVELD2	_831insTCATGT	p.M277fs
XH_P36	5	70766233	70766233	Frame_Shift_Del	DEL	T	-	T	BDP1	c.931delT	p.F311fs
XH_P36	5	71501039	71501039	Silent	SNP	C	A	C	MAP1B	c.C7002A	p.S2334S
XH_P36	5	71756466	71756466	Silent	SNP	G	A	G	ZNF366	c.C858T	p.C286C
XH_P36	5	74016556	74016556	Nonsense_Mutation	INS	-	TTCTTAAATGTTA	-	HEXB	nsTTAATTCTTAA	.R533delinsLNSX
XH_P36	5	74134851	74134851	Missense_Mutation	SNP	T	C	T	FAM169A	c.A257G	p.Q86R
XH_P36	5	76358967	76358967	Frame_Shift_Del	DEL	A	-	A	AGGF1	c.2035delA	p.K679fs
XH_P36	5	79733694	79733694	Frame_Shift_Ins	INS	-	CATCTCTTGAAT	-	ZFYVE16	AATTTTCATCTC	p.F397fs
XH_P36	5	82816284	82816284	Missense_Mutation	SNP	T	A	T	VCAN	c.T2159A	p.V720D
XH_P36	5	82834130	82834130	Frame_Shift_Ins	INS	-	TGGTGAACATT	-	VCAN	_2348insTGGTG	p.R783fs
XH_P36	5	89939712	89939712	In_Frame_Ins	INS	-	AGAGGTGGTATC	-	ADGRV1	GACAAAAGAGGT	2delinsKGQKRWYHFF
XH_P36	5	96062499	96062499	Missense_Mutation	SNP	C	T	C	CAST	c.C23T	p.A8V
XH_P36	5	96443159	96443159	Missense_Mutation	SNP	C	T	C	LIX1	c.G292A	p.A98T
XH_P36	5	102895168	102895168	Frame_Shift_Ins	INS	-	TT	-	NUDT12	c.153_154insAA	p.C52fs
XH_P36	5	102895169	102895169	Nonsense_Mutation	INS	-	ATTCGTTAGGAA	-	NUDT12	CTTGGTTCCTAA	M51delinsISLGSX
XH_P36	5	111504782	111504782	In_Frame_Ins	INS	-	TACAGTTCATCA	-	EPB41L4A	1760insTGATGA	R587delinsLMNCS
XH_P36	5	111504784	111504784	Frame_Shift_Ins	INS	-	TTAGTAATAGT	-	EPB41L4A	_1758insACTATT	p.I586fs
XH_P36	5	112769886	112769886	Silent	SNP	G	A	G	TSSK1B	c.C651T	p.D217D
XH_P36	5	118324039	118324039	Missense_Mutation	SNP	C	A	C	DTWD2	c.G168T	p.W56C
XH_P36	5	121413097	121413097	Missense_Mutation	SNP	G	T	G	LOX	c.C584A	p.P195H
XH_P36	5	134050742	134050742	Missense_Mutation	SNP	G	A	G	SEC24A	c.G2756A	p.R919H
XH_P36	5	134364858	134364858	Missense_Mutation	SNP	C	T	C	PITX1	c.G556A	p.A186T
XH_P36	5	134367131	134367131	Silent	SNP	G	A	G	PITX1	c.C237T	p.G79G
XH_P36	5	137523102	137523102	Nonstop_Mutation	SNP	A	C	A	KIF20A	c.A2673C	p.X891Y
XH_P36	5	138265000	138265000	Silent	SNP	C	T	C	CTNNA1	c.C516T	p.S172S
XH_P36	5	140047894	140047894	Silent	SNP	T	C	T	WDR55	c.T267C	p.A89A
XH_P36	5	140049102	140049102	Frame_Shift_Del	DEL	A	-	A	WDR55	c.1015delA	p.K339fs
XH_P36	5	140207965	140207965	Missense_Mutation	SNP	G	C	G	PCDHA6	c.G289C	p.G97R
XH_P36	5	140230590	140230590	Frame_Shift_Del	DEL	T	-	T	PCDHA9	c.2510delT	p.I837fs
XH_P36	5	140237134	140237134	Missense_Mutation	SNP	C	T	C	PCDHA10	c.C1501T	p.R501C
XH_P36	5	140475658	140475658	Missense_Mutation	SNP	C	G	C	PCDHB2	c.C1284G	p.F428L
XH_P36	5	140595217	140595217	Missense_Mutation	SNP	G	A	G	PCDHB13	c.G1522A	p.A508T
XH_P36	5	140604336	140604336	Missense_Mutation	SNP	C	T	C	PCDHB14	c.C1259T	p.T420M
XH_P36	5	140683367	140683367	Silent	SNP	C	T	C	SLC25A2	c.G66A	p.A22A
XH_P36	5	140769366	140769366	Missense_Mutation	SNP	C	T	C	PCDHGB4	c.C1915T	p.R639C
XH_P36	5	140778721	140778721	Nonsense_Mutation	INS	-	GCTGACTC	-	PCDHGB5	7_1028insGCTG	343_T344delinsGX

XH_P36	5	140953309	140953309	Frame_Shift_Del	DEL	G	-	G	DIAPH1	c.2081delC	p.P694fs
XH_P36	5	140953564	140953569	In_Frame_Del	DEL	GGAGGA	-	GGAGGA	DIAPH1	c.1821_1826del	p.607_609del
XH_P36	5	143586393	143586393	Missense_Mutation	SNP	G	A	G	KCTD16	c.G116A	p.R39H
XH_P36	5	145547779	145547779	Frame_Shift_Del	DEL	G	-	G	LARS	c.182delC	p.P61fs
XH_P36	5	146754725	146754725	Frame_Shift_Del	DEL	A	-	A	STK32A	c.976delA	p.K326fs
XH_P36	5	147861099	147861099	Frame_Shift_Del	DEL	T	-	T	HTR4	c.1170delA	p.K390fs
XH_P36	5	148407222	148407222	Silent	SNP	G	T	G	SH3TC2	c.C2073A	p.A691A
XH_P36	5	149460507	149460507	Missense_Mutation	SNP	C	A	C	CSF1R	c.G130T	p.G44C
XH_P36	5	149602668	149602668	Silent	SNP	G	A	G	CAMK2A	c.C1317T	p.D439D
XH_P36	5	149901203	149901203	Silent	SNP	C	T	C	NDST1	c.C387T	p.G129G
XH_P36	5	154173390	154173390	Frame_Shift_Del	DEL	C	-	C	LARP1	c.668delC	p.A223fs
XH_P36	5	158139199	158139199	Silent	SNP	G	T	G	EBF1	c.C1419A	p.T473T
XH_P36	5	162917426	162917426	Frame_Shift_Del	DEL	A	-	A	HMMR	c.1732delA	p.K578fs
XH_P36	5	167841357	167841357	Missense_Mutation	SNP	G	A	G	WWC1	c.G946A	p.A316T
XH_P36	5	168098430	168098430	Silent	SNP	G	A	G	SLIT3	c.C3921T	p.G1307G
XH_P36	5	168149299	168149299	Frame_Shift_Del	DEL	G	-	G	SLIT3	c.2445delC	p.P815fs
XH_P36	5	169129368	169129368	Missense_Mutation	SNP	G	T	G	DOCK2	c.G1320T	p.K440N
XH_P36	5	171523524	171523524	Missense_Mutation	SNP	C	T	C	STK10	c.G911A	p.R304Q
XH_P36	5	171638950	171638950	Frame_Shift_Del	DEL	A	-	A	UBTD2	c.589delT	p.S197fs
XH_P36	5	171765667	171765667	Silent	SNP	G	A	G	SH3PXD2B	c.C2442T	p.G814G
XH_P36	5	174868783	174868783	In_Frame_Ins	INS	-	CAAATACATTCCA	-	DRD1	320insCTGGAAT	440delinsHWNVFG
XH_P36	5	174869195	174869195	In_Frame_Ins	INS	-	ATGTTT	-	DRD1	907_908insAAAC	p.T303delinsKTS
XH_P36	5	175775279	175775279	Silent	SNP	C	T	C	KIAA1191	c.G297A	p.P99P
XH_P36	5	175843200	175843200	Silent	SNP	C	T	C	CLTB	c.G165A	p.A55A
XH_P36	5	176813456	176813456	Missense_Mutation	SNP	G	A	G	SLC34A1	c.G421A	p.A141T
XH_P36	5	176863433	176863433	Frame_Shift_Del	DEL	G	-	G	GRK6	c.1306delG	p.G436fs
XH_P36	5	176885292	176885292	Missense_Mutation	SNP	C	T	C	DBN1	c.G1543A	p.V515M
XH_P36	5	177548988	177548988	Missense_Mutation	SNP	T	C	T	N4BP3	c.T1621C	p.S541P
XH_P36	5	178309945	178309945	In_Frame_Ins	INS	-	ATTTTAGGTGTG	-	ZNF354B	493insATTTTAG	G164delinsGILGV
XH_P36	5	178460353	178460353	Frame_Shift_Ins	INS	-	ATTCTTTGCATGC	-	ZNF879	405insATTCTTT	p.H468fs
XH_P36	5	179151749	179151749	Frame_Shift_Ins	INS	-	TTACATGTTCATC	-	CANX	TTTTGTTTACATC	p.K537fs
XH_P36	6	15374448	15374448	Missense_Mutation	SNP	C	T	C	JARID2	c.C146T	p.A49V
XH_P36	6	21594968	21594968	Missense_Mutation	SNP	T	C	T	SOX4	c.T203C	p.V68A
XH_P36	6	22290504	22290504	Missense_Mutation	SNP	G	A	G	PRL	c.C391T	p.R131C
XH_P36	6	24576806	24576806	Nonsense_Mutation	INS	-	GCCTTCAGACTT	-	KIAA0319	89insAAAAGTCT	.S463delinsSKVX
XH_P36	6	25606400	25606400	Nonsense_Mutation	INS	-	TTAAGAGTATTT	-	CARMIL1	GATGTTTTAAGA	43_S1244delinsSRMFX
XH_P36	6	25606402	25606402	Missense_Mutation	SNP	A	T	A	CARMIL1	c.A3730T	p.S1244C
XH_P36	6	25799032	25799032	Missense_Mutation	SNP	T	C	T	SLC17A1	c.A1385G	p.K462R
XH_P36	6	27861432	27861432	Missense_Mutation	SNP	C	A	C	HIST1H2BO	c.C192A	p.N64K
XH_P36	6	28212908	28212908	Frame_Shift_Del	DEL	T	-	T	ZKSCAN4	c.1159delA	p.T387fs
XH_P36	6	28359148	28359148	Frame_Shift_Ins	INS	-	GAAAAATGCTATC	-	ZSCAN12	9insCTGATAGCA	p.C307fs
XH_P36	6	33653236	33653236	Missense_Mutation	SNP	C	T	C	ITPR3	c.C5408T	p.T1803M
XH_P36	6	35426163	35426163	Silent	SNP	T	C	T	FANCE	c.T1059C	p.P353P
XH_P36	6	36931061	36931061	Nonsense_Mutation	INS	-	GAGTCTGATGC	-	PI16	944insGAGTCTC	15_P316delinsRVX
XH_P36	6	41652798	41652798	Nonsense_Mutation	SNP	G	A	G	TFEB	c.C715T	p.R239X
XH_P36	6	42073370	42073372	In_Frame_Del	DEL	AGG	-	AGG	C6orf132	c.2278_2280del	p.760_760del
XH_P36	6	42235991	42235991	Missense_Mutation	SNP	G	C	G	TRERF1	c.C1338G	p.S446R
XH_P36	6	42237313	42237313	Missense_Mutation	SNP	G	T	G	TRERF1	c.C16A	p.L6M
XH_P36	6	43307375	43307375	Frame_Shift_Ins	INS	-	AGTGAGA	-	ZNF318	60_4361insTCTC	p.P1454fs
XH_P36	6	43418816	43418816	Missense_Mutation	SNP	C	T	C	DLK2	c.G502A	p.A168T
XH_P36	6	44223300	44223300	Missense_Mutation	SNP	G	A	G	SLC35B2	c.C43T	p.R15C
XH_P36	6	44269193	44269193	Frame_Shift_Del	DEL	C	-	C	AARS2	c.2607delG	p.K869fs
XH_P36	6	46111312	46111312	Nonsense_Mutation	INS	-	TAGGACAAAATA	-	ENPP4	298insTTAGGAC	33_V434delinsFRTKX
XH_P36	6	46111314	46111314	Frame_Shift_Ins	INS	-	TGCTTCTA	-	ENPP4	99_1300insTGCT	p.S433fs
XH_P36	6	46826739	46826739	Frame_Shift_Del	DEL	C	-	C	ADGRF5	c.2901delG	p.G967fs
XH_P36	6	51947326	51947326	Nonsense_Mutation	INS	-	ACGGAGGATGAC	-	PKHD1	insACTCTCATCC	.V49delinsTLILRX
XH_P36	6	52343894	52343894	Silent	SNP	C	T	C	EFHC1	c.C1338T	p.T446T
XH_P36	6	53363590	53363590	Silent	SNP	T	C	T	GCLC	c.A1764G	p.V588V
XH_P36	6	56480539	56480539	In_Frame_Ins	INS	-	TCTTCCTAA	-	DST	5_7726insTTAGC	.I2576delinsLGRI
XH_P36	6	56480541	56480541	In_Frame_Ins	INS	-	GGACATGTTTAA	-	DST	7724insTTAAAC	52575delinsFKHVP
XH_P36	6	62995837	62995837	Missense_Mutation	SNP	T	C	T	KHDRBS2	c.A17G	p.Y6C
XH_P36	6	64395565	64395565	Nonsense_Mutation	INS	-	TTTTTCACAGATT	-	PHF3	943insGTTTTTCA	E648delinsGFSQIX
XH_P36	6	66012756	66012756	Missense_Mutation	SNP	G	T	G	LOC441155	c.G342T	p.K114N
XH_P36	6	73952255	73952255	Splice_Site	INS	-	TCATCA	-	KHDC1	.	.
XH_P36	6	76660622	76660622	Nonsense_Mutation	INS	-	TCTTTGTTGTTAT	-	IMPG1	ATAAAATAACAA	416_G417delinsQX
XH_P36	6	79675743	79675743	Missense_Mutation	SNP	G	A	G	PHIP	c.C3236T	p.A1079V
XH_P36	6	80197020	80197020	Frame_Shift_Ins	INS	-	GTGAAGGAAGAA	-	LCA5	1795insCTTCTTC	p.E599fs
XH_P36	6	80751910	80751910	Frame_Shift_Del	DEL	A	-	A	TTK	c.2562delA	p.G854fs
XH_P36	6	83847704	83847704	Frame_Shift_Ins	INS	-	TTTAATCGTCTCT	-	DOPEY1	917insTTTTAATC	p.N1306fs
XH_P36	6	85446693	85446693	In_Frame_Ins	INS	-	ACATATTTTCTTT	-	TBX18	sAGAAAAAGAA	512delinsRKRKYVRQ
XH_P36	6	85446694	85446694	Missense_Mutation	SNP	G	T	G	TBX18	c.C1533A	p.N511K
XH_P36	6	86324630	86324630	Frame_Shift_Ins	INS	-	TTTCCCTTTATGTA	-	SYNCRIP	GATATATACATAA	p.Q537fs
XH_P36	6	88318837	88318837	Frame_Shift_Ins	INS	-	A	-	ORC3	c.175dupA	p.S58fs
XH_P36	6	89793782	89793782	Frame_Shift_Ins	INS	-	A	-	PNRC1	c.851_852insA	p.F284fs
XH_P36	6	89793784	89793784	Frame_Shift_Ins	INS	-	TTTCTGTCTGTTT	-	PNRC1	TTGCTTTTCTGTC	p.S285fs
XH_P36	6	90459404	90459404	Frame_Shift_Ins	INS	-	GTAGTAAGAATG	-	MDN1	AACATTTCATTC	p.T1158fs
XH_P36	6	102074256	102074256	Silent	SNP	C	A	C	GRIK2	c.C285A	p.A95A
XH_P36	6	116263666	116263666	Nonsense_Mutation	SNP	G	A	G	FRK	c.C1429T	p.R477X
XH_P36	6	116720492	116720492	Missense_Mutation	SNP	G	A	G	DSE	c.G79A	p.E27K
XH_P36	6	116938286	116938286	Frame_Shift_Ins	INS	-	GTCTTGT	-	RSPH4A	00_501insGTCT	p.S167fs
XH_P36	6	117609822	117609822	In_Frame_Ins	INS	-	TTTGGTTTCAGTT	-	ROS1	877insCAACTGA	2293delinsQLKPNG
XH_P36	6	118229019	118229019	Frame_Shift_Ins	INS	-	C	-	SLC35F1	c.131dupC	p.S44fs
XH_P36	6	128403762	128403762	Frame_Shift_Ins	INS	-	TTTACAAATAGGA	-	PTPRK	nsATCAATCCTAT	p.S404fs
XH_P36	6	130031231	130031231	Frame_Shift_Del	DEL	G	-	G	ARHGAP18	c.51delC	p.P17fs
XH_P36	6	134323264	134323264	Missense_Mutation	SNP	A	C	A	SLC2A12	c.T1571G	p.L524R
XH_P36	6	136599393	136599393	Missense_Mutation	SNP	G	A	G	BCLAF1	c.C620T	p.S207F
XH_P36	6	139097330	139097330	Frame_Shift_Del	DEL	A	-	A	CCDC28A	c.343delA	p.K115fs
XH_P36	6	142409404	142409404	Missense_Mutation	SNP	A	G	A	NMBR	c.T392C	p.V131A
XH_P36	6	142711439	142711439	Missense_Mutation	SNP	C	T	C	ADGRG6	c.C1183T	p.R395C
XH_P36	6	146988003	146988003	Silent	SNP	G	A	G	ADGB	c.G804A	p.A268A
XH_P36	6	147136338	147136338	In_Frame_Ins	INS	-	TTCCTTTACCCT	-	ADGB	4990insTTCCTT	K1663delinsKFLYP
XH_P36	6	155153431	155153431	Frame_Shift_Ins	INS	-	G	-	SCAF8	c.2718_2719ins	p.Q906fs
XH_P36	6	155153432	155153432	Frame_Shift_Ins	INS	-	ATTTCAGAATT	-	SCAF8	2720insATTTCA	p.N907fs
XH_P36	6	155571012	155571012	Missense_Mutation	SNP	C	T	C	TIAM2	c.C635T	p.A212V
XH_P36	6	158508008	158508008	Frame_Shift_Ins	INS	-	C	-	SYNJ2	c.2620dupC	p.R873fs
XH_P36	6	158900956	158900956	Frame_Shift_Ins	INS	-	C	-	TULP4	c.1201dupC	p.L400fs
XH_P36	6	160679514	160679514	Silent	SNP	G	A	G	SLC22A2	c.C276T	p.Y92Y
XH_P36	6	163149397	163149397	Missense_Mutation	SNP	G	T	G	PACRG	c.G130T	p.V44F

XH_P36	6	170110415	170110415	Missense_Mutation	SNP	G	T	G	PHF10	c.C1030A	p.Q344K
XH_P36	6	170110416	170110416	Nonsense_Mutation	INS	-	TTTGCTTTATTTC	-	PHF10	029insGAAATA/	343_Q344delinsRKX
XH_P36	7	1482030	1482030	Silent	SNP	C	T	C	MICALL2	c.G1509A	p.S503S
XH_P36	7	4841442	4841442	Frame_Shift_Del	DEL	G	-	G	RADIL	c.2684delC	p.P895fs
XH_P36	7	4862166	4862166	Missense_Mutation	SNP	C	T	C	RADIL	c.G1474A	p.A492T
XH_P36	7	5780887	5780887	Missense_Mutation	SNP	C	T	C	RNF216	c.G761A	p.R254Q
XH_P36	7	5781027	5781027	Frame_Shift_Ins	INS	-	AGTTAGCAAGTG	-	RNF216	621insTCACTTG(	p.E207fs
XH_P36	7	6502731	6502731	Missense_Mutation	SNP	G	A	G	KDELR2	c.C427T	p.R143W
XH_P36	7	6662266	6662266	Missense_Mutation	SNP	G	T	G	ZNF853	c.G1644T	p.K548N
XH_P36	7	7612451	7612451	Frame_Shift_Ins	INS	-	TCTATCAACATG	-	MIOS	46insATCTATCA	p.Q115fs
XH_P36	7	12420215	12420215	Frame_Shift_Ins	INS	-	TATTTTCTTTTT(	-	VWDE	AAAACAAAAAGA	p.R229fs
XH_P36	7	13975433	13975433	Frame_Shift_Ins	INS	-	GTCATTTATAAA(	-	ETV1	0insATGTTTATA/	p.S94fs
XH_P36	7	23728890	23728890	Missense_Mutation	SNP	A	T	A	FAM221A	c.A68T	p.Y23F
XH_P36	7	24932053	24932053	Frame_Shift_Del	DEL	T	-	T	OSBPL3	c.39delA	p.K13fs
XH_P36	7	27183075	27183075	Missense_Mutation	SNP	C	T	C	HOXA5	c.G152A	p.G51D
XH_P36	7	30705147	30705147	Missense_Mutation	SNP	G	A	G	CRHR2	c.C421T	p.R141W
XH_P36	7	31682378	31682378	Missense_Mutation	SNP	C	T	C	CCDC129	c.C1394T	p.S465F
XH_P36	7	31793063	31793063	Missense_Mutation	SNP	T	C	T	PDE1C	c.A2065G	p.M689V
XH_P36	7	37889877	37889877	Frame_Shift_Ins	INS	-	A	-	NME8	c.10dupA	p.S3fs
XH_P36	7	44560659	44560659	Silent	SNP	C	T	C	NPC1L1	c.G3012A	p.S1004S
XH_P36	7	45124001	45124001	Missense_Mutation	SNP	T	C	T	NACAD	c.A1778G	p.D593G
XH_P36	7	45141471	45141471	Missense_Mutation	SNP	G	A	G	TBRG4	c.C1190T	p.A397V
XH_P36	7	53103788	53103788	Missense_Mutation	SNP	G	A	G	POM121L12	c.G424A	p.A142T
XH_P36	7	56156631	56156631	Missense_Mutation	SNP	T	C	T	PHKG1	c.A4G	p.T2A
XH_P36	7	57528754	57528754	In_Frame_Ins	INS	-	ATCTTCTCA	-	ZNF716	7_588insATCTT(	.M196delinsISSQ
XH_P36	7	57528755	57528755	Nonsense_Mutation	INS	-	ACTTTTGGAGT	-	ZNF716	isTATCACTTTT(	6_L197delinsMYHFLEX
XH_P36	7	63674391	63674391	Missense_Mutation	SNP	G	A	G	ZNF735	c.G167A	p.G56D
XH_P36	7	64451956	64451956	Nonsense_Mutation	SNP	C	T	C	ERV3-1	c.G1449A	p.W483X
XH_P36	7	70255576	70255576	In_Frame_Ins	INS	-	CCA	-	AUTS2	3302_3303insC(	.S1101delinsSH
XH_P36	7	70597900	70597900	Missense_Mutation	SNP	G	A	G	GALNT17	c.G112A	p.A38T
XH_P36	7	72436399	72436399	Missense_Mutation	SNP	C	T	C	TRIM74	c.G290A	p.S97N
XH_P36	7	73814818	73814818	Missense_Mutation	SNP	C	T	C	CLIP2	c.C2894T	p.A965V
XH_P36	7	77379007	77379007	Frame_Shift_Ins	INS	-	TTCTTTTTCTTCT	-	RSBN1L	insTTTTTCTTTTT	p.P324fs
XH_P36	7	77379009	77379009	Silent	SNP	A	C	A	RSBN1L	c.A972C	p.P324P
XH_P36	7	77408086	77408086	Silent	SNP	C	T	C	RSBN1L	c.C2142T	p.S714S
XH_P36	7	86813908	86813908	Nonsense_Mutation	INS	-	ACCCTCTGGT	-	DMTF1	_753insACCCTC	V251_T252delinsX
XH_P36	7	89939531	89939531	Frame_Shift_Del	DEL	A	-	A	CFAP69	c.2805delA	p.P935fs
XH_P36	7	91631743	91631743	Frame_Shift_Ins	INS	-	A	-	AKAP9	c.2513dupA	p.Q838fs
XH_P36	7	91631745	91631745	Frame_Shift_Ins	INS	-	GGTTTTCTTTTCA	-	AKAP9	2515insGGTTTT(	p.Q838fs
XH_P36	7	94037202	94037202	Missense_Mutation	SNP	C	G	C	COL1A2	c.C638G	p.T213R
XH_P36	7	94919558	94919558	Nonsense_Mutation	INS	-	AACTAGTTACAT	-	PPP1R9A	3217insAACTAG	072_S1073delinsKNX
XH_P36	7	94919560	94919560	Missense_Mutation	SNP	C	G	C	PPP1R9A	c.C3218G	p.S1073C
XH_P36	7	99621198	99621198	Silent	SNP	C	T	C	ZKSCAN1	c.C69T	p.I23I
XH_P36	7	99758575	99758575	Frame_Shift_Ins	INS	-	TTCCCCCAGA	-	GAL3ST4	_437insTCTGGG	p.Q146fs
XH_P36	7	100451920	100451920	Missense_Mutation	SNP	C	T	C	SLC12A9	c.C101T	p.A34V
XH_P36	7	100552727	100552727	Missense_Mutation	SNP	G	T	G	MUC3A	c.G3308T	p.R1103L
XH_P36	7	100641373	100641373	Missense_Mutation	SNP	G	A	G	MUC12	c.G7529A	p.S2510N
XH_P36	7	100676246	100676246	Missense_Mutation	SNP	T	C	T	MUC17	c.T1549C	p.S517P
XH_P36	7	100677523	100677523	Missense_Mutation	SNP	A	C	A	MUC17	c.A2826C	p.R942S
XH_P36	7	100677944	100677944	Missense_Mutation	SNP	T	C	T	MUC17	c.T3247C	p.S1083P
XH_P36	7	100681171	100681171	Missense_Mutation	SNP	C	A	C	MUC17	c.C6474A	p.D2158E
XH_P36	7	100681172	100681172	Missense_Mutation	SNP	A	G	A	MUC17	c.A6475G	p.R2159G
XH_P36	7	100681278	100681278	Missense_Mutation	SNP	A	C	A	MUC17	c.A6581C	p.N2194T
XH_P36	7	100683382	100683382	Silent	SNP	T	C	T	MUC17	c.T8685C	p.D2895D
XH_P36	7	100683723	100683723	Missense_Mutation	SNP	G	C	G	MUC17	c.G9026C	p.R3009T
XH_P36	7	100730930	100730930	Frame_Shift_Del	DEL	G	-	G	TRIM56	c.337delG	p.G113fs
XH_P36	7	100816663	100816663	Missense_Mutation	SNP	G	A	G	NAT16	c.C451T	p.H151Y
XH_P36	7	102964991	102964991	Frame_Shift_Ins	INS	-	T	-	DNAJC2	c.590dupA	p.N197fs
XH_P36	7	107234511	107234511	Nonsense_Mutation	INS	-	TCTATAAATGGA	-	BCAP29	GGTGCTCTATAA	N103delinsSRCSINGHX
XH_P36	7	112407656	112407656	Missense_Mutation	SNP	T	C	T	TMEM168	c.A1690G	p.I564V
XH_P36	7	116397525	116397525	Missense_Mutation	SNP	C	T	C	MET	c.C607T	p.H203Y
XH_P36	7	117397929	117397929	Missense_Mutation	SNP	C	A	C	CTTNBP2	c.G3268T	p.G1090C
XH_P36	7	122033359	122033359	Nonsense_Mutation	INS	-	TTTATTTGAGTTT	-	CADPS2	CTTAAAAACTCA	N971_S972delinsX
XH_P36	7	127978355	127978355	Missense_Mutation	SNP	G	T	G	RBM28	c.C490A	p.L164I
XH_P36	7	128455922	128455922	Silent	SNP	C	T	C	CCDC136	c.C3300T	p.D1100D
XH_P36	7	128485259	128485259	Missense_Mutation	SNP	C	T	C	FLNC	c.C3740T	p.A1247V
XH_P36	7	128502976	128502976	Silent	SNP	G	A	G	ATP6V1F	c.G18A	p.K6K
XH_P36	7	130040097	130040097	Splice_Site	INS	-	GATAATGTTTGG	-	CEP41	.	.
XH_P36	7	131012702	131012702	Missense_Mutation	SNP	T	G	T	MKLN1	c.T44G	p.L15R
XH_P36	7	131831415	131831415	Missense_Mutation	SNP	G	A	G	PLXNA4	c.C4909T	p.R1637W
XH_P36	7	137569816	137569816	Missense_Mutation	SNP	C	T	C	CREB3L2	c.G1006A	p.G336R
XH_P36	7	138363260	138363260	Silent	SNP	C	T	C	SVOPL	c.G93A	p.T31T
XH_P36	7	139724377	139724377	Missense_Mutation	SNP	A	T	A	PARP12	c.T2089A	p.F697I
XH_P36	7	141631618	141631618	Frame_Shift_Ins	INS	-	TTTTTAATAAATG	-	CLEC5A	AGAAAACATTTA	p.L95fs
XH_P36	7	141750074	141750074	In_Frame_Ins	INS	-	TCCTTATGGATGA	-	MGAM	nsCTTTTCCTTAT	76delinsSFSLWMNN
XH_P36	7	141795497	141795497	Missense_Mutation	SNP	C	T	C	MGAM	c.C4903T	p.R1635W
XH_P36	7	141920186	141920186	Missense_Mutation	SNP	G	A	G	MGAM2	c.G5875A	p.D1959N
XH_P36	7	142565462	142565462	Missense_Mutation	SNP	C	T	C	EPHB6	c.C971T	p.A324V
XH_P36	7	150325284	150325284	Missense_Mutation	SNP	C	T	C	GIMAP6	c.G178A	p.V60I
XH_P36	7	150417615	150417615	Missense_Mutation	SNP	C	T	C	GIMAP1	c.C523T	p.R175C
XH_P36	7	150761288	150761288	Splice_Site	SNP	G	A	G	SLC4A2	.	.
XH_P36	7	151874148	151874148	Frame_Shift_Del	DEL	T	-	T	KMT2C	c.8390delA	p.K2797fs
XH_P36	7	151875020	151875020	Frame_Shift_Ins	INS	-	GGCCAGACTT	-	KMT2C	_7518insAAGTC	p.Q2506fs
XH_P36	7	151945313	151945313	Silent	SNP	A	G	A	KMT2C	c.T2206C	p.L736L
XH_P36	7	152345760	152345760	Frame_Shift_Del	DEL	A	-	A	XRCC2	c.810delT	p.F270fs
XH_P36	7	158527147	158527147	Missense_Mutation	SNP	C	T	C	ESYT2	c.G2602A	p.A868T
XH_P36	7	158937413	158937413	Frame_Shift_Del	DEL	G	-	G	VIPR2	c.51delC	p.P17fs
XH_P36	8	2813125	2813125	Missense_Mutation	SNP	G	A	G	CSMD1	c.C9980T	p.S3327L
XH_P36	8	2824227	2824227	Missense_Mutation	SNP	T	A	T	CSMD1	c.A8965T	p.I2989F
XH_P36	8	3226804	3226804	Silent	SNP	C	G	C	CSMD1	c.G2871C	p.T957T
XH_P36	8	3265726	3265726	Missense_Mutation	SNP	G	A	G	CSMD1	c.C1766T	p.T589M
XH_P36	8	6289099	6289099	Frame_Shift_Del	DEL	A	-	A	MCPH1	c.313delA	p.K105fs
XH_P36	8	7806651	7806651	Missense_Mutation	SNP	G	A	G	ZNF705B	c.G13A	p.E5K
XH_P36	8	8234868	8234868	In_Frame_Ins	INS	-	GCCACT	-	PRAG1	50_1051insAGT	.A351delinsSGA
XH_P36	8	9953753	9953753	Splice_Site	SNP	G	A	G	MSRA	.	.
XH_P36	8	10755632	10755632	Missense_Mutation	SNP	T	C	T	XKR6	c.A1756G	p.K586E
XH_P36	8	15998555	15998555	Splice_Site	INS	-	ATGCTCATACCT(	-	MSR1	.	.
XH_P36	8	17402081	17402081	Frame_Shift_Del	DEL	T	-	T	SLC7A2	c.498delT	p.D166fs

XH_P36	8	21981180	21981180	Missense_Mutation	SNP	G	A	G	HR	c.C1897T	p.R633W
XH_P36	8	22548022	22548022	Frame_Shift_Del	DEL	G	-	G	EGR3	c.1014delC	p.P338fs
XH_P36	8	23560102	23560102	Silent	SNP	G	A	G	NKX2-6	c.C768T	p.Y256Y
XH_P36	8	36644913	36644913	Frame_Shift_Ins	INS	-	ATATTTCATAAA	-	KCNU1	ATTCAATATTTCA	p.L95fs
XH_P36	8	37553551	37553551	Silent	SNP	C	T	C	ZNF703	c.C54T	p.S18S
XH_P36	8	37822872	37822872	Silent	SNP	G	A	G	ADRB3	c.C1116T	p.C372C
XH_P36	8	38186894	38186894	Splice_Site	SNP	A	G	A	NSD3	.	.
XH_P36	8	38205127	38205127	Nonsense_Mutation	INS	-	CTGATGACCTCG	-	NSD3	insAACCGAGGT	S188_K189delinsX
XH_P36	8	38874860	38874860	Missense_Mutation	SNP	C	A	C	ADAM9	c.C533A	p.S178Y
XH_P36	8	42040260	42040260	Silent	SNP	G	A	G	PLAT	c.C513T	p.G171G
XH_P36	8	43037381	43037381	Missense_Mutation	SNP	C	A	C	HGSNAT	c.C1106A	p.P369H
XH_P36	8	61736495	61736495	Missense_Mutation	SNP	C	T	C	CHD7	c.C3298T	p.R1100C
XH_P36	8	67417717	67417717	Silent	SNP	C	T	C	C8orf46	c.C234T	p.T78T
XH_P36	8	67873649	67873649	Missense_Mutation	SNP	C	T	C	TCF24	c.G352A	p.G118S
XH_P36	8	68151020	68151020	Missense_Mutation	SNP	T	C	T	ARFGEF1	c.A3088G	p.I1030V
XH_P36	8	72951256	72951256	Nonsense_Mutation	INS	-	ATCAGGACTTTAT	-	TRPA1	TTTATAATAAAG	13_Y714delinsAGYLX
XH_P36	8	77619814	77619814	Missense_Mutation	SNP	C	T	C	ZFHX4	c.C2624T	p.A875V
XH_P36	8	86019547	86019547	Missense_Mutation	SNP	C	T	C	LRRCC1	c.C17T	p.A6V
XH_P36	8	92030736	92030736	Missense_Mutation	SNP	C	T	C	TMEM55A	c.G368A	p.R123Q
XH_P36	8	95895095	95895095	Nonsense_Mutation	INS	-	TGCTTTAAGAGT	-	CCNE2	AAAGAACTCTTA	287delinsKSQRTLKAELX
XH_P36	8	98703408	98703408	Frame_Shift_Ins	INS	-	ATGATTGCTTGA	-	MTDH	CATTTCTATGATT	p.S347fs
XH_P36	8	99146236	99146236	Silent	SNP	G	A	G	POP1	c.G795A	p.A265A
XH_P36	8	100789078	100789078	Nonsense_Mutation	INS	-	GCCACTGCTCGCT	-	VPS13B	ACTTTGCCACTG	V2467delinsRVDSCFTPGX
XH_P36	8	100831011	100831011	Missense_Mutation	SNP	G	T	G	VPS13B	c.G8591T	p.R2864M
XH_P36	8	104897908	104897908	Nonsense_Mutation	SNP	C	T	C	RIMS2	c.C505T	p.R169X
XH_P36	8	105361517	105361517	Nonsense_Mutation	INS	-	GGAGAC	-	DCSTAMP	37_738insGGAG	p.Y246delinsX
XH_P36	8	105361519	105361519	Missense_Mutation	SNP	A	T	A	DCSTAMP	c.A739T	p.I247F
XH_P36	8	105521191	105521191	Missense_Mutation	SNP	T	G	T	LRP12	c.A191C	p.N64T
XH_P36	8	108970494	108970494	Frame_Shift_Ins	INS	-	AAAAA	-	RSPO2	228_229insTTTT	p.G77fs
XH_P36	8	108970495	108970495	In_Frame_Ins	INS	-	AAGTATTTA	-	RSPO2	7_228insTAAAT	p.E76delinsDKYL
XH_P36	8	110457532	110457532	Frame_Shift_Ins	INS	-	AACTT	-	PKHD1L1	434_5435insAA	p.V1812fs
XH_P36	8	110464995	110464995	Frame_Shift_Ins	INS	-	G	-	PKHD1L1	c.6557dupG	p.W2186fs
XH_P36	8	110478806	110478806	Splice_Site	SNP	G	T	G	PKHD1L1	.	.
XH_P36	8	110587336	110587336	Silent	SNP	G	A	G	SYBU	c.C1401T	p.G467G
XH_P36	8	110986446	110986446	Missense_Mutation	SNP	A	T	A	KCNV1	c.T172A	p.S58T
XH_P36	8	125326260	125326260	Missense_Mutation	SNP	A	G	A	TMEM65	c.T664C	p.F222L
XH_P36	8	125989809	125989809	In_Frame_Ins	INS	-	TTGATAAGG	-	ZNF572	9_1300insTTGAT	p.T433delinsTLIR
XH_P36	8	125989810	125989810	In_Frame_Ins	INS	-	TTTCTCCTGTATG	-	ZNF572	301insTTTTCTCC	434delinsLFSCMY
XH_P36	8	128750864	128750864	Missense_Mutation	SNP	C	T	C	MYC	c.C401T	p.P134L
XH_P36	8	133764167	133764167	Missense_Mutation	SNP	T	C	T	TMEM71	c.A178G	p.T60A
XH_P36	8	139606411	139606411	Silent	SNP	G	A	G	COL22A1	c.C4464T	p.P1488P
XH_P36	8	139638440	139638440	Missense_Mutation	SNP	C	T	C	COL22A1	c.G3710A	p.G1237E
XH_P36	8	142151365	142151365	Frame_Shift_Ins	INS	-	GTCGAAGTCTA	-	DENND3	_326insGTCGAA	p.A109fs
XH_P36	8	143956491	143956491	Missense_Mutation	SNP	C	T	C	CYP11B1	c.G1280A	p.R427H
XH_P36	8	144732584	144732584	Frame_Shift_Ins	INS	-	GA	-	ZNF623	c.542_543insGA	p.C181fs
XH_P36	8	144732585	144732585	Frame_Shift_Ins	INS	-	CTTCC	-	ZNF623	543_544insCTT	p.C181fs
XH_P36	8	144940503	144940503	Missense_Mutation	SNP	C	T	C	EPPK1	c.G6919A	p.V2307I
XH_P36	8	144998402	144998402	Missense_Mutation	SNP	G	A	G	PLEC	c.C5653T	p.R1885C
XH_P36	8	145109481	145109481	Frame_Shift_Del	DEL	C	-	C	OPLAH	c.2669delG	p.G890fs
XH_P36	8	145154303	145154303	Missense_Mutation	SNP	C	T	C	SHARPIN	c.G799A	p.V267M
XH_P36	8	145650341	145650341	Silent	SNP	G	A	G	VPS28	c.C288T	p.C96C
XH_P36	8	145693999	145693999	Silent	SNP	G	A	G	KIFC2	c.G969A	p.R323R
XH_P36	8	145737394	145737394	Missense_Mutation	SNP	G	A	G	RECQL4	c.C3293T	p.T1098I
XH_P36	9	977357	977357	Missense_Mutation	SNP	A	C	A	DMRT3	c.A356C	p.Q119P
XH_P36	9	1051712	1051712	Frame_Shift_Ins	INS	-	C	-	DMRT2	c.100dupC	p.T33fs
XH_P36	9	2047307	2047307	Missense_Mutation	SNP	C	T	C	SMARCA2	c.C869T	p.A290V
XH_P36	9	5233659	5233659	Missense_Mutation	SNP	G	A	G	INSL4	c.G202A	p.V68M
XH_P36	9	5742994	5742994	Missense_Mutation	SNP	A	G	A	RIC1	c.A1027G	p.T343A
XH_P36	9	5968044	5968044	Frame_Shift_Del	DEL	T	-	T	KIAA2026	c.2187delA	p.K729fs
XH_P36	9	6007719	6007719	Silent	SNP	G	T	G	KIAA2026	c.C69A	p.A23A
XH_P36	9	8524968	8524968	Silent	SNP	G	A	G	PTPRD	c.C627T	p.S209S
XH_P36	9	14841546	14841546	Frame_Shift_Ins	INS	-	TTTGAAAGACTT	-	FREM1	80insAAAAGTCT	p.G594fs
XH_P36	9	14841548	14841548	Nonsense_Mutation	INS	-	ATCTATGTATTC	-	FREM1	insCACTGAATAC	93_G594delinsTLNTX
XH_P36	9	16738425	16738425	Frame_Shift_Ins	INS	-	CACTTCTT	-	BNC2	1_62insAAGAAG	p.L21fs
XH_P36	9	18906821	18906821	Missense_Mutation	SNP	G	A	G	ADAMTSL1	c.G5093A	p.R1698H
XH_P36	9	27220063	27220063	Silent	SNP	C	T	C	TEK	c.C2676T	p.C892C
XH_P36	9	27548256	27548256	Missense_Mutation	SNP	C	T	C	C9orf72	c.G1424A	p.R475Q
XH_P36	9	34623991	34623991	Missense_Mutation	SNP	C	T	C	ARID3C	c.G445A	p.A149T
XH_P36	9	35091693	35091693	Frame_Shift_Del	DEL	G	-	G	PIGO	c.2191delC	p.R731fs
XH_P36	9	35103049	35103049	Silent	SNP	T	G	T	STOML2	c.A43C	p.R15R
XH_P36	9	35105796	35105796	Nonsense_Mutation	INS	-	TTTTAAATTTTTAT	-	FAM214B	CATACATAAAAA	450delinsYICTYIHKNLKINX
XH_P36	9	35404027	35404027	Nonsense_Mutation	INS	-	CAAGATAGAAGT	-	UNC13B	74insACAAGATA	S1591delinsSTRX
XH_P36	9	35607405	35607405	In_Frame_Ins	INS	-	ACCCAAAGCCCT	-	TESK1	620insACCCAAA	R207delinsNPKPW
XH_P36	9	35722212	35722212	Frame_Shift_Ins	INS	-	CCTAAGATTCCCA	-	TLN1	52insTGGGAAT	p.K284fs
XH_P36	9	40772354	40772354	Missense_Mutation	SNP	G	A	G	ZNF658	c.C2921T	p.T974M
XH_P36	9	67968558	67968558	Missense_Mutation	SNP	C	T	C	ANKRD20A1	c.C2117T	p.T706I
XH_P36	9	67968560	67968560	Missense_Mutation	SNP	C	A	C	ANKRD20A1	c.C2119A	p.Q707K
XH_P36	9	72965343	72965343	Missense_Mutation	SNP	G	T	G	SMC5	c.G3121T	p.A1041S
XH_P36	9	72965344	72965344	Frame_Shift_Ins	INS	-	TTTCACCTCCA	-	SMC5	_3123insTTTCAC	p.A1041fs
XH_P36	9	73399023	73399023	Silent	SNP	G	A	G	TRPM3	c.C1146T	p.G382G
XH_P36	9	74332948	74332948	Frame_Shift_Ins	INS	-	GCTGAATTTTGAA	-	TMEM2	TTATCGTTCAAAA	p.A709fs
XH_P36	9	84606936	84606936	Silent	SNP	T	C	T	SPATA31D1	c.T1551C	p.P517P
XH_P36	9	84607834	84607834	Missense_Mutation	SNP	T	G	T	SPATA31D1	c.T2449G	p.S817A
XH_P36	9	90582462	90582462	Frame_Shift_Ins	INS	-	G	-	CDK20	c.892dupC	p.H298fs
XH_P36	9	91991856	91991856	Missense_Mutation	SNP	C	T	C	SEMA4D	c.G1774A	p.A592T
XH_P36	9	95610784	95610784	Frame_Shift_Ins	INS	-	TTTGGAGCCTAT	-	ZNF484	177insGATAGGC	p.V59fs
XH_P36	9	96422612	96422612	Frame_Shift_Del	DEL	A	-	A	PHF2	c.1468delA	p.K490fs
XH_P36	9	99381507	99381507	Missense_Mutation	SNP	T	C	T	CDC14B	c.A154G	p.I52V
XH_P36	9	100850088	100850088	Silent	SNP	C	T	C	TRIM14	c.G993A	p.A331A
XH_P36	9	105767905	105767905	Missense_Mutation	SNP	C	A	C	CYLC2	c.C992A	p.A331E
XH_P36	9	107298869	107298869	In_Frame_Ins	INS	-	GGCATTTGATCG	-	OR13C3	226insCGATCAA	p.I76delinsRSNAI
XH_P36	9	113192205	113192205	Silent	SNP	T	C	T	SVEP1	c.A5610G	p.T1870T
XH_P36	9	114090063	114090063	Nonsense_Mutation	INS	-	TATGAAGAAATT	-	OR2K2	51insGCAATTTCT	p.Y217delinsX
XH_P36	9	114449150	114449150	In_Frame_Ins	INS	-	ATATGGCATGTA	-	C9orf84	CAATGTTACATG	373delinsAMLHAIFHC
XH_P36	9	115932129	115932129	Nonsense_Mutation	INS	-	GTGTCAGGGAA	-	FKBP15	2860insCTTCCC	p.R954delinsLPX
XH_P36	9	116276868	116276868	Silent	SNP	G	A	G	RGS3	c.G765A	p.T255T
XH_P36	9	117139586	117139586	Missense_Mutation	SNP	C	A	C	AKNA	c.G144T	p.Q48H
XH_P36	9	117848395	117848395	Nonsense_Mutation	SNP	G	A	G	TNC	c.C1615T	p.Q539X

XH_P36	9	117848781	117848781	Missense_Mutation	SNP	T	C	T	TNC	c.A1229G	p.N410S
XH_P36	9	127920568	127920568	Nonsense_Mutation	SNP	G	A	G	PPP6C	c.C265T	p.R89X
XH_P36	9	130947831	130947831	Nonsense_Mutation	SNP	G	A	G	CIZ1	c.C511T	p.R171X
XH_P36	9	131483691	131483691	Missense_Mutation	SNP	C	T	C	ZDHHC12	c.G601A	p.V201I
XH_P36	9	131709317	131709317	Missense_Mutation	SNP	G	T	G	DOLK	c.C266A	p.P89H
XH_P36	9	135277175	135277175	In_Frame_Ins	INS	-	ACAGTCCCAACC	-	TTF1	insTGAGGTTGG	345delinsVRLGLWK
XH_P36	9	135926232	135926232	Missense_Mutation	SNP	G	A	G	GTF3C5	c.G113A	p.R38H
XH_P36	9	137801701	137801701	Silent	SNP	C	T	C	FCN1	c.G924A	p.A308A
XH_P36	9	138395457	138395457	Silent	SNP	G	A	G	MRPS2	c.G369A	p.T123T
XH_P36	9	139371084	139371084	Silent	SNP	C	T	C	SEC16A	c.G984A	p.R328R
XH_P36	9	139390736	139390736	Frame_Shift_Del	DEL	G	-	G	NOTCH1	c.7455delC	p.P2485fs
XH_P36	9	139996032	139996032	Missense_Mutation	SNP	C	T	C	MAN1B1	c.C1162T	p.R388W
XH_P36	9	140001244	140001244	Missense_Mutation	SNP	C	A	C	MAN1B1	c.C1549A	p.R517S
XH_P36	9	140081973	140081973	Missense_Mutation	SNP	G	A	G	ANAPC2	c.C700T	p.R234C
XH_P36	9	140140197	140140197	Silent	SNP	C	T	C	FAM166A	c.G165A	p.V55V
XH_P36	10	94773	94773	Missense_Mutation	SNP	C	T	C	TUBB8	c.G137A	p.R46H
XH_P36	10	8019267	8019267	Missense_Mutation	SNP	G	A	G	TAF3	c.G2296A	p.G766S
XH_P36	10	8100708	8100708	Missense_Mutation	SNP	G	A	G	GATA3	c.G682A	p.E228K
XH_P36	10	13481346	13481346	Nonsense_Mutation	INS	-	TCTTGCTTATTTAT	-	BEND7	CACACATAAATA	11_R412delinsTHTX
XH_P36	10	14816335	14816335	Missense_Mutation	SNP	C	T	C	FAM107B	c.G328A	p.G110R
XH_P36	10	19981225	19981225	Missense_Mutation	SNP	G	T	G	MALRD1	c.G6152T	p.W2051L
XH_P36	10	20432247	20432247	Missense_Mutation	SNP	G	A	G	PLXDC2	c.G418A	p.V140I
XH_P36	10	25284588	25284588	Missense_Mutation	SNP	T	C	T	ENKUR	c.A248G	p.Y83C
XH_P36	10	35484127	35484127	Frame_Shift_Ins	INS	-	TAAACCAT	-	CREM	10_11insTAAACC	p.T4fs
XH_P36	10	44788267	44788267	Frame_Shift_Del	DEL	C	-	C	C10orf142	c.59delC	p.T20fs
XH_P36	10	46999854	46999854	Missense_Mutation	SNP	C	T	C	GPRIN2	c.C974T	p.A325V
XH_P36	10	50732583	50732583	Frame_Shift_Ins	INS	-	GCAGAGGGAGA	-	ERCC6	_893insTCTCCCT	p.A298fs
XH_P36	10	50953853	50953853	Silent	SNP	G	A	G	OGDHL	c.C840T	p.V280V
XH_P36	10	52005124	52005124	Missense_Mutation	SNP	G	A	G	ASAH2	c.C218T	p.S73F
XH_P36	10	52005126	52005126	In_Frame_Ins	INS	-	CAAGTAATT	-	ASAH2	5_216insAATTA	p.H72delinsQITC
XH_P36	10	64159513	64159513	Frame_Shift_Del	DEL	A	-	A	ZNF365	c.1189delA	p.K397fs
XH_P36	10	70332889	70332889	Missense_Mutation	SNP	A	G	A	TET1	c.A794G	p.N265S
XH_P36	10	70723214	70723214	Missense_Mutation	SNP	C	T	C	DDX21	c.C571T	p.R191C
XH_P36	10	71142432	71142432	Silent	SNP	G	A	G	HK1	c.G1359A	p.L453L
XH_P36	10	73466731	73466731	Missense_Mutation	SNP	G	A	G	CDH23	c.G3031A	p.E1011K
XH_P36	10	74666426	74666426	Nonsense_Mutation	INS	-	TCCAGTATCATCA	-	OIT3	18insATCCAGTAT	p.C206delinsX
XH_P36	10	75435427	75435427	In_Frame_Ins	INS	-	AAAAAGAAACAC	-	AGAP5	_991insGTGTTTG	.I331delinsVFLFI
XH_P36	10	78729786	78729786	Frame_Shift_Del	DEL	T	-	T	KCNMA1	c.2132delA	p.K711fs
XH_P36	10	81317157	81317157	Silent	SNP	G	T	G	SFTPA2	c.C585A	p.A195A
XH_P36	10	91497459	91497459	Frame_Shift_Ins	INS	-	TGCTCTCAGTCCT	-	KIF20B	2862insTGCTCTC	p.N954fs
XH_P36	10	92617011	92617011	Missense_Mutation	SNP	C	T	C	HTR7	c.G418A	p.V140I
XH_P36	10	93601946	93601946	Frame_Shift_Del	DEL	A	-	A	TNKS2	c.1857delA	p.T619fs
XH_P36	10	93999314	93999314	Missense_Mutation	SNP	C	T	C	CPEB3	c.G794A	p.G265D
XH_P36	10	95093644	95093644	Nonsense_Mutation	INS	-	CTCATTTTACAG	-	MYOF	51insACCTGTAA	17_D1518delinsPPVKX
XH_P36	10	98080475	98080475	Missense_Mutation	SNP	T	C	T	DNTT	c.T608C	p.F203S
XH_P36	10	98741863	98741863	In_Frame_Ins	INS	-	AGCTGTCTTGATC	-	LCOR	47insGAGCTGTC	P549delinsPSCLDG
XH_P36	10	102242535	102242535	Frame_Shift_Ins	INS	-	G	-	WNT8B	c.1019dupG	p.R340fs
XH_P36	10	102749036	102749036	Missense_Mutation	SNP	C	T	C	TWNK	c.C1069T	p.R357C
XH_P36	10	103530166	103530166	Missense_Mutation	SNP	G	A	G	FGF8	c.C343T	p.R115C
XH_P36	10	103917038	103917038	Missense_Mutation	SNP	G	A	G	NOLC1	c.G269A	p.S90N
XH_P36	10	103990510	103990510	Frame_Shift_Ins	INS	-	G	-	PITX3	c.669dupC	p.G224fs
XH_P36	10	104404668	104404668	Frame_Shift_Del	DEL	C	-	C	TRIM8	c.294delC	p.G98fs
XH_P36	10	104632345	104632345	Frame_Shift_Ins	INS	-	TAAATCTTATACA	-	AS3MT	ACCACTAAATCTT	p.T104fs
XH_P36	10	105233174	105233174	In_Frame_Ins	INS	-	GTCTCAGGCACT	-	CALHM3	nsCGCAGTGCCT	277delinsPAVPETP
XH_P36	10	114925317	114925317	Frame_Shift_Del	DEL	A	-	A	TCF7L2	c.1151delA	p.E384fs
XH_P36	10	115601182	115601182	Missense_Mutation	SNP	T	C	T	DCLRE1A	c.A2803G	p.M935V
XH_P36	10	118427804	118427804	Missense_Mutation	SNP	A	G	A	C10orf82	c.T20C	p.F7S
XH_P36	10	120789359	120789359	Frame_Shift_Ins	INS	-	C	-	NANOS1	c.47dupC	p.A16fs
XH_P36	10	121196221	121196221	Missense_Mutation	SNP	T	C	T	GRK5	c.T797C	p.M266T
XH_P36	10	127541779	127541779	Silent	SNP	A	G	A	BCCIP	c.A936G	p.G312G
XH_P36	10	128150079	128150079	Frame_Shift_Ins	INS	-	TCCCCTAA	-	C10orf90	9_1610insTTAG	p.P537fs
XH_P36	10	128150081	128150081	Frame_Shift_Ins	INS	-	TT	-	C10orf90	.1607_1608insA	p.E536fs
XH_P36	10	128973731	128973731	Missense_Mutation	SNP	G	A	G	FAM196A	c.C929T	p.P310L
XH_P36	10	129903319	129903319	Missense_Mutation	SNP	A	G	A	MKI67	c.T5705C	p.V1902A
XH_P36	10	133955443	133955443	Missense_Mutation	SNP	C	T	C	JAKMIP3	c.C1277T	p.T426M
XH_P36	10	134563094	134563094	Missense_Mutation	SNP	G	A	G	INPP5A	c.G614A	p.R205H
XH_P36	10	134942117	134942117	Missense_Mutation	SNP	C	T	C	ADGRA1	c.C494T	p.T165M
XH_P36	10	135012703	135012703	Silent	SNP	G	A	G	KNDC1	c.G2691A	p.T897T
XH_P36	10	135176380	135176380	Missense_Mutation	SNP	C	A	C	ECHS1	c.G865T	p.D289Y
XH_P36	11	308213	308213	Silent	SNP	C	A	C	IFITM2	c.C21A	p.T7T
XH_P36	11	618713	618713	Missense_Mutation	SNP	C	T	C	CDHR5	c.G1828A	p.G610S
XH_P36	11	1016425	1016425	Frame_Shift_Ins	INS	-	CCTCTACTACC	-	MUC6	6376insGGTAGT	p.Q2126fs
XH_P36	11	1016513	1016513	Frame_Shift_Ins	INS	-	ATCGCCTTCTG	-	MUC6	6288insCAGAAC	p.Q2096fs
XH_P36	11	2432968	2432968	Missense_Mutation	SNP	C	T	C	TRPM5	c.G2504A	p.R835H
XH_P36	11	3114178	3114178	Missense_Mutation	SNP	C	T	C	OSBPL5	c.G1847A	p.R616Q
XH_P36	11	3848989	3848989	Missense_Mutation	SNP	C	T	C	RHOG	c.G380A	p.R127Q
XH_P36	11	4661623	4661623	Silent	SNP	C	A	C	OR51D1	c.C603A	p.S201S
XH_P36	11	4881339	4881339	Silent	SNP	G	A	G	OR51H1	c.C456T	p.T152T
XH_P36	11	5275557	5275557	Missense_Mutation	SNP	A	G	A	HBG2	c.T280C	p.C94R
XH_P36	11	5364256	5364256	Missense_Mutation	SNP	A	G	A	OR51B5	c.T499C	p.C167R
XH_P36	11	5686526	5686526	Missense_Mutation	SNP	C	T	C	TRIM5	c.G995A	p.R332Q
XH_P36	11	5878544	5878544	Frame_Shift_Ins	INS	-	TATCTCTCTTGTT	-	OR52E8	insGTAACAAGAC	p.I130fs
XH_P36	11	6472654	6472654	In_Frame_Ins	INS	-	AAGATTCTGCTT	-	TRIM3	1191insAAGCAG	.E397delinsESRIL
XH_P36	11	7817937	7817937	Frame_Shift_Ins	INS	-	GTGGTGTCTGTC	-	OR5P2	3insAACACAGAC	p.C185fs
XH_P36	11	7960556	7960556	Frame_Shift_Ins	INS	-	CTTACAC	-	OR10A3	11_512insGTGTA	p.P171fs
XH_P36	11	9861142	9861142	Missense_Mutation	SNP	G	A	G	SBF2	c.C3358T	p.R1120C
XH_P36	11	12316345	12316353	In_Frame_Del	DEL	CTCCTCCTC	-	CTCCTCCTC	MICALCL	c.1367_1375del	p.456_459del
XH_P36	11	14498554	14498554	Nonsense_Mutation	SNP	G	A	G	COPB1	c.C1366T	p.R456X
XH_P36	11	17758073	17758073	Missense_Mutation	SNP	G	A	G	KCNC1	c.G524A	p.R175H
XH_P36	11	18428876	18428876	Frame_Shift_Ins	INS	-	GACTTACAGTCTC	-	LDHA	CCAAGACTTACA	p.L260fs
XH_P36	11	20066788	20066788	Silent	SNP	C	T	C	NAV2	c.C732T	p.L244L
XH_P36	11	20181600	20181600	Missense_Mutation	SNP	G	A	G	DBX1	c.C271T	p.R91W
XH_P36	11	20676326	20676326	Missense_Mutation	SNP	G	A	G	SLC6A5	c.G1604A	p.R535H
XH_P36	11	27520343	27520343	Frame_Shift_Ins	INS	-	TG	-	LIN7C	c.446_447insCA	p.E149fs
XH_P36	11	27520345	27520345	Frame_Shift_Ins	INS	-	TTATAAATGCCTA	-	LIN7C	sAAAATAGGCAT	p.E149fs
XH_P36	11	30255300	30255300	Nonsense_Mutation	SNP	C	T	C	FSHB	c.C343T	p.R115X
XH_P36	11	32608690	32608690	Frame_Shift_Ins	INS	-	ACAATCTCAAATT	-	EIF3M	6insCACAATCTC	p.D59fs
XH_P36	11	32635937	32635937	Frame_Shift_Ins	INS	-	AAGA	-	CCDC73	1926_1927insTC	p.Q643fs

XH_P36	11	32635939	32635939	In_Frame_Ins	INS	-	ATAAAAATGTCC	-	CCDC73	nsACTATGGACA	642delinsYYGHFYLS
XH_P36	11	33308862	33308862	Frame_Shift_Ins	INS	-	AAGAAAAT	-	HIPK3	2_903insAAGAA	p.P301fs
XH_P36	11	33308864	33308864	Frame_Shift_Ins	INS	-	AGGAC	-	HIPK3	904_905insAGG	p.I302fs
XH_P36	11	33596351	33596351	Missense_Mutation	SNP	T	G	T	KIAA1549L	c.T3443G	p.I1148S
XH_P36	11	34489930	34489930	Silent	SNP	C	T	C	CAT	c.C1422T	p.I474I
XH_P36	11	43425610	43425613	Frame_Shift_Del	DEL	GAGT	-	GAGT	TTC17	c.1395_1398del	p.Q465fs
XH_P36	11	45671512	45671512	Missense_Mutation	SNP	C	G	C	CHST1	c.G962C	p.G321A
XH_P36	11	47261725	47261725	Missense_Mutation	SNP	C	T	C	ACP2	c.G1025A	p.R342Q
XH_P36	11	47611863	47611863	Missense_Mutation	SNP	C	T	C	C1QTNF4	c.G500A	p.R167H
XH_P36	11	48347449	48347449	Frame_Shift_Del	DEL	A	-	A	OR4C3	c.957delA	p.V319fs
XH_P36	11	55798310	55798310	Missense_Mutation	SNP	G	A	G	OR5AS1	c.G416A	p.R139K
XH_P36	11	55860958	55860958	Frame_Shift_Ins	INS	-	TAATCAAATT	-	OR8I2	_176insTAATCA	p.M59fs
XH_P36	11	55860960	55860960	In_Frame_Ins	INS	-	ACT	-	OR8I2	c.177_178insAC	p.M59delinsMT
XH_P36	11	56237708	56237708	Frame_Shift_Ins	INS	-	T	-	OR5M3	c.265dupA	p.T89fs
XH_P36	11	57310811	57310811	Frame_Shift_Ins	INS	-	GTCTCTGTCATT	-	SMTNL1	97insTGTCTCTG	p.A232fs
XH_P36	11	60506456	60506456	Missense_Mutation	SNP	C	A	C	MS4A18	c.C610A	p.L204I
XH_P36	11	61018732	61018732	Silent	SNP	C	T	C	PGA5	c.C1146T	p.V382V
XH_P36	11	62289913	62289913	Silent	SNP	T	A	T	AHNAK	c.A11976T	p.P3992P
XH_P36	11	62295576	62295576	Missense_Mutation	SNP	G	A	G	AHNAK	c.C6313T	p.L2105F
XH_P36	11	63277289	63277289	Missense_Mutation	SNP	C	T	C	LGALS12	c.C295T	p.R99W
XH_P36	11	64037690	64037690	Frame_Shift_Del	DEL	G	-	G	BAD	c.498delC	p.P166fs
XH_P36	11	64572093	64572093	Frame_Shift_Del	DEL	G	-	G	MEN1	c.1561delC	p.R521fs
XH_P36	11	64596977	64596977	Silent	SNP	G	A	G	CDC42BPG	c.C3933T	p.H1311H
XH_P36	11	65307555	65307555	Frame_Shift_Del	DEL	C	-	C	LTBP3	c.3314delG	p.R1105fs
XH_P36	11	65396411	65396411	Silent	SNP	C	T	C	PCNX3	c.C3933T	p.R1311R
XH_P36	11	65733371	65733371	Missense_Mutation	SNP	C	T	C	SART1	c.C752T	p.A251V
XH_P36	11	65780794	65780794	Missense_Mutation	SNP	C	T	C	CST6	c.C373T	p.R125C
XH_P36	11	66083251	66083251	Silent	SNP	C	T	C	CD248	c.G1248A	p.E416E
XH_P36	11	66999319	66999319	Frame_Shift_Ins	INS	-	CTCCAAATCTAC	-	KDM2A	68insAACTCCAA	p.F456fs
XH_P36	11	67072394	67072394	Silent	SNP	C	A	C	SSH3	c.C255A	p.S85S
XH_P36	11	67187007	67187007	Missense_Mutation	SNP	G	A	G	CARNS1	c.G533A	p.R178H
XH_P36	11	67258296	67258296	Missense_Mutation	SNP	C	T	C	AIP	c.C817T	p.R273C
XH_P36	11	67261809	67261809	Missense_Mutation	SNP	G	A	G	PITPNM1	c.C2762T	p.A921V
XH_P36	11	67264838	67264838	Silent	SNP	G	A	G	PITPNM1	c.C2010T	p.P670P
XH_P36	11	67800693	67800693	Silent	SNP	A	G	A	NDUFS8	c.A315G	p.P105P
XH_P36	11	68080214	68080214	In_Frame_Ins	INS	-	GCT	-	LRP5	c.32_33insGCT	p.P11delinsPL
XH_P36	11	68334537	68334537	Missense_Mutation	SNP	G	A	G	PPP6R3	c.G1031A	p.R344Q
XH_P36	11	68517872	68517872	Frame_Shift_Del	DEL	C	-	C	TESMIN	c.257delG	p.G86fs
XH_P36	11	70319341	70319341	Frame_Shift_Ins	INS	-	TCACTTA	-	SHANK2	18_3419insTAA	p.A1140fs
XH_P36	11	71195477	71195477	Missense_Mutation	SNP	C	T	C	NADSYN1	c.C1439T	p.A480V
XH_P36	11	71712654	71712654	Frame_Shift_Del	DEL	G	-	G	IL18BP	c.563delG	p.W188fs
XH_P36	11	71727133	71727133	Missense_Mutation	SNP	C	A	C	NUMA1	c.G1416T	p.Q472H
XH_P36	11	75623053	75623053	Missense_Mutation	SNP	G	A	G	UVRAG	c.G563A	p.R188H
XH_P36	11	82966931	82966931	Frame_Shift_Del	DEL	A	-	A	ANKRD42	c.1509delA	p.V503fs
XH_P36	11	83676491	83676491	In_Frame_Ins	INS	-	ATACTTCAGAGG	-	DLG2	sTACTCCCTCTG	203delinsDTPSEVFP
XH_P36	11	85420388	85420388	Frame_Shift_Del	DEL	T	-	T	SYTL2	c.279delA	p.K93fs
XH_P36	11	88583231	88583231	Missense_Mutation	SNP	C	T	C	GRM5	c.G754A	p.A252T
XH_P36	11	89223652	89223652	Missense_Mutation	SNP	A	G	A	NOX4	c.T127C	p.Y43H
XH_P36	11	89447527	89447527	Frame_Shift_Ins	INS	-	TCAGCTATTCTG	-	TRIM77	CCTTATCAGCTA	p.L225fs
XH_P36	11	89914834	89914834	Nonsense_Mutation	INS	-	TAGAAAATATAA	-	NAALAD2	AATTAGTAGAAA	602_F603delinsDPX
XH_P36	11	92532469	92532469	Missense_Mutation	SNP	C	T	C	FAT3	c.C6290T	p.A2097V
XH_P36	11	94599175	94599175	Missense_Mutation	SNP	G	A	G	AMOTL1	c.G2180A	p.R727H
XH_P36	11	95546700	95546700	Nonsense_Mutation	SNP	C	T	C	CEP57	c.C451T	p.R151X
XH_P36	11	101323754	101323754	Missense_Mutation	SNP	C	A	C	TRPC6	c.G2728T	p.A910S
XH_P36	11	101833918	101833918	In_Frame_Ins	INS	-	TGACTGTTTCA	-	CEP126	nsTGAATGACTG	18delinsVNDCFQHC
XH_P36	11	102587116	102587116	Frame_Shift_Ins	INS	-	ATCGGAATTTTT	-	MMP8	nsTATAAAAAATT	p.P273fs
XH_P36	11	102661218	102661218	In_Frame_Ins	INS	-	TTTAGAATGTAG	-	MMP1	AAAGGGCTACAT	delinsYQYKKGYILNSKKIC
XH_P36	11	108384793	108384793	Frame_Shift_Del	DEL	A	-	A	EXPH5	c.877delT	p.W293fs
XH_P36	11	113694379	113694379	Frame_Shift_Ins	INS	-	CT	-	USP28	c.231_232insAG	p.C78fs
XH_P36	11	115109355	115109355	Missense_Mutation	SNP	A	C	A	CADM1	c.T289G	p.F97V
XH_P36	11	118526386	118526386	Missense_Mutation	SNP	C	T	C	PHLDB1	c.C3805T	p.R1269C
XH_P36	11	119216273	119216273	Frame_Shift_Del	DEL	G	-	G	MFRP	c.498delC	p.P166fs
XH_P36	11	121000749	121000749	Missense_Mutation	SNP	C	A	C	TECTA	c.C2770A	p.L924M
XH_P36	11	121475863	121475863	Missense_Mutation	SNP	G	T	G	SORL1	c.G4693T	p.A1565S
XH_P36	11	123448124	123448124	Missense_Mutation	SNP	C	T	C	GRAMD1B	c.C73T	p.R25W
XH_P36	11	123814451	123814451	Frame_Shift_Ins	INS	-	CTGACTTC	-	OR6T1	4_95insGAAGTC	p.M32fs
XH_P36	11	124266349	124266349	Missense_Mutation	SNP	A	G	A	OR8B3	c.T899C	p.L300P
XH_P36	11	124637789	124637789	Frame_Shift_Ins	INS	-	GGAA	-	MSANTD2	.272_273insTTC	p.N91fs
XH_P36	11	124637790	124637790	In_Frame_Ins	INS	-	GTTGTTCAA	-	MSANTD2	1_272insTTGAA	p.N91delinsIEQH
XH_P36	11	124845049	124845049	Frame_Shift_Del	DEL	A	-	A	CCDC15	c.574delA	p.K192fs
XH_P36	11	125221249	125221249	Silent	SNP	G	A	G	PKNOX2	c.G48A	p.T16T
XH_P36	12	2907038	2907041	Splice_Site	DEL	GTGA	-	GTGA	FKBP4	.	.
XH_P36	12	4735969	4735969	Missense_Mutation	SNP	G	A	G	AKAP3	c.C2099T	p.S700F
XH_P36	12	4766968	4766968	Missense_Mutation	SNP	G	A	G	NDUFA9	c.G373A	p.V125M
XH_P36	12	6131192	6131192	Missense_Mutation	SNP	A	G	A	VWF	c.T3548C	p.L1183P
XH_P36	12	9221371	9221371	Missense_Mutation	SNP	G	A	G	A2M	c.C3881T	p.P1294L
XH_P36	12	10762506	10762506	In_Frame_Ins	INS	-	TTAATTTTA	-	MAGOHB	9_50insTAAAAT	p.K17delinsIKLK
XH_P36	12	10762508	10762508	Frame_Shift_Ins	INS	-	TCTT	-	MAGOHB	c.47_48insAAGA	p.L16fs
XH_P36	12	11286385	11286385	Nonsense_Mutation	INS	-	TCATATCAGTTT	-	TAS2R30	insTACAAACTG	53_W154delinsVTNX
XH_P36	12	12814973	12814973	Missense_Mutation	SNP	G	A	G	GPR19	c.C410T	p.T137M
XH_P36	12	13238177	13238177	Frame_Shift_Ins	INS	-	CA	-	GSG1	c.485_486insTG	p.Y162fs
XH_P36	12	13238179	13238179	Splice_Site	INS	-	CTGTCTCTG	-	GSG1	.	.
XH_P36	12	18876416	18876416	Missense_Mutation	SNP	T	A	T	PLCZ1	c.A196T	p.I66F
XH_P36	12	26752988	26752988	In_Frame_Ins	INS	-	GTG	-	ITPR2	3732_3733insCA	p.F1245delinsHF
XH_P36	12	26752989	26752989	Frame_Shift_Ins	INS	-	ATATCTTTTTTTT	-	ITPR2	3732insAAAAAA	p.N1244fs
XH_P36	12	30805220	30805220	Nonsense_Mutation	INS	-	TATTTTTCATTT	-	IPO8	AATACAAATGA	88_M489delinsKYKX
XH_P36	12	30863059	30863059	Missense_Mutation	SNP	A	G	A	CAPRIN2	c.T2159C	p.L720P
XH_P36	12	40672017	40672017	Missense_Mutation	SNP	G	A	G	LRRK2	c.G2195A	p.G732E
XH_P36	12	42523647	42523648	Frame_Shift_Del	DEL	TT	-	TT	GXYLT1	c.227_228del	p.K76fs
XH_P36	12	43862460	43862460	Nonsense_Mutation	INS	-	TATTAGTGTAAT	-	ADAMTS20	insGAAAATTACA	9_N390delinsRKLHX
XH_P36	12	44149036	44149036	Frame_Shift_Ins	INS	-	TTAGAAGATGGA	-	PUS7L	insCTTCCATCTT	p.T5fs
XH_P36	12	48141379	48141379	Nonsense_Mutation	SNP	G	A	G	RAPGEF3	c.C1378T	p.R460X
XH_P36	12	49521893	49521893	Missense_Mutation	SNP	G	A	G	TUBA1B	c.C1204T	p.R402C
XH_P36	12	50747903	50747903	Frame_Shift_Ins	INS	-	AGCAACCCTTGT	-	FAM186A	insAAACACAAG	p.E904fs
XH_P36	12	50747905	50747905	Nonsense_Mutation	INS	-	ATTT	-	FAM186A	2709_2710insAA	904_E905delinsKX
XH_P36	12	51399180	51399180	Missense_Mutation	SNP	C	T	C	SLC11A2	c.G211A	p.G71R
XH_P36	12	52684997	52684997	Missense_Mutation	SNP	C	T	C	KRT81	c.G253A	p.E85K
XH_P36	12	53038849	53038849	Missense_Mutation	SNP	C	T	C	KRT2	c.G1874A	p.S625N

XH_P36	12	53162773	53162775	In_Frame_Del	DEL	ACT	-	ACT	KRT76	c.1639_1641del	p.547_547del
XH_P36	12	56398615	56398615	Missense_Mutation	SNP	G	A	G	SUOX	c.G1442A	p.R481H
XH_P36	12	56494905	56494905	Missense_Mutation	SNP	C	T	C	ERBB3	c.C3262T	p.P1088S
XH_P36	12	56558476	56558476	Missense_Mutation	SNP	A	T	A	SMARCC2	c.T3179A	p.M1060K
XH_P36	12	56558477	56558477	In_Frame_Ins	INS	-	CCTTGCCTC	-	SMARCC2	_3178insGAGG	M1060delinsEARM
XH_P36	12	56559113	56559113	Frame_Shift_Del	DEL	G	-	G	SMARCC2	c.3128delC	p.P1043fs
XH_P36	12	56572601	56572601	Missense_Mutation	SNP	G	A	G	SMARCC2	c.C1172T	p.T391M
XH_P36	12	56661723	56661723	Missense_Mutation	SNP	G	T	G	COQ10A	c.G184T	p.G62W
XH_P36	12	56994169	56994169	Nonsense_Mutation	SNP	C	A	C	BAZ2A	c.G4708T	p.G1570X
XH_P36	12	57389231	57389231	Missense_Mutation	SNP	C	T	C	GPR182	c.C238T	p.R80C
XH_P36	12	57906594	57906594	Frame_Shift_Ins	INS	-	GTCCCTTC	-	MARS	4_1815insGTCC	p.D605fs
XH_P36	12	57911097	57911097	Silent	SNP	T	C	T	DDIT3	c.A162G	p.S54S
XH_P36	12	58025103	58025103	Frame_Shift_Del	DEL	C	-	C	B4GALNT1	c.263delG	p.G88fs
XH_P36	12	63087738	63087738	Frame_Shift_Ins	INS	-	G	-	PPM1H	c.1114dupC	p.L372fs
XH_P36	12	64055225	64055225	Missense_Mutation	SNP	C	A	C	DPY19L2	c.G487T	p.A163S
XH_P36	12	65226072	65226072	Nonsense_Mutation	INS	-	ATTTTCAAAAATA	-	TBC1D30	CAATATTTTCAA	_D195delinsGKQYFQKX
XH_P36	12	65226073	65226073	Missense_Mutation	SNP	G	T	G	TBC1D30	c.G583T	p.D195Y
XH_P36	12	71834023	71834023	Missense_Mutation	SNP	C	A	C	LGR5	c.C163A	p.L55M
XH_P36	12	75687029	75687029	Missense_Mutation	SNP	T	A	T	CAPS2	c.A974T	p.K325I
XH_P36	12	75804272	75804272	Missense_Mutation	SNP	C	T	C	GLIPR1L2	c.C293T	p.T98M
XH_P36	12	77244718	77244718	Nonsense_Mutation	INS	-	ATACATGAATT	-	ZDHHC17	_1853insATACAT	618_I619delinsNTX
XH_P36	12	78400815	78400815	Frame_Shift_Ins	INS	-	GTTAAAACCTTC	-	NAV3	3insACTGTTAAA	p.T499fs
XH_P36	12	78515938	78515938	In_Frame_Ins	INS	-	AAG	-	NAV3	3968_3969insA	p.I1323delinsIR
XH_P36	12	78593137	78593137	Missense_Mutation	SNP	A	G	A	NAV3	c.A6475G	p.T2159A
XH_P36	12	88487681	88487681	Nonsense_Mutation	DEL	T	-	T	CEP290	c.3175delA	p.I1059X
XH_P36	12	88589253	88589253	Frame_Shift_Ins	INS	-	GATTGTGTTTTCC	-	TMTC3	573insGATTGTG	p.I858fs
XH_P36	12	89745700	89745700	Silent	SNP	G	A	G	DUSP6	c.C117T	p.C39C
XH_P36	12	89997947	89997947	Silent	SNP	G	A	G	ATP2B1	c.C2619T	p.G873G
XH_P36	12	91498041	91498041	Silent	SNP	G	T	G	LUM	c.C918A	p.I306I
XH_P36	12	91498042	91498042	Nonsense_Mutation	INS	-	AATTTAGTGTTT	-	LUM	ATAAAAAAACAC	306_K307delinsKX
XH_P36	12	93173078	93173078	Splice_Site	INS	-	CTTCCTTTTTCAC	-	EEA1	.	.
XH_P36	12	94543030	94543030	Frame_Shift_Del	DEL	C	-	C	PLXNC1	c.283delC	p.P95fs
XH_P36	12	94797019	94797019	Nonsense_Mutation	INS	-	GGTGCGTTTCTTT	-	CEP83	AAAATAAAGAAA	p.L115delinsX
XH_P36	12	97186181	97186181	Silent	SNP	G	A	G	CFAP54	c.G8754A	p.T2918T
XH_P36	12	101473003	101473003	Nonsense_Mutation	SNP	C	T	C	ANO4	c.C1345T	p.R449X
XH_P36	12	104697824	104697824	Missense_Mutation	SNP	C	T	C	EID3	c.C112T	p.R38C
XH_P36	12	107236579	107236579	Frame_Shift_Ins	INS	-	CACTT	-	RIC8B	001_1002insCA	p.E334fs
XH_P36	12	107236581	107236581	Frame_Shift_Ins	INS	-	CACTTACTATG	-	RIC8B	_1004insCACTTA	p.K335fs
XH_P36	12	108169390	108169390	Missense_Mutation	SNP	C	T	C	ASCL4	c.C398T	p.A133V
XH_P36	12	108954836	108954836	Missense_Mutation	SNP	G	T	G	SART3	c.C95A	p.A32D
XH_P36	12	109536183	109536183	Missense_Mutation	SNP	C	T	C	UNG	c.C52T	p.P18S
XH_P36	12	113445730	113445730	Missense_Mutation	SNP	G	A	G	OAS2	c.G1877A	p.S626N
XH_P36	12	117217097	117217097	Missense_Mutation	SNP	G	A	G	RNFT2	c.G826A	p.A276T
XH_P36	12	118681299	118681299	Nonsense_Mutation	INS	-	GTTATCAGTATT	-	TAOK3	insTTAAATACTC	72_V73delinsVKYX
XH_P36	12	118681301	118681301	Missense_Mutation	SNP	C	A	C	TAOK3	c.G213T	p.K71N
XH_P36	12	120760118	120760118	Nonsense_Mutation	INS	-	TATTTATTTTTTC	-	PLA2G1B	nsCTAGAAAAAA	9_N110delinsLEKINTX
XH_P36	12	123875311	123875311	Silent	SNP	C	T	C	KMT5A	c.C96T	p.A32A
XH_P36	12	124832387	124832387	Silent	SNP	G	A	G	NCOR2	c.C4026T	p.R1342R
XH_P36	12	124887093	124887093	In_Frame_Ins	INS	-	TGT	-	NCOR2	1493_1494insA	p.Q498delinsQQ
XH_P36	12	125396488	125396488	Silent	SNP	T	C	T	UBC	c.A1830G	p.Q610Q
XH_P36	12	125397037	125397037	Silent	SNP	C	T	C	UBC	c.G1281A	p.G427G
XH_P36	12	125397439	125397439	Silent	SNP	A	G	A	UBC	c.T879C	p.S293S
XH_P36	12	130647740	130647740	Missense_Mutation	SNP	T	G	T	FZD10	c.T253G	p.F85V
XH_P36	12	130830392	130830392	Missense_Mutation	SNP	G	T	G	PIWIL1	c.G285T	p.Q95H
XH_P36	12	130830394	130830394	In_Frame_Ins	INS	-	AACAAT	-	PIWIL1	287_288insAACA	p.N96delinsKTI
XH_P36	12	130935836	130935836	Silent	SNP	C	T	C	RIMBP2	c.G357A	p.R119R
XH_P36	12	131620642	131620642	Silent	SNP	C	T	C	ADGRD1	c.C2328T	p.G776G
XH_P36	12	133327262	133327262	Frame_Shift_Del	DEL	T	-	T	ANKLE2	c.814delA	p.T272fs
XH_P36	12	133463877	133463877	Missense_Mutation	SNP	G	T	G	CHFR	c.C38A	p.P13Q
XH_P36	12	133696963	133696963	Silent	SNP	T	C	T	ZNF891	c.A1542G	p.K514K
XH_P36	13	20244471	20244471	In_Frame_Ins	INS	-	CTGGAAAAGACT	-	MPHOSPH8	2426insCTGGAA	L809delinsPGKDL
XH_P36	13	20333558	20333558	Frame_Shift_Ins	INS	-	GTCTACATGGAA	-	PSPC1	5insCATTCCATG	p.T225fs
XH_P36	13	25670813	25670813	Silent	SNP	C	T	C	PABPC3	c.C477T	p.N159N
XH_P36	13	26542799	26542799	Missense_Mutation	SNP	G	A	G	ATP8A2	c.G3164A	p.R1055Q
XH_P36	13	26789433	26789433	Nonsense_Mutation	SNP	G	A	G	RNF6	c.C586T	p.R196X
XH_P36	13	27216440	27216440	Silent	SNP	G	A	G	WASF3	c.G33A	p.R11R
XH_P36	13	28712860	28712910	In_Frame_Del	DEL	CCCGCCGGG	-	GCCCCGCCGGGG	PAN3	c.66_116del	p.22_39del
XH_P36	13	30801573	30801573	Frame_Shift_Del	DEL	T	-	T	KATNAL1	c.1123delA	p.R375fs
XH_P36	13	31714404	31714404	Nonsense_Mutation	INS	-	GAGAACCTATCA	-	HSPH1	AATATTTGATAG	_A558delinsKYLIGSLFX
XH_P36	13	32910998	32910998	Frame_Shift_Ins	INS	-	AATGTTGTACTG	-	BRCA2	sTTTTGAATGTT	p.P836fs
XH_P36	13	33344888	33344888	Frame_Shift_Del	DEL	A	-	A	PDS5B	c.4161delA	p.P1387fs
XH_P36	13	36905543	36905543	Missense_Mutation	SNP	C	T	C	SPG20	c.G1001A	p.R334Q
XH_P36	13	39265390	39265390	Silent	SNP	G	A	G	FREM2	c.G3909A	p.T1303T
XH_P36	13	40175192	40175192	Silent	SNP	C	T	C	LHFP	c.G162A	p.V54V
XH_P36	13	45008838	45008838	Frame_Shift_Del	DEL	G	-	G	TSC22D1	c.185delC	p.P62fs
XH_P36	13	46632361	46632361	Missense_Mutation	SNP	A	G	A	CPB2	c.T841C	p.F281L
XH_P36	13	52604544	52604544	Frame_Shift_Ins	INS	-	C	-	UTP14C	c.1604_1605ins	p.Q535fs
XH_P36	13	52604545	52604545	Missense_Mutation	SNP	G	C	G	UTP14C	c.G1605C	p.Q535H
XH_P36	13	52639565	52639565	Missense_Mutation	SNP	C	T	C	NEK5	c.G2105A	p.S702N
XH_P36	13	53421509	53421509	Missense_Mutation	SNP	C	T	C	PCDH8	c.G1063A	p.A355T
XH_P36	13	77529556	77529556	Missense_Mutation	SNP	C	T	C	ACOD1	c.C355T	p.P119S
XH_P36	13	77641983	77641983	Nonsense_Mutation	INS	-	GATTTTCAATTC	-	MYCBP2	3insCCTGGAATT	63_R4064delinsTWNX
XH_P36	13	84454271	84454271	Missense_Mutation	SNP	C	T	C	SLITRK1	c.G1372A	p.A458T
XH_P36	13	92560239	92560239	Frame_Shift_Ins	INS	-	TA	-	GPC5	.1329_1330insT	p.N443fs
XH_P36	13	92560240	92560240	Nonsense_Mutation	INS	-	GTTTTTTGATAC	-	GPC5	31insTTGTTTTT	44_E445delinsLVFX
XH_P36	13	95121040	95121040	Frame_Shift_Del	DEL	A	-	A	DCT	c.555delT	p.F185fs
XH_P36	13	98828558	98828558	Silent	SNP	C	T	C	RNF113B	c.G933A	p.L311L
XH_P36	13	99091450	99091450	Silent	SNP	G	A	G	FARP1	c.G2433A	p.T811T
XH_P36	13	103390256	103390256	Missense_Mutation	SNP	T	C	T	CCDC168	c.A12791G	p.E4264G
XH_P36	13	103515102	103515102	Frame_Shift_Ins	INS	-	GAGCTCTCTT	-	BIVM-ERCC5	_1604insGAGCT	p.K535fs
XH_P36	13	110434690	110434690	Silent	SNP	C	T	C	IRS2	c.G3711A	p.S1237S
XH_P36	13	111110449	111110449	Missense_Mutation	SNP	C	T	C	COL4A2-AS2	c.G257A	p.R86Q
XH_P36	13	111371669	111371669	Missense_Mutation	SNP	C	T	C	ING1	c.C179T	p.A60V
XH_P36	13	113439523	113439523	Missense_Mutation	SNP	G	T	G	ATP11A	c.G114T	p.E38D
XH_P36	14	19553760	19553760	Missense_Mutation	SNP	G	A	G	POTEG	c.G344A	p.S115N
XH_P36	14	21550439	21550439	Silent	SNP	T	C	T	ARHGEF40	c.T1146C	p.C382C
XH_P36	14	21555623	21555623	Splice_Site	SNP	G	A	G	ARHGEF40	.	.
XH_P36	14	21569511	21569511	Silent	SNP	T	C	T	TMEM253	c.T9C	p.D3D

XH_P36	14	23745293	23745293	In_Frame_Ins	INS	-	GATATACAACCC	-	HOMEZ	1144insGGGTTC	R382delinsGLYIR
XH_P36	14	31642477	31642477	Silent	SNP	G	A	G	HECTD1	c.C1041T	p.G347G
XH_P36	14	37736246	37736246	Nonsense_Mutation	INS	-	AGCTTTAAACAT	-	MIPOL1	CTCTTTAGCTTTA	S42delinsKHSLALNIX
XH_P36	14	39649809	39649809	Missense_Mutation	SNP	G	A	G	PNN	c.G896A	p.R299H
XH_P36	14	39901278	39901278	Missense_Mutation	SNP	A	C	A	FBXO33	c.T89G	p.L30R
XH_P36	14	50626388	50626388	Missense_Mutation	SNP	G	A	G	SOS2	c.C1613T	p.A538V
XH_P36	14	51210171	51210171	Missense_Mutation	SNP	G	A	G	NIN	c.C3125T	p.A1042V
XH_P36	14	57269043	57269043	Missense_Mutation	SNP	G	A	G	OTX2	c.C304T	p.R102C
XH_P36	14	59107555	59107555	In_Frame_Ins	INS	-	TTCACTAAA	-	DACT1	7_448insTTCACT	E149delinsEFTK
XH_P36	14	59992099	59992099	Frame_Shift_Ins	INS	-	T	-	CCDC175	c.1895dupA	p.N632fs
XH_P36	14	60712698	60712698	Frame_Shift_Ins	INS	-	TCCTGTCCTGTG	-	PPM1A	34insATCCTGTC	p.R45fs
XH_P36	14	61186779	61186779	Missense_Mutation	SNP	C	A	C	SIX4	c.G1248T	p.Q416H
XH_P36	14	68044071	68044071	Silent	SNP	C	T	C	PLEKHH1	c.C2514T	p.Y838Y
XH_P36	14	68045918	68045918	Missense_Mutation	SNP	C	T	C	PLEKHH1	c.C2917T	p.R973W
XH_P36	14	71199421	71199421	Frame_Shift_Ins	INS	-	TCTTTCCACTTC	-	MAP3K9	4insCCGAAGTG	p.K622fs
XH_P36	14	71206835	71206835	Frame_Shift_Ins	INS	-	GTCTCTCTCCA	-	MAP3K9	696insTGGAGA	p.K232fs
XH_P36	14	72056027	72056027	Missense_Mutation	SNP	T	C	T	SIPA1L1	c.T1438C	p.Y480H
XH_P36	14	74138302	74138302	Splice_Site	INS	-	ATAATTTCATAA	-	DNAL1	.	.
XH_P36	14	74203800	74203800	Silent	SNP	G	A	G	ELMSAN1	c.C1650T	p.D550D
XH_P36	14	75359617	75359617	Frame_Shift_Del	DEL	C	-	C	DLST	c.523delC	p.P175fs
XH_P36	14	75513561	75513561	In_Frame_Ins	INS	-	AGGTGA	-	MLH3	797_2798insTCA	G933delinsVTC
XH_P36	14	75513562	75513562	In_Frame_Ins	INS	-	CAAAATGGA	-	MLH3	6_2797insTCCAT	G933delinsSILG
XH_P36	14	75515373	75515376	Frame_Shift_Del	DEL	TCAA	-	TCAA	MLH3	c.983_986del	p.I328fs
XH_P36	14	76429801	76429801	Nonsense_Mutation	SNP	C	A	C	TGFB3	c.G784T	p.G262X
XH_P36	14	86088585	86088585	Missense_Mutation	SNP	T	C	T	FLRT2	c.T727C	p.S243P
XH_P36	14	91444862	91444862	Frame_Shift_Ins	INS	-	TTTGGGT	-	RPS6KA5	31_182insACCC	p.G61fs
XH_P36	14	91444863	91444863	Frame_Shift_Ins	INS	-	TTAAGTT	-	RPS6KA5	30_181insAACT	p.G61fs
XH_P36	14	92471769	92471769	In_Frame_Ins	INS	-	GCAAAAGAAGG	-	TRIP11	sTCCATTTCCTTC	350delinsSISFFCSCK
XH_P36	14	93404844	93404844	Missense_Mutation	SNP	C	T	C	ITPK1	c.G929A	p.G310D
XH_P36	14	93761193	93761193	Frame_Shift_Del	DEL	T	-	T	BTBD7	c.173delA	p.K58fs
XH_P36	14	94107567	94107567	Silent	SNP	C	T	C	UNC79	c.C5463T	p.D1821D
XH_P36	14	94395508	94395508	Nonstop_Mutation	SNP	T	C	T	FAM181A	c.T877C	p.X293Q
XH_P36	14	94417346	94417346	Silent	SNP	G	A	G	ASB2	c.C735T	p.Y245Y
XH_P36	14	95675732	95675732	Silent	SNP	G	A	G	CLMN	c.C885T	p.A295A
XH_P36	14	97022519	97022519	Silent	SNP	G	A	G	PAPOLA	c.G1023A	p.S341S
XH_P36	14	99642353	99642353	Missense_Mutation	SNP	C	T	C	BCL11B	c.G604A	p.V202M
XH_P36	14	100126651	100126651	Silent	SNP	G	T	G	HHIPL1	c.G1410T	p.T470T
XH_P36	14	103336573	103336573	Missense_Mutation	SNP	C	T	C	TRAF3	c.C35T	p.A12V
XH_P36	14	103996592	103996592	Missense_Mutation	SNP	G	A	G	TRMT61A	c.G277A	p.A93T
XH_P36	14	105353222	105353222	Frame_Shift_Ins	INS	-	C	-	CEP170B	c.2437dupC	p.K812fs
XH_P36	14	105408144	105408144	Missense_Mutation	SNP	C	A	C	AHNAK2	c.G13344T	p.K4448N
XH_P36	14	105408615	105408615	Silent	SNP	T	C	T	AHNAK2	c.A12873G	p.V4291V
XH_P36	14	105408617	105408617	Missense_Mutation	SNP	C	T	C	AHNAK2	c.G12871A	p.V4291I
XH_P36	14	105944635	105944635	Missense_Mutation	SNP	C	T	C	CRIP2	c.C298T	p.H100Y
XH_P36	15	22742754	22742754	Missense_Mutation	SNP	G	A	G	GOLGA6L1	c.G1139A	p.R380Q
XH_P36	15	22743174	22743174	Missense_Mutation	SNP	G	A	G	GOLGA6L1	c.G1559A	p.R520K
XH_P36	15	22743191	22743191	Silent	SNP	A	C	A	GOLGA6L1	c.A1576C	p.R526R
XH_P36	15	23021239	23021239	Frame_Shift_Del	DEL	T	-	T	NIPA2	c.98delA	p.K33fs
XH_P36	15	23889902	23889902	Silent	SNP	G	A	G	MAGEL2	c.C2988T	p.V996V
XH_P36	15	23891598	23891598	Missense_Mutation	SNP	C	T	C	MAGEL2	c.G1292A	p.R431Q
XH_P36	15	24921544	24921544	Missense_Mutation	SNP	G	T	G	NPAP1	c.G530T	p.R177M
XH_P36	15	24924254	24924254	Silent	SNP	A	G	A	NPAP1	c.A3240G	p.G1080G
XH_P36	15	24924255	24924255	Missense_Mutation	SNP	T	C	T	NPAP1	c.T3241C	p.Y1081H
XH_P36	15	26806145	26806145	Silent	SNP	C	T	C	GABRB3	c.G759A	p.R253R
XH_P36	15	27160004	27160004	Silent	SNP	G	A	G	GABRA5	c.G552A	p.A184A
XH_P36	15	27188381	27188381	Silent	SNP	C	T	C	GABRA5	c.C897T	p.T299T
XH_P36	15	28413663	28413663	Missense_Mutation	SNP	C	T	C	HERC2	c.G10303A	p.A3435T
XH_P36	15	28419605	28419605	Silent	SNP	C	T	C	HERC2	c.G9993A	p.T3331T
XH_P36	15	28437288	28437288	Missense_Mutation	SNP	G	A	G	HERC2	c.C8270T	p.A2757V
XH_P36	15	30659672	30659672	Silent	SNP	G	A	G	CHRFAM7A	c.C396T	p.I132I
XH_P36	15	35178833	35178833	Nonsense_Mutation	INS	-	TTTTTGAGTAG	-	AQR	TTCTAATCTACTC	R905delinsPYILIYSKKX
XH_P36	15	39874834	39874834	Missense_Mutation	SNP	G	A	G	THBS1	c.G508A	p.D170N
XH_P36	15	42159792	42159792	Nonsense_Mutation	SNP	G	A	G	SPTBN5	c.C6256T	p.Q2086X
XH_P36	15	42300063	42300063	Frame_Shift_Ins	INS	-	TGTTTGCCCCT	-	PLA2G4E	257insAGGGGC	p.L86fs
XH_P36	15	43814792	43814792	Frame_Shift_Ins	INS	-	AGGTCCT	-	MAP1A	21_1122insAGG	p.P374fs
XH_P36	15	43896265	43896265	Missense_Mutation	SNP	T	C	T	STRC	c.A4304G	p.E1435G
XH_P36	15	44907750	44907750	Frame_Shift_Del	DEL	A	-	A	SPG11	c.2849delT	p.L950fs
XH_P36	15	45409422	45409422	Missense_Mutation	SNP	G	A	G	DUOXA2	c.G688A	p.V230M
XH_P36	15	49776644	49776644	Frame_Shift_Del	DEL	A	-	A	FGF7	c.528delA	p.G176fs
XH_P36	15	50773790	50773790	Missense_Mutation	SNP	G	A	G	USP8	c.G1100A	p.R367H
XH_P36	15	50925185	50925185	Missense_Mutation	SNP	G	A	G	TRPM7	c.C1012T	p.L338F
XH_P36	15	51857323	51857323	Nonsense_Mutation	DEL	A	-	A	DMXL2	c.326delT	p.L109X
XH_P36	15	52901069	52901069	Frame_Shift_Del	DEL	T	-	T	FAM214A	c.2063delA	p.N688fs
XH_P36	15	55621922	55621922	Frame_Shift_Del	DEL	T	-	T	PIGB	c.523delT	p.F175fs
XH_P36	15	55759217	55759217	Nonsense_Mutation	INS	-	GTAAATCTATTC	-	DNAAF4	insTGTGAATAG	183_K184delinsLX
XH_P36	15	55912219	55912219	Frame_Shift_Del	DEL	G	-	G	PRTG	c.3444delC	p.P1148fs
XH_P36	15	62986516	62986516	Missense_Mutation	SNP	A	G	A	TLN2	c.A1217G	p.K406R
XH_P36	15	63102142	63102142	Missense_Mutation	SNP	G	A	G	TLN2	c.G6682A	p.D2228N
XH_P36	15	64791850	64791850	Frame_Shift_Ins	INS	-	CTTTCCTATT	-	ZNF609	2_233insCTTTCC	p.V78fs
XH_P36	15	65499170	65499170	Missense_Mutation	SNP	C	T	C	CILP	c.G374A	p.R125Q
XH_P36	15	66030257	66030257	Nonsense_Mutation	INS	-	TTGACAGATCTT	-	DENND4A	ATGGAAAGATC	p.Y276delinsX
XH_P36	15	69331298	69331298	Silent	SNP	C	T	C	NOX5	c.C1389T	p.P463P
XH_P36	15	73660540	73660540	Nonsense_Mutation	SNP	C	T	C	HCN4	c.G72A	p.W24X
XH_P36	15	75498563	75498563	Silent	SNP	C	T	C	C15orf39	c.C174T	p.A58A
XH_P36	15	75969242	75969242	Missense_Mutation	SNP	T	C	T	CSPG4	c.A5618G	p.Q1873R
XH_P36	15	79058543	79058543	Missense_Mutation	SNP	C	T	C	ADAMTS7	c.G3710A	p.S1237N
XH_P36	15	85164712	85164712	Missense_Mutation	SNP	G	A	G	ZSCAN2	c.G1286A	p.R429H
XH_P36	15	85407795	85407795	Missense_Mutation	SNP	C	T	C	ALPK3	c.C5228T	p.P1743L
XH_P36	15	89398826	89398826	Missense_Mutation	SNP	A	G	A	ACAN	c.A3010G	p.T1004A
XH_P36	15	89719201	89719201	Missense_Mutation	SNP	G	A	G	ABHD2	c.G697A	p.V233M
XH_P36	15	100591929	100591929	Missense_Mutation	SNP	A	G	A	ADAMTS17	c.T2303C	p.L768S
XH_P36	15	102242559	102242559	Silent	SNP	C	T	C	TARSL2	c.G1104A	p.P368P
XH_P36	15	102346763	102346763	Missense_Mutation	SNP	G	A	G	OR4F6	c.G841A	p.V281I
XH_P36	16	314836	314836	Missense_Mutation	SNP	G	A	G	FAM234A	c.G1474A	p.A492T
XH_P36	16	842546	842546	Frame_Shift_Del	DEL	G	-	G	CHTF18	c.1434delG	p.E478fs
XH_P36	16	1412305	1412305	Frame_Shift_Del	DEL	C	-	C	GNPTG	c.510delC	p.H170fs
XH_P36	16	1709929	1709929	Missense_Mutation	SNP	G	A	G	CRAMP1	c.G2278A	p.A760T
XH_P36	16	1779576	1779600	Frame_Shift_Del	DEL	GCGGGGCGC	-	CGCGGGGCGCG	MAPK8IP3	c.599_602del	p.R200fs

XH_P36	16	2762695	2762695	Missense_Mutation	SNP	C	T	C	PRSS27	c.G493A	p.A165T
XH_P36	16	2903887	2903887	Missense_Mutation	SNP	C	A	C	PRSS22	c.G696T	p.E232D
XH_P36	16	2983964	2983964	Silent	SNP	G	A	G	FLYWCH1	c.G1497A	p.A499A
XH_P36	16	3119304	3119304	Frame_Shift_Ins	INS	-	G	-	IL32	c.488_489insG	p.D163fs
XH_P36	16	3170007	3170007	Missense_Mutation	SNP	C	T	C	ZNF205	c.C1346T	p.P449L
XH_P36	16	4802553	4802553	Missense_Mutation	SNP	G	A	G	ZNF500	c.C1267T	p.H423Y
XH_P36	16	5061152	5061152	Frame_Shift_Del	DEL	C	-	C	SEC14L5	c.1857delC	p.S619fs
XH_P36	16	10867203	10867203	Frame_Shift_Del	DEL	A	-	A	TVP23A	c.420delT	p.F140fs
XH_P36	16	14970537	14970537	Missense_Mutation	SNP	A	C	A	NOMO1	c.A2533C	p.S845R
XH_P36	16	15457764	15457764	Missense_Mutation	SNP	G	A	G	NPIPA5	c.C805T	p.R269C
XH_P36	16	16228273	16228273	Missense_Mutation	SNP	G	A	G	ABCC1	c.G4033A	p.E1345K
XH_P36	16	19063046	19063046	Silent	SNP	G	A	G	TMC7	c.G753A	p.P251P
XH_P36	16	19126240	19126240	Missense_Mutation	SNP	C	T	C	ITPRIPL2	c.C457T	p.R153C
XH_P36	16	19883839	19883839	Missense_Mutation	SNP	G	A	G	GPRC5B	c.C722T	p.T241M
XH_P36	16	20359850	20359850	Nonsense_Mutation	SNP	C	T	C	UMOD	c.G773A	p.W258X
XH_P36	16	20948007	20948007	Silent	SNP	G	A	G	DNAH3	c.C11685T	p.Y3895Y
XH_P36	16	22326502	22326502	Nonsense_Mutation	SNP	G	A	G	POLR3E	c.G507A	p.W169X
XH_P36	16	24582721	24582721	Nonsense_Mutation	INS	-	TCTCAGGATACT	-	RBBP6	TTTCTTTCTCAG	_S1412delinsHFLSQDTX
XH_P36	16	24807240	24807240	Frame_Shift_Del	DEL	A	-	A	TNRC6A	c.3541delA	p.K1181fs
XH_P36	16	28354502	28354502	Missense_Mutation	SNP	A	C	A	NPIPB6	c.T704G	p.M235R
XH_P36	16	28354503	28354503	Frame_Shift_Ins	INS	-	CTCC	-	NPIPB6	702_703insGGA	p.M235fs
XH_P36	16	28898983	28898983	Missense_Mutation	SNP	C	T	C	ATP2A1	c.C493T	p.R165C
XH_P36	16	28913639	28913639	Frame_Shift_Ins	INS	-	C	-	ATP2A1	c.2082dupC	p.R694fs
XH_P36	16	29891248	29891248	Frame_Shift_Ins	INS	-	G	-	SEZ6L2	c.1167dupC	p.N390fs
XH_P36	16	30018105	30018105	Splice_Site	SNP	C	T	C	DOC2A	.	.
XH_P36	16	30680561	30680561	Silent	SNP	C	A	C	FBRS	c.C2538A	p.A846A
XH_P36	16	30793666	30793666	Silent	SNP	G	T	G	ZNF629	c.C1983A	p.T661T
XH_P36	16	30794963	30795046	In_Frame_Del	DEL	AGCTGAAGC	-	CAGCTGAAGCA	ZNF629	c.603_686del	p.201_229del
XH_P36	16	31121549	31121549	Silent	SNP	C	A	C	BCKDK	c.C447A	p.A149A
XH_P36	16	31142287	31142287	Nonsense_Mutation	SNP	C	T	C	KAT8	c.C1378T	p.Q460X
XH_P36	16	46836971	46836971	In_Frame_Ins	INS	-	TTACCAAGGAG	-	C16orf87	75insTACTCCTTG	K59delinsYSLVNK
XH_P36	16	48595224	48595224	Nonsense_Mutation	INS	-	AACCATTATTGC	-	N4BP1	AAGAGCAATAAT	445delinsVFLESFIFFFNFLX
XH_P36	16	49670972	49670972	Silent	SNP	G	A	G	ZNF423	c.C1740T	p.C580C
XH_P36	16	53265642	53265642	Frame_Shift_Del	DEL	A	-	A	CHD9	c.2457delA	p.A819fs
XH_P36	16	53706815	53706815	Missense_Mutation	SNP	A	T	A	RPGRIP1L	c.T996A	p.H332Q
XH_P36	16	55983808	55983808	Frame_Shift_Ins	INS	-	CTCACCTCTGGT	-	CES5A	sCCAACCAGAG	p.R17fs
XH_P36	16	67006630	67006630	Missense_Mutation	SNP	A	T	A	CES3	c.A418T	p.T140S
XH_P36	16	67472507	67472507	Missense_Mutation	SNP	C	T	C	ATP6V0D1	c.G980A	p.R327H
XH_P36	16	67687145	67687145	Frame_Shift_Del	DEL	G	-	G	CARMIL2	c.3108delG	p.P1036fs
XH_P36	16	67964372	67964372	Silent	SNP	G	A	G	CTRL	c.C438T	p.N146N
XH_P36	16	68021792	68021792	Missense_Mutation	SNP	C	T	C	DPEP2	c.G695A	p.R232H
XH_P36	16	70328814	70328814	Frame_Shift_Del	DEL	G	-	G	DDX19B	c.66delG	p.V22fs
XH_P36	16	72138457	72138457	Frame_Shift_Del	DEL	T	-	T	DHX38	c.2083delT	p.F695fs
XH_P36	16	72142164	72142164	Silent	SNP	C	T	C	DHX38	c.C3003T	p.F1001F
XH_P36	16	72153907	72153907	Silent	SNP	G	A	G	PMFBP1	c.C2850T	p.A950A
XH_P36	16	74660379	74660379	Silent	SNP	G	A	G	RFWD3	c.C2043T	p.I681I
XH_P36	16	75258768	75258768	Missense_Mutation	SNP	C	T	C	CTRB1	c.C662T	p.P221L
XH_P36	16	75564074	75564074	Missense_Mutation	SNP	G	A	G	CHST5	c.C209T	p.S70L
XH_P36	16	77325354	77325354	Missense_Mutation	SNP	C	T	C	ADAMTS18	c.G2695A	p.G899S
XH_P36	16	81047739	81047739	Splice_Site	INS	-	ACTTTTAACTTT	-	CENPN	.	.
XH_P36	16	81047740	81047740	Splice_Site	INS	-	TTAAAAATTAAC	-	CENPN	.	.
XH_P36	16	81095653	81095653	Missense_Mutation	SNP	C	T	C	C16orf46	c.G301A	p.D101N
XH_P36	16	81301689	81301689	Missense_Mutation	SNP	C	T	C	BCO1	c.C796T	p.R266W
XH_P36	16	84494362	84494362	Silent	SNP	C	T	C	ATP2C2	c.C1983T	p.S661S
XH_P36	16	85721176	85721176	Frame_Shift_Ins	INS	-	C	-	GINS2	c.94dupG	p.D32fs
XH_P36	16	87636771	87636771	Missense_Mutation	SNP	T	C	T	JPH3	c.T19C	p.F7L
XH_P36	16	88496785	88496785	Silent	SNP	G	A	G	ZNF469	c.G2907A	p.S969S
XH_P36	16	88870993	88870993	Missense_Mutation	SNP	C	A	C	CDT1	c.C269A	p.A90D
XH_P36	16	89614422	89614422	Missense_Mutation	SNP	G	A	G	SPG7	c.G1564A	p.A522T
XH_P36	16	90001937	90001937	Missense_Mutation	SNP	G	A	G	TUBB3	c.G862A	p.G288S
XH_P36	17	3563601	3563601	Missense_Mutation	SNP	G	A	G	CTNS	c.G1042A	p.V348I
XH_P36	17	3643130	3643130	Nonsense_Mutation	INS	-	CTTATTATCTCA	-	ITGAE	491insTTGAGAT	831_E832delinsLRX
XH_P36	17	3643131	3643131	Silent	SNP	G	T	G	ITGAE	c.C2490A	p.V830V
XH_P36	17	6531438	6531438	Frame_Shift_Del	DEL	T	-	T	KIAA0753	c.717delA	p.K239fs
XH_P36	17	7127698	7127698	Missense_Mutation	SNP	C	T	C	ACADVL	c.C1525T	p.R509W
XH_P36	17	7217630	7217630	Frame_Shift_Del	DEL	T	-	T	GPS2	c.297delA	p.K99fs
XH_P36	17	15234782	15234782	Nonsense_Mutation	INS	-	TAACCAACTATCA	-	TEKT3	121insTGATAGT	.N41_L42delinsX
XH_P36	17	15961041	15961041	In_Frame_Ins	INS	-	GACAAA	-	NCOR1	869_5870insTTT	.T1957delinsICP
XH_P36	17	15961042	15961042	In_Frame_Ins	INS	-	CCAGGG	-	NCOR1	868_5869insCCC	.T1957delinsPWT
XH_P36	17	16097954	16097954	Splice_Site	INS	-	TATCTTTTTTTTT	-	NCOR1	.	.
XH_P36	17	17696400	17696400	Silent	SNP	C	T	C	RAI1	c.C138T	p.L46L
XH_P36	17	18651334	18651334	Missense_Mutation	SNP	T	C	T	FBXW10	c.T586C	p.W196R
XH_P36	17	20163582	20163582	Missense_Mutation	SNP	G	A	G	SPECC1	c.G2672A	p.C891Y
XH_P36	17	21318768	21318768	Silent	SNP	G	A	G	KCNJ12	c.G114A	p.T38T
XH_P36	17	25910015	25910015	Frame_Shift_Ins	INS	-	C	-	KSR1	c.454dupC	p.T151fs
XH_P36	17	26708249	26708249	Silent	SNP	C	T	C	SARM1	c.C495T	p.G165G
XH_P36	17	26712159	26712159	Missense_Mutation	SNP	C	T	C	SARM1	c.C1492T	p.R498C
XH_P36	17	27002460	27002460	Frame_Shift_Del	DEL	A	-	A	SUPT6H	c.580delA	p.K194fs
XH_P36	17	27044426	27044426	Missense_Mutation	SNP	T	A	T	RAB34	c.A301T	p.T101S
XH_P36	17	27239943	27239943	Missense_Mutation	SNP	C	T	C	PHF12	c.G1646A	p.G549D
XH_P36	17	27903116	27903116	Missense_Mutation	SNP	C	T	C	GIT1	c.G1645A	p.V549I
XH_P36	17	28380517	28380517	Nonsense_Mutation	INS	-	AGCTATTAAATT	-	EFCAB5	insTTAGAGCTAT	9_Q460delinsELELLNX
XH_P36	17	28380902	28380902	Frame_Shift_Ins	INS	-	TCTACTTGACCC	-	EFCAB5	3insCCTTCTACTT	p.I588fs
XH_P36	17	28706682	28706682	Frame_Shift_Ins	INS	-	C	-	CPD	c.685dupC	p.E228fs
XH_P36	17	29527509	29527509	Missense_Mutation	SNP	G	A	G	NF1	c.G958A	p.A320T
XH_P36	17	29632268	29632268	Silent	SNP	G	A	G	EVI2B	c.C360T	p.A120A
XH_P36	17	33348680	33348680	Frame_Shift_Ins	INS	-	C	-	RFFL	c.300dupG	p.L101fs
XH_P36	17	33348682	33348682	Missense_Mutation	SNP	T	A	T	RFFL	c.A299T	p.E100V
XH_P36	17	34062267	34062267	Missense_Mutation	SNP	C	T	C	RASL10B	c.C64T	p.R22C
XH_P36	17	34893857	34893857	Missense_Mutation	SNP	C	T	C	PIGW	c.C907T	p.R303C
XH_P36	17	35914047	35914047	Frame_Shift_Ins	INS	-	CTTTGCAGATTTT	-	SYNRG	95insATAAAATC	p.P432fs
XH_P36	17	37650828	37650828	Nonsense_Mutation	INS	-	CTTACACGAGAT	-	CDK12	TTAATGCTTACA	p.F767delinsLLX
XH_P36	17	38936698	38936698	Missense_Mutation	SNP	C	T	C	KRT27	c.G538A	p.E180K
XH_P36	17	39340696	39340696	Silent	SNP	G	A	G	KRTAP4-1	c.C354T	p.C118C
XH_P36	17	40042503	40042503	Missense_Mutation	SNP	T	G	T	ACLY	c.A2066C	p.N689T
XH_P36	17	40940193	40940193	Frame_Shift_Ins	INS	-	C	-	WNK4	c.902dupC	p.S301fs
XH_P36	17	41154724	41154724	Missense_Mutation	SNP	G	A	G	RPL27	c.G286A	p.V96I
XH_P36	17	45234298	45234298	Missense_Mutation	SNP	G	C	G	CDC27	c.C640G	p.L214V

XH_P36	17	45234343	45234343	Missense_Mutation	SNP	T	G	T	CDC27	c.A595C	p.N199H
XH_P36	17	46154355	46154355	Frame_Shift_Del	DEL	T	-	T	CBX1	c.12delA	p.K4fs
XH_P36	17	46925466	46925466	Missense_Mutation	SNP	G	T	G	CALCOCO2	c.G5T	p.W2L
XH_P36	17	48470257	48470257	Missense_Mutation	SNP	G	A	G	LRRC59	c.C167T	p.S56L
XH_P36	17	48647125	48647125	Missense_Mutation	SNP	C	T	C	CACNA1G	c.C547T	p.R183C
XH_P36	17	48676979	48676979	Missense_Mutation	SNP	G	A	G	CACNA1G	c.G3380A	p.R1127Q
XH_P36	17	48692826	48692826	Missense_Mutation	SNP	G	A	G	CACNA1G	c.G4762A	p.G1588R
XH_P36	17	53392628	53392628	Silent	SNP	T	C	T	HLF	c.T237C	p.I79I
XH_P36	17	54520274	54520274	Missense_Mutation	SNP	C	T	C	ANKFN1	c.C1088T	p.P363L
XH_P36	17	56329792	56329792	Missense_Mutation	SNP	C	A	C	LPO	c.C781A	p.L261M
XH_P36	17	56385030	56385030	Missense_Mutation	SNP	G	A	G	TSPOAP1	c.C4745T	p.S1582L
XH_P36	17	56388417	56388417	Missense_Mutation	SNP	G	A	G	TSPOAP1	c.C3059T	p.P1020L
XH_P36	17	56400068	56400068	Missense_Mutation	SNP	G	A	G	TSPOAP1	c.C1084T	p.R362C
XH_P36	17	56438293	56438293	Nonsense_Mutation	SNP	G	A	G	RNF43	c.C319T	p.Q107X
XH_P36	17	56676693	56676693	Silent	SNP	C	T	C	TEX14	c.G2031A	p.A677A
XH_P36	17	57746175	57746175	Missense_Mutation	SNP	C	A	C	CLTC	c.C2178A	p.S726R
XH_P36	17	59465981	59465981	Frame_Shift_Del	DEL	A	-	A	BCAS3	c.2662delA	p.K888fs
XH_P36	17	59560599	59560599	Missense_Mutation	SNP	C	G	C	TBX4	c.C1360G	p.R454G
XH_P36	17	59560601	59560601	Frame_Shift_Ins	INS	-	CTGGGGTGGAC	-	TBX4	3insTGTCTGGG(	p.R454fs
XH_P36	17	60042378	60042378	Missense_Mutation	SNP	A	C	A	MED13	c.T4833G	p.D1611E
XH_P36	17	61497894	61497894	Frame_Shift_Del	DEL	C	-	C	TANC2	c.4551delC	p.S1517fs
XH_P36	17	65908303	65908303	Nonsense_Mutation	INS	-	TTATTTGATCAC	-	BPTF	TTAGCTTTATTT	435_V1436delinsIFX
XH_P36	17	66416494	66416494	Missense_Mutation	SNP	C	A	C	ARSG	c.C1468A	p.L490I
XH_P36	17	73555417	73555417	Silent	SNP	C	T	C	LLGL2	c.C456T	p.N152N
XH_P36	17	73646720	73646720	Nonstop_Mutation	SNP	T	C	T	RECQL5	c.A1308G	p.X436W
XH_P36	17	73759512	73759512	Frame_Shift_Del	DEL	G	-	G	GALK1	c.364delC	p.L122fs
XH_P36	17	73914025	73914025	Silent	SNP	G	A	G	FBF1	c.C2373T	p.D791D
XH_P36	17	74015081	74015081	Missense_Mutation	SNP	G	A	G	EVPL	c.C1198T	p.R400W
XH_P36	17	74072983	74072983	Missense_Mutation	SNP	G	A	G	GALR2	c.G635A	p.R212H
XH_P36	17	77758181	77758181	Silent	SNP	C	T	C	CBX2	c.C939T	p.S313S
XH_P36	17	78090823	78090823	Missense_Mutation	SNP	C	T	C	GAA	c.C2246T	p.A749V
XH_P36	17	79210603	79210603	Splice_Site	SNP	T	C	T	TEPSIN	.	.
XH_P36	17	79249771	79249771	Missense_Mutation	SNP	G	T	G	SLC38A10	c.C910A	p.Q304K
XH_P36	17	79769608	79769608	Missense_Mutation	SNP	C	A	C	GCGR	c.C739A	p.L247M
XH_P36	17	79792754	79792754	Missense_Mutation	SNP	C	T	C	PPP1R27	c.G76A	p.V26M
XH_P36	17	79826463	79826463	Frame_Shift_Del	DEL	G	-	G	ARHGDIA	c.792delC	p.P264fs
XH_P36	18	2775745	2775745	Missense_Mutation	SNP	C	T	C	SMCHD1	c.C5189T	p.A1730V
XH_P36	18	3116424	3116424	Frame_Shift_Del	DEL	C	-	C	MYOM1	c.2920delG	p.V974fs
XH_P36	18	8824907	8824907	Silent	SNP	C	T	C	MTCL1	c.C3399T	p.A1133A
XH_P36	18	9221979	9221979	Missense_Mutation	SNP	G	A	G	ANKRD12	c.G856A	p.D286N
XH_P36	18	9221980	9221980	Frame_Shift_Ins	INS	-	CCTCAACTAC	-	ANKRD12	7_858insCCTCAA	p.D286fs
XH_P36	18	9530853	9530853	Nonsense_Mutation	INS	-	TGTG	-	RALBP1	1369_1370insTG	Q457_E458delinsLX
XH_P36	18	9530855	9530855	In_Frame_Ins	INS	-	ACTCAATTCTGGT	-	RALBP1	nsAAATACTCAAT	457delinsQKYSILVN
XH_P36	18	10807272	10807272	Frame_Shift_Ins	INS	-	T	-	PIEZO2	c.917_918insA	p.R306fs
XH_P36	18	10807273	10807273	Splice_Site	INS	-	AGTGTTCTGGGT	-	PIEZO2	.	.
XH_P36	18	13057634	13057634	Missense_Mutation	SNP	G	A	G	CEP192	c.G4159A	p.G1387R
XH_P36	18	13114225	13114225	Frame_Shift_Ins	INS	-	ACTCTGTC	-	CEP192	64_7265insACTC	p.S2422fs
XH_P36	18	13731800	13731800	Nonsense_Mutation	INS	-	CTTGTAAACACTA	-	RNMT	TATTACTTGTAAA	95_G96delinsWYYLX
XH_P36	18	13746253	13746253	Frame_Shift_Del	DEL	A	-	A	RNMT	c.1174delA	p.K392fs
XH_P36	18	14538206	14538206	Silent	SNP	T	C	T	POTEC	c.A564G	p.V188V
XH_P36	18	21921518	21921518	Missense_Mutation	SNP	C	T	C	OSBPL1A	c.G387A	p.M129I
XH_P36	18	22057387	22057387	In_Frame_Ins	INS	-	GAA	-	HRH4	c.770_771insGA	p.W257delinsWK
XH_P36	18	22057389	22057389	Frame_Shift_Ins	INS	-	AATTGTATTGCTA	-	HRH4	773insAATTGTAT	p.L258fs
XH_P36	18	24127013	24127013	Frame_Shift_Del	DEL	G	-	G	KCTD1	c.1488delC	p.P496fs
XH_P36	18	28710536	28710536	Nonsense_Mutation	INS	-	GTTTGTTAATTCT	-	DSC1	AAAAGAGAATTA	F876delinsHIKENX
XH_P36	18	32825748	32825748	Missense_Mutation	SNP	G	A	G	ZNF397	c.G1079A	p.R360Q
XH_P36	18	43223957	43223957	Missense_Mutation	SNP	G	A	G	SLC14A2	c.G1183A	p.V395M
XH_P36	18	44102168	44102168	Missense_Mutation	SNP	G	A	G	LOXHD1	c.C1360T	p.R454C
XH_P36	18	47753971	47753971	Frame_Shift_Ins	INS	-	TTCTTTTTTCCCT	-	CFAP53	5insTCAGGGAAA	p.R442fs
XH_P36	18	47753973	47753973	Frame_Shift_Ins	INS	-	TTGTGTAA	-	CFAP53	22_1323insTTAC	p.Q441fs
XH_P36	18	55103822	55103822	Missense_Mutation	SNP	C	A	C	ONECUT2	c.C874A	p.L292M
XH_P36	18	56651438	56651438	Missense_Mutation	SNP	G	A	G	ZNF532	c.G3646A	p.V1216I
XH_P36	18	57013194	57013194	Frame_Shift_Del	DEL	T	-	T	LMAN1	c.912delA	p.K304fs
XH_P36	18	57022886	57022886	In_Frame_Ins	INS	-	TCTTTTGACAATG	-	LMAN1	CCATCATTGTCAA	3delinsAFPSLSKESKN
XH_P36	18	57022888	57022888	Missense_Mutation	SNP	C	T	C	LMAN1	c.G217A	p.A73T
XH_P36	18	66377374	66377374	Missense_Mutation	SNP	G	A	G	TMX3	c.C149T	p.A50V
XH_P36	18	74091072	74091072	Missense_Mutation	SNP	G	A	G	ZNF516	c.C2998T	p.R1000C
XH_P36	18	76753476	76753476	Silent	SNP	G	A	G	SALL3	c.G1485A	p.S495S
XH_P36	18	77624107	77624107	Missense_Mutation	SNP	C	T	C	KCNG2	c.C440T	p.A147V
XH_P36	19	436668	436668	Missense_Mutation	SNP	G	T	G	SHC2	c.C736A	p.H246N
XH_P36	19	629605	629605	Missense_Mutation	SNP	G	A	G	POLRMT	c.C757T	p.R253W
XH_P36	19	808433	808433	Silent	SNP	C	T	C	PTBP1	c.C1149T	p.D383D
XH_P36	19	1000483	1000483	Missense_Mutation	SNP	G	A	G	GRIN3B	c.G47A	p.G16E
XH_P36	19	1012099	1012099	Silent	SNP	G	A	G	TMEM259	c.C807T	p.S269S
XH_P36	19	1254317	1254317	Frame_Shift_Del	DEL	G	-	G	MIDN	c.536delG	p.R179fs
XH_P36	19	1585169	1585169	Missense_Mutation	SNP	T	C	T	MBD3	c.A155G	p.Y52C
XH_P36	19	1611736	1611736	Silent	SNP	G	A	G	TCF3	c.C1926T	p.S642S
XH_P36	19	1816767	1816767	Missense_Mutation	SNP	C	T	C	REXO1	c.G3247A	p.D1083N
XH_P36	19	1819941	1819941	Missense_Mutation	SNP	C	T	C	REXO1	c.G2642A	p.G881D
XH_P36	19	3831790	3831790	Missense_Mutation	SNP	C	T	C	ZFR2	c.G466A	p.G156R
XH_P36	19	3907867	3907867	Missense_Mutation	SNP	G	A	G	ATCAY	c.G494A	p.R165H
XH_P36	19	4207239	4207239	Missense_Mutation	SNP	C	T	C	ANKRD24	c.C467T	p.A156V
XH_P36	19	4322990	4322990	Silent	SNP	G	A	G	FSD1	c.G1047A	p.T349T
XH_P36	19	5245795	5245795	Missense_Mutation	SNP	G	A	G	PTPRS	c.C941T	p.T314M
XH_P36	19	6047495	6047495	Missense_Mutation	SNP	C	T	C	RFX2	c.G13A	p.E5K
XH_P36	19	6213976	6213976	Frame_Shift_Del	DEL	G	-	G	MLLT1	c.1381delC	p.Q461fs
XH_P36	19	6477251	6477251	Frame_Shift_Del	DEL	G	-	G	DENND1C	c.359delC	p.P120fs
XH_P36	19	6750380	6750380	Frame_Shift_Del	DEL	C	-	C	TRIP10	c.1305delC	p.D435fs
XH_P36	19	7970711	7970711	Missense_Mutation	SNP	G	T	G	MAP2K7	c.G143T	p.S48I
XH_P36	19	8156455	8156455	Missense_Mutation	SNP	C	A	C	FBN3	c.G5925T	p.E1975D
XH_P36	19	9018533	9018533	Silent	SNP	G	T	G	MUC16	c.C37641A	p.T12547T
XH_P36	19	9046704	9046704	In_Frame_Ins	INS	-	GACAACAATCTC	-	MUC16	34927insGAGAT	11643delinsEIVVS
XH_P36	19	9067934	9067934	In_Frame_Ins	INS	-	ATAGCAGTAACC	-	MUC16	12insATTGGTTA	504delinsSLVTAMF
XH_P36	19	9068827	9068827	Frame_Shift_Ins	INS	-	AG	-	MUC16	18618_18619ins	p.V6207fs
XH_P36	19	9068829	9068829	In_Frame_Ins	INS	-	ACACACACAATG	-	MUC16	18617insCATTG	D6206delinsALCVY
XH_P36	19	9084742	9084742	In_Frame_Ins	INS	-	ACACCTATTCTCT	-	MUC16	nsATCAAGAGAA	358delinsHQENRCYW
XH_P36	19	9086879	9086879	Missense_Mutation	SNP	C	A	C	MUC16	c.G4936T	p.G1646C
XH_P36	19	9087185	9087185	In_Frame_Ins	INS	-	ACAAACATT	-	MUC16	9_4630insAATG	V1544delinsNVCW

XH_P36	19	9271744	9271744	Missense_Mutation	SNP	G	A	G	ZNF317	c.G1327A	p.E443K
XH_P36	19	9577251	9577251	Nonstop_Mutation	INS	-	TTTTTTTTTTTTTG	-	ZNF560	nsCATCAAAAAA	685delinsSSKKKKE
XH_P36	19	9767278	9767278	Missense_Mutation	SNP	C	T	C	ZNF562	c.G77A	p.R26Q
XH_P36	19	9965152	9965152	Missense_Mutation	SNP	G	A	G	OLFM2	c.C841T	p.R281W
XH_P36	19	10446365	10446365	Frame_Shift_Del	DEL	G	-	G	ICAM3	c.631delC	p.R211fs
XH_P36	19	10577862	10577862	Silent	SNP	C	T	C	PDE4A	c.C1509T	p.V503V
XH_P36	19	10812678	10812678	Missense_Mutation	SNP	C	A	C	QTRT1	c.C296A	p.P99H
XH_P36	19	11141507	11141507	Missense_Mutation	SNP	G	A	G	SMARCA4	c.G3484A	p.G1162S
XH_P36	19	11725438	11725438	Frame_Shift_Ins	INS	-	CAAACATTTATT	-	ZNF627	nsTTTCCAAACAT	p.R34fs
XH_P36	19	11728278	11728278	In_Frame_Ins	INS	-	ATAAGGTTTCTC	-	ZNF627	insTTCATAAGGT	210delinsAFIRFLS
XH_P36	19	11891280	11891280	Frame_Shift_Ins	INS	-	GAGGTCTTTCATC	-	ZNF441	542insGAGGTCT	p.L214fs
XH_P36	19	12060335	12060335	Frame_Shift_Ins	INS	-	TGCTTGC	-	ZNF700	05_1506insTGC	p.I502fs
XH_P36	19	12060336	12060336	Frame_Shift_Ins	INS	-	TTTAT	-	ZNF700	506_1507insTT	p.I502fs
XH_P36	19	12461585	12461585	Frame_Shift_Ins	INS	-	AAGACACATGAT	-	ZNF442	4insTTATCATGT	p.K272fs
XH_P36	19	12502590	12502590	Frame_Shift_Del	DEL	A	-	A	ZNF799	c.622delT	p.W208fs
XH_P36	19	12812581	12812581	Silent	SNP	C	T	C	TNPO2	c.G2496A	p.P832P
XH_P36	19	13246192	13246192	Silent	SNP	C	T	C	NACC1	c.C171T	p.D57D
XH_P36	19	13319692	13319694	In_Frame_Del	DEL	GAT	-	GAT	CACNA1A	c.6659_6661del	p.2220_2221del
XH_P36	19	14584381	14584381	Missense_Mutation	SNP	C	T	C	PTGER1	c.G752A	p.R251H
XH_P36	19	14938341	14938341	Nonsense_Mutation	INS	-	AACTTGCTCCTTC	-	OR7A5	sAATAGAAGGAC	F238_S239delinsX
XH_P36	19	14939035	14939035	Frame_Shift_Ins	INS	-	TACTTCCACC	-	OR7A5	_19insGGTGGA	p.T7fs
XH_P36	19	14939037	14939037	Missense_Mutation	SNP	T	C	T	OR7A5	c.A17G	p.D6G
XH_P36	19	15291600	15291600	Missense_Mutation	SNP	C	T	C	NOTCH3	c.G3034A	p.G1012R
XH_P36	19	15562864	15562864	Missense_Mutation	SNP	T	C	T	RASAL3	c.A2884G	p.K962E
XH_P36	19	16633994	16633994	Frame_Shift_Del	DEL	G	-	G	CHERP	c.1849delC	p.H617fs
XH_P36	19	17397466	17397501	In_Frame_Del	DEL	GTGTGTGTGT	-	TGTGTGTGTGTG	ANKLE1	c.1898_1933del	p.633_645del
XH_P36	19	17449476	17449476	Missense_Mutation	SNP	C	T	C	GTPBP3	c.C517T	p.R173W
XH_P36	19	17611422	17611422	Silent	SNP	C	T	C	SLC27A1	c.C1452T	p.G484G
XH_P36	19	17716896	17716896	Silent	SNP	G	A	G	UNC13A	c.C5079T	p.S1693S
XH_P36	19	18377405	18377405	Frame_Shift_Ins	INS	-	ATGCCCACAA	-	KIAA1683	_945insTTGTGG	p.Q315fs
XH_P36	19	19137021	19137021	Nonsense_Mutation	INS	-	CAGGTCA	-	SUGP2	35_136insTGAC	.V46_P47delinsX
XH_P36	19	19368961	19368961	Missense_Mutation	SNP	C	T	C	HAPLN4	c.G874A	p.A292T
XH_P36	19	19646214	19646214	Missense_Mutation	SNP	G	T	G	YJEFN3	c.G348T	p.Q116H
XH_P36	19	19654762	19654762	Frame_Shift_Del	DEL	C	-	C	CILP2	c.1408delC	p.P470fs
XH_P36	19	20116817	20116817	Silent	SNP	C	T	C	ZNF682	c.G1398A	p.T466T
XH_P36	19	21132847	21132847	Silent	SNP	A	C	A	ZNF85	c.A1335C	p.P445P
XH_P36	19	21366317	21366317	Missense_Mutation	SNP	T	A	T	ZNF431	c.T1214A	p.V405E
XH_P36	19	21366318	21366318	Silent	SNP	G	A	G	ZNF431	c.G1215A	p.V405V
XH_P36	19	21476830	21476830	Missense_Mutation	SNP	C	A	C	ZNF708	c.G746T	p.C249F
XH_P36	19	21476831	21476831	Missense_Mutation	SNP	A	G	A	ZNF708	c.T745C	p.C249R
XH_P36	19	21606900	21606900	Missense_Mutation	SNP	G	A	G	ZNF493	c.G1055A	p.R352K
XH_P36	19	21991466	21991466	Missense_Mutation	SNP	A	T	A	ZNF43	c.T1178A	p.V393E
XH_P36	19	22155346	22155346	Silent	SNP	T	C	T	ZNF208	c.A2490G	p.E830E
XH_P36	19	22156714	22156714	Silent	SNP	G	A	G	ZNF208	c.C1122T	p.C374C
XH_P36	19	22363189	22363189	Missense_Mutation	SNP	C	T	C	ZNF676	c.G1330A	p.A444T
XH_P36	19	22375871	22375871	Nonsense_Mutation	INS	-	TTACAGTGATAG	-	ZNF676	TTGGCTATCACT	6_G27delinsLWLSLX
XH_P36	19	22497408	22497408	Missense_Mutation	SNP	G	A	G	ZNF729	c.G1189A	p.V397I
XH_P36	19	22499464	22499464	Missense_Mutation	SNP	A	G	A	ZNF729	c.A3245G	p.D1082G
XH_P36	19	22574593	22574593	Missense_Mutation	SNP	C	T	C	ZNF98	c.G1444A	p.V482I
XH_P36	19	22941717	22941717	Missense_Mutation	SNP	G	T	G	ZNF99	c.C994A	p.Q332K
XH_P36	19	23543701	23543701	Missense_Mutation	SNP	T	C	T	ZNF91	c.A1984G	p.T662A
XH_P36	19	23544625	23544625	Missense_Mutation	SNP	C	T	C	ZNF91	c.G1060A	p.A354T
XH_P36	19	23927290	23927290	Silent	SNP	G	A	G	ZNF681	c.C1062T	p.T354T
XH_P36	19	30312660	30312660	Missense_Mutation	SNP	C	T	C	CCNE1	c.C494T	p.A165V
XH_P36	19	30313453	30313453	Silent	SNP	C	T	C	CCNE1	c.C918T	p.G306G
XH_P36	19	30500119	30500124	In_Frame_Del	DEL	TGATGA	-	TGATGA	URI1	c.840_845del	p.280_282del
XH_P36	19	30934880	30934880	Frame_Shift_Del	DEL	G	-	G	ZNF536	c.411delG	p.K137fs
XH_P36	19	30934881	30934881	Missense_Mutation	SNP	C	A	C	ZNF536	c.C412A	p.R138S
XH_P36	19	31768042	31768042	Missense_Mutation	SNP	G	A	G	TSHZ3	c.C2657T	p.T886M
XH_P36	19	31768155	31768155	Silent	SNP	G	A	G	TSHZ3	c.C2544T	p.S848S
XH_P36	19	33167417	33167417	Frame_Shift_Ins	INS	-	G	-	RGS9BP	c.249dupG	p.S83fs
XH_P36	19	33167776	33167776	Frame_Shift_Del	DEL	G	-	G	RGS9BP	c.607delG	p.G203fs
XH_P36	19	35449607	35449607	Silent	SNP	A	G	A	ZNF792	c.T1152C	p.H384H
XH_P36	19	35524868	35524868	Missense_Mutation	SNP	C	T	C	SCN1B	c.C673T	p.R225C
XH_P36	19	36018393	36018393	Missense_Mutation	SNP	A	G	A	SBSN	c.T791C	p.V264A
XH_P36	19	36223175	36223175	Frame_Shift_Del	DEL	C	-	C	KMT2B	c.5725delC	p.P1909fs
XH_P36	19	36303630	36303630	Silent	SNP	G	A	G	PRODH2	c.C306T	p.G102G
XH_P36	19	36362899	36362899	Missense_Mutation	SNP	C	T	C	APLP1	c.C811T	p.P271S
XH_P36	19	36370092	36370092	Silent	SNP	C	T	C	APLP1	c.C1830T	p.Y610Y
XH_P36	19	36940815	36940815	Frame_Shift_Del	DEL	T	-	T	ZNF566	c.9delA	p.K3fs
XH_P36	19	37005981	37005981	Missense_Mutation	SNP	A	T	A	ZNF260	c.T160A	p.S54T
XH_P36	19	37039180	37039180	Nonsense_Mutation	INS	-	CATACTGGTGGA	-	ZNF529	GTTTTTTCCACCA	.D76_I77delinsX
XH_P36	19	37240584	37240584	Missense_Mutation	SNP	T	C	T	ZNF850	c.A1262G	p.E421G
XH_P36	19	37368718	37368718	Frame_Shift_Ins	INS	-	CTTACCACATTCA	-	ZNF345	AGGCCTTACCAC	p.L329fs
XH_P36	19	38028415	38028415	Frame_Shift_Del	DEL	T	-	T	ZNF793	c.855delT	p.C285fs
XH_P36	19	38655461	38655461	Missense_Mutation	SNP	G	A	G	SIPA1L3	c.G4123A	p.G1375S
XH_P36	19	38885396	38885396	Frame_Shift_Ins	INS	-	C	-	SPRED3	c.538dupC	p.T179fs
XH_P36	19	39200039	39200039	Missense_Mutation	SNP	A	G	A	ACTN4	c.A656G	p.D219G
XH_P36	19	39360822	39360822	Missense_Mutation	SNP	A	G	A	RINL	c.T1103C	p.L368P
XH_P36	19	39379738	39379738	Missense_Mutation	SNP	A	G	A	SIRT2	c.T379C	p.C127R
XH_P36	19	39734782	39734782	Missense_Mutation	SNP	C	T	C	IFNL3	c.G274A	p.V92M
XH_P36	19	41518269	41518269	Missense_Mutation	SNP	C	T	C	CYP2B6	c.C1031T	p.A344V
XH_P36	19	41631436	41631436	Silent	SNP	C	T	C	CYP2F1	c.C1191T	p.Y397Y
XH_P36	19	43528843	43528843	Silent	SNP	G	A	G	PSG11	c.C430T	p.L144L
XH_P36	19	44169554	44169554	Frame_Shift_Ins	INS	-	ATCCCCAACTCAT	-	PLAUR	24insATGAGTTC	p.R75fs
XH_P36	19	44660972	44660972	Nonsense_Mutation	INS	-	TGAGATGTAA	-	ZNF234	_804insTGAGAT	68_F269delinsAEMX
XH_P36	19	44660973	44660973	Frame_Shift_Ins	INS	-	AGAAGCTT	-	ZNF234	4_805insAGAAC	p.A268fs
XH_P36	19	44933181	44933181	Frame_Shift_Ins	INS	-	TGAATAAGAAGA	-	ZNF229	nsTCGGTTCTTCT	p.R586fs
XH_P36	19	44980744	44980744	Missense_Mutation	SNP	G	T	G	ZNF180	c.C1879A	p.Q627K
XH_P36	19	44980745	44980745	Frame_Shift_Ins	INS	-	ATTTTAAGGTAGT	-	ZNF180	ACATGTACTACCT	p.H626fs
XH_P36	19	45573321	45573321	Missense_Mutation	SNP	G	A	G	CLASRP	c.G1730A	p.R577H
XH_P36	19	46116858	46116858	Missense_Mutation	SNP	C	T	C	EML2	c.G1765A	p.A589T
XH_P36	19	46289347	46289347	Silent	SNP	C	T	C	DMWD	c.G1407A	p.T469T
XH_P36	19	48184060	48184060	Missense_Mutation	SNP	G	A	G	BICRA	c.G1633A	p.A545T
XH_P36	19	49000877	49000877	Missense_Mutation	SNP	A	G	A	LMTK3	c.T3536C	p.L1179P
XH_P36	19	49657889	49657889	In_Frame_Ins	INS	-	TCCTCC	-	HRC	05_606insGGAG	p.E202delinsEEE
XH_P36	19	49860534	49860534	Nonsense_Mutation	SNP	G	T	G	TEAD2	c.C335A	p.S112X
XH_P36	19	51170969	51170969	Silent	SNP	C	T	C	SHANK1	c.G4248A	p.A1416A

XH_P36	19	51601912	51601912	Frame_Shift_Del	DEL	C	-	C	CTU1	c.993delG	p.G331fs
XH_P36	19	52716080	52716080	Silent	SNP	C	T	C	PPP2R1A	c.C645T	p.D215D
XH_P36	19	53086110	53086110	Silent	SNP	T	A	T	ZNF701	c.T798A	p.G266G
XH_P36	19	53643823	53643823	Missense_Mutation	SNP	G	A	G	ZNF347	c.C2261T	p.A754V
XH_P36	19	53644695	53644695	Silent	SNP	G	A	G	ZNF347	c.C1389T	p.C463C
XH_P36	19	53855284	53855284	Silent	SNP	G	A	G	ZNF845	c.G1356A	p.S452S
XH_P36	19	55481600	55481600	Missense_Mutation	SNP	C	A	C	NLRP2	c.C217A	p.L73I
XH_P36	19	56127331	56127331	Frame_Shift_Del	DEL	G	-	G	ZNF865	c.2347delG	p.G783fs
XH_P36	19	56953857	56953857	Frame_Shift_Ins	INS	-	GATGTCATCCCT	-	ZNF667	GTAAAAGGGAT	p.E169fs
XH_P36	19	57036570	57036570	In_Frame_Ins	INS	-	CTTACATTTATAT	-	ZNF471	13insCCTTACATT	I304delinsIPYIYM
XH_P36	19	57050528	57050528	Silent	SNP	G	A	G	ZFP28	c.G141A	p.P47P
XH_P36	19	57133248	57133248	Missense_Mutation	SNP	G	A	G	ZNF71	c.G593A	p.R198H
XH_P36	19	57640277	57640277	Nonsense_Mutation	INS	-	CATCCACATATA	-	USP29	5insCTCATCCAC	78_N79delinsNLIHIX
XH_P36	19	57932842	57932842	Frame_Shift_Ins	INS	-	ATTTG	-	ZNF17	982_1983insAT	p.T661fs
XH_P36	19	58049281	58049281	In_Frame_Ins	INS	-	CCCTGCTTC	-	ZNF549	0_871insCCCTG	.S290delinsSPCF
XH_P36	19	58050159	58050159	Nonsense_Mutation	INS	-	TTTGTGATGCTT	-	ZNF549	GTGGATTTTGTG	Y583_K584delinsX
XH_P36	19	58152336	58152336	Missense_Mutation	SNP	C	T	C	ZNF211	c.C299T	p.A100V
XH_P36	19	58213374	58213374	Missense_Mutation	SNP	T	C	T	ZNF154	c.A943G	p.S315G
XH_P36	19	58485478	58485478	Missense_Mutation	SNP	G	A	G	C19orf18	c.C215T	p.S72L
XH_P36	19	58629726	58629726	Missense_Mutation	SNP	G	A	G	ZSCAN18	c.C38T	p.T13M
XH_P36	20	1460483	1460483	Frame_Shift_Ins	INS	-	AACAATACATAT	-	SIRPB2	TACAAATATGTA	p.P105fs
XH_P36	20	3003413	3003413	Silent	SNP	C	T	C	PTPRA	c.C1380T	p.D460D
XH_P36	20	3003414	3003414	Missense_Mutation	SNP	G	A	G	PTPRA	c.G1381A	p.V461M
XH_P36	20	4228843	4228843	Silent	SNP	G	A	G	ADRA1D	c.C762T	p.Y254Y
XH_P36	20	5903414	5903414	Frame_Shift_Del	DEL	A	-	A	CHGB	c.624delA	p.I208fs
XH_P36	20	14307546	14307546	Missense_Mutation	SNP	G	A	G	FLRT3	c.C607T	p.R203C
XH_P36	20	16347868	16347868	Missense_Mutation	SNP	C	T	C	KIF16B	c.G4102A	p.A1368T
XH_P36	20	18286958	18286958	Missense_Mutation	SNP	A	T	A	ZNF133	c.A180T	p.K60N
XH_P36	20	18296347	18296347	Silent	SNP	G	A	G	ZNF133	c.G795A	p.T265T
XH_P36	20	23345348	23345348	Frame_Shift_Ins	INS	-	AG	-	GZF1	c.328_329insAG	p.L110fs
XH_P36	20	23345349	23345349	Nonsense_Mutation	INS	-	GGTTAAATGGG	-	GZF1	30insAGGTTAAA	L110_E111delinsLGX
XH_P36	20	25000780	25000780	Missense_Mutation	SNP	C	T	C	ACSS1	c.G749A	p.R250Q
XH_P36	20	25457670	25457670	Missense_Mutation	SNP	C	T	C	NINL	c.G2257A	p.A753T
XH_P36	20	30606904	30606904	Frame_Shift_Del	DEL	G	-	G	CCM2L	c.625delG	p.G209fs
XH_P36	20	32664877	32664877	Silent	SNP	C	T	C	RALY	c.C654T	p.G218G
XH_P36	20	33328811	33328811	In_Frame_Ins	INS	-	CCC	-	NCOA6	5248_5249insG	.S1750delinsWA
XH_P36	20	33328812	33328812	Frame_Shift_Ins	INS	-	GTCT	-	NCOA6	247_5248insAG	p.S1750fs
XH_P36	20	33345744	33345744	Silent	SNP	C	T	C	NCOA6	c.G807A	p.Q269Q
XH_P36	20	33589096	33589096	Missense_Mutation	SNP	G	A	G	MYH7B	c.G5650A	p.E1884K
XH_P36	20	34022391	34022391	Missense_Mutation	SNP	C	T	C	GDF5	c.C802T	p.R268W
XH_P36	20	34208670	34208670	Missense_Mutation	SNP	C	T	C	SPAG4	c.C911T	p.S304L
XH_P36	20	36031782	36031782	Nonstop_Mutation	SNP	G	T	G	SRC	c.G1611T	p.X537Y
XH_P36	20	37357196	37357196	Missense_Mutation	SNP	G	A	G	SLC32A1	c.G1492A	p.V498I
XH_P36	20	42788776	42788776	Silent	SNP	G	A	G	JPH2	c.C651T	p.G217G
XH_P36	20	43850774	43850774	Nonsense_Mutation	INS	-	TATGTTGTTGGT	-	SEMG2	ATTTAGTATGTTG	.W167delinsWIX
XH_P36	20	44530971	44530971	Silent	SNP	G	A	G	PLTP	c.C825T	p.D275D
XH_P36	20	45002133	45002133	Frame_Shift_Del	DEL	G	-	G	ELMO2	c.1321delC	p.H441fs
XH_P36	20	56090848	56090848	Missense_Mutation	SNP	G	A	G	CTCFL	c.C316T	p.R106C
XH_P36	20	57768838	57768838	Missense_Mutation	SNP	C	A	C	ZNF831	c.C2764A	p.P922T
XH_P36	20	57768886	57768886	Frame_Shift_Ins	INS	-	C	-	ZNF831	c.2813dupC	p.S938fs
XH_P36	20	60584133	60584133	Missense_Mutation	SNP	A	G	A	TAF4	c.T1859C	p.V620A
XH_P36	20	60705321	60705321	Silent	SNP	C	T	C	LSM14B	c.C642T	p.D214D
XH_P36	20	60776082	60776082	Silent	SNP	C	T	C	MTG2	c.C1170T	p.D390D
XH_P36	20	61982271	61982271	Silent	SNP	G	A	G	CHRNA4	c.C492T	p.D164D
XH_P36	20	62196336	62196336	Missense_Mutation	SNP	A	G	A	HELZ2	c.T2132C	p.L711P
XH_P36	20	62199927	62199927	Missense_Mutation	SNP	C	G	C	HELZ2	c.G1514C	p.R505T
XH_P36	20	62591287	62591287	Frame_Shift_Del	DEL	C	-	C	ZNF512B	c.2633delG	p.G878fs
XH_P36	21	10908835	10908835	Nonsense_Mutation	SNP	C	A	C	TPTE	c.G1096T	p.E366X
XH_P36	21	10908837	10908837	Frame_Shift_Ins	INS	-	TTGTTCTTGAGA	-	TPTE	AGTGGGTCTCAA	p.I365fs
XH_P36	21	22664532	22664532	Missense_Mutation	SNP	A	T	A	NCAM2	c.A590T	p.D197V
XH_P36	21	30343059	30343059	Nonsense_Mutation	INS	-	ATTCATTAAATAC	-	LTN1	TTAAAAGTATTT	p.C376delinsX
XH_P36	21	32589885	32589885	Missense_Mutation	SNP	C	T	C	TIAM1	c.G2126A	p.R709Q
XH_P36	21	42866456	42866456	Missense_Mutation	SNP	G	A	G	TMPRSS2	c.C176T	p.P59L
XH_P36	21	43510462	43510462	Missense_Mutation	SNP	G	A	G	UMODL1	c.G845A	p.C282Y
XH_P36	21	45389100	45389100	Missense_Mutation	SNP	G	T	G	AGPAT3	c.G450T	p.E150D
XH_P36	21	45877262	45877262	Silent	SNP	C	T	C	LRRC3	c.C735T	p.S245S
XH_P36	21	47648492	47648492	Silent	SNP	G	C	G	LSS	c.C36G	p.G12G
XH_P36	22	17601702	17601702	Frame_Shift_Ins	INS	-	G	-	CECR6	c.315dupC	p.A106fs
XH_P36	22	18300932	18300932	Frame_Shift_Del	DEL	G	-	G	MICAL3	c.4495delC	p.R1499fs
XH_P36	22	19808804	19808804	Silent	SNP	C	T	C	GNB1L	c.G75A	p.A25A
XH_P36	22	20843349	20843349	Silent	SNP	G	A	G	KLHL22	c.C150T	p.F50F
XH_P36	22	21800025	21800025	Frame_Shift_Del	DEL	C	-	C	HIC2	c.841delC	p.P281fs
XH_P36	22	24124503	24124503	Missense_Mutation	SNP	C	T	C	MMP11	c.C1166T	p.A389V
XH_P36	22	24836696	24836696	Missense_Mutation	SNP	G	A	G	ADORA2A	c.G478A	p.G160R
XH_P36	22	24837032	24837032	Missense_Mutation	SNP	C	A	C	ADORA2A	c.C814A	p.L272M
XH_P36	22	25625546	25625546	Splice_Site	SNP	G	A	G	CRYBB2	.	.
XH_P36	22	26239838	26239838	Silent	SNP	C	A	C	MYO18B	c.C3348A	p.P1116P
XH_P36	22	29446275	29446275	Frame_Shift_Del	DEL	G	-	G	ZNRF3	c.2106delG	p.K702fs
XH_P36	22	29837600	29837600	Missense_Mutation	SNP	G	A	G	RFPL1	c.G443A	p.R148Q
XH_P36	22	30074208	30074208	Silent	SNP	G	A	G	NF2	c.G1221A	p.P407P
XH_P36	22	31998711	31998711	Missense_Mutation	SNP	A	G	A	SFI1	c.A1580G	p.H527R
XH_P36	22	32937634	32937634	Silent	SNP	G	A	G	SYN3	c.C840T	p.Y280Y
XH_P36	22	34022242	34022242	Silent	SNP	G	A	G	LARGE1	c.C477T	p.S159S
XH_P36	22	36624038	36624038	Missense_Mutation	SNP	T	G	T	APOL2	c.A426C	p.E142D
XH_P36	22	37770438	37770438	Silent	SNP	C	T	C	ELFN2	c.G1137A	p.T379T
XH_P36	22	38621499	38621499	Missense_Mutation	SNP	A	T	A	TMEM184B	c.T719A	p.L240H
XH_P36	22	38621500	38621500	Missense_Mutation	SNP	G	T	G	TMEM184B	c.C718A	p.L240I
XH_P36	22	39262986	39262986	Missense_Mutation	SNP	G	A	G	CBX6	c.C413T	p.T138M
XH_P36	22	40800400	40800400	Missense_Mutation	SNP	C	T	C	SGSM3	c.C118T	p.R40C
XH_P36	22	40802644	40802644	Missense_Mutation	SNP	G	A	G	SGSM3	c.G974A	p.G325D
XH_P36	22	40815071	40815071	Frame_Shift_Del	DEL	G	-	G	MKL1	c.1371delC	p.P457fs
XH_P36	22	42024155	42024155	Frame_Shift_Del	DEL	T	-	T	XRCC6	c.116delT	p.I39fs
XH_P36	22	44276750	44276750	Silent	SNP	G	A	G	PNPLA5	c.C873T	p.R291R
XH_P36	22	44681360	44681360	Missense_Mutation	SNP	G	A	G	KIAA1644	c.C547T	p.R183W
XH_P36	22	46654519	46654519	Frame_Shift_Ins	INS	-	ACGAGGA	-	PKDREJ	00_4701insTCCT	p.K1567fs
XH_P36	22	46932163	46932163	Missense_Mutation	SNP	G	A	G	CELSR1	c.C905T	p.T302M
XH_P36	22	50297990	50297990	Frame_Shift_Ins	INS	-	G	-	ALG12	c.1156dupC	p.Q386fs
XH_P36	22	50591520	50591520	Missense_Mutation	SNP	T	C	T	MOV10L1	c.T320C	p.M107T

XH_P36	22	50658895	50658895	Frame_Shift_Del	DEL	G	-	G	TUBGCP6	c.3893delC	p.P1298fs
XH_P36	22	50728589	50728589	Missense_Mutation	SNP	G	A	G	PLXNB2	c.C425T	p.S142F
XH_P36	22	50987509	50987509	Missense_Mutation	SNP	G	A	G	KLHDC7B	c.G914A	p.G305D
XH_P36	X	2773043	2773043	Missense_Mutation	SNP	A	G	A	GYG2	c.A334G	p.N112D
XH_P36	X	3240378	3240378	Nonsense_Mutation	INS	-	AGCACCTGACATT	-	MXRA5	8insTTAATGTCA	116_V1117delinsTX
XH_P36	X	5811016	5811016	Missense_Mutation	SNP	G	A	G	NLGN4X	c.C2293T	p.R765C
XH_P36	X	5821876	5821876	Silent	SNP	G	A	G	NLGN4X	c.C843T	p.G281G
XH_P36	X	11197525	11197525	Frame_Shift_Ins	INS	-	TTTCCTGATAACT	-	ARHGAP6	AAAAAGTTATCA	p.E279fs
XH_P36	X	12904643	12904643	Frame_Shift_Ins	INS	-	C	-	TLR7	c.1017dupC	p.L339fs
XH_P36	X	14599388	14599388	Frame_Shift_Ins	INS	-	T	-	GLRA2	c.355dupT	p.D118fs
XH_P36	X	17750296	17750296	Silent	SNP	C	T	C	NHS	c.C4074T	p.S1358S
XH_P36	X	21444726	21444726	Missense_Mutation	SNP	G	A	G	CNKSR2	c.G176A	p.R59H
XH_P36	X	22056595	22056595	Missense_Mutation	SNP	G	T	G	PHEX	c.G127T	p.G43C
XH_P36	X	22292144	22292144	Frame_Shift_Ins	INS	-	A	-	ZNF645	c.1037dupA	p.Q346fs
XH_P36	X	23410920	23410920	Missense_Mutation	SNP	G	T	G	PTCHD1	c.G1285T	p.G429C
XH_P36	X	24734566	24734566	Missense_Mutation	SNP	G	A	G	POLA1	c.G613A	p.A205T
XH_P36	X	24859773	24859773	Silent	SNP	T	C	T	POLA1	c.T3741C	p.L1247L
XH_P36	X	27481249	27481249	Missense_Mutation	SNP	C	A	C	PPP4R3CP	c.G165T	p.Q55H
XH_P36	X	30327013	30327013	Silent	SNP	C	T	C	NR0B1	c.G468A	p.T156T
XH_P36	X	30327352	30327352	Silent	SNP	G	A	G	NR0B1	c.C129T	p.C43C
XH_P36	X	30877624	30877624	Missense_Mutation	SNP	C	T	C	TAB3	c.G82A	p.V28M
XH_P36	X	31986601	31986601	Missense_Mutation	SNP	T	C	T	DMD	c.A2446G	p.T816A
XH_P36	X	32361392	32361392	Frame_Shift_Ins	INS	-	TGATGCATTTCC	-	DMD	75insAAGGAAAT	p.S525fs
XH_P36	X	36321999	36321999	Missense_Mutation	SNP	C	T	C	CFAP47	c.C8006T	p.T2669M
XH_P36	X	37028698	37028698	Missense_Mutation	SNP	C	T	C	FAM47C	c.C2215T	p.R739C
XH_P36	X	39923654	39923654	Missense_Mutation	SNP	T	C	T	BCOR	c.A3383G	p.E1128G
XH_P36	X	45023492	45023492	Frame_Shift_Ins	INS	-	GC	-	CXorf36	c.500_501insGC	p.D167fs
XH_P36	X	45023493	45023493	Frame_Shift_Ins	INS	-	A	-	CXorf36	c.499_500insT	p.D167fs
XH_P36	X	47271966	47271966	Frame_Shift_Ins	INS	-	GTATAAAATTAA	-	ZNF157	5insCAGTATAAA	p.H165fs
XH_P36	X	47516631	47516631	Silent	SNP	G	A	G	UXT	c.C343T	p.L115L
XH_P36	X	48418176	48418176	Missense_Mutation	SNP	C	T	C	TBC1D25	c.C928T	p.R310C
XH_P36	X	48559092	48559092	Missense_Mutation	SNP	G	A	G	SUV39H1	c.G809A	p.R270H
XH_P36	X	49088214	49088214	Silent	SNP	C	T	C	CACNA1F	c.G201A	p.R67R
XH_P36	X	53106544	53106544	Silent	SNP	C	T	C	GPR173	c.C741T	p.A247A
XH_P36	X	53115191	53115191	Silent	SNP	C	T	C	TSPYL2	c.C1617T	p.Y539Y
XH_P36	X	53564586	53564586	Missense_Mutation	SNP	C	T	C	HUWE1	c.G12068A	p.R4023H
XH_P36	X	54112348	54112348	In_Frame_Ins	INS	-	AAGAATGAGGT	-	FAM120C	nsTAAATACCTCA	880delinsVNTSFFGT
XH_P36	X	66765862	66765862	Missense_Mutation	SNP	G	T	G	AR	c.G874T	p.G292C
XH_P36	X	69510571	69510571	Frame_Shift_Ins	INS	-	CTTCCTTC	-	KIF4A	51_152insCTTCC	p.Y51fs
XH_P36	X	69510573	69510573	Silent	SNP	C	T	C	KIF4A	c.C153T	p.Y51Y
XH_P36	X	69669650	69669650	Missense_Mutation	SNP	G	T	G	DLG3	c.G644T	p.R215L
XH_P36	X	70472854	70472854	Frame_Shift_Del	DEL	C	-	C	ZMYM3	c.252delG	p.G84fs
XH_P36	X	70586340	70586340	Missense_Mutation	SNP	A	T	A	TAF1	c.A176T	p.D59V
XH_P36	X	71426778	71426778	Nonsense_Mutation	INS	-	AGAAAGCTCAA	-	ERCC6L	_1839insTTGAG	613_N614delinsNX
XH_P36	X	74318786	74318786	Silent	SNP	A	G	A	ABCB7	c.T366C	p.G122G
XH_P36	X	76938995	76938995	In_Frame_Ins	INS	-	AGATGAAGAATC	-	ATRX	_1639insCATTCT	E547delinsHSSSE
XH_P36	X	77224989	77224989	Silent	SNP	G	T	G	PGAM4	c.C147A	p.G49G
XH_P36	X	77268382	77268382	Missense_Mutation	SNP	G	A	G	ATP7A	c.G2179A	p.G727R
XH_P36	X	79699009	79699009	Nonsense_Mutation	INS	-	TCAGGTTTCCTGC	-	FAM46D	TCTCTTCAGGTTT	L324_I325delinsLX
XH_P36	X	79989683	79989683	Frame_Shift_Ins	INS	-	AATTT	-	BRWD3	019_1020insAA	p.H340fs
XH_P36	X	82763673	82763673	Frame_Shift_Del	DEL	G	-	G	POU3F4	c.341delG	p.W114fs
XH_P36	X	91132649	91132649	Silent	SNP	C	T	C	PCDH11X	c.C1410T	p.F470F
XH_P36	X	91133364	91133364	Missense_Mutation	SNP	G	A	G	PCDH11X	c.G2125A	p.A709T
XH_P36	X	95993734	95993734	Missense_Mutation	SNP	A	T	A	DIAPH2	c.A315T	p.E105D
XH_P36	X	99884976	99884976	Nonstop_Mutation	INS	-	CCCTCTGAAACA	-	TSPAN6	sCCAGTGTTTCA	138delinsSSVSEGY
XH_P36	X	100297082	100297082	Frame_Shift_Ins	INS	-	T	-	TRMT2B	c.196dupA	p.S66fs
XH_P36	X	100503224	100503224	Missense_Mutation	SNP	A	G	A	DRP2	c.A1165G	p.N389D
XH_P36	X	100667681	100667681	Frame_Shift_Ins	INS	-	ATTCTTTCCTTGT	-	HNRNPH2	06insCTATTCTTT	p.A235fs
XH_P36	X	100747417	100747417	Missense_Mutation	SNP	G	A	G	ARMCX4	c.G3841A	p.A1281T
XH_P36	X	101970716	101970716	Missense_Mutation	SNP	A	G	A	RMCX5-GPRASF	c.A919G	p.R307G
XH_P36	X	102864157	102864157	Frame_Shift_Ins	INS	-	CTTCATTTTATT	-	TCEAL3	insTTCCTTCATT	p.G55fs
XH_P36	X	103080247	103080247	Missense_Mutation	SNP	T	G	T	RAB9B	c.A468C	p.K156N
XH_P36	X	105450411	105450411	Missense_Mutation	SNP	A	T	A	MUM1L1	c.A986T	p.D329V
XH_P36	X	105450735	105450735	Missense_Mutation	SNP	G	A	G	MUM1L1	c.G1310A	p.R437K
XH_P36	X	106038848	106038848	Frame_Shift_Ins	INS	-	CCTCTAATAATTA	-	RNF128	15insTACCTCTAA	p.V372fs
XH_P36	X	106038850	106038850	In_Frame_Ins	INS	-	ATTTAT	-	RNF128	116_1117insATT	p.V372delinsVIY
XH_P36	X	106845186	106845186	Frame_Shift_Del	DEL	G	-	G	FRMPD3	c.4016delG	p.R1339fs
XH_P36	X	107978741	107978741	Missense_Mutation	SNP	C	A	C	IRS4	c.G834T	p.Q278H
XH_P36	X	109693960	109693960	Missense_Mutation	SNP	G	A	G	RTL9	c.G115A	p.V39I
XH_P36	X	112065649	112065649	Nonsense_Mutation	SNP	G	A	G	AMOT	c.C706T	p.R236X
XH_P36	X	114082653	114082653	Frame_Shift_Ins	INS	-	AGATTATGTCTG	-	HTR2C	TCTTTCAGATTAT	p.C146fs
XH_P36	X	114424574	114424574	Silent	SNP	C	A	C	RBMXL3	c.C570A	p.G190G
XH_P36	X	114426364	114426364	Missense_Mutation	SNP	T	A	T	RBMXL3	c.T2360A	p.L787H
XH_P36	X	117043403	117043403	Nonsense_Mutation	INS	-	TGTAGTTAGGGT	-	KLHL13	AAATAAAACCCT	p.D403delinsEX
XH_P36	X	118723709	118723709	Frame_Shift_Ins	INS	-	GAGATAGGCTCC	-	NKRF	insTACGGGAGC	p.R560fs
XH_P36	X	118920652	118920652	Missense_Mutation	SNP	G	T	G	RPL39	c.C151A	p.L51I
XH_P36	X	119602985	119602985	Missense_Mutation	SNP	G	A	G	LAMP2	c.C40T	p.L14F
XH_P36	X	119674393	119674393	In_Frame_Ins	INS	-	GGTTGCTTAGCTC	-	CUL4B	ATATACCAGCTAA	0delinsDIPAKQPQTN
XH_P36	X	119694376	119694376	Nonsense_Mutation	INS	-	AATAAGAGTATA	-	CUL4B	insTTAATATACTC	0_L41delinsLIYSYFX
XH_P36	X	122387364	122387364	Missense_Mutation	SNP	A	G	A	GRIA3	c.A479G	p.K160R
XH_P36	X	123200251	123200251	Missense_Mutation	SNP	C	A	C	STAG2	c.C2230A	p.Q744K
XH_P36	X	123514660	123514660	Missense_Mutation	SNP	T	C	T	TENM1	c.A7904G	p.Y2635C
XH_P36	X	123637491	123637491	Missense_Mutation	SNP	C	T	C	TENM1	c.G3364A	p.V1122I
XH_P36	X	123663816	123663816	Missense_Mutation	SNP	C	T	C	TENM1	c.G2669A	p.R890Q
XH_P36	X	123680932	123680932	In_Frame_Ins	INS	-	ACA	-	TENM1	2442_2443insTC	p.T815delinsCT
XH_P36	X	123680934	123680934	Frame_Shift_Ins	INS	-	TTATTTTATTCCT	-	TENM1	CACTAAGGAATA	p.L814fs
XH_P36	X	124454042	124454042	Missense_Mutation	SNP	T	C	T	TEX13C	c.T74C	p.V25A
XH_P36	X	124454575	124454575	Missense_Mutation	SNP	C	T	C	TEX13C	c.C607T	p.P203S
XH_P36	X	125685317	125685317	Missense_Mutation	SNP	T	G	T	DCAF12L1	c.A1275C	p.E425D
XH_P36	X	128621013	128621013	Missense_Mutation	SNP	A	T	A	SMARCA1	c.T2163A	p.D721E
XH_P36	X	129147458	129147458	Missense_Mutation	SNP	T	A	T	BCORL1	c.T710A	p.V237D
XH_P36	X	129147932	129147932	Missense_Mutation	SNP	C	A	C	BCORL1	c.C1184A	p.T395K
XH_P36	X	129270637	129270637	Missense_Mutation	SNP	T	C	T	AIFM1	c.A128G	p.K43R
XH_P36	X	131212863	131212863	Silent	SNP	C	T	C	FRMD7	c.G1137A	p.E379E
XH_P36	X	135313108	135313108	Frame_Shift_Ins	INS	-	AAAAAGGGATTT	-	MAP7D3	1326insTAAATC	p.K442fs
XH_P36	X	135630842	135630842	Silent	SNP	C	T	C	VGLL1	c.C309T	p.N103N
XH_P36	X	136649264	136649264	Silent	SNP	G	A	G	ZIC3	c.G414A	p.G138G
XH_P36	X	142718126	142718126	Frame_Shift_Ins	INS	-	AAAAATATACAC	-	SLITRK4	ATAGACTGTATA	p.G267fs

XH_P36	X	148037594	148037594	Frame_Shift_Ins	INS	-	TTTCTGGAATTT	-	AFF2	nsTTTGTTTCTGG	p.A314fs
XH_P36	X	148627313	148627313	Missense_Mutation	SNP	G	T	G	CXorf40A	c.G137T	p.R46M
XH_P36	X	148794840	148794840	Frame_Shift_Ins	INS	-	G	-	MAGEA11	c.22dupG	p.R7fs
XH_P36	X	150906974	150906974	Missense_Mutation	SNP	G	T	G	CNGA2	c.G19T	p.G7C
XH_P36	X	152139042	152139042	Missense_Mutation	SNP	G	A	G	ZNF185	c.G941A	p.R314H
XH_P36	X	152686163	152686163	Frame_Shift_Del	DEL	G	-	G	ZFP92	c.328delG	p.G110fs
XH_P36	X	152710513	152710513	Missense_Mutation	SNP	G	T	G	TREX2	c.C376A	p.L126M
XH_P36	X	153048467	153048467	Silent	SNP	G	A	G	SRPK3	c.G642A	p.K214K
XH_P36	X	153050304	153050304	Missense_Mutation	SNP	G	A	G	SRPK3	c.G1345A	p.A449T
XH_P36	X	153178731	153178731	Missense_Mutation	SNP	C	T	C	ARHGAP4	c.G1352A	p.R451Q
XH_P36	X	153692348	153692348	Missense_Mutation	SNP	G	T	G	PLXNA3	c.G1602T	p.E534D
XH_P36	X	153714260	153714260	Silent	SNP	G	A	G	UBL4A	c.C213T	p.V71V
XH_P36	X	153944567	153944567	Missense_Mutation	SNP	C	T	C	GAB3	c.G110A	p.R37H
XH_P36	X	154754286	154754286	Frame_Shift_Del	DEL	T	-	T	TMLHE	c.189delA	p.K63fs
XH_P36	1	115258747	115258747	Missense_Mutation	SNV	C	T	T	NRAS	c.35G>A	P.G12D
XH_P36	1	1268888	1268888	Missense_Mutation	SNV	G	A	A	TAS1R3	c.1603G>A	P.D535N
XH_P36	1	13635049	13635049	Missense_Mutation	SNV	C	T	T	PRAMEF33P	c.1195C>T	P.R399C
XH_P36	1	144922225	144922225	Nonsense_Mutation	SNV	G	A	A	PDE4DIP	c.1141C>T	P.R381*
XH_P36	1	1461921	1461921	Splice_Site	SNV	C	T	T	ATAD3A	c.1337+10C>T	
XH_P36	1	148341948	148341948	Missense_Mutation	SNV	T	C	C	NBPF9	c.625A>G	P.I209V
XH_P36	1	149895835	149895835	Missense_Mutation	SNV	A	T	T	SF3B4	c.985T>A	P.S329T
XH_P36	1	151804203	151804203	Missense_Mutation	SNV	C	T	T	RORC	c.38G>A	P.R13Q
XH_P36	1	152275289	152275289	Missense_Mutation	SNV	G	A	A	FLG	c.12073C>T	P.P4025S
XH_P36	1	152770502	152770502	Missense_Mutation	SNV	C	T	T	LCE1D	c.232C>T	P.R78C
XH_P36	1	154246391	154246391	Frame_Shift_Del	INDEL	C	-	-	HAX1	c.319delC	P.Q107fs
XH_P36	1	155148566	155148566	Frame_Shift_Ins	INDEL	-	C	C	TRIM46	c.464dupC	P.P156fs
XH_P36	1	155580268	155580268	Frame_Shift_Ins	INDEL	-	C	C	MSTO1	c.141dupC	P.D48fs
XH_P36	1	155722346	155722346	Nonsense_Mutation	SNV	G	A	A	GON4L	c.5878C>T	P.R1960*
XH_P36	1	158325766	158325766	Missense_Mutation	SNV	G	A	A	CD1E	c.775G>A	P.V259I
XH_P36	1	158735706	158735706	Missense_Mutation	SNV	A	G	G	OR6N1	c.767T>C	P.L256P
XH_P36	1	158985772	158985772	Missense_Mutation	SNV	G	C	C	IFI16	c.376G>C	P.A126P
XH_P36	1	159779252	159779252	Missense_Mutation	SNV	A	G	G	FCRL6	c.665A>G	P.D222G
XH_P36	1	161953527	161953527	Missense_Mutation	SNV	G	A	A	OLFML2B	c.2194C>T	P.R732C
XH_P36	1	162731166	162731166	Nonsense_Mutation	SNV	C	T	T	DDR2	c.1021C>T	P.R341*
XH_P36	1	162766447	162766447	Silent	SNV	T	G	G	HSD17B7	c.312T>G	P.L104L
XH_P36	1	164769110	164769110	Nonsense_Mutation	SNV	C	T	T	PBX1	c.685C>T	P.R229*
XH_P36	1	166896414	166896414	Missense_Mutation	SNV	C	T	T	ILDR2	c.884G>A	P.R295H
XH_P36	1	171553156	171553156	Missense_Mutation	SNV	G	A	A	PRRC2C	c.7465G>A	P.A2489T
XH_P36	1	173772575	173772575	Silent	SNV	G	A	A	CENPL	c.627C>T	P.C209C
XH_P36	1	174200408	174200408	Missense_Mutation	SNV	C	T	T	RABGAP1L	c.457C>T	P.R153W
XH_P36	1	175355157	175355157	Splice_Site	SNV	C	T	T	TNR	c.778+11G>A	
XH_P36	1	176659361	176659361	Silent	SNV	C	T	T	PAPPA2	c.2226C>T	P.Y742Y
XH_P36	1	179604781	179604781	Splice_Site	INDEL	T	-	-	TDRD5	c.1300-12delT	
XH_P36	1	182550367	182550367	Missense_Mutation	SNV	G	A	A	RNASEL	c.1898C>T	P.T633M
XH_P36	1	19180874	19180874	Missense_Mutation	SNV	C	T	T	TAS1R2	c.1090G>A	P.D364N
XH_P36	1	19183876	19183876	Silent	SNV	G	T	T	TAS1R2	c.432C>A	P.S144S
XH_P36	1	19566360	19566360	Silent	SNV	C	T	T	EMC1	c.906G>A	P.L302L
XH_P36	1	196309709	196309709	Splice_Site	INDEL	A	-	-	KCNT2	c.1554-9delT	
XH_P36	1	197091176	197091176	Splice_Site	INDEL	A	-	-	ASPM	c.3742-3delT	
XH_P36	1	197297540	197297540	Splice_Site	INDEL	A	-	-	CRB1	c.71-12delA	
XH_P36	1	203668744	203668744	Missense_Mutation	SNV	G	A	A	ATP2B4	c.548G>A	P.R183H
XH_P36	1	203821253	203821253	Splice_Site	INDEL	-	T	T	ZC3H11A	c.2175-8dupT	
XH_P36	1	206758543	206758543	Frame_Shift_Del	INDEL	C	-	-	RASSF5	c.1024delC	P.L342fs
XH_P36	1	21267982	21267982	Splice_Site	SNV	A	G	G	EIF4G3	c.1492+2T>C	
XH_P36	1	2238077	2238077	Missense_Mutation	SNV	A	G	G	SKI	c.2060A>G	P.D687G
XH_P36	1	226340081	226340081	Missense_Mutation	SNV	T	C	C	ACBD3	c.1330A>G	P.S444G
XH_P36	1	228399732	228399732	Missense_Mutation	SNV	G	A	A	OBSCN	c.248G>A	P.R83H
XH_P36	1	228494956	228494956	Splice_Site	SNV	G	A	A	OBSCN	c.12194-4G>A	
XH_P36	1	228506757	228506757	Silent	SNV	G	A	A	OBSCN	c.14304G>A	P.S4768S
XH_P36	1	233464042	233464042	Missense_Mutation	SNV	G	T	T	MLK4	c.268G>T	P.G90C
XH_P36	1	235275431	235275431	Splice_Site	INDEL	A	-	-	TOMM20	c.394-8delT	
XH_P36	1	235658047	235658047	Silent	SNV	G	A	A	B3GALNT2	c.327C>T	P.N109N
XH_P36	1	235940392	235940392	Missense_Mutation	SNV	T	A	A	LYST	c.5431A>T	P.T1811S
XH_P36	1	242042491	242042491	Missense_Mutation	SNV	C	T	T	EXO1	c.1952C>T	P.S651L
XH_P36	1	245927412	245927412	Silent	SNV	T	C	C	SMYD3	c.1116A>G	P.Q372Q
XH_P36	1	248028086	248028086	Missense_Mutation	SNV	G	A	A	TRIM58	c.596G>A	P.R199Q
XH_P36	1	248436586	248436586	Silent	SNV	G	A	A	OR2T33	c.531C>T	P.C177C
XH_P36	1	248436712	248436712	Missense_Mutation	SNV	G	T	T	OR2T33	c.405C>A	P.S135R
XH_P36	1	2572878	2572878	Missense_Mutation	SNV	C	T	T	TTC34	c.1630G>A	P.D544N
XH_P36	1	25889626	25889626	Frame_Shift_Ins	INDEL	-	C	C	LDLRAP1	c.603dupC	P.S202fs
XH_P36	1	28116067	28116067	Splice_Site	INDEL	C	-	-	STX12	c.119-3delC	
XH_P36	1	28785730	28785730	Frame_Shift_Del	INDEL	A	-	-	PHACTR4	c.160delA	P.S54fs
XH_P36	1	32208473	32208473	Silent	SNV	C	T	T	ADGRB2	c.1218G>A	P.L406L
XH_P36	1	34666397	34666397	Frame_Shift_Ins	INDEL	-	A	A	C1orf94	c.1040dupA	P.Q349fs
XH_P36	1	35226919	35226919	Missense_Mutation	SNV	C	T	T	GJB4	c.64C>T	P.R22C
XH_P36	1	3541774	3541774	Silent	SNV	C	T	T	TPRG1L	c.138C>T	P.L46L
XH_P36	1	36030900	36030900	Missense_Mutation	SNV	C	T	T	NCDN	c.1826C>T	P.A609V
XH_P36	1	36367904	36367904	Missense_Mutation	SNV	G	A	A	AGO1	c.1363G>A	P.A455T
XH_P36	1	36380979	36380979	Nonsense_Mutation	SNV	C	T	T	AGO1	c.1864C>T	P.R622*
XH_P36	1	38265467	38265467	Missense_Mutation	SNV	G	A	A	MANEAL	c.300G>A	P.M100I
XH_P36	1	39823033	39823033	Splice_Site	INDEL	T	-	-	MACF1	c.5239-6delT	
XH_P36	1	42047087	42047087	Missense_Mutation	SNV	C	T	T	HIVEP3	c.3382G>A	P.V1128I
XH_P36	1	44466534	44466534	Missense_Mutation	SNV	G	A	A	SLC6A9	c.1441C>T	P.R481W
XH_P36	1	47748086	47748086	Silent	SNV	G	A	A	STIL	c.1179C>T	P.H393H
XH_P36	1	48917261	48917261	Silent	SNV	G	A	A	SPATA6	c.201C>T	P.D67D
XH_P36	1	50941243	50941243	Missense_Mutation	SNV	G	A	A	FAF1	c.1762C>T	P.R588W
XH_P36	1	52825774	52825774	Missense_Mutation	SNV	G	C	C	CC2D1B	c.735C>G	P.D245E
XH_P36	1	63876949	63876949	Frame_Shift_Ins	INDEL	-	T	T	ALG6	c.634dupT	P.C212fs
XH_P36	1	67147843	67147843	Missense_Mutation	SNV	A	G	G	SGIP1	c.1106A>G	P.N369S
XH_P36	1	76257129	76257129	Splice_Site	INDEL	T	-	-	RABGGTB	c.469-9delT	
XH_P36	1	78309060	78309060	Missense_Mutation	SNV	G	A	A	MIGA1	c.964G>A	P.A322T
XH_P36	1	78433883	78433883	Silent	SNV	T	C	C	FUBP1	c.216A>G	P.Q72Q
XH_P36	1	86591275	86591275	Silent	SNV	G	A	A	COL24A1	c.744C>T	P.Y248Y
XH_P36	1	874456	874456	Missense_Mutation	SNV	G	A	A	SAMD11	c.467G>A	P.R156H
XH_P36	1	90179646	90179646	Frame_Shift_Ins	INDEL	-	C	C	LRRC8C	c.1524dupC	P.W509fs
XH_P36	1	91967357	91967357	Frame_Shift_Del	INDEL	A	-	-	CDC7	c.92delA	P.N31fs
XH_P36	1	93306137	93306137	Silent	SNV	T	G	G	RPL5	c.735T>G	P.A245A
XH_P36	1	94140186	94140186	Missense_Mutation	SNV	C	T	T	BCAR3	c.301G>A	P.E101K

XH_P36	1	94502756	94502756	Missense_Mutation	SNV	G	A	A	ABCA4	c.3758C>T	P.T1253M
XH_P36	1	99772074	99772074	Silent	SNV	G	A	A	PLPPR4	c.1626G>A	P.T542T
XH_P36	10	102242535	102242535	Frame_Shift_Ins	INDEL	-	G	G	WNT8B	c.1025dupG	P.A343fs
XH_P36	10	102250651	102250651	Splice_Site	INDEL	A	-	-	SEC31B	c.2473-11delT	
XH_P36	10	102749036	102749036	Missense_Mutation	SNV	C	T	T	C10orf2	c.1069C>T	P.R357C
XH_P36	10	114154663	114154663	Splice_Site	INDEL	T	-	-	ACSL5	c.140-4delT	
XH_P36	10	114925317	114925317	Frame_Shift_Del	INDEL	A	-	-	TCF7L2	c.1385delA	P.K462fs
XH_P36	10	115601182	115601182	Missense_Mutation	SNV	T	C	C	DCLRE1A	c.2803A>G	P.M935V
XH_P36	10	120789359	120789359	Frame_Shift_Ins	INDEL	-	C	C	NANOS1	c.53dupC	P.P19fs
XH_P36	10	121196221	121196221	Missense_Mutation	SNV	T	C	C	GRK5	c.797T>C	P.M266T
XH_P36	10	124917228	124917228	Splice_Site	INDEL	T	-	-	BUB3	c.266-9delT	
XH_P36	10	12708722	12708722	Splice_Site	INDEL	T	-	-	CAMK1D	c.225-4delT	
XH_P36	10	128780162	128780162	Splice_Site	INDEL	T	-	-	DOCK1	c.172-11delT	
XH_P36	10	133955443	133955443	Missense_Mutation	SNV	C	T	T	JAKMIP3	c.1493C>T	P.T498M
XH_P36	10	134942117	134942117	Missense_Mutation	SNV	C	T	T	ADGRA1	c.785C>T	P.T262M
XH_P36	10	135012703	135012703	Silent	SNV	G	A	A	KNDC1	c.2691G>A	P.T897T
XH_P36	10	17363356	17363356	Splice_Site	INDEL	A	-	-	ST8SIA6	c.729-11delT	
XH_P36	10	19981225	19981225	Missense_Mutation	SNV	G	T	T	MALRD1	c.6152G>T	P.W2051L
XH_P36	10	26534838	26534838	Splice_Site	INDEL	T	-	-	GAD2	c.841-5delT	
XH_P36	10	46999854	46999854	Missense_Mutation	SNV	C	T	T	GPRIN2	c.974C>T	P.A325V
XH_P36	10	50953853	50953853	Silent	SNV	G	A	A	OGDHL	c.1296C>T	P.V432V
XH_P36	10	64159513	64159513	Frame_Shift_Del	INDEL	A	-	-	ZNF365	c.1196delA	P.K399fs
XH_P36	10	70332889	70332889	Missense_Mutation	SNV	A	G	G	TET1	c.794A>G	P.N265S
XH_P36	10	70723214	70723214	Missense_Mutation	SNV	C	T	T	DDX21	c.571C>T	P.R191C
XH_P36	10	71142432	71142432	Silent	SNV	G	A	A	HK1	c.1455G>A	P.L485L
XH_P36	10	73466731	73466731	Missense_Mutation	SNV	G	A	A	CDH23	c.3031G>A	P.E1011K
XH_P36	10	78729786	78729786	Frame_Shift_Del	INDEL	T	-	-	KCNMA1	c.2144delA	P.K715fs
XH_P36	10	8019267	8019267	Missense_Mutation	SNV	G	A	A	TAF3	c.2296G>A	P.G766S
XH_P36	10	8100708	8100708	Missense_Mutation	SNV	G	A	A	GATA3	c.682G>A	P.E228K
XH_P36	10	81317157	81317157	Silent	SNV	G	T	T	SFTPA2	c.555C>A	P.A185A
XH_P36	10	89272866	89272866	Splice_Site	INDEL	T	-	-	MINPP1	c.836-9delT	
XH_P36	10	92617011	92617011	Missense_Mutation	SNV	C	T	T	HTR7	c.418G>A	P.V140I
XH_P36	10	93601946	93601946	Frame_Shift_Del	INDEL	A	-	-	TNKS2	c.1865delA	P.N622fs
XH_P36	10	93999314	93999314	Missense_Mutation	SNV	C	T	T	CPEB3	c.794G>A	P.G265D
XH_P36	10	94773	94773	Missense_Mutation	SNV	C	T	T	TUBB8	c.137G>A	P.R46H
XH_P36	10	95995659	95995659	Splice_Site	INDEL	T	-	-	PLCE1	c.1291-5delT	
XH_P36	10	98080475	98080475	Missense_Mutation	SNV	T	C	C	DNTT	c.608T>C	P.F203S
XH_P36	11	101323754	101323754	Missense_Mutation	SNV	C	A	A	TRPC6	c.2728G>T	P.A910S
XH_P36	11	108384793	108384793	Frame_Shift_Del	INDEL	A	-	-	EXPH5	c.1213delT	P.W405fs
XH_P36	11	111366044	111366044	Splice_Site	INDEL	A	-	-	BTG4	c.511-5delT	
XH_P36	11	112020784	112020784	Splice_Site	INDEL	A	-	-	IL18	c.214+11delT	
XH_P36	11	113694379	113694379	Frame_Shift_Ins	INDEL	-	CT	CT	USP28	.854_855dupA	P.C286fs
XH_P36	11	123448124	123448124	Missense_Mutation	SNV	C	T	T	GRAMD1B	c.73C>T	P.R25W
XH_P36	11	124845049	124845049	Frame_Shift_Del	INDEL	A	-	-	CCDC15	c.581delA	P.K194fs
XH_P36	11	125221249	125221249	Silent	SNV	G	A	A	PKNOX2	c.48G>A	P.T16T
XH_P36	11	130066453	130066453	Splice_Site	INDEL	C	-	-	ST14	c.1224-6delC	
XH_P36	11	14280928	14280928	Missense_Mutation	SNV	C	T	T	SPON1	c.1592C>T	P.P531L
XH_P36	11	14793470	14793470	Splice_Site	INDEL	T	-	-	PDE3B	c.979-5delT	
XH_P36	11	17758073	17758073	Missense_Mutation	SNV	G	A	A	KCNC1	c.524G>A	P.R175H
XH_P36	11	20066788	20066788	Silent	SNV	C	T	T	NAV2	c.3282C>T	P.L1094L
XH_P36	11	2432968	2432968	Missense_Mutation	SNV	C	T	T	TRPM5	c.2504G>A	P.R835H
XH_P36	11	27405964	27405964	Splice_Site	INDEL	A	-	-	LGR4	c.618-10delT	
XH_P36	11	30255300	30255300	Nonsense_Mutation	SNV	C	T	T	FSHB	c.343C>T	P.R115*
XH_P36	11	3114178	3114178	Missense_Mutation	SNV	C	T	T	OSBPL5	c.1847G>A	P.R616Q
XH_P36	11	33596351	33596351	Missense_Mutation	SNV	T	G	G	KIAA1549L	c.3443T>G	P.I1148S
XH_P36	11	34489930	34489930	Silent	SNV	C	T	T	CAT	c.1422C>T	P.I474I
XH_P36	11	3848989	3848989	Missense_Mutation	SNV	C	T	T	RHOG	c.380G>A	P.R127Q
XH_P36	11	43425610	43425613	Frame_Shift_Del	INDEL	GAGT	-	-	TTC17	397_1400delGT	P.S466fs
XH_P36	11	45671512	45671512	Missense_Mutation	SNV	C	G	G	CHST1	c.962G>C	P.G321A
XH_P36	11	4661623	4661623	Silent	SNV	C	A	A	OR51D1	c.603C>A	P.S201S
XH_P36	11	47611863	47611863	Missense_Mutation	SNV	C	T	T	C1QTNF4	c.500G>A	P.R167H
XH_P36	11	48347449	48347449	Frame_Shift_Del	INDEL	A	-	-	OR4C3	c.962delA	P.N321fs
XH_P36	11	5275557	5275557	Missense_Mutation	SNV	A	G	G	HBG2	c.280T>C	P.C94R
XH_P36	11	5364256	5364256	Missense_Mutation	SNV	A	G	G	OR51B5	c.499T>C	P.C167R
XH_P36	11	60506456	60506456	Missense_Mutation	SNV	C	A	A	MS4A18	c.610C>A	P.L204I
XH_P36	11	60666410	60666410	Splice_Site	INDEL	A	-	-	PRPF19	c.985-7delT	
XH_P36	11	63277289	63277289	Missense_Mutation	SNV	C	T	T	LGALS12	c.481C>T	P.R161W
XH_P36	11	63997883	63997885	Splice_Site	INDEL	TTT	-	-	DNAJC4	23-11_-423-9delTTT	
XH_P36	11	65396411	65396411	Silent	SNV	C	T	T	PCNX3	c.3933C>T	P.R1311R
XH_P36	11	65733371	65733371	Missense_Mutation	SNV	C	T	T	SART1	c.752C>T	P.A251V
XH_P36	11	65780794	65780794	Missense_Mutation	SNV	C	T	T	CST6	c.373C>T	P.R125C
XH_P36	11	67072394	67072394	Silent	SNV	C	A	A	SSH3	c.255C>A	P.S85S
XH_P36	11	67187007	67187007	Missense_Mutation	SNV	G	A	A	CARNS1	c.902G>A	P.R301H
XH_P36	11	67258296	67258296	Missense_Mutation	SNV	C	T	T	AIP	c.817C>T	P.R273C
XH_P36	11	67261809	67261809	Missense_Mutation	SNV	G	A	A	PITPNM1	c.2762C>T	P.A921V
XH_P36	11	67263644	67263644	Splice_Site	SNV	C	A	A	PITPNM1	c.2314+5G>T	
XH_P36	11	67264838	67264838	Silent	SNV	G	A	A	PITPNM1	c.2010C>T	P.P670P
XH_P36	11	67800693	67800693	Silent	SNV	A	G	G	NDUFS8	c.315A>G	P.P105P
XH_P36	11	69962552	69962552	Splice_Site	INDEL	T	-	-	ANO1	c.856-6delT	
XH_P36	11	7059779	7059779	Splice_Site	INDEL	T	-	-	NLRP14	c.-21-11delT	
XH_P36	11	71195477	71195477	Missense_Mutation	SNV	C	T	T	NADSYN1	c.1439C>T	P.A480V
XH_P36	11	71850756	71850756	Missense_Mutation	SNV	G	A	A	FOLR3	c.616G>A	P.G206S
XH_P36	11	75623053	75623053	Missense_Mutation	SNV	G	A	A	UVRAG	c.563G>A	P.R188H
XH_P36	11	78282493	78282493	Splice_Site	SNV	A	C	C	NARS2	c.-540-4T>G	
XH_P36	11	82536174	82536174	Splice_Site	INDEL	A	-	-	PRCP	c.960-10delT	
XH_P36	11	82966931	82966931	Frame_Shift_Del	INDEL	A	-	-	ANKRD42	c.1517delA	P.K506fs
XH_P36	11	85420388	85420388	Frame_Shift_Del	INDEL	T	-	-	SYTL2	c.1986delA	P.K662fs
XH_P36	11	865495	865495	Splice_Site	INDEL	C	-	-	TSPAN4	c.331-11delC	
XH_P36	11	88068257	88068259	Splice_Site	INDEL	AAA	-	-	CTSC	73-9_173-7delTTT	
XH_P36	11	88583231	88583231	Missense_Mutation	SNV	C	T	T	GRM5	c.754G>A	P.A252T
XH_P36	11	92532469	92532469	Missense_Mutation	SNV	C	T	T	FAT3	c.6290C>T	P.A2097V
XH_P36	11	93439896	93439896	Splice_Site	INDEL	T	-	-	CEP295	c.5597-10delT	
XH_P36	11	94599175	94599175	Missense_Mutation	SNV	G	A	A	AMOTL1	c.2180G>A	P.R727H
XH_P36	11	9861142	9861142	Missense_Mutation	SNV	G	A	A	SBF2	c.3358C>T	P.R1120C
XH_P36	12	100489623	100489623	Splice_Site	INDEL	A	-	-	UHRF1BP1L	c.670-8delT	
XH_P36	12	101473003	101473003	Nonsense_Mutation	SNV	C	T	T	ANO4	c.1345C>T	P.R449*
XH_P36	12	101689237	101689237	Splice_Site	INDEL	T	-	-	UTP20	c.1252-12delT	
XH_P36	12	104697824	104697824	Missense_Mutation	SNV	C	T	T	EID3	c.112C>T	P.R38C

XH_P36	12	109536183	109536183	Missense_Mutation	SNV	C	T	T	UNG	c.52C>T	P.P18S
XH_P36	12	113445730	113445730	Missense_Mutation	SNV	G	A	A	OAS2	c.1877G>A	P.S626N
XH_P36	12	117217097	117217097	Missense_Mutation	SNV	G	A	A	RNFT2	c.826G>A	P.A276T
XH_P36	12	124832387	124832387	Silent	SNV	G	A	A	NCOR2	c.4026C>T	P.R1342R
XH_P36	12	130647740	130647740	Missense_Mutation	SNV	T	G	G	FZD10	c.253T>G	P.F85V
XH_P36	12	130935836	130935836	Silent	SNV	C	T	T	RIMBP2	c.357G>A	P.R119R
XH_P36	12	130941035	130941035	Splice_Site	SNV	G	A	A	RIMBP2	c.307+6C>T	
XH_P36	12	131620642	131620642	Silent	SNV	C	T	T	ADGRD1	c.2328C>T	P.G776G
XH_P36	12	133463877	133463877	Missense_Mutation	SNV	G	T	T	CHFR	c.38C>A	P.P13Q
XH_P36	12	18876416	18876416	Missense_Mutation	SNV	T	A	A	PLCZ1	c.196A>T	P.I66F
XH_P36	12	21727271	21727271	Splice_Site	INDEL	A	-	-	GYS2	c.496-11delT	
XH_P36	12	2907038	2907041	Splice_Site	INDEL	GTGA	-	-	FKBP4	3+4_393+7delAGTG	
XH_P36	12	29614779	29614779	Splice_Site	SNV	C	T	T	OVCH1	c.2280+8G>A	
XH_P36	12	40838232	40838232	Silent	SNV	C	A	A	MUC19	c.4494C>A	P.A1498A
XH_P36	12	4766968	4766968	Missense_Mutation	SNV	G	A	A	NDUFA9	c.373G>A	P.V125M
XH_P36	12	48141379	48141379	Nonsense_Mutation	SNV	G	A	A	RAPGEF3	c.1504C>T	P.R502*
XH_P36	12	49992526	49992526	Splice_Site	SNV	C	T	T	FAM186B	c.2364+12G>A	
XH_P36	12	50026780	50026781	Splice_Site	INDEL	CC	-	-	PRPF40B	349-8_349-7delCC	
XH_P36	12	51399180	51399180	Missense_Mutation	SNV	C	T	T	SLC11A2	c.223G>A	P.G75R
XH_P36	12	52684997	52684997	Missense_Mutation	SNV	C	T	T	KRT81	c.253G>A	P.E85K
XH_P36	12	53038849	53038849	Missense_Mutation	SNV	C	T	T	KRT2	c.1874G>A	P.S625N
XH_P36	12	56398615	56398615	Missense_Mutation	SNV	G	A	A	SUOX	c.1442G>A	P.R481H
XH_P36	12	56494905	56494905	Missense_Mutation	SNV	C	T	T	ERBB3	c.3262C>T	P.P1088S
XH_P36	12	56559113	56559113	Frame_Shift_Del	INDEL	G	-	-	SMARCC2	c.3221delC	P.P1074fs
XH_P36	12	56661723	56661723	Missense_Mutation	SNV	G	T	T	COQ10A	c.184G>T	P.G62W
XH_P36	12	57389231	57389231	Missense_Mutation	SNV	C	T	T	GPR182	c.238C>T	P.R80C
XH_P36	12	57911097	57911097	Silent	SNV	T	C	C	DDIT3	c.162A>G	P.S54S
XH_P36	12	64055225	64055225	Missense_Mutation	SNV	C	A	A	DPY19L2	c.487G>T	P.A163S
XH_P36	12	69085736	69085736	Splice_Site	INDEL	T	-	-	NUP107	c.217-4delT	
XH_P36	12	71537999	71537999	Splice_Site	INDEL	-	A	A	TSPAN8	c.61-7dupT	
XH_P36	12	72009100	72009100	Splice_Site	INDEL	A	-	-	ZFC3H1	c.5145-4delT	
XH_P36	12	72024509	72024509	Splice_Site	INDEL	A	-	-	ZFC3H1	c.3603-8delT	
XH_P36	12	75687029	75687029	Missense_Mutation	SNV	T	A	A	CAPS2	c.974A>T	P.K325I
XH_P36	12	75804272	75804272	Missense_Mutation	SNV	C	T	T	GLIPR1L2	c.293C>T	P.T98M
XH_P36	12	78593137	78593137	Missense_Mutation	SNV	A	G	G	NAV3	c.6541A>G	P.T2181A
XH_P36	12	89745700	89745700	Silent	SNV	G	A	A	DUSP6	c.117C>T	P.C39C
XH_P36	12	9221371	9221371	Missense_Mutation	SNV	G	A	A	A2M	c.4331C>T	P.P1444L
XH_P36	12	94543030	94543030	Frame_Shift_Del	INDEL	C	-	-	PLXNC1	c.288delC	P.A97fs
XH_P36	12	97186181	97186181	Silent	SNV	G	A	A	CFAP54	c.8754G>A	P.T2918T
XH_P36	13	103390256	103390256	Missense_Mutation	SNV	T	C	C	CCDC168	c.12791A>G	P.E4264G
XH_P36	13	110434690	110434690	Silent	SNV	C	T	T	IRS2	c.3711G>A	P.S1237S
XH_P36	13	111110449	111110449	Missense_Mutation	SNV	C	T	T	COL4A2-AS2	c.257G>A	P.R86Q
XH_P36	13	113439523	113439523	Missense_Mutation	SNV	G	T	T	ATP11A	c.114G>T	P.E38D
XH_P36	13	114538617	114538617	Splice_Site	INDEL	A	-	-	GAS6	c.590-9delT	
XH_P36	13	20656139	20656141	Splice_Site	INDEL	TTT	-	-	ZMYM2	454-7_3454-5delTTT	
XH_P36	13	26542799	26542799	Missense_Mutation	SNV	G	A	A	ATP8A2	c.3164G>A	P.R1055Q
XH_P36	13	26789433	26789433	Nonsense_Mutation	SNV	G	A	A	RNF6	c.586C>T	P.R196*
XH_P36	13	27216440	27216440	Silent	SNV	G	A	A	WASF3	c.33G>A	P.R11R
XH_P36	13	31036852	31036852	Splice_Site	INDEL	A	-	-	HMGB1	c.297-3delT	
XH_P36	13	36905543	36905543	Missense_Mutation	SNV	C	T	T	SPG20	c.1001G>A	P.R334Q
XH_P36	13	39265390	39265390	Silent	SNV	G	A	A	FREM2	c.3909G>A	P.T1303T
XH_P36	13	41897181	41897181	Splice_Site	INDEL	T	-	-	NAA16	c.403-3delT	
XH_P36	13	46632361	46632361	Missense_Mutation	SNV	A	G	G	CPB2	c.841T>C	P.F281L
XH_P36	13	50087175	50087175	Splice_Site	INDEL	T	-	-	PHF11	c.217-12delT	
XH_P36	13	53007887	53007887	Splice_Site	INDEL	A	-	-	VPS36	c.415-4delT	
XH_P36	13	73547716	73547716	Splice_Site	INDEL	T	-	-	PIBF1	c.1965-4delT	
XH_P36	13	77529556	77529556	Missense_Mutation	SNV	C	T	T	ACOD1	c.355C>T	P.P119S
XH_P36	13	79908574	79908574	Splice_Site	INDEL	-	A	A	RBM26	c.2740-10dupT	
XH_P36	13	84454271	84454271	Missense_Mutation	SNV	C	T	T	SLITRK1	c.1372G>A	P.A458T
XH_P36	14	100126651	100126651	Silent	SNV	G	T	T	HHIPL1	c.1410G>T	P.T470T
XH_P36	14	100380645	100380645	Splice_Site	SNV	C	T	T	EML1	c.1677+4C>T	
XH_P36	14	103336573	103336573	Missense_Mutation	SNV	C	T	T	TRAF3	c.35C>T	P.A12V
XH_P36	14	103996592	103996592	Missense_Mutation	SNV	G	A	A	TRMT61A	c.277G>A	P.A93T
XH_P36	14	105353222	105353222	Frame_Shift_Ins	INDEL	-	C	C	CEP170B	c.2653dupC	P.H885fs
XH_P36	14	105408144	105408144	Missense_Mutation	SNV	C	A	A	AHNAK2	c.13644G>T	P.K4548N
XH_P36	14	105944635	105944635	Missense_Mutation	SNV	C	T	T	CRIP2	c.298C>T	P.H100Y
XH_P36	14	20002302	20002302	Splice_Site	INDEL	A	-	-	POTEM	c.1127-7delT	
XH_P36	14	21569511	21569511	Silent	SNV	T	C	C	TMEM253	c.9T>C	P.D3D
XH_P36	14	21957314	21957314	Splice_Site	INDEL	-	T	T	TOX4	c.511-12dupT	
XH_P36	14	31642477	31642477	Silent	SNV	G	A	A	HECTD1	c.1041C>T	P.G347G
XH_P36	14	35497275	35497275	Splice_Site	INDEL	T	-	-	SRP54	c.1181-9delT	
XH_P36	14	39649809	39649809	Missense_Mutation	SNV	G	A	A	PNN	c.896G>A	P.R299H
XH_P36	14	39901278	39901278	Missense_Mutation	SNV	A	C	C	FBXO33	c.89T>G	P.L30R
XH_P36	14	50626388	50626388	Missense_Mutation	SNV	G	A	A	SOS2	c.1613C>T	P.A538V
XH_P36	14	51210171	51210171	Missense_Mutation	SNV	G	A	A	NIN	c.3125C>T	P.A1042V
XH_P36	14	53101641	53101641	Splice_Site	INDEL	T	-	-	GPR137C	c.1113-8delT	
XH_P36	14	55203729	55203729	Splice_Site	INDEL	T	-	-	SAMD4A	c.716-5delT	
XH_P36	14	57103323	57103323	Splice_Site	SNV	T	C	C	TMEM260	c.1869+7T>C	
XH_P36	14	57269043	57269043	Missense_Mutation	SNV	G	A	A	OTX2	c.280C>T	P.R94C
XH_P36	14	60581403	60581403	Splice_Site	INDEL	T	-	-	PCNX4	c.690-7delT	
XH_P36	14	61186779	61186779	Missense_Mutation	SNV	C	A	A	SIX4	c.1248G>T	P.Q416H
XH_P36	14	61278692	61278692	Splice_Site	INDEL	-	T	T	MNAT1	c.421-8dupT	
XH_P36	14	68044071	68044071	Silent	SNV	C	T	T	PLEKHH1	c.2514C>T	P.Y838Y
XH_P36	14	68045918	68045918	Missense_Mutation	SNV	C	T	T	PLEKHH1	c.2917C>T	P.R973W
XH_P36	14	72056027	72056027	Missense_Mutation	SNV	T	C	C	SIPA1L1	c.1438T>C	P.Y480H
XH_P36	14	74203800	74203800	Silent	SNV	G	A	A	ELMSAN1	c.1650C>T	P.D550D
XH_P36	14	74534275	74534275	Splice_Site	INDEL	A	-	-	ALDH6A1	c.814-3delT	
XH_P36	14	75359617	75359617	Frame_Shift_Del	INDEL	C	-	-	DLST	c.527delC	P.P176fs
XH_P36	14	75515373	75515376	Frame_Shift_Del	INDEL	TCAA	-	-	MLH3	983_986delTTG	P.I328fs
XH_P36	14	76429801	76429801	Nonsense_Mutation	SNV	C	A	A	TGFB3	c.784G>T	P.G262*
XH_P36	14	94107567	94107567	Silent	SNV	C	T	T	UNC79	c.5463C>T	P.D1821D
XH_P36	14	94417346	94417346	Splice_Site	SNV	G	A	A	ASB2	c.879C>T	P.Y293Y
XH_P36	14	95675732	95675732	Splice_Site	SNV	G	A	A	CLMN	c.885C>T	P.A295A
XH_P36	14	97022519	97022519	Silent	SNV	G	A	A	PAPOLA	c.1770G>A	P.S590S
XH_P36	15	100591929	100591929	Missense_Mutation	SNV	A	G	G	ADAMTS17	c.2303T>C	P.L768S
XH_P36	15	101865128	101865128	Silent	SNV	G	A	A	PCSK6	c.2079C>T	P.R693R
XH_P36	15	102242559	102242559	Silent	SNV	C	T	T	TARSL2	c.1104G>A	P.P368P
XH_P36	15	102346763	102346763	Missense_Mutation	SNV	G	A	A	OR4F6	c.841G>A	P.V281I

XH_P36	15	22742754	22742754	Missense_Mutation	SNV	G	A	A	GOLGA6L22	c.1103G>A	P.R368Q
XH_P36	15	23891598	23891598	Missense_Mutation	SNV	C	T	T	MAGEL2	c.1292G>A	P.R431Q
XH_P36	15	27160004	27160004	Silent	SNV	G	A	A	GABRA5	c.552G>A	P.A184A
XH_P36	15	27188381	27188381	Silent	SNV	C	T	T	GABRA5	c.897C>T	P.T299T
XH_P36	15	28263537	28263537	Splice_Site	SNV	C	T	T	OCA2	c.807+6G>A	
XH_P36	15	28437288	28437288	Missense_Mutation	SNV	G	A	A	HERC2	c.8270C>T	P.A2757V
XH_P36	15	30659672	30659672	Silent	SNV	G	A	A	CHRFAM7A	c.669C>T	P.I223I
XH_P36	15	40510642	40510642	Splice_Site	INDEL	T	-	-	BUB1B	c.2851-11delT	
XH_P36	15	42159792	42159792	Nonsense_Mutation	SNV	G	A	A	SPTBN5	c.6256C>T	P.Q2086*
XH_P36	15	44964203	44964203	Splice_Site	SNV	C	T	T	PATL2	c.657+10G>A	
XH_P36	15	45409422	45409422	Missense_Mutation	SNV	G	A	A	DUOXA2	c.688G>A	P.V230M
XH_P36	15	50773790	50773790	Missense_Mutation	SNV	G	A	A	USP8	c.1331G>A	P.R444H
XH_P36	15	50925185	50925185	Missense_Mutation	SNV	G	A	A	TRPM7	c.1012C>T	P.L338F
XH_P36	15	55621922	55621922	Frame_Shift_Del	INDEL	T	-	-	PIGB	c.529delT	P.C177fs
XH_P36	15	56720550	56720550	Splice_Site	INDEL	T	-	-	TEX9	c.874-6delT	
XH_P36	15	57545439	57545439	Splice_Site	INDEL	T	-	-	TCF12	c.751-12delT	
XH_P36	15	60747651	60747651	Splice_Site	INDEL	A	-	-	ICE2	c.256-10delT	
XH_P36	15	69331298	69331298	Silent	SNV	C	T	T	NOX5	c.1389C>T	P.P463P
XH_P36	15	69747754	69747755	Splice_Site	INDEL	TT	-	-	RPLP1	266-6_266-5delTT	
XH_P36	15	73660540	73660540	Nonsense_Mutation	SNV	C	T	T	HCN4	c.72G>A	P.W24*
XH_P36	15	75498563	75498563	Silent	SNV	C	T	T	C15orf39	c.174C>T	P.A58A
XH_P36	15	75969242	75969242	Missense_Mutation	SNV	T	C	C	CSPG4	c.5618A>G	P.Q1873R
XH_P36	15	79058543	79058543	Missense_Mutation	SNV	C	T	T	ADAMTS7	c.3710G>A	P.S1237N
XH_P36	15	85164712	85164712	Missense_Mutation	SNV	G	A	A	ZSCAN2	c.1286G>A	P.R429H
XH_P36	15	89719201	89719201	Missense_Mutation	SNV	G	A	A	ABHD2	c.697G>A	P.V233M
XH_P36	16	1412305	1412305	Frame_Shift_Del	INDEL	C	-	-	GNPTG	c.514delC	P.H172fs
XH_P36	16	14970537	14970537	Missense_Mutation	SNV	A	C	C	NOMO1	c.2533A>C	P.S845R
XH_P36	16	1709929	1709929	Missense_Mutation	SNV	G	A	A	CRAMP1	c.2278G>A	P.A760T
XH_P36	16	19063046	19063046	Silent	SNV	G	A	A	TMC7	c.1449G>A	P.P483P
XH_P36	16	19126240	19126240	Missense_Mutation	SNV	C	T	T	ITPRIPL2	c.457C>T	P.R153C
XH_P36	16	19883839	19883839	Missense_Mutation	SNV	G	A	A	GPRC5B	c.722C>T	P.T241M
XH_P36	16	20948007	20948007	Silent	SNV	G	A	A	DNAH3	c.11823C>T	P.Y3941Y
XH_P36	16	2279560	2279560	Splice_Site	SNV	C	A	A	E4F1	c.310-11C>A	
XH_P36	16	2762695	2762695	Missense_Mutation	SNV	C	T	T	PRSS27	c.493G>A	P.A165T
XH_P36	16	28913639	28913639	Frame_Shift_Ins	INDEL	-	C	C	ATP2A1	c.2089dupC	P.R697fs
XH_P36	16	2903887	2903887	Missense_Mutation	SNV	C	A	A	PRSS22	c.696G>T	P.E232D
XH_P36	16	2983964	2983964	Silent	SNV	G	A	A	FLYWCH1	c.1497G>A	P.A499A
XH_P36	16	29891248	29891248	Frame_Shift_Ins	INDEL	-	G	G	SEZ6L2	c.1299dupC	P.N434fs
XH_P36	16	30018105	30018105	Splice_Site	SNV	C	T	T	DOC2A	c.878+1G>A	
XH_P36	16	30680561	30680561	Silent	SNV	C	A	A	FBRS	c.2538C>A	P.A846A
XH_P36	16	31121549	31121549	Silent	SNV	C	A	A	BCKDK	c.447C>A	P.A149A
XH_P36	16	31142287	31142287	Nonsense_Mutation	SNV	C	T	T	KAT8	c.1378C>T	P.Q460*
XH_P36	16	314836	314836	Missense_Mutation	SNV	G	A	A	FAM234A	c.1474G>A	P.A492T
XH_P36	16	46726279	46726279	Splice_Site	INDEL	T	-	-	ORC6	c.196-11delT	
XH_P36	16	4855316	4855316	Splice_Site	INDEL	A	-	-	GLYR1	c.1570-5delT	
XH_P36	16	49670972	49670972	Silent	SNV	G	A	A	ZNF423	c.1911C>T	P.C637C
XH_P36	16	53265642	53265642	Frame_Shift_Del	INDEL	A	-	-	CHD9	c.2461delA	P.I821fs
XH_P36	16	53706815	53706815	Missense_Mutation	SNV	A	T	T	RPGRIP1L	c.996T>A	P.H332Q
XH_P36	16	67006630	67006630	Missense_Mutation	SNV	A	T	T	CES3	c.418A>T	P.T140S
XH_P36	16	67687145	67687145	Frame_Shift_Del	INDEL	G	-	-	CARMIL2	c.3113delG	P.G1038fs
XH_P36	16	67964372	67964372	Silent	SNV	G	A	A	CTRL	c.438C>T	P.N146N
XH_P36	16	68021792	68021792	Missense_Mutation	SNV	C	T	T	DPEP2	c.695G>A	P.R232H
XH_P36	16	70328814	70328814	Frame_Shift_Del	INDEL	G	-	-	DDX19B	c.68delG	P.G23fs
XH_P36	16	72142164	72142164	Silent	SNV	C	T	T	DHX38	c.3003C>T	P.F1001F
XH_P36	16	72153907	72153907	Silent	SNV	G	A	A	PMFBP1	c.2475C>T	P.A825A
XH_P36	16	74660379	74660379	Silent	SNV	G	A	A	RFWD3	c.2043C>T	P.I681I
XH_P36	16	75258768	75258768	Missense_Mutation	SNV	C	T	T	CTRB1	c.662C>T	P.P221L
XH_P36	16	75564074	75564074	Missense_Mutation	SNV	G	A	A	CHST5	c.209C>T	P.S70L
XH_P36	16	77325354	77325354	Missense_Mutation	SNV	C	T	T	ADAMTS18	c.2695G>A	P.G899S
XH_P36	16	81058306	81058306	Splice_Site	INDEL	T	-	-	CENPN	c.634-7delT	
XH_P36	16	81095653	81095653	Missense_Mutation	SNV	C	T	T	C16orf46	c.301G>A	P.D101N
XH_P36	16	81173227	81173227	Silent	SNV	C	T	T	PKD1L2	c.3453G>A	P.A1151A
XH_P36	16	81301689	81301689	Missense_Mutation	SNV	C	T	T	BCO1	c.796C>T	P.R266W
XH_P36	16	83214458	83214458	Splice_Site	INDEL	T	-	-	CDH13	c.484-8delT	
XH_P36	16	842546	842546	Frame_Shift_Del	INDEL	G	-	-	CHTF18	c.1440delG	P.L481fs
XH_P36	16	84494362	84494362	Silent	SNV	C	T	T	ATP2C2	c.2523C>T	P.S841S
XH_P36	16	89614422	89614422	Missense_Mutation	SNV	G	A	A	SPG7	c.1564G>A	P.A522T
XH_P36	16	89778933	89778933	Splice_Site	INDEL	G	-	-	VPS9D1	c.544-3delC	
XH_P36	16	90001937	90001937	Missense_Mutation	SNV	G	A	A	TUBB3	c.862G>A	P.G288S
XH_P36	17	16676799	16676799	Splice_Site	INDEL	T	-	-	CCDC144A	c.4252-4delT	
XH_P36	17	17898307	17898307	Splice_Site	SNV	C	A	A	DRC3	c.712-6C>A	
XH_P36	17	20163582	20163582	Missense_Mutation	SNV	G	A	A	SPECC1	c.2915G>A	P.C972Y
XH_P36	17	20859933	20859933	Splice_Site	INDEL	T	-	-	LOC339260	n.641-7delT	
XH_P36	17	25910015	25910015	Frame_Shift_Ins	INDEL	-	C	C	KSR1	c.461dupC	P.P155fs
XH_P36	17	26708249	26708249	Silent	SNV	C	T	T	SARM1	c.492C>T	P.G164G
XH_P36	17	26712159	26712159	Missense_Mutation	SNV	C	T	T	SARM1	c.1489C>T	P.R497C
XH_P36	17	27002460	27002460	Frame_Shift_Del	INDEL	A	-	-	SUPT6H	c.585delA	P.K195fs
XH_P36	17	27044426	27044426	Missense_Mutation	SNV	T	A	A	NARR	c.301A>T	P.T101S
XH_P36	17	27239943	27239943	Missense_Mutation	SNV	C	T	T	PHF12	c.1646G>A	P.G549D
XH_P36	17	27903116	27903116	Missense_Mutation	SNV	C	T	T	GIT1	c.1672G>A	P.V558I
XH_P36	17	28706682	28706682	Frame_Shift_Ins	INDEL	-	C	C	CPD	c.690dupC	P.A231fs
XH_P36	17	29632268	29632268	Silent	SNV	G	A	A	EVI2B	c.360C>T	P.A120A
XH_P36	17	34062267	34062267	Missense_Mutation	SNV	C	T	T	RASL10B	c.64C>T	P.R22C
XH_P36	17	34893857	34893857	Missense_Mutation	SNV	C	T	T	PIGW	c.907C>T	P.R303C
XH_P36	17	3563601	3563601	Missense_Mutation	SNV	G	A	A	CTNS	c.1042G>A	P.V348I
XH_P36	17	39888649	39888649	Splice_Site	INDEL	G	-	-	HAP1	c.574-3delC	
XH_P36	17	40042503	40042503	Missense_Mutation	SNV	T	G	G	ACLY	c.1934A>C	P.N645T
XH_P36	17	40940193	40940193	Frame_Shift_Ins	INDEL	-	C	C	WNK4	c.906dupC	P.G303fs
XH_P36	17	41154724	41154724	Missense_Mutation	SNV	G	A	A	RPL27	c.286G>A	P.V96I
XH_P36	17	48470257	48470257	Missense_Mutation	SNV	G	A	A	LRRC59	c.167C>T	P.S56L
XH_P36	17	48647125	48647125	Missense_Mutation	SNV	C	T	T	CACNA1G	c.547C>T	P.R183C
XH_P36	17	53392628	53392628	Silent	SNV	T	C	C	HLF	c.492T>C	P.I164I
XH_P36	17	54520274	54520274	Missense_Mutation	SNV	C	T	T	ANKFN1	c.1088C>T	P.P363L
XH_P36	17	56400068	56400068	Missense_Mutation	SNV	G	A	A	TSPOAP1	c.1264C>T	P.R422C
XH_P36	17	56438293	56438293	Nonsense_Mutation	SNV	G	A	A	RNF43	c.700C>T	P.Q234*
XH_P36	17	56676693	56676693	Silent	SNV	C	T	T	TEX14	c.2031G>A	P.A677A
XH_P36	17	57746175	57746175	Missense_Mutation	SNV	C	A	A	CLTC	c.2178C>A	P.S726R
XH_P36	17	59465981	59465981	Frame_Shift_Del	INDEL	A	-	-	BCAS3	c.2670delA	P.G891fs

XH_P36	17	60042378	60042378	Missense_Mutation	SNV	A	C	C	MED13	c.4833T>G	P.D1611E
XH_P36	17	61497894	61497894	Frame_Shift_Del	INDEL	C	-	-	TANC2	c.4556delC	P.P1519fs
XH_P36	17	7127698	7127698	Missense_Mutation	SNV	C	T	T	ACADVL	c.1591C>T	P.R531W
XH_P36	17	73555417	73555417	Silent	SNV	C	T	T	LLGL2	c.456C>T	P.N152N
XH_P36	17	73646720	73646720	Nonstop_Mutation	SNV	T	C	C	RECQL5	c.1308A>G	P.Ter436Wext*?
XH_P36	17	73914025	73914025	Silent	SNV	G	A	A	FBF1	c.2373C>T	P.D791D
XH_P36	17	74015081	74015081	Missense_Mutation	SNV	G	A	A	EVPL	c.1198C>T	P.R400W
XH_P36	17	74072983	74072983	Missense_Mutation	SNV	G	A	A	GALR2	c.635G>A	P.R212H
XH_P36	17	77758181	77758181	Silent	SNV	C	T	T	CBX2	c.939C>T	P.S313S
XH_P36	17	79210603	79210603	Splice_Site	SNV	T	C	C	TEPSIN	c.214-2A>G	
XH_P36	17	79249771	79249771	Missense_Mutation	SNV	G	T	T	SLC38A10	c.910C>A	P.Q304K
XH_P36	17	79769608	79769608	Missense_Mutation	SNV	C	A	A	GCGR	c.739C>A	P.L247M
XH_P36	17	79792754	79792754	Missense_Mutation	SNV	C	T	T	PPP1R27	c.76G>A	P.V26M
XH_P36	17	80129734	80129734	Splice_Site	INDEL	-	A	A	CCDC57	c.1730-6dupT	
XH_P36	18	13057634	13057634	Missense_Mutation	SNV	G	A	A	CEP192	c.4159G>A	P.G1387R
XH_P36	18	13746253	13746253	Frame_Shift_Del	INDEL	A	-	-	RNMT	c.1180delA	P.T394fs
XH_P36	18	14523456	14523456	Splice_Site	SNV	G	A	A	POTEC	c.1242+6C>T	
XH_P36	18	14538206	14538206	Silent	SNV	T	C	C	POTEC	c.564A>G	P.V188V
XH_P36	18	21921518	21921518	Missense_Mutation	SNV	C	T	T	OSBPL1A	c.387G>A	P.M129I
XH_P36	18	265533	265533	Splice_Site	INDEL	A	-	-	THOC1	c.55-3delT	
XH_P36	18	2775745	2775745	Missense_Mutation	SNV	C	T	T	SMCHD1	c.5189C>T	P.A1730V
XH_P36	18	30275527	30275528	Splice_Site	INDEL	AA	-	-	KLHL14	70-13_1070-12delTT	
XH_P36	18	3116424	3116424	Frame_Shift_Del	INDEL	C	-	-	MYOM1	c.3208delG	P.V1070fs
XH_P36	18	55103822	55103822	Missense_Mutation	SNV	C	A	A	ONECUT2	c.874C>A	P.L292M
XH_P36	18	56651438	56651438	Missense_Mutation	SNV	G	A	A	ZNF532	c.3646G>A	P.V1216I
XH_P36	18	66377374	66377374	Missense_Mutation	SNV	G	A	A	TMX3	c.149C>T	P.A50V
XH_P36	18	66678157	66678157	Splice_Site	INDEL	T	-	-	CCDC102B	c.1264-11delT	
XH_P36	18	74091072	74091072	Missense_Mutation	SNV	G	A	A	ZNF516	c.2998C>T	P.R1000C
XH_P36	18	76753476	76753476	Silent	SNV	G	A	A	SALL3	c.1485G>A	P.S495S
XH_P36	18	77624107	77624107	Missense_Mutation	SNV	C	T	T	KCNG2	c.440C>T	P.A147V
XH_P36	18	8824907	8824907	Silent	SNV	C	T	T	MTCL1	c.3399C>T	P.A1133A
XH_P36	19	10577862	10577862	Silent	SNV	C	T	T	PDE4A	c.2226C>T	P.V742V
XH_P36	19	10812678	10812678	Missense_Mutation	SNV	C	A	A	QTRT1	c.296C>A	P.P99H
XH_P36	19	1105178	1105178	Splice_Site	SNV	C	A	A	GPX4	c.85-7C>A	
XH_P36	19	11141507	11141507	Missense_Mutation	SNV	G	A	A	SMARCA4	c.3484G>A	P.G1162S
XH_P36	19	13319692	13319694	Missense_Mutation	INDEL	GAT	-	-	CACNA1A	6668_6670delA	P.H2223del
XH_P36	19	1585169	1585169	Missense_Mutation	SNV	T	C	C	MBD3	c.155A>G	P.Y52C
XH_P36	19	1819941	1819941	Missense_Mutation	SNV	C	T	T	REXO1	c.2642G>A	P.G881D
XH_P36	19	19654762	19654762	Frame_Shift_Del	INDEL	C	-	-	CILP2	c.1412delC	P.P471fs
XH_P36	19	20116817	20116817	Silent	SNV	C	T	T	ZNF682	c.1398G>A	P.T466T
XH_P36	19	23927290	23927290	Silent	SNV	G	A	A	ZNF681	c.1062C>T	P.T354T
XH_P36	19	30312660	30312660	Missense_Mutation	SNV	C	T	T	CCNE1	c.641C>T	P.A214V
XH_P36	19	30313453	30313453	Silent	SNV	C	T	T	CCNE1	c.1053C>T	P.G351G
XH_P36	19	31768042	31768042	Missense_Mutation	SNV	G	A	A	TSHZ3	c.2657C>T	P.T886M
XH_P36	19	31768155	31768155	Silent	SNV	G	A	A	TSHZ3	c.2544C>T	P.S848S
XH_P36	19	33167417	33167417	Frame_Shift_Ins	INDEL	-	G	G	RGS9BP	c.251dupG	P.C85fs
XH_P36	19	35524868	35524868	Missense_Mutation	SNV	C	T	T	SCN1B	c.673C>T	P.R225C
XH_P36	19	36223175	36223175	Frame_Shift_Del	INDEL	C	-	-	KMT2B	c.5730delC	P.R1911fs
XH_P36	19	36362899	36362899	Missense_Mutation	SNV	C	T	T	APLP1	c.811C>T	P.P271S
XH_P36	19	36370092	36370092	Silent	SNV	C	T	T	APLP1	c.1830C>T	P.Y610Y
XH_P36	19	36940815	36940815	Frame_Shift_Del	INDEL	T	-	-	ZNF566	c.324delA	P.K108fs
XH_P36	19	37005981	37005981	Missense_Mutation	SNV	A	T	T	ZNF260	c.160T>A	P.S54T
XH_P36	19	38028415	38028415	Frame_Shift_Del	INDEL	T	-	-	ZNF793	c.862delT	P.C288fs
XH_P36	19	3831790	3831790	Missense_Mutation	SNV	C	T	T	ZFR2	c.466G>A	P.G156R
XH_P36	19	38655461	38655461	Missense_Mutation	SNV	G	A	A	SIPA1L3	c.4123G>A	P.G1375S
XH_P36	19	38885396	38885396	Frame_Shift_Ins	INDEL	-	C	C	SPRED3	c.544dupC	P.Q182fs
XH_P36	19	39360822	39360822	Missense_Mutation	SNV	A	G	G	RINL	c.1103T>C	P.L368P
XH_P36	19	39379738	39379738	Missense_Mutation	SNV	A	G	G	SIRT2	c.379T>C	P.C127R
XH_P36	19	41518269	41518269	Missense_Mutation	SNV	C	T	T	CYP2B6	c.1031C>T	P.A344V
XH_P36	19	41631436	41631436	Silent	SNV	C	T	T	CYP2F1	c.1191C>T	P.Y397Y
XH_P36	19	4207239	4207239	Missense_Mutation	SNV	C	T	T	ANKRD24	c.467C>T	P.A156V
XH_P36	19	4445624	4445624	Splice_Site	SNV	C	A	A	UBXN6	c.1042-4G>T	
XH_P36	19	45803051	45803051	Splice_Site	INDEL	-	CCCCT	CCCCT	MARK4	8-7_1878-3dupCCCCT	
XH_P36	19	46116858	46116858	Missense_Mutation	SNV	C	T	T	EML2	c.2368G>A	P.A790T
XH_P36	19	46289347	46289347	Silent	SNV	C	T	T	DMWD	c.1407G>A	P.T469T
XH_P36	19	48184060	48184060	Missense_Mutation	SNV	G	A	A	GLTSCR1	c.1633G>A	P.A545T
XH_P36	19	49000877	49000877	Missense_Mutation	SNV	A	G	G	LMTK3	c.3536T>C	P.L1179P
XH_P36	19	49657889	49657889	Missense_Mutation	INDEL	-	TCCTCC	TCCTCC	HRC	00_605dupGGA	P.E201_E202dup
XH_P36	19	49860534	49860534	Nonsense_Mutation	SNV	G	T	T	TEAD2	c.335C>A	P.S112*
XH_P36	19	49934716	49934716	Splice_Site	INDEL	G	-	-	SLC17A7	c.1151-11delC	
XH_P36	19	5245795	5245795	Missense_Mutation	SNV	G	A	A	PTPRS	c.980C>T	P.T327M
XH_P36	19	52716080	52716080	Silent	SNV	C	T	T	PPP2R1A	c.645C>T	P.D215D
XH_P36	19	53644695	53644695	Silent	SNV	G	A	A	ZNF347	c.1389C>T	P.C463C
XH_P36	19	55481600	55481600	Missense_Mutation	SNV	C	A	A	NLRP2	c.217C>A	P.L73I
XH_P36	19	55598073	55598073	Splice_Site	SNV	C	T	T	EPS8L1	c.1397-9C>T	
XH_P36	19	56127331	56127331	Frame_Shift_Del	INDEL	G	-	-	ZNF865	c.2351delG	P.G784fs
XH_P36	19	58152336	58152336	Missense_Mutation	SNV	C	T	T	ZNF211	c.677C>T	P.A226V
XH_P36	19	58213374	58213374	Missense_Mutation	SNV	T	C	C	ZNF154	c.943A>G	P.S315G
XH_P36	19	58485478	58485478	Missense_Mutation	SNV	G	A	A	C19orf18	c.215C>T	P.S72L
XH_P36	19	6750380	6750380	Frame_Shift_Del	INDEL	C	-	-	TRIP10	c.1479delC	P.A494fs
XH_P36	19	7512066	7512066	Splice_Site	SNV	T	A	A	ARHGEF18	c.1173+12T>A	
XH_P36	19	8156455	8156455	Missense_Mutation	SNV	C	A	A	FBN3	c.5925G>T	P.E1975D
XH_P36	19	9018533	9018533	Silent	SNV	G	T	T	MUC16	c.37641C>A	P.T12547T
XH_P36	2	102482928	102482928	Missense_Mutation	SNV	A	C	C	MAP4K4	c.2009A>C	P.K670T
XH_P36	2	112705139	112705139	Missense_Mutation	SNV	T	C	C	MERTK	c.752T>C	P.V251A
XH_P36	2	112942828	112942828	Missense_Mutation	SNV	T	C	C	FBLN7	c.721T>C	P.C241R
XH_P36	2	120303807	120303807	Frame_Shift_Del	INDEL	A	-	-	CFAP221	c.106delA	P.R36fs
XH_P36	2	120677822	120677822	Splice_Site	SNV	G	A	A	PTPN4	c.1001+5G>A	
XH_P36	2	122205058	122205058	Silent	SNV	C	T	T	CLASP1	c.1767G>A	P.V589V
XH_P36	2	128467115	128467115	Missense_Mutation	SNV	G	A	A	WDR33	c.3514C>T	P.R1172C
XH_P36	2	128477850	128477850	Silent	SNV	G	A	A	WDR33	c.1749C>T	P.L583L
XH_P36	2	131803707	131803707	Missense_Mutation	SNV	G	A	A	ARHGEF4	c.2018G>A	P.R673H
XH_P36	2	141773495	141773495	Splice_Site	INDEL	A	-	-	LRP1B	c.1971-11delT	
XH_P36	2	143798030	143798030	Frame_Shift_Del	INDEL	A	-	-	KYNU	c.1080delA	P.K360fs
XH_P36	2	148657041	148657041	Frame_Shift_Del	INDEL	A	-	-	ACVR2A	c.285delA	P.D96fs
XH_P36	2	148683686	148683686	Frame_Shift_Del	INDEL	A	-	-	ACVR2A	c.1310delA	P.K437fs
XH_P36	2	160075810	160075810	Missense_Mutation	SNV	C	T	T	TANC1	c.3416C>T	P.T1139M
XH_P36	2	166785799	166785799	Missense_Mutation	SNV	C	T	T	TTC21B	c.1232G>A	P.R411Q

XH_P36	2	170366973	170366973	Frame_Shift_Ins	INDEL	-	A	A	KLHL41	c.690dupA	P.Y231fs
XH_P36	2	170460581	170460581	Frame_Shift_Del	INDEL	T	-	-	PPIG	c.36delT	P.F12fs
XH_P36	2	172549339	172549339	Frame_Shift_Del	INDEL	A	-	-	DYNC1I2	c.169delA	P.R57fs
XH_P36	2	179478981	179478981	Silent	SNV	A	G	G	TTN	c.44220T>C	P.S14740S
XH_P36	2	179575449	179575449	Missense_Mutation	SNV	C	T	T	TTN	c.27424G>A	P.V9142I
XH_P36	2	182763804	182763804	Silent	SNV	C	A	A	SSFA2	c.468C>A	P.S156S
XH_P36	2	186662103	186662103	Frame_Shift_Del	INDEL	A	-	-	FSIP2	c.10247delA	P.K3416fs
XH_P36	2	187615880	187615880	Silent	SNV	G	A	A	FAM171B	c.744G>A	P.L248L
XH_P36	2	189866275	189866275	Frame_Shift_Del	INDEL	C	-	-	COL3A1	c.2355delC	P.G786fs
XH_P36	2	191941055	191941055	Splice_Site	INDEL	A	-	-	STAT4	c.274-4delT	
XH_P36	2	202149545	202149545	Missense_Mutation	SNV	T	C	C	CASP8	c.860T>C	P.L287S
XH_P36	2	207172364	207172364	Missense_Mutation	SNV	G	A	A	ZDBF2	c.3106G>A	P.G1036S
XH_P36	2	207174428	207174428	Frame_Shift_Del	INDEL	A	-	-	ZDBF2	c.5178delA	P.K1726fs
XH_P36	2	20870507	20870507	Frame_Shift_Del	INDEL	C	-	-	GDF7	c.682delC	P.R228fs
XH_P36	2	210840978	210840978	Missense_Mutation	SNV	G	A	A	UNC80	c.8525G>A	P.R2842Q
XH_P36	2	219892362	219892362	Missense_Mutation	SNV	G	A	A	CFAP65	c.2188C>T	P.R730C
XH_P36	2	219896326	219896326	Missense_Mutation	SNV	C	T	T	CFAP65	c.667G>A	P.G223S
XH_P36	2	233410400	233410400	Missense_Mutation	SNV	C	T	T	CHRNG	c.1528C>T	P.R510C
XH_P36	2	234112838	234112838	Silent	SNV	C	T	T	INPP5D	c.2955C>T	P.P985P
XH_P36	2	234365952	234365952	Frame_Shift_Del	INDEL	G	-	-	DGKD	c.2432delG	P.G811fs
XH_P36	2	23865497	23865497	Silent	SNV	C	T	T	KLHL29	c.717C>T	P.P239P
XH_P36	2	239057734	239057734	Missense_Mutation	SNV	A	G	G	KLHL30	c.1426A>G	P.I476V
XH_P36	2	239258015	239258015	Missense_Mutation	SNV	C	T	T	TRAF3IP1	c.1219C>T	P.R407W
XH_P36	2	242179074	242179074	Silent	SNV	G	A	A	HDLBP	c.2454C>T	P.G818G
XH_P36	2	27248517	27248517	Frame_Shift_Del	INDEL	C	-	-	MAPRE3	c.543delC	P.C182fs
XH_P36	2	29016762	29016762	Nonsense_Mutation	SNV	C	T	T	PPP1CB	c.778C>T	P.R260*
XH_P36	2	29455174	29455174	Silent	SNV	G	A	A	ALK	c.2628C>T	P.A876A
XH_P36	2	31010045	31010045	Missense_Mutation	SNV	C	A	A	CAPN13	c.147G>T	P.Q49H
XH_P36	2	36704119	36704119	Frame_Shift_Del	INDEL	A	-	-	CRIM1	c.1080delA	P.G362fs
XH_P36	2	37347123	37347123	Frame_Shift_Del	INDEL	T	-	-	EIF2AK2	c.1227delA	P.K409fs
XH_P36	2	3750103	3750103	Missense_Mutation	SNV	C	T	T	ALLC	c.1126C>T	P.R376W
XH_P36	2	39559136	39559136	Splice_Site	INDEL	A	-	-	MAP4K3	c.458-6delT	
XH_P36	2	42720141	42720141	Silent	SNV	C	T	T	KCNG3	c.501G>A	P.S167S
XH_P36	2	42922889	42922891	Splice_Site	INDEL	TTT	-	-	MTA3	724-6_724-4delTTT	
XH_P36	2	50765682	50765682	Frame_Shift_Ins	INDEL	-	C	C	NRXN1	c.1971dupG	P.L658fs
XH_P36	2	54028522	54028522	Splice_Site	INDEL	T	-	-	ERLEC1	c.526-4delT	
XH_P36	2	60987361	60987361	Missense_Mutation	SNV	A	G	G	PAPOLG	c.110A>G	P.Y37C
XH_P36	2	61577527	61577527	Splice_Site	INDEL	A	-	-	USP34	c.1378-3delT	
XH_P36	2	71046551	71046551	Silent	SNV	C	T	T	CLEC4F	c.204G>A	P.P68P
XH_P36	2	73518631	73518631	Missense_Mutation	SNV	T	C	C	EGR4	c.1724A>G	P.K575R
XH_P36	2	74326498	74326498	Silent	SNV	G	T	T	TET3	c.3363G>T	P.T1121T
XH_P36	2	74900900	74900900	Missense_Mutation	SNV	G	A	A	SEMA4F	c.767G>A	P.R256Q
XH_P36	2	84942754	84942754	Splice_Site	INDEL	A	-	-	DNAH6	c.9611+9delA	
XH_P36	2	85043102	85043102	Missense_Mutation	SNV	C	T	T	DNAH6	c.12268C>T	P.P4090S
XH_P36	2	97475131	97475131	Silent	SNV	G	A	A	CNNM4	c.2205G>A	P.G735G
XH_P36	2	97638040	97638040	Silent	SNV	G	A	A	FAM178B	c.162C>T	P.T54T
XH_P36	20	14307546	14307546	Missense_Mutation	SNV	G	A	A	FLRT3	c.607C>T	P.R203C
XH_P36	20	16347868	16347868	Missense_Mutation	SNV	C	T	T	KIF16B	c.4102G>A	P.A1368T
XH_P36	20	18286958	18286958	Missense_Mutation	SNV	A	T	T	ZNF133	c.138A>T	P.K46N
XH_P36	20	25000780	25000780	Missense_Mutation	SNV	C	T	T	ACSS1	c.1112G>A	P.R371Q
XH_P36	20	25457670	25457670	Missense_Mutation	SNV	C	T	T	NINL	c.2257G>A	P.A753T
XH_P36	20	259074	259074	Splice_Site	SNV	C	T	T	C20orf96	c.463-4G>A	
XH_P36	20	3003414	3003414	Missense_Mutation	SNV	G	A	A	PTPRA	c.1408G>A	P.V470M
XH_P36	20	30606904	30606904	Frame_Shift_Del	INDEL	G	-	-	CCM2L	c.629delG	P.G210fs
XH_P36	20	33345744	33345744	Silent	SNV	C	T	T	NCOA6	c.807G>A	P.Q269Q
XH_P36	20	33589096	33589096	Missense_Mutation	SNV	G	A	A	MYH7B	c.5650G>A	P.E1884K
XH_P36	20	34022391	34022391	Silent	SNV	C	T	T	GDF5	c.822G>A	P.P274P
XH_P36	20	34208670	34208670	Missense_Mutation	SNV	C	T	T	SPAG4	c.911C>T	P.S304L
XH_P36	20	36031782	36031782	Nonstop_Mutation	SNV	G	T	T	SRC	c.1611G>T	P.Ter537Yext*?
XH_P36	20	37357196	37357196	Missense_Mutation	SNV	G	A	A	SLC32A1	c.1492G>A	P.V498I
XH_P36	20	42204858	42204858	Splice_Site	INDEL	C	-	-	SGK2	c.694-3delC	
XH_P36	20	4228843	4228843	Silent	SNV	G	A	A	ADRA1D	c.762C>T	P.Y254Y
XH_P36	20	42788776	42788776	Silent	SNV	G	A	A	JPH2	c.651C>T	P.G217G
XH_P36	20	44530971	44530971	Splice_Site	SNV	G	A	A	PLTP	c.825C>T	P.D275D
XH_P36	20	56090848	56090848	Missense_Mutation	SNV	G	A	A	CTCFL	c.1102C>T	P.R368C
XH_P36	20	57768838	57768838	Missense_Mutation	SNV	C	A	A	ZNF831	c.2764C>A	P.P922T
XH_P36	20	57768886	57768886	Frame_Shift_Ins	INDEL	-	C	C	ZNF831	c.2817dupC	P.K940fs
XH_P36	20	5903414	5903414	Frame_Shift_Del	INDEL	A	-	-	CHGB	c.630delA	P.E211fs
XH_P36	20	60705321	60705321	Silent	SNV	C	T	T	LSM14B	c.642C>T	P.D214D
XH_P36	20	60963347	60963348	Splice_Site	INDEL	TT	-	-	RPS21	187-8_187-7delTT	
XH_P36	20	62196336	62196336	Missense_Mutation	SNV	A	G	G	HELZ2	c.3839T>C	P.L1280P
XH_P36	20	62199927	62199927	Missense_Mutation	SNV	C	G	G	HELZ2	c.1514G>C	P.R505T
XH_P36	21	17203704	17203704	Splice_Site	INDEL	T	-	-	USP25	c.1765-10delT	
XH_P36	21	22664532	22664532	Missense_Mutation	SNV	A	T	T	NCAM2	c.590A>T	P.D197V
XH_P36	21	42866456	42866456	Missense_Mutation	SNV	G	A	A	TMPRSS2	c.176C>T	P.P59L
XH_P36	21	43510462	43510462	Missense_Mutation	SNV	G	A	A	UMODL1	c.845G>A	P.C282Y
XH_P36	21	45877262	45877262	Silent	SNV	C	T	T	LRRC3	c.735C>T	P.S245S
XH_P36	22	16269950	16269950	Splice_Site	INDEL	A	-	-	POTEH	c.1238-7delT	
XH_P36	22	18096096	18096096	Splice_Site	SNV	A	C	C	ATP6V1E1	c.34-10T>G	
XH_P36	22	20843349	20843349	Silent	SNV	G	A	A	KLHL22	c.150C>T	P.F50F
XH_P36	22	25625546	25625546	Splice_Site	SNV	G	A	A	CRYBB2	c.449+1G>A	
XH_P36	22	26239838	26239838	Silent	SNV	C	A	A	MYO18B	c.3348C>A	P.P1116P
XH_P36	22	29446275	29446275	Frame_Shift_Del	INDEL	G	-	-	ZNRF3	c.2111delG	P.G704fs
XH_P36	22	30074208	30074208	Silent	SNV	G	A	A	NF2	c.1470G>A	P.P490P
XH_P36	22	32937634	32937634	Silent	SNV	G	A	A	SYN3	c.837C>T	P.Y279Y
XH_P36	22	34022242	34022242	Silent	SNV	G	A	A	LARGE1	c.477C>T	P.S159S
XH_P36	22	36624038	36624038	Missense_Mutation	SNV	T	G	G	APOL2	c.426A>C	P.E142D
XH_P36	22	39262986	39262986	Missense_Mutation	SNV	G	A	A	CBX6	c.413C>T	P.T138M
XH_P36	22	42024155	42024155	Frame_Shift_Del	INDEL	T	-	-	XRCC6	c.122delT	P.L41fs
XH_P36	22	44583645	44583645	Splice_Site	SNV	G	A	A	PARVG	c.145-11G>A	
XH_P36	22	44681360	44681360	Missense_Mutation	SNV	G	A	A	KIAA1644	c.547C>T	P.R183W
XH_P36	22	46932163	46932163	Missense_Mutation	SNV	G	A	A	CELSR1	c.905C>T	P.T302M
XH_P36	22	50591520	50591520	Missense_Mutation	SNV	T	C	C	MOV10L1	c.2939T>C	P.M980T
XH_P36	22	50728589	50728589	Missense_Mutation	SNV	G	A	A	PLXNB2	c.425C>T	P.S142F
XH_P36	22	50987509	50987509	Missense_Mutation	SNV	G	A	A	KLHDC7B	c.914G>A	P.G305D
XH_P36	22	51042673	51042673	Silent	SNV	C	T	T	MAPK8IP2	c.945C>T	P.A315A
XH_P36	3	10301806	10301806	Splice_Site	INDEL	T	-	-	TATDN2	c.415-6delT	
XH_P36	3	108139971	108139971	Silent	SNV	G	A	A	MYH15	c.3853C>T	P.L1285L

XH_P36	3	111828413	111828413	Silent	SNV	G	A	A	C3orf52	c.420G>A	P.S140S
XH_P36	3	111940075	111940075	Splice_Site	INDEL	A	-	-	SLC9C1	c.1429-3delT	
XH_P36	3	112538716	112538716	Silent	SNV	A	G	G	CD200R1L	c.706T>C	P.L236L
XH_P36	3	112648129	112648129	Missense_Mutation	SNV	C	T	T	CD200R1	c.428G>A	P.R143H
XH_P36	3	113377483	113377483	Missense_Mutation	SNV	T	G	G	USF3	c.3046A>C	P.N1016H
XH_P36	3	122811188	122811188	Splice_Site	INDEL	T	-	-	PDIA5	c.170-5delT	
XH_P36	3	130107403	130107403	Splice_Site	INDEL	T	-	-	COL6A5	c.1862-12delT	
XH_P36	3	132051057	132051057	Missense_Mutation	SNV	G	A	A	ACPP	c.325G>A	P.V109I
XH_P36	3	136191339	136191339	Splice_Site	INDEL	-	A	A	STAG1	c.1126-6dupT	
XH_P36	3	1371501	1371501	Frame_Shift_Del	INDEL	A	-	-	CNTN6	c.1253delA	P.K418fs
XH_P36	3	138739338	138739338	Missense_Mutation	SNV	C	T	T	PRR23B	c.166G>A	P.A56T
XH_P36	3	148459721	148459721	Frame_Shift_Del	INDEL	T	-	-	AGTR1	c.904delT	P.Y302fs
XH_P36	3	150285470	150285470	Splice_Site	INDEL	T	-	-	EIF2A	c.401-3delT	
XH_P36	3	153842199	153842199	Frame_Shift_Del	INDEL	A	-	-	ARHGEF26	c.1090delA	P.M364fs
XH_P36	3	160231939	160231939	Missense_Mutation	SNV	C	T	T	KPNA4	c.1063G>A	P.A355T
XH_P36	3	161221015	161221015	Missense_Mutation	SNV	A	G	G	OTOL1	c.719A>G	P.D240G
XH_P36	3	175042056	175042056	Frame_Shift_Ins	INDEL	-	T	T	NAALADL2	c.1034dupT	P.M346fs
XH_P36	3	178952085	178952085	Missense_Mutation	SNV	A	G	G	PIK3CA	c.3140A>G	P.H1047R
XH_P36	3	183888195	183888195	Missense_Mutation	SNV	C	G	G	DVL3	c.1803C>G	P.S601R
XH_P36	3	195295892	195295892	Missense_Mutation	SNV	G	A	A	APOD	c.449C>T	P.A150V
XH_P36	3	25654189	25654189	Silent	SNV	A	G	G	TOP2B	c.3588T>C	P.S1196S
XH_P36	3	25657082	25657082	Splice_Site	INDEL	A	-	-	TOP2B	c.3337-5delT	
XH_P36	3	30691872	30691873	Frame_Shift_Del	INDEL	AA	-	-	TGFBR2	c.457_458delAA	P.K153fs
XH_P36	3	367670	367670	Silent	SNV	G	A	A	CHL1	c.120G>A	P.Q40Q
XH_P36	3	44692631	44692631	Silent	SNV	A	G	G	ZNF35	c.72A>G	P.E24E
XH_P36	3	50005145	50005145	Frame_Shift_Del	INDEL	G	-	-	RBM6	c.293delG	P.G98fs
XH_P36	3	50324240	50324240	Missense_Mutation	SNV	C	T	T	LSMEM2	c.305C>T	P.A102V
XH_P36	3	52437315	52437315	Splice_Site	SNV	C	T	T	BAP1	c.1730-1G>A	
XH_P36	3	53263342	53263342	Silent	SNV	G	A	A	TKT	c.1218C>T	P.S406S
XH_P36	3	55018615	55018615	Splice_Site	INDEL	T	-	-	CACNA2D3	c.2557-11delT	
XH_P36	3	57440505	57440505	Silent	SNV	C	T	T	DNAH12	c.3453G>A	P.T1151T
XH_P36	3	77657037	77657037	Frame_Shift_Ins	INDEL	-	C	C	ROBO2	c.3173dupC	P.V1059fs
XH_P36	3	9970326	9970326	Splice_Site	SNV	G	A	A	IL17RC	c.1110+12G>A	
XH_P36	4	107156505	107156505	Frame_Shift_Del	INDEL	T	-	-	TBCK	c.1370delA	P.N457fs
XH_P36	4	114286281	114286281	Missense_Mutation	SNV	C	T	T	ANK2	c.4693C>T	P.R1565C
XH_P36	4	120189503	120189503	Missense_Mutation	SNV	C	A	A	USP53	c.1216C>A	P.Q406K
XH_P36	4	123270296	123270296	Splice_Site	INDEL	T	-	-	KIAA1109	c.13281-11delT	
XH_P36	4	134071649	134071649	Frame_Shift_Del	INDEL	C	-	-	PCDH10	c.360delC	P.S121fs
XH_P36	4	134073341	134073341	Silent	SNV	C	A	A	PCDH10	c.2046C>A	P.A682A
XH_P36	4	138442370	138442370	Missense_Mutation	SNV	C	T	T	PCDH18	c.3218G>A	P.G1073E
XH_P36	4	155467079	155467079	Splice_Site	SNV	G	T	T	PLRG1	c.378-4C>A	
XH_P36	4	155470090	155470090	Splice_Site	INDEL	A	-	-	PLRG1	c.10-3delT	
XH_P36	4	184431426	184431426	Splice_Site	INDEL	T	-	-	ING2	c.53-3delT	
XH_P36	4	189022306	189022306	Missense_Mutation	SNV	C	A	A	TRIML2	c.384G>T	P.Q128H
XH_P36	4	22820431	22820431	Missense_Mutation	SNV	G	A	A	GBA3	c.374G>A	P.R125Q
XH_P36	4	25278714	25278714	Missense_Mutation	SNV	C	T	T	PI4K2B	c.1351C>T	P.R451C
XH_P36	4	2896294	2896294	Splice_Site	INDEL	T	-	-	ADD1	c.592-9delT	
XH_P36	4	38126762	38126762	Splice_Site	SNV	A	G	G	TBC1D1	c.3105+10A>G	
XH_P36	4	39511531	39511532	Splice_Site	INDEL	AA	-	-	UGDH	463-5_463-4delTT	
XH_P36	4	39864524	39864524	Missense_Mutation	SNV	C	T	T	PDS5A	c.2936G>A	P.R979H
XH_P36	4	4304807	4304807	Missense_Mutation	SNV	G	A	A	ZBTB49	c.1244G>A	P.R415Q
XH_P36	4	5018651	5018651	Missense_Mutation	SNV	G	A	A	CYTL1	c.239C>T	P.S80L
XH_P36	4	57179502	57179502	Frame_Shift_Ins	INDEL	-	A	A	KIAA1211	c.501dupA	P.Q168fs
XH_P36	4	68488908	68488908	Nonsense_Mutation	SNV	C	T	T	UBA6	c.2873G>A	P.W958*
XH_P36	4	70825805	70825805	Splice_Site	INDEL	A	-	-	CSN2	c.52-8delT	
XH_P36	4	81791254	81791254	Frame_Shift_Del	INDEL	A	-	-	C4orf22	c.499delA	P.R167fs
XH_P36	4	89653284	89653284	Silent	SNV	G	A	A	FAM13A	c.1734C>T	P.T578T
XH_P36	4	95173909	95173909	Frame_Shift_Ins	INDEL	-	A	A	SMARCAD1	c.1040dupA	P.N347fs
XH_P36	4	95191919	95191919	Silent	SNV	C	T	T	SMARCAD1	c.1522C>T	P.L508L
XH_P36	4	961739	961739	Missense_Mutation	SNV	G	A	A	DGKQ	c.740C>T	P.A247V
XH_P36	4	99339795	99339795	Splice_Site	INDEL	T	-	-	RAP1GDS1	c.1043-4delT	
XH_P36	5	112346433	112346436	Splice_Site	INDEL	TTTT	-	-	DCP2	43-10_943-7delTTTT	
XH_P36	5	112769886	112769886	Silent	SNV	G	A	A	TSSK1B	c.651C>T	P.D217D
XH_P36	5	118324039	118324039	Missense_Mutation	SNV	C	A	A	DTWD2	c.168G>T	P.W56C
XH_P36	5	1212555	1212555	Missense_Mutation	SNV	G	A	A	SLC6A19	c.619G>A	P.V207I
XH_P36	5	121413097	121413097	Missense_Mutation	SNV	G	T	T	LOX	c.584C>A	P.P195H
XH_P36	5	134050742	134050742	Missense_Mutation	SNV	G	A	A	SEC24A	c.2756G>A	P.R919H
XH_P36	5	138265000	138265000	Silent	SNV	C	T	T	CTNNA1	c.1965C>T	P.S655S
XH_P36	5	140047894	140047894	Silent	SNV	T	C	C	WDR55	c.267T>C	P.A89A
XH_P36	5	140049102	140049102	Frame_Shift_Del	INDEL	A	-	-	WDR55	c.1022delA	P.K341fs
XH_P36	5	140230590	140230590	Frame_Shift_Del	INDEL	T	-	-	PCDHA9	c.2517delT	P.L840fs
XH_P36	5	140237134	140237134	Missense_Mutation	SNV	C	T	T	PCDHA10	c.1501C>T	P.R501C
XH_P36	5	140568218	140568218	Silent	SNV	G	A	A	PCDHB9	c.1326G>A	P.P442P
XH_P36	5	140604336	140604336	Missense_Mutation	SNV	C	T	T	PCDHB14	c.1259C>T	P.T420M
XH_P36	5	140683367	140683367	Silent	SNV	C	T	T	SLC25A2	c.66G>A	P.A22A
XH_P36	5	140769366	140769366	Missense_Mutation	SNV	C	T	T	PCDHGB4	c.1915C>T	P.R639C
XH_P36	5	140953564	140953569	Missense_Mutation	INDEL	GGAGGA	-	-	DIAPH1	321_1826delTCC	P.P608_P609del
XH_P36	5	143586393	143586393	Missense_Mutation	SNV	G	A	A	KCTD16	c.116G>A	P.R39H
XH_P36	5	145547779	145547779	Frame_Shift_Del	INDEL	G	-	-	LARS	c.344delC	P.P115fs
XH_P36	5	146754725	146754725	Frame_Shift_Del	INDEL	A	-	-	STK32A	c.983delA	P.K328fs
XH_P36	5	148407222	148407222	Silent	SNV	G	T	T	SH3TC2	c.2073C>A	P.A691A
XH_P36	5	149602668	149602668	Silent	SNV	G	A	A	CAMK2A	c.1350C>T	P.D450D
XH_P36	5	149901203	149901203	Silent	SNV	C	T	T	NDST1	c.387C>T	P.G129G
XH_P36	5	154173390	154173390	Frame_Shift_Del	INDEL	C	-	-	LARP1	c.675delC	P.T226fs
XH_P36	5	15928091	15928091	Missense_Mutation	SNV	T	C	C	FBXL7	c.79T>C	P.S27P
XH_P36	5	16179661	16179661	Silent	SNV	G	A	A	MARCH11	c.24C>T	P.G8G
XH_P36	5	162917426	162917426	Frame_Shift_Del	INDEL	A	-	-	HMMR	c.2001delA	P.K667fs
XH_P36	5	167841357	167841357	Missense_Mutation	SNV	G	A	A	WWC1	c.946G>A	P.A316T
XH_P36	5	169129368	169129368	Missense_Mutation	SNV	G	T	T	DOCK2	c.1320G>T	P.K440N
XH_P36	5	171523524	171523524	Missense_Mutation	SNV	C	T	T	STK10	c.911G>A	P.R304Q
XH_P36	5	175775279	175775279	Silent	SNV	C	T	T	KIAA1191	c.483G>A	P.P161P
XH_P36	5	176813456	176813456	Missense_Mutation	SNV	G	A	A	SLC34A1	c.421G>A	P.A141T
XH_P36	5	176863433	176863433	Frame_Shift_Del	INDEL	G	-	-	GRK6	c.1310delG	P.G437fs
XH_P36	5	176916634	176916634	Splice_Site	SNV	G	A	A	PDLIM7	c.635-6C>T	
XH_P36	5	178140631	178140631	Splice_Site	INDEL	A	-	-	ZNF354A	c.131-9delT	
XH_P36	5	1882750	1882750	Silent	SNV	C	T	T	IRX4	c.12G>A	P.P4P
XH_P36	5	32091037	32091037	Missense_Mutation	SNV	A	G	G	PDZD2	c.7483A>G	P.K2495E
XH_P36	5	37438050	37438050	Silent	SNV	G	A	A	WDR70	c.519G>A	P.S173S

XH_P36	5	38903971	38903971	Splice_Site	INDEL	T	-	-	OSMR	c.992-6delT	
XH_P36	5	482774	482774	Silent	SNV	G	A	A	SLC9A3	c.1245C>T	P.R415R
XH_P36	5	5466430	5466430	Splice_Site	INDEL	T	-	-	ICE1	c.5893-9delT	
XH_P36	5	61847115	61847115	Frame_Shift_Del	INDEL	T	-	-	IPO11	c.2539delT	P.S847fs
XH_P36	5	64867698	64867698	Missense_Mutation	SNV	G	A	A	PPWD1	c.464G>A	P.C155Y
XH_P36	5	70766233	70766233	Frame_Shift_Del	INDEL	T	-	-	BDP1	c.938delT	P.L313fs
XH_P36	5	71501039	71501039	Silent	SNV	C	A	A	MAP1B	c.7002C>A	P.S2334S
XH_P36	5	71756466	71756466	Silent	SNV	G	A	A	ZNF366	c.858C>T	P.C286C
XH_P36	5	76358967	76358967	Frame_Shift_Del	INDEL	A	-	-	AGGF1	c.2042delA	P.N681fs
XH_P36	5	82816284	82816284	Missense_Mutation	SNV	T	A	A	VCAN	c.2159T>A	P.V720D
XH_P36	5	86705190	86705190	Splice_Site	INDEL	A	-	-	CCNH	c.82-10delT	
XH_P36	5	87502325	87502328	Splice_Site	INDEL	GAAA	-	-	TMEM161B	99-12_599-9delTTTC	
XH_P36	5	9190436	9190436	Missense_Mutation	SNV	C	T	T	SEMA5A	c.1216G>A	P.A406T
XH_P36	5	96062499	96062499	Missense_Mutation	SNV	C	T	T	CAST	c.23C>T	P.A8V
XH_P36	5	96443159	96443159	Missense_Mutation	SNV	C	T	T	LIX1	c.292G>A	P.A98T
XH_P36	6	102074256	102074256	Splice_Site	SNV	C	A	A	GRIK2	c.285C>A	P.A95A
XH_P36	6	116263666	116263666	Nonsense_Mutation	SNV	G	A	A	FRK	c.1429C>T	P.R477*
XH_P36	6	118229019	118229019	Frame_Shift_Ins	INDEL	-	C	C	SLC35F1	c.133dupC	P.R45fs
XH_P36	6	118635181	118635181	Splice_Site	INDEL	-	T	T	SLC35F1	c.1003-3dupT	
XH_P36	6	132190481	132190481	Splice_Site	INDEL	T	-	-	ENPP1	c.1274-8delT	
XH_P36	6	139097330	139097330	Frame_Shift_Del	INDEL	A	-	-	CCDC28A	c.350delA	P.N117fs
XH_P36	6	142409404	142409404	Missense_Mutation	SNV	A	G	G	NMBR	c.392T>C	P.V131A
XH_P36	6	142711439	142711439	Missense_Mutation	SNV	C	T	T	ADGRG6	c.1183C>T	P.R395C
XH_P36	6	15374448	15374448	Missense_Mutation	SNV	C	T	T	JARID2	c.146C>T	P.A49V
XH_P36	6	160431705	160431705	Splice_Site	INDEL	T	-	-	IGF2R	c.415-6delT	
XH_P36	6	163149397	163149397	Missense_Mutation	SNV	G	T	T	PACRG	c.130G>T	P.V44F
XH_P36	6	21594968	21594968	Missense_Mutation	SNV	T	C	C	SOX4	c.203T>C	P.V68A
XH_P36	6	22290504	22290504	Missense_Mutation	SNV	G	A	A	PRL	c.391C>T	P.R131C
XH_P36	6	25799032	25799032	Missense_Mutation	SNV	T	C	C	SLC17A1	c.1385A>G	P.K462R
XH_P36	6	26598138	26598138	Splice_Site	SNV	G	A	A	ABT1	c.242-4G>A	
XH_P36	6	33653236	33653236	Missense_Mutation	SNV	C	T	T	ITPR3	c.5408C>T	P.T1803M
XH_P36	6	35426163	35426163	Silent	SNV	T	C	C	FANCE	c.1059T>C	P.P353P
XH_P36	6	41652798	41652798	Nonsense_Mutation	SNV	G	A	A	TFEB	c.970C>T	P.R324*
XH_P36	6	42235991	42235991	Missense_Mutation	SNV	G	C	C	TRERF1	c.1338C>G	P.S446R
XH_P36	6	43418816	43418816	Missense_Mutation	SNV	C	T	T	DLK2	c.502G>A	P.A168T
XH_P36	6	43420878	43420878	Splice_Site	SNV	C	T	T	DLK2	c.141-5G>A	
XH_P36	6	43476493	43476493	Splice_Site	SNV	C	T	T	LRRC73	c.433+5G>A	
XH_P36	6	52343894	52343894	Silent	SNV	C	T	T	EFHC1	c.1281C>T	P.T427T
XH_P36	6	52962507	52962507	Splice_Site	INDEL	T	-	-	FBXO9	c.1236-5delT	
XH_P36	6	53363590	53363590	Silent	SNV	T	C	C	GCLC	c.1764A>G	P.V588V
XH_P36	6	56047462	56047462	Splice_Site	INDEL	-	A	A	COL21A1	c.-38-9dupT	
XH_P36	6	62995837	62995837	Missense_Mutation	SNV	T	C	C	KHDRBS2	c.17A>G	P.Y6C
XH_P36	6	66012756	66012756	Missense_Mutation	SNV	G	T	T	LOC441155	c.342G>T	P.K114N
XH_P36	6	69785876	69785876	Splice_Site	INDEL	T	-	-	ADGRB3	c.2437-3delT	
XH_P36	6	80751910	80751910	Frame_Shift_Del	INDEL	A	-	-	TTK	c.2568delA	P.K856fs
XH_P36	6	84645930	84645930	Splice_Site	INDEL	T	-	-	CYB5R4	c.956-5delT	
XH_P36	6	86387221	86387222	Splice_Site	INDEL	AA	-	-	SNHG5	54-12_254-11delTT	
XH_P36	6	88221098	88221099	Splice_Site	INDEL	TT	-	-	SLC35A1	710-10_710-9delTT	
XH_P36	6	88318837	88318837	Frame_Shift_Ins	INDEL	-	A	A	ORC3	c.181dupA	P.R61fs
XH_P36	7	100730930	100730930	Frame_Shift_Del	INDEL	G	-	-	TRIM56	c.342delG	P.P115fs
XH_P36	7	103301977	103301977	Splice_Site	INDEL	A	-	-	RELN	c.1290-3delT	
XH_P36	7	112407656	112407656	Missense_Mutation	SNV	T	C	C	TMEM168	c.1690A>G	P.I564V
XH_P36	7	116397525	116397525	Missense_Mutation	SNV	C	T	T	MET	c.1897C>T	P.H633Y
XH_P36	7	127978355	127978355	Missense_Mutation	SNV	G	T	T	RBM28	c.490C>A	P.L164I
XH_P36	7	128485259	128485259	Missense_Mutation	SNV	C	T	T	FLNC	c.3740C>T	P.A1247V
XH_P36	7	128502976	128502976	Silent	SNV	G	A	A	ATP6V1F	c.18G>A	P.K6K
XH_P36	7	131012702	131012702	Missense_Mutation	SNV	T	G	G	MKLN1	c.44T>G	P.L15R
XH_P36	7	131831415	131831415	Missense_Mutation	SNV	G	A	A	PLXNA4	c.4909C>T	P.R1637W
XH_P36	7	134852563	134852563	Splice_Site	INDEL	A	-	-	C7orf49	c.175-4delT	
XH_P36	7	137151696	137151696	Splice_Site	INDEL	A	-	-	DGKI	c.2444-8delT	
XH_P36	7	138363260	138363260	Silent	SNV	C	T	T	SVOPL	c.93G>A	P.T31T
XH_P36	7	139724377	139724377	Missense_Mutation	SNV	A	T	T	PARP12	c.2089T>A	P.F697I
XH_P36	7	142565462	142565462	Missense_Mutation	SNV	C	T	T	EPHB6	c.971C>T	P.A324V
XH_P36	7	150721484	150721484	Frame_Shift_Del	INDEL	C	-	-	ATG9B	c.27delG	P.R10fs
XH_P36	7	150761288	150761288	Splice_Site	SNV	G	A	A	SLC4A2	c.52-1G>A	
XH_P36	7	151945313	151945313	Silent	SNV	A	G	G	KMT2C	c.2206T>C	P.L736L
XH_P36	7	158527147	158527147	Missense_Mutation	SNV	C	T	T	ESYT2	c.2602G>A	P.A868T
XH_P36	7	27183075	27183075	Missense_Mutation	SNV	C	T	T	HOXA5	c.152G>A	P.G51D
XH_P36	7	28858716	28858716	Splice_Site	INDEL	T	-	-	CREB5	c.947-9delT	
XH_P36	7	31682378	31682378	Missense_Mutation	SNV	C	T	T	CCDC129	c.1424C>T	P.S475F
XH_P36	7	31793063	31793063	Missense_Mutation	SNV	T	C	C	PDE1C	c.2245A>G	P.M749V
XH_P36	7	34997686	34997686	Splice_Site	SNV	G	A	A	DPY19L1	c.874-4C>T	
XH_P36	7	37889877	37889877	Frame_Shift_Ins	INDEL	-	A	A	NME8	c.15dupA	P.R6fs
XH_P36	7	44560659	44560659	Silent	SNV	C	T	T	NPC1L1	c.3012G>A	P.S1004S
XH_P36	7	45124001	45124001	Missense_Mutation	SNV	T	C	C	NACAD	c.1778A>G	P.D593G
XH_P36	7	53103788	53103788	Missense_Mutation	SNV	G	A	A	POM121L12	c.424G>A	P.A142T
XH_P36	7	56156631	56156631	Missense_Mutation	SNV	T	C	C	PHKG1	c.4A>G	P.T2A
XH_P36	7	5780887	5780887	Missense_Mutation	SNV	C	T	T	RNF216	c.761G>A	P.R254Q
XH_P36	7	64451956	64451956	Nonsense_Mutation	SNV	C	T	T	ERV3-1	c.1449G>A	P.W483*
XH_P36	7	6485741	6485742	Splice_Site	INDEL	AA	-	-	DAGLB	.96-7_96-6delTT	
XH_P36	7	6502731	6502731	Missense_Mutation	SNV	G	A	A	KDELR2	c.427C>T	P.R143W
XH_P36	7	6565850	6565850	Splice_Site	SNV	C	T	T	GRID2IP	c.919+5G>A	
XH_P36	7	6662266	6662266	Missense_Mutation	SNV	G	T	T	ZNF853	c.1644G>T	P.K548N
XH_P36	7	70597900	70597900	Missense_Mutation	SNV	G	A	A	WBSCR17	c.112G>A	P.A38T
XH_P36	7	73814818	73814818	Missense_Mutation	SNV	C	T	T	CLIP2	c.2999C>T	P.A1000V
XH_P36	7	77408086	77408086	Silent	SNV	C	T	T	RSBN1L	c.2142C>T	P.S714S
XH_P36	7	84671610	84671610	Splice_Site	INDEL	A	-	-	SEMA3D	c.862-9delT	
XH_P36	7	89939531	89939531	Frame_Shift_Del	INDEL	A	-	-	CFAP69	c.2810delA	P.K937fs
XH_P36	7	94037202	94037202	Missense_Mutation	SNV	C	G	G	COL1A2	c.638C>G	P.T213R
XH_P36	7	99312312	99312312	Splice_Site	INDEL	A	-	-	CYP3A7	c.671-7delT	
XH_P36	8	104897908	104897908	Nonsense_Mutation	SNV	C	T	T	RIMS2	c.1081C>T	P.R361*
XH_P36	8	105521191	105521191	Missense_Mutation	SNV	T	G	G	LRP12	c.191A>C	P.N64T
XH_P36	8	10755632	10755632	Missense_Mutation	SNV	T	C	C	XKR6	c.1756A>G	P.K586E
XH_P36	8	110464995	110464995	Frame_Shift_Ins	INDEL	-	G	G	PKHD1L1	c.6563dupG	P.S2190fs
XH_P36	8	110478806	110478806	Splice_Site	SNV	G	T	T	PKHD1L1	c.8414-1G>T	
XH_P36	8	110587336	110587336	Silent	SNV	G	A	A	SYBU	c.1788C>T	P.G596G
XH_P36	8	110986446	110986446	Missense_Mutation	SNV	A	T	T	KCNV1	c.172T>A	P.S58T
XH_P36	8	117862854	117862854	Splice_Site	SNV	T	C	C	RAD21	c.1620+3A>G	

XH_P36	8	121587323	121587323	Splice_Site	SNV	T	A	A	SNTB1	c.1136+3A>T	
XH_P36	8	125326260	125326260	Missense_Mutation	SNV	A	G	G	TMEM65	c.664T>C	P.F222L
XH_P36	8	128750864	128750864	Missense_Mutation	SNV	C	T	T	MYC	c.401C>T	P.P134L
XH_P36	8	133056743	133056743	Splice_Site	SNV	G	A	A	OC90	c.113-7C>T	
XH_P36	8	133764167	133764167	Missense_Mutation	SNV	T	C	C	TMEM71	c.178A>G	P.T60A
XH_P36	8	139638440	139638440	Missense_Mutation	SNV	C	T	T	COL22A1	c.3710G>A	P.G1237E
XH_P36	8	143956491	143956491	Missense_Mutation	SNV	C	T	T	CYP11B1	c.1280G>A	P.R427H
XH_P36	8	144940503	144940503	Missense_Mutation	SNV	C	T	T	EPPK1	c.6919G>A	P.V2307I
XH_P36	8	144998402	144998402	Missense_Mutation	SNV	G	A	A	PLEC	c.5776C>T	P.R1926C
XH_P36	8	145650341	145650341	Silent	SNV	G	A	A	VPS28	c.288C>T	P.C96C
XH_P36	8	145693999	145693999	Silent	SNV	G	A	A	KIFC2	c.969G>A	P.R323R
XH_P36	8	145737394	145737394	Missense_Mutation	SNV	G	A	A	RECQL4	c.3293C>T	P.T1098I
XH_P36	8	17402081	17402081	Frame_Shift_Del	INDEL	T	-	-	SLC7A2	c.504delT	P.F168fs
XH_P36	8	21981180	21981180	Missense_Mutation	SNV	G	A	A	HR	c.1897C>T	P.R633W
XH_P36	8	2813125	2813125	Missense_Mutation	SNV	G	A	A	CSMD1	c.9980C>T	P.S3327L
XH_P36	8	2824227	2824227	Missense_Mutation	SNV	T	A	A	CSMD1	c.8965A>T	P.I2989F
XH_P36	8	3226804	3226804	Silent	SNV	C	G	G	CSMD1	c.2871G>C	P.T957T
XH_P36	8	37553551	37553551	Silent	SNV	C	T	T	ZNF703	c.54C>T	P.S18S
XH_P36	8	38186894	38186894	Splice_Site	SNV	A	G	G	WHSC1L1	c.1581+2T>C	
XH_P36	8	38874860	38874860	Missense_Mutation	SNV	C	A	A	ADAM9	c.533C>A	P.S178Y
XH_P36	8	42040260	42040260	Silent	SNV	G	A	A	PLAT	c.780C>T	P.G260G
XH_P36	8	61736495	61736495	Missense_Mutation	SNV	C	T	T	CHD7	c.3298C>T	P.R1100C
XH_P36	8	6289099	6289099	Frame_Shift_Del	INDEL	A	-	-	MCPH1	c.321delA	P.K107fs
XH_P36	8	65517426	65517426	Splice_Site	SNV	A	C	C	CYP7B1	c.1058-12T>G	
XH_P36	8	67417717	67417717	Silent	SNV	C	T	T	C8orf46	c.234C>T	P.T78T
XH_P36	8	68151020	68151020	Missense_Mutation	SNV	T	C	C	ARFGEF1	c.3088A>G	P.I1030V
XH_P36	8	77619814	77619814	Missense_Mutation	SNV	C	T	T	ZFHX4	c.2624C>T	P.A875V
XH_P36	8	82588523	82588523	Splice_Site	SNV	A	T	T	IMPA1	c.480-9T>A	
XH_P36	8	92030736	92030736	Missense_Mutation	SNV	C	T	T	TMEM55A	c.368G>A	P.R123Q
XH_P36	8	9609110	9609110	Splice_Site	INDEL	T	-	-	TNKS	c.2833-3delT	
XH_P36	8	99146236	99146236	Silent	SNV	G	A	A	POP1	c.795G>A	P.A265A
XH_P36	8	9953753	9953753	Splice_Site	SNV	G	A	A	MSRA	c.-131-1G>A	
XH_P36	9	105340676	105340676	Splice_Site	SNV	C	T	T	LINC00587	n.196C>T	
XH_P36	9	106885379	106885379	Splice_Site	INDEL	T	-	-	SMC2	c.2133-6delT	
XH_P36	9	113192205	113192205	Silent	SNV	T	C	C	SVEP1	c.5610A>G	P.T1870T
XH_P36	9	116276868	116276868	Silent	SNV	G	A	A	RGS3	c.1278G>A	P.T426T
XH_P36	9	117139586	117139586	Missense_Mutation	SNV	C	A	A	AKNA	c.501G>T	P.Q167H
XH_P36	9	117848395	117848395	Nonsense_Mutation	SNV	G	A	A	TNC	c.1615C>T	P.Q539*
XH_P36	9	124089770	124089770	Splice_Site	SNV	G	A	A	GSN	c.1915+10G>A	
XH_P36	9	124545797	124545797	Splice_Site	INDEL	T	-	-	DAB2IP	c.3390-11delT	
XH_P36	9	127920568	127920568	Nonsense_Mutation	SNV	G	A	A	PPP6C	c.442C>T	P.R148*
XH_P36	9	128691915	128691915	Splice_Site	INDEL	T	-	-	PBX3	c.292-11delT	
XH_P36	9	130947831	130947831	Nonsense_Mutation	SNV	G	A	A	CIZ1	c.583C>T	P.R195*
XH_P36	9	131483691	131483691	Missense_Mutation	SNV	C	T	T	ZDHHC12	c.601G>A	P.V201I
XH_P36	9	131709317	131709317	Missense_Mutation	SNV	G	T	T	DOLK	c.266C>A	P.P89H
XH_P36	9	133231492	133231492	Missense_Mutation	SNV	G	A	A	HMCN2	c.3599G>A	P.R1200H
XH_P36	9	133269250	133269250	Frame_Shift_Ins	INDEL	-	G	G	HMCN2	c.8686dupG	P.E2896fs
XH_P36	9	133281350	133281350	Silent	SNV	C	T	T	HMCN2	c.10674C>T	P.D3558D
XH_P36	9	135271808	135271808	Splice_Site	INDEL	-	T	T	TTF1	c.311+11dupA	
XH_P36	9	135534128	135534128	Splice_Site	INDEL	A	-	-	DDX31	c.839-6delT	
XH_P36	9	135926232	135926232	Missense_Mutation	SNV	G	A	A	GTF3C5	c.635G>A	P.R212H
XH_P36	9	137801701	137801701	Silent	SNV	C	T	T	FCN1	c.924G>A	P.A308A
XH_P36	9	138395457	138395457	Silent	SNV	G	A	A	MRPS2	c.369G>A	P.T123T
XH_P36	9	139371084	139371084	Silent	SNV	C	T	T	SEC16A	c.984G>A	P.R328R
XH_P36	9	139996032	139996032	Missense_Mutation	SNV	C	T	T	MAN1B1	c.1162C>T	P.R388W
XH_P36	9	140001244	140001244	Missense_Mutation	SNV	C	A	A	MAN1B1	c.1549C>A	P.R517S
XH_P36	9	140081973	140081973	Missense_Mutation	SNV	G	A	A	ANAPC2	c.700C>T	P.R234C
XH_P36	9	140126103	140126103	Splice_Site	INDEL	-	C	C	SLC34A3	c.-39-6dupC	
XH_P36	9	140140197	140140197	Silent	SNV	C	T	T	FAM166A	c.165G>A	P.V55V
XH_P36	9	140732961	140732961	Splice_Site	INDEL	C	-	-	MIR602	n.96delC	
XH_P36	9	18906821	18906821	Missense_Mutation	SNV	G	A	A	ADAMTSL1	c.5093G>A	P.R1698H
XH_P36	9	19300171	19300171	Splice_Site	INDEL	T	-	-	DENND4C	n.1253-7delT	
XH_P36	9	2047307	2047307	Missense_Mutation	SNV	C	T	T	SMARCA2	c.869C>T	P.A290V
XH_P36	9	26906080	26906081	Splice_Site	INDEL	AA	-	-	PLAA	823-7_1823-6delTT	
XH_P36	9	26928300	26928300	Splice_Site	SNV	C	A	A	PLAA	c.444+6G>T	
XH_P36	9	27220063	27220063	Silent	SNV	C	T	T	TEK	c.3120C>T	P.C1040C
XH_P36	9	27548256	27548256	Missense_Mutation	SNV	C	T	T	C9orf72	c.1424G>A	P.R475Q
XH_P36	9	2827162	2827162	Splice_Site	INDEL	A	-	-	PUM3	c.957-11delT	
XH_P36	9	35103049	35103049	Splice_Site	SNV	T	G	G	STOML2	c.43A>C	P.R15R
XH_P36	9	36102078	36102078	Splice_Site	INDEL	T	-	-	RECK	c.915-5delT	
XH_P36	9	37733708	37733708	Splice_Site	INDEL	T	-	-	FRMPD1	c.1123-11delT	
XH_P36	9	40772354	40772354	Missense_Mutation	SNV	G	A	A	ZNF658	c.2921C>T	P.T974M
XH_P36	9	5233659	5233659	Missense_Mutation	SNV	G	A	A	INSL4	c.202G>A	P.V68M
XH_P36	9	5742994	5742994	Missense_Mutation	SNV	A	G	G	RIC1	c.1027A>G	P.T343A
XH_P36	9	73399023	73399023	Splice_Site	SNV	G	A	A	TRPM3	c.687C>T	P.G229G
XH_P36	9	73458046	73458046	Splice_Site	INDEL	A	-	-	TRPM3	c.218-3delT	
XH_P36	9	84606936	84606936	Silent	SNV	T	C	C	SPATA31D1	c.1551T>C	P.P517P
XH_P36	9	8524968	8524968	Silent	SNV	G	A	A	PTPRD	c.636C>T	P.S212S
XH_P36	9	86503511	86503511	Splice_Site	INDEL	A	-	-	KIF27	c.1980-4delT	
XH_P36	9	90582462	90582462	Frame_Shift_Ins	INDEL	-	G	G	CDK20	c.955dupC	P.H319fs
XH_P36	9	96422612	96422612	Frame_Shift_Del	INDEL	A	-	-	PHF2	c.1475delA	P.K492fs
XH_P36	9	977357	977357	Missense_Mutation	SNV	A	C	C	DMRT3	c.356A>C	P.Q119P
XH_P36	9	99381507	99381507	Missense_Mutation	SNV	T	C	C	CDC14B	c.154A>G	P.I52V
XH_P36	X	100503224	100503224	Missense_Mutation	SNV	A	G	G	DRP2	c.1165A>G	P.N389D
XH_P36	X	101970716	101970716	Missense_Mutation	SNV	A	G	G	GPRASP2	c.919A>G	P.R307G
XH_P36	X	105450411	105450411	Missense_Mutation	SNV	A	T	T	MUM1L1	c.986A>T	P.D329V
XH_P36	X	106845186	106845186	Frame_Shift_Del	INDEL	G	-	-	FRMPD3	c.4022delG	P.G1341fs
XH_P36	X	114347904	114347904	Splice_Site	INDEL	-	A	A	LRCH2	c.2128-7dupT	
XH_P36	X	114424574	114424574	Silent	SNV	C	A	A	RBMXL3	c.570C>A	P.G190G
XH_P36	X	117530913	117530914	Splice_Site	INDEL	TT	-	-	WDR44	)54-10_1054-9delTT	
XH_P36	X	118920652	118920652	Missense_Mutation	SNV	G	T	T	RPL39	c.151C>A	P.L51I
XH_P36	X	123514660	123514660	Missense_Mutation	SNV	T	C	C	TENM1	c.7925A>G	P.Y2642C
XH_P36	X	123663816	123663816	Missense_Mutation	SNV	C	T	T	TENM1	c.2669G>A	P.R890Q
XH_P36	X	125685317	125685317	Missense_Mutation	SNV	T	G	G	DCAF12L1	c.1275A>C	P.E425D
XH_P36	X	12904643	12904643	Frame_Shift_Ins	INDEL	-	C	C	TLR7	c.1020dupC	P.S341fs
XH_P36	X	129147932	129147932	Missense_Mutation	SNV	C	A	A	BCORL1	c.1184C>A	P.T395K
XH_P36	X	129270637	129270637	Missense_Mutation	SNV	T	C	C	AIFM1	c.128A>G	P.K43R
XH_P36	X	14599388	14599388	Frame_Shift_Ins	INDEL	-	T	T	GLRA2	c.356dupT	P.L119fs

XH_P36	X	147003514	147003514	Splice_Site	SNV	C	T	T	FMR1	c.104+11C>T	
XH_P36	X	148627313	148627313	Missense_Mutation	SNV	G	T	T	CXorf40A	c.137G>T	P.R46M
XH_P36	X	148794840	148794840	Frame_Shift_Ins	INDEL	-	G	G	MAGEA11	c.26dupG	P.L10fs
XH_P36	X	151808938	151808938	Splice_Site	SNV	T	A	A	GABRQ	c.238+11T>A	
XH_P36	X	152686163	152686163	Frame_Shift_Del	INDEL	G	-	-	ZFP92	c.331delG	P.A111fs
XH_P36	X	153178731	153178731	Missense_Mutation	SNV	C	T	T	ARHGAP4	c.1352G>A	P.R451Q
XH_P36	X	153692348	153692348	Missense_Mutation	SNV	G	T	T	PLXNA3	c.1602G>T	P.E534D
XH_P36	X	153944567	153944567	Missense_Mutation	SNV	C	T	T	GAB3	c.110G>A	P.R37H
XH_P36	X	17750296	17750296	Silent	SNV	C	T	T	NHS	c.4137C>T	P.S1379S
XH_P36	X	22056595	22056595	Missense_Mutation	SNV	G	T	T	PHEX	c.127G>T	P.G43C
XH_P36	X	22292144	22292144	Frame_Shift_Ins	INDEL	-	A	A	ZNF645	c.1040dupA	P.N347fs
XH_P36	X	23397692	23397692	Splice_Site	INDEL	T	-	-	PTCHD1	c.352-10delT	
XH_P36	X	23410920	23410920	Missense_Mutation	SNV	G	T	T	PTCHD1	c.1285G>T	P.G429C
XH_P36	X	2773043	2773043	Missense_Mutation	SNV	A	G	G	GYG2	c.334A>G	P.N112D
XH_P36	X	30327013	30327013	Silent	SNV	C	T	T	NR0B1	c.468G>A	P.T156T
XH_P36	X	30877624	30877624	Missense_Mutation	SNV	C	T	T	TAB3	c.82G>A	P.V28M
XH_P36	X	32366648	32366648	Splice_Site	INDEL	A	-	-	DMD	c.5302-3delT	
XH_P36	X	36321999	36321999	Missense_Mutation	SNV	C	T	T	CFAP47	c.8006C>T	P.T2669M
XH_P36	X	37028698	37028698	Missense_Mutation	SNV	C	T	T	FAM47C	c.2215C>T	P.R739C
XH_P36	X	47516631	47516631	Silent	SNV	G	A	A	UXT	c.307C>T	P.L103L
XH_P36	X	53115191	53115191	Silent	SNV	C	T	T	TSPYL2	c.1617C>T	P.Y539Y
XH_P36	X	53564586	53564586	Missense_Mutation	SNV	C	T	T	HUWE1	c.12068G>A	P.R4023H
XH_P36	X	54569370	54569370	Splice_Site	INDEL	T	-	-	GNL3L	c.307-10delT	
XH_P36	X	65822649	65822649	Splice_Site	INDEL	A	-	-	EDA2R	c.353-10delT	
XH_P36	X	7023887	7023887	Splice_Site	INDEL	A	-	-	PUDP	c.131-8delT	
XH_P36	X	70586340	70586340	Missense_Mutation	SNV	A	T	T	TAF1	c.176A>T	P.D59V
XH_P36	X	74318786	74318786	Silent	SNV	A	G	G	ABCB7	c.444T>C	P.G148G
XH_P36	X	76845413	76845413	Splice_Site	INDEL	A	-	-	ATRX	c.6111-3delT	
XH_P36	X	77224989	77224989	Silent	SNV	G	T	T	PGAM4	c.147C>A	P.G49G
XH_P36	X	77268382	77268382	Missense_Mutation	SNV	G	A	A	ATP7A	c.2179G>A	P.G727R
XH_P36	X	79279670	79279670	Splice_Site	SNV	G	T	T	TBX22	c.458+7G>T	
XH_P36	X	82763673	82763673	Frame_Shift_Del	INDEL	G	-	-	POU3F4	c.346delG	P.A116fs
XH_P36	X	95993734	95993734	Missense_Mutation	SNV	A	T	T	DIAPH2	c.315A>T	P.E105D
XH_P37	12	25398284	25398284	Missense_Mutation	SNP	C	T	T	KRAS	c.35G>A	p.G12D
XH_P37	1	78426078	78426078	Missense_Mutation	SNP	G	T	T	FUBP1	c.1447C>A	p.P483T
XH_P37	1	977395	977395	Missense_Mutation	SNP	C	A	C	AGRN	c.C1237A	p.R413S
XH_P37	1	1858140	1858140	Frame_Shift_Ins	INS	-	TCTCCCGAGG	-	CFAP74	_3723insCCTCG	p.V1241fs
XH_P37	1	3742991	3742991	Nonsense_Mutation	INS	-	TAACTTAAAATA	-	CEP104	AAAGGATATTTT	G733delinsAERIFX
XH_P37	1	4772675	4772675	In_Frame_Ins	INS	-	ATGAACTCA	-	AJAP1	5_746insATGAA	.G249delinsDELR
XH_P37	1	5993378	5993378	Silent	SNP	G	A	G	NPHP4	c.C1131T	p.V377V
XH_P37	1	5993379	5993379	Nonsense_Mutation	INS	-	TAGGTATTTCTT	-	NPHP4	ATGCATAAGAA	377_T378delinsDAX
XH_P37	1	6712973	6712973	Frame_Shift_Ins	INS	-	ACAACTCAGCCA	-	DNAJC11	TCTATTGGCTG	p.G182fs
XH_P37	1	7798488	7798488	Frame_Shift_Ins	INS	-	AGAGGCT	-	CAMTA1	57_1258insAGA	p.S419fs
XH_P37	1	9796012	9796012	Silent	SNP	G	A	G	CLSTN1	c.C1608T	p.I536I
XH_P37	1	10357279	10357279	Frame_Shift_Ins	INS	-	AGGAG	-	KIF1B	952_1953insAG	p.D651fs
XH_P37	1	10468171	10468171	Frame_Shift_Ins	INS	-	CTAA	-	PGD	.427_428insCTA	p.G143fs
XH_P37	1	10468173	10468173	Frame_Shift_Ins	INS	-	CTACAGGT	-	PGD	9_430insCTACA	p.G143fs
XH_P37	1	12837291	12837291	Missense_Mutation	SNP	T	C	T	PRAMEF12	c.T1001C	p.F334S
XH_P37	1	14108446	14108446	Nonsense_Mutation	INS	-	AAGCTTGGGTA	-	PRDM2	554insAAAGCT	1185_D1186delinsX
XH_P37	1	15382667	15382667	Silent	SNP	T	C	T	KAZN	c.T525C	p.N175N
XH_P37	1	15495247	15495247	Frame_Shift_Ins	INS	-	ACAGC	-	C1orf195	124_125insGCT	p.V42fs
XH_P37	1	15987798	15987798	Nonsense_Mutation	INS	-	TTCTGATTGACA	-	RSC1A1	insTGGTTTCTGA	9_T480delinsMVSDX
XH_P37	1	16134066	16134066	Frame_Shift_Ins	INS	-	AACTTGAAATA	-	UQCRHL	ACATTTATTTCA	p.V27fs
XH_P37	1	16245978	16245978	Missense_Mutation	SNP	C	T	C	SPEN	c.C1601T	p.T534I
XH_P37	1	16245980	16245980	Missense_Mutation	SNP	C	G	C	SPEN	c.C1603G	p.R535G
XH_P37	1	16641752	16641752	Nonsense_Mutation	INS	-	CTCCCTTAAATG	-	FBXO42	AATACATTTAAG	V54_L55delinsVX
XH_P37	1	17084504	17084504	Missense_Mutation	SNP	G	A	G	MST1L	c.C1594T	p.R532W
XH_P37	1	17084505	17084505	Missense_Mutation	SNP	C	G	C	MST1L	c.G1593C	p.Q531H
XH_P37	1	19524505	19524505	Nonsense_Mutation	INS	-	CGGAGAAGCTA	-	UBR4	687insGTAGCTT	p.Q229delinsQX
XH_P37	1	19524507	19524507	Frame_Shift_Ins	INS	-	CGTGT	-	UBR4	684_685insACA	p.Q229fs
XH_P37	1	20020998	20020998	In_Frame_Ins	INS	-	AAAGAT	-	TMCO4	428_1429insAT	p.L477delinsIFL
XH_P37	1	20020999	20020999	Frame_Shift_Ins	INS	-	ATAAGAATTTAT	-	TMCO4	1428insAATAAA	p.Q476fs
XH_P37	1	22175460	22175460	Frame_Shift_Ins	INS	-	CATCACCT	-	HSPG2	3_7514insAGGT	p.R2505fs
XH_P37	1	22198813	22198813	Nonsense_Mutation	SNP	G	A	G	HSPG2	c.C4090T	p.R1364X
XH_P37	1	23520411	23520411	Nonsense_Mutation	INS	-	GCCTTCTACA	-	HTR1D	_302insTGTAGA	101_H102delinsMX
XH_P37	1	23885748	23885748	Frame_Shift_Ins	INS	-	TTCCCTCTTTTCC	-	ID3	70insAGGAAAAG	p.G57fs
XH_P37	1	24020359	24020359	Frame_Shift_Ins	INS	-	ATTTTCCCAT	-	RPL11	0_221insATTTTC	p.V74fs
XH_P37	1	24997974	24997974	Frame_Shift_Ins	INS	-	GTATTCAAAAGT	-	SRRM1	9insAAGTATTCA	p.K833fs
XH_P37	1	26751971	26751971	Frame_Shift_Ins	INS	-	AGGAACTCTTAT	-	LIN28A	nsACAAGGAACT	p.G136fs
XH_P37	1	27684800	27684800	Frame_Shift_Ins	INS	-	ACTCTAACTTG	-	MAP3K6	_2763insCAAGT	p.S921fs
XH_P37	1	28320072	28320072	Splice_Site	INS	-	TTTTA	-	EYA3	.	.
XH_P37	1	28477055	28477055	Nonsense_Mutation	INS	-	CTGTTTCCTCAC	-	PTAFR	TCTTGGTGAGGA	160delinsFLVRKQX
XH_P37	1	29314413	29314413	Frame_Shift_Ins	INS	-	GGAGAATAGTC	-	EPB41	insGAGGAGAAT	p.T155fs
XH_P37	1	31414971	31414971	Splice_Site	INS	-	GAATCTTGTGGT	-	PUM1	.	.
XH_P37	1	31532354	31532354	In_Frame_Ins	INS	-	CTACTTCTTTCAG	-	PUM1	insTCTGAAAGA	P20delinsPLKEVV
XH_P37	1	32118445	32118445	Frame_Shift_Ins	INS	-	T	-	COL16A1	c.4621_4622ins	p.G1541fs
XH_P37	1	32118446	32118446	In_Frame_Ins	INS	-	AGGTTGTGAAT	-	COL16A1	AAAAAACATTCA	41delinsKKHSQPVTG
XH_P37	1	32154468	32154468	Nonsense_Mutation	INS	-	ACATAATTCCTAT	-	COL16A1	761insATAGGA	587_L588delinsGX
XH_P37	1	32790122	32790122	In_Frame_Ins	INS	-	CTGCATCAAGTT	-	HDAC1	324insCTGCATC	.E108delinsDCIKL
XH_P37	1	34023424	34023424	Missense_Mutation	SNP	G	A	G	CSMD2	c.C8653T	p.R2885W
XH_P37	1	35480736	35480736	Frame_Shift_Ins	INS	-	TGTATGTGTGTG	-	ZMYM6	insAAACACACA	p.I152fs
XH_P37	1	35579116	35579116	Frame_Shift_Ins	INS	-	AACAACTGGCAT	-	ZMYM1	GGCAGAACAACT	p.L487fs
XH_P37	1	36028099	36028099	Frame_Shift_Ins	INS	-	ATAATACAACTA	-	NCDN	nsAGAAATAATA	p.L400fs
XH_P37	1	36215412	36215412	Frame_Shift_Ins	INS	-	A	-	CLSPN	c.1836_1837ins	p.T613fs
XH_P37	1	36215413	36215413	Splice_Site	INS	-	GGTAAG	-	CLSPN	.	.
XH_P37	1	36299771	36299771	Frame_Shift_Ins	INS	-	TTTTTCATTTCAG	-	AGO4	ACGGTTTTTTCA	p.V520fs
XH_P37	1	38338723	38338723	Nonsense_Mutation	INS	-	ATGCCTACC	-	INPP5B	3_1574insGGTA	p.K525delinsRX
XH_P37	1	39341321	39341321	Frame_Shift_Ins	INS	-	GCAATCTATAGC	-	GJA9	ATAGTGGCTATA	p.R150fs
XH_P37	1	39816687	39816687	Nonsense_Mutation	INS	-	CCTGTAAATCCT	-	MACF1	TGTCATCCTGTA	670delinsECHPVNPX
XH_P37	1	39877142	39877142	Nonsense_Mutation	INS	-	TCCATCATA	-	KIAA0754	5_1206insTCCAT	.P402delinsPPSX
XH_P37	1	39877144	39877144	Missense_Mutation	SNP	G	T	G	KIAA0754	c.G1207T	p.A403S
XH_P37	1	40704533	40704533	Frame_Shift_Ins	INS	-	CTGCCTATTGGT	-	RLF	TATGGCTGCCTA	p.H1387fs
XH_P37	1	40960979	40960979	In_Frame_Ins	INS	-	GAGACTTTTCTC	-	ZFP69	833insGAGACTT	E278delinsGDFSQ
XH_P37	1	40960981	40960981	Frame_Shift_Ins	INS	-	CGTGGAAT	-	ZFP69	4_835insCGTGG	p.E278fs
XH_P37	1	41012624	41012624	In_Frame_Ins	INS	-	GCG	-	ZNF684	c.629_630insGC	p.N210delinsKR
XH_P37	1	41012625	41012625	Nonsense_Mutation	INS	-	TCTATTATATTTA	-	ZNF684	TAACTTCTATTA	G211delinsNFLQYNFYYIX
XH_P37	1	41514547	41514547	In_Frame_Ins	INS	-	AAGGCA	-	SCMH1	616_617insTGCC	.N206delinsMPY
XH_P37	1	42046033	42046033	Frame_Shift_Ins	INS	-	GTCAGAAACG	-	HIVEP3	_4436insCGTTT	p.K1479fs

XH_P37	1	43055136	43055136	Frame_Shift_Ins	INS	-	GTATGTGTCTAT	-	CCDC30	sGTAGGGTATGT	p.E455fs
XH_P37	1	43896275	43896275	In_Frame_Ins	INS	-	TGAAAACTCCAG	-	SZT2	4419insTGAAAA	1473delinsSENSR
XH_P37	1	43903330	43903330	Frame_Shift_Ins	INS	-	TGGTAAACAAAC	-	SZT2	185insCTGGTAA	p.L2062fs
XH_P37	1	45292590	45292590	Frame_Shift_Ins	INS	-	TTCCTCTT	-	PTCH2	8_2679insAAGA	p.T893fs
XH_P37	1	45292591	45292591	Missense_Mutation	SNP	G	C	G	PTCH2	c.C2678G	p.T893R
XH_P37	1	45478986	45478986	Frame_Shift_Ins	INS	-	GTTTTATCTAGAT	-	UROD	237insGTTTTATC	p.D79fs
XH_P37	1	45484528	45484528	In_Frame_Ins	INS	-	TCGCCACTATGG	-	ZSWIM5	3156insCCATAG	1052delinsLHSGE
XH_P37	1	46070690	46070690	Splice_Site	INS	-	AAGTGACATTAA	-	NASP	.	.
XH_P37	1	46543274	46543274	Nonsense_Mutation	INS	-	TTTCCAGTCTGGC	-	C110117498-PIK	AATTAGCCAGAC	22_D123delinsILNVX
XH_P37	1	47110905	47110905	Silent	SNP	C	T	C	ATPAF1	c.G348A	p.R116R
XH_P37	1	47110907	47110907	In_Frame_Ins	INS	-	ACTACTTCTGGT	-	ATPAF1	6insGAACCAGA	R116delinsEPEVVR
XH_P37	1	47746544	47746544	Nonsense_Mutation	INS	-	AAGCCCTTCAAA	-	STIL	AATGCTTTTGAA	.G529delinsECFX
XH_P37	1	48460195	48460195	Frame_Shift_Ins	INS	-	CTATGCTGACTAC	-	TRABD2B	insAGGTAGTCAC	p.V59fs
XH_P37	1	52442702	52442702	Frame_Shift_Ins	INS	-	TCCAGGAGGTTT	-	RAB3B	ACATAAAACCTCC	p.N30fs
XH_P37	1	52891135	52891135	Frame_Shift_Ins	INS	-	ACAAAATCTCCA	-	ZCCHC11	AAGTAATGGAGA	p.A1586fs
XH_P37	1	53504718	53504718	Missense_Mutation	SNP	G	A	G	SCP2	c.G256A	p.D86N
XH_P37	1	53504719	53504719	Splice_Site	INS	-	TGTTTAAATAGAT	-	SCP2	.	.
XH_P37	1	55080512	55080512	Frame_Shift_Ins	INS	-	CC	-	FAM151A	c.435_436insGG	p.N146fs
XH_P37	1	55080514	55080514	Frame_Shift_Ins	INS	-	CTTCCTCCAA	-	FAM151A	_434insTTGGAG	p.K145fs
XH_P37	1	56977824	56977824	Frame_Shift_Ins	INS	-	TAAGTAGCAT	-	PLPP3	_634insATGCTA	p.L212fs
XH_P37	1	57233602	57233602	Frame_Shift_Ins	INS	-	ACCATCTTATTTC	-	C1orf168	insAAGAAATAAC	p.N321fs
XH_P37	1	58999933	58999933	Nonsense_Mutation	INS	-	TTCACTGGATTTT	-	OMA1	TTTAAAAATCCA	7_C268delinsHYLKIQX
XH_P37	1	59148073	59148073	Nonsense_Mutation	INS	-	CACTGTATATTT	-	MYSM1	AGGATAAATATA	p.E215delinsX
XH_P37	1	60499180	60499180	Missense_Mutation	SNP	G	C	G	C1orf87	c.C997G	p.R333G
XH_P37	1	61554336	61554336	In_Frame_Ins	INS	-	GCTTTTCATGTT	-	NFIA	544insGCTTTTC	Y181delinsYAFHV
XH_P37	1	63097073	63097073	Frame_Shift_Ins	INS	-	TTTTTTGAGATG	-	DOCK7	120insCCATCTC	p.K374fs
XH_P37	1	63999881	63999881	Frame_Shift_Ins	INS	-	CTTCTAATAAGAA	-	EFCAB7	TGGGCTTCTAAT	p.L266fs
XH_P37	1	65858477	65858477	Frame_Shift_Ins	INS	-	TGCTTTTTGCTGC	-	DNAJC6	1833insTGCTTTT	p.A611fs
XH_P37	1	66102089	66102089	Missense_Mutation	SNP	C	G	C	LEPR	c.C2889G	p.N963K
XH_P37	1	66102091	66102091	Frame_Shift_Ins	INS	-	TAAAGAACATAA	-	LEPR	sAATAATAAAGA	p.F964fs
XH_P37	1	67359077	67359077	Frame_Shift_Ins	INS	-	TTTCTTTCTTTCT	-	WDR78	AGAAAGAAAGA	p.T122fs
XH_P37	1	67558986	67558986	In_Frame_Ins	INS	-	TGCCTCATAAAAT	-	C1orf141	ATAATATTTTATC	delinsIAAYIIFYEAFKISFD
XH_P37	1	68624857	68624857	In_Frame_Ins	INS	-	TTTCCT	-	WLS	79_180insAGGA	p.M60delinsIGK
XH_P37	1	68624858	68624858	Frame_Shift_Ins	INS	-	ACTTTGC	-	WLS	78_179insGCAA	p.M60fs
XH_P37	1	68947873	68947873	Nonsense_Mutation	INS	-	AGTTGCTTAATTT	-	DEPDC1	18insAAAAATTA	540_G541delinsKNX
XH_P37	1	70226052	70226052	Nonsense_Mutation	INS	-	TTCTTGTAATAT	-	LRRC7	AGGCATTCTTGT	N56delinsALGRHSCNIX
XH_P37	1	70883662	70883662	Frame_Shift_Ins	INS	-	AAAATCT	-	CTH	93_294insAAAA	p.T98fs
XH_P37	1	70883664	70883664	In_Frame_Ins	INS	-	TTTTCT	-	CTH	295_296insTTTT	p.H99delinsLFY
XH_P37	1	71478166	71478166	In_Frame_Ins	INS	-	ATAATGAGATAG	-	PTGER3	99insGCCTATCTC	I300delinsSLSHYL
XH_P37	1	71536613	71536613	Nonsense_Mutation	INS	-	AAATTTAATTCAA	-	ZRANB2	sTGATTTGAATT	p.N194delinsX
XH_P37	1	74670532	74670532	In_Frame_Ins	INS	-	AAA	-	FPGT	.840_841insAA	p.Y280delinsYK
XH_P37	1	74670533	74670533	Frame_Shift_Ins	INS	-	TTCAATATCA	-	FPGT	_842insTTCAAT	p.V281fs
XH_P37	1	76878101	76878101	Frame_Shift_Ins	INS	-	CAACTCGAATCAT	-	ST6GALNAC3	428insCAACTCGA	p.R143fs
XH_P37	1	77620307	77620307	Splice_Site	INS	-	AAGAGGCAAAAC	-	PIGK	.	.
XH_P37	1	77629619	77629619	Nonsense_Mutation	INS	-	GTTTTTAAAAGA	-	PIGK	GTTTTTCTTTTAA	_N167delinsNSQFFFX
XH_P37	1	78958788	78958788	In_Frame_Ins	INS	-	GCAGGTTGTGTT	-	PTGFR	361insGCAGGTT	L120delinsLAGCV
XH_P37	1	85589805	85589805	Silent	SNP	A	T	A	WDR63	c.A1866T	p.S622S
XH_P37	1	86123593	86123593	Nonsense_Mutation	INS	-	TCTAAGAGCGTA	-	ZNHIT6	AGATTATACGCT	37_Y438delinsKIIRSX
XH_P37	1	86437068	86437068	In_Frame_Ins	INS	-	GTT	-	COL24A1	.272_273insAAC	p.L91delinsLT
XH_P37	1	86437069	86437069	Nonsense_Mutation	INS	-	TTATATATTTAGA	-	COL24A1	CTCTGTCTAAATA	1_G92delinsQDSVX
XH_P37	1	87031076	87031076	Frame_Shift_Ins	INS	-	CTATGGTACCATC	-	CLCA4	678insCTATGGTA	p.F226fs
XH_P37	1	87031078	87031078	Missense_Mutation	SNP	C	A	C	CLCA4	c.C679A	p.P227T
XH_P37	1	89846188	89846188	In_Frame_Ins	INS	-	ATGTTTGAACTC	-	GBP6	ATTGGATGTTTG	L60delinsKIGCLNSGI
XH_P37	1	90178875	90178875	Missense_Mutation	SNP	G	T	G	LRRC8C	c.G746T	p.R249M
XH_P37	1	90178876	90178876	In_Frame_Ins	INS	-	TTGTTCTTC	-	LRRC8C	7_748insTTGTTC	.R249delinsRLFF
XH_P37	1	90493113	90493113	Frame_Shift_Ins	INS	-	TTTTACTTGCC	-	ZNF326	_985insTTTTACT	p.N328fs
XH_P37	1	91406594	91406594	Frame_Shift_Ins	INS	-	TTGTTATCAGATC	-	ZNF644	sCTACATCTGAT	p.E106fs
XH_P37	1	91978805	91978805	Nonsense_Mutation	INS	-	GTAACATTGTAAT	-	CDC7	64insAGTAACAT	p.S255delinsX
XH_P37	1	92446722	92446722	In_Frame_Ins	INS	-	AACTGTTGTTTC	-	BRDT	0insATGAACTGT	533delinsPMNCCFL
XH_P37	1	92642493	92642493	In_Frame_Ins	INS	-	ATATTTTATGTTT	-	KIAA1107	ATATAATATTTTA	delinsSLTYNILCFQKP
XH_P37	1	92642755	92642755	Frame_Shift_Ins	INS	-	TTTATACTATTTCT	-	KIAA1107	TTTTATTTATACT	p.A156fs
XH_P37	1	92646066	92646066	Nonsense_Mutation	INS	-	GAGACTGAAGAT	-	KIAA1107	nsTGAAGAGACT	429_P430delinsPX
XH_P37	1	92944237	92944237	Frame_Shift_Ins	INS	-	GTTATTCTCTGCT	-	GFI1	98insGAGCAGAC	p.I333fs
XH_P37	1	93575967	93575967	Frame_Shift_Ins	INS	-	A	-	MTF2	c.186_187insA	p.Y62fs
XH_P37	1	93575968	93575968	Nonsense_Mutation	INS	-	TATTTTATATGTT	-	MTF2	GGGATATTTTAT	63delinsPVGYFICX
XH_P37	1	93736231	93736231	Frame_Shift_Ins	INS	-	AAATTTTGGATCT	-	CCDC18	1insGCAAATTTT	p.K1404fs
XH_P37	1	94645455	94645455	Frame_Shift_Ins	INS	-	AAAG	-	ARHGAP29	2113_2114insCT	p.E705fs
XH_P37	1	94645456	94645456	Frame_Shift_Ins	INS	-	TAGAACATTTAT	-	ARHGAP29	CCATTAATAAAT	p.E705fs
XH_P37	1	95712410	95712410	Frame_Shift_Ins	INS	-	ATTATACCTTTTG	-	RWDD3	9insAAATTATAC	p.L263fs
XH_P37	1	98039406	98039406	In_Frame_Ins	INS	-	AGTTAAGAAGTA	-	DPYD	TGCTTTTACTTCT	17delinsCFYFLTRRG
XH_P37	1	98165039	98165039	Nonsense_Mutation	INS	-	GCTCACAGATGG	-	DPYD	548insACCATCTC	83_E184delinsYHLX
XH_P37	1	100214245	100214245	Frame_Shift_Ins	INS	-	AGTAGTCTGTGT	-	FRRS1	0insTACACAGAC	p.K27fs
XH_P37	1	100480954	100480954	In_Frame_Ins	INS	-	CCAAATAATTTT	-	SLC35A3	insATTCCAAATA	T286delinsTFQIILH
XH_P37	1	100598841	100598841	Missense_Mutation	SNP	A	T	A	TRMT13	c.A117T	p.R39S
XH_P37	1	100598842	100598842	Nonsense_Mutation	INS	-	AGAAGC	-	TRMT13	18_119insAGAA	p.F40delinsX
XH_P37	1	100949886	100949886	Missense_Mutation	SNP	G	A	G	CDC14A	c.G137A	p.R46Q
XH_P37	1	104118078	104118078	Nonsense_Mutation	INS	-	TAAATTCTTTCTC	-	AMY2B	AAGATTAAATTC	39_V340delinsASEDX
XH_P37	1	104118080	104118080	Frame_Shift_Ins	INS	-	TATTC	-	AMY2B	019_1020insTAT	p.V340fs
XH_P37	1	109241942	109241942	Nonsense_Mutation	INS	-	AATAAAACATAT	-	PRPF38B	CAATGAATAAAA	9_R170delinsENFNEX
XH_P37	1	109268510	109268510	Missense_Mutation	SNP	C	A	C	FNDC7	c.C995A	p.T332K
XH_P37	1	109268512	109268512	Nonsense_Mutation	INS	-	TTTAATGAC	-	FNDC7	7_998insTTTAAT	p.S333delinsFX
XH_P37	1	109493048	109493048	Frame_Shift_Ins	INS	-	ATGATTTTAGTTC	-	CLCC1	CTAAGAACTAAA	p.S4fs
XH_P37	1	110022151	110022151	Nonsense_Mutation	INS	-	AATTTGTGAGAA	-	SYPL2	01insGAATTTGT	67_A268delinsGNLX
XH_P37	1	110051264	110051264	Nonsense_Mutation	INS	-	AAACTCTACTT	-	AMIGO1	_271insAAGTAG	.L91_L92delinsKX
XH_P37	1	112042935	112042935	In_Frame_Ins	INS	-	CATCATCTACTTT	-	ADORA3	CATTAAAGTAGA	98delinsLTIKVDDAVD
XH_P37	1	112308541	112308541	Nonsense_Mutation	INS	-	GTAAGACCCATCC	-	DDX20	496insTGTAAGA	p.N499delinsMX
XH_P37	1	112318853	112318853	Nonsense_Mutation	INS	-	CTATATACAACTT	-	KCND3	ATTACCAAGTTGT	586delinsYQKLPSCIX
XH_P37	1	112999050	112999050	Frame_Shift_Ins	INS	-	GTTGTTCCCTTTC	-	CTTNBP2NL	TCTCAGTTGTTCC	p.C312fs
XH_P37	1	114165602	114165602	Frame_Shift_Ins	INS	-	CTCTTGTGAAGT	-	MAGI3	47insATCTCTTGT	p.G449fs
XH_P37	1	114680299	114680299	Missense_Mutation	SNP	T	A	T	SYT6	c.A634T	p.N212Y
XH_P37	1	114680300	114680300	Frame_Shift_Ins	INS	-	AGCCCTGT	-	SYT6	2_633insACAGG	p.E211fs
XH_P37	1	114968344	114968344	Frame_Shift_Ins	INS	-	ATAGAAATTATGA	-	TRIM33	2insGCTTCATAAT	p.G474fs
XH_P37	1	120296007	120296007	Nonsense_Mutation	INS	-	TTATTATTAATAG	-	HMGCS2	nsTTTGCTATTAA	.H355delinsLCYX
XH_P37	1	120387075	120387075	Nonsense_Mutation	INS	-	ACTAAATGCTCT	-	NBPF7	84insAGAGCATT	p.W28delinsX
XH_P37	1	120438805	120438805	Missense_Mutation	SNP	C	A	C	ADAM30	c.G155T	p.R52L
XH_P37	1	120438806	120438806	Frame_Shift_Ins	INS	-	TTTTGAACAT	-	ADAM30	_154insATGTTC	p.R52fs
XH_P37	1	120468290	120468290	Nonsense_Mutation	INS	-	ATTATATCAACAA	-	NOTCH2	149insGTTGTTGA	C1383delinsWLLIX
XH_P37	1	144881520	144881520	Missense_Mutation	SNP	G	A	G	PDE4DIP	c.C4165T	p.R1389C

XH_P37	1	144892529	144892529	Frame_Shift_Ins	INS	-	ACCCACCTTTG	-	PDE4DIP	3365insCAAAG(	p.E1122fs
XH_P37	1	144911948	144911948	In_Frame_Ins	INS	-	CTGAAGAAC	-	PDE4DIP	9_2650insGTTCT	.P884delinsVLQP
XH_P37	1	144911950	144911950	Missense_Mutation	SNP	A	G	A	PDE4DIP	c.T2648C	p.I883T
XH_P37	1	145608291	145608291	Frame_Shift_Ins	INS	-	TTGTGGATTCTT	-	POLR3C	sCAAGAAGAATC	p.G149fs
XH_P37	1	145608636	145608636	Frame_Shift_Ins	INS	-	GCAAACTGACA	-	POLR3C	_210insTGTCAG	p.L70fs
XH_P37	1	145608638	145608638	Missense_Mutation	SNP	G	T	G	POLR3C	c.C208A	p.L70I
XH_P37	1	145650529	145650529	In_Frame_Ins	INS	-	CTATTAAAGGAA	-	RNF115	209insCTATTAA	.H70delinsPIKGN
XH_P37	1	147092428	147092428	Nonsense_Mutation	INS	-	TCTTCTGAGGT	-	BCL9	_2468insTCTTCT	823_P824delinsIFX
XH_P37	1	149783767	149783767	Frame_Shift_Ins	INS	-	GAGTGTGCAAG	-	HIST2H2BF	112insCCTTGCA	p.Y38fs
XH_P37	1	150936029	150936029	Frame_Shift_Ins	INS	-	TTTTTTTTCAAG	-	SETDB1	82insGTTTTTTT	p.V1161fs
XH_P37	1	152127601	152127601	In_Frame_Ins	INS	-	TCAGCCCCTCAC(	-	RPTN	74insTGGTGAG(	I658delinsHGEGLS
XH_P37	1	152187559	152187559	In_Frame_Ins	INS	-	CTAGGA	-	HRNR	545_6546insTCC	.S2182delinsSPS
XH_P37	1	152191626	152191626	In_Frame_Ins	INS	-	CGAGGA	-	HRNR	478_2479insTCC	.G827delinsSSG
XH_P37	1	152192114	152192114	Missense_Mutation	SNP	C	T	C	HRNR	c.G1991A	p.R664Q
XH_P37	1	152192683	152192683	Missense_Mutation	SNP	A	C	A	HRNR	c.T1422G	p.H474Q
XH_P37	1	152193659	152193659	Frame_Shift_Ins	INS	-	CAGTCAGCATGC	-	HRNR	46insTCCATGCT	p.K149fs
XH_P37	1	152280355	152280355	In_Frame_Ins	INS	-	GGA	-	FLG	7006_7007insT(	.Q2336delinsLQ
XH_P37	1	152281490	152281490	Missense_Mutation	SNP	C	T	C	FLG	c.G5872A	p.G1958R
XH_P37	1	152281491	152281491	Silent	SNP	A	G	A	FLG	c.T5871C	p.P1957P
XH_P37	1	152327966	152327966	Missense_Mutation	SNP	C	G	C	FLG2	c.G2296C	p.G766R
XH_P37	1	152636719	152636742	In_Frame_Del	DEL	GGTCCCAGCT	-	TGGTCCCAGCTC	LCE2D	c.138_161del	p.46_54del
XH_P37	1	153641033	153641033	Nonsense_Mutation	INS	-	AGGGTTTAATTG	-	ILF2	09insAACAATTA	p.C37delinsNNX
XH_P37	1	153945498	153945498	Frame_Shift_Ins	INS	-	ACGCT	-	CREB3L4	687_688insACG	p.Q229fs
XH_P37	1	153945929	153945929	Frame_Shift_Ins	INS	-	ATGTACTC	-	CREB3L4	6_887insATGTA	p.T296fs
XH_P37	1	153974459	153974459	Frame_Shift_Ins	INS	-	TCGGGGAGATA	-	NUP210L	sTACTCTTATCTC	p.V1645fs
XH_P37	1	154145660	154145660	Nonsense_Mutation	INS	-	GCTTCCTTATTC	-	TPM3	nsGAGAATAAGC	29_N30delinsGEX
XH_P37	1	154569676	154569676	Missense_Mutation	SNP	C	T	C	ADAR	c.G1117A	p.A373T
XH_P37	1	154680718	154680718	Nonsense_Mutation	INS	-	CAGTTGCTAAAT	-	KCNN3	0insTTTATTTAG	p.E644delinsFIX
XH_P37	1	155142305	155142305	Missense_Mutation	SNP	A	T	A	KRTCAP2	c.T330A	p.F110L
XH_P37	1	155142306	155142306	Nonsense_Mutation	INS	-	GTGTCTTCCCT	-	KRTCAP2	329insAGGGAA	F110_A111delinsX
XH_P37	1	155160832	155160832	Frame_Shift_Ins	INS	-	CTGTCCACACCC	-	MUC1	insAAGGGTGTG	p.T232fs
XH_P37	1	155174744	155174744	Frame_Shift_Ins	INS	-	CTCCAATACACC	-	THBS3	48insGGGTGTAT	p.F183fs
XH_P37	1	155207975	155207975	Nonsense_Mutation	INS	-	TTTTTATTTTTCT	-	GBA	AAGAAGAAAAA	0_G151delinsKKEEKX
XH_P37	1	155209781	155209781	Missense_Mutation	SNP	G	A	G	GBA	c.C203T	p.P68L
XH_P37	1	155218094	155218094	In_Frame_Ins	INS	-	TTATTGATGATG	-	FAM189B	1292insCATCAT	G431delinsASSIR
XH_P37	1	155218095	155218095	Splice_Site	INS	-	CCTTTCTTG	-	FAM189B	.	.
XH_P37	1	155447927	155447927	In_Frame_Ins	INS	-	GACACT	-	ASH1L	733_4734insAGT	.D1578delinsEVS
XH_P37	1	155450723	155450723	Nonsense_Mutation	INS	-	TTAAAAACGTTT	-	ASH1L	938insGAAACG	6_S647delinsMKRFX
XH_P37	1	155646500	155646500	Nonsense_Mutation	INS	-	TTACACTGGGG	-	YY1AP1	TTAAACCCCAGT	H44_Q45delinsGX
XH_P37	1	155698914	155698914	Splice_Site	INS	-	AGTTCATTT	-	DAP3	.	.
XH_P37	1	155912213	155912213	Frame_Shift_Ins	INS	-	CTGTGAT	-	RXFP4	13_714insCTGT(	p.R238fs
XH_P37	1	156235714	156235714	Frame_Shift_Ins	INS	-	AATGACCTCTC	-	SMG5	1575insGAGAG	p.S525fs
XH_P37	1	156263355	156263355	Frame_Shift_Ins	INS	-	GCCCTGGC	-	GLMP	2_553insGCCAG	p.L185fs
XH_P37	1	156268868	156268868	Frame_Shift_Ins	INS	-	TTAA	-	VHLL	.112_113insTTA	p.A38fs
XH_P37	1	156445012	156445012	Frame_Shift_Ins	INS	-	TACTTATGGTTA	-	MEF2D	3insGTTAACCAT	p.L291fs
XH_P37	1	156928653	156928653	Frame_Shift_Ins	INS	-	TT	-	ARHGEF11	.1262_1263insA	p.T421fs
XH_P37	1	156928654	156928654	Frame_Shift_Ins	INS	-	CCTGA	-	ARHGEF11	261_1262insTCA	p.T421fs
XH_P37	1	157665202	157665202	Nonsense_Mutation	INS	-	TCTTCCTAGTGAT	-	FCRL3	nsTGTAATCACTA	p.A443delinsVX
XH_P37	1	158390303	158390303	Nonsense_Mutation	INS	-	GATTGTCATCTTC	-	OR10K2	GCATGAAGATG	G119delinsIQSMKMTIX
XH_P37	1	158390535	158390535	Nonsense_Mutation	INS	-	TGCTCTCACTCC	-	OR10K2	2insAGGAGTGA	41_N42delinsKEX
XH_P37	1	158607956	158607956	Nonsense_Mutation	INS	-	AGCACTACTTCA	-	SPTA1	CAAACATGAAG	6_K1687delinsANMKX
XH_P37	1	158653270	158653270	Missense_Mutation	SNP	T	G	T	SPTA1	c.A281C	p.H94P
XH_P37	1	158653272	158653272	Frame_Shift_Ins	INS	-	ATGATGGGATAT	-	SPTA1	CGAAGATATCCC	p.K93fs
XH_P37	1	158908296	158908296	Silent	SNP	C	A	C	PYHIN1	c.C375A	p.L125L
XH_P37	1	158946506	158946506	Nonsense_Mutation	INS	-	AAAGGGAAACTT	-	PYHIN1	insGCTAAAGGC	p.D459delinsGX
XH_P37	1	159021716	159021716	Missense_Mutation	SNP	G	A	G	IFI16	c.G1745A	p.R582H
XH_P37	1	159505073	159505073	Nonsense_Mutation	INS	-	CTTAATGGATTG	-	OR10J5	ATAATCCAATCC	242_H243delinsX
XH_P37	1	160000980	160000980	Frame_Shift_Ins	INS	-	ACTTTTCTCCGTA	-	PIGM	0insAGTACGGAC	p.V184fs
XH_P37	1	160268972	160268972	Frame_Shift_Ins	INS	-	TTTTTTTTTTCTT	-	COPA	7insAAAAGAAA	p.E593fs
XH_P37	1	160523764	160523764	Frame_Shift_Ins	INS	-	C	-	CD84	c.218_219insG	p.Q73fs
XH_P37	1	160970882	160970882	Frame_Shift_Ins	INS	-	CACTGGGACAT	-	F11R	_169insATGTCC	p.S57fs
XH_P37	1	161173285	161173285	Missense_Mutation	SNP	A	T	A	NDUFS2	c.A154T	p.M52L
XH_P37	1	161173286	161173286	Nonsense_Mutation	INS	-	TTTTGTTTTTTGT	-	NDUFS2	TTTTGTTTTGTT	Y53delinsILFCFVFCFFX
XH_P37	1	161206305	161206305	Nonsense_Mutation	INS	-	GGCATCCTACAT	-	NR1I3	1insGATGTAGG	17_A18delinsQMX
XH_P37	1	161967854	161967854	Frame_Shift_Ins	INS	-	TGACATCCGGAA	-	OLFML2B	238insATTCCGG	p.P413fs
XH_P37	1	162487951	162487951	Nonsense_Mutation	INS	-	CAACACATAGTA	-	UHMK1	AAGGCAACACA	V288delinsEGNTX
XH_P37	1	162492339	162492339	Frame_Shift_Ins	INS	-	CCACCTTTAATAT	-	UHMK1	1038insCCACCT	.X346delinsYHLX
XH_P37	1	163318837	163318837	Nonsense_Mutation	INS	-	AACACAATTCTA	-	NUF2	sAGGCAAACACA	D410delinsKRQTQFX
XH_P37	1	167358984	167358984	Nonsense_Mutation	INS	-	AAAAACACGTGA	-	POU2F1	GAGGAAAAACA	262_G263delinsQX
XH_P37	1	169762110	169762110	Missense_Mutation	SNP	C	A	C	METTL18	c.G727T	p.D243Y
XH_P37	1	169822119	169822119	Frame_Shift_Ins	INS	-	ATATAAAGAACC	-	C1orf112	9insAAATATAAA	p.T716fs
XH_P37	1	169890891	169890891	Nonsense_Mutation	INS	-	TGGGTCTGTGTT	-	KIFAP3	AAAACAAACACA	I726delinsIKIKTNTDPQX
XH_P37	1	171070276	171070276	Frame_Shift_Ins	INS	-	TCTCCATCTTAA	-	FMO3	AATCTCTCCATCT	p.M1fs
XH_P37	1	171080113	171080113	Frame_Shift_Ins	INS	-	TT	-	FMO3	c.613_614insTT	p.N205fs
XH_P37	1	171080114	171080114	Nonsense_Mutation	INS	-	CATTTGTAGTGC	-	FMO3	GTGACCATTTGT	I205_Y206delinsKX
XH_P37	1	171605699	171605699	Frame_Shift_Ins	INS	-	TGACTTGG	-	MYOC	0_881insCCAAG	p.D294fs
XH_P37	1	171605701	171605701	Silent	SNP	C	T	C	MYOC	c.G879A	p.T293T
XH_P37	1	172501694	172501694	In_Frame_Ins	INS	-	TTTTCC	-	SUCO	.82_83insTTTTC	p.G28delinsVFR
XH_P37	1	172501695	172501695	Frame_Shift_Ins	INS	-	CAATCAGATGAC	-	SUCO	CAACCAATCAGA	p.G28fs
XH_P37	1	172558670	172558670	Frame_Shift_Ins	INS	-	ACTGAGAAAGA	-	SUCO	41insGACTGAGA	p.D247fs
XH_P37	1	172571847	172571847	Nonsense_Mutation	INS	-	GAATTCCACATT	-	SUCO	TCCTTTGAATTC	Y500delinsSSFEFHIX
XH_P37	1	172579276	172579276	In_Frame_Ins	INS	-	GAAAAG	-	SUCO	53_1954insGAA	.K651delinsKEK
XH_P37	1	172579278	172579278	Frame_Shift_Ins	INS	-	AATGATACTTCC	-	SUCO	insAGTAATGAT	p.L652fs
XH_P37	1	173503730	173503730	Missense_Mutation	SNP	G	A	G	SLC9C2	c.C1867T	p.P623S
XH_P37	1	173503732	173503732	Frame_Shift_Ins	INS	-	TTTGAATTTTCCA	-	SLC9C2	AAACTTGGAAAA	p.Y622fs
XH_P37	1	173545865	173545865	Nonsense_Mutation	INS	-	CAG	-	SLC9C2	c.836_837insCT	p.L279delinsFX
XH_P37	1	173545867	173545867	Frame_Shift_Ins	INS	-	ACCAT	-	SLC9C2	834_835insATG	p.L279fs
XH_P37	1	173840118	173840118	Frame_Shift_Ins	INS	-	CTTAGCTCTCT	-	ZBTB37	_756insCTTAGC	p.E252fs
XH_P37	1	175375683	175375683	Frame_Shift_Ins	INS	-	CGAGATGCTG	-	TNR	_168insCAGCAT	p.T56fs
XH_P37	1	179077263	179077263	Frame_Shift_Ins	INS	-	TAGC	-	ABL2	075_3076insGC	p.P1026fs
XH_P37	1	179084127	179084127	Nonsense_Mutation	INS	-	TTTAAAGAGTTTA	-	ABL2	1384insTAAACT	p.M462delinsX
XH_P37	1	179084129	179084129	Nonsense_Mutation	INS	-	TCAGCCAGTTGT	-	ABL2	sATATGACAACT	M462delinsDMTTGX
XH_P37	1	179414304	179414304	Missense_Mutation	SNP	A	T	A	AXDND1	c.A1763T	p.Y588F
XH_P37	1	179414305	179414305	Frame_Shift_Ins	INS	-	CATTCAGGATCTA	-	AXDND1	TTTTCTCATTCAG	p.Y588fs
XH_P37	1	180044257	180044257	Frame_Shift_Ins	INS	-	ACTACTCTTCTCT	-	CEP350	69insATACTACT	p.R1890fs
XH_P37	1	180471302	180471302	Frame_Shift_Ins	INS	-	AATGCCACATCT	-	ACBD6	GGGAGAAGATGT	p.P34fs
XH_P37	1	183498669	183498669	Splice_Site	INS	-	AGGCACTGGC	-	SMG7	.	.
XH_P37	1	186063458	186063458	Frame_Shift_Ins	INS	-	AAATG	-	HMCN1	247_10248insAA	p.S3416fs

XH_P37	1	186063460	186063460	In_Frame_Ins	INS	-	ATTTATTCT	-	HMCN1	9_10250insATT	N3417delinsNLFY
XH_P37	1	186123113	186123113	Nonsense_Mutation	INS	-	TAATTTTTTTCCC	-	HMCN1	CTCATACTAATT	_K5085delinsWLILIFFPX
XH_P37	1	186275835	186275835	Frame_Shift_Ins	INS	-	TTTATTGTGTTG	-	PRG4	insACTTTATTGT	p.L194fs
XH_P37	1	186286733	186286733	Splice_Site	INS	-	AACTTGTTTGT	-	TPR	.	.
XH_P37	1	186302419	186302419	Frame_Shift_Ins	INS	-	TATATATCTTGA	-	TPR	GAATACTCAAG	p.L1764fs
XH_P37	1	186355199	186355199	Nonsense_Mutation	INS	-	ATAAAACTATCT	-	C1orf27	TATAAATAAAAC	105delinsHLKTTLX
XH_P37	1	193038362	193038362	Frame_Shift_Ins	INS	-	GAAAA	-	TROVE2	178_179insGAA	p.A60fs
XH_P37	1	193038363	193038363	In_Frame_Ins	INS	-	TTTACTGAAATG	-	TROVE2	GTAGGTTTACTG	delinsAKCRFTEMLLTL
XH_P37	1	193150487	193150487	Frame_Shift_Ins	INS	-	GCTTAAGTATT	-	B3GALT2	206insTAATACT	p.S69fs
XH_P37	1	193150489	193150489	Silent	SNP	C	G	C	B3GALT2	c.G204C	p.R68R
XH_P37	1	196227577	196227577	In_Frame_Ins	INS	-	AAAACTACT	-	KCNT2	5_2886insAGTA	.S962delinsSVVF
XH_P37	1	196227578	196227578	Frame_Shift_Ins	INS	-	TGATTTCTTGCC	-	KCNT2	ACTAATGGCAAG	p.S962fs
XH_P37	1	196642293	196642293	Frame_Shift_Ins	INS	-	TTCTTCTTGGAG	-	CFH	TGTATTTCTTCT	p.K82fs
XH_P37	1	196646746	196646746	Nonsense_Mutation	INS	-	TGGAATTTTAAA	-	CFH	TCTAATGGAATT	_H191delinsNTCNVFX
XH_P37	1	197072248	197072248	Nonsense_Mutation	INS	-	TTTAAAATGCAT	-	ASPM	TGACTGATGCAT	_A2046delinsLFLTDAFX
XH_P37	1	197169310	197169310	Frame_Shift_Ins	INS	-	GGAAGACTATC	-	ZBTB41	TTATTTGATAGT	p.E98fs
XH_P37	1	197407801	197407801	Nonsense_Mutation	INS	-	AGCTACAAATGC	-	CRB1	39insGAGCTAC	1180delinsGATNAX
XH_P37	1	197627500	197627500	Splice_Site	INS	-	CATTGTATCATC	-	DENND1B	.	.
XH_P37	1	198668822	198668822	Nonsense_Mutation	INS	-	ATTGCGATTATA	-	PTPRC	429insATTGCGA	43_A144delinsKLRLX
XH_P37	1	198725307	198725307	Nonsense_Mutation	INS	-	TTCTTGATGGTT	-	PTPRC	TTTTATCTTCTTG	45_X1146delinsSILSSX
XH_P37	1	200376643	200376643	Frame_Shift_Ins	INS	-	ATCACTAACTCT	-	ZNF281	sCCATTAGAGTT	p.S731fs
XH_P37	1	200534609	200534609	Nonsense_Mutation	INS	-	ATCCAGTCACTT	-	KIF14	377insTAAGTGA	793_H794delinsX
XH_P37	1	200539051	200539051	Nonsense_Mutation	INS	-	ATTTCAAAGCCT	-	KIF14	insAAAAGGCTT	.S726delinsKRLX
XH_P37	1	200817364	200817364	Frame_Shift_Ins	INS	-	TTTGAAATTACA	-	CAMSAP2	insTTATTTGAA	p.E473fs
XH_P37	1	201038658	201038658	Frame_Shift_Ins	INS	-	TACTGTT	-	CACNA1S	31_2432insAAC	p.L811fs
XH_P37	1	201176107	201176107	Frame_Shift_Ins	INS	-	AA	-	IGFN1	.2086_2087insA	p.G696fs
XH_P37	1	201176109	201176109	Frame_Shift_Ins	INS	-	CTCTA	-	IGFN1	088_2089insCT	p.G696fs
XH_P37	1	201195059	201195059	Missense_Mutation	SNP	T	G	T	IGFN1	c.T10594G	p.Y3532D
XH_P37	1	202397994	202397994	In_Frame_Ins	INS	-	CCTTTAAATATA	-	PPP1R12B	insAATCCTTTAA	286delinsPNPLNIE
XH_P37	1	202702982	202702982	Frame_Shift_Ins	INS	-	AGAGAAAA	-	KDM5B	20_3321insTTTT	p.E1107fs
XH_P37	1	202702983	202702983	Frame_Shift_Ins	INS	-	TCTG	-	KDM5B	319_3320insCA	p.E1107fs
XH_P37	1	203036913	203036913	Frame_Shift_Ins	INS	-	ACTCCCTTTCTCT	-	PPFIA4	insGTTACTCCCT	p.E1038fs
XH_P37	1	203036915	203036915	Missense_Mutation	SNP	T	A	T	PPFIA4	c.T3116A	p.I1039N
XH_P37	1	204091066	204091066	Frame_Shift_Ins	INS	-	A	-	SOX13	c.814_815insA	p.P272fs
XH_P37	1	204091067	204091067	Frame_Shift_Ins	INS	-	TCTAAAT	-	SOX13	15_816insTCTA	p.P272fs
XH_P37	1	204192711	204192711	Nonsense_Mutation	INS	-	AGCCATCTATGC	-	PLEKHA6	3034insGCATAG	.Q1012delinsAX
XH_P37	1	205589694	205589694	Frame_Shift_Ins	INS	-	GGAG	-	ELK4	.479_480insCTC	p.V160fs
XH_P37	1	207196539	207196539	Frame_Shift_Ins	INS	-	AAACA	-	C1orf116	569_570insTGT	p.A190fs
XH_P37	1	207196540	207196540	Nonsense_Mutation	INS	-	TCTCAAC	-	C1orf116	68_569insGTTG	190_L191delinsGX
XH_P37	1	212560386	212560386	In_Frame_Ins	INS	-	GTATTTGTGGTA	-	TMEM206	190insTACCACA	.N64delinsYHKYN
XH_P37	1	214543029	214543029	Nonsense_Mutation	INS	-	TTTTATGTAAGT	-	PTPN14	AAAAACACTTA	4_G1015delinsGKTLTX
XH_P37	1	214788329	214788329	Nonsense_Mutation	INS	-	GATACAGCTGC	-	CENPF	nsCTGAGATACA	106_G107delinsSX
XH_P37	1	214815121	214815121	In_Frame_Ins	INS	-	GGTGTTCCTTT	-	CENPF	AATTCTTGGTGT	47delinsKNSWCSFANA
XH_P37	1	214820283	214820283	In_Frame_Ins	INS	-	ATTTTTGCTTTT	-	CENPF	1insTCTATTTTT	2457delinsLLFLLLG
XH_P37	1	214820672	214820672	Nonsense_Mutation	INS	-	CACTCTCTCATT	-	CENPF	7760insCACTCT	87_S2588delinsPLSHX
XH_P37	1	214830554	214830554	Nonsense_Mutation	INS	-	AAG	-	CENPF	8764_8765insA	p.S2922delinsX
XH_P37	1	214830556	214830556	Frame_Shift_Ins	INS	-	AGGAACAG	-	CENPF	6_8767insAGGA	p.S2922fs
XH_P37	1	215793626	215793626	Nonsense_Mutation	INS	-	CTGCTTCATGG	-	KCTD3	insGTAGCTGCT	N703_I704delinsKX
XH_P37	1	215848937	215848937	Frame_Shift_Ins	INS	-	AAAAATAATTCG	-	USH2A	sTTCATAGCGAA	p.G4106fs
XH_P37	1	215953192	215953192	In_Frame_Ins	INS	-	ATCAAACACACA	-	USH2A	sCAGGGGTGTG	644delinsTRGVCLIYE
XH_P37	1	215953194	215953194	Missense_Mutation	SNP	T	G	T	USH2A	c.A10930C	p.T3644P
XH_P37	1	216166380	216166380	Missense_Mutation	SNP	C	T	C	USH2A	c.G6787A	p.E2263K
XH_P37	1	216172382	216172382	In_Frame_Ins	INS	-	CTCCCC	-	USH2A	03_6504insGGG	.K2168delinsKGR
XH_P37	1	220152861	220152861	Nonsense_Mutation	INS	-	CTGTGTGTATTA	-	EPRS	insAAATAATAC	.Q1270delinsKX
XH_P37	1	220363108	220363108	In_Frame_Ins	INS	-	TGAGAGGCTGA	-	RAB3GAP2	716insCTTCAGC	572delinsDFSLSC
XH_P37	1	220363110	220363110	Splice_Site	INS	-	TCTTTTCACATT	-	RAB3GAP2	.	.
XH_P37	1	220835431	220835431	Frame_Shift_Ins	INS	-	CCCT	-	MARK1	2200_2201insC	p.R734fs
XH_P37	1	220835432	220835432	Frame_Shift_Ins	INS	-	ATGTA	-	MARK1	201_2202insAT	p.R734fs
XH_P37	1	222802636	222802636	Nonsense_Mutation	INS	-	AGAACTAATTCC	-	MIA3	075insAAGAACT	p.F692delinsX
XH_P37	1	225555701	225555701	In_Frame_Ins	INS	-	TAACTATTTTCA	-	DNAH14	2007insGTAACT	4002delinsNVTIFK
XH_P37	1	225607437	225607437	Nonsense_Mutation	INS	-	TTAATGCAGTA	-	LBR	TAATGCTACTG	144_P145delinsHX
XH_P37	1	228475843	228475843	Frame_Shift_Ins	INS	-	A	-	OBSCN	.9893_9894ins	p.S3298fs
XH_P37	1	228475845	228475845	In_Frame_Ins	INS	-	CTTACC	-	OBSCN	395_9896insCTT	.K3299delinsTYQ
XH_P37	1	228646198	228646198	Nonsense_Mutation	INS	-	GTAGATAGAA	-	HIST3H2BB	_369insGTAGAT	123_S124delinsTX
XH_P37	1	228646199	228646199	Silent	SNP	C	T	C	HIST3H2BB	c.C369T	p.T123T
XH_P37	1	231401514	231401514	In_Frame_Ins	INS	-	TGAACAAACAC	-	GNPAT	sCTTCCTGAACA	90delinsKLPEQTQL
XH_P37	1	231678354	231678354	Frame_Shift_Ins	INS	-	CT	-	TSNAX	c.364_365insCT	p.T122fs
XH_P37	1	231678356	231678356	Frame_Shift_Ins	INS	-	ACTATTCATAGT	-	TSNAX	7insAAACTATTC	p.T122fs
XH_P37	1	232942566	232942566	In_Frame_Ins	INS	-	TCGTGTGCTGCA	-	MAP10	1798insTCGTGT	C599delinsCSCAA
XH_P37	1	234596085	234596085	Frame_Shift_Ins	INS	-	TAATATTGGGA	-	TARBP1	ATCCCAATCCCA	p.P486fs
XH_P37	1	235973306	235973306	Missense_Mutation	SNP	A	T	A	LYST	c.T812A	p.F271Y
XH_P37	1	235973307	235973307	Frame_Shift_Ins	INS	-	GAGCCCTT	-	LYST	0_811insAAGG	p.F271fs
XH_P37	1	236205459	236205459	Frame_Shift_Ins	INS	-	GATGTGGATG	-	NID1	_886insCATCCA	p.Y296fs
XH_P37	1	236990174	236990174	Nonsense_Mutation	INS	-	TTATACCATTTA	-	MTR	ATCCTTTATACC	25_S326delinsEILYTIX
XH_P37	1	237791334	237791334	Frame_Shift_Ins	INS	-	AAAGTCATTATG	-	RYR2	sGCTGCAAAGTC	p.V2132fs
XH_P37	1	237947363	237947363	Frame_Shift_Ins	INS	-	CGTGTAGTGCTT	-	RYR2	2352insGCGTGT	p.T4117fs
XH_P37	1	241767869	241767869	Frame_Shift_Ins	INS	-	CCTTTGTGCTTT	-	OPN3	TAAGAAAAGCA	p.I129fs
XH_P37	1	242253238	242253238	Missense_Mutation	SNP	C	G	C	PLD5	c.G905C	p.C302S
XH_P37	1	242253240	242253240	Frame_Shift_Ins	INS	-	AACACTTTAATAT	-	PLD5	GAGACATATTA	p.N301fs
XH_P37	1	243388519	243388519	Frame_Shift_Ins	INS	-	AAGTTCTAAGTC	-	CEP170	TCAAGACTTAGA	p.M22fs
XH_P37	1	244724247	244724247	Frame_Shift_Ins	INS	-	AGAATGGATCC	-	CATSPERE	sAAGAAGAATG	p.F285fs
XH_P37	1	246755170	246755170	Frame_Shift_Ins	INS	-	CTATTTGCAGAT	-	CNST	TGGTTCTATTTG	p.L102fs
XH_P37	1	247013112	247013112	Nonsense_Mutation	INS	-	TGGTTATTTGTC	-	AHCTF1	TTTTTAGGACAA	66_E2067delinsNHFX
XH_P37	1	247695765	247695772	Frame_Shift_Del	DEL	CCAGGAGG	-	CCAGGAGG	OR2C3	c.42_49del	p.V14fs
XH_P37	1	247751935	247751935	Frame_Shift_Ins	INS	-	TGCCGATGTATT	-	OR2G2	TCATATGCCGAT	p.M92fs
XH_P37	1	248020603	248020603	Missense_Mutation	SNP	T	C	T	TRIM58	c.T55C	p.C19R
XH_P37	1	248185868	248185868	Missense_Mutation	SNP	G	T	G	OR2L5	c.G619T	p.V207L
XH_P37	1	248185870	248185870	In_Frame_Ins	INS	-	CATACGGATGG	-	OR2L5	2insCTCATACGG	207delinsVLIRMG
XH_P37	1	248458482	248458482	In_Frame_Ins	INS	-	CGCTCT	-	OR2T12	98_399insAGAG	p.L133delinsLER
XH_P37	1	248487387	248487387	Frame_Shift_Ins	INS	-	AAAGCTTT	-	OR2M7	3_484insAAAG	p.A162fs
XH_P37	1	248487389	248487389	Frame_Shift_Ins	INS	-	CTACTTGTTC	-	OR2M7	_482insGAACAA	p.V161fs
XH_P37	1	248652023	248652023	Frame_Shift_Ins	INS	-	GATTTGAGTATA	-	OR2T5	35insGGATTTGA	p.G45fs
XH_P37	1	248790372	248790372	Missense_Mutation	SNP	C	A	C	OR2T11	c.G58T	p.A20S
XH_P37	1	248790373	248790373	Frame_Shift_Ins	INS	-	ATGGT	-	OR2T11	.56_57insACCA	p.E19fs
XH_P37	1	248844806	248844806	In_Frame_Ins	INS	-	AAAAAAAACCTG	-	OR14I1	_800insCAGGTT	.I267delinsTGFFF
XH_P37	1	248845216	248845216	Nonsense_Mutation	INS	-	TTATTCTCATGA	-	OR14I1	GATCATCATGA	p.Y130delinsX
XH_P37	2	1653285	1653285	Frame_Shift_Ins	INS	-	CTGCTTCTCTG	-	PXDN	2267insCAGAGA	p.W756fs
XH_P37	2	1653286	1653286	Missense_Mutation	SNP	A	T	A	PXDN	c.T2266A	p.W756R

XH_P37	2	1926932	1926932	Nonsense_Mutation	INS	-	ATTATGCAGACA	-	MYT1L	sCATGCTGTCTG	203delinsNMLSAX
XH_P37	2	3653819	3653819	Nonsense_Mutation	INS	-	CATAATTAAACAT	-	COLEC11	nsAACATAATTAA	p.P13delinsPTX
XH_P37	2	7030374	7030374	In_Frame_Ins	INS	-	ACTTTCAAAAAC	-	RSAD2	TATAAACTTTCA	delinsLLKYKLSKTCLDY
XH_P37	2	9595879	9595879	Frame_Shift_Ins	INS	-	GCACC	-	CPSF3	485_1486insGC	p.K495fs
XH_P37	2	9770460	9770460	Missense_Mutation	SNP	C	T	C	YWHAQ	c.G122A	p.R41H
XH_P37	2	11341261	11341261	Nonsense_Mutation	INS	-	TTTGATCTCCAG	-	ROCK2	AAGAGCTGGAG	.825_A826delinsX
XH_P37	2	11341263	11341263	Silent	SNP	A	T	A	ROCK2	c.T2472A	p.S824S
XH_P37	2	11341436	11341436	Missense_Mutation	SNP	C	G	C	ROCK2	c.G2465C	p.R822P
XH_P37	2	11341437	11341437	Frame_Shift_Ins	INS	-	AAGATGCTACAA	-	ROCK2	4insGCAATTGTA	p.R822fs
XH_P37	2	11761106	11761106	In_Frame_Ins	INS	-	CAAAGA	-	GREB1	20_4121insCAA	.V1374delinsAKI
XH_P37	2	11780490	11780490	Silent	SNP	C	T	C	GREB1	c.C5760T	p.D1920D
XH_P37	2	15417060	15417060	Nonsense_Mutation	INS	-	GCTAATCGACTTT	-	NBAS	ATAACTAAAGTC	3_C1769delinsKIITKVDX
XH_P37	2	15427299	15427299	Nonsense_Mutation	INS	-	TATA	-	NBAS	5035_5036insTA	679_E1680delinsVX
XH_P37	2	15427300	15427300	Nonsense_Mutation	INS	-	ACACCTTTACCAT	-	NBAS	AATGAATGGTA	579_E1680delinsHQX
XH_P37	2	15760478	15760478	Nonsense_Mutation	INS	-	GAACTTTTATAAT	-	DDX1	TGAGATGAACTT	.K451delinsKLRX
XH_P37	2	17890014	17890014	Frame_Shift_Ins	INS	-	AGAAACTGGTTG	-	SMC6	7insAAATCAACC	p.A579fs
XH_P37	2	21250721	21250721	In_Frame_Ins	INS	-	TGACTTCTTAAC	-	APOB	2046insGTTAAG	F682delinsLLRSH
XH_P37	2	21255429	21255429	Silent	SNP	A	T	A	APOB	c.T1149A	p.V383V
XH_P37	2	21255430	21255430	Frame_Shift_Ins	INS	-	TTTCT	-	APOB	147_1148insAG	p.V383fs
XH_P37	2	21361270	21361270	Nonsense_Mutation	INS	-	CATTTCCACAAT	-	TDRD15	insTAACATTTCC	311delinsLTFPQX
XH_P37	2	24261327	24261327	In_Frame_Ins	INS	-	AAA	-	WDCP	1037_1038insT	p.A346delinsAL
XH_P37	2	24261328	24261328	Frame_Shift_Ins	INS	-	AAAAAAGTTAAT	-	WDCP	ACTTTCAATTAAC	p.A346fs
XH_P37	2	25469121	25469121	Frame_Shift_Ins	INS	-	AA	-	DNMT3A	c.880_881insTT	p.A294fs
XH_P37	2	25469122	25469122	Missense_Mutation	SNP	C	G	C	DNMT3A	c.G880C	p.A294P
XH_P37	2	26676298	26676298	In_Frame_Ins	INS	-	GAAAAGTTTACA	-	DRC1	AAACCAGAAAAG	0delinsEEKPEKFTESD
XH_P37	2	27606331	27606331	Nonsense_Mutation	INS	-	ATTCCTGTGAAA	-	PPM1G	TAGTTATTTCAC	3_E369delinsLSYFTGIX
XH_P37	2	27668197	27668197	Missense_Mutation	SNP	G	T	G	IFT172	c.C5034A	p.S1678R
XH_P37	2	27668198	27668198	Nonsense_Mutation	INS	-	ATCATTTTT	-	IFT172	2_5033insAAAA	.S1678delinsKKX
XH_P37	2	27801022	27801022	In_Frame_Ins	INS	-	TTGTTAGCCCCAT	-	C2orf16	584insTTTGTTAC	T528delinsTLLAPL
XH_P37	2	27801632	27801632	Missense_Mutation	SNP	C	G	C	C2orf16	c.C2193G	p.F731L
XH_P37	2	27878435	27878435	Frame_Shift_Ins	INS	-	TT	-	SUPT7L	c.373_374insAA	p.I125fs
XH_P37	2	27878437	27878437	Frame_Shift_Ins	INS	-	AGTACTTTTTG	-	SUPT7L	372insCAAAAA	p.R124fs
XH_P37	2	27884020	27884020	Nonsense_Mutation	INS	-	TAATTTTTTGAGT	-	SUPT7L	AAGCACTCAAA	84delinsSKHSKNX
XH_P37	2	29039033	29039033	Frame_Shift_Ins	INS	-	TC	-	SPDYA	c.153_154insTC	p.H51fs
XH_P37	2	29039034	29039034	Frame_Shift_Ins	INS	-	TTTCATAT	-	SPDYA	54_155insTTTCA	p.N52fs
XH_P37	2	30748605	30748605	Nonsense_Mutation	INS	-	GATATAAAAAAG	-	LCLAT1	GAAAGATATAA	.W50_L51delinsX
XH_P37	2	37121145	37121145	Missense_Mutation	SNP	C	T	C	STRN	c.G827A	p.G276D
XH_P37	2	37255237	37255237	Nonsense_Mutation	INS	-	AATCAGTACTTT	-	HEATR5B	sGGGAAAAAGTA	28_T1229delinsGKKYX
XH_P37	2	37455137	37455137	Frame_Shift_Ins	INS	-	CTTTTTCGGACT	-	CEBPZ	199insAGTCCGA	p.T400fs
XH_P37	2	37518081	37518081	Frame_Shift_Ins	INS	-	G	-	PRKD3	c.488_489insC	p.K163fs
XH_P37	2	37518082	37518082	In_Frame_Ins	INS	-	TGACTAGGG	-	PRKD3	7_488insCCCTAC	.K163delinsTLVK
XH_P37	2	37543600	37543600	In_Frame_Ins	INS	-	CTGTCTTTATCT	-	PRKD3	58insAGATAAAG	.L23delinsQIKTV
XH_P37	2	39213128	39213128	Frame_Shift_Ins	INS	-	AGTTCCTCTGTA	-	SOS1	nsCCAGTACAGA	p.Q1280fs
XH_P37	2	45645665	45645665	In_Frame_Ins	INS	-	ATACAACAGTGA	-	SRBD1	2insTGCTCACTG	724delinsLAHCCIP
XH_P37	2	47184037	47184037	In_Frame_Ins	INS	-	AAATTCTCCTAT	-	TTC7A	insATAAAATTCT	136delinsSIKFSYE
XH_P37	2	48033493	48033493	Frame_Shift_Ins	INS	-	AAAGCAACATCC	-	MSH6	08insGAAAAGCA	p.H1136fs
XH_P37	2	48848396	48848396	Missense_Mutation	SNP	C	A	C	GTF2A1L	c.C112A	p.H38N
XH_P37	2	48915547	48915547	Frame_Shift_Ins	INS	-	ATTTGTGCTTGC	-	LHCGR	nsTGTAGCAAGC	p.E463fs
XH_P37	2	54001615	54001615	Nonsense_Mutation	INS	-	GTTGTGGTTCTGT	-	CHAC2	GACTGTTGTGG	p.L170delinsX
XH_P37	2	54080474	54080474	Missense_Mutation	SNP	G	A	G	GPR75	c.C1420T	p.P474S
XH_P37	2	54080476	54080476	Nonsense_Mutation	INS	-	GATCTTATGTAA	-	GPR75	AACTGTTTACAT	T473delinsKLFTX
XH_P37	2	54080632	54080632	In_Frame_Ins	INS	-	ACTTACTACAGC	-	GPR75	2insGATGCTGTA	421delinsRCCSKVQ
XH_P37	2	54859817	54859817	In_Frame_Ins	INS	-	AGGATCTCATTT	-	SPTBN1	641insAAGGATC	1214delinsEGSHFP
XH_P37	2	55253970	55253970	Nonsense_Mutation	INS	-	TTGTTAGGAGAT	-	RTN4	insGGATCTCCTA	216_K217delinsRISX
XH_P37	2	55523564	55523564	Frame_Shift_Ins	INS	-	TCAGA	-	CCDC88A	836_4837insTCT	p.S1613fs
XH_P37	2	55523566	55523566	Frame_Shift_Ins	INS	-	TTTTTTTTTTT	-	CCDC88A	4835insAAAAA	p.S1612fs
XH_P37	2	55615993	55615993	Frame_Shift_Ins	INS	-	GAATG	-	CCDC88A	193_194insCAT	p.N65fs
XH_P37	2	55615995	55615995	Frame_Shift_Ins	INS	-	TATTTAATAATTT	-	CCDC88A	TTAGAAAATTAT	p.V64fs
XH_P37	2	55785925	55785925	Nonsense_Mutation	INS	-	GTAGAATTTTATT	-	PPP4R3B	AAAATAAAATAA	29_T730delinsNKIKX
XH_P37	2	58373584	58373584	Nonsense_Mutation	INS	-	ATAATATATAAT	-	VRK2	AAGTAATAATAT	268_L269delinsGIEX
XH_P37	2	58386822	58386822	In_Frame_Ins	INS	-	TGGGAAACTGTT	-	VRK2	68insGTGGGAA	F389delinsFVGNCL
XH_P37	2	61546454	61546454	Frame_Shift_Ins	INS	-	GTTGTTTACACT	-	USP34	nsAAACAGTGTA	p.L1008fs
XH_P37	2	61575319	61575319	Nonsense_Mutation	INS	-	TGGGAATGAATT	-	USP34	sTTAAATAATTCA	657_S658delinsDX
XH_P37	2	62066865	62066865	Frame_Shift_Ins	INS	-	TAAGATGTGCAG	-	FAM161A	74insCACCTGCA	p.R425fs
XH_P37	2	62450075	62450075	Frame_Shift_Ins	INS	-	G	-	B3GNT2	c.721dupG	p.F240fs
XH_P37	2	62450077	62450077	In_Frame_Ins	INS	-	TCCCTGCGTTGC	-	B3GNT2	723insTCCCTGC	V241delinsVPCVA
XH_P37	2	64323729	64323729	Nonsense_Mutation	INS	-	TACTAGCTTTCA	-	PELI1	TGGAATGAAAG	07_S108delinsWNESX
XH_P37	2	67631307	67631307	Nonsense_Mutation	INS	-	CTTGACGTATTT	-	ETAA1	nsTTGCTCTTGAC	98_V499delinsICSX
XH_P37	2	67631641	67631641	Nonsense_Mutation	INS	-	ACACTGGTTTTAC	-	ETAA1	TTACTAACACTG	09_L610delinsLGYX
XH_P37	2	68692191	68692191	Frame_Shift_Ins	INS	-	GAAATGAGTTA	-	FBXO48	nsCTTTAACTCAT	p.E29fs
XH_P37	2	68692193	68692193	Missense_Mutation	SNP	C	T	C	FBXO48	c.G85A	p.E29K
XH_P37	2	69741837	69741837	In_Frame_Ins	INS	-	CAGCGG	-	AAK1	41_1542insCCG	.Q514delinsHRW
XH_P37	2	69741838	69741838	Frame_Shift_Ins	INS	-	AA	-	AAK1	.1540_1541insT	p.Q514fs
XH_P37	2	70052627	70052627	Nonsense_Mutation	INS	-	ATATAGCCTATT	-	ANXA4	TTAACAATATAG	16_C317delinsHLTIX
XH_P37	2	70070394	70070394	Splice_Site	INS	-	GAAAAGAAAGAA	-	GMCL1	.	.
XH_P37	2	70106073	70106073	Frame_Shift_Ins	INS	-	C	-	GMCL1	c.1486dupC	p.L495fs
XH_P37	2	70106075	70106075	Frame_Shift_Ins	INS	-	ACTGTAAGATGG	-	GMCL1	488insCACTGTA	p.L496fs
XH_P37	2	70408629	70408629	In_Frame_Ins	INS	-	GCGGTAGGTGGT	-	C2orf42	89insATACCACCT	P163delinsPYHLPL
XH_P37	2	71351640	71351640	Frame_Shift_Ins	INS	-	TGGAGTATCTGT	-	MCEE	AACAACAGATAC	p.T25fs
XH_P37	2	71592759	71592759	Frame_Shift_Ins	INS	-	CT	-	ZNF638	.1918_1919insC	p.K640fs
XH_P37	2	71592760	71592760	Nonsense_Mutation	INS	-	TTCTAATGCTTTT	-	ZNF638	nsCATCTTCTAAT	640_S641delinsNIFX
XH_P37	2	71645759	71645759	Nonsense_Mutation	INS	-	ACACACAATAAC	-	ZNF638	0insGTCACACAC	7_K1098delinsGHTQX
XH_P37	2	71650361	71650361	Frame_Shift_Ins	INS	-	TCTTCTTTGAC	-	ZNF638	_3718insTCTTCT	p.I1239fs
XH_P37	2	72692440	72692440	Frame_Shift_Ins	INS	-	TTTCCTTATAATT	-	EXOC6B	sAACTAAATTAT	p.I565fs
XH_P37	2	73437917	73437917	Frame_Shift_Ins	INS	-	ATTCATTCATCTC	-	NOTO	nsCACCATTCATT	p.N206fs
XH_P37	2	73679956	73679956	Frame_Shift_Ins	INS	-	GGGCTTCTCTAT	-	ALMS1	00insTTGGGCTT	p.S2100fs
XH_P37	2	73989984	73989984	Nonsense_Mutation	INS	-	TGACTTTTAATTA	-	DUSP11	GAACTAATTAA	p.K313delinsTX
XH_P37	2	74043031	74043031	Nonsense_Mutation	INS	-	TGATTTACCTTG	-	C2orf78	82insCCTGATTT	561_I562delinsTX
XH_P37	2	74044025	74044025	Frame_Shift_Ins	INS	-	ACAAGATGATGT	-	C2orf78	76insGAACAAGA	p.N892fs
XH_P37	2	80101217	80101217	Frame_Shift_Ins	INS	-	CCTCTTCA	-	CTNNA2	01_602insCCTCT	p.H201fs
XH_P37	2	80101219	80101219	Frame_Shift_Ins	INS	-	CTCT	-	CTNNA2	.603_604insCTC	p.H201fs
XH_P37	2	84838942	84838942	Frame_Shift_Ins	INS	-	CTTTTTCCTTGAC	-	DNAH6	CTGTATCTTTTTC	p.R1147fs
XH_P37	2	84940357	84940357	Nonsense_Mutation	INS	-	TTTAAAATGGATA	-	DNAH6	AAGCAATTAAAA	_P3174delinsKKQLKWIAX
XH_P37	2	85768421	85768421	Frame_Shift_Ins	INS	-	TCAGTTTCTTTA	-	MAT2A	CTGGCTCAGTTT	p.I71fs
XH_P37	2	86371519	86371519	Nonsense_Mutation	INS	-	GCAGGTTTACAA	-	IMMT	sCAGTACTTGTA	16_R717delinsQYLX
XH_P37	2	96259924	96259924	Missense_Mutation	SNP	C	G	C	TRIM43	c.C153G	p.N51K
XH_P37	2	96259925	96259925	In_Frame_Ins	INS	-	TTTGAATCAGATC	-	TRIM43	155insTTGAATC	.C52delinsFESDR
XH_P37	2	96521901	96521901	In_Frame_Ins	INS	-	AAACGATAGCCT	-	ANKRD36C	5206insAGGCTA	L1736delinsRLSFL

XH_P37	2	96952204	96952204	Frame_Shift_Ins	INS	-	ATGTGCTCTGT	-	SNRNP200	3848insACAGA	p.P1283fs
XH_P37	2	96993664	96993664	In_Frame_Ins	INS	-	TGAAGCTGGTG	-	ITPRIPL1	TGTTCCTGAAG	40delinsHLFLKLVGRF
XH_P37	2	96993852	96993852	Frame_Shift_Ins	INS	-	TTTAAAACAGCT	-	ITPRIPL1	ACCATTTTAAA	p.I503fs
XH_P37	2	97637754	97637754	Missense_Mutation	SNP	G	C	G	FAM178B	c.C448G	p.L150V
XH_P37	2	97637755	97637755	Silent	SNP	C	T	C	FAM178B	c.G447A	p.E149E
XH_P37	2	97847519	97847519	Frame_Shift_Ins	INS	-	ATCACTCACATG	-	ANKRD36	27insTAATCACT	p.Q609fs
XH_P37	2	98173423	98173423	Missense_Mutation	SNP	A	G	A	ANKRD36B	c.T1046C	p.M349T
XH_P37	2	98377355	98377355	Frame_Shift_Ins	INS	-	TTGAACGTCCAT	-	TMEM131	12insCAATGGA	p.N1638fs
XH_P37	2	98428976	98428976	Nonsense_Mutation	INS	-	TTTAATTATCTT	-	TMEM131	TTACAAAAAGGA	E592delinsLVLQKDNX
XH_P37	2	98866899	98866899	Nonsense_Mutation	INS	-	TACATAGTTGTA	-	VWA3B	TACATCTACATA	K588delinsNTSTX
XH_P37	2	99013119	99013134	Frame_Shift_Del	DEL	AAGCTGCGA	-	GAAGCTGCGAC	CNGA3	c.1432_1447del	p.L478fs
XH_P37	2	100019486	100019486	Nonsense_Mutation	SNP	T	A	T	REV1	c.A3247T	p.K1083X
XH_P37	2	100019488	100019488	Nonsense_Mutation	INS	-	TAATATACCTGG	-	REV1	ACATTATCCAGG	2_K1083delinsHIIQVYX
XH_P37	2	100910699	100910699	Nonsense_Mutation	INS	-	TTTTCTTTATCCC	-	LONRF2	insATGGGGATA	E583delinsEWGX
XH_P37	2	102460702	102460702	Nonsense_Mutation	INS	-	ATATCTTCTTTTA	-	MAP4K4	AGTCCCATATCT	3delinsIYNSIVPYLLLLIX
XH_P37	2	103068372	103068372	Nonsense_Mutation	INS	-	ATTTGCAATGGA	-	IL18RAP	nsGATTATTTGCA	p.L511delinsX
XH_P37	2	108487451	108487451	Frame_Shift_Ins	INS	-	CTTTCCTTTTCTT	-	RGPD4	2992insCTTTCC	p.S997fs
XH_P37	2	108487453	108487453	Missense_Mutation	SNP	G	A	G	RGPD4	c.G2993A	p.G998D
XH_P37	2	108487693	108487693	In_Frame_Ins	INS	-	GTTTGCTTTATTG	-	RGPD4	4insGTGTTTGCT	1078delinsKCLLYWP
XH_P37	2	108626789	108626789	Nonsense_Mutation	INS	-	CAAAGAATGAGA	-	SLC5A7	TTAGGCAAAGAA	p.L300delinsLIX
XH_P37	2	109116167	109116167	Frame_Shift_Ins	INS	-	AACTCATGTATT	-	GCC2	nsACTGAACTCAT	p.Q1647fs
XH_P37	2	109347339	109347339	In_Frame_Ins	INS	-	CCAAATTTTTCT	-	RANBP2	sTTTACCAAATT	84delinsIYQIFLQW
XH_P37	2	109380677	109380677	Nonsense_Mutation	INS	-	ATGATTCTTGTT	-	RANBP2	insTCTCATGATT	228_K1229delinsVSX
XH_P37	2	109382444	109382444	Nonsense_Mutation	INS	-	GTTTGATTTTGT	-	RANBP2	50insTAGTTTGA	817_P1818delinsVVX
XH_P37	2	109382958	109382958	In_Frame_Ins	INS	-	TGAATCCTTGCT	-	RANBP2	4insATTTGAATC	1988delinsKFESLLC
XH_P37	2	112767580	112767580	Frame_Shift_Ins	INS	-	CTTTCAGA	-	MERTK	6_2017insCTTT	p.P672fs
XH_P37	2	113174876	113174876	Frame_Shift_Ins	INS	-	TCATTATTTCTAT	-	RGPD5	sCATTATAGAAA	p.S226fs
XH_P37	2	113260636	113260636	In_Frame_Ins	INS	-	TGCATATTATATA	-	TTL	sTTTCTGCATAT	251delinsKFLHIIYI
XH_P37	2	113332919	113332919	Frame_Shift_Ins	INS	-	TTAACATCCCAA	-	POLR1B	389insATTAACA	p.S796fs
XH_P37	2	114202593	114202593	Missense_Mutation	SNP	G	A	G	CBWD2	c.G391A	p.D131N
XH_P37	2	114202594	114202594	Frame_Shift_Ins	INS	-	CTCACTTCTAAC	-	CBWD2	393insCTCACTT	p.D131fs
XH_P37	2	118854223	118854223	Frame_Shift_Ins	INS	-	CGGTACCACAT	-	INSIG2	insAACGGTACC	p.R31fs
XH_P37	2	120414028	120414028	Frame_Shift_Ins	INS	-	GTTAGTGATAGT	-	CFAP221	2506insGTTAGT	p.T835fs
XH_P37	2	120620219	120620219	In_Frame_Ins	INS	-	TTTATGCCAGAG	-	PTPN4	247insTTTATGC	P82delinsPFMPE
XH_P37	2	120925501	120925501	In_Frame_Ins	INS	-	GTTGTTTTGTTT	-	EPB41L5	ATGACTGTTTGT	delinsIIMTVCFVFLNYS
XH_P37	2	122106266	122106266	Frame_Shift_Ins	INS	-	TGCCTAACTTGA	-	CLASP1	insTACTTCAAGT	p.S1345fs
XH_P37	2	122144748	122144748	Frame_Shift_Ins	INS	-	AATGTTCTCTC	-	CLASP1	3240insGAGAG	p.T1080fs
XH_P37	2	128707517	128707517	Frame_Shift_Ins	INS	-	TGTGTGTATTTA	-	SAP130	insCCAATAAATA	p.L872fs
XH_P37	2	129025797	129025797	Missense_Mutation	SNP	G	A	G	HS6ST1	c.C1175T	p.A392V
XH_P37	2	130934202	130934202	Frame_Shift_Ins	INS	-	CTACAGGACCT	-	SMPD4	nsCCAAGGTCCT	p.A27fs
XH_P37	2	130952659	130952659	Missense_Mutation	SNP	C	T	C	TUBA3E	c.G368A	p.R123H
XH_P37	2	132235907	132235907	Nonsense_Mutation	INS	-	AATCGATATTTC	-	TUBA3D	TTCACAATCGAT	8_G59delinsAFTIDISX
XH_P37	2	133070479	133070479	Frame_Shift_Ins	INS	-	TGTGTCCTTA	-	ZNF806	_54insTGTGTC	p.T18fs
XH_P37	2	135711691	135711691	Frame_Shift_Ins	INS	-	ACTCTTTTCCTTT	-	CCNT2	667insCACTCTT	p.H556fs
XH_P37	2	135711884	135711884	Frame_Shift_Ins	INS	-	CT	-	CCNT2	.1859_1860ins	p.R620fs
XH_P37	2	135711885	135711885	Frame_Shift_Ins	INS	-	TGTCTTT	-	CCNT2	60_1861insTGT	p.R620fs
XH_P37	2	135745134	135745134	Frame_Shift_Ins	INS	-	ATGACAAGCATA	-	MAP3K19	CATCTTATGCTT	p.E323fs
XH_P37	2	135908069	135908069	In_Frame_Ins	INS	-	ACAAGATGAGTA	-	RAB3GAP1	2055insACAAGA	S685delinsSQDEY
XH_P37	2	135988261	135988261	Frame_Shift_Ins	INS	-	T	-	ZRANB3	c.1775dupA	p.E592fs
XH_P37	2	135988263	135988263	In_Frame_Ins	INS	-	GATAACCTGTGC	-	ZRANB3	774insAAGCACA	E592delinsKHRLSE
XH_P37	2	136615557	136615557	Nonsense_Mutation	INS	-	TCAGTGTGTAAT	-	MCM6	sACAGCATTACA	E461delinsEQHYTLKX
XH_P37	2	138722089	138722089	Nonsense_Mutation	INS	-	GAATTTAA	-	HNMT	8_29insGAATTT	.S10_D11delinsX
XH_P37	2	138722090	138722090	Frame_Shift_Ins	INS	-	A	-	HNMT	c.29_30insA	p.S10fs
XH_P37	2	139322517	139322517	Nonsense_Mutation	INS	-	AATGAAAACATA	-	SPOPL	TCCAAAATGAAA	0_L331delinsAYYPKX
XH_P37	2	141115676	141115676	Frame_Shift_Ins	INS	-	ACTAAAATGT	-	LRP1B	_11267insACAT	p.T3756fs
XH_P37	2	141253294	141253294	Frame_Shift_Ins	INS	-	TATACATA	-	LRP1B	3_8874insTATG	p.Q2958fs
XH_P37	2	141253296	141253296	Frame_Shift_Ins	INS	-	AATTTTATAGCC	-	LRP1B	TAAGAACGGCTA	p.Q2958fs
XH_P37	2	141598610	141598610	Nonsense_Mutation	INS	-	C	-	LRP1B	c.4990_4991ins	1664_W1665delinsX
XH_P37	2	141598611	141598611	Frame_Shift_Ins	INS	-	TAAAGTATGATTA	-	LRP1B	AAAAAAATAATC	p.Y1664fs
XH_P37	2	148701091	148701091	Nonsense_Mutation	INS	-	TTTTATGGGAATT	-	ORC4	TAATCAATTCCC	171_L172delinsVX
XH_P37	2	152267851	152267851	Nonsense_Mutation	INS	-	TCTAACTCTTGCA	-	RIF1	TACTTTCTAACT	61_T62delinsKITFX
XH_P37	2	152512936	152512936	Frame_Shift_Ins	INS	-	A	-	NEB	c.6225_6226ins	p.V2076fs
XH_P37	2	152512937	152512937	Nonsense_Mutation	INS	-	ACTACAAGCACC	-	NEB	25insCAGGTGCT	75_V2076delinsIRCLX
XH_P37	2	153000509	153000509	Nonsense_Mutation	INS	-	AGTGTATTTAAA	-	STAM2	TATGATTTAAAT	179_E180delinsLLX
XH_P37	2	153000511	153000511	Silent	SNP	C	A	C	STAM2	c.G534T	p.L178L
XH_P37	2	153006702	153006702	In_Frame_Ins	INS	-	ACAGCAAGACCA	-	STAM2	82insTGGTCTTG	L28delinsWSCCL
XH_P37	2	157182444	157182444	Frame_Shift_Ins	INS	-	AGAGATAGCCG	-	NR4A2	_1609insCGGCT	p.D537fs
XH_P37	2	160086784	160086784	Missense_Mutation	SNP	C	T	C	TANC1	c.C4244T	p.P1415L
XH_P37	2	160086786	160086791	In_Frame_Del	DEL	GCACAC	-	GCACAC	TANC1	c.4246_4251del	p.1416_1417del
XH_P37	2	160721429	160721429	Frame_Shift_Ins	INS	-	ATAAAAACAGA	-	LY75	_2120insTCTGT	p.G707fs
XH_P37	2	160737723	160737723	Frame_Shift_Ins	INS	-	CTGTTTT	-	LY75	74_1275insAAAA	p.L425fs
XH_P37	2	160737724	160737724	Frame_Shift_Ins	INS	-	AATATTAAGACT	-	LY75	CAAAGAAGTCT	p.L425fs
XH_P37	2	162087932	162087932	Frame_Shift_Ins	INS	-	TGTAAGAAAGTT	-	TANK	GCTATGTAAGA	p.D324fs
XH_P37	2	163055376	163055376	Nonsense_Mutation	INS	-	AAGGTATTATTA	-	FAP	218insATAATAA	406_Y407delinsRX
XH_P37	2	163228584	163228584	Nonsense_Mutation	INS	-	TATTT	-	KCNH7	345_3346insAA	116_K1117delinsKX
XH_P37	2	163228586	163228586	Frame_Shift_Ins	INS	-	ATCCTCTCTACTT	-	KCNH7	344insAAGTAG	p.L1115fs
XH_P37	2	163291887	163291887	Frame_Shift_Ins	INS	-	ATGTTGATTGG	-	KCNH7	1754insCCAAT	p.G585fs
XH_P37	2	163291888	163291888	Frame_Shift_Ins	INS	-	TG	-	KCNH7	.1752_1753ins	p.G585fs
XH_P37	2	164466660	164466660	Nonsense_Mutation	INS	-	ACAGCACAATTA	-	FIGN	1649insTAATTG	G550delinsVIVLX
XH_P37	2	164467727	164467727	Nonsense_Mutation	INS	-	TCTCTCAAAAG	-	FIGN	582insCTTTTGA	194_L195delinsAFX
XH_P37	2	164467729	164467729	Missense_Mutation	SNP	C	A	C	FIGN	c.G580T	p.A194S
XH_P37	2	165578792	165578792	In_Frame_Ins	INS	-	TCCTCTTACCTAA	-	COBLL1	9insGTTAGGTAA	/263delinsVLGKRK
XH_P37	2	165584645	165584645	Frame_Shift_Ins	INS	-	CAACAGA	-	COBLL1	94_495insTCTGT	p.P165fs
XH_P37	2	165584647	165584647	Nonsense_Mutation	INS	-	TTATTTATTTAGT	-	COBLL1	ATCCCACTAAATA	H166delinsTNIHIPLNKX
XH_P37	2	166020190	166020190	Nonsense_Mutation	INS	-	TCAAAAAATGCT	-	SCN3A	2insTTAGCATTT	211_V212delinsISIFX
XH_P37	2	166532964	166532964	Frame_Shift_Ins	INS	-	AATAAGAGACT	-	CSRNP3	52insAAATAAGA	p.A184fs
XH_P37	2	166848796	166848796	Frame_Shift_Ins	INS	-	AATTTTCTTTTTT	-	SCN1A	ACTGACAAAAAA	p.L1663fs
XH_P37	2	167056294	167056294	In_Frame_Ins	INS	-	CCT	-	SCN9A	4821_4822insA	.R1608delinsRR
XH_P37	2	167056295	167056295	Frame_Shift_Ins	INS	-	TT	-	SCN9A	.4820_4821insA	p.G1607fs
XH_P37	2	167168170	167168170	Frame_Shift_Ins	INS	-	C	-	SCN9A	c.96_97insG	p.K33fs
XH_P37	2	167168172	167168172	Nonsense_Mutation	INS	-	TATAAAAATT	-	SCN9A	4_95insAATTTTT	.S32_K33delinsX
XH_P37	2	167289045	167289045	Frame_Shift_Ins	INS	-	GATGGAGG	-	SCN7A	74_2375insCCT	p.D792fs
XH_P37	2	167298238	167298238	Missense_Mutation	SNP	A	C	A	SCN7A	c.T1825G	p.Y609D
XH_P37	2	167298240	167298240	In_Frame_Ins	INS	-	GGTGTA	-	SCN7A	822_1823insTA	.K608delinsIHQ
XH_P37	2	168101119	168101119	In_Frame_Ins	INS	-	CAAATTTAATTA	-	XIRP2	2insCATCAAATT	/851delinsASNLIKL
XH_P37	2	168104370	168104370	In_Frame_Ins	INS	-	TTGAAGTTAATT	-	XIRP2	803insGTTGAAG	1934delinsTVEVNC
XH_P37	2	168115692	168115692	Nonsense_Mutation	INS	-	GAGATGATTGAA	-	XIRP2	971insGAGATG	657_V658delinsERX
XH_P37	2	168920070	168920070	Nonsense_Mutation	INS	-	GAATCGTTAGCT	-	STK39	TCAAATAAGCTA	439_A440delinsIKX

XH_P37	2	170060777	170060777	In_Frame_Ins	INS	-	TGTGAA	-	LRP2	719_7720insTTC	.R2574delinsFTR
XH_P37	2	170060778	170060778	Nonsense_Mutation	INS	-	AGTTTAA	-	LRP2	18_7719insTTAA	573_R2574delinsHX
XH_P37	2	170063328	170063328	Frame_Shift_Ins	INS	-	CCTGAAAACCAT	-	LRP2	902insATGGTTT	p.F2301fs
XH_P37	2	170667793	170667793	In_Frame_Ins	INS	-	ACTTCCCAA	-	SSB	8_1099insACTTC	.S366delinsSTSQ
XH_P37	2	170667794	170667794	In_Frame_Ins	INS	-	ACTTCTTTGTTCC	-	SSB	0insTCACTTCTT	367delinsVTSLFLN
XH_P37	2	171917623	171917623	Frame_Shift_Ins	INS	-	ATGTTGCCCTTA	-	TLK1	insATAAGGGCA	p.R29fs
XH_P37	2	171917625	171917625	Missense_Mutation	SNP	C	G	C	TLK1	c.G84C	p.K28N
XH_P37	2	172582767	172582767	Frame_Shift_Ins	INS	-	ACTTAAAAATTC	-	DYNC1I2	1insAAACTTAAA	p.K270fs
XH_P37	2	172582769	172582769	Frame_Shift_Ins	INS	-	GAGTGCAAGAT	-	DYNC1I2	813insGAGTGCA	p.H271fs
XH_P37	2	174228148	174228148	Nonsense_Mutation	INS	-	AACTGAACA	-	CDCA7	2_343insAACTGA	p.F114delinsFNX
XH_P37	2	174228150	174228150	Frame_Shift_Ins	INS	-	GATACCTCTG	-	CDCA7	_345insGATACC	p.L115fs
XH_P37	2	175268943	175268943	Frame_Shift_Ins	INS	-	AGAAAAGTCAT	-	SCRN3	34insAAGAAAA	p.E211fs
XH_P37	2	175268945	175268945	In_Frame_Ins	INS	-	CATAAAGCATGT	-	SCRN3	nsTAACATAAAG	212delinsFNIKHVS
XH_P37	2	175436470	175436470	In_Frame_Ins	INS	-	ATTTCTGTTGGT	-	WIPF1	insAACACCAAC	355delinsNTNRNIG
XH_P37	2	175439961	175439961	Nonsense_Mutation	INS	-	ACAGAACTATGA	-	WIPF1	9insTTTCATAGT	110_L111delinsISX
XH_P37	2	175666522	175666522	Missense_Mutation	SNP	T	A	T	CHN1	c.A746T	p.E249V
XH_P37	2	175666523	175666523	In_Frame_Ins	INS	-	AGAAGGAATGCT	-	CHN1	ATTTTAGCATTC	249delinsIFSIPSQLE
XH_P37	2	176959316	176959316	Frame_Shift_Ins	INS	-	TTTGGTCTTTAT	-	HOXD13	sGCTGTTTGGTC	p.I297fs
XH_P37	2	178080604	178080604	Frame_Shift_Ins	INS	-	TCTTAAGTTACCT	-	HNRNPA3	nsTACTCTTAAGT	p.K86fs
XH_P37	2	178081619	178081619	Frame_Shift_Ins	INS	-	CTGCATCTCTT	-	HNRNPA3	_706insCTGCAT	p.R235fs
XH_P37	2	178416535	178416535	Frame_Shift_Ins	INS	-	TTGAAAATTATC	-	TTC30B	nsATTGGATAAT	p.P319fs
XH_P37	2	178592818	178592818	Frame_Shift_Ins	INS	-	ACCAGAAGAGAT	-	PDE11A	9insACATCTCTT	p.I180fs
XH_P37	2	178705102	178705102	Nonsense_Mutation	INS	-	GCAAGATGGTT	-	PDE11A	nsGATAACCATC	E15_S16delinsGX
XH_P37	2	179253856	179253856	Frame_Shift_Ins	INS	-	T	-	OSBPL6	c.2184_2185insT	p.C728fs
XH_P37	2	179253857	179253857	In_Frame_Ins	INS	-	ATGAGAATATTC	-	OSBPL6	2186insATGAGA	.I729delinsNENIL
XH_P37	2	179341938	179341938	In_Frame_Ins	INS	-	GGAAAGGAA	-	FKBP7	3_224insTTCCT	p.R75delinsLPFR
XH_P37	2	179398216	179398216	Missense_Mutation	SNP	C	T	C	TTN	c.G75931A	p.E25311K
XH_P37	2	179399194	179399194	Frame_Shift_Ins	INS	-	ATGGGTGCTGGA	-	TTN	4953insCTCCAG	p.V24985fs
XH_P37	2	179413138	179413138	In_Frame_Ins	INS	-	TTCTGGGTTGAA	-	TTN	66020insTTCAA	22007delinsLQPRS
XH_P37	2	179418086	179418086	Frame_Shift_Ins	INS	-	TACAGGTTAAAC	-	TTN	346insATGTTTA	p.T20782fs
XH_P37	2	179424482	179424482	In_Frame_Ins	INS	-	AAGAAAGCATGC	-	TTN	59182insCCATG	19728delinsPCFLT
XH_P37	2	179435839	179435839	Frame_Shift_Ins	INS	-	AG	-	TTN	47824_47825ins	p.C15942fs
XH_P37	2	179435841	179435841	In_Frame_Ins	INS	-	ATGAGTTCC	-	TTN	2_47823insGGA	P15941delinsPELI
XH_P37	2	179437942	179437942	Frame_Shift_Ins	INS	-	CCTGAGACTT	-	TTN	_45722insAAGT	p.S15241fs
XH_P37	2	179449625	179449625	Nonsense_Mutation	INS	-	AGTT	-	TTN	7547_37548insA	2516_H12517delinsX
XH_P37	2	179449626	179449626	In_Frame_Ins	INS	-	CCATTTGGT	-	TTN	_37547insACCA	12516delinsYQMG
XH_P37	2	179449903	179449903	Nonsense_Mutation	INS	-	TACGTATCAAATC	-	TTN	AACAAGGATTTC	12458delinsRTRIX
XH_P37	2	179452445	179452445	In_Frame_Ins	INS	-	AAAATGAATTTG	-	TTN	96insTAACAAAT	.2132delinsINKFIFE
XH_P37	2	179522491	179522491	Nonsense_Mutation	INS	-	GATCTATCTCTT	-	TTN	877insACAAGAG	26_E12627delinsHKRX
XH_P37	2	179537423	179537423	Frame_Shift_Ins	INS	-	TGTTGCATGCAA	-	TTN	TAAAGGATTGCA	p.K10297fs
XH_P37	2	179574422	179574422	Frame_Shift_Ins	INS	-	ATGAGCATTTCTT	-	TTN	TATCATAAGAAA	p.I8298fs
XH_P37	2	179584475	179584475	Nonsense_Mutation	INS	-	AGATTTTTATGAA	-	TTN	TTGTATATTCATT	1_P6672delinsNIVYSX
XH_P37	2	179589227	179589227	Frame_Shift_Ins	INS	-	GAAGACTGAAAA	-	TTN	143insTATTTTTC	p.V5715fs
XH_P37	2	179604496	179604496	Frame_Shift_Ins	INS	-	TCTGACGAAAA	-	TTN	75insTGTTTTTC	p.L4125fs
XH_P37	2	179616165	179616165	Missense_Mutation	SNP	C	A	C	TTN	c.G10962T	p.M3654I
XH_P37	2	180348051	180348051	Frame_Shift_Ins	INS	-	CTTCACGGGAAC	-	ZNF385B	TAATCCTTCCCGT	p.A130fs
XH_P37	2	182543288	182543288	Frame_Shift_Ins	INS	-	CCTCGGAC	-	NEUROD1	9_300insGTCCG	p.K100fs
XH_P37	2	182766892	182766892	Nonsense_Mutation	INS	-	TCGAGTTAAAGG	-	SSFA2	54insTTCGAGTT	18_E219delinsTSSX
XH_P37	2	183053773	183053773	Nonsense_Mutation	INS	-	ATTTTTAAATGG	-	PDE1A	ATAGATCCATTT	2_E363delinsAPHRSIX
XH_P37	2	186657903	186657903	Nonsense_Mutation	INS	-	AAATTTGTTAATA	-	FSIP2	TTTCTAAATTTG	4_K2015delinsSSKSFX
XH_P37	2	186668085	186668085	Nonsense_Mutation	INS	-	ATAACTGCCTTCT	-	FSIP2	GTATAAAATAAC	.L4684delinsLVX
XH_P37	2	186668420	186668420	In_Frame_Ins	INS	-	ATTGGGCACGA	-	FSIP2	insTCTTTATTGG	796delinsNLYWARLP
XH_P37	2	186668422	186668422	Missense_Mutation	SNP	A	T	A	FSIP2	c.A14389T	p.I4797L
XH_P37	2	189867759	189867759	Nonsense_Mutation	INS	-	TAAAAACAAGGC	-	COL3A1	AAAATTTAAAAA	G843delinsEIKFKNKAFX
XH_P37	2	190339505	190339505	Frame_Shift_Ins	INS	-	GCCAAGAG	-	WDR75	9_2260insGCCA	p.N753fs
XH_P37	2	190339506	190339506	Nonsense_Mutation	INS	-	GAAA	-	WDR75	260_2261insGA	S754_L755delinsX
XH_P37	2	190531114	190531114	Frame_Shift_Ins	INS	-	CTTGATTTTGGT	-	ASNSD1	TATTTCTTGATT	p.E86fs
XH_P37	2	191302177	191302177	In_Frame_Ins	INS	-	CTGGTAATTCCT	-	MFSD6	insGATCTGGTA	474delinsDDLVIPV
XH_P37	2	192550490	192550490	Frame_Shift_Ins	INS	-	A	-	NABP1	c.612dupA	p.R204fs
XH_P37	2	192550492	192550492	Nonstop_Mutation	INS	-	TATTTTCAGAGG	-	NABP1	ATCACTATTTTCA	elinsYKNSSLFSEVVYRSR
XH_P37	2	192700824	192700824	Frame_Shift_Ins	INS	-	TAATCATTGACC	-	CAVIN2	CTATATGGTCAA	p.M368fs
XH_P37	2	196593033	196593033	Missense_Mutation	SNP	T	G	T	SLC39A10	c.T2297G	p.V766G
XH_P37	2	196593035	196593035	Frame_Shift_Ins	INS	-	AGCAAGCAATT	-	SLC39A10	CTGTAAAGCAAC	p.T767fs
XH_P37	2	196619155	196619155	Frame_Shift_Ins	INS	-	AG	-	DNAH7	11669_11670ins	p.Q3890fs
XH_P37	2	196619157	196619157	Frame_Shift_Ins	INS	-	ATATGAGCCGAC	-	DNAH7	nsAATGAGTCGG	p.Q3890fs
XH_P37	2	196737189	196737189	Nonsense_Mutation	INS	-	TTGAAATGGGAT	-	DNAH7	CACATAATCCCA	140_F2141delinsLTX
XH_P37	2	196866581	196866581	Nonsense_Mutation	INS	-	ATTTTTAATTATT	-	DNAH7	CTGAAATAATTA	1_F332delinsRCYFPLX
XH_P37	2	197565845	197565845	Nonsense_Mutation	INS	-	TATATTAACTTTT	-	CCDC150	TTTGTATATTA	delinsSLLVLYINFFCYVSX
XH_P37	2	197656112	197656112	Nonsense_Mutation	SNP	G	A	G	GTF3C3	c.C484T	p.R162X
XH_P37	2	198413128	198413128	Frame_Shift_Ins	INS	-	GTAAGGTTTATT	-	HSPE1-MOB4	AATAAGTAAGGT	p.Y120fs
XH_P37	2	200213653	200213653	In_Frame_Ins	INS	-	ATCTACCAGCAA	-	SATB2	944insTTGCTGG	H315delinsLAGRY
XH_P37	2	200826615	200826615	Frame_Shift_Ins	INS	-	GCAGCAGTTATA	-	MAIP1	sATTGGCAGCAG	p.N254fs
XH_P37	2	201437494	201437494	Nonsense_Mutation	INS	-	CTTCTTATTAACT	-	SGO2	6insAGCTTCTTA	809delinsKLLINFX
XH_P37	2	201437529	201437529	Frame_Shift_Ins	INS	-	GTCTTCC	-	SGO2	60_2461insGTC	p.P820fs
XH_P37	2	201437531	201437531	Nonsense_Mutation	INS	-	TCTGCCTAAGCT	-	SGO2	TTACTTTCTGCC	1_T822delinsDLLSAX
XH_P37	2	201438146	201438146	Frame_Shift_Ins	INS	-	TATGATAATGTC	-	SGO2	GTGTTTATTGAT	p.I1026fs
XH_P37	2	201857695	201857695	Frame_Shift_Ins	INS	-	TTTCCTTTTAGT	-	FAM126B	AAGTACTAAAA	p.E220fs
XH_P37	2	201862252	201862252	Frame_Shift_Ins	INS	-	GAGCTTGCATTC	-	FAM126B	insAGGAATGCA	p.L181fs
XH_P37	2	202357813	202357813	Nonsense_Mutation	INS	-	GGACATATCACCA	-	C2CD6	1insCTTGGTGA	84_S1085delinsPWX
XH_P37	2	202598158	202598158	Nonsense_Mutation	INS	-	CCAAGGATCATA	-	ALS2	21insACTATGAT	p.D807delinsELX
XH_P37	2	202938407	202938407	Missense_Mutation	SNP	G	C	G	KIAA2012	c.G57C	p.Q19H
XH_P37	2	202938408	202938408	Nonsense_Mutation	INS	-	GGCCTTTTATTAA	-	KIAA2012	insGGGCCTTTTA	20_L21delinsRAFYX
XH_P37	2	203160559	203160559	Frame_Shift_Ins	INS	-	GAATGCACCTCC	-	NOP58	TACAAAGAATGC	p.K357fs
XH_P37	2	204231691	204231691	In_Frame_Ins	INS	-	ACTGAACTCACTA	-	ABI2	TATTGACTGAAC	0delinsKLLTELTNVF
XH_P37	2	204305261	204305261	Frame_Shift_Ins	INS	-	AAAAAGACCAG	-	RAPH1	_2652insCTGGT	p.K884fs
XH_P37	2	205551005	205551005	In_Frame_Ins	INS	-	AATGACCTAATC	-	PARD3B	insTTTAATGACC	K74delinsKFNDLIY
XH_P37	2	205551007	205551007	Splice_Site	INS	-	TAAACACATTTTA	-	PARD3B	.	.
XH_P37	2	206921096	206921096	Frame_Shift_Ins	INS	-	AAAGATTCTGTA	-	INO80D	insAAATACAGA	p.L264fs
XH_P37	2	207174503	207174503	Nonsense_Mutation	INS	-	TCCACAGCTCTTA	-	ZDBF2	ATTATATCCACA	delinsPLEPLYPQLLSSESIX
XH_P37	2	207639093	207639093	Frame_Shift_Ins	INS	-	TCTATTAATTCTT	-	FASTKD2	ATCTGTTCTATT	p.N467fs
XH_P37	2	207655364	207655364	Missense_Mutation	SNP	C	A	C	FASTKD2	c.C1967A	p.A656D
XH_P37	2	207655366	207655366	Frame_Shift_Ins	INS	-	CTTAGTCCTTCA	-	FASTKD2	TTCCTGTCTTAGT	p.M657fs
XH_P37	2	207804365	207804365	Frame_Shift_Ins	INS	-	TACCATTT	-	CPO	42_43insTACCAT	p.L14fs
XH_P37	2	207804366	207804366	In_Frame_Ins	INS	-	ATATTTCATTTC	-	CPO	44insATATTTCA	.V15delinsDISFL
XH_P37	2	210558164	210558164	Nonsense_Mutation	INS	-	GTTTCTGTAACTA	-	MAP2	nsCGATGTTTCTC	0424delinsAMFLX
XH_P37	2	210559797	210559797	In_Frame_Ins	INS	-	AATGGGACTGTG	-	MAP2	nsTGGCAATGGG	68delinsDGNGTVYL
XH_P37	2	210685201	210685201	Frame_Shift_Ins	INS	-	ATCT	-	UNC80	2129_2130insAT	p.R710fs
XH_P37	2	210685202	210685202	In_Frame_Ins	INS	-	TTATTCTGTTTAA	-	UNC80	131insATTATTCT	R710delinsRIILFN

XH_P37	2	210787017	210787017	Nonsense_Mutation	INS	-	GTAGTTAATGAA	-	UNC80	TTGAAAGTAGTT	1792delinsKVESSX
XH_P37	2	211512680	211512680	Nonsense_Mutation	INS	-	TACTTTCTGGCAC	-	CPS1	AATTTTTACTTTC	_P629delinsKFLLSGTX
XH_P37	2	215645853	215645853	Frame_Shift_Ins	INS	-	TTTGTTAAGCA	-	BARD1	688insTGCTTAA	p.I230fs
XH_P37	2	215807703	215807703	Nonsense_Mutation	INS	-	TTTAAATTATTTT	-	ABCA12	28insATAAAATA	43_M2144delinsNKIIX
XH_P37	2	215807705	215807705	Nonsense_Mutation	INS	-	TGAATTTTTACTT	-	ABCA12	ATATTTAAGTAA	42_I2143delinsAKIFKX
XH_P37	2	215868957	215868957	Silent	SNP	G	A	G	ABCA12	c.C1705T	p.L569L
XH_P37	2	215868959	215868959	Frame_Shift_Ins	INS	-	ATAATGTAGGC	-	ABCA12	_1703insGCCTAC	p.E568fs
XH_P37	2	217364707	217364707	Frame_Shift_Ins	INS	-	AGACATCT	-	RPL37A	8_169insAGACA	p.H56fs
XH_P37	2	217364709	217364709	Frame_Shift_Ins	INS	-	GGAGT	-	RPL37A	170_171insGGA	p.C57fs
XH_P37	2	218674638	218674638	Frame_Shift_Ins	INS	-	TCTACCTTCT	-	TNS1	4806insAGAAG	p.S1602fs
XH_P37	2	218674639	218674639	Missense_Mutation	SNP	G	C	G	TNS1	c.C4805G	p.S1602C
XH_P37	2	219496954	219496954	Frame_Shift_Ins	INS	-	TAGCTGCTTGATA	-	PLCD4	59insATTAGCTGC	p.E456fs
XH_P37	2	219562294	219562294	Nonsense_Mutation	INS	-	AAAAAAATAAAC	-	STK36	2808insAAAAAAA	36_L937delinsQKKX
XH_P37	2	219870919	219870919	Frame_Shift_Ins	INS	-	CCAACCCA	-	CFAP65	5_4746insTGGG	p.Q1582fs
XH_P37	2	220037818	220037818	Frame_Shift_Ins	INS	-	ACTC	-	CNPPD1	722_723insGAG	p.S241fs
XH_P37	2	220045811	220045811	In_Frame_Ins	INS	-	CCACAGTTACTAA	-	RETREG2	TCTTCCACAGTTA	16delinsVSLPQLLILK
XH_P37	2	220103443	220103443	In_Frame_Ins	INS	-	CTCTTTTATTTCT	-	GLB1L	AAACAGAAATAA	284delinsQTEIKEDT
XH_P37	2	223773815	223773815	Nonsense_Mutation	INS	-	TTCATAAGTACT	-	ACSL3	sGTTATTCATAA	09_V110delinsGYSX
XH_P37	2	225360650	225360650	Frame_Shift_Ins	INS	-	AGTCTTTCTTATT	-	CUL3	543insAATAAGA	p.Q515fs
XH_P37	2	225360652	225360652	Frame_Shift_Ins	INS	-	TAACCTTTAGAGA	-	CUL3	41insACTCTCTAA	p.A514fs
XH_P37	2	227662984	227662984	In_Frame_Ins	INS	-	GGATGA	-	IRS1	470_471insTCAT	p.G157delinsGHP
XH_P37	2	227662985	227662985	Frame_Shift_Ins	INS	-	T	-	IRS1	c.469_470insA	p.G157fs
XH_P37	2	227886767	227886767	Nonsense_Mutation	INS	-	CACATCCCTTCTA	-	COL4A4	AAATAATAGAAG	p.P1405delinsKX
XH_P37	2	228860402	228860402	Nonsense_Mutation	INS	-	TCTAAAAATGCA	-	SPHKAP	TTAAAATGCATT	486_D1487delinsHX
XH_P37	2	228884708	228884708	Nonsense_Mutation	INS	-	GCTTCTGAATGA	-	SPHKAP	TATTATCATTCAG	N289delinsSHTLLSFRSFX
XH_P37	2	230662505	230662505	Nonsense_Mutation	INS	-	GACTTTTACCTT	-	TRIP12	90insATAAGGTA	830_V831delinsLX
XH_P37	2	231256899	231256899	Frame_Shift_Ins	INS	-	GTCTGGCCTTC	-	SP140L	883insGTCTGG	p.R294fs
XH_P37	2	231338147	231338147	Frame_Shift_Ins	INS	-	TTTTTCTTTTGTG	-	SP100	AATCTCTTTTTCT	p.D513fs
XH_P37	2	231338149	231338149	In_Frame_Ins	INS	-	CTGTAT	-	SP100	540_1541insCTG	p.M514delinsTVL
XH_P37	2	231927370	231927370	Nonsense_Mutation	INS	-	TAGGAAGTACAG	-	PSMD1	AAATTAGGAAG	95_Y96delinsERNX
XH_P37	2	232458083	232458083	Frame_Shift_Ins	INS	-	TGGAA	-	TEX44	421_422insTGGA	p.P141fs
XH_P37	2	232458084	232458084	Frame_Shift_Ins	INS	-	AACTGCACTCT	-	TEX44	_423insAACTGC	p.P141fs
XH_P37	2	233431616	233431616	Frame_Shift_Ins	INS	-	TCATATCACTTT	-	EIF4E2	AGAAGTCATATC	p.L106fs
XH_P37	2	233636012	233636012	Frame_Shift_Ins	INS	-	A	-	KCNJ13	c.60_61insT	p.V21fs
XH_P37	2	233636013	233636013	In_Frame_Ins	INS	-	ACTCACAATTGG	-	KCNJ13	0insAACCAATTG	M20delinsITNCEL
XH_P37	2	234186258	234186258	Frame_Shift_Ins	INS	-	ATTATCATTTTAT	-	ATG16L1	ATTCTATTATCAT	p.G170fs
XH_P37	2	234474132	234474132	In_Frame_Ins	INS	-	TGGGTGATGACA	-	USP40	ATTATTGTCATC	35delinsNIIVITQDI
XH_P37	2	234526800	234526800	Frame_Shift_Ins	INS	-	GTATGAGGTTGA	-	UGT1A8	AGAGTGTATGAG	p.P149fs
XH_P37	2	234602146	234602146	Frame_Shift_Ins	INS	-	GG	-	UGT1A6	c.496_497insGG	p.P166fs
XH_P37	2	234602147	234602147	Frame_Shift_Ins	INS	-	TAGATTTTTCTTC	-	UGT1A6	CTGGATAGATTT	p.P166fs
XH_P37	2	234681087	234681087	Frame_Shift_Ins	INS	-	TGTAA	-	UGT1A1	484_1485insTGT	p.L495fs
XH_P37	2	234681088	234681088	Nonsense_Mutation	INS	-	AACACAAAGACA	-	UGT1A1	36insTAAACACA	495_L496delinsLX
XH_P37	2	234698270	234698270	In_Frame_Ins	INS	-	ATTCCTCCATTTC	-	MROH2A	05insCATTCCTC	P135delinsPIPPFL
XH_P37	2	234746295	234746295	Frame_Shift_Ins	INS	-	GC	-	HJURP	1919_1920insG	p.G640fs
XH_P37	2	238257003	238257003	Frame_Shift_Ins	INS	-	T	-	COL6A3	.5133_5134insA	p.P1712fs
XH_P37	2	238257004	238257004	Nonsense_Mutation	INS	-	TGAGATTTAAAT	-	COL6A3	insTTTTATTTAA	711_P1712delinsYFIX
XH_P37	2	238662004	238662004	Frame_Shift_Ins	INS	-	TGGAAATCCCCC	-	LRRFIP1	69insATGGAAAT	p.A123fs
XH_P37	2	238729809	238729809	In_Frame_Ins	INS	-	TGT	-	RBM44	2012_2013insTG	p.K671delinsNV
XH_P37	2	238729810	238729810	Nonsense_Mutation	INS	-	AGAGACGGGGC	-	RBM44	insTTGTAGAGAC	671_I672delinsKLX
XH_P37	2	240957991	240957991	Frame_Shift_Ins	INS	-	T	-	NDUFA10	c.525_526insA	p.Q176fs
XH_P37	2	240957992	240957992	Frame_Shift_Ins	INS	-	AAAGCAGA	-	NDUFA10	24_525insTCTGC	p.N175fs
XH_P37	2	242026822	242026822	Nonsense_Mutation	SNP	C	A	C	SNED1	c.C4137A	p.Y1379X
XH_P37	2	242026823	242026823	Frame_Shift_Ins	INS	-	GTGATTTGTAAT	-	SNED1	GGTCATGTGATT	p.R1380fs
XH_P37	2	242373658	242373658	Frame_Shift_Ins	INS	-	GATAGGGAATT	-	FARP2	954insGATAGG	p.T318fs
XH_P37	2	242373659	242373659	Nonsense_Mutation	INS	-	ATTAAAAATCAGT	-	FARP2	CAAAATTAAAAA	T318delinsTQNX
XH_P37	2	242501866	242501866	Frame_Shift_Ins	INS	-	T	-	BOK	c.324_325insT	p.A108fs
XH_P37	2	242501867	242501867	In_Frame_Ins	INS	-	TTCCTCACT	-	BOK	5_326insTTCCTC	V109delinsVPHL
XH_P37	3	9489446	9489446	Nonsense_Mutation	INS	-	ATTCACTTTAAG	-	SETD5	60insCTATTCAC	20_R621delinsSYSLX
XH_P37	3	10084834	10084834	Nonsense_Mutation	INS	-	ATGTTGGACATT	-	FANCD2	TTTAAATGTTGG	0delinsRKKFKCWTFX
XH_P37	3	12641888	12641888	Nonsense_Mutation	INS	-	CCTGCCAATTAG	-	RAF1	861insCTAATTG	p.S287delinsSX
XH_P37	3	12641890	12641890	Missense_Mutation	SNP	A	G	A	RAF1	c.T859C	p.S287P
XH_P37	3	16310760	16310760	In_Frame_Ins	INS	-	ATTTTTCTTTTTTC	-	OXNAD1	TCCATTTTTCTTT	3delinsPCFHFSFLIIY
XH_P37	3	16312474	16312474	In_Frame_Ins	INS	-	TTG	-	OXNAD1	c.69_70insTTG	p.A23delinsAL
XH_P37	3	16312476	16312476	Nonsense_Mutation	INS	-	ATAATAGCACCA	-	OXNAD1	ACTCATAATAGC	p.V24delinsVVTX
XH_P37	3	17052979	17052979	Frame_Shift_Ins	INS	-	ACGACACC	-	PLCL2	3_1764insACGA	p.N588fs
XH_P37	3	17052980	17052980	In_Frame_Ins	INS	-	GAA	-	PLCL2	1764_1765insGA	p.N588delinsNE
XH_P37	3	20216412	20216412	Nonsense_Mutation	INS	-	AAGTAAGCAAA	-	SGO1	11insTCTTTGCT	Q204delinsLFAYFX
XH_P37	3	25778885	25778885	Frame_Shift_Ins	INS	-	GT	-	NGLY1	c.942_943insAC	p.C315fs
XH_P37	3	25778886	25778886	Frame_Shift_Ins	INS	-	ATACCAACCTTCC	-	NGLY1	sACCTGGAAGGT	p.N314fs
XH_P37	3	25820121	25820121	In_Frame_Ins	INS	-	TCTTTAAAATAAC	-	NGLY1	sGACCCTTATTTT	64delinsDPYFKELR
XH_P37	3	25833425	25833425	Nonsense_Mutation	INS	-	ATACAAGAATC	-	OXSM	915insATACAAG	305_G306delinsX
XH_P37	3	28295256	28295256	In_Frame_Ins	INS	-	ATTTGGATCCCTC	-	CMC1	TCTATATTTGGA	1delinsIVSIFGSLFFVY
XH_P37	3	29628622	29628622	Nonsense_Mutation	INS	-	GTGGTCCTTAATC	-	RBMS3	3insAGTGGTCC	p.F108delinsX
XH_P37	3	32576104	32576104	Frame_Shift_Ins	INS	-	A	-	DYNC1LI1	c.518_519insT	p.E173fs
XH_P37	3	32576105	32576105	Nonsense_Mutation	INS	-	CTTGATTTATCTA	-	DYNC1LI1	ATAATAGATAAA	E173delinsGNNRX
XH_P37	3	33135360	33135360	Frame_Shift_Ins	INS	-	CTCTCAGACA	-	TMPPE	_328insTGTCTG	p.E110fs
XH_P37	3	37034060	37034060	Frame_Shift_Ins	INS	-	TTGGAATGTCAT	-	EPM2AIP1	TAATGAATGACA	p.R170fs
XH_P37	3	37365907	37365907	In_Frame_Ins	INS	-	GATGATTTTTCTG	-	GOLGA4	1insGTGATGAT	344delinsGDDFSVQ
XH_P37	3	37367511	37367511	In_Frame_Ins	INS	-	GAG	-	GOLGA4	4134_4135insGA	p.D1378delinsDE
XH_P37	3	37367512	37367512	Nonsense_Mutation	INS	-	AGCC	-	GOLGA4	4135_4136insAG	1379_N1380delinsX
XH_P37	3	38037304	38037304	Missense_Mutation	SNP	A	G	A	VILL	c.A416G	p.H139R
XH_P37	3	38224579	38224579	Frame_Shift_Ins	INS	-	ATGCTCTAAAGA	-	OXSR1	57insAATGCTCTA	p.K52fs
XH_P37	3	38438616	38438616	Frame_Shift_Ins	INS	-	GCCTTAAGAGGA	-	XYLB	nsACTGCCTTAAC	p.S331fs
XH_P37	3	39111231	39111231	Frame_Shift_Ins	INS	-	A	-	WDR48	c.422_423insA	p.T141fs
XH_P37	3	39111232	39111232	Frame_Shift_Ins	INS	-	CCTCTTTATCTTG	-	WDR48	424insCCTCTTTA	p.T141fs
XH_P37	3	40557737	40557737	In_Frame_Ins	INS	-	TCCTCTTTG	-	ZNF620	0_311insTCCTCT	.H104delinsLLFD
XH_P37	3	40557738	40557738	Frame_Shift_Ins	INS	-	TTTGTCTTAATTA	-	ZNF620	12insCTTTGTCTT	p.H104fs
XH_P37	3	42661181	42661181	Frame_Shift_Ins	INS	-	GTAAAATATATG	-	NKTR	CATCTGTAAAAT	p.I89fs
XH_P37	3	42674292	42674292	In_Frame_Ins	INS	-	GTTTCTTCCTTTT	-	NKTR	GGTTGGTTTCTTC	50delinsNELVSSFFSQ
XH_P37	3	42676851	42676851	Nonsense_Mutation	INS	-	TTTAGTTTTTAAC	-	NKTR	TTTTTTTTTAGTT	D387delinsVLFFFFFSFX
XH_P37	3	42678521	42678521	Frame_Shift_Ins	INS	-	TCATGCGGTGAT	-	NKTR	TAATATTCATGCC	p.G442fs
XH_P37	3	42681052	42681052	Frame_Shift_Ins	INS	-	TAGCCAAGCTA	-	NKTR	3857insTAGCCA	p.R1286fs
XH_P37	3	43074841	43074841	Missense_Mutation	SNP	C	A	C	FAM198A	c.C1086A	p.D362E
XH_P37	3	43344817	43344817	Nonsense_Mutation	INS	-	AAAATAGATCTTC	-	SNRK	TGTTTAAAATAG	LdelinsKIFRLFKIDLLIX
XH_P37	3	44285337	44285337	Frame_Shift_Ins	INS	-	TGCTTCTTTACCA	-	TOPAZ1	sGTCTCTGCTTCT	p.K447fs
XH_P37	3	44763426	44763426	Nonsense_Mutation	INS	-	TGATGAATAAGC	-	ZNF502	sTCTGATGATGA	I373_T374delinsLX
XH_P37	3	44763727	44763727	Frame_Shift_Ins	INS	-	TTATATGGTTTT	-	ZNF502	TTACACTTATATC	p.A473fs
XH_P37	3	45436140	45436140	Frame_Shift_Ins	INS	-	AACCCAATCTCTC	-	LARS2	196insAACCCAAT	p.Q65fs

XH_P37	3	45988901	45988901	Nonsense_Mutation	INS	-	AGCAGTGTTTTC	-	CXCR6	CATGAAGCAGTC	_Y311delinsQHEAVFX
XH_P37	3	46063154	46063154	Frame_Shift_Ins	INS	-	AACCTCTCTT	-	XCR1	286insAAGAAG	p.V96fs
XH_P37	3	47125629	47125629	Frame_Shift_Ins	INS	-	GGAGTAGCTGT	-	SETD2	GTCACAACAGC	p.L1837fs
XH_P37	3	47165686	47165686	Frame_Shift_Ins	INS	-	CAGGACACTATA	-	SETD2	ATTAGATATAGT	p.H103fs
XH_P37	3	47318793	47318793	Frame_Shift_Ins	INS	-	ACACAGAAGA	-	KIF9	_72insTCTTCTG	p.I24fs
XH_P37	3	47318795	47318795	Nonsense_Mutation	INS	-	TGACATTTGTTCA	-	KIF9	70insTGAACAAA	.I24_R25delinsX
XH_P37	3	47969822	47969822	In_Frame_Ins	INS	-	TATTTTAAATACA	-	MAP4	ATAATGTATTTA	04delinsHNVFKIGF
XH_P37	3	48466010	48466010	Missense_Mutation	SNP	A	T	A	PLXNB1	c.T11A	p.L4Q
XH_P37	3	48466012	48466012	Silent	SNP	A	G	A	PLXNB1	c.T9C	p.A3A
XH_P37	3	48607914	48607914	Frame_Shift_Ins	INS	-	TGAACCTAATGT	-	COL7A1	64insTGACATTA	p.G2455fs
XH_P37	3	48618052	48618052	Missense_Mutation	SNP	T	C	T	COL7A1	c.A5014G	p.K1672E
XH_P37	3	48697796	48697796	Nonsense_Mutation	INS	-	GATCACTGCTA	-	CELSR3	2272insTAGCA	758_K759delinsX
XH_P37	3	49049990	49049990	Frame_Shift_Ins	INS	-	ACTGATAAGGTA	-	WDR6	46insACTGATA	p.C315fs
XH_P37	3	49064039	49064039	Missense_Mutation	SNP	A	T	A	IMPDH2	c.T823A	p.S275T
XH_P37	3	49064040	49064040	In_Frame_Ins	INS	-	GCTCCATCTGCA	-	IMPDH2	822insTGCAGAT	274delinsDADGA
XH_P37	3	49095207	49095207	In_Frame_Ins	INS	-	CTT	-	QRICH1	.425_426insAA	.Q142delinsQR
XH_P37	3	49095209	49095210	Frame_Shift_Del	DEL	GG	-	GG	QRICH1	c.423_424del	p.G141fs
XH_P37	3	49308675	49308675	Frame_Shift_Ins	INS	-	TTTGAATTCCAT	-	C3orf62	2insCAATGGAAT	p.T248fs
XH_P37	3	49841923	49841923	In_Frame_Ins	INS	-	AGACTGAGTCCT	-	FAM212A	368insAGACTGA	P123delinsQTESS
XH_P37	3	50005731	50005731	In_Frame_Ins	INS	-	CTG	-	RBM6	.873_874insCT	p.N291delinsNL
XH_P37	3	50005733	50005733	Frame_Shift_Ins	INS	-	AAATCCC	-	RBM6	75_876insAAAT	p.I292fs
XH_P37	3	50597133	50597133	Nonsense_Mutation	INS	-	CTTTAGACTCTA	-	C3orf18	44insTTAGAGTC	148_S149delinsPX
XH_P37	3	51265475	51265475	Frame_Shift_Ins	INS	-	A	-	DOCK3	c.1603_1604ins	p.G535fs
XH_P37	3	51265476	51265476	Nonsense_Mutation	INS	-	GCTGAAATGAGT	-	DOCK3	AATGTGCTGAA	5_T536delinsGNVLKX
XH_P37	3	52783733	52783733	Missense_Mutation	SNP	A	G	A	NEK4	c.T1214C	p.I405T
XH_P37	3	53326507	53326507	Frame_Shift_Ins	INS	-	TCTCCAGAAAC	-	DCP1A	498insGTTTCTG	p.E166fs
XH_P37	3	53326812	53326812	In_Frame_Ins	INS	-	CCCCAGCTT	-	DCP1A	_193insAAGCTC	.G65delinsKLGG
XH_P37	3	56647774	56647774	Frame_Shift_Ins	INS	-	ATCTAAGCAGGA	-	CCDC66	461insATCTAAG	p.K487fs
XH_P37	3	56651516	56651516	Frame_Shift_Ins	INS	-	TTGTTTGTTTCTC	-	CCDC66	sGTGTTTTGTTT	p.H706fs
XH_P37	3	57303683	57303683	Frame_Shift_Ins	INS	-	TCTGTCATAAAT	-	APPL1	ACTTCTTCTGTC	p.E700fs
XH_P37	3	58090920	58090920	Nonsense_Mutation	INS	-	TATGTCAACAAT	-	FLNB	TACTGTTATGTC	75delinsIATVMSTIX
XH_P37	3	58853662	58853662	Nonsense_Mutation	INS	-	TTAAATAGACAG	-	C3orf67	1insTACTGTCTA	84_A285delinsVLSIX
XH_P37	3	58853664	58853664	Splice_Site	SNP	T	C	T	C3orf67	.	.
XH_P37	3	63649762	63649762	In_Frame_Ins	INS	-	GGCTTACAATTCT	-	SNTN	36insTGGCTTAC	I145delinsIWLTIL
XH_P37	3	63973833	63973833	Silent	SNP	G	A	G	ATXN7	c.G759A	p.L253L
XH_P37	3	65369274	65369274	Frame_Shift_Ins	INS	-	CTTTCTAAAAAGT	-	MAGI1	ATACAAACTTTT	p.I814fs
XH_P37	3	69153769	69153769	Frame_Shift_Ins	INS	-	ACTCCCTCTCTTC	-	ARL6IP5	ATTACACTCCCT	p.I183fs
XH_P37	3	69273839	69273839	In_Frame_Ins	INS	-	GCCTCCATTCAGA	-	FRMD4B	4insTTCTGAATC	195delinsVLNGGY
XH_P37	3	77623837	77623837	Frame_Shift_Ins	INS	-	TCCTCATAACTAT	-	ROBO2	TTTCTGTCCTCAT	p.Q720fs
XH_P37	3	77651424	77651424	Missense_Mutation	SNP	T	C	T	ROBO2	c.T2918C	p.I973T
XH_P37	3	78685062	78685062	Frame_Shift_Ins	INS	-	AAACTTGTCTGCC	-	ROBO1	insAGTCGCAGA	p.L978fs
XH_P37	3	78706363	78706363	Nonsense_Mutation	INS	-	AGCTTTACTTAAT	-	ROBO1	1insATATTAAGT	797_T798delinsSYX
XH_P37	3	78717697	78717697	Frame_Shift_Ins	INS	-	TATGTTATCTGCA	-	ROBO1	sATAAATGCAGA	p.Y487fs
XH_P37	3	78719447	78719447	Frame_Shift_Ins	INS	-	GATCCGAT	-	ROBO1	8_1239insATCG	p.I413fs
XH_P37	3	78766995	78766995	Nonsense_Mutation	INS	-	AGACAGTCAGC	-	ROBO1	GCCGAGCTGAC	.K193delinsKPSX
XH_P37	3	93733357	93733357	Nonsense_Mutation	INS	-	AGTTGTAATTTTC	-	STX19	insGGGAAAATT	3_V254delinsGKITTX
XH_P37	3	97655736	97655736	Frame_Shift_Ins	INS	-	AAAGTATCTGCA	-	CRYBG3	sCATCAAAAGTA	p.L2830fs
XH_P37	3	98110040	98110040	Frame_Shift_Ins	INS	-	G	-	OR5K3	c.531_532insG	p.F177fs
XH_P37	3	98110041	98110041	In_Frame_Ins	INS	-	AAAAGAAGTTCT	-	OR5K3	CTCAGAAAAGAA	178delinsSSEKKFSNI
XH_P37	3	98518535	98518535	In_Frame_Ins	INS	-	AGCCAG	-	DCBLD2	08_2009insCTG	p.L670delinsPGF
XH_P37	3	100536328	100536328	Nonsense_Mutation	INS	-	TATCT	-	ABI3BP	697_2698insAG	900_L901delinsRX
XH_P37	3	100536330	100536330	Nonsense_Mutation	INS	-	TCATCTTCTGC	-	ABI3BP	2696insGCAGA	99_V900delinsSRRX
XH_P37	3	100551174	100551174	Frame_Shift_Ins	INS	-	TGTACATATAAT	-	ABI3BP	993insGATTATA	p.R665fs
XH_P37	3	100551176	100551176	Missense_Mutation	SNP	T	A	T	ABI3BP	c.A1991T	p.K664I
XH_P37	3	101476792	101476792	Nonsense_Mutation	INS	-	TCATTTCGAGAA	-	CEP97	insAGATCATTTC	389_D390delinsX
XH_P37	3	105271357	105271357	Nonsense_Mutation	INS	-	AAAATTACTTAT	-	ALCAM	GCTAAGAAAATT	518_I519delinsGSX
XH_P37	3	105423000	105423000	In_Frame_Ins	INS	-	CCCACATTCATA	-	CBLB	1278insTATGAA	I426delinsNMNVG
XH_P37	3	107097035	107097035	In_Frame_Ins	INS	-	GTCACCTTCTTCT	-	CCDC54	insCTGTCACCTT	201delinsSVTFFFN
XH_P37	3	107097037	107097037	Frame_Shift_Ins	INS	-	G	-	CCDC54	c.603_604insG	p.Y201fs
XH_P37	3	107492134	107492134	Frame_Shift_Ins	INS	-	TCTTCTTTTTCTT	-	BBX	nsCTTTTCTTCTT	p.L522fs
XH_P37	3	107492136	107492136	Missense_Mutation	SNP	A	C	A	BBX	c.A1568C	p.D523A
XH_P37	3	108281996	108281996	Frame_Shift_Ins	INS	-	TAAATGTTCTTCA	-	KIAA1524	611insTGAAGA	p.P537fs
XH_P37	3	110837698	110837698	Nonsense_Mutation	INS	-	GACAACTGCATA	-	NECTIN3	TAAGGACAACT	233delinsLKDNCIX
XH_P37	3	111764725	111764725	Frame_Shift_Ins	INS	-	GTTATATGTATTC	-	TMPRSS7	288insGTTATAT	p.I96fs
XH_P37	3	111764727	111764727	Missense_Mutation	SNP	A	G	A	TMPRSS7	c.A289G	p.N97D
XH_P37	3	112299933	112299933	Nonsense_Mutation	INS	-	AGCCATTGAAGA	-	SLC35A5	ATATTAGCCATT	I205delinsIIDISHX
XH_P37	3	113120579	113120579	Nonsense_Mutation	INS	-	GTTTTTATTAAAT	-	CFAP44	ATTGAAATTTAA	393_F394delinsVX
XH_P37	3	113169309	113169309	Nonsense_Mutation	INS	-	GAAATATGAAGC	-	SPICE1	CTGAAAACCTTC	34delinsSRKLKTFIFLVFKSX
XH_P37	3	113187735	113187735	Nonsense_Mutation	INS	-	ATGTCTCCTACCA	-	SPICE1	CATGTGGTAGG	55_T256delinsYMWX
XH_P37	3	118865982	118865982	Frame_Shift_Ins	INS	-	TC	-	C3orf30	c.946_947insTC	p.E316fs
XH_P37	3	118865984	118865984	Frame_Shift_Ins	INS	-	T	-	C3orf30	c.948_949insT	p.E316fs
XH_P37	3	119156952	119156952	Splice_Site	INS	-	CATTCGCAATGAA	-	TMEM39A	.	.
XH_P37	3	120050121	120050121	Frame_Shift_Ins	INS	-	TG	-	LRRC58	.1041_1042insC	p.S348fs
XH_P37	3	120050122	120050122	In_Frame_Ins	INS	-	ATAAAACATTTTC	-	LRRC58	041insACAAAAT	347delinsTQNVLY
XH_P37	3	120424944	120424944	Nonsense_Mutation	INS	-	CTTTTCTTCTATGC	-	RABL3	CAATCCATAGAA	6delinsKSTIHRRKEEX
XH_P37	3	121151774	121151774	Frame_Shift_Ins	INS	-	AGAAGAGTATGA	-	POLQ	insCATCTCATAC	p.D2550fs
XH_P37	3	121151776	121151776	Nonsense_Mutation	INS	-	TTTTTATTTA	-	POLQ	_7648insTAAAT	2550_V2551delinsX
XH_P37	3	121200540	121200540	Nonsense_Mutation	INS	-	TATCAAAAA	-	POLQ	9_6090insTTTTT	.Q2030delinsHFX
XH_P37	3	121200542	121200542	Frame_Shift_Ins	INS	-	GGTA	-	POLQ	6087_6088insTA	p.Q2030fs
XH_P37	3	121208048	121208048	Frame_Shift_Ins	INS	-	GAAGATAGTTTCT	-	POLQ	CCAGATAGAAAC	p.T1244fs
XH_P37	3	121208707	121208707	Frame_Shift_Ins	INS	-	CTAAAAATGTAT	-	POLQ	071insGATACAT	p.K1024fs
XH_P37	3	121208709	121208709	In_Frame_Ins	INS	-	TGAGTG	-	POLQ	068_3069insCAC	.T1023delinsTTQ
XH_P37	3	121256034	121256034	Nonsense_Mutation	INS	-	GTAATATTTTTC	-	POLQ	CAATAAGAAAAA	18_H219delinsYYTX
XH_P37	3	121340958	121340958	Missense_Mutation	SNP	G	T	G	FBXO40	c.G682T	p.A228S
XH_P37	3	121341223	121341223	Missense_Mutation	SNP	G	A	G	FBXO40	c.G947A	p.R316Q
XH_P37	3	121412681	121412681	In_Frame_Ins	INS	-	TGG	-	GOLGB1	6448_6449insCC	p.E2150delinsAK
XH_P37	3	121412682	121412682	Nonsense_Mutation	INS	-	TGTCCATGGAAC	-	GOLGB1	nsTATATCTTCCA	_I2151delinsYIFHGHLX
XH_P37	3	121508920	121508920	Nonsense_Mutation	INS	-	TTTCTTATCCTTC	-	IQCB1	ATCTGAAGGATA	p.G244delinsIIX
XH_P37	3	122496735	122496735	Nonsense_Mutation	INS	-	TGTTTTTGTCTG	-	HSPBAP1	TATACAGACAAA	.F28_K29delinsX
XH_P37	3	124457079	124457079	In_Frame_Ins	INS	-	ATAGACACAGAT	-	UMPS	976insATAGACA	Q325delinsQIDTD
XH_P37	3	124536550	124536550	Frame_Shift_Ins	INS	-	CTCTTCT	-	ITGB5	45_1046insAGA	p.T349fs
XH_P37	3	124629344	124629344	Missense_Mutation	SNP	A	C	A	MUC13	c.T1252G	p.F418V
XH_P37	3	124646578	124646578	Frame_Shift_Ins	INS	-	GCACCGCTTTA	-	MUC13	312insTAAAGC	p.D104fs
XH_P37	3	124731653	124731653	Nonsense_Mutation	INS	-	CTAAAATGCTTC	-	HEG1	0insTAGGAAGCA	p.A924delinsX
XH_P37	3	124896777	124896777	Frame_Shift_Ins	INS	-	TTGTAGGTGTGA	-	SLC12A8	432insTCACACCT	p.S144fs
XH_P37	3	124951520	124951520	Frame_Shift_Ins	INS	-	AATTTCCCTTGGT	-	ZNF148	CATTCACCAAGC	p.Q684fs
XH_P37	3	126190998	126190998	Nonsense_Mutation	INS	-	GTTTTTTCTGTGT	-	ZXDC	AAGTATACACAG	_G354delinsKSIHRKNX
XH_P37	3	128627939	128627939	Silent	SNP	T	G	T	ACAD9	c.T1482G	p.L494L

XH_P37	3	129156119	129156119	Nonsense_Mutation	INS	-	AAAAGGATGTG	-	MBD4	8insAAACACATG	S123_L124delinsX
XH_P37	3	129218800	129218800	Missense_Mutation	SNP	C	A	C	IFT122	c.C1931A	p.A644D
XH_P37	3	129289773	129289773	Frame_Shift_Ins	INS	-	AGCAGAGCTTTA	-	PLXND1	06insTGTAAAGC	p.E1202fs
XH_P37	3	129290596	129290596	Frame_Shift_Ins	INS	-	CACATTG	-	PLXND1	58_3169insCAAT	p.V1057fs
XH_P37	3	130188306	130188306	Frame_Shift_Ins	INS	-	ATTACAAAGATA	-	COL6A5	CAGATATTACAA	p.P2486fs
XH_P37	3	130300705	130300705	Frame_Shift_Ins	INS	-	AAGGAAAAGCAT	-	COL6A6	849insAAAGGAA	p.S1283fs
XH_P37	3	130405224	130405224	Nonsense_Mutation	INS	-	AGTTTTTTCCTCT	-	PIK3R4	sACAAAGAGGA	102delinsQQRGKNX
XH_P37	3	130447527	130447527	Frame_Shift_Ins	INS	-	ATAGTATAGTTG	-	PIK3R4	AACATACCAACTA	p.E529fs
XH_P37	3	130673854	130673854	Splice_Site	INS	-	TGATTTAAACTCT	-	ATP2C1	.	.
XH_P37	3	130686063	130686063	Frame_Shift_Ins	INS	-	AAAACTATAAT	-	ATP2C1	1183insAAAACT	p.P394fs
XH_P37	3	130686064	130686064	Frame_Shift_Ins	INS	-	AGTT	-	ATP2C1	.183_1184insAG	p.A395fs
XH_P37	3	132166265	132166265	In_Frame_Ins	INS	-	CAATATGTATTC	-	DNAJC13	insATCCAATATG	82delinsTSNMYSY
XH_P37	3	132400801	132400801	Nonsense_Mutation	INS	-	AAAAGAATTTTC	-	NPHP3	TAATAGGAAAAT	T1317delinsYNRKILLNNX
XH_P37	3	133379918	133379918	Nonsense_Mutation	INS	-	AAGTACATCATTA	-	TOPBP1	TACATAATGATG	p.D18delinsDTX
XH_P37	3	134911560	134911560	Frame_Shift_Ins	INS	-	AT	-	EPHB1	.2025_2026insA	p.F675fs
XH_P37	3	134911561	134911561	In_Frame_Ins	INS	-	CAAAAGGATTAC	-	EPHB1	AAACACAAAAG	76delinsGKHKRITKVH
XH_P37	3	135721304	135721304	Nonsense_Mutation	INS	-	TTTATGCATTGT	-	PPP2R3A	TTTTCTTTATGC	p.F322delinsX
XH_P37	3	138256168	138256168	Nonsense_Mutation	INS	-	TTTTTTGTTCTAG	-	CEP70	CCTCTAGAACAA	p.K11delinsDLX
XH_P37	3	138762838	138762838	Frame_Shift_Ins	INS	-	TAGATGCTTTTTT	-	PRR23C	insTCAAAAAAG	p.R209fs
XH_P37	3	140675488	140675488	In_Frame_Ins	INS	-	TTTGCCAACATA	-	SLC25A36	162insTTTGCCA	.A54delinsALPTY
XH_P37	3	140692776	140692776	Nonsense_Mutation	INS	-	TTACTGAT	-	SLC25A36	71_672insTTACT	224_D225delinsSYX
XH_P37	3	140692778	140692778	Nonsense_Mutation	INS	-	TTTAAAGTAAGTA	-	SLC25A36	GTTTTTTAAAGT	D225delinsGFLKX
XH_P37	3	141164711	141164711	Frame_Shift_Ins	INS	-	CTCCAGTATGGA	-	ZBTB38	nsCTTTTCTCCAG	p.V1161fs
XH_P37	3	142272215	142272215	Nonsense_Mutation	INS	-	ATATTTATACAT	-	ATR	ATAAAACATGTA	887_A888delinsKX
XH_P37	3	142522994	142522994	Nonsense_Mutation	INS	-	TGTAAAGACAAT	-	TRPC1	insACCTGTAAAG	611_H612delinsHLX
XH_P37	3	142746017	142746017	Nonsense_Mutation	INS	-	TTAATACCAAAAA	-	U2SURP	5insTTAATACCA	M25_I26delinsIX
XH_P37	3	142746018	142746018	Nonsense_Mutation	INS	-	TAACCTGGAGCA	-	U2SURP	76insTAACCTGG	M25_I26delinsMX
XH_P37	3	143412145	143412145	Frame_Shift_Ins	INS	-	AA	-	SLC9A9	c.537_538insTT	p.I180fs
XH_P37	3	143412147	143412147	Nonsense_Mutation	INS	-	TTTCTATTTTTCT	-	SLC9A9	insGAAGAAAAA	L179_I180delinsX
XH_P37	3	143691687	143691687	Silent	SNP	C	T	C	C3orf58	c.C513T	p.P171P
XH_P37	3	147113798	147113798	Frame_Shift_Ins	INS	-	TGGGGAAG	-	ZIC4	78_679insCTTCC	p.E227fs
XH_P37	3	148459207	148459207	Frame_Shift_Ins	INS	-	CGAATTTGTTGCA	-	AGTR1	insGTCGAATTTG	p.A129fs
XH_P37	3	148714156	148714156	In_Frame_Ins	INS	-	TGATGCTTT	-	GYG1	1_212insTGATG	p.H71delinsLMLY
XH_P37	3	148930338	148930338	Frame_Shift_Ins	INS	-	AACTATTTTTTTT	-	CP	nsTAAAAAAAAA	p.K98fs
XH_P37	3	149042680	149042680	Nonsense_Mutation	INS	-	CATTCAATGTCT	-	TM4SF18	CCTCAAGACATT	3_T134delinsPQDIEX
XH_P37	3	149238759	149238759	In_Frame_Ins	INS	-	CACTACCATTGT	-	WWTR1	insGTTACAATG	346delinsVTMVVIT
XH_P37	3	149485171	149485171	Frame_Shift_Ins	INS	-	GTCCTCCTTCTCA	-	ANKUB1	78insGATGAGAA	p.N426fs
XH_P37	3	149684339	149684339	In_Frame_Ins	INS	-	ATTCTGTTCACTT	-	PFN2	AACAAAAGTGAA	20delinsQNKSEQNYF
XH_P37	3	150908686	150908686	Frame_Shift_Ins	INS	-	GTGCTGCTCTTCT	-	MED12L	insCTGGTGCTG	p.H646fs
XH_P37	3	150908687	150908687	In_Frame_Ins	INS	-	CATCCGCCATGA	-	MED12L	8insGTTCATCCG	646delinsQFIRHDV
XH_P37	3	151163319	151163319	Nonsense_Mutation	INS	-	TTACATTCTACTC	-	IGSF10	CATGGGAGTAG	84_A1485delinsAWEX
XH_P37	3	154002700	154002700	Nonsense_Mutation	INS	-	GTTTTATTAGAAT	-	DHX36	AATGACATTCTAA	689_K690delinsEX
XH_P37	3	156745908	156745908	Nonsense_Mutation	INS	-	TTGGAAATATAT	-	LEKR1	GCACTTTTGGAA	2delinsTWALLEIYKFLX
XH_P37	3	156983447	156983447	Frame_Shift_Ins	INS	-	AGTTG	-	VEPH1	997_1998insCA	p.V666fs
XH_P37	3	156983449	156983449	Frame_Shift_Ins	INS	-	TTTTTAGTTG	-	VEPH1	_1996insCAACT	p.V666fs
XH_P37	3	158262051	158262051	Frame_Shift_Ins	INS	-	CTCACCTTTGTAC	-	RSRC1	insAACTCACCTT	p.S273fs
XH_P37	3	158402343	158402343	Nonsense_Mutation	INS	-	AAAATGTTTAAC	-	GFM1	TTTTTTAAAAATG	p.L599delinsQKX
XH_P37	3	160679649	160679649	In_Frame_Ins	INS	-	GTCATTTTTAGTA	-	PPM1L	insTCGTCATTTT	S48delinsSSSFLVS
XH_P37	3	165504041	165504041	Frame_Shift_Ins	INS	-	AACTCTTACTATA	-	BCHE	.576insTATAGTA	p.Q526fs
XH_P37	3	167006728	167006728	Nonsense_Mutation	INS	-	TTAGGAAATAAT	-	ZBBX	GCAAATATTATT	delinsAISLTTFCKYYFLIHCYRKX
XH_P37	3	167034877	167034877	Nonsense_Mutation	INS	-	AATTTTTTTGGAT	-	ZBBX	.10insCATCCAAA	/370delinsVIQKNX
XH_P37	3	167034879	167034879	Missense_Mutation	SNP	C	A	C	ZBBX	c.G1108T	p.V370L
XH_P37	3	167189551	167189551	Nonsense_Mutation	INS	-	GTTCAGGATAAA	-	SERPINI2	ATTTCTTTATCCT	Q24_K25delinsHX
XH_P37	3	167189553	167189553	Nonsense_Mutation	SNP	G	A	G	SERPINI2	c.C70T	p.Q24X
XH_P37	3	167728064	167728064	Frame_Shift_Ins	INS	-	GGAATATATTTTG	-	GOLIM4	insTTATCAAAAT	p.E667fs
XH_P37	3	167728065	167728065	Frame_Shift_Ins	INS	-	GT	-	GOLIM4	.1998_1999insA	p.E667fs
XH_P37	3	168833508	168833508	Frame_Shift_Ins	INS	-	G	-	MECOM	c.1587_1588ins	p.L530fs
XH_P37	3	168833509	168833509	Frame_Shift_Ins	INS	-	GTGTATGGAA	-	MECOM	_1587insTTCCA	p.S529fs
XH_P37	3	173996814	173996814	Nonsense_Mutation	INS	-	TTTTAGATGAT	-	NLGN1	_1024insTTTTAG	341_L342delinsEFX
XH_P37	3	173996816	173996816	Frame_Shift_Ins	INS	-	TTGGGTAA	-	NLGN1	5_1026insTTGG	p.L342fs
XH_P37	3	178919265	178919265	Frame_Shift_Ins	INS	-	ATTACTTTATCCA	-	PIK3CA	CTATCATTACTTT	p.Y250fs
XH_P37	3	179051684	179051684	Frame_Shift_Ins	INS	-	TGGTAGAGAAAC	-	ZNF639	nsTATTGGTAGA	p.H311fs
XH_P37	3	182538122	182538122	Nonsense_Mutation	INS	-	TTAGCTGGTCAG	-	ATP11B	TTTATTTAGCTG	p.Y34delinsYCX
XH_P37	3	182871823	182871823	Frame_Shift_Ins	INS	-	AGGACTATAACA	-	LAMP3	GATAGTGTTATA	p.P136fs
XH_P37	3	183245744	183245744	Frame_Shift_Ins	INS	-	AGCTGGGC	-	KLHL6	7_348insGCCCA	p.K116fs
XH_P37	3	183707163	183707163	Nonsense_Mutation	INS	-	GTT	-	ABCC5	.137_138insAA	p.C46delinsX
XH_P37	3	183707164	183707164	Frame_Shift_Ins	INS	-	AAGTG	-	ABCC5	136_137insCAC	p.C46fs
XH_P37	3	184020536	184020536	In_Frame_Ins	INS	-	ACCAACTCCATGT	-	PSMD2	sTTCTACCAACT	181delinsVFYQLHVI
XH_P37	3	184093778	184093778	Frame_Shift_Ins	INS	-	ACCATAGCTCTC	-	THPO	sAGAGAGAGCT	p.L13fs
XH_P37	3	185993440	185993440	Frame_Shift_Ins	INS	-	ATTGTCCCAA	-	DGKG	_806insTTGGGA	p.D269fs
XH_P37	3	185997718	185997718	Frame_Shift_Ins	INS	-	T	-	DGKG	c.713_714insA	p.G238fs
XH_P37	3	185997720	185997720	Nonsense_Mutation	INS	-	TACATTATTTTGT	-	DGKG	GGATACAAAATA	G238_F239delinsX
XH_P37	3	186272407	186272407	Frame_Shift_Ins	INS	-	T	-	TBCCD1	c.891dupA	p.G298fs
XH_P37	3	186272409	186272409	Nonsense_Mutation	INS	-	ATTCTATGTATTT	-	TBCCD1	sTAATAAATACA	97_G298delinsIINTX
XH_P37	3	193365903	193365903	Nonsense_Mutation	INS	-	AACTGAATTTCT	-	OPA1	TGTATTAACTGA	p.R548delinsLYX
XH_P37	3	194061852	194061852	Missense_Mutation	SNP	G	A	G	CPN2	c.C1580T	p.S527L
XH_P37	3	194158105	194158105	Frame_Shift_Ins	INS	-	CAGTCATCATCAT	-	ATP13A3	.934insATGATGA	p.R645fs
XH_P37	3	195515902	195515902	Frame_Shift_Ins	INS	-	ACAAGAAC	-	MUC4	48_2549insGTTC	p.A850fs
XH_P37	3	195515904	195515904	Silent	SNP	G	A	G	MUC4	c.C2547T	p.S849S
XH_P37	3	196230026	196230026	Nonsense_Mutation	INS	-	AGAATCAGAGG	-	RNF168	insTTCCTCTGAT	p.A7delinsFLX
XH_P37	3	196386792	196386792	Missense_Mutation	SNP	T	C	T	NRROS	c.T278C	p.L93P
XH_P37	3	196675428	196675428	Missense_Mutation	SNP	C	T	C	PIGZ	c.G340A	p.G114R
XH_P37	3	196743242	196743242	Splice_Site	INS	-	CACCAGGGA	-	MELTF	.	.
XH_P37	3	196812573	196812573	Nonsense_Mutation	INS	-	TTAATTTGTGAC	-	DLG1	ATGTTGTCACAA	delinsLHPCCHKLKKIX
XH_P37	3	196888573	196888573	Nonsense_Mutation	INS	-	TTATAAAAATTTC	-	DLG1	AAATGAAATTT	p.P174delinsKX
XH_P37	3	197423867	197423867	In_Frame_Ins	INS	-	CTGCCATTTCTC	-	RUBCN	1315insGAGAAA	439delinsEKWQS
XH_P37	4	154943	154943	Frame_Shift_Ins	INS	-	TTTCTCATGTCCT	-	ZNF718	CCACGTTTCTCAT	p.S156fs
XH_P37	4	366608	366608	Nonsense_Mutation	INS	-	TTACAAATTATC	-	ZNF141	AAAAATTACAAA	G53delinsEIKITNYPTX
XH_P37	4	1389361	1389457	Frame_Shift_Del	DEL	ATGTGGAGT	-	CCATGTGGAGTG	CRIPAK	c.1062_1158del	p.L354fs
XH_P37	4	2083462	2083462	Nonsense_Mutation	INS	-	GTCATCTTTGTA	-	POLN	206insCTACAAA	36_S737delinsLQRX
XH_P37	4	2955304	2955304	In_Frame_Ins	INS	-	CCTGCTGTGTGTA	-	NOP14	1insTTACACACA	227delinsDYTQQV
XH_P37	4	3134640	3134640	Splice_Site	INS	-	TCTAATG	-	HTT	.	.
XH_P37	4	3205873	3205873	Nonsense_Mutation	INS	-	TTTAAT	-	HTT	716_5717insTTT	p.V1906delinsVX
XH_P37	4	3205875	3205875	Nonsense_Mutation	INS	-	TCTAAAACAGAC	-	HTT	nsTAGTTCTAAAA	p.V1906delinsVX
XH_P37	4	4242118	4242118	Nonsense_Mutation	INS	-	CTAGTTATTTAA	-	TMEM128	GTGCTTTAAATAA	110delinsHHLCFKX
XH_P37	4	4304019	4304022	Frame_Shift_Del	DEL	CCCC	-	CCCC	ZBTB49	c.456_459del	p.Y152fs
XH_P37	4	7000864	7000864	Nonsense_Mutation	INS	-	TAACATCTA	-	TBC1D14	8_259insTAACAT	p.V86delinsVX
XH_P37	4	7000865	7000865	Frame_Shift_Ins	INS	-	AC	-	TBC1D14	c.259_260insAC	p.E87fs

XH_P37	4	7056211	7056211	Frame_Shift_Ins	INS	-	ACAGAGA	-	TADA2B	93_694insACAG/	p.A231fs
XH_P37	4	7056212	7056212	Frame_Shift_Ins	INS	-	G	-	TADA2B	c.694_695insG	p.F232fs
XH_P37	4	9213868	9213868	Missense_Mutation	SNP	G	C	G	USP17L10	c.G1486C	p.D496H
XH_P37	4	9251647	9251647	Frame_Shift_Ins	INS	-	CAGAAGTGATGC	-	USP17L11	1293insCAGAAGT	p.E431fs
XH_P37	4	10447574	10447574	Missense_Mutation	SNP	C	T	C	ZNF518B	c.G379A	p.V127I
XH_P37	4	10447575	10447575	Nonsense_Mutation	INS	-	GTGACTATGGTG	-	ZNF518B	AATAGCACCATA	26_V127delinsNHFX
XH_P37	4	10567701	10567701	Frame_Shift_Ins	INS	-	TCTCTTTCTGAA	-	CLNK	sTGTTTTCAGAA	p.R75fs
XH_P37	4	13370268	13370268	Missense_Mutation	SNP	C	T	C	RAB28	c.G580A	p.V194M
XH_P37	4	13370270	13370270	In_Frame_Ins	INS	-	TTGCTATTG	-	RAB28	7_578insCAATA(	V193delinsAIAM
XH_P37	4	13605245	13605245	In_Frame_Ins	INS	-	ATGAATTGAGA	-	BOD1L1	9insAAGTTCTCA	1093delinsTSSQFIL
XH_P37	4	13612692	13612692	In_Frame_Ins	INS	-	AGA	-	BOD1L1	1356_1357insT(	p.T453delinsST
XH_P37	4	13612693	13612693	Frame_Shift_Ins	INS	-	ACATGTGACAGA	-	BOD1L1	insCATCTCTGTC	p.K452fs
XH_P37	4	13616325	13616325	Frame_Shift_Ins	INS	-	AACAGGAAAGCA	-	BOD1L1	69insCTGCTTTC	p.E223fs
XH_P37	4	15518365	15518365	Frame_Shift_Ins	INS	-	ACATATATATTT(	-	CC2D2A	GTGTCAACATAT	p.T379fs
XH_P37	4	15688732	15688732	Frame_Shift_Ins	INS	-	TTGAATCGAAT	-	FAM200B	_133insTTGAAT(	p.Q44fs
XH_P37	4	15688734	15688734	Missense_Mutation	SNP	C	T	C	FAM200B	c.C134T	p.T45I
XH_P37	4	15689956	15689956	Nonsense_Mutation	INS	-	GCACCATTATTAG	-	FAM200B	ACACAGCACCAT	52delinsDSTQHHYX
XH_P37	4	16026917	16026917	Frame_Shift_Ins	INS	-	G	-	PROM1	c.500_501insC	p.G167fs
XH_P37	4	16026919	16026919	In_Frame_Ins	INS	-	AGAATTGGTGTT	-	PROM1	AATAAAAACACC	67delinsKIKTPILKFG
XH_P37	4	17885899	17885899	Frame_Shift_Ins	INS	-	AA	-	LCORL	.1252_1253insT	p.R418fs
XH_P37	4	17885900	17885900	Frame_Shift_Ins	INS	-	TGTTGACTCT	-	LCORL	_1252insAGAGT	p.R418fs
XH_P37	4	20706377	20706377	Frame_Shift_Ins	INS	-	CCCTCTGATT	-	PACRGL	7_148insCCCTCT	p.E49fs
XH_P37	4	24531358	24531358	Frame_Shift_Ins	INS	-	CCCTTCCTTT	-	DHX15	_2136insAAAGG	p.T712fs
XH_P37	4	25256870	25256870	In_Frame_Ins	INS	-	GTAAAAATCTTAT	-	PI4K2B	AAAAGTAAAAAT	03delinsKKVKILLDF
XH_P37	4	36230629	36230629	In_Frame_Ins	INS	-	AAATGGAACAAA	-	ARAP2	480insTTTGTTC	H160delinsHLFHF
XH_P37	4	37447439	37447439	Frame_Shift_Ins	INS	-	AACAGAAAGAC	-	NWD2	3830insAACAGA	p.I1277fs
XH_P37	4	37448332	37448332	Frame_Shift_Ins	INS	-	CC	-	NWD2	.4722_4723insC	p.R1574fs
XH_P37	4	37848718	37848718	Frame_Shift_Ins	INS	-	ACTCTAAAGT	-	PGM2	_1175insACTCT	p.G392fs
XH_P37	4	38798825	38798825	Frame_Shift_Ins	INS	-	GATCTGCCCTGG	-	TLR1	628insACCAGG(	p.S543fs
XH_P37	4	38990576	38990576	Nonsense_Mutation	INS	-	ATCTTTCTTCTAC	-	TMEM156	insGAGTAGAAG	1212_K213delinsEX
XH_P37	4	39274644	39274644	Frame_Shift_Ins	INS	-	T	-	WDR19	c.3048_3049ins	p.L1016fs
XH_P37	4	39274646	39274646	Frame_Shift_Ins	INS	-	GCTCTGCTTTTCA	-	WDR19	051insGCTCTGC	p.I1017fs
XH_P37	4	39310386	39310386	Frame_Shift_Ins	INS	-	GTGCAGTACT	-	RFC1	_1755insAGTAC	p.L585fs
XH_P37	4	39344065	39344065	Nonsense_Mutation	INS	-	TTGCTTTGTTGA(	-	RFC1	ATAACTCAACAA	p.L77delinsLX
XH_P37	4	39408870	39408870	Frame_Shift_Ins	INS	-	ATTCCTATAGCGT	-	KLB	ATTGTATTCCTAT	p.K101fs
XH_P37	4	40099007	40099007	Nonsense_Mutation	INS	-	GCAATTAAAAA(	-	N4BP2	GAAAGCAATTAA	p.T16delinsTX
XH_P37	4	40103933	40103933	Frame_Shift_Ins	INS	-	CTTGGTATCAGT	-	N4BP2	9insATCTTGGTA	p.S156fs
XH_P37	4	40104353	40104353	Missense_Mutation	SNP	C	A	C	N4BP2	c.C888A	p.N296K
XH_P37	4	40104355	40104355	In_Frame_Ins	INS	-	TGTGAAGGTAAT	-	N4BP2	nsGAATGTGAAG	297delinsLNVKVMG
XH_P37	4	41635675	41635675	In_Frame_Ins	INS	-	AATCTCCCT	-	LIMCH1	0_1941insAATC	).D647delinsEISL
XH_P37	4	42457378	42457378	Frame_Shift_Ins	INS	-	GATA	-	ATP8A1	2707_2708insTA	p.E903fs
XH_P37	4	42457379	42457379	Nonsense_Mutation	INS	-	GGCAGAGAAGG	-	ATP8A1	2707insACCTTC	03_N904delinsTFSAX
XH_P37	4	47427734	47427734	Missense_Mutation	SNP	G	A	G	GABRB1	c.G1124A	p.R375Q
XH_P37	4	47559835	47559835	Nonsense_Mutation	INS	-	ATTAGATATAAA	-	ATP10D	AAATAAATTAGA	60_P661delinsLEINX
XH_P37	4	47850332	47850332	Nonsense_Mutation	INS	-	GGACAGAATGTT	-	NFXL1	AAAGTTTAACAT	p.N862delinsX
XH_P37	4	48581108	48581108	In_Frame_Ins	INS	-	TCAGCTTTCATA	-	FRYL	10insCATATGAA	804delinsHMKADS
XH_P37	4	52883425	52883425	Frame_Shift_Ins	INS	-	GGAA	-	LRRC66	.354_355insTTC	p.N119fs
XH_P37	4	52883427	52883427	In_Frame_Ins	INS	-	TATAGTGTG	-	LRRC66	2_353insCACAC	N118delinsTHYN
XH_P37	4	55156615	55156615	Nonsense_Mutation	INS	-	ATAAACATTCAT	-	PDGFRA	AACTCTATAAAC	L1007delinsEFFHNSINIHX
XH_P37	4	57265517	57265517	Nonsense_Mutation	INS	-	AATAAATTAACTT	-	PPAT	TAAAAAAAGTTA	355delinsIKKVNLLYX
XH_P37	4	57397076	57397076	Silent	SNP	G	A	G	THEGL	c.G234A	p.E78E
XH_P37	4	57397077	57397077	Frame_Shift_Ins	INS	-	AGAAACTCTTGG	-	THEGL	6insTTAGAAACT	p.P79fs
XH_P37	4	57796663	57796663	In_Frame_Ins	INS	-	CTCTTTTGAGAC	-	REST	sCTTTTTCTCTTT	547delinsAFSLLRHLL
XH_P37	4	66217285	66217285	Nonsense_Mutation	INS	-	AGAAAGAGAGAG	-	EPHA5	CCTCATTCTCTCT	_V757delinsPLILSLSFX
XH_P37	4	68472071	68472071	Frame_Shift_Ins	INS	-	GGTGTGTTTAGT	-	STAP1	85insTGGTGTGT	p.A295fs
XH_P37	4	68497606	68497606	Nonsense_Mutation	INS	-	CTTTAATGTTAAA	-	UBA6	AAATAATTTAAC	1_F722delinsWDEIIX
XH_P37	4	69962362	69962362	Nonsense_Mutation	INS	-	GATCTGTTCACAC	-	UGT2B7	AAATGATCTGTT	2_L43delinsNEGKX
XH_P37	4	69964374	69964374	Frame_Shift_Ins	INS	-	CAAGATAATTAC	-	UGT2B7	39insACAAGATA	p.L280fs
XH_P37	4	70066372	70066372	Missense_Mutation	SNP	C	A	C	UGT2B11	c.G1376T	p.R459L
XH_P37	4	70066374	70066374	Frame_Shift_Ins	INS	-	GAAGGGAAAAA	-	UGT2B11	CTAATCTCTTTTT	p.D458fs
XH_P37	4	70146411	70146411	Missense_Mutation	SNP	G	A	G	UGT2B28	c.G193A	p.D65N
XH_P37	4	70146412	70146412	Frame_Shift_Ins	INS	-	GTCACTGTTTCTT	-	UGT2B28	195insGTCACTG	p.D65fs
XH_P37	4	70599137	70599137	Frame_Shift_Ins	INS	-	CTGCAATTATTT(	-	SULT1B1	insAGGAAATAAT	p.M197fs
XH_P37	4	71472097	71472097	Nonsense_Mutation	INS	-	CACATGCTATTA	-	AMBN	sTCATCACATGC	N332delinsIITCYX
XH_P37	4	71507937	71507937	Frame_Shift_Ins	INS	-	GAAATCAATAATT	-	ENAM	AATGGAAATCA	p.N265fs
XH_P37	4	71509605	71509605	Frame_Shift_Ins	INS	-	CCTTGGTTTTGAT	-	ENAM	53insTCCCTTGGT	p.P821fs
XH_P37	4	71509607	71509607	Missense_Mutation	SNP	A	T	A	ENAM	c.A2464T	p.I822L
XH_P37	4	71555610	71555610	In_Frame_Ins	INS	-	TAGGTAACAAG(	-	UTP3	TTTCGGTAGGTA	06delinsISVGNKPFY
XH_P37	4	71698131	71698131	Frame_Shift_Ins	INS	-	AATATTTATAGA	-	GRSF1	CAAAATCTATAA	p.M74fs
XH_P37	4	72306492	72306492	Splice_Site	INS	-	TCCTGTGG	-	SLC4A4	.	.
XH_P37	4	73013492	73013492	Missense_Mutation	SNP	A	G	A	NPFFR2	c.A1532G	p.E511G
XH_P37	4	73013493	73013493	Frame_Shift_Ins	INS	-	TTGACACTGCT	-	NPFFR2	_1534insTTGACA	p.E511fs
XH_P37	4	73185128	73185128	Frame_Shift_Ins	INS	-	AA	-	ADAMTS3	.1272_1273insT	p.M425fs
XH_P37	4	73185129	73185129	Nonsense_Mutation	INS	-	AATATAAGTAAA	-	ADAMTS3	sTAAAAGTTTACT	_M425delinsVKSLLIFX
XH_P37	4	73186550	73186550	Frame_Shift_Ins	INS	-	ATACATT	-	ADAMTS3	82_983insAATGT	p.S328fs
XH_P37	4	73959942	73959942	Nonsense_Mutation	INS	-	TAGCTTAATGAA	-	ANKRD17	CTGTATTTCATTA	K1477delinsSHMAVFHX
XH_P37	4	74008083	74008083	Frame_Shift_Ins	INS	-	AGTTCTGTTTGTA	-	ANKRD17	CATATTTACAAAC	p.I589fs
XH_P37	4	76452388	76452388	Frame_Shift_Ins	INS	-	ATTACACTGTAG(	-	THAP6	3insCCATTACACT	p.C169fs
XH_P37	4	76956486	76956486	Nonsense_Mutation	INS	-	GTGGACAACTAC	-	CXCL11	TTTCTGTAGTTGT	24_F25delinsRIFLX
XH_P37	4	77033573	77033573	In_Frame_Ins	INS	-	AATTTT	-	ART3	005_1006insAAT	).S335delinsSNF
XH_P37	4	77278640	77278640	Frame_Shift_Ins	INS	-	TGAGAAATATAC	-	CCDC158	TACTATTGTATAT	p.S687fs
XH_P37	4	78641711	78641711	In_Frame_Ins	INS	-	GGTTTCTTTCATT	-	CNOT6L	542insTAATGAAA	514delinsDNERNL
XH_P37	4	79359737	79359737	Frame_Shift_Ins	INS	-	CTCTTTGATAACA	-	FRAS1	AATTCCTCTTTG	p.P1744fs
XH_P37	4	79847859	79847859	Frame_Shift_Ins	INS	-	GAGTGTAGTTAA	-	PAQR3	AAAATATTAACT	p.V173fs
XH_P37	4	81207609	81207609	Frame_Shift_Ins	INS	-	GTTGTGGCGAT(	-	FGF5	TTTAAGTTGTGG	p.K54fs
XH_P37	4	81866074	81866074	Nonsense_Mutation	INS	-	TATAATGCATTA	-	C4orf22	AGATGTATAATG	.I196delinsINIYX
XH_P37	4	81866076	81866076	Missense_Mutation	SNP	C	A	C	C4orf22	c.C589A	p.L197I
XH_P37	4	82366778	82366778	Frame_Shift_Ins	INS	-	GTATG	-	RASGEF1B	703_704insCATA	p.V235fs
XH_P37	4	82366779	82366779	Nonsense_Mutation	INS	-	ATTGTTGGAATG	-	RASGEF1B	sTAAAACATTCCA	p.V235delinsX
XH_P37	4	83763552	83763552	In_Frame_Ins	INS	-	AGGGATTTG	-	SEC31A	1_2592insCAAA	).T864delinsTKSL
XH_P37	4	83772756	83772756	In_Frame_Ins	INS	-	AATTAGAATTGA	-	SEC31A	2039insTCAATT	D680delinsVNSNY
XH_P37	4	83772758	83772758	Splice_Site	INS	-	A	-	SEC31A	.	.
XH_P37	4	83788085	83788085	Nonsense_Mutation	INS	-	ATACCAAAGTGT	-	SEC31A	ATAATAACACTT	353_F354delinsLX
XH_P37	4	84511383	84511383	Frame_Shift_Ins	INS	-	CAATC	-	GPAT3	676_677insCAAT	p.V226fs
XH_P37	4	85724601	85724601	Frame_Shift_Ins	INS	-	GG	-	WDFY3	.2448_2449insC	p.S817fs
XH_P37	4	85724602	85724602	Frame_Shift_Ins	INS	-	GAAAGTTAATAT	-	WDFY3	2448insAATATTA	p.V816fs
XH_P37	4	85750297	85750297	Nonsense_Mutation	INS	-	AGAATGTATTTT	-	WDFY3	CATTTAAAATAC	272_D273delinsDIX
XH_P37	4	87662971	87662971	Splice_Site	INS	-	TTTGGCAGACCT	-	PTPN13	.	.

XH_P37	4	87671831	87671831	Missense_Mutation	SNP	G	T	G	PTPN13	c.G2859T	p.K953N
XH_P37	4	87671833	87671833	Nonsense_Mutation	INS	-	TAAGCAGCATGC	-	PTPN13	ATAAATAAGCA	954delinsNYVNKX
XH_P37	4	88766967	88766967	Frame_Shift_Ins	INS	-	ATGGGTGCTTTT	-	MEPE	48insGATGGGT	p.T316fs
XH_P37	4	88766969	88766969	Missense_Mutation	SNP	A	T	A	MEPE	c.A949T	p.I317F
XH_P37	4	90169810	90169810	Frame_Shift_Ins	INS	-	AAGAACCAGGA	-	GPRIN3	insTGCTCTCCTG	p.G484fs
XH_P37	4	90856248	90856248	Frame_Shift_Ins	INS	-	T	-	MMRN1	c.1417_1418ins	p.T473fs
XH_P37	4	90856250	90856250	Nonsense_Mutation	INS	-	GTACATTAATGC	-	MMRN1	1420insGTACAT	73_C474delinsTVHX
XH_P37	4	94411822	94411822	Nonsense_Mutation	INS	-	TTACCTTTTATTA	-	GRID2	AATTAATTACCT	536_M537delinsLX
XH_P37	4	94411824	94411824	Silent	SNP	A	T	A	GRID2	c.A1608T	p.R536R
XH_P37	4	99812397	99812397	Nonsense_Mutation	INS	-	GAATTGGTGATT	-	EIF4E	insCATAATCAC	D91_F92delinsAX
XH_P37	4	99812398	99812398	Nonsense_Mutation	INS	-	TTTA	-	EIF4E	270_271insTAA	.D91_F92delinsX
XH_P37	4	100512921	100512921	Frame_Shift_Ins	INS	-	TGAAATGGGTC	-	MTTP	sCAATTGAAATG	p.Q271fs
XH_P37	4	100522869	100522869	In_Frame_Ins	INS	-	ATTTGCTTTGA	-	MTTP	4insGAATTTGC	475delinsRILLLRQ
XH_P37	4	100851764	100851764	In_Frame_Ins	INS	-	TGG	-	DNAJB14	c.147_148insC	p.I50delinsPI
XH_P37	4	100851766	100851766	Frame_Shift_Ins	INS	-	TTAATGTACT	-	DNAJB14	_146insAGTACA	p.I49fs
XH_P37	4	102984291	102984291	In_Frame_Ins	INS	-	ATTCCATGTTGT	-	BANK1	0insGTCATTCCA	603delinsVVIPCCR
XH_P37	4	103987655	103987655	Frame_Shift_Ins	INS	-	ATGGTAAGGAT	-	SLC9B2	0insTTCATCCTT	p.V34fs
XH_P37	4	103988642	103988642	Frame_Shift_Ins	INS	-	TATGAAACGCTT	-	SLC9B2	6insAAAGCGTTT	p.T22fs
XH_P37	4	104016396	104016396	In_Frame_Ins	INS	-	GATAAAAATAAT	-	BDH2	insGTTATTATTT	N39delinsVIIFIIN
XH_P37	4	105412195	105412195	Frame_Shift_Ins	INS	-	AAGAAAAAACCT	-	CXXC4	765insCAGGTTTT	p.T255fs
XH_P37	4	106158017	106158017	Frame_Shift_Ins	INS	-	TGTGACTTCTGC	-	TET2	919insTTGTGAC	p.C973fs
XH_P37	4	106158192	106158192	Frame_Shift_Ins	INS	-	GCTTTGAGTGT	-	TET2	3094insGCTTTTG	p.H1031fs
XH_P37	4	106164912	106164912	Silent	SNP	T	C	T	TET2	c.T3780C	p.N1260N
XH_P37	4	106164913	106164913	Frame_Shift_Ins	INS	-	AAACAAAACAAG	-	TET2	782insAAAACAA	p.R1261fs
XH_P37	4	106193719	106193719	Splice_Site	INS	-	GAAAATAGCAAC	-	TET2	.	.
XH_P37	4	106197656	106197656	Frame_Shift_Ins	INS	-	TTTTT	-	TET2	989_5990insTT	p.P1997fs
XH_P37	4	106197657	106197657	In_Frame_Ins	INS	-	AGCCAT	-	TET2	90_5991insAGC	.P1997delinsPAI
XH_P37	4	106604317	106604317	Frame_Shift_Ins	INS	-	AACTAAGTGGG	-	INTS12	2insATTCCCACT	p.P321fs
XH_P37	4	108866284	108866284	Frame_Shift_Ins	INS	-	TA	-	CYP2U1	c.649_650insTA	p.P217fs
XH_P37	4	108866286	108866286	Frame_Shift_Ins	INS	-	ATGGAGTGCA	-	CYP2U1	_652insATGGAG	p.P217fs
XH_P37	4	110864539	110864539	In_Frame_Ins	INS	-	ACAACAAAATTA	-	EGF	458insACAACAA	.L153delinsHNKII
XH_P37	4	111471041	111471041	Frame_Shift_Ins	INS	-	AT	-	ENPEP	.2500_2501insA	p.R834fs
XH_P37	4	111471042	111471042	Nonsense_Mutation	INS	-	TTCTAAGCATAA	-	ENPEP	insTGTCTTCTAA	34_Y835delinsSVFX
XH_P37	4	113187838	113187838	Nonsense_Mutation	INS	-	AATTATCTGTCTC	-	AP1AR	TAACAATTATCT	70_T171delinsSRFILX
XH_P37	4	113189527	113189527	Frame_Shift_Ins	INS	-	TTTT	-	AP1AR	.772_773insTTT	p.G258fs
XH_P37	4	113189528	113189528	Frame_Shift_Ins	INS	-	GAACGTTCTCT	-	AP1AR	_774insGAACGT	p.G258fs
XH_P37	4	113481933	113481933	Nonsense_Mutation	INS	-	GTGATGGCTAAG	-	ZGRF1	TTCATAACTTAGC	.V1581delinsDEX
XH_P37	4	113508855	113508855	Frame_Shift_Ins	INS	-	AGAAAGTACTTT	-	ZGRF1	TCAGAGAAAGT	p.E1062fs
XH_P37	4	113510998	113510998	Nonsense_Mutation	INS	-	GATGAAGTTTTT	-	ZGRF1	CAAATAAAAAAC	46delinsFSVIISQIKNFILNEX
XH_P37	4	114275990	114275990	Silent	SNP	C	A	C	ANK2	c.C6216A	p.S2072S
XH_P37	4	114275991	114275991	In_Frame_Ins	INS	-	TCCTCTCTGTTT	-	ANK2	6218insTCCTCT	I2073delinsILSVL
XH_P37	4	114277101	114277101	Frame_Shift_Ins	INS	-	AATCATCTTGTC	-	ANK2	7328insAATCAT	p.L2443fs
XH_P37	4	114277991	114277991	Frame_Shift_Ins	INS	-	TTTGGGTTCTTT	-	ANK2	sTCTTGTTTGGG	p.S2739fs
XH_P37	4	114280072	114280072	Frame_Shift_Ins	INS	-	TGTCTTCTGG	-	ANK2	_10299insTGTC	p.T3433fs
XH_P37	4	115544459	115544459	Nonsense_Mutation	INS	-	TAGATGGGGCG	-	UGT8	424insTAGATGG	141_M142delinsDX
XH_P37	4	115769457	115769457	Frame_Shift_Ins	INS	-	AAAAAACATTAT	-	NDST4	854insCATAATG	p.P618fs
XH_P37	4	118005966	118005966	Nonsense_Mutation	INS	-	TGTTTCCGTACT	-	TRAM1L1	CAGTGAGTACG	95_Q196delinsQQX
XH_P37	4	119644806	119644806	Nonsense_Mutation	INS	-	CTCTGTCATTGAT	-	SEC24D	GCAACATCAATC	9_I990delinsMQHQX
XH_P37	4	119736768	119736768	Nonsense_Mutation	INS	-	CTTTGTGTTACC	-	SEC24D	11insGGGTAACA	171_P172delinsGX
XH_P37	4	122254154	122254154	Nonsense_Mutation	INS	-	CTCA	-	QRFPR	618_619insTGA	S207_P208delinsX
XH_P37	4	122254155	122254155	Frame_Shift_Ins	INS	-	CCTCTAGGTAAT	-	QRFPR	insTAAATTACCT	p.T206fs
XH_P37	4	122740015	122740015	Silent	SNP	T	G	T	CCNA2	c.A1056C	p.P352P
XH_P37	4	122740017	122740017	Nonsense_Mutation	INS	-	TTGCCTAGATGT	-	CCNA2	054insAACATCT	352_S353delinsNIX
XH_P37	4	123267880	123267880	Missense_Mutation	SNP	G	A	G	KIAA1109	c.G12836A	p.R4279H
XH_P37	4	123305067	123305067	In_Frame_Ins	INS	-	ACCAACTTTACA	-	ADAD1	TAAACACCAACT	59delinsHKHQLYKSF
XH_P37	4	123663361	123663361	Frame_Shift_Ins	INS	-	A	-	BBS12	c.314_315insA	p.L105fs
XH_P37	4	123663362	123663362	Nonsense_Mutation	INS	-	TGGATGATTTTAC	-	BBS12	AATTTGGATGA	5_G106delinsLNKFGX
XH_P37	4	123664018	123664018	Frame_Shift_Ins	INS	-	AGTATTATCTGT	-	BBS12	CCTTCAGTATTA	p.P324fs
XH_P37	4	125590492	125590492	Frame_Shift_Ins	INS	-	GAACAATGCAAG	-	ANKRD50	03insTATCTTGCA	p.G1135fs
XH_P37	4	126239555	126239555	Frame_Shift_Ins	INS	-	ACATCATACC	-	FAT4	_1990insACATC	p.D663fs
XH_P37	4	128732702	128732702	Frame_Shift_Ins	INS	-	A	-	HSPA4L	c.1366_1367ins	p.V456fs
XH_P37	4	128732703	128732703	Frame_Shift_Ins	INS	-	TAACTTATTC	-	HSPA4L	7_1368insTAACT	p.V456fs
XH_P37	4	129043100	129043100	Frame_Shift_Ins	INS	-	AGAAAGGATTAC	-	LARP1B	ATAAAGAAAGG	p.T186fs
XH_P37	4	140811436	140811436	Missense_Mutation	SNP	A	C	A	MAML3	c.T1154G	p.F385C
XH_P37	4	140811438	140811438	Nonsense_Mutation	INS	-	CTCAAACA	-	MAML3	1_1152insTGTT	384_F385delinsPVX
XH_P37	4	141320150	141320150	Nonsense_Mutation	INS	-	ATTTTTTTAAACA	-	CLGN	CTGATGTTTAAA	7_V248delinsSSSDVX
XH_P37	4	141847194	141847194	Nonsense_Mutation	INS	-	ATTTCTCCCCT	-	RNF150	924insAGGGGA	W308_L309delinsX
XH_P37	4	144449139	144449139	Nonsense_Mutation	INS	-	GAGGTGGGAATA	-	SMARCA5	AAAAGAGGTGG	307_D308delinsIX
XH_P37	4	145628399	145628399	Frame_Shift_Ins	INS	-	CCATGACTGTTA	-	HHIP	ACAATACCATGA	p.M382fs
XH_P37	4	149075973	149075973	Frame_Shift_Ins	INS	-	AAAGGTACTTCC	-	NR3C2	094insTGGAAGT	p.P698fs
XH_P37	4	154523693	154523693	Splice_Site	INS	-	AGACGAAACA	-	TMEM131L	.	.
XH_P37	4	154523695	154523695	Nonsense_Mutation	INS	-	TATAGGAAGAGC	-	TMEM131L	2456insTATAGG	p.F819delinsLX
XH_P37	4	154525255	154525255	Frame_Shift_Ins	INS	-	AGGCAGTTCT	-	TMEM131L	_3092insAGGCA	p.I1031fs
XH_P37	4	155156087	155156087	Frame_Shift_Ins	INS	-	TTTTTCTCCATCT	-	DCHS2	352insAGATGG	p.S2784fs
XH_P37	4	155506813	155506813	In_Frame_Ins	INS	-	ATTGAAACTACA	-	FGA	AAAAAGTGTAG	0delinsQKSVVSMTCN
XH_P37	4	156294391	156294391	Nonsense_Mutation	INS	-	ATATATTTAAGA	-	MAP9	TTTTTTTCTTAAA	_K127delinsVKSVFFSX
XH_P37	4	156775253	156775253	In_Frame_Ins	INS	-	ATTTAACACAAGA	-	ASIC5	ATATCCTCTTGTG	7delinsLKYPLVLNCFS
XH_P37	4	158257727	158257727	Frame_Shift_Ins	INS	-	GAAAAAGGAAT	-	GRIA2	insTGGTGAAAAA	p.S558fs
XH_P37	4	158257846	158257846	Nonsense_Mutation	INS	-	GAGCAATTGCAA	-	GRIA2	TAATGGAGCAA	597_L598delinsSX
XH_P37	4	159627447	159627447	In_Frame_Ins	INS	-	AAATACGCTAAG	-	ETFDH	1210insAAATAC	V403delinsVKYAK
XH_P37	4	159782777	159782777	Frame_Shift_Ins	INS	-	ATTTATCTGTTCT	-	FNIP2	TTTTTATTTATCT	p.L84fs
XH_P37	4	159782779	159782779	Missense_Mutation	SNP	C	G	C	FNIP2	c.C254G	p.T85S
XH_P37	4	164247261	164247261	Nonsense_Mutation	INS	-	TTTGTTTTATATT	-	NPY1R	sAATAAATATAA	149_P150delinsKX
XH_P37	4	164247263	164247263	Missense_Mutation	SNP	C	G	C	NPY1R	c.G444C	p.W148C
XH_P37	4	169173752	169173752	Frame_Shift_Ins	INS	-	TGCTTAATAAGA	-	DDX60	CATATATCTTATT	p.A1205fs
XH_P37	4	170028106	170028106	Frame_Shift_Ins	INS	-	GTTTCTTAATGG	-	SH3RF1	90insACCCATTA	p.R797fs
XH_P37	4	170506627	170506627	Nonsense_Mutation	INS	-	GTGTGCCTTTTT	-	NEK1	30insGAAAAAGC	Y227_S228delinsX
XH_P37	4	170506629	170506629	Nonsense_Mutation	INS	-	ACTACATTAG	-	NEK1	_678insCTAATG	226_Y227delinsHX
XH_P37	4	170613443	170613443	Nonsense_Mutation	INS	-	GAATCCACTTAA	-	CLCN3	ATGATGAATCCA	Y276_S277delinsX
XH_P37	4	170618693	170618693	Frame_Shift_Ins	INS	-	GGTACCATGGTG	-	CLCN3	1insAAGGTACCA	p.K430fs
XH_P37	4	175897678	175897678	Nonsense_Mutation	INS	-	CAATGAGGTTTT	-	ADAM29	insCTACAATGA	34_G335delinsLLQX
XH_P37	4	177032740	177032740	Nonsense_Mutation	INS	-	GACAGGAACTCC	-	WDR17	insTGACAGGAA	.Q3_V4delinsQX
XH_P37	4	177632805	177632805	Splice_Site	INS	-	GTGAAATTTACT	-	VEGFC	.	.
XH_P37	4	186085300	186085300	Nonsense_Mutation	INS	-	CA	-	CFAP97	.1353_1354insT	T452_V453delinsX
XH_P37	4	186283777	186283777	Missense_Mutation	SNP	G	C	G	SNX25	c.G2354C	p.S785T
XH_P37	4	187517755	187517755	In_Frame_Ins	INS	-	ACTTTTTTGGAT	-	FAT1	12939insATCCA	P4313delinsPSKKV
XH_P37	4	187524621	187524621	Nonsense_Mutation	INS	-	TGAAATGTCTGT	-	FAT1	1059insTAACAG	p.E3687delinsX
XH_P37	4	187524845	187524845	Frame_Shift_Ins	INS	-	G	-	FAT1	c.10834dupC	p.L3612fs

XH_P37	4	187524847	187524847	Nonsense_Mutation	INS	-	AATCATTGG	-	FAT1	2_10833insCCA	.L3611delinsLQX
XH_P37	4	187539653	187539653	Missense_Mutation	SNP	G	A	G	FAT1	c.C8087T	p.P2696L
XH_P37	4	187541497	187541497	Frame_Shift_Ins	INS	-	CCTT	-	FAT1	242_6243insAA	p.F2081fs
XH_P37	4	187541498	187541498	Nonsense_Mutation	INS	-	AAAGAATATCTT	-	FAT1	2insAACAAGAT	p.F2081delinsX
XH_P37	4	187542585	187542585	Frame_Shift_Ins	INS	-	AA	-	FAT1	.5154_5155insT	p.I1719fs
XH_P37	4	187542587	187542587	Frame_Shift_Ins	INS	-	ACTCAAATGCT	-	FAT1	_5153insAGCAT	p.T1718fs
XH_P37	4	187557349	187557349	In_Frame_Ins	INS	-	TGCTTTCAACTA	-	FAT1	ATATTATAGTTG	338delinsNIIVESKFS
XH_P37	4	187628850	187628850	Frame_Shift_Ins	INS	-	AAGACAAGGAG	-	FAT1	32insTACCTCCT	p.A711fs
XH_P37	4	189012829	189012829	Frame_Shift_Ins	INS	-	AAGAATTTTTAT	-	TRIML2	12insTTTATAAA	p.K338fs
XH_P37	4	189022359	189022359	Frame_Shift_Ins	INS	-	ATATGAGGATGT	-	TRIML2	AAACAACATCCT	p.M111fs
XH_P37	4	189022361	189022361	Missense_Mutation	SNP	C	A	C	TRIML2	c.G329T	p.S110I
XH_P37	5	1064042	1064042	Missense_Mutation	SNP	C	T	C	SLC12A7	c.G2656A	p.D886N
XH_P37	5	1081819	1081819	In_Frame_Ins	INS	-	CTCTCTTTTTTG	-	SLC12A7	1170insCAAAAA	V390delinsVKKER
XH_P37	5	1088434	1088434	Silent	SNP	G	A	G	SLC12A7	c.C531T	p.N177N
XH_P37	5	1321918	1321918	Silent	SNP	A	T	A	CLPTM1L	c.T1332A	p.G444G
XH_P37	5	1321919	1321919	Frame_Shift_Ins	INS	-	CCTT	-	CLPTM1L	330_1331insAA	p.G444fs
XH_P37	5	5461609	5461609	Frame_Shift_Ins	INS	-	TTTAAAGTTTTAG	-	ICE1	ATGTGTTTAAAG	p.S721fs
XH_P37	5	5462595	5462595	Frame_Shift_Ins	INS	-	TG	-	ICE1	.3148_3149insT	p.K1050fs
XH_P37	5	5462596	5462596	Frame_Shift_Ins	INS	-	GGAT	-	ICE1	149_3150insGG	p.K1050fs
XH_P37	5	5463633	5463633	Frame_Shift_Ins	INS	-	G	-	ICE1	c.4187dupG	p.C1396fs
XH_P37	5	5463635	5463635	Frame_Shift_Ins	INS	-	ATGTTGTTATTT	-	ICE1	189insCATGTTG	p.C1396fs
XH_P37	5	5464054	5464054	Nonsense_Mutation	INS	-	TGACACTGAAA	-	ICE1	insTATCTGACA	536_S1537delinsAIX
XH_P37	5	5464384	5464384	In_Frame_Ins	INS	-	AAGTTTTTACTG	-	ICE1	nsGCTGAAGTTT	.646delinsTLKFLLVC
XH_P37	5	7878349	7878349	In_Frame_Ins	INS	-	ATACAAAAAATA	-	MTRR	95insAATACAAA	V232delinsEYKKYL
XH_P37	5	9202083	9202083	Nonsense_Mutation	INS	-	TTTGTGAGACTG	-	SEMA5A	AATACCAGTCTC	p.I306delinsX
XH_P37	5	10426619	10426619	Missense_Mutation	SNP	G	A	G	MARCH6	c.G2176A	p.V726I
XH_P37	5	10426620	10426620	Nonsense_Mutation	INS	-	CAACATAAGCAT	-	MARCH6	78insATCAACAT	26_V727delinsVSTX
XH_P37	5	11199753	11199753	Nonsense_Mutation	INS	-	GAATCATTATTA	-	CTNND2	ATAATTTAATAA	257_Q258delinsIIX
XH_P37	5	13911505	13911505	In_Frame_Ins	INS	-	AAA	-	DNAH5	1633_1634insT	p.N545delinsIY
XH_P37	5	13911507	13911507	Frame_Shift_Ins	INS	-	GCTTTGGCTAAT	-	DNAH5	2insATCATTAG	p.T544fs
XH_P37	5	14508475	14508475	In_Frame_Ins	INS	-	AGGCAG	-	TRIO	38_9239insAGG	.R3080delinsQAG
XH_P37	5	14508477	14508477	Silent	SNP	A	T	A	TRIO	c.A9240T	p.R3080R
XH_P37	5	16682120	16682120	Frame_Shift_Ins	INS	-	GATTTTTAATTTG	-	MYO10	ATATCACAAATT	p.P1350fs
XH_P37	5	23526662	23526662	In_Frame_Ins	INS	-	GATTGCGTTCTA	-	PRDM9	nsGAGTGATTGC	489delinsGVIAFYML
XH_P37	5	32780891	32780891	Nonsense_Mutation	INS	-	TTCCTAAATTCTT	-	NPR3	60insATTCCTAA	0_M421delinsAFLNSX
XH_P37	5	33448805	33448805	Nonsense_Mutation	INS	-	AGTAAATATTGC	-	TARS	TGATCAGTAAAT	K99_T100delinsKX
XH_P37	5	33998923	33998923	Nonsense_Mutation	INS	-	ATGTTCTTTGGTT	-	AMACR	nsTAGAACCAAA	p.T188delinsX
XH_P37	5	34813735	34813735	Nonsense_Mutation	INS	-	CTTACTTTATCA	-	RAI14	ACATTTCTTACTT	A267delinsKSSYISYFIKIX
XH_P37	5	35065430	35065430	Frame_Shift_Ins	INS	-	TAGCTTGACTAA	-	PRLR	ATTCCATTAGTC	p.K443fs
XH_P37	5	36985621	36985621	Frame_Shift_Ins	INS	-	GTCTATTTTCAT	-	NIPBL	sAGACAGTCTAT	p.S780fs
XH_P37	5	37014856	37014856	Missense_Mutation	SNP	C	G	C	NIPBL	c.C4632G	p.I1544M
XH_P37	5	37173943	37173943	Frame_Shift_Ins	INS	-	CTTT	-	C5orf42	084_6085insAA	p.Q2029fs
XH_P37	5	37173944	37173944	Nonsense_Mutation	INS	-	TACTTTTCATTCA	-	C5orf42	4insATTTGAATG	.T2028delinsTFEX
XH_P37	5	38445796	38445796	In_Frame_Ins	INS	-	GAAATCTCT	-	EGFLAM	0_2481insGAAA	.C827delinsWKSL
XH_P37	5	38463096	38463096	In_Frame_Ins	INS	-	GCAAAAAGCAAA	-	EGFLAM	8insAGCAAAAA	N96delinsKAKSKC
XH_P37	5	38949930	38949930	Nonsense_Mutation	INS	-	ATACAAAATGTTA	-	RICTOR	CCTGGCTAACAT	1340delinsSVKPGX
XH_P37	5	39153623	39153623	In_Frame_Ins	INS	-	AGTTCCATGGGA	-	FYB	249insTTTCCCAT	P417delinsFPMELP
XH_P37	5	39377063	39377063	Frame_Shift_Ins	INS	-	AAAGAAATGTTT	-	DAB2	763insTTAAACAT	p.P588fs
XH_P37	5	40767741	40767741	Nonsense_Mutation	INS	-	AAGTTATCAGAT	-	PRKAA1	TATCAGATCTGA	16_A217delinsYYQIX
XH_P37	5	41917392	41917392	Frame_Shift_Ins	INS	-	CAGCAGATGAA	-	C5orf51	877insCAGCAG	p.R292fs
XH_P37	5	43298814	43298814	Frame_Shift_Ins	INS	-	A	-	HMGCS1	c.253_254insT	p.D85fs
XH_P37	5	43298815	43298815	Frame_Shift_Ins	INS	-	GCAAACTTTTGT	-	HMGCS1	253insTACAAAA	p.D85fs
XH_P37	5	45262689	45262689	Frame_Shift_Ins	INS	-	C	-	HCN1	c.2006_2007ins	p.T669fs
XH_P37	5	52204839	52204839	In_Frame_Ins	INS	-	TAGTTGGCA	-	ITGA1	7_1568insTAGT	.Q523delinsLVGK
XH_P37	5	55201951	55201951	Nonsense_Mutation	INS	-	AGTAAGTGGCT	-	IL31RA	_1031insAGTAA	344_M345delinsEX
XH_P37	5	55201953	55201953	In_Frame_Ins	INS	-	TTA	-	IL31RA	1032_1033insT	p.V344delinsVL
XH_P37	5	56219835	56219835	Frame_Shift_Ins	INS	-	CAGAAGCTTTGG	-	MIER3	CCCACCCAAAG	p.F325fs
XH_P37	5	56527135	56527135	Nonsense_Mutation	INS	-	ATTCTTTAAAAAT	-	GPBP1	AAAACATTCTTTA	140delinsESHQNILX
XH_P37	5	58284371	58284371	Nonsense_Mutation	INS	-	TTTTAGTGATAG	-	PDE4D	CATGAACTATCA	277_H278delinsLTX
XH_P37	5	60455957	60455957	Nonsense_Mutation	INS	-	AGAGGAAAAT	-	SMIM15	_42insATTTTCC	.W14_A15delinsX
XH_P37	5	61689861	61689861	Frame_Shift_Ins	INS	-	GAATAATTTGAT	-	DIMT1	CCTTGCATCAAA	p.N195fs
XH_P37	5	64756155	64756155	Frame_Shift_Ins	INS	-	GTATAGTAAGG	-	ADAMTS6	_473insCCTTACT	p.I158fs
XH_P37	5	65349539	65349539	Frame_Shift_Ins	INS	-	CTTTGAACTTTT	-	ERBIN	insGAGCTTTGA	p.I798fs
XH_P37	5	66461413	66461413	Frame_Shift_Del	DEL	C	-	C	MAST4	c.5623delC	p.P1875fs
XH_P37	5	66481845	66481845	In_Frame_Ins	INS	-	TAAGTTAAAATA	-	CD180	ACTGTATTTTAA	1delinsHILYFNLNKIK
XH_P37	5	68581252	68581252	Frame_Shift_Ins	INS	-	CAAATTC	-	CCDC125	63_964insGAAT	p.C322fs
XH_P37	5	68581253	68581253	Frame_Shift_Ins	INS	-	TAAACTT	-	CCDC125	62_963insAAGT	p.D321fs
XH_P37	5	70805913	70805913	Nonsense_Mutation	INS	-	TTCCAGTTTCTTT	-	BDP1	nsTCTTCTTCCAG	_P999delinsGSSSSFFX
XH_P37	5	70819894	70819894	Frame_Shift_Ins	INS	-	C	-	BDP1	c.5516_5517ins	p.N1839fs
XH_P37	5	70819895	70819895	Frame_Shift_Ins	INS	-	AGTGAAATTCCA	-	BDP1	sTTTAGAGTGAA	p.N1839fs
XH_P37	5	71493956	71493956	In_Frame_Ins	INS	-	AAATGGACATCT	-	MAP1B	4397insAAATGG	/1466delinsEMDIL
XH_P37	5	72873829	72873829	Missense_Mutation	SNP	G	A	G	UTP15	c.G473A	p.R158Q
XH_P37	5	72874956	72874956	Nonsense_Mutation	INS	-	CAGGGTAAGTA	-	UTP15	ATATGCAGGGTA	_L232delinsGNMQGKX
XH_P37	5	73932272	73932272	Silent	SNP	G	T	G	ENC1	c.C39A	p.A13A
XH_P37	5	73932273	73932273	Frame_Shift_Ins	INS	-	ACTATGCGTA	-	ENC1	_38insTACGCAT	p.A13fs
XH_P37	5	74016556	74016556	Nonsense_Mutation	INS	-	TTCTTAAATGTTA	-	HEXB	nsTTAATTCTTAA	.R533delinsLNSX
XH_P37	5	74109838	74109838	In_Frame_Ins	INS	-	TGGAACCTTAGG	-	FAM169A	GAGAGCCTAAG	.66delinsREPKVPNL
XH_P37	5	74137492	74137492	Frame_Shift_Ins	INS	-	ACTTTATGGCTT	-	FAM169A	TTAAAGCCATAA	p.P4fs
XH_P37	5	74651001	74651001	Nonsense_Mutation	INS	-	CAAACTTAATCCT	-	HMGCR	TATCTTCAAACTT	62delinsQYNYLQTX
XH_P37	5	74955197	74955197	In_Frame_Ins	INS	-	ATTTGTCTTTTCA	-	ANKDD1B	ACTTTCCATTTGT	62delinsAILSICLFTVQ
XH_P37	5	75896661	75896661	Frame_Shift_Ins	INS	-	CATTAGA	-	IQGAP2	46_947insCATTA	p.L316fs
XH_P37	5	75896663	75896663	In_Frame_Ins	INS	-	ATC	-	IQGAP2	c.948_949insAT	p.L316delinsLI
XH_P37	5	75914348	75914348	Frame_Shift_Ins	INS	-	GATTTTCTTTTTT	-	F2RL2	AACACAAAAAAG	p.A40fs
XH_P37	5	75927829	75927829	Frame_Shift_Ins	INS	-	TTCAACATTTTA	-	IQGAP2	TATAATTCAACA	p.A139fs
XH_P37	5	76989045	76989045	Frame_Shift_Ins	INS	-	CCAGCAATGTCT	-	TBCA	92insAAGACATT	p.Q98fs
XH_P37	5	78422006	78422006	Frame_Shift_Ins	INS	-	TGGTTTCTGGTG	-	BHMT	64insCTGGTTTC	p.D255fs
XH_P37	5	78422007	78422007	Nonsense_Mutation	INS	-	CTAAGTCTATCAT	-	BHMT	nsTCCCTAAGTC	255_C256delinsDPX
XH_P37	5	79026386	79026386	Frame_Shift_Ins	INS	-	TCAGGGGTAATA	-	CMYA5	99insGTTCAGGG	p.V600fs
XH_P37	5	79026962	79026962	In_Frame_Ins	INS	-	AACGGGGACTT	-	CMYA5	nsATATTAACGG	792delinsNINGDLRS
XH_P37	5	79028784	79028784	In_Frame_Ins	INS	-	TCAATTTCTTTTT	-	CMYA5	97insGTTCAATTT	1399delinsGFNFFFN
XH_P37	5	79031434	79031434	In_Frame_Ins	INS	-	TCCCTTTTCCTCA	-	CMYA5	847insTTCCCTTT	2282delinsSFPFPQ
XH_P37	5	79033190	79033190	Missense_Mutation	SNP	G	T	G	CMYA5	c.G8602T	p.V2868F
XH_P37	5	79033192	79033192	Frame_Shift_Ins	INS	-	CTGTGATCTGGC	-	CMYA5	nsCTTCACTGTGA	p.V2868fs
XH_P37	5	79033774	79033774	Frame_Shift_Ins	INS	-	CTGCTCTTTA	-	CMYA5	_9187insCTGCT	p.N3062fs
XH_P37	5	79033776	79033776	In_Frame_Ins	INS	-	TGACAAGCAACT	-	CMYA5	GTTTTATGACAA	63delinsMFYDKQLFH
XH_P37	5	79732898	79732898	Nonsense_Mutation	INS	-	AATCATATGCAT	-	ZFYVE16	GTTAGAATCATA	132_M133delinsGX
XH_P37	5	79732900	79732900	In_Frame_Ins	INS	-	ATCTTGGAG	-	ZFYVE16	_397insATCTTG	.D132delinsDILE
XH_P37	5	79733694	79733694	Frame_Shift_Ins	INS	-	ATTTTCATCTCTT	-	ZFYVE16	insGAAATTTTCA	p.F397fs

XH_P37	5	79797738	79797738	Splice_Site	INS	-	TTTTATAGGGTA	-	FAM151B	.	.
XH_P37	5	82816272	82816272	Missense_Mutation	SNP	C	G	C	VCAN	c.C2147G	p.T716R
XH_P37	5	82834886	82834886	In_Frame_Ins	INS	-	AATCACTATTTC(	-	VCAN	104insAAATCAC	V1035delinsEITISL
XH_P37	5	86670701	86670701	In_Frame_Ins	INS	-	TTTTGTATTTGT(	-	RASA1	0insATGTTTTGT	660delinsKCFVFVG
XH_P37	5	86674314	86674314	Frame_Shift_Ins	INS	-	ATTACAATGACA	-	RASA1	sTACTAATTACA	p.D816fs
XH_P37	5	88018662	88018662	Missense_Mutation	SNP	G	T	G	MEF2C	c.C1013A	p.P338H
XH_P37	5	88018663	88018663	Nonsense_Mutation	INS	-	ATTATTACTTACT	-	MEF2C	AAAACTAGTAAG	P338delinsKTSKX
XH_P37	5	88100560	88100560	In_Frame_Ins	INS	-	GTTCTTTTTCTTA	-	MEF2C	ATCTTAAGAAAA	8delinsHLKKKNMM
XH_P37	5	94050589	94050589	Frame_Shift_Ins	INS	-	TGAAAGACTTTG	-	MCTP1	1692insACAAAG	p.D564fs
XH_P37	5	95757639	95757639	In_Frame_Ins	INS	-	CTTCTT	-	PCSK1	64_565insAAGA	.F189delinsKKF
XH_P37	5	95757640	95757640	Frame_Shift_Ins	INS	-	TTATGAAATTCT	-	PCSK1	4insGGAGAATTT	p.D188fs
XH_P37	5	96314943	96314943	Nonsense_Mutation	INS	-	ATTAAATAAGAT	-	LNPEP	ACTGAATTAAATA	41_D42delinsLLNX
XH_P37	5	96364235	96364235	Nonstop_Mutation	INS	-	CTGCTATCTTGA	-	LNPEP	077insGCTGCTA	X1026delinsCCYLE
XH_P37	5	98129002	98129002	Frame_Shift_Ins	INS	-	ATCTGTCACA	-	RGMB	_983insATCTGT	p.R328fs
XH_P37	5	98236951	98236951	Frame_Shift_Ins	INS	-	ATGATGAAGAAG	-	CHD1	CATAATCTTCTTC	p.D176fs
XH_P37	5	98236953	98236953	Missense_Mutation	SNP	C	T	C	CHD1	c.G524A	p.C175Y
XH_P37	5	101627275	101627275	Frame_Shift_Ins	INS	-	G	-	SLCO4C1	c.390_391insC	p.S131fs
XH_P37	5	101627277	101627277	In_Frame_Ins	INS	-	GGGTCTCTTTTTC	-	SLCO4C1	CCTTCAAAAAGA	0delinsTYLQKETPIYI
XH_P37	5	102432481	102432481	Frame_Shift_Ins	INS	-	CCCTTCTTCA	-	GIN1	617insTGAAGA	p.K206fs
XH_P37	5	102895000	102895000	In_Frame_Ins	INS	-	AGAAGATCACCT	-	NUDT12	AATCAAGGTGA	08delinsNQGDLLRL
XH_P37	5	102895168	102895168	Frame_Shift_Ins	INS	-	TT	-	NUDT12	c.153_154insAA	p.C52fs
XH_P37	5	102895169	102895169	Nonsense_Mutation	INS	-	CATTCGTTAGGA	-	NUDT12	TGGTTCCTAACG	.M51delinsILGSX
XH_P37	5	106717027	106717027	Frame_Shift_Ins	INS	-	TGTCAGATTTTT	-	EFNA5	AAAACAAAAAT	p.Q206fs
XH_P37	5	108714446	108714446	In_Frame_Ins	INS	-	AAAACTGATAAG	-	PJA2	TTGAACTTATCA	248delinsLELISFLTF
XH_P37	5	111504782	111504782	In_Frame_Ins	INS	-	CATCAGGTAGTA	-	EPB41L4A	TACTATTACTAAG	37delinsLTITNLMNCS
XH_P37	5	112769991	112769991	Nonsense_Mutation	INS	-	TCAACAAGGAGG	-	TSSK1B	46insCCCTCCTT	32_A183delinsAPPCX
XH_P37	5	112769993	112769993	Missense_Mutation	SNP	C	G	C	TSSK1B	c.G544C	p.A182P
XH_P37	5	112860804	112860804	Silent	SNP	T	C	T	YTHDC2	c.T405C	p.P135P
XH_P37	5	112878160	112878160	Frame_Shift_Ins	INS	-	ACATTATCACTC	-	YTHDC2	ACACAAACATTAT	p.F185fs
XH_P37	5	112878161	112878161	In_Frame_Ins	INS	-	TAT	-	YTHDC2	c.556_557insTAT	p.A186delinsVS
XH_P37	5	112917273	112917273	Missense_Mutation	SNP	C	A	C	YTHDC2	c.C2614A	p.P872T
XH_P37	5	112917274	112917274	Nonsense_Mutation	INS	-	CATATAATTTAC	-	YTHDC2	ACATTTCATATA	S873delinsPYFTFHIIYYX
XH_P37	5	114860347	114860347	Nonsense_Mutation	INS	-	AATCCAGCCTATA	-	FEM1C	ATGATTTATAGG	04_F505delinsNVVX
XH_P37	5	114861034	114861034	Frame_Shift_Ins	INS	-	TTAGATATAGAG(	-	FEM1C	CAGCGCCTCTAT	p.M275fs
XH_P37	5	121488320	121488320	Frame_Shift_Ins	INS	-	GCAACTTGCTGT	-	ZNF474	36insGGCAACTT	p.C212fs
XH_P37	5	121759035	121759035	In_Frame_Ins	INS	-	CTTTCATTGTTC	-	SNCAIP	604insCTTTCAT	.S201delinsSLSLF
XH_P37	5	122515880	122515880	In_Frame_Ins	INS	-	CAAATGTTAGGG	-	PRDM6	37insGCAAATGT	512delinsQANVRV
XH_P37	5	122515882	122515882	Missense_Mutation	SNP	C	T	C	PRDM6	c.C1538T	p.P513L
XH_P37	5	125896792	125896792	In_Frame_Ins	INS	-	TTGTTTTTTCTT	-	ALDH7A1	896insAAGAAAA	G299delinsEEKTR
XH_P37	5	125939361	125939361	Frame_Shift_Ins	INS	-	TTGCCATTCCAAA	-	PHAX	7insCTTTGCCATT	p.S66fs
XH_P37	5	126154742	126154742	In_Frame_Ins	INS	-	CTATTTTAAATCA	-	LMNB1	sGCAGCTATTTTA	146delinsQAAILNHF
XH_P37	5	127624162	127624162	Nonsense_Mutation	INS	-	GTTAAACTAAAA	-	FBN2	AAGATGTTTTAG	242_F2243delinsEX
XH_P37	5	130766630	130766630	Frame_Shift_Ins	INS	-	ACAAACAAGTTA	-	RAPGEF6	387insATAACTT	p.S1463fs
XH_P37	5	132018259	132018259	Frame_Shift_Ins	INS	-	GTTACTGTATAC	-	IL4	95insGGTTACTG	p.Y132fs
XH_P37	5	132097316	132097316	Frame_Shift_Ins	INS	-	TGGCT	-	SEPT8	795_796insAGC	p.E266fs
XH_P37	5	134086612	134086612	Frame_Shift_Ins	INS	-	TTTTTAACCGAA	-	CAMLG	64insGTTTTTAA	p.L288fs
XH_P37	5	134223506	134223506	Frame_Shift_Ins	INS	-	TTCAGAGTCATA	-	TXNDC15	nsCATTCAGAGT	p.A7fs
XH_P37	5	135700940	135700940	Splice_Site	SNP	C	G	C	TRPC7	.	.
XH_P37	5	135700941	135700941	Nonsense_Mutation	INS	-	GATCTGAGATCT	-	TRPC7	GAAAGATCTCAG	L2delinsTNKKDLRSIX
XH_P37	5	138651795	138651795	Frame_Shift_Ins	INS	-	TT	-	MATR3	c.183_184insTT	p.T61fs
XH_P37	5	138651796	138651796	Nonsense_Mutation	INS	-	AATACAAATTACT	-	MATR3	AAAGAATACAAA	P63delinsNNKRIQITTX
XH_P37	5	138658597	138658597	Frame_Shift_Ins	INS	-	TTTTGGATTTCTT	-	MATR3	1226insTTTTGGA	p.D409fs
XH_P37	5	140048341	140048341	In_Frame_Ins	INS	-	GCTCTC	-	WDR55	525_526insGCTC	.A175delinsAAL
XH_P37	5	140053882	140053882	In_Frame_Ins	INS	-	GGTTATTTTATTG	-	HARS	AATAATCAATAA	383delinsVIINKITIIK
XH_P37	5	140073250	140073250	Frame_Shift_Ins	INS	-	TTGTATTTACAA	-	HARS2	CTATGTTGTATT	p.K61fs
XH_P37	5	140076941	140076941	Frame_Shift_Ins	INS	-	AAGATTACAG	-	HARS2	_716insAAGATT	p.L239fs
XH_P37	5	140166126	140166126	Frame_Shift_Ins	INS	-	TCCAATCATTTA	-	PCDHA1	AATATTTCCAATG	p.L84fs
XH_P37	5	140201420	140201420	Silent	SNP	T	C	T	PCDHA5	c.T60C	p.L20L
XH_P37	5	140214444	140214444	Frame_Shift_Ins	INS	-	CACAAACAAAAT	-	PCDHA7	GAATTCACAAAC	p.D159fs
XH_P37	5	140220935	140220935	Frame_Shift_Ins	INS	-	CTATTTCTTTATG	-	PCDHA8	ACTTTCTATTTCT	p.G10fs
XH_P37	5	140256137	140256137	In_Frame_Ins	INS	-	AATCATTTT	-	PCDHA12	0_1081insAATCA	Q360delinsQNHF
XH_P37	5	140256138	140256138	Frame_Shift_Ins	INS	-	ATCCCAT	-	PCDHA12	81_1082insATCC	p.E361fs
XH_P37	5	140262403	140262403	In_Frame_Ins	INS	-	CAAAATGCC	-	PCDHA13	0_551insCAAAAT	.N184delinsTKCH
XH_P37	5	140530021	140530021	Frame_Shift_Ins	INS	-	ACACATCTATCTC	-	PCDHB6	84insACACATCT	p.R61fs
XH_P37	5	140574045	140574045	Silent	SNP	C	G	C	PCDHB10	c.C1920G	p.L640L
XH_P37	5	140626468	140626468	Frame_Shift_Ins	INS	-	GTGATGAAATAC	-	PCDHB15	3insAAGTGATG	p.V441fs
XH_P37	5	140740501	140740501	Nonsense_Mutation	INS	-	TAATCTTAGTCTC	-	PCDHGB2	TAAATTAATCTTA	267_D268delinsNFX
XH_P37	5	140746162	140746162	In_Frame_Ins	INS	-	AAGATAGAGTGT	-	PCDHGA5	2266insAAGATA	H755delinsHKIEC
XH_P37	5	140856315	140856315	In_Frame_Ins	INS	-	ATTTCATTTCCT	-	PCDHGC3	633insATTTCAT	Q211delinsQFHFL
XH_P37	5	141694670	141694670	Frame_Shift_Ins	INS	-	TTAGACCA	-	SPRY4	3_4insTGGTCTA	p.E2fs
XH_P37	5	142780008	142780008	Frame_Shift_Ins	INS	-	AAAGAGA	-	NR3C1	96_397insTCTCT	p.T133fs
XH_P37	5	142780009	142780009	Frame_Shift_Ins	INS	-	AGAGTCCTTGGA	-	NR3C1	TGATCTCCAAGG	p.S132fs
XH_P37	5	147661734	147661734	Frame_Shift_Ins	INS	-	CTATCATCTCTTC	-	SPINK13	nsCATCCTATCAT	p.P59fs
XH_P37	5	147781661	147781661	Frame_Shift_Ins	INS	-	GACTTTTTGGTTT	-	FBXO38	TTATTGACTTTTT	p.S127fs
XH_P37	5	147863916	147863916	In_Frame_Ins	INS	-	CTTAAGGTCTAG	-	HTR4	103insAACTAGA	I368delinsKLDLKF
XH_P37	5	149357391	149357391	In_Frame_Ins	INS	-	TTTGTCTTGGTT	-	SLC26A2	177insTTTGTCT	Q59delinsHLSWL
XH_P37	5	149677948	149677948	Nonsense_Mutation	INS	-	C	-	ARSI	c.538_539insG	Y180_D181delinsX
XH_P37	5	149677949	149677949	Frame_Shift_Ins	INS	-	TTCTCCAGTGACA	-	ARSI	insATTGTCACTG	p.Y180fs
XH_P37	5	150050199	150050199	Missense_Mutation	SNP	C	T	C	MYOZ3	c.C215T	p.A72V
XH_P37	5	150050200	150050200	Nonsense_Mutation	INS	-	TCATGATTGTTT	-	MYOZ3	217insTCATGAT	p.A72delinsASX
XH_P37	5	150660641	150660641	Nonsense_Mutation	INS	-	TTCTTTGTGACC	-	SLC36A3	78insTAGGTCAC	Q360_V361delinsX
XH_P37	5	154281037	154281037	Nonsense_Mutation	INS	-	CAACTAATTTTTT	-	GEMIN5	873insTAAAAAAT	.H958delinsLKNX
XH_P37	5	154281039	154281039	Nonsense_Mutation	SNP	G	T	G	GEMIN5	c.C2871A	p.Y957X
XH_P37	5	154395684	154395684	Frame_Shift_Ins	INS	-	GTTCTTTCAACT	-	KIF4B	66insTCGTTCTT	p.V755fs
XH_P37	5	154395686	154395686	Missense_Mutation	SNP	G	T	G	KIF4B	c.G2267T	p.S756I
XH_P37	5	156522370	156522370	In_Frame_Ins	INS	-	TAA	-	HAVCR2	c.622_623insTTA	p.G208delinsVR
XH_P37	5	156522371	156522371	Nonsense_Mutation	INS	-	AATTTTTATTTAA	-	HAVCR2	AAAATATTAAATA	08_I209delinsHNKYX
XH_P37	5	156675883	156675883	Frame_Shift_Ins	INS	-	AGATGGACCCTT	-	ITK	1658insAGATGG	p.S553fs
XH_P37	5	157221945	157221945	Frame_Shift_Ins	INS	-	T	-	CLINT1	c.1047_1048insA	p.F350fs
XH_P37	5	157221946	157221946	Nonsense_Mutation	INS	-	TTTTGGTTACAG	-	CLINT1	sGTAGTCTGTAA	349_F350delinsEX
XH_P37	5	158621791	158621791	Nonsense_Mutation	INS	-	TGACATTTTATT	-	RNF145	16insAAATAAAA	I106_L107delinsKX
XH_P37	5	159521058	159521058	Frame_Shift_Ins	INS	-	ATAACCAATCAA	-	PWWP2A	9insTATTGATTG	p.G200fs
XH_P37	5	159996621	159996621	Frame_Shift_Ins	INS	-	AA	-	ATP10B	.3819_3820insT	p.A1274fs
XH_P37	5	159996623	159996623	In_Frame_Ins	INS	-	AACATGGAC	-	ATP10B	7_3818insGTCCA	N1273delinsSPCY
XH_P37	5	160886695	160886695	Silent	SNP	G	A	G	GABRB2	c.C393T	p.H131H
XH_P37	5	161113998	161113998	Frame_Shift_Ins	INS	-	ATTTAGAATTTAT	-	GABRA6	CCTTATTTAGAA	p.D72fs
XH_P37	5	162939148	162939148	In_Frame_Ins	INS	-	TCTCTCAAATAT	-	MAT2B	ACAGTTCTCTCA	68delinsENSSLKYIR
XH_P37	5	168189559	168189559	Frame_Shift_Ins	INS	-	TTCTGAACACCTA	-	SLIT3	1595insTAGGTGT	p.E532fs

XH_P37	5	169685144	169685144	In_Frame_Ins	INS	-	CAGGCAGAT	-	LCP2	6_997insATCTG	.A333delinsICLA
XH_P37	5	172396471	172396471	Nonsense_Mutation	INS	-	AATAGGAACAC	-	RPL26L1	TACAAAATAGG	.K122delinsNTKX
XH_P37	5	173376594	173376594	Frame_Shift_Ins	INS	-	TTTCTCCCTCTAT	-	CPEB4	1466insTTTCTC	p.P489fs
XH_P37	5	174868783	174868783	In_Frame_Ins	INS	-	CAAATACATTCC	-	DRD1	20insCTGGAAT	440delinsHWNVFG
XH_P37	5	176562785	176562785	Frame_Shift_Ins	INS	-	GTCTTTGTTACT	-	NSD1	insATAGTCTTTG	p.L227fs
XH_P37	5	176637253	176637253	In_Frame_Ins	INS	-	GTGGCCCTTTTT	-	NSD1	TTGTATGTGGC	8delinsNCMWPFFTL
XH_P37	5	176734136	176734136	Nonsense_Mutation	INS	-	CAAGTATTTATT	-	MXD3	ACACTCAATAAA	_S190delinsRHSINTCX
XH_P37	5	176895901	176895901	Splice_Site	SNP	C	T	C	DBN1	.	.
XH_P37	5	178541069	178541069	Frame_Shift_Ins	INS	-	TCCTGATTCCAA	-	ADAMTS2	insCCTTTTGGAA	p.N1145fs
XH_P37	5	178541188	178541188	Frame_Shift_Ins	INS	-	AAAAGACCAGA	-	ADAMTS2	316insTTTCTGG	p.I1106fs
XH_P37	5	179136924	179136924	Frame_Shift_Ins	INS	-	AAAGCTAACTAC	-	CANX	nsGGAAAAGCTA	p.K193fs
XH_P37	5	179151749	179151749	Nonsense_Mutation	INS	-	CATGTATTATGT	-	CANX	CATGTTCATGTA	538delinsNFVYMFMYYVIX
XH_P37	5	179300202	179300202	Frame_Shift_Ins	INS	-	AACAGAGGCTGA	-	TBC1D9B	insACTTTCAGCC	p.P745fs
XH_P37	5	180335794	180335794	Frame_Shift_Ins	INS	-	GATAAAT	-	BTNL8	58_259insGATA	p.K86fs
XH_P37	5	180335795	180335795	In_Frame_Ins	INS	-	ATCAAAACCCTT	-	BTNL8	260insATCAAAA	.L87delinsHQNPL
XH_P37	6	572585	572585	Frame_Shift_Ins	INS	-	AGGTGGTTGTA	-	EXOC2	1378insCTACAA	p.L460fs
XH_P37	6	2779651	2779651	Frame_Shift_Ins	INS	-	A	-	WRNIP1	c.1411_1412ins	p.P471fs
XH_P37	6	2779653	2779653	Frame_Shift_Ins	INS	-	TGTCAATGTGCT	-	WRNIP1	14insTTTGTCAA	p.P471fs
XH_P37	6	5216916	5216916	Frame_Shift_Ins	INS	-	ATGAAACT	-	LYRM4	1_142insAGTTT	p.D48fs
XH_P37	6	7386031	7386031	Frame_Shift_Ins	INS	-	GGCTGTTTTTAT	-	CAGE1	TGAAGATAAAA	p.D90fs
XH_P37	6	7579754	7579754	Frame_Shift_Ins	INS	-	AGAAGCAGTACT	-	DSP	332insAAGAAG	p.I1111fs
XH_P37	6	7580957	7580957	In_Frame_Ins	INS	-	GGCCTTTTGCTG	-	DSP	4535insGGCCTT	1512delinsWPFAD
XH_P37	6	7584273	7584273	Nonsense_Mutation	INS	-	CGTAACTGACCT	-	DSP	4982insCGTAAC	p.I1661delinsTX
XH_P37	6	7584886	7584886	Frame_Shift_Ins	INS	-	A	-	DSP	c.5594_5595ins	p.R1865fs
XH_P37	6	7584887	7584887	Nonsense_Mutation	INS	-	TGTGCTTCTAAT	-	DSP	596insCTGTGCT	65_R1866delinsRLCFX
XH_P37	6	7606393	7606393	Frame_Shift_Ins	INS	-	AATGTTAATCA	-	SNRNP48	_937insAATGTT	p.K312fs
XH_P37	6	7606395	7606395	Missense_Mutation	SNP	A	T	A	SNRNP48	c.A938T	p.D313V
XH_P37	6	10586649	10586649	Nonsense_Mutation	INS	-	ATTCTGGGTCAG	-	GCNT2	28insGATTCTGG	L143_L144delinsX
XH_P37	6	10586651	10586651	In_Frame_Ins	INS	-	ATC	-	GCNT2	c.429_430insAT	p.L143delinsLI
XH_P37	6	12125990	12125990	Frame_Shift_Ins	INS	-	TTGGATGT	-	HIVEP1	2_5963insTTGG	p.L1988fs
XH_P37	6	12125992	12125992	Frame_Shift_Ins	INS	-	A	-	HIVEP1	c.5964_5965ins	p.L1988fs
XH_P37	6	12136121	12136121	Frame_Shift_Ins	INS	-	TTTGGTATCTATA	-	HIVEP1	4insAATTTGGT	p.E2161fs
XH_P37	6	16307040	16307040	Frame_Shift_Ins	INS	-	ATGCT	-	ATXN1	967_1968insAG	p.G656fs
XH_P37	6	16307042	16307042	Missense_Mutation	SNP	C	T	C	ATXN1	c.G1966A	p.G656S
XH_P37	6	17637647	17637647	Frame_Shift_Ins	INS	-	G	-	NUP153	c.2074dupC	p.Q692fs
XH_P37	6	17637649	17637649	Nonsense_Mutation	INS	-	GGAGTAATTTTG	-	NUP153	AATAAGCAAAA	L_Q692delinsVISKITPX
XH_P37	6	17646367	17646367	Nonsense_Mutation	INS	-	ACCTTTTTGACG	-	NUP153	AAGCACCGTCAA	51delinsKKHRQKGX
XH_P37	6	20102617	20102617	Frame_Shift_Ins	INS	-	TTACATGCATCT	-	MBOAT1	AAAATAAAGATG	p.I463fs
XH_P37	6	24533902	24533902	Frame_Shift_Ins	INS	-	CATTAAAGAC	-	ALDH5A1	_1571insCATTA	p.Y524fs
XH_P37	6	25913674	25913674	Nonsense_Mutation	INS	-	GCAGTTCTT	-	SLC17A2	_1160insAAGA	p.L387delinsX
XH_P37	6	25914888	25914888	In_Frame_Ins	INS	-	AAAAATATAAGC	-	SLC17A2	074insAAGCTTA	V358delinsVSLYFS
XH_P37	6	25914890	25914890	Missense_Mutation	SNP	C	T	C	SLC17A2	c.G1072A	p.V358I
XH_P37	6	26056277	26056277	Frame_Shift_Ins	INS	-	TCGTATGGGAGT	-	HIST1H1C	insGTAACTCCC	p.K127fs
XH_P37	6	26197293	26197293	Silent	SNP	C	G	C	HIST1H3D	c.G186C	p.L62L
XH_P37	6	26273494	26273494	Frame_Shift_Ins	INS	-	CCCTCATTATTTA	-	HIST1H2BI	292insCCCTCAT	p.T97fs
XH_P37	6	26273495	26273495	Missense_Mutation	SNP	G	C	G	HIST1H2BI	c.G292C	p.A98P
XH_P37	6	26637707	26637707	Frame_Shift_Ins	INS	-	G	-	ZNF322	c.1074_1075ins	p.S359fs
XH_P37	6	26637708	26637708	Nonsense_Mutation	INS	-	TACCAATATTAA	-	ZNF322	74insTTTTTAATA	358_S359delinsNFX
XH_P37	6	27100406	27100406	Frame_Shift_Ins	INS	-	CGCTAAGTAAAC	-	HIST1H2BJ	nsCACTGTTTACT	p.V42fs
XH_P37	6	27278647	27278647	In_Frame_Ins	INS	-	CCTCTC	-	POM121L2	02_1303insGAG	p.L435delinsERL
XH_P37	6	27368429	27368429	Frame_Shift_Ins	INS	-	GG	-	ZNF391	c.280_281insGG	p.S94fs
XH_P37	6	27368430	27368430	In_Frame_Ins	INS	-	CTTCGTTTTTAT	-	ZNF391	_282insCTTCGTT	.S94delinsSFVFI
XH_P37	6	28056993	28056993	In_Frame_Ins	INS	-	TTTTTCTCCAGCA	-	ZNF165	nsGATTTTTTTCT	01delinsGDFFLQHK
XH_P37	6	28227664	28227664	Frame_Shift_Ins	INS	-	ACTCCAA	-	NKAPL	15_516insACTC	p.S172fs
XH_P37	6	28268784	28268784	Frame_Shift_Ins	INS	-	AAAATCCTCAAT	-	PGBD1	insATAAAAATC	p.F385fs
XH_P37	6	35199593	35199593	Frame_Shift_Ins	INS	-	CCTTGTCAGCTA	-	SCUBE3	nsACACCCTTGTC	p.F142fs
XH_P37	6	35201038	35201038	Frame_Shift_Ins	INS	-	CCAATTATCTCA	-	SCUBE3	70insCCCAATTA	p.H223fs
XH_P37	6	35280317	35280317	Splice_Site	INS	-	ATTCCA	-	DEF6	.	.
XH_P37	6	36297945	36297946	Frame_Shift_Del	DEL	GG	-	GG	C6orf222	c.522_523del	p.D174fs
XH_P37	6	36297946	36297946	Frame_Shift_Ins	INS	-	TCTTCTTTCTC	-	C6orf222	522insGAGAAA	p.D174fs
XH_P37	6	38813525	38813525	In_Frame_Ins	INS	-	GATATGCAAATA	-	DNAH8	22insAGATATG	1674delinsVDMQIS
XH_P37	6	38831780	38831780	In_Frame_Ins	INS	-	GTAAAG	-	DNAH8	42_6443insGTA	.F2148delinsCKV
XH_P37	6	38831782	38831782	Nonsense_Mutation	INS	-	AATATACCTTCT	-	DNAH8	CTCTATAATATA	148delinsFLLYNIPSX
XH_P37	6	38877467	38877467	Frame_Shift_Ins	INS	-	CAGAGGGGTT	-	DNAH8	_9688insCAGAG	p.T3229fs
XH_P37	6	38994426	38994426	In_Frame_Ins	INS	-	TGTCTCAGTGTT	-	DNAH8	insCATTTGTCT	607delinsTFVSVFRF
XH_P37	6	41001906	41001906	Frame_Shift_Ins	INS	-	TTATCTTCAT	-	UNC5CL	_400insATGAAG	p.G134fs
XH_P37	6	41884595	41884595	Frame_Shift_Ins	INS	-	TAAAGGTGGTAT	-	MED20	97insAATACCAC	p.A33fs
XH_P37	6	42986652	42986652	Missense_Mutation	SNP	G	A	G	KLHDC3	c.G872A	p.R291H
XH_P37	6	43014274	43014274	Nonsense_Mutation	INS	-	CCCCAAGACCTA	-	CUL7	715insATAGGTC	905_V906delinsEX
XH_P37	6	43034245	43034245	In_Frame_Ins	INS	-	AAA	-	KLC4	c.542_543insAA	p.I181delinsIN
XH_P37	6	43034246	43034246	In_Frame_Ins	INS	-	AAAGGA	-	KLC4	43_544insAAAG	p.I181delinsIKG
XH_P37	6	43153831	43153831	Nonsense_Mutation	SNP	C	T	C	CUL9	c.C889T	p.Q297X
XH_P37	6	43416877	43416877	Nonsense_Mutation	INS	-	AATGACAT	-	ABCC10	4_4055insAATG	352_S1353delinsQX
XH_P37	6	43477337	43477337	Missense_Mutation	SNP	G	C	G	LRRC73	c.C187G	p.L63V
XH_P37	6	43488950	43488950	In_Frame_Ins	INS	-	TTTTTACCT	-	POLR1C	3_954insTTTTTA	.P318delinsPFYL
XH_P37	6	43491668	43491668	Missense_Mutation	SNP	T	C	T	XPO5	c.A3553G	p.M1185V
XH_P37	6	43491670	43491670	In_Frame_Ins	INS	-	TCA	-	XPO5	3550_3551insT	.P1184delinsLT
XH_P37	6	43519132	43519132	In_Frame_Ins	INS	-	CACACC	-	XPO5	30_1631insGGT	.K544delinsRCE
XH_P37	6	43555218	43555218	Nonsense_Mutation	INS	-	TAACAGAGACTA	-	POLH	GGCTTAACAGA	.V161delinsVAX
XH_P37	6	43582076	43582076	In_Frame_Ins	INS	-	TCTGTTGGTAAA	-	POLH	nsATACTCTGTTG	518delinsDTLLVKSL
XH_P37	6	43623389	43623389	In_Frame_Ins	INS	-	TTTTCTTCCTTT	-	RSPH9	_440insTTTTCTT	.T147delinsIFFLS
XH_P37	6	44218099	44218099	In_Frame_Ins	INS	-	TCCTGCACGTTA	-	HSP90AB1	insACATCCTGCA	240delinsETSCTLS
XH_P37	6	44982601	44982601	Frame_Shift_Ins	INS	-	ATTATACC	-	SUPT3H	2_43insGGTATA	p.M15fs
XH_P37	6	44982602	44982602	Nonsense_Mutation	INS	-	TGTTACC	-	SUPT3H	41_42insGGTAA	.Y14_M15delinsX
XH_P37	6	46657444	46657444	Frame_Shift_Ins	INS	-	TTGCCTGGCT	-	TDRD6	_1580insTTGCC	p.A527fs
XH_P37	6	46660990	46660990	Nonsense_Mutation	INS	-	GAACATAATCCT	-	TDRD6	sCTTGAGAACAT	709_E1710delinsSX
XH_P37	6	47522500	47522500	In_Frame_Ins	INS	-	AAA	-	CD2AP	c.539_540insAA	p.S180delinsSK
XH_P37	6	47522502	47522502	Frame_Shift_Ins	INS	-	AAAAACAACTTC	-	CD2AP	42insCAAAAACA	p.E181fs
XH_P37	6	50682942	50682942	Silent	SNP	C	T	C	TFAP2D	c.C153T	p.T51T
XH_P37	6	50807890	50807890	Frame_Shift_Ins	INS	-	CATCCCCTGAAT	-	TFAP2B	63insACATCCCC	p.R321fs
XH_P37	6	50811103	50811103	Nonstop_Mutation	INS	-	CACCCTCAGTGT	-	TFAP2B	1382insCACCCTC	X461delinsSPSVS
XH_P37	6	52147651	52147651	Nonsense_Mutation	INS	-	CACTAAATGTAA	-	MCM3	230insCTTACATT	N77_N78delinsTYIX
XH_P37	6	52993184	52993184	Frame_Shift_Ins	INS	-	CTTTTTTCTTTTT	-	GCM1	AAAAAGAAAAA	p.Q377fs
XH_P37	6	56437777	56437777	Frame_Shift_Ins	INS	-	T	-	DST	c.5452_5453ins	p.P1818fs
XH_P37	6	56437778	56437778	Frame_Shift_Ins	INS	-	GAAAAGATGTTA	-	DST	insTCAGTAACAT	p.P1818fs
[truncated: 3,408,869 more chars]
